# Unraveling the Causal Links Between Immune Cells, Lipids, and Cardiovascular Diseases: Insights from Mendelian Randomization

**DOI:** 10.5334/gh.1444

**Published:** 2025-07-03

**Authors:** Fengwei He, Tian Yang, Wentao Zhang, Ming Liu, Hao Wu

**Affiliations:** 1 Department of Cardiology, The First Hospital of Shanxi Medical University, Taiyuan 030001, China; 2 Department of Cell Biology and Medical Genetics, School of Basic Medical Science, Shanxi Medical University, Taiyuan 030001, China

**Keywords:** Causality, Immune Cells, Lipid Profiles, Cardiovascular Diseases, Mediator, Mendelian Randomization

## Abstract

**Background and Aim:**

Cardiovascular diseases (CVD), including coronary artery disease (CAD), myocardial infarction (MI), atrial fibrillation (AF), and ischemic stroke (IS), are major causes of morbidity and mortality worldwide. Immune cells play crucial roles in CVD, but causal links between immune cell subtypes and CVD risk remain unclear. This study used Mendelian randomization (MR) to investigate these associations.

**Methods and Results:**

Exposure data were analyzed with a P < 1 × 10^–5^ threshold, excluding linkage disequilibrium SNPs. MR of 731 immune cell types used the inverse variance weighted (IVW) method, with pleiotropy and heterogeneity tests. Lipid profiles (HDL, LDL, VLDL, triglycerides) were assessed as mediators.

Increased CD27 on unswitched memory B cells, CD28^–^ DN T cells, and CX3CR1 on CD14^–^ CD16^+^ monocytes raised CVD risk, while CD28 on Tregs and HLA DR^++^ monocytes were protective. For CAD, CD24^+^ CD27^+^ %B cells and SSC-A on HLA DR^+^ NK cells were protective, with certain T cells increasing risk. Similar trends were observed for MI, AF, and IS. Reverse MR showed no CVD effects on these positive immune traits. Lipid profiles mediated CVD risk, with HDL protective and LDL, VLDL, and triglycerides increasing risk. Mediation analyses showed LDL and triglycerides partially mediated CX3CR1-monocyte effects on MI risk. Functional enrichment identified cytokine signaling and inflammation in CVD.

**Conclusions:**

Our findings highlight immune cell subtypes and lipid traits in CVD risk. Regulatory T cells and protective phenotypes are therapeutic targets, while LDL and triglycerides mediate immune-disease pathways, emphasizing immune-lipid interactions for targeted therapies.

## Introduction

Cardiovascular disease (CVD) is a leading cause of mortality worldwide, particularly coronary artery disease (CAD), myocardial infarction (MI), and heart failure (HF), imposing a significant public health and economic burden (1). In addition to traditional risk factors such as hypertension, diabetes, smoking, and inactivity, growing evidence suggests that immune dysregulation and lipid metabolism disorders play crucial roles in the development and progression of CVD (2). Understanding the interplay between these mechanisms is essential for developing new preventive and therapeutic strategies.

Immune cells are key players in the progression of CVD, particularly in the formation and rupture of atherosclerotic plaques, a hallmark of CVD (3). Macrophages, T cells, and B cells are involved in plaque development, contributing to cardiovascular events such as MI (4). However, due to the complexity of CVD, observational studies alone are insufficient to establish a causal role for immune cells. Thus, a robust method to infer causality and minimize confounding factors is needed.

Dysregulation of lipid metabolism, particularly levels of high-density lipoprotein (HDL), low-density lipoprotein (LDL), very low-density lipoprotein (VLDL), and triglycerides, is well-recognized as a key risk factor for CVD (5). LDL is a major contributor to plaque formation, while HDL has protective effects by clearing cholesterol from artery walls (6). The interaction between immune cells and lipid metabolism may be pivotal in CVD pathogenesis (7).

Mendelian Randomization (MR) is a powerful method that uses genetic variants as instrumental variables (IVs) to infer causality between exposures and outcomes (8). MR can mitigate the effects of confounding factors, as genetic variants are randomly assigned at birth, akin to a natural randomized trial (9). This method is ideal for investigating the causal relationships between immune cells, lipid metabolism, and CVD.

Although both immune cells and lipid metabolism are linked to CVD, the causal pathways remain unclear. Specifically, this study seeks to address:

- Do immune cells mediate CVD through effects on lipid metabolism?
- Which specific immune cell subtypes affect CVD risk via particular lipid particles?

This study aims to use a two-step MR analysis to assess the causal impact of immune cells on CVD and investigate whether lipid metabolism acts as a mediator, thus elucidating the interplay between the immune system, lipids, and CVD.

This research will fill a critical gap by clarifying the causal relationships between immune cells, lipid metabolism, and CVD. By identifying specific immune cells and lipid pathways involved in CVD, the study may provide novel targets for personalized prevention and treatment strategies for CVD.

## Materials and Methods

### Study design

**Figure 1:**
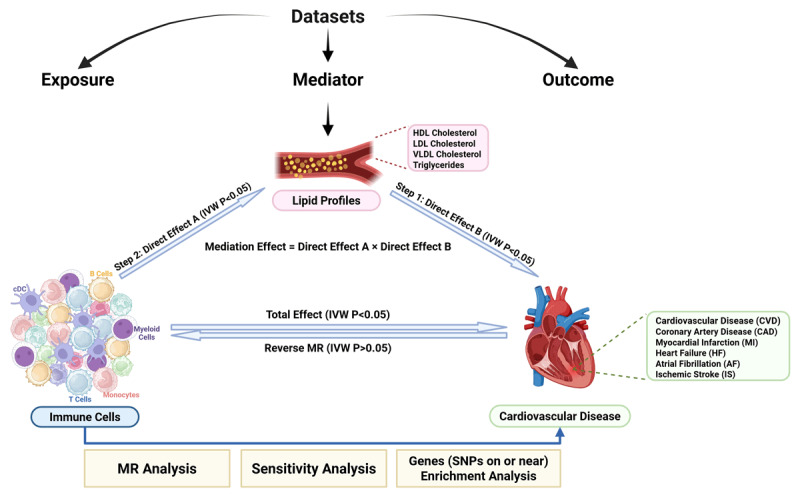
Overview of the study process using Mendelian Randomization (MR) to assess the causal relationships between immune cells, lipid profiles, and cardiovascular disease (CVD). Immune cells (exposure) affect lipid profiles (mediator), which in turn influence CVD outcomes (outcome). The Two-Step MR approach involves **Step 1** (Direct Effect B) linking lipid profiles to CVD and **Step 2** (Direct Effect A) linking immune cells to lipid profiles. The mediation effect is calculated as the product of these effects. Total and reverse MR analyses assess overall impact and causality, with sensitivity and enrichment analyses examining genetic factors.

Figure 1 illustrated our study’s methodology, using MR to explore the causal pathways between immune cells, lipid profiles, and CVD. We employed a two-step MR approach to analyze how lipid profiles mediate the impact of immune cells on CVD, following three MR assumptions.

Initially, we performed a two-sample MR to identify causal relationships between immune cell traits and CVD. Concurrently, we used reverse MR with SNPs linked to these diseases as IVs to exclude any immune cell traits with positive reverse MR findings. We then measured the causal effects from immune cells to lipid profiles (Direct Effect A) and from lipid profiles to CVD (Direct Effect B) to see if lipid profiles mediated these relationships. We quantified the mediation effect and the proportion mediated by lipid profiles. Finally, we performed functional enrichment analysis on SNPs-related genes from immune cells in CVD to detail the biological relevance of these variants at various disease stages.

### Data sources

For this two-sample MR study, we systematically identify the causal impacts of various immune cells and lipid particles on CVD risk. The exposure data for 731 immune cell types are sourced from the IEU Open GWAS project, covering datasets from ebi-a-GCST90001391 to ebi-a-GCST90002121. This dataset details various immune cells, including B cells, dendritic cells (cDC), different T cell stages, regulatory T cells (Treg), lymphocyte subsets, monocytes, and myeloid cells (Table S1). Lipid data are focusing on four lipid particles: HDL (ieu-b-109), LDL (ieu-b-110), VLDL (met-d-VLDL_C), and triglycerides (ieu-b-111). Outcome data for CVD and related conditions were retrieved using relevant keywords from the GWAS database, selecting the largest datasets available from European descent participants to minimize population stratification bias. Table 1 lists the detailed information of datasets used in this study.

**Table 1:** Characteristics of selected GWAS data.


NAME	GWAS ID	SNPs	NCASE	NCONTROL	SAMPLE SIZE	POPULATION	PMID

**Exposure**							

Immune cell	ebi-a-GCST90001391-- ebi-a-GCST90002121	18,622	NA	NA	2,309,119	European	32929287

**Mediation**							

HDL cholesterol	ieu-b-109	12,321,875	NA	NA	403,943	European	32203549

LDL cholesterol	ieu-b-110	12,321,875	NA	NA	440,546	European	32203549

VLDL cholesterol	met-d-VLDL_C	12,321,875	NA	NA	115,078	European	NA

Triglycerides	ieu-b-111	12,321,875	NA	NA	441,016	European	32203549

**Outcome**							

Cardiovascular disease	ebi-a-GCST90086055	14,485,079	15,009	41,628	56,637	European	33893285

Coronary artery disease	ebi-a-GCST003116	8,597,751	42,096	361	141,217	European	26343387

Myocardial infarction	ebi-a-GCST90018877	24,172,914	20,917	440,906	461,823	European	34594039

	ebi-a-GCST011364	10,290,368	14,825	2,680	395,795	European	33532862

	ebi-a-GCST011365	8,106,745	14,825	44,000	395,795	European	33532862

Heart failure	ukb-d-HEARTFAIL	9,858,439	1,405	359,789	361,194	European	NA

Atrial fibrillation	ebi-a-GCST006414	33,519,037	60,620	970,216	1,030,836	European	30061737

Ischemic stroke	ebi-a-GCST90018864	24,174,314	11,929	472,192	484,121	European	34594039

	ebi-a-GCST005843	7,537,579	34,217	406,111	440,328	European	29531354


### Selection criteria for instrumental variable

To investigate the causal pathways between immune cells, lipid profiles, and CVD, we collected SNP data as IVs for both exposures and outcomes. This approach ensures that our effect estimates are free from confounding factors and reverse causal influences, similar to random allocation in randomized controlled trials. Drawing from current MR research related to immune cells, we selected genetic IVs with significant genetic correlations at *P* < 10^–5^. We excluded SNPs exhibiting linkage disequilibrium (LD) with r^2^ > 0.001 within a 10,000 kb range, and SNPs with F-statistics <10 were deemed weak IVs and excluded from further analysis. The remaining SNPs meeting these criteria were utilized for MR analysis. This included a first-step MR from lipid profiles to CVD, and a second-step MR from immune cells to lipid profiles, following the same principles for instrumental variable selection.

### Enrichment analysis of variants

To investigate the roles of genetic variants in diseases, we identified the corresponding or nearby genes of causal SNPs using the Ensembl Genome Browser. We then performed functional enrichment analysis for each disease using the Metascape website (https://metascape.org/gp/index.html), which provides a comprehensive set of default ontologies including gene ontology (GO) processes, Kyoto Encyclopedia of Genes and Genomes (KEGG) pathways, Reactome gene sets, and canonical pathways. The results of these analyses were downloaded and subsequently visualized using the online bioinformatics platform (http://www.bioinformatics.com.cn/).

### Statistical analysis

The primary method in this study was MR analysis to explore the causal effects of immune cells – lipid profiles – CVD, utilizing genetic variants as instruments. We used R (version 4.2.2) for data analysis and visualization, employing the “TwoSampleMR,” “VariantAnnotation,” and “ieugwasr” packages for two-sample MR analysis. Methods including MR Egger, inverse variance weighting (IVW), weighted median, simple mode, and weighted mode were used, with IVW as the principal method and a *P* < 0.05 indicating statistical significance. The odds ratio (OR) highlighted risk (OR > 1) and protective (OR < 1) factors.

Heterogeneity among SNPs was evaluated using Cochran’s Q statistic and MR pleiotropy residual sum and outlier (MR-PRESSO) tests, where a *P* > 0.05 suggested no significant heterogeneity. We further analyzed pleiotropy and sensitivity using the MR-Egger and Leave-one-out methods; an MR-Egger intercept close to zero with a *P* > 0.05 indicated no significant horizontal pleiotropy. Scatter plots, funnel plots, and MR leave-one-out sensitivity analysis are used to analyze the data. The scatter plot shows that outcomes are unaffected by outliers, while the funnel plot illustrates the correlation strength and absence of heterogeneity. MR leave-one-out analysis involves removing each SNP one at a time and recalculating the meta effect to assess sensitivity. If the result changes significantly, it indicates a SNP with substantial impact. Thus, the scatter plot reaffirms the results’ robustness against outliers. Significant associations between immune cell traits and CVD, lipid profiles and CVD, immune cell traits and lipid profiles were adjusted using the Benjamini-Hochberg method within the False Discovery Rate (FDR) framework, setting a significance threshold at *P* < 0.05.

## Results

### Causal associations between immune cells and cardiovascular diseases

**Figure 2:**
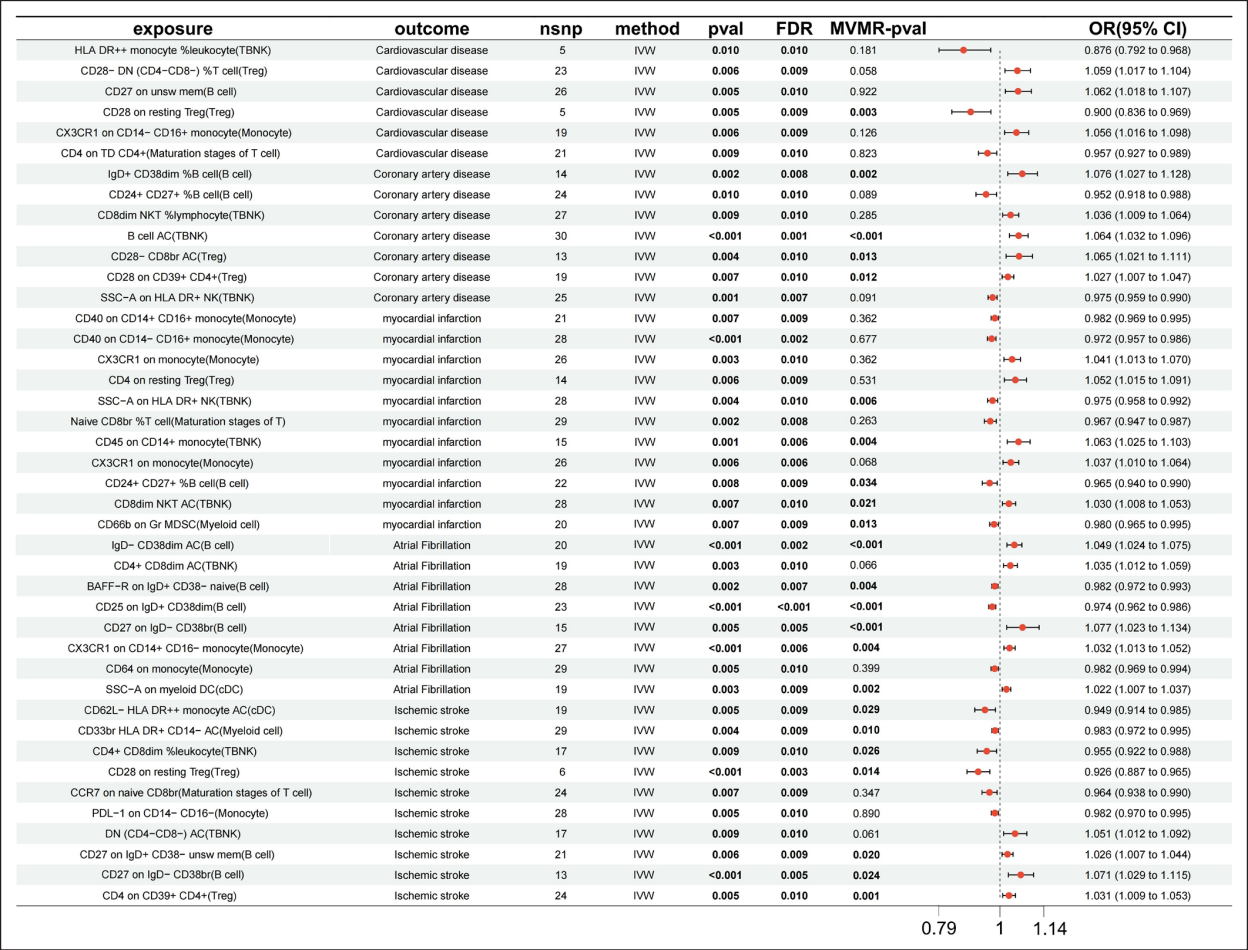
Forest plot summarizing the MR analysis results for the association between different immune cell phenotypes (exposure) and various cardiovascular outcomes (CVD, coronary artery disease, myocardial infarction, atrial fibrillation, ischemic stroke). The table includes details such as the number of SNPs used (nsnp), method (Inverse variance weighted), p-value, false discovery rate (FDR), and odds ratio (OR) with 95% confidence intervals (CI). Statistically significant associations are highlighted, providing insights into the potential causal roles of specific immune cell types in cardiovascular diseases.

Exposure data were extracted, and an association analysis was conducted using an R package with a significance threshold of P < 1e-05, excluding linkage disequilibrium SNPs (kb = 10,000; r^2^ = 0.001). All SNPs had F-statistic values exceeding 10, eliminating weak IVs (Table S2). We performed MR analysis on 731 immune cell types (Table S3) using the IVW method, with criteria of P < 0.05 and pleiotropy test P > 0.05 to identify positive immune cells. The causal effects of these cells on CVD, including CAD, MI, atrial fibrillation (AF), and ischemic stroke (IS), are summarized in Figure 2 and Supplementary Table S4.

The study revealed significant associations between various immunophenotypes and CVD risk. Increased expression of CD27 on unswitched memory B cells (OR = 1.062, P = 0.010), CD28^–^ DN (CD4^–^ CD8^–^) T cells (OR = 1.059, P = 0.009), and CX3CR1 on CD14^–^ CD16^+^ monocytes (OR = 1.056, P = 0.009) correlated with higher CVD risk. In contrast, CD28 on resting Tregs (OR = 0.009, P = 0.009) and HLA DR^++^ monocyte %leukocytes (OR = 0.876, P = 0.01) showed protective effects, suggesting that greater expression may aid in prevention.

For CAD, CD24^+^ CD27^+^ %B cells (OR = 0.952, 95% CI: 0.918–0.988, P = 0.01) and SSC-A on HLA DR^+^ NK cells (OR = 0.975, 95% CI: 0.959–0.990, P = 0.007) were protective, while lymphocyte subgroups like B cell AC and CD28- CD8br AC indicated increased risk. Notably, markers such as CD28 on CD39^+^ CD4^+^ and CD8dim NKT %lymphocytes also displayed significant associations with CAD.

Regarding MI, CD45 on CD14^+^ monocytes, CX3CR1 on monocytes, and CD4 on resting Tregs were linked to increased risk. Conversely, CD40 on CD14^–^ CD16^+^ monocytes, naive CD8br %T cells, and CD24^+^ CD27^+^ %B cells exhibited protective effects. Other populations, such as CD8dim NKT AC and CD40 on CD14^+^ CD16^+^ monocytes, also influenced MI risk.

For AF, CD25 on IgD^+^ CD38dim (OR = 0.974, 95% CI: 0.962–0.986, P < 0.001), BAFF-R on IgD^+^ CD38- naive B cells (OR = 0.982, 95% CI: 0.972–0.993, P = 0.007), and CD64 on monocytes (OR = 0.982, 95% CI: 0.969–0.994, P = 0.01) were inversely correlated, indicating protective roles. However, IgD^–^ CD38dim AC and other markers were associated with increased AF risk.

In IS, higher CD28 expression on resting Tregs, along with CD33br HLA DR^+^ CD14^–^ AC and other markers, correlated with reduced risk, suggesting protective effects. Conversely, increased levels of B cells (CD27 on IgD^–^ CD38br and CD27 on IgD^+^ CD38^–^ unswitched memory), CD4 on CD39^+^ CD4^+^ (Treg), and DN (CD4^–^CD8^–^) AC (TBNK) were linked to higher ischemic stroke risk, indicating hazardous effects.

Overall, Regulatory T cells (CD28 on resting Tregs) play a protective role in CVD and IS. CD24^+^ CD27^+^ % B cell and SSC-A on SSC^–^A on HLA DR^+^ NK also offer protection against CAD and MI. On the other hand, B cells (CD27 on IgD^–^ CD38br) are associated with significant risk factors for MI, AF, and IS (Figure S1 and Table S5).

Based on the reverse MR analysis of positive immune cells, we assessed the causal relationship between CVD and positive immune cells (Table S6). CVD, such as CAD, MI, AF, and IS, were considered the exposure variables, while positive immune cells were treated as the outcome variables. The results suggest that there may be no causal relationship between CVD and the risk associated with positive immune cells.

Sensitivity analysis including heterogeneity and pleiotropy test indicated the positive SNPs exhibit neither heterogeneity nor horizontal pleiotropy (Table S7–8). The forest plot, scatter plot, funnel plot, and leave-one-out analysis of identified immune cell traits with causal effect on CVD were presented in Figure S2.

### Causal Relationships between Lipid Profiles and CVDs

**Figure 3:**
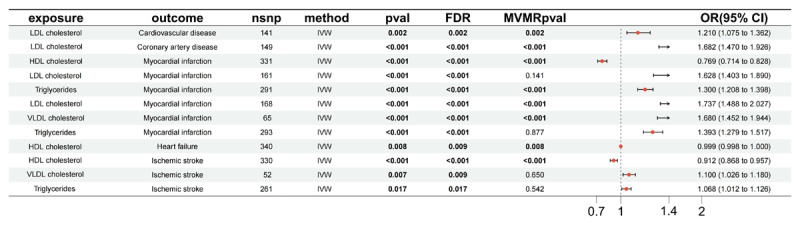
Forest plot summarizing MR analysis results for the association between lipid profiles (exposure) and various cardiovascular outcomes.

Dyslipidemia, marked by high LDL, VLDL, and triglycerides and low HDL, increases CVD risk through arterial plaque buildup. To further examine the causal relationships between lipid profiles and CVD, MR analysis was conducted, with detailed results available in Supplementary Table S9. The analysis adhered to three criteria: application of the IVW method, a significance threshold of P < 0.05, and pleiotropy tests with P > 0.05. Key findings indicated that HDL cholesterol plays a protective role against MI, HF and IS, while elevated LDL cholesterol, VLDL cholesterol, and triglycerides act as risk factors. These results highlight the distinct roles lipid profiles play in cardiovascular health, with more detailed relationships illustrated in Figure 3 and Table S10.

Supplementary Table S11–12 is the sensitivity analysis results. The forest plot, scatter plot, funnel plot and leave-one-out analysis of identified lipid profiles traits with causal effect on CVD were presented in Supplementary Figure S3.

### Role of positive immune cells in lipid profiles

**Figure 4:**
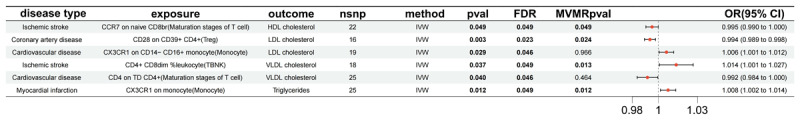
Forest plot of MR analysis showing associations between immune cell phenotypes (exposure) and lipid profiles (outcome) for different cardiovascular diseases.

To evaluate the causal relationships between specific positive immune cell subtypes and lipid profiles, MR analyses were conducted (Table S13). IVW results indicated that increased CCR7 on naive CD8br (maturation stage of T cells) is associated with decreased HDL cholesterol (OR = 0.995, 95% CI = 0.990–1.000, P = 0.049). Increased CD28 on CD39^+^ CD4^+^ (Tregs) correlates with reduced LDL cholesterol, suggesting a protective effect. Conversely, CX3CR1 on CD14^–^ CD16^+^ monocytes is linked to higher LDL cholesterol, while increased CD4+ CD8dim %leukocyte (TBNK) is associated with increased VLDL cholesterol, and increased CD4 on TD CD4^+^ (another T cell maturation stage) is linked to reduced VLDL cholesterol. CX3CR1 on monocytes is also associated with higher triglycerides, suggesting a potential risk factor (Figure 4). These findings emphasize how immune cell subtypes may impact lipid metabolism and inform potential targeted therapies, with further details provided in Table S14.

### Analysis of immune cells’ influence on cardiovascular disease via lipid profiles

**Figure 5:**
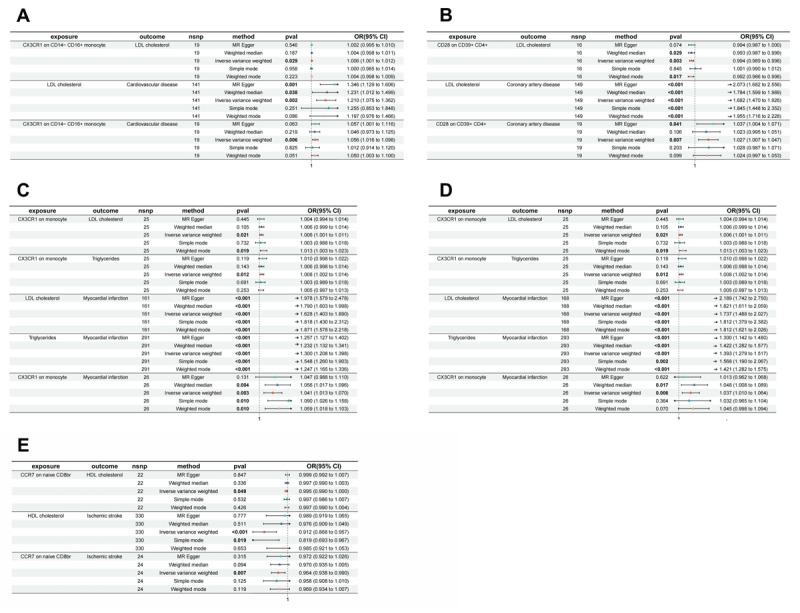
Forest plot presenting MR results for associations between immune cell phenotypes and lipid profiles and cardiovascular diseases. Panels **A-E** show odds ratios (OR) and 95% confidence intervals (CI) from multiple MR methods (MR-Egger, weighted median, inverse variance weighted, simple mode, weighted mode). Asterisks indicate significance. **(A)** CX3CR1 on CD14^+^CD16^+^ monocytes: LDL cholesterol, cardiovascular disease. **(B)** CD28 on CD39^+^ CD4^+^ T cells: LDL cholesterol, coronary artery disease. **(C)** CX3CR1 on monocytes: LDL cholesterol, triglycerides, myocardial infarction (ebi-a-GCST90018877). **(D)** CX3CR1 on monocytes: LDL cholesterol, triglycerides, myocardial infarction (ebi-a-GCST90018877). **(E)** CCR7 on naive CD8^+^ T cells: HDL cholesterol, ischemic stroke. nsnp = number of SNPs. Error bars represent 95% CI.

Based on the results of the analysis, the mediation effects of lipid traits on the immune cell traits causally associated with cardiovascular and cerebrovascular diseases were evaluated. The results presented in the mediation analysis table indicate that the β value of the mediation effect ranged from –0.003 to 0.003, and the mediated proportion varied from –12.8% to 9.2% (Table 2). The risk causal effects of CX3CR1 on CD14^–^ CD16^+^ monocytes on CVD are mediated by LDL cholesterol, with a small mediation effect of β = 0.001 and a mediation proportion of 2.2%, indicating a partial role of LDL cholesterol in this pathway (Figure 5A). For CAD, CD28 on CD39^+^ CD4^+^ T cells showed a complex effect through LDL cholesterol. The mediation effect was negative (β = –0.003), and the mediated proportion was –12.8%, suggesting that the lipid mediation might counteract the direct effect of CD28 expression (Figure 5B). If we perform a more stringent analysis, considering only the results with significant mediation effects, CX3CR1 on monocytes consistently demonstrates a risk causal effect on MI through LDL cholesterol and triglycerides, with a mediation effect of β = 0.00 and a mediation proportion of 7.26% and 9.2%, respectively. These mediation effects indicate that both LDL cholesterol and triglycerides act as significant mediators in the causal pathway linking CX3CR1 expression on monocytes to MI, underscoring the role of lipid traits in this immune-disease relationship (Figure 5C-D). Based on the analysis of direct and total effects, the expression of CCR7 on naive CD8br T cells is negatively correlated with ischemic stroke (total effect β = –0.037, 95% CI: –0.064 to –0.010), with both direct effects A and B being negative, suggesting a potential protective role. Although the mediation effect is positive (β = 0.001), the mediation proportion is negative (–1.23%), which may indicate that HDL cholesterol plays a complex role in the overall causal pathway (Figure 5E).

**Table 2:** Mediation analysis of the causal effects of immune cell traits on cardiovascular disease via blood lipid traits.


TRAITS OF IMMUNE CELL	TRAITS OF BLOOD LIPID	TRAITS OF DISEASE	TOTAL EFFECT	DIRECT EFFECT A	DIRECT EFFECT B	MEDIATION EFFECT	MEDIATED PROPORTION (%)

			β(95% CI)	β(95% CI)	β(95% CI)	β(95% CI)	

CX3CR1 on CD 14- CD 16+ monocyte (Monocyte)	LDL cholesterol	Cardiovascular disease	0.054 (0.016, 0.093)	0.006 (0.001, 0.012)	0.191 (0.072, 0.309)	0.001 (0.001, 0.002)	2.2% (0.225%, 4.17%)

CD28 on CD39+ CD4+(Treg)	LDL cholesterol	Coronary artery disease	0.027 (0.007, 0.046)	–0.007 (–0.011, –0.002)	0.520 (0.385, 0.655)	–0.003 (–0.006, –0.001)	–12.8% (–21.1%, –4.38%)

CX3CR1 on monocyte(Monocyte)	LDL cholesterol	Myocardial infarction (ebi-a-GCST90018877)	0.040 (0.013, 0.067)	0.006 (0.001, 0.011)	0.488 (0.339, 0.637)	0.003 (0.001, 0.005)	7.26% (1.11%, 13.4%)
	
	Triglycerides			0.008 (0.002, 0.013)	0.262 (0.189, 0.335)	0.003 (0.001, 0.005)	7.26% (1.11%, 13.4%)

CX3CR1 on monocyte(Monocyte)	LDL cholesterol	Myocardial infarction (ebi-a-GCST011364)	0.036 (0.010, 0.062)	0.006 (0.001, 0.011)	0.552 (0.397, 0.707)	0.003 (0.001, 0.006)	9.2% (1.4%, 17%)
	
	Triglycerides			0.008 (0.002, 0.013)	0.332 (0.246, 0.417)	0.003 (0.001, 0.006)	9.2% (1.4%, 17%)

CCR7 on naive CD8br (Maturation stages of T cell)	HDL cholesterol	Ischemic stroke	–0.037 (–0.064, –0.010)	–0.005 (–0.010, –0.001)	–0.093 (–0.142, –0.044)	0.001 (0.001, 0.001)	–1.23% (–0.003%, –2.45%)


These findings emphasize the importance of lipid traits as mediators in the relationship between immune cell activation and cardiovascular disease outcomes, suggesting potential therapeutic targets for managing cardiovascular risk through modulation of both immune and metabolic pathways.

### Functional enrichment analysis of causal effect SNPs

**Figure 6:**
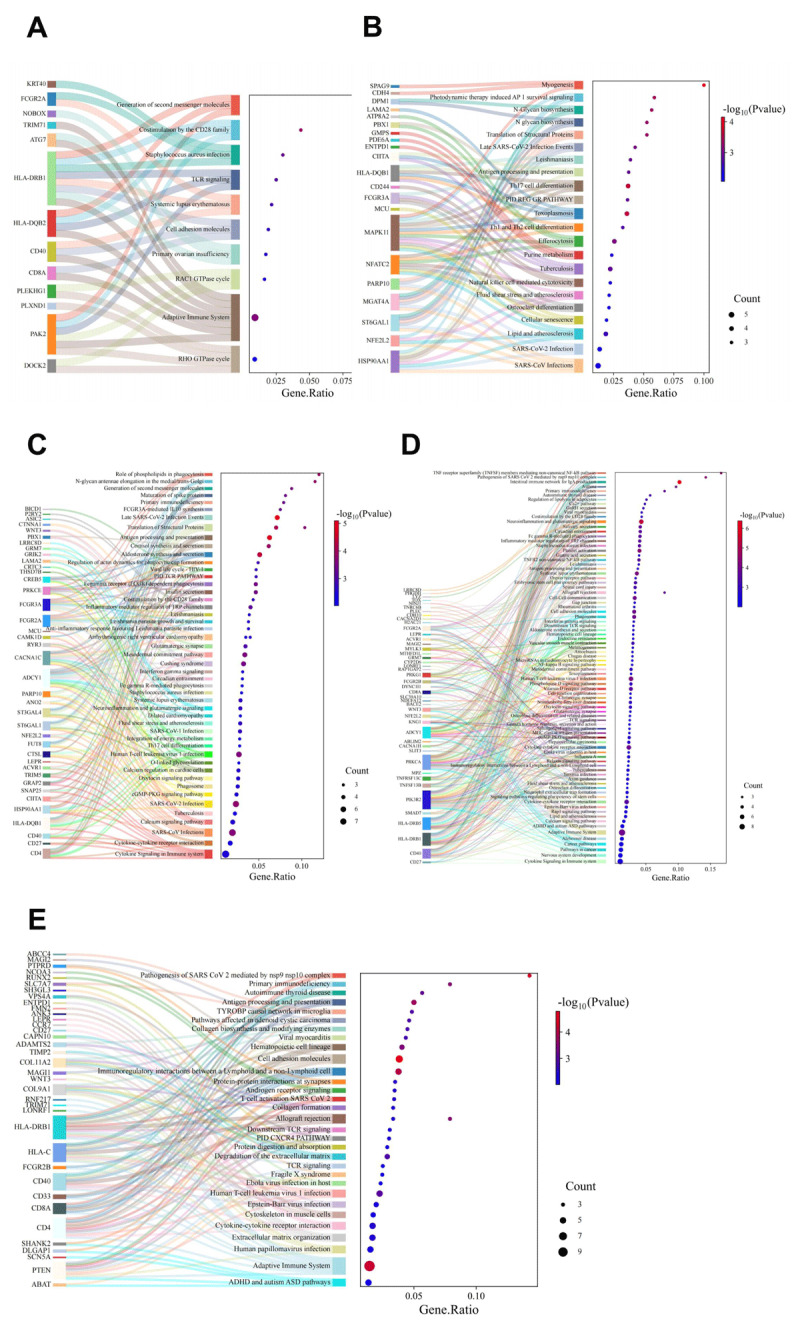
Enrichment analysis of genes linked to causal SNPs to reveal immune cell roles in cardiovascular disease. **(A–E)** Sankey diagrams and dot plots depicting the results of enrichment analysis for immune cell traits associated with CVD **(A)**, CAD **(B)**, MI **(C)**, AF **(D)**, and IS **(E)**.

To further elucidate the biological significance of immune cells in CVD, we performed enrichment analysis on genes associated with causal SNPs identified in the MR analysis, aiming to explore the involved biological pathways (Figure 6). Detailed results are provided in Tables S15–19. The analysis revealed significant enrichment in cytokine signaling pathways, particularly “Cytokine-cytokine receptor interaction” and “Cytokine signaling in the immune system” pathways in MI, AF, and IS. These findings underscore the crucial role of cytokines in mediating interactions between immune cells and cardiovascular tissues.

Additionally, our results indicated enrichment in pathways related to inflammatory and autoimmune conditions, such as “Systemic lupus erythematosus” and “Neuroinflammation and glutamatergic signaling,” which are associated with CVD and AF risks, respectively. Moreover, our analysis linked infectious disease pathways to cardiovascular conditions, including “SARS-CoV-2 infection” in CAD and “Staphylococcus aureus infection” in CVD. These findings suggest that infectious agents may impact cardiovascular health through immune-mediated mechanisms, highlighting potential genetic predispositions that could heighten immune responses and increase cardiovascular risk.

## Discussion

Our study provides a comprehensive overview of the causal associations between immune cells and CVD, including CAD, MI, AF and IS. By utilizing MR analysis, we identified key immune cell subtypes that either increase or decrease the risk of these conditions, highlighting the complex and dynamic role of the immune system in cardiovascular health. These findings align with recent literature that emphasizes the intricate balance between pro-inflammatory and regulatory immune responses in cardiovascular pathogenesis (10,11).

The results underscore a dual role of the immune system in CVD, wherein specific immune cells contribute to disease progression while others provide protective effects. CD27 on unswitched memory B cells, CD28^–^negative double-negative (CD4^–^ CD8^–^) T cells, and CX3CR1 on CD14^–^ CD16^+^ monocytes were associated with increased risk, suggesting that these subtypes promote a pro-inflammatory environment. This inflammatory milieu is known to contribute to endothelial dysfunction, plaque formation, and plaque instability-key steps in the pathogenesis of atherosclerosis and other cardiovascular conditions (12,13). These immune cell subsets may play pro-inflammatory roles in cardiovascular pathology. CX3CR1, for instance, mediates leukocyte adhesion and migration, critical steps in atherogenesis and plaque formation (14). Elevated levels of CX3CR1-expressing monocytes could enhance vascular inflammation, promoting the development of atherosclerotic lesions (15).

Conversely, higher expression of CD28 on resting regulatory T cells (Tregs) and increased percentages of HLA-DR^++^ monocytes demonstrated protective effects against CVD. Tregs are pivotal in maintaining immune homeostasis and suppressing excessive inflammatory responses (16,17). The expression of CD28, a co-stimulatory molecule, on Tregs may enhance their regulatory functions, thereby mitigating inflammation and vascular damage (18). Similarly, HLA-DR^++^ monocytes have been implicated in anti-inflammatory processes, which could confer protective effects against CVD (19,20). These findings are consistent with recent studies suggesting that enhancing Treg activity can reduce inflammation and stabilize atherosclerotic plaques, ultimately decreasing cardiovascular risk (21,22).

In CAD and MI, distinct immune subtypes showed divergent roles in influencing disease outcomes. The protective effect of CD24^+^ CD27^+^ B cells and HLA DR^+^ NK cells in CAD suggests that these immune cells may mitigate inflammation within atherosclerotic plaques. B cells, particularly those expressing anti-inflammatory markers, have been implicated in the production of regulatory cytokines like IL-6, which can dampen the inflammatory response and stabilize plaques (23,24,25). NK cells, on the other hand, have been shown to selectively target and eliminate activated macrophages, limiting the inflammatory process within plaques and promoting plaque stability (26,27).

Conversely, monocyte-driven inflammation, particularly involving CD45-expressing CD14^+^ monocytes and CX3CR1-expressing monocytes, was associated with an increased risk of MI. Monocytes are among the first responders to myocardial injury, and their excessive recruitment and activation can lead to adverse remodeling and impaired healing post-MI (28,29). Targeting monocyte recruitment or activation could therefore be an effective strategy to limit myocardial injury and improve outcomes (30,31).

The involvement of immune cells in AF and IS highlights the diverse mechanisms by which the immune system impacts cardiovascular health. CD25-expressing IgD^+^ CD38dim and BAFF-R on IgD^+^ CD38^–^ naive B cells demonstrated a protective effect against AF, which may indicate the involvement of B cells that help maintain immune homeostasis and prevent excessive inflammation (32). B cells produce anti-inflammatory cytokines, such as IL-10, which may reduce the inflammatory environment that predisposes individuals to AF (33). The involvement of CD64 on monocytes suggests that enhanced phagocytic capabilities may help clear pro-inflammatory stimuli, reducing AF risk (34). In contrast, CX3CR1 on CD14^–^ CD16^+^ monocytes may contribute to a pro-inflammatory state and atrial fibrosis, promoting AF. These monocytes, known for their pro-inflammatory properties, may promote atrial inflammation and remodeling (35). They are recruited to inflamed tissues, where they exacerbate local inflammation, which is critical for AF development (36). Targeting these pathways could offer a novel therapeutic approach for AF by reducing atrial fibrosis and promoting normal atrial function.

Increased expression of markers such as CD28 on resting Tregs, PDL-1 on CD14^–^ CD16^–^ cells, CD62 L- HLA DR^++^ on monocyte ACs, CCR7 on naive CD8^+^ T cells, was associated with reduced IS risk. These cells help modulate immune responses, maintain tolerance, and limit excessive inflammation, which are key in reducing stroke risk. PDL-1 play roles in immune suppression, maintaining vascular homeostasis, and preventing excessive immune activation (37,38). Increased anti-inflammatory activity by monocytes and T cells also contributes to reduced IS risk (39,40,41). Markers associated with increased IS risk point to a pro-inflammatory profile that contributes to stroke pathogenesis. Pro-inflammatory B cells can amplify inflammatory cascades, increasing blood-brain barrier permeability and risk of ischemic events (42). DN T cells are linked to immune dysregulation and promote neuroinflammation (43).

Comprehensively, enhancing Treg function may provide a therapeutic approach to reducing inflammation and stabilizing cardiovascular health. NK cells expressing SSC-A on HLA DR^+^ are known for their cytotoxic activity, which may limit inflammation and promote plaque stability. Additionally, targeting pro-inflammatory B cells may help reduce cardiovascular risk and prevent adverse outcomes.

Our reverse MR analysis aimed to assess whether CVD and other cardiovascular conditions, such as CAD, MI, AF, and IS, have a causal effect on the abundance of positive immune cells. The results indicated no significant causal relationship, suggesting that the observed associations between cardiovascular conditions and immune cell subtypes are likely due to immune modulation influencing disease risk rather than the reverse. This finding underscores the primary role of immune dysregulation in driving cardiovascular pathology, supporting the notion that modulating immune responses could be a key strategy in preventing and managing CVD, while also highlighting the potential of targeting immune cells for therapeutic interventions (44).

Abnormal blood lipid levels, known as dyslipidemia, are closely linked to CVD. Elevated levels of LDL cholesterol, VLDL cholesterol, and triglycerides increase the risk of conditions such as heart attack, heart failure, and stroke, as they contribute to plaque buildup in the arteries (atherosclerosis). Conversely, higher levels of HDL cholesterol are protective, helping to reduce this risk by removing excess cholesterol from the bloodstream. Our study highlights key lipid biomarkers for cardiovascular health. HDL cholesterol shows a protective role against MI, HF and IS, while LDL cholesterol, VLDL cholesterol, and triglycerides are risk factors. These findings align with previous studies linking HDL cholesterol to cardiovascular benefits through reverse cholesterol transport and anti-inflammatory properties (45). LDL, VLDL, and triglycerides are confirmed risk factors, consistent with their role in atherosclerosis (46,47). These results have implications for personalized medicine. Targeting LDL and triglyceride remains crucial for cardiovascular risk management, supported by advances like PCSK9 inhibitors (48,49). The evidence for HDL’s protective effect may prompt further exploration of enhancing HDL functionality.

Further MR analysis identified associations between immune cell subtypes and lipid profiles, suggesting a complex interplay between immune function and lipid metabolism with implications for cardiovascular risk. Increased CCR7 expression on naive CD8^+^ T cells was linked to lower HDL cholesterol, highlighting the need for further research on how naive T cell differentiation influences HDL metabolism. Similarly, increased CD28 expression on CD39^+^ CD4^+^ regulatory T cells (Tregs) was associated with lower LDL cholesterol, suggesting a protective role. Given that Tregs reduce inflammation, their activity may improve lipid metabolism and lower LDL levels (50). Enhancing Treg function could offer a strategy for managing hyperlipidemia. CD4^+^ CD8dim T cells appeared to contribute to VLDL production, while terminally differentiated T cells supported lipid balance. CX3CR1 expression on CD14^–^ CD16^+^ monocytes was linked to elevated LDL cholesterol, suggesting their role in lipid accumulation within atherosclerotic plaques and subsequent disease progression (51). Furthermore, this expression was associated with elevated triglycerides, highlighting the connection between inflammation and dyslipidemia. Targeting CX3CR1-expressing monocytes may help mitigate atherosclerosis, as chronic inflammation from these cells likely exacerbates dyslipidemia and increases cardiovascular risk.

The mediation analysis highlights the role of lipid traits in linking immune cell activation to cardiovascular and cerebrovascular diseases. Lipid traits, particularly LDL cholesterol and triglycerides, significantly mediate the effects of immune cells on cardiovascular outcomes. CX3CR1 on monocytes showed a partial mediation effect on CVD risk through LDL cholesterol, indicating lipid metabolism’s role in the immune-disease link. The relationship between CD28 on T cells and CAD through LDL cholesterol suggests a complex interaction. For CX3CR1 on monocytes and MI, LDL cholesterol and triglycerides showed significant mediation, underscoring the importance of these lipid markers. These findings highlight an immuno-lipid connection in MI. While the partial increase in MI risk via LDL and triglycerides suggests that lipid-lowering is crucial, targeting immune pathways may offer additional benefits. Further mechanisms remain unknown, necessitating broader strategies for MI prevention. CCR7 on naive T cells showed a protective effect against IS, with HDL cholesterol playing a complex role that could this weaken the protection. Recent studies have similarly demonstrated that Tregs can play both protective and pathogenic roles in CVD (52), emphasizing the complexity of these interactions and the need for further research. Overall, these findings emphasize lipid traits as key mediators in the immune-cardiovascular relationship and suggest potential therapeutic targets by modulating immune responses and lipid metabolism. Further research should investigate how regulatory immune cells modulate lipid metabolism and the implications for cardiovascular risk management.

The enrichment analysis provides insights into the role of immune cells in CVD progression. By examining genes associated with causal SNPs from MR analysis, we identified key roles for immune signaling in cardiovascular health.

We observed significant enrichment in cytokine signaling pathways across MI, AF, and IS, highlighting the importance of cytokines in mediating immune cell interactions with cardiovascular tissues. This enrichment suggests that targeting cytokine signaling could be a potential strategy for modulating cardiovascular outcomes, as supported by recent studies emphasizing the role of cytokine networks in inflammation and immune responses (53).

We also found enrichment in pathways related to inflammatory and autoimmune conditions, which are linked to increased risks of CVD and AF, indicating that chronic inflammation can heighten cardiovascular risk (54). Furthermore, our analysis linked infectious disease pathways to cardiovascular conditions, suggesting that infectious agents may influence cardiovascular health through immune-mediated mechanisms. Recent evidence shows that infections, particularly COVID-19, can exacerbate cardiovascular complications through immune activation and endothelial dysfunction (55,56). Overall, our findings highlight the complex role of immune-mediated mechanisms, including cytokine signaling, autoimmune pathways, and infectious agents, as significant contributors to cardiovascular risk.

## Conclusion and Future Directions

Our study underscores the complexity of immune regulation in CVD, revealing both protective and harmful roles of various immune cell subtypes. Regulatory immune cells like Tregs show the protective effects, indicating their potential as therapeutic targets. Enhancing Treg function could reduce inflammation and stabilize plaques, offering a promising approach to lower cardiovascular events. Furthermore, our findings suggest that modulating immune cells affecting lipid metabolism may effectively reduce cardiovascular risk.

Future research should explore how these immune cells influence cardiovascular outcomes and assess their viability as therapeutic targets. Incorporating multi-omics approaches, such as single-cell RNA sequencing and proteomics, could yield deeper insights into the pathways and immune cell subtypes involved, leading to more targeted treatments. Advances in immunotherapy and lipid-targeted therapies offer exciting opportunities for clinical application, potentially paving the way for novel strategies to reduce cardiovascular morbidity and mortality. Targeting immune modulation appears to be a promising strategy for managing CVD.

## Data Accessibility Statement

All the data supporting the findings of this study are included in this article and its supporting information.

## Additional Files

The additional files for this article can be found as follows:

10.5334/gh.1444.s1 Supplementary Figures. Figure S1. Venn diagrams showing the overlap of significant immune cell traits associated with different cardiovascular outcomes.
A. Overlap between myocardial infarction (MI) and coronary artery disease (CAD).
B. Overlap between ischemic stroke (IS) and cardiovascular disease (CVD).
C. Overlap among MI, atrial fibrillation (AF), and IS.
D. Overlap among CAD, CVD, IS, MI, and AF.

10.5334/gh.1444.s2 Supplementary Figures. Figure S2. The forest plot, scatter plot, funnel plot, and leave-one-out analysis of identified immune cell traits with causal effect on different cardiovascular diseases.

10.5334/gh.1444.s3 Supplementary Figures. Figure S3. The forest plot, scatter plot, funnel plot, and leave-one-out analysis of identified lipid profiles traits with causal effect on different cardiovascular diseases.

10.5334/gh.1444.s4 Supplementary Tables. Table S1. More information about genome-wide association studies (GWASs) of 731 immune cells.

10.5334/gh.1444.s5 Supplementary Tables. Table S2. Genetic instrumental variables (IVs) for immune cells. More information about GWASs for 91 inflammatory proteins.

10.5334/gh.1444.s6 Supplementary Tables. Table S3. Mendelian randomization (MR) analysis of immune cells and cardiovascular disease. Genetic instrumental variables (IVs) for lipidomes.

10.5334/gh.1444.s7 Supplementary Tables. Table S4. Details of immune cells associated with cardiovascular disease.

10.5334/gh.1444.s8 Supplementary Tables. Table S5. Overlap of immune cell traits associated with cardiovascular disease outcomes.

10.5334/gh.1444.s9 Supplementary Tables. Table S6. Reverse MR analysis assessing causal relationships between cardiovascular diseases and positive immune cells.

10.5334/gh.1444.s10 Supplementary Tables. Table S7–8. Heterogeneity and pleiotropy test of immune cells and cardiovascular diseases.

10.5334/gh.1444.s11 Supplementary Tables. Table S9. MR analysis of lipid profiles and cardiovascular diseases.

10.5334/gh.1444.s12 Supplementary Tables. Table S10. Details of positive lipid profiles associated with cardiovascular disease.

10.5334/gh.1444.s13 Supplementary Tables. Table S11–12. Heterogeneity and pleiotropy test of immune cells and cardiovascular diseases.

10.5334/gh.1444.s14 Supplementary Tables. Table S13. MR estimates of positive immune cells and lipid profiles.

10.5334/gh.1444.s15 Supplementary Tables. Table S14. Details of lipid-related positive immune cells.

10.5334/gh.1444.s16 Supplementary Tables. Table S15–19. Enrichment analysis of immune cell-related SNPs in cardiovascular disease, coronary artery disease, myocardial infarction, atrial fibrillation and ischemic stroke, respectively.
