# Supplementary figures and images for: Unraveling the Causal Links Between Immune Cells, Lipids, and Cardiovascular Diseases: Insights from Mendelian Randomization
Source: Glob Heart. 2025 Jul 3;20(1):57. doi: 10.5334/gh.1444 (PMC12227087; doi:10.5334/gh.1444)

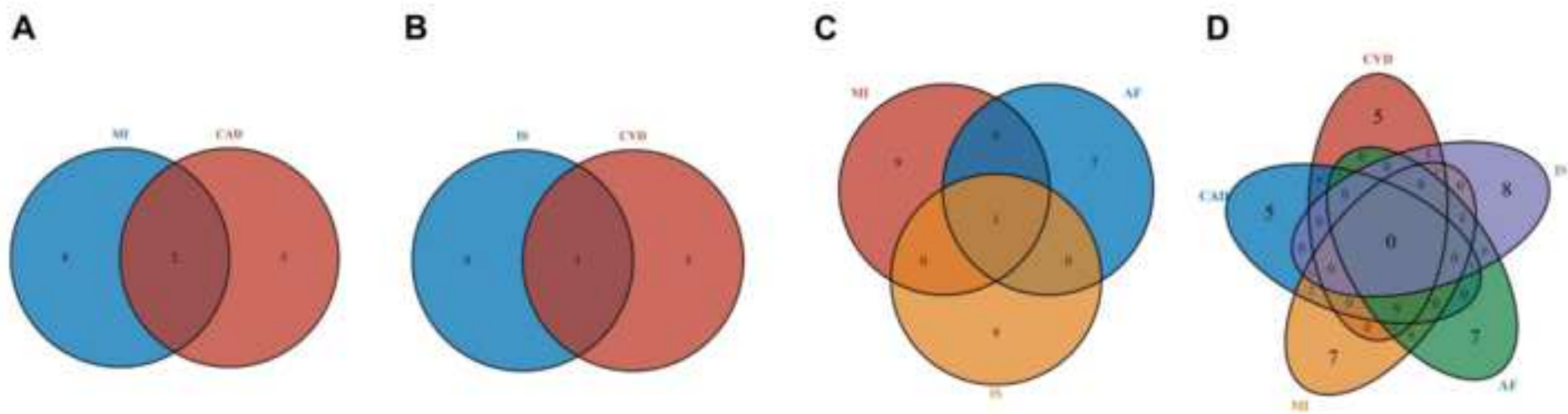

Supplement: Supplementary Figures. Figure S1. — Venn diagrams showing the overlap of significant immune cell traits associated with different cardiovascular outcomes. A. Overlap between myocardial infarction (MI) and coronary artery disease (CAD). B. Overlap between ischemic stroke (IS) and cardiovascular disease (CVD). C. Overlap among MI, atrial fibrillation (AF), and IS. D. Overlap among CAD, CVD, IS, MI, and AF. [file gh-20-1-1444-s1.pdf]
