# Supplementary material for: Unraveling the Causal Links Between Immune Cells, Lipids, and Cardiovascular Diseases: Insights from Mendelian Randomization
Source: Glob Heart. 2025 Jul 3;20(1):57. doi: 10.5334/gh.1444 (PMC12227087; doi:10.5334/gh.1444)
Supplement: Supplementary Figures. Figure S3. — The forest plot, scatter plot, funnel plot, and leave-one-out analysis of identified lipid profiles traits with causal effect on different cardiovascular diseases. [file gh-20-1-1444-s3.pdf]

Figure S3

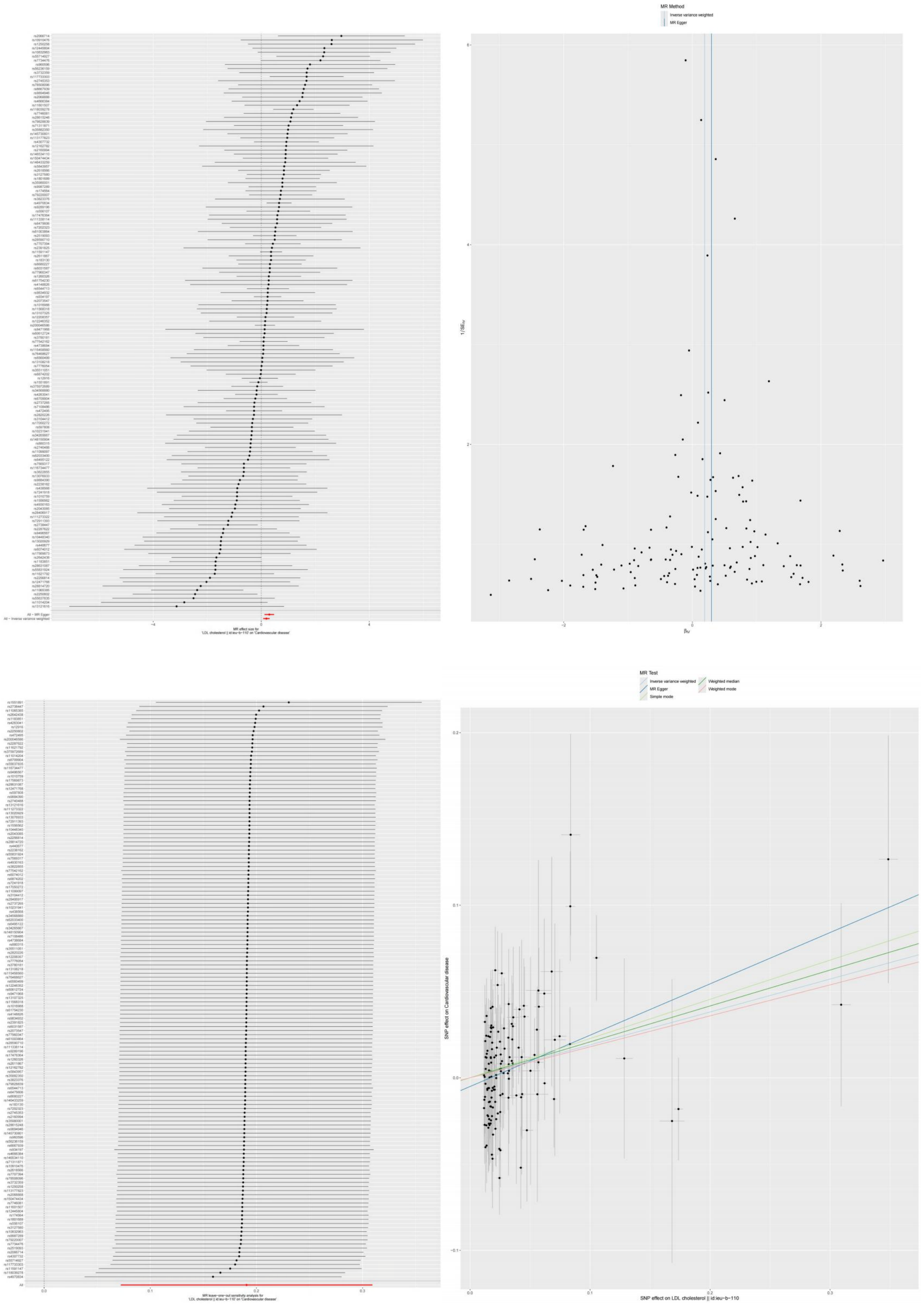

The forest plot, scatter plot, funnel plot and leave- one-out analysis of identified LDL cholesterol traits with causal effect on Cardiovascular disease.

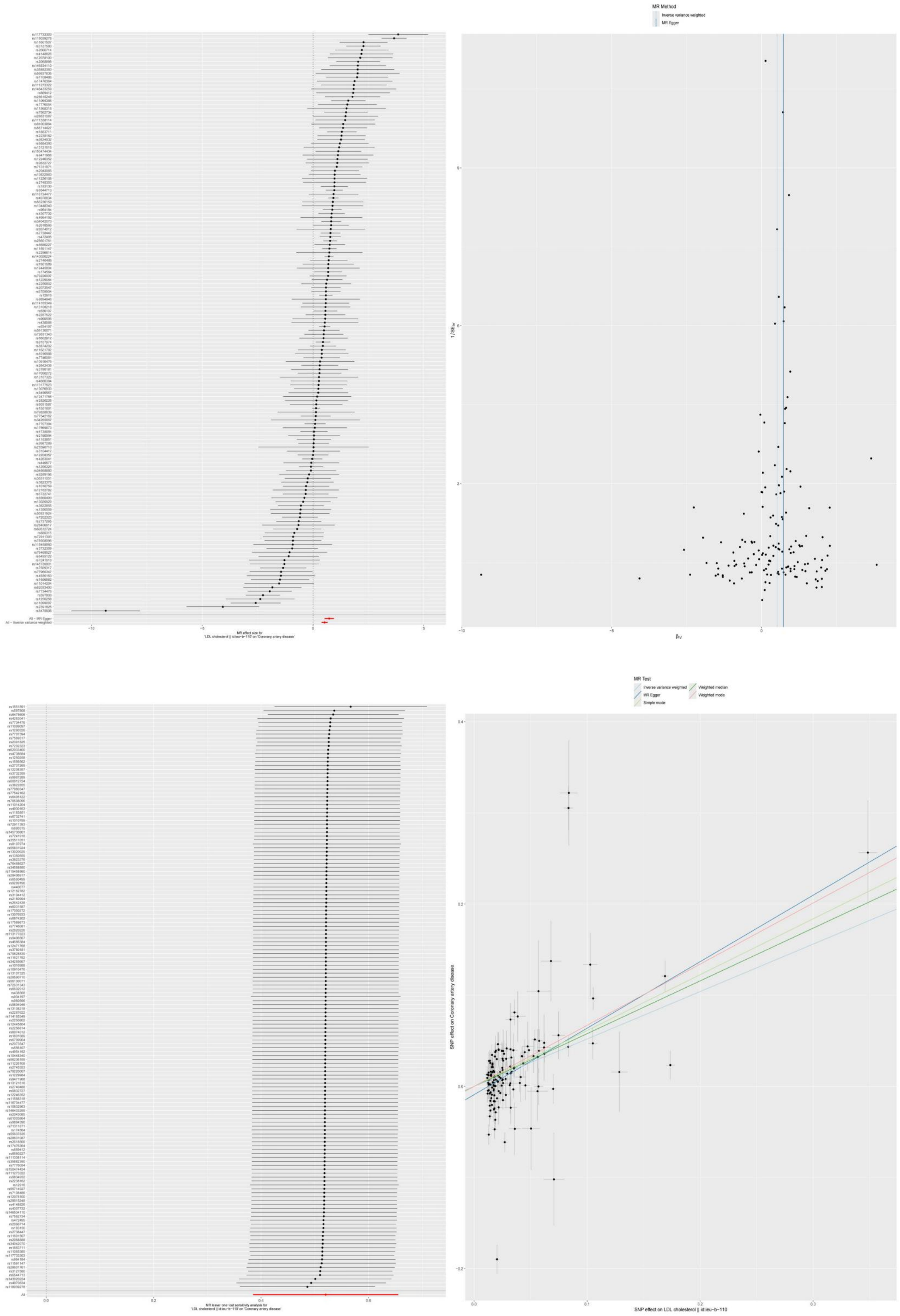

The forest plot, scatter plot, funnel plot and leave- one-out analysis of identified LDL cholesterol traits with causal effect on Coronary artery disease.

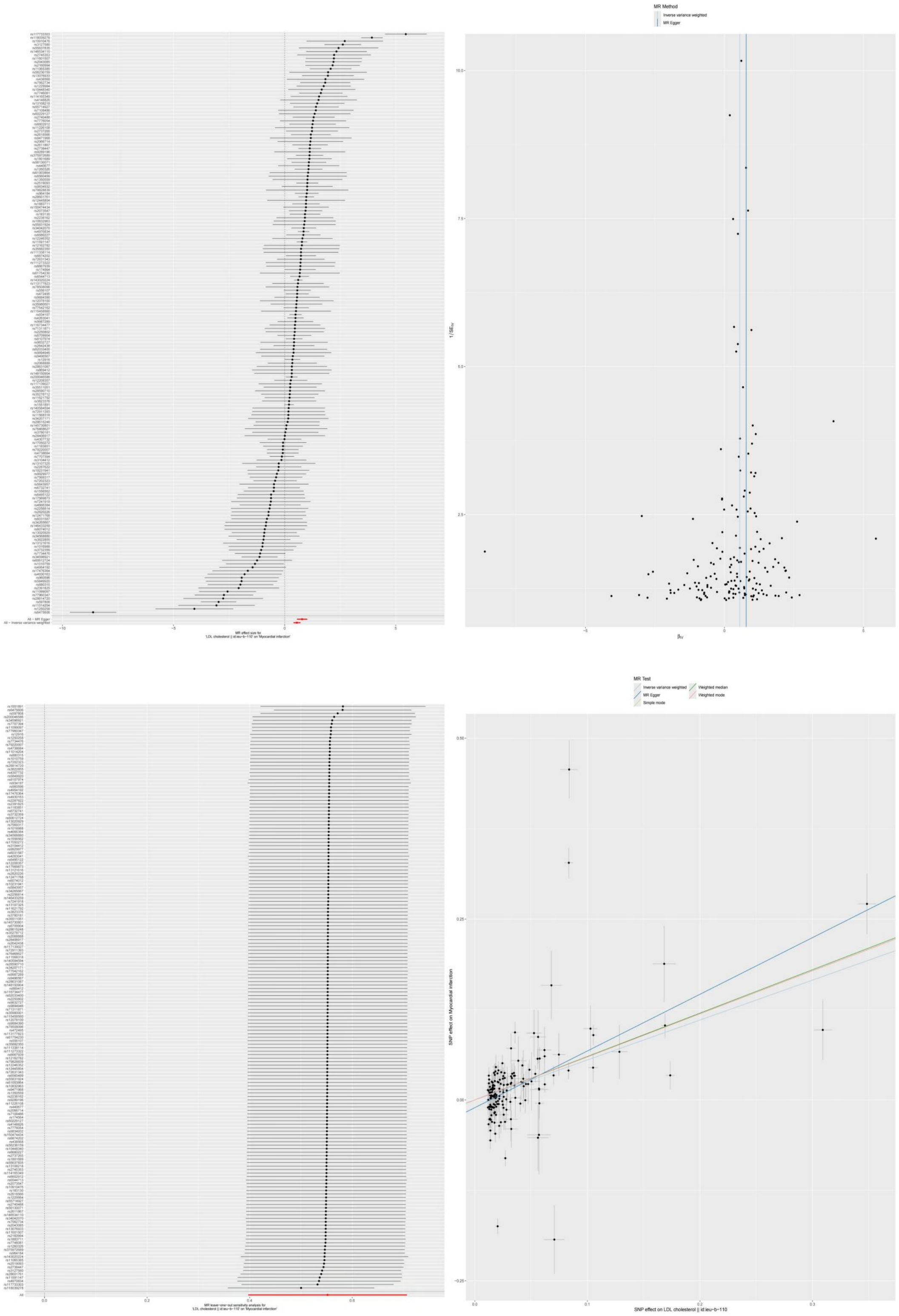

The forest plot, scatter plot, funnel plot and leave- one-out analysis of identified LDL cholesterol traits with causal effect on Myocardial infarction(ebi-a-GCST90018877).

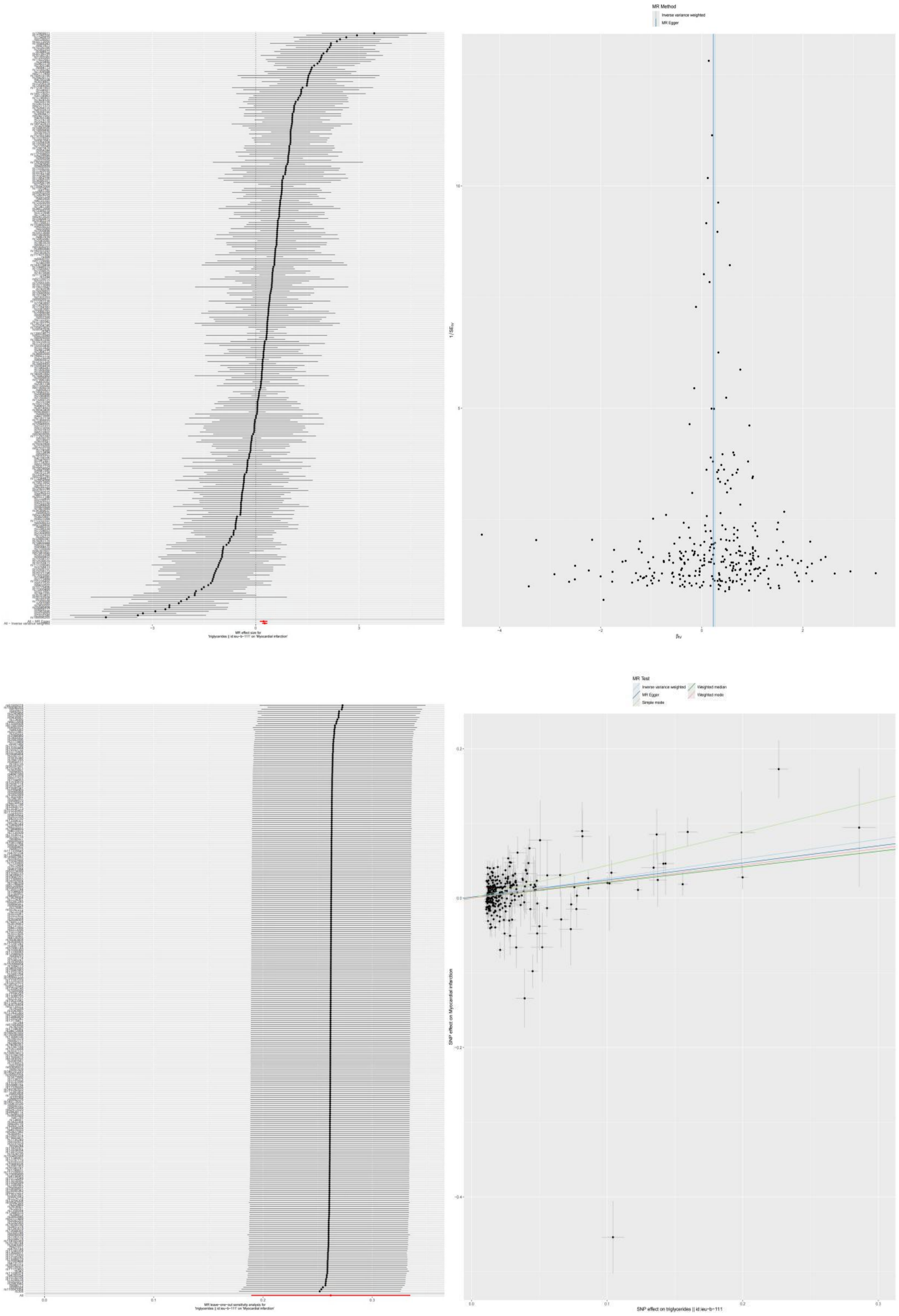

The forest plot, scatter plot, funnel plot and leave- one-out analysis of identified Triglycerides traits with causal effect on Myocardial infarction(ebi-a-GCST90018877).

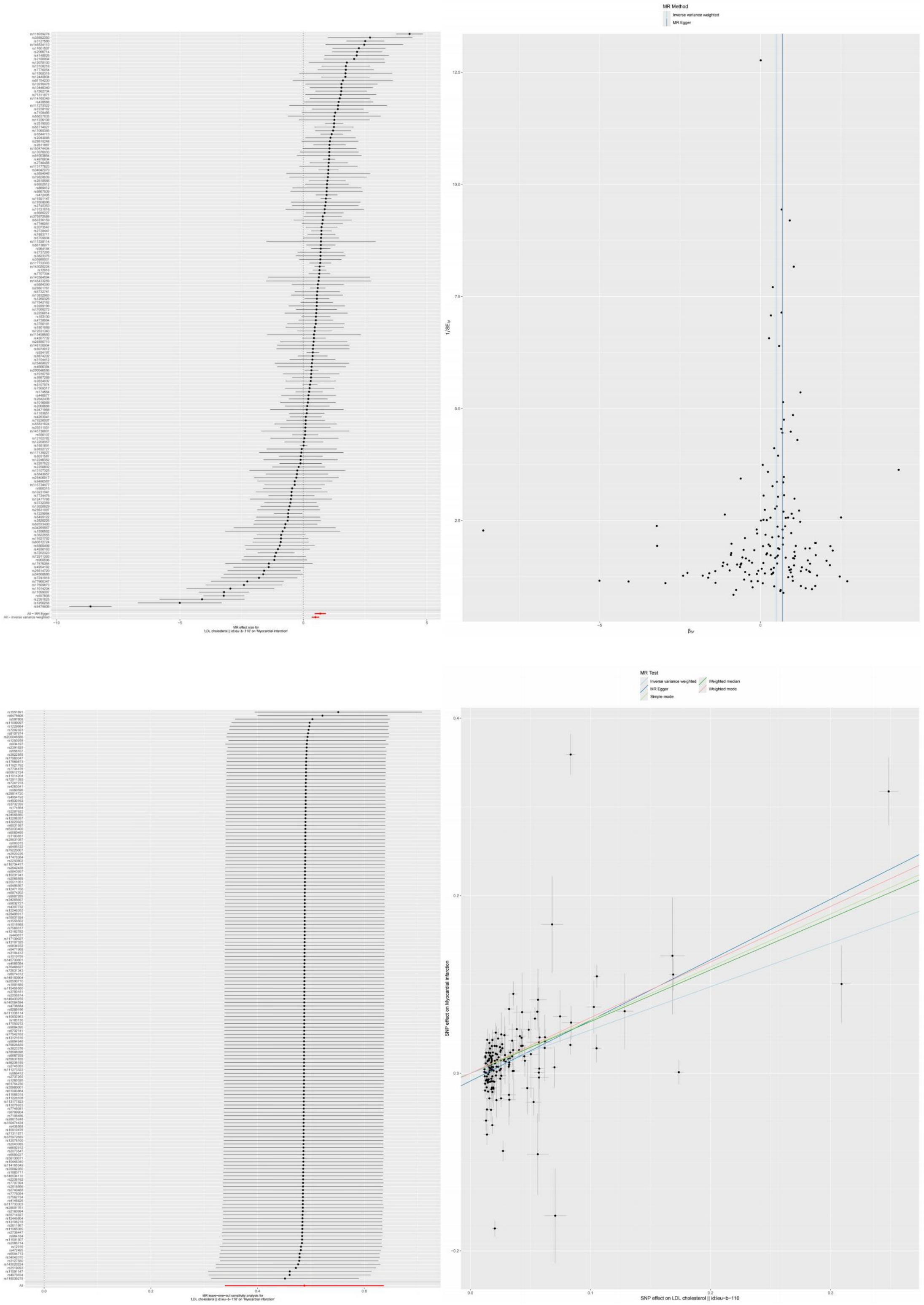

The forest plot, scatter plot, funnel plot and leave- one-out analysis of identified LDL cholesterol traits with causal effect on Myocardial infarction(ebi-a-GCST011364).

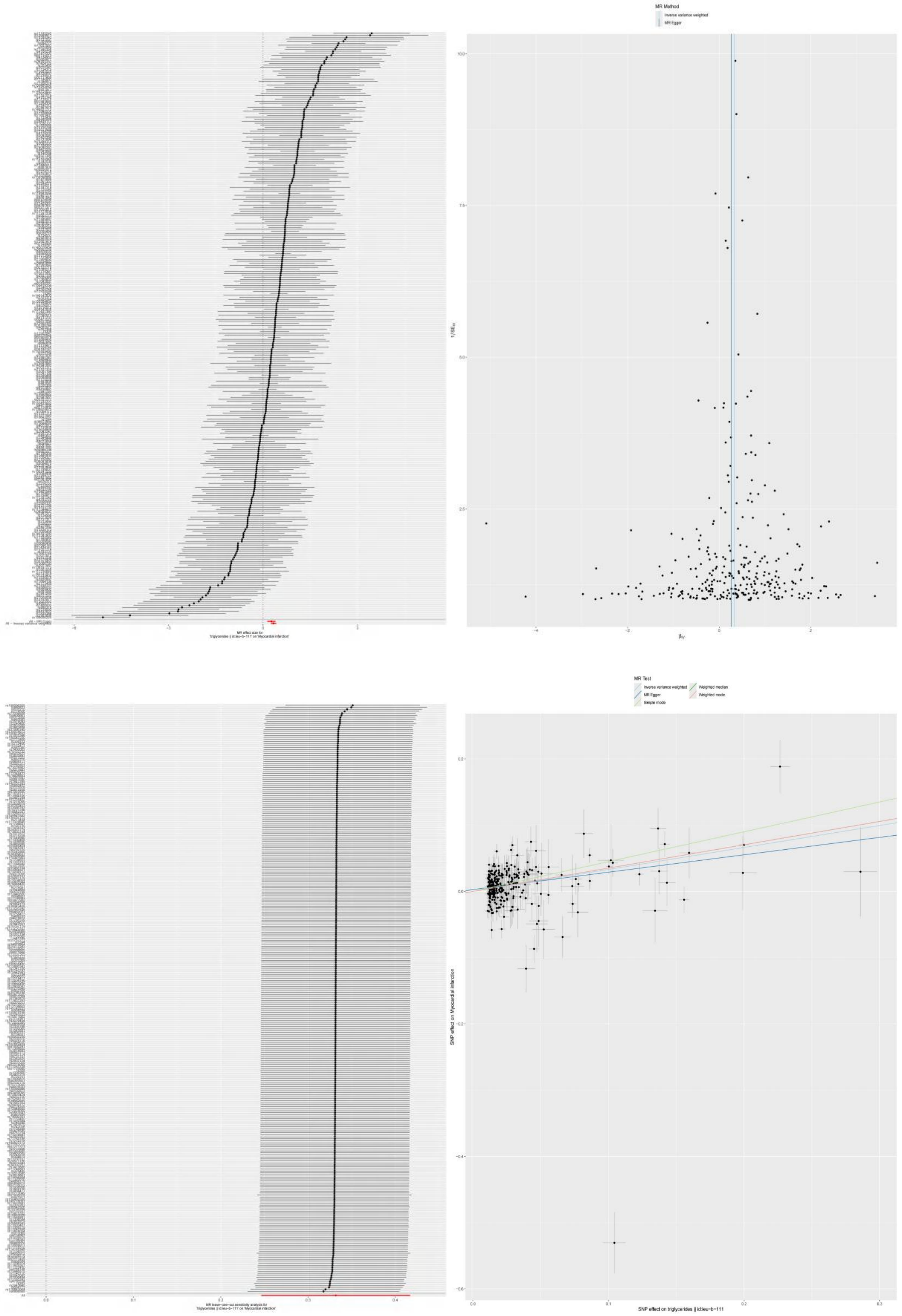

The forest plot, scatter plot, funnel plot and leave- one-out analysis of identified Triglycerides traits with causal effect on Myocardial infarction(ebi-a-GCST011364).

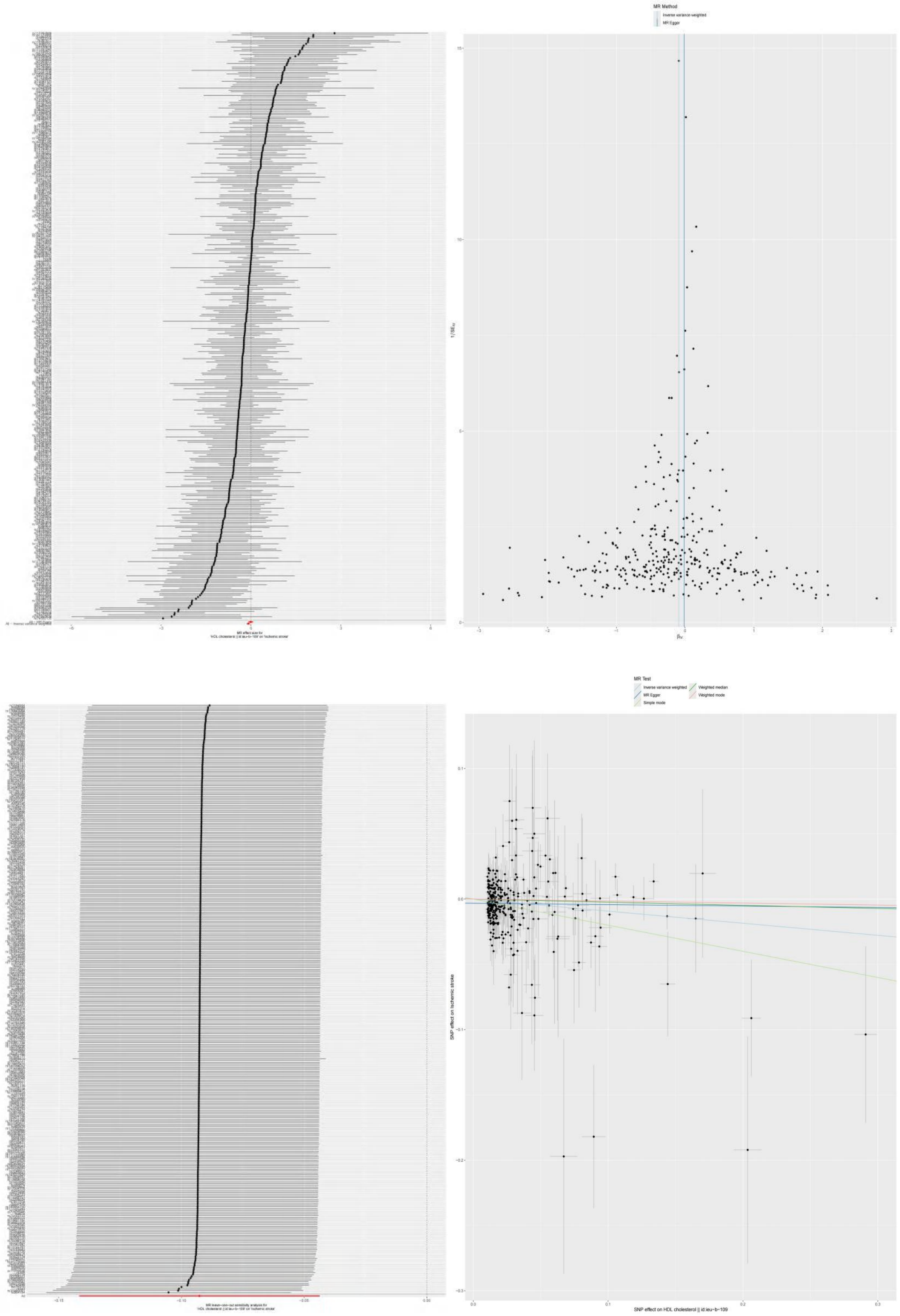

The forest plot, scatter plot, funnel plot and leave- one-out analysis of identified HDL cholesterol traits with causal effect on Ischemic stroke.
