# Supplementary material for: Unraveling the Causal Links Between Immune Cells, Lipids, and Cardiovascular Diseases: Insights from Mendelian Randomization
Source: Glob Heart. 2025 Jul 3;20(1):57. doi: 10.5334/gh.1444 (PMC12227087; doi:10.5334/gh.1444)
Supplement: Supplementary Tables. Table S1. — More information about genome-wide association studies (GWASs) of 731 immune cells. [file gh-20-1-1444-s4.pdf]

Details on Genome-Wide Association Studies (GWAS) of 73

ID	CatalogID	reportedTrait
ebi-a-GCST90001391	GCST90001391	IgD+ %B cell
ebi-a-GCST90001392	GCST90001392	IgD+ CD38br AC
ebi-a-GCST90001393	GCST90001393	IgD+ CD38dim %B cell
ebi-a-GCST90001394	GCST90001394	IgD+ CD38dim AC
ebi-a-GCST90001395	GCST90001395	IgD+ CD38- %B cell
ebi-a-GCST90001396	GCST90001396	IgD+ CD38- AC
ebi-a-GCST90001397	GCST90001397	Unsw mem %B cell
ebi-a-GCST90001398	GCST90001398	Unsw mem AC
ebi-a-GCST90001399	GCST90001399	IgD- CD27- %B cell
ebi-a-GCST90001400	GCST90001400	IgD+ AC
ebi-a-GCST90001401	GCST90001401	IgD- CD27- AC
ebi-a-GCST90001402	GCST90001402	Sw mem %B cell
ebi-a-GCST90001403	GCST90001403	Sw mem AC
ebi-a-GCST90001404	GCST90001404	PB/PC %B cell
ebi-a-GCST90001405	GCST90001405	PB/PC AC
ebi-a-GCST90001406	GCST90001406	Memory B cell %B cell
ebi-a-GCST90001407	GCST90001407	Memory B cell AC
ebi-a-GCST90001408	GCST90001408	Naive-mature B cell %B cell
ebi-a-GCST90001409	GCST90001409	Naive-mature B cell AC
ebi-a-GCST90001410	GCST90001410	IgD- CD38br %B cell
ebi-a-GCST90001411	GCST90001411	IgD+ CD24+ %B cell
ebi-a-GCST90001412	GCST90001412	IgD+ CD24+ AC
ebi-a-GCST90001413	GCST90001413	IgD- CD24- %B cell
ebi-a-GCST90001414	GCST90001414	IgD- CD24- AC
ebi-a-GCST90001415	GCST90001415	IgD+ CD24- %B cell
ebi-a-GCST90001416	GCST90001416	IgD+ CD24- AC
ebi-a-GCST90001417	GCST90001417	CD24+ CD27+ %B cell
ebi-a-GCST90001418	GCST90001418	CD24+ CD27+ AC
ebi-a-GCST90001419	GCST90001419	CD20- %B cell
ebi-a-GCST90001420	GCST90001420	IgD- CD38br AC
ebi-a-GCST90001421	GCST90001421	CD20- AC
ebi-a-GCST90001422	GCST90001422	CD20- CD38- %B cell
ebi-a-GCST90001423	GCST90001423	CD20- CD38- AC
ebi-a-GCST90001424	GCST90001424	IgD+ %Lymphocyte
ebi-a-GCST90001425	GCST90001425	IgD- CD38br %lymphocyte
ebi-a-GCST90001426	GCST90001426	IgD- CD38dim %lymphocyte
ebi-a-GCST90001427	GCST90001427	IgD- CD38- %lymphocyte
ebi-a-GCST90001428	GCST90001428	IgD- CD38dim %B cell
ebi-a-GCST90001429	GCST90001429	IgD+ CD38br %lymphocyte
ebi-a-GCST90001430	GCST90001430	IgD+ CD38dim %lymphocyte
ebi-a-GCST90001431	GCST90001431	IgD+ CD38- %lymphocyte
ebi-a-GCST90001432	GCST90001432	Unsw Mem %lymphocyte
ebi-a-GCST90001433	GCST90001433	IgD- CD27- %lymphocyte
ebi-a-GCST90001434	GCST90001434	Sw mem %lymphocyte
ebi-a-GCST90001435	GCST90001435	PB/PC %lymphocyte

ebi-a-GCST90001436	GCST90001436	Memory B cell %lymphocyte
ebi-a-GCST90001437	GCST90001437	Naive-mature B cell %lymphocyte
ebi-a-GCST90001438	GCST90001438	IgD- CD38dim AC
ebi-a-GCST90001439	GCST90001439	IgD+ CD24+ %lymphocyte
ebi-a-GCST90001440	GCST90001440	IgD- CD24- %lymphocyte
ebi-a-GCST90001441	GCST90001441	IgD+ CD24- %lymphocyte
ebi-a-GCST90001442	GCST90001442	CD24+ CD27+ %lymphocyte
ebi-a-GCST90001443	GCST90001443	CD20- %lymphocyte
ebi-a-GCST90001444	GCST90001444	CD20- CD38- %lymphocyte
ebi-a-GCST90001445	GCST90001445	IgD- CD38- %B cell
ebi-a-GCST90001446	GCST90001446	IgD- CD38- AC
ebi-a-GCST90001447	GCST90001447	IgD+ CD38br %B cell
ebi-a-GCST90001448	GCST90001448	CD11c+ monocyte AC
ebi-a-GCST90001449	GCST90001449	CD11c+ monocyte %monocyte
ebi-a-GCST90001450	GCST90001450	CD62L- monocyte AC
ebi-a-GCST90001451	GCST90001451	CD62L- monocyte %monocyte
ebi-a-GCST90001452	GCST90001452	CD11c+ CD62L- monocyte AC
ebi-a-GCST90001453	GCST90001453	CD11c+ CD62L- monocyte %monocyt
ebi-a-GCST90001454	GCST90001454	CD62L- HLA DR++ monocyte AC
ebi-a-GCST90001455	GCST90001455	CD62L- HLA DR++ monocyte %monc
ebi-a-GCST90001456	GCST90001456	CD11c+ HLA DR++ monocyte AC
ebi-a-GCST90001457	GCST90001457	CD11c+ HLA DR++ monocyte %monc
ebi-a-GCST90001458	GCST90001458	Myeloid DC AC
ebi-a-GCST90001459	GCST90001459	Myeloid DC %DC
ebi-a-GCST90001460	GCST90001460	Plasmacytoid DC AC
ebi-a-GCST90001461	GCST90001461	DC AC
ebi-a-GCST90001462	GCST90001462	CD62L- DC AC
ebi-a-GCST90001463	GCST90001463	CD62L- DC %DC
ebi-a-GCST90001464	GCST90001464	CD86+ myeloid DC AC
ebi-a-GCST90001465	GCST90001465	CD86+ myeloid DC %DC
ebi-a-GCST90001466	GCST90001466	CD86+ plasmacytoid DC AC
ebi-a-GCST90001467	GCST90001467	CD86+ plasmacytoid DC %DC
ebi-a-GCST90001468	GCST90001468	CD62L- myeloid DC AC
ebi-a-GCST90001469	GCST90001469	CD62L- myeloid DC %DC
ebi-a-GCST90001470	GCST90001470	CD62L- plasmacytoid DC AC
ebi-a-GCST90001471	GCST90001471	CD62L- plasmacytoid DC %DC
ebi-a-GCST90001472	GCST90001472	CD62L- CD86+ myeloid DC AC
ebi-a-GCST90001473	GCST90001473	CD62L- CD86+ myeloid DC %DC
ebi-a-GCST90001474	GCST90001474	Plasmacytoid DC %DC
ebi-a-GCST90001475	GCST90001475	HLA DR++ monocyte %monocyte
ebi-a-GCST90001476	GCST90001476	HLA DR++ monocyte %leukocyte
ebi-a-GCST90001477	GCST90001477	HLA DR++ monocyte AC
ebi-a-GCST90001478	GCST90001478	CD4 Treg %CD4
ebi-a-GCST90001479	GCST90001479	CD4 Treg %T cell
ebi-a-GCST90001480	GCST90001480	Resting Treg AC
ebi-a-GCST90001481	GCST90001481	Resting Treg % CD4 Treg
ebi-a-GCST90001482	GCST90001482	Resting Treg %CD4
ebi-a-GCST90001483	GCST90001483	CD39+ resting Treg AC

ebi-a-GCST90001484	GCST90001484	CD39+ resting Treg %resting Treg
ebi-a-GCST90001485	GCST90001485	CD39+ resting Treg % CD4 Treg
ebi-a-GCST90001486	GCST90001486	Activated Treg AC
ebi-a-GCST90001487	GCST90001487	Activated Treg %CD4 Treg
ebi-a-GCST90001488	GCST90001488	Activated Treg %CD4
ebi-a-GCST90001489	GCST90001489	CD39+ activated Treg AC
ebi-a-GCST90001490	GCST90001490	CD39+ activated Treg %activated Treg
ebi-a-GCST90001491	GCST90001491	CD39+ activated Treg %CD4 Treg
ebi-a-GCST90001492	GCST90001492	Secreting Treg AC
ebi-a-GCST90001493	GCST90001493	Secreting Treg % CD4 Treg
ebi-a-GCST90001494	GCST90001494	Secreting Treg %CD4
ebi-a-GCST90001495	GCST90001495	CD39+ secreting Treg AC
ebi-a-GCST90001496	GCST90001496	CD39+ secreting Treg %secreting Treg
ebi-a-GCST90001497	GCST90001497	CD39+ secreting Treg %CD4 Treg
ebi-a-GCST90001498	GCST90001498	Activated & resting Treg AC
ebi-a-GCST90001499	GCST90001499	Activated & resting Treg % CD4 Treg
ebi-a-GCST90001500	GCST90001500	Activated & resting Treg %CD4+
ebi-a-GCST90001501	GCST90001501	Activated & secreting Treg AC
ebi-a-GCST90001502	GCST90001502	Activated & secreting Treg %CD4 Treg
ebi-a-GCST90001503	GCST90001503	Activated & secreting Treg %CD4+
ebi-a-GCST90001504	GCST90001504	CD25hi AC
ebi-a-GCST90001505	GCST90001505	CD25hi %CD4+
ebi-a-GCST90001506	GCST90001506	CD25hi %T cell
ebi-a-GCST90001507	GCST90001507	CD25hi CD45RA+ CD4 not Treg AC
ebi-a-GCST90001508	GCST90001508	CD25hi CD45RA+ CD4 not Treg %CI
ebi-a-GCST90001509	GCST90001509	CD25hi CD45RA+ CD4 not Treg %T
ebi-a-GCST90001510	GCST90001510	CD25hi CD45RA- CD4 not Treg AC
ebi-a-GCST90001511	GCST90001511	CD25hi CD45RA- CD4 not Treg %CD
ebi-a-GCST90001512	GCST90001512	CD25hi CD45RA- CD4 not Treg %T c
ebi-a-GCST90001513	GCST90001513	CD4 Treg AC
ebi-a-GCST90001514	GCST90001514	HSC AC
ebi-a-GCST90001515	GCST90001515	Im MDSC AC
ebi-a-GCST90001516	GCST90001516	Im MDSC %CD33dim HLA DR- CD6
ebi-a-GCST90001517	GCST90001517	CD33br HLA DR+ AC
ebi-a-GCST90001518	GCST90001518	CD33br HLA DR+ CD14- AC
ebi-a-GCST90001519	GCST90001519	CD33br HLA DR+ CD14- %CD33br H
ebi-a-GCST90001520	GCST90001520	CD33br HLA DR+ CD14dim AC
ebi-a-GCST90001521	GCST90001521	CD33br HLA DR+ CD14dim %CD33b
ebi-a-GCST90001522	GCST90001522	CD33- HLA DR- AC
ebi-a-GCST90001523	GCST90001523	CD33- HLA DR+ AC
ebi-a-GCST90001524	GCST90001524	Gr MDSC AC
ebi-a-GCST90001525	GCST90001525	CD33dim HLA DR+ CD11b+ AC
ebi-a-GCST90001526	GCST90001526	CD33dim HLA DR+ CD11b+ %CD33
ebi-a-GCST90001527	GCST90001527	CD33dim HLA DR+ CD11b- AC
ebi-a-GCST90001528	GCST90001528	CD33dim HLA DR+ CD11b- %CD33c
ebi-a-GCST90001529	GCST90001529	CD66b++ myeloid cell AC
ebi-a-GCST90001530	GCST90001530	Mo MDSC AC
ebi-a-GCST90001531	GCST90001531	CD33dim HLA DR- AC

ebi-a-GCST90001532	GCST90001532	Basophil AC
ebi-a-GCST90001533	GCST90001533	Basophil %CD33dim HLA DR- CD66l
ebi-a-GCST90001534	GCST90001534	CD45RA- CD4+ AC
ebi-a-GCST90001535	GCST90001535	CD45RA- CD4+ %CD4+
ebi-a-GCST90001536	GCST90001536	CD45RA- CD4+ %T cell
ebi-a-GCST90001537	GCST90001537	CM CD4+ AC
ebi-a-GCST90001538	GCST90001538	CM CD4+ %CD4+
ebi-a-GCST90001539	GCST90001539	CM CD4+ %T cell
ebi-a-GCST90001540	GCST90001540	Naive CD4+ AC
ebi-a-GCST90001541	GCST90001541	Naive CD4+ %CD4+
ebi-a-GCST90001542	GCST90001542	EM CD4+ AC
ebi-a-GCST90001543	GCST90001543	EM CD4+ %CD4+
ebi-a-GCST90001544	GCST90001544	EM CD4+ %T cell
ebi-a-GCST90001545	GCST90001545	TD CD4+ AC
ebi-a-GCST90001546	GCST90001546	TD CD4+ %CD4+
ebi-a-GCST90001547	GCST90001547	TD CD4+ %T cell
ebi-a-GCST90001548	GCST90001548	CM CD8br AC
ebi-a-GCST90001549	GCST90001549	CM CD8br %CD8br
ebi-a-GCST90001550	GCST90001550	CM CD8br %T cell
ebi-a-GCST90001551	GCST90001551	Naive CD8br AC
ebi-a-GCST90001552	GCST90001552	Naive CD8br %CD8br
ebi-a-GCST90001553	GCST90001553	Naive CD8br %T cell
ebi-a-GCST90001554	GCST90001554	EM CD8br AC
ebi-a-GCST90001555	GCST90001555	EM CD8br %CD8br
ebi-a-GCST90001556	GCST90001556	EM CD8br %T cell
ebi-a-GCST90001557	GCST90001557	TD CD8br AC
ebi-a-GCST90001558	GCST90001558	TD CD8br %CD8br
ebi-a-GCST90001559	GCST90001559	TD CD8br %T cell
ebi-a-GCST90001560	GCST90001560	CD45RA+ CD8br AC
ebi-a-GCST90001561	GCST90001561	CD45RA+ CD8br %CD8br
ebi-a-GCST90001562	GCST90001562	CD45RA+ CD8br %T cell
ebi-a-GCST90001563	GCST90001563	CM DN (CD4-CD8-) AC
ebi-a-GCST90001564	GCST90001564	CM DN (CD4-CD8-) %DN
ebi-a-GCST90001565	GCST90001565	CM DN (CD4-CD8-) %T cell
ebi-a-GCST90001566	GCST90001566	Naive DN (CD4-CD8-) AC
ebi-a-GCST90001567	GCST90001567	Naive DN (CD4-CD8-) %DN
ebi-a-GCST90001568	GCST90001568	Naive DN (CD4-CD8-) %T cell
ebi-a-GCST90001569	GCST90001569	EM DN (CD4-CD8-) AC
ebi-a-GCST90001570	GCST90001570	EM DN (CD4-CD8-) %DN
ebi-a-GCST90001571	GCST90001571	EM DN (CD4-CD8-) %T cell
ebi-a-GCST90001572	GCST90001572	TD DN (CD4-CD8-) AC
ebi-a-GCST90001573	GCST90001573	TD DN (CD4-CD8-) %DN
ebi-a-GCST90001574	GCST90001574	TD DN (CD4-CD8-) %T cell
ebi-a-GCST90001575	GCST90001575	Naive CD4+ %T cell
ebi-a-GCST90001576	GCST90001576	Transitional %B cell
ebi-a-GCST90001577	GCST90001577	Transitional AC
ebi-a-GCST90001578	GCST90001578	Transitional %lymphocyte
ebi-a-GCST90001579	GCST90001579	CD14- CD16+ monocyte AC

ebi-a-GCST90001580	GCST90001580	CD14+ CD16+ monocyte AC
ebi-a-GCST90001581	GCST90001581	CD14- CD16- AC
ebi-a-GCST90001582	GCST90001582	CD14+ CD16- monocyte AC
ebi-a-GCST90001583	GCST90001583	Monocyte AC
ebi-a-GCST90001584	GCST90001584	CD14- CD16+ monocyte %monocyte
ebi-a-GCST90001585	GCST90001585	CD14+ CD16+ monocyte %monocyte
ebi-a-GCST90001586	GCST90001586	CD14+ CD16- monocyte %monocyte
ebi-a-GCST90001587	GCST90001587	CD16+ monocyte %monocyte
ebi-a-GCST90001588	GCST90001588	T/B
ebi-a-GCST90001589	GCST90001589	CD4/CD8br
ebi-a-GCST90001590	GCST90001590	CD4+ AC
ebi-a-GCST90001591	GCST90001591	CD4+ %T cell
ebi-a-GCST90001592	GCST90001592	CD8br AC
ebi-a-GCST90001593	GCST90001593	CD8br %T cell
ebi-a-GCST90001594	GCST90001594	DP (CD4+CD8+) AC
ebi-a-GCST90001595	GCST90001595	DP (CD4+CD8+) %T cell
ebi-a-GCST90001596	GCST90001596	CD8dim AC
ebi-a-GCST90001597	GCST90001597	CD8dim %T cell
ebi-a-GCST90001598	GCST90001598	DN (CD4-CD8-) AC
ebi-a-GCST90001599	GCST90001599	DN (CD4-CD8-) %T cell
ebi-a-GCST90001600	GCST90001600	Leukocyte AC
ebi-a-GCST90001601	GCST90001601	Lymphocyte AC
ebi-a-GCST90001602	GCST90001602	Lymphocyte %leukocyte
ebi-a-GCST90001603	GCST90001603	T cell AC
ebi-a-GCST90001604	GCST90001604	T cell %lymphocyte
ebi-a-GCST90001605	GCST90001605	T cell %leukocyte
ebi-a-GCST90001606	GCST90001606	CD4+ %leukocyte
ebi-a-GCST90001607	GCST90001607	CD8br %leukocyte
ebi-a-GCST90001608	GCST90001608	DP (CD4+CD8+) %leukocyte
ebi-a-GCST90001609	GCST90001609	CD4+ CD8dim AC
ebi-a-GCST90001610	GCST90001610	CD4+ CD8dim %lymphocyte
ebi-a-GCST90001611	GCST90001611	CD4+ CD8dim %leukocyte
ebi-a-GCST90001612	GCST90001612	CD8dim %leukocyte
ebi-a-GCST90001613	GCST90001613	DN (CD4-CD8-) %leukocyte
ebi-a-GCST90001614	GCST90001614	CD8br and CD8dim %leukocyte
ebi-a-GCST90001615	GCST90001615	TCRgd AC
ebi-a-GCST90001616	GCST90001616	TCRgd %T cell
ebi-a-GCST90001617	GCST90001617	TCRgd %lymphocyte
ebi-a-GCST90001618	GCST90001618	HLA DR+ T cell AC
ebi-a-GCST90001619	GCST90001619	HLA DR+ T cell%T cell
ebi-a-GCST90001620	GCST90001620	HLA DR+ T cell%lymphocyte
ebi-a-GCST90001621	GCST90001621	NKT AC
ebi-a-GCST90001622	GCST90001622	NKT %T cell
ebi-a-GCST90001623	GCST90001623	NKT %lymphocyte
ebi-a-GCST90001624	GCST90001624	HLA DR+ CD4+ AC
ebi-a-GCST90001625	GCST90001625	HLA DR+ CD4+ %T cell
ebi-a-GCST90001626	GCST90001626	HLA DR+ CD4+ %lymphocyte
ebi-a-GCST90001627	GCST90001627	HLA DR+ CD8br AC

ebi-a-GCST90001628	GCST90001628	HLA DR+ CD8br %T cell
ebi-a-GCST90001629	GCST90001629	HLA DR+ CD8br %lymphocyte
ebi-a-GCST90001630	GCST90001630	CD8br NKT AC
ebi-a-GCST90001631	GCST90001631	CD8br NKT %T cell
ebi-a-GCST90001632	GCST90001632	CD8br NKT %lymphocyte
ebi-a-GCST90001633	GCST90001633	CD8dim NKT AC
ebi-a-GCST90001634	GCST90001634	CD8dim NKT %T cell
ebi-a-GCST90001635	GCST90001635	CD8dim NKT %lymphocyte
ebi-a-GCST90001636	GCST90001636	DN (CD4-CD8-) NKT AC
ebi-a-GCST90001637	GCST90001637	DN (CD4-CD8-) NKT %T cell
ebi-a-GCST90001638	GCST90001638	DN (CD4-CD8-) NKT %lymphocyte
ebi-a-GCST90001639	GCST90001639	CD3- lymphocyte AC
ebi-a-GCST90001640	GCST90001640	CD3- lymphocyte %lymphocyte
ebi-a-GCST90001641	GCST90001641	CD3- lymphocyte %leukocyte
ebi-a-GCST90001642	GCST90001642	B cell AC
ebi-a-GCST90001643	GCST90001643	B cell % CD3- lymphocyte
ebi-a-GCST90001644	GCST90001644	B cell %lymphocyte
ebi-a-GCST90001645	GCST90001645	NK AC
ebi-a-GCST90001646	GCST90001646	NK %CD3- lymphocyte
ebi-a-GCST90001647	GCST90001647	NK %lymphocyte
ebi-a-GCST90001648	GCST90001648	HLA DR+ NK AC
ebi-a-GCST90001649	GCST90001649	HLA DR+ NK %NK
ebi-a-GCST90001650	GCST90001650	HLA DR+ NK %CD3- lymphocyte
ebi-a-GCST90001651	GCST90001651	Granulocyte AC
ebi-a-GCST90001652	GCST90001652	Granulocyte %leukocyte
ebi-a-GCST90001653	GCST90001653	CD28- DN (CD4-CD8-) %DN
ebi-a-GCST90001654	GCST90001654	CD28- DN (CD4-CD8-) AC
ebi-a-GCST90001655	GCST90001655	CD28+ DN (CD4-CD8-) %T cell
ebi-a-GCST90001656	GCST90001656	CD28+ DN (CD4-CD8-) %DN
ebi-a-GCST90001657	GCST90001657	CD28+ DN (CD4-CD8-) AC
ebi-a-GCST90001658	GCST90001658	CD39+ CD4+ %T cell
ebi-a-GCST90001659	GCST90001659	CD39+ CD4+ %CD4+
ebi-a-GCST90001660	GCST90001660	CD39+ CD4+ AC
ebi-a-GCST90001661	GCST90001661	CD28- CD8dim %T cell
ebi-a-GCST90001662	GCST90001662	CD28- CD8dim %CD8dim
ebi-a-GCST90001663	GCST90001663	CD28- CD8dim AC
ebi-a-GCST90001664	GCST90001664	CD28+ CD45RA+ CD8dim %T cell
ebi-a-GCST90001665	GCST90001665	CD28+ CD45RA+ CD8dim %CD8dim
ebi-a-GCST90001666	GCST90001666	CD28+ CD45RA+ CD8dim AC
ebi-a-GCST90001667	GCST90001667	CD28+ CD45RA- CD8dim %T cell
ebi-a-GCST90001668	GCST90001668	CD28+ CD45RA- CD8dim %CD8dim
ebi-a-GCST90001669	GCST90001669	CD28+ CD45RA- CD8dim AC
ebi-a-GCST90001670	GCST90001670	CD39+ CD8br %T cell
ebi-a-GCST90001671	GCST90001671	CD39+ CD8br %CD8br
ebi-a-GCST90001672	GCST90001672	CD39+ CD8br AC
ebi-a-GCST90001673	GCST90001673	CD28- CD127- CD25++ CD8br %T ce
ebi-a-GCST90001674	GCST90001674	CD28- CD127- CD25++ CD8br %CD8
ebi-a-GCST90001675	GCST90001675	CD28- CD127- CD25++ CD8br AC

ebi-a-GCST90001676	GCST90001676	CD28- CD25++ CD8br %T cell
ebi-a-GCST90001677	GCST90001677	CD28- CD25++ CD8br %CD8br
ebi-a-GCST90001678	GCST90001678	CD28- CD25++ CD8br AC
ebi-a-GCST90001679	GCST90001679	CD25++ CD8br %T cell
ebi-a-GCST90001680	GCST90001680	CD25++ CD8br %CD8br
ebi-a-GCST90001681	GCST90001681	CD25++ CD8br AC
ebi-a-GCST90001682	GCST90001682	CD127- CD8br %T cell
ebi-a-GCST90001683	GCST90001683	CD127- CD8br %CD8br
ebi-a-GCST90001684	GCST90001684	CD127- CD8br AC
ebi-a-GCST90001685	GCST90001685	CD28- CD8br %T cell
ebi-a-GCST90001686	GCST90001686	CD28- CD8br %CD8br
ebi-a-GCST90001687	GCST90001687	CD28- CD8br AC
ebi-a-GCST90001688	GCST90001688	CD28+ CD45RA+ CD8br %T cell
ebi-a-GCST90001689	GCST90001689	CD28+ CD45RA+ CD8br %CD8br
ebi-a-GCST90001690	GCST90001690	CD28+ CD45RA+ CD8br AC
ebi-a-GCST90001691	GCST90001691	CD28+ CD45RA- CD8br %T cell
ebi-a-GCST90001692	GCST90001692	CD28+ CD45RA- CD8br %CD8br
ebi-a-GCST90001693	GCST90001693	CD28+ CD45RA- CD8br AC
ebi-a-GCST90001694	GCST90001694	CD28- DN (CD4-CD8-) %T cell
ebi-a-GCST90001695	GCST90001695	CD45RA- CD28- CD8br AC
ebi-a-GCST90001696	GCST90001696	CD45RA- CD28- CD8br %CD8br
ebi-a-GCST90001697	GCST90001697	CD45RA- CD28- CD8br %T cell
ebi-a-GCST90001698	GCST90001698	CD45RA+ CD28- CD8br AC
ebi-a-GCST90001699	GCST90001699	CD45RA+ CD28- CD8br %CD8br
ebi-a-GCST90001700	GCST90001700	CD45RA+ CD28- CD8br %T cell
ebi-a-GCST90001701	GCST90001701	BAFF-R on CD20- CD38-
ebi-a-GCST90001702	GCST90001702	BAFF-R on CD24+ CD27+
ebi-a-GCST90001703	GCST90001703	BAFF-R on IgD+ CD24+
ebi-a-GCST90001704	GCST90001704	BAFF-R on IgD+ CD24-
ebi-a-GCST90001705	GCST90001705	BAFF-R on IgD+ CD38-
ebi-a-GCST90001706	GCST90001706	BAFF-R on IgD+ CD38- naive
ebi-a-GCST90001707	GCST90001707	BAFF-R on IgD+ CD38- unsw mem
ebi-a-GCST90001708	GCST90001708	BAFF-R on IgD+ CD38br
ebi-a-GCST90001709	GCST90001709	BAFF-R on IgD+ CD38dim
ebi-a-GCST90001710	GCST90001710	BAFF-R on IgD- CD24-
ebi-a-GCST90001711	GCST90001711	BAFF-R on IgD- CD27-
ebi-a-GCST90001712	GCST90001712	BAFF-R on IgD- CD38-
ebi-a-GCST90001713	GCST90001713	BAFF-R on IgD- CD38br
ebi-a-GCST90001714	GCST90001714	BAFF-R on IgD- CD38dim
ebi-a-GCST90001715	GCST90001715	BAFF-R on memory B cell
ebi-a-GCST90001716	GCST90001716	BAFF-R on naive-mature B cell
ebi-a-GCST90001717	GCST90001717	BAFF-R on unsw mem
ebi-a-GCST90001718	GCST90001718	BAFF-R on sw mem
ebi-a-GCST90001719	GCST90001719	BAFF-R on IgD+
ebi-a-GCST90001720	GCST90001720	BAFF-R on transitional
ebi-a-GCST90001721	GCST90001721	CD19 on CD20-
ebi-a-GCST90001722	GCST90001722	CD19 on CD20- CD38-
ebi-a-GCST90001723	GCST90001723	CD19 on CD24+ CD27+

ebi-a-GCST90001724	GCST90001724	CD19 on IgD+ CD24+
ebi-a-GCST90001725	GCST90001725	CD19 on IgD+ CD24-
ebi-a-GCST90001726	GCST90001726	CD19 on IgD+ CD38-
ebi-a-GCST90001727	GCST90001727	CD19 on IgD+ CD38- naive
ebi-a-GCST90001728	GCST90001728	CD19 on IgD+ CD38- unsw mem
ebi-a-GCST90001729	GCST90001729	CD19 on IgD+ CD38br
ebi-a-GCST90001730	GCST90001730	CD19 on IgD+ CD38dim
ebi-a-GCST90001731	GCST90001731	CD19 on IgD- CD24-
ebi-a-GCST90001732	GCST90001732	CD19 on IgD- CD27-
ebi-a-GCST90001733	GCST90001733	CD19 on IgD- CD38-
ebi-a-GCST90001734	GCST90001734	CD19 on IgD- CD38br
ebi-a-GCST90001735	GCST90001735	CD19 on IgD- CD38dim
ebi-a-GCST90001736	GCST90001736	CD19 on memory B cell
ebi-a-GCST90001737	GCST90001737	CD19 on naive-mature B cell
ebi-a-GCST90001738	GCST90001738	CD19 on unsw mem
ebi-a-GCST90001739	GCST90001739	CD19 on PB/PC
ebi-a-GCST90001740	GCST90001740	CD19 on sw mem
ebi-a-GCST90001741	GCST90001741	CD19 on IgD+
ebi-a-GCST90001742	GCST90001742	CD19 on transitional
ebi-a-GCST90001743	GCST90001743	CD20 on B cell
ebi-a-GCST90001744	GCST90001744	CD20 on CD20- CD38-
ebi-a-GCST90001745	GCST90001745	CD20 on CD24+ CD27+
ebi-a-GCST90001746	GCST90001746	CD20 on IgD+ CD24+
ebi-a-GCST90001747	GCST90001747	CD20 on IgD+ CD24-
ebi-a-GCST90001748	GCST90001748	CD20 on IgD+ CD38-
ebi-a-GCST90001749	GCST90001749	CD20 on IgD+ CD38- naive
ebi-a-GCST90001750	GCST90001750	CD20 on IgD+ CD38- unsw mem
ebi-a-GCST90001751	GCST90001751	CD20 on IgD+ CD38br
ebi-a-GCST90001752	GCST90001752	CD20 on IgD+ CD38dim
ebi-a-GCST90001753	GCST90001753	CD20 on IgD- CD24-
ebi-a-GCST90001754	GCST90001754	CD20 on IgD- CD27-
ebi-a-GCST90001755	GCST90001755	CD20 on IgD- CD38-
ebi-a-GCST90001756	GCST90001756	CD20 on IgD- CD38br
ebi-a-GCST90001757	GCST90001757	CD20 on IgD- CD38dim
ebi-a-GCST90001758	GCST90001758	CD20 on memory B cell
ebi-a-GCST90001759	GCST90001759	CD20 on naive-mature B cell
ebi-a-GCST90001760	GCST90001760	CD20 on unsw mem
ebi-a-GCST90001761	GCST90001761	CD20 on sw mem
ebi-a-GCST90001762	GCST90001762	CD20 on IgD+
ebi-a-GCST90001763	GCST90001763	CD20 on transitional
ebi-a-GCST90001764	GCST90001764	CD24 on CD24+ CD27+
ebi-a-GCST90001765	GCST90001765	CD24 on IgD+ CD24+
ebi-a-GCST90001766	GCST90001766	CD24 on IgD+ CD38-
ebi-a-GCST90001767	GCST90001767	CD24 on IgD+ CD38- unsw mem
ebi-a-GCST90001768	GCST90001768	CD24 on IgD+ CD38br
ebi-a-GCST90001769	GCST90001769	CD24 on IgD- CD38-
ebi-a-GCST90001770	GCST90001770	CD24 on IgD- CD38dim
ebi-a-GCST90001771	GCST90001771	CD24 on memory B cell

ebi-a-GCST90001772	GCST90001772	CD24 on unsw mem
ebi-a-GCST90001773	GCST90001773	CD24 on sw mem
ebi-a-GCST90001774	GCST90001774	CD24 on transitional
ebi-a-GCST90001775	GCST90001775	CD25 on B cell
ebi-a-GCST90001776	GCST90001776	CD25 on CD20- CD38-
ebi-a-GCST90001777	GCST90001777	CD25 on CD24+ CD27+
ebi-a-GCST90001778	GCST90001778	CD25 on IgD+ CD24+
ebi-a-GCST90001779	GCST90001779	CD25 on IgD+ CD24-
ebi-a-GCST90001780	GCST90001780	CD25 on IgD+ CD38-
ebi-a-GCST90001781	GCST90001781	CD25 on IgD+ CD38- naive
ebi-a-GCST90001782	GCST90001782	CD25 on IgD+ CD38- unsw mem
ebi-a-GCST90001783	GCST90001783	CD25 on IgD+ CD38br
ebi-a-GCST90001784	GCST90001784	CD25 on IgD+ CD38dim
ebi-a-GCST90001785	GCST90001785	CD25 on IgD- CD24-
ebi-a-GCST90001786	GCST90001786	CD25 on IgD- CD27-
ebi-a-GCST90001787	GCST90001787	CD25 on IgD- CD38-
ebi-a-GCST90001788	GCST90001788	CD25 on IgD- CD38br
ebi-a-GCST90001789	GCST90001789	CD25 on IgD- CD38dim
ebi-a-GCST90001790	GCST90001790	CD25 on memory B cell
ebi-a-GCST90001791	GCST90001791	CD25 on naive-mature B cell
ebi-a-GCST90001792	GCST90001792	CD25 on unsw mem
ebi-a-GCST90001793	GCST90001793	CD25 on sw mem
ebi-a-GCST90001794	GCST90001794	CD25 on IgD+
ebi-a-GCST90001795	GCST90001795	CD25 on transitional
ebi-a-GCST90001796	GCST90001796	CD27 on CD20-
ebi-a-GCST90001797	GCST90001797	CD27 on CD20- CD38-
ebi-a-GCST90001798	GCST90001798	CD27 on CD24+ CD27+
ebi-a-GCST90001799	GCST90001799	CD27 on T cell
ebi-a-GCST90001800	GCST90001800	CD27 on IgD+ CD24+
ebi-a-GCST90001801	GCST90001801	CD27 on IgD+ CD38- unsw mem
ebi-a-GCST90001802	GCST90001802	CD27 on IgD- CD38-
ebi-a-GCST90001803	GCST90001803	CD27 on IgD- CD38br
ebi-a-GCST90001804	GCST90001804	CD27 on IgD- CD38dim
ebi-a-GCST90001805	GCST90001805	CD27 on memory B cell
ebi-a-GCST90001806	GCST90001806	CD27 on unsw mem
ebi-a-GCST90001807	GCST90001807	CD27 on PB/PC
ebi-a-GCST90001808	GCST90001808	CD27 on sw mem
ebi-a-GCST90001809	GCST90001809	CD38 on CD20-
ebi-a-GCST90001810	GCST90001810	CD38 on CD3- CD19-
ebi-a-GCST90001811	GCST90001811	CD38 on IgD+ CD24-
ebi-a-GCST90001812	GCST90001812	CD38 on IgD+ CD38br
ebi-a-GCST90001813	GCST90001813	CD38 on IgD+ CD38dim
ebi-a-GCST90001814	GCST90001814	CD38 on IgD- CD38br
ebi-a-GCST90001815	GCST90001815	CD38 on IgD- CD38dim
ebi-a-GCST90001816	GCST90001816	CD38 on naive-mature B cell
ebi-a-GCST90001817	GCST90001817	CD38 on PB/PC
ebi-a-GCST90001818	GCST90001818	CD38 on IgD+
ebi-a-GCST90001819	GCST90001819	CD38 on transitional

ebi-a-GCST90001820	GCST90001820	IgD on IgD+ CD24+
ebi-a-GCST90001821	GCST90001821	IgD on IgD+ CD24-
ebi-a-GCST90001822	GCST90001822	IgD on IgD+ CD38-
ebi-a-GCST90001823	GCST90001823	IgD on IgD+ CD38- unsw mem
ebi-a-GCST90001824	GCST90001824	IgD on IgD+ CD38br
ebi-a-GCST90001825	GCST90001825	IgD on IgD+ CD38dim
ebi-a-GCST90001826	GCST90001826	IgD on unsw mem
ebi-a-GCST90001827	GCST90001827	IgD on IgD+
ebi-a-GCST90001828	GCST90001828	IgD on transitional
ebi-a-GCST90001829	GCST90001829	BAFF-R on B cell
ebi-a-GCST90001830	GCST90001830	BAFF-R on CD20-
ebi-a-GCST90001831	GCST90001831	CD62L on CD62L+ myeloid DC
ebi-a-GCST90001832	GCST90001832	CD62L on CD62L+ plasmacytoid DC
ebi-a-GCST90001833	GCST90001833	CD62L on CD62L+ DC
ebi-a-GCST90001834	GCST90001834	CD62L on monocyte
ebi-a-GCST90001835	GCST90001835	CD62L on granulocyte
ebi-a-GCST90001836	GCST90001836	CD66b on CD66b++ myeloid cell
ebi-a-GCST90001837	GCST90001837	CD66b on Gr MDSC
ebi-a-GCST90001838	GCST90001838	CD3 on naive CD8br
ebi-a-GCST90001839	GCST90001839	CD3 on EM CD8br
ebi-a-GCST90001840	GCST90001840	CD3 on TD CD8br
ebi-a-GCST90001841	GCST90001841	CD3 on CM CD4+
ebi-a-GCST90001842	GCST90001842	CD3 on Naive CD4+
ebi-a-GCST90001843	GCST90001843	CD3 on EM CD4+
ebi-a-GCST90001844	GCST90001844	CD3 on TD CD4+
ebi-a-GCST90001845	GCST90001845	CD3 on CD45RA- CD4+
ebi-a-GCST90001846	GCST90001846	CD3 on CM CD8br
ebi-a-GCST90001847	GCST90001847	CD3 on HLA DR+ T cell
ebi-a-GCST90001848	GCST90001848	CD3 on NKT
ebi-a-GCST90001849	GCST90001849	CD3 on HLA DR+ CD4+
ebi-a-GCST90001850	GCST90001850	CD3 on HLA DR+ CD8br
ebi-a-GCST90001851	GCST90001851	CD3 on T cell
ebi-a-GCST90001852	GCST90001852	CD3 on CD39+ resting Treg
ebi-a-GCST90001853	GCST90001853	CD3 on activated Treg
ebi-a-GCST90001854	GCST90001854	CD3 on CD39+ activated Treg
ebi-a-GCST90001855	GCST90001855	CD3 on secreting Treg
ebi-a-GCST90001856	GCST90001856	CD3 on CD39+ secreting Treg
ebi-a-GCST90001857	GCST90001857	CD3 on activated & secreting Treg
ebi-a-GCST90001858	GCST90001858	CD3 on CD45RA+ CD4+
ebi-a-GCST90001859	GCST90001859	CD3 on CD8br
ebi-a-GCST90001860	GCST90001860	CD3 on CD39+ CD4+
ebi-a-GCST90001861	GCST90001861	CD3 on CD28+ CD4+
ebi-a-GCST90001862	GCST90001862	CD3 on CD28+ DN (CD4-CD8-)
ebi-a-GCST90001863	GCST90001863	CD3 on CD28+ CD45RA- CD8br
ebi-a-GCST90001864	GCST90001864	CD3 on CD28+ CD45RA+ CD8br
ebi-a-GCST90001865	GCST90001865	CD3 on CD28- CD8br
ebi-a-GCST90001866	GCST90001866	CD3 on CD39+ CD8br
ebi-a-GCST90001867	GCST90001867	CD3 on CD4+

ebi-a-GCST90001868	GCST90001868	CD3 on CD4 Treg
ebi-a-GCST90001869	GCST90001869	CD3 on resting Treg
ebi-a-GCST90001870	GCST90001870	CD34 on HSC
ebi-a-GCST90001871	GCST90001871	HVEM on T cell
ebi-a-GCST90001872	GCST90001872	HVEM on naive CD8br
ebi-a-GCST90001873	GCST90001873	HVEM on EM CD8br
ebi-a-GCST90001874	GCST90001874	HVEM on TD CD8br
ebi-a-GCST90001875	GCST90001875	HVEM on CD4+
ebi-a-GCST90001876	GCST90001876	HVEM on CM CD4+
ebi-a-GCST90001877	GCST90001877	HVEM on naive CD4+
ebi-a-GCST90001878	GCST90001878	HVEM on EM CD4+
ebi-a-GCST90001879	GCST90001879	HVEM on TD CD4+
ebi-a-GCST90001880	GCST90001880	HVEM on CD45RA- CD4+
ebi-a-GCST90001881	GCST90001881	HVEM on CD8br
ebi-a-GCST90001882	GCST90001882	HVEM on CM CD8br
ebi-a-GCST90001883	GCST90001883	CD16-CD56 on NKT
ebi-a-GCST90001884	GCST90001884	CD16-CD56 on NK
ebi-a-GCST90001885	GCST90001885	CD16-CD56 on HLA DR+ NK
ebi-a-GCST90001886	GCST90001886	CD28 on CD39+ activated Treg
ebi-a-GCST90001887	GCST90001887	CD28 on secreting Treg
ebi-a-GCST90001888	GCST90001888	CD28 on CD39+ secreting Treg
ebi-a-GCST90001889	GCST90001889	CD28 on activated & secreting Treg
ebi-a-GCST90001890	GCST90001890	CD28 on CD45RA+ CD4+
ebi-a-GCST90001891	GCST90001891	CD28 on CD45RA- CD4 not Treg
ebi-a-GCST90001892	GCST90001892	CD28 on CD39+ CD4+
ebi-a-GCST90001893	GCST90001893	CD28 on CD28+ CD45RA- CD8br
ebi-a-GCST90001894	GCST90001894	CD28 on CD28+ CD4+
ebi-a-GCST90001895	GCST90001895	CD28 on CD28+ DN (CD4-CD8-)
ebi-a-GCST90001896	GCST90001896	CD28 on CD28+ CD45RA+ CD8br
ebi-a-GCST90001897	GCST90001897	CD28 on CD39+ CD8br
ebi-a-GCST90001898	GCST90001898	CD28 on CD4+
ebi-a-GCST90001899	GCST90001899	CD28 on CD4 Treg
ebi-a-GCST90001900	GCST90001900	CD28 on resting Treg
ebi-a-GCST90001901	GCST90001901	CD28 on CD39+ resting Treg
ebi-a-GCST90001902	GCST90001902	CD28 on activated Treg
ebi-a-GCST90001903	GCST90001903	CD86 on myeloid DC
ebi-a-GCST90001904	GCST90001904	CD86 on CD62L+ myeloid DC
ebi-a-GCST90001905	GCST90001905	CD86 on monocyte
ebi-a-GCST90001906	GCST90001906	CD86 on granulocyte
ebi-a-GCST90001907	GCST90001907	CCR7 on naive CD4+
ebi-a-GCST90001908	GCST90001908	CCR7 on naive CD8br
ebi-a-GCST90001909	GCST90001909	CD45 on CD14+ monocyte
ebi-a-GCST90001910	GCST90001910	CD45 on B cell
ebi-a-GCST90001911	GCST90001911	CD45 on NK
ebi-a-GCST90001912	GCST90001912	CD45 on HLA DR+ NK
ebi-a-GCST90001913	GCST90001913	CD45 on granulocyte
ebi-a-GCST90001914	GCST90001914	CD45 on lymphocyte
ebi-a-GCST90001915	GCST90001915	CD45 on T cell

ebi-a-GCST90001916	GCST90001916	CD45 on CD4+
ebi-a-GCST90001917	GCST90001917	CD45 on CD8br
ebi-a-GCST90001918	GCST90001918	CD45 on HLA DR+ T cell
ebi-a-GCST90001919	GCST90001919	CD45 on NKT
ebi-a-GCST90001920	GCST90001920	CD45 on HLA DR+ CD4+
ebi-a-GCST90001921	GCST90001921	CD45 on HLA DR+ CD8br
ebi-a-GCST90001922	GCST90001922	CD127 on T cell
ebi-a-GCST90001923	GCST90001923	CD127 on CD45RA- CD4 not Treg
ebi-a-GCST90001924	GCST90001924	CD127 on CD28+ CD4+
ebi-a-GCST90001925	GCST90001925	CD127 on CD28+ DN (CD4-CD8-)
ebi-a-GCST90001926	GCST90001926	CD127 on granulocyte
ebi-a-GCST90001927	GCST90001927	CD127 on CD8br
ebi-a-GCST90001928	GCST90001928	CD127 on CD28+ CD45RA- CD8br
ebi-a-GCST90001929	GCST90001929	CD127 on CD28+ CD45RA+ CD8br
ebi-a-GCST90001930	GCST90001930	CD127 on CD28- CD8br
ebi-a-GCST90001931	GCST90001931	CD127 on CD4+
ebi-a-GCST90001932	GCST90001932	CD127 on CD45RA+ CD4+
ebi-a-GCST90001933	GCST90001933	CD25 on CD45RA- CD4 not Treg
ebi-a-GCST90001934	GCST90001934	CD25 on CD45RA+ CD4 not Treg
ebi-a-GCST90001935	GCST90001935	CD25 on CD39+ CD4 Treg
ebi-a-GCST90001936	GCST90001936	CD25 on CD4 Treg
ebi-a-GCST90001937	GCST90001937	CD25 on resting Treg
ebi-a-GCST90001938	GCST90001938	CD25 on CD39+ resting Treg
ebi-a-GCST90001939	GCST90001939	CD25 on activated Treg
ebi-a-GCST90001940	GCST90001940	CD25 on CD39+ activated Treg
ebi-a-GCST90001941	GCST90001941	CD25 on secreting Treg
ebi-a-GCST90001942	GCST90001942	CD25 on CD39+ secreting Treg
ebi-a-GCST90001943	GCST90001943	CD25 on activated & secreting Treg
ebi-a-GCST90001944	GCST90001944	CD123 on plasmacytoid DC
ebi-a-GCST90001945	GCST90001945	CD123 on CD62L+ plasmacytoid DC
ebi-a-GCST90001946	GCST90001946	CD33 on CD14+ monocyte
ebi-a-GCST90001947	GCST90001947	CD33 on CD33br HLA DR+ CD14dim
ebi-a-GCST90001948	GCST90001948	CD33 on CD33dim HLA DR+ CD11b-
ebi-a-GCST90001949	GCST90001949	CD33 on CD33dim HLA DR+ CD11b-
ebi-a-GCST90001950	GCST90001950	CD33 on Gr MDSC
ebi-a-GCST90001951	GCST90001951	CD33 on CD66b++ myeloid cell
ebi-a-GCST90001952	GCST90001952	CD33 on Mo MDSC
ebi-a-GCST90001953	GCST90001953	CD33 on CD33dim HLA DR-
ebi-a-GCST90001954	GCST90001954	CD33 on basophil
ebi-a-GCST90001955	GCST90001955	CD33 on Im MDSC
ebi-a-GCST90001956	GCST90001956	CD33 on CD33br HLA DR+
ebi-a-GCST90001957	GCST90001957	CD33 on CD33br HLA DR+ CD14-
ebi-a-GCST90001958	GCST90001958	CD4 on monocyte
ebi-a-GCST90001959	GCST90001959	CD4 on HLA DR+ CD4+
ebi-a-GCST90001960	GCST90001960	CD25 on CD4+
ebi-a-GCST90001961	GCST90001961	CD25 on CD39+ CD4+
ebi-a-GCST90001962	GCST90001962	CD25 on CD28+ CD4+
ebi-a-GCST90001963	GCST90001963	FSC-A on myeloid DC

ebi-a-GCST90001964	GCST90001964	FSC-A on plasmacytoid DC
ebi-a-GCST90001965	GCST90001965	FSC-A on monocyte
ebi-a-GCST90001966	GCST90001966	FSC-A on granulocyte
ebi-a-GCST90001967	GCST90001967	FSC-A on CD14+ monocyte
ebi-a-GCST90001968	GCST90001968	FSC-A on B cell
ebi-a-GCST90001969	GCST90001969	FSC-A on NK
ebi-a-GCST90001970	GCST90001970	FSC-A on HLA DR+ NK
ebi-a-GCST90001971	GCST90001971	FSC-A on lymphocyte
ebi-a-GCST90001972	GCST90001972	FSC-A on T cell
ebi-a-GCST90001973	GCST90001973	FSC-A on CD4+
ebi-a-GCST90001974	GCST90001974	FSC-A on CD8br
ebi-a-GCST90001975	GCST90001975	FSC-A on HLA DR+ T cell
ebi-a-GCST90001976	GCST90001976	FSC-A on NKT
ebi-a-GCST90001977	GCST90001977	FSC-A on HLA DR+ CD4+
ebi-a-GCST90001978	GCST90001978	FSC-A on HLA DR+ CD8br
ebi-a-GCST90001979	GCST90001979	CD16 on CD14- CD16+ monocyte
ebi-a-GCST90001980	GCST90001980	CD40 on CD14+ CD16- monocyte
ebi-a-GCST90001981	GCST90001981	CD40 on CD14+ CD16+ monocyte
ebi-a-GCST90001982	GCST90001982	CCR2 on CD14- CD16+ monocyte
ebi-a-GCST90001983	GCST90001983	CD14 on CD14+ CD16+ monocyte
ebi-a-GCST90001984	GCST90001984	HLA DR on CD14- CD16+ monocyte
ebi-a-GCST90001985	GCST90001985	CD40 on monocytes
ebi-a-GCST90001986	GCST90001986	CD14 on CD14+ CD16- monocyte
ebi-a-GCST90001987	GCST90001987	CD64 on CD14+ CD16- monocyte
ebi-a-GCST90001988	GCST90001988	HLA DR on CD14+ CD16- monocyte
ebi-a-GCST90001989	GCST90001989	CD40 on CD14- CD16+ monocyte
ebi-a-GCST90001990	GCST90001990	CD64 on CD14- CD16+ monocyte
ebi-a-GCST90001991	GCST90001991	HLA DR on CD14+ monocyte
ebi-a-GCST90001992	GCST90001992	CCR2 on CD14+ CD16+ monocyte
ebi-a-GCST90001993	GCST90001993	PDL-1 on CD14+ CD16- monocyte
ebi-a-GCST90001994	GCST90001994	CX3CR1 on CD14- CD16-
ebi-a-GCST90001995	GCST90001995	CX3CR1 on monocyte
ebi-a-GCST90001996	GCST90001996	CX3CR1 on CD14+ CD16+ monocyte
ebi-a-GCST90001997	GCST90001997	CX3CR1 on CD14+ CD16- monocyte
ebi-a-GCST90001998	GCST90001998	PDL-1 on CD14+ CD16+ monocyte
ebi-a-GCST90001999	GCST90001999	PDL-1 on CD14- CD16+ monocyte
ebi-a-GCST90002000	GCST90002000	PDL-1 on CD14- CD16-
ebi-a-GCST90002001	GCST90002001	CD64 on CD14- CD16-
ebi-a-GCST90002002	GCST90002002	PDL-1 on monocyte
ebi-a-GCST90002003	GCST90002003	CCR2 on CD14- CD16-
ebi-a-GCST90002004	GCST90002004	CCR2 on CD14+ CD16- monocyte
ebi-a-GCST90002005	GCST90002005	CD16 on CD14+ CD16+ monocyte
ebi-a-GCST90002006	GCST90002006	CD64 on monocyte
ebi-a-GCST90002007	GCST90002007	HLA DR on CD14+ CD16+ monocyte
ebi-a-GCST90002008	GCST90002008	CCR2 on monocyte
ebi-a-GCST90002009	GCST90002009	HLA DR on CD14- CD16-
ebi-a-GCST90002010	GCST90002010	HLA DR on monocyte
ebi-a-GCST90002011	GCST90002011	CD64 on CD14+ CD16+ monocyte

ebi-a-GCST90002012	GCST90002012	CX3CR1 on CD14- CD16+ monocyte
ebi-a-GCST90002013	GCST90002013	CCR2 on myeloid DC
ebi-a-GCST90002014	GCST90002014	CCR2 on CD62L+ myeloid DC
ebi-a-GCST90002015	GCST90002015	CCR2 on plasmacytoid DC
ebi-a-GCST90002016	GCST90002016	CCR2 on CD62L+ plasmacytoid DC
ebi-a-GCST90002017	GCST90002017	CCR2 on monocyte
ebi-a-GCST90002018	GCST90002018	CCR2 on granulocyte
ebi-a-GCST90002019	GCST90002019	CD14 on Mo MDSC
ebi-a-GCST90002020	GCST90002020	CD14 on CD33br HLA DR+ CD14dim
ebi-a-GCST90002021	GCST90002021	CD14 on CD33dim HLA DR+ CD11b
ebi-a-GCST90002022	GCST90002022	CD4 on CD4+
ebi-a-GCST90002023	GCST90002023	CD4 on CM CD4 +
ebi-a-GCST90002024	GCST90002024	CD4 on naive CD4+
ebi-a-GCST90002025	GCST90002025	CD4 on EM CD4+
ebi-a-GCST90002026	GCST90002026	CD4 on TD CD4+
ebi-a-GCST90002027	GCST90002027	CD4 on CD45RA+ CD4+
ebi-a-GCST90002028	GCST90002028	CD19 on B cell
ebi-a-GCST90002029	GCST90002029	CD39 on CD39+ CD8br
ebi-a-GCST90002030	GCST90002030	CD39 on CD39+ activated Treg
ebi-a-GCST90002031	GCST90002031	CD39 on CD39+ secreting Treg
ebi-a-GCST90002032	GCST90002032	CD39 on CD39+ CD4+
ebi-a-GCST90002033	GCST90002033	CD39 on granulocyte
ebi-a-GCST90002034	GCST90002034	CD39 on monocyte
ebi-a-GCST90002035	GCST90002035	CD80 on myeloid DC
ebi-a-GCST90002036	GCST90002036	CD80 on CD62L+ myeloid DC
ebi-a-GCST90002037	GCST90002037	CD80 on plasmacytoid DC
ebi-a-GCST90002038	GCST90002038	CD80 on CD62L+ plasmacytoid DC
ebi-a-GCST90002039	GCST90002039	CD80 on monocyte
ebi-a-GCST90002040	GCST90002040	CD80 on granulocyte
ebi-a-GCST90002041	GCST90002041	CD45 on lymphocyte
ebi-a-GCST90002042	GCST90002042	CD45 on CD33br HLA DR+ CD14-
ebi-a-GCST90002043	GCST90002043	CD45 on CD33br HLA DR+ CD14dim
ebi-a-GCST90002044	GCST90002044	CD45 on CD33dim HLA DR+ CD11b-
ebi-a-GCST90002045	GCST90002045	CD45 on CD33- HLA DR-
ebi-a-GCST90002046	GCST90002046	CD45 on CD33- HLA DR+
ebi-a-GCST90002047	GCST90002047	CD45 on Gr MDSC
ebi-a-GCST90002048	GCST90002048	CD45 on CD66b++ myelod cell
ebi-a-GCST90002049	GCST90002049	CD45 on Mo MDSC
ebi-a-GCST90002050	GCST90002050	CD45 on CD33dim HLA DR-
ebi-a-GCST90002051	GCST90002051	CD45 on basophil
ebi-a-GCST90002052	GCST90002052	CD45 on Im MDSC
ebi-a-GCST90002053	GCST90002053	CD45 on CD33br HLA DR+
ebi-a-GCST90002054	GCST90002054	CD8 on CM CD8br
ebi-a-GCST90002055	GCST90002055	CD8 on naive CD8br
ebi-a-GCST90002056	GCST90002056	CD8 on EM CD8br
ebi-a-GCST90002057	GCST90002057	CD8 on TD CD8br
ebi-a-GCST90002058	GCST90002058	CD8 on CD8br
ebi-a-GCST90002059	GCST90002059	CD8 on NKT

ebi-a-GCST90002060	GCST90002060	CD8 on HLA DR+ CD8br
ebi-a-GCST90002061	GCST90002061	CD4 on CD39+ CD4+
ebi-a-GCST90002062	GCST90002062	CD4 on CD28+ CD4+
ebi-a-GCST90002063	GCST90002063	CD4 on CD4 Treg
ebi-a-GCST90002064	GCST90002064	CD4 on resting Treg
ebi-a-GCST90002065	GCST90002065	CD4 on CD39+ resting Treg
ebi-a-GCST90002066	GCST90002066	CD4 on activated Treg
ebi-a-GCST90002067	GCST90002067	CD4 on CD39+ activated Treg
ebi-a-GCST90002068	GCST90002068	CD4 on secreting Treg
ebi-a-GCST90002069	GCST90002069	CD4 on CD39+ secreting Treg
ebi-a-GCST90002070	GCST90002070	CD4 on activated & secreting Treg
ebi-a-GCST90002071	GCST90002071	SSC-A on myeloid DC
ebi-a-GCST90002072	GCST90002072	SSC-A on plasmacytoid DC
ebi-a-GCST90002073	GCST90002073	SSC-A on monocyte
ebi-a-GCST90002074	GCST90002074	SSC-A on CD14+ monocyte
ebi-a-GCST90002075	GCST90002075	SSC-A on B cell
ebi-a-GCST90002076	GCST90002076	SSC-A on NK
ebi-a-GCST90002077	GCST90002077	SSC-A on HLA DR+ NK
ebi-a-GCST90002078	GCST90002078	SSC-A on granulocyte
ebi-a-GCST90002079	GCST90002079	SSC-A on lymphocyte
ebi-a-GCST90002080	GCST90002080	SSC-A on T cell
ebi-a-GCST90002081	GCST90002081	SSC-A on CD4+
ebi-a-GCST90002082	GCST90002082	SSC-A on CD8br
ebi-a-GCST90002083	GCST90002083	SSC-A on HLA DR+ T cell
ebi-a-GCST90002084	GCST90002084	SSC-A on NKT
ebi-a-GCST90002085	GCST90002085	SSC-A on HLA DR+ CD4+
ebi-a-GCST90002086	GCST90002086	SSC-A on HLA DR+ CD8br
ebi-a-GCST90002087	GCST90002087	CD11c on myeloid DC
ebi-a-GCST90002088	GCST90002088	CD11c on CD62L+ myeloid DC
ebi-a-GCST90002089	GCST90002089	CD11c on monocyte
ebi-a-GCST90002090	GCST90002090	CD11c on granulocyte
ebi-a-GCST90002091	GCST90002091	CD11b on CD14+ monocyte
ebi-a-GCST90002092	GCST90002092	CD11b on Gr MDSC
ebi-a-GCST90002093	GCST90002093	CD11b on CD66b++ myeloid cell
ebi-a-GCST90002094	GCST90002094	CD11b on Mo MDSC
ebi-a-GCST90002095	GCST90002095	CD11b on CD33dim HLA DR-
ebi-a-GCST90002096	GCST90002096	CD11b on basophil
ebi-a-GCST90002097	GCST90002097	CD11b on CD33br HLA DR+ CD14di
ebi-a-GCST90002098	GCST90002098	CD45RA on naive CD4+
ebi-a-GCST90002099	GCST90002099	CD4RA on TD CD4+
ebi-a-GCST90002100	GCST90002100	CD45RA on naive CD8br
ebi-a-GCST90002101	GCST90002101	CD45RA on TD CD8br
ebi-a-GCST90002102	GCST90002102	CD45RA on resting Treg
ebi-a-GCST90002103	GCST90002103	CD45RA on CD39+ resting Treg
ebi-a-GCST90002104	GCST90002104	HLA DR on myeloid DC
ebi-a-GCST90002105	GCST90002105	HLA DR on plasmacytoid DC
ebi-a-GCST90002106	GCST90002106	HLA DR on DC
ebi-a-GCST90002107	GCST90002107	HLA DR on HSC

ebi-a-GCST90002108	GCST90002108	HLA DR on CD33br HLA DR+ CD14[illegible]
ebi-a-GCST90002109	GCST90002109	HLA DR on CD33br HLA DR+ CD14[illegible]
ebi-a-GCST90002110	GCST90002110	HLA DR on CD33dim HLA DR+ CD1[illegible]
ebi-a-GCST90002111	GCST90002111	HLA DR on CD33dim HLA DR+ CD1[illegible]
ebi-a-GCST90002112	GCST90002112	HLA DR on CD33- HLA DR+
ebi-a-GCST90002113	GCST90002113	HLA DR on HLA DR+ T cell
ebi-a-GCST90002114	GCST90002114	HLA DR on HLA DR+ CD4+
ebi-a-GCST90002115	GCST90002115	HLA DR on HLA DR+ CD8br
ebi-a-GCST90002116	GCST90002116	HLA DR on B cell
ebi-a-GCST90002117	GCST90002117	HLA DR on HLA DR+ NK
ebi-a-GCST90002118	GCST90002118	CD8 on CD28+ CD45RA- CD8br
ebi-a-GCST90002119	GCST90002119	CD8 on CD28+ CD45RA+ CD8br
ebi-a-GCST90002120	GCST90002120	CD8 on CD28- CD8br
ebi-a-GCST90002121	GCST90002121	CD8 on CD39+ CD8br

[illegible]31 Immune Cell Traits

Statistical_trait_name	Panel
BC_Trait1	B cell
BC_Trait10	B cell
BC_Trait11	B cell
BC_Trait12	B cell
BC_Trait13	B cell
BC_Trait14	B cell
BC_Trait17	B cell
BC_Trait18	B cell
BC_Trait19	B cell
BC_Trait2	B cell
BC_Trait20	B cell
BC_Trait21	B cell
BC_Trait22	B cell
BC_Trait23	B cell
BC_Trait24	B cell
BC_Trait25	B cell
BC_Trait26	B cell
BC_Trait27	B cell
BC_Trait28	B cell
BC_Trait3	B cell
BC_Trait31	B cell
BC_Trait32	B cell
BC_Trait33	B cell
BC_Trait34	B cell
BC_Trait35	B cell
BC_Trait36	B cell
BC_Trait37	B cell
BC_Trait38	B cell
BC_Trait39	B cell
BC_Trait4	B cell
BC_Trait40	B cell
BC_Trait43	B cell
BC_Trait44	B cell
BC_Trait46	B cell
BC_Trait47	B cell
BC_Trait48	B cell
BC_Trait49	B cell
BC_Trait5	B cell
BC_Trait50	B cell
BC_Trait51	B cell
BC_Trait52	B cell
BC_Trait54	B cell
BC_Trait55	B cell
BC_Trait56	B cell
BC_Trait57	B cell

BC_Trait58	B cell
BC_Trait59	B cell
BC_Trait6	B cell
BC_Trait61	B cell
BC_Trait62	B cell
BC_Trait63	B cell
BC_Trait64	B cell
BC_Trait65	B cell
BC_Trait67	B cell
BC_Trait7	B cell
BC_Trait8	B cell
BC_Trait9	B cell
DC_Trait524	cDC
DC_Trait525	cDC
DC_Trait526	cDC
DC_Trait527	cDC
DC_Trait530	cDC
DC_Trait531	cDC
DC_Trait532	cDC
DC_Trait533	cDC
DC_Trait534	cDC
DC_Trait535	cDC
f_DC_Trait502	cDC
f_DC_Trait503	cDC
f_DC_Trait504	cDC
f_DC_Trait505	cDC
f_DC_Trait508	cDC
f_DC_Trait509	cDC
f_DC_Trait512	cDC
f_DC_Trait513	cDC
f_DC_Trait514	cDC
f_DC_Trait515	cDC
f_DC_Trait516	cDC
f_DC_Trait517	cDC
f_DC_Trait518	cDC
f_DC_Trait519	cDC
f_DC_Trait520	cDC
f_DC_Trait521	cDC
f_DC_Trait522	cDC
f_MONO_Trait503	TBNK
f_MONO_Trait504	TBNK
f_MONO_Trait505	TBNK
f_Treg_Trait501	Treg
f_Treg_Trait502	Treg
f_Treg_Trait503	Treg
f_Treg_Trait504	Treg
f_Treg_Trait505	Treg
f_Treg_Trait506	Treg

f_Treg_Trait507	Treg
f_Treg_Trait508	Treg
f_Treg_Trait509	Treg
f_Treg_Trait510	Treg
f_Treg_Trait511	Treg
f_Treg_Trait512	Treg
f_Treg_Trait513	Treg
f_Treg_Trait514	Treg
f_Treg_Trait515	Treg
f_Treg_Trait516	Treg
f_Treg_Trait517	Treg
f_Treg_Trait518	Treg
f_Treg_Trait519	Treg
f_Treg_Trait520	Treg
f_Treg_Trait521	Treg
f_Treg_Trait522	Treg
f_Treg_Trait523	Treg
f_Treg_Trait527	Treg
f_Treg_Trait528	Treg
f_Treg_Trait529	Treg
f_Treg_Trait533	Treg
f_Treg_Trait534	Treg
f_Treg_Trait535	Treg
f_Treg_Trait536	Treg
f_Treg_Trait537	Treg
f_Treg_Trait538	Treg
f_Treg_Trait539	Treg
f_Treg_Trait540	Treg
f_Treg_Trait541	Treg
f_Treg_Trait542	Treg
MDSC_Trait1	Myeloid cell
MDSC_Trait10	Myeloid cell
MDSC_Trait11	Myeloid cell
MDSC_Trait12	Myeloid cell
MDSC_Trait13	Myeloid cell
MDSC_Trait14	Myeloid cell
MDSC_Trait15	Myeloid cell
MDSC_Trait16	Myeloid cell
MDSC_Trait22	Myeloid cell
MDSC_Trait23	Myeloid cell
MDSC_Trait24	Myeloid cell
MDSC_Trait25	Myeloid cell
MDSC_Trait26	Myeloid cell
MDSC_Trait27	Myeloid cell
MDSC_Trait28	Myeloid cell
MDSC_Trait3	Myeloid cell
MDSC_Trait4	Myeloid cell
MDSC_Trait5	Myeloid cell

MDSC_Trait8	Myeloid cell
MDSC_Trait9	Myeloid cell
MT_ry_Trait514	Maturation stages of T cell
MT_ry_Trait515	Maturation stages of T cell
MT_ry_Trait516	Maturation stages of T cell
MT_Trait503	Maturation stages of T cell
MT_Trait504	Maturation stages of T cell
MT_Trait505	Maturation stages of T cell
MT_Trait506	Maturation stages of T cell
MT_Trait507	Maturation stages of T cell
MT_Trait508	Maturation stages of T cell
MT_Trait509	Maturation stages of T cell
MT_Trait510	Maturation stages of T cell
MT_Trait511	Maturation stages of T cell
MT_Trait512	Maturation stages of T cell
MT_Trait513	Maturation stages of T cell
MT_Trait522	Maturation stages of T cell
MT_Trait523	Maturation stages of T cell
MT_Trait524	Maturation stages of T cell
MT_Trait525	Maturation stages of T cell
MT_Trait526	Maturation stages of T cell
MT_Trait527	Maturation stages of T cell
MT_Trait528	Maturation stages of T cell
MT_Trait529	Maturation stages of T cell
MT_Trait530	Maturation stages of T cell
MT_Trait531	Maturation stages of T cell
MT_Trait532	Maturation stages of T cell
MT_Trait533	Maturation stages of T cell
MT_Trait537	Maturation stages of T cell
MT_Trait538	Maturation stages of T cell
MT_Trait539	Maturation stages of T cell
MT_Trait542	Maturation stages of T cell
MT_Trait543	Maturation stages of T cell
MT_Trait544	Maturation stages of T cell
MT_Trait545	Maturation stages of T cell
MT_Trait546	Maturation stages of T cell
MT_Trait547	Maturation stages of T cell
MT_Trait548	Maturation stages of T cell
MT_Trait549	Maturation stages of T cell
MT_Trait550	Maturation stages of T cell
MT_Trait551	Maturation stages of T cell
MT_Trait552	Maturation stages of T cell
MT_Trait553	Maturation stages of T cell
MT_Trait560	Maturation stages of T cell
N_BC_Trait3	B cell
N_BC_Trait4	B cell
N_BC_Trait6	B cell
N_MONO_Trait501	Monocyte

N_MONO_Trait503	Monocyte
N_MONO_Trait505	Monocyte
N_MONO_Trait507	Monocyte
N_MONO_Trait513	Monocyte
N_MONO_Trait514	Monocyte
N_MONO_Trait515	Monocyte
N_MONO_Trait516	Monocyte
N_MONO_Trait517	Monocyte
RATIO_CD3_B	TBNK
RATIO_CD4_CD8B	TBNK
TB_ry_Trait512	TBNK
TB_ry_Trait513	TBNK
TB_ry_Trait515	TBNK
TB_ry_Trait516	TBNK
TB_ry_Trait518	TBNK
TB_ry_Trait519	TBNK
TB_ry_Trait524	TBNK
TB_ry_Trait525	TBNK
TB_ry_Trait527	TBNK
TB_ry_Trait528	TBNK
TB_Trait501	TBNK
TB_Trait507	TBNK
TB_Trait508	TBNK
TB_Trait509	TBNK
TB_Trait510	TBNK
TB_Trait511	TBNK
TB_Trait514	TBNK
TB_Trait517	TBNK
TB_Trait520	TBNK
TB_Trait521	TBNK
TB_Trait522	TBNK
TB_Trait523	TBNK
TB_Trait526	TBNK
TB_Trait529	TBNK
TB_Trait532	TBNK
TB_Trait533	TBNK
TB_Trait534	TBNK
TB_Trait535	TBNK
TB_Trait536	TBNK
TB_Trait537	TBNK
TB_Trait538	TBNK
TB_Trait539	TBNK
TB_Trait540	TBNK
TB_Trait541	TBNK
TB_Trait542	TBNK
TB_Trait543	TBNK
TB_Trait544	TBNK
TB_Trait545	TBNK

TB_Trait546	TBNK
TB_Trait547	TBNK
TB_Trait548	TBNK
TB_Trait549	TBNK
TB_Trait550	TBNK
TB_Trait551	TBNK
TB_Trait552	TBNK
TB_Trait553	TBNK
TB_Trait554	TBNK
TB_Trait555	TBNK
TB_Trait556	TBNK
TB_Trait560	TBNK
TB_Trait561	TBNK
TB_Trait562	TBNK
TB_Trait563	TBNK
TB_Trait564	TBNK
TB_Trait565	TBNK
TB_Trait566	TBNK
TB_Trait567	TBNK
TB_Trait568	TBNK
TB_Trait569	TBNK
TB_Trait570	TBNK
TB_Trait571	TBNK
TB_Trait575	TBNK
TB_Trait576	TBNK
Treg_Trait505	Treg
Treg_Trait506	Treg
Treg_Trait507	Treg
Treg_Trait508	Treg
Treg_Trait509	Treg
Treg_Trait512	Treg
Treg_Trait513	Treg
Treg_Trait514	Treg
Treg_Trait574	Treg
Treg_Trait575	Treg
Treg_Trait576	Treg
Treg_Trait577	Treg
Treg_Trait578	Treg
Treg_Trait579	Treg
Treg_Trait580	Treg
Treg_Trait581	Treg
Treg_Trait582	Treg
Treg_Trait585	Treg
Treg_Trait586	Treg
Treg_Trait587	Treg
Treg_Trait588	Treg
Treg_Trait589	Treg
Treg_Trait590	Treg

Treg_Trait591	Treg
Treg_Trait592	Treg
Treg_Trait593	Treg
Treg_Trait594	Treg
Treg_Trait595	Treg
Treg_Trait596	Treg
Treg_Trait597	Treg
Treg_Trait598	Treg
Treg_Trait599	Treg
Treg_Trait600	Treg
Treg_Trait601	Treg
Treg_Trait602	Treg
Treg_Trait603	Treg
Treg_Trait604	Treg
Treg_Trait605	Treg
Treg_Trait606	Treg
Treg_Trait607	Treg
Treg_Trait608	Treg
Treg_Trait611	Treg
Treg_Trait700	Treg
Treg_Trait701	Treg
Treg_Trait702	Treg
Treg_Trait706	Treg
Treg_Trait707	Treg
Treg_Trait708	Treg
Bcells.trait111	B cell
Bcells.trait112	B cell
Bcells.trait137	B cell
Bcells.trait138	B cell
Bcells.trait139	B cell
Bcells.trait140	B cell
Bcells.trait141	B cell
Bcells.trait142	B cell
Bcells.trait143	B cell
Bcells.trait147	B cell
Bcells.trait148	B cell
Bcells.trait149	B cell
Bcells.trait150	B cell
Bcells.trait151	B cell
Bcells.trait154	B cell
Bcells.trait156	B cell
Bcells.trait157	B cell
Bcells.trait159	B cell
Bcells.trait160	B cell
Bcells.trait161	B cell
Bcells.trait255	B cell
Bcells.trait282	B cell
Bcells.trait283	B cell

Bcells.trait308	B cell
Bcells.trait309	B cell
Bcells.trait310	B cell
Bcells.trait311	B cell
Bcells.trait312	B cell
Bcells.trait313	B cell
Bcells.trait314	B cell
Bcells.trait318	B cell
Bcells.trait319	B cell
Bcells.trait320	B cell
Bcells.trait321	B cell
Bcells.trait322	B cell
Bcells.trait325	B cell
Bcells.trait327	B cell
Bcells.trait328	B cell
Bcells.trait329	B cell
Bcells.trait331	B cell
Bcells.trait332	B cell
Bcells.trait333	B cell
Bcells.trait335	B cell
Bcells.trait354	B cell
Bcells.trait355	B cell
Bcells.trait378	B cell
Bcells.trait379	B cell
Bcells.trait380	B cell
Bcells.trait381	B cell
Bcells.trait382	B cell
Bcells.trait383	B cell
Bcells.trait384	B cell
Bcells.trait388	B cell
Bcells.trait389	B cell
Bcells.trait390	B cell
Bcells.trait391	B cell
Bcells.trait392	B cell
Bcells.trait395	B cell
Bcells.trait397	B cell
Bcells.trait398	B cell
Bcells.trait399	B cell
Bcells.trait400	B cell
Bcells.trait401	B cell
Bcells.trait418	B cell
Bcells.trait438	B cell
Bcells.trait439	B cell
Bcells.trait441	B cell
Bcells.trait442	B cell
Bcells.trait445	B cell
Bcells.trait446	B cell
Bcells.trait448	B cell

Bcells.trait449	B cell
Bcells.trait450	B cell
Bcells.trait451	B cell
Bcells.trait453	B cell
Bcells.trait470	B cell
Bcells.trait471	B cell
Bcells.trait490	B cell
Bcells.trait491	B cell
Bcells.trait492	B cell
Bcells.trait493	B cell
Bcells.trait494	B cell
Bcells.trait495	B cell
Bcells.trait496	B cell
Bcells.trait500	B cell
Bcells.trait501	B cell
Bcells.trait502	B cell
Bcells.trait503	B cell
Bcells.trait504	B cell
Bcells.trait506	B cell
Bcells.trait508	B cell
Bcells.trait509	B cell
Bcells.trait510	B cell
Bcells.trait511	B cell
Bcells.trait512	B cell
Bcells.trait514	B cell
Bcells.trait536	B cell
Bcells.trait537	B cell
Bcells.trait539	B cell
Bcells.trait561	B cell
Bcells.trait563	B cell
Bcells.trait565	B cell
Bcells.trait566	B cell
Bcells.trait567	B cell
Bcells.trait570	B cell
Bcells.trait571	B cell
Bcells.trait572	B cell
Bcells.trait574	B cell
Bcells.trait597	B cell
Bcells.trait619	B cell
Bcells.trait637	B cell
Bcells.trait640	B cell
Bcells.trait641	B cell
Bcells.trait643	B cell
Bcells.trait644	B cell
Bcells.trait646	B cell
Bcells.trait647	B cell
Bcells.trait649	B cell
Bcells.trait650	B cell

Bcells.trait775	B cell
Bcells.trait776	B cell
Bcells.trait777	B cell
Bcells.trait779	B cell
Bcells.trait780	B cell
Bcells.trait781	B cell
Bcells.trait785	B cell
Bcells.trait786	B cell
Bcells.trait787	B cell
Bcells.trait84	B cell
Bcells.trait85	B cell
Blue.530.30.cDC.trait1	cDC
Blue.530.30.cDC.trait2	cDC
Blue.530.30.cDC.trait3	cDC
Blue.530.30.cDC.trait4	cDC
Blue.530.30.cDC.trait5	cDC
Blue.530.30.MDSC.trait1	Myeloid cell
Blue.530.30.MDSC.trait2	Myeloid cell
Blue.530.30.MT.trait10	Maturation stages of T cell
Blue.530.30.MT.trait11	Maturation stages of T cell
Blue.530.30.MT.trait12	Maturation stages of T cell
Blue.530.30.MT.trait3	Maturation stages of T cell
Blue.530.30.MT.trait4	Maturation stages of T cell
Blue.530.30.MT.trait5	Maturation stages of T cell
Blue.530.30.MT.trait6	Maturation stages of T cell
Blue.530.30.MT.trait7	Maturation stages of T cell
Blue.530.30.MT.trait9	Maturation stages of T cell
Blue.530.30.TBNK.trait4	TBNK
Blue.530.30.TBNK.trait5	TBNK
Blue.530.30.TBNK.trait6	TBNK
Blue.530.30.TBNK.trait7	TBNK
Blue.530.30.Treg.trait1	Treg
Blue.530.30.Treg.trait10	Treg
Blue.530.30.Treg.trait11	Treg
Blue.530.30.Treg.trait12	Treg
Blue.530.30.Treg.trait13	Treg
Blue.530.30.Treg.trait14	Treg
Blue.530.30.Treg.trait15	Treg
Blue.530.30.Treg.trait17	Treg
Blue.530.30.Treg.trait2	Treg
Blue.530.30.Treg.trait21	Treg
Blue.530.30.Treg.trait22	Treg
Blue.530.30.Treg.trait23	Treg
Blue.530.30.Treg.trait3	Treg
Blue.530.30.Treg.trait4	Treg
Blue.530.30.Treg.trait5	Treg
Blue.530.30.Treg.trait6	Treg
Blue.530.30.Treg.trait7	Treg

Blue.530.30.Treg.trait8	Treg
Blue.530.30.Treg.trait9	Treg
Blue.585.42.MDSC.trait1	Myeloid cell
HVEM1000_150630.trait1	Maturation stages of T cell
HVEM1000_150630.trait10	Maturation stages of T cell
HVEM1000_150630.trait11	Maturation stages of T cell
HVEM1000_150630.trait12	Maturation stages of T cell
HVEM1000_150630.trait2	Maturation stages of T cell
HVEM1000_150630.trait3	Maturation stages of T cell
HVEM1000_150630.trait4	Maturation stages of T cell
HVEM1000_150630.trait5	Maturation stages of T cell
HVEM1000_150630.trait6	Maturation stages of T cell
HVEM1000_150630.trait7	Maturation stages of T cell
HVEM1000_150630.trait8	Maturation stages of T cell
HVEM1000_150630.trait9	Maturation stages of T cell
Blue.585.42.TBNK.trait1	TBNK
Blue.585.42.TBNK.trait2	TBNK
Blue.585.42.TBNK.trait3	TBNK
Blue.585.42.Treg.trait10	Treg
Blue.585.42.Treg.trait11	Treg
Blue.585.42.Treg.trait12	Treg
Blue.585.42.Treg.trait13	Treg
Blue.585.42.Treg.trait15	Treg
Blue.585.42.Treg.trait18	Treg
Blue.585.42.Treg.trait19	Treg
Blue.585.42.Treg.trait2	Treg
Blue.585.42.Treg.trait20	Treg
Blue.585.42.Treg.trait21	Treg
Blue.585.42.Treg.trait3	Treg
Blue.585.42.Treg.trait4	Treg
Blue.585.42.Treg.trait5	Treg
Blue.585.42.Treg.trait6	Treg
Blue.585.42.Treg.trait7	Treg
Blue.585.42.Treg.trait8	Treg
Blue.585.42.Treg.trait9	Treg
Blue.670.LP.cDC.trait1	cDC
Blue.670.LP.cDC.trait2	cDC
Blue.670.LP.cDC.trait3	cDC
Blue.670.LP.cDC.trait4	cDC
Blue.670.LP.MT.trait1	Maturation stages of T cell
Blue.670.LP.MT.trait3	Maturation stages of T cell
Blue.670.LP.TBNK.trait1	TBNK
Blue.670.LP.TBNK.trait10	TBNK
Blue.670.LP.TBNK.trait11	TBNK
Blue.670.LP.TBNK.trait12	TBNK
Blue.670.LP.TBNK.trait13	TBNK
Blue.670.LP.TBNK.trait2	TBNK
Blue.670.LP.TBNK.trait3	TBNK

Blue.670.LP.TBNK.trait4	TBNK
Blue.670.LP.TBNK.trait5	TBNK
Blue.670.LP.TBNK.trait6	TBNK
Blue.670.LP.TBNK.trait7	TBNK
Blue.670.LP.TBNK.trait8	TBNK
Blue.670.LP.TBNK.trait9	TBNK
Blue.670.LP.Treg.trait1	Treg
Blue.670.LP.Treg.trait10	Treg
Blue.670.LP.Treg.trait11	Treg
Blue.670.LP.Treg.trait12	Treg
Blue.670.LP.Treg.trait13	Treg
Blue.670.LP.Treg.trait2	Treg
Blue.670.LP.Treg.trait3	Treg
Blue.670.LP.Treg.trait4	Treg
Blue.670.LP.Treg.trait5	Treg
Blue.670.LP.Treg.trait6	Treg
Blue.670.LP.Treg.trait7	Treg
Blue.780.60.CD25.trait1	Treg
Blue.780.60.CD25.trait13	Treg
Blue.780.60.CD25.trait10	Treg
Blue.780.60.CD25.trait2	Treg
Blue.780.60.CD25.trait3	Treg
Blue.780.60.CD25.trait4	Treg
Blue.780.60.CD25.trait5	Treg
Blue.780.60.CD25.trait6	Treg
Blue.780.60.CD25.trait7	Treg
Blue.780.60.CD25.trait8	Treg
Blue.780.60.CD25.trait9	Treg
Blue.780.60.cDC.trait1	cDC
Blue.780.60.cDC.trait2	cDC
Blue.780.60.MDSC.trait1	Myeloid cell
Blue.780.60.MDSC.trait10	Myeloid cell
Blue.780.60.MDSC.trait12	Myeloid cell
Blue.780.60.MDSC.trait13	Myeloid cell
Blue.780.60.MDSC.trait14	Myeloid cell
Blue.780.60.MDSC.trait2	Myeloid cell
Blue.780.60.MDSC.trait3	Myeloid cell
Blue.780.60.MDSC.trait4	Myeloid cell
Blue.780.60.MDSC.trait6	Myeloid cell
Blue.780.60.MDSC.trait7	Myeloid cell
Blue.780.60.MDSC.trait8	Myeloid cell
Blue.780.60.MDSC.trait9	Myeloid cell
Blue.780.60.TBNK.trait1	TBNK
Blue.780.60.TBNK.trait3	TBNK
Blue.780.60.Treg.trait4	Treg
Blue.780.60.Treg.trait5	Treg
Blue.780.60.Treg.trait6	Treg
FSC.cDC.trait2	cDC

FSC.cDC.trait3	cDC
FSC.cDC.trait4	cDC
FSC.cDC.trait5	cDC
FSC.TBNK.trait1	TBNK
FSC.TBNK.trait10	TBNK
FSC.TBNK.trait11	TBNK
FSC.TBNK.trait12	TBNK
FSC.TBNK.trait2	TBNK
FSC.TBNK.trait3	TBNK
FSC.TBNK.trait4	TBNK
FSC.TBNK.trait5	TBNK
FSC.TBNK.trait6	TBNK
FSC.TBNK.trait7	TBNK
FSC.TBNK.trait8	TBNK
FSC.TBNK.trait9	TBNK
mono.trait1	Monocyte
mono.trait10	Monocyte
mono.trait11	Monocyte
mono.trait12	Monocyte
mono.trait14	Monocyte
mono.trait16	Monocyte
mono.trait17	Monocyte
mono.trait19	Monocyte
mono.trait2	Monocyte
mono.trait21	Monocyte
mono.trait22	Monocyte
mono.trait24	Monocyte
mono.trait25	Monocyte
mono.trait28	Monocyte
mono.trait29	Monocyte
mono.trait3	Monocyte
mono.trait33	Monocyte
mono.trait35	Monocyte
mono.trait36	Monocyte
mono.trait39	Monocyte
mono.trait4	Monocyte
mono.trait41	Monocyte
mono.trait42	Monocyte
mono.trait43	Monocyte
mono.trait44	Monocyte
mono.trait46	Monocyte
mono.trait47	Monocyte
mono.trait48	Monocyte
mono.trait50	Monocyte
mono.trait52	Monocyte
mono.trait53	Monocyte
mono.trait54	Monocyte
mono.trait6	Monocyte

mono.trait7	Monocyte
Red.660.20.cDC.trait1	cDC
Red.660.20.cDC.trait2	cDC
Red.660.20.cDC.trait3	cDC
Red.660.20.cDC.trait4	cDC
Red.660.20.cDC.trait5	cDC
Red.660.20.cDC.trait6	cDC
Red.660.20.MDSC.trait2	Myeloid cell
Red.660.20.MDSC.trait3	Myeloid cell
Red.660.20.MDSC.trait4	Myeloid cell
Red.660.20.MT.trait1	Maturation stages of T cell
Red.660.20.MT.trait2	Maturation stages of T cell
Red.660.20.MT.trait3	Maturation stages of T cell
Red.660.20.MT.trait4	Maturation stages of T cell
Red.660.20.MT.trait5	Maturation stages of T cell
Red.660.20.MT.trait6	Maturation stages of T cell
Red.660.20.TBNK.trait2	TBNK
Red.660.20.Treg.trait1	Treg
Red.660.20.Treg.trait2	Treg
Red.660.20.Treg.trait3	Treg
Red.660.20.Treg.trait5	Treg
Red.660.20.Treg.trait6	Treg
Red.660.20.Treg.trait7	Treg
Red.780.60.cDC.trait1	cDC
Red.780.60.cDC.trait2	cDC
Red.780.60.cDC.trait3	cDC
Red.780.60.cDC.trait4	cDC
Red.780.60.cDC.trait5	cDC
Red.780.60.cDC.trait6	cDC
Red.780.60.MDSC.trait1	Myeloid cell
Red.780.60.MDSC.trait10	Myeloid cell
Red.780.60.MDSC.trait11	Myeloid cell
Red.780.60.MDSC.trait13	Myeloid cell
Red.780.60.MDSC.trait14	Myeloid cell
Red.780.60.MDSC.trait15	Myeloid cell
Red.780.60.MDSC.trait16	Myeloid cell
Red.780.60.MDSC.trait3	Myeloid cell
Red.780.60.MDSC.trait4	Myeloid cell
Red.780.60.MDSC.trait5	Myeloid cell
Red.780.60.MDSC.trait7	Myeloid cell
Red.780.60.MDSC.trait8	Myeloid cell
Red.780.60.MDSC.trait9	Myeloid cell
Red.780.60.MT.trait2	Maturation stages of T cell
Red.780.60.MT.trait3	Maturation stages of T cell
Red.780.60.MT.trait4	Maturation stages of T cell
Red.780.60.MT.trait5	Maturation stages of T cell
Red.780.60.TBNK.trait1	TBNK
Red.780.60.TBNK.trait2	TBNK

Red.780.60.TBNK.trait3	TBNK
Red.780.60.Treg.trait15	Treg
Red.780.60.Treg.trait16	Treg
Red.780.60.Treg.trait2	Treg
Red.780.60.Treg.trait3	Treg
Red.780.60.Treg.trait4	Treg
Red.780.60.Treg.trait5	Treg
Red.780.60.Treg.trait6	Treg
Red.780.60.Treg.trait7	Treg
Red.780.60.Treg.trait8	Treg
Red.780.60.Treg.trait9	Treg
SSC.cDC.trait2	cDC
SSC.cDC.trait3	cDC
SSC.cDC.trait4	cDC
SSC.TBNK.trait1	TBNK
SSC.TBNK.trait10	TBNK
SSC.TBNK.trait11	TBNK
SSC.TBNK.trait12	TBNK
SSC.TBNK.trait13	TBNK
SSC.TBNK.trait2	TBNK
SSC.TBNK.trait3	TBNK
SSC.TBNK.trait4	TBNK
SSC.TBNK.trait5	TBNK
SSC.TBNK.trait6	TBNK
SSC.TBNK.trait7	TBNK
SSC.TBNK.trait8	TBNK
SSC.TBNK.trait9	TBNK
Violet.450.50.cDC.trait1	cDC
Violet.450.50.cDC.trait2	cDC
Violet.450.50.cDC.trait3	cDC
Violet.450.50.cDC.trait4	cDC
Violet.450.50.MDSC.trait1	Myeloid cell
Violet.450.50.MDSC.trait13	Myeloid cell
Violet.450.50.MDSC.trait2	Myeloid cell
Violet.450.50.MDSC.trait3	Myeloid cell
Violet.450.50.MDSC.trait4	Myeloid cell
Violet.450.50.MDSC.trait6	Myeloid cell
Violet.450.50.MDSC.trait9	Myeloid cell
Violet.450.50.MT.trait1	Maturation stages of T cell
Violet.450.50.MT.trait2	Maturation stages of T cell
Violet.450.50.MT.trait3	Maturation stages of T cell
Violet.450.50.MT.trait4	Maturation stages of T cell
Violet.450.50.Treg.trait2	Treg
Violet.450.50.Treg.trait3	Treg
Violet.510.50.cDC.trait2	cDC
Violet.510.50.cDC.trait3	cDC
Violet.510.50.cDC.trait4	cDC
Violet.510.50.MDSC.trait1	Myeloid cell

Violet.510.50.MDSC.trait3	Myeloid cell
Violet.510.50.MDSC.trait4	Myeloid cell
Violet.510.50.MDSC.trait5	Myeloid cell
Violet.510.50.MDSC.trait6	Myeloid cell
Violet.510.50.MDSC.trait7	Myeloid cell
Violet.510.50.TBNK.trait2	TBNK
Violet.510.50.TBNK.trait3	TBNK
Violet.510.50.TBNK.trait4	TBNK
Violet.510.50.TBNK.trait5	TBNK
Violet.510.50.TBNK.trait6	TBNK
Violet.510.50.Treg.trait2	Treg
Violet.510.50.Treg.trait3	Treg
Violet.510.50.Treg.trait4	Treg
Violet.510.50.Treg.trait5	Treg

Genetic instrumenta

Id.exposure	SNP	exposure	Chr	Pos
ebi-a-GCST90001391	rs72757888	IgD+ %B cell	1	233045963
ebi-a-GCST90001391	rs56209800	IgD+ %B cell	2	238560295
ebi-a-GCST90001391	rs7597061	IgD+ %B cell	2	66001846
ebi-a-GCST90001391	rs138919897	IgD+ %B cell	3	106834795
ebi-a-GCST90001391	rs4691917	IgD+ %B cell	4	164344795
ebi-a-GCST90001391	rs17405603	IgD+ %B cell	5	144608340
ebi-a-GCST90001391	rs72952600	IgD+ %B cell	6	114522609
ebi-a-GCST90001391	rs9265870	IgD+ %B cell	6	31312892
ebi-a-GCST90001391	rs12204136	IgD+ %B cell	6	91791081
ebi-a-GCST90001391	rs10226722	IgD+ %B cell	7	68025695
ebi-a-GCST90001391	rs2450220	IgD+ %B cell	8	119323401
ebi-a-GCST90001391	rs140191306	IgD+ %B cell	9	2570753
ebi-a-GCST90001391	rs11068290	IgD+ %B cell	12	110228055
ebi-a-GCST90001391	rs11616703	IgD+ %B cell	13	22879729
ebi-a-GCST90001391	rs62057183	IgD+ %B cell	16	30744826
ebi-a-GCST90001391	rs73426855	IgD+ %B cell	17	77637123
ebi-a-GCST90001391	rs148496020	IgD+ %B cell	17	51092001
ebi-a-GCST90001392	rs1919856	IgD+ CD38br AC	1	247700906
ebi-a-GCST90001392	rs116285595	IgD+ CD38br AC	1	200858159
ebi-a-GCST90001392	rs116104104	IgD+ CD38br AC	1	214476260
ebi-a-GCST90001392	rs12036556	IgD+ CD38br AC	1	99149370
ebi-a-GCST90001392	rs1294253	IgD+ CD38br AC	1	233503875
ebi-a-GCST90001392	rs146959444	IgD+ CD38br AC	1	167027268
ebi-a-GCST90001392	rs113965271	IgD+ CD38br AC	2	128428350
ebi-a-GCST90001392	rs733555	IgD+ CD38br AC	2	16464227
ebi-a-GCST90001392	rs78188887	IgD+ CD38br AC	2	155636739
ebi-a-GCST90001392	rs140380950	IgD+ CD38br AC	2	105587803
ebi-a-GCST90001392	rs374727947	IgD+ CD38br AC	3	163801921
ebi-a-GCST90001392	rs794185	IgD+ CD38br AC	3	4437358
ebi-a-GCST90001392	rs76112278	IgD+ CD38br AC	4	15915069
ebi-a-GCST90001392	rs7667070	IgD+ CD38br AC	4	38379833
ebi-a-GCST90001392	rs80330977	IgD+ CD38br AC	5	158276610
ebi-a-GCST90001392	rs78658073	IgD+ CD38br AC	5	498773
ebi-a-GCST90001392	rs76660971	IgD+ CD38br AC	5	18919526
ebi-a-GCST90001392	rs117772462	IgD+ CD38br AC	7	48791017
ebi-a-GCST90001392	rs10265656	IgD+ CD38br AC	7	187171
ebi-a-GCST90001392	rs143397127	IgD+ CD38br AC	7	12847391
ebi-a-GCST90001392	rs1695681	IgD+ CD38br AC	8	118255950
ebi-a-GCST90001392	rs16868356	IgD+ CD38br AC	8	102770464
ebi-a-GCST90001392	rs2395904	IgD+ CD38br AC	8	130617819
ebi-a-GCST90001392	rs10821937	IgD+ CD38br AC	10	63723909
ebi-a-GCST90001392	rs61878035	IgD+ CD38br AC	11	15328898
ebi-a-GCST90001392	rs11068290	IgD+ CD38br AC	12	110228055
ebi-a-GCST90001392	rs118090735	IgD+ CD38br AC	12	78790468
ebi-a-GCST90001392	rs11159480	IgD+ CD38br AC	14	81466662

ebi-a-GCST90001392	rs7169431	IgD+ CD38br AC	15	56340896
ebi-a-GCST90001392	rs76608654	IgD+ CD38br AC	16	55450479
ebi-a-GCST90001392	rs12939565	IgD+ CD38br AC	17	38038389
ebi-a-GCST90001393	rs75766272	IgD+ CD38dim %B	1	8009985
ebi-a-GCST90001393	rs7595353	IgD+ CD38dim %B	2	229232050
ebi-a-GCST90001393	rs9865821	IgD+ CD38dim %B	3	7014131
ebi-a-GCST90001393	rs6835759	IgD+ CD38dim %B	4	170328209
ebi-a-GCST90001393	rs2301101	IgD+ CD38dim %B	5	83420407
ebi-a-GCST90001393	rs9459562	IgD+ CD38dim %B	6	166502048
ebi-a-GCST90001393	rs6998850	IgD+ CD38dim %B	8	61848028
ebi-a-GCST90001393	rs546147740	IgD+ CD38dim %B	8	4318676
ebi-a-GCST90001393	rs62488766	IgD+ CD38dim %B	8	12518295
ebi-a-GCST90001393	rs61912200	IgD+ CD38dim %B	12	1637126
ebi-a-GCST90001393	rs78269238	IgD+ CD38dim %B	14	65673804
ebi-a-GCST90001393	rs60521331	IgD+ CD38dim %B	17	8639905
ebi-a-GCST90001393	rs34157595	IgD+ CD38dim %B	17	49200947
ebi-a-GCST90001393	rs329966	IgD+ CD38dim %B	19	53070617
ebi-a-GCST90001393	rs4811007	IgD+ CD38dim %B	20	48839604
ebi-a-GCST90001393	rs73909843	IgD+ CD38dim %B	20	49554764
ebi-a-GCST90001393	rs78958229	IgD+ CD38dim %B	21	39363957
ebi-a-GCST90001394	rs72691696	IgD+ CD38dim AC	1	166598682
ebi-a-GCST90001394	rs7690529	IgD+ CD38dim AC	4	91412392
ebi-a-GCST90001394	rs147207149	IgD+ CD38dim AC	5	128786753
ebi-a-GCST90001394	rs17056318	IgD+ CD38dim AC	5	158285953
ebi-a-GCST90001394	rs1510970	IgD+ CD38dim AC	5	118216738
ebi-a-GCST90001394	rs115150472	IgD+ CD38dim AC	5	146138788
ebi-a-GCST90001394	rs141880653	IgD+ CD38dim AC	5	166970737
ebi-a-GCST90001394	rs115951641	IgD+ CD38dim AC	6	52336715
ebi-a-GCST90001394	rs12197153	IgD+ CD38dim AC	6	100821460
ebi-a-GCST90001394	rs143397127	IgD+ CD38dim AC	7	12847391
ebi-a-GCST90001394	rs190448231	IgD+ CD38dim AC	7	87868543
ebi-a-GCST90001394	rs2395904	IgD+ CD38dim AC	8	130617819
ebi-a-GCST90001394	rs283340	IgD+ CD38dim AC	8	70193536
ebi-a-GCST90001394	rs150663332	IgD+ CD38dim AC	8	2044843
ebi-a-GCST90001394	rs139801247	IgD+ CD38dim AC	9	138747854
ebi-a-GCST90001394	rs116851238	IgD+ CD38dim AC	10	33792014
ebi-a-GCST90001394	rs12799241	IgD+ CD38dim AC	11	119980729
ebi-a-GCST90001394	rs75474045	IgD+ CD38dim AC	13	72428296
ebi-a-GCST90001394	rs12906621	IgD+ CD38dim AC	15	37177413
ebi-a-GCST90001394	rs117518951	IgD+ CD38dim AC	16	55449193
ebi-a-GCST90001394	rs143176410	IgD+ CD38dim AC	16	84305239
ebi-a-GCST90001394	rs8076302	IgD+ CD38dim AC	17	79265701
ebi-a-GCST90001394	rs12939566	IgD+ CD38dim AC	17	38038390
ebi-a-GCST90001394	rs4792571	IgD+ CD38dim AC	17	14931234
ebi-a-GCST90001394	rs655580	IgD+ CD38dim AC	19	53039110
ebi-a-GCST90001394	rs34764986	IgD+ CD38dim AC	20	59926554
ebi-a-GCST90001395	rs12122084	IgD+ CD38- %B ce	1	111914431
ebi-a-GCST90001395	rs1512044	IgD+ CD38- %B ce	3	188624425

ebi-a-GCST90001395	rs76376526	IgD+ CD38- %B ce	4	109408667
ebi-a-GCST90001395	rs78009119	IgD+ CD38- %B ce	5	136421498
ebi-a-GCST90001395	rs34843244	IgD+ CD38- %B ce	5	90267368
ebi-a-GCST90001395	rs115802369	IgD+ CD38- %B ce	6	5380890
ebi-a-GCST90001395	rs2621369	IgD+ CD38- %B ce	6	32768285
ebi-a-GCST90001395	rs113398446	IgD+ CD38- %B ce	6	161871755
ebi-a-GCST90001395	rs80032720	IgD+ CD38- %B ce	6	32384933
ebi-a-GCST90001395	rs10254361	IgD+ CD38- %B ce	7	119564205
ebi-a-GCST90001395	rs2251199	IgD+ CD38- %B ce	8	14671281
ebi-a-GCST90001395	rs62502412	IgD+ CD38- %B ce	8	28265475
ebi-a-GCST90001395	rs10111625	IgD+ CD38- %B ce	8	79595404
ebi-a-GCST90001395	rs7012048	IgD+ CD38- %B ce	8	29631839
ebi-a-GCST90001395	rs148952274	IgD+ CD38- %B ce	9	37982527
ebi-a-GCST90001395	rs79870102	IgD+ CD38- %B ce	12	66341509
ebi-a-GCST90001395	rs1959342	IgD+ CD38- %B ce	14	20673865
ebi-a-GCST90001395	rs77762131	IgD+ CD38- %B ce	14	96205838
ebi-a-GCST90001395	rs10444793	IgD+ CD38- %B ce	15	44517689
ebi-a-GCST90001395	rs147135384	IgD+ CD38- %B ce	15	40195637
ebi-a-GCST90001395	rs11150426	IgD+ CD38- %B ce	16	81986583
ebi-a-GCST90001395	rs181771926	IgD+ CD38- %B ce	16	52294553
ebi-a-GCST90001395	rs329966	IgD+ CD38- %B ce	19	53070617
ebi-a-GCST90001395	rs73909843	IgD+ CD38- %B ce	20	49554764
ebi-a-GCST90001395	rs35050775	IgD+ CD38- %B ce	20	8003880
ebi-a-GCST90001396	rs10493766	IgD+ CD38- AC	1	85945660
ebi-a-GCST90001396	rs149701635	IgD+ CD38- AC	3	44741083
ebi-a-GCST90001396	rs79207264	IgD+ CD38- AC	3	96406408
ebi-a-GCST90001396	rs71615254	IgD+ CD38- AC	4	178658413
ebi-a-GCST90001396	rs146127735	IgD+ CD38- AC	5	502938
ebi-a-GCST90001396	rs1158784	IgD+ CD38- AC	6	32758761
ebi-a-GCST90001396	rs12216018	IgD+ CD38- AC	6	130514590
ebi-a-GCST90001396	rs7809268	IgD+ CD38- AC	7	96302607
ebi-a-GCST90001396	rs144625305	IgD+ CD38- AC	7	7871676
ebi-a-GCST90001396	rs10866982	IgD+ CD38- AC	8	5127400
ebi-a-GCST90001396	rs12763172	IgD+ CD38- AC	10	85447519
ebi-a-GCST90001396	rs7909504	IgD+ CD38- AC	10	32174610
ebi-a-GCST90001396	rs146687492	IgD+ CD38- AC	11	125619694
ebi-a-GCST90001396	rs12874404	IgD+ CD38- AC	13	108993494
ebi-a-GCST90001396	rs77762131	IgD+ CD38- AC	14	96205838
ebi-a-GCST90001396	rs10444793	IgD+ CD38- AC	15	44517689
ebi-a-GCST90001396	rs1529727	IgD+ CD38- AC	19	11164067
ebi-a-GCST90001396	rs62235484	IgD+ CD38- AC	21	41083050
ebi-a-GCST90001397	rs7412746	Unsw mem %B cell	1	150860471
ebi-a-GCST90001397	rs146430312	Unsw mem %B cell	1	236060214
ebi-a-GCST90001397	rs75766272	Unsw mem %B cell	1	8009985
ebi-a-GCST90001397	rs72686460	Unsw mem %B cell	1	45867753
ebi-a-GCST90001397	rs77201712	Unsw mem %B cell	2	157311822
ebi-a-GCST90001397	rs17006489	Unsw mem %B cell	2	28304630
ebi-a-GCST90001397	rs72844588	Unsw mem %B cell	2	138420154

ebi-a-GCST90001397	rs45608038	Unsw mem %B cell	2	73679013
ebi-a-GCST90001397	rs73041424	Unsw mem %B cell	3	16414269
ebi-a-GCST90001397	rs183471524	Unsw mem %B cell	4	27108645
ebi-a-GCST90001397	rs116525939	Unsw mem %B cell	5	97611924
ebi-a-GCST90001397	rs895377	Unsw mem %B cell	5	58050530
ebi-a-GCST90001397	rs73032183	Unsw mem %B cell	6	169456830
ebi-a-GCST90001397	rs4710879	Unsw mem %B cell	6	170426027
ebi-a-GCST90001397	rs12539793	Unsw mem %B cell	7	25971860
ebi-a-GCST90001397	rs11779181	Unsw mem %B cell	8	9063027
ebi-a-GCST90001397	rs1679013	Unsw mem %B cell	9	22206987
ebi-a-GCST90001397	rs1930922	Unsw mem %B cell	9	93297086
ebi-a-GCST90001397	rs12380223	Unsw mem %B cell	9	7639660
ebi-a-GCST90001397	rs143553941	Unsw mem %B cell	12	109368119
ebi-a-GCST90001397	rs11519226	Unsw mem %B cell	12	20322449
ebi-a-GCST90001397	rs7322292	Unsw mem %B cell	13	51370899
ebi-a-GCST90001397	rs73192651	Unsw mem %B cell	13	50212885
ebi-a-GCST90001397	rs2793491	Unsw mem %B cell	13	24826051
ebi-a-GCST90001397	rs73518104	Unsw mem %B cell	13	70177578
ebi-a-GCST90001397	rs117004076	Unsw mem %B cell	14	97702226
ebi-a-GCST90001397	rs77762131	Unsw mem %B cell	14	96205838
ebi-a-GCST90001397	rs143666202	Unsw mem %B cell	14	77579227
ebi-a-GCST90001397	rs150839591	Unsw mem %B cell	17	260039
ebi-a-GCST90001397	rs1596383	Unsw mem %B cell	18	7993103
ebi-a-GCST90001397	rs1883832	Unsw mem %B cell	20	44746982
ebi-a-GCST90001397	rs138380722	Unsw mem %B cell	20	56790125
ebi-a-GCST90001397	rs146720368	Unsw mem %B cell	22	32003463
ebi-a-GCST90001398	rs75766272	Unsw mem AC	1	8009985
ebi-a-GCST90001398	rs11737576	Unsw mem AC	4	55923201
ebi-a-GCST90001398	rs79831025	Unsw mem AC	6	40981212
ebi-a-GCST90001398	rs4076316	Unsw mem AC	6	167859623
ebi-a-GCST90001398	rs144625305	Unsw mem AC	7	7871676
ebi-a-GCST90001398	rs2436878	Unsw mem AC	8	103767171
ebi-a-GCST90001398	rs118092259	Unsw mem AC	8	89432719
ebi-a-GCST90001398	rs11794578	Unsw mem AC	9	808529
ebi-a-GCST90001398	rs112839090	Unsw mem AC	12	119727983
ebi-a-GCST90001398	rs12874404	Unsw mem AC	13	108993494
ebi-a-GCST90001398	rs557577736	Unsw mem AC	13	46224092
ebi-a-GCST90001398	rs145371905	Unsw mem AC	13	70325772
ebi-a-GCST90001398	rs73192651	Unsw mem AC	13	50212885
ebi-a-GCST90001398	rs77762131	Unsw mem AC	14	96205838
ebi-a-GCST90001398	rs143666202	Unsw mem AC	14	77579227
ebi-a-GCST90001398	rs8014978	Unsw mem AC	14	94936964
ebi-a-GCST90001398	rs880311	Unsw mem AC	16	8963504
ebi-a-GCST90001398	rs11657118	Unsw mem AC	17	2714118
ebi-a-GCST90001398	rs12461001	Unsw mem AC	19	14885525
ebi-a-GCST90001398	rs7254039	Unsw mem AC	19	14993354
ebi-a-GCST90001398	rs6032660	Unsw mem AC	20	44730245
ebi-a-GCST90001398	rs55712190	Unsw mem AC	22	22613458

ebi-a-GCST90001399	rs76625665	IgD- CD27- %B cel	1	233049739
ebi-a-GCST90001399	rs116473015	IgD- CD27- %B cel	2	147859010
ebi-a-GCST90001399	rs9842571	IgD- CD27- %B cel	3	125931267
ebi-a-GCST90001399	rs35574803	IgD- CD27- %B cel	3	32533246
ebi-a-GCST90001399	rs79430021	IgD- CD27- %B cel	4	25037515
ebi-a-GCST90001399	rs35661323	IgD- CD27- %B cel	7	44284783
ebi-a-GCST90001399	rs2250246	IgD- CD27- %B cel	12	6553016
ebi-a-GCST90001399	rs7224129	IgD- CD27- %B cel	17	38075426
ebi-a-GCST90001399	rs11658240	IgD- CD27- %B cel	17	80484139
ebi-a-GCST90001399	rs7209088	IgD- CD27- %B cel	17	8030165
ebi-a-GCST90001399	rs72897237	IgD- CD27- %B cel	18	37553257
ebi-a-GCST90001399	rs645554	IgD- CD27- %B cel	19	14389948
ebi-a-GCST90001399	rs4810485	IgD- CD27- %B cel	20	44747947
ebi-a-GCST90001399	rs6073079	IgD- CD27- %B cel	20	42024537
ebi-a-GCST90001399	rs11086984	IgD- CD27- %B cel	20	44511627
ebi-a-GCST90001399	rs62223906	IgD- CD27- %B cel	21	38699025
ebi-a-GCST90001400	rs71615254	IgD+ AC	4	178658413
ebi-a-GCST90001400	rs12197153	IgD+ AC	6	100821460
ebi-a-GCST90001400	rs9354413	IgD+ AC	6	67039330
ebi-a-GCST90001400	rs2390504	IgD+ AC	7	21321670
ebi-a-GCST90001400	rs146233710	IgD+ AC	7	106139682
ebi-a-GCST90001400	rs150663332	IgD+ AC	8	2044843
ebi-a-GCST90001400	rs3019260	IgD+ AC	8	102068804
ebi-a-GCST90001400	rs701839	IgD+ AC	10	102955294
ebi-a-GCST90001400	rs189761842	IgD+ AC	10	125485345
ebi-a-GCST90001400	rs116906133	IgD+ AC	13	100023905
ebi-a-GCST90001400	rs4145212	IgD+ AC	13	108960188
ebi-a-GCST90001400	rs3811215	IgD+ AC	14	22954777
ebi-a-GCST90001400	rs11638959	IgD+ AC	15	25171150
ebi-a-GCST90001400	rs143176410	IgD+ AC	16	84305239
ebi-a-GCST90001400	rs11653761	IgD+ AC	17	2716535
ebi-a-GCST90001400	rs62067029	IgD+ AC	17	38038391
ebi-a-GCST90001400	rs67017503	IgD+ AC	17	74280991
ebi-a-GCST90001400	rs12461001	IgD+ AC	19	14885525
ebi-a-GCST90001400	rs77110486	IgD+ AC	20	56359017
ebi-a-GCST90001401	rs61786059	IgD- CD27- AC	1	29035758
ebi-a-GCST90001401	rs7518494	IgD- CD27- AC	1	217233446
ebi-a-GCST90001401	rs185621536	IgD- CD27- AC	1	113524501
ebi-a-GCST90001401	rs12129123	IgD- CD27- AC	1	10083709
ebi-a-GCST90001401	rs72953104	IgD- CD27- AC	1	41379583
ebi-a-GCST90001401	rs113442137	IgD- CD27- AC	1	160254955
ebi-a-GCST90001401	rs11688492	IgD- CD27- AC	2	64865804
ebi-a-GCST90001401	rs7606024	IgD- CD27- AC	2	56616595
ebi-a-GCST90001401	rs114551034	IgD- CD27- AC	2	66709035
ebi-a-GCST90001401	rs35574803	IgD- CD27- AC	3	32533246
ebi-a-GCST90001401	rs13099360	IgD- CD27- AC	3	60476875
ebi-a-GCST90001401	rs59951351	IgD- CD27- AC	4	38152090
ebi-a-GCST90001401	rs74356874	IgD- CD27- AC	5	22936562

ebi-a-GCST90001401	rs144306835	IgD- CD27- AC	8	96356983
ebi-a-GCST90001401	rs112432445	IgD- CD27- AC	9	4162626
ebi-a-GCST90001401	rs78763642	IgD- CD27- AC	9	25294308
ebi-a-GCST90001401	rs117493606	IgD- CD27- AC	10	28340929
ebi-a-GCST90001401	rs117833074	IgD- CD27- AC	12	77551907
ebi-a-GCST90001401	rs1047792	IgD- CD27- AC	12	6649099
ebi-a-GCST90001401	rs2250246	IgD- CD27- AC	12	6553016
ebi-a-GCST90001401	rs11843493	IgD- CD27- AC	13	47527957
ebi-a-GCST90001401	rs421516	IgD- CD27- AC	17	63135589
ebi-a-GCST90001401	rs11661511	IgD- CD27- AC	18	60852258
ebi-a-GCST90001401	rs72977632	IgD- CD27- AC	19	1930174
ebi-a-GCST90001401	rs406298	IgD- CD27- AC	19	16409903
ebi-a-GCST90001401	rs77126700	IgD- CD27- AC	20	47313887
ebi-a-GCST90001401	rs4239702	IgD- CD27- AC	20	44749251
ebi-a-GCST90001401	rs909692	IgD- CD27- AC	22	50710633
ebi-a-GCST90001401	rs9615714	IgD- CD27- AC	22	48376963
ebi-a-GCST90001402	rs72757888	Sw mem %B cell	1	233045963
ebi-a-GCST90001402	rs11204752	Sw mem %B cell	1	150958133
ebi-a-GCST90001402	rs1519529	Sw mem %B cell	2	136974257
ebi-a-GCST90001402	rs138919897	Sw mem %B cell	3	106834795
ebi-a-GCST90001402	rs55658041	Sw mem %B cell	4	164291862
ebi-a-GCST90001402	rs17405603	Sw mem %B cell	5	144608340
ebi-a-GCST90001402	rs830682	Sw mem %B cell	5	165597506
ebi-a-GCST90001402	rs9265880	Sw mem %B cell	6	31313054
ebi-a-GCST90001402	rs2450220	Sw mem %B cell	8	119323401
ebi-a-GCST90001402	rs140191306	Sw mem %B cell	9	2570753
ebi-a-GCST90001402	rs2105800	Sw mem %B cell	11	90592716
ebi-a-GCST90001402	rs11068290	Sw mem %B cell	12	110228055
ebi-a-GCST90001402	rs114917376	Sw mem %B cell	17	51764168
ebi-a-GCST90001402	rs73426855	Sw mem %B cell	17	77637123
ebi-a-GCST90001403	rs142587763	Sw mem AC	1	165680636
ebi-a-GCST90001403	rs115414430	Sw mem AC	2	36720835
ebi-a-GCST90001403	rs1519526	Sw mem AC	2	136967396
ebi-a-GCST90001403	rs7595246	Sw mem AC	2	238554657
ebi-a-GCST90001403	rs73185679	Sw mem AC	3	186485863
ebi-a-GCST90001403	rs141352577	Sw mem AC	3	8492010
ebi-a-GCST90001403	rs115218350	Sw mem AC	3	104146883
ebi-a-GCST90001403	rs80076964	Sw mem AC	4	94935625
ebi-a-GCST90001403	rs4697617	Sw mem AC	4	25771115
ebi-a-GCST90001403	rs452973	Sw mem AC	5	11308990
ebi-a-GCST90001403	rs4076316	Sw mem AC	6	167859623
ebi-a-GCST90001403	rs1161387	Sw mem AC	6	50598963
ebi-a-GCST90001403	rs60508745	Sw mem AC	7	40554169
ebi-a-GCST90001403	rs11777835	Sw mem AC	8	144986103
ebi-a-GCST90001403	rs62521107	Sw mem AC	8	118851954
ebi-a-GCST90001403	rs2454536	Sw mem AC	10	53764740
ebi-a-GCST90001403	rs192544457	Sw mem AC	13	42337460
ebi-a-GCST90001403	rs73178168	Sw mem AC	13	47809629

ebi-a-GCST90001403	rs9520836	Sw mem AC	13	108957063
ebi-a-GCST90001403	rs11653761	Sw mem AC	17	2716535
ebi-a-GCST90001403	rs421516	Sw mem AC	17	63135589
ebi-a-GCST90001403	rs117605782	Sw mem AC	18	46442174
ebi-a-GCST90001403	rs149462070	Sw mem AC	21	14412199
ebi-a-GCST90001403	rs77741094	Sw mem AC	21	42362418
ebi-a-GCST90001403	rs5994482	Sw mem AC	22	32570873
ebi-a-GCST90001404	rs187723151	PB/PC %B cell	1	242186219
ebi-a-GCST90001404	rs2792563	PB/PC %B cell	1	213100225
ebi-a-GCST90001404	rs150152228	PB/PC %B cell	2	235278654
ebi-a-GCST90001404	rs79423067	PB/PC %B cell	4	143873369
ebi-a-GCST90001404	rs78804535	PB/PC %B cell	4	144419847
ebi-a-GCST90001404	rs140642786	PB/PC %B cell	5	148927495
ebi-a-GCST90001404	rs2019885	PB/PC %B cell	5	163177849
ebi-a-GCST90001404	rs193552	PB/PC %B cell	5	148172599
ebi-a-GCST90001404	rs34629	PB/PC %B cell	5	60527506
ebi-a-GCST90001404	rs61294310	PB/PC %B cell	6	5266557
ebi-a-GCST90001404	rs11754551	PB/PC %B cell	6	145631063
ebi-a-GCST90001404	rs78370279	PB/PC %B cell	7	71338898
ebi-a-GCST90001404	rs74326785	PB/PC %B cell	8	24549984
ebi-a-GCST90001404	rs138542577	PB/PC %B cell	8	16074805
ebi-a-GCST90001404	rs4458909	PB/PC %B cell	8	118447297
ebi-a-GCST90001404	rs3138500	PB/PC %B cell	9	92219945
ebi-a-GCST90001404	rs12412378	PB/PC %B cell	10	130483163
ebi-a-GCST90001404	rs185836310	PB/PC %B cell	10	73504848
ebi-a-GCST90001404	rs76195952	PB/PC %B cell	11	129797755
ebi-a-GCST90001404	rs55728331	PB/PC %B cell	11	117526440
ebi-a-GCST90001404	rs10849436	PB/PC %B cell	12	6409619
ebi-a-GCST90001404	rs62012610	PB/PC %B cell	15	34006114
ebi-a-GCST90001404	rs8191161	PB/PC %B cell	16	82103595
ebi-a-GCST90001404	rs12709039	PB/PC %B cell	16	59861376
ebi-a-GCST90001404	rs80148662	PB/PC %B cell	17	52836239
ebi-a-GCST90001404	rs186329	PB/PC %B cell	19	56065534
ebi-a-GCST90001404	rs8132059	PB/PC %B cell	21	16916284
ebi-a-GCST90001404	rs11089305	PB/PC %B cell	22	19729361
ebi-a-GCST90001405	rs80297308	PB/PC AC	1	102294391
ebi-a-GCST90001405	rs10800645	PB/PC AC	1	199586940
ebi-a-GCST90001405	rs74458303	PB/PC AC	2	121699864
ebi-a-GCST90001405	rs148362575	PB/PC AC	2	187165335
ebi-a-GCST90001405	rs148389014	PB/PC AC	3	188082431
ebi-a-GCST90001405	rs116540222	PB/PC AC	3	117331256
ebi-a-GCST90001405	rs149048155	PB/PC AC	3	957228
ebi-a-GCST90001405	rs62353585	PB/PC AC	4	168102710
ebi-a-GCST90001405	rs76709581	PB/PC AC	5	179574693
ebi-a-GCST90001405	rs7449190	PB/PC AC	5	1297918
ebi-a-GCST90001405	rs61294310	PB/PC AC	6	5266557
ebi-a-GCST90001405	rs4506076	PB/PC AC	6	8860293
ebi-a-GCST90001405	rs149559870	PB/PC AC	8	39638049

ebi-a-GCST90001405	rs74565954	PB/PC AC	8	24520468
ebi-a-GCST90001405	rs62519719	PB/PC AC	8	118376967
ebi-a-GCST90001405	rs12412378	PB/PC AC	10	130483163
ebi-a-GCST90001405	rs1681588	PB/PC AC	14	23098304
ebi-a-GCST90001405	rs17765677	PB/PC AC	16	65999760
ebi-a-GCST90001405	rs7185719	PB/PC AC	16	59859266
ebi-a-GCST90001405	rs112471525	PB/PC AC	17	52797903
ebi-a-GCST90001405	rs72883992	PB/PC AC	18	24475181
ebi-a-GCST90001405	rs4806937	PB/PC AC	19	3443405
ebi-a-GCST90001405	rs6014246	PB/PC AC	20	53613034
ebi-a-GCST90001405	rs4820558	PB/PC AC	22	21022887
ebi-a-GCST90001405	rs28652751	PB/PC AC	22	35861208
ebi-a-GCST90001406	rs75766272	Memory B cell %B	1	8009985
ebi-a-GCST90001406	rs4658141	Memory B cell %B	1	89814066
ebi-a-GCST90001406	rs149876490	Memory B cell %B	1	16668240
ebi-a-GCST90001406	rs7412746	Memory B cell %B	1	150860471
ebi-a-GCST90001406	rs72635970	Memory B cell %B	2	73868977
ebi-a-GCST90001406	rs116391651	Memory B cell %B	2	183843344
ebi-a-GCST90001406	rs72844588	Memory B cell %B	2	138420154
ebi-a-GCST90001406	rs55710170	Memory B cell %B	3	16416145
ebi-a-GCST90001406	rs60515154	Memory B cell %B	4	47914545
ebi-a-GCST90001406	rs35698683	Memory B cell %B	4	11590243
ebi-a-GCST90001406	rs77312528	Memory B cell %B	4	36385921
ebi-a-GCST90001406	rs895377	Memory B cell %B	5	58050530
ebi-a-GCST90001406	rs690488	Memory B cell %B	5	40183220
ebi-a-GCST90001406	rs115405874	Memory B cell %B	5	119742816
ebi-a-GCST90001406	rs62398379	Memory B cell %B	6	37585447
ebi-a-GCST90001406	rs9270558	Memory B cell %B	6	32560667
ebi-a-GCST90001406	rs9492229	Memory B cell %B	6	129409652
ebi-a-GCST90001406	rs633090	Memory B cell %B	8	12933368
ebi-a-GCST90001406	rs140191306	Memory B cell %B	9	2570753
ebi-a-GCST90001406	rs145123647	Memory B cell %B	10	26959594
ebi-a-GCST90001406	rs10750504	Memory B cell %B	11	132156175
ebi-a-GCST90001406	rs61896866	Memory B cell %B	11	111318165
ebi-a-GCST90001406	rs112570989	Memory B cell %B	12	2866164
ebi-a-GCST90001406	rs9584780	Memory B cell %B	13	98901269
ebi-a-GCST90001406	rs76477280	Memory B cell %B	13	50147180
ebi-a-GCST90001406	rs116844769	Memory B cell %B	14	65713895
ebi-a-GCST90001406	rs77762131	Memory B cell %B	14	96205838
ebi-a-GCST90001406	rs72914692	Memory B cell %B	18	54422995
ebi-a-GCST90001406	rs329966	Memory B cell %B	19	53070617
ebi-a-GCST90001407	rs16867231	Memory B cell AC	2	181050023
ebi-a-GCST90001407	rs10018308	Memory B cell AC	4	120657899
ebi-a-GCST90001407	rs17708396	Memory B cell AC	5	148410250
ebi-a-GCST90001407	rs12216018	Memory B cell AC	6	130514590
ebi-a-GCST90001407	rs74565175	Memory B cell AC	7	81370578
ebi-a-GCST90001407	rs77643931	Memory B cell AC	9	129171041
ebi-a-GCST90001407	rs12763172	Memory B cell AC	10	85447519

ebi-a-GCST90001407	rs2879633	Memory B cell AC	10	53757050
ebi-a-GCST90001407	rs36072728	Memory B cell AC	11	29448646
ebi-a-GCST90001407	rs557577736	Memory B cell AC	13	46224092
ebi-a-GCST90001407	rs12874404	Memory B cell AC	13	108993494
ebi-a-GCST90001407	rs61997631	Memory B cell AC	14	104600920
ebi-a-GCST90001407	rs77762131	Memory B cell AC	14	96205838
ebi-a-GCST90001407	rs139196765	Memory B cell AC	15	24733764
ebi-a-GCST90001407	rs11653761	Memory B cell AC	17	2716535
ebi-a-GCST90001407	rs144295598	Memory B cell AC	22	45164171
ebi-a-GCST90001407	rs9614226	Memory B cell AC	22	44688150
ebi-a-GCST90001408	rs4658141	Naive-mature B cel	1	89814066
ebi-a-GCST90001408	rs7412746	Naive-mature B cel	1	150860471
ebi-a-GCST90001408	rs75766272	Naive-mature B cel	1	8009985
ebi-a-GCST90001408	rs149876490	Naive-mature B cel	1	16668240
ebi-a-GCST90001408	rs72844588	Naive-mature B cel	2	138420154
ebi-a-GCST90001408	rs13421263	Naive-mature B cel	2	169182294
ebi-a-GCST90001408	rs72635970	Naive-mature B cel	2	73868977
ebi-a-GCST90001408	rs55710170	Naive-mature B cel	3	16416145
ebi-a-GCST90001408	rs35698683	Naive-mature B cel	4	11590243
ebi-a-GCST90001408	rs77312528	Naive-mature B cel	4	36385921
ebi-a-GCST90001408	rs115405874	Naive-mature B cel	5	119742816
ebi-a-GCST90001408	rs690488	Naive-mature B cel	5	40183220
ebi-a-GCST90001408	rs895377	Naive-mature B cel	5	58050530
ebi-a-GCST90001408	rs62398379	Naive-mature B cel	6	37585447
ebi-a-GCST90001408	rs9492229	Naive-mature B cel	6	129409652
ebi-a-GCST90001408	rs9270578	Naive-mature B cel	6	32561093
ebi-a-GCST90001408	rs633090	Naive-mature B cel	8	12933368
ebi-a-GCST90001408	rs145123647	Naive-mature B cel	10	26959594
ebi-a-GCST90001408	rs61896866	Naive-mature B cel	11	111318165
ebi-a-GCST90001408	rs2334904	Naive-mature B cel	11	132133144
ebi-a-GCST90001408	rs112570989	Naive-mature B cel	12	2866164
ebi-a-GCST90001408	rs76477280	Naive-mature B cel	13	50147180
ebi-a-GCST90001408	rs9584780	Naive-mature B cel	13	98901269
ebi-a-GCST90001408	rs2476213	Naive-mature B cel	13	102612226
ebi-a-GCST90001408	rs77762131	Naive-mature B cel	14	96205838
ebi-a-GCST90001408	rs974368	Naive-mature B cel	14	63009549
ebi-a-GCST90001408	rs116844769	Naive-mature B cel	14	65713895
ebi-a-GCST90001408	rs72914692	Naive-mature B cel	18	54422995
ebi-a-GCST90001408	rs9614418	Naive-mature B cel	22	44674143
ebi-a-GCST90001409	rs139077655	Naive-mature B cel	2	132741527
ebi-a-GCST90001409	rs76981518	Naive-mature B cel	4	152777207
ebi-a-GCST90001409	rs115150472	Naive-mature B cel	5	146138788
ebi-a-GCST90001409	rs10042327	Naive-mature B cel	5	58278611
ebi-a-GCST90001409	rs17056318	Naive-mature B cel	5	158285953
ebi-a-GCST90001409	rs9354413	Naive-mature B cel	6	67039330
ebi-a-GCST90001409	rs35607968	Naive-mature B cel	7	67653879
ebi-a-GCST90001409	rs2390504	Naive-mature B cel	7	21321670
ebi-a-GCST90001409	rs143397127	Naive-mature B cel	7	12847391

ebi-a-GCST90001409	rs2395904	Naive-mature B cel	8	130617819
ebi-a-GCST90001409	rs283340	Naive-mature B cel	8	70193536
ebi-a-GCST90001409	rs189761842	Naive-mature B cel	10	125485345
ebi-a-GCST90001409	rs117344365	Naive-mature B cel	10	33779474
ebi-a-GCST90001409	rs12799241	Naive-mature B cel	11	119980729
ebi-a-GCST90001409	rs2286599	Naive-mature B cel	12	6499533
ebi-a-GCST90001409	rs58114937	Naive-mature B cel	12	67638132
ebi-a-GCST90001409	rs9564767	Naive-mature B cel	13	71534535
ebi-a-GCST90001409	rs3811215	Naive-mature B cel	14	22954777
ebi-a-GCST90001409	rs76263497	Naive-mature B cel	15	60878804
ebi-a-GCST90001409	rs143176410	Naive-mature B cel	16	84305239
ebi-a-GCST90001409	rs77983244	Naive-mature B cel	17	7316027
ebi-a-GCST90001409	rs62067029	Naive-mature B cel	17	38038391
ebi-a-GCST90001409	rs62115322	Naive-mature B cel	19	53848484
ebi-a-GCST90001409	rs34764986	Naive-mature B cel	20	59926554
ebi-a-GCST90001410	rs2792563	IgD- CD38br %B c	1	213100225
ebi-a-GCST90001410	rs1250061	IgD- CD38br %B c	2	216542855
ebi-a-GCST90001410	rs12636371	IgD- CD38br %B c	3	125894785
ebi-a-GCST90001410	rs12493961	IgD- CD38br %B c	3	11244825
ebi-a-GCST90001410	rs13075403	IgD- CD38br %B c	3	28830276
ebi-a-GCST90001410	rs11938966	IgD- CD38br %B c	4	55386841
ebi-a-GCST90001410	rs76912165	IgD- CD38br %B c	5	92965344
ebi-a-GCST90001410	rs139843122	IgD- CD38br %B c	6	107939307
ebi-a-GCST90001410	rs148377259	IgD- CD38br %B c	7	106306631
ebi-a-GCST90001410	rs13235778	IgD- CD38br %B c	7	57550983
ebi-a-GCST90001410	rs10270812	IgD- CD38br %B c	7	68026886
ebi-a-GCST90001410	rs188550599	IgD- CD38br %B c	8	15730211
ebi-a-GCST90001410	rs138542577	IgD- CD38br %B c	8	16074805
ebi-a-GCST90001410	rs137930158	IgD- CD38br %B c	8	109663198
ebi-a-GCST90001410	rs113087021	IgD- CD38br %B c	11	97765784
ebi-a-GCST90001410	rs478251	IgD- CD38br %B c	11	127827727
ebi-a-GCST90001410	rs238926	IgD- CD38br %B c	11	113975210
ebi-a-GCST90001410	rs7185719	IgD- CD38br %B c	16	59859266
ebi-a-GCST90001410	rs11705673	IgD- CD38br %B c	22	45016637
ebi-a-GCST90001411	rs75766272	IgD+ CD24+ %B c	1	8009985
ebi-a-GCST90001411	rs11584183	IgD+ CD24+ %B c	1	230130712
ebi-a-GCST90001411	rs45608038	IgD+ CD24+ %B c	2	73679013
ebi-a-GCST90001411	rs6781675	IgD+ CD24+ %B c	3	4408435
ebi-a-GCST90001411	rs11134931	IgD+ CD24+ %B c	5	175234476
ebi-a-GCST90001411	rs34418535	IgD+ CD24+ %B c	6	32553200
ebi-a-GCST90001411	rs45575344	IgD+ CD24+ %B c	6	133003242
ebi-a-GCST90001411	rs147867176	IgD+ CD24+ %B c	6	169106376
ebi-a-GCST90001411	rs9369326	IgD+ CD24+ %B c	6	42013868
ebi-a-GCST90001411	rs7457479	IgD+ CD24+ %B c	7	66493985
ebi-a-GCST90001411	rs62562996	IgD+ CD24+ %B c	9	21576571
ebi-a-GCST90001411	rs112570989	IgD+ CD24+ %B c	12	2866164
ebi-a-GCST90001411	rs12874404	IgD+ CD24+ %B c	13	108993494
ebi-a-GCST90001411	rs7988851	IgD+ CD24+ %B c	13	51367583

ebi-a-GCST90001411	rs76477280	IgD+ CD24+ %B c	13	50147180
ebi-a-GCST90001411	rs145371905	IgD+ CD24+ %B c	13	70325772
ebi-a-GCST90001411	rs77762131	IgD+ CD24+ %B c	14	96205838
ebi-a-GCST90001411	rs78269238	IgD+ CD24+ %B c	14	65673804
ebi-a-GCST90001411	rs35130455	IgD+ CD24+ %B c	14	93256694
ebi-a-GCST90001411	rs17256322	IgD+ CD24+ %B c	16	4127619
ebi-a-GCST90001411	rs144479315	IgD+ CD24+ %B c	18	23575118
ebi-a-GCST90001412	rs7586359	IgD+ CD24+ AC	2	237195419
ebi-a-GCST90001412	rs10513789	IgD+ CD24+ AC	3	182760073
ebi-a-GCST90001412	rs13132141	IgD+ CD24+ AC	4	156739300
ebi-a-GCST90001412	rs147556975	IgD+ CD24+ AC	5	148456360
ebi-a-GCST90001412	rs4702913	IgD+ CD24+ AC	5	4700587
ebi-a-GCST90001412	rs10942726	IgD+ CD24+ AC	5	74257985
ebi-a-GCST90001412	rs144607178	IgD+ CD24+ AC	7	63248560
ebi-a-GCST90001412	rs10749417	IgD+ CD24+ AC	10	85446387
ebi-a-GCST90001412	rs11047113	IgD+ CD24+ AC	12	23968609
ebi-a-GCST90001412	rs183466766	IgD+ CD24+ AC	13	108710731
ebi-a-GCST90001412	rs139981802	IgD+ CD24+ AC	13	108488351
ebi-a-GCST90001412	rs557577736	IgD+ CD24+ AC	13	46224092
ebi-a-GCST90001412	rs145371905	IgD+ CD24+ AC	13	70325772
ebi-a-GCST90001412	rs9314892	IgD+ CD24+ AC	13	114873368
ebi-a-GCST90001412	rs12874404	IgD+ CD24+ AC	13	108993494
ebi-a-GCST90001412	rs73192651	IgD+ CD24+ AC	13	50212885
ebi-a-GCST90001412	rs8014978	IgD+ CD24+ AC	14	94936964
ebi-a-GCST90001412	rs77762131	IgD+ CD24+ AC	14	96205838
ebi-a-GCST90001412	rs12595433	IgD+ CD24+ AC	15	63290645
ebi-a-GCST90001412	rs880312	IgD+ CD24+ AC	16	8963501
ebi-a-GCST90001412	rs11653761	IgD+ CD24+ AC	17	2716535
ebi-a-GCST90001412	rs7254039	IgD+ CD24+ AC	19	14993354
ebi-a-GCST90001412	rs9614226	IgD+ CD24+ AC	22	44688150
ebi-a-GCST90001413	rs111237501	IgD- CD24- %B cel	1	163272701
ebi-a-GCST90001413	rs11887461	IgD- CD24- %B cel	2	128660247
ebi-a-GCST90001413	rs13020544	IgD- CD24- %B cel	2	217809198
ebi-a-GCST90001413	rs145137909	IgD- CD24- %B cel	2	215618238
ebi-a-GCST90001413	rs114209430	IgD- CD24- %B cel	4	83018399
ebi-a-GCST90001413	rs17044633	IgD- CD24- %B cel	4	113331648
ebi-a-GCST90001413	rs183821560	IgD- CD24- %B cel	5	62072324
ebi-a-GCST90001413	rs9398107	IgD- CD24- %B cel	6	107155687
ebi-a-GCST90001413	rs73045003	IgD- CD24- %B cel	7	2651996
ebi-a-GCST90001413	rs76377003	IgD- CD24- %B cel	8	112881828
ebi-a-GCST90001413	rs112898282	IgD- CD24- %B cel	10	28462184
ebi-a-GCST90001413	rs7966251	IgD- CD24- %B cel	12	103731395
ebi-a-GCST90001413	rs142305616	IgD- CD24- %B cel	14	103946237
ebi-a-GCST90001413	rs7198605	IgD- CD24- %B cel	16	6107421
ebi-a-GCST90001413	rs35192093	IgD- CD24- %B cel	18	29387367
ebi-a-GCST90001413	rs8109503	IgD- CD24- %B cel	19	19073798
ebi-a-GCST90001413	rs6073000	IgD- CD24- %B cel	20	41777449
ebi-a-GCST90001413	rs115218594	IgD- CD24- %B cel	22	27642688

ebi-a-GCST90001414	rs72953104	IgD- CD24- AC	1	41379583
ebi-a-GCST90001414	rs111237501	IgD- CD24- AC	1	163272701
ebi-a-GCST90001414	rs114114265	IgD- CD24- AC	2	79806712
ebi-a-GCST90001414	rs7615875	IgD- CD24- AC	3	29345411
ebi-a-GCST90001414	rs76499400	IgD- CD24- AC	3	191888629
ebi-a-GCST90001414	rs4697616	IgD- CD24- AC	4	25767183
ebi-a-GCST90001414	rs17044633	IgD- CD24- AC	4	113331648
ebi-a-GCST90001414	rs76225955	IgD- CD24- AC	4	31594004
ebi-a-GCST90001414	rs74356874	IgD- CD24- AC	5	22936562
ebi-a-GCST90001414	rs145638783	IgD- CD24- AC	6	1238442
ebi-a-GCST90001414	rs112898282	IgD- CD24- AC	10	28462184
ebi-a-GCST90001414	rs75453701	IgD- CD24- AC	13	55229646
ebi-a-GCST90001414	rs7982713	IgD- CD24- AC	13	71376322
ebi-a-GCST90001414	rs191543445	IgD- CD24- AC	14	31505138
ebi-a-GCST90001414	rs496760	IgD- CD24- AC	15	54695686
ebi-a-GCST90001414	rs421516	IgD- CD24- AC	17	63135589
ebi-a-GCST90001414	rs72977632	IgD- CD24- AC	19	1930174
ebi-a-GCST90001414	rs451367	IgD- CD24- AC	19	16412842
ebi-a-GCST90001415	rs11184230	IgD+ CD24- %B ce	1	105159724
ebi-a-GCST90001415	rs7412746	IgD+ CD24- %B ce	1	150860471
ebi-a-GCST90001415	rs75766272	IgD+ CD24- %B ce	1	8009985
ebi-a-GCST90001415	rs45608038	IgD+ CD24- %B ce	2	73679013
ebi-a-GCST90001415	rs12465275	IgD+ CD24- %B ce	2	4082267
ebi-a-GCST90001415	rs56335940	IgD+ CD24- %B ce	3	106579815
ebi-a-GCST90001415	rs1972883	IgD+ CD24- %B ce	4	47951945
ebi-a-GCST90001415	rs35698683	IgD+ CD24- %B ce	4	11590243
ebi-a-GCST90001415	rs6910508	IgD+ CD24- %B ce	6	166531345
ebi-a-GCST90001415	rs533239981	IgD+ CD24- %B ce	8	75515052
ebi-a-GCST90001415	rs2450220	IgD+ CD24- %B ce	8	119323401
ebi-a-GCST90001415	rs7870388	IgD+ CD24- %B ce	9	8660693
ebi-a-GCST90001415	rs140191306	IgD+ CD24- %B ce	9	2570753
ebi-a-GCST90001415	rs145123647	IgD+ CD24- %B ce	10	26959594
ebi-a-GCST90001415	rs116844769	IgD+ CD24- %B ce	14	65713895
ebi-a-GCST90001415	rs182854545	IgD+ CD24- %B ce	14	54016223
ebi-a-GCST90001415	rs73426855	IgD+ CD24- %B ce	17	77637123
ebi-a-GCST90001415	rs2159343	IgD+ CD24- %B ce	17	49451837
ebi-a-GCST90001415	rs78958229	IgD+ CD24- %B ce	21	39363957
ebi-a-GCST90001415	rs58706313	IgD+ CD24- %B ce	22	44492562
ebi-a-GCST90001416	rs71615254	IgD+ CD24- AC	4	178658413
ebi-a-GCST90001416	rs17056318	IgD+ CD24- AC	5	158285953
ebi-a-GCST90001416	rs141108367	IgD+ CD24- AC	5	21916806
ebi-a-GCST90001416	rs115150472	IgD+ CD24- AC	5	146138788
ebi-a-GCST90001416	rs12197153	IgD+ CD24- AC	6	100821460
ebi-a-GCST90001416	rs9354413	IgD+ CD24- AC	6	67039330
ebi-a-GCST90001416	rs143397127	IgD+ CD24- AC	7	12847391
ebi-a-GCST90001416	rs190448231	IgD+ CD24- AC	7	87868543
ebi-a-GCST90001416	rs34910780	IgD+ CD24- AC	7	77469688
ebi-a-GCST90001416	rs2395904	IgD+ CD24- AC	8	130617819

ebi-a-GCST90001416	rs150663332	IgD+ CD24- AC	8	2044843
ebi-a-GCST90001416	rs117190494	IgD+ CD24- AC	8	75563339
ebi-a-GCST90001416	rs701839	IgD+ CD24- AC	10	102955294
ebi-a-GCST90001416	rs189761842	IgD+ CD24- AC	10	125485345
ebi-a-GCST90001416	rs12799241	IgD+ CD24- AC	11	119980729
ebi-a-GCST90001416	rs34771848	IgD+ CD24- AC	12	14302579
ebi-a-GCST90001416	rs76578043	IgD+ CD24- AC	13	105214810
ebi-a-GCST90001416	rs75474045	IgD+ CD24- AC	13	72428296
ebi-a-GCST90001416	rs3811215	IgD+ CD24- AC	14	22954777
ebi-a-GCST90001416	rs143176410	IgD+ CD24- AC	16	84305239
ebi-a-GCST90001416	rs73426855	IgD+ CD24- AC	17	77637123
ebi-a-GCST90001416	rs77983244	IgD+ CD24- AC	17	7316027
ebi-a-GCST90001416	rs62067029	IgD+ CD24- AC	17	38038391
ebi-a-GCST90001416	rs34764986	IgD+ CD24- AC	20	59926554
ebi-a-GCST90001417	rs145208372	CD24+ CD27+ %B	1	83678678
ebi-a-GCST90001417	rs7412746	CD24+ CD27+ %B	1	150860471
ebi-a-GCST90001417	rs72758947	CD24+ CD27+ %B	1	207087053
ebi-a-GCST90001417	rs4658141	CD24+ CD27+ %B	1	89814066
ebi-a-GCST90001417	rs75766272	CD24+ CD27+ %B	1	8009985
ebi-a-GCST90001417	rs72944931	CD24+ CD27+ %B	2	178116262
ebi-a-GCST90001417	rs45608038	CD24+ CD27+ %B	2	73679013
ebi-a-GCST90001417	rs72844588	CD24+ CD27+ %B	2	138420154
ebi-a-GCST90001417	rs62233948	CD24+ CD27+ %B	3	7831627
ebi-a-GCST90001417	rs145442262	CD24+ CD27+ %B	3	186702869
ebi-a-GCST90001417	rs55710170	CD24+ CD27+ %B	3	16416145
ebi-a-GCST90001417	rs35698683	CD24+ CD27+ %B	4	11590243
ebi-a-GCST90001417	rs115405874	CD24+ CD27+ %B	5	119742816
ebi-a-GCST90001417	rs681014	CD24+ CD27+ %B	5	140636323
ebi-a-GCST90001417	rs9492229	CD24+ CD27+ %B	6	129409652
ebi-a-GCST90001417	rs148702078	CD24+ CD27+ %B	6	100574892
ebi-a-GCST90001417	rs62459310	CD24+ CD27+ %B	7	56242182
ebi-a-GCST90001417	rs10505439	CD24+ CD27+ %B	8	124662104
ebi-a-GCST90001417	rs10743163	CD24+ CD27+ %B	11	11033950
ebi-a-GCST90001417	rs265612	CD24+ CD27+ %B	12	130112732
ebi-a-GCST90001417	rs112294465	CD24+ CD27+ %B	12	123744082
ebi-a-GCST90001417	rs76477280	CD24+ CD27+ %B	13	50147180
ebi-a-GCST90001417	rs117004076	CD24+ CD27+ %B	14	97702226
ebi-a-GCST90001417	rs77762131	CD24+ CD27+ %B	14	96205838
ebi-a-GCST90001417	rs116844769	CD24+ CD27+ %B	14	65713895
ebi-a-GCST90001417	rs7221794	CD24+ CD27+ %B	17	76979729
ebi-a-GCST90001418	rs75766272	CD24+ CD27+ AC	1	8009985
ebi-a-GCST90001418	rs16867231	CD24+ CD27+ AC	2	181050023
ebi-a-GCST90001418	rs79207264	CD24+ CD27+ AC	3	96406408
ebi-a-GCST90001418	rs7640784	CD24+ CD27+ AC	3	176029498
ebi-a-GCST90001418	rs149085540	CD24+ CD27+ AC	4	110550623
ebi-a-GCST90001418	rs17708396	CD24+ CD27+ AC	5	148410250
ebi-a-GCST90001418	rs10942726	CD24+ CD27+ AC	5	74257985
ebi-a-GCST90001418	rs35057381	CD24+ CD27+ AC	5	26412301

ebi-a-GCST90001418	rs56033406	CD24+ CD27+ AC	6	41084740
ebi-a-GCST90001418	rs4076316	CD24+ CD27+ AC	6	167859623
ebi-a-GCST90001418	rs144625305	CD24+ CD27+ AC	7	7871676
ebi-a-GCST90001418	rs184496770	CD24+ CD27+ AC	8	124255582
ebi-a-GCST90001418	rs118092259	CD24+ CD27+ AC	8	89432719
ebi-a-GCST90001418	rs10759006	CD24+ CD27+ AC	9	8855603
ebi-a-GCST90001418	rs10762541	CD24+ CD27+ AC	10	53790461
ebi-a-GCST90001418	rs138329462	CD24+ CD27+ AC	12	11634360
ebi-a-GCST90001418	rs557577736	CD24+ CD27+ AC	13	46224092
ebi-a-GCST90001418	rs12874404	CD24+ CD27+ AC	13	108993494
ebi-a-GCST90001418	rs73192651	CD24+ CD27+ AC	13	50212885
ebi-a-GCST90001418	rs76552872	CD24+ CD27+ AC	13	70167316
ebi-a-GCST90001418	rs77762131	CD24+ CD27+ AC	14	96205838
ebi-a-GCST90001418	rs11653761	CD24+ CD27+ AC	17	2716535
ebi-a-GCST90001418	rs11649808	CD24+ CD27+ AC	17	62258665
ebi-a-GCST90001418	rs7254039	CD24+ CD27+ AC	19	14993354
ebi-a-GCST90001418	rs728776	CD24+ CD27+ AC	22	45400116
ebi-a-GCST90001418	rs144295598	CD24+ CD27+ AC	22	45164171
ebi-a-GCST90001419	rs529500582	CD20- %B cell	1	11620068
ebi-a-GCST90001419	rs187723151	CD20- %B cell	1	242186219
ebi-a-GCST90001419	rs112180455	CD20- %B cell	2	221466858
ebi-a-GCST90001419	rs58352426	CD20- %B cell	3	154090291
ebi-a-GCST90001419	rs75951284	CD20- %B cell	3	187638796
ebi-a-GCST90001419	rs74786971	CD20- %B cell	4	90909176
ebi-a-GCST90001419	rs316122	CD20- %B cell	5	93014071
ebi-a-GCST90001419	rs9270911	CD20- %B cell	6	32572202
ebi-a-GCST90001419	rs9477367	CD20- %B cell	6	17249496
ebi-a-GCST90001419	rs111761453	CD20- %B cell	7	137173666
ebi-a-GCST90001419	rs75368642	CD20- %B cell	7	150969808
ebi-a-GCST90001419	rs7875273	CD20- %B cell	9	9023522
ebi-a-GCST90001419	rs118084343	CD20- %B cell	11	17687837
ebi-a-GCST90001419	rs3885787	CD20- %B cell	12	92375948
ebi-a-GCST90001419	rs4244568	CD20- %B cell	15	39319915
ebi-a-GCST90001419	rs140134875	CD20- %B cell	15	80306120
ebi-a-GCST90001419	rs4889373	CD20- %B cell	16	81747602
ebi-a-GCST90001419	rs2107459	CD20- %B cell	16	74140113
ebi-a-GCST90001419	rs7265879	CD20- %B cell	20	6103341
ebi-a-GCST90001419	rs57840701	CD20- %B cell	20	46686437
ebi-a-GCST90001420	rs115156019	IgD- CD38br AC	1	168227395
ebi-a-GCST90001420	rs2792563	IgD- CD38br AC	1	213100225
ebi-a-GCST90001420	rs138158989	IgD- CD38br AC	2	204476635
ebi-a-GCST90001420	rs114044800	IgD- CD38br AC	2	237823477
ebi-a-GCST90001420	rs148362575	IgD- CD38br AC	2	187165335
ebi-a-GCST90001420	rs66491584	IgD- CD38br AC	3	20980398
ebi-a-GCST90001420	rs141490841	IgD- CD38br AC	3	149265655
ebi-a-GCST90001420	rs62353585	IgD- CD38br AC	4	168102710
ebi-a-GCST90001420	rs3105149	IgD- CD38br AC	5	36474634
ebi-a-GCST90001420	rs78678130	IgD- CD38br AC	7	105792831

ebi-a-GCST90001420	rs138058221	IgD- CD38br AC	7	132571443
ebi-a-GCST90001420	rs10815908	IgD- CD38br AC	9	8537763
ebi-a-GCST90001420	rs912755	IgD- CD38br AC	10	7484185
ebi-a-GCST90001420	rs11605946	IgD- CD38br AC	11	19547027
ebi-a-GCST90001420	rs79801395	IgD- CD38br AC	11	97756814
ebi-a-GCST90001420	rs478251	IgD- CD38br AC	11	127827727
ebi-a-GCST90001420	rs147319637	IgD- CD38br AC	11	111211623
ebi-a-GCST90001420	rs11609248	IgD- CD38br AC	12	14317207
ebi-a-GCST90001420	rs17037450	IgD- CD38br AC	12	106098314
ebi-a-GCST90001420	rs2075365	IgD- CD38br AC	12	31092966
ebi-a-GCST90001420	rs74011406	IgD- CD38br AC	15	47631034
ebi-a-GCST90001420	rs72834736	IgD- CD38br AC	16	81821361
ebi-a-GCST90001421	rs6712095	CD20- AC	2	205623497
ebi-a-GCST90001421	rs57641238	CD20- AC	2	75536553
ebi-a-GCST90001421	rs34625183	CD20- AC	3	146097919
ebi-a-GCST90001421	rs36123742	CD20- AC	3	133253719
ebi-a-GCST90001421	rs74786971	CD20- AC	4	90909176
ebi-a-GCST90001421	rs116699251	CD20- AC	5	142419461
ebi-a-GCST90001421	rs41494	CD20- AC	5	9688280
ebi-a-GCST90001421	rs7773849	CD20- AC	6	128553518
ebi-a-GCST90001421	rs9273241	CD20- AC	6	32614025
ebi-a-GCST90001421	rs2102662	CD20- AC	7	56828069
ebi-a-GCST90001421	rs111761453	CD20- AC	7	137173666
ebi-a-GCST90001421	rs38182	CD20- AC	7	15900650
ebi-a-GCST90001421	rs6586822	CD20- AC	8	19139514
ebi-a-GCST90001421	rs6482467	CD20- AC	10	25320223
ebi-a-GCST90001421	rs16936840	CD20- AC	11	19531128
ebi-a-GCST90001421	rs2212443	CD20- AC	11	88131197
ebi-a-GCST90001421	rs12146943	CD20- AC	13	21076875
ebi-a-GCST90001421	rs77553819	CD20- AC	13	24882401
ebi-a-GCST90001421	rs71431666	CD20- AC	14	95266847
ebi-a-GCST90001421	rs252298	CD20- AC	16	29232967
ebi-a-GCST90001421	rs56736306	CD20- AC	16	87840577
ebi-a-GCST90001421	rs2637364	CD20- AC	17	32078878
ebi-a-GCST90001421	rs6029245	CD20- AC	20	39267990
ebi-a-GCST90001421	rs6052993	CD20- AC	20	4944838
ebi-a-GCST90001421	rs6008703	CD20- AC	22	48837650
ebi-a-GCST90001422	rs75839951	CD20- CD38- %B (	2	34491589
ebi-a-GCST90001422	rs4832496	CD20- CD38- %B (	2	17752538
ebi-a-GCST90001422	rs141595421	CD20- CD38- %B (	3	63492195
ebi-a-GCST90001422	rs396692	CD20- CD38- %B (	4	163553861
ebi-a-GCST90001422	rs182720746	CD20- CD38- %B (	4	113092883
ebi-a-GCST90001422	rs2215848	CD20- CD38- %B (	4	25065221
ebi-a-GCST90001422	rs62311900	CD20- CD38- %B (	4	74145187
ebi-a-GCST90001422	rs2339996	CD20- CD38- %B (	5	173070505
ebi-a-GCST90001422	rs13176237	CD20- CD38- %B (	5	42353044
ebi-a-GCST90001422	rs72492305	CD20- CD38- %B (	6	32509333
ebi-a-GCST90001422	rs56284619	CD20- CD38- %B (	6	90013983

ebi-a-GCST90001422	rs10988372	CD20- CD38- %B c	9	132193568
ebi-a-GCST90001422	rs118073973	CD20- CD38- %B c	9	19948908
ebi-a-GCST90001422	rs72652459	CD20- CD38- %B c	9	22099416
ebi-a-GCST90001422	rs55767134	CD20- CD38- %B c	10	17048435
ebi-a-GCST90001422	rs34666228	CD20- CD38- %B c	11	123385425
ebi-a-GCST90001422	rs78301150	CD20- CD38- %B c	12	82233392
ebi-a-GCST90001422	rs55897487	CD20- CD38- %B c	12	261706
ebi-a-GCST90001422	rs117308259	CD20- CD38- %B c	14	31993501
ebi-a-GCST90001422	rs17099538	CD20- CD38- %B c	14	33254318
ebi-a-GCST90001422	rs77911577	CD20- CD38- %B c	14	28669022
ebi-a-GCST90001422	rs2113943	CD20- CD38- %B c	15	60895636
ebi-a-GCST90001422	rs35639639	CD20- CD38- %B c	16	5488091
ebi-a-GCST90001422	rs16955339	CD20- CD38- %B c	17	52971060
ebi-a-GCST90001422	rs36071180	CD20- CD38- %B c	20	765164
ebi-a-GCST90001422	rs6125184	CD20- CD38- %B c	20	46576714
ebi-a-GCST90001423	rs182720746	CD20- CD38- AC	4	113092883
ebi-a-GCST90001423	rs62311900	CD20- CD38- AC	4	74145187
ebi-a-GCST90001423	rs2339996	CD20- CD38- AC	5	173070505
ebi-a-GCST90001423	rs72492305	CD20- CD38- AC	6	32509333
ebi-a-GCST90001423	rs6906886	CD20- CD38- AC	6	85038851
ebi-a-GCST90001423	rs11203884	CD20- CD38- AC	8	17516564
ebi-a-GCST90001423	rs10988372	CD20- CD38- AC	9	132193568
ebi-a-GCST90001423	rs118073973	CD20- CD38- AC	9	19948908
ebi-a-GCST90001423	rs75609575	CD20- CD38- AC	11	97639659
ebi-a-GCST90001423	rs308820	CD20- CD38- AC	11	88142031
ebi-a-GCST90001423	rs61906277	CD20- CD38- AC	11	123384801
ebi-a-GCST90001423	rs117308259	CD20- CD38- AC	14	31993501
ebi-a-GCST90001423	rs16955339	CD20- CD38- AC	17	52971060
ebi-a-GCST90001423	rs77510701	CD20- CD38- AC	18	34838106
ebi-a-GCST90001423	rs80339814	CD20- CD38- AC	19	53396009
ebi-a-GCST90001423	rs6125184	CD20- CD38- AC	20	46576714
ebi-a-GCST90001423	rs36071180	CD20- CD38- AC	20	765164
ebi-a-GCST90001424	rs143109147	IgD+ %Lymphocyte	1	40387371
ebi-a-GCST90001424	rs16837671	IgD+ %Lymphocyte	2	206655953
ebi-a-GCST90001424	rs141070091	IgD+ %Lymphocyte	4	46715880
ebi-a-GCST90001424	rs76845048	IgD+ %Lymphocyte	5	115785493
ebi-a-GCST90001424	rs4361609	IgD+ %Lymphocyte	6	31240635
ebi-a-GCST90001424	rs73078428	IgD+ %Lymphocyte	7	20106029
ebi-a-GCST90001424	rs111830700	IgD+ %Lymphocyte	7	121332400
ebi-a-GCST90001424	rs34910780	IgD+ %Lymphocyte	7	77469688
ebi-a-GCST90001424	rs2395904	IgD+ %Lymphocyte	8	130617819
ebi-a-GCST90001424	rs533239981	IgD+ %Lymphocyte	8	75515052
ebi-a-GCST90001424	rs1977295	IgD+ %Lymphocyte	10	7188484
ebi-a-GCST90001424	rs75319860	IgD+ %Lymphocyte	12	110312229
ebi-a-GCST90001424	rs116906133	IgD+ %Lymphocyte	13	100023905
ebi-a-GCST90001424	rs12874404	IgD+ %Lymphocyte	13	108993494
ebi-a-GCST90001424	rs2402519	IgD+ %Lymphocyte	14	95373465
ebi-a-GCST90001424	rs11657531	IgD+ %Lymphocyte	17	2731370

ebi-a-GCST90001424	rs12939565	IgD+ %Lymphocyte	17	38038389
ebi-a-GCST90001424	rs75956343	IgD+ %Lymphocyte	17	70210257
ebi-a-GCST90001424	rs55658072	IgD+ %Lymphocyte	19	14873284
ebi-a-GCST90001424	rs6131010	IgD+ %Lymphocyte	20	44724305
ebi-a-GCST90001425	rs970081	IgD- CD38br %lym	1	213101844
ebi-a-GCST90001425	rs78196740	IgD- CD38br %lym	1	63802723
ebi-a-GCST90001425	rs148362575	IgD- CD38br %lym	2	187165335
ebi-a-GCST90001425	rs62160934	IgD- CD38br %lym	2	136941539
ebi-a-GCST90001425	rs138158989	IgD- CD38br %lym	2	204476635
ebi-a-GCST90001425	rs66491584	IgD- CD38br %lym	3	20980398
ebi-a-GCST90001425	rs116540222	IgD- CD38br %lym	3	117331256
ebi-a-GCST90001425	rs79812543	IgD- CD38br %lym	3	157649504
ebi-a-GCST90001425	rs151009998	IgD- CD38br %lym	3	11712287
ebi-a-GCST90001425	rs11938966	IgD- CD38br %lym	4	55386841
ebi-a-GCST90001425	rs114428590	IgD- CD38br %lym	4	61089291
ebi-a-GCST90001425	rs79264549	IgD- CD38br %lym	5	89585751
ebi-a-GCST90001425	rs6554754	IgD- CD38br %lym	5	1297258
ebi-a-GCST90001425	rs139101653	IgD- CD38br %lym	6	31274174
ebi-a-GCST90001425	rs76624801	IgD- CD38br %lym	6	41935797
ebi-a-GCST90001425	rs187459109	IgD- CD38br %lym	6	40333488
ebi-a-GCST90001425	rs75346886	IgD- CD38br %lym	7	127285830
ebi-a-GCST90001425	rs138058221	IgD- CD38br %lym	7	132571443
ebi-a-GCST90001425	rs148377259	IgD- CD38br %lym	7	106306631
ebi-a-GCST90001425	rs12340211	IgD- CD38br %lym	9	8531036
ebi-a-GCST90001425	rs113087021	IgD- CD38br %lym	11	97765784
ebi-a-GCST90001425	rs478251	IgD- CD38br %lym	11	127827727
ebi-a-GCST90001425	rs6488283	IgD- CD38br %lym	12	10496783
ebi-a-GCST90001425	rs7294591	IgD- CD38br %lym	12	52932031
ebi-a-GCST90001425	rs11609248	IgD- CD38br %lym	12	14317207
ebi-a-GCST90001425	rs17037450	IgD- CD38br %lym	12	106098314
ebi-a-GCST90001425	rs72686093	IgD- CD38br %lym	14	22129890
ebi-a-GCST90001425	rs2627305	IgD- CD38br %lym	15	74539114
ebi-a-GCST90001425	rs4785074	IgD- CD38br %lym	16	59885737
ebi-a-GCST90001425	rs72834736	IgD- CD38br %lym	16	81821361
ebi-a-GCST90001425	rs76348547	IgD- CD38br %lym	22	31013921
ebi-a-GCST90001426	rs500586	IgD- CD38dim %ly	1	41075209
ebi-a-GCST90001426	rs72691171	IgD- CD38dim %ly	1	119873920
ebi-a-GCST90001426	rs143785715	IgD- CD38dim %ly	1	19500582
ebi-a-GCST90001426	rs10922490	IgD- CD38dim %ly	1	89286072
ebi-a-GCST90001426	rs11687597	IgD- CD38dim %ly	2	136926146
ebi-a-GCST90001426	rs7595246	IgD- CD38dim %ly	2	238554657
ebi-a-GCST90001426	rs71612685	IgD- CD38dim %ly	4	100279851
ebi-a-GCST90001426	rs1867929	IgD- CD38dim %ly	4	1628280
ebi-a-GCST90001426	rs114839471	IgD- CD38dim %ly	5	89545035
ebi-a-GCST90001426	rs17405603	IgD- CD38dim %ly	5	144608340
ebi-a-GCST90001426	rs146936090	IgD- CD38dim %ly	6	41401285
ebi-a-GCST90001426	rs6972846	IgD- CD38dim %ly	7	22048853
ebi-a-GCST90001426	rs78027513	IgD- CD38dim %ly	7	154618679

ebi-a-GCST90001426	rs7840395	IgD- CD38dim %ly	8	144984956
ebi-a-GCST90001426	rs2137277	IgD- CD38dim %ly	8	40734662
ebi-a-GCST90001426	rs72816858	IgD- CD38dim %ly	10	22940992
ebi-a-GCST90001426	rs12298227	IgD- CD38dim %ly	12	54786414
ebi-a-GCST90001426	rs593370	IgD- CD38dim %ly	12	53022961
ebi-a-GCST90001426	rs9520836	IgD- CD38dim %ly	13	108957063
ebi-a-GCST90001426	rs118086652	IgD- CD38dim %ly	15	76646870
ebi-a-GCST90001426	rs11653761	IgD- CD38dim %ly	17	2716535
ebi-a-GCST90001426	rs145744014	IgD- CD38dim %ly	18	61418402
ebi-a-GCST90001426	rs652344	IgD- CD38dim %ly	19	36290177
ebi-a-GCST90001426	rs2328245	IgD- CD38dim %ly	20	18194427
ebi-a-GCST90001426	rs134927	IgD- CD38dim %ly	22	27421843
ebi-a-GCST90001427	rs61790670	IgD- CD38- %lymp	1	84127219
ebi-a-GCST90001427	rs116661843	IgD- CD38- %lymp	1	178417994
ebi-a-GCST90001427	rs12128328	IgD- CD38- %lymp	1	59700800
ebi-a-GCST90001427	rs12624265	IgD- CD38- %lymp	2	119468602
ebi-a-GCST90001427	rs12992631	IgD- CD38- %lymp	2	232313158
ebi-a-GCST90001427	rs2084194	IgD- CD38- %lymp	2	47117185
ebi-a-GCST90001427	rs6723786	IgD- CD38- %lymp	2	238550072
ebi-a-GCST90001427	rs114551034	IgD- CD38- %lymp	2	66709035
ebi-a-GCST90001427	rs75524570	IgD- CD38- %lymp	3	191905058
ebi-a-GCST90001427	rs150378941	IgD- CD38- %lymp	4	133090875
ebi-a-GCST90001427	rs72942964	IgD- CD38- %lymp	4	134727496
ebi-a-GCST90001427	rs7676571	IgD- CD38- %lymp	4	56547047
ebi-a-GCST90001427	rs4697617	IgD- CD38- %lymp	4	25771115
ebi-a-GCST90001427	rs72711540	IgD- CD38- %lymp	4	186920475
ebi-a-GCST90001427	rs17405603	IgD- CD38- %lymp	5	144608340
ebi-a-GCST90001427	rs144625305	IgD- CD38- %lymp	7	7871676
ebi-a-GCST90001427	rs11777835	IgD- CD38- %lymp	8	144986103
ebi-a-GCST90001427	rs190799514	IgD- CD38- %lymp	8	55620993
ebi-a-GCST90001427	rs75116337	IgD- CD38- %lymp	8	55914981
ebi-a-GCST90001427	rs814418	IgD- CD38- %lymp	8	9936753
ebi-a-GCST90001427	rs4756916	IgD- CD38- %lymp	11	12544361
ebi-a-GCST90001427	rs17234595	IgD- CD38- %lymp	11	23667143
ebi-a-GCST90001427	rs12426629	IgD- CD38- %lymp	12	54797571
ebi-a-GCST90001427	rs9514859	IgD- CD38- %lymp	13	109144845
ebi-a-GCST90001427	rs12874404	IgD- CD38- %lymp	13	108993494
ebi-a-GCST90001427	rs4474666	IgD- CD38- %lymp	16	85313662
ebi-a-GCST90001427	rs11653761	IgD- CD38- %lymp	17	2716535
ebi-a-GCST90001427	rs34157595	IgD- CD38- %lymp	17	49200947
ebi-a-GCST90001427	rs7236090	IgD- CD38- %lymp	18	60947111
ebi-a-GCST90001427	rs77741094	IgD- CD38- %lymp	21	42362418
ebi-a-GCST90001428	rs112904733	IgD- CD38dim %B	1	214958496
ebi-a-GCST90001428	rs143109147	IgD- CD38dim %B	1	40387371
ebi-a-GCST90001428	rs12118242	IgD- CD38dim %B	1	232094153
ebi-a-GCST90001428	rs9424499	IgD- CD38dim %B	1	233050029
ebi-a-GCST90001428	rs16832731	IgD- CD38dim %B	2	136864703
ebi-a-GCST90001428	rs138919897	IgD- CD38dim %B	3	106834795

ebi-a-GCST90001428	rs73205209	IgD- CD38dim %B	3	106275239
ebi-a-GCST90001428	rs111854570	IgD- CD38dim %B	3	8695105
ebi-a-GCST90001428	rs114839471	IgD- CD38dim %B	5	89545035
ebi-a-GCST90001428	rs17405749	IgD- CD38dim %B	5	144609363
ebi-a-GCST90001428	rs80088375	IgD- CD38dim %B	5	11638274
ebi-a-GCST90001428	rs75273600	IgD- CD38dim %B	6	125594743
ebi-a-GCST90001428	rs849133	IgD- CD38dim %B	7	28192280
ebi-a-GCST90001428	rs146267894	IgD- CD38dim %B	9	75147206
ebi-a-GCST90001428	rs2397752	IgD- CD38dim %B	10	132983821
ebi-a-GCST90001428	rs117987395	IgD- CD38dim %B	10	10573290
ebi-a-GCST90001428	rs80216562	IgD- CD38dim %B	11	128322602
ebi-a-GCST90001428	rs10743163	IgD- CD38dim %B	11	11033950
ebi-a-GCST90001428	rs61946381	IgD- CD38dim %B	12	121343559
ebi-a-GCST90001428	rs1846398	IgD- CD38dim %B	12	57302799
ebi-a-GCST90001428	rs4038038	IgD- CD38dim %B	13	19499423
ebi-a-GCST90001428	rs117227598	IgD- CD38dim %B	15	77402748
ebi-a-GCST90001428	rs11548656	IgD- CD38dim %B	16	81916912
ebi-a-GCST90001428	rs75659776	IgD- CD38dim %B	17	49605668
ebi-a-GCST90001428	rs117541917	IgD- CD38dim %B	17	41946947
ebi-a-GCST90001428	rs113530462	IgD- CD38dim %B	20	6688542
ebi-a-GCST90001429	rs1919856	IgD+ CD38br %lyn	1	247700906
ebi-a-GCST90001429	rs11682311	IgD+ CD38br %lyn	2	16464977
ebi-a-GCST90001429	rs4907205	IgD+ CD38br %lyn	2	97558150
ebi-a-GCST90001429	rs140380950	IgD+ CD38br %lyn	2	105587803
ebi-a-GCST90001429	rs2686423	IgD+ CD38br %lyn	3	183195708
ebi-a-GCST90001429	rs76112278	IgD+ CD38br %lyn	4	15915069
ebi-a-GCST90001429	rs7667070	IgD+ CD38br %lyn	4	38379833
ebi-a-GCST90001429	rs78658073	IgD+ CD38br %lyn	5	498773
ebi-a-GCST90001429	rs80330977	IgD+ CD38br %lyn	5	158276610
ebi-a-GCST90001429	rs76660971	IgD+ CD38br %lyn	5	18919526
ebi-a-GCST90001429	rs2857599	IgD+ CD38br %lyn	6	31562150
ebi-a-GCST90001429	rs117772462	IgD+ CD38br %lyn	7	48791017
ebi-a-GCST90001429	rs11185560	IgD+ CD38br %lyn	7	190115
ebi-a-GCST90001429	rs10103048	IgD+ CD38br %lyn	8	130602281
ebi-a-GCST90001429	rs1695681	IgD+ CD38br %lyn	8	118255950
ebi-a-GCST90001429	rs13300113	IgD+ CD38br %lyn	9	115678594
ebi-a-GCST90001429	rs10821937	IgD+ CD38br %lyn	10	63723909
ebi-a-GCST90001429	rs10832409	IgD+ CD38br %lyn	11	15377699
ebi-a-GCST90001429	rs11068290	IgD+ CD38br %lyn	12	110228055
ebi-a-GCST90001429	rs59879144	IgD+ CD38br %lyn	12	75564449
ebi-a-GCST90001429	rs10877626	IgD+ CD38br %lyn	12	61758121
ebi-a-GCST90001429	rs17408660	IgD+ CD38br %lyn	12	27184482
ebi-a-GCST90001429	rs75589249	IgD+ CD38br %lyn	14	43915569
ebi-a-GCST90001429	rs3811315	IgD+ CD38br %lyn	14	22499870
ebi-a-GCST90001429	rs2216762	IgD+ CD38br %lyn	16	84759446
ebi-a-GCST90001429	rs60521331	IgD+ CD38br %lyn	17	8639905
ebi-a-GCST90001429	rs55708924	IgD+ CD38br %lyn	21	16767346
ebi-a-GCST90001430	rs143109147	IgD+ CD38dim %ly	1	40387371

ebi-a-GCST90001430	rs55877424	IgD+ CD38dim %ly	2	12243809
ebi-a-GCST90001430	rs149290264	IgD+ CD38dim %ly	3	41659628
ebi-a-GCST90001430	rs182174914	IgD+ CD38dim %ly	3	80085882
ebi-a-GCST90001430	rs10933716	IgD+ CD38dim %ly	3	193840329
ebi-a-GCST90001430	rs28399382	IgD+ CD38dim %ly	3	115393238
ebi-a-GCST90001430	rs28654727	IgD+ CD38dim %ly	4	61631854
ebi-a-GCST90001430	rs6831487	IgD+ CD38dim %ly	4	170331972
ebi-a-GCST90001430	rs75512885	IgD+ CD38dim %ly	5	158317602
ebi-a-GCST90001430	rs1510971	IgD+ CD38dim %ly	5	118216796
ebi-a-GCST90001430	rs12516105	IgD+ CD38dim %ly	5	160198880
ebi-a-GCST90001430	rs4361609	IgD+ CD38dim %ly	6	31240635
ebi-a-GCST90001430	rs9459562	IgD+ CD38dim %ly	6	166502048
ebi-a-GCST90001430	rs34910780	IgD+ CD38dim %ly	7	77469688
ebi-a-GCST90001430	rs62474511	IgD+ CD38dim %ly	7	123275484
ebi-a-GCST90001430	rs4716961	IgD+ CD38dim %ly	7	155224134
ebi-a-GCST90001430	rs7840067	IgD+ CD38dim %ly	8	130582787
ebi-a-GCST90001430	rs533239981	IgD+ CD38dim %ly	8	75515052
ebi-a-GCST90001430	rs60953943	IgD+ CD38dim %ly	8	60077245
ebi-a-GCST90001430	rs76400856	IgD+ CD38dim %ly	10	56638876
ebi-a-GCST90001430	rs74151247	IgD+ CD38dim %ly	10	97801073
ebi-a-GCST90001430	rs75319860	IgD+ CD38dim %ly	12	110312229
ebi-a-GCST90001430	rs139695291	IgD+ CD38dim %ly	13	104519594
ebi-a-GCST90001430	rs75474045	IgD+ CD38dim %ly	13	72428296
ebi-a-GCST90001430	rs12874404	IgD+ CD38dim %ly	13	108993494
ebi-a-GCST90001430	rs116844769	IgD+ CD38dim %ly	14	65713895
ebi-a-GCST90001430	rs16974449	IgD+ CD38dim %ly	16	84754964
ebi-a-GCST90001430	rs60521331	IgD+ CD38dim %ly	17	8639905
ebi-a-GCST90001430	rs11657531	IgD+ CD38dim %ly	17	2731370
ebi-a-GCST90001430	rs2291516	IgD+ CD38dim %ly	19	11508177
ebi-a-GCST90001430	rs655580	IgD+ CD38dim %ly	19	53039110
ebi-a-GCST90001430	rs6131010	IgD+ CD38dim %ly	20	44724305
ebi-a-GCST90001430	rs55708924	IgD+ CD38dim %ly	21	16767346
ebi-a-GCST90001430	rs143518319	IgD+ CD38dim %ly	21	36295623
ebi-a-GCST90001430	rs142838362	IgD+ CD38dim %ly	21	47606966
ebi-a-GCST90001431	rs149701635	IgD+ CD38- %lymp	3	44741083
ebi-a-GCST90001431	rs35088452	IgD+ CD38- %lymp	3	8847483
ebi-a-GCST90001431	rs6895553	IgD+ CD38- %lymp	5	114821667
ebi-a-GCST90001431	rs78009119	IgD+ CD38- %lymp	5	136421498
ebi-a-GCST90001431	rs77055472	IgD+ CD38- %lymp	5	157655991
ebi-a-GCST90001431	rs12197754	IgD+ CD38- %lymp	6	130521070
ebi-a-GCST90001431	rs144625305	IgD+ CD38- %lymp	7	7871676
ebi-a-GCST90001431	rs12763172	IgD+ CD38- %lymp	10	85447519
ebi-a-GCST90001431	rs12146653	IgD+ CD38- %lymp	11	21411891
ebi-a-GCST90001431	rs12829274	IgD+ CD38- %lymp	12	129349167
ebi-a-GCST90001431	rs3730631	IgD+ CD38- %lymp	12	69228178
ebi-a-GCST90001431	rs12874404	IgD+ CD38- %lymp	13	108993494
ebi-a-GCST90001431	rs112782756	IgD+ CD38- %lymp	13	34506073
ebi-a-GCST90001431	rs2003291	IgD+ CD38- %lymp	13	21566923

ebi-a-GCST90001431	rs77762131	IgD+ CD38- %lymp	14	96205838
ebi-a-GCST90001431	rs72706301	IgD+ CD38- %lymp	14	94852219
ebi-a-GCST90001431	rs149731447	IgD+ CD38- %lymp	14	52536948
ebi-a-GCST90001431	rs10444793	IgD+ CD38- %lymp	15	44517689
ebi-a-GCST90001431	rs28594458	IgD+ CD38- %lymp	17	2728777
ebi-a-GCST90001431	rs62235484	IgD+ CD38- %lymp	21	41083050
ebi-a-GCST90001431	rs78007261	IgD+ CD38- %lymp	22	45666517
ebi-a-GCST90001432	rs75766272	Unsw Mem %lymph	1	8009985
ebi-a-GCST90001432	rs56326639	Unsw Mem %lymph	3	32512523
ebi-a-GCST90001432	rs6557137	Unsw Mem %lymph	6	150197022
ebi-a-GCST90001432	rs144625305	Unsw Mem %lymph	7	7871676
ebi-a-GCST90001432	rs2956955	Unsw Mem %lymph	8	1552537
ebi-a-GCST90001432	rs141247883	Unsw Mem %lymph	8	144986071
ebi-a-GCST90001432	rs57757402	Unsw Mem %lymph	9	37390411
ebi-a-GCST90001432	rs11794578	Unsw Mem %lymph	9	808529
ebi-a-GCST90001432	rs10119513	Unsw Mem %lymph	9	16899526
ebi-a-GCST90001432	rs10840410	Unsw Mem %lymph	11	10437096
ebi-a-GCST90001432	rs10894530	Unsw Mem %lymph	11	132159459
ebi-a-GCST90001432	rs143553941	Unsw Mem %lymph	12	109368119
ebi-a-GCST90001432	rs12829274	Unsw Mem %lymph	12	129349167
ebi-a-GCST90001432	rs73192651	Unsw Mem %lymph	13	50212885
ebi-a-GCST90001432	rs12874404	Unsw Mem %lymph	13	108993494
ebi-a-GCST90001432	rs557577736	Unsw Mem %lymph	13	46224092
ebi-a-GCST90001432	rs77762131	Unsw Mem %lymph	14	96205838
ebi-a-GCST90001432	rs8014978	Unsw Mem %lymph	14	94936964
ebi-a-GCST90001432	rs112135308	Unsw Mem %lymph	16	86794463
ebi-a-GCST90001432	rs11657759	Unsw Mem %lymph	17	2715961
ebi-a-GCST90001432	rs7254039	Unsw Mem %lymph	19	14993354
ebi-a-GCST90001432	rs6032660	Unsw Mem %lymph	20	44730245
ebi-a-GCST90001432	rs76762356	Unsw Mem %lymph	20	10454080
ebi-a-GCST90001432	rs55712190	Unsw Mem %lymph	22	22613458
ebi-a-GCST90001433	rs2789568	IgD- CD27- %lymp	1	217179038
ebi-a-GCST90001433	rs113442137	IgD- CD27- %lymp	1	160254955
ebi-a-GCST90001433	rs185621536	IgD- CD27- %lymp	1	113524501
ebi-a-GCST90001433	rs7606024	IgD- CD27- %lymp	2	56616595
ebi-a-GCST90001433	rs35574803	IgD- CD27- %lymp	3	32533246
ebi-a-GCST90001433	rs78756830	IgD- CD27- %lymp	4	99437210
ebi-a-GCST90001433	rs12654363	IgD- CD27- %lymp	5	84064142
ebi-a-GCST90001433	rs191610741	IgD- CD27- %lymp	6	4201348
ebi-a-GCST90001433	rs807894	IgD- CD27- %lymp	6	164610887
ebi-a-GCST90001433	rs1978170	IgD- CD27- %lymp	7	38757409
ebi-a-GCST90001433	rs78763642	IgD- CD27- %lymp	9	25294308
ebi-a-GCST90001433	rs2250246	IgD- CD27- %lymp	12	6553016
ebi-a-GCST90001433	rs77114347	IgD- CD27- %lymp	16	7452768
ebi-a-GCST90001433	rs4941188	IgD- CD27- %lymp	18	60857029
ebi-a-GCST90001433	rs111407982	IgD- CD27- %lymp	19	8814367
ebi-a-GCST90001433	rs7260348	IgD- CD27- %lymp	19	15011442
ebi-a-GCST90001433	rs77126700	IgD- CD27- %lymp	20	47313887

ebi-a-GCST90001433	rs4810485	IgD- CD27- %lymp	20	44747947
ebi-a-GCST90001434	rs7595246	Sw mem %lymphoc	2	238554657
ebi-a-GCST90001434	rs1519526	Sw mem %lymphoc	2	136967396
ebi-a-GCST90001434	rs7640978	Sw mem %lymphoc	3	32533010
ebi-a-GCST90001434	rs17029267	Sw mem %lymphoc	4	100572746
ebi-a-GCST90001434	rs62382785	Sw mem %lymphoc	5	165581566
ebi-a-GCST90001434	rs17405603	Sw mem %lymphoc	5	144608340
ebi-a-GCST90001434	rs190799514	Sw mem %lymphoc	8	55620993
ebi-a-GCST90001434	rs7840395	Sw mem %lymphoc	8	144984956
ebi-a-GCST90001434	rs814418	Sw mem %lymphoc	8	9936753
ebi-a-GCST90001434	rs118076634	Sw mem %lymphoc	11	94589940
ebi-a-GCST90001434	rs12426629	Sw mem %lymphoc	12	54797571
ebi-a-GCST90001434	rs726449	Sw mem %lymphoc	13	109151489
ebi-a-GCST90001434	rs9520836	Sw mem %lymphoc	13	108957063
ebi-a-GCST90001434	rs11653761	Sw mem %lymphoc	17	2716535
ebi-a-GCST90001434	rs145744014	Sw mem %lymphoc	18	61418402
ebi-a-GCST90001434	rs138728953	Sw mem %lymphoc	19	14698416
ebi-a-GCST90001434	rs117231919	Sw mem %lymphoc	19	652628
ebi-a-GCST90001434	rs377621503	Sw mem %lymphoc	19	645485
ebi-a-GCST90001435	rs141365416	PB/PC %lymphocy	1	102236814
ebi-a-GCST90001435	rs148362575	PB/PC %lymphocy	2	187165335
ebi-a-GCST90001435	rs116540222	PB/PC %lymphocy	3	117331256
ebi-a-GCST90001435	rs7449190	PB/PC %lymphocy	5	1297918
ebi-a-GCST90001435	rs61294310	PB/PC %lymphocy	6	5266557
ebi-a-GCST90001435	rs13244540	PB/PC %lymphocy	7	151002534
ebi-a-GCST90001435	rs62519719	PB/PC %lymphocy	8	118376967
ebi-a-GCST90001435	rs74326785	PB/PC %lymphocy	8	24549984
ebi-a-GCST90001435	rs3138500	PB/PC %lymphocy	9	92219945
ebi-a-GCST90001435	rs12412378	PB/PC %lymphocy	10	130483163
ebi-a-GCST90001435	rs1938685	PB/PC %lymphocy	11	69277078
ebi-a-GCST90001435	rs897989	PB/PC %lymphocy	11	91973528
ebi-a-GCST90001435	rs55728331	PB/PC %lymphocy	11	117526440
ebi-a-GCST90001435	rs10849436	PB/PC %lymphocy	12	6409619
ebi-a-GCST90001435	rs1838357	PB/PC %lymphocy	13	93740906
ebi-a-GCST90001435	rs4785074	PB/PC %lymphocy	16	59885737
ebi-a-GCST90001435	rs7255292	PB/PC %lymphocy	19	49529379
ebi-a-GCST90001435	rs8132059	PB/PC %lymphocy	21	16916284
ebi-a-GCST90001435	rs11089305	PB/PC %lymphocy	22	19729361
ebi-a-GCST90001436	rs75414548	Memory B cell %ly	1	39385714
ebi-a-GCST90001436	rs75766272	Memory B cell %ly	1	8009985
ebi-a-GCST90001436	rs878471	Memory B cell %ly	1	150547747
ebi-a-GCST90001436	rs12636848	Memory B cell %ly	3	147613080
ebi-a-GCST90001436	rs143003356	Memory B cell %ly	4	142016471
ebi-a-GCST90001436	rs17708396	Memory B cell %ly	5	148410250
ebi-a-GCST90001436	rs6895553	Memory B cell %ly	5	114821667
ebi-a-GCST90001436	rs1770731	Memory B cell %ly	6	107001456
ebi-a-GCST90001436	rs74505387	Memory B cell %ly	6	101891742
ebi-a-GCST90001436	rs410173	Memory B cell %ly	6	1163541

ebi-a-GCST90001436	rs144625305	Memory B cell %ly	7	7871676
ebi-a-GCST90001436	rs6558507	Memory B cell %ly	8	1626220
ebi-a-GCST90001436	rs34173062	Memory B cell %ly	8	145158607
ebi-a-GCST90001436	rs12763172	Memory B cell %ly	10	85447519
ebi-a-GCST90001436	rs10791220	Memory B cell %ly	11	132145598
ebi-a-GCST90001436	rs11225155	Memory B cell %ly	11	102043400
ebi-a-GCST90001436	rs74839056	Memory B cell %ly	13	107914821
ebi-a-GCST90001436	rs557577736	Memory B cell %ly	13	46224092
ebi-a-GCST90001436	rs12874404	Memory B cell %ly	13	108993494
ebi-a-GCST90001436	rs9514859	Memory B cell %ly	13	109144845
ebi-a-GCST90001436	rs73192651	Memory B cell %ly	13	50212885
ebi-a-GCST90001436	rs77762131	Memory B cell %ly	14	96205838
ebi-a-GCST90001436	rs974368	Memory B cell %ly	14	63009549
ebi-a-GCST90001436	rs139196765	Memory B cell %ly	15	24733764
ebi-a-GCST90001436	rs11653761	Memory B cell %ly	17	2716535
ebi-a-GCST90001436	rs7254039	Memory B cell %ly	19	14993354
ebi-a-GCST90001436	rs551529415	Memory B cell %ly	19	1157330
ebi-a-GCST90001436	rs3810559	Memory B cell %ly	20	3207749
ebi-a-GCST90001436	rs4640493	Memory B cell %ly	22	45387786
ebi-a-GCST90001436	rs9614226	Memory B cell %ly	22	44688150
ebi-a-GCST90001437	rs11937214	Naive-mature B cel	4	162634026
ebi-a-GCST90001437	rs76845048	Naive-mature B cel	5	115785493
ebi-a-GCST90001437	rs115204570	Naive-mature B cel	5	135756457
ebi-a-GCST90001437	rs17056318	Naive-mature B cel	5	158285953
ebi-a-GCST90001437	rs4361609	Naive-mature B cel	6	31240635
ebi-a-GCST90001437	rs35607968	Naive-mature B cel	7	67653879
ebi-a-GCST90001437	rs111830700	Naive-mature B cel	7	121332400
ebi-a-GCST90001437	rs140197720	Naive-mature B cel	7	108191234
ebi-a-GCST90001437	rs2395904	Naive-mature B cel	8	130617819
ebi-a-GCST90001437	rs4361743	Naive-mature B cel	8	132657435
ebi-a-GCST90001437	rs533239981	Naive-mature B cel	8	75515052
ebi-a-GCST90001437	rs2978582	Naive-mature B cel	8	35485504
ebi-a-GCST90001437	rs74151247	Naive-mature B cel	10	97801073
ebi-a-GCST90001437	rs56097028	Naive-mature B cel	11	43575416
ebi-a-GCST90001437	rs74397782	Naive-mature B cel	12	25018659
ebi-a-GCST90001437	rs78433555	Naive-mature B cel	12	107821321
ebi-a-GCST90001437	rs116844769	Naive-mature B cel	14	65713895
ebi-a-GCST90001437	rs112850074	Naive-mature B cel	14	103229069
ebi-a-GCST90001437	rs12939565	Naive-mature B cel	17	38038389
ebi-a-GCST90001437	rs11653761	Naive-mature B cel	17	2716535
ebi-a-GCST90001437	rs4805840	Naive-mature B cel	19	33468482
ebi-a-GCST90001437	rs55658072	Naive-mature B cel	19	14873284
ebi-a-GCST90001437	rs7270264	Naive-mature B cel	20	42078615
ebi-a-GCST90001437	rs55708924	Naive-mature B cel	21	16767346
ebi-a-GCST90001438	rs77848220	IgD- CD38dim AC	1	61251434
ebi-a-GCST90001438	rs7595246	IgD- CD38dim AC	2	238554657
ebi-a-GCST90001438	rs115414430	IgD- CD38dim AC	2	36720835
ebi-a-GCST90001438	rs1519524	IgD- CD38dim AC	2	136934125

ebi-a-GCST90001438	rs141352577	IgD- CD38dim AC	3	8492010
ebi-a-GCST90001438	rs73185679	IgD- CD38dim AC	3	186485863
ebi-a-GCST90001438	rs7840395	IgD- CD38dim AC	8	144984956
ebi-a-GCST90001438	rs16925715	IgD- CD38dim AC	10	53772842
ebi-a-GCST90001438	rs149006488	IgD- CD38dim AC	11	67523715
ebi-a-GCST90001438	rs61910510	IgD- CD38dim AC	11	130557122
ebi-a-GCST90001438	rs4936106	IgD- CD38dim AC	11	130360402
ebi-a-GCST90001438	rs79407211	IgD- CD38dim AC	13	109119262
ebi-a-GCST90001438	rs73178168	IgD- CD38dim AC	13	47809629
ebi-a-GCST90001438	rs192544457	IgD- CD38dim AC	13	42337460
ebi-a-GCST90001438	rs9520836	IgD- CD38dim AC	13	108957063
ebi-a-GCST90001438	rs60035843	IgD- CD38dim AC	15	69123222
ebi-a-GCST90001438	rs11653761	IgD- CD38dim AC	17	2716535
ebi-a-GCST90001438	rs117605782	IgD- CD38dim AC	18	46442174
ebi-a-GCST90001438	rs533086	IgD- CD38dim AC	19	16411841
ebi-a-GCST90001438	rs78893742	IgD- CD38dim AC	20	36100011
ebi-a-GCST90001439	rs61788860	IgD+ CD24+ %lym	1	60020239
ebi-a-GCST90001439	rs75766272	IgD+ CD24+ %lym	1	8009985
ebi-a-GCST90001439	rs2015983	IgD+ CD24+ %lym	2	237150166
ebi-a-GCST90001439	rs6781675	IgD+ CD24+ %lym	3	4408435
ebi-a-GCST90001439	rs1382818	IgD+ CD24+ %lym	6	153645785
ebi-a-GCST90001439	rs4937689	IgD+ CD24+ %lym	11	132141201
ebi-a-GCST90001439	rs183466766	IgD+ CD24+ %lym	13	108710731
ebi-a-GCST90001439	rs12874404	IgD+ CD24+ %lym	13	108993494
ebi-a-GCST90001439	rs141107540	IgD+ CD24+ %lym	13	109038407
ebi-a-GCST90001439	rs73192651	IgD+ CD24+ %lym	13	50212885
ebi-a-GCST90001439	rs77762131	IgD+ CD24+ %lym	14	96205838
ebi-a-GCST90001439	rs8014978	IgD+ CD24+ %lym	14	94936964
ebi-a-GCST90001439	rs11657118	IgD+ CD24+ %lym	17	2714118
ebi-a-GCST90001439	rs7254039	IgD+ CD24+ %lym	19	14993354
ebi-a-GCST90001439	rs3810559	IgD+ CD24+ %lym	20	3207749
ebi-a-GCST90001440	rs6687573	IgD- CD24- %lymp	1	35281308
ebi-a-GCST90001440	rs55756125	IgD- CD24- %lymp	1	59740965
ebi-a-GCST90001440	rs111237501	IgD- CD24- %lymp	1	163272701
ebi-a-GCST90001440	rs11674994	IgD- CD24- %lymp	2	60125363
ebi-a-GCST90001440	rs72990172	IgD- CD24- %lymp	2	238526279
ebi-a-GCST90001440	rs76225955	IgD- CD24- %lymp	4	31594004
ebi-a-GCST90001440	rs17044633	IgD- CD24- %lymp	4	113331648
ebi-a-GCST90001440	rs183821560	IgD- CD24- %lymp	5	62072324
ebi-a-GCST90001440	rs807894	IgD- CD24- %lymp	6	164610887
ebi-a-GCST90001440	rs9398107	IgD- CD24- %lymp	6	107155687
ebi-a-GCST90001440	rs75464772	IgD- CD24- %lymp	6	19788226
ebi-a-GCST90001440	rs6991364	IgD- CD24- %lymp	8	145050345
ebi-a-GCST90001440	rs146221269	IgD- CD24- %lymp	11	94920856
ebi-a-GCST90001440	rs73277142	IgD- CD24- %lymp	12	11974332
ebi-a-GCST90001440	rs11066272	IgD- CD24- %lymp	12	112802224
ebi-a-GCST90001440	rs139509958	IgD- CD24- %lymp	12	54727027
ebi-a-GCST90001440	rs191543445	IgD- CD24- %lymp	14	31505138

ebi-a-GCST90001440	rs17102161	IgD- CD24- %lymp	14	65283958
ebi-a-GCST90001440	rs496760	IgD- CD24- %lymp	15	54695686
ebi-a-GCST90001440	rs75150138	IgD- CD24- %lymp	16	49238584
ebi-a-GCST90001440	rs11862268	IgD- CD24- %lymp	16	81239867
ebi-a-GCST90001440	rs58904842	IgD- CD24- %lymp	16	3463156
ebi-a-GCST90001440	rs30146	IgD- CD24- %lymp	16	14342183
ebi-a-GCST90001440	rs8098848	IgD- CD24- %lymp	18	60857793
ebi-a-GCST90001440	rs451367	IgD- CD24- %lymp	19	16412842
ebi-a-GCST90001441	rs16837671	IgD+ CD24- %lymp	2	206655953
ebi-a-GCST90001441	rs520786	IgD+ CD24- %lymp	3	187559064
ebi-a-GCST90001441	rs34712193	IgD+ CD24- %lymp	3	1814140
ebi-a-GCST90001441	rs17056318	IgD+ CD24- %lymp	5	158285953
ebi-a-GCST90001441	rs76845048	IgD+ CD24- %lymp	5	115785493
ebi-a-GCST90001441	rs115204570	IgD+ CD24- %lymp	5	135756457
ebi-a-GCST90001441	rs140523784	IgD+ CD24- %lymp	6	16887609
ebi-a-GCST90001441	rs9354413	IgD+ CD24- %lymp	6	67039330
ebi-a-GCST90001441	rs4361609	IgD+ CD24- %lymp	6	31240635
ebi-a-GCST90001441	rs111830700	IgD+ CD24- %lymp	7	121332400
ebi-a-GCST90001441	rs34910780	IgD+ CD24- %lymp	7	77469688
ebi-a-GCST90001441	rs12542190	IgD+ CD24- %lymp	8	130594761
ebi-a-GCST90001441	rs2978582	IgD+ CD24- %lymp	8	35485504
ebi-a-GCST90001441	rs533239981	IgD+ CD24- %lymp	8	75515052
ebi-a-GCST90001441	rs71483382	IgD+ CD24- %lymp	10	83763586
ebi-a-GCST90001441	rs75319860	IgD+ CD24- %lymp	12	110312229
ebi-a-GCST90001441	rs76578043	IgD+ CD24- %lymp	13	105214810
ebi-a-GCST90001441	rs2994690	IgD+ CD24- %lymp	14	95363646
ebi-a-GCST90001441	rs116844769	IgD+ CD24- %lymp	14	65713895
ebi-a-GCST90001441	rs9900847	IgD+ CD24- %lymp	17	2717218
ebi-a-GCST90001441	rs12939565	IgD+ CD24- %lymp	17	38038389
ebi-a-GCST90001441	rs73426855	IgD+ CD24- %lymp	17	77637123
ebi-a-GCST90001441	rs4805840	IgD+ CD24- %lymp	19	33468482
ebi-a-GCST90001441	rs6131010	IgD+ CD24- %lymp	20	44724305
ebi-a-GCST90001442	rs75766272	CD24+ CD27+ %ly	1	8009985
ebi-a-GCST90001442	rs76720471	CD24+ CD27+ %ly	2	222274805
ebi-a-GCST90001442	rs1519524	CD24+ CD27+ %ly	2	136934125
ebi-a-GCST90001442	rs112542522	CD24+ CD27+ %ly	2	4031340
ebi-a-GCST90001442	rs12636848	CD24+ CD27+ %ly	3	147613080
ebi-a-GCST90001442	rs4689417	CD24+ CD27+ %ly	4	4392393
ebi-a-GCST90001442	rs17708396	CD24+ CD27+ %ly	5	148410250
ebi-a-GCST90001442	rs35057381	CD24+ CD27+ %ly	5	26412301
ebi-a-GCST90001442	rs4076316	CD24+ CD27+ %ly	6	167859623
ebi-a-GCST90001442	rs74505387	CD24+ CD27+ %ly	6	101891742
ebi-a-GCST90001442	rs144625305	CD24+ CD27+ %ly	7	7871676
ebi-a-GCST90001442	rs6997704	CD24+ CD27+ %ly	8	140978857
ebi-a-GCST90001442	rs1332199	CD24+ CD27+ %ly	9	9503477
ebi-a-GCST90001442	rs144440894	CD24+ CD27+ %ly	10	53795073
ebi-a-GCST90001442	rs6486433	CD24+ CD27+ %ly	11	9594690
ebi-a-GCST90001442	rs4763704	CD24+ CD27+ %ly	12	11698759

ebi-a-GCST90001442	rs12829274	CD24+ CD27+ %ly	12	129349167
ebi-a-GCST90001442	rs12874404	CD24+ CD27+ %ly	13	108993494
ebi-a-GCST90001442	rs557577736	CD24+ CD27+ %ly	13	46224092
ebi-a-GCST90001442	rs73192651	CD24+ CD27+ %ly	13	50212885
ebi-a-GCST90001442	rs112782756	CD24+ CD27+ %ly	13	34506073
ebi-a-GCST90001442	rs9578529	CD24+ CD27+ %ly	13	23613190
ebi-a-GCST90001442	rs77762131	CD24+ CD27+ %ly	14	96205838
ebi-a-GCST90001442	rs11657759	CD24+ CD27+ %ly	17	2715961
ebi-a-GCST90001442	rs7254039	CD24+ CD27+ %ly	19	14993354
ebi-a-GCST90001443	rs6712095	CD20- %lymphocyt	2	205623497
ebi-a-GCST90001443	rs34625183	CD20- %lymphocyt	3	146097919
ebi-a-GCST90001443	rs75951284	CD20- %lymphocyt	3	187638796
ebi-a-GCST90001443	rs186229706	CD20- %lymphocyt	4	2029089
ebi-a-GCST90001443	rs4647601	CD20- %lymphocyt	4	185570032
ebi-a-GCST90001443	rs72791978	CD20- %lymphocyt	5	108070072
ebi-a-GCST90001443	rs9273241	CD20- %lymphocyt	6	32614025
ebi-a-GCST90001443	rs7773849	CD20- %lymphocyt	6	128553518
ebi-a-GCST90001443	rs13244540	CD20- %lymphocyt	7	151002534
ebi-a-GCST90001443	rs38201	CD20- %lymphocyt	7	15911408
ebi-a-GCST90001443	rs34313582	CD20- %lymphocyt	8	84919811
ebi-a-GCST90001443	rs72825207	CD20- %lymphocyt	10	62909955
ebi-a-GCST90001443	rs147937648	CD20- %lymphocyt	12	82008771
ebi-a-GCST90001443	rs77553819	CD20- %lymphocyt	13	24882401
ebi-a-GCST90001443	rs12432015	CD20- %lymphocyt	14	49052782
ebi-a-GCST90001443	rs4244568	CD20- %lymphocyt	15	39319915
ebi-a-GCST90001443	rs252294	CD20- %lymphocyt	16	29235533
ebi-a-GCST90001443	rs4782702	CD20- %lymphocyt	16	85477299
ebi-a-GCST90001443	rs11662133	CD20- %lymphocyt	18	74434633
ebi-a-GCST90001443	rs186355283	CD20- %lymphocyt	20	46615708
ebi-a-GCST90001443	rs6052993	CD20- %lymphocyt	20	4944838
ebi-a-GCST90001444	rs75839951	CD20- CD38- %lyn	2	34491589
ebi-a-GCST90001444	rs4619804	CD20- CD38- %lyn	3	18674644
ebi-a-GCST90001444	rs709082	CD20- CD38- %lyn	3	191447446
ebi-a-GCST90001444	rs2215848	CD20- CD38- %lyn	4	25065221
ebi-a-GCST90001444	rs182720746	CD20- CD38- %lyn	4	113092883
ebi-a-GCST90001444	rs62311900	CD20- CD38- %lyn	4	74145187
ebi-a-GCST90001444	rs2339996	CD20- CD38- %lyn	5	173070505
ebi-a-GCST90001444	rs72492305	CD20- CD38- %lyn	6	32509333
ebi-a-GCST90001444	rs6906886	CD20- CD38- %lyn	6	85038851
ebi-a-GCST90001444	rs10279688	CD20- CD38- %lyn	7	9851008
ebi-a-GCST90001444	rs11203884	CD20- CD38- %lyn	8	17516564
ebi-a-GCST90001444	rs118073973	CD20- CD38- %lyn	9	19948908
ebi-a-GCST90001444	rs55767134	CD20- CD38- %lyn	10	17048435
ebi-a-GCST90001444	rs308820	CD20- CD38- %lyn	11	88142031
ebi-a-GCST90001444	rs34666228	CD20- CD38- %lyn	11	123385425
ebi-a-GCST90001444	rs78301150	CD20- CD38- %lyn	12	82233392
ebi-a-GCST90001444	rs117308259	CD20- CD38- %lyn	14	31993501
ebi-a-GCST90001444	rs35639639	CD20- CD38- %lyn	16	5488091

ebi-a-GCST90001444	rs111737298	CD20- CD38- %lyr	17	52847291
ebi-a-GCST90001444	rs16955339	CD20- CD38- %lyr	17	52971060
ebi-a-GCST90001444	rs77510701	CD20- CD38- %lyr	18	34838106
ebi-a-GCST90001444	rs80339814	CD20- CD38- %lyr	19	53396009
ebi-a-GCST90001444	rs6125184	CD20- CD38- %lyr	20	46576714
ebi-a-GCST90001444	rs59902361	CD20- CD38- %lyr	20	38334734
ebi-a-GCST90001444	rs36071180	CD20- CD38- %lyr	20	765164
ebi-a-GCST90001445	rs61790670	IgD- CD38- %B cel	1	84127219
ebi-a-GCST90001445	rs115576360	IgD- CD38- %B cel	4	156996644
ebi-a-GCST90001445	rs4691920	IgD- CD38- %B cel	4	164351901
ebi-a-GCST90001445	rs7726548	IgD- CD38- %B cel	5	116807553
ebi-a-GCST90001445	rs17405603	IgD- CD38- %B cel	5	144608340
ebi-a-GCST90001445	rs62417843	IgD- CD38- %B cel	6	91779902
ebi-a-GCST90001445	rs9265864	IgD- CD38- %B cel	6	31312747
ebi-a-GCST90001445	rs2450220	IgD- CD38- %B cel	8	119323401
ebi-a-GCST90001445	rs140191306	IgD- CD38- %B cel	9	2570753
ebi-a-GCST90001445	rs138977782	IgD- CD38- %B cel	9	4744416
ebi-a-GCST90001445	rs11068290	IgD- CD38- %B cel	12	110228055
ebi-a-GCST90001445	rs7994585	IgD- CD38- %B cel	13	111083495
ebi-a-GCST90001445	rs7215243	IgD- CD38- %B cel	17	78261805
ebi-a-GCST90001446	rs77146037	IgD- CD38- AC	1	178195174
ebi-a-GCST90001446	rs61790670	IgD- CD38- AC	1	84127219
ebi-a-GCST90001446	rs114551034	IgD- CD38- AC	2	66709035
ebi-a-GCST90001446	rs115218350	IgD- CD38- AC	3	104146883
ebi-a-GCST90001446	rs112964950	IgD- CD38- AC	3	73697496
ebi-a-GCST90001446	rs75524570	IgD- CD38- AC	3	191905058
ebi-a-GCST90001446	rs56741207	IgD- CD38- AC	4	134733663
ebi-a-GCST90001446	rs4697617	IgD- CD38- AC	4	25771115
ebi-a-GCST90001446	rs144625305	IgD- CD38- AC	7	7871676
ebi-a-GCST90001446	rs62521107	IgD- CD38- AC	8	118851954
ebi-a-GCST90001446	rs118121266	IgD- CD38- AC	10	20380753
ebi-a-GCST90001446	rs79416092	IgD- CD38- AC	12	6775503
ebi-a-GCST90001446	rs421516	IgD- CD38- AC	17	63135589
ebi-a-GCST90001446	rs77741094	IgD- CD38- AC	21	42362418
ebi-a-GCST90001446	rs144295598	IgD- CD38- AC	22	45164171
ebi-a-GCST90001447	rs74907325	IgD+ CD38br %B c	1	218429679
ebi-a-GCST90001447	rs76084861	IgD+ CD38br %B c	2	16114539
ebi-a-GCST90001447	rs16867231	IgD+ CD38br %B c	2	181050023
ebi-a-GCST90001447	rs140380950	IgD+ CD38br %B c	2	105587803
ebi-a-GCST90001447	rs76112278	IgD+ CD38br %B c	4	15915069
ebi-a-GCST90001447	rs28393044	IgD+ CD38br %B c	4	157437839
ebi-a-GCST90001447	rs78658073	IgD+ CD38br %B c	5	498773
ebi-a-GCST90001447	rs139996875	IgD+ CD38br %B c	5	78010341
ebi-a-GCST90001447	rs13196511	IgD+ CD38br %B c	6	44643196
ebi-a-GCST90001447	rs117772462	IgD+ CD38br %B c	7	48791017
ebi-a-GCST90001447	rs17739715	IgD+ CD38br %B c	8	9957457
ebi-a-GCST90001447	rs17771512	IgD+ CD38br %B c	8	120047880
ebi-a-GCST90001447	rs57998613	IgD+ CD38br %B c	9	93109339

ebi-a-GCST90001447	rs28491938	IgD+ CD38br %B c	9	41980474
ebi-a-GCST90001447	rs11002952	IgD+ CD38br %B c	10	81145177
ebi-a-GCST90001447	rs2362966	IgD+ CD38br %B c	10	125560687
ebi-a-GCST90001447	rs35060907	IgD+ CD38br %B c	10	132636158
ebi-a-GCST90001447	rs61880664	IgD+ CD38br %B c	11	15343532
ebi-a-GCST90001447	rs11068290	IgD+ CD38br %B c	12	110228055
ebi-a-GCST90001447	rs59879144	IgD+ CD38br %B c	12	75564449
ebi-a-GCST90001447	rs4903967	IgD+ CD38br %B c	14	81485149
ebi-a-GCST90001447	rs17278914	IgD+ CD38br %B c	14	44595691
ebi-a-GCST90001447	rs76840770	IgD+ CD38br %B c	14	54814017
ebi-a-GCST90001447	rs79487320	IgD+ CD38br %B c	14	43717096
ebi-a-GCST90001447	rs147226531	IgD+ CD38br %B c	16	54686774
ebi-a-GCST90001447	rs2216762	IgD+ CD38br %B c	16	84759446
ebi-a-GCST90001447	rs8059803	IgD+ CD38br %B c	16	81603001
ebi-a-GCST90001447	rs72927038	IgD+ CD38br %B c	18	51760939
ebi-a-GCST90001448	rs114693598	CD11c+ monocyte .	1	161500712
ebi-a-GCST90001448	rs2404849	CD11c+ monocyte .	1	224272792
ebi-a-GCST90001448	rs72724781	CD11c+ monocyte .	1	89327063
ebi-a-GCST90001448	rs3040901	CD11c+ monocyte .	2	79926958
ebi-a-GCST90001448	rs74610143	CD11c+ monocyte .	3	172744541
ebi-a-GCST90001448	rs13104041	CD11c+ monocyte .	4	173569449
ebi-a-GCST90001448	rs72835655	CD11c+ monocyte .	5	167349999
ebi-a-GCST90001448	rs2295270	CD11c+ monocyte .	6	47216562
ebi-a-GCST90001448	rs151194174	CD11c+ monocyte .	7	20995778
ebi-a-GCST90001448	rs2587473	CD11c+ monocyte .	10	72515785
ebi-a-GCST90001448	rs72830905	CD11c+ monocyte .	10	85138481
ebi-a-GCST90001448	rs34936922	CD11c+ monocyte .	12	115758242
ebi-a-GCST90001448	rs113800324	CD11c+ monocyte .	12	69766606
ebi-a-GCST90001448	rs9894233	CD11c+ monocyte .	17	45778747
ebi-a-GCST90001448	rs113451095	CD11c+ monocyte .	17	9975038
ebi-a-GCST90001448	rs73445777	CD11c+ monocyte .	18	46954097
ebi-a-GCST90001448	rs8175830	CD11c+ monocyte .	19	27851156
ebi-a-GCST90001448	rs150248455	CD11c+ monocyte .	21	46373171
ebi-a-GCST90001449	rs191730825	CD11c+ monocyte '	1	46613239
ebi-a-GCST90001449	rs115058278	CD11c+ monocyte '	3	101915678
ebi-a-GCST90001449	rs74610143	CD11c+ monocyte '	3	172744541
ebi-a-GCST90001449	rs11921245	CD11c+ monocyte '	3	65576520
ebi-a-GCST90001449	rs35237330	CD11c+ monocyte '	4	182768743
ebi-a-GCST90001449	rs13186506	CD11c+ monocyte '	5	59111915
ebi-a-GCST90001449	rs75256410	CD11c+ monocyte '	5	176914227
ebi-a-GCST90001449	rs13241407	CD11c+ monocyte '	7	153684820
ebi-a-GCST90001449	rs11766273	CD11c+ monocyte '	7	22775663
ebi-a-GCST90001449	rs10227905	CD11c+ monocyte '	7	122075261
ebi-a-GCST90001449	rs73073066	CD11c+ monocyte '	7	23486970
ebi-a-GCST90001449	rs17689291	CD11c+ monocyte '	8	94037230
ebi-a-GCST90001449	rs4922033	CD11c+ monocyte '	8	19326303
ebi-a-GCST90001449	rs703009	CD11c+ monocyte '	10	26889823
ebi-a-GCST90001449	rs11610005	CD11c+ monocyte '	12	27966210

ebi-a-GCST90001449	rs57566659	CD11c+ monocyte	12	42973243
ebi-a-GCST90001449	rs76968110	CD11c+ monocyte	14	31160823
ebi-a-GCST90001449	rs117909439	CD11c+ monocyte	14	29918210
ebi-a-GCST90001449	rs62002392	CD11c+ monocyte	15	37200279
ebi-a-GCST90001449	rs16952634	CD11c+ monocyte	16	53934556
ebi-a-GCST90001449	rs8065103	CD11c+ monocyte	17	45062476
ebi-a-GCST90001449	rs1381984	CD11c+ monocyte	20	52655304
ebi-a-GCST90001449	rs35149557	CD11c+ monocyte	20	5421681
ebi-a-GCST90001450	rs4656708	CD62L- monocyte	1	169701466
ebi-a-GCST90001450	rs4987369	CD62L- monocyte	1	169662742
ebi-a-GCST90001450	rs62167331	CD62L- monocyte	2	56524393
ebi-a-GCST90001450	rs13068785	CD62L- monocyte	3	74703663
ebi-a-GCST90001450	rs1907980	CD62L- monocyte	4	4859590
ebi-a-GCST90001450	rs11935230	CD62L- monocyte	4	136447876
ebi-a-GCST90001450	rs278949	CD62L- monocyte	4	40461712
ebi-a-GCST90001450	rs17082777	CD62L- monocyte	4	63361327
ebi-a-GCST90001450	rs11249570	CD62L- monocyte	5	177033794
ebi-a-GCST90001450	rs79930588	CD62L- monocyte	5	6308011
ebi-a-GCST90001450	rs528135834	CD62L- monocyte	7	41362245
ebi-a-GCST90001450	rs4484736	CD62L- monocyte	8	66059237
ebi-a-GCST90001450	rs530377	CD62L- monocyte	9	135337621
ebi-a-GCST90001450	rs143153359	CD62L- monocyte	9	76145588
ebi-a-GCST90001450	rs77287889	CD62L- monocyte	10	89954638
ebi-a-GCST90001450	rs116691483	CD62L- monocyte	11	111990120
ebi-a-GCST90001450	rs1800973	CD62L- monocyte	12	69744014
ebi-a-GCST90001450	rs4899265	CD62L- monocyte	14	69321561
ebi-a-GCST90001450	rs146793376	CD62L- monocyte	16	31775655
ebi-a-GCST90001450	rs435806	CD62L- monocyte	19	2993427
ebi-a-GCST90001450	rs149543954	CD62L- monocyte	20	15302488
ebi-a-GCST90001451	rs4987360	CD62L- monocyte	1	169664867
ebi-a-GCST90001451	rs4656708	CD62L- monocyte	1	169701466
ebi-a-GCST90001451	rs141287837	CD62L- monocyte	1	99082513
ebi-a-GCST90001451	rs11694457	CD62L- monocyte	2	356029
ebi-a-GCST90001451	rs9824107	CD62L- monocyte	3	174360449
ebi-a-GCST90001451	rs62236339	CD62L- monocyte	3	16446085
ebi-a-GCST90001451	rs9859349	CD62L- monocyte	3	31661754
ebi-a-GCST90001451	rs36141595	CD62L- monocyte	4	32848987
ebi-a-GCST90001451	rs1816932	CD62L- monocyte	4	56771414
ebi-a-GCST90001451	rs17624563	CD62L- monocyte	5	110456263
ebi-a-GCST90001451	rs11249570	CD62L- monocyte	5	177033794
ebi-a-GCST90001451	rs2399610	CD62L- monocyte	5	5737796
ebi-a-GCST90001451	rs76717104	CD62L- monocyte	7	17644035
ebi-a-GCST90001451	rs717824	CD62L- monocyte	7	14032498
ebi-a-GCST90001451	rs2124027	CD62L- monocyte	8	14941111
ebi-a-GCST90001451	rs530377	CD62L- monocyte	9	135337621
ebi-a-GCST90001451	rs117765367	CD62L- monocyte	11	2024995
ebi-a-GCST90001451	rs7139370	CD62L- monocyte	13	109976273
ebi-a-GCST90001451	rs117309559	CD62L- monocyte	14	31030304

ebi-a-GCST90001451	rs4899389	CD62L- monocyte (	14	71667863
ebi-a-GCST90001451	rs45487297	CD62L- monocyte (	14	30153119
ebi-a-GCST90001451	rs7150584	CD62L- monocyte (	14	58316355
ebi-a-GCST90001451	rs9807014	CD62L- monocyte (	17	76108637
ebi-a-GCST90001451	rs9944612	CD62L- monocyte (	18	60116421
ebi-a-GCST90001451	rs55859756	CD62L- monocyte (	22	37139371
ebi-a-GCST90001452	rs2404849	CD11c+ CD62L- m	1	224272792
ebi-a-GCST90001452	rs1407142	CD11c+ CD62L- m	1	86940820
ebi-a-GCST90001452	rs55971447	CD11c+ CD62L- m	1	161515326
ebi-a-GCST90001452	rs72660177	CD11c+ CD62L- m	1	34019363
ebi-a-GCST90001452	rs72724781	CD11c+ CD62L- m	1	89327063
ebi-a-GCST90001452	rs3040901	CD11c+ CD62L- m	2	79926958
ebi-a-GCST90001452	rs13104041	CD11c+ CD62L- m	4	173569449
ebi-a-GCST90001452	rs72835655	CD11c+ CD62L- m	5	167349999
ebi-a-GCST90001452	rs75220521	CD11c+ CD62L- m	6	81660130
ebi-a-GCST90001452	rs11772116	CD11c+ CD62L- m	7	23594922
ebi-a-GCST90001452	rs1268974	CD11c+ CD62L- m	10	80852378
ebi-a-GCST90001452	rs11213272	CD11c+ CD62L- m	11	109986288
ebi-a-GCST90001452	rs12277397	CD11c+ CD62L- m	11	134569102
ebi-a-GCST90001452	rs10891433	CD11c+ CD62L- m	11	112642209
ebi-a-GCST90001452	rs76524078	CD11c+ CD62L- m	12	26355696
ebi-a-GCST90001452	rs113800324	CD11c+ CD62L- m	12	69766606
ebi-a-GCST90001452	rs17474599	CD11c+ CD62L- m	13	100593548
ebi-a-GCST90001452	rs77694582	CD11c+ CD62L- m	14	102895904
ebi-a-GCST90001452	rs4899265	CD11c+ CD62L- m	14	69321561
ebi-a-GCST90001452	rs4359381	CD11c+ CD62L- m	15	61748527
ebi-a-GCST90001452	rs12708874	CD11c+ CD62L- m	16	66062093
ebi-a-GCST90001452	rs9894233	CD11c+ CD62L- m	17	45778747
ebi-a-GCST90001452	rs34142458	CD11c+ CD62L- m	17	2792530
ebi-a-GCST90001452	rs150248455	CD11c+ CD62L- m	21	46373171
ebi-a-GCST90001452	rs117545845	CD11c+ CD62L- m	22	25622885
ebi-a-GCST90001453	rs116249234	CD11c+ CD62L- m	1	56894640
ebi-a-GCST90001453	rs80087840	CD11c+ CD62L- m	1	17966505
ebi-a-GCST90001453	rs191730825	CD11c+ CD62L- m	1	46613239
ebi-a-GCST90001453	rs10924239	CD11c+ CD62L- m	1	245721841
ebi-a-GCST90001453	rs114693598	CD11c+ CD62L- m	1	161500712
ebi-a-GCST90001453	rs114857887	CD11c+ CD62L- m	2	47354461
ebi-a-GCST90001453	rs10804278	CD11c+ CD62L- m	2	220533049
ebi-a-GCST90001453	rs74610143	CD11c+ CD62L- m	3	172744541
ebi-a-GCST90001453	rs11921245	CD11c+ CD62L- m	3	65576520
ebi-a-GCST90001453	rs138567833	CD11c+ CD62L- m	4	182783833
ebi-a-GCST90001453	rs13241407	CD11c+ CD62L- m	7	153684820
ebi-a-GCST90001453	rs73073066	CD11c+ CD62L- m	7	23486970
ebi-a-GCST90001453	rs4922033	CD11c+ CD62L- m	8	19326303
ebi-a-GCST90001453	rs1469503	CD11c+ CD62L- m	11	112076693
ebi-a-GCST90001453	rs57566659	CD11c+ CD62L- m	12	42973243
ebi-a-GCST90001453	rs1800973	CD11c+ CD62L- m	12	69744014
ebi-a-GCST90001453	rs11610005	CD11c+ CD62L- m	12	27966210

ebi-a-GCST90001453	rs117909439	CD11c+ CD62L- m	14	29918210
ebi-a-GCST90001453	rs62002392	CD11c+ CD62L- m	15	37200279
ebi-a-GCST90001453	rs16952634	CD11c+ CD62L- m	16	53934556
ebi-a-GCST90001453	rs4468691	CD11c+ CD62L- m	17	45064678
ebi-a-GCST90001454	rs74805957	CD62L- HLA DR+	2	112941273
ebi-a-GCST90001454	rs115431394	CD62L- HLA DR+	3	1641354
ebi-a-GCST90001454	rs1320172	CD62L- HLA DR+	3	61569753
ebi-a-GCST90001454	rs111224423	CD62L- HLA DR+	4	1531162
ebi-a-GCST90001454	rs7437802	CD62L- HLA DR+	4	148180897
ebi-a-GCST90001454	rs3797612	CD62L- HLA DR+	5	178631699
ebi-a-GCST90001454	rs10948829	CD62L- HLA DR+	6	54476928
ebi-a-GCST90001454	rs10759491	CD62L- HLA DR+	9	113852341
ebi-a-GCST90001454	rs10980077	CD62L- HLA DR+	9	112600304
ebi-a-GCST90001454	rs10887149	CD62L- HLA DR+	10	124167004
ebi-a-GCST90001454	rs28680389	CD62L- HLA DR+	11	134566275
ebi-a-GCST90001454	rs938447	CD62L- HLA DR+	12	108746427
ebi-a-GCST90001454	rs148420500	CD62L- HLA DR+	13	76034172
ebi-a-GCST90001454	rs4899265	CD62L- HLA DR+	14	69321561
ebi-a-GCST90001454	rs139554006	CD62L- HLA DR+	14	53831558
ebi-a-GCST90001454	rs144348353	CD62L- HLA DR+	15	88341038
ebi-a-GCST90001454	rs7190107	CD62L- HLA DR+	16	50273295
ebi-a-GCST90001454	rs2240899	CD62L- HLA DR+	18	3595011
ebi-a-GCST90001454	rs117545845	CD62L- HLA DR+	22	25622885
ebi-a-GCST90001455	rs76234991	CD62L- HLA DR+	1	89120861
ebi-a-GCST90001455	rs9919190	CD62L- HLA DR+	1	165676784
ebi-a-GCST90001455	rs4668750	CD62L- HLA DR+	2	12351867
ebi-a-GCST90001455	rs6430066	CD62L- HLA DR+	2	145504787
ebi-a-GCST90001455	rs356651	CD62L- HLA DR+	2	101541798
ebi-a-GCST90001455	rs2955643	CD62L- HLA DR+	3	9386094
ebi-a-GCST90001455	rs12485444	CD62L- HLA DR+	3	188135783
ebi-a-GCST90001455	rs151240896	CD62L- HLA DR+	3	9228763
ebi-a-GCST90001455	rs189575603	CD62L- HLA DR+	4	83340880
ebi-a-GCST90001455	rs111224423	CD62L- HLA DR+	4	1531162
ebi-a-GCST90001455	rs11766800	CD62L- HLA DR+	7	50435617
ebi-a-GCST90001455	rs1969797	CD62L- HLA DR+	9	85828008
ebi-a-GCST90001455	rs78736203	CD62L- HLA DR+	10	18422972
ebi-a-GCST90001455	rs35079623	CD62L- HLA DR+	14	31868358
ebi-a-GCST90001455	rs765044	CD62L- HLA DR+	19	2560423
ebi-a-GCST90001455	rs5761319	CD62L- HLA DR+	22	26322953
ebi-a-GCST90001456	rs506388	CD11c+ HLA DR+	1	229571061
ebi-a-GCST90001456	rs561934182	CD11c+ HLA DR+	1	103631826
ebi-a-GCST90001456	rs56186608	CD11c+ HLA DR+	1	45056124
ebi-a-GCST90001456	rs72660177	CD11c+ HLA DR+	1	34019363
ebi-a-GCST90001456	rs2404849	CD11c+ HLA DR+	1	224272792
ebi-a-GCST90001456	rs116484930	CD11c+ HLA DR+	1	76985141
ebi-a-GCST90001456	rs6808893	CD11c+ HLA DR+	3	188133439
ebi-a-GCST90001456	rs548306594	CD11c+ HLA DR+	3	87446622
ebi-a-GCST90001456	rs74610143	CD11c+ HLA DR+	3	172744541

ebi-a-GCST90001456	rs55651060	CD11c+ HLA DR+	6	67292748
ebi-a-GCST90001456	rs2295270	CD11c+ HLA DR+	6	47216562
ebi-a-GCST90001456	rs151194174	CD11c+ HLA DR+	7	20995778
ebi-a-GCST90001456	rs2587473	CD11c+ HLA DR+	10	72515785
ebi-a-GCST90001456	rs12277397	CD11c+ HLA DR+	11	134569102
ebi-a-GCST90001456	rs112353331	CD11c+ HLA DR+	13	100594821
ebi-a-GCST90001456	rs148420500	CD11c+ HLA DR+	13	76034172
ebi-a-GCST90001456	rs9894233	CD11c+ HLA DR+	17	45778747
ebi-a-GCST90001456	rs76376209	CD11c+ HLA DR+	17	35215045
ebi-a-GCST90001456	rs117545845	CD11c+ HLA DR+	22	25622885
ebi-a-GCST90001457	rs77361483	CD11c+ HLA DR+	1	56892198
ebi-a-GCST90001457	rs191730825	CD11c+ HLA DR+	1	46613239
ebi-a-GCST90001457	rs6808893	CD11c+ HLA DR+	3	188133439
ebi-a-GCST90001457	rs74610143	CD11c+ HLA DR+	3	172744541
ebi-a-GCST90001457	rs76838015	CD11c+ HLA DR+	4	141946812
ebi-a-GCST90001457	rs35237330	CD11c+ HLA DR+	4	182768743
ebi-a-GCST90001457	rs6942832	CD11c+ HLA DR+	7	24465888
ebi-a-GCST90001457	rs78175301	CD11c+ HLA DR+	7	23629053
ebi-a-GCST90001457	rs11785252	CD11c+ HLA DR+	8	117400280
ebi-a-GCST90001457	rs2587473	CD11c+ HLA DR+	10	72515785
ebi-a-GCST90001457	rs1469503	CD11c+ HLA DR+	11	112076693
ebi-a-GCST90001457	rs117853201	CD11c+ HLA DR+	11	80035628
ebi-a-GCST90001457	rs7101558	CD11c+ HLA DR+	11	115387449
ebi-a-GCST90001457	rs76968110	CD11c+ HLA DR+	14	31160823
ebi-a-GCST90001457	rs9897015	CD11c+ HLA DR+	17	45202758
ebi-a-GCST90001457	rs76376209	CD11c+ HLA DR+	17	35215045
ebi-a-GCST90001457	rs6038180	CD11c+ HLA DR+	20	5465449
ebi-a-GCST90001457	rs143643742	CD11c+ HLA DR+	20	45975692
ebi-a-GCST90001458	rs141320646	Myeloid DC AC	1	73622958
ebi-a-GCST90001458	rs115805162	Myeloid DC AC	1	161410253
ebi-a-GCST90001458	rs71632979	Myeloid DC AC	1	161526397
ebi-a-GCST90001458	rs147678401	Myeloid DC AC	1	161035780
ebi-a-GCST90001458	rs569911	Myeloid DC AC	1	160798209
ebi-a-GCST90001458	rs142472859	Myeloid DC AC	2	40026310
ebi-a-GCST90001458	rs17017403	Myeloid DC AC	2	112435483
ebi-a-GCST90001458	rs72794768	Myeloid DC AC	2	40472950
ebi-a-GCST90001458	rs115389169	Myeloid DC AC	3	1981195
ebi-a-GCST90001458	rs4681269	Myeloid DC AC	3	145530567
ebi-a-GCST90001458	rs17036497	Myeloid DC AC	4	106927356
ebi-a-GCST90001458	rs79275634	Myeloid DC AC	5	112543958
ebi-a-GCST90001458	rs76168011	Myeloid DC AC	7	4372544
ebi-a-GCST90001458	rs62504404	Myeloid DC AC	8	38806068
ebi-a-GCST90001458	rs16938204	Myeloid DC AC	8	73348626
ebi-a-GCST90001458	rs138949944	Myeloid DC AC	9	21870164
ebi-a-GCST90001458	rs10746769	Myeloid DC AC	9	88374754
ebi-a-GCST90001458	rs10763776	Myeloid DC AC	10	30413417
ebi-a-GCST90001458	rs17711429	Myeloid DC AC	11	34866130
ebi-a-GCST90001458	rs206131	Myeloid DC AC	13	32875763

ebi-a-GCST90001458	rs1664690	Myeloid DC AC	19	4079922
ebi-a-GCST90001458	rs147711004	Myeloid DC AC	19	45337918
ebi-a-GCST90001458	rs6048352	Myeloid DC AC	20	22780354
ebi-a-GCST90001458	rs147431504	Myeloid DC AC	20	2470273
ebi-a-GCST90001459	rs143140559	Myeloid DC %DC	1	160972255
ebi-a-GCST90001459	rs71639910	Myeloid DC %DC	1	161937774
ebi-a-GCST90001459	rs71632979	Myeloid DC %DC	1	161526397
ebi-a-GCST90001459	rs74341264	Myeloid DC %DC	1	161561151
ebi-a-GCST90001459	rs66730637	Myeloid DC %DC	2	65193712
ebi-a-GCST90001459	rs142472859	Myeloid DC %DC	2	40026310
ebi-a-GCST90001459	rs7577824	Myeloid DC %DC	2	111876763
ebi-a-GCST90001459	rs141267173	Myeloid DC %DC	2	211051993
ebi-a-GCST90001459	rs11715127	Myeloid DC %DC	3	384169
ebi-a-GCST90001459	rs62272723	Myeloid DC %DC	3	151963160
ebi-a-GCST90001459	rs114586806	Myeloid DC %DC	6	36323614
ebi-a-GCST90001459	rs196051	Myeloid DC %DC	6	22056131
ebi-a-GCST90001459	rs78726632	Myeloid DC %DC	7	14195723
ebi-a-GCST90001459	rs876036	Myeloid DC %DC	7	50307710
ebi-a-GCST90001459	rs4921823	Myeloid DC %DC	8	17810983
ebi-a-GCST90001459	rs34547718	Myeloid DC %DC	8	130647985
ebi-a-GCST90001459	rs17767335	Myeloid DC %DC	8	71747316
ebi-a-GCST90001459	rs1571857	Myeloid DC %DC	9	135295345
ebi-a-GCST90001459	rs145330472	Myeloid DC %DC	9	31374800
ebi-a-GCST90001459	rs1330145	Myeloid DC %DC	9	110467582
ebi-a-GCST90001459	rs4752481	Myeloid DC %DC	10	122700529
ebi-a-GCST90001459	rs10735429	Myeloid DC %DC	12	108726677
ebi-a-GCST90001459	rs73180904	Myeloid DC %DC	13	44765322
ebi-a-GCST90001459	rs79691782	Myeloid DC %DC	16	111247
ebi-a-GCST90001459	rs79631949	Myeloid DC %DC	17	12078483
ebi-a-GCST90001459	rs73064521	Myeloid DC %DC	19	58148090
ebi-a-GCST90001459	rs6079813	Myeloid DC %DC	20	15407093
ebi-a-GCST90001459	rs12628370	Myeloid DC %DC	22	27665142
ebi-a-GCST90001460	rs6751072	Plasmacytoid DC A	2	1970675
ebi-a-GCST90001460	rs6708784	Plasmacytoid DC A	2	111927379
ebi-a-GCST90001460	rs12616455	Plasmacytoid DC A	2	85696551
ebi-a-GCST90001460	rs7560153	Plasmacytoid DC A	2	221584311
ebi-a-GCST90001460	rs115638174	Plasmacytoid DC A	2	178694935
ebi-a-GCST90001460	rs2164210	Plasmacytoid DC A	2	136580287
ebi-a-GCST90001460	rs192992420	Plasmacytoid DC A	3	19131454
ebi-a-GCST90001460	rs77908862	Plasmacytoid DC A	3	104696995
ebi-a-GCST90001460	rs2545798	Plasmacytoid DC A	5	176855627
ebi-a-GCST90001460	rs62354582	Plasmacytoid DC A	5	36514924
ebi-a-GCST90001460	rs144152085	Plasmacytoid DC A	6	67895544
ebi-a-GCST90001460	rs73064418	Plasmacytoid DC A	7	21574985
ebi-a-GCST90001460	rs186765793	Plasmacytoid DC A	7	11535712
ebi-a-GCST90001460	rs876039	Plasmacytoid DC A	7	50308811
ebi-a-GCST90001460	rs117039412	Plasmacytoid DC A	8	34472241
ebi-a-GCST90001460	rs11776881	Plasmacytoid DC A	8	131805603

ebi-a-GCST90001460	rs2862050	Plasmacytoid DC A	10	99691593
ebi-a-GCST90001460	rs17108993	Plasmacytoid DC A	10	95364033
ebi-a-GCST90001460	rs55901425	Plasmacytoid DC A	11	80180769
ebi-a-GCST90001460	rs117839924	Plasmacytoid DC A	12	101344076
ebi-a-GCST90001460	rs117112573	Plasmacytoid DC A	12	53745426
ebi-a-GCST90001460	rs17582557	Plasmacytoid DC A	13	101828654
ebi-a-GCST90001460	rs76924856	Plasmacytoid DC A	14	100464091
ebi-a-GCST90001460	rs217667	Plasmacytoid DC A	14	62358847
ebi-a-GCST90001460	rs138423727	Plasmacytoid DC A	15	43005653
ebi-a-GCST90001460	rs142129101	Plasmacytoid DC A	16	3995624
ebi-a-GCST90001460	rs147983983	Plasmacytoid DC A	16	83921634
ebi-a-GCST90001460	rs16977238	Plasmacytoid DC A	17	70427321
ebi-a-GCST90001460	rs16944211	Plasmacytoid DC A	17	10991039
ebi-a-GCST90001460	rs117990934	Plasmacytoid DC A	17	35059123
ebi-a-GCST90001460	rs10853636	Plasmacytoid DC A	18	51412543
ebi-a-GCST90001461	rs191217950	DC AC	1	160940671
ebi-a-GCST90001461	rs911228	DC AC	1	160145298
ebi-a-GCST90001461	rs55971447	DC AC	1	161515326
ebi-a-GCST90001461	rs75072970	DC AC	1	161058351
ebi-a-GCST90001461	rs12144062	DC AC	1	120123990
ebi-a-GCST90001461	rs17438107	DC AC	1	162920561
ebi-a-GCST90001461	rs2322660	DC AC	2	136557319
ebi-a-GCST90001461	rs17017403	DC AC	2	112435483
ebi-a-GCST90001461	rs7355664	DC AC	2	47496463
ebi-a-GCST90001461	rs6782857	DC AC	3	31185662
ebi-a-GCST90001461	rs115751481	DC AC	3	41531792
ebi-a-GCST90001461	rs11748731	DC AC	5	30878060
ebi-a-GCST90001461	rs79275634	DC AC	5	112543958
ebi-a-GCST90001461	rs2490034	DC AC	6	3870889
ebi-a-GCST90001461	rs76168011	DC AC	7	4372544
ebi-a-GCST90001461	rs35802809	DC AC	7	46017435
ebi-a-GCST90001461	rs28854187	DC AC	8	73320315
ebi-a-GCST90001461	rs62504404	DC AC	8	38806068
ebi-a-GCST90001461	rs138192280	DC AC	9	88411604
ebi-a-GCST90001461	rs142917473	DC AC	12	133840072
ebi-a-GCST90001461	rs9590896	DC AC	13	32877696
ebi-a-GCST90001461	rs115102594	DC AC	17	35251042
ebi-a-GCST90001461	rs147431504	DC AC	20	2470273
ebi-a-GCST90001462	rs74341264	CD62L- DC AC	1	161561151
ebi-a-GCST90001462	rs143140559	CD62L- DC AC	1	160972255
ebi-a-GCST90001462	rs71632979	CD62L- DC AC	1	161526397
ebi-a-GCST90001462	rs147678401	CD62L- DC AC	1	161035780
ebi-a-GCST90001462	rs71639910	CD62L- DC AC	1	161937774
ebi-a-GCST90001462	rs17438107	CD62L- DC AC	1	162920561
ebi-a-GCST90001462	rs75055130	CD62L- DC AC	2	155187137
ebi-a-GCST90001462	rs9855547	CD62L- DC AC	3	163257483
ebi-a-GCST90001462	rs35740507	CD62L- DC AC	4	90807488
ebi-a-GCST90001462	rs139894456	CD62L- DC AC	8	141396189

ebi-a-GCST90001462	rs771894	CD62L- DC AC	9	1642516
ebi-a-GCST90001462	rs116910026	CD62L- DC AC	11	19877912
ebi-a-GCST90001462	rs149812305	CD62L- DC AC	11	5480718
ebi-a-GCST90001462	rs73231298	CD62L- DC AC	12	127036033
ebi-a-GCST90001462	rs35519924	CD62L- DC AC	15	81119490
ebi-a-GCST90001462	rs151030554	CD62L- DC AC	15	57324322
ebi-a-GCST90001462	rs139058442	CD62L- DC AC	16	74973365
ebi-a-GCST90001462	rs112701148	CD62L- DC AC	18	10175131
ebi-a-GCST90001462	rs6048352	CD62L- DC AC	20	22780354
ebi-a-GCST90001463	rs1515318	CD62L- DC %DC	1	105108278
ebi-a-GCST90001463	rs143140559	CD62L- DC %DC	1	160972255
ebi-a-GCST90001463	rs74341264	CD62L- DC %DC	1	161561151
ebi-a-GCST90001463	rs143032729	CD62L- DC %DC	1	160353789
ebi-a-GCST90001463	rs6537835	CD62L- DC %DC	1	115108914
ebi-a-GCST90001463	rs79979402	CD62L- DC %DC	1	62527805
ebi-a-GCST90001463	rs341029	CD62L- DC %DC	1	77072414
ebi-a-GCST90001463	rs56199187	CD62L- DC %DC	1	161520931
ebi-a-GCST90001463	rs4680245	CD62L- DC %DC	3	155938532
ebi-a-GCST90001463	rs112985320	CD62L- DC %DC	3	67260176
ebi-a-GCST90001463	rs62300075	CD62L- DC %DC	4	43118540
ebi-a-GCST90001463	rs116473196	CD62L- DC %DC	5	179543345
ebi-a-GCST90001463	rs75220521	CD62L- DC %DC	6	81660130
ebi-a-GCST90001463	rs11754319	CD62L- DC %DC	6	8259337
ebi-a-GCST90001463	rs78548019	CD62L- DC %DC	6	148373443
ebi-a-GCST90001463	rs702185	CD62L- DC %DC	9	1621830
ebi-a-GCST90001463	rs138315340	CD62L- DC %DC	11	19889219
ebi-a-GCST90001463	rs41275192	CD62L- DC %DC	11	6479487
ebi-a-GCST90001463	rs149812305	CD62L- DC %DC	11	5480718
ebi-a-GCST90001463	rs2332920	CD62L- DC %DC	14	73286458
ebi-a-GCST90001463	rs7163051	CD62L- DC %DC	15	68361528
ebi-a-GCST90001463	rs148188224	CD62L- DC %DC	16	65149970
ebi-a-GCST90001463	rs2024391	CD62L- DC %DC	16	11907102
ebi-a-GCST90001463	rs5767014	CD62L- DC %DC	22	48474288
ebi-a-GCST90001464	rs72711599	CD86+ myeloid DC	1	162752670
ebi-a-GCST90001464	rs12134798	CD86+ myeloid DC	1	120115384
ebi-a-GCST90001464	rs147678401	CD86+ myeloid DC	1	161035780
ebi-a-GCST90001464	rs139939515	CD86+ myeloid DC	1	163958750
ebi-a-GCST90001464	rs71639910	CD86+ myeloid DC	1	161937774
ebi-a-GCST90001464	rs6656063	CD86+ myeloid DC	1	157964856
ebi-a-GCST90001464	rs71632979	CD86+ myeloid DC	1	161526397
ebi-a-GCST90001464	rs113995133	CD86+ myeloid DC	1	163485197
ebi-a-GCST90001464	rs3789359	CD86+ myeloid DC	1	181020181
ebi-a-GCST90001464	rs74341264	CD86+ myeloid DC	1	161561151
ebi-a-GCST90001464	rs143140559	CD86+ myeloid DC	1	160972255
ebi-a-GCST90001464	rs115849513	CD86+ myeloid DC	3	1827937
ebi-a-GCST90001464	rs112966518	CD86+ myeloid DC	4	76221611
ebi-a-GCST90001464	rs112186537	CD86+ myeloid DC	6	73397623
ebi-a-GCST90001464	rs923967	CD86+ myeloid DC	8	116153312

ebi-a-GCST90001464	rs16938204	CD86+ myeloid DC	8	73348626
ebi-a-GCST90001464	rs12005879	CD86+ myeloid DC	9	133737649
ebi-a-GCST90001464	rs9557311	CD86+ myeloid DC	13	100459317
ebi-a-GCST90001464	rs75700156	CD86+ myeloid DC	14	44042820
ebi-a-GCST90001464	rs185312213	CD86+ myeloid DC	14	51937110
ebi-a-GCST90001464	rs140205436	CD86+ myeloid DC	15	25969797
ebi-a-GCST90001464	rs79497887	CD86+ myeloid DC	16	57725145
ebi-a-GCST90001464	rs72846852	CD86+ myeloid DC	17	77253136
ebi-a-GCST90001465	rs143140559	CD86+ myeloid DC	1	160972255
ebi-a-GCST90001465	rs6656063	CD86+ myeloid DC	1	157964856
ebi-a-GCST90001465	rs71639910	CD86+ myeloid DC	1	161937774
ebi-a-GCST90001465	rs3789359	CD86+ myeloid DC	1	181020181
ebi-a-GCST90001465	rs113995133	CD86+ myeloid DC	1	163485197
ebi-a-GCST90001465	rs74341264	CD86+ myeloid DC	1	161561151
ebi-a-GCST90001465	rs139939515	CD86+ myeloid DC	1	163958750
ebi-a-GCST90001465	rs71632979	CD86+ myeloid DC	1	161526397
ebi-a-GCST90001465	rs950485	CD86+ myeloid DC	1	82817239
ebi-a-GCST90001465	rs114566711	CD86+ myeloid DC	2	191451636
ebi-a-GCST90001465	rs1996740	CD86+ myeloid DC	4	78748977
ebi-a-GCST90001465	rs143512471	CD86+ myeloid DC	5	63081773
ebi-a-GCST90001465	rs113404917	CD86+ myeloid DC	6	147461264
ebi-a-GCST90001465	rs78169719	CD86+ myeloid DC	7	157588588
ebi-a-GCST90001465	rs144259606	CD86+ myeloid DC	7	109678733
ebi-a-GCST90001465	rs77148097	CD86+ myeloid DC	7	98162546
ebi-a-GCST90001465	rs76522264	CD86+ myeloid DC	8	113032809
ebi-a-GCST90001465	rs117690664	CD86+ myeloid DC	8	107693714
ebi-a-GCST90001465	rs143555771	CD86+ myeloid DC	10	89944259
ebi-a-GCST90001465	rs75358870	CD86+ myeloid DC	14	30170464
ebi-a-GCST90001465	rs140205436	CD86+ myeloid DC	15	25969797
ebi-a-GCST90001465	rs72745440	CD86+ myeloid DC	15	67606602
ebi-a-GCST90001465	rs113345111	CD86+ myeloid DC	18	22455983
ebi-a-GCST90001465	rs77768825	CD86+ myeloid DC	19	30509788
ebi-a-GCST90001466	rs56164117	CD86+ plasmacytoi	1	45173351
ebi-a-GCST90001466	rs1991164	CD86+ plasmacytoi	2	1732855
ebi-a-GCST90001466	rs13088702	CD86+ plasmacytoi	3	121714019
ebi-a-GCST90001466	rs78379738	CD86+ plasmacytoi	5	148318809
ebi-a-GCST90001466	rs10248630	CD86+ plasmacytoi	7	67115438
ebi-a-GCST90001466	rs2725015	CD86+ plasmacytoi	8	4465101
ebi-a-GCST90001466	rs79116372	CD86+ plasmacytoi	8	36308379
ebi-a-GCST90001466	rs140090913	CD86+ plasmacytoi	8	18584959
ebi-a-GCST90001466	rs139557155	CD86+ plasmacytoi	9	77583476
ebi-a-GCST90001466	rs61852527	CD86+ plasmacytoi	10	90380589
ebi-a-GCST90001466	rs9513487	CD86+ plasmacytoi	13	99428046
ebi-a-GCST90001466	rs142245023	CD86+ plasmacytoi	14	69634234
ebi-a-GCST90001466	rs11852100	CD86+ plasmacytoi	14	36478046
ebi-a-GCST90001466	rs75070627	CD86+ plasmacytoi	15	96560797
ebi-a-GCST90001466	rs114468547	CD86+ plasmacytoi	16	78055434
ebi-a-GCST90001466	rs118148569	CD86+ plasmacytoi	16	13019834

ebi-a-GCST90001466	rs112496823	CD86+ plasmacytoi	17	33829583
ebi-a-GCST90001466	rs150206669	CD86+ plasmacytoi	18	66297295
ebi-a-GCST90001466	rs313778	CD86+ plasmacytoi	19	3313321
ebi-a-GCST90001466	rs11701450	CD86+ plasmacytoi	21	34628034
ebi-a-GCST90001467	rs3845548	CD86+ plasmacytoi	1	161507448
ebi-a-GCST90001467	rs513617	CD86+ plasmacytoi	1	34413398
ebi-a-GCST90001467	rs11811863	CD86+ plasmacytoi	1	247577856
ebi-a-GCST90001467	rs11166208	CD86+ plasmacytoi	1	99738054
ebi-a-GCST90001467	rs10170363	CD86+ plasmacytoi	2	15272805
ebi-a-GCST90001467	rs66967216	CD86+ plasmacytoi	2	190942111
ebi-a-GCST90001467	rs1915087	CD86+ plasmacytoi	3	121838791
ebi-a-GCST90001467	rs78379738	CD86+ plasmacytoi	5	148318809
ebi-a-GCST90001467	rs6930632	CD86+ plasmacytoi	6	155772981
ebi-a-GCST90001467	rs6463191	CD86+ plasmacytoi	7	43492179
ebi-a-GCST90001467	rs116872766	CD86+ plasmacytoi	8	77883134
ebi-a-GCST90001467	rs55966096	CD86+ plasmacytoi	8	4474949
ebi-a-GCST90001467	rs140090913	CD86+ plasmacytoi	8	18584959
ebi-a-GCST90001467	rs55732541	CD86+ plasmacytoi	9	8370207
ebi-a-GCST90001467	rs139557155	CD86+ plasmacytoi	9	77583476
ebi-a-GCST90001467	rs117919713	CD86+ plasmacytoi	9	77145787
ebi-a-GCST90001467	rs72810797	CD86+ plasmacytoi	10	30336672
ebi-a-GCST90001467	rs61852527	CD86+ plasmacytoi	10	90380589
ebi-a-GCST90001467	rs11231693	CD86+ plasmacytoi	11	63862612
ebi-a-GCST90001467	rs12311260	CD86+ plasmacytoi	12	18094072
ebi-a-GCST90001467	rs9513487	CD86+ plasmacytoi	13	99428046
ebi-a-GCST90001467	rs34692286	CD86+ plasmacytoi	13	28137152
ebi-a-GCST90001467	rs11852100	CD86+ plasmacytoi	14	36478046
ebi-a-GCST90001467	rs75070627	CD86+ plasmacytoi	15	96560797
ebi-a-GCST90001467	rs2955695	CD86+ plasmacytoi	15	35034619
ebi-a-GCST90001467	rs112496823	CD86+ plasmacytoi	17	33829583
ebi-a-GCST90001467	rs71381356	CD86+ plasmacytoi	17	49908577
ebi-a-GCST90001467	rs150206669	CD86+ plasmacytoi	18	66297295
ebi-a-GCST90001467	rs62131897	CD86+ plasmacytoi	19	10622478
ebi-a-GCST90001467	rs9607320	CD86+ plasmacytoi	22	22202164
ebi-a-GCST90001468	rs74341264	CD62L- myeloid D	1	161561151
ebi-a-GCST90001468	rs71632979	CD62L- myeloid D	1	161526397
ebi-a-GCST90001468	rs17438107	CD62L- myeloid D	1	162920561
ebi-a-GCST90001468	rs143140559	CD62L- myeloid D	1	160972255
ebi-a-GCST90001468	rs71639910	CD62L- myeloid D	1	161937774
ebi-a-GCST90001468	rs147678401	CD62L- myeloid D	1	161035780
ebi-a-GCST90001468	rs10779896	CD62L- myeloid D	2	4613050
ebi-a-GCST90001468	rs73172229	CD62L- myeloid D	3	179194521
ebi-a-GCST90001468	rs13148506	CD62L- myeloid D	4	43123482
ebi-a-GCST90001468	rs116910026	CD62L- myeloid D	11	19877912
ebi-a-GCST90001468	rs4997747	CD62L- myeloid D	12	127036550
ebi-a-GCST90001468	rs11830537	CD62L- myeloid D	12	48157703
ebi-a-GCST90001468	rs17071426	CD62L- myeloid D	13	48733325
ebi-a-GCST90001468	rs35519924	CD62L- myeloid D	15	81119490

ebi-a-GCST90001468	rs112701148	CD62L- myeloid D	18	10175131
ebi-a-GCST90001468	rs112501424	CD62L- myeloid D	19	3045627
ebi-a-GCST90001468	rs5767014	CD62L- myeloid D	22	48474288
ebi-a-GCST90001469	rs6537835	CD62L- myeloid D	1	115108914
ebi-a-GCST90001469	rs74341264	CD62L- myeloid D	1	161561151
ebi-a-GCST90001469	rs147678401	CD62L- myeloid D	1	161035780
ebi-a-GCST90001469	rs34558449	CD62L- myeloid D	1	192566347
ebi-a-GCST90001469	rs143140559	CD62L- myeloid D	1	160972255
ebi-a-GCST90001469	rs17438107	CD62L- myeloid D	1	162920561
ebi-a-GCST90001469	rs71632979	CD62L- myeloid D	1	161526397
ebi-a-GCST90001469	rs71639910	CD62L- myeloid D	1	161937774
ebi-a-GCST90001469	rs10779896	CD62L- myeloid D	2	4613050
ebi-a-GCST90001469	rs143586943	CD62L- myeloid D	3	55606125
ebi-a-GCST90001469	rs16864449	CD62L- myeloid D	3	189220585
ebi-a-GCST90001469	rs17637703	CD62L- myeloid D	3	45440721
ebi-a-GCST90001469	rs530300575	CD62L- myeloid D	4	3387723
ebi-a-GCST90001469	rs62300075	CD62L- myeloid D	4	43118540
ebi-a-GCST90001469	rs75220521	CD62L- myeloid D	6	81660130
ebi-a-GCST90001469	rs3012723	CD62L- myeloid D	9	134645623
ebi-a-GCST90001469	rs181905377	CD62L- myeloid D	9	138681023
ebi-a-GCST90001469	rs149812305	CD62L- myeloid D	11	5480718
ebi-a-GCST90001469	rs116910026	CD62L- myeloid D	11	19877912
ebi-a-GCST90001469	rs7300606	CD62L- myeloid D	12	108719667
ebi-a-GCST90001469	rs6492701	CD62L- myeloid D	13	95043651
ebi-a-GCST90001469	rs7148140	CD62L- myeloid D	14	88587741
ebi-a-GCST90001469	rs17780615	CD62L- myeloid D	14	73029609
ebi-a-GCST90001469	rs72743439	CD62L- myeloid D	15	67410088
ebi-a-GCST90001469	rs116473040	CD62L- myeloid D	17	80291652
ebi-a-GCST90001469	rs5767014	CD62L- myeloid D	22	48474288
ebi-a-GCST90001470	rs4462104	CD62L- plasmacyto	1	36975893
ebi-a-GCST90001470	rs12061996	CD62L- plasmacyto	1	167814385
ebi-a-GCST90001470	rs17224524	CD62L- plasmacyto	2	182346146
ebi-a-GCST90001470	rs116054627	CD62L- plasmacyto	2	51168498
ebi-a-GCST90001470	rs75472065	CD62L- plasmacyto	3	77076673
ebi-a-GCST90001470	rs4928176	CD62L- plasmacyto	3	98972835
ebi-a-GCST90001470	rs191779135	CD62L- plasmacyto	4	82502357
ebi-a-GCST90001470	rs6535445	CD62L- plasmacyto	4	84151427
ebi-a-GCST90001470	rs1472757	CD62L- plasmacyto	5	6455872
ebi-a-GCST90001470	rs1391986	CD62L- plasmacyto	5	52071311
ebi-a-GCST90001470	rs9502274	CD62L- plasmacyto	6	566060
ebi-a-GCST90001470	rs13201703	CD62L- plasmacyto	6	22921471
ebi-a-GCST90001470	rs9403901	CD62L- plasmacyto	6	148286133
ebi-a-GCST90001470	rs56374915	CD62L- plasmacyto	7	115086436
ebi-a-GCST90001470	rs72716416	CD62L- plasmacyto	8	131993230
ebi-a-GCST90001470	rs118054784	CD62L- plasmacyto	8	130658684
ebi-a-GCST90001470	rs116894787	CD62L- plasmacyto	10	12240106
ebi-a-GCST90001470	rs7114664	CD62L- plasmacyto	11	22793001
ebi-a-GCST90001470	rs61936377	CD62L- plasmacyto	12	63530291

ebi-a-GCST90001470	rs16957038	CD62L- plasmacyto	13	100542252
ebi-a-GCST90001470	rs170697	CD62L- plasmacyto	14	83856849
ebi-a-GCST90001470	rs9900969	CD62L- plasmacyto	17	76194489
ebi-a-GCST90001470	rs11659751	CD62L- plasmacyto	18	55736723
ebi-a-GCST90001470	rs150918748	CD62L- plasmacyto	22	43446390
ebi-a-GCST90001471	rs4462104	CD62L- plasmacyto	1	36975893
ebi-a-GCST90001471	rs55971447	CD62L- plasmacyto	1	161515326
ebi-a-GCST90001471	rs116054627	CD62L- plasmacyto	2	51168498
ebi-a-GCST90001471	rs148594241	CD62L- plasmacyto	2	236709624
ebi-a-GCST90001471	rs79976443	CD62L- plasmacyto	3	78714454
ebi-a-GCST90001471	rs75472065	CD62L- plasmacyto	3	77076673
ebi-a-GCST90001471	rs62297340	CD62L- plasmacyto	4	5407389
ebi-a-GCST90001471	rs79659729	CD62L- plasmacyto	4	84265416
ebi-a-GCST90001471	rs6535445	CD62L- plasmacyto	4	84151427
ebi-a-GCST90001471	rs191779135	CD62L- plasmacyto	4	82502357
ebi-a-GCST90001471	rs148863428	CD62L- plasmacyto	4	107554477
ebi-a-GCST90001471	rs1835019	CD62L- plasmacyto	5	66583829
ebi-a-GCST90001471	rs56374915	CD62L- plasmacyto	7	115086436
ebi-a-GCST90001471	rs62495198	CD62L- plasmacyto	8	13705385
ebi-a-GCST90001471	rs61859443	CD62L- plasmacyto	10	43792983
ebi-a-GCST90001471	rs146839224	CD62L- plasmacyto	10	131367364
ebi-a-GCST90001471	rs11000032	CD62L- plasmacyto	10	73619601
ebi-a-GCST90001471	rs182833362	CD62L- plasmacyto	16	27972325
ebi-a-GCST90001471	rs8044705	CD62L- plasmacyto	16	48914991
ebi-a-GCST90001471	rs3827963	CD62L- plasmacyto	20	20371744
ebi-a-GCST90001472	rs147678401	CD62L- CD86+ my	1	161035780
ebi-a-GCST90001472	rs74341264	CD62L- CD86+ my	1	161561151
ebi-a-GCST90001472	rs2788088	CD62L- CD86+ my	1	2897093
ebi-a-GCST90001472	rs71639910	CD62L- CD86+ my	1	161937774
ebi-a-GCST90001472	rs61808511	CD62L- CD86+ my	1	120116588
ebi-a-GCST90001472	rs113995133	CD62L- CD86+ my	1	163485197
ebi-a-GCST90001472	rs71632979	CD62L- CD86+ my	1	161526397
ebi-a-GCST90001472	rs6656063	CD62L- CD86+ my	1	157964856
ebi-a-GCST90001472	rs77107932	CD62L- CD86+ my	1	162042160
ebi-a-GCST90001472	rs143140559	CD62L- CD86+ my	1	160972255
ebi-a-GCST90001472	rs10779896	CD62L- CD86+ my	2	4613050
ebi-a-GCST90001472	rs146507670	CD62L- CD86+ my	3	172101731
ebi-a-GCST90001472	rs162588	CD62L- CD86+ my	3	364098
ebi-a-GCST90001472	rs144101090	CD62L- CD86+ my	4	60446347
ebi-a-GCST90001472	rs112186537	CD62L- CD86+ my	6	73397623
ebi-a-GCST90001472	rs117044176	CD62L- CD86+ my	7	11604044
ebi-a-GCST90001472	rs117690664	CD62L- CD86+ my	8	107693714
ebi-a-GCST90001472	rs62489014	CD62L- CD86+ my	8	1144980
ebi-a-GCST90001472	rs139956891	CD62L- CD86+ my	10	127556162
ebi-a-GCST90001472	rs11830537	CD62L- CD86+ my	12	48157703
ebi-a-GCST90001472	rs135299	CD62L- CD86+ my	22	48479731
ebi-a-GCST90001473	rs2788088	CD62L- CD86+ my	1	2897093
ebi-a-GCST90001473	rs147678401	CD62L- CD86+ my	1	161035780

ebi-a-GCST90001473	rs71639910	CD62L- CD86+ my	1	161937774
ebi-a-GCST90001473	rs113995133	CD62L- CD86+ my	1	163485197
ebi-a-GCST90001473	rs6537835	CD62L- CD86+ my	1	115108914
ebi-a-GCST90001473	rs79078281	CD62L- CD86+ my	1	147922051
ebi-a-GCST90001473	rs74341264	CD62L- CD86+ my	1	161561151
ebi-a-GCST90001473	rs71632979	CD62L- CD86+ my	1	161526397
ebi-a-GCST90001473	rs77107932	CD62L- CD86+ my	1	162042160
ebi-a-GCST90001473	rs143140559	CD62L- CD86+ my	1	160972255
ebi-a-GCST90001473	rs10779896	CD62L- CD86+ my	2	4613050
ebi-a-GCST90001473	rs162588	CD62L- CD86+ my	3	364098
ebi-a-GCST90001473	rs117690664	CD62L- CD86+ my	8	107693714
ebi-a-GCST90001473	rs144030104	CD62L- CD86+ my	8	40841454
ebi-a-GCST90001473	rs72659736	CD62L- CD86+ my	8	60780923
ebi-a-GCST90001473	rs139956891	CD62L- CD86+ my	10	127556162
ebi-a-GCST90001473	rs4761369	CD62L- CD86+ my	12	77743499
ebi-a-GCST90001473	rs113704374	CD62L- CD86+ my	12	106818887
ebi-a-GCST90001473	rs500809	CD62L- CD86+ my	20	3013136
ebi-a-GCST90001473	rs5767014	CD62L- CD86+ my	22	48474288
ebi-a-GCST90001474	rs10910893	Plasmacytoid DC %	1	181160955
ebi-a-GCST90001474	rs143032729	Plasmacytoid DC %	1	160353789
ebi-a-GCST90001474	rs74341264	Plasmacytoid DC %	1	161561151
ebi-a-GCST90001474	rs56199187	Plasmacytoid DC %	1	161520931
ebi-a-GCST90001474	rs7593638	Plasmacytoid DC %	2	111876941
ebi-a-GCST90001474	rs2164210	Plasmacytoid DC %	2	136580287
ebi-a-GCST90001474	rs62234997	Plasmacytoid DC %	3	20754619
ebi-a-GCST90001474	rs62272723	Plasmacytoid DC %	3	151963160
ebi-a-GCST90001474	rs11715127	Plasmacytoid DC %	3	384169
ebi-a-GCST90001474	rs7566	Plasmacytoid DC %	5	179156449
ebi-a-GCST90001474	rs196051	Plasmacytoid DC %	6	22056131
ebi-a-GCST90001474	rs876036	Plasmacytoid DC %	7	50307710
ebi-a-GCST90001474	rs78726632	Plasmacytoid DC %	7	14195723
ebi-a-GCST90001474	rs4398989	Plasmacytoid DC %	9	110238601
ebi-a-GCST90001474	rs10759202	Plasmacytoid DC %	9	99462625
ebi-a-GCST90001474	rs4752481	Plasmacytoid DC %	10	122700529
ebi-a-GCST90001474	rs79053113	Plasmacytoid DC %	10	133341271
ebi-a-GCST90001474	rs607867	Plasmacytoid DC %	11	107542351
ebi-a-GCST90001474	rs75812106	Plasmacytoid DC %	11	108509865
ebi-a-GCST90001474	rs28595889	Plasmacytoid DC %	12	61698548
ebi-a-GCST90001474	rs10735429	Plasmacytoid DC %	12	108726677
ebi-a-GCST90001474	rs73180904	Plasmacytoid DC %	13	44765322
ebi-a-GCST90001474	rs690924	Plasmacytoid DC %	15	50067248
ebi-a-GCST90001474	rs12951038	Plasmacytoid DC %	17	77544550
ebi-a-GCST90001474	rs203393	Plasmacytoid DC %	20	56242563
ebi-a-GCST90001475	rs76950373	HLA DR++ monoc	1	70942862
ebi-a-GCST90001475	rs9439731	HLA DR++ monoc	1	19059471
ebi-a-GCST90001475	rs7538058	HLA DR++ monoc	1	166381821
ebi-a-GCST90001475	rs116670542	HLA DR++ monoc	2	217877436
ebi-a-GCST90001475	rs187260865	HLA DR++ monoc	2	106940570

ebi-a-GCST90001475	rs17023874	HLA DR++ monoc	2	39734023
ebi-a-GCST90001475	rs7624992	HLA DR++ monoc	3	3233271
ebi-a-GCST90001475	rs2878953	HLA DR++ monoc	3	180110875
ebi-a-GCST90001475	rs62395272	HLA DR++ monoc	6	31394424
ebi-a-GCST90001475	rs9271281	HLA DR++ monoc	6	32581059
ebi-a-GCST90001475	rs9271768	HLA DR++ monoc	6	32594188
ebi-a-GCST90001475	rs9296042	HLA DR++ monoc	6	32736005
ebi-a-GCST90001475	rs9469094	HLA DR++ monoc	6	32223191
ebi-a-GCST90001475	rs116322630	HLA DR++ monoc	6	15221128
ebi-a-GCST90001475	rs2090888	HLA DR++ monoc	6	109646216
ebi-a-GCST90001475	rs2113347	HLA DR++ monoc	8	3175873
ebi-a-GCST90001475	rs61835728	HLA DR++ monoc	10	10550265
ebi-a-GCST90001475	rs150066324	HLA DR++ monoc	12	51947803
ebi-a-GCST90001475	rs7953257	HLA DR++ monoc	12	112684221
ebi-a-GCST90001475	rs12436392	HLA DR++ monoc	14	81293101
ebi-a-GCST90001475	rs62083470	HLA DR++ monoc	18	35262843
ebi-a-GCST90001475	rs10410907	HLA DR++ monoc	19	41033109
ebi-a-GCST90001475	rs6010756	HLA DR++ monoc	20	61265397
ebi-a-GCST90001476	rs9439732	HLA DR++ monoc	1	19059473
ebi-a-GCST90001476	rs35478452	HLA DR++ monoc	1	248035843
ebi-a-GCST90001476	rs115589615	HLA DR++ monoc	2	106954936
ebi-a-GCST90001476	rs9271768	HLA DR++ monoc	6	32594188
ebi-a-GCST90001476	rs62395272	HLA DR++ monoc	6	31394424
ebi-a-GCST90001476	rs9270591	HLA DR++ monoc	6	32561424
ebi-a-GCST90001476	rs9296042	HLA DR++ monoc	6	32736005
ebi-a-GCST90001476	rs76115039	HLA DR++ monoc	21	34394714
ebi-a-GCST90001476	rs9615878	HLA DR++ monoc	22	48806386
ebi-a-GCST90001477	rs12410580	HLA DR++ monoc	1	160126020
ebi-a-GCST90001477	rs10753675	HLA DR++ monoc	1	165314847
ebi-a-GCST90001477	rs115589615	HLA DR++ monoc	2	106954936
ebi-a-GCST90001477	rs138955284	HLA DR++ monoc	3	154844527
ebi-a-GCST90001477	rs114141703	HLA DR++ monoc	3	197226213
ebi-a-GCST90001477	rs74827437	HLA DR++ monoc	4	109128418
ebi-a-GCST90001477	rs12659610	HLA DR++ monoc	5	65872759
ebi-a-GCST90001477	rs73576356	HLA DR++ monoc	6	147436169
ebi-a-GCST90001477	rs9272459	HLA DR++ monoc	6	32605578
ebi-a-GCST90001477	rs62395272	HLA DR++ monoc	6	31394424
ebi-a-GCST90001477	rs9275271	HLA DR++ monoc	6	32662533
ebi-a-GCST90001477	rs112700313	HLA DR++ monoc	6	127699589
ebi-a-GCST90001477	rs28610544	HLA DR++ monoc	7	106771703
ebi-a-GCST90001477	rs117433119	HLA DR++ monoc	7	45664941
ebi-a-GCST90001477	rs16901587	HLA DR++ monoc	8	127636944
ebi-a-GCST90001477	rs35842632	HLA DR++ monoc	9	25608163
ebi-a-GCST90001477	rs4244738	HLA DR++ monoc	9	66536402
ebi-a-GCST90001477	rs7310615	HLA DR++ monoc	12	111865049
ebi-a-GCST90001477	rs11169475	HLA DR++ monoc	12	50914563
ebi-a-GCST90001477	rs111801150	HLA DR++ monoc	13	114508277
ebi-a-GCST90001477	rs55952218	HLA DR++ monoc	16	73388512

ebi-a-GCST90001477	rs183181627	HLA DR++ monocy	16	69003214
ebi-a-GCST90001477	rs34795364	HLA DR++ monocy	17	34011303
ebi-a-GCST90001477	rs139181645	HLA DR++ monocy	17	76469857
ebi-a-GCST90001477	rs61289229	HLA DR++ monocy	19	46392004
ebi-a-GCST90001477	rs5995959	HLA DR++ monocy	22	22750190
ebi-a-GCST90001478	rs181741769	CD4 Treg %CD4	1	26999658
ebi-a-GCST90001478	rs13410035	CD4 Treg %CD4	2	161139489
ebi-a-GCST90001478	rs17009374	CD4 Treg %CD4	2	30532493
ebi-a-GCST90001478	rs4685760	CD4 Treg %CD4	3	4602747
ebi-a-GCST90001478	rs12186662	CD4 Treg %CD4	5	90356197
ebi-a-GCST90001478	rs77142596	CD4 Treg %CD4	6	163753664
ebi-a-GCST90001478	rs2187822	CD4 Treg %CD4	6	32449726
ebi-a-GCST90001478	rs191172032	CD4 Treg %CD4	7	148149143
ebi-a-GCST90001478	rs77615103	CD4 Treg %CD4	7	37406370
ebi-a-GCST90001478	rs138899232	CD4 Treg %CD4	8	10768753
ebi-a-GCST90001478	rs1571025	CD4 Treg %CD4	10	6134869
ebi-a-GCST90001478	rs6602392	CD4 Treg %CD4	10	6078079
ebi-a-GCST90001478	rs73018933	CD4 Treg %CD4	11	128174781
ebi-a-GCST90001478	rs77071786	CD4 Treg %CD4	12	23501010
ebi-a-GCST90001478	rs149595513	CD4 Treg %CD4	14	56535269
ebi-a-GCST90001478	rs146090095	CD4 Treg %CD4	14	25035095
ebi-a-GCST90001478	rs79028902	CD4 Treg %CD4	16	84427121
ebi-a-GCST90001478	rs72821912	CD4 Treg %CD4	17	30183388
ebi-a-GCST90001478	rs2909902	CD4 Treg %CD4	19	47667326
ebi-a-GCST90001478	rs6016476	CD4 Treg %CD4	20	39570656
ebi-a-GCST90001479	rs79327855	CD4 Treg %T cell	3	144158111
ebi-a-GCST90001479	rs76239041	CD4 Treg %T cell	5	153002618
ebi-a-GCST90001479	rs219948	CD4 Treg %T cell	6	12155899
ebi-a-GCST90001479	rs2975007	CD4 Treg %T cell	6	29902327
ebi-a-GCST90001479	rs971864	CD4 Treg %T cell	6	164597687
ebi-a-GCST90001479	rs115707165	CD4 Treg %T cell	7	81108132
ebi-a-GCST90001479	rs28445004	CD4 Treg %T cell	8	34560021
ebi-a-GCST90001479	rs6602392	CD4 Treg %T cell	10	6078079
ebi-a-GCST90001479	rs1571025	CD4 Treg %T cell	10	6134869
ebi-a-GCST90001479	rs509240	CD4 Treg %T cell	11	30568643
ebi-a-GCST90001479	rs61907712	CD4 Treg %T cell	11	128161142
ebi-a-GCST90001479	rs139191636	CD4 Treg %T cell	12	23591661
ebi-a-GCST90001479	rs146090095	CD4 Treg %T cell	14	25035095
ebi-a-GCST90001479	rs2526941	CD4 Treg %T cell	14	73155735
ebi-a-GCST90001479	rs11869223	CD4 Treg %T cell	17	33775713
ebi-a-GCST90001479	rs62055610	CD4 Treg %T cell	17	5640871
ebi-a-GCST90001479	rs56043453	CD4 Treg %T cell	17	80189590
ebi-a-GCST90001479	rs10212061	CD4 Treg %T cell	22	49579609
ebi-a-GCST90001480	rs12044365	Resting Treg AC	1	210431761
ebi-a-GCST90001480	rs116794175	Resting Treg AC	1	194010208
ebi-a-GCST90001480	rs17583875	Resting Treg AC	1	197924770
ebi-a-GCST90001480	rs6751481	Resting Treg AC	2	38897810
ebi-a-GCST90001480	rs79244630	Resting Treg AC	2	130435634

ebi-a-GCST90001480	rs17607399	Resting Treg AC	2	41884212
ebi-a-GCST90001480	rs408686	Resting Treg AC	2	39177320
ebi-a-GCST90001480	rs111714509	Resting Treg AC	3	124800996
ebi-a-GCST90001480	rs979640	Resting Treg AC	4	132525794
ebi-a-GCST90001480	rs247636	Resting Treg AC	5	142990496
ebi-a-GCST90001480	rs36058358	Resting Treg AC	6	32636282
ebi-a-GCST90001480	rs9272050	Resting Treg AC	6	32599071
ebi-a-GCST90001480	rs1618199	Resting Treg AC	6	31275850
ebi-a-GCST90001480	rs182630194	Resting Treg AC	7	71331239
ebi-a-GCST90001480	rs147199078	Resting Treg AC	7	80542211
ebi-a-GCST90001480	rs7787732	Resting Treg AC	7	151861663
ebi-a-GCST90001480	rs7899451	Resting Treg AC	10	118980344
ebi-a-GCST90001480	rs16922479	Resting Treg AC	10	22863004
ebi-a-GCST90001480	rs3016770	Resting Treg AC	11	128694040
ebi-a-GCST90001480	rs75958070	Resting Treg AC	11	79203049
ebi-a-GCST90001480	rs73060045	Resting Treg AC	12	12168131
ebi-a-GCST90001480	rs72702642	Resting Treg AC	14	99672909
ebi-a-GCST90001480	rs117432559	Resting Treg AC	15	40508902
ebi-a-GCST90001480	rs8088253	Resting Treg AC	18	64904859
ebi-a-GCST90001480	rs11703793	Resting Treg AC	22	27946507
ebi-a-GCST90001480	rs111941642	Resting Treg AC	22	28079761
ebi-a-GCST90001481	rs187089343	Resting Treg % CD	1	182091538
ebi-a-GCST90001481	rs78144227	Resting Treg % CD	1	203602440
ebi-a-GCST90001481	rs150956189	Resting Treg % CD	1	214625781
ebi-a-GCST90001481	rs61828007	Resting Treg % CD	1	203502968
ebi-a-GCST90001481	rs12133465	Resting Treg % CD	1	195430336
ebi-a-GCST90001481	rs146899923	Resting Treg % CD	1	195919849
ebi-a-GCST90001481	rs60220946	Resting Treg % CD	1	193505625
ebi-a-GCST90001481	rs147071297	Resting Treg % CD	1	191648600
ebi-a-GCST90001481	rs7530076	Resting Treg % CD	1	195825276
ebi-a-GCST90001481	rs17583875	Resting Treg % CD	1	197924770
ebi-a-GCST90001481	rs138915779	Resting Treg % CD	1	190079170
ebi-a-GCST90001481	rs147958143	Resting Treg % CD	1	238096255
ebi-a-GCST90001481	rs112553628	Resting Treg % CD	2	38077329
ebi-a-GCST90001481	rs413431	Resting Treg % CD	2	39176695
ebi-a-GCST90001481	rs139681117	Resting Treg % CD	2	23924920
ebi-a-GCST90001481	rs12993274	Resting Treg % CD	2	42378770
ebi-a-GCST90001481	rs76584473	Resting Treg % CD	2	73109350
ebi-a-GCST90001481	rs6751481	Resting Treg % CD	2	38897810
ebi-a-GCST90001481	rs17607399	Resting Treg % CD	2	41884212
ebi-a-GCST90001481	rs138960714	Resting Treg % CD	4	123422719
ebi-a-GCST90001481	rs10059100	Resting Treg % CD	5	120100784
ebi-a-GCST90001481	rs1634783	Resting Treg % CD	6	31283604
ebi-a-GCST90001481	rs9274712	Resting Treg % CD	6	32637298
ebi-a-GCST90001481	rs76109501	Resting Treg % CD	8	28323922
ebi-a-GCST90001481	rs273421	Resting Treg % CD	8	132542825
ebi-a-GCST90001481	rs12722496	Resting Treg % CD	10	6096667
ebi-a-GCST90001481	rs192123974	Resting Treg % CD	11	13923816

ebi-a-GCST90001481	rs138463974	Resting Treg % CD	12	132114547
ebi-a-GCST90001481	rs116850494	Resting Treg % CD	13	60389778
ebi-a-GCST90001481	rs73124650	Resting Treg % CD	20	47523429
ebi-a-GCST90001481	rs79670457	Resting Treg % CD	20	47437969
ebi-a-GCST90001482	rs147958143	Resting Treg %CD4	1	238096255
ebi-a-GCST90001482	rs7530076	Resting Treg %CD4	1	195825276
ebi-a-GCST90001482	rs146899923	Resting Treg %CD4	1	195919849
ebi-a-GCST90001482	rs12141656	Resting Treg %CD4	1	189081342
ebi-a-GCST90001482	rs147071297	Resting Treg %CD4	1	191648600
ebi-a-GCST90001482	rs150956189	Resting Treg %CD4	1	214625781
ebi-a-GCST90001482	rs12133465	Resting Treg %CD4	1	195430336
ebi-a-GCST90001482	rs17583875	Resting Treg %CD4	1	197924770
ebi-a-GCST90001482	rs28401504	Resting Treg %CD4	1	203497175
ebi-a-GCST90001482	rs138915779	Resting Treg %CD4	1	190079170
ebi-a-GCST90001482	rs115383271	Resting Treg %CD4	2	39367533
ebi-a-GCST90001482	rs17607399	Resting Treg %CD4	2	41884212
ebi-a-GCST90001482	rs6751481	Resting Treg %CD4	2	38897810
ebi-a-GCST90001482	rs408686	Resting Treg %CD4	2	39177320
ebi-a-GCST90001482	rs112553628	Resting Treg %CD4	2	38077329
ebi-a-GCST90001482	rs247636	Resting Treg %CD4	5	142990496
ebi-a-GCST90001482	rs1084331	Resting Treg %CD4	6	125213827
ebi-a-GCST90001482	rs4959016	Resting Treg %CD4	6	31297833
ebi-a-GCST90001482	rs62405562	Resting Treg %CD4	6	32451834
ebi-a-GCST90001482	rs56090633	Resting Treg %CD4	7	51393519
ebi-a-GCST90001482	rs17165751	Resting Treg %CD4	7	131301614
ebi-a-GCST90001482	rs73579441	Resting Treg %CD4	8	49024059
ebi-a-GCST90001482	rs192123974	Resting Treg %CD4	11	13923816
ebi-a-GCST90001482	rs79505492	Resting Treg %CD4	11	79167993
ebi-a-GCST90001482	rs3016770	Resting Treg %CD4	11	128694040
ebi-a-GCST90001482	rs145833948	Resting Treg %CD4	12	41804479
ebi-a-GCST90001482	rs76384362	Resting Treg %CD4	12	15365872
ebi-a-GCST90001482	rs148054039	Resting Treg %CD4	13	110095055
ebi-a-GCST90001482	rs147345848	Resting Treg %CD4	14	69803533
ebi-a-GCST90001482	rs72744142	Resting Treg %CD4	15	81518947
ebi-a-GCST90001482	rs113376235	Resting Treg %CD4	16	75514711
ebi-a-GCST90001482	rs117680001	Resting Treg %CD4	17	13652401
ebi-a-GCST90001482	rs188418491	Resting Treg %CD4	19	777304
ebi-a-GCST90001482	rs79524476	Resting Treg %CD4	19	40973561
ebi-a-GCST90001482	rs34441774	Resting Treg %CD4	22	49440757
ebi-a-GCST90001483	rs187089343	CD39+ resting Treg	1	182091538
ebi-a-GCST90001483	rs17583875	CD39+ resting Treg	1	197924770
ebi-a-GCST90001483	rs190023756	CD39+ resting Treg	1	199471227
ebi-a-GCST90001483	rs146899923	CD39+ resting Treg	1	195919849
ebi-a-GCST90001483	rs138915779	CD39+ resting Treg	1	190079170
ebi-a-GCST90001483	rs11890666	CD39+ resting Treg	2	38745822
ebi-a-GCST90001483	rs78096816	CD39+ resting Treg	2	53370856
ebi-a-GCST90001483	rs12712610	CD39+ resting Treg	2	38897249
ebi-a-GCST90001483	rs11718018	CD39+ resting Treg	3	60364770

ebi-a-GCST90001483	rs138807420	CD39+ resting Treg	5	6231929
ebi-a-GCST90001483	rs62421103	CD39+ resting Treg	6	113160344
ebi-a-GCST90001483	rs10086961	CD39+ resting Treg	8	132897157
ebi-a-GCST90001483	rs111534725	CD39+ resting Treg	10	98139264
ebi-a-GCST90001483	rs117678654	CD39+ resting Treg	10	101548054
ebi-a-GCST90001483	rs7079761	CD39+ resting Treg	10	97842115
ebi-a-GCST90001483	rs35909109	CD39+ resting Treg	10	98014850
ebi-a-GCST90001483	rs77520132	CD39+ resting Treg	10	98160835
ebi-a-GCST90001483	rs143132810	CD39+ resting Treg	10	96890004
ebi-a-GCST90001483	rs117572834	CD39+ resting Treg	10	109896541
ebi-a-GCST90001483	rs192831774	CD39+ resting Treg	10	95387682
ebi-a-GCST90001483	rs185474383	CD39+ resting Treg	10	96311553
ebi-a-GCST90001483	rs4918971	CD39+ resting Treg	10	97569998
ebi-a-GCST90001483	rs72809762	CD39+ resting Treg	10	97294154
ebi-a-GCST90001483	rs6488275	CD39+ resting Treg	12	10379180
ebi-a-GCST90001483	rs78843390	CD39+ resting Treg	12	130031612
ebi-a-GCST90001483	rs4321083	CD39+ resting Treg	13	84031916
ebi-a-GCST90001483	rs78654607	CD39+ resting Treg	16	83070118
ebi-a-GCST90001483	rs56158191	CD39+ resting Treg	19	57436943
ebi-a-GCST90001483	rs2326054	CD39+ resting Treg	20	3033175
ebi-a-GCST90001484	rs115328872	CD39+ resting Treg	1	197921246
ebi-a-GCST90001484	rs146899923	CD39+ resting Treg	1	195919849
ebi-a-GCST90001484	rs183612488	CD39+ resting Treg	2	97689175
ebi-a-GCST90001484	rs75834156	CD39+ resting Treg	2	38911130
ebi-a-GCST90001484	rs77718305	CD39+ resting Treg	2	85424907
ebi-a-GCST90001484	rs11676839	CD39+ resting Treg	2	197121290
ebi-a-GCST90001484	rs11718018	CD39+ resting Treg	3	60364770
ebi-a-GCST90001484	rs141700904	CD39+ resting Treg	6	57764436
ebi-a-GCST90001484	rs62416192	CD39+ resting Treg	6	93571691
ebi-a-GCST90001484	rs73196633	CD39+ resting Treg	7	86574111
ebi-a-GCST90001484	rs78809247	CD39+ resting Treg	8	4556480
ebi-a-GCST90001484	rs7077127	CD39+ resting Treg	10	18717348
ebi-a-GCST90001484	rs117572834	CD39+ resting Treg	10	109896541
ebi-a-GCST90001484	rs192831774	CD39+ resting Treg	10	95387682
ebi-a-GCST90001484	rs4917830	CD39+ resting Treg	10	100125216
ebi-a-GCST90001484	rs118112011	CD39+ resting Treg	10	97204949
ebi-a-GCST90001484	rs4918962	CD39+ resting Treg	10	97475574
ebi-a-GCST90001484	rs1923692	CD39+ resting Treg	10	98173715
ebi-a-GCST90001484	rs10882701	CD39+ resting Treg	10	97741071
ebi-a-GCST90001484	rs4293064	CD39+ resting Treg	10	107757380
ebi-a-GCST90001484	rs12778837	CD39+ resting Treg	10	96998744
ebi-a-GCST90001484	rs72809757	CD39+ resting Treg	10	97285715
ebi-a-GCST90001484	rs7963011	CD39+ resting Treg	12	10389074
ebi-a-GCST90001484	rs7328393	CD39+ resting Treg	13	44805761
ebi-a-GCST90001484	rs143895795	CD39+ resting Treg	15	94569845
ebi-a-GCST90001484	rs2127464	CD39+ resting Treg	15	94581009
ebi-a-GCST90001484	rs116988060	CD39+ resting Treg	18	34080354
ebi-a-GCST90001484	rs2326054	CD39+ resting Treg	20	3033175

ebi-a-GCST90001485	rs61821998	CD39+ resting Treg	1	193263354
ebi-a-GCST90001485	rs34645541	CD39+ resting Treg	1	195875605
ebi-a-GCST90001485	rs150956189	CD39+ resting Treg	1	214625781
ebi-a-GCST90001485	rs116794175	CD39+ resting Treg	1	194010208
ebi-a-GCST90001485	rs113435341	CD39+ resting Treg	1	198151688
ebi-a-GCST90001485	rs11579717	CD39+ resting Treg	1	199999121
ebi-a-GCST90001485	rs187089343	CD39+ resting Treg	1	182091538
ebi-a-GCST90001485	rs78096816	CD39+ resting Treg	2	53370856
ebi-a-GCST90001485	rs12712610	CD39+ resting Treg	2	38897249
ebi-a-GCST90001485	rs115977640	CD39+ resting Treg	2	160238519
ebi-a-GCST90001485	rs11890666	CD39+ resting Treg	2	38745822
ebi-a-GCST90001485	rs884599	CD39+ resting Treg	5	171129907
ebi-a-GCST90001485	rs12518588	CD39+ resting Treg	5	85325148
ebi-a-GCST90001485	rs113264169	CD39+ resting Treg	7	46328226
ebi-a-GCST90001485	rs357363	CD39+ resting Treg	7	137976565
ebi-a-GCST90001485	rs78809247	CD39+ resting Treg	8	4556480
ebi-a-GCST90001485	rs10086961	CD39+ resting Treg	8	132897157
ebi-a-GCST90001485	rs11818051	CD39+ resting Treg	10	97972294
ebi-a-GCST90001485	rs4918969	CD39+ resting Treg	10	97562938
ebi-a-GCST90001485	rs35909109	CD39+ resting Treg	10	98014850
ebi-a-GCST90001485	rs117572834	CD39+ resting Treg	10	109896541
ebi-a-GCST90001485	rs182702572	CD39+ resting Treg	10	96658384
ebi-a-GCST90001485	rs12258674	CD39+ resting Treg	10	68394054
ebi-a-GCST90001485	rs1410600	CD39+ resting Treg	10	97070694
ebi-a-GCST90001485	rs12778837	CD39+ resting Treg	10	96998744
ebi-a-GCST90001485	rs111534725	CD39+ resting Treg	10	98139264
ebi-a-GCST90001485	rs17111341	CD39+ resting Treg	10	97881031
ebi-a-GCST90001485	rs77520132	CD39+ resting Treg	10	98160835
ebi-a-GCST90001485	rs7963011	CD39+ resting Treg	12	10389074
ebi-a-GCST90001485	rs7310854	CD39+ resting Treg	12	52495599
ebi-a-GCST90001485	rs78843390	CD39+ resting Treg	12	130031612
ebi-a-GCST90001485	rs78654607	CD39+ resting Treg	16	83070118
ebi-a-GCST90001485	rs116988060	CD39+ resting Treg	18	34080354
ebi-a-GCST90001485	rs2326054	CD39+ resting Treg	20	3033175
ebi-a-GCST90001485	rs5755717	CD39+ resting Treg	22	35786713
ebi-a-GCST90001486	rs113435341	Activated Treg AC	1	198151688
ebi-a-GCST90001486	rs139138505	Activated Treg AC	1	225667295
ebi-a-GCST90001486	rs138915779	Activated Treg AC	1	190079170
ebi-a-GCST90001486	rs34596162	Activated Treg AC	2	195102952
ebi-a-GCST90001486	rs6901124	Activated Treg AC	6	109035704
ebi-a-GCST90001486	rs146746694	Activated Treg AC	6	155566192
ebi-a-GCST90001486	rs17509082	Activated Treg AC	7	120927706
ebi-a-GCST90001486	rs62489142	Activated Treg AC	7	135616195
ebi-a-GCST90001486	rs11593335	Activated Treg AC	10	6160702
ebi-a-GCST90001486	rs148991751	Activated Treg AC	12	53114409
ebi-a-GCST90001486	rs145600407	Activated Treg AC	13	19280601
ebi-a-GCST90001486	rs142987815	Activated Treg AC	15	98954485
ebi-a-GCST90001486	rs8064831	Activated Treg AC	17	10150766

ebi-a-GCST90001486	rs10515986	Activated Treg AC	18	60125602
ebi-a-GCST90001486	rs66939386	Activated Treg AC	19	45564915
ebi-a-GCST90001486	rs12626270	Activated Treg AC	21	34223997
ebi-a-GCST90001487	rs17583875	Activated Treg %C	1	197924770
ebi-a-GCST90001487	rs138915779	Activated Treg %C	1	190079170
ebi-a-GCST90001487	rs138751156	Activated Treg %C	1	77300547
ebi-a-GCST90001487	rs76151354	Activated Treg %C	1	104827341
ebi-a-GCST90001487	rs7530076	Activated Treg %C	1	195825276
ebi-a-GCST90001487	rs11185398	Activated Treg %C	1	104762898
ebi-a-GCST90001487	rs147071297	Activated Treg %C	1	191648600
ebi-a-GCST90001487	rs16845263	Activated Treg %C	2	161728621
ebi-a-GCST90001487	rs115862633	Activated Treg %C	2	229924038
ebi-a-GCST90001487	rs34185703	Activated Treg %C	3	94030311
ebi-a-GCST90001487	rs76204832	Activated Treg %C	3	71513336
ebi-a-GCST90001487	rs75839545	Activated Treg %C	3	174387584
ebi-a-GCST90001487	rs9878966	Activated Treg %C	3	14636829
ebi-a-GCST90001487	rs55990322	Activated Treg %C	3	124899665
ebi-a-GCST90001487	rs77506321	Activated Treg %C	4	121611469
ebi-a-GCST90001487	rs368899879	Activated Treg %C	4	29697653
ebi-a-GCST90001487	rs551105604	Activated Treg %C	5	136049136
ebi-a-GCST90001487	rs7784817	Activated Treg %C	7	23752731
ebi-a-GCST90001487	rs117779957	Activated Treg %C	8	19490663
ebi-a-GCST90001487	rs139502175	Activated Treg %C	8	131952722
ebi-a-GCST90001487	rs73697379	Activated Treg %C	8	106542599
ebi-a-GCST90001487	rs7035176	Activated Treg %C	9	119577258
ebi-a-GCST90001487	rs3793566	Activated Treg %C	9	112150516
ebi-a-GCST90001487	rs10897856	Activated Treg %C	11	81359416
ebi-a-GCST90001487	rs72969219	Activated Treg %C	11	38338290
ebi-a-GCST90001487	rs11066188	Activated Treg %C	12	112610714
ebi-a-GCST90001487	rs12441465	Activated Treg %C	15	80970517
ebi-a-GCST90001487	rs2030207	Activated Treg %C	15	92551913
ebi-a-GCST90001487	rs2356747	Activated Treg %C	16	21252443
ebi-a-GCST90001487	rs145044935	Activated Treg %C	18	48643554
ebi-a-GCST90001487	rs117140551	Activated Treg %C	19	23103264
ebi-a-GCST90001488	rs138751156	Activated Treg %C	1	77300547
ebi-a-GCST90001488	rs56295335	Activated Treg %C	1	10062088
ebi-a-GCST90001488	rs112009416	Activated Treg %C	1	199163362
ebi-a-GCST90001488	rs11579717	Activated Treg %C	1	199999121
ebi-a-GCST90001488	rs138915779	Activated Treg %C	1	190079170
ebi-a-GCST90001488	rs139138505	Activated Treg %C	1	225667295
ebi-a-GCST90001488	rs149143064	Activated Treg %C	2	39755475
ebi-a-GCST90001488	rs10511150	Activated Treg %C	3	94952474
ebi-a-GCST90001488	rs4109437	Activated Treg %C	4	190769223
ebi-a-GCST90001488	rs16903407	Activated Treg %C	5	2165975
ebi-a-GCST90001488	rs10116430	Activated Treg %C	9	11644911
ebi-a-GCST90001488	rs3793566	Activated Treg %C	9	112150516
ebi-a-GCST90001488	rs7078614	Activated Treg %C	10	6075831
ebi-a-GCST90001488	rs597808	Activated Treg %C	12	111973358

ebi-a-GCST90001488	rs17038606	Activated Treg %C	12	107042891
ebi-a-GCST90001488	rs139191636	Activated Treg %C	12	23591661
ebi-a-GCST90001488	rs1820840	Activated Treg %C	16	84640780
ebi-a-GCST90001488	rs117140551	Activated Treg %C	19	23103264
ebi-a-GCST90001488	rs8112021	Activated Treg %C	19	22667560
ebi-a-GCST90001489	rs7432284	CD39+ activated T	3	186094684
ebi-a-GCST90001489	rs139015125	CD39+ activated T	4	165512454
ebi-a-GCST90001489	rs12648210	CD39+ activated T	4	89266460
ebi-a-GCST90001489	rs142422333	CD39+ activated T	5	155375550
ebi-a-GCST90001489	rs10059458	CD39+ activated T	5	73681489
ebi-a-GCST90001489	rs78027772	CD39+ activated T	7	126248228
ebi-a-GCST90001489	rs12707040	CD39+ activated T	7	80480551
ebi-a-GCST90001489	rs139577399	CD39+ activated T	7	23203415
ebi-a-GCST90001489	rs11777493	CD39+ activated T	8	113921646
ebi-a-GCST90001489	rs35163827	CD39+ activated T	8	9606332
ebi-a-GCST90001489	rs191262582	CD39+ activated T	9	85042639
ebi-a-GCST90001489	rs11795139	CD39+ activated T	9	86150415
ebi-a-GCST90001489	rs7088584	CD39+ activated T	10	97607644
ebi-a-GCST90001489	rs72809762	CD39+ activated T	10	97294154
ebi-a-GCST90001489	rs2251521	CD39+ activated T	10	109471331
ebi-a-GCST90001489	rs10882701	CD39+ activated T	10	97741071
ebi-a-GCST90001489	rs11188757	CD39+ activated T	10	98205222
ebi-a-GCST90001489	rs111806244	CD39+ activated T	10	47637202
ebi-a-GCST90001489	rs10786199	CD39+ activated T	10	97069472
ebi-a-GCST90001489	rs11002579	CD39+ activated T	10	80258836
ebi-a-GCST90001489	rs202211780	CD39+ activated T	10	97016742
ebi-a-GCST90001489	rs4321083	CD39+ activated T	13	84031916
ebi-a-GCST90001489	rs4441121	CD39+ activated T	13	19650584
ebi-a-GCST90001489	rs77020191	CD39+ activated T	15	59643449
ebi-a-GCST90001489	rs56197234	CD39+ activated T	15	70222702
ebi-a-GCST90001489	rs289708	CD39+ activated T	16	57038162
ebi-a-GCST90001489	rs139917391	CD39+ activated T	16	18152860
ebi-a-GCST90001489	rs8076447	CD39+ activated T	17	75229883
ebi-a-GCST90001489	rs618250	CD39+ activated T	18	362116
ebi-a-GCST90001489	rs7267949	CD39+ activated T	20	17586584
ebi-a-GCST90001489	rs4811602	CD39+ activated T	20	36849088
ebi-a-GCST90001490	rs118006237	CD39+ activated T	1	100480230
ebi-a-GCST90001490	rs2171655	CD39+ activated T	2	127229166
ebi-a-GCST90001490	rs72805325	CD39+ activated T	2	60887023
ebi-a-GCST90001490	rs6432076	CD39+ activated T	2	10368044
ebi-a-GCST90001490	rs12106894	CD39+ activated T	3	127066325
ebi-a-GCST90001490	rs115545577	CD39+ activated T	4	114230061
ebi-a-GCST90001490	rs77630931	CD39+ activated T	5	80287625
ebi-a-GCST90001490	rs7774484	CD39+ activated T	6	7465057
ebi-a-GCST90001491	rs61818169	CD39+ activated T	1	113522834
ebi-a-GCST90001491	rs7681044	CD39+ activated T	4	99477128
ebi-a-GCST90001491	rs12648210	CD39+ activated T	4	89266460
ebi-a-GCST90001491	rs79629290	CD39+ activated T	5	56760264

ebi-a-GCST90001491	rs115722307	CD39+ activated Tı	5	172789806
ebi-a-GCST90001491	rs62390650	CD39+ activated Tı	6	1652500
ebi-a-GCST90001491	rs138483328	CD39+ activated Tı	6	108484291
ebi-a-GCST90001491	rs117008259	CD39+ activated Tı	8	68482844
ebi-a-GCST90001491	rs7035176	CD39+ activated Tı	9	119577258
ebi-a-GCST90001491	rs72809762	CD39+ activated Tı	10	97294154
ebi-a-GCST90001491	rs12268044	CD39+ activated Tı	10	98794615
ebi-a-GCST90001491	rs111806244	CD39+ activated Tı	10	47637202
ebi-a-GCST90001491	rs202211780	CD39+ activated Tı	10	97016742
ebi-a-GCST90001491	rs61856855	CD39+ activated Tı	10	98558540
ebi-a-GCST90001491	rs141018090	CD39+ activated Tı	10	98169221
ebi-a-GCST90001491	rs148430006	CD39+ activated Tı	10	95923091
ebi-a-GCST90001491	rs10882655	CD39+ activated Tı	10	97474371
ebi-a-GCST90001491	rs10882701	CD39+ activated Tı	10	97741071
ebi-a-GCST90001491	rs148903835	CD39+ activated Tı	10	108283818
ebi-a-GCST90001491	rs10786199	CD39+ activated Tı	10	97069472
ebi-a-GCST90001491	rs117858605	CD39+ activated Tı	12	81434565
ebi-a-GCST90001491	rs74018295	CD39+ activated Tı	15	57870174
ebi-a-GCST90001491	rs117344160	CD39+ activated Tı	15	45274540
ebi-a-GCST90001491	rs72715941	CD39+ activated Tı	15	27293286
ebi-a-GCST90001491	rs146341199	CD39+ activated Tı	19	12479291
ebi-a-GCST90001491	rs4550723	CD39+ activated Tı	20	36838101
ebi-a-GCST90001492	rs11579717	Secreting Treg AC	1	199999121
ebi-a-GCST90001492	rs138915779	Secreting Treg AC	1	190079170
ebi-a-GCST90001492	rs113435341	Secreting Treg AC	1	198151688
ebi-a-GCST90001492	rs76202906	Secreting Treg AC	1	113476831
ebi-a-GCST90001492	rs72915529	Secreting Treg AC	2	54970478
ebi-a-GCST90001492	rs138960714	Secreting Treg AC	4	123422719
ebi-a-GCST90001492	rs114547596	Secreting Treg AC	4	97974344
ebi-a-GCST90001492	rs114437347	Secreting Treg AC	4	86967620
ebi-a-GCST90001492	rs507492	Secreting Treg AC	6	109031005
ebi-a-GCST90001492	rs75280379	Secreting Treg AC	6	121512597
ebi-a-GCST90001492	rs6942586	Secreting Treg AC	7	36530276
ebi-a-GCST90001492	rs17717645	Secreting Treg AC	7	18003774
ebi-a-GCST90001492	rs56397630	Secreting Treg AC	10	105304351
ebi-a-GCST90001492	rs61839660	Secreting Treg AC	10	6094697
ebi-a-GCST90001492	rs11217768	Secreting Treg AC	11	120086680
ebi-a-GCST90001492	rs12297353	Secreting Treg AC	12	105434300
ebi-a-GCST90001492	rs75596315	Secreting Treg AC	12	122520112
ebi-a-GCST90001492	rs3184504	Secreting Treg AC	12	111884608
ebi-a-GCST90001492	rs145600407	Secreting Treg AC	13	19280601
ebi-a-GCST90001492	rs7162344	Secreting Treg AC	15	26030485
ebi-a-GCST90001492	rs153054	Secreting Treg AC	16	69446054
ebi-a-GCST90001492	rs149951966	Secreting Treg AC	17	41137136
ebi-a-GCST90001492	rs1041737	Secreting Treg AC	21	34318056
ebi-a-GCST90001493	rs11579717	Secreting Treg % C	1	199999121
ebi-a-GCST90001493	rs116794175	Secreting Treg % C	1	194010208
ebi-a-GCST90001493	rs187089343	Secreting Treg % C	1	182091538

ebi-a-GCST90001493	rs11577174	Secreting Treg % C	1	230492271
ebi-a-GCST90001493	rs34645541	Secreting Treg % C	1	195875605
ebi-a-GCST90001493	rs60220946	Secreting Treg % C	1	193505625
ebi-a-GCST90001493	rs12744266	Secreting Treg % C	1	248047667
ebi-a-GCST90001493	rs113435341	Secreting Treg % C	1	198151688
ebi-a-GCST90001493	rs75981740	Secreting Treg % C	1	5012020
ebi-a-GCST90001493	rs17607399	Secreting Treg % C	2	41884212
ebi-a-GCST90001493	rs413431	Secreting Treg % C	2	39176695
ebi-a-GCST90001493	rs6751481	Secreting Treg % C	2	38897810
ebi-a-GCST90001493	rs72966214	Secreting Treg % C	2	208014310
ebi-a-GCST90001493	rs12993274	Secreting Treg % C	2	42378770
ebi-a-GCST90001493	rs62322628	Secreting Treg % C	4	122780838
ebi-a-GCST90001493	rs11739607	Secreting Treg % C	5	27694229
ebi-a-GCST90001493	rs10059100	Secreting Treg % C	5	120100784
ebi-a-GCST90001493	rs9265947	Secreting Treg % C	6	31315165
ebi-a-GCST90001493	rs7825970	Secreting Treg % C	8	3414155
ebi-a-GCST90001493	rs73579441	Secreting Treg % C	8	49024059
ebi-a-GCST90001493	rs273421	Secreting Treg % C	8	132542825
ebi-a-GCST90001493	rs79224901	Secreting Treg % C	8	3651438
ebi-a-GCST90001493	rs117608988	Secreting Treg % C	9	86285391
ebi-a-GCST90001493	rs79607503	Secreting Treg % C	9	135102542
ebi-a-GCST90001493	rs7893324	Secreting Treg % C	10	6111075
ebi-a-GCST90001493	rs4759006	Secreting Treg % C	12	56009543
ebi-a-GCST90001493	rs186940149	Secreting Treg % C	13	36528979
ebi-a-GCST90001493	rs2148708	Secreting Treg % C	13	21794935
ebi-a-GCST90001493	rs79235233	Secreting Treg % C	14	92726328
ebi-a-GCST90001493	rs562562	Secreting Treg % C	18	24263725
ebi-a-GCST90001493	rs369976766	Secreting Treg % C	22	28835840
ebi-a-GCST90001494	rs113435341	Secreting Treg %Cl	1	198151688
ebi-a-GCST90001494	rs143290109	Secreting Treg %Cl	1	103543193
ebi-a-GCST90001494	rs10925924	Secreting Treg %Cl	1	239767145
ebi-a-GCST90001494	rs35378667	Secreting Treg %Cl	1	205001095
ebi-a-GCST90001494	rs11579717	Secreting Treg %Cl	1	199999121
ebi-a-GCST90001494	rs138915779	Secreting Treg %Cl	1	190079170
ebi-a-GCST90001494	rs12712610	Secreting Treg %Cl	2	38897249
ebi-a-GCST90001494	rs11124653	Secreting Treg %Cl	2	39112166
ebi-a-GCST90001494	rs73155096	Secreting Treg %Cl	3	149610852
ebi-a-GCST90001494	rs6438325	Secreting Treg %Cl	3	116384674
ebi-a-GCST90001494	rs358809	Secreting Treg %Cl	3	55349822
ebi-a-GCST90001494	rs9838548	Secreting Treg %Cl	3	126747367
ebi-a-GCST90001494	rs7651919	Secreting Treg %Cl	3	4597367
ebi-a-GCST90001494	rs186715000	Secreting Treg %Cl	4	1589324
ebi-a-GCST90001494	rs2668533	Secreting Treg %Cl	4	54555201
ebi-a-GCST90001494	rs114437347	Secreting Treg %Cl	4	86967620
ebi-a-GCST90001494	rs12642085	Secreting Treg %Cl	4	57549528
ebi-a-GCST90001494	rs138960714	Secreting Treg %Cl	4	123422719
ebi-a-GCST90001494	rs9689649	Secreting Treg %Cl	6	162101178
ebi-a-GCST90001494	rs185363826	Secreting Treg %Cl	7	128186610

ebi-a-GCST90001494	rs74830391	Secreting Treg %Cl	7	37427289
ebi-a-GCST90001494	rs6602392	Secreting Treg %Cl	10	6078079
ebi-a-GCST90001494	rs7893324	Secreting Treg %Cl	10	6111075
ebi-a-GCST90001494	rs10905879	Secreting Treg %Cl	10	6177083
ebi-a-GCST90001494	rs149375589	Secreting Treg %Cl	11	67333370
ebi-a-GCST90001494	rs10772894	Secreting Treg %Cl	12	16263287
ebi-a-GCST90001495	rs114114791	CD39+ secreting Tı	1	25840181
ebi-a-GCST90001495	rs7570197	CD39+ secreting Tı	2	79140712
ebi-a-GCST90001495	rs139577399	CD39+ secreting Tı	7	23203415
ebi-a-GCST90001495	rs76464802	CD39+ secreting Tı	7	67769786
ebi-a-GCST90001495	rs75270901	CD39+ secreting Tı	7	80556281
ebi-a-GCST90001495	rs57516532	CD39+ secreting Tı	7	1172869
ebi-a-GCST90001495	rs78027772	CD39+ secreting Tı	7	126248228
ebi-a-GCST90001495	rs150315125	CD39+ secreting Tı	8	10090715
ebi-a-GCST90001495	rs74791100	CD39+ secreting Tı	9	86052403
ebi-a-GCST90001495	rs10882324	CD39+ secreting Tı	10	82674931
ebi-a-GCST90001495	rs76710109	CD39+ secreting Tı	10	131817745
ebi-a-GCST90001495	rs10786199	CD39+ secreting Tı	10	97069472
ebi-a-GCST90001495	rs11598645	CD39+ secreting Tı	10	68351521
ebi-a-GCST90001495	rs11254133	CD39+ secreting Tı	10	16697829
ebi-a-GCST90001495	rs72809762	CD39+ secreting Tı	10	97294154
ebi-a-GCST90001495	rs10882655	CD39+ secreting Tı	10	97474371
ebi-a-GCST90001495	rs11188757	CD39+ secreting Tı	10	98205222
ebi-a-GCST90001495	rs202211780	CD39+ secreting Tı	10	97016742
ebi-a-GCST90001495	rs10882701	CD39+ secreting Tı	10	97741071
ebi-a-GCST90001495	rs7937686	CD39+ secreting Tı	11	130313067
ebi-a-GCST90001495	rs149951966	CD39+ secreting Tı	17	41137136
ebi-a-GCST90001495	rs11655883	CD39+ secreting Tı	17	75234757
ebi-a-GCST90001495	rs56216441	CD39+ secreting Tı	18	65545793
ebi-a-GCST90001495	rs111461378	CD39+ secreting Tı	20	15575550
ebi-a-GCST90001495	rs7267949	CD39+ secreting Tı	20	17586584
ebi-a-GCST90001495	rs2836025	CD39+ secreting Tı	21	39232216
ebi-a-GCST90001495	rs6005121	CD39+ secreting Tı	22	27065117
ebi-a-GCST90001496	rs282011	CD39+ secreting Tı	1	91858207
ebi-a-GCST90001496	rs147911368	CD39+ secreting Tı	1	97319841
ebi-a-GCST90001496	rs118006237	CD39+ secreting Tı	1	100480230
ebi-a-GCST90001496	rs2316911	CD39+ secreting Tı	2	236543890
ebi-a-GCST90001496	rs78458369	CD39+ secreting Tı	2	74164120
ebi-a-GCST90001496	rs2171655	CD39+ secreting Tı	2	127229166
ebi-a-GCST90001496	rs73037918	CD39+ secreting Tı	3	10923961
ebi-a-GCST90001496	rs74346764	CD39+ secreting Tı	3	145230069
ebi-a-GCST90001496	rs35796547	CD39+ secreting Tı	3	74432399
ebi-a-GCST90001496	rs11930070	CD39+ secreting Tı	4	54265667
ebi-a-GCST90001496	rs17571942	CD39+ secreting Tı	5	29581038
ebi-a-GCST90001496	rs7774484	CD39+ secreting Tı	6	7465057
ebi-a-GCST90001496	rs2569847	CD39+ secreting Tı	6	1651973
ebi-a-GCST90001496	rs10247216	CD39+ secreting Tı	7	97140811
ebi-a-GCST90001496	rs117748540	CD39+ secreting Tı	10	95002810

ebi-a-GCST90001496	rs114844216	CD39+ secreting Tı	10	98158952
ebi-a-GCST90001496	rs4918960	CD39+ secreting Tı	10	97472570
ebi-a-GCST90001496	rs146426185	CD39+ secreting Tı	10	99452494
ebi-a-GCST90001496	rs7910217	CD39+ secreting Tı	10	96999873
ebi-a-GCST90001496	rs147121076	CD39+ secreting Tı	10	95924521
ebi-a-GCST90001496	rs118047459	CD39+ secreting Tı	10	69891249
ebi-a-GCST90001496	rs117200502	CD39+ secreting Tı	10	97070990
ebi-a-GCST90001496	rs145557266	CD39+ secreting Tı	10	95850055
ebi-a-GCST90001496	rs10882701	CD39+ secreting Tı	10	97741071
ebi-a-GCST90001496	rs11188950	CD39+ secreting Tı	10	98619863
ebi-a-GCST90001496	rs118111902	CD39+ secreting Tı	11	19624351
ebi-a-GCST90001496	rs7984101	CD39+ secreting Tı	13	43764296
ebi-a-GCST90001496	rs111461378	CD39+ secreting Tı	20	15575550
ebi-a-GCST90001496	rs5769126	CD39+ secreting Tı	22	47135205
ebi-a-GCST90001497	rs282011	CD39+ secreting Tı	1	91858207
ebi-a-GCST90001497	rs118006237	CD39+ secreting Tı	1	100480230
ebi-a-GCST90001497	rs2171655	CD39+ secreting Tı	2	127229166
ebi-a-GCST90001497	rs78458369	CD39+ secreting Tı	2	74164120
ebi-a-GCST90001497	rs74346764	CD39+ secreting Tı	3	145230069
ebi-a-GCST90001497	rs6805627	CD39+ secreting Tı	3	76155328
ebi-a-GCST90001497	rs11930070	CD39+ secreting Tı	4	54265667
ebi-a-GCST90001497	rs17571942	CD39+ secreting Tı	5	29581038
ebi-a-GCST90001497	rs2569847	CD39+ secreting Tı	6	1651973
ebi-a-GCST90001497	rs115364130	CD39+ secreting Tı	7	83238053
ebi-a-GCST90001497	rs59428330	CD39+ secreting Tı	9	138051433
ebi-a-GCST90001497	rs114844216	CD39+ secreting Tı	10	98158952
ebi-a-GCST90001497	rs117200502	CD39+ secreting Tı	10	97070990
ebi-a-GCST90001497	rs61873892	CD39+ secreting Tı	10	100118038
ebi-a-GCST90001497	rs145557266	CD39+ secreting Tı	10	95850055
ebi-a-GCST90001497	rs7910217	CD39+ secreting Tı	10	96999873
ebi-a-GCST90001497	rs10882701	CD39+ secreting Tı	10	97741071
ebi-a-GCST90001497	rs146426185	CD39+ secreting Tı	10	99452494
ebi-a-GCST90001497	rs7080854	CD39+ secreting Tı	10	116367153
ebi-a-GCST90001497	rs147121076	CD39+ secreting Tı	10	95924521
ebi-a-GCST90001497	rs4918960	CD39+ secreting Tı	10	97472570
ebi-a-GCST90001497	rs111461378	CD39+ secreting Tı	20	15575550
ebi-a-GCST90001497	rs79919890	CD39+ secreting Tı	22	47078385
ebi-a-GCST90001498	rs144404462	Activated & resting	1	198005348
ebi-a-GCST90001498	rs6751481	Activated & resting	2	38897810
ebi-a-GCST90001498	rs55732147	Activated & resting	2	241675786
ebi-a-GCST90001498	rs408686	Activated & resting	2	39177320
ebi-a-GCST90001498	rs146746694	Activated & resting	6	155566192
ebi-a-GCST90001498	rs2524084	Activated & resting	6	31241639
ebi-a-GCST90001498	rs9272226	Activated & resting	6	32602396
ebi-a-GCST90001498	rs147199078	Activated & resting	7	80542211
ebi-a-GCST90001498	rs2108908	Activated & resting	7	152112789
ebi-a-GCST90001498	rs182630194	Activated & resting	7	71331239
ebi-a-GCST90001498	rs1819445	Activated & resting	9	96772051

ebi-a-GCST90001498	rs12049737	Activated & resting	10	2614502
ebi-a-GCST90001498	rs78409263	Activated & resting	11	79202514
ebi-a-GCST90001498	rs11850806	Activated & resting	14	78669137
ebi-a-GCST90001498	rs66762238	Activated & resting	17	20792822
ebi-a-GCST90001498	rs8064831	Activated & resting	17	10150766
ebi-a-GCST90001498	rs9709738	Activated & resting	18	60137303
ebi-a-GCST90001498	rs79684619	Activated & resting	18	64907349
ebi-a-GCST90001498	rs6074425	Activated & resting	20	1295889
ebi-a-GCST90001499	rs6587385	Activated & resting	1	248047256
ebi-a-GCST90001499	rs113435341	Activated & resting	1	198151688
ebi-a-GCST90001499	rs11579717	Activated & resting	1	199999121
ebi-a-GCST90001499	rs187089343	Activated & resting	1	182091538
ebi-a-GCST90001499	rs11577174	Activated & resting	1	230492271
ebi-a-GCST90001499	rs60220946	Activated & resting	1	193505625
ebi-a-GCST90001499	rs116794175	Activated & resting	1	194010208
ebi-a-GCST90001499	rs34645541	Activated & resting	1	195875605
ebi-a-GCST90001499	rs75981740	Activated & resting	1	5012020
ebi-a-GCST90001499	rs12993274	Activated & resting	2	42378770
ebi-a-GCST90001499	rs6751481	Activated & resting	2	38897810
ebi-a-GCST90001499	rs17607399	Activated & resting	2	41884212
ebi-a-GCST90001499	rs413431	Activated & resting	2	39176695
ebi-a-GCST90001499	rs62322628	Activated & resting	4	122780838
ebi-a-GCST90001499	rs11739607	Activated & resting	5	27694229
ebi-a-GCST90001499	rs10059100	Activated & resting	5	120100784
ebi-a-GCST90001499	rs9265947	Activated & resting	6	31315165
ebi-a-GCST90001499	rs7825970	Activated & resting	8	3414155
ebi-a-GCST90001499	rs273421	Activated & resting	8	132542825
ebi-a-GCST90001499	rs79224901	Activated & resting	8	3651438
ebi-a-GCST90001499	rs73579441	Activated & resting	8	49024059
ebi-a-GCST90001499	rs79607503	Activated & resting	9	135102542
ebi-a-GCST90001499	rs7893324	Activated & resting	10	6111075
ebi-a-GCST90001499	rs4759006	Activated & resting	12	56009543
ebi-a-GCST90001499	rs2148708	Activated & resting	13	21794935
ebi-a-GCST90001499	rs186940149	Activated & resting	13	36528979
ebi-a-GCST90001499	rs79235233	Activated & resting	14	92726328
ebi-a-GCST90001499	rs562562	Activated & resting	18	24263725
ebi-a-GCST90001499	rs369976766	Activated & resting	22	28835840
ebi-a-GCST90001500	rs76882749	Activated & resting	1	54532130
ebi-a-GCST90001500	rs147104104	Activated & resting	1	208455675
ebi-a-GCST90001500	rs17583875	Activated & resting	1	197924770
ebi-a-GCST90001500	rs116794175	Activated & resting	1	194010208
ebi-a-GCST90001500	rs190023756	Activated & resting	1	199471227
ebi-a-GCST90001500	rs572410764	Activated & resting	1	239509035
ebi-a-GCST90001500	rs6751481	Activated & resting	2	38897810
ebi-a-GCST90001500	rs408686	Activated & resting	2	39177320
ebi-a-GCST90001500	rs6724848	Activated & resting	2	38678651
ebi-a-GCST90001500	rs1371033	Activated & resting	2	148248261
ebi-a-GCST90001500	rs6733868	Activated & resting	2	25499867

ebi-a-GCST90001500	rs13410035	Activated & resting	2	161139489
ebi-a-GCST90001500	rs13006059	Activated & resting	2	43022720
ebi-a-GCST90001500	rs73009664	Activated & resting	3	2721613
ebi-a-GCST90001500	rs62285107	Activated & resting	4	1731279
ebi-a-GCST90001500	rs17780836	Activated & resting	5	58622934
ebi-a-GCST90001500	rs62390348	Activated & resting	6	14892248
ebi-a-GCST90001500	rs9269074	Activated & resting	6	32440792
ebi-a-GCST90001500	rs137997033	Activated & resting	6	124005085
ebi-a-GCST90001500	rs2708609	Activated & resting	7	112101909
ebi-a-GCST90001500	rs56090633	Activated & resting	7	51393519
ebi-a-GCST90001500	rs76225340	Activated & resting	8	3654985
ebi-a-GCST90001500	rs145165211	Activated & resting	8	21371903
ebi-a-GCST90001500	rs114400243	Activated & resting	9	11577096
ebi-a-GCST90001500	rs514150	Activated & resting	10	8084573
ebi-a-GCST90001500	rs1247730	Activated & resting	11	79922768
ebi-a-GCST90001500	rs11109601	Activated & resting	12	99174032
ebi-a-GCST90001500	rs61924635	Activated & resting	12	25650645
ebi-a-GCST90001500	rs77071786	Activated & resting	12	23501010
ebi-a-GCST90001500	rs370689709	Activated & resting	13	67699975
ebi-a-GCST90001500	rs186940149	Activated & resting	13	36528979
ebi-a-GCST90001500	rs113376235	Activated & resting	16	75514711
ebi-a-GCST90001500	rs60174426	Activated & resting	16	29355773
ebi-a-GCST90001500	rs461709	Activated & resting	19	47666711
ebi-a-GCST90001501	rs11579717	Activated & secreti	1	199999121
ebi-a-GCST90001501	rs113435341	Activated & secreti	1	198151688
ebi-a-GCST90001501	rs76202906	Activated & secreti	1	113476831
ebi-a-GCST90001501	rs112622820	Activated & secreti	1	225934912
ebi-a-GCST90001501	rs138915779	Activated & secreti	1	190079170
ebi-a-GCST90001501	rs115307639	Activated & secreti	4	98004266
ebi-a-GCST90001501	rs138960714	Activated & secreti	4	123422719
ebi-a-GCST90001501	rs114437347	Activated & secreti	4	86967620
ebi-a-GCST90001501	rs75280379	Activated & secreti	6	121512597
ebi-a-GCST90001501	rs1268153	Activated & secreti	6	109035696
ebi-a-GCST90001501	rs17717645	Activated & secreti	7	18003774
ebi-a-GCST90001501	rs575051668	Activated & secreti	7	31426904
ebi-a-GCST90001501	rs61839660	Activated & secreti	10	6094697
ebi-a-GCST90001501	rs10905876	Activated & secreti	10	6174601
ebi-a-GCST90001501	rs56397630	Activated & secreti	10	105304351
ebi-a-GCST90001501	rs75596315	Activated & secreti	12	122520112
ebi-a-GCST90001501	rs145600407	Activated & secreti	13	19280601
ebi-a-GCST90001501	rs7162344	Activated & secreti	15	26030485
ebi-a-GCST90001501	rs153054	Activated & secreti	16	69446054
ebi-a-GCST90001501	rs147638541	Activated & secreti	17	66206097
ebi-a-GCST90001501	rs149951966	Activated & secreti	17	41137136
ebi-a-GCST90001501	rs72833913	Activated & secreti	17	5713335
ebi-a-GCST90001501	rs9709738	Activated & secreti	18	60137303
ebi-a-GCST90001502	rs61828007	Activated & secreti	1	203502968
ebi-a-GCST90001502	rs11579717	Activated & secreti	1	199999121

ebi-a-GCST90001502	rs72651876	Activated & secreti	1	19206027
ebi-a-GCST90001502	rs138915779	Activated & secreti	1	190079170
ebi-a-GCST90001502	rs60220946	Activated & secreti	1	193505625
ebi-a-GCST90001502	rs150956189	Activated & secreti	1	214625781
ebi-a-GCST90001502	rs146899923	Activated & secreti	1	195919849
ebi-a-GCST90001502	rs187089343	Activated & secreti	1	182091538
ebi-a-GCST90001502	rs113435341	Activated & secreti	1	198151688
ebi-a-GCST90001502	rs3917863	Activated & secreti	1	169592401
ebi-a-GCST90001502	rs7530076	Activated & secreti	1	195825276
ebi-a-GCST90001502	rs139681117	Activated & secreti	2	23924920
ebi-a-GCST90001502	rs13000564	Activated & secreti	2	41596014
ebi-a-GCST90001502	rs115669925	Activated & secreti	2	111442886
ebi-a-GCST90001502	rs413431	Activated & secreti	2	39176695
ebi-a-GCST90001502	rs189383	Activated & secreti	2	8709770
ebi-a-GCST90001502	rs115907036	Activated & secreti	2	37537677
ebi-a-GCST90001502	rs112553628	Activated & secreti	2	38077329
ebi-a-GCST90001502	rs17607399	Activated & secreti	2	41884212
ebi-a-GCST90001502	rs6751481	Activated & secreti	2	38897810
ebi-a-GCST90001502	rs78181865	Activated & secreti	3	84481659
ebi-a-GCST90001502	rs138960714	Activated & secreti	4	123422719
ebi-a-GCST90001502	rs10059100	Activated & secreti	5	120100784
ebi-a-GCST90001502	rs112637846	Activated & secreti	6	149114553
ebi-a-GCST90001502	rs1634783	Activated & secreti	6	31283604
ebi-a-GCST90001502	rs11969660	Activated & secreti	6	14395373
ebi-a-GCST90001502	rs9274712	Activated & secreti	6	32637298
ebi-a-GCST90001502	rs273421	Activated & secreti	8	132542825
ebi-a-GCST90001502	rs76109501	Activated & secreti	8	28323922
ebi-a-GCST90001502	rs73579441	Activated & secreti	8	49024059
ebi-a-GCST90001502	rs12722496	Activated & secreti	10	6096667
ebi-a-GCST90001502	rs192123974	Activated & secreti	11	13923816
ebi-a-GCST90001502	rs145406940	Activated & secreti	12	23093493
ebi-a-GCST90001502	rs138463974	Activated & secreti	12	132114547
ebi-a-GCST90001502	rs116850494	Activated & secreti	13	60389778
ebi-a-GCST90001502	rs191384725	Activated & secreti	16	7628264
ebi-a-GCST90001502	rs568546879	Activated & secreti	18	33878112
ebi-a-GCST90001502	rs112455863	Activated & secreti	20	20514860
ebi-a-GCST90001502	rs113000944	Activated & secreti	20	48836592
ebi-a-GCST90001503	rs11579717	Activated & secreti	1	199999121
ebi-a-GCST90001503	rs146512168	Activated & secreti	1	182579163
ebi-a-GCST90001503	rs10925924	Activated & secreti	1	239767145
ebi-a-GCST90001503	rs143290109	Activated & secreti	1	103543193
ebi-a-GCST90001503	rs138915779	Activated & secreti	1	190079170
ebi-a-GCST90001503	rs113435341	Activated & secreti	1	198151688
ebi-a-GCST90001503	rs35378667	Activated & secreti	1	205001095
ebi-a-GCST90001503	rs12712610	Activated & secreti	2	38897249
ebi-a-GCST90001503	rs73155096	Activated & secreti	3	149610852
ebi-a-GCST90001503	rs4685759	Activated & secreti	3	4602572
ebi-a-GCST90001503	rs6438325	Activated & secreti	3	116384674

ebi-a-GCST90001503	rs114437347	Activated & secreti	4	86967620
ebi-a-GCST90001503	rs186715000	Activated & secreti	4	1589324
ebi-a-GCST90001503	rs2668533	Activated & secreti	4	54555201
ebi-a-GCST90001503	rs138960714	Activated & secreti	4	123422719
ebi-a-GCST90001503	rs12642085	Activated & secreti	4	57549528
ebi-a-GCST90001503	rs117947776	Activated & secreti	6	103491045
ebi-a-GCST90001503	rs74830391	Activated & secreti	7	37427289
ebi-a-GCST90001503	rs2958568	Activated & secreti	8	19426186
ebi-a-GCST90001503	rs61839660	Activated & secreti	10	6094697
ebi-a-GCST90001503	rs11256966	Activated & secreti	10	6185310
ebi-a-GCST90001503	rs149375589	Activated & secreti	11	67333370
ebi-a-GCST90001503	rs11221698	Activated & secreti	11	129253988
ebi-a-GCST90001503	rs17465223	Activated & secreti	12	23612754
ebi-a-GCST90001503	rs145444215	Activated & secreti	14	103258906
ebi-a-GCST90001503	rs4901222	Activated & secreti	14	38241882
ebi-a-GCST90001503	rs1465510	Activated & secreti	15	39046198
ebi-a-GCST90001503	rs149703905	Activated & secreti	16	88710635
ebi-a-GCST90001503	rs140078626	Activated & secreti	16	28651774
ebi-a-GCST90001503	rs11655776	Activated & secreti	17	2761413
ebi-a-GCST90001503	rs143166038	Activated & secreti	19	43502925
ebi-a-GCST90001503	rs6065288	Activated & secreti	20	39567774
ebi-a-GCST90001503	rs147838148	Activated & secreti	22	47814108
ebi-a-GCST90001504	rs12145444	CD25hi AC	1	198008884
ebi-a-GCST90001504	rs61817905	CD25hi AC	1	113666355
ebi-a-GCST90001504	rs6541307	CD25hi AC	1	230100836
ebi-a-GCST90001504	rs78193200	CD25hi AC	1	149148053
ebi-a-GCST90001504	rs150956240	CD25hi AC	2	21238984
ebi-a-GCST90001504	rs7560118	CD25hi AC	2	225186492
ebi-a-GCST90001504	rs188501834	CD25hi AC	3	165293521
ebi-a-GCST90001504	rs79749232	CD25hi AC	5	141144312
ebi-a-GCST90001504	rs9343914	CD25hi AC	6	80166444
ebi-a-GCST90001504	rs507492	CD25hi AC	6	109031005
ebi-a-GCST90001504	rs17717645	CD25hi AC	7	18003774
ebi-a-GCST90001504	rs140960913	CD25hi AC	7	158180337
ebi-a-GCST90001504	rs61839660	CD25hi AC	10	6094697
ebi-a-GCST90001504	rs137903114	CD25hi AC	11	130770585
ebi-a-GCST90001504	rs11217768	CD25hi AC	11	120086680
ebi-a-GCST90001504	rs79596262	CD25hi AC	13	38859265
ebi-a-GCST90001504	rs145600407	CD25hi AC	13	19280601
ebi-a-GCST90001504	rs117676560	CD25hi AC	13	74976559
ebi-a-GCST90001504	rs142289362	CD25hi AC	16	77778611
ebi-a-GCST90001504	rs188505795	CD25hi AC	16	28223373
ebi-a-GCST90001504	rs117614870	CD25hi AC	17	63857170
ebi-a-GCST90001504	rs149951966	CD25hi AC	17	41137136
ebi-a-GCST90001504	rs11697981	CD25hi AC	20	43093014
ebi-a-GCST90001504	rs6131700	CD25hi AC	20	15742230
ebi-a-GCST90001504	rs2019002	CD25hi AC	22	25652094
ebi-a-GCST90001505	rs140655813	CD25hi %CD4+	1	163746986

ebi-a-GCST90001505	rs181741769	CD25hi %CD4+	1	26999658
ebi-a-GCST90001505	rs115815681	CD25hi %CD4+	1	149047683
ebi-a-GCST90001505	rs60148251	CD25hi %CD4+	1	11819596
ebi-a-GCST90001505	rs79327855	CD25hi %CD4+	3	144158111
ebi-a-GCST90001505	rs56107686	CD25hi %CD4+	3	189510063
ebi-a-GCST90001505	rs3732449	CD25hi %CD4+	3	170198898
ebi-a-GCST90001505	rs419099	CD25hi %CD4+	5	102089442
ebi-a-GCST90001505	rs9268791	CD25hi %CD4+	6	32421073
ebi-a-GCST90001505	rs7831142	CD25hi %CD4+	8	1673599
ebi-a-GCST90001505	rs117760133	CD25hi %CD4+	8	129187397
ebi-a-GCST90001505	rs2448157	CD25hi %CD4+	8	98470579
ebi-a-GCST90001505	rs114400243	CD25hi %CD4+	9	11577096
ebi-a-GCST90001505	rs71479760	CD25hi %CD4+	10	6183872
ebi-a-GCST90001505	rs61839660	CD25hi %CD4+	10	6094697
ebi-a-GCST90001505	rs11603619	CD25hi %CD4+	11	12094804
ebi-a-GCST90001505	rs11221698	CD25hi %CD4+	11	129253988
ebi-a-GCST90001505	rs116928928	CD25hi %CD4+	11	32030595
ebi-a-GCST90001505	rs7305147	CD25hi %CD4+	12	23524261
ebi-a-GCST90001505	rs537403506	CD25hi %CD4+	12	69385641
ebi-a-GCST90001505	rs12819777	CD25hi %CD4+	12	39382086
ebi-a-GCST90001505	rs140078626	CD25hi %CD4+	16	28651774
ebi-a-GCST90001505	rs74553250	CD25hi %CD4+	17	40063525
ebi-a-GCST90001505	rs117614870	CD25hi %CD4+	17	63857170
ebi-a-GCST90001505	rs12458491	CD25hi %CD4+	18	35307820
ebi-a-GCST90001505	rs80266752	CD25hi %CD4+	20	52010540
ebi-a-GCST90001505	rs6131700	CD25hi %CD4+	20	15742230
ebi-a-GCST90001506	rs78193200	CD25hi %T cell	1	149148053
ebi-a-GCST90001506	rs115815681	CD25hi %T cell	1	149047683
ebi-a-GCST90001506	rs113339346	CD25hi %T cell	2	42801648
ebi-a-GCST90001506	rs79327855	CD25hi %T cell	3	144158111
ebi-a-GCST90001506	rs6771179	CD25hi %T cell	3	14769598
ebi-a-GCST90001506	rs2403273	CD25hi %T cell	4	1760374
ebi-a-GCST90001506	rs1558501	CD25hi %T cell	4	55482148
ebi-a-GCST90001506	rs419099	CD25hi %T cell	5	102089442
ebi-a-GCST90001506	rs62392327	CD25hi %T cell	5	145144060
ebi-a-GCST90001506	rs4587210	CD25hi %T cell	6	1543191
ebi-a-GCST90001506	rs117469911	CD25hi %T cell	7	79791344
ebi-a-GCST90001506	rs183422804	CD25hi %T cell	8	90678452
ebi-a-GCST90001506	rs139753341	CD25hi %T cell	9	124831580
ebi-a-GCST90001506	rs2004605	CD25hi %T cell	9	37391292
ebi-a-GCST90001506	rs61839660	CD25hi %T cell	10	6094697
ebi-a-GCST90001506	rs62626343	CD25hi %T cell	10	6128374
ebi-a-GCST90001506	rs71506285	CD25hi %T cell	10	59399542
ebi-a-GCST90001506	rs58498656	CD25hi %T cell	11	127075073
ebi-a-GCST90001506	rs12325554	CD25hi %T cell	16	84053417
ebi-a-GCST90001506	rs11869223	CD25hi %T cell	17	33775713
ebi-a-GCST90001506	rs2702579	CD25hi %T cell	18	71659838
ebi-a-GCST90001506	rs141382158	CD25hi %T cell	20	590294

ebi-a-GCST90001506	rs67691565	CD25hi %T cell	20	52169857
ebi-a-GCST90001507	rs144350975	CD25hi CD45RA+	1	188000439
ebi-a-GCST90001507	rs141541045	CD25hi CD45RA+	1	182131075
ebi-a-GCST90001507	rs180839256	CD25hi CD45RA+	1	200021804
ebi-a-GCST90001507	rs55728470	CD25hi CD45RA+	1	244367408
ebi-a-GCST90001507	rs116794175	CD25hi CD45RA+	1	194010208
ebi-a-GCST90001507	rs116517602	CD25hi CD45RA+	1	198922068
ebi-a-GCST90001507	rs11124653	CD25hi CD45RA+	2	39112166
ebi-a-GCST90001507	rs114780506	CD25hi CD45RA+	2	104762054
ebi-a-GCST90001507	rs12712610	CD25hi CD45RA+	2	38897249
ebi-a-GCST90001507	rs17607399	CD25hi CD45RA+	2	41884212
ebi-a-GCST90001507	rs11693193	CD25hi CD45RA+	2	120485053
ebi-a-GCST90001507	rs116368402	CD25hi CD45RA+	3	145915913
ebi-a-GCST90001507	rs76537963	CD25hi CD45RA+	4	93093634
ebi-a-GCST90001507	rs28446907	CD25hi CD45RA+	5	109149543
ebi-a-GCST90001507	rs75798204	CD25hi CD45RA+	5	129494174
ebi-a-GCST90001507	rs73777152	CD25hi CD45RA+	5	106225233
ebi-a-GCST90001507	rs10905719	CD25hi CD45RA+	10	6115171
ebi-a-GCST90001507	rs75958070	CD25hi CD45RA+	11	79203049
ebi-a-GCST90001507	rs112238613	CD25hi CD45RA+	11	110766468
ebi-a-GCST90001507	rs79536602	CD25hi CD45RA+	15	78621679
ebi-a-GCST90001507	rs1645894	CD25hi CD45RA+	16	57340615
ebi-a-GCST90001507	rs117882514	CD25hi CD45RA+	18	6105334
ebi-a-GCST90001507	rs116034876	CD25hi CD45RA+	19	17018604
ebi-a-GCST90001507	rs75952429	CD25hi CD45RA+	19	15750527
ebi-a-GCST90001507	rs148388592	CD25hi CD45RA+	19	16483486
ebi-a-GCST90001507	rs1018576	CD25hi CD45RA+	20	1298257
ebi-a-GCST90001507	rs57061667	CD25hi CD45RA+	22	24096941
ebi-a-GCST90001508	rs182812626	CD25hi CD45RA+	1	189980053
ebi-a-GCST90001508	rs116517602	CD25hi CD45RA+	1	198922068
ebi-a-GCST90001508	rs77180728	CD25hi CD45RA+	1	226986331
ebi-a-GCST90001508	rs180839256	CD25hi CD45RA+	1	200021804
ebi-a-GCST90001508	rs61836370	CD25hi CD45RA+	1	188691469
ebi-a-GCST90001508	rs141541045	CD25hi CD45RA+	1	182131075
ebi-a-GCST90001508	rs17022951	CD25hi CD45RA+	2	38807441
ebi-a-GCST90001508	rs11124653	CD25hi CD45RA+	2	39112166
ebi-a-GCST90001508	rs13024840	CD25hi CD45RA+	2	38895920
ebi-a-GCST90001508	rs114780506	CD25hi CD45RA+	2	104762054
ebi-a-GCST90001508	rs1430018	CD25hi CD45RA+	2	38132316
ebi-a-GCST90001508	rs56000618	CD25hi CD45RA+	2	175199869
ebi-a-GCST90001508	rs71339620	CD25hi CD45RA+	2	87854860
ebi-a-GCST90001508	rs114437347	CD25hi CD45RA+	4	86967620
ebi-a-GCST90001508	rs28446907	CD25hi CD45RA+	5	109149543
ebi-a-GCST90001508	rs75798204	CD25hi CD45RA+	5	129494174
ebi-a-GCST90001508	rs73329062	CD25hi CD45RA+	7	44763296
ebi-a-GCST90001508	rs73579441	CD25hi CD45RA+	8	49024059
ebi-a-GCST90001508	rs13260343	CD25hi CD45RA+	8	14028645
ebi-a-GCST90001508	rs1264473	CD25hi CD45RA+	8	102431583

ebi-a-GCST90001508	rs150635171	CD25hi CD45RA+	9	137525725
ebi-a-GCST90001508	rs117626397	CD25hi CD45RA+	10	131423918
ebi-a-GCST90001508	rs10905719	CD25hi CD45RA+	10	6115171
ebi-a-GCST90001508	rs11195783	CD25hi CD45RA+	10	113754024
ebi-a-GCST90001508	rs75958070	CD25hi CD45RA+	11	79203049
ebi-a-GCST90001508	rs149541387	CD25hi CD45RA+	12	15408104
ebi-a-GCST90001508	rs116850494	CD25hi CD45RA+	13	60389778
ebi-a-GCST90001508	rs79536602	CD25hi CD45RA+	15	78621679
ebi-a-GCST90001508	rs1645894	CD25hi CD45RA+	16	57340615
ebi-a-GCST90001508	rs28592611	CD25hi CD45RA+	16	78381297
ebi-a-GCST90001508	rs12958644	CD25hi CD45RA+	18	6060693
ebi-a-GCST90001508	rs149429639	CD25hi CD45RA+	18	42913522
ebi-a-GCST90001508	rs188418491	CD25hi CD45RA+	19	777304
ebi-a-GCST90001508	rs274814	CD25hi CD45RA+	19	20836911
ebi-a-GCST90001508	rs738595	CD25hi CD45RA+	22	49372052
ebi-a-GCST90001509	rs180839256	CD25hi CD45RA+	1	200021804
ebi-a-GCST90001509	rs61836370	CD25hi CD45RA+	1	188691469
ebi-a-GCST90001509	rs76432001	CD25hi CD45RA+	1	245389000
ebi-a-GCST90001509	rs144350975	CD25hi CD45RA+	1	188000439
ebi-a-GCST90001509	rs116517602	CD25hi CD45RA+	1	198922068
ebi-a-GCST90001509	rs11124653	CD25hi CD45RA+	2	39112166
ebi-a-GCST90001509	rs72797312	CD25hi CD45RA+	2	38999003
ebi-a-GCST90001509	rs13024840	CD25hi CD45RA+	2	38895920
ebi-a-GCST90001509	rs17607399	CD25hi CD45RA+	2	41884212
ebi-a-GCST90001509	rs78032501	CD25hi CD45RA+	2	227684292
ebi-a-GCST90001509	rs114780506	CD25hi CD45RA+	2	104762054
ebi-a-GCST90001509	rs114437347	CD25hi CD45RA+	4	86967620
ebi-a-GCST90001509	rs75798204	CD25hi CD45RA+	5	129494174
ebi-a-GCST90001509	rs28446907	CD25hi CD45RA+	5	109149543
ebi-a-GCST90001509	rs67017865	CD25hi CD45RA+	8	138060119
ebi-a-GCST90001509	rs4876568	CD25hi CD45RA+	8	115299703
ebi-a-GCST90001509	rs1264473	CD25hi CD45RA+	8	102431583
ebi-a-GCST90001509	rs117626397	CD25hi CD45RA+	10	131423918
ebi-a-GCST90001509	rs10905719	CD25hi CD45RA+	10	6115171
ebi-a-GCST90001509	rs144105723	CD25hi CD45RA+	10	119138812
ebi-a-GCST90001509	rs75958070	CD25hi CD45RA+	11	79203049
ebi-a-GCST90001509	rs112238613	CD25hi CD45RA+	11	110766468
ebi-a-GCST90001509	rs79536602	CD25hi CD45RA+	15	78621679
ebi-a-GCST90001509	rs28592611	CD25hi CD45RA+	16	78381297
ebi-a-GCST90001509	rs149429639	CD25hi CD45RA+	18	42913522
ebi-a-GCST90001509	rs117882514	CD25hi CD45RA+	18	6105334
ebi-a-GCST90001509	rs117520868	CD25hi CD45RA+	19	16580644
ebi-a-GCST90001509	rs188418491	CD25hi CD45RA+	19	777304
ebi-a-GCST90001510	rs78193200	CD25hi CD45RA- (	1	149148053
ebi-a-GCST90001510	rs11579717	CD25hi CD45RA- (	1	199999121
ebi-a-GCST90001510	rs61817905	CD25hi CD45RA- (	1	113666355
ebi-a-GCST90001510	rs113435341	CD25hi CD45RA- (	1	198151688
ebi-a-GCST90001510	rs138915779	CD25hi CD45RA- (	1	190079170

ebi-a-GCST90001510	rs150956240	CD25hi CD45RA- (	2	21238984
ebi-a-GCST90001510	rs115575166	CD25hi CD45RA- (	2	38573789
ebi-a-GCST90001510	rs60349936	CD25hi CD45RA- (	4	32957041
ebi-a-GCST90001510	rs139310498	CD25hi CD45RA- (	6	168197693
ebi-a-GCST90001510	rs73176863	CD25hi CD45RA- (	7	156112045
ebi-a-GCST90001510	rs2596120	CD25hi CD45RA- (	8	76660230
ebi-a-GCST90001510	rs61839660	CD25hi CD45RA- (	10	6094697
ebi-a-GCST90001510	rs137903114	CD25hi CD45RA- (	11	130770585
ebi-a-GCST90001510	rs61045067	CD25hi CD45RA- (	12	47505424
ebi-a-GCST90001510	rs79596262	CD25hi CD45RA- (	13	38859265
ebi-a-GCST90001510	rs144999003	CD25hi CD45RA- (	15	33593233
ebi-a-GCST90001510	rs143283244	CD25hi CD45RA- (	15	94643151
ebi-a-GCST90001510	rs142289362	CD25hi CD45RA- (	16	77778611
ebi-a-GCST90001510	rs117614870	CD25hi CD45RA- (	17	63857170
ebi-a-GCST90001510	rs72942206	CD25hi CD45RA- (	18	55275584
ebi-a-GCST90001510	rs187133679	CD25hi CD45RA- (	19	34592058
ebi-a-GCST90001510	rs11697981	CD25hi CD45RA- (	20	43093014
ebi-a-GCST90001510	rs6131700	CD25hi CD45RA- (	20	15742230
ebi-a-GCST90001511	rs17583875	CD25hi CD45RA- (	1	197924770
ebi-a-GCST90001511	rs116794175	CD25hi CD45RA- (	1	194010208
ebi-a-GCST90001511	rs17666424	CD25hi CD45RA- (	1	159187530
ebi-a-GCST90001511	rs151238138	CD25hi CD45RA- (	2	171484062
ebi-a-GCST90001511	rs115575166	CD25hi CD45RA- (	2	38573789
ebi-a-GCST90001511	rs9836170	CD25hi CD45RA- (	3	169654216
ebi-a-GCST90001511	rs4955262	CD25hi CD45RA- (	3	32259389
ebi-a-GCST90001511	rs139048853	CD25hi CD45RA- (	4	69570859
ebi-a-GCST90001511	rs72818906	CD25hi CD45RA- (	5	180045604
ebi-a-GCST90001511	rs9268791	CD25hi CD45RA- (	6	32421073
ebi-a-GCST90001511	rs76420648	CD25hi CD45RA- (	8	24588282
ebi-a-GCST90001511	rs62518962	CD25hi CD45RA- (	8	138341531
ebi-a-GCST90001511	rs35825512	CD25hi CD45RA- (	9	20167602
ebi-a-GCST90001511	rs61839660	CD25hi CD45RA- (	10	6094697
ebi-a-GCST90001511	rs116928928	CD25hi CD45RA- (	11	32030595
ebi-a-GCST90001511	rs7105186	CD25hi CD45RA- (	11	122746450
ebi-a-GCST90001511	rs10785396	CD25hi CD45RA- (	12	43312063
ebi-a-GCST90001511	rs149406003	CD25hi CD45RA- (	13	101206935
ebi-a-GCST90001511	rs143283244	CD25hi CD45RA- (	15	94643151
ebi-a-GCST90001511	rs4238686	CD25hi CD45RA- (	16	88788934
ebi-a-GCST90001511	rs117614870	CD25hi CD45RA- (	17	63857170
ebi-a-GCST90001511	rs12458491	CD25hi CD45RA- (	18	35307820
ebi-a-GCST90001511	rs80266752	CD25hi CD45RA- (	20	52010540
ebi-a-GCST90001511	rs148871284	CD25hi CD45RA- (	20	15546558
ebi-a-GCST90001512	rs4497244	CD25hi CD45RA- (	1	197997953
ebi-a-GCST90001512	rs116794175	CD25hi CD45RA- (	1	194010208
ebi-a-GCST90001512	rs115815681	CD25hi CD45RA- (	1	149047683
ebi-a-GCST90001512	rs78193200	CD25hi CD45RA- (	1	149148053
ebi-a-GCST90001512	rs11579717	CD25hi CD45RA- (	1	199999121
ebi-a-GCST90001512	rs17666424	CD25hi CD45RA- (	1	159187530

ebi-a-GCST90001512	rs151238138	CD25hi CD45RA- (	2	171484062
ebi-a-GCST90001512	rs6771179	CD25hi CD45RA- (	3	14769598
ebi-a-GCST90001512	rs79327855	CD25hi CD45RA- (	3	144158111
ebi-a-GCST90001512	rs139048853	CD25hi CD45RA- (	4	69570859
ebi-a-GCST90001512	rs1558501	CD25hi CD45RA- (	4	55482148
ebi-a-GCST90001512	rs419099	CD25hi CD45RA- (	5	102089442
ebi-a-GCST90001512	rs12374668	CD25hi CD45RA- (	6	100229605
ebi-a-GCST90001512	rs6926235	CD25hi CD45RA- (	6	11130482
ebi-a-GCST90001512	rs6912413	CD25hi CD45RA- (	6	22960927
ebi-a-GCST90001512	rs4726924	CD25hi CD45RA- (	7	147873501
ebi-a-GCST90001512	rs183422804	CD25hi CD45RA- (	8	90678452
ebi-a-GCST90001512	rs7017671	CD25hi CD45RA- (	8	128467754
ebi-a-GCST90001512	rs2004605	CD25hi CD45RA- (	9	37391292
ebi-a-GCST90001512	rs12006014	CD25hi CD45RA- (	9	20160505
ebi-a-GCST90001512	rs61839660	CD25hi CD45RA- (	10	6094697
ebi-a-GCST90001512	rs116928928	CD25hi CD45RA- (	11	32030595
ebi-a-GCST90001512	rs149406003	CD25hi CD45RA- (	13	101206935
ebi-a-GCST90001512	rs143283244	CD25hi CD45RA- (	15	94643151
ebi-a-GCST90001512	rs76694846	CD25hi CD45RA- (	15	90197310
ebi-a-GCST90001512	rs12325554	CD25hi CD45RA- (	16	84053417
ebi-a-GCST90001512	rs12926701	CD25hi CD45RA- (	16	24473291
ebi-a-GCST90001512	rs117614870	CD25hi CD45RA- (	17	63857170
ebi-a-GCST90001512	rs12458491	CD25hi CD45RA- (	18	35307820
ebi-a-GCST90001512	rs2702579	CD25hi CD45RA- (	18	71659838
ebi-a-GCST90001512	rs148871284	CD25hi CD45RA- (	20	15546558
ebi-a-GCST90001512	rs6131700	CD25hi CD45RA- (	20	15742230
ebi-a-GCST90001512	rs67691565	CD25hi CD45RA- (	20	52169857
ebi-a-GCST90001512	rs147838148	CD25hi CD45RA- (	22	47814108
ebi-a-GCST90001513	rs76202906	CD4 Treg AC	1	113476831
ebi-a-GCST90001513	rs78890192	CD4 Treg AC	4	107059453
ebi-a-GCST90001513	rs77040981	CD4 Treg AC	4	184321385
ebi-a-GCST90001513	rs1268153	CD4 Treg AC	6	109035696
ebi-a-GCST90001513	rs1028334	CD4 Treg AC	6	18584683
ebi-a-GCST90001513	rs9343914	CD4 Treg AC	6	80166444
ebi-a-GCST90001513	rs17717645	CD4 Treg AC	7	18003774
ebi-a-GCST90001513	rs11772999	CD4 Treg AC	7	113991516
ebi-a-GCST90001513	rs140960913	CD4 Treg AC	7	158180337
ebi-a-GCST90001513	rs140842863	CD4 Treg AC	8	35828286
ebi-a-GCST90001513	rs61839660	CD4 Treg AC	10	6094697
ebi-a-GCST90001513	rs78409263	CD4 Treg AC	11	79202514
ebi-a-GCST90001513	rs75596315	CD4 Treg AC	12	122520112
ebi-a-GCST90001513	rs3184504	CD4 Treg AC	12	111884608
ebi-a-GCST90001513	rs145600407	CD4 Treg AC	13	19280601
ebi-a-GCST90001513	rs78334049	CD4 Treg AC	14	93385046
ebi-a-GCST90001513	rs7162344	CD4 Treg AC	15	26030485
ebi-a-GCST90001513	rs188505795	CD4 Treg AC	16	28223373
ebi-a-GCST90001513	rs149951966	CD4 Treg AC	17	41137136
ebi-a-GCST90001513	rs72833913	CD4 Treg AC	17	5713335

ebi-a-GCST90001513	rs79684619	CD4 Treg AC	18	64907349
ebi-a-GCST90001513	rs9709738	CD4 Treg AC	18	60137303
ebi-a-GCST90001514	rs80237985	HSC AC	1	217396264
ebi-a-GCST90001514	rs76038700	HSC AC	2	135226197
ebi-a-GCST90001514	rs1432243	HSC AC	2	137773206
ebi-a-GCST90001514	rs115937242	HSC AC	2	136121922
ebi-a-GCST90001514	rs74529033	HSC AC	2	137747326
ebi-a-GCST90001514	rs112258784	HSC AC	2	191015741
ebi-a-GCST90001514	rs2887466	HSC AC	3	131748507
ebi-a-GCST90001514	rs10080768	HSC AC	6	2760245
ebi-a-GCST90001514	rs142114110	HSC AC	7	82240518
ebi-a-GCST90001514	rs77538571	HSC AC	8	4006968
ebi-a-GCST90001514	rs34526926	HSC AC	8	78599881
ebi-a-GCST90001514	rs143685973	HSC AC	9	136023292
ebi-a-GCST90001514	rs10809987	HSC AC	9	13585363
ebi-a-GCST90001514	rs2724803	HSC AC	10	12404560
ebi-a-GCST90001514	rs115076910	HSC AC	11	121009383
ebi-a-GCST90001514	rs2709155	HSC AC	11	4597980
ebi-a-GCST90001514	rs77058454	HSC AC	15	81301816
ebi-a-GCST90001514	rs6416872	HSC AC	17	2750333
ebi-a-GCST90001514	rs466736	HSC AC	21	20337514
ebi-a-GCST90001515	rs74227709	Im MDSC AC	1	247722588
ebi-a-GCST90001515	rs6682502	Im MDSC AC	1	234866398
ebi-a-GCST90001515	rs347282	Im MDSC AC	1	162312753
ebi-a-GCST90001515	rs423524	Im MDSC AC	2	147899621
ebi-a-GCST90001515	rs113696385	Im MDSC AC	2	176971968
ebi-a-GCST90001515	rs9829934	Im MDSC AC	3	113575708
ebi-a-GCST90001515	rs143216098	Im MDSC AC	3	14039647
ebi-a-GCST90001515	rs329820	Im MDSC AC	3	78947955
ebi-a-GCST90001515	rs76522245	Im MDSC AC	4	182229502
ebi-a-GCST90001515	rs2451361	Im MDSC AC	6	34029204
ebi-a-GCST90001515	rs1421603	Im MDSC AC	7	131327890
ebi-a-GCST90001515	rs72841673	Im MDSC AC	10	125501892
ebi-a-GCST90001515	rs56236288	Im MDSC AC	10	11698793
ebi-a-GCST90001515	rs2420243	Im MDSC AC	10	117921053
ebi-a-GCST90001515	rs10843345	Im MDSC AC	12	29371578
ebi-a-GCST90001515	rs144437325	Im MDSC AC	13	52130641
ebi-a-GCST90001515	rs141792521	Im MDSC AC	16	58686745
ebi-a-GCST90001515	rs7259307	Im MDSC AC	19	51540750
ebi-a-GCST90001515	rs17801681	Im MDSC AC	19	51676649
ebi-a-GCST90001515	rs2455069	Im MDSC AC	19	51728641
ebi-a-GCST90001515	rs1654530	Im MDSC AC	19	51476281
ebi-a-GCST90001515	rs4499342	Im MDSC AC	19	8786913
ebi-a-GCST90001515	rs1546826	Im MDSC AC	21	19549947
ebi-a-GCST90001515	rs76978157	Im MDSC AC	21	47549410
ebi-a-GCST90001516	rs12094489	Im MDSC %CD33d	1	247286936
ebi-a-GCST90001516	rs72923436	Im MDSC %CD33d	2	176970247
ebi-a-GCST90001516	rs9829934	Im MDSC %CD33d	3	113575708

ebi-a-GCST90001516	rs469318	Im MDSC %CD33c	5	179340006
ebi-a-GCST90001516	rs79899973	Im MDSC %CD33c	6	10417917
ebi-a-GCST90001516	rs115076391	Im MDSC %CD33c	6	873131
ebi-a-GCST90001516	rs182908654	Im MDSC %CD33c	7	141084101
ebi-a-GCST90001516	rs41928	Im MDSC %CD33c	7	117925703
ebi-a-GCST90001516	rs147302158	Im MDSC %CD33c	9	32093300
ebi-a-GCST90001516	rs12350919	Im MDSC %CD33c	9	129479345
ebi-a-GCST90001516	rs139228307	Im MDSC %CD33c	10	49759225
ebi-a-GCST90001516	rs7907229	Im MDSC %CD33c	10	10423376
ebi-a-GCST90001516	rs2420243	Im MDSC %CD33c	10	117921053
ebi-a-GCST90001516	rs3829176	Im MDSC %CD33c	10	127752441
ebi-a-GCST90001516	rs12878160	Im MDSC %CD33c	14	93613399
ebi-a-GCST90001516	rs12443996	Im MDSC %CD33c	16	74210489
ebi-a-GCST90001516	rs4499342	Im MDSC %CD33c	19	8786913
ebi-a-GCST90001516	rs1701958	Im MDSC %CD33c	19	51476715
ebi-a-GCST90001516	rs151124788	Im MDSC %CD33c	19	47622471
ebi-a-GCST90001516	rs2455069	Im MDSC %CD33c	19	51728641
ebi-a-GCST90001516	rs1537122	Im MDSC %CD33c	21	46598506
ebi-a-GCST90001516	rs12482767	Im MDSC %CD33c	21	47428075
ebi-a-GCST90001517	rs2703989	CD33br HLA DR+	1	90495703
ebi-a-GCST90001517	rs11161969	CD33br HLA DR+	1	87752464
ebi-a-GCST90001517	rs5020267	CD33br HLA DR+	2	147077389
ebi-a-GCST90001517	rs150886675	CD33br HLA DR+	2	227300526
ebi-a-GCST90001517	rs3770997	CD33br HLA DR+	2	206649338
ebi-a-GCST90001517	rs143438920	CD33br HLA DR+	3	15416436
ebi-a-GCST90001517	rs115418145	CD33br HLA DR+	4	130012204
ebi-a-GCST90001517	rs73248200	CD33br HLA DR+	4	27723538
ebi-a-GCST90001517	rs505769	CD33br HLA DR+	5	29036863
ebi-a-GCST90001517	rs1902950	CD33br HLA DR+	5	162397650
ebi-a-GCST90001517	rs141849324	CD33br HLA DR+	5	133807831
ebi-a-GCST90001517	rs77906933	CD33br HLA DR+	6	125326624
ebi-a-GCST90001517	rs183489799	CD33br HLA DR+	6	135523741
ebi-a-GCST90001517	rs34908874	CD33br HLA DR+	6	19571542
ebi-a-GCST90001517	rs142786778	CD33br HLA DR+	6	23419953
ebi-a-GCST90001517	rs1647900	CD33br HLA DR+	7	134917158
ebi-a-GCST90001517	rs182908654	CD33br HLA DR+	7	141084101
ebi-a-GCST90001517	rs11777538	CD33br HLA DR+	8	127623130
ebi-a-GCST90001517	rs182483660	CD33br HLA DR+	10	90000206
ebi-a-GCST90001517	rs10740011	CD33br HLA DR+	10	61890937
ebi-a-GCST90001517	rs143612223	CD33br HLA DR+	10	112429560
ebi-a-GCST90001517	rs61916462	CD33br HLA DR+	11	101619255
ebi-a-GCST90001517	rs12297491	CD33br HLA DR+	12	1731406
ebi-a-GCST90001517	rs4020134	CD33br HLA DR+	14	78420741
ebi-a-GCST90001517	rs117019089	CD33br HLA DR+	16	69342268
ebi-a-GCST90001517	rs35948572	CD33br HLA DR+	16	5666157
ebi-a-GCST90001517	rs13334605	CD33br HLA DR+	16	8615510
ebi-a-GCST90001517	rs111715212	CD33br HLA DR+	18	5327059
ebi-a-GCST90001517	rs74253876	CD33br HLA DR+	18	5858361

ebi-a-GCST90001517	rs117285079	CD33br HLA DR+	18	4762780
ebi-a-GCST90001517	rs3865444	CD33br HLA DR+	19	51727962
ebi-a-GCST90001518	rs144410606	CD33br HLA DR+	1	187381911
ebi-a-GCST90001518	rs2703989	CD33br HLA DR+	1	90495703
ebi-a-GCST90001518	rs17046269	CD33br HLA DR+	2	54973519
ebi-a-GCST90001518	rs150886675	CD33br HLA DR+	2	227300526
ebi-a-GCST90001518	rs5020267	CD33br HLA DR+	2	147077389
ebi-a-GCST90001518	rs9990730	CD33br HLA DR+	4	183610911
ebi-a-GCST90001518	rs6858113	CD33br HLA DR+	4	27713713
ebi-a-GCST90001518	rs115418145	CD33br HLA DR+	4	130012204
ebi-a-GCST90001518	rs505806	CD33br HLA DR+	5	29036851
ebi-a-GCST90001518	rs1902950	CD33br HLA DR+	5	162397650
ebi-a-GCST90001518	rs77906933	CD33br HLA DR+	6	125326624
ebi-a-GCST90001518	rs34908874	CD33br HLA DR+	6	19571542
ebi-a-GCST90001518	rs182908654	CD33br HLA DR+	7	141084101
ebi-a-GCST90001518	rs1647900	CD33br HLA DR+	7	134917158
ebi-a-GCST90001518	rs4733718	CD33br HLA DR+	8	130647103
ebi-a-GCST90001518	rs138272175	CD33br HLA DR+	9	945397
ebi-a-GCST90001518	rs10740011	CD33br HLA DR+	10	61890937
ebi-a-GCST90001518	rs35248101	CD33br HLA DR+	10	13171617
ebi-a-GCST90001518	rs117032925	CD33br HLA DR+	10	103744374
ebi-a-GCST90001518	rs75873877	CD33br HLA DR+	10	89722783
ebi-a-GCST90001518	rs143612223	CD33br HLA DR+	10	112429560
ebi-a-GCST90001518	rs61916496	CD33br HLA DR+	11	101684475
ebi-a-GCST90001518	rs144437325	CD33br HLA DR+	13	52130641
ebi-a-GCST90001518	rs4020134	CD33br HLA DR+	14	78420741
ebi-a-GCST90001518	rs4357922	CD33br HLA DR+	15	97966655
ebi-a-GCST90001518	rs57812633	CD33br HLA DR+	16	5690721
ebi-a-GCST90001518	rs13334605	CD33br HLA DR+	16	8615510
ebi-a-GCST90001518	rs117019089	CD33br HLA DR+	16	69342268
ebi-a-GCST90001518	rs117306448	CD33br HLA DR+	17	68540131
ebi-a-GCST90001518	rs16988845	CD33br HLA DR+	19	51549527
ebi-a-GCST90001518	rs3865444	CD33br HLA DR+	19	51727962
ebi-a-GCST90001519	rs10494422	CD33br HLA DR+	1	164781015
ebi-a-GCST90001519	rs10799466	CD33br HLA DR+	1	228317685
ebi-a-GCST90001519	rs12033128	CD33br HLA DR+	1	85552939
ebi-a-GCST90001519	rs16865573	CD33br HLA DR+	2	6895049
ebi-a-GCST90001519	rs116534482	CD33br HLA DR+	2	142334140
ebi-a-GCST90001519	rs218773	CD33br HLA DR+	4	14437080
ebi-a-GCST90001519	rs115205981	CD33br HLA DR+	4	36025181
ebi-a-GCST90001519	rs6844114	CD33br HLA DR+	4	190745892
ebi-a-GCST90001519	rs62350505	CD33br HLA DR+	4	165304812
ebi-a-GCST90001519	rs2501720	CD33br HLA DR+	6	90899561
ebi-a-GCST90001519	rs11982860	CD33br HLA DR+	7	67501241
ebi-a-GCST90001519	rs142412842	CD33br HLA DR+	7	140274216
ebi-a-GCST90001519	rs111391334	CD33br HLA DR+	9	83651275
ebi-a-GCST90001519	rs7116432	CD33br HLA DR+	11	35244058
ebi-a-GCST90001519	rs2529690	CD33br HLA DR+	17	12239004

ebi-a-GCST90001519	rs113548770	CD33br HLA DR+	18	70222212
ebi-a-GCST90001519	rs7245846	CD33br HLA DR+	19	51731176
ebi-a-GCST90001519	rs111695695	CD33br HLA DR+	19	51897760
ebi-a-GCST90001519	rs62197298	CD33br HLA DR+	20	61646108
ebi-a-GCST90001520	rs115048709	CD33br HLA DR+	1	87894430
ebi-a-GCST90001520	rs5020267	CD33br HLA DR+	2	147077389
ebi-a-GCST90001520	rs3770997	CD33br HLA DR+	2	206649338
ebi-a-GCST90001520	rs76694877	CD33br HLA DR+	3	27966223
ebi-a-GCST90001520	rs112790132	CD33br HLA DR+	3	115423600
ebi-a-GCST90001520	rs75779179	CD33br HLA DR+	3	14581493
ebi-a-GCST90001520	rs139406769	CD33br HLA DR+	3	178041850
ebi-a-GCST90001520	rs56151283	CD33br HLA DR+	3	150895562
ebi-a-GCST90001520	rs142206516	CD33br HLA DR+	4	130010719
ebi-a-GCST90001520	rs1865405	CD33br HLA DR+	5	136614606
ebi-a-GCST90001520	rs142786778	CD33br HLA DR+	6	23419953
ebi-a-GCST90001520	rs187876923	CD33br HLA DR+	6	125217867
ebi-a-GCST90001520	rs55652412	CD33br HLA DR+	6	135488750
ebi-a-GCST90001520	rs145382040	CD33br HLA DR+	6	25721374
ebi-a-GCST90001520	rs182908654	CD33br HLA DR+	7	141084101
ebi-a-GCST90001520	rs10112702	CD33br HLA DR+	8	127626066
ebi-a-GCST90001520	rs143612223	CD33br HLA DR+	10	112429560
ebi-a-GCST90001520	rs7074380	CD33br HLA DR+	10	43466121
ebi-a-GCST90001520	rs2695824	CD33br HLA DR+	11	131554552
ebi-a-GCST90001520	rs9548748	CD33br HLA DR+	13	39950178
ebi-a-GCST90001520	rs11618675	CD33br HLA DR+	13	49307675
ebi-a-GCST90001520	rs7180375	CD33br HLA DR+	15	79157379
ebi-a-GCST90001520	rs187512831	CD33br HLA DR+	16	12806949
ebi-a-GCST90001520	rs74253876	CD33br HLA DR+	18	5858361
ebi-a-GCST90001520	rs117285079	CD33br HLA DR+	18	4762780
ebi-a-GCST90001520	rs113771439	CD33br HLA DR+	18	5326964
ebi-a-GCST90001520	rs3865444	CD33br HLA DR+	19	51727962
ebi-a-GCST90001520	rs73039451	CD33br HLA DR+	19	33617508
ebi-a-GCST90001520	rs77594041	CD33br HLA DR+	20	20080535
ebi-a-GCST90001521	rs17262293	CD33br HLA DR+	1	29062427
ebi-a-GCST90001521	rs187359230	CD33br HLA DR+	2	35143865
ebi-a-GCST90001521	rs6544660	CD33br HLA DR+	2	43688496
ebi-a-GCST90001521	rs6720231	CD33br HLA DR+	2	158761398
ebi-a-GCST90001521	rs76092047	CD33br HLA DR+	2	236068499
ebi-a-GCST90001521	rs182987179	CD33br HLA DR+	3	41352496
ebi-a-GCST90001521	rs61797471	CD33br HLA DR+	4	36058097
ebi-a-GCST90001521	rs115038761	CD33br HLA DR+	4	136462637
ebi-a-GCST90001521	rs6844114	CD33br HLA DR+	4	190745892
ebi-a-GCST90001521	rs12655500	CD33br HLA DR+	5	163124522
ebi-a-GCST90001521	rs111231787	CD33br HLA DR+	5	112408670
ebi-a-GCST90001521	rs2982905	CD33br HLA DR+	6	162155632
ebi-a-GCST90001521	rs1504215	CD33br HLA DR+	6	91006227
ebi-a-GCST90001521	rs6951925	CD33br HLA DR+	7	108801624
ebi-a-GCST90001521	rs625405	CD33br HLA DR+	7	4184036

ebi-a-GCST90001521	rs138834110	CD33br HLA DR+	8	5338183
ebi-a-GCST90001521	rs2811828	CD33br HLA DR+	9	17952241
ebi-a-GCST90001521	rs480590	CD33br HLA DR+	11	117356979
ebi-a-GCST90001521	rs72719861	CD33br HLA DR+	14	72634959
ebi-a-GCST90001521	rs12933012	CD33br HLA DR+	16	81880726
ebi-a-GCST90001521	rs9912578	CD33br HLA DR+	17	70578744
ebi-a-GCST90001521	rs117734358	CD33br HLA DR+	18	13088666
ebi-a-GCST90001521	rs12459419	CD33br HLA DR+	19	51728477
ebi-a-GCST90001521	rs73030477	CD33br HLA DR+	19	28098310
ebi-a-GCST90001521	rs10409679	CD33br HLA DR+	19	50983930
ebi-a-GCST90001521	rs740867	CD33br HLA DR+	19	57195149
ebi-a-GCST90001522	rs59118111	CD33- HLA DR- A	1	4044791
ebi-a-GCST90001522	rs930273	CD33- HLA DR- A	3	10659768
ebi-a-GCST90001522	rs17644026	CD33- HLA DR- A	4	53828606
ebi-a-GCST90001522	rs145308006	CD33- HLA DR- A	6	103221111
ebi-a-GCST90001522	rs72826499	CD33- HLA DR- A	6	19014908
ebi-a-GCST90001522	rs6918752	CD33- HLA DR- A	6	139658866
ebi-a-GCST90001522	rs2893591	CD33- HLA DR- A	7	105343363
ebi-a-GCST90001522	rs144148395	CD33- HLA DR- A	10	38761057
ebi-a-GCST90001522	rs7079561	CD33- HLA DR- A	10	128153755
ebi-a-GCST90001522	rs12276131	CD33- HLA DR- A	11	4839164
ebi-a-GCST90001522	rs55867246	CD33- HLA DR- A	14	54236212
ebi-a-GCST90001522	rs145397048	CD33- HLA DR- A	14	101739270
ebi-a-GCST90001522	rs67418059	CD33- HLA DR- A	15	65664499
ebi-a-GCST90001522	rs77571541	CD33- HLA DR- A	16	84165036
ebi-a-GCST90001522	rs9916257	CD33- HLA DR- A	17	33797371
ebi-a-GCST90001522	rs150206406	CD33- HLA DR- A	18	35502118
ebi-a-GCST90001522	rs117195035	CD33- HLA DR- A	18	72338465
ebi-a-GCST90001522	rs145540967	CD33- HLA DR- A	19	31193989
ebi-a-GCST90001522	rs142340400	CD33- HLA DR- A	19	3958937
ebi-a-GCST90001522	rs1205315	CD33- HLA DR- A	19	54838294
ebi-a-GCST90001522	rs116851051	CD33- HLA DR- A	20	15544052
ebi-a-GCST90001523	rs144484750	CD33- HLA DR+ A	1	175194000
ebi-a-GCST90001523	rs372474797	CD33- HLA DR+ A	2	90197043
ebi-a-GCST90001523	rs11695642	CD33- HLA DR+ A	2	43952695
ebi-a-GCST90001523	rs17382372	CD33- HLA DR+ A	2	136921761
ebi-a-GCST90001523	rs74940075	CD33- HLA DR+ A	3	105338298
ebi-a-GCST90001523	rs4955865	CD33- HLA DR+ A	3	55733390
ebi-a-GCST90001523	rs114548614	CD33- HLA DR+ A	3	196493486
ebi-a-GCST90001523	rs66733222	CD33- HLA DR+ A	3	171668815
ebi-a-GCST90001523	rs78557978	CD33- HLA DR+ A	4	115638601
ebi-a-GCST90001523	rs6841247	CD33- HLA DR+ A	4	31899436
ebi-a-GCST90001523	rs149494397	CD33- HLA DR+ A	6	46669724
ebi-a-GCST90001523	rs149034028	CD33- HLA DR+ A	6	138310176
ebi-a-GCST90001523	rs115139705	CD33- HLA DR+ A	7	120959087
ebi-a-GCST90001523	rs118141414	CD33- HLA DR+ A	7	141885631
ebi-a-GCST90001523	rs876036	CD33- HLA DR+ A	7	50307710
ebi-a-GCST90001523	rs2705609	CD33- HLA DR+ A	7	3560919

ebi-a-GCST90001523	rs117757905	CD33- HLA DR+ A	9	20121195
ebi-a-GCST90001523	rs140563508	CD33- HLA DR+ A	9	140249209
ebi-a-GCST90001523	rs185521042	CD33- HLA DR+ A	10	81460339
ebi-a-GCST90001523	rs139348198	CD33- HLA DR+ A	11	14699945
ebi-a-GCST90001523	rs73023658	CD33- HLA DR+ A	11	130596271
ebi-a-GCST90001523	rs145935372	CD33- HLA DR+ A	12	61820975
ebi-a-GCST90001523	rs2464634	CD33- HLA DR+ A	12	120316206
ebi-a-GCST90001523	rs3916927	CD33- HLA DR+ A	13	103927135
ebi-a-GCST90001523	rs143360411	CD33- HLA DR+ A	14	60625341
ebi-a-GCST90001523	rs117803686	CD33- HLA DR+ A	15	25457565
ebi-a-GCST90001523	rs118076832	CD33- HLA DR+ A	15	52532599
ebi-a-GCST90001523	rs17883048	CD33- HLA DR+ A	17	7570956
ebi-a-GCST90001523	rs117117531	CD33- HLA DR+ A	17	50899429
ebi-a-GCST90001523	rs2108605	CD33- HLA DR+ A	17	10352579
ebi-a-GCST90001523	rs116926801	CD33- HLA DR+ A	17	65652250
ebi-a-GCST90001523	rs7207960	CD33- HLA DR+ A	17	32010983
ebi-a-GCST90001523	rs73049161	CD33- HLA DR+ A	19	41969867
ebi-a-GCST90001523	rs140072356	CD33- HLA DR+ A	19	41677568
ebi-a-GCST90001523	rs35720624	CD33- HLA DR+ A	19	53014851
ebi-a-GCST90001523	rs1697573	CD33- HLA DR+ A	19	51718317
ebi-a-GCST90001523	rs34318562	CD33- HLA DR+ A	19	52180033
ebi-a-GCST90001523	rs6074118	CD33- HLA DR+ A	20	10283445
ebi-a-GCST90001524	rs2070901	Gr MDSC AC	1	161185058
ebi-a-GCST90001524	rs6710092	Gr MDSC AC	2	166018627
ebi-a-GCST90001524	rs2017989	Gr MDSC AC	2	242099961
ebi-a-GCST90001524	rs111636975	Gr MDSC AC	2	176606506
ebi-a-GCST90001524	rs1434218	Gr MDSC AC	2	79555347
ebi-a-GCST90001524	rs114244809	Gr MDSC AC	2	5834493
ebi-a-GCST90001524	rs2351156	Gr MDSC AC	2	50325488
ebi-a-GCST90001524	rs75747211	Gr MDSC AC	3	48261366
ebi-a-GCST90001524	rs114469087	Gr MDSC AC	4	90493819
ebi-a-GCST90001524	rs1018969	Gr MDSC AC	4	5468752
ebi-a-GCST90001524	rs114772249	Gr MDSC AC	5	54008468
ebi-a-GCST90001524	rs10872783	Gr MDSC AC	6	96696878
ebi-a-GCST90001524	rs140075808	Gr MDSC AC	7	115503233
ebi-a-GCST90001524	rs72845259	Gr MDSC AC	10	132711992
ebi-a-GCST90001524	rs2468595	Gr MDSC AC	11	23114913
ebi-a-GCST90001524	rs12588021	Gr MDSC AC	14	72955384
ebi-a-GCST90001524	rs78384212	Gr MDSC AC	14	60291874
ebi-a-GCST90001524	rs8098513	Gr MDSC AC	18	67052180
ebi-a-GCST90001524	rs655539	Gr MDSC AC	18	54281790
ebi-a-GCST90001524	rs3865444	Gr MDSC AC	19	51727962
ebi-a-GCST90001524	rs16988872	Gr MDSC AC	19	51580161
ebi-a-GCST90001525	rs1801274	CD33dim HLA DR	1	161479745
ebi-a-GCST90001525	rs148937000	CD33dim HLA DR	1	94437964
ebi-a-GCST90001525	rs4606283	CD33dim HLA DR	1	227581017
ebi-a-GCST90001525	rs62146115	CD33dim HLA DR	2	95456961
ebi-a-GCST90001525	rs2868992	CD33dim HLA DR	2	56232555

ebi-a-GCST90001525	rs143383466	CD33dim HLA DR	3	196068066
ebi-a-GCST90001525	rs11709025	CD33dim HLA DR	3	177439366
ebi-a-GCST90001525	rs71596080	CD33dim HLA DR	4	82676237
ebi-a-GCST90001525	rs10025005	CD33dim HLA DR	4	181288587
ebi-a-GCST90001525	rs10011663	CD33dim HLA DR	4	190338080
ebi-a-GCST90001525	rs78173101	CD33dim HLA DR	5	148830537
ebi-a-GCST90001525	rs62362950	CD33dim HLA DR	5	83003910
ebi-a-GCST90001525	rs74851637	CD33dim HLA DR	5	144893255
ebi-a-GCST90001525	rs17721845	CD33dim HLA DR	6	149234718
ebi-a-GCST90001525	rs73716755	CD33dim HLA DR	7	116308013
ebi-a-GCST90001525	rs144894656	CD33dim HLA DR	9	118222268
ebi-a-GCST90001525	rs11594395	CD33dim HLA DR	10	2817998
ebi-a-GCST90001525	rs192715019	CD33dim HLA DR	12	83087530
ebi-a-GCST90001525	rs74638799	CD33dim HLA DR	12	11652069
ebi-a-GCST90001525	rs9524403	CD33dim HLA DR	13	94940936
ebi-a-GCST90001525	rs9510371	CD33dim HLA DR	13	23410009
ebi-a-GCST90001525	rs141080550	CD33dim HLA DR	13	111287945
ebi-a-GCST90001525	rs11631677	CD33dim HLA DR	15	86647805
ebi-a-GCST90001525	rs58051708	CD33dim HLA DR	17	72998954
ebi-a-GCST90001525	rs145231100	CD33dim HLA DR	18	33141890
ebi-a-GCST90001525	rs1201940	CD33dim HLA DR	20	59281507
ebi-a-GCST90001525	rs76640602	CD33dim HLA DR	22	39737217
ebi-a-GCST90001526	rs3820097	CD33dim HLA DR	1	161049358
ebi-a-GCST90001526	rs1801274	CD33dim HLA DR	1	161479745
ebi-a-GCST90001526	rs55720217	CD33dim HLA DR	1	28277072
ebi-a-GCST90001526	rs35006063	CD33dim HLA DR	1	105261254
ebi-a-GCST90001526	rs75239369	CD33dim HLA DR	2	180132004
ebi-a-GCST90001526	rs139393711	CD33dim HLA DR	3	113287778
ebi-a-GCST90001526	rs78096163	CD33dim HLA DR	3	49663488
ebi-a-GCST90001526	rs263715	CD33dim HLA DR	3	6504785
ebi-a-GCST90001526	rs6820873	CD33dim HLA DR	4	31077545
ebi-a-GCST90001526	rs115483539	CD33dim HLA DR	5	50273359
ebi-a-GCST90001526	rs7723240	CD33dim HLA DR	5	82990740
ebi-a-GCST90001526	rs35152411	CD33dim HLA DR	5	125675454
ebi-a-GCST90001526	rs7754966	CD33dim HLA DR	6	151449284
ebi-a-GCST90001526	rs9260301	CD33dim HLA DR	6	29916465
ebi-a-GCST90001526	rs73203458	CD33dim HLA DR	7	112318350
ebi-a-GCST90001526	rs79573082	CD33dim HLA DR	8	20155237
ebi-a-GCST90001526	rs111944709	CD33dim HLA DR	8	4886462
ebi-a-GCST90001526	rs4641163	CD33dim HLA DR	9	130194536
ebi-a-GCST90001526	rs78671194	CD33dim HLA DR	10	73360670
ebi-a-GCST90001526	rs79027682	CD33dim HLA DR	10	30519550
ebi-a-GCST90001526	rs12306859	CD33dim HLA DR	12	52497538
ebi-a-GCST90001526	rs117713744	CD33dim HLA DR	13	39990754
ebi-a-GCST90001526	rs7156568	CD33dim HLA DR	14	102533672
ebi-a-GCST90001526	rs3826007	CD33dim HLA DR	15	80263217
ebi-a-GCST90001526	rs6105661	CD33dim HLA DR	20	16785806
ebi-a-GCST90001526	rs2097380	CD33dim HLA DR	22	49658809

ebi-a-GCST90001527	rs72762130	CD33dim HLA DR	1	237056710
ebi-a-GCST90001527	rs1801274	CD33dim HLA DR	1	161479745
ebi-a-GCST90001527	rs116772525	CD33dim HLA DR	1	184082076
ebi-a-GCST90001527	rs116600349	CD33dim HLA DR	1	7584588
ebi-a-GCST90001527	rs72702556	CD33dim HLA DR	1	150680115
ebi-a-GCST90001527	rs10921381	CD33dim HLA DR	1	193500034
ebi-a-GCST90001527	rs62172936	CD33dim HLA DR	2	154682039
ebi-a-GCST90001527	rs1427642	CD33dim HLA DR	2	82739796
ebi-a-GCST90001527	rs77839823	CD33dim HLA DR	2	117657692
ebi-a-GCST90001527	rs1377398	CD33dim HLA DR	2	19918761
ebi-a-GCST90001527	rs115342053	CD33dim HLA DR	2	178319837
ebi-a-GCST90001527	rs79141836	CD33dim HLA DR	2	99513380
ebi-a-GCST90001527	rs113181189	CD33dim HLA DR	3	2089038
ebi-a-GCST90001527	rs4467644	CD33dim HLA DR	4	115266184
ebi-a-GCST90001527	rs139262012	CD33dim HLA DR	5	99798403
ebi-a-GCST90001527	rs34228085	CD33dim HLA DR	7	144809718
ebi-a-GCST90001527	rs76259509	CD33dim HLA DR	7	86296884
ebi-a-GCST90001527	rs34574947	CD33dim HLA DR	7	18953422
ebi-a-GCST90001527	rs185048341	CD33dim HLA DR	9	107953420
ebi-a-GCST90001527	rs17658872	CD33dim HLA DR	10	74886544
ebi-a-GCST90001527	rs80075776	CD33dim HLA DR	13	70565458
ebi-a-GCST90001527	rs11616439	CD33dim HLA DR	13	102675461
ebi-a-GCST90001527	rs34476866	CD33dim HLA DR	14	37002449
ebi-a-GCST90001527	rs61987643	CD33dim HLA DR	14	54058233
ebi-a-GCST90001527	rs67760360	CD33dim HLA DR	15	80260872
ebi-a-GCST90001527	rs184208453	CD33dim HLA DR	17	62996369
ebi-a-GCST90001527	rs12965906	CD33dim HLA DR	18	49061830
ebi-a-GCST90001527	rs112332228	CD33dim HLA DR	18	36935582
ebi-a-GCST90001527	rs1201943	CD33dim HLA DR	20	59282409
ebi-a-GCST90001528	rs3820097	CD33dim HLA DR	1	161049358
ebi-a-GCST90001528	rs35006063	CD33dim HLA DR	1	105261254
ebi-a-GCST90001528	rs1801274	CD33dim HLA DR	1	161479745
ebi-a-GCST90001528	rs75239369	CD33dim HLA DR	2	180132004
ebi-a-GCST90001528	rs263715	CD33dim HLA DR	3	6504785
ebi-a-GCST90001528	rs139393711	CD33dim HLA DR	3	113287778
ebi-a-GCST90001528	rs6820873	CD33dim HLA DR	4	31077545
ebi-a-GCST90001528	rs115483539	CD33dim HLA DR	5	50273359
ebi-a-GCST90001528	rs114746765	CD33dim HLA DR	5	125978376
ebi-a-GCST90001528	rs942382	CD33dim HLA DR	6	151472251
ebi-a-GCST90001528	rs112808189	CD33dim HLA DR	6	159180712
ebi-a-GCST90001528	rs73203461	CD33dim HLA DR	7	112321582
ebi-a-GCST90001528	rs117229031	CD33dim HLA DR	7	84944448
ebi-a-GCST90001528	rs79573082	CD33dim HLA DR	8	20155237
ebi-a-GCST90001528	rs111944709	CD33dim HLA DR	8	4886462
ebi-a-GCST90001528	rs112330535	CD33dim HLA DR	8	97415144
ebi-a-GCST90001528	rs11789887	CD33dim HLA DR	9	130168840
ebi-a-GCST90001528	rs79027682	CD33dim HLA DR	10	30519550
ebi-a-GCST90001528	rs78671194	CD33dim HLA DR	10	73360670

ebi-a-GCST90001528	rs72857552	CD33dim HLA DR	11	11239846
ebi-a-GCST90001528	rs7156568	CD33dim HLA DR	14	102533672
ebi-a-GCST90001528	rs3826007	CD33dim HLA DR	15	80263217
ebi-a-GCST90001528	rs17443769	CD33dim HLA DR	16	9531278
ebi-a-GCST90001528	rs6105661	CD33dim HLA DR	20	16785806
ebi-a-GCST90001528	rs2097380	CD33dim HLA DR	22	49658809
ebi-a-GCST90001529	rs12239179	CD66b++ myeloid	1	198976492
ebi-a-GCST90001529	rs2070901	CD66b++ myeloid	1	161185058
ebi-a-GCST90001529	rs10803923	CD66b++ myeloid	2	179931060
ebi-a-GCST90001529	rs59277927	CD66b++ myeloid	2	223074399
ebi-a-GCST90001529	rs2630166	CD66b++ myeloid	3	60042425
ebi-a-GCST90001529	rs116795543	CD66b++ myeloid	3	188082404
ebi-a-GCST90001529	rs114469087	CD66b++ myeloid	4	90493819
ebi-a-GCST90001529	rs1047483	CD66b++ myeloid	5	52249612
ebi-a-GCST90001529	rs71536865	CD66b++ myeloid	7	11873259
ebi-a-GCST90001529	rs72845259	CD66b++ myeloid	10	132711992
ebi-a-GCST90001529	rs11218704	CD66b++ myeloid	11	122458787
ebi-a-GCST90001529	rs11114486	CD66b++ myeloid	12	80899901
ebi-a-GCST90001529	rs1899009	CD66b++ myeloid	12	74096868
ebi-a-GCST90001529	rs78384212	CD66b++ myeloid	14	60291874
ebi-a-GCST90001529	rs2911266	CD66b++ myeloid	16	89509001
ebi-a-GCST90001529	rs111580523	CD66b++ myeloid	18	57183504
ebi-a-GCST90001529	rs10419309	CD66b++ myeloid	19	52138563
ebi-a-GCST90001529	rs16979810	CD66b++ myeloid	20	18935780
ebi-a-GCST90001529	rs148676149	CD66b++ myeloid	22	47075772
ebi-a-GCST90001530	rs12736179	Mo MDSC AC	1	20887141
ebi-a-GCST90001530	rs12082268	Mo MDSC AC	1	217288095
ebi-a-GCST90001530	rs10458377	Mo MDSC AC	1	226154277
ebi-a-GCST90001530	rs10176753	Mo MDSC AC	2	133046981
ebi-a-GCST90001530	rs147643522	Mo MDSC AC	3	185678332
ebi-a-GCST90001530	rs9271768	Mo MDSC AC	6	32594188
ebi-a-GCST90001530	rs9270591	Mo MDSC AC	6	32561424
ebi-a-GCST90001530	rs2071473	Mo MDSC AC	6	32782605
ebi-a-GCST90001530	rs80032720	Mo MDSC AC	6	32384933
ebi-a-GCST90001530	rs7757733	Mo MDSC AC	6	5580339
ebi-a-GCST90001530	rs112611150	Mo MDSC AC	7	186897
ebi-a-GCST90001530	rs9792404	Mo MDSC AC	9	8429048
ebi-a-GCST90001530	rs11112649	Mo MDSC AC	12	106064969
ebi-a-GCST90001530	rs1800973	Mo MDSC AC	12	69744014
ebi-a-GCST90001530	rs10082958	Mo MDSC AC	12	89653407
ebi-a-GCST90001530	rs12885009	Mo MDSC AC	14	85063049
ebi-a-GCST90001530	rs3007125	Mo MDSC AC	14	47267652
ebi-a-GCST90001530	rs78949787	Mo MDSC AC	19	51591138
ebi-a-GCST90001530	rs1354106	Mo MDSC AC	19	51737991
ebi-a-GCST90001531	rs114590598	CD33dim HLA DR	1	185852065
ebi-a-GCST90001531	rs10515946	CD33dim HLA DR	2	208038537
ebi-a-GCST90001531	rs9842941	CD33dim HLA DR	3	128441690
ebi-a-GCST90001531	rs138503959	CD33dim HLA DR	3	89320290

ebi-a-GCST90001531	rs146071030	CD33dim HLA DR	3	134673672
ebi-a-GCST90001531	rs6851258	CD33dim HLA DR	4	113247850
ebi-a-GCST90001531	rs74831462	CD33dim HLA DR	5	95242577
ebi-a-GCST90001531	rs116166244	CD33dim HLA DR	5	110463783
ebi-a-GCST90001531	rs403939	CD33dim HLA DR	8	144555482
ebi-a-GCST90001531	rs962695	CD33dim HLA DR	8	80656206
ebi-a-GCST90001531	rs9297816	CD33dim HLA DR	8	132011279
ebi-a-GCST90001531	rs11203701	CD33dim HLA DR	8	15256015
ebi-a-GCST90001531	rs78915471	CD33dim HLA DR	10	18406409
ebi-a-GCST90001531	rs78338533	CD33dim HLA DR	10	11630331
ebi-a-GCST90001531	rs72776409	CD33dim HLA DR	10	759450
ebi-a-GCST90001531	rs142419042	CD33dim HLA DR	11	104445613
ebi-a-GCST90001531	rs117780942	CD33dim HLA DR	11	105062174
ebi-a-GCST90001531	rs7987975	CD33dim HLA DR	13	114578893
ebi-a-GCST90001531	rs117197348	CD33dim HLA DR	14	90893228
ebi-a-GCST90001531	rs77388515	CD33dim HLA DR	19	34133960
ebi-a-GCST90001531	rs33978622	CD33dim HLA DR	19	51736383
ebi-a-GCST90001531	rs78744187	CD33dim HLA DR	19	33754548
ebi-a-GCST90001531	rs55678760	CD33dim HLA DR	20	58069558
ebi-a-GCST90001531	rs2013837	CD33dim HLA DR	22	39271617
ebi-a-GCST90001531	rs1475853	CD33dim HLA DR	22	25145753
ebi-a-GCST90001532	rs114590598	Basophil AC	1	185852065
ebi-a-GCST90001532	rs138503959	Basophil AC	3	89320290
ebi-a-GCST90001532	rs9842941	Basophil AC	3	128441690
ebi-a-GCST90001532	rs6446560	Basophil AC	4	4257901
ebi-a-GCST90001532	rs116166244	Basophil AC	5	110463783
ebi-a-GCST90001532	rs74831462	Basophil AC	5	95242577
ebi-a-GCST90001532	rs3897098	Basophil AC	8	83786650
ebi-a-GCST90001532	rs9297816	Basophil AC	8	132011279
ebi-a-GCST90001532	rs403939	Basophil AC	8	144555482
ebi-a-GCST90001532	rs35406957	Basophil AC	8	130634816
ebi-a-GCST90001532	rs11203701	Basophil AC	8	15256015
ebi-a-GCST90001532	rs78915471	Basophil AC	10	18406409
ebi-a-GCST90001532	rs78338533	Basophil AC	10	11630331
ebi-a-GCST90001532	rs117780942	Basophil AC	11	105062174
ebi-a-GCST90001532	rs142419042	Basophil AC	11	104445613
ebi-a-GCST90001532	rs117197348	Basophil AC	14	90893228
ebi-a-GCST90001532	rs3865444	Basophil AC	19	51727962
ebi-a-GCST90001532	rs77388515	Basophil AC	19	34133960
ebi-a-GCST90001532	rs75773078	Basophil AC	19	51742145
ebi-a-GCST90001532	rs78744187	Basophil AC	19	33754548
ebi-a-GCST90001532	rs7351079	Basophil AC	19	51574038
ebi-a-GCST90001532	rs55678760	Basophil AC	20	58069558
ebi-a-GCST90001532	rs2013837	Basophil AC	22	39271617
ebi-a-GCST90001532	rs1475853	Basophil AC	22	25145753
ebi-a-GCST90001533	rs61823496	Basophil %CD33di	1	223653616
ebi-a-GCST90001533	rs75165408	Basophil %CD33di	3	67867718
ebi-a-GCST90001533	rs4350951	Basophil %CD33di	3	169241641

ebi-a-GCST90001533	rs4679209	Basophil %CD33di	3	125887424
ebi-a-GCST90001533	rs72777352	Basophil %CD33di	5	94316089
ebi-a-GCST90001533	rs118101102	Basophil %CD33di	6	79907097
ebi-a-GCST90001533	rs2392579	Basophil %CD33di	7	38540948
ebi-a-GCST90001533	rs13237906	Basophil %CD33di	7	101155469
ebi-a-GCST90001533	rs16872136	Basophil %CD33di	8	106003701
ebi-a-GCST90001533	rs49195	Basophil %CD33di	8	71550708
ebi-a-GCST90001533	rs72714455	Basophil %CD33di	8	131925751
ebi-a-GCST90001533	rs1924240	Basophil %CD33di	9	85792653
ebi-a-GCST90001533	rs73163591	Basophil %CD33di	13	28146072
ebi-a-GCST90001533	rs62024809	Basophil %CD33di	15	53420725
ebi-a-GCST90001533	rs208569	Basophil %CD33di	16	22868035
ebi-a-GCST90001533	rs7351079	Basophil %CD33di	19	51574038
ebi-a-GCST90001533	rs117417480	Basophil %CD33di	19	54429001
ebi-a-GCST90001533	rs3865444	Basophil %CD33di	19	51727962
ebi-a-GCST90001533	rs4499342	Basophil %CD33di	19	8786913
ebi-a-GCST90001533	rs422720	Basophil %CD33di	21	46578954
ebi-a-GCST90001533	rs2244104	Basophil %CD33di	22	39410500
ebi-a-GCST90001534	rs150956189	CD45RA- CD4+ A(	1	214625781
ebi-a-GCST90001534	rs541367304	CD45RA- CD4+ A(	1	198283058
ebi-a-GCST90001534	rs6743943	CD45RA- CD4+ A(	2	118512809
ebi-a-GCST90001534	rs148716007	CD45RA- CD4+ A(	5	120094917
ebi-a-GCST90001534	rs117563350	CD45RA- CD4+ A(	6	151699396
ebi-a-GCST90001534	rs55905714	CD45RA- CD4+ A(	6	68723598
ebi-a-GCST90001534	rs62447160	CD45RA- CD4+ A(	7	36527098
ebi-a-GCST90001534	rs73176863	CD45RA- CD4+ A(	7	156112045
ebi-a-GCST90001534	rs41306808	CD45RA- CD4+ A(	10	12164786
ebi-a-GCST90001534	rs137903114	CD45RA- CD4+ A(	11	130770585
ebi-a-GCST90001534	rs11062774	CD45RA- CD4+ A(	12	3830558
ebi-a-GCST90001534	rs653178	CD45RA- CD4+ A(	12	112007756
ebi-a-GCST90001534	rs7162344	CD45RA- CD4+ A(	15	26030485
ebi-a-GCST90001534	rs4357954	CD45RA- CD4+ A(	16	7395086
ebi-a-GCST90001534	rs145638437	CD45RA- CD4+ A(	16	27137016
ebi-a-GCST90001534	rs9961909	CD45RA- CD4+ A(	18	49180597
ebi-a-GCST90001534	rs184600191	CD45RA- CD4+ A(	18	14436972
ebi-a-GCST90001535	rs116794175	CD45RA- CD4+ %	1	194010208
ebi-a-GCST90001535	rs12121018	CD45RA- CD4+ %	1	94176595
ebi-a-GCST90001535	rs114825362	CD45RA- CD4+ %	1	101526164
ebi-a-GCST90001535	rs150607181	CD45RA- CD4+ %	1	239680551
ebi-a-GCST90001535	rs76201922	CD45RA- CD4+ %	1	226764439
ebi-a-GCST90001535	rs146620586	CD45RA- CD4+ %	1	198524264
ebi-a-GCST90001535	rs146674398	CD45RA- CD4+ %	1	187940731
ebi-a-GCST90001535	rs11579717	CD45RA- CD4+ %	1	199999121
ebi-a-GCST90001535	rs10432663	CD45RA- CD4+ %	2	41955462
ebi-a-GCST90001535	rs11124653	CD45RA- CD4+ %	2	39112166
ebi-a-GCST90001535	rs72613709	CD45RA- CD4+ %	2	197060767
ebi-a-GCST90001535	rs12712610	CD45RA- CD4+ %	2	38897249
ebi-a-GCST90001535	rs183293285	CD45RA- CD4+ %	3	75917650

ebi-a-GCST90001535	rs10013469	CD45RA- CD4+ %	4	185924276
ebi-a-GCST90001535	rs931555	CD45RA- CD4+ %	5	35803577
ebi-a-GCST90001535	rs62341785	CD45RA- CD4+ %	5	7272022
ebi-a-GCST90001535	rs7700617	CD45RA- CD4+ %	5	39546706
ebi-a-GCST90001535	rs9271766	CD45RA- CD4+ %	6	32594169
ebi-a-GCST90001535	rs71569312	CD45RA- CD4+ %	6	35830034
ebi-a-GCST90001535	rs10230422	CD45RA- CD4+ %	7	16431997
ebi-a-GCST90001535	rs10269315	CD45RA- CD4+ %	7	119060402
ebi-a-GCST90001535	rs76139558	CD45RA- CD4+ %	10	2787360
ebi-a-GCST90001535	rs1250600	CD45RA- CD4+ %	10	81010850
ebi-a-GCST90001535	rs141350785	CD45RA- CD4+ %	11	5240669
ebi-a-GCST90001535	rs73189225	CD45RA- CD4+ %	12	108927798
ebi-a-GCST90001535	rs1849779	CD45RA- CD4+ %	12	43674787
ebi-a-GCST90001535	rs146329634	CD45RA- CD4+ %	14	89433599
ebi-a-GCST90001535	rs2252009	CD45RA- CD4+ %	16	3214442
ebi-a-GCST90001535	rs7216330	CD45RA- CD4+ %	17	4102387
ebi-a-GCST90001535	rs76516339	CD45RA- CD4+ %	18	74287337
ebi-a-GCST90001536	rs11579717	CD45RA- CD4+ %	1	199999121
ebi-a-GCST90001536	rs146620586	CD45RA- CD4+ %	1	198524264
ebi-a-GCST90001536	rs150422994	CD45RA- CD4+ %	1	21018820
ebi-a-GCST90001536	rs1581998	CD45RA- CD4+ %	1	157535842
ebi-a-GCST90001536	rs1380699	CD45RA- CD4+ %	2	57906216
ebi-a-GCST90001536	rs72795179	CD45RA- CD4+ %	2	38931153
ebi-a-GCST90001536	rs12712610	CD45RA- CD4+ %	2	38897249
ebi-a-GCST90001536	rs190665193	CD45RA- CD4+ %	2	106438361
ebi-a-GCST90001536	rs6771179	CD45RA- CD4+ %	3	14769598
ebi-a-GCST90001536	rs7675607	CD45RA- CD4+ %	4	37884855
ebi-a-GCST90001536	rs10013442	CD45RA- CD4+ %	4	185924195
ebi-a-GCST90001536	rs12498745	CD45RA- CD4+ %	4	99216768
ebi-a-GCST90001536	rs138143706	CD45RA- CD4+ %	6	13138376
ebi-a-GCST90001536	rs9271766	CD45RA- CD4+ %	6	32594169
ebi-a-GCST90001536	rs74911099	CD45RA- CD4+ %	6	85644512
ebi-a-GCST90001536	rs1051488	CD45RA- CD4+ %	6	31322911
ebi-a-GCST90001536	rs79647440	CD45RA- CD4+ %	7	142438302
ebi-a-GCST90001536	rs144196931	CD45RA- CD4+ %	7	139232896
ebi-a-GCST90001536	rs1550856	CD45RA- CD4+ %	8	117805397
ebi-a-GCST90001536	rs62539662	CD45RA- CD4+ %	9	10237013
ebi-a-GCST90001536	rs11201613	CD45RA- CD4+ %	10	87172617
ebi-a-GCST90001536	rs188628982	CD45RA- CD4+ %	11	86965976
ebi-a-GCST90001536	rs7963505	CD45RA- CD4+ %	12	131363533
ebi-a-GCST90001536	rs142403852	CD45RA- CD4+ %	12	86979143
ebi-a-GCST90001536	rs543009646	CD45RA- CD4+ %	16	16334247
ebi-a-GCST90001536	rs12448994	CD45RA- CD4+ %	16	989381
ebi-a-GCST90001536	rs1626874	CD45RA- CD4+ %	18	66895282
ebi-a-GCST90001536	rs73196629	CD45RA- CD4+ %	21	19399885
ebi-a-GCST90001536	rs12160006	CD45RA- CD4+ %	22	32667243
ebi-a-GCST90001537	rs12043060	CM CD4+ AC	1	157059350
ebi-a-GCST90001537	rs541367304	CM CD4+ AC	1	198283058

ebi-a-GCST90001537	rs10206629	CM CD4+ AC	2	38920333
ebi-a-GCST90001537	rs2875222	CM CD4+ AC	3	75433850
ebi-a-GCST90001537	rs79668094	CM CD4+ AC	4	64094440
ebi-a-GCST90001537	rs11725186	CM CD4+ AC	4	94012466
ebi-a-GCST90001537	rs76664097	CM CD4+ AC	5	178428451
ebi-a-GCST90001537	rs9271622	CM CD4+ AC	6	32591845
ebi-a-GCST90001537	rs13207893	CM CD4+ AC	6	32586922
ebi-a-GCST90001537	rs34327811	CM CD4+ AC	6	148552958
ebi-a-GCST90001537	rs75024452	CM CD4+ AC	6	7780498
ebi-a-GCST90001537	rs7760353	CM CD4+ AC	6	102207604
ebi-a-GCST90001537	rs17717645	CM CD4+ AC	7	18003774
ebi-a-GCST90001537	rs11990065	CM CD4+ AC	8	17277790
ebi-a-GCST90001537	rs118048878	CM CD4+ AC	9	27446846
ebi-a-GCST90001537	rs183058497	CM CD4+ AC	12	65373483
ebi-a-GCST90001537	rs1913821	CM CD4+ AC	12	20885314
ebi-a-GCST90001537	rs3184504	CM CD4+ AC	12	111884608
ebi-a-GCST90001537	rs11062774	CM CD4+ AC	12	3830558
ebi-a-GCST90001537	rs151245760	CM CD4+ AC	12	16811638
ebi-a-GCST90001537	rs55873755	CM CD4+ AC	12	26948822
ebi-a-GCST90001537	rs1980708	CM CD4+ AC	13	100586353
ebi-a-GCST90001537	rs78720100	CM CD4+ AC	15	97948444
ebi-a-GCST90001537	rs7162344	CM CD4+ AC	15	26030485
ebi-a-GCST90001537	rs117066544	CM CD4+ AC	17	7887429
ebi-a-GCST90001537	rs144079997	CM CD4+ AC	17	72909337
ebi-a-GCST90001537	rs1043118	CM CD4+ AC	20	56223607
ebi-a-GCST90001537	rs61148192	CM CD4+ AC	20	10891892
ebi-a-GCST90001537	rs17584809	CM CD4+ AC	21	33131559
ebi-a-GCST90001538	rs116640816	CM CD4+ %CD4+	1	233503332
ebi-a-GCST90001538	rs76756517	CM CD4+ %CD4+	1	27383717
ebi-a-GCST90001538	rs4497244	CM CD4+ %CD4+	1	197997953
ebi-a-GCST90001538	rs12409155	CM CD4+ %CD4+	1	110998348
ebi-a-GCST90001538	rs72817100	CM CD4+ %CD4+	2	100426114
ebi-a-GCST90001538	rs4473342	CM CD4+ %CD4+	2	160994845
ebi-a-GCST90001538	rs963730	CM CD4+ %CD4+	2	39216773
ebi-a-GCST90001538	rs59491101	CM CD4+ %CD4+	2	62624970
ebi-a-GCST90001538	rs6751481	CM CD4+ %CD4+	2	38897810
ebi-a-GCST90001538	rs6780334	CM CD4+ %CD4+	3	177085939
ebi-a-GCST90001538	rs186961945	CM CD4+ %CD4+	3	34562966
ebi-a-GCST90001538	rs12503846	CM CD4+ %CD4+	4	181498148
ebi-a-GCST90001538	rs10782230	CM CD4+ %CD4+	6	126228512
ebi-a-GCST90001538	rs9396752	CM CD4+ %CD4+	6	9941223
ebi-a-GCST90001538	rs10245930	CM CD4+ %CD4+	7	21822311
ebi-a-GCST90001538	rs6954594	CM CD4+ %CD4+	7	144239162
ebi-a-GCST90001538	rs13233481	CM CD4+ %CD4+	7	130980127
ebi-a-GCST90001538	rs72694085	CM CD4+ %CD4+	9	4651962
ebi-a-GCST90001538	rs113269772	CM CD4+ %CD4+	9	79870974
ebi-a-GCST90001538	rs143395500	CM CD4+ %CD4+	10	69536834
ebi-a-GCST90001538	rs151245760	CM CD4+ %CD4+	12	16811638

ebi-a-GCST90001538	rs12231781	CM CD4+ %CD4+	12	16272142
ebi-a-GCST90001538	rs143624012	CM CD4+ %CD4+	13	74308892
ebi-a-GCST90001538	rs2816718	CM CD4+ %CD4+	14	49245824
ebi-a-GCST90001538	rs4784296	CM CD4+ %CD4+	16	51479349
ebi-a-GCST90001538	rs117765614	CM CD4+ %CD4+	17	39786505
ebi-a-GCST90001538	rs186686893	CM CD4+ %CD4+	17	37015917
ebi-a-GCST90001538	rs8067124	CM CD4+ %CD4+	17	38869490
ebi-a-GCST90001538	rs56350470	CM CD4+ %CD4+	19	53813407
ebi-a-GCST90001538	rs73021131	CM CD4+ %CD4+	19	16505722
ebi-a-GCST90001538	rs13041294	CM CD4+ %CD4+	20	48024935
ebi-a-GCST90001538	rs74510800	CM CD4+ %CD4+	22	36127324
ebi-a-GCST90001539	rs10495355	CM CD4+ %T cell	1	235018206
ebi-a-GCST90001539	rs12409155	CM CD4+ %T cell	1	110998348
ebi-a-GCST90001539	rs12734326	CM CD4+ %T cell	1	240644644
ebi-a-GCST90001539	rs143570471	CM CD4+ %T cell	2	110018647
ebi-a-GCST90001539	rs6751481	CM CD4+ %T cell	2	38897810
ebi-a-GCST90001539	rs73171187	CM CD4+ %T cell	3	177034032
ebi-a-GCST90001539	rs148365679	CM CD4+ %T cell	3	194035416
ebi-a-GCST90001539	rs73206223	CM CD4+ %T cell	4	5821635
ebi-a-GCST90001539	rs715997	CM CD4+ %T cell	4	182072648
ebi-a-GCST90001539	rs149778510	CM CD4+ %T cell	4	112434921
ebi-a-GCST90001539	rs17230697	CM CD4+ %T cell	4	55480792
ebi-a-GCST90001539	rs114753591	CM CD4+ %T cell	5	43356042
ebi-a-GCST90001539	rs2127105	CM CD4+ %T cell	5	172260234
ebi-a-GCST90001539	rs7757458	CM CD4+ %T cell	6	103618303
ebi-a-GCST90001539	rs13233481	CM CD4+ %T cell	7	130980127
ebi-a-GCST90001539	rs1983285	CM CD4+ %T cell	7	131783062
ebi-a-GCST90001539	rs62525675	CM CD4+ %T cell	8	87705654
ebi-a-GCST90001539	rs72694085	CM CD4+ %T cell	9	4651962
ebi-a-GCST90001539	rs12805205	CM CD4+ %T cell	11	97164068
ebi-a-GCST90001539	rs151245760	CM CD4+ %T cell	12	16811638
ebi-a-GCST90001539	rs10846286	CM CD4+ %T cell	12	16252521
ebi-a-GCST90001539	rs7963505	CM CD4+ %T cell	12	131363533
ebi-a-GCST90001539	rs4784296	CM CD4+ %T cell	16	51479349
ebi-a-GCST90001539	rs12453732	CM CD4+ %T cell	17	38177839
ebi-a-GCST90001539	rs13041294	CM CD4+ %T cell	20	48024935
ebi-a-GCST90001539	rs10485474	CM CD4+ %T cell	20	59473649
ebi-a-GCST90001540	rs9998896	Naive CD4+ AC	4	94816722
ebi-a-GCST90001540	rs186690287	Naive CD4+ AC	4	109425453
ebi-a-GCST90001540	rs4282192	Naive CD4+ AC	4	38320205
ebi-a-GCST90001540	rs72776301	Naive CD4+ AC	5	79555714
ebi-a-GCST90001540	rs151100670	Naive CD4+ AC	5	141405792
ebi-a-GCST90001540	rs7704364	Naive CD4+ AC	5	8105516
ebi-a-GCST90001540	rs143021470	Naive CD4+ AC	6	81026437
ebi-a-GCST90001540	rs17205612	Naive CD4+ AC	6	32637333
ebi-a-GCST90001540	rs139014478	Naive CD4+ AC	6	51524409
ebi-a-GCST90001540	rs9271772	Naive CD4+ AC	6	32594275
ebi-a-GCST90001540	rs3129891	Naive CD4+ AC	6	32415080

ebi-a-GCST90001540	rs77253141	Naive CD4+ AC	8	24504854
ebi-a-GCST90001540	rs71501086	Naive CD4+ AC	9	11010939
ebi-a-GCST90001540	rs55996464	Naive CD4+ AC	9	130525294
ebi-a-GCST90001540	rs78628680	Naive CD4+ AC	10	114300633
ebi-a-GCST90001540	rs75958070	Naive CD4+ AC	11	79203049
ebi-a-GCST90001540	rs3184504	Naive CD4+ AC	12	111884608
ebi-a-GCST90001540	rs980169	Naive CD4+ AC	15	96720568
ebi-a-GCST90001540	rs75139198	Naive CD4+ AC	15	96213762
ebi-a-GCST90001540	rs12916593	Naive CD4+ AC	15	26130511
ebi-a-GCST90001540	rs113039817	Naive CD4+ AC	16	7395808
ebi-a-GCST90001540	rs12607959	Naive CD4+ AC	18	65226341
ebi-a-GCST90001540	rs79538978	Naive CD4+ AC	18	54960800
ebi-a-GCST90001540	rs78280695	Naive CD4+ AC	19	15667049
ebi-a-GCST90001540	rs78897171	Naive CD4+ AC	20	60443251
ebi-a-GCST90001540	rs113148171	Naive CD4+ AC	22	46993034
ebi-a-GCST90001541	rs11579717	Naive CD4+ %CD4	1	199999121
ebi-a-GCST90001541	rs146620586	Naive CD4+ %CD4	1	198524264
ebi-a-GCST90001541	rs12121018	Naive CD4+ %CD4	1	94176595
ebi-a-GCST90001541	rs114825362	Naive CD4+ %CD4	1	101526164
ebi-a-GCST90001541	rs150607181	Naive CD4+ %CD4	1	239680551
ebi-a-GCST90001541	rs12712610	Naive CD4+ %CD4	2	38897249
ebi-a-GCST90001541	rs11124653	Naive CD4+ %CD4	2	39112166
ebi-a-GCST90001541	rs72613709	Naive CD4+ %CD4	2	197060767
ebi-a-GCST90001541	rs11716653	Naive CD4+ %CD4	3	174932703
ebi-a-GCST90001541	rs62274365	Naive CD4+ %CD4	3	103225168
ebi-a-GCST90001541	rs7636470	Naive CD4+ %CD4	3	186606581
ebi-a-GCST90001541	rs36088117	Naive CD4+ %CD4	4	11877955
ebi-a-GCST90001541	rs140125890	Naive CD4+ %CD4	4	40884597
ebi-a-GCST90001541	rs4702116	Naive CD4+ %CD4	5	15902603
ebi-a-GCST90001541	rs4959365	Naive CD4+ %CD4	6	6026750
ebi-a-GCST90001541	rs148031710	Naive CD4+ %CD4	6	31148873
ebi-a-GCST90001541	rs9271536	Naive CD4+ %CD4	6	32589978
ebi-a-GCST90001541	rs113243185	Naive CD4+ %CD4	6	32585071
ebi-a-GCST90001541	rs17205612	Naive CD4+ %CD4	6	32637333
ebi-a-GCST90001541	rs7798224	Naive CD4+ %CD4	7	103375873
ebi-a-GCST90001541	rs113763504	Naive CD4+ %CD4	10	81110536
ebi-a-GCST90001541	rs76139558	Naive CD4+ %CD4	10	2787360
ebi-a-GCST90001541	rs73189225	Naive CD4+ %CD4	12	108927798
ebi-a-GCST90001541	rs1849779	Naive CD4+ %CD4	12	43674787
ebi-a-GCST90001541	rs146180953	Naive CD4+ %CD4	13	100509066
ebi-a-GCST90001541	rs146329634	Naive CD4+ %CD4	14	89433599
ebi-a-GCST90001541	rs77572771	Naive CD4+ %CD4	15	63059380
ebi-a-GCST90001541	rs8071815	Naive CD4+ %CD4	17	47988216
ebi-a-GCST90001541	rs66679234	Naive CD4+ %CD4	17	4224639
ebi-a-GCST90001541	rs11652705	Naive CD4+ %CD4	17	41761741
ebi-a-GCST90001541	rs12607959	Naive CD4+ %CD4	18	65226341
ebi-a-GCST90001541	rs8091016	Naive CD4+ %CD4	18	74288206
ebi-a-GCST90001541	rs79538978	Naive CD4+ %CD4	18	54960800

ebi-a-GCST90001541	rs6046769	Naive CD4+ %CD4	20	20238128
ebi-a-GCST90001541	rs117262034	Naive CD4+ %CD4	22	19597257
ebi-a-GCST90001541	rs5767274	Naive CD4+ %CD4	22	46980774
ebi-a-GCST90001542	rs541367304	EM CD4+ AC	1	198283058
ebi-a-GCST90001542	rs11579717	EM CD4+ AC	1	199999121
ebi-a-GCST90001542	rs150956189	EM CD4+ AC	1	214625781
ebi-a-GCST90001542	rs6729741	EM CD4+ AC	2	118599600
ebi-a-GCST90001542	rs142002778	EM CD4+ AC	3	75855397
ebi-a-GCST90001542	rs17376950	EM CD4+ AC	3	60085026
ebi-a-GCST90001542	rs75603168	EM CD4+ AC	4	5108593
ebi-a-GCST90001542	rs115531274	EM CD4+ AC	5	18401623
ebi-a-GCST90001542	rs7737130	EM CD4+ AC	5	123727627
ebi-a-GCST90001542	rs115795061	EM CD4+ AC	6	32731861
ebi-a-GCST90001542	rs117563350	EM CD4+ AC	6	151699396
ebi-a-GCST90001542	rs62447160	EM CD4+ AC	7	36527098
ebi-a-GCST90001542	rs75275482	EM CD4+ AC	8	121950150
ebi-a-GCST90001542	rs74443612	EM CD4+ AC	8	77653957
ebi-a-GCST90001542	rs148709760	EM CD4+ AC	9	136923252
ebi-a-GCST90001542	rs143648086	EM CD4+ AC	9	139422055
ebi-a-GCST90001542	rs17183358	EM CD4+ AC	10	2738100
ebi-a-GCST90001542	rs112785791	EM CD4+ AC	12	114413657
ebi-a-GCST90001542	rs77802035	EM CD4+ AC	13	49298909
ebi-a-GCST90001542	rs12894769	EM CD4+ AC	14	79210529
ebi-a-GCST90001542	rs72705882	EM CD4+ AC	15	26023978
ebi-a-GCST90001542	rs74010649	EM CD4+ AC	16	7400983
ebi-a-GCST90001542	rs9967156	EM CD4+ AC	18	73833744
ebi-a-GCST90001542	rs9961909	EM CD4+ AC	18	49180597
ebi-a-GCST90001542	rs78082758	EM CD4+ AC	22	26812132
ebi-a-GCST90001543	rs72668680	EM CD4+ %CD4+	1	53240377
ebi-a-GCST90001543	rs146620586	EM CD4+ %CD4+	1	198524264
ebi-a-GCST90001543	rs11579717	EM CD4+ %CD4+	1	199999121
ebi-a-GCST90001543	rs114825362	EM CD4+ %CD4+	1	101526164
ebi-a-GCST90001543	rs12712610	EM CD4+ %CD4+	2	38897249
ebi-a-GCST90001543	rs114348846	EM CD4+ %CD4+	2	241731435
ebi-a-GCST90001543	rs1991423	EM CD4+ %CD4+	2	137242053
ebi-a-GCST90001543	rs114499519	EM CD4+ %CD4+	3	102911185
ebi-a-GCST90001543	rs1920658	EM CD4+ %CD4+	3	160357646
ebi-a-GCST90001543	rs114174358	EM CD4+ %CD4+	4	1091560
ebi-a-GCST90001543	rs9267484	EM CD4+ %CD4+	6	31505724
ebi-a-GCST90001543	rs35900954	EM CD4+ %CD4+	6	124439410
ebi-a-GCST90001543	rs4959028	EM CD4+ %CD4+	6	32383138
ebi-a-GCST90001543	rs9269702	EM CD4+ %CD4+	6	32547143
ebi-a-GCST90001543	rs7798224	EM CD4+ %CD4+	7	103375873
ebi-a-GCST90001543	rs28844071	EM CD4+ %CD4+	8	127475071
ebi-a-GCST90001543	rs439782	EM CD4+ %CD4+	8	68568325
ebi-a-GCST90001543	rs72858657	EM CD4+ %CD4+	11	14128767
ebi-a-GCST90001543	rs79505492	EM CD4+ %CD4+	11	79167993
ebi-a-GCST90001543	rs73060045	EM CD4+ %CD4+	12	12168131

ebi-a-GCST90001543	rs113498448	EM CD4+ %CD4+	12	27421151
ebi-a-GCST90001543	rs12428126	EM CD4+ %CD4+	13	21012562
ebi-a-GCST90001543	rs72744142	EM CD4+ %CD4+	15	81518947
ebi-a-GCST90001543	rs183743052	EM CD4+ %CD4+	17	51049212
ebi-a-GCST90001543	rs157386	EM CD4+ %CD4+	17	19699121
ebi-a-GCST90001543	rs75010615	EM CD4+ %CD4+	17	38896451
ebi-a-GCST90001543	rs79538978	EM CD4+ %CD4+	18	54960800
ebi-a-GCST90001543	rs12607959	EM CD4+ %CD4+	18	65226341
ebi-a-GCST90001543	rs6046769	EM CD4+ %CD4+	20	20238128
ebi-a-GCST90001544	rs114825362	EM CD4+ %T cell	1	101526164
ebi-a-GCST90001544	rs146620586	EM CD4+ %T cell	1	198524264
ebi-a-GCST90001544	rs11579717	EM CD4+ %T cell	1	199999121
ebi-a-GCST90001544	rs80060305	EM CD4+ %T cell	2	46131295
ebi-a-GCST90001544	rs58905133	EM CD4+ %T cell	2	38887964
ebi-a-GCST90001544	rs113243185	EM CD4+ %T cell	6	32585071
ebi-a-GCST90001544	rs9267484	EM CD4+ %T cell	6	31505724
ebi-a-GCST90001544	rs113642164	EM CD4+ %T cell	6	100662893
ebi-a-GCST90001544	rs9271536	EM CD4+ %T cell	6	32589978
ebi-a-GCST90001544	rs3131014	EM CD4+ %T cell	6	31116627
ebi-a-GCST90001544	rs2500513	EM CD4+ %T cell	6	149287703
ebi-a-GCST90001544	rs11770479	EM CD4+ %T cell	7	70906148
ebi-a-GCST90001544	rs7464374	EM CD4+ %T cell	8	144768532
ebi-a-GCST90001544	rs387663	EM CD4+ %T cell	8	68568308
ebi-a-GCST90001544	rs72858657	EM CD4+ %T cell	11	14128767
ebi-a-GCST90001544	rs2981595	EM CD4+ %T cell	12	10583297
ebi-a-GCST90001544	rs73189225	EM CD4+ %T cell	12	108927798
ebi-a-GCST90001544	rs73060045	EM CD4+ %T cell	12	12168131
ebi-a-GCST90001544	rs2606175	EM CD4+ %T cell	15	88060366
ebi-a-GCST90001544	rs17776339	EM CD4+ %T cell	15	43260378
ebi-a-GCST90001544	rs183743052	EM CD4+ %T cell	17	51049212
ebi-a-GCST90001544	rs12607959	EM CD4+ %T cell	18	65226341
ebi-a-GCST90001544	rs6132693	EM CD4+ %T cell	20	299759
ebi-a-GCST90001545	rs114780135	TD CD4+ AC	1	197674188
ebi-a-GCST90001545	rs12712610	TD CD4+ AC	2	38897249
ebi-a-GCST90001545	rs13096849	TD CD4+ AC	3	184212137
ebi-a-GCST90001545	rs116761394	TD CD4+ AC	3	122030442
ebi-a-GCST90001545	rs62323288	TD CD4+ AC	4	139844715
ebi-a-GCST90001545	rs143706635	TD CD4+ AC	5	67078101
ebi-a-GCST90001545	rs9269202	TD CD4+ AC	6	32449523
ebi-a-GCST90001545	rs9271537	TD CD4+ AC	6	32589982
ebi-a-GCST90001545	rs3105008	TD CD4+ AC	8	96535247
ebi-a-GCST90001545	rs71497247	TD CD4+ AC	10	18763343
ebi-a-GCST90001545	rs79700278	TD CD4+ AC	12	65625955
ebi-a-GCST90001545	rs1304374	TD CD4+ AC	21	32198368
ebi-a-GCST90001546	rs28832396	TD CD4+ %CD4+	1	109586432
ebi-a-GCST90001546	rs113435341	TD CD4+ %CD4+	1	198151688
ebi-a-GCST90001546	rs597606	TD CD4+ %CD4+	2	224660972
ebi-a-GCST90001546	rs1897327	TD CD4+ %CD4+	2	38966307

ebi-a-GCST90001546	rs13024840	TD CD4+ %CD4+	2	38895920
ebi-a-GCST90001546	rs4674422	TD CD4+ %CD4+	2	220606931
ebi-a-GCST90001546	rs116761394	TD CD4+ %CD4+	3	122030442
ebi-a-GCST90001546	rs497623	TD CD4+ %CD4+	3	170418340
ebi-a-GCST90001546	rs62400929	TD CD4+ %CD4+	6	31159546
ebi-a-GCST90001546	rs9501598	TD CD4+ %CD4+	6	31853808
ebi-a-GCST90001546	rs113243185	TD CD4+ %CD4+	6	32585071
ebi-a-GCST90001546	rs9501107	TD CD4+ %CD4+	6	31392047
ebi-a-GCST90001546	rs9271536	TD CD4+ %CD4+	6	32589978
ebi-a-GCST90001546	rs1184536	TD CD4+ %CD4+	10	129021087
ebi-a-GCST90001546	rs7075830	TD CD4+ %CD4+	10	1091650
ebi-a-GCST90001546	rs10750395	TD CD4+ %CD4+	11	99630378
ebi-a-GCST90001546	rs34977393	TD CD4+ %CD4+	11	104086486
ebi-a-GCST90001546	rs191878389	TD CD4+ %CD4+	12	17957041
ebi-a-GCST90001546	rs140464654	TD CD4+ %CD4+	12	41374965
ebi-a-GCST90001546	rs7131851	TD CD4+ %CD4+	12	129434577
ebi-a-GCST90001546	rs12148245	TD CD4+ %CD4+	15	80278961
ebi-a-GCST90001546	rs4778983	TD CD4+ %CD4+	15	82503078
ebi-a-GCST90001546	rs140178705	TD CD4+ %CD4+	18	5785815
ebi-a-GCST90001547	rs28832396	TD CD4+ %T cell	1	109586432
ebi-a-GCST90001547	rs17583875	TD CD4+ %T cell	1	197924770
ebi-a-GCST90001547	rs114171033	TD CD4+ %T cell	2	142418583
ebi-a-GCST90001547	rs55633297	TD CD4+ %T cell	2	121565142
ebi-a-GCST90001547	rs1897327	TD CD4+ %T cell	2	38966307
ebi-a-GCST90001547	rs1509520	TD CD4+ %T cell	2	65076497
ebi-a-GCST90001547	rs13024840	TD CD4+ %T cell	2	38895920
ebi-a-GCST90001547	rs6766978	TD CD4+ %T cell	3	188878471
ebi-a-GCST90001547	rs143346058	TD CD4+ %T cell	3	141674361
ebi-a-GCST90001547	rs140596675	TD CD4+ %T cell	4	119558596
ebi-a-GCST90001547	rs9271536	TD CD4+ %T cell	6	32589978
ebi-a-GCST90001547	rs113243185	TD CD4+ %T cell	6	32585071
ebi-a-GCST90001547	rs9501598	TD CD4+ %T cell	6	31853808
ebi-a-GCST90001547	rs62400929	TD CD4+ %T cell	6	31159546
ebi-a-GCST90001547	rs9501107	TD CD4+ %T cell	6	31392047
ebi-a-GCST90001547	rs113667295	TD CD4+ %T cell	8	138632388
ebi-a-GCST90001547	rs7075830	TD CD4+ %T cell	10	1091650
ebi-a-GCST90001547	rs10750395	TD CD4+ %T cell	11	99630378
ebi-a-GCST90001547	rs7131851	TD CD4+ %T cell	12	129434577
ebi-a-GCST90001547	rs191878389	TD CD4+ %T cell	12	17957041
ebi-a-GCST90001547	rs140239764	TD CD4+ %T cell	12	25084629
ebi-a-GCST90001547	rs140464654	TD CD4+ %T cell	12	41374965
ebi-a-GCST90001547	rs74385090	TD CD4+ %T cell	14	51932689
ebi-a-GCST90001547	rs12148245	TD CD4+ %T cell	15	80278961
ebi-a-GCST90001547	rs4778983	TD CD4+ %T cell	15	82503078
ebi-a-GCST90001547	rs3786686	TD CD4+ %T cell	19	6831355
ebi-a-GCST90001547	rs1304372	TD CD4+ %T cell	21	32198305
ebi-a-GCST90001548	rs113435341	CM CD8br AC	1	198151688
ebi-a-GCST90001548	rs78746216	CM CD8br AC	2	221949923

ebi-a-GCST90001548	rs138265071	CM CD8br AC	2	233371652
ebi-a-GCST90001548	rs78389248	CM CD8br AC	3	29218896
ebi-a-GCST90001548	rs116293582	CM CD8br AC	3	152670506
ebi-a-GCST90001548	rs78805150	CM CD8br AC	5	52037812
ebi-a-GCST90001548	rs7723922	CM CD8br AC	5	171961688
ebi-a-GCST90001548	rs80120480	CM CD8br AC	6	31411457
ebi-a-GCST90001548	rs57031530	CM CD8br AC	6	97286991
ebi-a-GCST90001548	rs59128750	CM CD8br AC	7	68448742
ebi-a-GCST90001548	rs142789041	CM CD8br AC	7	16682991
ebi-a-GCST90001548	rs149060712	CM CD8br AC	8	60906565
ebi-a-GCST90001548	rs13290078	CM CD8br AC	9	27934690
ebi-a-GCST90001548	rs73377526	CM CD8br AC	10	117424380
ebi-a-GCST90001548	rs71467473	CM CD8br AC	11	69919703
ebi-a-GCST90001548	rs4565951	CM CD8br AC	12	108304102
ebi-a-GCST90001548	rs118038203	CM CD8br AC	13	90021753
ebi-a-GCST90001548	rs12149562	CM CD8br AC	16	12222651
ebi-a-GCST90001548	rs78160658	CM CD8br AC	16	4623421
ebi-a-GCST90001548	rs62047982	CM CD8br AC	16	53154832
ebi-a-GCST90001548	rs9924010	CM CD8br AC	16	7398995
ebi-a-GCST90001548	rs117534688	CM CD8br AC	17	31444517
ebi-a-GCST90001548	rs8079667	CM CD8br AC	17	70743564
ebi-a-GCST90001548	rs3179285	CM CD8br AC	17	41476397
ebi-a-GCST90001548	rs143819377	CM CD8br AC	18	4603336
ebi-a-GCST90001548	rs6073949	CM CD8br AC	20	44531878
ebi-a-GCST90001548	rs61735519	CM CD8br AC	22	45749966
ebi-a-GCST90001549	rs74559159	CM CD8br %CD8l	1	3835304
ebi-a-GCST90001549	rs541367304	CM CD8br %CD8l	1	198283058
ebi-a-GCST90001549	rs75518360	CM CD8br %CD8l	1	14881805
ebi-a-GCST90001549	rs55699240	CM CD8br %CD8l	2	113838725
ebi-a-GCST90001549	rs11887842	CM CD8br %CD8l	2	102839478
ebi-a-GCST90001549	rs67145759	CM CD8br %CD8l	2	38902624
ebi-a-GCST90001549	rs183732116	CM CD8br %CD8l	2	168003021
ebi-a-GCST90001549	rs35121986	CM CD8br %CD8l	2	187690214
ebi-a-GCST90001549	rs116515477	CM CD8br %CD8l	3	68134416
ebi-a-GCST90001549	rs66550246	CM CD8br %CD8l	3	159822228
ebi-a-GCST90001549	rs16881729	CM CD8br %CD8l	4	29389538
ebi-a-GCST90001549	rs10035567	CM CD8br %CD8l	5	173005364
ebi-a-GCST90001549	rs16878206	CM CD8br %CD8l	5	59816794
ebi-a-GCST90001549	rs4868220	CM CD8br %CD8l	5	172266325
ebi-a-GCST90001549	rs62463257	CM CD8br %CD8l	7	71923809
ebi-a-GCST90001549	rs35069110	CM CD8br %CD8l	7	157454257
ebi-a-GCST90001549	rs117299036	CM CD8br %CD8l	7	17645647
ebi-a-GCST90001549	rs2040680	CM CD8br %CD8l	7	88785449
ebi-a-GCST90001549	rs6558408	CM CD8br %CD8l	8	145033734
ebi-a-GCST90001549	rs78845536	CM CD8br %CD8l	9	10866626
ebi-a-GCST90001549	rs192829014	CM CD8br %CD8l	9	5750067
ebi-a-GCST90001549	rs116858348	CM CD8br %CD8l	10	92780623
ebi-a-GCST90001549	rs2123414	CM CD8br %CD8l	10	130834276

ebi-a-GCST90001549	rs78719136	CM CD8br %CD8l	14	21355951
ebi-a-GCST90001549	rs350280	CM CD8br %CD8l	16	12231997
ebi-a-GCST90001549	rs62047982	CM CD8br %CD8l	16	53154832
ebi-a-GCST90001549	rs62037700	CM CD8br %CD8l	16	12102157
ebi-a-GCST90001549	rs2713981	CM CD8br %CD8l	17	70743094
ebi-a-GCST90001549	rs4796089	CM CD8br %CD8l	17	33814758
ebi-a-GCST90001549	rs2866112	CM CD8br %CD8l	19	54310961
ebi-a-GCST90001549	rs2840005	CM CD8br %CD8l	19	56314899
ebi-a-GCST90001549	rs2424193	CM CD8br %CD8l	20	18417321
ebi-a-GCST90001549	rs13037846	CM CD8br %CD8l	20	59379480
ebi-a-GCST90001550	rs541367304	CM CD8br %T cell	1	198283058
ebi-a-GCST90001550	rs10865142	CM CD8br %T cell	2	38892836
ebi-a-GCST90001550	rs76516141	CM CD8br %T cell	3	33874255
ebi-a-GCST90001550	rs4702732	CM CD8br %T cell	5	10806765
ebi-a-GCST90001550	rs78708224	CM CD8br %T cell	7	26455241
ebi-a-GCST90001550	rs117410040	CM CD8br %T cell	7	155951234
ebi-a-GCST90001550	rs117299036	CM CD8br %T cell	7	17645647
ebi-a-GCST90001550	rs35069110	CM CD8br %T cell	7	157454257
ebi-a-GCST90001550	rs7819099	CM CD8br %T cell	8	144992862
ebi-a-GCST90001550	rs149060712	CM CD8br %T cell	8	60906565
ebi-a-GCST90001550	rs75442393	CM CD8br %T cell	8	2918034
ebi-a-GCST90001550	rs192459369	CM CD8br %T cell	9	10726150
ebi-a-GCST90001550	rs10868399	CM CD8br %T cell	9	88850694
ebi-a-GCST90001550	rs75837226	CM CD8br %T cell	11	67813671
ebi-a-GCST90001550	rs59708244	CM CD8br %T cell	11	121805674
ebi-a-GCST90001550	rs8003606	CM CD8br %T cell	14	68973250
ebi-a-GCST90001550	rs139325044	CM CD8br %T cell	16	59183550
ebi-a-GCST90001550	rs56392104	CM CD8br %T cell	16	11828567
ebi-a-GCST90001550	rs173262	CM CD8br %T cell	16	12230161
ebi-a-GCST90001550	rs140690408	CM CD8br %T cell	16	53339004
ebi-a-GCST90001550	rs34353535	CM CD8br %T cell	19	45038267
ebi-a-GCST90001550	rs61735519	CM CD8br %T cell	22	45749966
ebi-a-GCST90001551	rs2629830	Naive CD8br AC	1	114684259
ebi-a-GCST90001551	rs116590755	Naive CD8br AC	2	173592729
ebi-a-GCST90001551	rs73926977	Naive CD8br AC	2	38260823
ebi-a-GCST90001551	rs78908164	Naive CD8br AC	2	127884901
ebi-a-GCST90001551	rs10178070	Naive CD8br AC	2	18727639
ebi-a-GCST90001551	rs79752907	Naive CD8br AC	2	237010723
ebi-a-GCST90001551	rs4849134	Naive CD8br AC	2	111615015
ebi-a-GCST90001551	rs79777035	Naive CD8br AC	3	25717920
ebi-a-GCST90001551	rs140786980	Naive CD8br AC	3	174970460
ebi-a-GCST90001551	rs11735841	Naive CD8br AC	4	25612506
ebi-a-GCST90001551	rs11931315	Naive CD8br AC	4	16601009
ebi-a-GCST90001551	rs28465400	Naive CD8br AC	6	29839523
ebi-a-GCST90001551	rs72851020	Naive CD8br AC	6	32501393
ebi-a-GCST90001551	rs1374984	Naive CD8br AC	6	127949276
ebi-a-GCST90001551	rs72826622	Naive CD8br AC	6	20193704
ebi-a-GCST90001551	rs1150829	Naive CD8br AC	6	587532

ebi-a-GCST90001551	rs2853995	Naive CD8br AC	6	31333019
ebi-a-GCST90001551	rs12555210	Naive CD8br AC	9	33428882
ebi-a-GCST90001551	rs12420840	Naive CD8br AC	11	126523412
ebi-a-GCST90001551	rs10783874	Naive CD8br AC	12	58534768
ebi-a-GCST90001551	rs34099171	Naive CD8br AC	12	79413333
ebi-a-GCST90001551	rs113911480	Naive CD8br AC	14	29113167
ebi-a-GCST90001551	rs17685199	Naive CD8br AC	15	36405909
ebi-a-GCST90001551	rs28678122	Naive CD8br AC	15	56956314
ebi-a-GCST90001551	rs11652705	Naive CD8br AC	17	41761741
ebi-a-GCST90001551	rs62065924	Naive CD8br AC	17	39984813
ebi-a-GCST90001551	rs143416600	Naive CD8br AC	18	37173101
ebi-a-GCST90001551	rs62229789	Naive CD8br AC	20	50100758
ebi-a-GCST90001551	rs117120215	Naive CD8br AC	21	38377520
ebi-a-GCST90001552	rs4658869	Naive CD8br %CD	1	243020284
ebi-a-GCST90001552	rs2629824	Naive CD8br %CD	1	114687316
ebi-a-GCST90001552	rs150213926	Naive CD8br %CD	1	200202822
ebi-a-GCST90001552	rs76668354	Naive CD8br %CD	2	103587624
ebi-a-GCST90001552	rs1533299	Naive CD8br %CD	2	111616141
ebi-a-GCST90001552	rs142772175	Naive CD8br %CD	2	120240225
ebi-a-GCST90001552	rs1064086	Naive CD8br %CD	2	237034061
ebi-a-GCST90001552	rs12631411	Naive CD8br %CD	3	106253801
ebi-a-GCST90001552	rs116495677	Naive CD8br %CD	4	6612728
ebi-a-GCST90001552	rs149655274	Naive CD8br %CD	4	154029285
ebi-a-GCST90001552	rs4862490	Naive CD8br %CD	4	185923329
ebi-a-GCST90001552	rs13359589	Naive CD8br %CD	5	73116673
ebi-a-GCST90001552	rs72811823	Naive CD8br %CD	5	145928905
ebi-a-GCST90001552	rs76508332	Naive CD8br %CD	6	167353961
ebi-a-GCST90001552	rs114559146	Naive CD8br %CD	6	31395062
ebi-a-GCST90001552	rs9260902	Naive CD8br %CD	6	29947740
ebi-a-GCST90001552	rs9792050	Naive CD8br %CD	7	31164655
ebi-a-GCST90001552	rs117111088	Naive CD8br %CD	7	8319424
ebi-a-GCST90001552	rs141596789	Naive CD8br %CD	7	28687227
ebi-a-GCST90001552	rs13253487	Naive CD8br %CD	8	62835179
ebi-a-GCST90001552	rs77779289	Naive CD8br %CD	8	18461126
ebi-a-GCST90001552	rs62572630	Naive CD8br %CD	9	114635416
ebi-a-GCST90001552	rs185397210	Naive CD8br %CD	9	12807800
ebi-a-GCST90001552	rs72757070	Naive CD8br %CD	9	113053653
ebi-a-GCST90001552	rs77986171	Naive CD8br %CD	10	54725683
ebi-a-GCST90001552	rs78038265	Naive CD8br %CD	12	85949580
ebi-a-GCST90001552	rs7981825	Naive CD8br %CD	13	98313824
ebi-a-GCST90001552	rs9577271	Naive CD8br %CD	13	114096507
ebi-a-GCST90001552	rs1879612	Naive CD8br %CD	15	99398708
ebi-a-GCST90001552	rs8057009	Naive CD8br %CD	16	85990923
ebi-a-GCST90001552	rs205358	Naive CD8br %CD	16	28114995
ebi-a-GCST90001552	rs181235482	Naive CD8br %CD	16	21569690
ebi-a-GCST90001552	rs5011724	Naive CD8br %CD	17	41759061
ebi-a-GCST90001552	rs8067835	Naive CD8br %CD	17	31363767
ebi-a-GCST90001552	rs143788880	Naive CD8br %CD	17	57361218

ebi-a-GCST90001552	rs34001747	Naive CD8br %CD	17	16863687
ebi-a-GCST90001552	rs275837	Naive CD8br %CD	19	48876190
ebi-a-GCST90001552	rs139415527	Naive CD8br %CD	21	46904641
ebi-a-GCST90001552	rs118003243	Naive CD8br %CD	21	43795055
ebi-a-GCST90001553	rs13386443	Naive CD8br %T c	2	111722118
ebi-a-GCST90001553	rs10183338	Naive CD8br %T c	2	111610816
ebi-a-GCST90001553	rs1064086	Naive CD8br %T c	2	237034061
ebi-a-GCST90001553	rs116590755	Naive CD8br %T c	2	173592729
ebi-a-GCST90001553	rs6833962	Naive CD8br %T c	4	103558731
ebi-a-GCST90001553	rs10474365	Naive CD8br %T c	5	90986313
ebi-a-GCST90001553	rs73404248	Naive CD8br %T c	6	31410352
ebi-a-GCST90001553	rs114581570	Naive CD8br %T c	6	19906546
ebi-a-GCST90001553	rs116562707	Naive CD8br %T c	6	31360602
ebi-a-GCST90001553	rs145607970	Naive CD8br %T c	6	32624017
ebi-a-GCST90001553	rs141596789	Naive CD8br %T c	7	28687227
ebi-a-GCST90001553	rs8180936	Naive CD8br %T c	8	61151574
ebi-a-GCST90001553	rs75368383	Naive CD8br %T c	8	144789624
ebi-a-GCST90001553	rs12555210	Naive CD8br %T c	9	33428882
ebi-a-GCST90001553	rs72815242	Naive CD8br %T c	10	29316021
ebi-a-GCST90001553	rs656841	Naive CD8br %T c	11	126244173
ebi-a-GCST90001553	rs4764066	Naive CD8br %T c	12	14385601
ebi-a-GCST90001553	rs7981825	Naive CD8br %T c	13	98313824
ebi-a-GCST90001553	rs11843149	Naive CD8br %T c	13	24258336
ebi-a-GCST90001553	rs17767232	Naive CD8br %T c	14	65199087
ebi-a-GCST90001553	rs73328313	Naive CD8br %T c	14	93019211
ebi-a-GCST90001553	rs17685199	Naive CD8br %T c	15	36405909
ebi-a-GCST90001553	rs112725843	Naive CD8br %T c	16	28238440
ebi-a-GCST90001553	rs11652705	Naive CD8br %T c	17	41761741
ebi-a-GCST90001553	rs143788880	Naive CD8br %T c	17	57361218
ebi-a-GCST90001553	rs34742163	Naive CD8br %T c	17	3040277
ebi-a-GCST90001553	rs8067835	Naive CD8br %T c	17	31363767
ebi-a-GCST90001553	rs10411666	Naive CD8br %T c	19	779416
ebi-a-GCST90001553	rs117120215	Naive CD8br %T c	21	38377520
ebi-a-GCST90001553	rs116954000	Naive CD8br %T c	22	40298491
ebi-a-GCST90001554	rs11166573	EM CD8br AC	1	101733861
ebi-a-GCST90001554	rs17583875	EM CD8br AC	1	197924770
ebi-a-GCST90001554	rs150956189	EM CD8br AC	1	214625781
ebi-a-GCST90001554	rs76668354	EM CD8br AC	2	103587624
ebi-a-GCST90001554	rs115083752	EM CD8br AC	2	145856378
ebi-a-GCST90001554	rs58905133	EM CD8br AC	2	38887964
ebi-a-GCST90001554	rs57159407	EM CD8br AC	2	22978736
ebi-a-GCST90001554	rs113985770	EM CD8br AC	2	210377930
ebi-a-GCST90001554	rs60998309	EM CD8br AC	3	24959272
ebi-a-GCST90001554	rs2378966	EM CD8br AC	4	179936308
ebi-a-GCST90001554	rs13187183	EM CD8br AC	5	117976098
ebi-a-GCST90001554	rs9382165	EM CD8br AC	6	52836410
ebi-a-GCST90001554	rs11153123	EM CD8br AC	6	109038333
ebi-a-GCST90001554	rs3130685	EM CD8br AC	6	31206206

ebi-a-GCST90001554	rs9268932	EM CD8br AC	6	32433290
ebi-a-GCST90001554	rs593435	EM CD8br AC	7	132417404
ebi-a-GCST90001554	rs73195098	EM CD8br AC	7	112571685
ebi-a-GCST90001554	rs77753889	EM CD8br AC	8	130711599
ebi-a-GCST90001554	rs1322060	EM CD8br AC	9	117697660
ebi-a-GCST90001554	rs1183901	EM CD8br AC	10	90231718
ebi-a-GCST90001554	rs75744217	EM CD8br AC	10	68456634
ebi-a-GCST90001554	rs11060493	EM CD8br AC	12	130160924
ebi-a-GCST90001554	rs11080350	EM CD8br AC	17	33774635
ebi-a-GCST90001554	rs143237141	EM CD8br AC	22	45176439
ebi-a-GCST90001555	rs60220946	EM CD8br %CD8b	1	193505625
ebi-a-GCST90001555	rs150956189	EM CD8br %CD8b	1	214625781
ebi-a-GCST90001555	rs34645541	EM CD8br %CD8b	1	195875605
ebi-a-GCST90001555	rs150329295	EM CD8br %CD8b	1	241856210
ebi-a-GCST90001555	rs2629830	EM CD8br %CD8b	1	114684259
ebi-a-GCST90001555	rs116794175	EM CD8br %CD8b	1	194010208
ebi-a-GCST90001555	rs17583875	EM CD8br %CD8b	1	197924770
ebi-a-GCST90001555	rs11896593	EM CD8br %CD8b	2	38925155
ebi-a-GCST90001555	rs2463509	EM CD8br %CD8b	2	103672986
ebi-a-GCST90001555	rs75196020	EM CD8br %CD8b	2	241750301
ebi-a-GCST90001555	rs28578699	EM CD8br %CD8b	2	38899155
ebi-a-GCST90001555	rs10510244	EM CD8br %CD8b	3	3036671
ebi-a-GCST90001555	rs1479391	EM CD8br %CD8b	3	61970211
ebi-a-GCST90001555	rs116760504	EM CD8br %CD8b	3	47546791
ebi-a-GCST90001555	rs7709566	EM CD8br %CD8b	5	117881168
ebi-a-GCST90001555	rs146969910	EM CD8br %CD8b	5	171664213
ebi-a-GCST90001555	rs148770451	EM CD8br %CD8b	6	26044129
ebi-a-GCST90001555	rs28629976	EM CD8br %CD8b	6	32625221
ebi-a-GCST90001555	rs7867019	EM CD8br %CD8b	9	13378083
ebi-a-GCST90001555	rs7903206	EM CD8br %CD8b	10	28349473
ebi-a-GCST90001555	rs17588249	EM CD8br %CD8b	12	43493148
ebi-a-GCST90001555	rs14067	EM CD8br %CD8b	13	114110660
ebi-a-GCST90001555	rs117545347	EM CD8br %CD8b	15	98090362
ebi-a-GCST90001555	rs12952913	EM CD8br %CD8b	17	18020050
ebi-a-GCST90001555	rs4473273	EM CD8br %CD8b	18	27999885
ebi-a-GCST90001556	rs141730400	EM CD8br %T cell	1	244508870
ebi-a-GCST90001556	rs17583875	EM CD8br %T cell	1	197924770
ebi-a-GCST90001556	rs542493	EM CD8br %T cell	1	65508317
ebi-a-GCST90001556	rs76668354	EM CD8br %T cell	2	103587624
ebi-a-GCST90001556	rs58905133	EM CD8br %T cell	2	38887964
ebi-a-GCST90001556	rs7577330	EM CD8br %T cell	2	113613580
ebi-a-GCST90001556	rs4507432	EM CD8br %T cell	4	1748200
ebi-a-GCST90001556	rs1583072	EM CD8br %T cell	5	118002423
ebi-a-GCST90001556	rs13359589	EM CD8br %T cell	5	73116673
ebi-a-GCST90001556	rs111551789	EM CD8br %T cell	6	29720277
ebi-a-GCST90001556	rs2275854	EM CD8br %T cell	6	29976838
ebi-a-GCST90001556	rs7496	EM CD8br %T cell	6	52842839
ebi-a-GCST90001556	rs2442737	EM CD8br %T cell	6	31329303

ebi-a-GCST90001556	rs116944484	EM CD8br %T cell	10	83233799
ebi-a-GCST90001556	rs4802765	EM CD8br %T cell	19	51589473
ebi-a-GCST90001557	rs113702891	TD CD8br AC	1	9586393
ebi-a-GCST90001557	rs191753228	TD CD8br AC	1	196321168
ebi-a-GCST90001557	rs78397636	TD CD8br AC	2	36822684
ebi-a-GCST90001557	rs7583259	TD CD8br AC	2	38921934
ebi-a-GCST90001557	rs6707421	TD CD8br AC	2	38897781
ebi-a-GCST90001557	rs79293829	TD CD8br AC	4	37444327
ebi-a-GCST90001557	rs56669682	TD CD8br AC	4	136465712
ebi-a-GCST90001557	rs114799586	TD CD8br AC	5	27430294
ebi-a-GCST90001557	rs6915999	TD CD8br AC	6	31377851
ebi-a-GCST90001557	rs78245997	TD CD8br AC	7	67448185
ebi-a-GCST90001557	rs12352819	TD CD8br AC	9	133271663
ebi-a-GCST90001557	rs4593919	TD CD8br AC	10	118142847
ebi-a-GCST90001557	rs561315248	TD CD8br AC	10	68624972
ebi-a-GCST90001557	rs10829262	TD CD8br AC	10	18999976
ebi-a-GCST90001557	rs138437119	TD CD8br AC	10	129206876
ebi-a-GCST90001557	rs7989683	TD CD8br AC	13	96304050
ebi-a-GCST90001557	rs1958553	TD CD8br AC	14	34077214
ebi-a-GCST90001557	rs4796089	TD CD8br AC	17	33814758
ebi-a-GCST90001557	rs2837838	TD CD8br AC	21	42191862
ebi-a-GCST90001557	rs118009051	TD CD8br AC	21	17079538
ebi-a-GCST90001558	rs79479105	TD CD8br %CD8b	1	213569604
ebi-a-GCST90001558	rs113702891	TD CD8br %CD8b	1	9586393
ebi-a-GCST90001558	rs541367304	TD CD8br %CD8b	1	198283058
ebi-a-GCST90001558	rs17397520	TD CD8br %CD8b	1	99902142
ebi-a-GCST90001558	rs11579717	TD CD8br %CD8b	1	199999121
ebi-a-GCST90001558	rs13023673	TD CD8br %CD8b	2	38594483
ebi-a-GCST90001558	rs79827501	TD CD8br %CD8b	2	38431267
ebi-a-GCST90001558	rs12622920	TD CD8br %CD8b	2	38753514
ebi-a-GCST90001558	rs17034567	TD CD8br %CD8b	2	102062586
ebi-a-GCST90001558	rs7583259	TD CD8br %CD8b	2	38921934
ebi-a-GCST90001558	rs6707421	TD CD8br %CD8b	2	38897781
ebi-a-GCST90001558	rs112587592	TD CD8br %CD8b	5	104220406
ebi-a-GCST90001558	rs9263519	TD CD8br %CD8b	6	31067184
ebi-a-GCST90001558	rs79481260	TD CD8br %CD8b	8	7256096
ebi-a-GCST90001558	rs1328299	TD CD8br %CD8b	9	16047293
ebi-a-GCST90001558	rs12352819	TD CD8br %CD8b	9	133271663
ebi-a-GCST90001558	rs561315248	TD CD8br %CD8b	10	68624972
ebi-a-GCST90001558	rs78799727	TD CD8br %CD8b	12	129218834
ebi-a-GCST90001558	rs118080839	TD CD8br %CD8b	12	53301100
ebi-a-GCST90001558	rs7989683	TD CD8br %CD8b	13	96304050
ebi-a-GCST90001558	rs56995664	TD CD8br %CD8b	17	71799960
ebi-a-GCST90001558	rs8070109	TD CD8br %CD8b	17	18598568
ebi-a-GCST90001558	rs77511728	TD CD8br %CD8b	17	2170021
ebi-a-GCST90001558	rs3829817	TD CD8br %CD8b	19	8161450
ebi-a-GCST90001558	rs9614433	TD CD8br %CD8b	22	44700197
ebi-a-GCST90001559	rs146620586	TD CD8br %T cell	1	198524264

ebi-a-GCST90001559	rs13023673	TD CD8br %T cell	2	38594483
ebi-a-GCST90001559	rs6707421	TD CD8br %T cell	2	38897781
ebi-a-GCST90001559	rs7583259	TD CD8br %T cell	2	38921934
ebi-a-GCST90001559	rs17034567	TD CD8br %T cell	2	102062586
ebi-a-GCST90001559	rs79827501	TD CD8br %T cell	2	38431267
ebi-a-GCST90001559	rs78735231	TD CD8br %T cell	4	134825979
ebi-a-GCST90001559	rs1450437	TD CD8br %T cell	4	139524885
ebi-a-GCST90001559	rs1864958	TD CD8br %T cell	5	149767083
ebi-a-GCST90001559	rs73338957	TD CD8br %T cell	5	163631995
ebi-a-GCST90001559	rs62367544	TD CD8br %T cell	5	104124640
ebi-a-GCST90001559	rs138437119	TD CD8br %T cell	10	129206876
ebi-a-GCST90001559	rs561315248	TD CD8br %T cell	10	68624972
ebi-a-GCST90001559	rs7104957	TD CD8br %T cell	11	122575669
ebi-a-GCST90001559	rs75690955	TD CD8br %T cell	12	96812894
ebi-a-GCST90001559	rs7989683	TD CD8br %T cell	13	96304050
ebi-a-GCST90001559	rs28621553	TD CD8br %T cell	17	71801578
ebi-a-GCST90001559	rs3865240	TD CD8br %T cell	17	1991460
ebi-a-GCST90001559	rs9968024	TD CD8br %T cell	22	51144731
ebi-a-GCST90001560	rs17655711	CD45RA+ CD8br A	1	237971051
ebi-a-GCST90001560	rs76444609	CD45RA+ CD8br A	1	249084997
ebi-a-GCST90001560	rs28578699	CD45RA+ CD8br A	2	38899155
ebi-a-GCST90001560	rs11896593	CD45RA+ CD8br A	2	38925155
ebi-a-GCST90001560	rs79777035	CD45RA+ CD8br A	3	25717920
ebi-a-GCST90001560	rs185587352	CD45RA+ CD8br A	3	117384981
ebi-a-GCST90001560	rs62321187	CD45RA+ CD8br A	4	120605817
ebi-a-GCST90001560	rs114799586	CD45RA+ CD8br A	5	27430294
ebi-a-GCST90001560	rs72826622	CD45RA+ CD8br A	6	20193704
ebi-a-GCST90001560	rs114968045	CD45RA+ CD8br A	6	32666960
ebi-a-GCST90001560	rs3104369	CD45RA+ CD8br A	6	32602482
ebi-a-GCST90001560	rs72933273	CD45RA+ CD8br A	6	109644613
ebi-a-GCST90001560	rs6943221	CD45RA+ CD8br A	7	10075089
ebi-a-GCST90001560	rs75402344	CD45RA+ CD8br A	8	5871144
ebi-a-GCST90001560	rs1550856	CD45RA+ CD8br A	8	117805397
ebi-a-GCST90001560	rs140940501	CD45RA+ CD8br A	9	128850262
ebi-a-GCST90001560	rs76552842	CD45RA+ CD8br A	11	83433494
ebi-a-GCST90001560	rs117284243	CD45RA+ CD8br A	11	122918979
ebi-a-GCST90001560	rs11110042	CD45RA+ CD8br A	12	100186724
ebi-a-GCST90001560	rs138746551	CD45RA+ CD8br A	12	48342339
ebi-a-GCST90001560	rs35644926	CD45RA+ CD8br A	15	71472800
ebi-a-GCST90001560	rs72791631	CD45RA+ CD8br A	16	57560762
ebi-a-GCST90001560	rs16968252	CD45RA+ CD8br A	17	31692448
ebi-a-GCST90001560	rs118124576	CD45RA+ CD8br A	18	13390635
ebi-a-GCST90001560	rs13381634	CD45RA+ CD8br A	18	62334258
ebi-a-GCST90001560	rs62077212	CD45RA+ CD8br A	18	27887990
ebi-a-GCST90001560	rs2837838	CD45RA+ CD8br A	21	42191862
ebi-a-GCST90001561	rs190023756	CD45RA+ CD8br	1	199471227
ebi-a-GCST90001561	rs116794175	CD45RA+ CD8br	1	194010208
ebi-a-GCST90001561	rs17583875	CD45RA+ CD8br	1	197924770

ebi-a-GCST90001561	rs34645541	CD45RA+ CD8br [illegible]	1	195875605
ebi-a-GCST90001561	rs150956189	CD45RA+ CD8br [illegible]	1	214625781
ebi-a-GCST90001561	rs28578699	CD45RA+ CD8br [illegible]	2	38899155
ebi-a-GCST90001561	rs2463509	CD45RA+ CD8br [illegible]	2	103672986
ebi-a-GCST90001561	rs11896593	CD45RA+ CD8br [illegible]	2	38925155
ebi-a-GCST90001561	rs75196020	CD45RA+ CD8br [illegible]	2	241750301
ebi-a-GCST90001561	rs10510244	CD45RA+ CD8br [illegible]	3	3036671
ebi-a-GCST90001561	rs181615229	CD45RA+ CD8br [illegible]	3	2937455
ebi-a-GCST90001561	rs62268448	CD45RA+ CD8br [illegible]	3	61307038
ebi-a-GCST90001561	rs115475145	CD45RA+ CD8br [illegible]	4	148303054
ebi-a-GCST90001561	rs146969910	CD45RA+ CD8br [illegible]	5	171664213
ebi-a-GCST90001561	rs140764369	CD45RA+ CD8br [illegible]	5	120985438
ebi-a-GCST90001561	rs148770451	CD45RA+ CD8br [illegible]	6	26044129
ebi-a-GCST90001561	rs118191043	CD45RA+ CD8br [illegible]	7	138310353
ebi-a-GCST90001561	rs77806002	CD45RA+ CD8br [illegible]	11	127735836
ebi-a-GCST90001561	rs117900773	CD45RA+ CD8br [illegible]	11	97410635
ebi-a-GCST90001561	rs76552842	CD45RA+ CD8br [illegible]	11	83433494
ebi-a-GCST90001561	rs17588249	CD45RA+ CD8br [illegible]	12	43493148
ebi-a-GCST90001561	rs14067	CD45RA+ CD8br [illegible]	13	114110660
ebi-a-GCST90001561	rs117545347	CD45RA+ CD8br [illegible]	15	98090362
ebi-a-GCST90001561	rs114542543	CD45RA+ CD8br [illegible]	17	55617067
ebi-a-GCST90001561	rs12952913	CD45RA+ CD8br [illegible]	17	18020050
ebi-a-GCST90001561	rs4473273	CD45RA+ CD8br [illegible]	18	27999885
ebi-a-GCST90001561	rs12958506	CD45RA+ CD8br [illegible]	18	75239395
ebi-a-GCST90001562	rs149720446	CD45RA+ CD8br [illegible]	1	78725368
ebi-a-GCST90001562	rs72932690	CD45RA+ CD8br [illegible]	1	68487020
ebi-a-GCST90001562	rs146620586	CD45RA+ CD8br [illegible]	1	198524264
ebi-a-GCST90001562	rs569064217	CD45RA+ CD8br [illegible]	1	241601336
ebi-a-GCST90001562	rs150956189	CD45RA+ CD8br [illegible]	1	214625781
ebi-a-GCST90001562	rs7583259	CD45RA+ CD8br [illegible]	2	38921934
ebi-a-GCST90001562	rs6707421	CD45RA+ CD8br [illegible]	2	38897781
ebi-a-GCST90001562	rs191749866	CD45RA+ CD8br [illegible]	2	39603005
ebi-a-GCST90001562	rs73101099	CD45RA+ CD8br [illegible]	3	61309584
ebi-a-GCST90001562	rs185587352	CD45RA+ CD8br [illegible]	3	117384981
ebi-a-GCST90001562	rs181615229	CD45RA+ CD8br [illegible]	3	2937455
ebi-a-GCST90001562	rs77031975	CD45RA+ CD8br [illegible]	4	618242
ebi-a-GCST90001562	rs140811183	CD45RA+ CD8br [illegible]	4	135317580
ebi-a-GCST90001562	rs11099574	CD45RA+ CD8br [illegible]	4	84074624
ebi-a-GCST90001562	rs2596459	CD45RA+ CD8br [illegible]	6	31417540
ebi-a-GCST90001562	rs3094228	CD45RA+ CD8br [illegible]	6	31429927
ebi-a-GCST90001562	rs71569429	CD45RA+ CD8br [illegible]	6	164449966
ebi-a-GCST90001562	rs6943827	CD45RA+ CD8br [illegible]	7	41966718
ebi-a-GCST90001562	rs75402344	CD45RA+ CD8br [illegible]	8	5871144
ebi-a-GCST90001562	rs117773023	CD45RA+ CD8br [illegible]	8	3996923
ebi-a-GCST90001562	rs16873557	CD45RA+ CD8br [illegible]	8	106724711
ebi-a-GCST90001562	rs8180936	CD45RA+ CD8br [illegible]	8	61151574
ebi-a-GCST90001562	rs116954492	CD45RA+ CD8br [illegible]	9	16497683
ebi-a-GCST90001562	rs77806002	CD45RA+ CD8br [illegible]	11	127735836

ebi-a-GCST90001562	rs76552842	CD45RA+ CD8br %	11	83433494
ebi-a-GCST90001562	rs11110042	CD45RA+ CD8br %	12	100186724
ebi-a-GCST90001562	rs11629948	CD45RA+ CD8br %	15	65376824
ebi-a-GCST90001562	rs192221507	CD45RA+ CD8br %	16	10650924
ebi-a-GCST90001562	rs79039330	CD45RA+ CD8br %	17	43385620
ebi-a-GCST90001562	rs118124576	CD45RA+ CD8br %	18	13390635
ebi-a-GCST90001562	rs143018694	CD45RA+ CD8br %	19	38512779
ebi-a-GCST90001562	rs2515898	CD45RA+ CD8br %	19	13712956
ebi-a-GCST90001562	rs62212949	CD45RA+ CD8br %	21	19196423
ebi-a-GCST90001562	rs116954000	CD45RA+ CD8br %	22	40298491
ebi-a-GCST90001563	rs62107683	CM DN (CD4-CD8	2	8430901
ebi-a-GCST90001563	rs149297162	CM DN (CD4-CD8	3	193372154
ebi-a-GCST90001563	rs71592595	CM DN (CD4-CD8	5	121763931
ebi-a-GCST90001563	rs149764572	CM DN (CD4-CD8	14	95378018
ebi-a-GCST90001564	rs335577	CM DN (CD4-CD8	1	214874967
ebi-a-GCST90001564	rs140471174	CM DN (CD4-CD8	1	55929294
ebi-a-GCST90001564	rs62107683	CM DN (CD4-CD8	2	8430901
ebi-a-GCST90001564	rs6443820	CM DN (CD4-CD8	3	182393540
ebi-a-GCST90001564	rs41297739	CM DN (CD4-CD8	4	70347949
ebi-a-GCST90001564	rs36108327	CM DN (CD4-CD8	6	134052042
ebi-a-GCST90001564	rs13439307	CM DN (CD4-CD8	8	123030662
ebi-a-GCST90001564	rs4421367	CM DN (CD4-CD8	8	3902849
ebi-a-GCST90001564	rs114405373	CM DN (CD4-CD8	10	88835399
ebi-a-GCST90001564	rs74902462	CM DN (CD4-CD8	12	59066617
ebi-a-GCST90001564	rs11643091	CM DN (CD4-CD8	16	5903304
ebi-a-GCST90001564	rs2681072	CM DN (CD4-CD8	17	33734417
ebi-a-GCST90001564	rs3744246	CM DN (CD4-CD8	17	38084350
ebi-a-GCST90001564	rs77759734	CM DN (CD4-CD8	18	56943484
ebi-a-GCST90001564	rs6056743	CM DN (CD4-CD8	20	9582756
ebi-a-GCST90001564	rs60174111	CM DN (CD4-CD8	20	20107611
ebi-a-GCST90001565	rs10923602	CM DN (CD4-CD8	1	119091238
ebi-a-GCST90001565	rs7419106	CM DN (CD4-CD8	1	4118900
ebi-a-GCST90001565	rs2363876	CM DN (CD4-CD8	1	61776742
ebi-a-GCST90001565	rs142709521	CM DN (CD4-CD8	1	978139
ebi-a-GCST90001565	rs116181048	CM DN (CD4-CD8	2	208596366
ebi-a-GCST90001565	rs60587253	CM DN (CD4-CD8	2	80073031
ebi-a-GCST90001565	rs301224	CM DN (CD4-CD8	3	176091938
ebi-a-GCST90001565	rs1816148	CM DN (CD4-CD8	5	51696375
ebi-a-GCST90001565	rs56017608	CM DN (CD4-CD8	7	149806486
ebi-a-GCST90001565	rs10976338	CM DN (CD4-CD8	9	7510150
ebi-a-GCST90001565	rs11592463	CM DN (CD4-CD8	10	34518442
ebi-a-GCST90001565	rs79148798	CM DN (CD4-CD8	11	132425661
ebi-a-GCST90001565	rs2681071	CM DN (CD4-CD8	17	33734665
ebi-a-GCST90001565	rs188074964	CM DN (CD4-CD8	19	49834820
ebi-a-GCST90001565	rs6093976	CM DN (CD4-CD8	20	43035780
ebi-a-GCST90001565	rs117646786	CM DN (CD4-CD8	22	33601370
ebi-a-GCST90001566	rs12139791	Naive DN (CD4-CI	1	182697886
ebi-a-GCST90001566	rs1679937	Naive DN (CD4-CI	1	53595255

ebi-a-GCST90001566	rs62174823	Naive DN (CD4-CI	2	156766527
ebi-a-GCST90001566	rs78905646	Naive DN (CD4-CI	2	189898847
ebi-a-GCST90001566	rs1348953	Naive DN (CD4-CI	2	84213945
ebi-a-GCST90001566	rs727239	Naive DN (CD4-CI	3	6358753
ebi-a-GCST90001566	rs116550072	Naive DN (CD4-CI	5	150605036
ebi-a-GCST90001566	rs3819288	Naive DN (CD4-CI	6	31322694
ebi-a-GCST90001566	rs2523614	Naive DN (CD4-CI	6	31320654
ebi-a-GCST90001566	rs73061672	Naive DN (CD4-CI	7	8326430
ebi-a-GCST90001566	rs149868216	Naive DN (CD4-CI	8	89666365
ebi-a-GCST90001566	rs7036108	Naive DN (CD4-CI	9	107284126
ebi-a-GCST90001566	rs34406534	Naive DN (CD4-CI	9	11740444
ebi-a-GCST90001566	rs10821586	Naive DN (CD4-CI	10	61519938
ebi-a-GCST90001566	rs9599032	Naive DN (CD4-CI	13	66450470
ebi-a-GCST90001566	rs35761894	Naive DN (CD4-CI	13	70921568
ebi-a-GCST90001566	rs113911480	Naive DN (CD4-CI	14	29113167
ebi-a-GCST90001566	rs12907798	Naive DN (CD4-CI	15	36349479
ebi-a-GCST90001566	rs17315924	Naive DN (CD4-CI	15	80725065
ebi-a-GCST90001566	rs4784694	Naive DN (CD4-CI	16	56631722
ebi-a-GCST90001567	rs332818	Naive DN (CD4-CI	1	61679283
ebi-a-GCST90001567	rs1252165	Naive DN (CD4-CI	1	237168044
ebi-a-GCST90001567	rs114187856	Naive DN (CD4-CI	2	240939082
ebi-a-GCST90001567	rs76313555	Naive DN (CD4-CI	3	59914042
ebi-a-GCST90001567	rs7699349	Naive DN (CD4-CI	4	149134594
ebi-a-GCST90001567	rs13359589	Naive DN (CD4-CI	5	73116673
ebi-a-GCST90001567	rs141935759	Naive DN (CD4-CI	6	145631468
ebi-a-GCST90001567	rs148768795	Naive DN (CD4-CI	6	150715047
ebi-a-GCST90001567	rs1264345	Naive DN (CD4-CI	6	30800068
ebi-a-GCST90001567	rs140929446	Naive DN (CD4-CI	7	57131203
ebi-a-GCST90001567	rs17779181	Naive DN (CD4-CI	7	23640174
ebi-a-GCST90001567	rs12537183	Naive DN (CD4-CI	7	153543926
ebi-a-GCST90001567	rs2331387	Naive DN (CD4-CI	7	45898582
ebi-a-GCST90001567	rs4386870	Naive DN (CD4-CI	7	22747751
ebi-a-GCST90001567	rs149868216	Naive DN (CD4-CI	8	89666365
ebi-a-GCST90001567	rs4504704	Naive DN (CD4-CI	9	78567264
ebi-a-GCST90001567	rs2988087	Naive DN (CD4-CI	10	32399394
ebi-a-GCST90001567	rs755280	Naive DN (CD4-CI	12	95116986
ebi-a-GCST90001567	rs1290035	Naive DN (CD4-CI	14	69856859
ebi-a-GCST90001567	rs139496000	Naive DN (CD4-CI	15	64652871
ebi-a-GCST90001567	rs13339407	Naive DN (CD4-CI	16	78156295
ebi-a-GCST90001567	rs17608925	Naive DN (CD4-CI	17	38082831
ebi-a-GCST90001567	rs55748312	Naive DN (CD4-CI	17	70184940
ebi-a-GCST90001568	rs1679937	Naive DN (CD4-CI	1	53595255
ebi-a-GCST90001568	rs57726666	Naive DN (CD4-CI	2	16203326
ebi-a-GCST90001568	rs4832054	Naive DN (CD4-CI	2	87058580
ebi-a-GCST90001568	rs73150883	Naive DN (CD4-CI	3	146048618
ebi-a-GCST90001568	rs116550072	Naive DN (CD4-CI	5	150605036
ebi-a-GCST90001568	rs2523614	Naive DN (CD4-CI	6	31320654
ebi-a-GCST90001568	rs3819288	Naive DN (CD4-CI	6	31322694

ebi-a-GCST90001568	rs73061672	Naive DN (CD4-CI	7	8326430
ebi-a-GCST90001568	rs13235626	Naive DN (CD4-CI	7	21281534
ebi-a-GCST90001568	rs12537183	Naive DN (CD4-CI	7	153543926
ebi-a-GCST90001568	rs117300256	Naive DN (CD4-CI	8	15118856
ebi-a-GCST90001568	rs79416719	Naive DN (CD4-CI	8	76291201
ebi-a-GCST90001568	rs149868216	Naive DN (CD4-CI	8	89666365
ebi-a-GCST90001568	rs77986171	Naive DN (CD4-CI	10	54725683
ebi-a-GCST90001568	rs10836189	Naive DN (CD4-CI	11	34364512
ebi-a-GCST90001568	rs7956917	Naive DN (CD4-CI	12	115792537
ebi-a-GCST90001568	rs73084801	Naive DN (CD4-CI	12	30233867
ebi-a-GCST90001568	rs35761894	Naive DN (CD4-CI	13	70921568
ebi-a-GCST90001568	rs9599032	Naive DN (CD4-CI	13	66450470
ebi-a-GCST90001568	rs664597	Naive DN (CD4-CI	13	37440708
ebi-a-GCST90001568	rs17315924	Naive DN (CD4-CI	15	80725065
ebi-a-GCST90001568	rs112522622	Naive DN (CD4-CI	16	51946807
ebi-a-GCST90001568	rs75858298	Naive DN (CD4-CI	16	59757006
ebi-a-GCST90001568	rs117020416	Naive DN (CD4-CI	17	57520937
ebi-a-GCST90001569	rs541367304	EM DN (CD4-CD8	1	198283058
ebi-a-GCST90001569	rs111902980	EM DN (CD4-CD8	1	157954450
ebi-a-GCST90001569	rs76685729	EM DN (CD4-CD8	2	188005200
ebi-a-GCST90001569	rs6710361	EM DN (CD4-CD8	2	87047145
ebi-a-GCST90001569	rs142885636	EM DN (CD4-CD8	2	73173482
ebi-a-GCST90001569	rs11124150	EM DN (CD4-CD8	2	98905012
ebi-a-GCST90001569	rs13170001	EM DN (CD4-CD8	5	2685550
ebi-a-GCST90001569	rs78522661	EM DN (CD4-CD8	5	179850567
ebi-a-GCST90001569	rs10947785	EM DN (CD4-CD8	6	39132818
ebi-a-GCST90001569	rs113500908	EM DN (CD4-CD8	6	100774499
ebi-a-GCST90001569	rs139249541	EM DN (CD4-CD8	6	102294761
ebi-a-GCST90001569	rs11980500	EM DN (CD4-CD8	7	14882428
ebi-a-GCST90001569	rs577044375	EM DN (CD4-CD8	9	138170904
ebi-a-GCST90001569	rs7869045	EM DN (CD4-CD8	9	124584495
ebi-a-GCST90001569	rs1573041	EM DN (CD4-CD8	10	135091802
ebi-a-GCST90001569	rs569669132	EM DN (CD4-CD8	15	78218071
ebi-a-GCST90001569	rs141519401	EM DN (CD4-CD8	16	14491395
ebi-a-GCST90001569	rs9896155	EM DN (CD4-CD8	17	33794697
ebi-a-GCST90001569	rs117142611	EM DN (CD4-CD8	19	48128615
ebi-a-GCST90001569	rs437936	EM DN (CD4-CD8	21	43364569
ebi-a-GCST90001569	rs9647225	EM DN (CD4-CD8	21	15259953
ebi-a-GCST90001570	rs2126332	EM DN (CD4-CD8	1	226705690
ebi-a-GCST90001570	rs34645541	EM DN (CD4-CD8	1	195875605
ebi-a-GCST90001570	rs111902980	EM DN (CD4-CD8	1	157954450
ebi-a-GCST90001570	rs61779793	EM DN (CD4-CD8	1	40193589
ebi-a-GCST90001570	rs7530076	EM DN (CD4-CD8	1	195825276
ebi-a-GCST90001570	rs541367304	EM DN (CD4-CD8	1	198283058
ebi-a-GCST90001570	rs1976208	EM DN (CD4-CD8	1	82322706
ebi-a-GCST90001570	rs6659397	EM DN (CD4-CD8	1	206796165
ebi-a-GCST90001570	rs12133465	EM DN (CD4-CD8	1	195430336
ebi-a-GCST90001570	rs6751481	EM DN (CD4-CD8	2	38897810

ebi-a-GCST90001570	rs6729180	EM DN (CD4-CD8	2	38920621
ebi-a-GCST90001570	rs6782825	EM DN (CD4-CD8	3	24484006
ebi-a-GCST90001570	rs1093425	EM DN (CD4-CD8	6	101417133
ebi-a-GCST90001570	rs9265144	EM DN (CD4-CD8	6	31288202
ebi-a-GCST90001570	rs6935399	EM DN (CD4-CD8	6	151026312
ebi-a-GCST90001570	rs9274712	EM DN (CD4-CD8	6	32637298
ebi-a-GCST90001570	rs7809394	EM DN (CD4-CD8	7	55145382
ebi-a-GCST90001570	rs112361235	EM DN (CD4-CD8	7	23599680
ebi-a-GCST90001570	rs6954002	EM DN (CD4-CD8	7	152739128
ebi-a-GCST90001570	rs117099260	EM DN (CD4-CD8	8	116338265
ebi-a-GCST90001570	rs116877116	EM DN (CD4-CD8	8	135490341
ebi-a-GCST90001570	rs12115038	EM DN (CD4-CD8	8	19929781
ebi-a-GCST90001570	rs4871042	EM DN (CD4-CD8	8	121144851
ebi-a-GCST90001570	rs117470554	EM DN (CD4-CD8	9	16436647
ebi-a-GCST90001570	rs74120911	EM DN (CD4-CD8	10	21616211
ebi-a-GCST90001570	rs827338	EM DN (CD4-CD8	10	72674619
ebi-a-GCST90001570	rs75679242	EM DN (CD4-CD8	11	44090370
ebi-a-GCST90001570	rs77924860	EM DN (CD4-CD8	11	14032701
ebi-a-GCST90001570	rs117301997	EM DN (CD4-CD8	14	46761853
ebi-a-GCST90001570	rs141687469	EM DN (CD4-CD8	17	38885512
ebi-a-GCST90001570	rs5756946	EM DN (CD4-CD8	22	38570219
ebi-a-GCST90001571	rs116230798	EM DN (CD4-CD8	1	95254974
ebi-a-GCST90001571	rs541367304	EM DN (CD4-CD8	1	198283058
ebi-a-GCST90001571	rs111902980	EM DN (CD4-CD8	1	157954450
ebi-a-GCST90001571	rs34546581	EM DN (CD4-CD8	2	87021659
ebi-a-GCST90001571	rs28555522	EM DN (CD4-CD8	3	141557676
ebi-a-GCST90001571	rs77784181	EM DN (CD4-CD8	4	130189351
ebi-a-GCST90001571	rs4583837	EM DN (CD4-CD8	4	108003824
ebi-a-GCST90001571	rs78522661	EM DN (CD4-CD8	5	179850567
ebi-a-GCST90001571	rs113075410	EM DN (CD4-CD8	6	101435600
ebi-a-GCST90001571	rs13210358	EM DN (CD4-CD8	6	102272234
ebi-a-GCST90001571	rs55781756	EM DN (CD4-CD8	6	32795706
ebi-a-GCST90001571	rs113500908	EM DN (CD4-CD8	6	100774499
ebi-a-GCST90001571	rs10225877	EM DN (CD4-CD8	7	55150822
ebi-a-GCST90001571	rs76747637	EM DN (CD4-CD8	7	79800654
ebi-a-GCST90001571	rs28647367	EM DN (CD4-CD8	8	142106083
ebi-a-GCST90001571	rs116877116	EM DN (CD4-CD8	8	135490341
ebi-a-GCST90001571	rs75679242	EM DN (CD4-CD8	11	44090370
ebi-a-GCST90001571	rs78118522	EM DN (CD4-CD8	11	107707464
ebi-a-GCST90001571	rs11180778	EM DN (CD4-CD8	12	76371057
ebi-a-GCST90001571	rs527345021	EM DN (CD4-CD8	13	107465525
ebi-a-GCST90001571	rs11635763	EM DN (CD4-CD8	15	27177182
ebi-a-GCST90001571	rs141519401	EM DN (CD4-CD8	16	14491395
ebi-a-GCST90001571	rs9896155	EM DN (CD4-CD8	17	33794697
ebi-a-GCST90001571	rs140247402	EM DN (CD4-CD8	17	1235562
ebi-a-GCST90001571	rs437936	EM DN (CD4-CD8	21	43364569
ebi-a-GCST90001572	rs9660907	TD DN (CD4-CD8-	1	245039866
ebi-a-GCST90001572	rs4497244	TD DN (CD4-CD8-	1	197997953

ebi-a-GCST90001572	rs1891587	TD DN (CD4-CD8·	1	190077813
ebi-a-GCST90001572	rs6431734	TD DN (CD4-CD8·	2	15874207
ebi-a-GCST90001572	rs6751481	TD DN (CD4-CD8·	2	38897810
ebi-a-GCST90001572	rs6729180	TD DN (CD4-CD8·	2	38920621
ebi-a-GCST90001572	rs6755452	TD DN (CD4-CD8·	2	87035076
ebi-a-GCST90001572	rs36060603	TD DN (CD4-CD8·	3	78637361
ebi-a-GCST90001572	rs10003087	TD DN (CD4-CD8·	4	172937884
ebi-a-GCST90001572	rs72731805	TD DN (CD4-CD8·	4	147400930
ebi-a-GCST90001572	rs114588192	TD DN (CD4-CD8·	5	146490228
ebi-a-GCST90001572	rs78513507	TD DN (CD4-CD8·	5	98468131
ebi-a-GCST90001572	rs2442720	TD DN (CD4-CD8·	6	31320277
ebi-a-GCST90001572	rs9461666	TD DN (CD4-CD8·	6	31207763
ebi-a-GCST90001572	rs7744522	TD DN (CD4-CD8·	6	11109693
ebi-a-GCST90001572	rs61126090	TD DN (CD4-CD8·	6	32586382
ebi-a-GCST90001572	rs75865132	TD DN (CD4-CD8·	7	27625612
ebi-a-GCST90001572	rs117257206	TD DN (CD4-CD8·	8	120880964
ebi-a-GCST90001572	rs191991810	TD DN (CD4-CD8·	12	38160865
ebi-a-GCST90001572	rs142576649	TD DN (CD4-CD8·	12	130856799
ebi-a-GCST90001572	rs17775026	TD DN (CD4-CD8·	16	26221619
ebi-a-GCST90001572	rs2242597	TD DN (CD4-CD8·	17	34245629
ebi-a-GCST90001572	rs10422969	TD DN (CD4-CD8·	19	30096708
ebi-a-GCST90001573	rs6659397	TD DN (CD4-CD8·	1	206796165
ebi-a-GCST90001573	rs2126332	TD DN (CD4-CD8·	1	226705690
ebi-a-GCST90001573	rs7530076	TD DN (CD4-CD8·	1	195825276
ebi-a-GCST90001573	rs111902980	TD DN (CD4-CD8·	1	157954450
ebi-a-GCST90001573	rs12133465	TD DN (CD4-CD8·	1	195430336
ebi-a-GCST90001573	rs541367304	TD DN (CD4-CD8·	1	198283058
ebi-a-GCST90001573	rs12712610	TD DN (CD4-CD8·	2	38897249
ebi-a-GCST90001573	rs7583259	TD DN (CD4-CD8·	2	38921934
ebi-a-GCST90001573	rs6782825	TD DN (CD4-CD8·	3	24484006
ebi-a-GCST90001573	rs34513732	TD DN (CD4-CD8·	3	140621480
ebi-a-GCST90001573	rs72729850	TD DN (CD4-CD8·	4	147300773
ebi-a-GCST90001573	rs35139945	TD DN (CD4-CD8·	6	32626267
ebi-a-GCST90001573	rs1265224	TD DN (CD4-CD8·	6	4514319
ebi-a-GCST90001573	rs7809394	TD DN (CD4-CD8·	7	55145382
ebi-a-GCST90001573	rs112374765	TD DN (CD4-CD8·	7	157846504
ebi-a-GCST90001573	rs10738134	TD DN (CD4-CD8·	8	121168145
ebi-a-GCST90001573	rs116877116	TD DN (CD4-CD8·	8	135490341
ebi-a-GCST90001573	rs12115038	TD DN (CD4-CD8·	8	19929781
ebi-a-GCST90001573	rs10987246	TD DN (CD4-CD8·	9	129079854
ebi-a-GCST90001573	rs117470554	TD DN (CD4-CD8·	9	16436647
ebi-a-GCST90001573	rs7085637	TD DN (CD4-CD8·	10	21616206
ebi-a-GCST90001573	rs75679242	TD DN (CD4-CD8·	11	44090370
ebi-a-GCST90001573	rs117694717	TD DN (CD4-CD8·	13	106560589
ebi-a-GCST90001573	rs80142288	TD DN (CD4-CD8·	13	75648872
ebi-a-GCST90001573	rs72676662	TD DN (CD4-CD8·	14	47362685
ebi-a-GCST90001573	rs141687469	TD DN (CD4-CD8·	17	38885512
ebi-a-GCST90001573	rs368478932	TD DN (CD4-CD8·	17	62913595

ebi-a-GCST90001573	rs5756946	TD DN (CD4-CD8-	22	38570219
ebi-a-GCST90001574	rs114892491	TD DN (CD4-CD8-	1	156921889
ebi-a-GCST90001574	rs9660907	TD DN (CD4-CD8-	1	245039866
ebi-a-GCST90001574	rs7530076	TD DN (CD4-CD8-	1	195825276
ebi-a-GCST90001574	rs541367304	TD DN (CD4-CD8-	1	198283058
ebi-a-GCST90001574	rs57305384	TD DN (CD4-CD8-	1	229002885
ebi-a-GCST90001574	rs10920583	TD DN (CD4-CD8-	1	190099416
ebi-a-GCST90001574	rs6755452	TD DN (CD4-CD8-	2	87035076
ebi-a-GCST90001574	rs78911530	TD DN (CD4-CD8-	2	67543324
ebi-a-GCST90001574	rs6729180	TD DN (CD4-CD8-	2	38920621
ebi-a-GCST90001574	rs6751481	TD DN (CD4-CD8-	2	38897810
ebi-a-GCST90001574	rs36060603	TD DN (CD4-CD8-	3	78637361
ebi-a-GCST90001574	rs10003087	TD DN (CD4-CD8-	4	172937884
ebi-a-GCST90001574	rs78513507	TD DN (CD4-CD8-	5	98468131
ebi-a-GCST90001574	rs142421834	TD DN (CD4-CD8-	5	52915612
ebi-a-GCST90001574	rs76673582	TD DN (CD4-CD8-	5	149696943
ebi-a-GCST90001574	rs9266797	TD DN (CD4-CD8-	6	31363510
ebi-a-GCST90001574	rs111983490	TD DN (CD4-CD8-	6	32205324
ebi-a-GCST90001574	rs28584364	TD DN (CD4-CD8-	6	32512048
ebi-a-GCST90001574	rs17692789	TD DN (CD4-CD8-	7	122641368
ebi-a-GCST90001574	rs117481225	TD DN (CD4-CD8-	7	146971931
ebi-a-GCST90001574	rs147717252	TD DN (CD4-CD8-	7	27567035
ebi-a-GCST90001574	rs146907982	TD DN (CD4-CD8-	9	120231514
ebi-a-GCST90001574	rs35706962	TD DN (CD4-CD8-	11	106402698
ebi-a-GCST90001574	rs116088408	TD DN (CD4-CD8-	12	27632799
ebi-a-GCST90001574	rs10876180	TD DN (CD4-CD8-	12	51836616
ebi-a-GCST90001574	rs11669637	TD DN (CD4-CD8-	19	12690226
ebi-a-GCST90001575	rs72721071	Naive CD4+ %T c	1	94170616
ebi-a-GCST90001575	rs17583875	Naive CD4+ %T c	1	197924770
ebi-a-GCST90001575	rs6576734	Naive CD4+ %T c	1	85383442
ebi-a-GCST90001575	rs114825362	Naive CD4+ %T c	1	101526164
ebi-a-GCST90001575	rs150607181	Naive CD4+ %T c	1	239680551
ebi-a-GCST90001575	rs145775506	Naive CD4+ %T c	2	209850246
ebi-a-GCST90001575	rs72613709	Naive CD4+ %T c	2	197060767
ebi-a-GCST90001575	rs150981153	Naive CD4+ %T c	2	57533997
ebi-a-GCST90001575	rs12712610	Naive CD4+ %T c	2	38897249
ebi-a-GCST90001575	rs9990031	Naive CD4+ %T c	3	129437836
ebi-a-GCST90001575	rs7636470	Naive CD4+ %T c	3	186606581
ebi-a-GCST90001575	rs4507432	Naive CD4+ %T c	4	1748200
ebi-a-GCST90001575	rs36088117	Naive CD4+ %T c	4	11877955
ebi-a-GCST90001575	rs1379152	Naive CD4+ %T c	4	64498422
ebi-a-GCST90001575	rs140125890	Naive CD4+ %T c	4	40884597
ebi-a-GCST90001575	rs57810789	Naive CD4+ %T c	4	185350356
ebi-a-GCST90001575	rs7715223	Naive CD4+ %T c	5	15900124
ebi-a-GCST90001575	rs76972929	Naive CD4+ %T c	5	153357272
ebi-a-GCST90001575	rs151100670	Naive CD4+ %T c	5	141405792
ebi-a-GCST90001575	rs9269702	Naive CD4+ %T c	6	32547143
ebi-a-GCST90001575	rs4959365	Naive CD4+ %T c	6	6026750

ebi-a-GCST90001575	rs2516718	Naive CD4+ %T c	6	30223903
ebi-a-GCST90001575	rs4959028	Naive CD4+ %T c	6	32383138
ebi-a-GCST90001575	rs77253141	Naive CD4+ %T c	8	24504854
ebi-a-GCST90001575	rs7042017	Naive CD4+ %T c	9	92779466
ebi-a-GCST90001575	rs76139558	Naive CD4+ %T c	10	2787360
ebi-a-GCST90001575	rs1849779	Naive CD4+ %T c	12	43674787
ebi-a-GCST90001575	rs146329634	Naive CD4+ %T c	14	89433599
ebi-a-GCST90001575	rs980169	Naive CD4+ %T c	15	96720568
ebi-a-GCST90001575	rs66679234	Naive CD4+ %T c	17	4224639
ebi-a-GCST90001575	rs4792002	Naive CD4+ %T c	17	7853356
ebi-a-GCST90001575	rs75010615	Naive CD4+ %T c	17	38896451
ebi-a-GCST90001575	rs157386	Naive CD4+ %T c	17	19699121
ebi-a-GCST90001575	rs79538978	Naive CD4+ %T c	18	54960800
ebi-a-GCST90001575	rs78137572	Naive CD4+ %T c	19	15677349
ebi-a-GCST90001575	rs6046769	Naive CD4+ %T c	20	20238128
ebi-a-GCST90001575	rs5767274	Naive CD4+ %T c	22	46980774
ebi-a-GCST90001576	rs1384161	Transitional %B cel	1	99311623
ebi-a-GCST90001576	rs2375523	Transitional %B cel	1	65152941
ebi-a-GCST90001576	rs1473575	Transitional %B cel	1	242619029
ebi-a-GCST90001576	rs62157336	Transitional %B cel	2	79220714
ebi-a-GCST90001576	rs145834662	Transitional %B cel	2	85296873
ebi-a-GCST90001576	rs76112278	Transitional %B cel	4	15915069
ebi-a-GCST90001576	rs6853622	Transitional %B cel	4	83375719
ebi-a-GCST90001576	rs29001568	Transitional %B cel	6	32447341
ebi-a-GCST90001576	rs9394619	Transitional %B cel	6	39574377
ebi-a-GCST90001576	rs28491938	Transitional %B cel	9	41980474
ebi-a-GCST90001576	rs117249531	Transitional %B cel	10	81026095
ebi-a-GCST90001576	rs190371014	Transitional %B cel	10	24415064
ebi-a-GCST90001576	rs35060907	Transitional %B cel	10	132636158
ebi-a-GCST90001576	rs59879144	Transitional %B cel	12	75564449
ebi-a-GCST90001576	rs117943886	Transitional %B cel	13	95198601
ebi-a-GCST90001576	rs4906270	Transitional %B cel	14	103364737
ebi-a-GCST90001576	rs2216762	Transitional %B cel	16	84759446
ebi-a-GCST90001577	rs1294253	Transitional AC	1	233503875
ebi-a-GCST90001577	rs116285595	Transitional AC	1	200858159
ebi-a-GCST90001577	rs1384162	Transitional AC	1	99311831
ebi-a-GCST90001577	rs140088532	Transitional AC	2	105581937
ebi-a-GCST90001577	rs78188887	Transitional AC	2	155636739
ebi-a-GCST90001577	rs3845834	Transitional AC	2	219236436
ebi-a-GCST90001577	rs113965271	Transitional AC	2	128428350
ebi-a-GCST90001577	rs733555	Transitional AC	2	16464227
ebi-a-GCST90001577	rs374727947	Transitional AC	3	163801921
ebi-a-GCST90001577	rs34161184	Transitional AC	3	74778821
ebi-a-GCST90001577	rs35949974	Transitional AC	4	42452429
ebi-a-GCST90001577	rs76112278	Transitional AC	4	15915069
ebi-a-GCST90001577	rs985222	Transitional AC	4	5229884
ebi-a-GCST90001577	rs75512885	Transitional AC	5	158317602
ebi-a-GCST90001577	rs929786	Transitional AC	5	143071654

ebi-a-GCST90001577	rs77886121	Transitional AC	5	153628389
ebi-a-GCST90001577	rs143397127	Transitional AC	7	12847391
ebi-a-GCST90001577	rs12535189	Transitional AC	7	121174063
ebi-a-GCST90001577	rs1639898	Transitional AC	7	2204473
ebi-a-GCST90001577	rs55798372	Transitional AC	8	3638250
ebi-a-GCST90001577	rs2395904	Transitional AC	8	130617819
ebi-a-GCST90001577	rs16868356	Transitional AC	8	102770464
ebi-a-GCST90001577	rs190371014	Transitional AC	10	24415064
ebi-a-GCST90001577	rs12799241	Transitional AC	11	119980729
ebi-a-GCST90001577	rs75505900	Transitional AC	11	115433689
ebi-a-GCST90001577	rs11159480	Transitional AC	14	81466662
ebi-a-GCST90001577	rs146347064	Transitional AC	16	8263526
ebi-a-GCST90001577	rs117518951	Transitional AC	16	55449193
ebi-a-GCST90001577	rs8045732	Transitional AC	16	88060989
ebi-a-GCST90001577	rs12939565	Transitional AC	17	38038389
ebi-a-GCST90001577	rs8092698	Transitional AC	18	75135714
ebi-a-GCST90001577	rs78943781	Transitional AC	19	32443306
ebi-a-GCST90001577	rs655580	Transitional AC	19	53039110
ebi-a-GCST90001578	rs10796440	Transitional %lymp	1	18491472
ebi-a-GCST90001578	rs1384162	Transitional %lymp	1	99311831
ebi-a-GCST90001578	rs10158474	Transitional %lymp	1	96015938
ebi-a-GCST90001578	rs140088532	Transitional %lymp	2	105581937
ebi-a-GCST90001578	rs62157336	Transitional %lymp	2	79220714
ebi-a-GCST90001578	rs733555	Transitional %lymp	2	16464227
ebi-a-GCST90001578	rs74720848	Transitional %lymp	4	164477154
ebi-a-GCST90001578	rs72673262	Transitional %lymp	4	126225945
ebi-a-GCST90001578	rs76112278	Transitional %lymp	4	15915069
ebi-a-GCST90001578	rs77886121	Transitional %lymp	5	153628389
ebi-a-GCST90001578	rs75512885	Transitional %lymp	5	158317602
ebi-a-GCST90001578	rs17206161	Transitional %lymp	6	32665490
ebi-a-GCST90001578	rs1639898	Transitional %lymp	7	2204473
ebi-a-GCST90001578	rs55798372	Transitional %lymp	8	3638250
ebi-a-GCST90001578	rs7005227	Transitional %lymp	8	130597858
ebi-a-GCST90001578	rs12360242	Transitional %lymp	10	132652504
ebi-a-GCST90001578	rs190371014	Transitional %lymp	10	24415064
ebi-a-GCST90001578	rs11068290	Transitional %lymp	12	110228055
ebi-a-GCST90001578	rs59879144	Transitional %lymp	12	75564449
ebi-a-GCST90001578	rs117943886	Transitional %lymp	13	95198601
ebi-a-GCST90001578	rs3811315	Transitional %lymp	14	22499870
ebi-a-GCST90001578	rs72805142	Transitional %lymp	16	85878540
ebi-a-GCST90001578	rs2216762	Transitional %lymp	16	84759446
ebi-a-GCST90001578	rs146347064	Transitional %lymp	16	8263526
ebi-a-GCST90001578	rs11657531	Transitional %lymp	17	2731370
ebi-a-GCST90001578	rs12939565	Transitional %lymp	17	38038389
ebi-a-GCST90001578	rs60521331	Transitional %lymp	17	8639905
ebi-a-GCST90001578	rs329966	Transitional %lymp	19	53070617
ebi-a-GCST90001579	rs12404245	CD14- CD16+ mon	1	77952299
ebi-a-GCST90001579	rs115383270	CD14- CD16+ mon	1	161531340

ebi-a-GCST90001579	rs74341264	CD14- CD16+ mon	1	161561151
ebi-a-GCST90001579	rs112150611	CD14- CD16+ mon	1	18034211
ebi-a-GCST90001579	rs116662407	CD14- CD16+ mon	1	18064955
ebi-a-GCST90001579	rs6684716	CD14- CD16+ mon	1	166649601
ebi-a-GCST90001579	rs74578127	CD14- CD16+ mon	2	232980015
ebi-a-GCST90001579	rs3853483	CD14- CD16+ mon	5	141962502
ebi-a-GCST90001579	rs12055035	CD14- CD16+ mon	5	173542221
ebi-a-GCST90001579	rs77983030	CD14- CD16+ mon	9	74645450
ebi-a-GCST90001579	rs117671592	CD14- CD16+ mon	10	14470275
ebi-a-GCST90001579	rs144707945	CD14- CD16+ mon	11	115147742
ebi-a-GCST90001579	rs10833398	CD14- CD16+ mon	11	20860026
ebi-a-GCST90001579	rs143984211	CD14- CD16+ mon	11	395746
ebi-a-GCST90001579	rs139922750	CD14- CD16+ mon	12	64437951
ebi-a-GCST90001579	rs8026803	CD14- CD16+ mon	15	80260014
ebi-a-GCST90001579	rs473026	CD14- CD16+ mon	18	24363797
ebi-a-GCST90001579	rs139025334	CD14- CD16+ mon	20	48944963
ebi-a-GCST90001579	rs9984228	CD14- CD16+ mon	21	47484045
ebi-a-GCST90001580	rs74341264	CD14+ CD16+ mor	1	161561151
ebi-a-GCST90001580	rs115451299	CD14+ CD16+ mor	1	110012633
ebi-a-GCST90001580	rs185631377	CD14+ CD16+ mor	1	161031616
ebi-a-GCST90001580	rs71632979	CD14+ CD16+ mor	1	161526397
ebi-a-GCST90001580	rs139795227	CD14+ CD16+ mor	1	92842367
ebi-a-GCST90001580	rs71639910	CD14+ CD16+ mor	1	161937774
ebi-a-GCST90001580	rs3829793	CD14+ CD16+ mor	1	161194400
ebi-a-GCST90001580	rs147218533	CD14+ CD16+ mor	3	167152276
ebi-a-GCST90001580	rs112831211	CD14+ CD16+ mor	4	182944203
ebi-a-GCST90001580	rs78723624	CD14+ CD16+ mor	4	33430567
ebi-a-GCST90001580	rs9313620	CD14+ CD16+ mor	5	172669254
ebi-a-GCST90001580	rs144601865	CD14+ CD16+ mor	5	38258302
ebi-a-GCST90001580	rs13260841	CD14+ CD16+ mor	8	19431500
ebi-a-GCST90001580	rs77305308	CD14+ CD16+ mor	8	30170640
ebi-a-GCST90001580	rs72649637	CD14+ CD16+ mor	8	51887026
ebi-a-GCST90001580	rs877544	CD14+ CD16+ mor	9	113895745
ebi-a-GCST90001580	rs10970605	CD14+ CD16+ mor	9	31933307
ebi-a-GCST90001580	rs142005394	CD14+ CD16+ mor	11	17377369
ebi-a-GCST90001580	rs847888	CD14+ CD16+ mor	12	112151742
ebi-a-GCST90001580	rs71436541	CD14+ CD16+ mor	13	80448111
ebi-a-GCST90001580	rs11856889	CD14+ CD16+ mor	15	24443663
ebi-a-GCST90001580	rs913678	CD14+ CD16+ mor	20	48955424
ebi-a-GCST90001581	rs1338629	CD14- CD16- AC	1	164446649
ebi-a-GCST90001581	rs144126567	CD14- CD16- AC	1	161510516
ebi-a-GCST90001581	rs78327733	CD14- CD16- AC	1	241079307
ebi-a-GCST90001581	rs3014270	CD14- CD16- AC	1	227562378
ebi-a-GCST90001581	rs147699975	CD14- CD16- AC	2	213883128
ebi-a-GCST90001581	rs79657767	CD14- CD16- AC	2	38775196
ebi-a-GCST90001581	rs2324472	CD14- CD16- AC	4	24260048
ebi-a-GCST90001581	rs2562375	CD14- CD16- AC	5	132729281
ebi-a-GCST90001581	rs73161489	CD14- CD16- AC	5	86880856

ebi-a-GCST90001581	rs17540814	CD14- CD16- AC	6	93853881
ebi-a-GCST90001581	rs6921349	CD14- CD16- AC	6	166039211
ebi-a-GCST90001581	rs445	CD14- CD16- AC	7	92408370
ebi-a-GCST90001581	rs149241803	CD14- CD16- AC	7	39649629
ebi-a-GCST90001581	rs138772443	CD14- CD16- AC	9	139345466
ebi-a-GCST90001581	rs17296114	CD14- CD16- AC	10	32693485
ebi-a-GCST90001581	rs12301658	CD14- CD16- AC	12	22349620
ebi-a-GCST90001581	rs76478186	CD14- CD16- AC	12	68363738
ebi-a-GCST90001581	rs1800973	CD14- CD16- AC	12	69744014
ebi-a-GCST90001581	rs11865775	CD14- CD16- AC	16	5750222
ebi-a-GCST90001581	rs115758894	CD14- CD16- AC	18	53138692
ebi-a-GCST90001581	rs4806508	CD14- CD16- AC	19	54337523
ebi-a-GCST90001581	rs8122111	CD14- CD16- AC	20	46542662
ebi-a-GCST90001581	rs17406122	CD14- CD16- AC	20	7719687
ebi-a-GCST90001581	rs761570	CD14- CD16- AC	20	912626
ebi-a-GCST90001581	rs2205245	CD14- CD16- AC	21	29959796
ebi-a-GCST90001582	rs78355921	CD14+ CD16- mon	1	159941852
ebi-a-GCST90001582	rs150649461	CD14+ CD16- mon	1	92925654
ebi-a-GCST90001582	rs17465420	CD14+ CD16- mon	1	38398588
ebi-a-GCST90001582	rs2904118	CD14+ CD16- mon	2	56402320
ebi-a-GCST90001582	rs1375493	CD14+ CD16- mon	2	182323766
ebi-a-GCST90001582	rs183716470	CD14+ CD16- mon	3	81299703
ebi-a-GCST90001582	rs58943387	CD14+ CD16- mon	3	190720967
ebi-a-GCST90001582	rs310730	CD14+ CD16- mon	3	4328811
ebi-a-GCST90001582	rs536943	CD14+ CD16- mon	3	173213883
ebi-a-GCST90001582	rs189962728	CD14+ CD16- mon	4	58124445
ebi-a-GCST90001582	rs13121143	CD14+ CD16- mon	4	190199873
ebi-a-GCST90001582	rs10029045	CD14+ CD16- mon	4	36008336
ebi-a-GCST90001582	rs12501003	CD14+ CD16- mon	4	92510918
ebi-a-GCST90001582	rs56010301	CD14+ CD16- mon	5	52645510
ebi-a-GCST90001582	rs445	CD14+ CD16- mon	7	92408370
ebi-a-GCST90001582	rs10094039	CD14+ CD16- mon	8	130589676
ebi-a-GCST90001582	rs111463078	CD14+ CD16- mon	8	2350689
ebi-a-GCST90001582	rs613224	CD14+ CD16- mon	8	2803266
ebi-a-GCST90001582	rs2418135	CD14+ CD16- mon	9	113901309
ebi-a-GCST90001582	rs11177104	CD14+ CD16- mon	12	68605883
ebi-a-GCST90001582	rs9599503	CD14+ CD16- mon	13	35314819
ebi-a-GCST90001582	rs41331448	CD14+ CD16- mon	13	21289312
ebi-a-GCST90001582	rs149927978	CD14+ CD16- mon	14	105638349
ebi-a-GCST90001582	rs2247998	CD14+ CD16- mon	16	85801445
ebi-a-GCST90001582	rs58581511	CD14+ CD16- mon	17	57950621
ebi-a-GCST90001582	rs9747839	CD14+ CD16- mon	17	81084522
ebi-a-GCST90001582	rs8076666	CD14+ CD16- mon	17	78223817
ebi-a-GCST90001582	rs2120231	CD14+ CD16- mon	17	1015996
ebi-a-GCST90001582	rs55779804	CD14+ CD16- mon	19	6371660
ebi-a-GCST90001582	rs28437286	CD14+ CD16- mon	21	43063279
ebi-a-GCST90001582	rs118111037	CD14+ CD16- mon	22	35958024
ebi-a-GCST90001583	rs115451299	Monocyte AC	1	110012633

ebi-a-GCST90001583	rs55971447	Monocyte AC	1	161515326
ebi-a-GCST90001583	rs150649461	Monocyte AC	1	92925654
ebi-a-GCST90001583	rs1896857	Monocyte AC	2	198164530
ebi-a-GCST90001583	rs1375493	Monocyte AC	2	182323766
ebi-a-GCST90001583	rs6708174	Monocyte AC	2	134344243
ebi-a-GCST90001583	rs10192574	Monocyte AC	2	56278216
ebi-a-GCST90001583	rs183716470	Monocyte AC	3	81299703
ebi-a-GCST90001583	rs189962728	Monocyte AC	4	58124445
ebi-a-GCST90001583	rs13121143	Monocyte AC	4	190199873
ebi-a-GCST90001583	rs12501003	Monocyte AC	4	92510918
ebi-a-GCST90001583	rs445	Monocyte AC	7	92408370
ebi-a-GCST90001583	rs145530955	Monocyte AC	7	65604757
ebi-a-GCST90001583	rs2541899	Monocyte AC	7	35423859
ebi-a-GCST90001583	rs77765378	Monocyte AC	8	77490437
ebi-a-GCST90001583	rs78805826	Monocyte AC	8	49196586
ebi-a-GCST90001583	rs75791731	Monocyte AC	8	135163467
ebi-a-GCST90001583	rs12346499	Monocyte AC	9	113902881
ebi-a-GCST90001583	rs1249347	Monocyte AC	10	28612860
ebi-a-GCST90001583	rs139761995	Monocyte AC	11	36967796
ebi-a-GCST90001583	rs11177104	Monocyte AC	12	68605883
ebi-a-GCST90001583	rs11061521	Monocyte AC	12	131900390
ebi-a-GCST90001583	rs116981568	Monocyte AC	13	28964708
ebi-a-GCST90001583	rs9599503	Monocyte AC	13	35314819
ebi-a-GCST90001583	rs142584082	Monocyte AC	14	98059388
ebi-a-GCST90001583	rs28648481	Monocyte AC	14	97964046
ebi-a-GCST90001583	rs2120231	Monocyte AC	17	1015996
ebi-a-GCST90001583	rs58581511	Monocyte AC	17	57950621
ebi-a-GCST90001583	rs28437286	Monocyte AC	21	43063279
ebi-a-GCST90001584	rs74975395	CD14- CD16+ mon	1	166026114
ebi-a-GCST90001584	rs115383270	CD14- CD16+ mon	1	161531340
ebi-a-GCST90001584	rs141826566	CD14- CD16+ mon	1	30332508
ebi-a-GCST90001584	rs74915645	CD14- CD16+ mon	2	9134527
ebi-a-GCST90001584	rs115440084	CD14- CD16+ mon	2	150006610
ebi-a-GCST90001584	rs155144	CD14- CD16+ mon	2	182315630
ebi-a-GCST90001584	rs12637779	CD14- CD16+ mon	3	109439443
ebi-a-GCST90001584	rs7613930	CD14- CD16+ mon	3	188340672
ebi-a-GCST90001584	rs185857822	CD14- CD16+ mon	5	1285334
ebi-a-GCST90001584	rs1202098	CD14- CD16+ mon	6	73301721
ebi-a-GCST90001584	rs9357234	CD14- CD16+ mon	6	37030217
ebi-a-GCST90001584	rs7834258	CD14- CD16+ mon	8	13209667
ebi-a-GCST90001584	rs78829712	CD14- CD16+ mon	10	82443865
ebi-a-GCST90001584	rs143356597	CD14- CD16+ mon	11	588659
ebi-a-GCST90001584	rs144707945	CD14- CD16+ mon	11	115147742
ebi-a-GCST90001584	rs10765883	CD14- CD16+ mon	11	11658286
ebi-a-GCST90001584	rs73046238	CD14- CD16+ mon	12	2608522
ebi-a-GCST90001584	rs17200456	CD14- CD16+ mon	14	25243751
ebi-a-GCST90001584	rs4999522	CD14- CD16+ mon	14	95963238
ebi-a-GCST90001584	rs3826007	CD14- CD16+ mon	15	80263217

ebi-a-GCST90001584	rs316369	CD14- CD16+ mon	16	9728962
ebi-a-GCST90001584	rs4783204	CD14- CD16+ mon	16	85474706
ebi-a-GCST90001584	rs137976321	CD14- CD16+ mon	20	50183940
ebi-a-GCST90001585	rs71639910	CD14+ CD16+ mo	1	161937774
ebi-a-GCST90001585	rs74341264	CD14+ CD16+ mo	1	161561151
ebi-a-GCST90001585	rs3829793	CD14+ CD16+ mo	1	161194400
ebi-a-GCST90001585	rs71632979	CD14+ CD16+ mo	1	161526397
ebi-a-GCST90001585	rs138308733	CD14+ CD16+ mo	1	158788928
ebi-a-GCST90001585	rs185631377	CD14+ CD16+ mo	1	161031616
ebi-a-GCST90001585	rs11466597	CD14+ CD16+ mo	1	92185351
ebi-a-GCST90001585	rs7604795	CD14+ CD16+ mo	2	215457940
ebi-a-GCST90001585	rs75738537	CD14+ CD16+ mo	3	172984421
ebi-a-GCST90001585	rs147218533	CD14+ CD16+ mo	3	167152276
ebi-a-GCST90001585	rs114032392	CD14+ CD16+ mo	4	122320941
ebi-a-GCST90001585	rs6853013	CD14+ CD16+ mo	4	161408429
ebi-a-GCST90001585	rs112831211	CD14+ CD16+ mo	4	182944203
ebi-a-GCST90001585	rs77936882	CD14+ CD16+ mo	4	148945287
ebi-a-GCST90001585	rs1903578	CD14+ CD16+ mo	4	91255079
ebi-a-GCST90001585	rs141154517	CD14+ CD16+ mo	4	71103396
ebi-a-GCST90001585	rs2304647	CD14+ CD16+ mo	4	41646651
ebi-a-GCST90001585	rs34161492	CD14+ CD16+ mo	6	79237344
ebi-a-GCST90001585	rs76127744	CD14+ CD16+ mo	7	99531657
ebi-a-GCST90001585	rs445	CD14+ CD16+ mo	7	92408370
ebi-a-GCST90001585	rs77305308	CD14+ CD16+ mo	8	30170640
ebi-a-GCST90001585	rs72649637	CD14+ CD16+ mo	8	51887026
ebi-a-GCST90001585	rs10970605	CD14+ CD16+ mo	9	31933307
ebi-a-GCST90001585	rs56037947	CD14+ CD16+ mo	12	4104047
ebi-a-GCST90001585	rs847888	CD14+ CD16+ mo	12	112151742
ebi-a-GCST90001585	rs117302238	CD14+ CD16+ mo	15	75293128
ebi-a-GCST90001586	rs142340414	CD14+ CD16- mon	1	212967660
ebi-a-GCST90001586	rs659192	CD14+ CD16- mon	1	208200737
ebi-a-GCST90001586	rs4656993	CD14+ CD16- mon	1	161176137
ebi-a-GCST90001586	rs141826566	CD14+ CD16- mon	1	30332508
ebi-a-GCST90001586	rs111690368	CD14+ CD16- mon	1	91084546
ebi-a-GCST90001586	rs115383270	CD14+ CD16- mon	1	161531340
ebi-a-GCST90001586	rs74341264	CD14+ CD16- mon	1	161561151
ebi-a-GCST90001586	rs155144	CD14+ CD16- mon	2	182315630
ebi-a-GCST90001586	rs12637779	CD14+ CD16- mon	3	109439443
ebi-a-GCST90001586	rs149602828	CD14+ CD16- mon	4	43503165
ebi-a-GCST90001586	rs150125023	CD14+ CD16- mon	5	1812272
ebi-a-GCST90001586	rs13183256	CD14+ CD16- mon	5	37116496
ebi-a-GCST90001586	rs138743668	CD14+ CD16- mon	5	26070685
ebi-a-GCST90001586	rs139379948	CD14+ CD16- mon	7	103732660
ebi-a-GCST90001586	rs78047442	CD14+ CD16- mon	7	153317246
ebi-a-GCST90001586	rs56347556	CD14+ CD16- mon	8	29823363
ebi-a-GCST90001586	rs116915583	CD14+ CD16- mon	9	108747182
ebi-a-GCST90001586	rs79562353	CD14+ CD16- mon	10	17069964
ebi-a-GCST90001586	rs143984211	CD14+ CD16- mon	11	395746

ebi-a-GCST90001586	rs10833398	CD14+ CD16- mon	11	20860026
ebi-a-GCST90001586	rs76293503	CD14+ CD16- mon	12	124510950
ebi-a-GCST90001586	rs67760360	CD14+ CD16- mon	15	80260872
ebi-a-GCST90001586	rs4783204	CD14+ CD16- mon	16	85474706
ebi-a-GCST90001586	rs13330943	CD14+ CD16- mon	16	8615778
ebi-a-GCST90001586	rs62083033	CD14+ CD16- mon	17	68030340
ebi-a-GCST90001586	rs137976321	CD14+ CD16- mon	20	50183940
ebi-a-GCST90001587	rs111690368	CD16+ monocyte %	1	91084546
ebi-a-GCST90001587	rs74341264	CD16+ monocyte %	1	161561151
ebi-a-GCST90001587	rs4656993	CD16+ monocyte %	1	161176137
ebi-a-GCST90001587	rs659192	CD16+ monocyte %	1	208200737
ebi-a-GCST90001587	rs115383270	CD16+ monocyte %	1	161531340
ebi-a-GCST90001587	rs150451378	CD16+ monocyte %	2	4795580
ebi-a-GCST90001587	rs115440084	CD16+ monocyte %	2	150006610
ebi-a-GCST90001587	rs155144	CD16+ monocyte %	2	182315630
ebi-a-GCST90001587	rs7613930	CD16+ monocyte %	3	188340672
ebi-a-GCST90001587	rs12637779	CD16+ monocyte %	3	109439443
ebi-a-GCST90001587	rs13183256	CD16+ monocyte %	5	37116496
ebi-a-GCST90001587	rs150125023	CD16+ monocyte %	5	1812272
ebi-a-GCST90001587	rs138743668	CD16+ monocyte %	5	26070685
ebi-a-GCST90001587	rs139379948	CD16+ monocyte %	7	103732660
ebi-a-GCST90001587	rs79562353	CD16+ monocyte %	10	17069964
ebi-a-GCST90001587	rs143356597	CD16+ monocyte %	11	588659
ebi-a-GCST90001587	rs10765883	CD16+ monocyte %	11	11658286
ebi-a-GCST90001587	rs144707945	CD16+ monocyte %	11	115147742
ebi-a-GCST90001587	rs12582907	CD16+ monocyte %	12	130524797
ebi-a-GCST90001587	rs67760360	CD16+ monocyte %	15	80260872
ebi-a-GCST90001587	rs4783204	CD16+ monocyte %	16	85474706
ebi-a-GCST90001587	rs13330943	CD16+ monocyte %	16	8615778
ebi-a-GCST90001587	rs72982284	CD16+ monocyte %	18	77678240
ebi-a-GCST90001587	rs137976321	CD16+ monocyte %	20	50183940
ebi-a-GCST90001588	rs17113367	T/B	1	95785786
ebi-a-GCST90001588	rs72941136	T/B	1	82245693
ebi-a-GCST90001588	rs3738088	T/B	1	18554670
ebi-a-GCST90001588	rs7634218	T/B	3	188392916
ebi-a-GCST90001588	rs13066480	T/B	3	177432204
ebi-a-GCST90001588	rs2679580	T/B	3	8845344
ebi-a-GCST90001588	rs1523154	T/B	4	31904141
ebi-a-GCST90001588	rs191132013	T/B	4	32930199
ebi-a-GCST90001588	rs9368867	T/B	6	11760807
ebi-a-GCST90001588	rs34147569	T/B	6	71753094
ebi-a-GCST90001588	rs1557987	T/B	7	26591929
ebi-a-GCST90001588	rs150774187	T/B	7	100536678
ebi-a-GCST90001588	rs77233730	T/B	8	118635981
ebi-a-GCST90001588	rs7000289	T/B	8	130597230
ebi-a-GCST90001588	rs73520190	T/B	9	118765180
ebi-a-GCST90001588	rs116735405	T/B	10	119550308
ebi-a-GCST90001588	rs6585493	T/B	10	85429828

ebi-a-GCST90001588	rs4758148	T/B	11	5876630
ebi-a-GCST90001588	rs142089808	T/B	11	116171120
ebi-a-GCST90001588	rs7990182	T/B	13	110384132
ebi-a-GCST90001588	rs9520905	T/B	13	109146439
ebi-a-GCST90001588	rs12874404	T/B	13	108993494
ebi-a-GCST90001588	rs11627066	T/B	14	66304049
ebi-a-GCST90001588	rs4789817	T/B	17	80607989
ebi-a-GCST90001588	rs3894193	T/B	17	38125346
ebi-a-GCST90001588	rs9892810	T/B	17	2716155
ebi-a-GCST90001588	rs144426024	T/B	18	56897917
ebi-a-GCST90001588	rs2223650	T/B	20	9831513
ebi-a-GCST90001588	rs73220980	T/B	21	44752768
ebi-a-GCST90001588	rs5754162	T/B	22	32991307
ebi-a-GCST90001589	rs11120194	CD4/CD8br	1	213863315
ebi-a-GCST90001589	rs12139740	CD4/CD8br	1	242332976
ebi-a-GCST90001589	rs141317121	CD4/CD8br	1	19649970
ebi-a-GCST90001589	rs13418326	CD4/CD8br	2	113732809
ebi-a-GCST90001589	rs798726	CD4/CD8br	4	1685211
ebi-a-GCST90001589	rs955275	CD4/CD8br	6	117320197
ebi-a-GCST90001589	rs73402222	CD4/CD8br	6	31388449
ebi-a-GCST90001589	rs2516466	CD4/CD8br	6	31414327
ebi-a-GCST90001589	rs74493069	CD4/CD8br	7	138725017
ebi-a-GCST90001589	rs1529247	CD4/CD8br	7	9337459
ebi-a-GCST90001589	rs10990269	CD4/CD8br	9	99054924
ebi-a-GCST90001589	rs2576166	CD4/CD8br	10	90271135
ebi-a-GCST90001589	rs117942997	CD4/CD8br	10	74476968
ebi-a-GCST90001589	rs495055	CD4/CD8br	17	74440890
ebi-a-GCST90001589	rs11080350	CD4/CD8br	17	33774635
ebi-a-GCST90001589	rs62130076	CD4/CD8br	19	1671549
ebi-a-GCST90001590	rs7537437	CD4+ AC	1	39439538
ebi-a-GCST90001590	rs75697875	CD4+ AC	1	23233895
ebi-a-GCST90001590	rs2972073	CD4+ AC	2	55293730
ebi-a-GCST90001590	rs75494288	CD4+ AC	2	59985834
ebi-a-GCST90001590	rs905461	CD4+ AC	3	104884917
ebi-a-GCST90001590	rs10051472	CD4+ AC	5	79563530
ebi-a-GCST90001590	rs10040274	CD4+ AC	5	114822737
ebi-a-GCST90001590	rs117563350	CD4+ AC	6	151699396
ebi-a-GCST90001590	rs9271772	CD4+ AC	6	32594275
ebi-a-GCST90001590	rs143021470	CD4+ AC	6	81026437
ebi-a-GCST90001590	rs3129891	CD4+ AC	6	32415080
ebi-a-GCST90001590	rs72960548	CD4+ AC	6	127873865
ebi-a-GCST90001590	rs73132830	CD4+ AC	7	55936249
ebi-a-GCST90001590	rs4425753	CD4+ AC	8	15369837
ebi-a-GCST90001590	rs139746725	CD4+ AC	9	68344045
ebi-a-GCST90001590	rs78628680	CD4+ AC	10	114300633
ebi-a-GCST90001590	rs137903114	CD4+ AC	11	130770585
ebi-a-GCST90001590	rs61935579	CD4+ AC	12	60195749
ebi-a-GCST90001590	rs3184504	CD4+ AC	12	111884608

ebi-a-GCST90001590	rs3211602	CD4+ AC	12	58147219
ebi-a-GCST90001590	rs28578320	CD4+ AC	15	96213772
ebi-a-GCST90001590	rs7162344	CD4+ AC	15	26030485
ebi-a-GCST90001590	rs414743	CD4+ AC	21	46512075
ebi-a-GCST90001590	rs1849876	CD4+ AC	22	45611053
ebi-a-GCST90001591	rs1932067	CD4+ %T cell	1	26322081
ebi-a-GCST90001591	rs75478218	CD4+ %T cell	1	167583792
ebi-a-GCST90001591	rs10497440	CD4+ %T cell	2	176311434
ebi-a-GCST90001591	rs6744796	CD4+ %T cell	2	212828793
ebi-a-GCST90001591	rs55699240	CD4+ %T cell	2	113838725
ebi-a-GCST90001591	rs114828374	CD4+ %T cell	2	149887929
ebi-a-GCST90001591	rs76397707	CD4+ %T cell	3	96614918
ebi-a-GCST90001591	rs147164127	CD4+ %T cell	3	132826728
ebi-a-GCST90001591	rs798726	CD4+ %T cell	4	1685211
ebi-a-GCST90001591	rs114574090	CD4+ %T cell	4	46796462
ebi-a-GCST90001591	rs9266287	CD4+ %T cell	6	31328612
ebi-a-GCST90001591	rs13210358	CD4+ %T cell	6	102272234
ebi-a-GCST90001591	rs2516660	CD4+ %T cell	6	30466596
ebi-a-GCST90001591	rs60342399	CD4+ %T cell	7	52601846
ebi-a-GCST90001591	rs56009006	CD4+ %T cell	7	132484052
ebi-a-GCST90001591	rs72735973	CD4+ %T cell	8	136778540
ebi-a-GCST90001591	rs2576166	CD4+ %T cell	10	90271135
ebi-a-GCST90001591	rs1020462	CD4+ %T cell	12	78570914
ebi-a-GCST90001591	rs74950524	CD4+ %T cell	12	5424569
ebi-a-GCST90001591	rs16958131	CD4+ %T cell	13	101667739
ebi-a-GCST90001591	rs3914424	CD4+ %T cell	17	68353355
ebi-a-GCST90001591	rs2590261	CD4+ %T cell	17	74602286
ebi-a-GCST90001591	rs11869223	CD4+ %T cell	17	33775713
ebi-a-GCST90001591	rs7280330	CD4+ %T cell	21	33068962
ebi-a-GCST90001591	rs73162393	CD4+ %T cell	22	28078166
ebi-a-GCST90001592	rs12130486	CD8br AC	1	81455274
ebi-a-GCST90001592	rs12119984	CD8br AC	1	246874501
ebi-a-GCST90001592	rs78032118	CD8br AC	2	66964383
ebi-a-GCST90001592	rs113985770	CD8br AC	2	210377930
ebi-a-GCST90001592	rs6858602	CD8br AC	4	179936294
ebi-a-GCST90001592	rs3130685	CD8br AC	6	31206206
ebi-a-GCST90001592	rs75424572	CD8br AC	6	127364239
ebi-a-GCST90001592	rs12195711	CD8br AC	6	50386986
ebi-a-GCST90001592	rs28895267	CD8br AC	6	32445916
ebi-a-GCST90001592	rs6915999	CD8br AC	6	31377851
ebi-a-GCST90001592	rs79647440	CD8br AC	7	142438302
ebi-a-GCST90001592	rs11772710	CD8br AC	7	132433567
ebi-a-GCST90001592	rs1550856	CD8br AC	8	117805397
ebi-a-GCST90001592	rs11197647	CD8br AC	10	118142066
ebi-a-GCST90001592	rs1980812	CD8br AC	10	90274583
ebi-a-GCST90001592	rs11060493	CD8br AC	12	130160924
ebi-a-GCST90001592	rs28415769	CD8br AC	14	94358762
ebi-a-GCST90001592	rs117391590	CD8br AC	17	42759948

ebi-a-GCST90001592	rs35238206	CD8br AC	17	75698346
ebi-a-GCST90001592	rs11080350	CD8br AC	17	33774635
ebi-a-GCST90001592	rs41276984	CD8br AC	20	37580670
ebi-a-GCST90001592	rs2837838	CD8br AC	21	42191862
ebi-a-GCST90001592	rs2267983	CD8br AC	22	50698157
ebi-a-GCST90001593	rs76595423	CD8br %T cell	1	102745011
ebi-a-GCST90001593	rs141317121	CD8br %T cell	1	19649970
ebi-a-GCST90001593	rs12139740	CD8br %T cell	1	242332976
ebi-a-GCST90001593	rs13418326	CD8br %T cell	2	113732809
ebi-a-GCST90001593	rs7670980	CD8br %T cell	4	41910384
ebi-a-GCST90001593	rs798726	CD8br %T cell	4	1685211
ebi-a-GCST90001593	rs40653	CD8br %T cell	5	9372480
ebi-a-GCST90001593	rs2337390	CD8br %T cell	5	71408867
ebi-a-GCST90001593	rs73402222	CD8br %T cell	6	31388449
ebi-a-GCST90001593	rs2516466	CD8br %T cell	6	31414327
ebi-a-GCST90001593	rs955275	CD8br %T cell	6	117320197
ebi-a-GCST90001593	rs74493069	CD8br %T cell	7	138725017
ebi-a-GCST90001593	rs62458311	CD8br %T cell	7	67315960
ebi-a-GCST90001593	rs7826289	CD8br %T cell	8	61782205
ebi-a-GCST90001593	rs10990269	CD8br %T cell	9	99054924
ebi-a-GCST90001593	rs116943003	CD8br %T cell	10	76644830
ebi-a-GCST90001593	rs117942997	CD8br %T cell	10	74476968
ebi-a-GCST90001593	rs2576166	CD8br %T cell	10	90271135
ebi-a-GCST90001593	rs74381175	CD8br %T cell	11	130326664
ebi-a-GCST90001593	rs1676877	CD8br %T cell	11	74879130
ebi-a-GCST90001593	rs12449027	CD8br %T cell	16	13172942
ebi-a-GCST90001593	rs495055	CD8br %T cell	17	74440890
ebi-a-GCST90001593	rs62130076	CD8br %T cell	19	1671549
ebi-a-GCST90001593	rs73516994	CD8br %T cell	19	16748884
ebi-a-GCST90001594	rs72728134	DP (CD4+CD8+) A	1	100596929
ebi-a-GCST90001594	rs12142860	DP (CD4+CD8+) A	1	215998585
ebi-a-GCST90001594	rs75275131	DP (CD4+CD8+) A	2	4425244
ebi-a-GCST90001594	rs62314120	DP (CD4+CD8+) A	4	126951113
ebi-a-GCST90001594	rs10002165	DP (CD4+CD8+) A	4	145233527
ebi-a-GCST90001594	rs11725463	DP (CD4+CD8+) A	4	94584105
ebi-a-GCST90001594	rs9493240	DP (CD4+CD8+) A	6	132534887
ebi-a-GCST90001594	rs77965313	DP (CD4+CD8+) A	9	120915115
ebi-a-GCST90001594	rs147688743	DP (CD4+CD8+) A	10	64810319
ebi-a-GCST90001594	rs117166897	DP (CD4+CD8+) A	10	10964820
ebi-a-GCST90001594	rs11025036	DP (CD4+CD8+) A	11	19175633
ebi-a-GCST90001594	rs2857229	DP (CD4+CD8+) A	12	6900616
ebi-a-GCST90001594	rs1764923	DP (CD4+CD8+) A	13	86328055
ebi-a-GCST90001594	rs72694502	DP (CD4+CD8+) A	14	85161244
ebi-a-GCST90001594	rs7160965	DP (CD4+CD8+) A	14	31506505
ebi-a-GCST90001594	rs7141604	DP (CD4+CD8+) A	14	21252046
ebi-a-GCST90001594	rs6606924	DP (CD4+CD8+) A	15	29054286
ebi-a-GCST90001594	rs10851760	DP (CD4+CD8+) A	15	66871840
ebi-a-GCST90001594	rs857241	DP (CD4+CD8+) A	20	3078879

ebi-a-GCST90001594	rs6022045	DP (CD4+CD8+) A	20	51305288
ebi-a-GCST90001595	rs73095609	DP (CD4+CD8+) %	1	226673287
ebi-a-GCST90001595	rs10490700	DP (CD4+CD8+) %	2	106615183
ebi-a-GCST90001595	rs74197595	DP (CD4+CD8+) %	2	231492412
ebi-a-GCST90001595	rs16987794	DP (CD4+CD8+) %	2	20748761
ebi-a-GCST90001595	rs4482524	DP (CD4+CD8+) %	2	242673146
ebi-a-GCST90001595	rs28459093	DP (CD4+CD8+) %	4	13725129
ebi-a-GCST90001595	rs45551732	DP (CD4+CD8+) %	5	169816374
ebi-a-GCST90001595	rs111582587	DP (CD4+CD8+) %	6	32649176
ebi-a-GCST90001596	rs192222368	CD8dim AC	2	222901838
ebi-a-GCST90001596	rs1554808	CD8dim AC	2	123656927
ebi-a-GCST90001596	rs11676464	CD8dim AC	2	86018262
ebi-a-GCST90001596	rs139090356	CD8dim AC	2	7451357
ebi-a-GCST90001596	rs4438746	CD8dim AC	4	8280496
ebi-a-GCST90001596	rs60409696	CD8dim AC	5	8374588
ebi-a-GCST90001596	rs2937553	CD8dim AC	5	36473128
ebi-a-GCST90001596	rs72974644	CD8dim AC	6	123644770
ebi-a-GCST90001596	rs72936510	CD8dim AC	6	99300812
ebi-a-GCST90001596	rs139249541	CD8dim AC	6	102294761
ebi-a-GCST90001596	rs111273508	CD8dim AC	6	25330739
ebi-a-GCST90001596	rs79251210	CD8dim AC	6	100468602
ebi-a-GCST90001596	rs180222	CD8dim AC	8	133899160
ebi-a-GCST90001596	rs35947132	CD8dim AC	10	72360387
ebi-a-GCST90001596	rs74508088	CD8dim AC	10	52148920
ebi-a-GCST90001596	rs34562168	CD8dim AC	10	110358510
ebi-a-GCST90001596	rs80109323	CD8dim AC	10	133227859
ebi-a-GCST90001596	rs145682498	CD8dim AC	10	54508949
ebi-a-GCST90001596	rs112072415	CD8dim AC	11	33183043
ebi-a-GCST90001596	rs2733684	CD8dim AC	12	32558024
ebi-a-GCST90001596	rs7152367	CD8dim AC	14	66292007
ebi-a-GCST90001596	rs62020708	CD8dim AC	15	101232186
ebi-a-GCST90001596	rs149916994	CD8dim AC	16	8346489
ebi-a-GCST90001596	rs11652532	CD8dim AC	17	33769883
ebi-a-GCST90001596	rs9916629	CD8dim AC	17	33796260
ebi-a-GCST90001596	rs12185529	CD8dim AC	19	3059754
ebi-a-GCST90001596	rs111748563	CD8dim AC	19	17901533
ebi-a-GCST90001596	rs62210845	CD8dim AC	21	15274585
ebi-a-GCST90001597	rs62287820	CD8dim %T cell	3	155664429
ebi-a-GCST90001597	rs2937553	CD8dim %T cell	5	36473128
ebi-a-GCST90001597	rs60409696	CD8dim %T cell	5	8374588
ebi-a-GCST90001597	rs79086781	CD8dim %T cell	6	100526652
ebi-a-GCST90001597	rs139249541	CD8dim %T cell	6	102294761
ebi-a-GCST90001597	rs9457418	CD8dim %T cell	6	159051908
ebi-a-GCST90001597	rs141447985	CD8dim %T cell	6	104062697
ebi-a-GCST90001597	rs112505246	CD8dim %T cell	6	100914991
ebi-a-GCST90001597	rs76747637	CD8dim %T cell	7	79800654
ebi-a-GCST90001597	rs143576769	CD8dim %T cell	7	156775440
ebi-a-GCST90001597	rs140922347	CD8dim %T cell	7	121099420

ebi-a-GCST90001597	rs77148456	CD8dim %T cell	9	137583362
ebi-a-GCST90001597	rs112072415	CD8dim %T cell	11	33183043
ebi-a-GCST90001597	rs12871857	CD8dim %T cell	13	26527159
ebi-a-GCST90001597	rs17700958	CD8dim %T cell	14	56993406
ebi-a-GCST90001597	rs8022863	CD8dim %T cell	14	77628472
ebi-a-GCST90001597	rs142704274	CD8dim %T cell	17	39899038
ebi-a-GCST90001597	rs111748563	CD8dim %T cell	19	17901533
ebi-a-GCST90001597	rs7274761	CD8dim %T cell	20	60128452
ebi-a-GCST90001598	rs115364254	DN (CD4-CD8-) A(	1	222677137
ebi-a-GCST90001598	rs11124150	DN (CD4-CD8-) A(	2	98905012
ebi-a-GCST90001598	rs6755452	DN (CD4-CD8-) A(	2	87035076
ebi-a-GCST90001598	rs6708291	DN (CD4-CD8-) A(	2	220541824
ebi-a-GCST90001598	rs111891110	DN (CD4-CD8-) A(	2	75659115
ebi-a-GCST90001598	rs13108209	DN (CD4-CD8-) A(	4	4354607
ebi-a-GCST90001598	rs6860349	DN (CD4-CD8-) A(	5	2133412
ebi-a-GCST90001598	rs111273508	DN (CD4-CD8-) A(	6	25330739
ebi-a-GCST90001598	rs4298345	DN (CD4-CD8-) A(	6	31240818
ebi-a-GCST90001598	rs139249541	DN (CD4-CD8-) A(	6	102294761
ebi-a-GCST90001598	rs79251210	DN (CD4-CD8-) A(	6	100468602
ebi-a-GCST90001598	rs4711821	DN (CD4-CD8-) A(	6	45556291
ebi-a-GCST90001598	rs7869045	DN (CD4-CD8-) A(	9	124584495
ebi-a-GCST90001598	rs577044375	DN (CD4-CD8-) A(	9	138170904
ebi-a-GCST90001598	rs11103734	DN (CD4-CD8-) A(	9	138082201
ebi-a-GCST90001598	rs639937	DN (CD4-CD8-) A(	10	96981668
ebi-a-GCST90001598	rs11610256	DN (CD4-CD8-) A(	12	111734121
ebi-a-GCST90001598	rs61974190	DN (CD4-CD8-) A(	14	23252363
ebi-a-GCST90001598	rs12904219	DN (CD4-CD8-) A(	15	97967499
ebi-a-GCST90001598	rs9896155	DN (CD4-CD8-) A(	17	33794697
ebi-a-GCST90001598	rs9978065	DN (CD4-CD8-) A(	21	15247433
ebi-a-GCST90001599	rs181508809	DN (CD4-CD8-) %	1	19032967
ebi-a-GCST90001599	rs11124150	DN (CD4-CD8-) %	2	98905012
ebi-a-GCST90001599	rs2177640	DN (CD4-CD8-) %	2	9803664
ebi-a-GCST90001599	rs7569868	DN (CD4-CD8-) %	2	87019683
ebi-a-GCST90001599	rs7664985	DN (CD4-CD8-) %	4	96189661
ebi-a-GCST90001599	rs13108209	DN (CD4-CD8-) %	4	4354607
ebi-a-GCST90001599	rs78522661	DN (CD4-CD8-) %	5	179850567
ebi-a-GCST90001599	rs4711821	DN (CD4-CD8-) %	6	45556291
ebi-a-GCST90001599	rs139249541	DN (CD4-CD8-) %	6	102294761
ebi-a-GCST90001599	rs79251210	DN (CD4-CD8-) %	6	100468602
ebi-a-GCST90001599	rs17060639	DN (CD4-CD8-) %	6	100915659
ebi-a-GCST90001599	rs7021333	DN (CD4-CD8-) %	9	98823528
ebi-a-GCST90001599	rs11180823	DN (CD4-CD8-) %	12	76477941
ebi-a-GCST90001599	rs9896155	DN (CD4-CD8-) %	17	33794697
ebi-a-GCST90001599	rs140247402	DN (CD4-CD8-) %	17	1235562
ebi-a-GCST90001599	rs117051230	DN (CD4-CD8-) %	20	18427743
ebi-a-GCST90001600	rs12033535	Leukocyte AC	1	158256296
ebi-a-GCST90001600	rs12124698	Leukocyte AC	1	246883687
ebi-a-GCST90001600	rs960562	Leukocyte AC	1	81138405

ebi-a-GCST90001600	rs74698222	Leukocyte AC	2	129448626
ebi-a-GCST90001600	rs9856598	Leukocyte AC	3	143886945
ebi-a-GCST90001600	rs78475199	Leukocyte AC	3	151512300
ebi-a-GCST90001600	rs78158338	Leukocyte AC	4	189016126
ebi-a-GCST90001600	rs77745857	Leukocyte AC	4	105523007
ebi-a-GCST90001600	rs185513166	Leukocyte AC	4	109188818
ebi-a-GCST90001600	rs11725186	Leukocyte AC	4	94012466
ebi-a-GCST90001600	rs75663237	Leukocyte AC	5	55340690
ebi-a-GCST90001600	rs72762395	Leukocyte AC	5	43904012
ebi-a-GCST90001601	rs2797531	Lymphocyte AC	1	111173494
ebi-a-GCST90001601	rs17824379	Lymphocyte AC	3	5810961
ebi-a-GCST90001601	rs115885535	Lymphocyte AC	4	99705681
ebi-a-GCST90001601	rs10040274	Lymphocyte AC	5	114822737
ebi-a-GCST90001601	rs9271629	Lymphocyte AC	6	32591976
ebi-a-GCST90001601	rs72960548	Lymphocyte AC	6	127873865
ebi-a-GCST90001601	rs55802874	Lymphocyte AC	7	55923072
ebi-a-GCST90001601	rs6942586	Lymphocyte AC	7	36530276
ebi-a-GCST90001601	rs12417637	Lymphocyte AC	11	128303735
ebi-a-GCST90001601	rs7302662	Lymphocyte AC	12	129172288
ebi-a-GCST90001601	rs597808	Lymphocyte AC	12	111973358
ebi-a-GCST90001601	rs11850806	Lymphocyte AC	14	78669137
ebi-a-GCST90001601	rs941767	Lymphocyte AC	14	91864872
ebi-a-GCST90001601	rs142551447	Lymphocyte AC	16	28203694
ebi-a-GCST90001601	rs79508330	Lymphocyte AC	16	86445957
ebi-a-GCST90001601	rs10410390	Lymphocyte AC	19	54603419
ebi-a-GCST90001601	rs117336432	Lymphocyte AC	20	62523193
ebi-a-GCST90001601	rs112839163	Lymphocyte AC	20	14708666
ebi-a-GCST90001601	rs11702153	Lymphocyte AC	21	14911701
ebi-a-GCST90001602	rs67017818	Lymphocyte %leuk	1	21016307
ebi-a-GCST90001602	rs6701133	Lymphocyte %leuk	1	62836414
ebi-a-GCST90001602	rs112405229	Lymphocyte %leuk	1	240584083
ebi-a-GCST90001602	rs62264159	Lymphocyte %leuk	3	127409116
ebi-a-GCST90001602	rs893978	Lymphocyte %leuk	3	74174855
ebi-a-GCST90001602	rs184624056	Lymphocyte %leuk	4	259809
ebi-a-GCST90001602	rs78277075	Lymphocyte %leuk	4	183543742
ebi-a-GCST90001602	rs7677240	Lymphocyte %leuk	4	105475366
ebi-a-GCST90001602	rs11960775	Lymphocyte %leuk	5	126715206
ebi-a-GCST90001602	rs76019287	Lymphocyte %leuk	5	28612713
ebi-a-GCST90001602	rs6912413	Lymphocyte %leuk	6	22960927
ebi-a-GCST90001602	rs7006104	Lymphocyte %leuk	8	18403786
ebi-a-GCST90001602	rs11998531	Lymphocyte %leuk	8	120860297
ebi-a-GCST90001602	rs56275163	Lymphocyte %leuk	9	140738816
ebi-a-GCST90001602	rs2434066	Lymphocyte %leuk	10	83891503
ebi-a-GCST90001602	rs8177701	Lymphocyte %leuk	10	6003312
ebi-a-GCST90001602	rs481252	Lymphocyte %leuk	11	134188542
ebi-a-GCST90001602	rs61415458	Lymphocyte %leuk	12	20726031
ebi-a-GCST90001602	rs74346935	Lymphocyte %leuk	14	100697727
ebi-a-GCST90001602	rs111373552	Lymphocyte %leuk	16	25912505

ebi-a-GCST90001602	rs77650974	Lymphocyte %leuk	18	12394263
ebi-a-GCST90001602	rs150146725	Lymphocyte %leuk	20	49694567
ebi-a-GCST90001603	rs12124698	T cell AC	1	246883687
ebi-a-GCST90001603	rs75308045	T cell AC	1	111150195
ebi-a-GCST90001603	rs17339549	T cell AC	2	80078045
ebi-a-GCST90001603	rs17283318	T cell AC	3	151749774
ebi-a-GCST90001603	rs76560336	T cell AC	4	33455497
ebi-a-GCST90001603	rs10051472	T cell AC	5	79563530
ebi-a-GCST90001603	rs9271622	T cell AC	6	32591845
ebi-a-GCST90001603	rs72960548	T cell AC	6	127873865
ebi-a-GCST90001603	rs13207893	T cell AC	6	32586922
ebi-a-GCST90001603	rs62447160	T cell AC	7	36527098
ebi-a-GCST90001603	rs1550856	T cell AC	8	117805397
ebi-a-GCST90001603	rs139994967	T cell AC	11	79921187
ebi-a-GCST90001603	rs3184504	T cell AC	12	111884608
ebi-a-GCST90001603	rs11850806	T cell AC	14	78669137
ebi-a-GCST90001603	rs12599179	T cell AC	16	86449253
ebi-a-GCST90001603	rs117391590	T cell AC	17	42759948
ebi-a-GCST90001603	rs35669214	T cell AC	17	71372662
ebi-a-GCST90001603	rs75995320	T cell AC	20	14839626
ebi-a-GCST90001603	rs414743	T cell AC	21	46512075
ebi-a-GCST90001603	rs11702153	T cell AC	21	14911701
ebi-a-GCST90001604	rs1874323	T cell %lymphocyte	3	65771684
ebi-a-GCST90001604	rs4701789	T cell %lymphocyte	5	7691703
ebi-a-GCST90001604	rs34147569	T cell %lymphocyte	6	71753094
ebi-a-GCST90001604	rs28832872	T cell %lymphocyte	6	32529015
ebi-a-GCST90001604	rs6904670	T cell %lymphocyte	6	31192797
ebi-a-GCST90001604	rs241580	T cell %lymphocyte	6	157895107
ebi-a-GCST90001604	rs17615220	T cell %lymphocyte	6	32677413
ebi-a-GCST90001604	rs10868215	T cell %lymphocyte	9	87234111
ebi-a-GCST90001604	rs7864642	T cell %lymphocyte	9	122187995
ebi-a-GCST90001604	rs55972028	T cell %lymphocyte	9	134712997
ebi-a-GCST90001604	rs4758148	T cell %lymphocyte	11	5876630
ebi-a-GCST90001604	rs11620475	T cell %lymphocyte	13	112079904
ebi-a-GCST90001604	rs149528313	T cell %lymphocyte	13	110658783
ebi-a-GCST90001604	rs735609	T cell %lymphocyte	15	57644456
ebi-a-GCST90001604	rs9916257	T cell %lymphocyte	17	33797371
ebi-a-GCST90001604	rs11870294	T cell %lymphocyte	17	77121593
ebi-a-GCST90001604	rs10512469	T cell %lymphocyte	17	33770802
ebi-a-GCST90001604	rs62096695	T cell %lymphocyte	18	77533989
ebi-a-GCST90001605	rs1609355	T cell %leukocyte	2	22988095
ebi-a-GCST90001605	rs78641454	T cell %leukocyte	3	126744167
ebi-a-GCST90001605	rs893978	T cell %leukocyte	3	74174855
ebi-a-GCST90001605	rs4325897	T cell %leukocyte	3	111423342
ebi-a-GCST90001605	rs76320442	T cell %leukocyte	4	1632499
ebi-a-GCST90001605	rs184624056	T cell %leukocyte	4	259809
ebi-a-GCST90001605	rs1501750	T cell %leukocyte	5	4747892
ebi-a-GCST90001605	rs76019287	T cell %leukocyte	5	28612713

ebi-a-GCST90001605	rs1114226	T cell %leukocyte	6	77834631
ebi-a-GCST90001605	rs6912413	T cell %leukocyte	6	22960927
ebi-a-GCST90001605	rs113185319	T cell %leukocyte	7	100618701
ebi-a-GCST90001605	rs11998531	T cell %leukocyte	8	120860297
ebi-a-GCST90001605	rs16908368	T cell %leukocyte	9	122185915
ebi-a-GCST90001605	rs55764198	T cell %leukocyte	9	140738996
ebi-a-GCST90001605	rs481252	T cell %leukocyte	11	134188542
ebi-a-GCST90001605	rs12426383	T cell %leukocyte	12	20722854
ebi-a-GCST90001605	rs4614661	T cell %leukocyte	14	104750712
ebi-a-GCST90001605	rs9916629	T cell %leukocyte	17	33796260
ebi-a-GCST90001605	rs77650974	T cell %leukocyte	18	12394263
ebi-a-GCST90001605	rs150146725	T cell %leukocyte	20	49694567
ebi-a-GCST90001606	rs60704551	CD4+ %leukocyte	1	14791436
ebi-a-GCST90001606	rs114905473	CD4+ %leukocyte	4	111370027
ebi-a-GCST90001606	rs798726	CD4+ %leukocyte	4	1685211
ebi-a-GCST90001606	rs13210358	CD4+ %leukocyte	6	102272234
ebi-a-GCST90001606	rs118114356	CD4+ %leukocyte	6	162243723
ebi-a-GCST90001606	rs116160694	CD4+ %leukocyte	6	67354817
ebi-a-GCST90001606	rs56157148	CD4+ %leukocyte	9	138662360
ebi-a-GCST90001606	rs168003	CD4+ %leukocyte	10	20133549
ebi-a-GCST90001606	rs59571548	CD4+ %leukocyte	10	130289895
ebi-a-GCST90001606	rs1830888	CD4+ %leukocyte	10	45339904
ebi-a-GCST90001606	rs2003177	CD4+ %leukocyte	11	87066709
ebi-a-GCST90001606	rs912657	CD4+ %leukocyte	13	22369133
ebi-a-GCST90001606	rs17520378	CD4+ %leukocyte	15	50900606
ebi-a-GCST90001606	rs62048802	CD4+ %leukocyte	16	84703205
ebi-a-GCST90001606	rs112782656	CD4+ %leukocyte	19	57486015
ebi-a-GCST90001607	rs115924617	CD8br %leukocyte	1	100383765
ebi-a-GCST90001607	rs11163186	CD8br %leukocyte	1	81483189
ebi-a-GCST90001607	rs78326687	CD8br %leukocyte	1	242316531
ebi-a-GCST90001607	rs13418326	CD8br %leukocyte	2	113732809
ebi-a-GCST90001607	rs73207267	CD8br %leukocyte	3	194033312
ebi-a-GCST90001607	rs3804348	CD8br %leukocyte	4	3431342
ebi-a-GCST90001607	rs29001478	CD8br %leukocyte	6	32447162
ebi-a-GCST90001607	rs6915999	CD8br %leukocyte	6	31377851
ebi-a-GCST90001607	rs3130685	CD8br %leukocyte	6	31206206
ebi-a-GCST90001607	rs62458311	CD8br %leukocyte	7	67315960
ebi-a-GCST90001607	rs6986151	CD8br %leukocyte	8	101519901
ebi-a-GCST90001607	rs10990269	CD8br %leukocyte	9	99054924
ebi-a-GCST90001607	rs2576166	CD8br %leukocyte	10	90271135
ebi-a-GCST90001607	rs3829858	CD8br %leukocyte	10	85994142
ebi-a-GCST90001607	rs117942997	CD8br %leukocyte	10	74476968
ebi-a-GCST90001607	rs12931073	CD8br %leukocyte	16	78208251
ebi-a-GCST90001607	rs9934960	CD8br %leukocyte	16	17253548
ebi-a-GCST90001607	rs11080350	CD8br %leukocyte	17	33774635
ebi-a-GCST90001607	rs62130076	CD8br %leukocyte	19	1671549
ebi-a-GCST90001608	rs6719918	DP (CD4+CD8+) %	2	5547219
ebi-a-GCST90001608	rs16987794	DP (CD4+CD8+) %	2	20748761

ebi-a-GCST90001608	rs4482524	DP (CD4+CD8+) %	2	242673146
ebi-a-GCST90001608	rs28459093	DP (CD4+CD8+) %	4	13725129
ebi-a-GCST90001608	rs45551732	DP (CD4+CD8+) %	5	169816374
ebi-a-GCST90001608	rs9493240	DP (CD4+CD8+) %	6	132534887
ebi-a-GCST90001608	rs7786572	DP (CD4+CD8+) %	7	4779905
ebi-a-GCST90001608	rs77965313	DP (CD4+CD8+) %	9	120915115
ebi-a-GCST90001608	rs117166897	DP (CD4+CD8+) %	10	10964820
ebi-a-GCST90001608	rs147688743	DP (CD4+CD8+) %	10	64810319
ebi-a-GCST90001608	rs11025036	DP (CD4+CD8+) %	11	19175633
ebi-a-GCST90001608	rs3759343	DP (CD4+CD8+) %	12	6833104
ebi-a-GCST90001608	rs2857229	DP (CD4+CD8+) %	12	6900616
ebi-a-GCST90001608	rs72694502	DP (CD4+CD8+) %	14	85161244
ebi-a-GCST90001608	rs189060675	DP (CD4+CD8+) %	14	106214819
ebi-a-GCST90001608	rs76251345	DP (CD4+CD8+) %	14	51336044
ebi-a-GCST90001608	rs7160965	DP (CD4+CD8+) %	14	31506505
ebi-a-GCST90001608	rs117340321	DP (CD4+CD8+) %	15	42829973
ebi-a-GCST90001608	rs4966346	DP (CD4+CD8+) %	15	29054740
ebi-a-GCST90001608	rs62022846	DP (CD4+CD8+) %	16	11640890
ebi-a-GCST90001608	rs529770358	DP (CD4+CD8+) %	19	1185530
ebi-a-GCST90001608	rs2983457	DP (CD4+CD8+) %	20	62838443
ebi-a-GCST90001608	rs6022045	DP (CD4+CD8+) %	20	51305288
ebi-a-GCST90001608	rs55928872	DP (CD4+CD8+) %	21	45713321
ebi-a-GCST90001609	rs2801996	CD4+ CD8dim AC	1	90372717
ebi-a-GCST90001609	rs150339178	CD4+ CD8dim AC	2	85647906
ebi-a-GCST90001609	rs35290870	CD4+ CD8dim AC	2	87023103
ebi-a-GCST90001609	rs35046345	CD4+ CD8dim AC	2	84947693
ebi-a-GCST90001609	rs150052968	CD4+ CD8dim AC	3	42512599
ebi-a-GCST90001609	rs5030065	CD4+ CD8dim AC	3	186454577
ebi-a-GCST90001609	rs4699432	CD4+ CD8dim AC	4	96480118
ebi-a-GCST90001609	rs116684688	CD4+ CD8dim AC	5	170530012
ebi-a-GCST90001609	rs28366280	CD4+ CD8dim AC	6	32560262
ebi-a-GCST90001609	rs73729106	CD4+ CD8dim AC	6	32514288
ebi-a-GCST90001609	rs4959028	CD4+ CD8dim AC	6	32383138
ebi-a-GCST90001609	rs78510378	CD4+ CD8dim AC	7	154998925
ebi-a-GCST90001609	rs2706889	CD4+ CD8dim AC	7	95424378
ebi-a-GCST90001609	rs2810499	CD4+ CD8dim AC	9	136770207
ebi-a-GCST90001609	rs66907047	CD4+ CD8dim AC	10	26886249
ebi-a-GCST90001609	rs7109274	CD4+ CD8dim AC	11	59720416
ebi-a-GCST90001609	rs12422526	CD4+ CD8dim AC	12	87302893
ebi-a-GCST90001609	rs10880825	CD4+ CD8dim AC	12	45991896
ebi-a-GCST90001609	rs61953641	CD4+ CD8dim AC	13	64026403
ebi-a-GCST90001609	rs111693583	CD4+ CD8dim AC	16	28583610
ebi-a-GCST90001610	rs17530643	CD4+ CD8dim %ly	1	83469264
ebi-a-GCST90001610	rs2816843	CD4+ CD8dim %ly	1	90384101
ebi-a-GCST90001610	rs150339178	CD4+ CD8dim %ly	2	85647906
ebi-a-GCST90001610	rs35290870	CD4+ CD8dim %ly	2	87023103
ebi-a-GCST90001610	rs147195040	CD4+ CD8dim %ly	3	196485831
ebi-a-GCST90001610	rs150052968	CD4+ CD8dim %ly	3	42512599

ebi-a-GCST90001610	rs4833069	CD4+ CD8dim %ly	4	36081878
ebi-a-GCST90001610	rs6870700	CD4+ CD8dim %ly	5	86468013
ebi-a-GCST90001610	rs79131379	CD4+ CD8dim %ly	6	33133040
ebi-a-GCST90001610	rs4959028	CD4+ CD8dim %ly	6	32383138
ebi-a-GCST90001610	rs73729106	CD4+ CD8dim %ly	6	32514288
ebi-a-GCST90001610	rs28366280	CD4+ CD8dim %ly	6	32560262
ebi-a-GCST90001610	rs755884	CD4+ CD8dim %ly	6	160524578
ebi-a-GCST90001610	rs78510378	CD4+ CD8dim %ly	7	154998925
ebi-a-GCST90001610	rs2338695	CD4+ CD8dim %ly	8	90257990
ebi-a-GCST90001610	rs149551255	CD4+ CD8dim %ly	8	68301618
ebi-a-GCST90001610	rs66907047	CD4+ CD8dim %ly	10	26886249
ebi-a-GCST90001610	rs511713	CD4+ CD8dim %ly	11	128099099
ebi-a-GCST90001610	rs10858526	CD4+ CD8dim %ly	12	87312997
ebi-a-GCST90001610	rs10880825	CD4+ CD8dim %ly	12	45991896
ebi-a-GCST90001610	rs28521508	CD4+ CD8dim %ly	15	84131989
ebi-a-GCST90001610	rs111693583	CD4+ CD8dim %ly	16	28583610
ebi-a-GCST90001610	rs903881	CD4+ CD8dim %ly	16	16206042
ebi-a-GCST90001611	rs2801996	CD4+ CD8dim %le	1	90372717
ebi-a-GCST90001611	rs17530643	CD4+ CD8dim %le	1	83469264
ebi-a-GCST90001611	rs114200183	CD4+ CD8dim %le	2	97350229
ebi-a-GCST90001611	rs150339178	CD4+ CD8dim %le	2	85647906
ebi-a-GCST90001611	rs35290870	CD4+ CD8dim %le	2	87023103
ebi-a-GCST90001611	rs150052968	CD4+ CD8dim %le	3	42512599
ebi-a-GCST90001611	rs28366280	CD4+ CD8dim %le	6	32560262
ebi-a-GCST90001611	rs4959028	CD4+ CD8dim %le	6	32383138
ebi-a-GCST90001611	rs79131379	CD4+ CD8dim %le	6	33133040
ebi-a-GCST90001611	rs73729106	CD4+ CD8dim %le	6	32514288
ebi-a-GCST90001611	rs78510378	CD4+ CD8dim %le	7	154998925
ebi-a-GCST90001611	rs66907047	CD4+ CD8dim %le	10	26886249
ebi-a-GCST90001611	rs511713	CD4+ CD8dim %le	11	128099099
ebi-a-GCST90001611	rs10858526	CD4+ CD8dim %le	12	87312997
ebi-a-GCST90001611	rs10880825	CD4+ CD8dim %le	12	45991896
ebi-a-GCST90001611	rs28521508	CD4+ CD8dim %le	15	84131989
ebi-a-GCST90001611	rs903881	CD4+ CD8dim %le	16	16206042
ebi-a-GCST90001611	rs111693583	CD4+ CD8dim %le	16	28583610
ebi-a-GCST90001612	rs11676464	CD8dim %leukocyt	2	86018262
ebi-a-GCST90001612	rs62287820	CD8dim %leukocyt	3	155664429
ebi-a-GCST90001612	rs148468972	CD8dim %leukocyt	4	184898221
ebi-a-GCST90001612	rs34740679	CD8dim %leukocyt	4	4261553
ebi-a-GCST90001612	rs60409696	CD8dim %leukocyt	5	8374588
ebi-a-GCST90001612	rs2937553	CD8dim %leukocyt	5	36473128
ebi-a-GCST90001612	rs79086781	CD8dim %leukocyt	6	100526652
ebi-a-GCST90001612	rs139249541	CD8dim %leukocyt	6	102294761
ebi-a-GCST90001612	rs112505246	CD8dim %leukocyt	6	100914991
ebi-a-GCST90001612	rs76747637	CD8dim %leukocyt	7	79800654
ebi-a-GCST90001612	rs143576769	CD8dim %leukocyt	7	156775440
ebi-a-GCST90001612	rs77148456	CD8dim %leukocyt	9	137583362
ebi-a-GCST90001612	rs112072415	CD8dim %leukocyt	11	33183043

ebi-a-GCST90001612	rs12871857	CD8dim %leukocyt	13	26527159
ebi-a-GCST90001612	rs17700958	CD8dim %leukocyt	14	56993406
ebi-a-GCST90001612	rs8022863	CD8dim %leukocyt	14	77628472
ebi-a-GCST90001612	rs9916257	CD8dim %leukocyt	17	33797371
ebi-a-GCST90001612	rs111748563	CD8dim %leukocyt	19	17901533
ebi-a-GCST90001613	rs72784218	DN (CD4-CD8-) %	2	19698493
ebi-a-GCST90001613	rs11124150	DN (CD4-CD8-) %	2	98905012
ebi-a-GCST90001613	rs62176864	DN (CD4-CD8-) %	2	156465928
ebi-a-GCST90001613	rs7569868	DN (CD4-CD8-) %	2	87019683
ebi-a-GCST90001613	rs185860761	DN (CD4-CD8-) %	2	45213218
ebi-a-GCST90001613	rs2177640	DN (CD4-CD8-) %	2	9803664
ebi-a-GCST90001613	rs7664985	DN (CD4-CD8-) %	4	96189661
ebi-a-GCST90001613	rs4711821	DN (CD4-CD8-) %	6	45556291
ebi-a-GCST90001613	rs34025101	DN (CD4-CD8-) %	6	135388494
ebi-a-GCST90001613	rs79251210	DN (CD4-CD8-) %	6	100468602
ebi-a-GCST90001613	rs17060639	DN (CD4-CD8-) %	6	100915659
ebi-a-GCST90001613	rs139249541	DN (CD4-CD8-) %	6	102294761
ebi-a-GCST90001613	rs76747637	DN (CD4-CD8-) %	7	79800654
ebi-a-GCST90001613	rs7021333	DN (CD4-CD8-) %	9	98823528
ebi-a-GCST90001613	rs7869045	DN (CD4-CD8-) %	9	124584495
ebi-a-GCST90001613	rs11103734	DN (CD4-CD8-) %	9	138082201
ebi-a-GCST90001613	rs11180778	DN (CD4-CD8-) %	12	76371057
ebi-a-GCST90001613	rs142716558	DN (CD4-CD8-) %	14	51308539
ebi-a-GCST90001613	rs9896155	DN (CD4-CD8-) %	17	33794697
ebi-a-GCST90001613	rs140247402	DN (CD4-CD8-) %	17	1235562
ebi-a-GCST90001613	rs139015432	DN (CD4-CD8-) %	18	55486198
ebi-a-GCST90001614	rs78326687	CD8br and CD8dim	1	242316531
ebi-a-GCST90001614	rs13418326	CD8br and CD8dim	2	113732809
ebi-a-GCST90001614	rs6849514	CD8br and CD8dim	4	186386587
ebi-a-GCST90001614	rs76556848	CD8br and CD8dim	4	37388167
ebi-a-GCST90001614	rs3804348	CD8br and CD8dim	4	3431342
ebi-a-GCST90001614	rs6595142	CD8br and CD8dim	5	117867191
ebi-a-GCST90001614	rs73402222	CD8br and CD8dim	6	31388449
ebi-a-GCST90001614	rs2275854	CD8br and CD8dim	6	29976838
ebi-a-GCST90001614	rs9501618	CD8br and CD8dim	6	32339784
ebi-a-GCST90001614	rs62458311	CD8br and CD8dim	7	67315960
ebi-a-GCST90001614	rs6986151	CD8br and CD8dim	8	101519901
ebi-a-GCST90001614	rs118063063	CD8br and CD8dim	10	83179890
ebi-a-GCST90001614	rs2576166	CD8br and CD8dim	10	90271135
ebi-a-GCST90001614	rs11080350	CD8br and CD8dim	17	33774635
ebi-a-GCST90001614	rs4796452	CD8br and CD8dim	17	6157164
ebi-a-GCST90001614	rs73162393	CD8br and CD8dim	22	28078166
ebi-a-GCST90001615	rs558986	TCRgd AC	1	18660295
ebi-a-GCST90001615	rs947107	TCRgd AC	1	245716969
ebi-a-GCST90001615	rs11897347	TCRgd AC	2	197856673
ebi-a-GCST90001615	rs149000203	TCRgd AC	4	111341950
ebi-a-GCST90001615	rs139500218	TCRgd AC	5	100863142
ebi-a-GCST90001615	rs9273390	TCRgd AC	6	32627042

ebi-a-GCST90001615	rs67851010	TCRgd AC	6	32501347
ebi-a-GCST90001615	rs139311612	TCRgd AC	6	79117697
ebi-a-GCST90001615	rs73353488	TCRgd AC	7	38336965
ebi-a-GCST90001615	rs3802329	TCRgd AC	8	19266727
ebi-a-GCST90001615	rs77743715	TCRgd AC	9	24238234
ebi-a-GCST90001615	rs10850589	TCRgd AC	12	109499490
ebi-a-GCST90001615	rs117810965	TCRgd AC	13	110758617
ebi-a-GCST90001615	rs7192379	TCRgd AC	16	16869754
ebi-a-GCST90001615	rs7194802	TCRgd AC	16	60665097
ebi-a-GCST90001615	rs9912354	TCRgd AC	17	33805730
ebi-a-GCST90001615	rs35702634	TCRgd AC	17	72943434
ebi-a-GCST90001615	rs8079417	TCRgd AC	17	35269469
ebi-a-GCST90001615	rs142067182	TCRgd AC	18	51354134
ebi-a-GCST90001615	rs148395664	TCRgd AC	20	6008247
ebi-a-GCST90001615	rs9974788	TCRgd AC	21	27926021
ebi-a-GCST90001616	rs17643159	TCRgd %T cell	1	216117735
ebi-a-GCST90001616	rs320359	TCRgd %T cell	1	118129970
ebi-a-GCST90001616	rs4952856	TCRgd %T cell	2	47185438
ebi-a-GCST90001616	rs17224872	TCRgd %T cell	2	182351184
ebi-a-GCST90001616	rs75122287	TCRgd %T cell	2	190980603
ebi-a-GCST90001616	rs35996042	TCRgd %T cell	3	2194745
ebi-a-GCST90001616	rs113438136	TCRgd %T cell	3	86907637
ebi-a-GCST90001616	rs149000203	TCRgd %T cell	4	111341950
ebi-a-GCST90001616	rs72845743	TCRgd %T cell	6	32528414
ebi-a-GCST90001616	rs139311612	TCRgd %T cell	6	79117697
ebi-a-GCST90001616	rs116965753	TCRgd %T cell	7	121036117
ebi-a-GCST90001616	rs56396450	TCRgd %T cell	7	138404064
ebi-a-GCST90001616	rs7818691	TCRgd %T cell	8	19353778
ebi-a-GCST90001616	rs76021379	TCRgd %T cell	9	24274574
ebi-a-GCST90001616	rs2094742	TCRgd %T cell	10	133884758
ebi-a-GCST90001616	rs10850589	TCRgd %T cell	12	109499490
ebi-a-GCST90001616	rs80341165	TCRgd %T cell	12	115642929
ebi-a-GCST90001616	rs322939	TCRgd %T cell	17	3445039
ebi-a-GCST90001616	rs113457024	TCRgd %T cell	18	51480039
ebi-a-GCST90001617	rs947107	TCRgd %lymphocy	1	245716969
ebi-a-GCST90001617	rs17643159	TCRgd %lymphocy	1	216117735
ebi-a-GCST90001617	rs72990536	TCRgd %lymphocy	2	237548169
ebi-a-GCST90001617	rs840941	TCRgd %lymphocy	2	65713102
ebi-a-GCST90001617	rs4952856	TCRgd %lymphocy	2	47185438
ebi-a-GCST90001617	rs35996042	TCRgd %lymphocy	3	2194745
ebi-a-GCST90001617	rs113438136	TCRgd %lymphocy	3	86907637
ebi-a-GCST90001617	rs149000203	TCRgd %lymphocy	4	111341950
ebi-a-GCST90001617	rs139500218	TCRgd %lymphocy	5	100863142
ebi-a-GCST90001617	rs139311612	TCRgd %lymphocy	6	79117697
ebi-a-GCST90001617	rs72845743	TCRgd %lymphocy	6	32528414
ebi-a-GCST90001617	rs9267908	TCRgd %lymphocy	6	32204952
ebi-a-GCST90001617	rs56396450	TCRgd %lymphocy	7	138404064
ebi-a-GCST90001617	rs7818691	TCRgd %lymphocy	8	19353778

ebi-a-GCST90001617	rs76021379	TCRgd %lymphocy	9	24274574
ebi-a-GCST90001617	rs2094742	TCRgd %lymphocy	10	133884758
ebi-a-GCST90001617	rs10850589	TCRgd %lymphocy	12	109499490
ebi-a-GCST90001617	rs150193285	TCRgd %lymphocy	15	75224360
ebi-a-GCST90001617	rs9912354	TCRgd %lymphocy	17	33805730
ebi-a-GCST90001617	rs113457024	TCRgd %lymphocy	18	51480039
ebi-a-GCST90001617	rs174698	TCRgd %lymphocy	22	19954306
ebi-a-GCST90001618	rs4949963	HLA DR+ T cell A	1	95906877
ebi-a-GCST90001618	rs6686155	HLA DR+ T cell A	1	120406847
ebi-a-GCST90001618	rs7538103	HLA DR+ T cell A	1	16147854
ebi-a-GCST90001618	rs6758681	HLA DR+ T cell A	2	158890790
ebi-a-GCST90001618	rs57068864	HLA DR+ T cell A	3	29043472
ebi-a-GCST90001618	rs113649280	HLA DR+ T cell A	3	10881996
ebi-a-GCST90001618	rs71312261	HLA DR+ T cell A	3	158900756
ebi-a-GCST90001618	rs147577369	HLA DR+ T cell A	4	137447868
ebi-a-GCST90001618	rs68174286	HLA DR+ T cell A	6	32503204
ebi-a-GCST90001618	rs9391848	HLA DR+ T cell A	6	31320145
ebi-a-GCST90001618	rs78648104	HLA DR+ T cell A	6	50683009
ebi-a-GCST90001618	rs79684162	HLA DR+ T cell A	6	142661594
ebi-a-GCST90001618	rs76997602	HLA DR+ T cell A	7	115451309
ebi-a-GCST90001618	rs117419608	HLA DR+ T cell A	8	11831788
ebi-a-GCST90001618	rs2181036	HLA DR+ T cell A	9	117649136
ebi-a-GCST90001618	rs2808067	HLA DR+ T cell A	10	32045932
ebi-a-GCST90001618	rs1549827	HLA DR+ T cell A	10	118188503
ebi-a-GCST90001618	rs7308816	HLA DR+ T cell A	12	85022716
ebi-a-GCST90001618	rs11054779	HLA DR+ T cell A	12	12457054
ebi-a-GCST90001618	rs75799568	HLA DR+ T cell A	13	50104582
ebi-a-GCST90001618	rs506151	HLA DR+ T cell A	13	110882752
ebi-a-GCST90001618	rs59231583	HLA DR+ T cell A	14	96082974
ebi-a-GCST90001618	rs111481219	HLA DR+ T cell A	16	11544197
ebi-a-GCST90001618	rs72779785	HLA DR+ T cell A	16	10569039
ebi-a-GCST90001618	rs3087456	HLA DR+ T cell A	16	10970902
ebi-a-GCST90001618	rs62029480	HLA DR+ T cell A	16	10651993
ebi-a-GCST90001618	rs1084559	HLA DR+ T cell A	16	10825434
ebi-a-GCST90001618	rs71387949	HLA DR+ T cell A	17	77801131
ebi-a-GCST90001618	rs7504737	HLA DR+ T cell A	18	70065436
ebi-a-GCST90001618	rs138213638	HLA DR+ T cell A	18	55985546
ebi-a-GCST90001618	rs116844973	HLA DR+ T cell A	19	787806
ebi-a-GCST90001618	rs12627305	HLA DR+ T cell A	21	21249973
ebi-a-GCST90001618	rs9613063	HLA DR+ T cell A	22	26382004
ebi-a-GCST90001619	rs79178843	HLA DR+ T cell%	2	13251154
ebi-a-GCST90001619	rs116797692	HLA DR+ T cell%	2	158854583
ebi-a-GCST90001619	rs145794521	HLA DR+ T cell%	3	74944494
ebi-a-GCST90001619	rs148323639	HLA DR+ T cell%	4	27183687
ebi-a-GCST90001619	rs78648104	HLA DR+ T cell%	6	50683009
ebi-a-GCST90001619	rs3104414	HLA DR+ T cell%	6	32582612
ebi-a-GCST90001619	rs10244964	HLA DR+ T cell%	7	46432552
ebi-a-GCST90001619	rs76997602	HLA DR+ T cell%	7	115451309

ebi-a-GCST90001619	rs138678763	HLA DR+ T cell%T	8	11817727
ebi-a-GCST90001619	rs117941717	HLA DR+ T cell%T	8	105030375
ebi-a-GCST90001619	rs7047722	HLA DR+ T cell%T	9	101548105
ebi-a-GCST90001619	rs144674286	HLA DR+ T cell%T	9	101028704
ebi-a-GCST90001619	rs7032773	HLA DR+ T cell%T	9	117645562
ebi-a-GCST90001619	rs687184	HLA DR+ T cell%T	9	22725624
ebi-a-GCST90001619	rs117014352	HLA DR+ T cell%T	10	76784567
ebi-a-GCST90001619	rs10788105	HLA DR+ T cell%T	10	122390777
ebi-a-GCST90001619	rs7088079	HLA DR+ T cell%T	10	3066899
ebi-a-GCST90001619	rs7127620	HLA DR+ T cell%T	11	86071947
ebi-a-GCST90001619	rs542160	HLA DR+ T cell%T	11	107676543
ebi-a-GCST90001619	rs79132531	HLA DR+ T cell%T	11	107259543
ebi-a-GCST90001619	rs7294683	HLA DR+ T cell%T	12	126542791
ebi-a-GCST90001619	rs11054779	HLA DR+ T cell%T	12	12457054
ebi-a-GCST90001619	rs9526689	HLA DR+ T cell%T	13	51299267
ebi-a-GCST90001619	rs506151	HLA DR+ T cell%T	13	110882752
ebi-a-GCST90001619	rs3087456	HLA DR+ T cell%T	16	10970902
ebi-a-GCST90001619	rs71381201	HLA DR+ T cell%T	16	11357860
ebi-a-GCST90001619	rs1084560	HLA DR+ T cell%T	16	10824872
ebi-a-GCST90001619	rs55724950	HLA DR+ T cell%T	16	10993712
ebi-a-GCST90001619	rs12601032	HLA DR+ T cell%T	17	11457702
ebi-a-GCST90001619	rs62068717	HLA DR+ T cell%T	17	50420836
ebi-a-GCST90001619	rs138213638	HLA DR+ T cell%T	18	55985546
ebi-a-GCST90001619	rs140410174	HLA DR+ T cell%T	19	1671156
ebi-a-GCST90001619	rs73030989	HLA DR+ T cell%T	19	38996871
ebi-a-GCST90001620	rs67501806	HLA DR+ T cell%l	2	158891420
ebi-a-GCST90001620	rs79178843	HLA DR+ T cell%l	2	13251154
ebi-a-GCST90001620	rs116797692	HLA DR+ T cell%l	2	158854583
ebi-a-GCST90001620	rs143916977	HLA DR+ T cell%l	3	33852454
ebi-a-GCST90001620	rs148323639	HLA DR+ T cell%l	4	27183687
ebi-a-GCST90001620	rs68174286	HLA DR+ T cell%l	6	32503204
ebi-a-GCST90001620	rs10244964	HLA DR+ T cell%l	7	46432552
ebi-a-GCST90001620	rs76997602	HLA DR+ T cell%l	7	115451309
ebi-a-GCST90001620	rs146309290	HLA DR+ T cell%l	8	11854977
ebi-a-GCST90001620	rs144674286	HLA DR+ T cell%l	9	101028704
ebi-a-GCST90001620	rs7032773	HLA DR+ T cell%l	9	117645562
ebi-a-GCST90001620	rs7088079	HLA DR+ T cell%l	10	3066899
ebi-a-GCST90001620	rs10787670	HLA DR+ T cell%l	10	118187405
ebi-a-GCST90001620	rs10788105	HLA DR+ T cell%l	10	122390777
ebi-a-GCST90001620	rs117014352	HLA DR+ T cell%l	10	76784567
ebi-a-GCST90001620	rs542160	HLA DR+ T cell%l	11	107676543
ebi-a-GCST90001620	rs7294683	HLA DR+ T cell%l	12	126542791
ebi-a-GCST90001620	rs1863851	HLA DR+ T cell%l	12	11396335
ebi-a-GCST90001620	rs11054779	HLA DR+ T cell%l	12	12457054
ebi-a-GCST90001620	rs506151	HLA DR+ T cell%l	13	110882752
ebi-a-GCST90001620	rs9526689	HLA DR+ T cell%l	13	51299267
ebi-a-GCST90001620	rs2414178	HLA DR+ T cell%l	15	53001538
ebi-a-GCST90001620	rs11070458	HLA DR+ T cell%l	15	45771751

ebi-a-GCST90001620	rs3087456	HLA DR+ T cell%l	16	10970902
ebi-a-GCST90001620	rs62029480	HLA DR+ T cell%l	16	10651993
ebi-a-GCST90001620	rs55724950	HLA DR+ T cell%l	16	10993712
ebi-a-GCST90001620	rs1091849	HLA DR+ T cell%l	16	10829280
ebi-a-GCST90001620	rs73030989	HLA DR+ T cell%l	19	38996871
ebi-a-GCST90001620	rs140410174	HLA DR+ T cell%l	19	1671156
ebi-a-GCST90001621	rs112708092	NKT AC	2	236468046
ebi-a-GCST90001621	rs72960531	NKT AC	3	127072857
ebi-a-GCST90001621	rs35164053	NKT AC	4	110173445
ebi-a-GCST90001621	rs114574090	NKT AC	4	46796462
ebi-a-GCST90001621	rs353293	NKT AC	5	148807226
ebi-a-GCST90001621	rs16868916	NKT AC	5	39445552
ebi-a-GCST90001621	rs75494211	NKT AC	6	101911859
ebi-a-GCST90001621	rs511427	NKT AC	6	100143688
ebi-a-GCST90001621	rs139249541	NKT AC	6	102294761
ebi-a-GCST90001621	rs117047316	NKT AC	6	100317414
ebi-a-GCST90001621	rs115536607	NKT AC	6	101417409
ebi-a-GCST90001621	rs2041561	NKT AC	7	52580478
ebi-a-GCST90001621	rs640339	NKT AC	7	132413024
ebi-a-GCST90001621	rs10256019	NKT AC	7	12934776
ebi-a-GCST90001621	rs7819099	NKT AC	8	144992862
ebi-a-GCST90001621	rs145191094	NKT AC	8	25541959
ebi-a-GCST90001621	rs2781107	NKT AC	9	122546242
ebi-a-GCST90001621	rs10972866	NKT AC	9	36351694
ebi-a-GCST90001621	rs113127552	NKT AC	10	88396393
ebi-a-GCST90001621	rs10750486	NKT AC	11	131463916
ebi-a-GCST90001621	rs12792298	NKT AC	11	112189184
ebi-a-GCST90001621	rs78268116	NKT AC	11	112705025
ebi-a-GCST90001621	rs12224032	NKT AC	11	112914680
ebi-a-GCST90001621	rs10765907	NKT AC	11	2306683
ebi-a-GCST90001621	rs73122728	NKT AC	12	61383255
ebi-a-GCST90001621	rs73109444	NKT AC	12	47944164
ebi-a-GCST90001621	rs12817873	NKT AC	12	71518033
ebi-a-GCST90001621	rs79649529	NKT AC	13	109580017
ebi-a-GCST90001621	rs12895031	NKT AC	14	99846398
ebi-a-GCST90001621	rs72717679	NKT AC	15	33137651
ebi-a-GCST90001621	rs11652532	NKT AC	17	33769883
ebi-a-GCST90001621	rs9916629	NKT AC	17	33796260
ebi-a-GCST90001621	rs7232006	NKT AC	18	61860478
ebi-a-GCST90001621	rs8104714	NKT AC	19	32872109
ebi-a-GCST90001621	rs9983338	NKT AC	21	29982668
ebi-a-GCST90001622	rs112565020	NKT %T cell	2	153816776
ebi-a-GCST90001622	rs77846504	NKT %T cell	2	137558728
ebi-a-GCST90001622	rs186023757	NKT %T cell	2	208244512
ebi-a-GCST90001622	rs149977848	NKT %T cell	2	242038726
ebi-a-GCST90001622	rs2886726	NKT %T cell	3	20997419
ebi-a-GCST90001622	rs115536607	NKT %T cell	6	101417409
ebi-a-GCST90001622	rs115889733	NKT %T cell	6	44207850

ebi-a-GCST90001622	rs201024668	NKT %T cell	6	98118004
ebi-a-GCST90001622	rs117147408	NKT %T cell	6	101980039
ebi-a-GCST90001622	rs79251210	NKT %T cell	6	100468602
ebi-a-GCST90001622	rs9488834	NKT %T cell	6	97889936
ebi-a-GCST90001622	rs2041561	NKT %T cell	7	52580478
ebi-a-GCST90001622	rs17158356	NKT %T cell	7	35204550
ebi-a-GCST90001622	rs7819099	NKT %T cell	8	144992862
ebi-a-GCST90001622	rs3012770	NKT %T cell	9	134699760
ebi-a-GCST90001622	rs4918328	NKT %T cell	10	109648240
ebi-a-GCST90001622	rs10765907	NKT %T cell	11	2306683
ebi-a-GCST90001622	rs77738700	NKT %T cell	11	112751407
ebi-a-GCST90001622	rs12224032	NKT %T cell	11	112914680
ebi-a-GCST90001622	rs10750486	NKT %T cell	11	131463916
ebi-a-GCST90001622	rs12792298	NKT %T cell	11	112189184
ebi-a-GCST90001622	rs73122728	NKT %T cell	12	61383255
ebi-a-GCST90001622	rs73109444	NKT %T cell	12	47944164
ebi-a-GCST90001622	rs74426495	NKT %T cell	12	89421923
ebi-a-GCST90001622	rs79649529	NKT %T cell	13	109580017
ebi-a-GCST90001622	rs16958131	NKT %T cell	13	101667739
ebi-a-GCST90001622	rs906930	NKT %T cell	14	99822918
ebi-a-GCST90001622	rs72717679	NKT %T cell	15	33137651
ebi-a-GCST90001622	rs7225505	NKT %T cell	17	7861810
ebi-a-GCST90001622	rs7218011	NKT %T cell	17	33773022
ebi-a-GCST90001622	rs9912354	NKT %T cell	17	33805730
ebi-a-GCST90001622	rs75659919	NKT %T cell	18	23907745
ebi-a-GCST90001622	rs6141832	NKT %T cell	20	31448648
ebi-a-GCST90001622	rs2286306	NKT %T cell	20	52558849
ebi-a-GCST90001622	rs9983338	NKT %T cell	21	29982668
ebi-a-GCST90001623	rs35203503	NKT %lymphocyte	1	23087573
ebi-a-GCST90001623	rs149977848	NKT %lymphocyte	2	242038726
ebi-a-GCST90001623	rs186023757	NKT %lymphocyte	2	208244512
ebi-a-GCST90001623	rs77846504	NKT %lymphocyte	2	137558728
ebi-a-GCST90001623	rs2886726	NKT %lymphocyte	3	20997419
ebi-a-GCST90001623	rs79251210	NKT %lymphocyte	6	100468602
ebi-a-GCST90001623	rs117147408	NKT %lymphocyte	6	101980039
ebi-a-GCST90001623	rs201024668	NKT %lymphocyte	6	98118004
ebi-a-GCST90001623	rs115536607	NKT %lymphocyte	6	101417409
ebi-a-GCST90001623	rs9488834	NKT %lymphocyte	6	97889936
ebi-a-GCST90001623	rs115889733	NKT %lymphocyte	6	44207850
ebi-a-GCST90001623	rs2041561	NKT %lymphocyte	7	52580478
ebi-a-GCST90001623	rs17158356	NKT %lymphocyte	7	35204550
ebi-a-GCST90001623	rs7819099	NKT %lymphocyte	8	144992862
ebi-a-GCST90001623	rs2781107	NKT %lymphocyte	9	122546242
ebi-a-GCST90001623	rs3012770	NKT %lymphocyte	9	134699760
ebi-a-GCST90001623	rs4918328	NKT %lymphocyte	10	109648240
ebi-a-GCST90001623	rs12224032	NKT %lymphocyte	11	112914680
ebi-a-GCST90001623	rs10765907	NKT %lymphocyte	11	2306683
ebi-a-GCST90001623	rs10750486	NKT %lymphocyte	11	131463916

ebi-a-GCST90001623	rs12792298	NKT %lymphocyte	11	112189184
ebi-a-GCST90001623	rs77738700	NKT %lymphocyte	11	112751407
ebi-a-GCST90001623	rs73109444	NKT %lymphocyte	12	47944164
ebi-a-GCST90001623	rs74426495	NKT %lymphocyte	12	89421923
ebi-a-GCST90001623	rs73122728	NKT %lymphocyte	12	61383255
ebi-a-GCST90001623	rs16958131	NKT %lymphocyte	13	101667739
ebi-a-GCST90001623	rs906930	NKT %lymphocyte	14	99822918
ebi-a-GCST90001623	rs72717679	NKT %lymphocyte	15	33137651
ebi-a-GCST90001623	rs7225505	NKT %lymphocyte	17	7861810
ebi-a-GCST90001623	rs9912354	NKT %lymphocyte	17	33805730
ebi-a-GCST90001623	rs7218011	NKT %lymphocyte	17	33773022
ebi-a-GCST90001623	rs75659919	NKT %lymphocyte	18	23907745
ebi-a-GCST90001623	rs7254941	NKT %lymphocyte	19	9077196
ebi-a-GCST90001623	rs2286306	NKT %lymphocyte	20	52558849
ebi-a-GCST90001623	rs9983338	NKT %lymphocyte	21	29982668
ebi-a-GCST90001623	rs13055438	NKT %lymphocyte	22	45374249
ebi-a-GCST90001624	rs2064600	HLA DR+ CD4+ A	1	1220504
ebi-a-GCST90001624	rs62166973	HLA DR+ CD4+ A	2	121359390
ebi-a-GCST90001624	rs6767083	HLA DR+ CD4+ A	3	179838990
ebi-a-GCST90001624	rs55849757	HLA DR+ CD4+ A	3	75122371
ebi-a-GCST90001624	rs17824379	HLA DR+ CD4+ A	3	5810961
ebi-a-GCST90001624	rs6891593	HLA DR+ CD4+ A	5	155005574
ebi-a-GCST90001624	rs3134968	HLA DR+ CD4+ A	6	32674059
ebi-a-GCST90001624	rs17198888	HLA DR+ CD4+ A	6	31266015
ebi-a-GCST90001624	rs62417423	HLA DR+ CD4+ A	6	114211974
ebi-a-GCST90001624	rs79769908	HLA DR+ CD4+ A	7	70660195
ebi-a-GCST90001624	rs2977197	HLA DR+ CD4+ A	8	1690475
ebi-a-GCST90001624	rs1322067	HLA DR+ CD4+ A	9	117660933
ebi-a-GCST90001624	rs10787670	HLA DR+ CD4+ A	10	118187405
ebi-a-GCST90001624	rs78229126	HLA DR+ CD4+ A	10	11713685
ebi-a-GCST90001624	rs11251832	HLA DR+ CD4+ A	10	3327039
ebi-a-GCST90001624	rs10845930	HLA DR+ CD4+ A	12	14382617
ebi-a-GCST90001624	rs10148838	HLA DR+ CD4+ A	14	20261108
ebi-a-GCST90001624	rs7150202	HLA DR+ CD4+ A	14	80335426
ebi-a-GCST90001624	rs117110835	HLA DR+ CD4+ A	15	45945654
ebi-a-GCST90001624	rs142283210	HLA DR+ CD4+ A	15	80129450
ebi-a-GCST90001624	rs7405251	HLA DR+ CD4+ A	16	10829272
ebi-a-GCST90001624	rs3087456	HLA DR+ CD4+ A	16	10970902
ebi-a-GCST90001624	rs609755	HLA DR+ CD4+ A	19	56601692
ebi-a-GCST90001624	rs9613063	HLA DR+ CD4+ A	22	26382004
ebi-a-GCST90001624	rs12166155	HLA DR+ CD4+ A	22	44999166
ebi-a-GCST90001625	rs80226392	HLA DR+ CD4+ %	3	8304753
ebi-a-GCST90001625	rs116253486	HLA DR+ CD4+ %	3	33925087
ebi-a-GCST90001625	rs10052603	HLA DR+ CD4+ %	5	10837029
ebi-a-GCST90001625	rs4867947	HLA DR+ CD4+ %	5	169629403
ebi-a-GCST90001625	rs1832967	HLA DR+ CD4+ %	6	66862504
ebi-a-GCST90001625	rs3104415	HLA DR+ CD4+ %	6	32582577
ebi-a-GCST90001625	rs150440365	HLA DR+ CD4+ %	8	104592705

ebi-a-GCST90001625	rs567806839	HLA DR+ CD4+ %	8	26422292
ebi-a-GCST90001625	rs117868023	HLA DR+ CD4+ %	8	103550528
ebi-a-GCST90001625	rs79563199	HLA DR+ CD4+ %	9	130974142
ebi-a-GCST90001625	rs7032773	HLA DR+ CD4+ %	9	117645562
ebi-a-GCST90001625	rs7088079	HLA DR+ CD4+ %	10	3066899
ebi-a-GCST90001625	rs117816082	HLA DR+ CD4+ %	10	27360466
ebi-a-GCST90001625	rs146016042	HLA DR+ CD4+ %	11	97693585
ebi-a-GCST90001625	rs73062743	HLA DR+ CD4+ %	12	10511923
ebi-a-GCST90001625	rs7972178	HLA DR+ CD4+ %	12	14354060
ebi-a-GCST90001625	rs4906573	HLA DR+ CD4+ %	14	45842287
ebi-a-GCST90001625	rs117110835	HLA DR+ CD4+ %	15	45945654
ebi-a-GCST90001625	rs646262	HLA DR+ CD4+ %	15	29720356
ebi-a-GCST90001625	rs8055544	HLA DR+ CD4+ %	16	11091561
ebi-a-GCST90001625	rs11641185	HLA DR+ CD4+ %	16	10986596
ebi-a-GCST90001625	rs7202106	HLA DR+ CD4+ %	16	84375706
ebi-a-GCST90001625	rs140410174	HLA DR+ CD4+ %	19	1671156
ebi-a-GCST90001625	rs12609975	HLA DR+ CD4+ %	19	39400504
ebi-a-GCST90001625	rs6071096	HLA DR+ CD4+ %	20	58624500
ebi-a-GCST90001625	rs11090233	HLA DR+ CD4+ %	22	23690325
ebi-a-GCST90001625	rs149004280	HLA DR+ CD4+ %	22	48226137
ebi-a-GCST90001626	rs116253486	HLA DR+ CD4+ %	3	33925087
ebi-a-GCST90001626	rs1608073	HLA DR+ CD4+ %	3	179820505
ebi-a-GCST90001626	rs4867947	HLA DR+ CD4+ %	5	169629403
ebi-a-GCST90001626	rs12716076	HLA DR+ CD4+ %	5	10838090
ebi-a-GCST90001626	rs3104414	HLA DR+ CD4+ %	6	32582612
ebi-a-GCST90001626	rs150440365	HLA DR+ CD4+ %	8	104592705
ebi-a-GCST90001626	rs112418658	HLA DR+ CD4+ %	8	26844259
ebi-a-GCST90001626	rs117868023	HLA DR+ CD4+ %	8	103550528
ebi-a-GCST90001626	rs7032773	HLA DR+ CD4+ %	9	117645562
ebi-a-GCST90001626	rs7088079	HLA DR+ CD4+ %	10	3066899
ebi-a-GCST90001626	rs7972178	HLA DR+ CD4+ %	12	14354060
ebi-a-GCST90001626	rs7964565	HLA DR+ CD4+ %	12	71660112
ebi-a-GCST90001626	rs4906573	HLA DR+ CD4+ %	14	45842287
ebi-a-GCST90001626	rs7202106	HLA DR+ CD4+ %	16	84375706
ebi-a-GCST90001626	rs3087456	HLA DR+ CD4+ %	16	10970902
ebi-a-GCST90001626	rs539211	HLA DR+ CD4+ %	18	20039728
ebi-a-GCST90001626	rs12609975	HLA DR+ CD4+ %	19	39400504
ebi-a-GCST90001626	rs140410174	HLA DR+ CD4+ %	19	1671156
ebi-a-GCST90001626	rs12610775	HLA DR+ CD4+ %	19	53134203
ebi-a-GCST90001626	rs6071096	HLA DR+ CD4+ %	20	58624500
ebi-a-GCST90001626	rs149004280	HLA DR+ CD4+ %	22	48226137
ebi-a-GCST90001626	rs11090233	HLA DR+ CD4+ %	22	23690325
ebi-a-GCST90001627	rs771140	HLA DR+ CD8br A	1	34612610
ebi-a-GCST90001627	rs6686155	HLA DR+ CD8br A	1	120406847
ebi-a-GCST90001627	rs12029484	HLA DR+ CD8br A	1	230111146
ebi-a-GCST90001627	rs61500216	HLA DR+ CD8br A	1	168428587
ebi-a-GCST90001627	rs78744340	HLA DR+ CD8br A	2	24474853
ebi-a-GCST90001627	rs57068864	HLA DR+ CD8br A	3	29043472

ebi-a-GCST90001627	rs17286630	HLA DR+ CD8br A	5	58125959
ebi-a-GCST90001627	rs11153123	HLA DR+ CD8br A	6	109038333
ebi-a-GCST90001627	rs78648104	HLA DR+ CD8br A	6	50683009
ebi-a-GCST90001627	rs9378213	HLA DR+ CD8br A	6	32448398
ebi-a-GCST90001627	rs79684162	HLA DR+ CD8br A	6	142661594
ebi-a-GCST90001627	rs2516465	HLA DR+ CD8br A	6	31414906
ebi-a-GCST90001627	rs76997602	HLA DR+ CD8br A	7	115451309
ebi-a-GCST90001627	rs75495211	HLA DR+ CD8br A	7	114787625
ebi-a-GCST90001627	rs74743779	HLA DR+ CD8br A	8	103349435
ebi-a-GCST90001627	rs138678763	HLA DR+ CD8br A	8	11817727
ebi-a-GCST90001627	rs2181036	HLA DR+ CD8br A	9	117649136
ebi-a-GCST90001627	rs2808067	HLA DR+ CD8br A	10	32045932
ebi-a-GCST90001627	rs11197647	HLA DR+ CD8br A	10	118142066
ebi-a-GCST90001627	rs567794	HLA DR+ CD8br A	11	107664900
ebi-a-GCST90001627	rs73365443	HLA DR+ CD8br A	12	85019560
ebi-a-GCST90001627	rs75799568	HLA DR+ CD8br A	13	50104582
ebi-a-GCST90001627	rs506151	HLA DR+ CD8br A	13	110882752
ebi-a-GCST90001627	rs59231583	HLA DR+ CD8br A	14	96082974
ebi-a-GCST90001627	rs75913581	HLA DR+ CD8br A	14	100653131
ebi-a-GCST90001627	rs10152591	HLA DR+ CD8br A	15	70048157
ebi-a-GCST90001627	rs1084560	HLA DR+ CD8br A	16	10824872
ebi-a-GCST90001627	rs3087456	HLA DR+ CD8br A	16	10970902
ebi-a-GCST90001627	rs112455120	HLA DR+ CD8br A	16	10380785
ebi-a-GCST90001627	rs72779785	HLA DR+ CD8br A	16	10569039
ebi-a-GCST90001627	rs35669214	HLA DR+ CD8br A	17	71372662
ebi-a-GCST90001627	rs138213638	HLA DR+ CD8br A	18	55985546
ebi-a-GCST90001627	rs76443179	HLA DR+ CD8br A	19	38669622
ebi-a-GCST90001627	rs117517764	HLA DR+ CD8br A	19	17485805
ebi-a-GCST90001627	rs78581629	HLA DR+ CD8br A	19	795371
ebi-a-GCST90001628	rs112935806	HLA DR+ CD8br %	1	182869895
ebi-a-GCST90001628	rs72757686	HLA DR+ CD8br %	1	243409313
ebi-a-GCST90001628	rs771140	HLA DR+ CD8br %	1	34612610
ebi-a-GCST90001628	rs12029484	HLA DR+ CD8br %	1	230111146
ebi-a-GCST90001628	rs9378213	HLA DR+ CD8br %	6	32448398
ebi-a-GCST90001628	rs78456529	HLA DR+ CD8br %	7	131627162
ebi-a-GCST90001628	rs6975360	HLA DR+ CD8br %	7	82348481
ebi-a-GCST90001628	rs78867731	HLA DR+ CD8br %	8	107775474
ebi-a-GCST90001628	rs138678763	HLA DR+ CD8br %	8	11817727
ebi-a-GCST90001628	rs75588949	HLA DR+ CD8br %	9	113087414
ebi-a-GCST90001628	rs1407309	HLA DR+ CD8br %	9	117651780
ebi-a-GCST90001628	rs7027771	HLA DR+ CD8br %	9	101552955
ebi-a-GCST90001628	rs111509656	HLA DR+ CD8br %	9	101005853
ebi-a-GCST90001628	rs11598687	HLA DR+ CD8br %	10	76609571
ebi-a-GCST90001628	rs79132531	HLA DR+ CD8br %	11	107259543
ebi-a-GCST90001628	rs72998075	HLA DR+ CD8br %	11	109402654
ebi-a-GCST90001628	rs542160	HLA DR+ CD8br %	11	107676543
ebi-a-GCST90001628	rs11054779	HLA DR+ CD8br %	12	12457054
ebi-a-GCST90001628	rs73365443	HLA DR+ CD8br %	12	85019560

ebi-a-GCST90001628	rs506151	HLA DR+ CD8br %	13	110882752
ebi-a-GCST90001628	rs34287418	HLA DR+ CD8br %	14	104939039
ebi-a-GCST90001628	rs3087456	HLA DR+ CD8br %	16	10970902
ebi-a-GCST90001628	rs1091849	HLA DR+ CD8br %	16	10829280
ebi-a-GCST90001628	rs72779785	HLA DR+ CD8br %	16	10569039
ebi-a-GCST90001628	rs62029480	HLA DR+ CD8br %	16	10651993
ebi-a-GCST90001628	rs112455120	HLA DR+ CD8br %	16	10380785
ebi-a-GCST90001628	rs7195518	HLA DR+ CD8br %	16	11485515
ebi-a-GCST90001628	rs138213638	HLA DR+ CD8br %	18	55985546
ebi-a-GCST90001628	rs117517764	HLA DR+ CD8br %	19	17485805
ebi-a-GCST90001628	rs76443179	HLA DR+ CD8br %	19	38669622
ebi-a-GCST90001628	rs59556129	HLA DR+ CD8br %	19	42107414
ebi-a-GCST90001628	rs6020784	HLA DR+ CD8br %	20	49449929
ebi-a-GCST90001628	rs4920093	HLA DR+ CD8br %	21	43304155
ebi-a-GCST90001629	rs12029484	HLA DR+ CD8br %	1	230111146
ebi-a-GCST90001629	rs147143327	HLA DR+ CD8br %	1	244492916
ebi-a-GCST90001629	rs771140	HLA DR+ CD8br %	1	34612610
ebi-a-GCST90001629	rs9378213	HLA DR+ CD8br %	6	32448398
ebi-a-GCST90001629	rs6975360	HLA DR+ CD8br %	7	82348481
ebi-a-GCST90001629	rs76997602	HLA DR+ CD8br %	7	115451309
ebi-a-GCST90001629	rs78456529	HLA DR+ CD8br %	7	131627162
ebi-a-GCST90001629	rs138678763	HLA DR+ CD8br %	8	11817727
ebi-a-GCST90001629	rs78867731	HLA DR+ CD8br %	8	107775474
ebi-a-GCST90001629	rs2146255	HLA DR+ CD8br %	9	101567115
ebi-a-GCST90001629	rs1407309	HLA DR+ CD8br %	9	117651780
ebi-a-GCST90001629	rs144674286	HLA DR+ CD8br %	9	101028704
ebi-a-GCST90001629	rs11598687	HLA DR+ CD8br %	10	76609571
ebi-a-GCST90001629	rs1431485	HLA DR+ CD8br %	10	118193216
ebi-a-GCST90001629	rs542160	HLA DR+ CD8br %	11	107676543
ebi-a-GCST90001629	rs79132531	HLA DR+ CD8br %	11	107259543
ebi-a-GCST90001629	rs73365443	HLA DR+ CD8br %	12	85019560
ebi-a-GCST90001629	rs11054779	HLA DR+ CD8br %	12	12457054
ebi-a-GCST90001629	rs506151	HLA DR+ CD8br %	13	110882752
ebi-a-GCST90001629	rs34287418	HLA DR+ CD8br %	14	104939039
ebi-a-GCST90001629	rs60923927	HLA DR+ CD8br %	16	10380869
ebi-a-GCST90001629	rs4527037	HLA DR+ CD8br %	16	11518708
ebi-a-GCST90001629	rs112455120	HLA DR+ CD8br %	16	10380785
ebi-a-GCST90001629	rs3087456	HLA DR+ CD8br %	16	10970902
ebi-a-GCST90001629	rs72779785	HLA DR+ CD8br %	16	10569039
ebi-a-GCST90001629	rs62029480	HLA DR+ CD8br %	16	10651993
ebi-a-GCST90001629	rs117517764	HLA DR+ CD8br %	19	17485805
ebi-a-GCST90001629	rs113162001	HLA DR+ CD8br %	19	53321356
ebi-a-GCST90001629	rs78581629	HLA DR+ CD8br %	19	795371
ebi-a-GCST90001629	rs73030989	HLA DR+ CD8br %	19	38996871
ebi-a-GCST90001629	rs6020784	HLA DR+ CD8br %	20	49449929
ebi-a-GCST90001629	rs4920093	HLA DR+ CD8br %	21	43304155
ebi-a-GCST90001630	rs17448682	CD8br NKT AC	1	15966713
ebi-a-GCST90001630	rs55699240	CD8br NKT AC	2	113838725

ebi-a-GCST90001630	rs60553229	CD8br NKT AC	3	183735909
ebi-a-GCST90001630	rs4235317	CD8br NKT AC	4	24439455
ebi-a-GCST90001630	rs115889733	CD8br NKT AC	6	44207850
ebi-a-GCST90001630	rs7756993	CD8br NKT AC	6	31382133
ebi-a-GCST90001630	rs9269109	CD8br NKT AC	6	32443266
ebi-a-GCST90001630	rs593435	CD8br NKT AC	7	132417404
ebi-a-GCST90001630	rs34575510	CD8br NKT AC	7	23209757
ebi-a-GCST90001630	rs2041561	CD8br NKT AC	7	52580478
ebi-a-GCST90001630	rs7003580	CD8br NKT AC	8	145007534
ebi-a-GCST90001630	rs78099351	CD8br NKT AC	9	10082863
ebi-a-GCST90001630	rs4745010	CD8br NKT AC	9	73024602
ebi-a-GCST90001630	rs1325907	CD8br NKT AC	10	90237398
ebi-a-GCST90001630	rs12792298	CD8br NKT AC	11	112189184
ebi-a-GCST90001630	rs78268116	CD8br NKT AC	11	112705025
ebi-a-GCST90001630	rs12224032	CD8br NKT AC	11	112914680
ebi-a-GCST90001630	rs144156455	CD8br NKT AC	11	70988762
ebi-a-GCST90001630	rs2074023	CD8br NKT AC	11	2325581
ebi-a-GCST90001630	rs73109444	CD8br NKT AC	12	47944164
ebi-a-GCST90001630	rs12422853	CD8br NKT AC	12	105063882
ebi-a-GCST90001630	rs61944019	CD8br NKT AC	13	27907115
ebi-a-GCST90001630	rs117122100	CD8br NKT AC	13	109403845
ebi-a-GCST90001630	rs11658693	CD8br NKT AC	17	33800249
ebi-a-GCST90001630	rs12940345	CD8br NKT AC	17	74612462
ebi-a-GCST90001630	rs1008425	CD8br NKT AC	19	32852145
ebi-a-GCST90001630	rs1077381	CD8br NKT AC	20	59623849
ebi-a-GCST90001631	rs61815774	CD8br NKT %T ce	1	208930710
ebi-a-GCST90001631	rs115294653	CD8br NKT %T ce	1	28413298
ebi-a-GCST90001631	rs147600774	CD8br NKT %T ce	1	115926543
ebi-a-GCST90001631	rs34994982	CD8br NKT %T ce	2	137472168
ebi-a-GCST90001631	rs7577330	CD8br NKT %T ce	2	113613580
ebi-a-GCST90001631	rs60553229	CD8br NKT %T ce	3	183735909
ebi-a-GCST90001631	rs12499194	CD8br NKT %T ce	4	24539202
ebi-a-GCST90001631	rs17256404	CD8br NKT %T ce	5	9391367
ebi-a-GCST90001631	rs115889733	CD8br NKT %T ce	6	44207850
ebi-a-GCST90001631	rs2275854	CD8br NKT %T ce	6	29976838
ebi-a-GCST90001631	rs34575510	CD8br NKT %T ce	7	23209757
ebi-a-GCST90001631	rs10276441	CD8br NKT %T ce	7	52581385
ebi-a-GCST90001631	rs11544989	CD8br NKT %T ce	8	145057868
ebi-a-GCST90001631	rs725683	CD8br NKT %T ce	10	90258876
ebi-a-GCST90001631	rs161249	CD8br NKT %T ce	10	31701343
ebi-a-GCST90001631	rs2074023	CD8br NKT %T ce	11	2325581
ebi-a-GCST90001631	rs12224032	CD8br NKT %T ce	11	112914680
ebi-a-GCST90001631	rs568739	CD8br NKT %T ce	11	128060429
ebi-a-GCST90001631	rs144156455	CD8br NKT %T ce	11	70988762
ebi-a-GCST90001631	rs145936741	CD8br NKT %T ce	11	116470289
ebi-a-GCST90001631	rs12792298	CD8br NKT %T ce	11	112189184
ebi-a-GCST90001631	rs76161425	CD8br NKT %T ce	11	112704995
ebi-a-GCST90001631	rs117122100	CD8br NKT %T ce	13	109403845

ebi-a-GCST90001631	rs116179687	CD8br NKT %T ce	13	108484563
ebi-a-GCST90001631	rs61944019	CD8br NKT %T ce	13	27907115
ebi-a-GCST90001631	rs11650003	CD8br NKT %T ce	17	7866604
ebi-a-GCST90001631	rs11658954	CD8br NKT %T ce	17	74953080
ebi-a-GCST90001631	rs56297703	CD8br NKT %T ce	18	67865495
ebi-a-GCST90001631	rs5769842	CD8br NKT %T ce	22	49437976
ebi-a-GCST90001632	rs147600774	CD8br NKT %lymp	1	115926543
ebi-a-GCST90001632	rs61815774	CD8br NKT %lymp	1	208930710
ebi-a-GCST90001632	rs55699240	CD8br NKT %lymp	2	113838725
ebi-a-GCST90001632	rs34994982	CD8br NKT %lymp	2	137472168
ebi-a-GCST90001632	rs10188255	CD8br NKT %lymp	2	122052948
ebi-a-GCST90001632	rs60553229	CD8br NKT %lymp	3	183735909
ebi-a-GCST90001632	rs12499194	CD8br NKT %lymp	4	24539202
ebi-a-GCST90001632	rs17256404	CD8br NKT %lymp	5	9391367
ebi-a-GCST90001632	rs115889733	CD8br NKT %lymp	6	44207850
ebi-a-GCST90001632	rs2395191	CD8br NKT %lymp	6	32447085
ebi-a-GCST90001632	rs34575510	CD8br NKT %lymp	7	23209757
ebi-a-GCST90001632	rs2041561	CD8br NKT %lymp	7	52580478
ebi-a-GCST90001632	rs11544989	CD8br NKT %lymp	8	145057868
ebi-a-GCST90001632	rs725683	CD8br NKT %lymp	10	90258876
ebi-a-GCST90001632	rs12363646	CD8br NKT %lymp	11	128067342
ebi-a-GCST90001632	rs12792298	CD8br NKT %lymp	11	112189184
ebi-a-GCST90001632	rs11216023	CD8br NKT %lymp	11	116460172
ebi-a-GCST90001632	rs2074023	CD8br NKT %lymp	11	2325581
ebi-a-GCST90001632	rs78268116	CD8br NKT %lymp	11	112705025
ebi-a-GCST90001632	rs12224032	CD8br NKT %lymp	11	112914680
ebi-a-GCST90001632	rs144156455	CD8br NKT %lymp	11	70988762
ebi-a-GCST90001632	rs61944019	CD8br NKT %lymp	13	27907115
ebi-a-GCST90001632	rs117122100	CD8br NKT %lymp	13	109403845
ebi-a-GCST90001632	rs116179687	CD8br NKT %lymp	13	108484563
ebi-a-GCST90001632	rs11658954	CD8br NKT %lymp	17	74953080
ebi-a-GCST90001632	rs11650003	CD8br NKT %lymp	17	7866604
ebi-a-GCST90001632	rs75361910	CD8br NKT %lymp	17	33793208
ebi-a-GCST90001632	rs56297703	CD8br NKT %lymp	18	67865495
ebi-a-GCST90001632	rs117499937	CD8br NKT %lymp	22	28030270
ebi-a-GCST90001633	rs116700304	CD8dim NKT AC	1	6519453
ebi-a-GCST90001633	rs113855303	CD8dim NKT AC	1	11524363
ebi-a-GCST90001633	rs11676464	CD8dim NKT AC	2	86018262
ebi-a-GCST90001633	rs4500996	CD8dim NKT AC	2	98843446
ebi-a-GCST90001633	rs139090356	CD8dim NKT AC	2	7451357
ebi-a-GCST90001633	rs62391967	CD8dim NKT AC	3	106910114
ebi-a-GCST90001633	rs145331774	CD8dim NKT AC	6	402147
ebi-a-GCST90001633	rs79251210	CD8dim NKT AC	6	100468602
ebi-a-GCST90001633	rs9488834	CD8dim NKT AC	6	97889936
ebi-a-GCST90001633	rs111273508	CD8dim NKT AC	6	25330739
ebi-a-GCST90001633	rs117147408	CD8dim NKT AC	6	101980039
ebi-a-GCST90001633	rs115536607	CD8dim NKT AC	6	101417409
ebi-a-GCST90001633	rs303054	CD8dim NKT AC	6	10416638

ebi-a-GCST90001633	rs6970303	CD8dim NKT AC	7	28913959
ebi-a-GCST90001633	rs34124324	CD8dim NKT AC	8	4205974
ebi-a-GCST90001633	rs11544989	CD8dim NKT AC	8	145057868
ebi-a-GCST90001633	rs145682498	CD8dim NKT AC	10	54508949
ebi-a-GCST90001633	rs80109323	CD8dim NKT AC	10	133227859
ebi-a-GCST90001633	rs10892953	CD8dim NKT AC	11	99100867
ebi-a-GCST90001633	rs77738700	CD8dim NKT AC	11	112751407
ebi-a-GCST90001633	rs73062743	CD8dim NKT AC	12	10511923
ebi-a-GCST90001633	rs7323005	CD8dim NKT AC	13	40673331
ebi-a-GCST90001633	rs7152367	CD8dim NKT AC	14	66292007
ebi-a-GCST90001633	rs35840380	CD8dim NKT AC	14	102546027
ebi-a-GCST90001633	rs7208329	CD8dim NKT AC	17	33128801
ebi-a-GCST90001633	rs7218011	CD8dim NKT AC	17	33773022
ebi-a-GCST90001633	rs9916257	CD8dim NKT AC	17	33797371
ebi-a-GCST90001633	rs62126783	CD8dim NKT AC	19	17596144
ebi-a-GCST90001633	rs62210845	CD8dim NKT AC	21	15274585
ebi-a-GCST90001633	rs8131290	CD8dim NKT AC	21	45423006
ebi-a-GCST90001634	rs113855303	CD8dim NKT %T (	1	11524363
ebi-a-GCST90001634	rs4500996	CD8dim NKT %T (	2	98843446
ebi-a-GCST90001634	rs139090356	CD8dim NKT %T (	2	7451357
ebi-a-GCST90001634	rs7589893	CD8dim NKT %T (	2	99932255
ebi-a-GCST90001634	rs117147408	CD8dim NKT %T (	6	101980039
ebi-a-GCST90001634	rs201024668	CD8dim NKT %T (	6	98118004
ebi-a-GCST90001634	rs115536607	CD8dim NKT %T (	6	101417409
ebi-a-GCST90001634	rs79251210	CD8dim NKT %T (	6	100468602
ebi-a-GCST90001634	rs9488834	CD8dim NKT %T (	6	97889936
ebi-a-GCST90001634	rs6970303	CD8dim NKT %T (	7	28913959
ebi-a-GCST90001634	rs11544989	CD8dim NKT %T (	8	145057868
ebi-a-GCST90001634	rs77738700	CD8dim NKT %T (	11	112751407
ebi-a-GCST90001634	rs61925057	CD8dim NKT %T (	12	61076597
ebi-a-GCST90001634	rs73062743	CD8dim NKT %T (	12	10511923
ebi-a-GCST90001634	rs12871857	CD8dim NKT %T (	13	26527159
ebi-a-GCST90001634	rs7323005	CD8dim NKT %T (	13	40673331
ebi-a-GCST90001634	rs7152367	CD8dim NKT %T (	14	66292007
ebi-a-GCST90001634	rs113413210	CD8dim NKT %T (	15	78982526
ebi-a-GCST90001634	rs112895628	CD8dim NKT %T (	17	79356092
ebi-a-GCST90001634	rs142704274	CD8dim NKT %T (	17	39899038
ebi-a-GCST90001634	rs9912354	CD8dim NKT %T (	17	33805730
ebi-a-GCST90001634	rs5998010	CD8dim NKT %T (	22	31889080
ebi-a-GCST90001635	rs28628425	CD8dim NKT %lyr	1	248782956
ebi-a-GCST90001635	rs7589893	CD8dim NKT %lyr	2	99932255
ebi-a-GCST90001635	rs4500996	CD8dim NKT %lyr	2	98843446
ebi-a-GCST90001635	rs139090356	CD8dim NKT %lyr	2	7451357
ebi-a-GCST90001635	rs11676464	CD8dim NKT %lyr	2	86018262
ebi-a-GCST90001635	rs73223508	CD8dim NKT %lyr	3	137239756
ebi-a-GCST90001635	rs62287820	CD8dim NKT %lyr	3	155664429
ebi-a-GCST90001635	rs9488834	CD8dim NKT %lyr	6	97889936
ebi-a-GCST90001635	rs117147408	CD8dim NKT %lyr	6	101980039

ebi-a-GCST90001635	rs201024668	CD8dim NKT %lyr	6	98118004
ebi-a-GCST90001635	rs79251210	CD8dim NKT %lyr	6	100468602
ebi-a-GCST90001635	rs115536607	CD8dim NKT %lyr	6	101417409
ebi-a-GCST90001635	rs6970303	CD8dim NKT %lyr	7	28913959
ebi-a-GCST90001635	rs11544989	CD8dim NKT %lyr	8	145057868
ebi-a-GCST90001635	rs77738700	CD8dim NKT %lyr	11	112751407
ebi-a-GCST90001635	rs10892953	CD8dim NKT %lyr	11	99100867
ebi-a-GCST90001635	rs73062743	CD8dim NKT %lyr	12	10511923
ebi-a-GCST90001635	rs61925057	CD8dim NKT %lyr	12	61076597
ebi-a-GCST90001635	rs12871857	CD8dim NKT %lyr	13	26527159
ebi-a-GCST90001635	rs7323005	CD8dim NKT %lyr	13	40673331
ebi-a-GCST90001635	rs7152367	CD8dim NKT %lyr	14	66292007
ebi-a-GCST90001635	rs35237642	CD8dim NKT %lyr	14	102605308
ebi-a-GCST90001635	rs113413210	CD8dim NKT %lyr	15	78982526
ebi-a-GCST90001635	rs191392302	CD8dim NKT %lyr	17	39953961
ebi-a-GCST90001635	rs9916257	CD8dim NKT %lyr	17	33797371
ebi-a-GCST90001635	rs112895628	CD8dim NKT %lyr	17	79356092
ebi-a-GCST90001635	rs7208329	CD8dim NKT %lyr	17	33128801
ebi-a-GCST90001635	rs17304450	CD8dim NKT %lyr	19	6095431
ebi-a-GCST90001635	rs8131290	CD8dim NKT %lyr	21	45423006
ebi-a-GCST90001635	rs5998010	CD8dim NKT %lyr	22	31889080
ebi-a-GCST90001636	rs11580805	DN (CD4-CD8-) N	1	56114818
ebi-a-GCST90001636	rs11485850	DN (CD4-CD8-) N	1	248784570
ebi-a-GCST90001636	rs12143791	DN (CD4-CD8-) N	1	178071170
ebi-a-GCST90001636	rs4233371	DN (CD4-CD8-) N	1	161473125
ebi-a-GCST90001636	rs11124150	DN (CD4-CD8-) N	2	98905012
ebi-a-GCST90001636	rs112708092	DN (CD4-CD8-) N	2	236468046
ebi-a-GCST90001636	rs1473232	DN (CD4-CD8-) N	2	8498179
ebi-a-GCST90001636	rs34613828	DN (CD4-CD8-) N	2	87022759
ebi-a-GCST90001636	rs6785704	DN (CD4-CD8-) N	3	194020431
ebi-a-GCST90001636	rs62290986	DN (CD4-CD8-) N	3	194467784
ebi-a-GCST90001636	rs13108209	DN (CD4-CD8-) N	4	4354607
ebi-a-GCST90001637	rs59209359	DN (CD4-CD8-) N	1	56133478
ebi-a-GCST90001637	rs11485850	DN (CD4-CD8-) N	1	248784570
ebi-a-GCST90001637	rs4233371	DN (CD4-CD8-) N	1	161473125
ebi-a-GCST90001637	rs41274520	DN (CD4-CD8-) N	1	11210450
ebi-a-GCST90001637	rs3102960	DN (CD4-CD8-) N	2	8452497
ebi-a-GCST90001637	rs7569868	DN (CD4-CD8-) N	2	87019683
ebi-a-GCST90001637	rs188455143	DN (CD4-CD8-) N	2	179782799
ebi-a-GCST90001637	rs149977848	DN (CD4-CD8-) N	2	242038726
ebi-a-GCST90001637	rs11124150	DN (CD4-CD8-) N	2	98905012
ebi-a-GCST90001637	rs6785704	DN (CD4-CD8-) N	3	194020431
ebi-a-GCST90001637	rs265772	DN (CD4-CD8-) N	3	1371260
ebi-a-GCST90001637	rs13108209	DN (CD4-CD8-) N	4	4354607
ebi-a-GCST90001637	rs250103	DN (CD4-CD8-) N	5	141986611
ebi-a-GCST90001637	rs192734627	DN (CD4-CD8-) N	5	126590324
ebi-a-GCST90001637	rs201024668	DN (CD4-CD8-) N	6	98118004
ebi-a-GCST90001637	rs115536607	DN (CD4-CD8-) N	6	101417409

ebi-a-GCST90001637	rs117147408	DN (CD4-CD8-) N	6	101980039
ebi-a-GCST90001637	rs79251210	DN (CD4-CD8-) N	6	100468602
ebi-a-GCST90001637	rs2674389	DN (CD4-CD8-) N	6	93510469
ebi-a-GCST90001637	rs113145300	DN (CD4-CD8-) N	6	92830899
ebi-a-GCST90001637	rs9488834	DN (CD4-CD8-) N	6	97889936
ebi-a-GCST90001637	rs76158038	DN (CD4-CD8-) N	7	38595761
ebi-a-GCST90001637	rs77738700	DN (CD4-CD8-) N	11	112751407
ebi-a-GCST90001637	rs12792298	DN (CD4-CD8-) N	11	112189184
ebi-a-GCST90001637	rs190676386	DN (CD4-CD8-) N	11	114445844
ebi-a-GCST90001637	rs61925057	DN (CD4-CD8-) N	12	61076597
ebi-a-GCST90001637	rs148936961	DN (CD4-CD8-) N	12	61264786
ebi-a-GCST90001637	rs140444563	DN (CD4-CD8-) N	12	31103286
ebi-a-GCST90001637	rs9509337	DN (CD4-CD8-) N	13	19245376
ebi-a-GCST90001637	rs76333352	DN (CD4-CD8-) N	14	65546932
ebi-a-GCST90001637	rs113413210	DN (CD4-CD8-) N	15	78982526
ebi-a-GCST90001637	rs9896155	DN (CD4-CD8-) N	17	33794697
ebi-a-GCST90001637	rs9983338	DN (CD4-CD8-) N	21	29982668
ebi-a-GCST90001638	rs11485850	DN (CD4-CD8-) N	1	248784570
ebi-a-GCST90001638	rs11579742	DN (CD4-CD8-) N	1	175296847
ebi-a-GCST90001638	rs4233371	DN (CD4-CD8-) N	1	161473125
ebi-a-GCST90001638	rs59209359	DN (CD4-CD8-) N	1	56133478
ebi-a-GCST90001638	rs72784218	DN (CD4-CD8-) N	2	19698493
ebi-a-GCST90001638	rs149977848	DN (CD4-CD8-) N	2	242038726
ebi-a-GCST90001638	rs7569868	DN (CD4-CD8-) N	2	87019683
ebi-a-GCST90001638	rs11124150	DN (CD4-CD8-) N	2	98905012
ebi-a-GCST90001638	rs188455143	DN (CD4-CD8-) N	2	179782799
ebi-a-GCST90001638	rs6785704	DN (CD4-CD8-) N	3	194020431
ebi-a-GCST90001638	rs265772	DN (CD4-CD8-) N	3	1371260
ebi-a-GCST90001638	rs73191859	DN (CD4-CD8-) N	4	4222121
ebi-a-GCST90001638	rs192734627	DN (CD4-CD8-) N	5	126590324
ebi-a-GCST90001638	rs9488834	DN (CD4-CD8-) N	6	97889936
ebi-a-GCST90001638	rs79251210	DN (CD4-CD8-) N	6	100468602
ebi-a-GCST90001638	rs115536607	DN (CD4-CD8-) N	6	101417409
ebi-a-GCST90001638	rs201024668	DN (CD4-CD8-) N	6	98118004
ebi-a-GCST90001638	rs11759822	DN (CD4-CD8-) N	6	99608776
ebi-a-GCST90001638	rs117147408	DN (CD4-CD8-) N	6	101980039
ebi-a-GCST90001638	rs76158038	DN (CD4-CD8-) N	7	38595761
ebi-a-GCST90001638	rs77738700	DN (CD4-CD8-) N	11	112751407
ebi-a-GCST90001638	rs12792298	DN (CD4-CD8-) N	11	112189184
ebi-a-GCST90001638	rs148936961	DN (CD4-CD8-) N	12	61264786
ebi-a-GCST90001638	rs61925057	DN (CD4-CD8-) N	12	61076597
ebi-a-GCST90001638	rs140444563	DN (CD4-CD8-) N	12	31103286
ebi-a-GCST90001638	rs9509337	DN (CD4-CD8-) N	13	19245376
ebi-a-GCST90001638	rs76333352	DN (CD4-CD8-) N	14	65546932
ebi-a-GCST90001638	rs113413210	DN (CD4-CD8-) N	15	78982526
ebi-a-GCST90001638	rs8054431	DN (CD4-CD8-) N	16	27226437
ebi-a-GCST90001638	rs9916629	DN (CD4-CD8-) N	17	33796260
ebi-a-GCST90001638	rs11652532	DN (CD4-CD8-) N	17	33769883

ebi-a-GCST90001638	rs9983338	DN (CD4-CD8-) N	21	29982668
ebi-a-GCST90001639	rs61766955	CD3- lymphocyte A	1	31073453
ebi-a-GCST90001639	rs12616821	CD3- lymphocyte A	2	99282659
ebi-a-GCST90001639	rs17824379	CD3- lymphocyte A	3	5810961
ebi-a-GCST90001639	rs76620602	CD3- lymphocyte A	3	31651652
ebi-a-GCST90001639	rs17617028	CD3- lymphocyte A	4	106064683
ebi-a-GCST90001639	rs55738889	CD3- lymphocyte A	5	43983956
ebi-a-GCST90001639	rs17605177	CD3- lymphocyte A	7	126074598
ebi-a-GCST90001639	rs35604965	CD3- lymphocyte A	10	102722625
ebi-a-GCST90001639	rs11822514	CD3- lymphocyte A	11	125133704
ebi-a-GCST90001639	rs184712	CD3- lymphocyte A	12	58141602
ebi-a-GCST90001639	rs4763964	CD3- lymphocyte A	12	13468008
ebi-a-GCST90001639	rs73234444	CD3- lymphocyte A	13	83976964
ebi-a-GCST90001639	rs935320	CD3- lymphocyte A	15	57644442
ebi-a-GCST90001639	rs12915752	CD3- lymphocyte A	15	24590168
ebi-a-GCST90001639	rs3894193	CD3- lymphocyte A	17	38125346
ebi-a-GCST90001639	rs4796089	CD3- lymphocyte A	17	33814758
ebi-a-GCST90001639	rs9894744	CD3- lymphocyte A	17	72932879
ebi-a-GCST90001639	rs3795066	CD3- lymphocyte A	19	1039626
ebi-a-GCST90001639	rs144770721	CD3- lymphocyte A	21	32074074
ebi-a-GCST90001640	rs1874323	CD3- lymphocyte %	3	65771684
ebi-a-GCST90001640	rs4701789	CD3- lymphocyte %	5	7691703
ebi-a-GCST90001640	rs28832872	CD3- lymphocyte %	6	32529015
ebi-a-GCST90001640	rs241580	CD3- lymphocyte %	6	157895107
ebi-a-GCST90001640	rs34147569	CD3- lymphocyte %	6	71753094
ebi-a-GCST90001640	rs17615220	CD3- lymphocyte %	6	32677413
ebi-a-GCST90001640	rs6904670	CD3- lymphocyte %	6	31192797
ebi-a-GCST90001641	rs144820681	CD3- lymphocyte %	1	157319909
ebi-a-GCST90001641	rs76901853	CD3- lymphocyte %	2	232025880
ebi-a-GCST90001641	rs17824379	CD3- lymphocyte %	3	5810961
ebi-a-GCST90001641	rs1874323	CD3- lymphocyte %	3	65771684
ebi-a-GCST90001641	rs146849959	CD3- lymphocyte %	4	90761054
ebi-a-GCST90001641	rs4701789	CD3- lymphocyte %	5	7691703
ebi-a-GCST90001641	rs10484980	CD3- lymphocyte %	6	54451626
ebi-a-GCST90001641	rs10282551	CD3- lymphocyte %	7	135898321
ebi-a-GCST90001641	rs521446	CD3- lymphocyte %	9	136618314
ebi-a-GCST90001641	rs1649973	CD3- lymphocyte %	10	83888949
ebi-a-GCST90001641	rs145179037	CD3- lymphocyte %	10	122435643
ebi-a-GCST90001641	rs146347528	CD3- lymphocyte %	13	112890557
ebi-a-GCST90001641	rs12874404	CD3- lymphocyte %	13	108993494
ebi-a-GCST90001641	rs4581669	CD3- lymphocyte %	15	24585534
ebi-a-GCST90001641	rs138446406	CD3- lymphocyte %	15	54830492
ebi-a-GCST90001641	rs11870294	CD3- lymphocyte %	17	77121593
ebi-a-GCST90001641	rs4796089	CD3- lymphocyte %	17	33814758
ebi-a-GCST90001641	rs1476462	CD3- lymphocyte %	17	2707605
ebi-a-GCST90001641	rs77650974	CD3- lymphocyte %	18	12394263
ebi-a-GCST90001641	rs3730790	CD3- lymphocyte %	18	51815699
ebi-a-GCST90001642	rs490416	B cell AC	1	20838219

ebi-a-GCST90001642	rs17113368	B cell AC	1	95786229
ebi-a-GCST90001642	rs72941136	B cell AC	1	82245693
ebi-a-GCST90001642	rs6750281	B cell AC	2	99274418
ebi-a-GCST90001642	rs9883798	B cell AC	3	16473863
ebi-a-GCST90001642	rs2679580	B cell AC	3	8845344
ebi-a-GCST90001642	rs182527768	B cell AC	4	109048591
ebi-a-GCST90001642	rs1897558	B cell AC	5	167552605
ebi-a-GCST90001642	rs13173982	B cell AC	5	149268532
ebi-a-GCST90001642	rs10052651	B cell AC	5	18686084
ebi-a-GCST90001642	rs9460146	B cell AC	6	170723710
ebi-a-GCST90001642	rs117693242	B cell AC	6	104445996
ebi-a-GCST90001642	rs75270114	B cell AC	6	147667242
ebi-a-GCST90001642	rs7834992	B cell AC	8	119556485
ebi-a-GCST90001642	rs2395904	B cell AC	8	130617819
ebi-a-GCST90001642	rs73520180	B cell AC	9	118756021
ebi-a-GCST90001642	rs4933299	B cell AC	10	85456128
ebi-a-GCST90001642	rs142089808	B cell AC	11	116171120
ebi-a-GCST90001642	rs58114937	B cell AC	12	67638132
ebi-a-GCST90001642	rs1057686	B cell AC	12	120533696
ebi-a-GCST90001642	rs12865434	B cell AC	13	108970631
ebi-a-GCST90001642	rs11627066	B cell AC	14	66304049
ebi-a-GCST90001642	rs7500855	B cell AC	16	87692225
ebi-a-GCST90001642	rs7210666	B cell AC	17	74980293
ebi-a-GCST90001642	rs1966302	B cell AC	17	2718946
ebi-a-GCST90001642	rs3894193	B cell AC	17	38125346
ebi-a-GCST90001642	rs35606919	B cell AC	17	4353359
ebi-a-GCST90001642	rs62061834	B cell AC	17	49836652
ebi-a-GCST90001642	rs74390224	B cell AC	18	65360311
ebi-a-GCST90001642	rs11666499	B cell AC	19	10372310
ebi-a-GCST90001642	rs2033489	B cell AC	19	56768215
ebi-a-GCST90001642	rs5009526	B cell AC	20	38895000
ebi-a-GCST90001642	rs144825213	B cell AC	21	15364014
ebi-a-GCST90001642	rs5756641	B cell AC	22	37746783
ebi-a-GCST90001643	rs183053322	B cell % CD3- lymp	1	25076059
ebi-a-GCST90001643	rs12087279	B cell % CD3- lymp	1	82243292
ebi-a-GCST90001643	rs2679580	B cell % CD3- lymp	3	8845344
ebi-a-GCST90001643	rs191368650	B cell % CD3- lymp	4	110029714
ebi-a-GCST90001643	rs190518013	B cell % CD3- lymp	4	109649839
ebi-a-GCST90001643	rs34588768	B cell % CD3- lymp	4	55012690
ebi-a-GCST90001643	rs77438219	B cell % CD3- lymp	5	66776987
ebi-a-GCST90001643	rs56311672	B cell % CD3- lymp	5	106196268
ebi-a-GCST90001643	rs11954223	B cell % CD3- lymp	5	67413499
ebi-a-GCST90001643	rs117732815	B cell % CD3- lymp	7	98089427
ebi-a-GCST90001643	rs2213306	B cell % CD3- lymp	8	28615433
ebi-a-GCST90001643	rs142527102	B cell % CD3- lymp	8	17769401
ebi-a-GCST90001643	rs117515961	B cell % CD3- lymp	9	128278931
ebi-a-GCST90001643	rs73520180	B cell % CD3- lymp	9	118756021
ebi-a-GCST90001643	rs1915649	B cell % CD3- lymp	10	66556168

ebi-a-GCST90001643	rs72810613	B cell % CD3- lymp	10	95494689
ebi-a-GCST90001643	rs17823772	B cell % CD3- lymp	10	105928767
ebi-a-GCST90001643	rs12422149	B cell % CD3- lymp	11	74883577
ebi-a-GCST90001643	rs75210520	B cell % CD3- lymp	11	86506048
ebi-a-GCST90001643	rs61928632	B cell % CD3- lymp	12	85235042
ebi-a-GCST90001643	rs9520836	B cell % CD3- lymp	13	108957063
ebi-a-GCST90001643	rs78240988	B cell % CD3- lymp	14	76781310
ebi-a-GCST90001643	rs77651542	B cell % CD3- lymp	15	50374150
ebi-a-GCST90001643	rs7216096	B cell % CD3- lymp	17	33772954
ebi-a-GCST90001643	rs4239087	B cell % CD3- lymp	17	63434313
ebi-a-GCST90001643	rs9916257	B cell % CD3- lymp	17	33797371
ebi-a-GCST90001643	rs78468114	B cell % CD3- lymp	18	8581509
ebi-a-GCST90001643	rs281407	B cell % CD3- lymp	19	49233335
ebi-a-GCST90001643	rs73174907	B cell % CD3- lymp	22	43064030
ebi-a-GCST90001644	rs72941136	B cell %lymphocyte	1	82245693
ebi-a-GCST90001644	rs17113367	B cell %lymphocyte	1	95785786
ebi-a-GCST90001644	rs35911859	B cell %lymphocyte	1	235098109
ebi-a-GCST90001644	rs3738088	B cell %lymphocyte	1	18554670
ebi-a-GCST90001644	rs72810052	B cell %lymphocyte	2	25478461
ebi-a-GCST90001644	rs2679580	B cell %lymphocyte	3	8845344
ebi-a-GCST90001644	rs74535587	B cell %lymphocyte	3	8296294
ebi-a-GCST90001644	rs13108273	B cell %lymphocyte	4	25699326
ebi-a-GCST90001644	rs112526427	B cell %lymphocyte	4	151386190
ebi-a-GCST90001644	rs34588768	B cell %lymphocyte	4	55012690
ebi-a-GCST90001644	rs191368650	B cell %lymphocyte	4	110029714
ebi-a-GCST90001644	rs115698234	B cell %lymphocyte	5	66793608
ebi-a-GCST90001644	rs9368867	B cell %lymphocyte	6	11760807
ebi-a-GCST90001644	rs150774187	B cell %lymphocyte	7	100536678
ebi-a-GCST90001644	rs1557987	B cell %lymphocyte	7	26591929
ebi-a-GCST90001644	rs2395904	B cell %lymphocyte	8	130617819
ebi-a-GCST90001644	rs6985401	B cell %lymphocyte	8	27753061
ebi-a-GCST90001644	rs77233730	B cell %lymphocyte	8	118635981
ebi-a-GCST90001644	rs73520190	B cell %lymphocyte	9	118765180
ebi-a-GCST90001644	rs1572133	B cell %lymphocyte	10	85432638
ebi-a-GCST90001644	rs4748742	B cell %lymphocyte	10	7177305
ebi-a-GCST90001644	rs536579180	B cell %lymphocyte	11	14756839
ebi-a-GCST90001644	rs142089808	B cell %lymphocyte	11	116171120
ebi-a-GCST90001644	rs7483614	B cell %lymphocyte	11	32729647
ebi-a-GCST90001644	rs58114937	B cell %lymphocyte	12	67638132
ebi-a-GCST90001644	rs7990182	B cell %lymphocyte	13	110384132
ebi-a-GCST90001644	rs12874404	B cell %lymphocyte	13	108993494
ebi-a-GCST90001644	rs1408899	B cell %lymphocyte	13	108634581
ebi-a-GCST90001644	rs9520905	B cell %lymphocyte	13	109146439
ebi-a-GCST90001644	rs11627066	B cell %lymphocyte	14	66304049
ebi-a-GCST90001644	rs66777340	B cell %lymphocyte	17	2716327
ebi-a-GCST90001644	rs3894193	B cell %lymphocyte	17	38125346
ebi-a-GCST90001644	rs144426024	B cell %lymphocyte	18	56897917
ebi-a-GCST90001644	rs55658072	B cell %lymphocyte	19	14873284

ebi-a-GCST90001644	rs2223650	B cell %lymphocyte	20	9831513
ebi-a-GCST90001644	rs5754162	B cell %lymphocyte	22	32991307
ebi-a-GCST90001645	rs183053322	NK AC	1	25076059
ebi-a-GCST90001645	rs55971447	NK AC	1	161515326
ebi-a-GCST90001645	rs3789102	NK AC	2	111724375
ebi-a-GCST90001645	rs17824379	NK AC	3	5810961
ebi-a-GCST90001645	rs7736582	NK AC	5	40991344
ebi-a-GCST90001645	rs74938505	NK AC	5	89498010
ebi-a-GCST90001645	rs17605177	NK AC	7	126074598
ebi-a-GCST90001645	rs2922076	NK AC	8	99861962
ebi-a-GCST90001645	rs59536859	NK AC	9	126947962
ebi-a-GCST90001645	rs13287806	NK AC	9	133252691
ebi-a-GCST90001645	rs184712	NK AC	12	58141602
ebi-a-GCST90001645	rs597808	NK AC	12	111973358
ebi-a-GCST90001645	rs78664638	NK AC	13	85391377
ebi-a-GCST90001645	rs34341378	NK AC	13	74994547
ebi-a-GCST90001645	rs541558027	NK AC	14	87892054
ebi-a-GCST90001645	rs1257331	NK AC	14	99784255
ebi-a-GCST90001645	rs11637587	NK AC	15	46249557
ebi-a-GCST90001645	rs935320	NK AC	15	57644442
ebi-a-GCST90001645	rs7216096	NK AC	17	33772954
ebi-a-GCST90001645	rs9916257	NK AC	17	33797371
ebi-a-GCST90001645	rs6607353	NK AC	17	35252013
ebi-a-GCST90001645	rs150434441	NK AC	19	10409323
ebi-a-GCST90001646	rs12087279	NK %CD3- lymphc	1	82243292
ebi-a-GCST90001646	rs183053322	NK %CD3- lymphc	1	25076059
ebi-a-GCST90001646	rs55971447	NK %CD3- lymphc	1	161515326
ebi-a-GCST90001646	rs190518013	NK %CD3- lymphc	4	109649839
ebi-a-GCST90001646	rs34588768	NK %CD3- lymphc	4	55012690
ebi-a-GCST90001646	rs191368650	NK %CD3- lymphc	4	110029714
ebi-a-GCST90001646	rs11954223	NK %CD3- lymphc	5	67413499
ebi-a-GCST90001646	rs77438219	NK %CD3- lymphc	5	66776987
ebi-a-GCST90001646	rs35483654	NK %CD3- lymphc	5	133009855
ebi-a-GCST90001646	rs56311672	NK %CD3- lymphc	5	106196268
ebi-a-GCST90001646	rs56289531	NK %CD3- lymphc	7	36221410
ebi-a-GCST90001646	rs2213306	NK %CD3- lymphc	8	28615433
ebi-a-GCST90001646	rs142527102	NK %CD3- lymphc	8	17769401
ebi-a-GCST90001646	rs2623730	NK %CD3- lymphc	8	3612222
ebi-a-GCST90001646	rs146445024	NK %CD3- lymphc	9	96368316
ebi-a-GCST90001646	rs73520180	NK %CD3- lymphc	9	118756021
ebi-a-GCST90001646	rs1915649	NK %CD3- lymphc	10	66556168
ebi-a-GCST90001646	rs17823772	NK %CD3- lymphc	10	105928767
ebi-a-GCST90001646	rs2433423	NK %CD3- lymphc	11	86428029
ebi-a-GCST90001646	rs12874404	NK %CD3- lymphc	13	108993494
ebi-a-GCST90001646	rs4239087	NK %CD3- lymphc	17	63434313
ebi-a-GCST90001646	rs9916257	NK %CD3- lymphc	17	33797371
ebi-a-GCST90001646	rs7218011	NK %CD3- lymphc	17	33773022
ebi-a-GCST90001646	rs111661128	NK %CD3- lymphc	17	76403698

ebi-a-GCST90001646	rs78468114	NK %CD3- lymphc	18	8581509
ebi-a-GCST90001646	rs281407	NK %CD3- lymphc	19	49233335
ebi-a-GCST90001646	rs73174907	NK %CD3- lymphc	22	43064030
ebi-a-GCST90001647	rs115644251	NK %lymphocyte	1	97773324
ebi-a-GCST90001647	rs183053322	NK %lymphocyte	1	25076059
ebi-a-GCST90001647	rs144650982	NK %lymphocyte	1	93553003
ebi-a-GCST90001647	rs115345454	NK %lymphocyte	3	163251716
ebi-a-GCST90001647	rs6552764	NK %lymphocyte	4	185104503
ebi-a-GCST90001647	rs77438219	NK %lymphocyte	5	66776987
ebi-a-GCST90001647	rs56311672	NK %lymphocyte	5	106196268
ebi-a-GCST90001647	rs11954223	NK %lymphocyte	5	67413499
ebi-a-GCST90001647	rs2546701	NK %lymphocyte	5	168938336
ebi-a-GCST90001647	rs10948071	NK %lymphocyte	6	43280713
ebi-a-GCST90001647	rs2269705	NK %lymphocyte	6	30656847
ebi-a-GCST90001647	rs182077215	NK %lymphocyte	8	132608541
ebi-a-GCST90001647	rs10118740	NK %lymphocyte	9	122199843
ebi-a-GCST90001647	rs149863465	NK %lymphocyte	9	88631496
ebi-a-GCST90001647	rs2433423	NK %lymphocyte	11	86428029
ebi-a-GCST90001647	rs73027720	NK %lymphocyte	11	122327282
ebi-a-GCST90001647	rs7998044	NK %lymphocyte	13	110673614
ebi-a-GCST90001647	rs10512469	NK %lymphocyte	17	33770802
ebi-a-GCST90001647	rs9916257	NK %lymphocyte	17	33797371
ebi-a-GCST90001647	rs187230794	NK %lymphocyte	17	16149107
ebi-a-GCST90001647	rs113667813	NK %lymphocyte	17	76674023
ebi-a-GCST90001647	rs281414	NK %lymphocyte	19	10450285
ebi-a-GCST90001647	rs62130952	NK %lymphocyte	19	57182897
ebi-a-GCST90001647	rs281407	NK %lymphocyte	19	49233335
ebi-a-GCST90001647	rs2426800	NK %lymphocyte	20	58174503
ebi-a-GCST90001647	rs3787412	NK %lymphocyte	20	60481054
ebi-a-GCST90001647	rs57576269	NK %lymphocyte	21	42516902
ebi-a-GCST90001647	rs73174907	NK %lymphocyte	22	43064030
ebi-a-GCST90001648	rs71639910	HLA DR+ NK AC	1	161937774
ebi-a-GCST90001648	rs74341264	HLA DR+ NK AC	1	161561151
ebi-a-GCST90001648	rs71632989	HLA DR+ NK AC	1	161533055
ebi-a-GCST90001648	rs2880640	HLA DR+ NK AC	1	162879819
ebi-a-GCST90001648	rs75673714	HLA DR+ NK AC	2	42135201
ebi-a-GCST90001648	rs6750412	HLA DR+ NK AC	2	155323352
ebi-a-GCST90001648	rs79676986	HLA DR+ NK AC	2	102532879
ebi-a-GCST90001648	rs370476	HLA DR+ NK AC	3	5258431
ebi-a-GCST90001648	rs2047610	HLA DR+ NK AC	3	20847169
ebi-a-GCST90001648	rs1862176	HLA DR+ NK AC	5	139730444
ebi-a-GCST90001648	rs111621170	HLA DR+ NK AC	5	34861285
ebi-a-GCST90001648	rs35089291	HLA DR+ NK AC	8	21132144
ebi-a-GCST90001648	rs59613483	HLA DR+ NK AC	11	46937611
ebi-a-GCST90001648	rs79168765	HLA DR+ NK AC	13	90033065
ebi-a-GCST90001648	rs8025803	HLA DR+ NK AC	15	80260273
ebi-a-GCST90001648	rs182834114	HLA DR+ NK AC	16	9558024
ebi-a-GCST90001648	rs4076588	HLA DR+ NK AC	17	70686441

ebi-a-GCST90001648	rs139025334	HLA DR+ NK AC	20	48944963
ebi-a-GCST90001648	rs111288272	HLA DR+ NK AC	22	46804519
ebi-a-GCST90001649	rs865239	HLA DR+ NK %N	1	156896164
ebi-a-GCST90001649	rs71632989	HLA DR+ NK %N	1	161533055
ebi-a-GCST90001649	rs72685367	HLA DR+ NK %N	1	78442686
ebi-a-GCST90001649	rs74341264	HLA DR+ NK %N	1	161561151
ebi-a-GCST90001649	rs4656984	HLA DR+ NK %N	1	161078909
ebi-a-GCST90001649	rs71424967	HLA DR+ NK %N	2	238315335
ebi-a-GCST90001649	rs80171191	HLA DR+ NK %N	2	69059451
ebi-a-GCST90001649	rs3135080	HLA DR+ NK %N	4	2399058
ebi-a-GCST90001649	rs6833916	HLA DR+ NK %N	4	24948155
ebi-a-GCST90001649	rs142307180	HLA DR+ NK %N	4	1488517
ebi-a-GCST90001649	rs1862176	HLA DR+ NK %N	5	139730444
ebi-a-GCST90001649	rs61745530	HLA DR+ NK %N	6	47976361
ebi-a-GCST90001649	rs592776	HLA DR+ NK %N	6	153846268
ebi-a-GCST90001649	rs10260467	HLA DR+ NK %N	7	11300995
ebi-a-GCST90001649	rs17307458	HLA DR+ NK %N	8	60104360
ebi-a-GCST90001649	rs145013190	HLA DR+ NK %N	8	98791630
ebi-a-GCST90001649	rs56329333	HLA DR+ NK %N	9	118136745
ebi-a-GCST90001649	rs117338457	HLA DR+ NK %N	11	69354886
ebi-a-GCST90001649	rs17119267	HLA DR+ NK %N	14	84382486
ebi-a-GCST90001649	rs7180804	HLA DR+ NK %N	15	80259074
ebi-a-GCST90001649	rs72770017	HLA DR+ NK %N	16	10973612
ebi-a-GCST90001649	rs9916257	HLA DR+ NK %N	17	33797371
ebi-a-GCST90001649	rs12462814	HLA DR+ NK %N	19	29496714
ebi-a-GCST90001649	rs148177889	HLA DR+ NK %N	20	709089
ebi-a-GCST90001650	rs72685367	HLA DR+ NK %C	1	78442686
ebi-a-GCST90001650	rs2880640	HLA DR+ NK %C	1	162879819
ebi-a-GCST90001650	rs74341264	HLA DR+ NK %C	1	161561151
ebi-a-GCST90001650	rs71632979	HLA DR+ NK %C	1	161526397
ebi-a-GCST90001650	rs71639910	HLA DR+ NK %C	1	161937774
ebi-a-GCST90001650	rs79676986	HLA DR+ NK %C	2	102532879
ebi-a-GCST90001650	rs71424967	HLA DR+ NK %C	2	238315335
ebi-a-GCST90001650	rs4684938	HLA DR+ NK %C	3	1412674
ebi-a-GCST90001650	rs3135080	HLA DR+ NK %C	4	2399058
ebi-a-GCST90001650	rs1862176	HLA DR+ NK %C	5	139730444
ebi-a-GCST90001650	rs61745530	HLA DR+ NK %C	6	47976361
ebi-a-GCST90001650	rs592776	HLA DR+ NK %C	6	153846268
ebi-a-GCST90001650	rs10260467	HLA DR+ NK %C	7	11300995
ebi-a-GCST90001650	rs145013190	HLA DR+ NK %C	8	98791630
ebi-a-GCST90001650	rs4582908	HLA DR+ NK %C	10	86885802
ebi-a-GCST90001650	rs11595870	HLA DR+ NK %C	10	12408809
ebi-a-GCST90001650	rs117338457	HLA DR+ NK %C	11	69354886
ebi-a-GCST90001650	rs78479083	HLA DR+ NK %C	12	104596209
ebi-a-GCST90001650	rs144018284	HLA DR+ NK %C	13	48165147
ebi-a-GCST90001650	rs436053	HLA DR+ NK %C	15	67252716
ebi-a-GCST90001650	rs8025803	HLA DR+ NK %C	15	80260273
ebi-a-GCST90001650	rs62028061	HLA DR+ NK %C	16	10961519

ebi-a-GCST90001650	rs28816203	HLA DR+ NK %C	16	53157625
ebi-a-GCST90001650	rs4076588	HLA DR+ NK %C	17	70686441
ebi-a-GCST90001650	rs150102419	HLA DR+ NK %C	17	61353821
ebi-a-GCST90001650	rs1609454	HLA DR+ NK %C	19	9110431
ebi-a-GCST90001651	rs11265198	Granulocyte AC	1	159420307
ebi-a-GCST90001651	rs34599082	Granulocyte AC	1	159175494
ebi-a-GCST90001651	rs12618511	Granulocyte AC	2	1874340
ebi-a-GCST90001651	rs6757897	Granulocyte AC	2	66830010
ebi-a-GCST90001651	rs13383581	Granulocyte AC	2	70824249
ebi-a-GCST90001651	rs1914722	Granulocyte AC	2	50925528
ebi-a-GCST90001651	rs9851753	Granulocyte AC	3	143886418
ebi-a-GCST90001651	rs9852120	Granulocyte AC	3	23767055
ebi-a-GCST90001651	rs1563320	Granulocyte AC	5	97763960
ebi-a-GCST90001651	rs72762395	Granulocyte AC	5	43904012
ebi-a-GCST90001651	rs7805092	Granulocyte AC	7	2768935
ebi-a-GCST90001651	rs4074900	Granulocyte AC	8	144276224
ebi-a-GCST90001651	rs1890595	Granulocyte AC	9	35679591
ebi-a-GCST90001651	rs75350000	Granulocyte AC	10	11312276
ebi-a-GCST90001651	rs481252	Granulocyte AC	11	134188542
ebi-a-GCST90001651	rs671160	Granulocyte AC	11	117952387
ebi-a-GCST90001651	rs78270456	Granulocyte AC	11	5229028
ebi-a-GCST90001651	rs77799904	Granulocyte AC	12	3152264
ebi-a-GCST90001651	rs11158220	Granulocyte AC	14	59282769
ebi-a-GCST90001651	rs12450800	Granulocyte AC	17	38144116
ebi-a-GCST90001651	rs77650974	Granulocyte AC	18	12394263
ebi-a-GCST90001651	rs17879224	Granulocyte AC	19	4951885
ebi-a-GCST90001651	rs150954312	Granulocyte AC	19	50754898
ebi-a-GCST90001651	rs35566998	Granulocyte AC	20	60651297
ebi-a-GCST90001651	rs185512917	Granulocyte AC	20	48187302
ebi-a-GCST90001652	rs9729039	Granulocyte %leuk	1	226280379
ebi-a-GCST90001652	rs112405229	Granulocyte %leuk	1	240584083
ebi-a-GCST90001652	rs6701133	Granulocyte %leuk	1	62836414
ebi-a-GCST90001652	rs34599082	Granulocyte %leuk	1	159175494
ebi-a-GCST90001652	rs11805388	Granulocyte %leuk	1	172302386
ebi-a-GCST90001652	rs1116294	Granulocyte %leuk	2	49764298
ebi-a-GCST90001652	rs265772	Granulocyte %leuk	3	1371260
ebi-a-GCST90001652	rs893978	Granulocyte %leuk	3	74174855
ebi-a-GCST90001652	rs114188428	Granulocyte %leuk	3	39765597
ebi-a-GCST90001652	rs78277075	Granulocyte %leuk	4	183543742
ebi-a-GCST90001652	rs4588497	Granulocyte %leuk	4	44447116
ebi-a-GCST90001652	rs184624056	Granulocyte %leuk	4	259809
ebi-a-GCST90001652	rs35640478	Granulocyte %leuk	6	22954240
ebi-a-GCST90001652	rs11998531	Granulocyte %leuk	8	120860297
ebi-a-GCST90001652	rs74674596	Granulocyte %leuk	9	137658621
ebi-a-GCST90001652	rs79040043	Granulocyte %leuk	10	85710935
ebi-a-GCST90001652	rs8177701	Granulocyte %leuk	10	6003312
ebi-a-GCST90001652	rs2434066	Granulocyte %leuk	10	83891503
ebi-a-GCST90001652	rs481252	Granulocyte %leuk	11	134188542

ebi-a-GCST90001652	rs61415458	Granulocyte %leuk	12	20726031
ebi-a-GCST90001652	rs79855296	Granulocyte %leuk	14	100617766
ebi-a-GCST90001652	rs145016200	Granulocyte %leuk	14	101601125
ebi-a-GCST90001652	rs137979325	Granulocyte %leuk	17	36460632
ebi-a-GCST90001652	rs7219272	Granulocyte %leuk	17	9142206
ebi-a-GCST90001652	rs77650974	Granulocyte %leuk	18	12394263
ebi-a-GCST90001652	rs2837766	Granulocyte %leuk	21	42028282
ebi-a-GCST90001653	rs78686313	CD28- DN (CD4-C	1	14588462
ebi-a-GCST90001653	rs17187533	CD28- DN (CD4-C	1	208712244
ebi-a-GCST90001653	rs6734907	CD28- DN (CD4-C	2	106914029
ebi-a-GCST90001653	rs10174322	CD28- DN (CD4-C	2	7659940
ebi-a-GCST90001653	rs11128263	CD28- DN (CD4-C	3	72391950
ebi-a-GCST90001653	rs66989520	CD28- DN (CD4-C	4	147357834
ebi-a-GCST90001653	rs11930576	CD28- DN (CD4-C	4	169595404
ebi-a-GCST90001653	rs143066848	CD28- DN (CD4-C	5	83073780
ebi-a-GCST90001653	rs114170505	CD28- DN (CD4-C	6	32666487
ebi-a-GCST90001653	rs9488834	CD28- DN (CD4-C	6	97889936
ebi-a-GCST90001653	rs74593262	CD28- DN (CD4-C	6	15826226
ebi-a-GCST90001653	rs79967252	CD28- DN (CD4-C	7	143153812
ebi-a-GCST90001653	rs56009006	CD28- DN (CD4-C	7	132484052
ebi-a-GCST90001653	rs79012126	CD28- DN (CD4-C	9	138405739
ebi-a-GCST90001653	rs141291061	CD28- DN (CD4-C	9	71650333
ebi-a-GCST90001653	rs4391487	CD28- DN (CD4-C	9	94905724
ebi-a-GCST90001653	rs1875167	CD28- DN (CD4-C	10	2967899
ebi-a-GCST90001653	rs12770129	CD28- DN (CD4-C	10	132175707
ebi-a-GCST90001653	rs35409720	CD28- DN (CD4-C	11	277365
ebi-a-GCST90001653	rs61894112	CD28- DN (CD4-C	11	99376355
ebi-a-GCST90001653	rs7134223	CD28- DN (CD4-C	12	73854918
ebi-a-GCST90001653	rs78409217	CD28- DN (CD4-C	14	67161374
ebi-a-GCST90001653	rs75187775	CD28- DN (CD4-C	14	22497057
ebi-a-GCST90001653	rs149302210	CD28- DN (CD4-C	14	25870244
ebi-a-GCST90001653	rs4796089	CD28- DN (CD4-C	17	33814758
ebi-a-GCST90001653	rs9914549	CD28- DN (CD4-C	17	80624957
ebi-a-GCST90001653	rs59211785	CD28- DN (CD4-C	17	80030971
ebi-a-GCST90001653	rs1675243	CD28- DN (CD4-C	18	3668971
ebi-a-GCST90001654	rs138517625	CD28- DN (CD4-C	1	172681556
ebi-a-GCST90001654	rs115364254	CD28- DN (CD4-C	1	222677137
ebi-a-GCST90001654	rs556844566	CD28- DN (CD4-C	2	224185075
ebi-a-GCST90001654	rs111891110	CD28- DN (CD4-C	2	75659115
ebi-a-GCST90001654	rs6710361	CD28- DN (CD4-C	2	87047145
ebi-a-GCST90001654	rs115953192	CD28- DN (CD4-C	2	45208257
ebi-a-GCST90001654	rs139755840	CD28- DN (CD4-C	2	204522552
ebi-a-GCST90001654	rs76549873	CD28- DN (CD4-C	2	75722749
ebi-a-GCST90001654	rs11128263	CD28- DN (CD4-C	3	72391950
ebi-a-GCST90001654	rs66989520	CD28- DN (CD4-C	4	147357834
ebi-a-GCST90001654	rs1580183	CD28- DN (CD4-C	5	83043360
ebi-a-GCST90001654	rs4298345	CD28- DN (CD4-C	6	31240818
ebi-a-GCST90001654	rs79251210	CD28- DN (CD4-C	6	100468602

ebi-a-GCST90001654	rs137931178	CD28- DN (CD4-C	6	32329150
ebi-a-GCST90001654	rs9488834	CD28- DN (CD4-C	6	97889936
ebi-a-GCST90001654	rs74593262	CD28- DN (CD4-C	6	15826226
ebi-a-GCST90001654	rs10948242	CD28- DN (CD4-C	6	45553259
ebi-a-GCST90001654	rs139249541	CD28- DN (CD4-C	6	102294761
ebi-a-GCST90001654	rs140542258	CD28- DN (CD4-C	7	66909295
ebi-a-GCST90001654	rs7789422	CD28- DN (CD4-C	7	132432591
ebi-a-GCST90001654	rs2388191	CD28- DN (CD4-C	8	103637374
ebi-a-GCST90001654	rs7869045	CD28- DN (CD4-C	9	124584495
ebi-a-GCST90001654	rs10757255	CD28- DN (CD4-C	9	21776631
ebi-a-GCST90001654	rs36037322	CD28- DN (CD4-C	10	96943194
ebi-a-GCST90001654	rs10996820	CD28- DN (CD4-C	10	67760890
ebi-a-GCST90001654	rs61894112	CD28- DN (CD4-C	11	99376355
ebi-a-GCST90001654	rs10850001	CD28- DN (CD4-C	12	112553032
ebi-a-GCST90001654	rs140444563	CD28- DN (CD4-C	12	31103286
ebi-a-GCST90001654	rs35765554	CD28- DN (CD4-C	14	51318805
ebi-a-GCST90001654	rs11856833	CD28- DN (CD4-C	15	86807884
ebi-a-GCST90001654	rs140413577	CD28- DN (CD4-C	17	48437860
ebi-a-GCST90001654	rs12453786	CD28- DN (CD4-C	17	33272507
ebi-a-GCST90001654	rs9896155	CD28- DN (CD4-C	17	33794697
ebi-a-GCST90001654	rs9978065	CD28- DN (CD4-C	21	15247433
ebi-a-GCST90001655	rs72740660	CD28+ DN (CD4-C	1	215938244
ebi-a-GCST90001655	rs79892959	CD28+ DN (CD4-C	2	119667997
ebi-a-GCST90001655	rs3111416	CD28+ DN (CD4-C	2	8452313
ebi-a-GCST90001655	rs34546581	CD28+ DN (CD4-C	2	87021659
ebi-a-GCST90001655	rs115661735	CD28+ DN (CD4-C	2	65358919
ebi-a-GCST90001655	rs9880099	CD28+ DN (CD4-C	3	136255776
ebi-a-GCST90001655	rs1979672	CD28+ DN (CD4-C	3	46274109
ebi-a-GCST90001655	rs4077189	CD28+ DN (CD4-C	3	185796785
ebi-a-GCST90001655	rs144380153	CD28+ DN (CD4-C	5	169402564
ebi-a-GCST90001655	rs6890802	CD28+ DN (CD4-C	5	12611030
ebi-a-GCST90001655	rs112667234	CD28+ DN (CD4-C	5	68594062
ebi-a-GCST90001655	rs73503175	CD28+ DN (CD4-C	6	71939663
ebi-a-GCST90001655	rs55781756	CD28+ DN (CD4-C	6	32795706
ebi-a-GCST90001655	rs190300559	CD28+ DN (CD4-C	6	30718782
ebi-a-GCST90001655	rs35662273	CD28+ DN (CD4-C	6	7668809
ebi-a-GCST90001655	rs147994116	CD28+ DN (CD4-C	7	110080212
ebi-a-GCST90001655	rs111486611	CD28+ DN (CD4-C	7	85756157
ebi-a-GCST90001655	rs75581458	CD28+ DN (CD4-C	8	76719148
ebi-a-GCST90001655	rs10987993	CD28+ DN (CD4-C	9	101625377
ebi-a-GCST90001655	rs75679242	CD28+ DN (CD4-C	11	44090370
ebi-a-GCST90001655	rs57672161	CD28+ DN (CD4-C	14	101688448
ebi-a-GCST90001655	rs532816650	CD28+ DN (CD4-C	15	77852215
ebi-a-GCST90001655	rs17774204	CD28+ DN (CD4-C	17	21265508
ebi-a-GCST90001655	rs34825674	CD28+ DN (CD4-C	19	56583279
ebi-a-GCST90001655	rs941206	CD28+ DN (CD4-C	20	45261041
ebi-a-GCST90001656	rs17187533	CD28+ DN (CD4-C	1	208712244
ebi-a-GCST90001656	rs78686313	CD28+ DN (CD4-C	1	14588462

ebi-a-GCST90001656	rs6734907	CD28+ DN (CD4-C	2	106914029
ebi-a-GCST90001656	rs10174322	CD28+ DN (CD4-C	2	7659940
ebi-a-GCST90001656	rs11128263	CD28+ DN (CD4-C	3	72391950
ebi-a-GCST90001656	rs11930576	CD28+ DN (CD4-C	4	169595404
ebi-a-GCST90001656	rs66989520	CD28+ DN (CD4-C	4	147357834
ebi-a-GCST90001656	rs143066848	CD28+ DN (CD4-C	5	83073780
ebi-a-GCST90001656	rs114170505	CD28+ DN (CD4-C	6	32666487
ebi-a-GCST90001656	rs9488834	CD28+ DN (CD4-C	6	97889936
ebi-a-GCST90001656	rs74593262	CD28+ DN (CD4-C	6	15826226
ebi-a-GCST90001656	rs56009006	CD28+ DN (CD4-C	7	132484052
ebi-a-GCST90001656	rs79967252	CD28+ DN (CD4-C	7	143153812
ebi-a-GCST90001656	rs4391487	CD28+ DN (CD4-C	9	94905724
ebi-a-GCST90001656	rs141291061	CD28+ DN (CD4-C	9	71650333
ebi-a-GCST90001656	rs79012126	CD28+ DN (CD4-C	9	138405739
ebi-a-GCST90001656	rs1875167	CD28+ DN (CD4-C	10	2967899
ebi-a-GCST90001656	rs12770129	CD28+ DN (CD4-C	10	132175707
ebi-a-GCST90001656	rs35409720	CD28+ DN (CD4-C	11	277365
ebi-a-GCST90001656	rs61894112	CD28+ DN (CD4-C	11	99376355
ebi-a-GCST90001656	rs7134223	CD28+ DN (CD4-C	12	73854918
ebi-a-GCST90001656	rs75187775	CD28+ DN (CD4-C	14	22497057
ebi-a-GCST90001656	rs149302210	CD28+ DN (CD4-C	14	25870244
ebi-a-GCST90001656	rs78409217	CD28+ DN (CD4-C	14	67161374
ebi-a-GCST90001656	rs59211785	CD28+ DN (CD4-C	17	80030971
ebi-a-GCST90001656	rs4796089	CD28+ DN (CD4-C	17	33814758
ebi-a-GCST90001656	rs9914549	CD28+ DN (CD4-C	17	80624957
ebi-a-GCST90001656	rs1675243	CD28+ DN (CD4-C	18	3668971
ebi-a-GCST90001657	rs35723887	CD28+ DN (CD4-C	2	87021043
ebi-a-GCST90001657	rs77008070	CD28+ DN (CD4-C	2	15471323
ebi-a-GCST90001657	rs77805826	CD28+ DN (CD4-C	3	185798025
ebi-a-GCST90001657	rs73829619	CD28+ DN (CD4-C	4	68686165
ebi-a-GCST90001657	rs3757248	CD28+ DN (CD4-C	6	90657536
ebi-a-GCST90001657	rs111474820	CD28+ DN (CD4-C	9	4488885
ebi-a-GCST90001657	rs11818898	CD28+ DN (CD4-C	10	87516854
ebi-a-GCST90001657	rs116884829	CD28+ DN (CD4-C	11	77173517
ebi-a-GCST90001657	rs7128994	CD28+ DN (CD4-C	11	71242209
ebi-a-GCST90001657	rs57672161	CD28+ DN (CD4-C	14	101688448
ebi-a-GCST90001657	rs7214041	CD28+ DN (CD4-C	17	70401476
ebi-a-GCST90001657	rs6044409	CD28+ DN (CD4-C	20	16812087
ebi-a-GCST90001658	rs6686124	CD39+ CD4+ %T c	1	168745427
ebi-a-GCST90001658	rs115665382	CD39+ CD4+ %T c	3	116305711
ebi-a-GCST90001658	rs74346764	CD39+ CD4+ %T c	3	145230069
ebi-a-GCST90001658	rs114309505	CD39+ CD4+ %T c	4	4345603
ebi-a-GCST90001658	rs62421103	CD39+ CD4+ %T c	6	113160344
ebi-a-GCST90001658	rs6569455	CD39+ CD4+ %T c	6	126445961
ebi-a-GCST90001658	rs7753111	CD39+ CD4+ %T c	6	7730944
ebi-a-GCST90001658	rs2907958	CD39+ CD4+ %T c	7	156739135
ebi-a-GCST90001658	rs150315125	CD39+ CD4+ %T c	8	10090715
ebi-a-GCST90001658	rs34664008	CD39+ CD4+ %T c	8	113175429

ebi-a-GCST90001658	rs1999472	CD39+ CD4+ %T c	9	16186705
ebi-a-GCST90001658	rs145557266	CD39+ CD4+ %T c	10	95850055
ebi-a-GCST90001658	rs10762791	CD39+ CD4+ %T c	10	80308802
ebi-a-GCST90001658	rs150156752	CD39+ CD4+ %T c	10	110589119
ebi-a-GCST90001658	rs144769320	CD39+ CD4+ %T c	10	99527224
ebi-a-GCST90001658	rs4918960	CD39+ CD4+ %T c	10	97472570
ebi-a-GCST90001658	rs61862821	CD39+ CD4+ %T c	10	94513122
ebi-a-GCST90001658	rs12778797	CD39+ CD4+ %T c	10	96998688
ebi-a-GCST90001658	rs61886297	CD39+ CD4+ %T c	10	95830221
ebi-a-GCST90001658	rs10882701	CD39+ CD4+ %T c	10	97741071
ebi-a-GCST90001658	rs1410600	CD39+ CD4+ %T c	10	97070694
ebi-a-GCST90001658	rs11598645	CD39+ CD4+ %T c	10	68351521
ebi-a-GCST90001658	rs114844216	CD39+ CD4+ %T c	10	98158952
ebi-a-GCST90001658	rs12587599	CD39+ CD4+ %T c	14	104575130
ebi-a-GCST90001658	rs55992940	CD39+ CD4+ %T c	15	59639147
ebi-a-GCST90001658	rs151077900	CD39+ CD4+ %T c	16	1037963
ebi-a-GCST90001658	rs118130438	CD39+ CD4+ %T c	17	35962041
ebi-a-GCST90001658	rs11658050	CD39+ CD4+ %T c	17	75220287
ebi-a-GCST90001658	rs75420474	CD39+ CD4+ %T c	18	39828718
ebi-a-GCST90001658	rs144764562	CD39+ CD4+ %T c	19	43539305
ebi-a-GCST90001658	rs2286306	CD39+ CD4+ %T c	20	52558849
ebi-a-GCST90001658	rs144700141	CD39+ CD4+ %T c	20	16016296
ebi-a-GCST90001659	rs116034038	CD39+ CD4+ %CD	1	65028833
ebi-a-GCST90001659	rs78390127	CD39+ CD4+ %CD	2	77501356
ebi-a-GCST90001659	rs117667220	CD39+ CD4+ %CD	2	60655811
ebi-a-GCST90001659	rs10184974	CD39+ CD4+ %CD	2	5560128
ebi-a-GCST90001659	rs74346764	CD39+ CD4+ %CD	3	145230069
ebi-a-GCST90001659	rs12648210	CD39+ CD4+ %CD	4	89266460
ebi-a-GCST90001659	rs2907958	CD39+ CD4+ %CD	7	156739135
ebi-a-GCST90001659	rs139405606	CD39+ CD4+ %CD	7	53796473
ebi-a-GCST90001659	rs11784945	CD39+ CD4+ %CD	8	135708247
ebi-a-GCST90001659	rs34664008	CD39+ CD4+ %CD	8	113175429
ebi-a-GCST90001659	rs147888698	CD39+ CD4+ %CD	10	96576314
ebi-a-GCST90001659	rs10882701	CD39+ CD4+ %CD	10	97741071
ebi-a-GCST90001659	rs114844216	CD39+ CD4+ %CD	10	98158952
ebi-a-GCST90001659	rs144769320	CD39+ CD4+ %CD	10	99527224
ebi-a-GCST90001659	rs61873892	CD39+ CD4+ %CD	10	100118038
ebi-a-GCST90001659	rs145557266	CD39+ CD4+ %CD	10	95850055
ebi-a-GCST90001659	rs12778797	CD39+ CD4+ %CD	10	96998688
ebi-a-GCST90001659	rs10762791	CD39+ CD4+ %CD	10	80308802
ebi-a-GCST90001659	rs61886297	CD39+ CD4+ %CD	10	95830221
ebi-a-GCST90001659	rs1410600	CD39+ CD4+ %CD	10	97070694
ebi-a-GCST90001659	rs4918960	CD39+ CD4+ %CD	10	97472570
ebi-a-GCST90001659	rs74773804	CD39+ CD4+ %CD	11	108602539
ebi-a-GCST90001659	rs12587599	CD39+ CD4+ %CD	14	104575130
ebi-a-GCST90001659	rs184287492	CD39+ CD4+ %CD	15	81451157
ebi-a-GCST90001659	rs2008368	CD39+ CD4+ %CD	17	30347397
ebi-a-GCST90001659	rs11658050	CD39+ CD4+ %CD	17	75220287

ebi-a-GCST90001659	rs142629161	CD39+ CD4+ %CD	21	29333825
ebi-a-GCST90001660	rs10800381	CD39+ CD4+ AC	1	168744353
ebi-a-GCST90001660	rs191265355	CD39+ CD4+ AC	2	123442119
ebi-a-GCST90001660	rs11718018	CD39+ CD4+ AC	3	60364770
ebi-a-GCST90001660	rs138807420	CD39+ CD4+ AC	5	6231929
ebi-a-GCST90001660	rs72968495	CD39+ CD4+ AC	6	119638473
ebi-a-GCST90001660	rs62421103	CD39+ CD4+ AC	6	113160344
ebi-a-GCST90001660	rs113087753	CD39+ CD4+ AC	7	80556308
ebi-a-GCST90001660	rs57516532	CD39+ CD4+ AC	7	1172869
ebi-a-GCST90001660	rs139577399	CD39+ CD4+ AC	7	23203415
ebi-a-GCST90001660	rs78027772	CD39+ CD4+ AC	7	126248228
ebi-a-GCST90001660	rs35163827	CD39+ CD4+ AC	8	9606332
ebi-a-GCST90001660	rs74791100	CD39+ CD4+ AC	9	86052403
ebi-a-GCST90001660	rs10882701	CD39+ CD4+ AC	10	97741071
ebi-a-GCST90001660	rs114844216	CD39+ CD4+ AC	10	98158952
ebi-a-GCST90001660	rs4918960	CD39+ CD4+ AC	10	97472570
ebi-a-GCST90001660	rs1002938	CD39+ CD4+ AC	10	99461284
ebi-a-GCST90001660	rs2251521	CD39+ CD4+ AC	10	109471331
ebi-a-GCST90001660	rs12778797	CD39+ CD4+ AC	10	96998688
ebi-a-GCST90001660	rs11598645	CD39+ CD4+ AC	10	68351521
ebi-a-GCST90001660	rs112392069	CD39+ CD4+ AC	10	98925325
ebi-a-GCST90001660	rs10882324	CD39+ CD4+ AC	10	82674931
ebi-a-GCST90001660	rs145557266	CD39+ CD4+ AC	10	95850055
ebi-a-GCST90001660	rs1410600	CD39+ CD4+ AC	10	97070694
ebi-a-GCST90001660	rs821702	CD39+ CD4+ AC	10	108095070
ebi-a-GCST90001660	rs140503524	CD39+ CD4+ AC	11	44646709
ebi-a-GCST90001660	rs4321083	CD39+ CD4+ AC	13	84031916
ebi-a-GCST90001660	rs17091864	CD39+ CD4+ AC	14	57103379
ebi-a-GCST90001660	rs149951966	CD39+ CD4+ AC	17	41137136
ebi-a-GCST90001660	rs8076447	CD39+ CD4+ AC	17	75229883
ebi-a-GCST90001660	rs59143200	CD39+ CD4+ AC	19	1092511
ebi-a-GCST90001661	rs75436543	CD28- CD8dim %T	1	239777743
ebi-a-GCST90001661	rs12986962	CD28- CD8dim %T	2	111808558
ebi-a-GCST90001661	rs115545560	CD28- CD8dim %T	2	7793905
ebi-a-GCST90001661	rs115125396	CD28- CD8dim %T	2	147306619
ebi-a-GCST90001661	rs7634877	CD28- CD8dim %T	3	44822675
ebi-a-GCST90001661	rs116411882	CD28- CD8dim %T	3	134126390
ebi-a-GCST90001661	rs1561856	CD28- CD8dim %T	3	137321623
ebi-a-GCST90001661	rs4869279	CD28- CD8dim %T	5	95628918
ebi-a-GCST90001661	rs113075410	CD28- CD8dim %T	6	101435600
ebi-a-GCST90001661	rs9457418	CD28- CD8dim %T	6	159051908
ebi-a-GCST90001661	rs115695595	CD28- CD8dim %T	6	45300097
ebi-a-GCST90001661	rs182663933	CD28- CD8dim %T	6	103174948
ebi-a-GCST90001661	rs117147408	CD28- CD8dim %T	6	101980039
ebi-a-GCST90001661	rs626030	CD28- CD8dim %T	7	51947089
ebi-a-GCST90001661	rs11544989	CD28- CD8dim %T	8	145057868
ebi-a-GCST90001661	rs12360943	CD28- CD8dim %T	11	81817326
ebi-a-GCST90001661	rs117661587	CD28- CD8dim %T	12	10807555

ebi-a-GCST90001661	rs2274015	CD28- CD8dim %T	13	78180771
ebi-a-GCST90001661	rs142716558	CD28- CD8dim %T	14	51308539
ebi-a-GCST90001661	rs7152367	CD28- CD8dim %T	14	66292007
ebi-a-GCST90001661	rs4796089	CD28- CD8dim %T	17	33814758
ebi-a-GCST90001661	rs111748563	CD28- CD8dim %T	19	17901533
ebi-a-GCST90001661	rs12185529	CD28- CD8dim %T	19	3059754
ebi-a-GCST90001661	rs976051	CD28- CD8dim %T	21	16234888
ebi-a-GCST90001661	rs13054887	CD28- CD8dim %T	22	43169818
ebi-a-GCST90001662	rs2009581	CD28- CD8dim %C	2	111807677
ebi-a-GCST90001662	rs12470307	CD28- CD8dim %C	2	106923501
ebi-a-GCST90001662	rs115125396	CD28- CD8dim %C	2	147306619
ebi-a-GCST90001662	rs7649029	CD28- CD8dim %C	3	46753355
ebi-a-GCST90001662	rs147207959	CD28- CD8dim %C	4	182642975
ebi-a-GCST90001662	rs2710824	CD28- CD8dim %C	4	169596133
ebi-a-GCST90001662	rs4869279	CD28- CD8dim %C	5	95628918
ebi-a-GCST90001662	rs137931178	CD28- CD8dim %C	6	32329150
ebi-a-GCST90001662	rs142531546	CD28- CD8dim %C	7	37829371
ebi-a-GCST90001662	rs140865509	CD28- CD8dim %C	9	104945924
ebi-a-GCST90001662	rs2389116	CD28- CD8dim %C	12	33034357
ebi-a-GCST90001662	rs149704972	CD28- CD8dim %C	14	102846088
ebi-a-GCST90001662	rs76919728	CD28- CD8dim %C	16	30933851
ebi-a-GCST90001662	rs4796089	CD28- CD8dim %C	17	33814758
ebi-a-GCST90001662	rs1788822	CD28- CD8dim %C	18	21116999
ebi-a-GCST90001662	rs117082312	CD28- CD8dim %C	22	31767385
ebi-a-GCST90001663	rs75436543	CD28- CD8dim AC	1	239777743
ebi-a-GCST90001663	rs12986962	CD28- CD8dim AC	2	111808558
ebi-a-GCST90001663	rs115125396	CD28- CD8dim AC	2	147306619
ebi-a-GCST90001663	rs139090356	CD28- CD8dim AC	2	7451357
ebi-a-GCST90001663	rs79597088	CD28- CD8dim AC	3	74344603
ebi-a-GCST90001663	rs9836603	CD28- CD8dim AC	3	191611972
ebi-a-GCST90001663	rs7448311	CD28- CD8dim AC	5	95643248
ebi-a-GCST90001663	rs12204116	CD28- CD8dim AC	6	91469768
ebi-a-GCST90001663	rs13207893	CD28- CD8dim AC	6	32586922
ebi-a-GCST90001663	rs139249541	CD28- CD8dim AC	6	102294761
ebi-a-GCST90001663	rs79122093	CD28- CD8dim AC	6	101593157
ebi-a-GCST90001663	rs115695595	CD28- CD8dim AC	6	45300097
ebi-a-GCST90001663	rs111273508	CD28- CD8dim AC	6	25330739
ebi-a-GCST90001663	rs626030	CD28- CD8dim AC	7	51947089
ebi-a-GCST90001663	rs535307	CD28- CD8dim AC	7	105345398
ebi-a-GCST90001663	rs76396396	CD28- CD8dim AC	7	17214739
ebi-a-GCST90001663	rs10107063	CD28- CD8dim AC	8	123562235
ebi-a-GCST90001663	rs35947132	CD28- CD8dim AC	10	72360387
ebi-a-GCST90001663	rs80109323	CD28- CD8dim AC	10	133227859
ebi-a-GCST90001663	rs483239	CD28- CD8dim AC	10	96989103
ebi-a-GCST90001663	rs7937886	CD28- CD8dim AC	11	81903744
ebi-a-GCST90001663	rs10772894	CD28- CD8dim AC	12	16263287
ebi-a-GCST90001663	rs7990259	CD28- CD8dim AC	13	85954085
ebi-a-GCST90001663	rs7152367	CD28- CD8dim AC	14	66292007

ebi-a-GCST90001663	rs58745116	CD28- CD8dim AC	17	33803592
ebi-a-GCST90001663	rs12185529	CD28- CD8dim AC	19	3059754
ebi-a-GCST90001663	rs111748563	CD28- CD8dim AC	19	17901533
ebi-a-GCST90001663	rs142837365	CD28- CD8dim AC	22	41271935
ebi-a-GCST90001664	rs7530076	CD28+ CD45RA+	1	195825276
ebi-a-GCST90001664	rs34645541	CD28+ CD45RA+	1	195875605
ebi-a-GCST90001664	rs116794175	CD28+ CD45RA+	1	194010208
ebi-a-GCST90001664	rs541367304	CD28+ CD45RA+	1	198283058
ebi-a-GCST90001664	rs1341624	CD28+ CD45RA+	1	191433917
ebi-a-GCST90001664	rs1980606	CD28+ CD45RA+	1	160654786
ebi-a-GCST90001664	rs11579717	CD28+ CD45RA+	1	199999121
ebi-a-GCST90001664	rs12712610	CD28+ CD45RA+	2	38897249
ebi-a-GCST90001664	rs78905646	CD28+ CD45RA+	2	189898847
ebi-a-GCST90001664	rs7557121	CD28+ CD45RA+	2	240520878
ebi-a-GCST90001664	rs4832054	CD28+ CD45RA+	2	87058580
ebi-a-GCST90001664	rs7583259	CD28+ CD45RA+	2	38921934
ebi-a-GCST90001664	rs79827501	CD28+ CD45RA+	2	38431267
ebi-a-GCST90001664	rs468979	CD28+ CD45RA+	3	13489292
ebi-a-GCST90001664	rs74577787	CD28+ CD45RA+	3	84799491
ebi-a-GCST90001664	rs73197004	CD28+ CD45RA+	3	108545856
ebi-a-GCST90001664	rs114437347	CD28+ CD45RA+	4	86967620
ebi-a-GCST90001664	rs17627427	CD28+ CD45RA+	4	25959751
ebi-a-GCST90001664	rs183337752	CD28+ CD45RA+	5	19034933
ebi-a-GCST90001664	rs17480640	CD28+ CD45RA+	5	119666172
ebi-a-GCST90001664	rs113098887	CD28+ CD45RA+	6	84554072
ebi-a-GCST90001664	rs2252937	CD28+ CD45RA+	6	31461613
ebi-a-GCST90001664	rs78470849	CD28+ CD45RA+	6	31382675
ebi-a-GCST90001664	rs9461666	CD28+ CD45RA+	6	31207763
ebi-a-GCST90001664	rs112637846	CD28+ CD45RA+	6	149114553
ebi-a-GCST90001664	rs117174937	CD28+ CD45RA+	8	17606838
ebi-a-GCST90001664	rs117626397	CD28+ CD45RA+	10	131423918
ebi-a-GCST90001664	rs4964285	CD28+ CD45RA+	12	109318834
ebi-a-GCST90001664	rs12424807	CD28+ CD45RA+	12	14397702
ebi-a-GCST90001664	rs141049772	CD28+ CD45RA+	14	52982783
ebi-a-GCST90001664	rs11848822	CD28+ CD45RA+	14	102531420
ebi-a-GCST90001664	rs538856056	CD28+ CD45RA+	15	36549557
ebi-a-GCST90001664	rs3861195	CD28+ CD45RA+	15	32997150
ebi-a-GCST90001664	rs925236	CD28+ CD45RA+	15	99661608
ebi-a-GCST90001664	rs1948650	CD28+ CD45RA+	15	36040048
ebi-a-GCST90001664	rs149948027	CD28+ CD45RA+	16	89718638
ebi-a-GCST90001664	rs117988108	CD28+ CD45RA+	17	71781635
ebi-a-GCST90001664	rs75024282	CD28+ CD45RA+	19	30042758
ebi-a-GCST90001664	rs145827119	CD28+ CD45RA+	20	57132122
ebi-a-GCST90001664	rs140816141	CD28+ CD45RA+	22	28323098
ebi-a-GCST90001665	rs116794175	CD28+ CD45RA+	1	194010208
ebi-a-GCST90001665	rs34645541	CD28+ CD45RA+	1	195875605
ebi-a-GCST90001665	rs11579717	CD28+ CD45RA+	1	199999121
ebi-a-GCST90001665	rs541367304	CD28+ CD45RA+	1	198283058

ebi-a-GCST90001665	rs72771954	CD28+ CD45RA+ (	1	247539505
ebi-a-GCST90001665	rs17401833	CD28+ CD45RA+ (	1	14788274
ebi-a-GCST90001665	rs2367376	CD28+ CD45RA+ (	2	87009742
ebi-a-GCST90001665	rs7583259	CD28+ CD45RA+ (	2	38921934
ebi-a-GCST90001665	rs116478736	CD28+ CD45RA+ (	2	18033652
ebi-a-GCST90001665	rs12712610	CD28+ CD45RA+ (	2	38897249
ebi-a-GCST90001665	rs13001936	CD28+ CD45RA+ (	2	240573745
ebi-a-GCST90001665	rs79827501	CD28+ CD45RA+ (	2	38431267
ebi-a-GCST90001665	rs78181865	CD28+ CD45RA+ (	3	84481659
ebi-a-GCST90001665	rs76313555	CD28+ CD45RA+ (	3	59914042
ebi-a-GCST90001665	rs116760504	CD28+ CD45RA+ (	3	47546791
ebi-a-GCST90001665	rs73184461	CD28+ CD45RA+ (	3	189014593
ebi-a-GCST90001665	rs555202	CD28+ CD45RA+ (	3	174486633
ebi-a-GCST90001665	rs114437347	CD28+ CD45RA+ (	4	86967620
ebi-a-GCST90001665	rs34767257	CD28+ CD45RA+ (	6	30932606
ebi-a-GCST90001665	rs113888828	CD28+ CD45RA+ (	6	149157641
ebi-a-GCST90001665	rs9274730	CD28+ CD45RA+ (	6	32637817
ebi-a-GCST90001665	rs12378196	CD28+ CD45RA+ (	9	14963519
ebi-a-GCST90001665	rs145682498	CD28+ CD45RA+ (	10	54508949
ebi-a-GCST90001665	rs67486389	CD28+ CD45RA+ (	12	19926432
ebi-a-GCST90001665	rs4984382	CD28+ CD45RA+ (	15	94882509
ebi-a-GCST90001665	rs74021626	CD28+ CD45RA+ (	15	66417100
ebi-a-GCST90001665	rs34854932	CD28+ CD45RA+ (	16	83875573
ebi-a-GCST90001665	rs8127300	CD28+ CD45RA+ (	21	43529642
ebi-a-GCST90001665	rs6001084	CD28+ CD45RA+ (	22	38681644
ebi-a-GCST90001666	rs2185480	CD28+ CD45RA+ (	1	176186935
ebi-a-GCST90001666	rs541367304	CD28+ CD45RA+ (	1	198283058
ebi-a-GCST90001666	rs1341624	CD28+ CD45RA+ (	1	191433917
ebi-a-GCST90001666	rs7530076	CD28+ CD45RA+ (	1	195825276
ebi-a-GCST90001666	rs11579717	CD28+ CD45RA+ (	1	199999121
ebi-a-GCST90001666	rs138298107	CD28+ CD45RA+ (	1	213203523
ebi-a-GCST90001666	rs116794175	CD28+ CD45RA+ (	1	194010208
ebi-a-GCST90001666	rs10875036	CD28+ CD45RA+ (	1	97288628
ebi-a-GCST90001666	rs78905646	CD28+ CD45RA+ (	2	189898847
ebi-a-GCST90001666	rs139618773	CD28+ CD45RA+ (	2	45403427
ebi-a-GCST90001666	rs12712610	CD28+ CD45RA+ (	2	38897249
ebi-a-GCST90001666	rs7557121	CD28+ CD45RA+ (	2	240520878
ebi-a-GCST90001666	rs61038907	CD28+ CD45RA+ (	2	87061070
ebi-a-GCST90001666	rs79827501	CD28+ CD45RA+ (	2	38431267
ebi-a-GCST90001666	rs35884179	CD28+ CD45RA+ (	2	26163168
ebi-a-GCST90001666	rs7583259	CD28+ CD45RA+ (	2	38921934
ebi-a-GCST90001666	rs115376296	CD28+ CD45RA+ (	2	230763199
ebi-a-GCST90001666	rs74577787	CD28+ CD45RA+ (	3	84799491
ebi-a-GCST90001666	rs56112048	CD28+ CD45RA+ (	3	137006597
ebi-a-GCST90001666	rs76313555	CD28+ CD45RA+ (	3	59914042
ebi-a-GCST90001666	rs468979	CD28+ CD45RA+ (	3	13489292
ebi-a-GCST90001666	rs146985291	CD28+ CD45RA+ (	3	170169608
ebi-a-GCST90001666	rs114437347	CD28+ CD45RA+ (	4	86967620

ebi-a-GCST90001666	rs35471013	CD28+ CD45RA+ (	4	37794059
ebi-a-GCST90001666	rs138960714	CD28+ CD45RA+ (	4	123422719
ebi-a-GCST90001666	rs574807	CD28+ CD45RA+ (	6	145543700
ebi-a-GCST90001666	rs112637846	CD28+ CD45RA+ (	6	149114553
ebi-a-GCST90001666	rs113098887	CD28+ CD45RA+ (	6	84554072
ebi-a-GCST90001666	rs117403703	CD28+ CD45RA+ (	6	93203850
ebi-a-GCST90001666	rs184082746	CD28+ CD45RA+ (	7	92791863
ebi-a-GCST90001666	rs80172536	CD28+ CD45RA+ (	7	110737584
ebi-a-GCST90001666	rs182236332	CD28+ CD45RA+ (	7	124954943
ebi-a-GCST90001666	rs13243221	CD28+ CD45RA+ (	7	80880540
ebi-a-GCST90001666	rs192475369	CD28+ CD45RA+ (	9	33552507
ebi-a-GCST90001666	rs7096922	CD28+ CD45RA+ (	10	119371841
ebi-a-GCST90001666	rs80194890	CD28+ CD45RA+ (	10	91332621
ebi-a-GCST90001666	rs117513254	CD28+ CD45RA+ (	12	126913667
ebi-a-GCST90001666	rs141049772	CD28+ CD45RA+ (	14	52982783
ebi-a-GCST90001666	rs1948650	CD28+ CD45RA+ (	15	36040048
ebi-a-GCST90001666	rs72763382	CD28+ CD45RA+ (	15	97953052
ebi-a-GCST90001666	rs12907798	CD28+ CD45RA+ (	15	36349479
ebi-a-GCST90001666	rs149948027	CD28+ CD45RA+ (	16	89718638
ebi-a-GCST90001666	rs117733745	CD28+ CD45RA+ (	16	15158014
ebi-a-GCST90001666	rs144591335	CD28+ CD45RA+ (	16	59010741
ebi-a-GCST90001666	rs1005513	CD28+ CD45RA+ (	17	49196918
ebi-a-GCST90001666	rs10451363	CD28+ CD45RA+ (	18	9939517
ebi-a-GCST90001666	rs77935824	CD28+ CD45RA+ (	18	68968361
ebi-a-GCST90001666	rs145827119	CD28+ CD45RA+ (	20	57132122
ebi-a-GCST90001666	rs62229786	CD28+ CD45RA+ (	20	50098150
ebi-a-GCST90001666	rs73193728	CD28+ CD45RA+ (	21	32665618
ebi-a-GCST90001666	rs140816141	CD28+ CD45RA+ (	22	28323098
ebi-a-GCST90001667	rs116759767	CD28+ CD45RA- C	1	189981116
ebi-a-GCST90001667	rs11579717	CD28+ CD45RA- C	1	199999121
ebi-a-GCST90001667	rs17130468	CD28+ CD45RA- C	1	68567192
ebi-a-GCST90001667	rs147612560	CD28+ CD45RA- C	1	201600103
ebi-a-GCST90001667	rs541367304	CD28+ CD45RA- C	1	198283058
ebi-a-GCST90001667	rs17216040	CD28+ CD45RA- C	2	129145907
ebi-a-GCST90001667	rs1400032	CD28+ CD45RA- C	2	86923183
ebi-a-GCST90001667	rs34973688	CD28+ CD45RA- C	2	133470672
ebi-a-GCST90001667	rs1975288	CD28+ CD45RA- C	2	45109063
ebi-a-GCST90001667	rs138232290	CD28+ CD45RA- C	4	40701822
ebi-a-GCST90001667	rs115370355	CD28+ CD45RA- C	4	92620279
ebi-a-GCST90001667	rs62358725	CD28+ CD45RA- C	5	51967646
ebi-a-GCST90001667	rs74463547	CD28+ CD45RA- C	5	25574181
ebi-a-GCST90001667	rs114968045	CD28+ CD45RA- C	6	32666960
ebi-a-GCST90001667	rs10456250	CD28+ CD45RA- C	6	22061048
ebi-a-GCST90001667	rs2177324	CD28+ CD45RA- C	8	123605760
ebi-a-GCST90001667	rs73184766	CD28+ CD45RA- C	8	1968214
ebi-a-GCST90001667	rs12235775	CD28+ CD45RA- C	9	23394831
ebi-a-GCST90001667	rs76359108	CD28+ CD45RA- C	10	68754597
ebi-a-GCST90001667	rs185416365	CD28+ CD45RA- C	13	42211264

ebi-a-GCST90001667	rs10134215	CD28+ CD45RA- (	14	34455424
ebi-a-GCST90001667	rs138678985	CD28+ CD45RA- (	14	37895643
ebi-a-GCST90001667	rs12914989	CD28+ CD45RA- (	15	26221496
ebi-a-GCST90001667	rs117455482	CD28+ CD45RA- (	15	90520252
ebi-a-GCST90001667	rs9912771	CD28+ CD45RA- (	17	14307085
ebi-a-GCST90001667	rs61744818	CD28+ CD45RA- (	17	74163759
ebi-a-GCST90001667	rs72848176	CD28+ CD45RA- (	17	65576640
ebi-a-GCST90001667	rs139641355	CD28+ CD45RA- (	19	52441118
ebi-a-GCST90001667	rs687264	CD28+ CD45RA- (	22	20172901
ebi-a-GCST90001668	rs541367304	CD28+ CD45RA- (	1	198283058
ebi-a-GCST90001668	rs138915779	CD28+ CD45RA- (	1	190079170
ebi-a-GCST90001668	rs11579717	CD28+ CD45RA- (	1	199999121
ebi-a-GCST90001668	rs72694845	CD28+ CD45RA- (	1	151909812
ebi-a-GCST90001668	rs78170509	CD28+ CD45RA- (	1	97340174
ebi-a-GCST90001668	rs55711612	CD28+ CD45RA- (	2	111845422
ebi-a-GCST90001668	rs78336738	CD28+ CD45RA- (	2	54934160
ebi-a-GCST90001668	rs111561108	CD28+ CD45RA- (	2	107926407
ebi-a-GCST90001668	rs7649029	CD28+ CD45RA- (	3	46753355
ebi-a-GCST90001668	rs2710824	CD28+ CD45RA- (	4	169596133
ebi-a-GCST90001668	rs147207959	CD28+ CD45RA- (	4	182642975
ebi-a-GCST90001668	rs7679241	CD28+ CD45RA- (	4	26288550
ebi-a-GCST90001668	rs10517119	CD28+ CD45RA- (	4	44859711
ebi-a-GCST90001668	rs1997323	CD28+ CD45RA- (	5	87220656
ebi-a-GCST90001668	rs137931178	CD28+ CD45RA- (	6	32329150
ebi-a-GCST90001668	rs142531546	CD28+ CD45RA- (	7	37829371
ebi-a-GCST90001668	rs78310542	CD28+ CD45RA- (	8	103052789
ebi-a-GCST90001668	rs2177324	CD28+ CD45RA- (	8	123605760
ebi-a-GCST90001668	rs118141156	CD28+ CD45RA- (	10	82705665
ebi-a-GCST90001668	rs117626397	CD28+ CD45RA- (	10	131423918
ebi-a-GCST90001668	rs72774994	CD28+ CD45RA- (	10	2957054
ebi-a-GCST90001668	rs12576570	CD28+ CD45RA- (	11	8243002
ebi-a-GCST90001668	rs2085746	CD28+ CD45RA- (	11	61924407
ebi-a-GCST90001668	rs7316570	CD28+ CD45RA- (	12	33069361
ebi-a-GCST90001668	rs12231396	CD28+ CD45RA- (	12	100112638
ebi-a-GCST90001668	rs149704972	CD28+ CD45RA- (	14	102846088
ebi-a-GCST90001668	rs71386709	CD28+ CD45RA- (	16	12613868
ebi-a-GCST90001668	rs76919728	CD28+ CD45RA- (	16	30933851
ebi-a-GCST90001668	rs4796089	CD28+ CD45RA- (	17	33814758
ebi-a-GCST90001668	rs56206359	CD28+ CD45RA- (	19	35329974
ebi-a-GCST90001669	rs72631887	CD28+ CD45RA- (	1	835092
ebi-a-GCST90001669	rs147612560	CD28+ CD45RA- (	1	201600103
ebi-a-GCST90001669	rs138915779	CD28+ CD45RA- (	1	190079170
ebi-a-GCST90001669	rs541367304	CD28+ CD45RA- (	1	198283058
ebi-a-GCST90001669	rs34645541	CD28+ CD45RA- (	1	195875605
ebi-a-GCST90001669	rs4129441	CD28+ CD45RA- (	1	241904201
ebi-a-GCST90001669	rs11579717	CD28+ CD45RA- (	1	199999121
ebi-a-GCST90001669	rs111432170	CD28+ CD45RA- (	1	175728032
ebi-a-GCST90001669	rs28527279	CD28+ CD45RA- (	2	86948835

ebi-a-GCST90001669	rs61269136	CD28+ CD45RA- (	3	61511035
ebi-a-GCST90001669	rs2670003	CD28+ CD45RA- (	3	9087967
ebi-a-GCST90001669	rs35035328	CD28+ CD45RA- (	3	46276078
ebi-a-GCST90001669	rs74732038	CD28+ CD45RA- (	4	174826726
ebi-a-GCST90001669	rs138232290	CD28+ CD45RA- (	4	40701822
ebi-a-GCST90001669	rs1450949	CD28+ CD45RA- (	5	51919136
ebi-a-GCST90001669	rs35222020	CD28+ CD45RA- (	6	149101822
ebi-a-GCST90001669	rs13231184	CD28+ CD45RA- (	7	28364598
ebi-a-GCST90001669	rs11770198	CD28+ CD45RA- (	7	142351498
ebi-a-GCST90001669	rs78971171	CD28+ CD45RA- (	7	86261382
ebi-a-GCST90001669	rs116499679	CD28+ CD45RA- (	8	28528105
ebi-a-GCST90001669	rs62488624	CD28+ CD45RA- (	8	36903125
ebi-a-GCST90001669	rs138728019	CD28+ CD45RA- (	8	6788445
ebi-a-GCST90001669	rs12235775	CD28+ CD45RA- (	9	23394831
ebi-a-GCST90001669	rs117626397	CD28+ CD45RA- (	10	131423918
ebi-a-GCST90001669	rs116884829	CD28+ CD45RA- (	11	77173517
ebi-a-GCST90001669	rs77547518	CD28+ CD45RA- (	11	9143785
ebi-a-GCST90001669	rs143559470	CD28+ CD45RA- (	11	115711814
ebi-a-GCST90001669	rs181777922	CD28+ CD45RA- (	12	1631566
ebi-a-GCST90001669	rs78228882	CD28+ CD45RA- (	13	107306559
ebi-a-GCST90001669	rs138678985	CD28+ CD45RA- (	14	37895643
ebi-a-GCST90001669	rs34487669	CD28+ CD45RA- (	15	26227349
ebi-a-GCST90001669	rs76098298	CD28+ CD45RA- (	17	54788402
ebi-a-GCST90001669	rs2585739	CD28+ CD45RA- (	17	74135619
ebi-a-GCST90001669	rs565801	CD28+ CD45RA- (	19	7837186
ebi-a-GCST90001669	rs117586381	CD28+ CD45RA- (	19	18683695
ebi-a-GCST90001669	rs687264	CD28+ CD45RA- (	22	20172901
ebi-a-GCST90001669	rs139127	CD28+ CD45RA- (	22	44578288
ebi-a-GCST90001670	rs7677831	CD39+ CD8br %T	4	82648490
ebi-a-GCST90001670	rs58895686	CD39+ CD8br %T	5	43984994
ebi-a-GCST90001670	rs202075317	CD39+ CD8br %T	6	68997428
ebi-a-GCST90001670	rs1861381	CD39+ CD8br %T	7	97100379
ebi-a-GCST90001670	rs78311694	CD39+ CD8br %T	7	69867672
ebi-a-GCST90001670	rs34664008	CD39+ CD8br %T	8	113175429
ebi-a-GCST90001670	rs16909016	CD39+ CD8br %T	9	96068328
ebi-a-GCST90001670	rs140079267	CD39+ CD8br %T	10	93272233
ebi-a-GCST90001670	rs11818051	CD39+ CD8br %T	10	97972294
ebi-a-GCST90001670	rs117648214	CD39+ CD8br %T	10	98816434
ebi-a-GCST90001670	rs12778618	CD39+ CD8br %T	10	95633120
ebi-a-GCST90001670	rs72812685	CD39+ CD8br %T	10	95845989
ebi-a-GCST90001670	rs12778837	CD39+ CD8br %T	10	96998744
ebi-a-GCST90001670	rs11812465	CD39+ CD8br %T	10	91738825
ebi-a-GCST90001670	rs111534725	CD39+ CD8br %T	10	98139264
ebi-a-GCST90001670	rs111899271	CD39+ CD8br %T	10	70317419
ebi-a-GCST90001670	rs17111341	CD39+ CD8br %T	10	97881031
ebi-a-GCST90001670	rs4918960	CD39+ CD8br %T	10	97472570
ebi-a-GCST90001670	rs2631687	CD39+ CD8br %T	10	93057020
ebi-a-GCST90001670	rs184287492	CD39+ CD8br %T	15	81451157

ebi-a-GCST90001670	rs7182699	CD39+ CD8br %T	15	94355818
ebi-a-GCST90001671	rs241207	CD39+ CD8br %Cl	1	4634084
ebi-a-GCST90001671	rs7677831	CD39+ CD8br %Cl	4	82648490
ebi-a-GCST90001671	rs73814898	CD39+ CD8br %Cl	4	53758992
ebi-a-GCST90001671	rs58895686	CD39+ CD8br %Cl	5	43984994
ebi-a-GCST90001671	rs142712866	CD39+ CD8br %Cl	8	124323593
ebi-a-GCST90001671	rs34664008	CD39+ CD8br %Cl	8	113175429
ebi-a-GCST90001671	rs140079267	CD39+ CD8br %Cl	10	93272233
ebi-a-GCST90001671	rs2631687	CD39+ CD8br %Cl	10	93057020
ebi-a-GCST90001671	rs72812685	CD39+ CD8br %Cl	10	95845989
ebi-a-GCST90001671	rs72826245	CD39+ CD8br %Cl	10	99903526
ebi-a-GCST90001671	rs4918960	CD39+ CD8br %Cl	10	97472570
ebi-a-GCST90001671	rs12778618	CD39+ CD8br %Cl	10	95633120
ebi-a-GCST90001671	rs2901833	CD39+ CD8br %Cl	10	96998591
ebi-a-GCST90001671	rs111534725	CD39+ CD8br %Cl	10	98139264
ebi-a-GCST90001671	rs146426185	CD39+ CD8br %Cl	10	99452494
ebi-a-GCST90001671	rs17111341	CD39+ CD8br %Cl	10	97881031
ebi-a-GCST90001671	rs11818051	CD39+ CD8br %Cl	10	97972294
ebi-a-GCST90001671	rs111899271	CD39+ CD8br %Cl	10	70317419
ebi-a-GCST90001671	rs1995763	CD39+ CD8br %Cl	11	134726590
ebi-a-GCST90001671	rs76225630	CD39+ CD8br %Cl	13	34074087
ebi-a-GCST90001671	rs1332595	CD39+ CD8br %Cl	13	77106508
ebi-a-GCST90001671	rs9899828	CD39+ CD8br %Cl	17	71595627
ebi-a-GCST90001672	rs62156361	CD39+ CD8br AC	2	99679099
ebi-a-GCST90001672	rs11718018	CD39+ CD8br AC	3	60364770
ebi-a-GCST90001672	rs113398680	CD39+ CD8br AC	3	145339577
ebi-a-GCST90001672	rs7677831	CD39+ CD8br AC	4	82648490
ebi-a-GCST90001672	rs149697028	CD39+ CD8br AC	4	42155788
ebi-a-GCST90001672	rs1861381	CD39+ CD8br AC	7	97100379
ebi-a-GCST90001672	rs34664008	CD39+ CD8br AC	8	113175429
ebi-a-GCST90001672	rs750587	CD39+ CD8br AC	9	362917
ebi-a-GCST90001672	rs12778797	CD39+ CD8br AC	10	96998688
ebi-a-GCST90001672	rs72812685	CD39+ CD8br AC	10	95845989
ebi-a-GCST90001672	rs35664	CD39+ CD8br AC	10	80490882
ebi-a-GCST90001672	rs111534725	CD39+ CD8br AC	10	98139264
ebi-a-GCST90001672	rs72826245	CD39+ CD8br AC	10	99903526
ebi-a-GCST90001672	rs4918960	CD39+ CD8br AC	10	97472570
ebi-a-GCST90001672	rs2631687	CD39+ CD8br AC	10	93057020
ebi-a-GCST90001672	rs140079267	CD39+ CD8br AC	10	93272233
ebi-a-GCST90001672	rs12778618	CD39+ CD8br AC	10	95633120
ebi-a-GCST90001672	rs11818051	CD39+ CD8br AC	10	97972294
ebi-a-GCST90001672	rs17111341	CD39+ CD8br AC	10	97881031
ebi-a-GCST90001672	rs146426185	CD39+ CD8br AC	10	99452494
ebi-a-GCST90001672	rs111899271	CD39+ CD8br AC	10	70317419
ebi-a-GCST90001672	rs11257350	CD39+ CD8br AC	10	11874754
ebi-a-GCST90001672	rs146940094	CD39+ CD8br AC	15	94946993
ebi-a-GCST90001672	rs17509944	CD39+ CD8br AC	15	50717609
ebi-a-GCST90001672	rs149574697	CD39+ CD8br AC	18	67477449

ebi-a-GCST90001673	rs58434301	CD28- CD127- CD	1	233952844
ebi-a-GCST90001673	rs142394211	CD28- CD127- CD	1	20454583
ebi-a-GCST90001673	rs35089045	CD28- CD127- CD	1	57910243
ebi-a-GCST90001673	rs146970469	CD28- CD127- CD	2	30292698
ebi-a-GCST90001673	rs62166861	CD28- CD127- CD	2	122691344
ebi-a-GCST90001673	rs9823305	CD28- CD127- CD	3	169748799
ebi-a-GCST90001673	rs186154	CD28- CD127- CD	3	60305117
ebi-a-GCST90001673	rs115642223	CD28- CD127- CD	4	141162325
ebi-a-GCST90001673	rs6864137	CD28- CD127- CD	5	49624091
ebi-a-GCST90001673	rs9269118	CD28- CD127- CD	6	32443641
ebi-a-GCST90001673	rs111856210	CD28- CD127- CD	7	7026180
ebi-a-GCST90001673	rs56098200	CD28- CD127- CD	8	62998411
ebi-a-GCST90001673	rs74139363	CD28- CD127- CD	10	71528846
ebi-a-GCST90001673	rs7963204	CD28- CD127- CD	12	52559164
ebi-a-GCST90001673	rs990962	CD28- CD127- CD	14	22567561
ebi-a-GCST90001673	rs1871360	CD28- CD127- CD	15	95276540
ebi-a-GCST90001673	rs79000044	CD28- CD127- CD	17	67264572
ebi-a-GCST90001673	rs12450916	CD28- CD127- CD	17	74615933
ebi-a-GCST90001673	rs10411958	CD28- CD127- CD	19	13113641
ebi-a-GCST90001673	rs6085043	CD28- CD127- CD	20	5222441
ebi-a-GCST90001673	rs1015011	CD28- CD127- CD	22	24893021
ebi-a-GCST90001674	rs35089045	CD28- CD127- CD	1	57910243
ebi-a-GCST90001674	rs79410072	CD28- CD127- CD	2	66204309
ebi-a-GCST90001674	rs13068352	CD28- CD127- CD	3	172724426
ebi-a-GCST90001674	rs4317130	CD28- CD127- CD	3	173079422
ebi-a-GCST90001674	rs921157	CD28- CD127- CD	3	63178517
ebi-a-GCST90001674	rs115642223	CD28- CD127- CD	4	141162325
ebi-a-GCST90001674	rs114824462	CD28- CD127- CD	5	166507188
ebi-a-GCST90001674	rs75836793	CD28- CD127- CD	5	73501148
ebi-a-GCST90001674	rs111856210	CD28- CD127- CD	7	7026180
ebi-a-GCST90001674	rs1711693	CD28- CD127- CD	8	62932228
ebi-a-GCST90001674	rs10998956	CD28- CD127- CD	10	71525881
ebi-a-GCST90001674	rs75532811	CD28- CD127- CD	12	18964607
ebi-a-GCST90001674	rs7963204	CD28- CD127- CD	12	52559164
ebi-a-GCST90001674	rs149389411	CD28- CD127- CD	15	36411730
ebi-a-GCST90001674	rs12599120	CD28- CD127- CD	16	25477950
ebi-a-GCST90001674	rs2120691	CD28- CD127- CD	16	86753615
ebi-a-GCST90001674	rs6085043	CD28- CD127- CD	20	5222441
ebi-a-GCST90001674	rs6004167	CD28- CD127- CD	22	24892018
ebi-a-GCST90001675	rs12038742	CD28- CD127- CD	1	188622706
ebi-a-GCST90001675	rs35089045	CD28- CD127- CD	1	57910243
ebi-a-GCST90001675	rs3879969	CD28- CD127- CD	1	120551817
ebi-a-GCST90001675	rs62166861	CD28- CD127- CD	2	122691344
ebi-a-GCST90001675	rs72929459	CD28- CD127- CD	2	200181613
ebi-a-GCST90001675	rs115134707	CD28- CD127- CD	3	60344346
ebi-a-GCST90001675	rs1839051	CD28- CD127- CD	3	76705353
ebi-a-GCST90001675	rs13068352	CD28- CD127- CD	3	172724426
ebi-a-GCST90001675	rs138505212	CD28- CD127- CD	4	161872855

ebi-a-GCST90001675	rs115642223	CD28- CD127- CD	4	141162325
ebi-a-GCST90001675	rs6881158	CD28- CD127- CD	5	20984750
ebi-a-GCST90001675	rs17203563	CD28- CD127- CD	6	32446459
ebi-a-GCST90001675	rs10952014	CD28- CD127- CD	7	7026245
ebi-a-GCST90001675	rs77397960	CD28- CD127- CD	8	62998415
ebi-a-GCST90001675	rs76210385	CD28- CD127- CD	9	4728523
ebi-a-GCST90001675	rs4459318	CD28- CD127- CD	11	119541778
ebi-a-GCST90001675	rs7963204	CD28- CD127- CD	12	52559164
ebi-a-GCST90001675	rs57153567	CD28- CD127- CD	13	24865664
ebi-a-GCST90001675	rs1871360	CD28- CD127- CD	15	95276540
ebi-a-GCST90001675	rs66790175	CD28- CD127- CD	16	86074353
ebi-a-GCST90001675	rs12450916	CD28- CD127- CD	17	74615933
ebi-a-GCST90001675	rs79000044	CD28- CD127- CD	17	67264572
ebi-a-GCST90001675	rs62063923	CD28- CD127- CD	17	29871886
ebi-a-GCST90001675	rs9319779	CD28- CD127- CD	18	67151077
ebi-a-GCST90001675	rs10413329	CD28- CD127- CD	19	13114434
ebi-a-GCST90001675	rs117591947	CD28- CD127- CD	20	18052894
ebi-a-GCST90001675	rs6085043	CD28- CD127- CD	20	5222441
ebi-a-GCST90001675	rs75632179	CD28- CD127- CD	20	33611208
ebi-a-GCST90001675	rs79172657	CD28- CD127- CD	21	41072897
ebi-a-GCST90001675	rs6004167	CD28- CD127- CD	22	24892018
ebi-a-GCST90001676	rs3010090	CD28- CD25++ CI	1	202164837
ebi-a-GCST90001676	rs6682100	CD28- CD25++ CI	1	159130400
ebi-a-GCST90001676	rs2377708	CD28- CD25++ CI	1	217982930
ebi-a-GCST90001676	rs58227542	CD28- CD25++ CI	2	218638794
ebi-a-GCST90001676	rs62166861	CD28- CD25++ CI	2	122691344
ebi-a-GCST90001676	rs115583593	CD28- CD25++ CI	2	180234098
ebi-a-GCST90001676	rs6549955	CD28- CD25++ CI	3	29825146
ebi-a-GCST90001676	rs9839296	CD28- CD25++ CI	3	169747938
ebi-a-GCST90001677	rs3010090	CD28- CD25++ CI	1	202164837
ebi-a-GCST90001677	rs532011071	CD28- CD25++ CI	1	18829486
ebi-a-GCST90001677	rs12756299	CD28- CD25++ CI	1	7758760
ebi-a-GCST90001677	rs58227542	CD28- CD25++ CI	2	218638794
ebi-a-GCST90001677	rs9839296	CD28- CD25++ CI	3	169747938
ebi-a-GCST90001677	rs79297644	CD28- CD25++ CI	4	189525284
ebi-a-GCST90001677	rs27905	CD28- CD25++ CI	5	50162788
ebi-a-GCST90001677	rs115466519	CD28- CD25++ CI	5	96883867
ebi-a-GCST90001677	rs9380237	CD28- CD25++ CI	6	31264392
ebi-a-GCST90001677	rs542482176	CD28- CD25++ CI	6	63661784
ebi-a-GCST90001677	rs9269182	CD28- CD25++ CI	6	32447857
ebi-a-GCST90001677	rs220702	CD28- CD25++ CI	6	46867717
ebi-a-GCST90001677	rs2353082	CD28- CD25++ CI	7	72913168
ebi-a-GCST90001677	rs2283017	CD28- CD25++ CI	7	99574758
ebi-a-GCST90001677	rs75532811	CD28- CD25++ CI	12	18964607
ebi-a-GCST90001677	rs61623247	CD28- CD25++ CI	15	67237900
ebi-a-GCST90001677	rs145868945	CD28- CD25++ CI	16	54688102
ebi-a-GCST90001677	rs149335969	CD28- CD25++ CI	17	1604250
ebi-a-GCST90001677	rs72865317	CD28- CD25++ CI	17	69346419

ebi-a-GCST90001677	rs57752884	CD28- CD25++ CD	17	62472
ebi-a-GCST90001677	rs3919557	CD28- CD25++ CD	18	53419914
ebi-a-GCST90001677	rs61383018	CD28- CD25++ CD	18	66588419
ebi-a-GCST90001677	rs2109199	CD28- CD25++ CD	18	66051556
ebi-a-GCST90001677	rs10404710	CD28- CD25++ CD	19	8751986
ebi-a-GCST90001677	rs3848525	CD28- CD25++ CD	19	58836339
ebi-a-GCST90001677	rs75692821	CD28- CD25++ CD	21	19732234
ebi-a-GCST90001678	rs2377708	CD28- CD25++ CD	1	217982930
ebi-a-GCST90001678	rs116011991	CD28- CD25++ CD	1	181205200
ebi-a-GCST90001678	rs79660525	CD28- CD25++ CD	2	66305842
ebi-a-GCST90001678	rs114664351	CD28- CD25++ CD	2	148331978
ebi-a-GCST90001678	rs72929459	CD28- CD25++ CD	2	200181613
ebi-a-GCST90001678	rs62166861	CD28- CD25++ CD	2	122691344
ebi-a-GCST90001678	rs58227542	CD28- CD25++ CD	2	218638794
ebi-a-GCST90001678	rs78643468	CD28- CD25++ CD	3	62204684
ebi-a-GCST90001678	rs115134707	CD28- CD25++ CD	3	60344346
ebi-a-GCST90001678	rs1839051	CD28- CD25++ CD	3	76705353
ebi-a-GCST90001678	rs75596593	CD28- CD25++ CD	4	161897744
ebi-a-GCST90001678	rs220702	CD28- CD25++ CD	6	46867717
ebi-a-GCST90001678	rs9269109	CD28- CD25++ CD	6	32443266
ebi-a-GCST90001678	rs1634734	CD28- CD25++ CD	6	31307885
ebi-a-GCST90001678	rs78414417	CD28- CD25++ CD	7	137053814
ebi-a-GCST90001678	rs142133440	CD28- CD25++ CD	9	128841396
ebi-a-GCST90001678	rs74969013	CD28- CD25++ CD	10	112991635
ebi-a-GCST90001678	rs12430530	CD28- CD25++ CD	13	24865057
ebi-a-GCST90001678	rs45446506	CD28- CD25++ CD	14	67938855
ebi-a-GCST90001678	rs66790175	CD28- CD25++ CD	16	86074353
ebi-a-GCST90001678	rs62063923	CD28- CD25++ CD	17	29871886
ebi-a-GCST90001678	rs3848525	CD28- CD25++ CD	19	58836339
ebi-a-GCST90001678	rs10413329	CD28- CD25++ CD	19	13114434
ebi-a-GCST90001678	rs7262847	CD28- CD25++ CD	20	40528509
ebi-a-GCST90001678	rs79172657	CD28- CD25++ CD	21	41072897
ebi-a-GCST90001679	rs61768382	CD25++ CD8br %T	1	53873140
ebi-a-GCST90001679	rs10789181	CD25++ CD8br %T	1	65816567
ebi-a-GCST90001679	rs78667279	CD25++ CD8br %T	2	54957709
ebi-a-GCST90001679	rs60503607	CD25++ CD8br %T	2	53610249
ebi-a-GCST90001679	rs56262900	CD25++ CD8br %T	2	23897725
ebi-a-GCST90001679	rs139199585	CD25++ CD8br %T	3	189800760
ebi-a-GCST90001679	rs2875404	CD25++ CD8br %T	3	76674553
ebi-a-GCST90001679	rs111237279	CD25++ CD8br %T	4	184874118
ebi-a-GCST90001679	rs115540740	CD25++ CD8br %T	5	2863938
ebi-a-GCST90001679	rs111066413	CD25++ CD8br %T	8	142715815
ebi-a-GCST90001679	rs61839660	CD25++ CD8br %T	10	6094697
ebi-a-GCST90001679	rs4565815	CD25++ CD8br %T	10	94519697
ebi-a-GCST90001679	rs76836923	CD25++ CD8br %T	13	68739557
ebi-a-GCST90001679	rs35186946	CD25++ CD8br %T	15	37811809
ebi-a-GCST90001679	rs12930684	CD25++ CD8br %T	16	7317447
ebi-a-GCST90001679	rs35800283	CD25++ CD8br %T	18	62294527

ebi-a-GCST90001679	rs9304487	CD25++ CD8br %T	18	23581998
ebi-a-GCST90001679	rs73158142	CD25++ CD8br %T	22	37308470
ebi-a-GCST90001680	rs10789181	CD25++ CD8br %C	1	65816567
ebi-a-GCST90001680	rs72915529	CD25++ CD8br %C	2	54970478
ebi-a-GCST90001680	rs11935691	CD25++ CD8br %C	4	166715039
ebi-a-GCST90001680	rs114170505	CD25++ CD8br %C	6	32666487
ebi-a-GCST90001680	rs143728584	CD25++ CD8br %C	6	63027335
ebi-a-GCST90001680	rs117858455	CD25++ CD8br %C	7	70457913
ebi-a-GCST90001680	rs2511723	CD25++ CD8br %C	8	103689301
ebi-a-GCST90001680	rs34291145	CD25++ CD8br %C	8	112918556
ebi-a-GCST90001680	rs7871096	CD25++ CD8br %C	9	9463556
ebi-a-GCST90001680	rs2891140	CD25++ CD8br %C	9	20150132
ebi-a-GCST90001680	rs7899856	CD25++ CD8br %C	10	76992690
ebi-a-GCST90001680	rs61839660	CD25++ CD8br %C	10	6094697
ebi-a-GCST90001680	rs60120845	CD25++ CD8br %C	10	56952514
ebi-a-GCST90001680	rs12319878	CD25++ CD8br %C	12	132289989
ebi-a-GCST90001680	rs34505526	CD25++ CD8br %C	13	21956272
ebi-a-GCST90001680	rs76404623	CD25++ CD8br %C	14	66336556
ebi-a-GCST90001680	rs1840899	CD25++ CD8br %C	15	37803811
ebi-a-GCST90001680	rs1713484	CD25++ CD8br %C	16	12745851
ebi-a-GCST90001680	rs117023413	CD25++ CD8br %C	16	77750381
ebi-a-GCST90001680	rs12930684	CD25++ CD8br %C	16	7317447
ebi-a-GCST90001680	rs9304487	CD25++ CD8br %C	18	23581998
ebi-a-GCST90001680	rs62085785	CD25++ CD8br %C	18	2446762
ebi-a-GCST90001680	rs57406526	CD25++ CD8br %C	20	12603931
ebi-a-GCST90001681	rs285429	CD25++ CD8br AC	1	165421240
ebi-a-GCST90001681	rs12090749	CD25++ CD8br AC	1	204152575
ebi-a-GCST90001681	rs72915262	CD25++ CD8br AC	2	199736794
ebi-a-GCST90001681	rs186153147	CD25++ CD8br AC	2	233285448
ebi-a-GCST90001681	rs147417158	CD25++ CD8br AC	2	28165568
ebi-a-GCST90001681	rs78667279	CD25++ CD8br AC	2	54957709
ebi-a-GCST90001681	rs139199585	CD25++ CD8br AC	3	189800760
ebi-a-GCST90001681	rs2875404	CD25++ CD8br AC	3	76674553
ebi-a-GCST90001681	rs79068129	CD25++ CD8br AC	4	40455868
ebi-a-GCST90001681	rs115540740	CD25++ CD8br AC	5	2863938
ebi-a-GCST90001681	rs2381937	CD25++ CD8br AC	5	174189338
ebi-a-GCST90001681	rs3112458	CD25++ CD8br AC	5	170912245
ebi-a-GCST90001681	rs67795607	CD25++ CD8br AC	6	148558828
ebi-a-GCST90001681	rs113792557	CD25++ CD8br AC	7	63496409
ebi-a-GCST90001681	rs111066413	CD25++ CD8br AC	8	142715815
ebi-a-GCST90001681	rs12722558	CD25++ CD8br AC	10	6070276
ebi-a-GCST90001681	rs145966061	CD25++ CD8br AC	10	110696905
ebi-a-GCST90001681	rs79217088	CD25++ CD8br AC	10	56038091
ebi-a-GCST90001681	rs9523198	CD25++ CD8br AC	13	91588376
ebi-a-GCST90001681	rs76836923	CD25++ CD8br AC	13	68739557
ebi-a-GCST90001681	rs12873951	CD25++ CD8br AC	13	86136478
ebi-a-GCST90001681	rs61975285	CD25++ CD8br AC	14	82650928
ebi-a-GCST90001681	rs77859155	CD25++ CD8br AC	14	66658886

ebi-a-GCST90001681	rs117603761	CD25++ CD8br AC	16	77741894
ebi-a-GCST90001681	rs117391590	CD25++ CD8br AC	17	42759948
ebi-a-GCST90001681	rs6502728	CD25++ CD8br AC	17	3394299
ebi-a-GCST90001681	rs1403755	CD25++ CD8br AC	18	30263492
ebi-a-GCST90001681	rs67641209	CD25++ CD8br AC	18	1728457
ebi-a-GCST90001681	rs268887	CD25++ CD8br AC	19	51460482
ebi-a-GCST90001681	rs6004851	CD25++ CD8br AC	22	26346108
ebi-a-GCST90001682	rs10171072	CD127- CD8br %T	2	113756563
ebi-a-GCST90001682	rs147338725	CD127- CD8br %T	3	52230641
ebi-a-GCST90001682	rs13187738	CD127- CD8br %T	5	82289904
ebi-a-GCST90001682	rs77729615	CD127- CD8br %T	6	129871947
ebi-a-GCST90001682	rs144005685	CD127- CD8br %T	6	147443476
ebi-a-GCST90001682	rs915895	CD127- CD8br %T	6	32190217
ebi-a-GCST90001682	rs6904669	CD127- CD8br %T	6	31192796
ebi-a-GCST90001682	rs143747981	CD127- CD8br %T	7	52171917
ebi-a-GCST90001682	rs117509659	CD127- CD8br %T	7	29475802
ebi-a-GCST90001682	rs144285452	CD127- CD8br %T	8	8977165
ebi-a-GCST90001682	rs1031000	CD127- CD8br %T	13	104945684
ebi-a-GCST90001682	rs176187	CD127- CD8br %T	16	77078642
ebi-a-GCST90001682	rs17792777	CD127- CD8br %T	17	74656860
ebi-a-GCST90001682	rs4920093	CD127- CD8br %T	21	43304155
ebi-a-GCST90001682	rs143237141	CD127- CD8br %T	22	45176439
ebi-a-GCST90001683	rs147839296	CD127- CD8br %C	1	97114345
ebi-a-GCST90001683	rs114065521	CD127- CD8br %C	1	178434882
ebi-a-GCST90001683	rs114672498	CD127- CD8br %C	1	4591565
ebi-a-GCST90001683	rs13016937	CD127- CD8br %C	2	237036590
ebi-a-GCST90001683	rs10178517	CD127- CD8br %C	2	110049342
ebi-a-GCST90001683	rs12504845	CD127- CD8br %C	4	134361180
ebi-a-GCST90001683	rs669537	CD127- CD8br %C	5	134593542
ebi-a-GCST90001683	rs2636362	CD127- CD8br %C	6	168395245
ebi-a-GCST90001683	rs6462248	CD127- CD8br %C	7	31160869
ebi-a-GCST90001683	rs139584477	CD127- CD8br %C	8	57056704
ebi-a-GCST90001683	rs6485566	CD127- CD8br %C	11	44737646
ebi-a-GCST90001683	rs8063013	CD127- CD8br %C	16	72356899
ebi-a-GCST90001683	rs12450916	CD127- CD8br %C	17	74615933
ebi-a-GCST90001683	rs16943541	CD127- CD8br %C	18	376543
ebi-a-GCST90001684	rs12130486	CD127- CD8br AC	1	81455274
ebi-a-GCST90001684	rs7533594	CD127- CD8br AC	1	205558402
ebi-a-GCST90001684	rs305404	CD127- CD8br AC	1	88236589
ebi-a-GCST90001684	rs17204781	CD127- CD8br AC	3	151747048
ebi-a-GCST90001684	rs661093	CD127- CD8br AC	5	3971674
ebi-a-GCST90001684	rs6915999	CD127- CD8br AC	6	31377851
ebi-a-GCST90001684	rs6908236	CD127- CD8br AC	6	33628922
ebi-a-GCST90001684	rs3130685	CD127- CD8br AC	6	31206206
ebi-a-GCST90001684	rs77729615	CD127- CD8br AC	6	129871947
ebi-a-GCST90001684	rs72849269	CD127- CD8br AC	6	32468939
ebi-a-GCST90001684	rs199651	CD127- CD8br AC	7	23366691
ebi-a-GCST90001684	rs72721355	CD127- CD8br AC	8	140355393

ebi-a-GCST90001684	rs1183901	CD127- CD8br AC	10	90231718
ebi-a-GCST90001684	rs11060493	CD127- CD8br AC	12	130160924
ebi-a-GCST90001684	rs34970500	CD127- CD8br AC	13	110075194
ebi-a-GCST90001684	rs35238206	CD127- CD8br AC	17	75698346
ebi-a-GCST90001684	rs2837838	CD127- CD8br AC	21	42191862
ebi-a-GCST90001684	rs2267983	CD127- CD8br AC	22	50698157
ebi-a-GCST90001685	rs60998309	CD28- CD8br %T c	3	24959272
ebi-a-GCST90001685	rs7728865	CD28- CD8br %T c	5	59819941
ebi-a-GCST90001685	rs9269109	CD28- CD8br %T c	6	32443266
ebi-a-GCST90001685	rs7756993	CD28- CD8br %T c	6	31382133
ebi-a-GCST90001685	rs149359490	CD28- CD8br %T c	6	42968121
ebi-a-GCST90001685	rs144285452	CD28- CD8br %T c	8	8977165
ebi-a-GCST90001685	rs7989683	CD28- CD8br %T c	13	96304050
ebi-a-GCST90001685	rs17757383	CD28- CD8br %T c	14	96102338
ebi-a-GCST90001685	rs74829844	CD28- CD8br %T c	16	49700293
ebi-a-GCST90001685	rs76108828	CD28- CD8br %T c	17	19407306
ebi-a-GCST90001685	rs4796089	CD28- CD8br %T c	17	33814758
ebi-a-GCST90001685	rs12940345	CD28- CD8br %T c	17	74612462
ebi-a-GCST90001685	rs8108431	CD28- CD8br %T c	19	9996687
ebi-a-GCST90001685	rs148460149	CD28- CD8br %T c	20	50115542
ebi-a-GCST90001685	rs56216385	CD28- CD8br %T c	21	16129270
ebi-a-GCST90001685	rs6009141	CD28- CD8br %T c	22	49445896
ebi-a-GCST90001686	rs193125329	CD28- CD8br %CD	3	172046675
ebi-a-GCST90001686	rs6781800	CD28- CD8br %CD	3	187595182
ebi-a-GCST90001686	rs669537	CD28- CD8br %CD	5	134593542
ebi-a-GCST90001686	rs2197002	CD28- CD8br %CD	5	78136988
ebi-a-GCST90001686	rs7728865	CD28- CD8br %CD	5	59819941
ebi-a-GCST90001686	rs149359490	CD28- CD8br %CD	6	42968121
ebi-a-GCST90001686	rs6904670	CD28- CD8br %CD	6	31192797
ebi-a-GCST90001686	rs57658648	CD28- CD8br %CD	6	32520731
ebi-a-GCST90001686	rs2275854	CD28- CD8br %CD	6	29976838
ebi-a-GCST90001686	rs7775227	CD28- CD8br %CD	6	31382147
ebi-a-GCST90001686	rs11544989	CD28- CD8br %CD	8	145057868
ebi-a-GCST90001686	rs62490686	CD28- CD8br %CD	8	37669867
ebi-a-GCST90001686	rs185397210	CD28- CD8br %CD	9	12807800
ebi-a-GCST90001686	rs34752208	CD28- CD8br %CD	9	137165566
ebi-a-GCST90001686	rs62530389	CD28- CD8br %CD	9	1143504
ebi-a-GCST90001686	rs78240333	CD28- CD8br %CD	11	122733658
ebi-a-GCST90001686	rs142216336	CD28- CD8br %CD	12	59178989
ebi-a-GCST90001686	rs57679712	CD28- CD8br %CD	12	14117043
ebi-a-GCST90001686	rs7989683	CD28- CD8br %CD	13	96304050
ebi-a-GCST90001686	rs139331134	CD28- CD8br %CD	14	20442676
ebi-a-GCST90001686	rs117279064	CD28- CD8br %CD	16	14352543
ebi-a-GCST90001686	rs12450916	CD28- CD8br %CD	17	74615933
ebi-a-GCST90001686	rs4796089	CD28- CD8br %CD	17	33814758
ebi-a-GCST90001686	rs1978254	CD28- CD8br %CD	19	57281224
ebi-a-GCST90001686	rs148460149	CD28- CD8br %CD	20	50115542
ebi-a-GCST90001686	rs56216385	CD28- CD8br %CD	21	16129270

ebi-a-GCST90001687	rs7533594	CD28- CD8br AC	1	205558402
ebi-a-GCST90001687	rs62280061	CD28- CD8br AC	3	187544579
ebi-a-GCST90001687	rs60998309	CD28- CD8br AC	3	24959272
ebi-a-GCST90001687	rs1451433	CD28- CD8br AC	4	179934673
ebi-a-GCST90001687	rs36058358	CD28- CD8br AC	6	32636282
ebi-a-GCST90001687	rs77729615	CD28- CD8br AC	6	129871947
ebi-a-GCST90001687	rs3130685	CD28- CD8br AC	6	31206206
ebi-a-GCST90001687	rs1266079	CD28- CD8br AC	6	31504774
ebi-a-GCST90001687	rs113098117	CD28- CD8br AC	7	54014110
ebi-a-GCST90001687	rs118079184	CD28- CD8br AC	8	9002188
ebi-a-GCST90001687	rs7989683	CD28- CD8br AC	13	96304050
ebi-a-GCST90001687	rs12940345	CD28- CD8br AC	17	74612462
ebi-a-GCST90001687	rs4796089	CD28- CD8br AC	17	33814758
ebi-a-GCST90001687	rs148460149	CD28- CD8br AC	20	50115542
ebi-a-GCST90001687	rs56216385	CD28- CD8br AC	21	16129270
ebi-a-GCST90001687	rs427055	CD28- CD8br AC	21	15326213
ebi-a-GCST90001687	rs2267983	CD28- CD8br AC	22	50698157
ebi-a-GCST90001688	rs113116201	CD28+ CD45RA+ (	1	198830942
ebi-a-GCST90001688	rs78743764	CD28+ CD45RA+ (	1	3301962
ebi-a-GCST90001688	rs145208064	CD28+ CD45RA+ (	1	187976146
ebi-a-GCST90001688	rs12755474	CD28+ CD45RA+ (	1	216352525
ebi-a-GCST90001688	rs141357353	CD28+ CD45RA+ (	1	58442574
ebi-a-GCST90001688	rs144593458	CD28+ CD45RA+ (	1	159355851
ebi-a-GCST90001688	rs142530179	CD28+ CD45RA+ (	1	62672889
ebi-a-GCST90001688	rs149023182	CD28+ CD45RA+ (	1	232754676
ebi-a-GCST90001688	rs77458346	CD28+ CD45RA+ (	1	247120912
ebi-a-GCST90001688	rs74926697	CD28+ CD45RA+ (	2	653215
ebi-a-GCST90001688	rs7567302	CD28+ CD45RA+ (	2	237035915
ebi-a-GCST90001688	rs111275136	CD28+ CD45RA+ (	2	52593468
ebi-a-GCST90001688	rs79112776	CD28+ CD45RA+ (	2	56230278
ebi-a-GCST90001688	rs113046419	CD28+ CD45RA+ (	2	66598636
ebi-a-GCST90001688	rs140064908	CD28+ CD45RA+ (	2	141593935
ebi-a-GCST90001688	rs7583259	CD28+ CD45RA+ (	2	38921934
ebi-a-GCST90001688	rs10183338	CD28+ CD45RA+ (	2	111610816
ebi-a-GCST90001688	rs72968960	CD28+ CD45RA+ (	2	128497022
ebi-a-GCST90001688	rs146985291	CD28+ CD45RA+ (	3	170169608
ebi-a-GCST90001688	rs116131582	CD28+ CD45RA+ (	3	177291240
ebi-a-GCST90001688	rs144547268	CD28+ CD45RA+ (	3	79405328
ebi-a-GCST90001688	rs114795342	CD28+ CD45RA+ (	3	24791112
ebi-a-GCST90001688	rs74577787	CD28+ CD45RA+ (	3	84799491
ebi-a-GCST90001688	rs13115676	CD28+ CD45RA+ (	4	139253983
ebi-a-GCST90001688	rs76604940	CD28+ CD45RA+ (	4	31397782
ebi-a-GCST90001688	rs6833962	CD28+ CD45RA+ (	4	103558731
ebi-a-GCST90001688	rs11735841	CD28+ CD45RA+ (	4	25612506
ebi-a-GCST90001688	rs115218435	CD28+ CD45RA+ (	4	67836131
ebi-a-GCST90001688	rs137858581	CD28+ CD45RA+ (	5	173002007
ebi-a-GCST90001688	rs117403703	CD28+ CD45RA+ (	6	93203850
ebi-a-GCST90001688	rs116562707	CD28+ CD45RA+ (	6	31360602

ebi-a-GCST90001688	rs2075682	CD28+ CD45RA+ (	6	29691097
ebi-a-GCST90001688	rs141878021	CD28+ CD45RA+ (	6	319617
ebi-a-GCST90001688	rs117475733	CD28+ CD45RA+ (	6	148775706
ebi-a-GCST90001688	rs73404248	CD28+ CD45RA+ (	6	31410352
ebi-a-GCST90001688	rs144788631	CD28+ CD45RA+ (	6	145827461
ebi-a-GCST90001688	rs112637846	CD28+ CD45RA+ (	6	149114553
ebi-a-GCST90001688	rs141596789	CD28+ CD45RA+ (	7	28687227
ebi-a-GCST90001688	rs184082746	CD28+ CD45RA+ (	7	92791863
ebi-a-GCST90001688	rs184134422	CD28+ CD45RA+ (	7	109990427
ebi-a-GCST90001688	rs182236332	CD28+ CD45RA+ (	7	124954943
ebi-a-GCST90001688	rs62477133	CD28+ CD45RA+ (	7	118626871
ebi-a-GCST90001688	rs112571466	CD28+ CD45RA+ (	7	111704231
ebi-a-GCST90001688	rs13243221	CD28+ CD45RA+ (	7	80880540
ebi-a-GCST90001688	rs111503027	CD28+ CD45RA+ (	8	59820131
ebi-a-GCST90001688	rs8180936	CD28+ CD45RA+ (	8	61151574
ebi-a-GCST90001688	rs143498795	CD28+ CD45RA+ (	8	140342260
ebi-a-GCST90001688	rs112779860	CD28+ CD45RA+ (	8	127602530
ebi-a-GCST90001688	rs118127261	CD28+ CD45RA+ (	8	39979373
ebi-a-GCST90001688	rs142967914	CD28+ CD45RA+ (	9	12800264
ebi-a-GCST90001688	rs192475369	CD28+ CD45RA+ (	9	33552507
ebi-a-GCST90001688	rs76325389	CD28+ CD45RA+ (	9	122676137
ebi-a-GCST90001688	rs73648165	CD28+ CD45RA+ (	9	36889554
ebi-a-GCST90001688	rs113447229	CD28+ CD45RA+ (	9	105346840
ebi-a-GCST90001688	rs12555210	CD28+ CD45RA+ (	9	33428882
ebi-a-GCST90001688	rs80194890	CD28+ CD45RA+ (	10	91332621
ebi-a-GCST90001688	rs35890579	CD28+ CD45RA+ (	10	129690837
ebi-a-GCST90001688	rs74723190	CD28+ CD45RA+ (	10	96183831
ebi-a-GCST90001688	rs12258605	CD28+ CD45RA+ (	10	125342204
ebi-a-GCST90001688	rs189801254	CD28+ CD45RA+ (	10	68336237
ebi-a-GCST90001688	rs141341926	CD28+ CD45RA+ (	11	119643891
ebi-a-GCST90001688	rs138653244	CD28+ CD45RA+ (	11	47745696
ebi-a-GCST90001688	rs111346930	CD28+ CD45RA+ (	11	91003050
ebi-a-GCST90001688	rs56125740	CD28+ CD45RA+ (	11	134093512
ebi-a-GCST90001688	rs117927481	CD28+ CD45RA+ (	12	49315200
ebi-a-GCST90001688	rs112258643	CD28+ CD45RA+ (	12	55672838
ebi-a-GCST90001688	rs77780671	CD28+ CD45RA+ (	12	129183573
ebi-a-GCST90001688	rs9592479	CD28+ CD45RA+ (	13	67157607
ebi-a-GCST90001688	rs58471414	CD28+ CD45RA+ (	14	28349764
ebi-a-GCST90001688	rs139463423	CD28+ CD45RA+ (	14	31143832
ebi-a-GCST90001688	rs117494082	CD28+ CD45RA+ (	14	56862154
ebi-a-GCST90001688	rs77670766	CD28+ CD45RA+ (	15	94589979
ebi-a-GCST90001688	rs17685199	CD28+ CD45RA+ (	15	36405909
ebi-a-GCST90001688	rs28460753	CD28+ CD45RA+ (	15	62991760
ebi-a-GCST90001688	rs138736730	CD28+ CD45RA+ (	15	87138558
ebi-a-GCST90001688	rs112731098	CD28+ CD45RA+ (	16	50350935
ebi-a-GCST90001688	rs179609	CD28+ CD45RA+ (	16	13818107
ebi-a-GCST90001688	rs111283043	CD28+ CD45RA+ (	16	79205197
ebi-a-GCST90001688	rs144591335	CD28+ CD45RA+ (	16	59010741

ebi-a-GCST90001688	rs149948027	CD28+ CD45RA+ (	16	89718638
ebi-a-GCST90001688	rs147066619	CD28+ CD45RA+ (	16	65488378
ebi-a-GCST90001688	rs117795441	CD28+ CD45RA+ (	17	54735696
ebi-a-GCST90001688	rs8067835	CD28+ CD45RA+ (	17	31363767
ebi-a-GCST90001688	rs11652705	CD28+ CD45RA+ (	17	41761741
ebi-a-GCST90001688	rs77935824	CD28+ CD45RA+ (	18	68968361
ebi-a-GCST90001688	rs56095154	CD28+ CD45RA+ (	18	50159833
ebi-a-GCST90001688	rs60455861	CD28+ CD45RA+ (	19	53953072
ebi-a-GCST90001688	rs142208889	CD28+ CD45RA+ (	19	51829114
ebi-a-GCST90001688	rs145827119	CD28+ CD45RA+ (	20	57132122
ebi-a-GCST90001688	rs149926070	CD28+ CD45RA+ (	21	41143748
ebi-a-GCST90001688	rs116954000	CD28+ CD45RA+ (	22	40298491
ebi-a-GCST90001689	rs17583875	CD28+ CD45RA+ (	1	197924770
ebi-a-GCST90001689	rs60220946	CD28+ CD45RA+ (	1	193505625
ebi-a-GCST90001689	rs2629824	CD28+ CD45RA+ (	1	114687316
ebi-a-GCST90001689	rs2802930	CD28+ CD45RA+ (	1	235096612
ebi-a-GCST90001689	rs76668354	CD28+ CD45RA+ (	2	103587624
ebi-a-GCST90001689	rs1064086	CD28+ CD45RA+ (	2	237034061
ebi-a-GCST90001689	rs146741472	CD28+ CD45RA+ (	2	120215812
ebi-a-GCST90001689	rs11896593	CD28+ CD45RA+ (	2	38925155
ebi-a-GCST90001689	rs76313555	CD28+ CD45RA+ (	3	59914042
ebi-a-GCST90001689	rs146985291	CD28+ CD45RA+ (	3	170169608
ebi-a-GCST90001689	rs10025910	CD28+ CD45RA+ (	4	25610903
ebi-a-GCST90001689	rs149655274	CD28+ CD45RA+ (	4	154029285
ebi-a-GCST90001689	rs79638560	CD28+ CD45RA+ (	4	183402200
ebi-a-GCST90001689	rs72723766	CD28+ CD45RA+ (	4	152131193
ebi-a-GCST90001689	rs117475733	CD28+ CD45RA+ (	6	148775706
ebi-a-GCST90001689	rs114559146	CD28+ CD45RA+ (	6	31395062
ebi-a-GCST90001689	rs9260902	CD28+ CD45RA+ (	6	29947740
ebi-a-GCST90001689	rs117403703	CD28+ CD45RA+ (	6	93203850
ebi-a-GCST90001689	rs9792050	CD28+ CD45RA+ (	7	31164655
ebi-a-GCST90001689	rs10242630	CD28+ CD45RA+ (	7	146469943
ebi-a-GCST90001689	rs182236332	CD28+ CD45RA+ (	7	124954943
ebi-a-GCST90001689	rs184082746	CD28+ CD45RA+ (	7	92791863
ebi-a-GCST90001689	rs73061672	CD28+ CD45RA+ (	7	8326430
ebi-a-GCST90001689	rs141596789	CD28+ CD45RA+ (	7	28687227
ebi-a-GCST90001689	rs34752208	CD28+ CD45RA+ (	9	137165566
ebi-a-GCST90001689	rs7033084	CD28+ CD45RA+ (	9	16904641
ebi-a-GCST90001689	rs62572596	CD28+ CD45RA+ (	9	114588604
ebi-a-GCST90001689	rs76014368	CD28+ CD45RA+ (	12	123513519
ebi-a-GCST90001689	rs10840675	CD28+ CD45RA+ (	12	17367587
ebi-a-GCST90001689	rs112258643	CD28+ CD45RA+ (	12	55672838
ebi-a-GCST90001689	rs9577271	CD28+ CD45RA+ (	13	114096507
ebi-a-GCST90001689	rs28576084	CD28+ CD45RA+ (	14	82062494
ebi-a-GCST90001689	rs1879612	CD28+ CD45RA+ (	15	99398708
ebi-a-GCST90001689	rs28460753	CD28+ CD45RA+ (	15	62991760
ebi-a-GCST90001689	rs143788880	CD28+ CD45RA+ (	17	57361218
ebi-a-GCST90001689	rs139580802	CD28+ CD45RA+ (	17	76213606

ebi-a-GCST90001689	rs5011724	CD28+ CD45RA+ (	17	41759061
ebi-a-GCST90001689	rs1978254	CD28+ CD45RA+ (	19	57281224
ebi-a-GCST90001689	rs145827119	CD28+ CD45RA+ (	20	57132122
ebi-a-GCST90001690	rs2629830	CD28+ CD45RA+ (	1	114684259
ebi-a-GCST90001690	rs142530179	CD28+ CD45RA+ (	1	62672889
ebi-a-GCST90001690	rs149023182	CD28+ CD45RA+ (	1	232754676
ebi-a-GCST90001690	rs4849134	CD28+ CD45RA+ (	2	111615015
ebi-a-GCST90001690	rs73926977	CD28+ CD45RA+ (	2	38260823
ebi-a-GCST90001690	rs78908164	CD28+ CD45RA+ (	2	127884901
ebi-a-GCST90001690	rs10178070	CD28+ CD45RA+ (	2	18727639
ebi-a-GCST90001690	rs11896593	CD28+ CD45RA+ (	2	38925155
ebi-a-GCST90001690	rs62189428	CD28+ CD45RA+ (	2	237016087
ebi-a-GCST90001690	rs111275136	CD28+ CD45RA+ (	2	52593468
ebi-a-GCST90001690	rs79777035	CD28+ CD45RA+ (	3	25717920
ebi-a-GCST90001690	rs62248512	CD28+ CD45RA+ (	3	26549717
ebi-a-GCST90001690	rs146985291	CD28+ CD45RA+ (	3	170169608
ebi-a-GCST90001690	rs142617762	CD28+ CD45RA+ (	4	609857
ebi-a-GCST90001690	rs11735841	CD28+ CD45RA+ (	4	25612506
ebi-a-GCST90001690	rs115574093	CD28+ CD45RA+ (	4	67743517
ebi-a-GCST90001690	rs73127385	CD28+ CD45RA+ (	4	31396800
ebi-a-GCST90001690	rs7707297	CD28+ CD45RA+ (	5	83559348
ebi-a-GCST90001690	rs148593283	CD28+ CD45RA+ (	5	42120894
ebi-a-GCST90001690	rs2853995	CD28+ CD45RA+ (	6	31333019
ebi-a-GCST90001690	rs1974459	CD28+ CD45RA+ (	6	32452142
ebi-a-GCST90001690	rs117403703	CD28+ CD45RA+ (	6	93203850
ebi-a-GCST90001690	rs3131627	CD28+ CD45RA+ (	6	31415065
ebi-a-GCST90001690	rs117475733	CD28+ CD45RA+ (	6	148775706
ebi-a-GCST90001690	rs72826622	CD28+ CD45RA+ (	6	20193704
ebi-a-GCST90001690	rs184082746	CD28+ CD45RA+ (	7	92791863
ebi-a-GCST90001690	rs182236332	CD28+ CD45RA+ (	7	124954943
ebi-a-GCST90001690	rs74968616	CD28+ CD45RA+ (	7	44125650
ebi-a-GCST90001690	rs113447229	CD28+ CD45RA+ (	9	105346840
ebi-a-GCST90001690	rs76325389	CD28+ CD45RA+ (	9	122676137
ebi-a-GCST90001690	rs142967914	CD28+ CD45RA+ (	9	12800264
ebi-a-GCST90001690	rs2987632	CD28+ CD45RA+ (	9	138307613
ebi-a-GCST90001690	rs192475369	CD28+ CD45RA+ (	9	33552507
ebi-a-GCST90001690	rs189801254	CD28+ CD45RA+ (	10	68336237
ebi-a-GCST90001690	rs80194890	CD28+ CD45RA+ (	10	91332621
ebi-a-GCST90001690	rs12575977	CD28+ CD45RA+ (	11	126507219
ebi-a-GCST90001690	rs55645768	CD28+ CD45RA+ (	11	79284506
ebi-a-GCST90001690	rs79293430	CD28+ CD45RA+ (	12	71950863
ebi-a-GCST90001690	rs117927481	CD28+ CD45RA+ (	12	49315200
ebi-a-GCST90001690	rs75723492	CD28+ CD45RA+ (	13	84241776
ebi-a-GCST90001690	rs9592479	CD28+ CD45RA+ (	13	67157607
ebi-a-GCST90001690	rs113911480	CD28+ CD45RA+ (	14	29113167
ebi-a-GCST90001690	rs58471414	CD28+ CD45RA+ (	14	28349764
ebi-a-GCST90001690	rs12901644	CD28+ CD45RA+ (	15	36456429
ebi-a-GCST90001690	rs144591335	CD28+ CD45RA+ (	16	59010741

ebi-a-GCST90001690	rs75985606	CD28+ CD45RA+ (	16	56102938
ebi-a-GCST90001690	rs111283043	CD28+ CD45RA+ (	16	79205197
ebi-a-GCST90001690	rs6503467	CD28+ CD45RA+ (	17	41767457
ebi-a-GCST90001690	rs2860255	CD28+ CD45RA+ (	18	27836611
ebi-a-GCST90001690	rs79934486	CD28+ CD45RA+ (	18	69051316
ebi-a-GCST90001690	rs302838	CD28+ CD45RA+ (	19	56414856
ebi-a-GCST90001690	rs60455861	CD28+ CD45RA+ (	19	53953072
ebi-a-GCST90001690	rs142208889	CD28+ CD45RA+ (	19	51829114
ebi-a-GCST90001690	rs145827119	CD28+ CD45RA+ (	20	57132122
ebi-a-GCST90001690	rs62229789	CD28+ CD45RA+ (	20	50100758
ebi-a-GCST90001690	rs149926070	CD28+ CD45RA+ (	21	41143748
ebi-a-GCST90001691	rs147071297	CD28+ CD45RA- (	1	191648600
ebi-a-GCST90001691	rs7530076	CD28+ CD45RA- (	1	195825276
ebi-a-GCST90001691	rs116794175	CD28+ CD45RA- (	1	194010208
ebi-a-GCST90001691	rs17583875	CD28+ CD45RA- (	1	197924770
ebi-a-GCST90001691	rs3117751	CD28+ CD45RA- (	1	177745454
ebi-a-GCST90001691	rs34645541	CD28+ CD45RA- (	1	195875605
ebi-a-GCST90001691	rs60220946	CD28+ CD45RA- (	1	193505625
ebi-a-GCST90001691	rs76557351	CD28+ CD45RA- (	2	46479929
ebi-a-GCST90001691	rs147285635	CD28+ CD45RA- (	3	84498712
ebi-a-GCST90001691	rs71621309	CD28+ CD45RA- (	3	66439115
ebi-a-GCST90001691	rs1450147	CD28+ CD45RA- (	4	183111829
ebi-a-GCST90001691	rs114547596	CD28+ CD45RA- (	4	97974344
ebi-a-GCST90001691	rs16996915	CD28+ CD45RA- (	4	78710538
ebi-a-GCST90001691	rs17318450	CD28+ CD45RA- (	5	35482314
ebi-a-GCST90001691	rs34933238	CD28+ CD45RA- (	5	23346191
ebi-a-GCST90001691	rs4425613	CD28+ CD45RA- (	6	130374792
ebi-a-GCST90001691	rs113888828	CD28+ CD45RA- (	6	149157641
ebi-a-GCST90001691	rs9378248	CD28+ CD45RA- (	6	31326289
ebi-a-GCST90001691	rs59052071	CD28+ CD45RA- (	6	32586307
ebi-a-GCST90001691	rs1722056	CD28+ CD45RA- (	7	109795429
ebi-a-GCST90001691	rs144061457	CD28+ CD45RA- (	9	108909972
ebi-a-GCST90001691	rs7069505	CD28+ CD45RA- (	10	956400
ebi-a-GCST90001691	rs74331648	CD28+ CD45RA- (	11	91720965
ebi-a-GCST90001691	rs180683226	CD28+ CD45RA- (	12	47277365
ebi-a-GCST90001691	rs145394193	CD28+ CD45RA- (	18	2967168
ebi-a-GCST90001691	rs1284408	CD28+ CD45RA- (	18	13548430
ebi-a-GCST90001691	rs9608041	CD28+ CD45RA- (	22	20851424
ebi-a-GCST90001692	rs146620586	CD28+ CD45RA- (	1	198524264
ebi-a-GCST90001692	rs60220946	CD28+ CD45RA- (	1	193505625
ebi-a-GCST90001692	rs11579717	CD28+ CD45RA- (	1	199999121
ebi-a-GCST90001692	rs138915779	CD28+ CD45RA- (	1	190079170
ebi-a-GCST90001692	rs76557351	CD28+ CD45RA- (	2	46479929
ebi-a-GCST90001692	rs144716339	CD28+ CD45RA- (	3	131340841
ebi-a-GCST90001692	rs147285635	CD28+ CD45RA- (	3	84498712
ebi-a-GCST90001692	rs114547596	CD28+ CD45RA- (	4	97974344
ebi-a-GCST90001692	rs6812204	CD28+ CD45RA- (	4	181208931
ebi-a-GCST90001692	rs138969403	CD28+ CD45RA- (	4	88757559

ebi-a-GCST90001692	rs12498745	CD28+ CD45RA- (	4	99216768
ebi-a-GCST90001692	rs4368632	CD28+ CD45RA- (	4	78946913
ebi-a-GCST90001692	rs28800474	CD28+ CD45RA- (	4	116872471
ebi-a-GCST90001692	rs17230697	CD28+ CD45RA- (	4	55480792
ebi-a-GCST90001692	rs9327711	CD28+ CD45RA- (	5	134576018
ebi-a-GCST90001692	rs62359129	CD28+ CD45RA- (	5	39623055
ebi-a-GCST90001692	rs1051488	CD28+ CD45RA- (	6	31322911
ebi-a-GCST90001692	rs9271513	CD28+ CD45RA- (	6	32589523
ebi-a-GCST90001692	rs111328168	CD28+ CD45RA- (	6	149103548
ebi-a-GCST90001692	rs12663276	CD28+ CD45RA- (	6	29906010
ebi-a-GCST90001692	rs77336134	CD28+ CD45RA- (	7	22504488
ebi-a-GCST90001692	rs3896278	CD28+ CD45RA- (	7	134137705
ebi-a-GCST90001692	rs7863844	CD28+ CD45RA- (	9	38410242
ebi-a-GCST90001692	rs7948625	CD28+ CD45RA- (	11	43997280
ebi-a-GCST90001692	rs1558728	CD28+ CD45RA- (	12	97559603
ebi-a-GCST90001692	rs12317609	CD28+ CD45RA- (	12	23030395
ebi-a-GCST90001692	rs79222153	CD28+ CD45RA- (	12	43252206
ebi-a-GCST90001692	rs2892496	CD28+ CD45RA- (	13	30488990
ebi-a-GCST90001692	rs11629948	CD28+ CD45RA- (	15	65376824
ebi-a-GCST90001692	rs8051285	CD28+ CD45RA- (	16	7862725
ebi-a-GCST90001692	rs1150437	CD28+ CD45RA- (	20	59223621
ebi-a-GCST90001692	rs79865452	CD28+ CD45RA- (	22	50795620
ebi-a-GCST90001693	rs12124698	CD28+ CD45RA- (	1	246883687
ebi-a-GCST90001693	rs11579717	CD28+ CD45RA- (	1	199999121
ebi-a-GCST90001693	rs6693121	CD28+ CD45RA- (	1	101744633
ebi-a-GCST90001693	rs138915779	CD28+ CD45RA- (	1	190079170
ebi-a-GCST90001693	rs541367304	CD28+ CD45RA- (	1	198283058
ebi-a-GCST90001693	rs60220946	CD28+ CD45RA- (	1	193505625
ebi-a-GCST90001693	rs186153147	CD28+ CD45RA- (	2	233285448
ebi-a-GCST90001693	rs2670003	CD28+ CD45RA- (	3	9087967
ebi-a-GCST90001693	rs114547596	CD28+ CD45RA- (	4	97974344
ebi-a-GCST90001693	rs76560336	CD28+ CD45RA- (	4	33455497
ebi-a-GCST90001693	rs35858834	CD28+ CD45RA- (	5	23325233
ebi-a-GCST90001693	rs145673143	CD28+ CD45RA- (	5	180271484
ebi-a-GCST90001693	rs7808299	CD28+ CD45RA- (	7	83422079
ebi-a-GCST90001693	rs1722056	CD28+ CD45RA- (	7	109795429
ebi-a-GCST90001693	rs1322060	CD28+ CD45RA- (	9	117697660
ebi-a-GCST90001693	rs7863863	CD28+ CD45RA- (	9	3374943
ebi-a-GCST90001693	rs12778529	CD28+ CD45RA- (	10	774793
ebi-a-GCST90001693	rs11595826	CD28+ CD45RA- (	10	111696639
ebi-a-GCST90001693	rs117198249	CD28+ CD45RA- (	10	28939784
ebi-a-GCST90001693	rs2488271	CD28+ CD45RA- (	10	90228162
ebi-a-GCST90001693	rs116884829	CD28+ CD45RA- (	11	77173517
ebi-a-GCST90001693	rs67077312	CD28+ CD45RA- (	13	86215727
ebi-a-GCST90001693	rs4644866	CD28+ CD45RA- (	16	25867450
ebi-a-GCST90001693	rs9924010	CD28+ CD45RA- (	16	7398995
ebi-a-GCST90001693	rs2160305	CD28+ CD45RA- (	16	54006217
ebi-a-GCST90001693	rs117391590	CD28+ CD45RA- (	17	42759948

ebi-a-GCST90001693	rs34317887	CD28+ CD45RA- (	18	74998706
ebi-a-GCST90001693	rs7241473	CD28+ CD45RA- (	18	13547966
ebi-a-GCST90001693	rs188418491	CD28+ CD45RA- (	19	777304
ebi-a-GCST90001694	rs3821224	CD28- DN (CD4-C	2	61404905
ebi-a-GCST90001694	rs115953192	CD28- DN (CD4-C	2	45208257
ebi-a-GCST90001694	rs6755452	CD28- DN (CD4-C	2	87035076
ebi-a-GCST90001694	rs76549873	CD28- DN (CD4-C	2	75722749
ebi-a-GCST90001694	rs75941798	CD28- DN (CD4-C	3	118074543
ebi-a-GCST90001694	rs144346829	CD28- DN (CD4-C	4	96715064
ebi-a-GCST90001694	rs66989520	CD28- DN (CD4-C	4	147357834
ebi-a-GCST90001694	rs1580183	CD28- DN (CD4-C	5	83043360
ebi-a-GCST90001694	rs139249541	CD28- DN (CD4-C	6	102294761
ebi-a-GCST90001694	rs79086781	CD28- DN (CD4-C	6	100526652
ebi-a-GCST90001694	rs74593262	CD28- DN (CD4-C	6	15826226
ebi-a-GCST90001694	rs9488834	CD28- DN (CD4-C	6	97889936
ebi-a-GCST90001694	rs6907169	CD28- DN (CD4-C	6	100905226
ebi-a-GCST90001694	rs2511751	CD28- DN (CD4-C	8	103602649
ebi-a-GCST90001694	rs958219	CD28- DN (CD4-C	9	21784225
ebi-a-GCST90001694	rs73560923	CD28- DN (CD4-C	9	138405168
ebi-a-GCST90001694	rs1875168	CD28- DN (CD4-C	10	2967977
ebi-a-GCST90001694	rs36037322	CD28- DN (CD4-C	10	96943194
ebi-a-GCST90001694	rs117477265	CD28- DN (CD4-C	10	115625047
ebi-a-GCST90001694	rs78936881	CD28- DN (CD4-C	11	16325844
ebi-a-GCST90001694	rs140444563	CD28- DN (CD4-C	12	31103286
ebi-a-GCST90001694	rs142716558	CD28- DN (CD4-C	14	51308539
ebi-a-GCST90001694	rs8077688	CD28- DN (CD4-C	17	33280360
ebi-a-GCST90001694	rs140247402	CD28- DN (CD4-C	17	1235562
ebi-a-GCST90001694	rs9896155	CD28- DN (CD4-C	17	33794697
ebi-a-GCST90001694	rs8113259	CD28- DN (CD4-C	19	21693075
ebi-a-GCST90001694	rs9978065	CD28- DN (CD4-C	21	15247433
ebi-a-GCST90001695	rs76732756	CD45RA- CD28- C	1	221000197
ebi-a-GCST90001695	rs78656354	CD45RA- CD28- C	1	166905650
ebi-a-GCST90001695	rs76595423	CD45RA- CD28- C	1	102745011
ebi-a-GCST90001695	rs182784388	CD45RA- CD28- C	1	18635367
ebi-a-GCST90001695	rs111978771	CD45RA- CD28- C	1	21025910
ebi-a-GCST90001695	rs76119911	CD45RA- CD28- C	1	247712613
ebi-a-GCST90001695	rs148620542	CD45RA- CD28- C	1	178035728
ebi-a-GCST90001695	rs78134440	CD45RA- CD28- C	1	215060336
ebi-a-GCST90001695	rs142707561	CD45RA- CD28- C	1	42381260
ebi-a-GCST90001695	rs140963286	CD45RA- CD28- C	1	24640467
ebi-a-GCST90001695	rs74827547	CD45RA- CD28- C	1	54853809
ebi-a-GCST90001695	rs140677128	CD45RA- CD28- C	1	229822729
ebi-a-GCST90001695	rs144571871	CD45RA- CD28- C	1	65133062
ebi-a-GCST90001695	rs61772921	CD45RA- CD28- C	1	80897562
ebi-a-GCST90001695	rs145224093	CD45RA- CD28- C	1	116035874
ebi-a-GCST90001695	rs117708200	CD45RA- CD28- C	1	121273212
ebi-a-GCST90001695	rs74637079	CD45RA- CD28- C	1	74493410
ebi-a-GCST90001695	rs149981836	CD45RA- CD28- C	1	110677725

ebi-a-GCST90001695	rs55881385	CD45RA- CD28- C	1	34724192
ebi-a-GCST90001695	rs139889394	CD45RA- CD28- C	1	41963906
ebi-a-GCST90001695	rs115486816	CD45RA- CD28- C	1	56588047
ebi-a-GCST90001695	rs113759128	CD45RA- CD28- C	1	28458334
ebi-a-GCST90001695	rs12062472	CD45RA- CD28- C	1	17431300
ebi-a-GCST90001695	rs114507999	CD45RA- CD28- C	1	21892266
ebi-a-GCST90001695	rs150650566	CD45RA- CD28- C	1	83046200
ebi-a-GCST90001695	rs150416566	CD45RA- CD28- C	1	108257502
ebi-a-GCST90001695	rs138010330	CD45RA- CD28- C	1	161526187
ebi-a-GCST90001695	rs138480948	CD45RA- CD28- C	1	64420637
ebi-a-GCST90001695	rs141289216	CD45RA- CD28- C	1	14539144
ebi-a-GCST90001695	rs138829583	CD45RA- CD28- C	1	79094990
ebi-a-GCST90001695	rs149124296	CD45RA- CD28- C	1	213124992
ebi-a-GCST90001695	rs75097808	CD45RA- CD28- C	1	154796811
ebi-a-GCST90001695	rs149737122	CD45RA- CD28- C	1	96560455
ebi-a-GCST90001695	rs139859886	CD45RA- CD28- C	1	98055569
ebi-a-GCST90001695	rs183203096	CD45RA- CD28- C	1	103246274
ebi-a-GCST90001695	rs79656514	CD45RA- CD28- C	1	235943573
ebi-a-GCST90001695	rs72815709	CD45RA- CD28- C	2	80415786
ebi-a-GCST90001695	rs150146567	CD45RA- CD28- C	2	12784208
ebi-a-GCST90001695	rs72929459	CD45RA- CD28- C	2	200181613
ebi-a-GCST90001695	rs80139012	CD45RA- CD28- C	2	6119988
ebi-a-GCST90001695	rs138799468	CD45RA- CD28- C	2	9441279
ebi-a-GCST90001695	rs112839187	CD45RA- CD28- C	2	238422350
ebi-a-GCST90001695	rs115954619	CD45RA- CD28- C	2	174896997
ebi-a-GCST90001695	rs114763811	CD45RA- CD28- C	2	191319109
ebi-a-GCST90001695	rs79932972	CD45RA- CD28- C	2	142744587
ebi-a-GCST90001695	rs144711723	CD45RA- CD28- C	2	145836600
ebi-a-GCST90001695	rs185697137	CD45RA- CD28- C	2	147044386
ebi-a-GCST90001695	rs74932477	CD45RA- CD28- C	2	229222112
ebi-a-GCST90001695	rs151288286	CD45RA- CD28- C	2	106552757
ebi-a-GCST90001695	rs58905133	CD45RA- CD28- C	2	38887964
ebi-a-GCST90001695	rs147427289	CD45RA- CD28- C	2	40489896
ebi-a-GCST90001695	rs148026776	CD45RA- CD28- C	2	155487782
ebi-a-GCST90001695	rs11899522	CD45RA- CD28- C	2	54396879
ebi-a-GCST90001695	rs138962348	CD45RA- CD28- C	2	146534594
ebi-a-GCST90001695	rs17008005	CD45RA- CD28- C	2	29596767
ebi-a-GCST90001695	rs147205787	CD45RA- CD28- C	2	144815972
ebi-a-GCST90001695	rs75828981	CD45RA- CD28- C	2	144233823
ebi-a-GCST90001695	rs77721642	CD45RA- CD28- C	2	154028459
ebi-a-GCST90001695	rs137978465	CD45RA- CD28- C	2	228411157
ebi-a-GCST90001695	rs112785730	CD45RA- CD28- C	2	127831527
ebi-a-GCST90001695	rs192932211	CD45RA- CD28- C	2	10422752
ebi-a-GCST90001695	rs12464465	CD45RA- CD28- C	2	176542288
ebi-a-GCST90001695	rs77843545	CD45RA- CD28- C	2	237100311
ebi-a-GCST90001695	rs72996438	CD45RA- CD28- C	2	211172214
ebi-a-GCST90001695	rs184901419	CD45RA- CD28- C	3	99971319
ebi-a-GCST90001695	rs115445664	CD45RA- CD28- C	3	126486072

ebi-a-GCST90001695	rs79397670	CD45RA- CD28- C	3	3816462
ebi-a-GCST90001695	rs148242949	CD45RA- CD28- C	3	59179373
ebi-a-GCST90001695	rs116779558	CD45RA- CD28- C	3	77069014
ebi-a-GCST90001695	rs140517857	CD45RA- CD28- C	3	70804684
ebi-a-GCST90001695	rs141023610	CD45RA- CD28- C	3	15790899
ebi-a-GCST90001695	rs79489889	CD45RA- CD28- C	3	39505173
ebi-a-GCST90001695	rs77577578	CD45RA- CD28- C	3	192050467
ebi-a-GCST90001695	rs11716645	CD45RA- CD28- C	3	29402908
ebi-a-GCST90001695	rs112545773	CD45RA- CD28- C	3	141081402
ebi-a-GCST90001695	rs142115354	CD45RA- CD28- C	3	42897614
ebi-a-GCST90001695	rs148658406	CD45RA- CD28- C	3	153311399
ebi-a-GCST90001695	rs115945428	CD45RA- CD28- C	3	110093567
ebi-a-GCST90001695	rs139772031	CD45RA- CD28- C	3	10035985
ebi-a-GCST90001695	rs35268412	CD45RA- CD28- C	3	141999791
ebi-a-GCST90001695	rs16864501	CD45RA- CD28- C	3	152385976
ebi-a-GCST90001695	rs115879183	CD45RA- CD28- C	3	194347376
ebi-a-GCST90001695	rs114057146	CD45RA- CD28- C	3	190504703
ebi-a-GCST90001695	rs79665127	CD45RA- CD28- C	3	190758619
ebi-a-GCST90001695	rs149994650	CD45RA- CD28- C	3	18550864
ebi-a-GCST90001695	rs545794900	CD45RA- CD28- C	3	3710729
ebi-a-GCST90001695	rs140311178	CD45RA- CD28- C	4	8174723
ebi-a-GCST90001695	rs143044126	CD45RA- CD28- C	4	31116547
ebi-a-GCST90001695	rs148799115	CD45RA- CD28- C	4	45244488
ebi-a-GCST90001695	rs55830085	CD45RA- CD28- C	4	106862213
ebi-a-GCST90001695	rs77152285	CD45RA- CD28- C	4	93935039
ebi-a-GCST90001695	rs183097749	CD45RA- CD28- C	4	171324708
ebi-a-GCST90001695	rs192021609	CD45RA- CD28- C	4	157192771
ebi-a-GCST90001695	rs1522563	CD45RA- CD28- C	4	171653752
ebi-a-GCST90001695	rs183382760	CD45RA- CD28- C	4	116470715
ebi-a-GCST90001695	rs115928015	CD45RA- CD28- C	4	156055689
ebi-a-GCST90001695	rs114619811	CD45RA- CD28- C	4	71875684
ebi-a-GCST90001695	rs117905416	CD45RA- CD28- C	4	48119663
ebi-a-GCST90001695	rs192553272	CD45RA- CD28- C	4	114347303
ebi-a-GCST90001695	rs35046933	CD45RA- CD28- C	4	57059988
ebi-a-GCST90001695	rs72673255	CD45RA- CD28- C	4	126220318
ebi-a-GCST90001695	rs62409210	CD45RA- CD28- C	4	25412773
ebi-a-GCST90001695	rs116807650	CD45RA- CD28- C	4	112605741
ebi-a-GCST90001695	rs76506268	CD45RA- CD28- C	4	187981624
ebi-a-GCST90001695	rs114109475	CD45RA- CD28- C	4	47216939
ebi-a-GCST90001695	rs139748481	CD45RA- CD28- C	4	13226947
ebi-a-GCST90001695	rs79870119	CD45RA- CD28- C	4	64142694
ebi-a-GCST90001695	rs112028149	CD45RA- CD28- C	4	60775910
ebi-a-GCST90001695	rs142857708	CD45RA- CD28- C	4	133864790
ebi-a-GCST90001695	rs111981818	CD45RA- CD28- C	4	2899648
ebi-a-GCST90001695	rs59951086	CD45RA- CD28- C	4	187679105
ebi-a-GCST90001695	rs138615640	CD45RA- CD28- C	4	46871059
ebi-a-GCST90001695	rs28622225	CD45RA- CD28- C	4	166260369
ebi-a-GCST90001695	rs77953154	CD45RA- CD28- C	5	179723042

ebi-a-GCST90001695	rs80036087	CD45RA- CD28- C	5	3678994
ebi-a-GCST90001695	rs114935210	CD45RA- CD28- C	5	134766276
ebi-a-GCST90001695	rs114216104	CD45RA- CD28- C	5	140131486
ebi-a-GCST90001695	rs115546994	CD45RA- CD28- C	5	159062854
ebi-a-GCST90001695	rs138324315	CD45RA- CD28- C	5	39721163
ebi-a-GCST90001695	rs116581718	CD45RA- CD28- C	5	29413612
ebi-a-GCST90001695	rs111731860	CD45RA- CD28- C	5	175121768
ebi-a-GCST90001695	rs78604443	CD45RA- CD28- C	5	179476195
ebi-a-GCST90001695	rs62369101	CD45RA- CD28- C	5	97959184
ebi-a-GCST90001695	rs12656604	CD45RA- CD28- C	5	72679056
ebi-a-GCST90001695	rs145266404	CD45RA- CD28- C	5	172300229
ebi-a-GCST90001695	rs1604388	CD45RA- CD28- C	5	81759022
ebi-a-GCST90001695	rs147014864	CD45RA- CD28- C	5	22132055
ebi-a-GCST90001695	rs114128029	CD45RA- CD28- C	5	79070021
ebi-a-GCST90001695	rs62383308	CD45RA- CD28- C	5	165460085
ebi-a-GCST90001695	rs76343453	CD45RA- CD28- C	5	81353920
ebi-a-GCST90001695	rs2071556	CD45RA- CD28- C	6	32904601
ebi-a-GCST90001695	rs78939697	CD45RA- CD28- C	6	67374008
ebi-a-GCST90001695	rs114801643	CD45RA- CD28- C	6	66316172
ebi-a-GCST90001695	rs77777749	CD45RA- CD28- C	6	118275279
ebi-a-GCST90001695	rs141926147	CD45RA- CD28- C	6	146863903
ebi-a-GCST90001695	rs187115825	CD45RA- CD28- C	6	382181
ebi-a-GCST90001695	rs149782409	CD45RA- CD28- C	6	14931262
ebi-a-GCST90001695	rs139613266	CD45RA- CD28- C	6	101930392
ebi-a-GCST90001695	rs144920942	CD45RA- CD28- C	6	104569120
ebi-a-GCST90001695	rs145336406	CD45RA- CD28- C	6	78745175
ebi-a-GCST90001695	rs17212021	CD45RA- CD28- C	6	32637474
ebi-a-GCST90001695	rs149957527	CD45RA- CD28- C	6	52375282
ebi-a-GCST90001695	rs130071	CD45RA- CD28- C	6	31116210
ebi-a-GCST90001695	rs147385696	CD45RA- CD28- C	6	143880059
ebi-a-GCST90001695	rs55779481	CD45RA- CD28- C	6	119136518
ebi-a-GCST90001695	rs145174666	CD45RA- CD28- C	6	6745192
ebi-a-GCST90001695	rs74651043	CD45RA- CD28- C	6	24233134
ebi-a-GCST90001695	rs115485721	CD45RA- CD28- C	6	65783601
ebi-a-GCST90001695	rs145613913	CD45RA- CD28- C	6	70855865
ebi-a-GCST90001695	rs58860560	CD45RA- CD28- C	6	79460848
ebi-a-GCST90001695	rs541695620	CD45RA- CD28- C	6	69157587
ebi-a-GCST90001695	rs34961721	CD45RA- CD28- C	6	83087501
ebi-a-GCST90001695	rs71567655	CD45RA- CD28- C	6	162968515
ebi-a-GCST90001695	rs115379408	CD45RA- CD28- C	6	18617129
ebi-a-GCST90001695	rs112857714	CD45RA- CD28- C	6	148462118
ebi-a-GCST90001695	rs143986791	CD45RA- CD28- C	7	62268900
ebi-a-GCST90001695	rs142522853	CD45RA- CD28- C	7	1084031
ebi-a-GCST90001695	rs185778924	CD45RA- CD28- C	7	148905542
ebi-a-GCST90001695	rs145137280	CD45RA- CD28- C	7	147207957
ebi-a-GCST90001695	rs140112233	CD45RA- CD28- C	7	115835641
ebi-a-GCST90001695	rs138358136	CD45RA- CD28- C	7	194075
ebi-a-GCST90001695	rs117469911	CD45RA- CD28- C	7	79791344

ebi-a-GCST90001695	rs143845801	CD45RA- CD28- C	7	84434549
ebi-a-GCST90001695	rs117845490	CD45RA- CD28- C	7	113248370
ebi-a-GCST90001695	rs151261173	CD45RA- CD28- C	7	70763152
ebi-a-GCST90001695	rs34592474	CD45RA- CD28- C	7	57279055
ebi-a-GCST90001695	rs79647440	CD45RA- CD28- C	7	142438302
ebi-a-GCST90001695	rs181551194	CD45RA- CD28- C	7	73339258
ebi-a-GCST90001695	rs77307127	CD45RA- CD28- C	7	151528768
ebi-a-GCST90001695	rs77164608	CD45RA- CD28- C	7	21673701
ebi-a-GCST90001695	rs141112932	CD45RA- CD28- C	7	30866360
ebi-a-GCST90001695	rs117271894	CD45RA- CD28- C	8	112881674
ebi-a-GCST90001695	rs59236867	CD45RA- CD28- C	8	42077996
ebi-a-GCST90001695	rs7011687	CD45RA- CD28- C	8	141472683
ebi-a-GCST90001695	rs116960471	CD45RA- CD28- C	8	1549975
ebi-a-GCST90001695	rs77654966	CD45RA- CD28- C	8	27058930
ebi-a-GCST90001695	rs141515499	CD45RA- CD28- C	8	57100983
ebi-a-GCST90001695	rs192253149	CD45RA- CD28- C	8	6255718
ebi-a-GCST90001695	rs62513410	CD45RA- CD28- C	8	142607179
ebi-a-GCST90001695	rs143825022	CD45RA- CD28- C	8	28561116
ebi-a-GCST90001695	rs3133836	CD45RA- CD28- C	8	104363338
ebi-a-GCST90001695	rs62488802	CD45RA- CD28- C	8	7162695
ebi-a-GCST90001695	rs111456999	CD45RA- CD28- C	8	3156062
ebi-a-GCST90001695	rs116959045	CD45RA- CD28- C	8	53929771
ebi-a-GCST90001695	rs72728362	CD45RA- CD28- C	8	127054086
ebi-a-GCST90001695	rs6586916	CD45RA- CD28- C	8	20371089
ebi-a-GCST90001695	rs118082680	CD45RA- CD28- C	8	21084293
ebi-a-GCST90001695	rs535005711	CD45RA- CD28- C	8	144233069
ebi-a-GCST90001695	rs118024980	CD45RA- CD28- C	9	9009338
ebi-a-GCST90001695	rs10759064	CD45RA- CD28- C	9	9405003
ebi-a-GCST90001695	rs62582217	CD45RA- CD28- C	9	134320210
ebi-a-GCST90001695	rs34609592	CD45RA- CD28- C	9	136915695
ebi-a-GCST90001695	rs72743927	CD45RA- CD28- C	9	98810796
ebi-a-GCST90001695	rs78849864	CD45RA- CD28- C	9	134242324
ebi-a-GCST90001695	rs75067089	CD45RA- CD28- C	9	14983311
ebi-a-GCST90001695	rs117196114	CD45RA- CD28- C	9	83853182
ebi-a-GCST90001695	rs75225279	CD45RA- CD28- C	9	25938569
ebi-a-GCST90001695	rs113447030	CD45RA- CD28- C	9	124865332
ebi-a-GCST90001695	rs151120803	CD45RA- CD28- C	9	96711406
ebi-a-GCST90001695	rs79407975	CD45RA- CD28- C	10	28109090
ebi-a-GCST90001695	rs144431284	CD45RA- CD28- C	10	30823850
ebi-a-GCST90001695	rs117006645	CD45RA- CD28- C	10	108143796
ebi-a-GCST90001695	rs145744161	CD45RA- CD28- C	10	108259029
ebi-a-GCST90001695	rs61864030	CD45RA- CD28- C	10	133431823
ebi-a-GCST90001695	rs117739788	CD45RA- CD28- C	10	56884368
ebi-a-GCST90001695	rs189683893	CD45RA- CD28- C	10	89153882
ebi-a-GCST90001695	rs75067680	CD45RA- CD28- C	10	133817829
ebi-a-GCST90001695	rs72773345	CD45RA- CD28- C	10	8494842
ebi-a-GCST90001695	rs117437702	CD45RA- CD28- C	10	110752679
ebi-a-GCST90001695	rs148889514	CD45RA- CD28- C	10	111548575

ebi-a-GCST90001695	rs146914701	CD45RA- CD28- C	10	109409332
ebi-a-GCST90001695	rs45507192	CD45RA- CD28- C	10	21304171
ebi-a-GCST90001695	rs150841550	CD45RA- CD28- C	10	11478633
ebi-a-GCST90001695	rs139513469	CD45RA- CD28- C	10	66332109
ebi-a-GCST90001695	rs143930177	CD45RA- CD28- C	10	70543706
ebi-a-GCST90001695	rs150427323	CD45RA- CD28- C	11	118189351
ebi-a-GCST90001695	rs528814030	CD45RA- CD28- C	11	117015217
ebi-a-GCST90001695	rs117431224	CD45RA- CD28- C	11	44356396
ebi-a-GCST90001695	rs11232730	CD45RA- CD28- C	11	81281823
ebi-a-GCST90001695	rs117413806	CD45RA- CD28- C	11	16290338
ebi-a-GCST90001695	rs12279408	CD45RA- CD28- C	11	84269972
ebi-a-GCST90001695	rs186889529	CD45RA- CD28- C	11	59109032
ebi-a-GCST90001695	rs141057204	CD45RA- CD28- C	11	130013797
ebi-a-GCST90001695	rs117277690	CD45RA- CD28- C	11	33322159
ebi-a-GCST90001695	rs111417549	CD45RA- CD28- C	11	36584481
ebi-a-GCST90001695	rs141656121	CD45RA- CD28- C	11	91872187
ebi-a-GCST90001695	rs185854402	CD45RA- CD28- C	11	116624918
ebi-a-GCST90001695	rs77315801	CD45RA- CD28- C	11	132912552
ebi-a-GCST90001695	rs17676161	CD45RA- CD28- C	11	65259911
ebi-a-GCST90001695	rs34499829	CD45RA- CD28- C	12	734001
ebi-a-GCST90001695	rs75005340	CD45RA- CD28- C	12	38141078
ebi-a-GCST90001695	rs61908026	CD45RA- CD28- C	12	2808166
ebi-a-GCST90001695	rs139243374	CD45RA- CD28- C	12	21581956
ebi-a-GCST90001695	rs17212998	CD45RA- CD28- C	12	13243168
ebi-a-GCST90001695	rs536210128	CD45RA- CD28- C	12	101687266
ebi-a-GCST90001695	rs17712916	CD45RA- CD28- C	12	64607264
ebi-a-GCST90001695	rs78788969	CD45RA- CD28- C	12	85026832
ebi-a-GCST90001695	rs117975109	CD45RA- CD28- C	12	52408142
ebi-a-GCST90001695	rs142611712	CD45RA- CD28- C	12	54827947
ebi-a-GCST90001695	rs139457426	CD45RA- CD28- C	12	47679643
ebi-a-GCST90001695	rs117785622	CD45RA- CD28- C	12	132283582
ebi-a-GCST90001695	rs142911819	CD45RA- CD28- C	12	114081812
ebi-a-GCST90001695	rs143230107	CD45RA- CD28- C	12	26115369
ebi-a-GCST90001695	rs75893788	CD45RA- CD28- C	12	84857033
ebi-a-GCST90001695	rs61939536	CD45RA- CD28- C	12	128643151
ebi-a-GCST90001695	rs74603821	CD45RA- CD28- C	12	101714936
ebi-a-GCST90001695	rs78420941	CD45RA- CD28- C	12	50294296
ebi-a-GCST90001695	rs74902462	CD45RA- CD28- C	12	59066617
ebi-a-GCST90001695	rs35078472	CD45RA- CD28- C	12	127715831
ebi-a-GCST90001695	rs113819655	CD45RA- CD28- C	13	100586942
ebi-a-GCST90001695	rs80142250	CD45RA- CD28- C	13	110903174
ebi-a-GCST90001695	rs117048727	CD45RA- CD28- C	13	44264686
ebi-a-GCST90001695	rs142470414	CD45RA- CD28- C	13	73498369
ebi-a-GCST90001695	rs35779961	CD45RA- CD28- C	13	102262527
ebi-a-GCST90001695	rs149043871	CD45RA- CD28- C	13	102446635
ebi-a-GCST90001695	rs80012883	CD45RA- CD28- C	13	94341740
ebi-a-GCST90001695	rs147398419	CD45RA- CD28- C	13	23525796
ebi-a-GCST90001695	rs190474871	CD45RA- CD28- C	13	25506269

ebi-a-GCST90001695	rs145022527	CD45RA- CD28- C	13	37118716
ebi-a-GCST90001695	rs141018536	CD45RA- CD28- C	13	90525482
ebi-a-GCST90001695	rs74444952	CD45RA- CD28- C	13	36209759
ebi-a-GCST90001695	rs75686073	CD45RA- CD28- C	14	68253542
ebi-a-GCST90001695	rs12882035	CD45RA- CD28- C	14	43977463
ebi-a-GCST90001695	rs111953852	CD45RA- CD28- C	14	84194540
ebi-a-GCST90001695	rs190267289	CD45RA- CD28- C	14	29011762
ebi-a-GCST90001695	rs140590797	CD45RA- CD28- C	14	62568446
ebi-a-GCST90001695	rs77243672	CD45RA- CD28- C	14	76846837
ebi-a-GCST90001695	rs116848911	CD45RA- CD28- C	14	79042294
ebi-a-GCST90001695	rs61988500	CD45RA- CD28- C	14	86640070
ebi-a-GCST90001695	rs150383571	CD45RA- CD28- C	14	43151367
ebi-a-GCST90001695	rs117475878	CD45RA- CD28- C	14	32709998
ebi-a-GCST90001695	rs116928874	CD45RA- CD28- C	14	22957038
ebi-a-GCST90001695	rs554483844	CD45RA- CD28- C	14	88292196
ebi-a-GCST90001695	rs17180391	CD45RA- CD28- C	14	72797255
ebi-a-GCST90001695	rs11160382	CD45RA- CD28- C	14	97572261
ebi-a-GCST90001695	rs76881655	CD45RA- CD28- C	15	26109688
ebi-a-GCST90001695	rs75884646	CD45RA- CD28- C	15	95776793
ebi-a-GCST90001695	rs79716258	CD45RA- CD28- C	15	55067476
ebi-a-GCST90001695	rs79888820	CD45RA- CD28- C	15	96888705
ebi-a-GCST90001695	rs186595909	CD45RA- CD28- C	15	78554710
ebi-a-GCST90001695	rs146940094	CD45RA- CD28- C	15	94946993
ebi-a-GCST90001695	rs145483124	CD45RA- CD28- C	15	85650544
ebi-a-GCST90001695	rs146986354	CD45RA- CD28- C	15	29116366
ebi-a-GCST90001695	rs112568609	CD45RA- CD28- C	15	27818525
ebi-a-GCST90001695	rs72739876	CD45RA- CD28- C	15	49106401
ebi-a-GCST90001695	rs62021713	CD45RA- CD28- C	15	50365389
ebi-a-GCST90001695	rs75477291	CD45RA- CD28- C	16	73579327
ebi-a-GCST90001695	rs140134394	CD45RA- CD28- C	16	17341866
ebi-a-GCST90001695	rs62033204	CD45RA- CD28- C	16	17388418
ebi-a-GCST90001695	rs140605634	CD45RA- CD28- C	16	68607748
ebi-a-GCST90001695	rs147802671	CD45RA- CD28- C	16	32022251
ebi-a-GCST90001695	rs151052734	CD45RA- CD28- C	16	51075587
ebi-a-GCST90001695	rs151237482	CD45RA- CD28- C	16	78214566
ebi-a-GCST90001695	rs150856260	CD45RA- CD28- C	16	26527473
ebi-a-GCST90001695	rs8056824	CD45RA- CD28- C	16	61800469
ebi-a-GCST90001695	rs188394601	CD45RA- CD28- C	16	70343845
ebi-a-GCST90001695	rs117362948	CD45RA- CD28- C	16	50169595
ebi-a-GCST90001695	rs117500319	CD45RA- CD28- C	16	3108127
ebi-a-GCST90001695	rs117262195	CD45RA- CD28- C	16	5991815
ebi-a-GCST90001695	rs116941636	CD45RA- CD28- C	16	79757046
ebi-a-GCST90001695	rs145957749	CD45RA- CD28- C	16	80690635
ebi-a-GCST90001695	rs149716976	CD45RA- CD28- C	16	75062625
ebi-a-GCST90001695	rs117863142	CD45RA- CD28- C	16	79245986
ebi-a-GCST90001695	rs2615702	CD45RA- CD28- C	16	81462243
ebi-a-GCST90001695	rs192302673	CD45RA- CD28- C	16	85685140
ebi-a-GCST90001695	rs146127940	CD45RA- CD28- C	17	29929827

ebi-a-GCST90001695	rs146164275	CD45RA- CD28- C	17	76005958
ebi-a-GCST90001695	rs140871773	CD45RA- CD28- C	17	13877125
ebi-a-GCST90001695	rs116876186	CD45RA- CD28- C	17	16446446
ebi-a-GCST90001695	rs72847598	CD45RA- CD28- C	17	71902183
ebi-a-GCST90001695	rs1007033	CD45RA- CD28- C	17	31695774
ebi-a-GCST90001695	rs148362510	CD45RA- CD28- C	18	3267975
ebi-a-GCST90001695	rs76383831	CD45RA- CD28- C	18	74630095
ebi-a-GCST90001695	rs151145927	CD45RA- CD28- C	18	38375388
ebi-a-GCST90001695	rs530410832	CD45RA- CD28- C	18	44380586
ebi-a-GCST90001695	rs148225159	CD45RA- CD28- C	18	56129994
ebi-a-GCST90001695	rs578157000	CD45RA- CD28- C	18	6304023
ebi-a-GCST90001695	rs147706586	CD45RA- CD28- C	18	3555698
ebi-a-GCST90001695	rs75346347	CD45RA- CD28- C	18	7862061
ebi-a-GCST90001695	rs139436159	CD45RA- CD28- C	18	60608014
ebi-a-GCST90001695	rs7238907	CD45RA- CD28- C	18	71686718
ebi-a-GCST90001695	rs149532201	CD45RA- CD28- C	18	26456104
ebi-a-GCST90001695	rs75420474	CD45RA- CD28- C	18	39828718
ebi-a-GCST90001695	rs71354045	CD45RA- CD28- C	19	57840862
ebi-a-GCST90001695	rs117428784	CD45RA- CD28- C	19	43422957
ebi-a-GCST90001695	rs116865617	CD45RA- CD28- C	19	14535355
ebi-a-GCST90001695	rs112362737	CD45RA- CD28- C	19	24522507
ebi-a-GCST90001695	rs62531041	CD45RA- CD28- C	19	48379346
ebi-a-GCST90001695	rs191604848	CD45RA- CD28- C	19	17591476
ebi-a-GCST90001695	rs6027989	CD45RA- CD28- C	20	59618023
ebi-a-GCST90001695	rs41276984	CD45RA- CD28- C	20	37580670
ebi-a-GCST90001695	rs77937827	CD45RA- CD28- C	20	20799344
ebi-a-GCST90001695	rs137942933	CD45RA- CD28- C	20	33104122
ebi-a-GCST90001695	rs148291061	CD45RA- CD28- C	20	3135837
ebi-a-GCST90001695	rs56067013	CD45RA- CD28- C	20	46616280
ebi-a-GCST90001695	rs75427771	CD45RA- CD28- C	20	59382138
ebi-a-GCST90001695	rs117036387	CD45RA- CD28- C	20	3521072
ebi-a-GCST90001695	rs192717771	CD45RA- CD28- C	21	28699078
ebi-a-GCST90001695	rs117514047	CD45RA- CD28- C	21	26316207
ebi-a-GCST90001695	rs140280232	CD45RA- CD28- C	21	46387655
ebi-a-GCST90001695	rs113432575	CD45RA- CD28- C	21	30128360
ebi-a-GCST90001695	rs142419740	CD45RA- CD28- C	21	47472186
ebi-a-GCST90001695	rs78680127	CD45RA- CD28- C	21	41099181
ebi-a-GCST90001695	rs5997061	CD45RA- CD28- C	22	26677514
ebi-a-GCST90001695	rs144599349	CD45RA- CD28- C	22	45887357
ebi-a-GCST90001695	rs143237141	CD45RA- CD28- C	22	45176439
ebi-a-GCST90001695	rs147132955	CD45RA- CD28- C	22	24296877
ebi-a-GCST90001695	rs191357081	CD45RA- CD28- C	22	41021471
ebi-a-GCST90001696	rs138758802	CD45RA- CD28- C	1	91894308
ebi-a-GCST90001696	rs55881385	CD45RA- CD28- C	1	34724192
ebi-a-GCST90001696	rs138754438	CD45RA- CD28- C	1	6329089
ebi-a-GCST90001696	rs17388638	CD45RA- CD28- C	1	209805394
ebi-a-GCST90001696	rs77145525	CD45RA- CD28- C	2	194765458
ebi-a-GCST90001696	rs17556536	CD45RA- CD28- C	2	40269291

ebi-a-GCST90001696	rs112774387	CD45RA- CD28- C	2	100757118
ebi-a-GCST90001696	rs114135956	CD45RA- CD28- C	2	8930530
ebi-a-GCST90001696	rs72815709	CD45RA- CD28- C	2	80415786
ebi-a-GCST90001696	rs450175	CD45RA- CD28- C	2	38400812
ebi-a-GCST90001696	rs58905133	CD45RA- CD28- C	2	38887964
ebi-a-GCST90001696	rs150146567	CD45RA- CD28- C	2	12784208
ebi-a-GCST90001696	rs66707192	CD45RA- CD28- C	3	186382065
ebi-a-GCST90001696	rs113679275	CD45RA- CD28- C	3	115470787
ebi-a-GCST90001696	rs72978357	CD45RA- CD28- C	3	133630044
ebi-a-GCST90001696	rs142507918	CD45RA- CD28- C	4	188764989
ebi-a-GCST90001696	rs116077033	CD45RA- CD28- C	5	114060904
ebi-a-GCST90001696	rs77953154	CD45RA- CD28- C	5	179723042
ebi-a-GCST90001696	rs114216104	CD45RA- CD28- C	5	140131486
ebi-a-GCST90001696	rs76343453	CD45RA- CD28- C	5	81353920
ebi-a-GCST90001696	rs151336928	CD45RA- CD28- C	5	23996586
ebi-a-GCST90001696	rs6941748	CD45RA- CD28- C	6	45557327
ebi-a-GCST90001696	rs145613913	CD45RA- CD28- C	6	70855865
ebi-a-GCST90001696	rs130071	CD45RA- CD28- C	6	31116210
ebi-a-GCST90001696	rs17212021	CD45RA- CD28- C	6	32637474
ebi-a-GCST90001696	rs1736913	CD45RA- CD28- C	6	29704400
ebi-a-GCST90001696	rs9468632	CD45RA- CD28- C	6	29775939
ebi-a-GCST90001696	rs149600977	CD45RA- CD28- C	6	9242018
ebi-a-GCST90001696	rs3133835	CD45RA- CD28- C	8	104363126
ebi-a-GCST90001696	rs149487182	CD45RA- CD28- C	8	131518018
ebi-a-GCST90001696	rs62488802	CD45RA- CD28- C	8	7162695
ebi-a-GCST90001696	rs10970967	CD45RA- CD28- C	9	3250025
ebi-a-GCST90001696	rs12791920	CD45RA- CD28- C	11	910259
ebi-a-GCST90001696	rs538264691	CD45RA- CD28- C	12	97040021
ebi-a-GCST90001696	rs61188057	CD45RA- CD28- C	12	59068194
ebi-a-GCST90001696	rs142399295	CD45RA- CD28- C	12	38143987
ebi-a-GCST90001696	rs66657257	CD45RA- CD28- C	13	93732550
ebi-a-GCST90001696	rs742867	CD45RA- CD28- C	14	68217724
ebi-a-GCST90001696	rs140385926	CD45RA- CD28- C	15	69903187
ebi-a-GCST90001696	rs149716976	CD45RA- CD28- C	16	75062625
ebi-a-GCST90001696	rs151237482	CD45RA- CD28- C	16	78214566
ebi-a-GCST90001696	rs190312656	CD45RA- CD28- C	16	81625215
ebi-a-GCST90001696	rs150856260	CD45RA- CD28- C	16	26527473
ebi-a-GCST90001696	rs62033204	CD45RA- CD28- C	16	17388418
ebi-a-GCST90001696	rs77946649	CD45RA- CD28- C	17	67795226
ebi-a-GCST90001696	rs148265436	CD45RA- CD28- C	17	71407818
ebi-a-GCST90001696	rs75420474	CD45RA- CD28- C	18	39828718
ebi-a-GCST90001696	rs75573571	CD45RA- CD28- C	18	72932358
ebi-a-GCST90001696	rs55896100	CD45RA- CD28- C	19	17509059
ebi-a-GCST90001696	rs117252706	CD45RA- CD28- C	19	3310194
ebi-a-GCST90001696	rs117428784	CD45RA- CD28- C	19	43422957
ebi-a-GCST90001696	rs77920451	CD45RA- CD28- C	20	55040109
ebi-a-GCST90001696	rs75427771	CD45RA- CD28- C	20	59382138
ebi-a-GCST90001696	rs6001335	CD45RA- CD28- C	22	39345966

ebi-a-GCST90001697	rs150650566	CD45RA- CD28- C	1	83046200
ebi-a-GCST90001697	rs144571871	CD45RA- CD28- C	1	65133062
ebi-a-GCST90001697	rs142707561	CD45RA- CD28- C	1	42381260
ebi-a-GCST90001697	rs138754438	CD45RA- CD28- C	1	6329089
ebi-a-GCST90001697	rs113971555	CD45RA- CD28- C	1	2776366
ebi-a-GCST90001697	rs55881385	CD45RA- CD28- C	1	34724192
ebi-a-GCST90001697	rs140434581	CD45RA- CD28- C	1	18070672
ebi-a-GCST90001697	rs55971473	CD45RA- CD28- C	1	117061778
ebi-a-GCST90001697	rs138758802	CD45RA- CD28- C	1	91894308
ebi-a-GCST90001697	rs61772921	CD45RA- CD28- C	1	80897562
ebi-a-GCST90001697	rs139859886	CD45RA- CD28- C	1	98055569
ebi-a-GCST90001697	rs115427760	CD45RA- CD28- C	1	3815578
ebi-a-GCST90001697	rs149124296	CD45RA- CD28- C	1	213124992
ebi-a-GCST90001697	rs111978771	CD45RA- CD28- C	1	21025910
ebi-a-GCST90001697	rs142702764	CD45RA- CD28- C	1	158276239
ebi-a-GCST90001697	rs114691245	CD45RA- CD28- C	1	77137266
ebi-a-GCST90001697	rs77103383	CD45RA- CD28- C	1	81910383
ebi-a-GCST90001697	rs114763811	CD45RA- CD28- C	2	191319109
ebi-a-GCST90001697	rs17508302	CD45RA- CD28- C	2	3878640
ebi-a-GCST90001697	rs58905133	CD45RA- CD28- C	2	38887964
ebi-a-GCST90001697	rs28362959	CD45RA- CD28- C	2	75060287
ebi-a-GCST90001697	rs115954619	CD45RA- CD28- C	2	174896997
ebi-a-GCST90001697	rs79190516	CD45RA- CD28- C	2	151197759
ebi-a-GCST90001697	rs12464465	CD45RA- CD28- C	2	176542288
ebi-a-GCST90001697	rs137978465	CD45RA- CD28- C	2	228411157
ebi-a-GCST90001697	rs74932477	CD45RA- CD28- C	2	229222112
ebi-a-GCST90001697	rs79415906	CD45RA- CD28- C	2	194776474
ebi-a-GCST90001697	rs11890933	CD45RA- CD28- C	2	177348686
ebi-a-GCST90001697	rs144434471	CD45RA- CD28- C	2	153006087
ebi-a-GCST90001697	rs151232118	CD45RA- CD28- C	2	242141013
ebi-a-GCST90001697	rs114135956	CD45RA- CD28- C	2	8930530
ebi-a-GCST90001697	rs190272290	CD45RA- CD28- C	2	9944052
ebi-a-GCST90001697	rs17024974	CD45RA- CD28- C	2	40275338
ebi-a-GCST90001697	rs112774387	CD45RA- CD28- C	2	100757118
ebi-a-GCST90001697	rs72815709	CD45RA- CD28- C	2	80415786
ebi-a-GCST90001697	rs150146567	CD45RA- CD28- C	2	12784208
ebi-a-GCST90001697	rs146323263	CD45RA- CD28- C	2	87376401
ebi-a-GCST90001697	rs115749134	CD45RA- CD28- C	2	199992008
ebi-a-GCST90001697	rs72995054	CD45RA- CD28- C	2	239863536
ebi-a-GCST90001697	rs149604038	CD45RA- CD28- C	3	185463545
ebi-a-GCST90001697	rs139772031	CD45RA- CD28- C	3	10035985
ebi-a-GCST90001697	rs149994650	CD45RA- CD28- C	3	18550864
ebi-a-GCST90001697	rs116802453	CD45RA- CD28- C	4	159714312
ebi-a-GCST90001697	rs111981818	CD45RA- CD28- C	4	2899648
ebi-a-GCST90001697	rs62409210	CD45RA- CD28- C	4	25412773
ebi-a-GCST90001697	rs183382760	CD45RA- CD28- C	4	116470715
ebi-a-GCST90001697	rs142507918	CD45RA- CD28- C	4	188764989
ebi-a-GCST90001697	rs115618542	CD45RA- CD28- C	4	70805245

ebi-a-GCST90001697	rs115336001	CD45RA- CD28- C	4	86157011
ebi-a-GCST90001697	rs76506268	CD45RA- CD28- C	4	187981624
ebi-a-GCST90001697	rs28722483	CD45RA- CD28- C	4	171668297
ebi-a-GCST90001697	rs114128029	CD45RA- CD28- C	5	79070021
ebi-a-GCST90001697	rs75196616	CD45RA- CD28- C	5	116600218
ebi-a-GCST90001697	rs12656604	CD45RA- CD28- C	5	72679056
ebi-a-GCST90001697	rs115353080	CD45RA- CD28- C	5	82672572
ebi-a-GCST90001697	rs115724263	CD45RA- CD28- C	5	139389602
ebi-a-GCST90001697	rs115546994	CD45RA- CD28- C	5	159062854
ebi-a-GCST90001697	rs76015360	CD45RA- CD28- C	5	28822020
ebi-a-GCST90001697	rs151336928	CD45RA- CD28- C	5	23996586
ebi-a-GCST90001697	rs6868378	CD45RA- CD28- C	5	179876723
ebi-a-GCST90001697	rs187898623	CD45RA- CD28- C	5	88105461
ebi-a-GCST90001697	rs62369101	CD45RA- CD28- C	5	97959184
ebi-a-GCST90001697	rs114216104	CD45RA- CD28- C	5	140131486
ebi-a-GCST90001697	rs76343453	CD45RA- CD28- C	5	81353920
ebi-a-GCST90001697	rs143022760	CD45RA- CD28- C	5	110729776
ebi-a-GCST90001697	rs563562215	CD45RA- CD28- C	6	73393170
ebi-a-GCST90001697	rs190399027	CD45RA- CD28- C	6	26077482
ebi-a-GCST90001697	rs1894743	CD45RA- CD28- C	6	31326794
ebi-a-GCST90001697	rs114170505	CD45RA- CD28- C	6	32666487
ebi-a-GCST90001697	rs10499012	CD45RA- CD28- C	6	97767591
ebi-a-GCST90001697	rs7752387	CD45RA- CD28- C	6	52903887
ebi-a-GCST90001697	rs130071	CD45RA- CD28- C	6	31116210
ebi-a-GCST90001697	rs145613913	CD45RA- CD28- C	6	70855865
ebi-a-GCST90001697	rs187115825	CD45RA- CD28- C	6	382181
ebi-a-GCST90001697	rs116227354	CD45RA- CD28- C	6	30102241
ebi-a-GCST90001697	rs76938777	CD45RA- CD28- C	7	31473901
ebi-a-GCST90001697	rs142647916	CD45RA- CD28- C	7	39125654
ebi-a-GCST90001697	rs185778924	CD45RA- CD28- C	7	148905542
ebi-a-GCST90001697	rs142522853	CD45RA- CD28- C	7	1084031
ebi-a-GCST90001697	rs140112233	CD45RA- CD28- C	7	115835641
ebi-a-GCST90001697	rs138339769	CD45RA- CD28- C	7	146509534
ebi-a-GCST90001697	rs11561957	CD45RA- CD28- C	7	135470972
ebi-a-GCST90001697	rs62439313	CD45RA- CD28- C	7	20898984
ebi-a-GCST90001697	rs2977034	CD45RA- CD28- C	8	88503714
ebi-a-GCST90001697	rs62488802	CD45RA- CD28- C	8	7162695
ebi-a-GCST90001697	rs147794754	CD45RA- CD28- C	8	130397472
ebi-a-GCST90001697	rs149487182	CD45RA- CD28- C	8	131518018
ebi-a-GCST90001697	rs3133836	CD45RA- CD28- C	8	104363338
ebi-a-GCST90001697	rs181997131	CD45RA- CD28- C	8	81285696
ebi-a-GCST90001697	rs117354956	CD45RA- CD28- C	8	61304968
ebi-a-GCST90001697	rs143825022	CD45RA- CD28- C	8	28561116
ebi-a-GCST90001697	rs10987410	CD45RA- CD28- C	9	129448120
ebi-a-GCST90001697	rs111710268	CD45RA- CD28- C	9	93695603
ebi-a-GCST90001697	rs139422413	CD45RA- CD28- C	9	9765228
ebi-a-GCST90001697	rs116985924	CD45RA- CD28- C	9	2716911
ebi-a-GCST90001697	rs146914701	CD45RA- CD28- C	10	109409332

ebi-a-GCST90001697	rs72773345	CD45RA- CD28- C	10	8494842
ebi-a-GCST90001697	rs35281771	CD45RA- CD28- C	10	71334832
ebi-a-GCST90001697	rs182940298	CD45RA- CD28- C	10	37524966
ebi-a-GCST90001697	rs142980650	CD45RA- CD28- C	10	109010961
ebi-a-GCST90001697	rs139513469	CD45RA- CD28- C	10	66332109
ebi-a-GCST90001697	rs117006645	CD45RA- CD28- C	10	108143796
ebi-a-GCST90001697	rs145744161	CD45RA- CD28- C	10	108259029
ebi-a-GCST90001697	rs143930177	CD45RA- CD28- C	10	70543706
ebi-a-GCST90001697	rs148384064	CD45RA- CD28- C	10	71016160
ebi-a-GCST90001697	rs144159369	CD45RA- CD28- C	11	126353889
ebi-a-GCST90001697	rs141057204	CD45RA- CD28- C	11	130013797
ebi-a-GCST90001697	rs141656121	CD45RA- CD28- C	11	91872187
ebi-a-GCST90001697	rs78722454	CD45RA- CD28- C	11	60213203
ebi-a-GCST90001697	rs12789925	CD45RA- CD28- C	11	11865406
ebi-a-GCST90001697	rs146319541	CD45RA- CD28- C	11	16659699
ebi-a-GCST90001697	rs146027781	CD45RA- CD28- C	11	54986405
ebi-a-GCST90001697	rs75845707	CD45RA- CD28- C	11	112719342
ebi-a-GCST90001697	rs11233425	CD45RA- CD28- C	11	82732689
ebi-a-GCST90001697	rs17676161	CD45RA- CD28- C	11	65259911
ebi-a-GCST90001697	rs139018186	CD45RA- CD28- C	11	10046307
ebi-a-GCST90001697	rs139457426	CD45RA- CD28- C	12	47679643
ebi-a-GCST90001697	rs186876963	CD45RA- CD28- C	12	34609393
ebi-a-GCST90001697	rs61908026	CD45RA- CD28- C	12	2808166
ebi-a-GCST90001697	rs142368429	CD45RA- CD28- C	12	121240098
ebi-a-GCST90001697	rs72648003	CD45RA- CD28- C	12	110018408
ebi-a-GCST90001697	rs117975109	CD45RA- CD28- C	12	52408142
ebi-a-GCST90001697	rs74902462	CD45RA- CD28- C	12	59066617
ebi-a-GCST90001697	rs78788969	CD45RA- CD28- C	12	85026832
ebi-a-GCST90001697	rs75446793	CD45RA- CD28- C	12	99748029
ebi-a-GCST90001697	rs538264691	CD45RA- CD28- C	12	97040021
ebi-a-GCST90001697	rs2058467	CD45RA- CD28- C	12	5924445
ebi-a-GCST90001697	rs111388649	CD45RA- CD28- C	12	28246470
ebi-a-GCST90001697	rs66657257	CD45RA- CD28- C	13	93732550
ebi-a-GCST90001697	rs113819655	CD45RA- CD28- C	13	100586942
ebi-a-GCST90001697	rs113972785	CD45RA- CD28- C	13	85399806
ebi-a-GCST90001697	rs75405648	CD45RA- CD28- C	13	108798780
ebi-a-GCST90001697	rs118053428	CD45RA- CD28- C	13	49828243
ebi-a-GCST90001697	rs117551202	CD45RA- CD28- C	14	45526169
ebi-a-GCST90001697	rs35673401	CD45RA- CD28- C	14	72914708
ebi-a-GCST90001697	rs742867	CD45RA- CD28- C	14	68217724
ebi-a-GCST90001697	rs117895563	CD45RA- CD28- C	14	92658912
ebi-a-GCST90001697	rs117475878	CD45RA- CD28- C	14	32709998
ebi-a-GCST90001697	rs76136072	CD45RA- CD28- C	14	22608720
ebi-a-GCST90001697	rs140590797	CD45RA- CD28- C	14	62568446
ebi-a-GCST90001697	rs62010321	CD45RA- CD28- C	15	69137961
ebi-a-GCST90001697	rs112568609	CD45RA- CD28- C	15	27818525
ebi-a-GCST90001697	rs147264423	CD45RA- CD28- C	15	78644995
ebi-a-GCST90001697	rs62021713	CD45RA- CD28- C	15	50365389

ebi-a-GCST90001697	rs72739876	CD45RA- CD28- C	15	49106401
ebi-a-GCST90001697	rs74707627	CD45RA- CD28- C	15	86484743
ebi-a-GCST90001697	rs150285115	CD45RA- CD28- C	15	67108460
ebi-a-GCST90001697	rs118106010	CD45RA- CD28- C	15	88790835
ebi-a-GCST90001697	rs77604796	CD45RA- CD28- C	15	55083216
ebi-a-GCST90001697	rs149716976	CD45RA- CD28- C	16	75062625
ebi-a-GCST90001697	rs151052734	CD45RA- CD28- C	16	51075587
ebi-a-GCST90001697	rs34375694	CD45RA- CD28- C	16	19193363
ebi-a-GCST90001697	rs151077900	CD45RA- CD28- C	16	1037963
ebi-a-GCST90001697	rs117262195	CD45RA- CD28- C	16	5991815
ebi-a-GCST90001697	rs113573055	CD45RA- CD28- C	16	15724919
ebi-a-GCST90001697	rs117134167	CD45RA- CD28- C	16	25778935
ebi-a-GCST90001697	rs192302673	CD45RA- CD28- C	16	85685140
ebi-a-GCST90001697	rs112018017	CD45RA- CD28- C	16	1532257
ebi-a-GCST90001697	rs79429801	CD45RA- CD28- C	16	61063115
ebi-a-GCST90001697	rs118189345	CD45RA- CD28- C	16	78415731
ebi-a-GCST90001697	rs12597262	CD45RA- CD28- C	16	86442575
ebi-a-GCST90001697	rs11079080	CD45RA- CD28- C	17	51509601
ebi-a-GCST90001697	rs148760558	CD45RA- CD28- C	17	72284938
ebi-a-GCST90001697	rs116876186	CD45RA- CD28- C	17	16446446
ebi-a-GCST90001697	rs2354767	CD45RA- CD28- C	17	72076433
ebi-a-GCST90001697	rs77946649	CD45RA- CD28- C	17	67795226
ebi-a-GCST90001697	rs71362776	CD45RA- CD28- C	18	75601988
ebi-a-GCST90001697	rs75573571	CD45RA- CD28- C	18	72932358
ebi-a-GCST90001697	rs75346347	CD45RA- CD28- C	18	7862061
ebi-a-GCST90001697	rs149532201	CD45RA- CD28- C	18	26456104
ebi-a-GCST90001697	rs75420474	CD45RA- CD28- C	18	39828718
ebi-a-GCST90001697	rs151145927	CD45RA- CD28- C	18	38375388
ebi-a-GCST90001697	rs75093468	CD45RA- CD28- C	19	46851328
ebi-a-GCST90001697	rs117428784	CD45RA- CD28- C	19	43422957
ebi-a-GCST90001697	rs191604848	CD45RA- CD28- C	19	17591476
ebi-a-GCST90001697	rs71363366	CD45RA- CD28- C	19	54261549
ebi-a-GCST90001697	rs117586879	CD45RA- CD28- C	20	17201328
ebi-a-GCST90001697	rs146663516	CD45RA- CD28- C	20	35238641
ebi-a-GCST90001697	rs77920451	CD45RA- CD28- C	20	55040109
ebi-a-GCST90001697	rs75427771	CD45RA- CD28- C	20	59382138
ebi-a-GCST90001697	rs79429254	CD45RA- CD28- C	21	16733570
ebi-a-GCST90001697	rs76797120	CD45RA- CD28- C	22	45793576
ebi-a-GCST90001697	rs149015425	CD45RA- CD28- C	22	50545634
ebi-a-GCST90001698	rs60470888	CD45RA+ CD28- C	1	164353833
ebi-a-GCST90001698	rs188687630	CD45RA+ CD28- C	1	7176852
ebi-a-GCST90001698	rs527693920	CD45RA+ CD28- C	1	17217922
ebi-a-GCST90001698	rs114672498	CD45RA+ CD28- C	1	4591565
ebi-a-GCST90001698	rs145583539	CD45RA+ CD28- C	1	66490103
ebi-a-GCST90001698	rs41299575	CD45RA+ CD28- C	1	113653257
ebi-a-GCST90001698	rs144942838	CD45RA+ CD28- C	1	111842326
ebi-a-GCST90001698	rs12118378	CD45RA+ CD28- C	1	68007080
ebi-a-GCST90001698	rs147591823	CD45RA+ CD28- C	1	47595103

ebi-a-GCST90001698	rs567145695	CD45RA+ CD28- (	1	246645088
ebi-a-GCST90001698	rs114021246	CD45RA+ CD28- (	1	46898789
ebi-a-GCST90001698	rs61784602	CD45RA+ CD28- (	1	46796909
ebi-a-GCST90001698	rs76444609	CD45RA+ CD28- (	1	249084997
ebi-a-GCST90001698	rs75312915	CD45RA+ CD28- (	1	2272160
ebi-a-GCST90001698	rs11576563	CD45RA+ CD28- (	1	6118693
ebi-a-GCST90001698	rs75255396	CD45RA+ CD28- (	1	35892939
ebi-a-GCST90001698	rs184269765	CD45RA+ CD28- (	1	36511989
ebi-a-GCST90001698	rs140963286	CD45RA+ CD28- (	1	24640467
ebi-a-GCST90001698	rs114791501	CD45RA+ CD28- (	1	81668305
ebi-a-GCST90001698	rs115175419	CD45RA+ CD28- (	1	100459655
ebi-a-GCST90001698	rs79183860	CD45RA+ CD28- (	1	21708196
ebi-a-GCST90001698	rs114230155	CD45RA+ CD28- (	1	102796337
ebi-a-GCST90001698	rs138905087	CD45RA+ CD28- (	1	87136899
ebi-a-GCST90001698	rs149050426	CD45RA+ CD28- (	1	160679804
ebi-a-GCST90001698	rs78085370	CD45RA+ CD28- (	1	186023973
ebi-a-GCST90001698	rs116446467	CD45RA+ CD28- (	1	187795006
ebi-a-GCST90001698	rs11572436	CD45RA+ CD28- (	1	217191870
ebi-a-GCST90001698	rs151086171	CD45RA+ CD28- (	1	223544915
ebi-a-GCST90001698	rs12130486	CD45RA+ CD28- (	1	81455274
ebi-a-GCST90001698	rs76636736	CD45RA+ CD28- (	1	99542806
ebi-a-GCST90001698	rs595254	CD45RA+ CD28- (	1	12228810
ebi-a-GCST90001698	rs78656354	CD45RA+ CD28- (	1	166905650
ebi-a-GCST90001698	rs72736883	CD45RA+ CD28- (	1	218388725
ebi-a-GCST90001698	rs111347604	CD45RA+ CD28- (	1	85641879
ebi-a-GCST90001698	rs114181559	CD45RA+ CD28- (	1	234239162
ebi-a-GCST90001698	rs554542958	CD45RA+ CD28- (	1	234912820
ebi-a-GCST90001698	rs114507999	CD45RA+ CD28- (	1	21892266
ebi-a-GCST90001698	rs1508603	CD45RA+ CD28- (	1	48031833
ebi-a-GCST90001698	rs550385698	CD45RA+ CD28- (	1	83698544
ebi-a-GCST90001698	rs138296471	CD45RA+ CD28- (	1	96963270
ebi-a-GCST90001698	rs34772471	CD45RA+ CD28- (	1	112269677
ebi-a-GCST90001698	rs573830319	CD45RA+ CD28- (	1	38393439
ebi-a-GCST90001698	rs373989598	CD45RA+ CD28- (	1	206572177
ebi-a-GCST90001698	rs12750174	CD45RA+ CD28- (	1	184765000
ebi-a-GCST90001698	rs148484526	CD45RA+ CD28- (	1	204459946
ebi-a-GCST90001698	rs116510159	CD45RA+ CD28- (	1	195636177
ebi-a-GCST90001698	rs75641434	CD45RA+ CD28- (	1	181280885
ebi-a-GCST90001698	rs532754584	CD45RA+ CD28- (	1	243352763
ebi-a-GCST90001698	rs139423647	CD45RA+ CD28- (	1	236238830
ebi-a-GCST90001698	rs141644068	CD45RA+ CD28- (	1	220781210
ebi-a-GCST90001698	rs186329934	CD45RA+ CD28- (	1	7815185
ebi-a-GCST90001698	rs12080172	CD45RA+ CD28- (	1	53470377
ebi-a-GCST90001698	rs143940797	CD45RA+ CD28- (	1	52644883
ebi-a-GCST90001698	rs77538429	CD45RA+ CD28- (	1	110742985
ebi-a-GCST90001698	rs138822361	CD45RA+ CD28- (	1	5236853
ebi-a-GCST90001698	rs113698814	CD45RA+ CD28- (	1	207678128
ebi-a-GCST90001698	rs76736944	CD45RA+ CD28- (	1	180881632

ebi-a-GCST90001698	rs142604048	CD45RA+ CD28- (	2	87503702
ebi-a-GCST90001698	rs184492468	CD45RA+ CD28- (	2	73477886
ebi-a-GCST90001698	rs138152896	CD45RA+ CD28- (	2	40546780
ebi-a-GCST90001698	rs146217030	CD45RA+ CD28- (	2	41343706
ebi-a-GCST90001698	rs77983529	CD45RA+ CD28- (	2	51721852
ebi-a-GCST90001698	rs141089930	CD45RA+ CD28- (	2	178028543
ebi-a-GCST90001698	rs3821186	CD45RA+ CD28- (	2	172953167
ebi-a-GCST90001698	rs111409203	CD45RA+ CD28- (	2	19743796
ebi-a-GCST90001698	rs148947489	CD45RA+ CD28- (	2	10326418
ebi-a-GCST90001698	rs79027575	CD45RA+ CD28- (	2	146371302
ebi-a-GCST90001698	rs180716798	CD45RA+ CD28- (	2	150956800
ebi-a-GCST90001698	rs141812086	CD45RA+ CD28- (	2	204682687
ebi-a-GCST90001698	rs112785730	CD45RA+ CD28- (	2	127831527
ebi-a-GCST90001698	rs192583129	CD45RA+ CD28- (	2	129405810
ebi-a-GCST90001698	rs183895386	CD45RA+ CD28- (	2	22576943
ebi-a-GCST90001698	rs149983528	CD45RA+ CD28- (	2	61772377
ebi-a-GCST90001698	rs190665193	CD45RA+ CD28- (	2	106438361
ebi-a-GCST90001698	rs35948774	CD45RA+ CD28- (	2	118664032
ebi-a-GCST90001698	rs377446032	CD45RA+ CD28- (	2	97762985
ebi-a-GCST90001698	rs7597139	CD45RA+ CD28- (	2	107560107
ebi-a-GCST90001698	rs115640110	CD45RA+ CD28- (	2	181840
ebi-a-GCST90001698	rs1508064	CD45RA+ CD28- (	2	72979172
ebi-a-GCST90001698	rs192562105	CD45RA+ CD28- (	2	53036426
ebi-a-GCST90001698	rs140702253	CD45RA+ CD28- (	2	194544900
ebi-a-GCST90001698	rs72931475	CD45RA+ CD28- (	2	159316835
ebi-a-GCST90001698	rs79038988	CD45RA+ CD28- (	2	210192689
ebi-a-GCST90001698	rs79391745	CD45RA+ CD28- (	2	83381537
ebi-a-GCST90001698	rs570386304	CD45RA+ CD28- (	2	233831336
ebi-a-GCST90001698	rs77317999	CD45RA+ CD28- (	2	52025038
ebi-a-GCST90001698	rs116034906	CD45RA+ CD28- (	2	118737657
ebi-a-GCST90001698	rs188021268	CD45RA+ CD28- (	2	67789879
ebi-a-GCST90001698	rs567695407	CD45RA+ CD28- (	2	107911125
ebi-a-GCST90001698	rs72836484	CD45RA+ CD28- (	2	84999809
ebi-a-GCST90001698	rs188292706	CD45RA+ CD28- (	2	162960872
ebi-a-GCST90001698	rs75561723	CD45RA+ CD28- (	2	199542954
ebi-a-GCST90001698	rs13010105	CD45RA+ CD28- (	2	42107980
ebi-a-GCST90001698	rs548679517	CD45RA+ CD28- (	2	17719245
ebi-a-GCST90001698	rs139404430	CD45RA+ CD28- (	2	7430709
ebi-a-GCST90001698	rs12616335	CD45RA+ CD28- (	2	159969605
ebi-a-GCST90001698	rs140929582	CD45RA+ CD28- (	2	169297185
ebi-a-GCST90001698	rs113513225	CD45RA+ CD28- (	2	192441892
ebi-a-GCST90001698	rs62175716	CD45RA+ CD28- (	2	152777402
ebi-a-GCST90001698	rs76568972	CD45RA+ CD28- (	2	1592272
ebi-a-GCST90001698	rs138567976	CD45RA+ CD28- (	2	8152821
ebi-a-GCST90001698	rs113206087	CD45RA+ CD28- (	2	21048037
ebi-a-GCST90001698	rs74620549	CD45RA+ CD28- (	2	36162712
ebi-a-GCST90001698	rs76316808	CD45RA+ CD28- (	2	123361495
ebi-a-GCST90001698	rs116039349	CD45RA+ CD28- (	2	132603936

ebi-a-GCST90001698	rs184350274	CD45RA+ CD28- (	2	228724105
ebi-a-GCST90001698	rs149131245	CD45RA+ CD28- (	2	234725994
ebi-a-GCST90001698	rs78530932	CD45RA+ CD28- (	2	81191156
ebi-a-GCST90001698	rs138367877	CD45RA+ CD28- (	2	64757961
ebi-a-GCST90001698	rs74499833	CD45RA+ CD28- (	2	77077801
ebi-a-GCST90001698	rs11885897	CD45RA+ CD28- (	2	225204126
ebi-a-GCST90001698	rs116086373	CD45RA+ CD28- (	2	223278095
ebi-a-GCST90001698	rs74932477	CD45RA+ CD28- (	2	229222112
ebi-a-GCST90001698	rs113883934	CD45RA+ CD28- (	2	104720870
ebi-a-GCST90001698	rs4853078	CD45RA+ CD28- (	2	75148081
ebi-a-GCST90001698	rs77678807	CD45RA+ CD28- (	2	208755904
ebi-a-GCST90001698	rs115517310	CD45RA+ CD28- (	2	170436614
ebi-a-GCST90001698	rs140173523	CD45RA+ CD28- (	2	195788562
ebi-a-GCST90001698	rs72996438	CD45RA+ CD28- (	2	211172214
ebi-a-GCST90001698	rs72908728	CD45RA+ CD28- (	2	174516215
ebi-a-GCST90001698	rs115945729	CD45RA+ CD28- (	2	199934892
ebi-a-GCST90001698	rs75903460	CD45RA+ CD28- (	2	162758257
ebi-a-GCST90001698	rs144528321	CD45RA+ CD28- (	2	156270948
ebi-a-GCST90001698	rs149876155	CD45RA+ CD28- (	2	24167677
ebi-a-GCST90001698	rs140778797	CD45RA+ CD28- (	2	130811172
ebi-a-GCST90001698	rs115027982	CD45RA+ CD28- (	2	120693004
ebi-a-GCST90001698	rs7426047	CD45RA+ CD28- (	2	107184424
ebi-a-GCST90001698	rs112137766	CD45RA+ CD28- (	2	41540149
ebi-a-GCST90001698	rs77156603	CD45RA+ CD28- (	2	104590904
ebi-a-GCST90001698	rs151250363	CD45RA+ CD28- (	2	240644273
ebi-a-GCST90001698	rs35811413	CD45RA+ CD28- (	2	90262802
ebi-a-GCST90001698	rs147774047	CD45RA+ CD28- (	3	23633832
ebi-a-GCST90001698	rs7613821	CD45RA+ CD28- (	3	105611213
ebi-a-GCST90001698	rs62272737	CD45RA+ CD28- (	3	152065838
ebi-a-GCST90001698	rs16864501	CD45RA+ CD28- (	3	152385976
ebi-a-GCST90001698	rs75660425	CD45RA+ CD28- (	3	129232254
ebi-a-GCST90001698	rs189065333	CD45RA+ CD28- (	3	170926536
ebi-a-GCST90001698	rs112057764	CD45RA+ CD28- (	3	176439376
ebi-a-GCST90001698	rs146756366	CD45RA+ CD28- (	3	76660068
ebi-a-GCST90001698	rs184789822	CD45RA+ CD28- (	3	180444592
ebi-a-GCST90001698	rs115660429	CD45RA+ CD28- (	3	195531004
ebi-a-GCST90001698	rs116719160	CD45RA+ CD28- (	3	130201546
ebi-a-GCST90001698	rs11921864	CD45RA+ CD28- (	3	140104765
ebi-a-GCST90001698	rs79418578	CD45RA+ CD28- (	3	141886501
ebi-a-GCST90001698	rs78741146	CD45RA+ CD28- (	3	182409985
ebi-a-GCST90001698	rs138401052	CD45RA+ CD28- (	3	166256176
ebi-a-GCST90001698	rs75551427	CD45RA+ CD28- (	3	168644376
ebi-a-GCST90001698	rs115052567	CD45RA+ CD28- (	3	169651872
ebi-a-GCST90001698	rs148658406	CD45RA+ CD28- (	3	153311399
ebi-a-GCST90001698	rs75968195	CD45RA+ CD28- (	3	157462992
ebi-a-GCST90001698	rs116521668	CD45RA+ CD28- (	3	43905713
ebi-a-GCST90001698	rs139791317	CD45RA+ CD28- (	3	146662353
ebi-a-GCST90001698	rs116349768	CD45RA+ CD28- (	3	84476620

ebi-a-GCST90001698	rs140527299	CD45RA+ CD28- (	3	63604132
ebi-a-GCST90001698	rs115821332	CD45RA+ CD28- (	3	115341107
ebi-a-GCST90001698	rs185034429	CD45RA+ CD28- (	3	90216749
ebi-a-GCST90001698	rs55787444	CD45RA+ CD28- (	3	185766414
ebi-a-GCST90001698	rs78193714	CD45RA+ CD28- (	3	186195530
ebi-a-GCST90001698	rs111777292	CD45RA+ CD28- (	3	192716368
ebi-a-GCST90001698	rs62247421	CD45RA+ CD28- (	3	64390467
ebi-a-GCST90001698	rs115212782	CD45RA+ CD28- (	3	74527776
ebi-a-GCST90001698	rs77521823	CD45RA+ CD28- (	3	130074113
ebi-a-GCST90001698	rs116589647	CD45RA+ CD28- (	3	122070556
ebi-a-GCST90001698	rs75816947	CD45RA+ CD28- (	3	3753451
ebi-a-GCST90001698	rs111902471	CD45RA+ CD28- (	3	14459099
ebi-a-GCST90001698	rs115040341	CD45RA+ CD28- (	3	64543612
ebi-a-GCST90001698	rs17878699	CD45RA+ CD28- (	3	3132754
ebi-a-GCST90001698	rs111745963	CD45RA+ CD28- (	3	67860216
ebi-a-GCST90001698	rs115879183	CD45RA+ CD28- (	3	194347376
ebi-a-GCST90001698	rs138871895	CD45RA+ CD28- (	3	162179186
ebi-a-GCST90001698	rs78313301	CD45RA+ CD28- (	3	11075184
ebi-a-GCST90001698	rs187990734	CD45RA+ CD28- (	3	144835290
ebi-a-GCST90001698	rs184977394	CD45RA+ CD28- (	3	81799701
ebi-a-GCST90001698	rs146900894	CD45RA+ CD28- (	3	94221769
ebi-a-GCST90001698	rs185587352	CD45RA+ CD28- (	3	117384981
ebi-a-GCST90001698	rs150027262	CD45RA+ CD28- (	3	72800530
ebi-a-GCST90001698	rs115135290	CD45RA+ CD28- (	3	105859785
ebi-a-GCST90001698	rs142609156	CD45RA+ CD28- (	4	11158267
ebi-a-GCST90001698	rs13104493	CD45RA+ CD28- (	4	32690871
ebi-a-GCST90001698	rs17476188	CD45RA+ CD28- (	4	165111111
ebi-a-GCST90001698	rs116399750	CD45RA+ CD28- (	4	176754667
ebi-a-GCST90001698	rs77814979	CD45RA+ CD28- (	4	131041099
ebi-a-GCST90001698	rs72660861	CD45RA+ CD28- (	4	85158464
ebi-a-GCST90001698	rs183356919	CD45RA+ CD28- (	4	9678634
ebi-a-GCST90001698	rs7663813	CD45RA+ CD28- (	4	57611830
ebi-a-GCST90001698	rs116536232	CD45RA+ CD28- (	4	67329531
ebi-a-GCST90001698	rs139819048	CD45RA+ CD28- (	4	67760555
ebi-a-GCST90001698	rs114619811	CD45RA+ CD28- (	4	71875684
ebi-a-GCST90001698	rs113555953	CD45RA+ CD28- (	4	97898706
ebi-a-GCST90001698	rs80331575	CD45RA+ CD28- (	4	109566866
ebi-a-GCST90001698	rs187204486	CD45RA+ CD28- (	4	116900766
ebi-a-GCST90001698	rs138943622	CD45RA+ CD28- (	4	114293931
ebi-a-GCST90001698	rs191331192	CD45RA+ CD28- (	4	160795044
ebi-a-GCST90001698	rs147026949	CD45RA+ CD28- (	4	100588891
ebi-a-GCST90001698	rs114559319	CD45RA+ CD28- (	4	85213802
ebi-a-GCST90001698	rs79395509	CD45RA+ CD28- (	4	176348299
ebi-a-GCST90001698	rs79888370	CD45RA+ CD28- (	4	180201786
ebi-a-GCST90001698	rs114421150	CD45RA+ CD28- (	4	16986930
ebi-a-GCST90001698	rs138954494	CD45RA+ CD28- (	4	32184694
ebi-a-GCST90001698	rs74718830	CD45RA+ CD28- (	4	35542069
ebi-a-GCST90001698	rs75993384	CD45RA+ CD28- (	4	55305871

ebi-a-GCST90001698	rs62321187	CD45RA+ CD28- (	4	120605817
ebi-a-GCST90001698	rs182176493	CD45RA+ CD28- (	4	83484083
ebi-a-GCST90001698	rs76679968	CD45RA+ CD28- (	4	129754843
ebi-a-GCST90001698	rs140367496	CD45RA+ CD28- (	4	131786765
ebi-a-GCST90001698	rs115307086	CD45RA+ CD28- (	4	131409763
ebi-a-GCST90001698	rs115677012	CD45RA+ CD28- (	4	167555345
ebi-a-GCST90001698	rs77689186	CD45RA+ CD28- (	4	176614955
ebi-a-GCST90001698	rs12499962	CD45RA+ CD28- (	4	99029457
ebi-a-GCST90001698	rs146374482	CD45RA+ CD28- (	4	85578601
ebi-a-GCST90001698	rs149844272	CD45RA+ CD28- (	4	87144762
ebi-a-GCST90001698	rs114685875	CD45RA+ CD28- (	4	138349095
ebi-a-GCST90001698	rs77434296	CD45RA+ CD28- (	4	22140311
ebi-a-GCST90001698	rs191607214	CD45RA+ CD28- (	4	123414413
ebi-a-GCST90001698	rs28622225	CD45RA+ CD28- (	4	166260369
ebi-a-GCST90001698	rs12501785	CD45RA+ CD28- (	4	180565400
ebi-a-GCST90001698	rs76763980	CD45RA+ CD28- (	4	151589256
ebi-a-GCST90001698	rs116498781	CD45RA+ CD28- (	4	183314518
ebi-a-GCST90001698	rs148590919	CD45RA+ CD28- (	4	187560601
ebi-a-GCST90001698	rs72723889	CD45RA+ CD28- (	4	146773005
ebi-a-GCST90001698	rs76825401	CD45RA+ CD28- (	4	84334746
ebi-a-GCST90001698	rs77146551	CD45RA+ CD28- (	4	108096534
ebi-a-GCST90001698	rs541823325	CD45RA+ CD28- (	4	49518806
ebi-a-GCST90001698	rs55975566	CD45RA+ CD28- (	4	3836469
ebi-a-GCST90001698	rs537461274	CD45RA+ CD28- (	5	119939807
ebi-a-GCST90001698	rs79725470	CD45RA+ CD28- (	5	94705605
ebi-a-GCST90001698	rs148816938	CD45RA+ CD28- (	5	71107850
ebi-a-GCST90001698	rs58243030	CD45RA+ CD28- (	5	180495180
ebi-a-GCST90001698	rs528672922	CD45RA+ CD28- (	5	79202265
ebi-a-GCST90001698	rs78676406	CD45RA+ CD28- (	5	118494935
ebi-a-GCST90001698	rs146314225	CD45RA+ CD28- (	5	141469416
ebi-a-GCST90001698	rs3822406	CD45RA+ CD28- (	5	180054853
ebi-a-GCST90001698	rs113960013	CD45RA+ CD28- (	5	178662302
ebi-a-GCST90001698	rs112357188	CD45RA+ CD28- (	5	16118218
ebi-a-GCST90001698	rs149898247	CD45RA+ CD28- (	5	129034231
ebi-a-GCST90001698	rs142922198	CD45RA+ CD28- (	5	145713781
ebi-a-GCST90001698	rs13360609	CD45RA+ CD28- (	5	180502672
ebi-a-GCST90001698	rs189369117	CD45RA+ CD28- (	5	142967016
ebi-a-GCST90001698	rs17642570	CD45RA+ CD28- (	5	148699825
ebi-a-GCST90001698	rs79194156	CD45RA+ CD28- (	5	145551085
ebi-a-GCST90001698	rs144613377	CD45RA+ CD28- (	5	81311763
ebi-a-GCST90001698	rs180927720	CD45RA+ CD28- (	5	11287269
ebi-a-GCST90001698	rs185483934	CD45RA+ CD28- (	5	16704585
ebi-a-GCST90001698	rs192556004	CD45RA+ CD28- (	5	135639453
ebi-a-GCST90001698	rs189412340	CD45RA+ CD28- (	5	75564943
ebi-a-GCST90001698	rs138800755	CD45RA+ CD28- (	5	77962505
ebi-a-GCST90001698	rs113884990	CD45RA+ CD28- (	5	96997109
ebi-a-GCST90001698	rs139800400	CD45RA+ CD28- (	5	134343662
ebi-a-GCST90001698	rs116581718	CD45RA+ CD28- (	5	29413612

ebi-a-GCST90001698	rs114871166	CD45RA+ CD28- (	5	24165437
ebi-a-GCST90001698	rs114425563	CD45RA+ CD28- (	5	176911575
ebi-a-GCST90001698	rs148939501	CD45RA+ CD28- (	5	12480500
ebi-a-GCST90001698	rs76726998	CD45RA+ CD28- (	5	93889241
ebi-a-GCST90001698	rs62337106	CD45RA+ CD28- (	5	1957403
ebi-a-GCST90001698	rs144585837	CD45RA+ CD28- (	5	120363630
ebi-a-GCST90001698	rs8177454	CD45RA+ CD28- (	5	150408639
ebi-a-GCST90001698	rs146135726	CD45RA+ CD28- (	5	154015729
ebi-a-GCST90001698	rs148291161	CD45RA+ CD28- (	5	168556910
ebi-a-GCST90001698	rs77587134	CD45RA+ CD28- (	5	3635021
ebi-a-GCST90001698	rs75760272	CD45RA+ CD28- (	5	10850589
ebi-a-GCST90001698	rs115755474	CD45RA+ CD28- (	5	168914162
ebi-a-GCST90001698	rs62406757	CD45RA+ CD28- (	5	180423556
ebi-a-GCST90001698	rs114245333	CD45RA+ CD28- (	5	112619226
ebi-a-GCST90001698	rs112973846	CD45RA+ CD28- (	5	128365521
ebi-a-GCST90001698	rs150287087	CD45RA+ CD28- (	6	125051261
ebi-a-GCST90001698	rs141389929	CD45RA+ CD28- (	6	142716026
ebi-a-GCST90001698	rs60537788	CD45RA+ CD28- (	6	897475
ebi-a-GCST90001698	rs62388146	CD45RA+ CD28- (	6	7624482
ebi-a-GCST90001698	rs149801155	CD45RA+ CD28- (	6	162457335
ebi-a-GCST90001698	rs138327786	CD45RA+ CD28- (	6	1730982
ebi-a-GCST90001698	rs117822024	CD45RA+ CD28- (	6	86346609
ebi-a-GCST90001698	rs149136965	CD45RA+ CD28- (	6	30958409
ebi-a-GCST90001698	rs73412501	CD45RA+ CD28- (	6	39011539
ebi-a-GCST90001698	rs9479093	CD45RA+ CD28- (	6	151992984
ebi-a-GCST90001698	rs140087548	CD45RA+ CD28- (	6	168136303
ebi-a-GCST90001698	rs73780868	CD45RA+ CD28- (	6	152137377
ebi-a-GCST90001698	rs116431337	CD45RA+ CD28- (	6	66416572
ebi-a-GCST90001698	rs6916896	CD45RA+ CD28- (	6	5734886
ebi-a-GCST90001698	rs180745907	CD45RA+ CD28- (	6	20965785
ebi-a-GCST90001698	rs145336406	CD45RA+ CD28- (	6	78745175
ebi-a-GCST90001698	rs79594290	CD45RA+ CD28- (	6	32066320
ebi-a-GCST90001698	rs78275221	CD45RA+ CD28- (	6	90900544
ebi-a-GCST90001698	rs62424171	CD45RA+ CD28- (	6	118769058
ebi-a-GCST90001698	rs114599999	CD45RA+ CD28- (	6	52376254
ebi-a-GCST90001698	rs2496150	CD45RA+ CD28- (	6	13374132
ebi-a-GCST90001698	rs117185099	CD45RA+ CD28- (	6	119514715
ebi-a-GCST90001698	rs10155782	CD45RA+ CD28- (	6	37156467
ebi-a-GCST90001698	rs75424381	CD45RA+ CD28- (	6	11805124
ebi-a-GCST90001698	rs111867376	CD45RA+ CD28- (	6	159962229
ebi-a-GCST90001698	rs149208831	CD45RA+ CD28- (	6	10017676
ebi-a-GCST90001698	rs145604620	CD45RA+ CD28- (	6	11491250
ebi-a-GCST90001698	rs75510626	CD45RA+ CD28- (	6	64401131
ebi-a-GCST90001698	rs62397109	CD45RA+ CD28- (	6	53785189
ebi-a-GCST90001698	rs79681496	CD45RA+ CD28- (	6	46004728
ebi-a-GCST90001698	rs140414198	CD45RA+ CD28- (	6	151971790
ebi-a-GCST90001698	rs149828559	CD45RA+ CD28- (	6	84898123
ebi-a-GCST90001698	rs117248086	CD45RA+ CD28- (	6	119632972

ebi-a-GCST90001698	rs75146109	CD45RA+ CD28- (	6	138346038
ebi-a-GCST90001698	rs79518488	CD45RA+ CD28- (	6	28389143
ebi-a-GCST90001698	rs35756014	CD45RA+ CD28- (	6	106606287
ebi-a-GCST90001698	rs12332976	CD45RA+ CD28- (	6	40616989
ebi-a-GCST90001698	rs12192395	CD45RA+ CD28- (	6	70784408
ebi-a-GCST90001698	rs144234028	CD45RA+ CD28- (	6	53258504
ebi-a-GCST90001698	rs192397067	CD45RA+ CD28- (	6	81984275
ebi-a-GCST90001698	rs9386578	CD45RA+ CD28- (	6	107128051
ebi-a-GCST90001698	rs112880431	CD45RA+ CD28- (	6	130091902
ebi-a-GCST90001698	rs142978760	CD45RA+ CD28- (	6	6047112
ebi-a-GCST90001698	rs189131343	CD45RA+ CD28- (	6	167014418
ebi-a-GCST90001698	rs9471016	CD45RA+ CD28- (	6	39196392
ebi-a-GCST90001698	rs80018878	CD45RA+ CD28- (	7	46985321
ebi-a-GCST90001698	rs143845801	CD45RA+ CD28- (	7	84434549
ebi-a-GCST90001698	rs137997289	CD45RA+ CD28- (	7	115706613
ebi-a-GCST90001698	rs149355173	CD45RA+ CD28- (	7	91861162
ebi-a-GCST90001698	rs74405982	CD45RA+ CD28- (	7	103435268
ebi-a-GCST90001698	rs75300104	CD45RA+ CD28- (	7	143729951
ebi-a-GCST90001698	rs191669640	CD45RA+ CD28- (	7	154881197
ebi-a-GCST90001698	rs77465871	CD45RA+ CD28- (	7	47504039
ebi-a-GCST90001698	rs115550848	CD45RA+ CD28- (	7	157555802
ebi-a-GCST90001698	rs17501774	CD45RA+ CD28- (	7	81422404
ebi-a-GCST90001698	rs117944233	CD45RA+ CD28- (	7	137569181
ebi-a-GCST90001698	rs146204142	CD45RA+ CD28- (	7	112317649
ebi-a-GCST90001698	rs142537480	CD45RA+ CD28- (	7	52813883
ebi-a-GCST90001698	rs35730839	CD45RA+ CD28- (	7	109248384
ebi-a-GCST90001698	rs80305889	CD45RA+ CD28- (	7	34681601
ebi-a-GCST90001698	rs117433099	CD45RA+ CD28- (	7	6934892
ebi-a-GCST90001698	rs147681963	CD45RA+ CD28- (	7	37590076
ebi-a-GCST90001698	rs10276305	CD45RA+ CD28- (	7	7885705
ebi-a-GCST90001698	rs77646481	CD45RA+ CD28- (	7	113821794
ebi-a-GCST90001698	rs180795781	CD45RA+ CD28- (	7	46290614
ebi-a-GCST90001698	rs142302016	CD45RA+ CD28- (	7	54517004
ebi-a-GCST90001698	rs554574739	CD45RA+ CD28- (	7	101754392
ebi-a-GCST90001698	rs117469911	CD45RA+ CD28- (	7	79791344
ebi-a-GCST90001698	rs73116391	CD45RA+ CD28- (	7	46417626
ebi-a-GCST90001698	rs118044214	CD45RA+ CD28- (	7	147886758
ebi-a-GCST90001698	rs115221944	CD45RA+ CD28- (	7	56724250
ebi-a-GCST90001698	rs545493508	CD45RA+ CD28- (	7	78599311
ebi-a-GCST90001698	rs74842782	CD45RA+ CD28- (	7	131413341
ebi-a-GCST90001698	rs78470553	CD45RA+ CD28- (	7	158334355
ebi-a-GCST90001698	rs184401135	CD45RA+ CD28- (	7	83368658
ebi-a-GCST90001698	rs146975368	CD45RA+ CD28- (	7	5907599
ebi-a-GCST90001698	rs145057921	CD45RA+ CD28- (	7	6231210
ebi-a-GCST90001698	rs186251573	CD45RA+ CD28- (	7	13558580
ebi-a-GCST90001698	rs138449036	CD45RA+ CD28- (	7	29025284
ebi-a-GCST90001698	rs79647440	CD45RA+ CD28- (	7	142438302
ebi-a-GCST90001698	rs559989071	CD45RA+ CD28- (	7	141814031

ebi-a-GCST90001698	rs35706199	CD45RA+ CD28- (	7	131191874
ebi-a-GCST90001698	rs118124593	CD45RA+ CD28- (	7	134350922
ebi-a-GCST90001698	rs150400236	CD45RA+ CD28- (	7	136855654
ebi-a-GCST90001698	rs28914837	CD45RA+ CD28- (	7	7759826
ebi-a-GCST90001698	rs117079032	CD45RA+ CD28- (	7	69510130
ebi-a-GCST90001698	rs185754925	CD45RA+ CD28- (	7	62917010
ebi-a-GCST90001698	rs144872818	CD45RA+ CD28- (	7	49894195
ebi-a-GCST90001698	rs111399817	CD45RA+ CD28- (	7	146550168
ebi-a-GCST90001698	rs113677798	CD45RA+ CD28- (	7	78657809
ebi-a-GCST90001698	rs149168806	CD45RA+ CD28- (	7	78834873
ebi-a-GCST90001698	rs73206236	CD45RA+ CD28- (	7	113679246
ebi-a-GCST90001698	rs116921833	CD45RA+ CD28- (	7	153895185
ebi-a-GCST90001698	rs142111513	CD45RA+ CD28- (	8	23682802
ebi-a-GCST90001698	rs8178689	CD45RA+ CD28- (	8	42060072
ebi-a-GCST90001698	rs117339150	CD45RA+ CD28- (	8	52563134
ebi-a-GCST90001698	rs77370289	CD45RA+ CD28- (	8	57917248
ebi-a-GCST90001698	rs186888992	CD45RA+ CD28- (	8	81419619
ebi-a-GCST90001698	rs34039708	CD45RA+ CD28- (	8	12500832
ebi-a-GCST90001698	rs72727978	CD45RA+ CD28- (	8	134999971
ebi-a-GCST90001698	rs113122796	CD45RA+ CD28- (	8	142876352
ebi-a-GCST90001698	rs181660070	CD45RA+ CD28- (	8	117040660
ebi-a-GCST90001698	rs117389556	CD45RA+ CD28- (	8	61340602
ebi-a-GCST90001698	rs117609491	CD45RA+ CD28- (	8	106840842
ebi-a-GCST90001698	rs117741105	CD45RA+ CD28- (	8	112234506
ebi-a-GCST90001698	rs117127715	CD45RA+ CD28- (	8	31315428
ebi-a-GCST90001698	rs117626443	CD45RA+ CD28- (	8	69864162
ebi-a-GCST90001698	rs555447585	CD45RA+ CD28- (	8	138817238
ebi-a-GCST90001698	rs79851411	CD45RA+ CD28- (	8	13716390
ebi-a-GCST90001698	rs35376347	CD45RA+ CD28- (	8	99217391
ebi-a-GCST90001698	rs1368539	CD45RA+ CD28- (	8	16526793
ebi-a-GCST90001698	rs75361870	CD45RA+ CD28- (	8	9202609
ebi-a-GCST90001698	rs79580432	CD45RA+ CD28- (	8	4778180
ebi-a-GCST90001698	rs34000508	CD45RA+ CD28- (	8	25574426
ebi-a-GCST90001698	rs140129399	CD45RA+ CD28- (	8	1322274
ebi-a-GCST90001698	rs139584477	CD45RA+ CD28- (	8	57056704
ebi-a-GCST90001698	rs117685161	CD45RA+ CD28- (	8	99344753
ebi-a-GCST90001698	rs150058365	CD45RA+ CD28- (	8	89800477
ebi-a-GCST90001698	rs147959386	CD45RA+ CD28- (	8	124197307
ebi-a-GCST90001698	rs13249758	CD45RA+ CD28- (	8	128271231
ebi-a-GCST90001698	rs117123362	CD45RA+ CD28- (	8	129915383
ebi-a-GCST90001698	rs149627909	CD45RA+ CD28- (	8	28381797
ebi-a-GCST90001698	rs117440863	CD45RA+ CD28- (	8	98513319
ebi-a-GCST90001698	rs62513341	CD45RA+ CD28- (	8	116858342
ebi-a-GCST90001698	rs117983267	CD45RA+ CD28- (	8	124174485
ebi-a-GCST90001698	rs79218577	CD45RA+ CD28- (	8	143859934
ebi-a-GCST90001698	rs183280627	CD45RA+ CD28- (	8	30627573
ebi-a-GCST90001698	rs117808048	CD45RA+ CD28- (	8	73079210
ebi-a-GCST90001698	rs144258173	CD45RA+ CD28- (	8	2859400

ebi-a-GCST90001698	rs74416723	CD45RA+ CD28- (	8	6769704
ebi-a-GCST90001698	rs144582155	CD45RA+ CD28- (	8	41997183
ebi-a-GCST90001698	rs77009235	CD45RA+ CD28- (	8	4499026
ebi-a-GCST90001698	rs117219726	CD45RA+ CD28- (	8	33264182
ebi-a-GCST90001698	rs76230052	CD45RA+ CD28- (	8	131835695
ebi-a-GCST90001698	rs118076041	CD45RA+ CD28- (	8	144562245
ebi-a-GCST90001698	rs117448261	CD45RA+ CD28- (	8	82400020
ebi-a-GCST90001698	rs45440296	CD45RA+ CD28- (	9	133917168
ebi-a-GCST90001698	rs151035277	CD45RA+ CD28- (	9	25887570
ebi-a-GCST90001698	rs374964617	CD45RA+ CD28- (	9	504491
ebi-a-GCST90001698	rs144206851	CD45RA+ CD28- (	9	77966779
ebi-a-GCST90001698	rs145563511	CD45RA+ CD28- (	9	106801353
ebi-a-GCST90001698	rs140899856	CD45RA+ CD28- (	9	111194567
ebi-a-GCST90001698	rs77733172	CD45RA+ CD28- (	9	140235501
ebi-a-GCST90001698	rs76543451	CD45RA+ CD28- (	9	122201008
ebi-a-GCST90001698	rs148380088	CD45RA+ CD28- (	9	28759554
ebi-a-GCST90001698	rs148602493	CD45RA+ CD28- (	9	23576735
ebi-a-GCST90001698	rs74336249	CD45RA+ CD28- (	9	135625608
ebi-a-GCST90001698	rs148835086	CD45RA+ CD28- (	9	10278984
ebi-a-GCST90001698	rs76017116	CD45RA+ CD28- (	9	107332377
ebi-a-GCST90001698	rs79727415	CD45RA+ CD28- (	9	121837015
ebi-a-GCST90001698	rs559632285	CD45RA+ CD28- (	9	45362596
ebi-a-GCST90001698	rs117702997	CD45RA+ CD28- (	9	83435830
ebi-a-GCST90001698	rs150336475	CD45RA+ CD28- (	9	111627658
ebi-a-GCST90001698	rs10965305	CD45RA+ CD28- (	9	22268719
ebi-a-GCST90001698	rs34609592	CD45RA+ CD28- (	9	136915695
ebi-a-GCST90001698	rs111373539	CD45RA+ CD28- (	9	107628892
ebi-a-GCST90001698	rs148629497	CD45RA+ CD28- (	9	10593877
ebi-a-GCST90001698	rs62567746	CD45RA+ CD28- (	9	38796687
ebi-a-GCST90001698	rs72704856	CD45RA+ CD28- (	9	10774031
ebi-a-GCST90001698	rs118062750	CD45RA+ CD28- (	9	29889453
ebi-a-GCST90001698	rs7850783	CD45RA+ CD28- (	9	100968226
ebi-a-GCST90001698	rs149649979	CD45RA+ CD28- (	9	10889621
ebi-a-GCST90001698	rs75489053	CD45RA+ CD28- (	9	22449820
ebi-a-GCST90001698	rs12378893	CD45RA+ CD28- (	9	79057643
ebi-a-GCST90001698	rs562356749	CD45RA+ CD28- (	9	67316488
ebi-a-GCST90001698	rs188376248	CD45RA+ CD28- (	9	102390885
ebi-a-GCST90001698	rs10122523	CD45RA+ CD28- (	9	99511751
ebi-a-GCST90001698	rs72768736	CD45RA+ CD28- (	9	137149408
ebi-a-GCST90001698	rs139145617	CD45RA+ CD28- (	9	138652160
ebi-a-GCST90001698	rs13293817	CD45RA+ CD28- (	9	138791163
ebi-a-GCST90001698	rs140322068	CD45RA+ CD28- (	10	1765793
ebi-a-GCST90001698	rs72810820	CD45RA+ CD28- (	10	92600913
ebi-a-GCST90001698	rs10159645	CD45RA+ CD28- (	10	106333320
ebi-a-GCST90001698	rs145835734	CD45RA+ CD28- (	10	66659421
ebi-a-GCST90001698	rs78622624	CD45RA+ CD28- (	10	33359425
ebi-a-GCST90001698	rs139781702	CD45RA+ CD28- (	10	38359540
ebi-a-GCST90001698	rs2816832	CD45RA+ CD28- (	10	52735936

ebi-a-GCST90001698	rs138948609	CD45RA+ CD28- (	10	132596864
ebi-a-GCST90001698	rs2384564	CD45RA+ CD28- (	10	36442592
ebi-a-GCST90001698	rs191325818	CD45RA+ CD28- (	10	120573449
ebi-a-GCST90001698	rs143395500	CD45RA+ CD28- (	10	69536834
ebi-a-GCST90001698	rs184899141	CD45RA+ CD28- (	10	82437388
ebi-a-GCST90001698	rs77390603	CD45RA+ CD28- (	10	4688192
ebi-a-GCST90001698	rs145743664	CD45RA+ CD28- (	10	16995517
ebi-a-GCST90001698	rs112517700	CD45RA+ CD28- (	10	91013835
ebi-a-GCST90001698	rs143772909	CD45RA+ CD28- (	10	101494118
ebi-a-GCST90001698	rs117003718	CD45RA+ CD28- (	10	124406240
ebi-a-GCST90001698	rs149860203	CD45RA+ CD28- (	10	31026572
ebi-a-GCST90001698	rs10824158	CD45RA+ CD28- (	10	53915868
ebi-a-GCST90001698	rs78793273	CD45RA+ CD28- (	10	55118190
ebi-a-GCST90001698	rs117098395	CD45RA+ CD28- (	10	107595273
ebi-a-GCST90001698	rs145021762	CD45RA+ CD28- (	10	22411454
ebi-a-GCST90001698	rs139445618	CD45RA+ CD28- (	10	24055451
ebi-a-GCST90001698	rs148109962	CD45RA+ CD28- (	10	17396637
ebi-a-GCST90001698	rs147968748	CD45RA+ CD28- (	10	54704473
ebi-a-GCST90001698	rs11202534	CD45RA+ CD28- (	10	89500755
ebi-a-GCST90001698	rs74157741	CD45RA+ CD28- (	10	121973769
ebi-a-GCST90001698	rs72780729	CD45RA+ CD28- (	10	17605945
ebi-a-GCST90001698	rs11596188	CD45RA+ CD28- (	10	1734511
ebi-a-GCST90001698	rs118084393	CD45RA+ CD28- (	10	6554827
ebi-a-GCST90001698	rs74322543	CD45RA+ CD28- (	10	85916001
ebi-a-GCST90001698	rs575794055	CD45RA+ CD28- (	10	119966169
ebi-a-GCST90001698	rs74853222	CD45RA+ CD28- (	10	133380798
ebi-a-GCST90001698	rs147563624	CD45RA+ CD28- (	10	5377215
ebi-a-GCST90001698	rs76385522	CD45RA+ CD28- (	11	10620991
ebi-a-GCST90001698	rs78922116	CD45RA+ CD28- (	11	25534610
ebi-a-GCST90001698	rs78524989	CD45RA+ CD28- (	11	94284134
ebi-a-GCST90001698	rs7102247	CD45RA+ CD28- (	11	133304292
ebi-a-GCST90001698	rs78085398	CD45RA+ CD28- (	11	33121166
ebi-a-GCST90001698	rs145665517	CD45RA+ CD28- (	11	75103559
ebi-a-GCST90001698	rs192346792	CD45RA+ CD28- (	11	74172698
ebi-a-GCST90001698	rs186370342	CD45RA+ CD28- (	11	92081132
ebi-a-GCST90001698	rs1106305	CD45RA+ CD28- (	11	12065087
ebi-a-GCST90001698	rs113653707	CD45RA+ CD28- (	11	43996684
ebi-a-GCST90001698	rs117270364	CD45RA+ CD28- (	11	60694375
ebi-a-GCST90001698	rs142869493	CD45RA+ CD28- (	11	87291175
ebi-a-GCST90001698	rs61759531	CD45RA+ CD28- (	11	130773279
ebi-a-GCST90001698	rs117467460	CD45RA+ CD28- (	11	99466406
ebi-a-GCST90001698	rs75157411	CD45RA+ CD28- (	11	95180478
ebi-a-GCST90001698	rs117284243	CD45RA+ CD28- (	11	122918979
ebi-a-GCST90001698	rs78881177	CD45RA+ CD28- (	11	127881478
ebi-a-GCST90001698	rs138259561	CD45RA+ CD28- (	11	76470121
ebi-a-GCST90001698	rs7130130	CD45RA+ CD28- (	11	59336329
ebi-a-GCST90001698	rs146252263	CD45RA+ CD28- (	11	2477438
ebi-a-GCST90001698	rs118014538	CD45RA+ CD28- (	11	3830358

ebi-a-GCST90001698	rs74540476	CD45RA+ CD28- (	11	29902098
ebi-a-GCST90001698	rs77695406	CD45RA+ CD28- (	11	70599488
ebi-a-GCST90001698	rs79741386	CD45RA+ CD28- (	11	109660227
ebi-a-GCST90001698	rs75093728	CD45RA+ CD28- (	11	123053955
ebi-a-GCST90001698	rs189796120	CD45RA+ CD28- (	11	109281106
ebi-a-GCST90001698	rs117420444	CD45RA+ CD28- (	11	31631366
ebi-a-GCST90001698	rs12279408	CD45RA+ CD28- (	11	84269972
ebi-a-GCST90001698	rs76552842	CD45RA+ CD28- (	11	83433494
ebi-a-GCST90001698	rs201928503	CD45RA+ CD28- (	11	43037421
ebi-a-GCST90001698	rs117573224	CD45RA+ CD28- (	11	1149087
ebi-a-GCST90001698	rs35733103	CD45RA+ CD28- (	11	3114190
ebi-a-GCST90001698	rs116916598	CD45RA+ CD28- (	11	121746998
ebi-a-GCST90001698	rs77485746	CD45RA+ CD28- (	11	129187484
ebi-a-GCST90001698	rs11060236	CD45RA+ CD28- (	12	129773605
ebi-a-GCST90001698	rs77577050	CD45RA+ CD28- (	12	116077199
ebi-a-GCST90001698	rs146822590	CD45RA+ CD28- (	12	130060949
ebi-a-GCST90001698	rs144682449	CD45RA+ CD28- (	12	53402638
ebi-a-GCST90001698	rs58817739	CD45RA+ CD28- (	12	46009960
ebi-a-GCST90001698	rs117785622	CD45RA+ CD28- (	12	132283582
ebi-a-GCST90001698	rs11112760	CD45RA+ CD28- (	12	106241673
ebi-a-GCST90001698	rs137943198	CD45RA+ CD28- (	12	10963475
ebi-a-GCST90001698	rs73115326	CD45RA+ CD28- (	12	60257747
ebi-a-GCST90001698	rs77364757	CD45RA+ CD28- (	12	88300742
ebi-a-GCST90001698	rs149037944	CD45RA+ CD28- (	12	103661467
ebi-a-GCST90001698	rs143604762	CD45RA+ CD28- (	12	131000132
ebi-a-GCST90001698	rs139075636	CD45RA+ CD28- (	12	102082782
ebi-a-GCST90001698	rs139898536	CD45RA+ CD28- (	12	109036912
ebi-a-GCST90001698	rs75013096	CD45RA+ CD28- (	12	44084962
ebi-a-GCST90001698	rs10505739	CD45RA+ CD28- (	12	10043518
ebi-a-GCST90001698	rs188790487	CD45RA+ CD28- (	12	43386402
ebi-a-GCST90001698	rs188074116	CD45RA+ CD28- (	12	19311236
ebi-a-GCST90001698	rs117004916	CD45RA+ CD28- (	12	114748976
ebi-a-GCST90001698	rs112529428	CD45RA+ CD28- (	12	129340399
ebi-a-GCST90001698	rs141976683	CD45RA+ CD28- (	12	71790032
ebi-a-GCST90001698	rs17042703	CD45RA+ CD28- (	12	77392215
ebi-a-GCST90001698	rs71466029	CD45RA+ CD28- (	12	82065434
ebi-a-GCST90001698	rs78773852	CD45RA+ CD28- (	12	71181930
ebi-a-GCST90001698	rs184910025	CD45RA+ CD28- (	12	117089403
ebi-a-GCST90001698	rs112121527	CD45RA+ CD28- (	12	119037578
ebi-a-GCST90001698	rs147107195	CD45RA+ CD28- (	12	48190275
ebi-a-GCST90001698	rs35275693	CD45RA+ CD28- (	12	88561479
ebi-a-GCST90001698	rs117613430	CD45RA+ CD28- (	12	93540934
ebi-a-GCST90001698	rs142686680	CD45RA+ CD28- (	12	29095512
ebi-a-GCST90001698	rs117293367	CD45RA+ CD28- (	12	52437438
ebi-a-GCST90001698	rs149313844	CD45RA+ CD28- (	12	4800867
ebi-a-GCST90001698	rs1381033	CD45RA+ CD28- (	12	15618105
ebi-a-GCST90001698	rs140053174	CD45RA+ CD28- (	12	71043237
ebi-a-GCST90001698	rs112004249	CD45RA+ CD28- (	12	82550260

ebi-a-GCST90001698	rs146661200	CD45RA+ CD28- (	12	100494123
ebi-a-GCST90001698	rs143435133	CD45RA+ CD28- (	12	129545747
ebi-a-GCST90001698	rs75391939	CD45RA+ CD28- (	12	133446245
ebi-a-GCST90001698	rs140524161	CD45RA+ CD28- (	13	37301576
ebi-a-GCST90001698	rs75550713	CD45RA+ CD28- (	13	112088993
ebi-a-GCST90001698	rs3848018	CD45RA+ CD28- (	13	99034552
ebi-a-GCST90001698	rs117075995	CD45RA+ CD28- (	13	19869255
ebi-a-GCST90001698	rs76911919	CD45RA+ CD28- (	13	112714493
ebi-a-GCST90001698	rs80133207	CD45RA+ CD28- (	13	113264513
ebi-a-GCST90001698	rs8002354	CD45RA+ CD28- (	13	109769788
ebi-a-GCST90001698	rs145720280	CD45RA+ CD28- (	13	43404037
ebi-a-GCST90001698	rs111648746	CD45RA+ CD28- (	13	28485345
ebi-a-GCST90001698	rs77214871	CD45RA+ CD28- (	13	84005540
ebi-a-GCST90001698	rs75592465	CD45RA+ CD28- (	13	105831838
ebi-a-GCST90001698	rs80128954	CD45RA+ CD28- (	13	76775102
ebi-a-GCST90001698	rs188618717	CD45RA+ CD28- (	13	114441229
ebi-a-GCST90001698	rs150476409	CD45RA+ CD28- (	13	113289664
ebi-a-GCST90001698	rs7985427	CD45RA+ CD28- (	13	47807626
ebi-a-GCST90001698	rs9584374	CD45RA+ CD28- (	13	97185631
ebi-a-GCST90001698	rs186400376	CD45RA+ CD28- (	13	31805158
ebi-a-GCST90001698	rs113007178	CD45RA+ CD28- (	13	40914557
ebi-a-GCST90001698	rs112111433	CD45RA+ CD28- (	13	85569939
ebi-a-GCST90001698	rs76256154	CD45RA+ CD28- (	13	95650711
ebi-a-GCST90001698	rs7139515	CD45RA+ CD28- (	13	96254406
ebi-a-GCST90001698	rs117177255	CD45RA+ CD28- (	13	77081029
ebi-a-GCST90001698	rs147848662	CD45RA+ CD28- (	13	112802184
ebi-a-GCST90001698	rs149582701	CD45RA+ CD28- (	13	62438680
ebi-a-GCST90001698	rs117304580	CD45RA+ CD28- (	14	72774395
ebi-a-GCST90001698	rs1381623	CD45RA+ CD28- (	14	84609045
ebi-a-GCST90001698	rs74565504	CD45RA+ CD28- (	14	89207147
ebi-a-GCST90001698	rs113699161	CD45RA+ CD28- (	14	58571589
ebi-a-GCST90001698	rs141481912	CD45RA+ CD28- (	14	97924775
ebi-a-GCST90001698	rs80334718	CD45RA+ CD28- (	14	33998519
ebi-a-GCST90001698	rs141309221	CD45RA+ CD28- (	14	95018285
ebi-a-GCST90001698	rs150889386	CD45RA+ CD28- (	14	100822892
ebi-a-GCST90001698	rs144275943	CD45RA+ CD28- (	14	34065832
ebi-a-GCST90001698	rs71419987	CD45RA+ CD28- (	14	34919717
ebi-a-GCST90001698	rs61755651	CD45RA+ CD28- (	14	72431599
ebi-a-GCST90001698	rs142461733	CD45RA+ CD28- (	14	22723226
ebi-a-GCST90001698	rs111730307	CD45RA+ CD28- (	14	49236170
ebi-a-GCST90001698	rs10400782	CD45RA+ CD28- (	14	35130401
ebi-a-GCST90001698	rs77191330	CD45RA+ CD28- (	14	79365684
ebi-a-GCST90001698	rs149134258	CD45RA+ CD28- (	14	93148478
ebi-a-GCST90001698	rs76545164	CD45RA+ CD28- (	14	36027615
ebi-a-GCST90001698	rs146937045	CD45RA+ CD28- (	14	46740502
ebi-a-GCST90001698	rs187775632	CD45RA+ CD28- (	14	76210029
ebi-a-GCST90001698	rs192467351	CD45RA+ CD28- (	14	76826605
ebi-a-GCST90001698	rs116853391	CD45RA+ CD28- (	14	104409277

ebi-a-GCST90001698	rs72694381	CD45RA+ CD28- (	14	24859553
ebi-a-GCST90001698	rs72672939	CD45RA+ CD28- (	14	45753508
ebi-a-GCST90001698	rs150055480	CD45RA+ CD28- (	14	55295865
ebi-a-GCST90001698	rs184184520	CD45RA+ CD28- (	14	45055780
ebi-a-GCST90001698	rs10484068	CD45RA+ CD28- (	14	99653123
ebi-a-GCST90001698	rs138183141	CD45RA+ CD28- (	14	101668310
ebi-a-GCST90001698	rs117862145	CD45RA+ CD28- (	14	33586273
ebi-a-GCST90001698	rs183436787	CD45RA+ CD28- (	14	41636680
ebi-a-GCST90001698	rs80331554	CD45RA+ CD28- (	15	59013643
ebi-a-GCST90001698	rs146982369	CD45RA+ CD28- (	15	25983333
ebi-a-GCST90001698	rs188087675	CD45RA+ CD28- (	15	29114742
ebi-a-GCST90001698	rs2441781	CD45RA+ CD28- (	15	52321843
ebi-a-GCST90001698	rs181228225	CD45RA+ CD28- (	15	84950824
ebi-a-GCST90001698	rs112310272	CD45RA+ CD28- (	15	26944526
ebi-a-GCST90001698	rs117801457	CD45RA+ CD28- (	15	29421113
ebi-a-GCST90001698	rs72750943	CD45RA+ CD28- (	15	63828258
ebi-a-GCST90001698	rs117294527	CD45RA+ CD28- (	15	100764857
ebi-a-GCST90001698	rs117397768	CD45RA+ CD28- (	15	68230533
ebi-a-GCST90001698	rs62009323	CD45RA+ CD28- (	15	96961294
ebi-a-GCST90001698	rs186824722	CD45RA+ CD28- (	15	24505612
ebi-a-GCST90001698	rs149438077	CD45RA+ CD28- (	15	30234373
ebi-a-GCST90001698	rs12442442	CD45RA+ CD28- (	15	40127028
ebi-a-GCST90001698	rs56041702	CD45RA+ CD28- (	15	50008133
ebi-a-GCST90001698	rs149908314	CD45RA+ CD28- (	15	54344919
ebi-a-GCST90001698	rs190589573	CD45RA+ CD28- (	15	79305514
ebi-a-GCST90001698	rs138020305	CD45RA+ CD28- (	15	95184966
ebi-a-GCST90001698	rs75669615	CD45RA+ CD28- (	15	39668760
ebi-a-GCST90001698	rs11633592	CD45RA+ CD28- (	15	25418271
ebi-a-GCST90001698	rs117211386	CD45RA+ CD28- (	15	51649599
ebi-a-GCST90001698	rs147590366	CD45RA+ CD28- (	15	63216446
ebi-a-GCST90001698	rs116922386	CD45RA+ CD28- (	15	68647510
ebi-a-GCST90001698	rs76066405	CD45RA+ CD28- (	16	78454375
ebi-a-GCST90001698	rs79548047	CD45RA+ CD28- (	16	11614838
ebi-a-GCST90001698	rs117555426	CD45RA+ CD28- (	16	13128176
ebi-a-GCST90001698	rs572954905	CD45RA+ CD28- (	16	31402773
ebi-a-GCST90001698	rs142015595	CD45RA+ CD28- (	16	83755289
ebi-a-GCST90001698	rs78888262	CD45RA+ CD28- (	16	88701572
ebi-a-GCST90001698	rs117291729	CD45RA+ CD28- (	16	11975137
ebi-a-GCST90001698	rs150007303	CD45RA+ CD28- (	16	23350716
ebi-a-GCST90001698	rs138937927	CD45RA+ CD28- (	16	51095561
ebi-a-GCST90001698	rs147054310	CD45RA+ CD28- (	16	85194229
ebi-a-GCST90001698	rs12934979	CD45RA+ CD28- (	16	87379050
ebi-a-GCST90001698	rs151246819	CD45RA+ CD28- (	16	23373955
ebi-a-GCST90001698	rs34265859	CD45RA+ CD28- (	16	25931790
ebi-a-GCST90001698	rs117898578	CD45RA+ CD28- (	16	50471284
ebi-a-GCST90001698	rs118065847	CD45RA+ CD28- (	16	54673316
ebi-a-GCST90001698	rs143946135	CD45RA+ CD28- (	16	69023689
ebi-a-GCST90001698	rs142076427	CD45RA+ CD28- (	16	5628078

ebi-a-GCST90001698	rs77145028	CD45RA+ CD28- (	16	54932285
ebi-a-GCST90001698	rs139120487	CD45RA+ CD28- (	16	755030
ebi-a-GCST90001698	rs142151887	CD45RA+ CD28- (	16	1071217
ebi-a-GCST90001698	rs138562176	CD45RA+ CD28- (	16	4207228
ebi-a-GCST90001698	rs187924827	CD45RA+ CD28- (	17	6329247
ebi-a-GCST90001698	rs147069965	CD45RA+ CD28- (	17	36231492
ebi-a-GCST90001698	rs117046665	CD45RA+ CD28- (	17	41774967
ebi-a-GCST90001698	rs55856613	CD45RA+ CD28- (	17	79094162
ebi-a-GCST90001698	rs150053624	CD45RA+ CD28- (	17	80299376
ebi-a-GCST90001698	rs56375820	CD45RA+ CD28- (	17	9999183
ebi-a-GCST90001698	rs118019384	CD45RA+ CD28- (	17	27826991
ebi-a-GCST90001698	rs143393137	CD45RA+ CD28- (	17	30967385
ebi-a-GCST90001698	rs144104650	CD45RA+ CD28- (	17	5023557
ebi-a-GCST90001698	rs78100215	CD45RA+ CD28- (	17	8298873
ebi-a-GCST90001698	rs117922931	CD45RA+ CD28- (	17	30028367
ebi-a-GCST90001698	rs79757812	CD45RA+ CD28- (	17	65896989
ebi-a-GCST90001698	rs139736375	CD45RA+ CD28- (	17	67625638
ebi-a-GCST90001698	rs184152921	CD45RA+ CD28- (	17	79175857
ebi-a-GCST90001698	rs113411752	CD45RA+ CD28- (	17	35489333
ebi-a-GCST90001698	rs34539402	CD45RA+ CD28- (	17	39959627
ebi-a-GCST90001698	rs17882143	CD45RA+ CD28- (	17	41836082
ebi-a-GCST90001698	rs74465961	CD45RA+ CD28- (	17	15099614
ebi-a-GCST90001698	rs55658055	CD45RA+ CD28- (	17	17705424
ebi-a-GCST90001698	rs113199364	CD45RA+ CD28- (	17	75157900
ebi-a-GCST90001698	rs117830252	CD45RA+ CD28- (	17	11465523
ebi-a-GCST90001698	rs117979973	CD45RA+ CD28- (	17	76931475
ebi-a-GCST90001698	rs568349042	CD45RA+ CD28- (	17	80344923
ebi-a-GCST90001698	rs77725246	CD45RA+ CD28- (	17	55639212
ebi-a-GCST90001698	rs143393656	CD45RA+ CD28- (	17	63493154
ebi-a-GCST90001698	rs192484154	CD45RA+ CD28- (	17	47652035
ebi-a-GCST90001698	rs150156077	CD45RA+ CD28- (	18	4348487
ebi-a-GCST90001698	rs150635355	CD45RA+ CD28- (	18	38662297
ebi-a-GCST90001698	rs150994087	CD45RA+ CD28- (	18	56708792
ebi-a-GCST90001698	rs62098570	CD45RA+ CD28- (	18	60238886
ebi-a-GCST90001698	rs150905742	CD45RA+ CD28- (	18	70586990
ebi-a-GCST90001698	rs117745825	CD45RA+ CD28- (	18	3305228
ebi-a-GCST90001698	rs11661882	CD45RA+ CD28- (	18	70504468
ebi-a-GCST90001698	rs7235263	CD45RA+ CD28- (	18	74408679
ebi-a-GCST90001698	rs80063983	CD45RA+ CD28- (	18	75193391
ebi-a-GCST90001698	rs112278099	CD45RA+ CD28- (	18	5319739
ebi-a-GCST90001698	rs145978106	CD45RA+ CD28- (	18	7839215
ebi-a-GCST90001698	rs147015694	CD45RA+ CD28- (	18	2896235
ebi-a-GCST90001698	rs116990016	CD45RA+ CD28- (	18	28163696
ebi-a-GCST90001698	rs78606202	CD45RA+ CD28- (	18	46581667
ebi-a-GCST90001698	rs536434085	CD45RA+ CD28- (	18	63832285
ebi-a-GCST90001698	rs73966235	CD45RA+ CD28- (	18	65092573
ebi-a-GCST90001698	rs76808079	CD45RA+ CD28- (	18	22735939
ebi-a-GCST90001698	rs75438005	CD45RA+ CD28- (	18	26017285

ebi-a-GCST90001698	rs17665147	CD45RA+ CD28- (	18	19383778
ebi-a-GCST90001698	rs116940971	CD45RA+ CD28- (	18	38279589
ebi-a-GCST90001698	rs147136626	CD45RA+ CD28- (	18	73901698
ebi-a-GCST90001698	rs114857658	CD45RA+ CD28- (	19	21585596
ebi-a-GCST90001698	rs118089863	CD45RA+ CD28- (	19	24477916
ebi-a-GCST90001698	rs146989801	CD45RA+ CD28- (	19	45630360
ebi-a-GCST90001698	rs200198801	CD45RA+ CD28- (	19	46387941
ebi-a-GCST90001698	rs112604811	CD45RA+ CD28- (	19	46587710
ebi-a-GCST90001698	rs143517669	CD45RA+ CD28- (	19	868083
ebi-a-GCST90001698	rs137963524	CD45RA+ CD28- (	19	8899157
ebi-a-GCST90001698	rs77955766	CD45RA+ CD28- (	19	17307102
ebi-a-GCST90001698	rs147370318	CD45RA+ CD28- (	19	31892272
ebi-a-GCST90001698	rs143824026	CD45RA+ CD28- (	19	33797284
ebi-a-GCST90001698	rs11879282	CD45RA+ CD28- (	19	48790968
ebi-a-GCST90001698	rs140800343	CD45RA+ CD28- (	19	50001170
ebi-a-GCST90001698	rs57121931	CD45RA+ CD28- (	19	55017690
ebi-a-GCST90001698	rs143871291	CD45RA+ CD28- (	19	1191667
ebi-a-GCST90001698	rs146101423	CD45RA+ CD28- (	19	6753000
ebi-a-GCST90001698	rs146650864	CD45RA+ CD28- (	19	22454852
ebi-a-GCST90001698	rs7254864	CD45RA+ CD28- (	19	9246656
ebi-a-GCST90001698	rs76648333	CD45RA+ CD28- (	19	33873371
ebi-a-GCST90001698	rs78703636	CD45RA+ CD28- (	19	33953595
ebi-a-GCST90001698	rs59745713	CD45RA+ CD28- (	19	34075835
ebi-a-GCST90001698	rs145410896	CD45RA+ CD28- (	19	34111992
ebi-a-GCST90001698	rs73034498	CD45RA+ CD28- (	19	44937435
ebi-a-GCST90001698	rs147572731	CD45RA+ CD28- (	19	49416171
ebi-a-GCST90001698	rs180972485	CD45RA+ CD28- (	20	24457030
ebi-a-GCST90001698	rs137942933	CD45RA+ CD28- (	20	33104122
ebi-a-GCST90001698	rs76312917	CD45RA+ CD28- (	20	38969262
ebi-a-GCST90001698	rs6094100	CD45RA+ CD28- (	20	43755180
ebi-a-GCST90001698	rs138246394	CD45RA+ CD28- (	20	54228961
ebi-a-GCST90001698	rs13039776	CD45RA+ CD28- (	20	62915126
ebi-a-GCST90001698	rs41276984	CD45RA+ CD28- (	20	37580670
ebi-a-GCST90001698	rs73102494	CD45RA+ CD28- (	20	41197100
ebi-a-GCST90001698	rs148727285	CD45RA+ CD28- (	20	48156477
ebi-a-GCST90001698	rs149483829	CD45RA+ CD28- (	20	49499194
ebi-a-GCST90001698	rs11538695	CD45RA+ CD28- (	20	50004560
ebi-a-GCST90001698	rs76720524	CD45RA+ CD28- (	20	1832785
ebi-a-GCST90001698	rs145250558	CD45RA+ CD28- (	20	15245578
ebi-a-GCST90001698	rs73091371	CD45RA+ CD28- (	20	4420029
ebi-a-GCST90001698	rs17791728	CD45RA+ CD28- (	20	17556587
ebi-a-GCST90001698	rs184911123	CD45RA+ CD28- (	20	18880832
ebi-a-GCST90001698	rs75600477	CD45RA+ CD28- (	20	40197928
ebi-a-GCST90001698	rs566794699	CD45RA+ CD28- (	20	25333520
ebi-a-GCST90001698	rs145263449	CD45RA+ CD28- (	20	12493170
ebi-a-GCST90001698	rs459056	CD45RA+ CD28- (	20	15614140
ebi-a-GCST90001698	rs113369914	CD45RA+ CD28- (	20	21420694
ebi-a-GCST90001698	rs118180479	CD45RA+ CD28- (	20	37027035

ebi-a-GCST90001698	rs148513824	CD45RA+ CD28- (	20	61268143
ebi-a-GCST90001698	rs118009051	CD45RA+ CD28- (	21	17079538
ebi-a-GCST90001698	rs117922572	CD45RA+ CD28- (	21	27289871
ebi-a-GCST90001698	rs75483199	CD45RA+ CD28- (	21	32526668
ebi-a-GCST90001698	rs111541596	CD45RA+ CD28- (	21	15989102
ebi-a-GCST90001698	rs6517382	CD45RA+ CD28- (	21	38156170
ebi-a-GCST90001698	rs79655468	CD45RA+ CD28- (	21	42790901
ebi-a-GCST90001698	rs7278151	CD45RA+ CD28- (	21	29724671
ebi-a-GCST90001698	rs111714479	CD45RA+ CD28- (	21	35532237
ebi-a-GCST90001698	rs7510888	CD45RA+ CD28- (	22	26185033
ebi-a-GCST90001698	rs77841631	CD45RA+ CD28- (	22	44942066
ebi-a-GCST90001698	rs73878713	CD45RA+ CD28- (	22	23055148
ebi-a-GCST90001698	rs117562840	CD45RA+ CD28- (	22	42680877
ebi-a-GCST90001698	rs146461857	CD45RA+ CD28- (	22	23709958
ebi-a-GCST90001698	rs189798499	CD45RA+ CD28- (	22	24402964
ebi-a-GCST90001698	rs117885295	CD45RA+ CD28- (	22	27731404
ebi-a-GCST90001698	rs11703161	CD45RA+ CD28- (	22	40335424
ebi-a-GCST90001698	rs79330384	CD45RA+ CD28- (	22	44472281
ebi-a-GCST90001698	rs13055531	CD45RA+ CD28- (	22	32562454
ebi-a-GCST90001698	rs117840149	CD45RA+ CD28- (	22	36506460
ebi-a-GCST90001698	rs132751	CD45RA+ CD28- (	22	36610967
ebi-a-GCST90001698	rs4821845	CD45RA+ CD28- (	22	39354391
ebi-a-GCST90001698	rs142505312	CD45RA+ CD28- (	22	50155532
ebi-a-GCST90001698	rs139939351	CD45RA+ CD28- (	22	50360292
ebi-a-GCST90001699	rs146620586	CD45RA+ CD28- (	1	198524264
ebi-a-GCST90001699	rs74055305	CD45RA+ CD28- (	1	4985225
ebi-a-GCST90001699	rs76444609	CD45RA+ CD28- (	1	249084997
ebi-a-GCST90001699	rs184701860	CD45RA+ CD28- (	2	146317203
ebi-a-GCST90001699	rs4853078	CD45RA+ CD28- (	2	75148081
ebi-a-GCST90001699	rs10176830	CD45RA+ CD28- (	2	83631928
ebi-a-GCST90001699	rs7605634	CD45RA+ CD28- (	2	133321163
ebi-a-GCST90001699	rs7559619	CD45RA+ CD28- (	2	38892207
ebi-a-GCST90001699	rs148524572	CD45RA+ CD28- (	3	185618728
ebi-a-GCST90001699	rs139329941	CD45RA+ CD28- (	3	166969119
ebi-a-GCST90001699	rs79638560	CD45RA+ CD28- (	4	183402200
ebi-a-GCST90001699	rs199797711	CD45RA+ CD28- (	4	120352238
ebi-a-GCST90001699	rs13133130	CD45RA+ CD28- (	4	67792751
ebi-a-GCST90001699	rs147841321	CD45RA+ CD28- (	4	130934266
ebi-a-GCST90001699	rs189420173	CD45RA+ CD28- (	4	4128043
ebi-a-GCST90001699	rs4648125	CD45RA+ CD28- (	4	103535765
ebi-a-GCST90001699	rs80181351	CD45RA+ CD28- (	5	1155401
ebi-a-GCST90001699	rs375356899	CD45RA+ CD28- (	6	165166578
ebi-a-GCST90001699	rs56306620	CD45RA+ CD28- (	6	108456796
ebi-a-GCST90001699	rs73719023	CD45RA+ CD28- (	6	6986777
ebi-a-GCST90001699	rs62461164	CD45RA+ CD28- (	7	77674010
ebi-a-GCST90001699	rs117858699	CD45RA+ CD28- (	7	128071585
ebi-a-GCST90001699	rs7819099	CD45RA+ CD28- (	8	144992862
ebi-a-GCST90001699	rs1747869	CD45RA+ CD28- (	9	139041212

ebi-a-GCST90001699	rs144378515	CD45RA+ CD28- (	10	60542770
ebi-a-GCST90001699	rs142420464	CD45RA+ CD28- (	10	12757694
ebi-a-GCST90001699	rs147119730	CD45RA+ CD28- (	10	15607755
ebi-a-GCST90001699	rs117284243	CD45RA+ CD28- (	11	122918979
ebi-a-GCST90001699	rs76552842	CD45RA+ CD28- (	11	83433494
ebi-a-GCST90001699	rs17175340	CD45RA+ CD28- (	12	23151353
ebi-a-GCST90001699	rs7989683	CD45RA+ CD28- (	13	96304050
ebi-a-GCST90001699	rs74828441	CD45RA+ CD28- (	15	30132737
ebi-a-GCST90001699	rs7186214	CD45RA+ CD28- (	16	79282813
ebi-a-GCST90001699	rs150885241	CD45RA+ CD28- (	17	49002799
ebi-a-GCST90001699	rs4796089	CD45RA+ CD28- (	17	33814758
ebi-a-GCST90001699	rs45609839	CD45RA+ CD28- (	18	22006601
ebi-a-GCST90001700	rs114021246	CD45RA+ CD28- (	1	46898789
ebi-a-GCST90001700	rs202047604	CD45RA+ CD28- (	1	223109887
ebi-a-GCST90001700	rs149050426	CD45RA+ CD28- (	1	160679804
ebi-a-GCST90001700	rs147600774	CD45RA+ CD28- (	1	115926543
ebi-a-GCST90001700	rs2152857	CD45RA+ CD28- (	1	81185936
ebi-a-GCST90001700	rs11580627	CD45RA+ CD28- (	1	207801545
ebi-a-GCST90001700	rs11576563	CD45RA+ CD28- (	1	6118693
ebi-a-GCST90001700	rs12068756	CD45RA+ CD28- (	1	233943028
ebi-a-GCST90001700	rs72723467	CD45RA+ CD28- (	1	175366612
ebi-a-GCST90001700	rs76444609	CD45RA+ CD28- (	1	249084997
ebi-a-GCST90001700	rs114230155	CD45RA+ CD28- (	1	102796337
ebi-a-GCST90001700	rs77676367	CD45RA+ CD28- (	1	27963297
ebi-a-GCST90001700	rs61746209	CD45RA+ CD28- (	1	238644579
ebi-a-GCST90001700	rs114672498	CD45RA+ CD28- (	1	4591565
ebi-a-GCST90001700	rs141656769	CD45RA+ CD28- (	1	58381604
ebi-a-GCST90001700	rs140963286	CD45RA+ CD28- (	1	24640467
ebi-a-GCST90001700	rs79027575	CD45RA+ CD28- (	2	146371302
ebi-a-GCST90001700	rs72931475	CD45RA+ CD28- (	2	159316835
ebi-a-GCST90001700	rs114581990	CD45RA+ CD28- (	2	235962017
ebi-a-GCST90001700	rs146627868	CD45RA+ CD28- (	2	81365670
ebi-a-GCST90001700	rs4670265	CD45RA+ CD28- (	2	38932777
ebi-a-GCST90001700	rs10176830	CD45RA+ CD28- (	2	83631928
ebi-a-GCST90001700	rs148947489	CD45RA+ CD28- (	2	10326418
ebi-a-GCST90001700	rs115943331	CD45RA+ CD28- (	2	240239970
ebi-a-GCST90001700	rs190665193	CD45RA+ CD28- (	2	106438361
ebi-a-GCST90001700	rs2192500	CD45RA+ CD28- (	2	59373057
ebi-a-GCST90001700	rs4853078	CD45RA+ CD28- (	2	75148081
ebi-a-GCST90001700	rs16864501	CD45RA+ CD28- (	3	152385976
ebi-a-GCST90001700	rs73218592	CD45RA+ CD28- (	3	109447837
ebi-a-GCST90001700	rs185587352	CD45RA+ CD28- (	3	117384981
ebi-a-GCST90001700	rs76128446	CD45RA+ CD28- (	3	192001379
ebi-a-GCST90001700	rs17878699	CD45RA+ CD28- (	3	3132754
ebi-a-GCST90001700	rs73105597	CD45RA+ CD28- (	3	73306180
ebi-a-GCST90001700	rs115879183	CD45RA+ CD28- (	3	194347376
ebi-a-GCST90001700	rs148658406	CD45RA+ CD28- (	3	153311399
ebi-a-GCST90001700	rs139329941	CD45RA+ CD28- (	3	166969119

ebi-a-GCST90001700	rs111747460	CD45RA+ CD28- (	3	74016318
ebi-a-GCST90001700	rs114219959	CD45RA+ CD28- (	3	106957827
ebi-a-GCST90001700	rs73173143	CD45RA+ CD28- (	3	177136644
ebi-a-GCST90001700	rs114827401	CD45RA+ CD28- (	3	31287377
ebi-a-GCST90001700	rs147841321	CD45RA+ CD28- (	4	130934266
ebi-a-GCST90001700	rs116399750	CD45RA+ CD28- (	4	176754667
ebi-a-GCST90001700	rs79395509	CD45RA+ CD28- (	4	176348299
ebi-a-GCST90001700	rs189420173	CD45RA+ CD28- (	4	4128043
ebi-a-GCST90001700	rs114421150	CD45RA+ CD28- (	4	16986930
ebi-a-GCST90001700	rs193135177	CD45RA+ CD28- (	4	177338168
ebi-a-GCST90001700	rs77689186	CD45RA+ CD28- (	4	176614955
ebi-a-GCST90001700	rs114559319	CD45RA+ CD28- (	4	85213802
ebi-a-GCST90001700	rs12499962	CD45RA+ CD28- (	4	99029457
ebi-a-GCST90001700	rs114685875	CD45RA+ CD28- (	4	138349095
ebi-a-GCST90001700	rs149021870	CD45RA+ CD28- (	4	135635954
ebi-a-GCST90001700	rs75993384	CD45RA+ CD28- (	4	55305871
ebi-a-GCST90001700	rs114430346	CD45RA+ CD28- (	4	102760648
ebi-a-GCST90001700	rs191607214	CD45RA+ CD28- (	4	123414413
ebi-a-GCST90001700	rs114619811	CD45RA+ CD28- (	4	71875684
ebi-a-GCST90001700	rs146374482	CD45RA+ CD28- (	4	85578601
ebi-a-GCST90001700	rs78088167	CD45RA+ CD28- (	4	130098084
ebi-a-GCST90001700	rs146047936	CD45RA+ CD28- (	4	97392739
ebi-a-GCST90001700	rs62321187	CD45RA+ CD28- (	4	120605817
ebi-a-GCST90001700	rs12501785	CD45RA+ CD28- (	4	180565400
ebi-a-GCST90001700	rs80181351	CD45RA+ CD28- (	5	1155401
ebi-a-GCST90001700	rs148291161	CD45RA+ CD28- (	5	168556910
ebi-a-GCST90001700	rs140976870	CD45RA+ CD28- (	5	120420356
ebi-a-GCST90001700	rs116581718	CD45RA+ CD28- (	5	29413612
ebi-a-GCST90001700	rs112254351	CD45RA+ CD28- (	5	169422728
ebi-a-GCST90001700	rs75146333	CD45RA+ CD28- (	5	65455207
ebi-a-GCST90001700	rs79725470	CD45RA+ CD28- (	5	94705605
ebi-a-GCST90001700	rs146171997	CD45RA+ CD28- (	5	52147101
ebi-a-GCST90001700	rs17642570	CD45RA+ CD28- (	5	148699825
ebi-a-GCST90001700	rs180927720	CD45RA+ CD28- (	5	11287269
ebi-a-GCST90001700	rs144613377	CD45RA+ CD28- (	5	81311763
ebi-a-GCST90001700	rs189412340	CD45RA+ CD28- (	5	75564943
ebi-a-GCST90001700	rs115715247	CD45RA+ CD28- (	5	68399621
ebi-a-GCST90001700	rs73400437	CD45RA+ CD28- (	6	24904561
ebi-a-GCST90001700	rs150287087	CD45RA+ CD28- (	6	125051261
ebi-a-GCST90001700	rs117708929	CD45RA+ CD28- (	6	124879601
ebi-a-GCST90001700	rs151244510	CD45RA+ CD28- (	6	74134014
ebi-a-GCST90001700	rs76602542	CD45RA+ CD28- (	6	82300125
ebi-a-GCST90001700	rs145604620	CD45RA+ CD28- (	6	11491250
ebi-a-GCST90001700	rs375356899	CD45RA+ CD28- (	6	165166578
ebi-a-GCST90001700	rs79518488	CD45RA+ CD28- (	6	28389143
ebi-a-GCST90001700	rs73719023	CD45RA+ CD28- (	6	6986777
ebi-a-GCST90001700	rs149208831	CD45RA+ CD28- (	6	10017676
ebi-a-GCST90001700	rs115541238	CD45RA+ CD28- (	6	44289942

ebi-a-GCST90001700	rs138327786	CD45RA+ CD28- (	6	1730982
ebi-a-GCST90001700	rs79647440	CD45RA+ CD28- (	7	142438302
ebi-a-GCST90001700	rs184379743	CD45RA+ CD28- (	7	83499414
ebi-a-GCST90001700	rs77465871	CD45RA+ CD28- (	7	47504039
ebi-a-GCST90001700	rs186251573	CD45RA+ CD28- (	7	13558580
ebi-a-GCST90001700	rs34947488	CD45RA+ CD28- (	7	125751
ebi-a-GCST90001700	rs149394991	CD45RA+ CD28- (	7	30944767
ebi-a-GCST90001700	rs71528265	CD45RA+ CD28- (	7	67397567
ebi-a-GCST90001700	rs117469911	CD45RA+ CD28- (	7	79791344
ebi-a-GCST90001700	rs111065494	CD45RA+ CD28- (	7	48369369
ebi-a-GCST90001700	rs10274897	CD45RA+ CD28- (	7	45344881
ebi-a-GCST90001700	rs185754925	CD45RA+ CD28- (	7	62917010
ebi-a-GCST90001700	rs190764862	CD45RA+ CD28- (	7	41114935
ebi-a-GCST90001700	rs150400236	CD45RA+ CD28- (	7	136855654
ebi-a-GCST90001700	rs78470553	CD45RA+ CD28- (	7	158334355
ebi-a-GCST90001700	rs143845801	CD45RA+ CD28- (	7	84434549
ebi-a-GCST90001700	rs62491100	CD45RA+ CD28- (	7	112589900
ebi-a-GCST90001700	rs117692894	CD45RA+ CD28- (	7	39587008
ebi-a-GCST90001700	rs116979366	CD45RA+ CD28- (	7	31134950
ebi-a-GCST90001700	rs117296998	CD45RA+ CD28- (	7	33902745
ebi-a-GCST90001700	rs117478414	CD45RA+ CD28- (	8	56081028
ebi-a-GCST90001700	rs17524895	CD45RA+ CD28- (	8	11660136
ebi-a-GCST90001700	rs117867588	CD45RA+ CD28- (	8	15699657
ebi-a-GCST90001700	rs7823439	CD45RA+ CD28- (	8	16615390
ebi-a-GCST90001700	rs72719812	CD45RA+ CD28- (	8	136875749
ebi-a-GCST90001700	rs6982554	CD45RA+ CD28- (	8	4172183
ebi-a-GCST90001700	rs79218577	CD45RA+ CD28- (	8	143859934
ebi-a-GCST90001700	rs117808048	CD45RA+ CD28- (	8	73079210
ebi-a-GCST90001700	rs34039708	CD45RA+ CD28- (	8	12500832
ebi-a-GCST90001700	rs79851411	CD45RA+ CD28- (	8	13716390
ebi-a-GCST90001700	rs138901088	CD45RA+ CD28- (	9	2517641
ebi-a-GCST90001700	rs142884906	CD45RA+ CD28- (	9	130626387
ebi-a-GCST90001700	rs559632285	CD45RA+ CD28- (	9	45362596
ebi-a-GCST90001700	rs9408992	CD45RA+ CD28- (	9	102347204
ebi-a-GCST90001700	rs111972324	CD45RA+ CD28- (	9	38278118
ebi-a-GCST90001700	rs374964617	CD45RA+ CD28- (	9	504491
ebi-a-GCST90001700	rs7044904	CD45RA+ CD28- (	9	16069316
ebi-a-GCST90001700	rs575794055	CD45RA+ CD28- (	10	119966169
ebi-a-GCST90001700	rs11202534	CD45RA+ CD28- (	10	89500755
ebi-a-GCST90001700	rs74157741	CD45RA+ CD28- (	10	121973769
ebi-a-GCST90001700	rs143395500	CD45RA+ CD28- (	10	69536834
ebi-a-GCST90001700	rs78069205	CD45RA+ CD28- (	10	9668124
ebi-a-GCST90001700	rs143129647	CD45RA+ CD28- (	10	125841271
ebi-a-GCST90001700	rs11200733	CD45RA+ CD28- (	10	85685084
ebi-a-GCST90001700	rs75772839	CD45RA+ CD28- (	10	31530534
ebi-a-GCST90001700	rs10824158	CD45RA+ CD28- (	10	53915868
ebi-a-GCST90001700	rs4919341	CD45RA+ CD28- (	10	101282501
ebi-a-GCST90001700	rs147968748	CD45RA+ CD28- (	10	54704473

ebi-a-GCST90001700	rs78922116	CD45RA+ CD28- (	11	25534610
ebi-a-GCST90001700	rs61759531	CD45RA+ CD28- (	11	130773279
ebi-a-GCST90001700	rs12279408	CD45RA+ CD28- (	11	84269972
ebi-a-GCST90001700	rs116916598	CD45RA+ CD28- (	11	121746998
ebi-a-GCST90001700	rs10769706	CD45RA+ CD28- (	11	6693054
ebi-a-GCST90001700	rs189796120	CD45RA+ CD28- (	11	109281106
ebi-a-GCST90001700	rs117284243	CD45RA+ CD28- (	11	122918979
ebi-a-GCST90001700	rs117173207	CD45RA+ CD28- (	11	24544063
ebi-a-GCST90001700	rs1106305	CD45RA+ CD28- (	11	12065087
ebi-a-GCST90001700	rs76552842	CD45RA+ CD28- (	11	83433494
ebi-a-GCST90001700	rs147107195	CD45RA+ CD28- (	12	48190275
ebi-a-GCST90001700	rs77113660	CD45RA+ CD28- (	12	83249816
ebi-a-GCST90001700	rs117785622	CD45RA+ CD28- (	12	132283582
ebi-a-GCST90001700	rs188074116	CD45RA+ CD28- (	12	19311236
ebi-a-GCST90001700	rs73129374	CD45RA+ CD28- (	12	77297125
ebi-a-GCST90001700	rs10505739	CD45RA+ CD28- (	12	10043518
ebi-a-GCST90001700	rs139898536	CD45RA+ CD28- (	12	109036912
ebi-a-GCST90001700	rs138410422	CD45RA+ CD28- (	12	93329083
ebi-a-GCST90001700	rs146822590	CD45RA+ CD28- (	12	130060949
ebi-a-GCST90001700	rs76337573	CD45RA+ CD28- (	12	79703102
ebi-a-GCST90001700	rs59615051	CD45RA+ CD28- (	13	114557883
ebi-a-GCST90001700	rs113007178	CD45RA+ CD28- (	13	40914557
ebi-a-GCST90001700	rs77214871	CD45RA+ CD28- (	13	84005540
ebi-a-GCST90001700	rs190658660	CD45RA+ CD28- (	13	93311613
ebi-a-GCST90001700	rs76942041	CD45RA+ CD28- (	13	24658539
ebi-a-GCST90001700	rs10492710	CD45RA+ CD28- (	13	98908125
ebi-a-GCST90001700	rs141850210	CD45RA+ CD28- (	14	54222912
ebi-a-GCST90001700	rs138547687	CD45RA+ CD28- (	14	29258015
ebi-a-GCST90001700	rs183436787	CD45RA+ CD28- (	14	41636680
ebi-a-GCST90001700	rs144275943	CD45RA+ CD28- (	14	34065832
ebi-a-GCST90001700	rs35068665	CD45RA+ CD28- (	14	101798387
ebi-a-GCST90001700	rs72758450	CD45RA+ CD28- (	15	69811049
ebi-a-GCST90001700	rs74828441	CD45RA+ CD28- (	15	30132737
ebi-a-GCST90001700	rs1879612	CD45RA+ CD28- (	15	99398708
ebi-a-GCST90001700	rs80331554	CD45RA+ CD28- (	15	59013643
ebi-a-GCST90001700	rs149908314	CD45RA+ CD28- (	15	54344919
ebi-a-GCST90001700	rs8040007	CD45RA+ CD28- (	15	101775026
ebi-a-GCST90001700	rs188087675	CD45RA+ CD28- (	15	29114742
ebi-a-GCST90001700	rs147093663	CD45RA+ CD28- (	15	81422902
ebi-a-GCST90001700	rs117038196	CD45RA+ CD28- (	15	60603443
ebi-a-GCST90001700	rs141044525	CD45RA+ CD28- (	16	1824187
ebi-a-GCST90001700	rs12596264	CD45RA+ CD28- (	16	1058881
ebi-a-GCST90001700	rs77467380	CD45RA+ CD28- (	16	78662086
ebi-a-GCST90001700	rs138937927	CD45RA+ CD28- (	16	51095561
ebi-a-GCST90001700	rs4796089	CD45RA+ CD28- (	17	33814758
ebi-a-GCST90001700	rs138697156	CD45RA+ CD28- (	17	29972138
ebi-a-GCST90001700	rs180697858	CD45RA+ CD28- (	17	1649801
ebi-a-GCST90001700	rs138765259	CD45RA+ CD28- (	17	47337653

ebi-a-GCST90001700	rs12450105	CD45RA+ CD28- (	17	54427514
ebi-a-GCST90001700	rs138281296	CD45RA+ CD28- (	17	22048470
ebi-a-GCST90001700	rs144104650	CD45RA+ CD28- (	17	5023557
ebi-a-GCST90001700	rs112048022	CD45RA+ CD28- (	17	20694000
ebi-a-GCST90001700	rs150885241	CD45RA+ CD28- (	17	49002799
ebi-a-GCST90001700	rs142866937	CD45RA+ CD28- (	17	51002048
ebi-a-GCST90001700	rs150156077	CD45RA+ CD28- (	18	4348487
ebi-a-GCST90001700	rs184040186	CD45RA+ CD28- (	18	1699838
ebi-a-GCST90001700	rs113598754	CD45RA+ CD28- (	18	9944924
ebi-a-GCST90001700	rs75438005	CD45RA+ CD28- (	18	26017285
ebi-a-GCST90001700	rs116915023	CD45RA+ CD28- (	18	62442252
ebi-a-GCST90001700	rs138766897	CD45RA+ CD28- (	18	7853372
ebi-a-GCST90001700	rs79290306	CD45RA+ CD28- (	19	2363095
ebi-a-GCST90001700	rs146650864	CD45RA+ CD28- (	19	22454852
ebi-a-GCST90001700	rs59745713	CD45RA+ CD28- (	19	34075835
ebi-a-GCST90001700	rs147572731	CD45RA+ CD28- (	19	49416171
ebi-a-GCST90001700	rs191923221	CD45RA+ CD28- (	19	18858754
ebi-a-GCST90001700	rs200198801	CD45RA+ CD28- (	19	46387941
ebi-a-GCST90001700	rs118089863	CD45RA+ CD28- (	19	24477916
ebi-a-GCST90001700	rs145410896	CD45RA+ CD28- (	19	34111992
ebi-a-GCST90001700	rs449647	CD45RA+ CD28- (	19	45408564
ebi-a-GCST90001700	rs61578886	CD45RA+ CD28- (	20	898372
ebi-a-GCST90001700	rs147207602	CD45RA+ CD28- (	20	41693849
ebi-a-GCST90001700	rs137942933	CD45RA+ CD28- (	20	33104122
ebi-a-GCST90001700	rs17791728	CD45RA+ CD28- (	20	17556587
ebi-a-GCST90001700	rs118180479	CD45RA+ CD28- (	20	37027035
ebi-a-GCST90001700	rs75600477	CD45RA+ CD28- (	20	40197928
ebi-a-GCST90001700	rs180972485	CD45RA+ CD28- (	20	24457030
ebi-a-GCST90001700	rs41276984	CD45RA+ CD28- (	20	37580670
ebi-a-GCST90001700	rs7276763	CD45RA+ CD28- (	21	35824714
ebi-a-GCST90001700	rs62218442	CD45RA+ CD28- (	22	23072586
ebi-a-GCST90001700	rs132751	CD45RA+ CD28- (	22	36610967
ebi-a-GCST90001700	rs8142081	CD45RA+ CD28- (	22	25196811
ebi-a-GCST90001701	rs4664360	BAFF-R on CD20-	2	161569376
ebi-a-GCST90001701	rs12630507	BAFF-R on CD20-	3	141650463
ebi-a-GCST90001701	rs35744279	BAFF-R on CD20-	3	190520073
ebi-a-GCST90001701	rs72664998	BAFF-R on CD20-	8	69444614
ebi-a-GCST90001701	rs12311737	BAFF-R on CD20-	12	131958370
ebi-a-GCST90001701	rs1628296	BAFF-R on CD20-	12	123309183
ebi-a-GCST90001701	rs4406935	BAFF-R on CD20-	13	106357764
ebi-a-GCST90001701	rs1780947	BAFF-R on CD20-	14	20326938
ebi-a-GCST90001701	rs111901500	BAFF-R on CD20-	14	48900270
ebi-a-GCST90001701	rs78083064	BAFF-R on CD20-	16	11821999
ebi-a-GCST90001701	rs117920411	BAFF-R on CD20-	16	16968389
ebi-a-GCST90001701	rs117783732	BAFF-R on CD20-	16	78797349
ebi-a-GCST90001701	rs139785721	BAFF-R on CD20-	17	27211620
ebi-a-GCST90001701	rs117457546	BAFF-R on CD20-	19	8466710
ebi-a-GCST90001701	rs191405504	BAFF-R on CD20-	22	41305796

ebi-a-GCST90001701	rs73163382	BAFF-R on CD20-	22	42251917
ebi-a-GCST90001701	rs185749756	BAFF-R on CD20-	22	39920299
ebi-a-GCST90001701	rs73165129	BAFF-R on CD20-	22	42315371
ebi-a-GCST90001702	rs4657041	BAFF-R on CD24+	1	161478859
ebi-a-GCST90001702	rs6680891	BAFF-R on CD24+	1	164083691
ebi-a-GCST90001702	rs2925323	BAFF-R on CD24+	3	101658926
ebi-a-GCST90001702	rs116470335	BAFF-R on CD24+	4	24492147
ebi-a-GCST90001702	rs56020760	BAFF-R on CD24+	10	72391681
ebi-a-GCST90001702	rs116897208	BAFF-R on CD24+	10	15100608
ebi-a-GCST90001702	rs143106266	BAFF-R on CD24+	13	29908421
ebi-a-GCST90001702	rs1885397	BAFF-R on CD24+	14	55233098
ebi-a-GCST90001702	rs185794015	BAFF-R on CD24+	22	44440180
ebi-a-GCST90001702	rs117086217	BAFF-R on CD24+	22	39876838
ebi-a-GCST90001702	rs9614288	BAFF-R on CD24+	22	44297686
ebi-a-GCST90001702	rs73165129	BAFF-R on CD24+	22	42315371
ebi-a-GCST90001702	rs5758649	BAFF-R on CD24+	22	42605548
ebi-a-GCST90001702	rs146069723	BAFF-R on CD24+	22	43339331
ebi-a-GCST90001702	rs187315152	BAFF-R on CD24+	22	41305795
ebi-a-GCST90001702	rs528009599	BAFF-R on CD24+	22	40568020
ebi-a-GCST90001702	rs13058590	BAFF-R on CD24+	22	39641412
ebi-a-GCST90001702	rs149228586	BAFF-R on CD24+	22	41608058
ebi-a-GCST90001702	rs2142827	BAFF-R on CD24+	22	42874781
ebi-a-GCST90001702	rs143944768	BAFF-R on CD24+	22	44378670
ebi-a-GCST90001703	rs72658947	BAFF-R on IgD+ C	1	30372026
ebi-a-GCST90001703	rs4657041	BAFF-R on IgD+ C	1	161478859
ebi-a-GCST90001703	rs6680891	BAFF-R on IgD+ C	1	164083691
ebi-a-GCST90001703	rs74594247	BAFF-R on IgD+ C	1	147057319
ebi-a-GCST90001703	rs114318843	BAFF-R on IgD+ C	2	164755835
ebi-a-GCST90001703	rs116470335	BAFF-R on IgD+ C	4	24492147
ebi-a-GCST90001703	rs56020760	BAFF-R on IgD+ C	10	72391681
ebi-a-GCST90001703	rs143106266	BAFF-R on IgD+ C	13	29908421
ebi-a-GCST90001703	rs1885397	BAFF-R on IgD+ C	14	55233098
ebi-a-GCST90001703	rs75056525	BAFF-R on IgD+ C	22	43349884
ebi-a-GCST90001703	rs2142827	BAFF-R on IgD+ C	22	42874781
ebi-a-GCST90001703	rs189503542	BAFF-R on IgD+ C	22	44698304
ebi-a-GCST90001703	rs117086217	BAFF-R on IgD+ C	22	39876838
ebi-a-GCST90001703	rs5758649	BAFF-R on IgD+ C	22	42605548
ebi-a-GCST90001703	rs73165129	BAFF-R on IgD+ C	22	42315371
ebi-a-GCST90001703	rs149228586	BAFF-R on IgD+ C	22	41608058
ebi-a-GCST90001703	rs5996232	BAFF-R on IgD+ C	22	43159810
ebi-a-GCST90001703	rs528009599	BAFF-R on IgD+ C	22	40568020
ebi-a-GCST90001703	rs9614288	BAFF-R on IgD+ C	22	44297686
ebi-a-GCST90001704	rs1801274	BAFF-R on IgD+ C	1	161479745
ebi-a-GCST90001704	rs6680891	BAFF-R on IgD+ C	1	164083691
ebi-a-GCST90001704	rs149153670	BAFF-R on IgD+ C	2	142280835
ebi-a-GCST90001704	rs2925323	BAFF-R on IgD+ C	3	101658926
ebi-a-GCST90001704	rs116470335	BAFF-R on IgD+ C	4	24492147
ebi-a-GCST90001704	rs2939924	BAFF-R on IgD+ C	10	132491701

ebi-a-GCST90001704	rs4880914	BAFF-R on IgD+ C	10	1746219
ebi-a-GCST90001704	rs1563991	BAFF-R on IgD+ C	11	37670518
ebi-a-GCST90001704	rs143106266	BAFF-R on IgD+ C	13	29908421
ebi-a-GCST90001704	rs35754161	BAFF-R on IgD+ C	15	51655048
ebi-a-GCST90001704	rs145868946	BAFF-R on IgD+ C	17	26045225
ebi-a-GCST90001704	rs148563640	BAFF-R on IgD+ C	17	48145906
ebi-a-GCST90001704	rs76146864	BAFF-R on IgD+ C	22	40246055
ebi-a-GCST90001704	rs5757678	BAFF-R on IgD+ C	22	39843409
ebi-a-GCST90001704	rs149806587	BAFF-R on IgD+ C	22	40774205
ebi-a-GCST90001704	rs149398740	BAFF-R on IgD+ C	22	23924845
ebi-a-GCST90001704	rs1894588	BAFF-R on IgD+ C	22	40629980
ebi-a-GCST90001704	rs1081003	BAFF-R on IgD+ C	22	42525756
ebi-a-GCST90001704	rs117705111	BAFF-R on IgD+ C	22	41957510
ebi-a-GCST90001704	rs117430146	BAFF-R on IgD+ C	22	41998660
ebi-a-GCST90001704	rs5759079	BAFF-R on IgD+ C	22	43393437
ebi-a-GCST90001704	rs73165129	BAFF-R on IgD+ C	22	42315371
ebi-a-GCST90001705	rs6680891	BAFF-R on IgD+ C	1	164083691
ebi-a-GCST90001705	rs1801274	BAFF-R on IgD+ C	1	161479745
ebi-a-GCST90001705	rs2925323	BAFF-R on IgD+ C	3	101658926
ebi-a-GCST90001705	rs116470335	BAFF-R on IgD+ C	4	24492147
ebi-a-GCST90001705	rs448333	BAFF-R on IgD+ C	6	97236851
ebi-a-GCST90001705	rs190757416	BAFF-R on IgD+ C	6	45358923
ebi-a-GCST90001705	rs2519807	BAFF-R on IgD+ C	9	136826108
ebi-a-GCST90001705	rs137911637	BAFF-R on IgD+ C	10	25305421
ebi-a-GCST90001705	rs56020760	BAFF-R on IgD+ C	10	72391681
ebi-a-GCST90001705	rs2939924	BAFF-R on IgD+ C	10	132491701
ebi-a-GCST90001705	rs1563991	BAFF-R on IgD+ C	11	37670518
ebi-a-GCST90001705	rs143106266	BAFF-R on IgD+ C	13	29908421
ebi-a-GCST90001705	rs2110699	BAFF-R on IgD+ C	14	89904438
ebi-a-GCST90001705	rs1894588	BAFF-R on IgD+ C	22	40629980
ebi-a-GCST90001705	rs149806587	BAFF-R on IgD+ C	22	40774205
ebi-a-GCST90001705	rs117430146	BAFF-R on IgD+ C	22	41998660
ebi-a-GCST90001705	rs76146864	BAFF-R on IgD+ C	22	40246055
ebi-a-GCST90001705	rs117705111	BAFF-R on IgD+ C	22	41957510
ebi-a-GCST90001705	rs1081003	BAFF-R on IgD+ C	22	42525756
ebi-a-GCST90001705	rs5757678	BAFF-R on IgD+ C	22	39843409
ebi-a-GCST90001705	rs5759079	BAFF-R on IgD+ C	22	43393437
ebi-a-GCST90001705	rs73165129	BAFF-R on IgD+ C	22	42315371
ebi-a-GCST90001706	rs2570487	BAFF-R on IgD+ C	2	104459443
ebi-a-GCST90001706	rs59598877	BAFF-R on IgD+ C	2	196505282
ebi-a-GCST90001706	rs6804241	BAFF-R on IgD+ C	3	61516583
ebi-a-GCST90001706	rs9815713	BAFF-R on IgD+ C	3	153568131
ebi-a-GCST90001706	rs10155189	BAFF-R on IgD+ C	4	38927396
ebi-a-GCST90001706	rs55846026	BAFF-R on IgD+ C	4	130179421
ebi-a-GCST90001706	rs57736830	BAFF-R on IgD+ C	5	168402085
ebi-a-GCST90001706	rs1368364	BAFF-R on IgD+ C	5	146926845
ebi-a-GCST90001706	rs17596501	BAFF-R on IgD+ C	5	1557372
ebi-a-GCST90001706	rs73451452	BAFF-R on IgD+ C	7	137804100

ebi-a-GCST90001706	rs115650989	BAFF-R on IgD+ C	8	145510474
ebi-a-GCST90001706	rs75589560	BAFF-R on IgD+ C	8	129239338
ebi-a-GCST90001706	rs150624416	BAFF-R on IgD+ C	10	57677034
ebi-a-GCST90001706	rs11599977	BAFF-R on IgD+ C	11	38419307
ebi-a-GCST90001706	rs79451942	BAFF-R on IgD+ C	11	109330334
ebi-a-GCST90001706	rs139758246	BAFF-R on IgD+ C	16	1227020
ebi-a-GCST90001706	rs9896701	BAFF-R on IgD+ C	17	68048182
ebi-a-GCST90001706	rs7504122	BAFF-R on IgD+ C	18	10226551
ebi-a-GCST90001706	rs35291683	BAFF-R on IgD+ C	21	42576275
ebi-a-GCST90001706	rs147730115	BAFF-R on IgD+ C	21	26858465
ebi-a-GCST90001706	rs6002543	BAFF-R on IgD+ C	22	42320547
ebi-a-GCST90001706	rs1894588	BAFF-R on IgD+ C	22	40629980
ebi-a-GCST90001706	rs76146864	BAFF-R on IgD+ C	22	40246055
ebi-a-GCST90001706	rs149806587	BAFF-R on IgD+ C	22	40774205
ebi-a-GCST90001706	rs117705111	BAFF-R on IgD+ C	22	41957510
ebi-a-GCST90001706	rs5757678	BAFF-R on IgD+ C	22	39843409
ebi-a-GCST90001706	rs117430146	BAFF-R on IgD+ C	22	41998660
ebi-a-GCST90001706	rs1081003	BAFF-R on IgD+ C	22	42525756
ebi-a-GCST90001706	rs2018340	BAFF-R on IgD+ C	22	43390319
ebi-a-GCST90001707	rs4689407	BAFF-R on IgD+ C	4	6341740
ebi-a-GCST90001707	rs57736830	BAFF-R on IgD+ C	5	168402085
ebi-a-GCST90001707	rs73451452	BAFF-R on IgD+ C	7	137804100
ebi-a-GCST90001707	rs115650989	BAFF-R on IgD+ C	8	145510474
ebi-a-GCST90001707	rs79451942	BAFF-R on IgD+ C	11	109330334
ebi-a-GCST90001707	rs80241990	BAFF-R on IgD+ C	11	3250248
ebi-a-GCST90001707	rs11599977	BAFF-R on IgD+ C	11	38419307
ebi-a-GCST90001707	rs117397694	BAFF-R on IgD+ C	12	5565515
ebi-a-GCST90001707	rs113973452	BAFF-R on IgD+ C	14	100402055
ebi-a-GCST90001707	rs7504122	BAFF-R on IgD+ C	18	10226551
ebi-a-GCST90001707	rs35291683	BAFF-R on IgD+ C	21	42576275
ebi-a-GCST90001707	rs147730115	BAFF-R on IgD+ C	21	26858465
ebi-a-GCST90001707	rs139290596	BAFF-R on IgD+ C	22	44349795
ebi-a-GCST90001707	rs5758649	BAFF-R on IgD+ C	22	42605548
ebi-a-GCST90001707	rs113530150	BAFF-R on IgD+ C	22	40567196
ebi-a-GCST90001707	rs146069723	BAFF-R on IgD+ C	22	43339331
ebi-a-GCST90001707	rs150612106	BAFF-R on IgD+ C	22	40175781
ebi-a-GCST90001707	rs73165129	BAFF-R on IgD+ C	22	42315371
ebi-a-GCST90001707	rs11090614	BAFF-R on IgD+ C	22	44300847
ebi-a-GCST90001707	rs187315152	BAFF-R on IgD+ C	22	41305795
ebi-a-GCST90001707	rs117826659	BAFF-R on IgD+ C	22	39919408
ebi-a-GCST90001708	rs74594247	BAFF-R on IgD+ C	1	147057319
ebi-a-GCST90001708	rs4657041	BAFF-R on IgD+ C	1	161478859
ebi-a-GCST90001708	rs72658947	BAFF-R on IgD+ C	1	30372026
ebi-a-GCST90001708	rs114318843	BAFF-R on IgD+ C	2	164755835
ebi-a-GCST90001708	rs141130967	BAFF-R on IgD+ C	3	101706582
ebi-a-GCST90001708	rs116023377	BAFF-R on IgD+ C	4	18858102
ebi-a-GCST90001708	rs35631270	BAFF-R on IgD+ C	4	80524348
ebi-a-GCST90001708	rs34006338	BAFF-R on IgD+ C	7	45957318

ebi-a-GCST90001708	rs11761810	BAFF-R on IgD+ C	7	152779596
ebi-a-GCST90001708	rs10273088	BAFF-R on IgD+ C	7	13005993
ebi-a-GCST90001708	rs116897208	BAFF-R on IgD+ C	10	15100608
ebi-a-GCST90001708	rs72805400	BAFF-R on IgD+ C	10	77064133
ebi-a-GCST90001708	rs1563991	BAFF-R on IgD+ C	11	37670518
ebi-a-GCST90001708	rs143106266	BAFF-R on IgD+ C	13	29908421
ebi-a-GCST90001708	rs72686437	BAFF-R on IgD+ C	14	22596030
ebi-a-GCST90001708	rs16957279	BAFF-R on IgD+ C	15	33954227
ebi-a-GCST90001708	rs148563640	BAFF-R on IgD+ C	17	48145906
ebi-a-GCST90001708	rs117499379	BAFF-R on IgD+ C	18	4343023
ebi-a-GCST90001708	rs5757678	BAFF-R on IgD+ C	22	39843409
ebi-a-GCST90001708	rs5759079	BAFF-R on IgD+ C	22	43393437
ebi-a-GCST90001708	rs117430146	BAFF-R on IgD+ C	22	41998660
ebi-a-GCST90001708	rs73165129	BAFF-R on IgD+ C	22	42315371
ebi-a-GCST90001708	rs1894588	BAFF-R on IgD+ C	22	40629980
ebi-a-GCST90001708	rs149806587	BAFF-R on IgD+ C	22	40774205
ebi-a-GCST90001708	rs76146864	BAFF-R on IgD+ C	22	40246055
ebi-a-GCST90001708	rs117705111	BAFF-R on IgD+ C	22	41957510
ebi-a-GCST90001708	rs1081003	BAFF-R on IgD+ C	22	42525756
ebi-a-GCST90001709	rs6680891	BAFF-R on IgD+ C	1	164083691
ebi-a-GCST90001709	rs1801274	BAFF-R on IgD+ C	1	161479745
ebi-a-GCST90001709	rs72658947	BAFF-R on IgD+ C	1	30372026
ebi-a-GCST90001709	rs9434824	BAFF-R on IgD+ C	1	7137637
ebi-a-GCST90001709	rs2925323	BAFF-R on IgD+ C	3	101658926
ebi-a-GCST90001709	rs148695055	BAFF-R on IgD+ C	4	137058391
ebi-a-GCST90001709	rs116470335	BAFF-R on IgD+ C	4	24492147
ebi-a-GCST90001709	rs73737980	BAFF-R on IgD+ C	6	33025017
ebi-a-GCST90001709	rs12535011	BAFF-R on IgD+ C	7	33667945
ebi-a-GCST90001709	rs1563991	BAFF-R on IgD+ C	11	37670518
ebi-a-GCST90001709	rs144136349	BAFF-R on IgD+ C	13	34499243
ebi-a-GCST90001709	rs143106266	BAFF-R on IgD+ C	13	29908421
ebi-a-GCST90001709	rs35754161	BAFF-R on IgD+ C	15	51655048
ebi-a-GCST90001709	rs148563640	BAFF-R on IgD+ C	17	48145906
ebi-a-GCST90001709	rs6079871	BAFF-R on IgD+ C	20	15504410
ebi-a-GCST90001709	rs149398740	BAFF-R on IgD+ C	22	23924845
ebi-a-GCST90001709	rs117430146	BAFF-R on IgD+ C	22	41998660
ebi-a-GCST90001709	rs1894588	BAFF-R on IgD+ C	22	40629980
ebi-a-GCST90001709	rs76146864	BAFF-R on IgD+ C	22	40246055
ebi-a-GCST90001709	rs149806587	BAFF-R on IgD+ C	22	40774205
ebi-a-GCST90001709	rs5759079	BAFF-R on IgD+ C	22	43393437
ebi-a-GCST90001709	rs5757678	BAFF-R on IgD+ C	22	39843409
ebi-a-GCST90001709	rs117705111	BAFF-R on IgD+ C	22	41957510
ebi-a-GCST90001709	rs73165129	BAFF-R on IgD+ C	22	42315371
ebi-a-GCST90001709	rs1081003	BAFF-R on IgD+ C	22	42525756
ebi-a-GCST90001710	rs6680891	BAFF-R on IgD- C	1	164083691
ebi-a-GCST90001710	rs4657041	BAFF-R on IgD- C	1	161478859
ebi-a-GCST90001710	rs10169360	BAFF-R on IgD- C	2	5558511
ebi-a-GCST90001710	rs9869373	BAFF-R on IgD- C	3	101645001

ebi-a-GCST90001710	rs7684683	BAFF-R on IgD- C	4	80423021
ebi-a-GCST90001710	rs2939924	BAFF-R on IgD- C	10	132491701
ebi-a-GCST90001710	rs2861184	BAFF-R on IgD- C	11	38422885
ebi-a-GCST90001710	rs143106266	BAFF-R on IgD- C	13	29908421
ebi-a-GCST90001710	rs4467081	BAFF-R on IgD- C	16	25853127
ebi-a-GCST90001710	rs11083093	BAFF-R on IgD- C	18	22393283
ebi-a-GCST90001710	rs528009599	BAFF-R on IgD- C	22	40568020
ebi-a-GCST90001710	rs5750432	BAFF-R on IgD- C	22	22402940
ebi-a-GCST90001710	rs73165129	BAFF-R on IgD- C	22	42315371
ebi-a-GCST90001710	rs9614288	BAFF-R on IgD- C	22	44297686
ebi-a-GCST90001710	rs146069723	BAFF-R on IgD- C	22	43339331
ebi-a-GCST90001710	rs113530150	BAFF-R on IgD- C	22	40567196
ebi-a-GCST90001710	rs142780018	BAFF-R on IgD- C	22	44395926
ebi-a-GCST90001710	rs149398740	BAFF-R on IgD- C	22	23924845
ebi-a-GCST90001710	rs150612106	BAFF-R on IgD- C	22	40175781
ebi-a-GCST90001710	rs6000850	BAFF-R on IgD- C	22	38032090
ebi-a-GCST90001710	rs13058590	BAFF-R on IgD- C	22	39641412
ebi-a-GCST90001710	rs9622984	BAFF-R on IgD- C	22	39659460
ebi-a-GCST90001710	rs5758649	BAFF-R on IgD- C	22	42605548
ebi-a-GCST90001710	rs191405504	BAFF-R on IgD- C	22	41305796
ebi-a-GCST90001711	rs4657041	BAFF-R on IgD- C	1	161478859
ebi-a-GCST90001711	rs72780844	BAFF-R on IgD- C	2	15727913
ebi-a-GCST90001711	rs2925323	BAFF-R on IgD- C	3	101658926
ebi-a-GCST90001711	rs2789845	BAFF-R on IgD- C	9	136799915
ebi-a-GCST90001711	rs2939924	BAFF-R on IgD- C	10	132491701
ebi-a-GCST90001711	rs2861184	BAFF-R on IgD- C	11	38422885
ebi-a-GCST90001711	rs143106266	BAFF-R on IgD- C	13	29908421
ebi-a-GCST90001711	rs2110699	BAFF-R on IgD- C	14	89904438
ebi-a-GCST90001711	rs145423893	BAFF-R on IgD- C	15	28254280
ebi-a-GCST90001711	rs142780018	BAFF-R on IgD- C	22	44395926
ebi-a-GCST90001711	rs528009599	BAFF-R on IgD- C	22	40568020
ebi-a-GCST90001711	rs191405504	BAFF-R on IgD- C	22	41305796
ebi-a-GCST90001711	rs73165129	BAFF-R on IgD- C	22	42315371
ebi-a-GCST90001711	rs13058590	BAFF-R on IgD- C	22	39641412
ebi-a-GCST90001711	rs146069723	BAFF-R on IgD- C	22	43339331
ebi-a-GCST90001711	rs5758649	BAFF-R on IgD- C	22	42605548
ebi-a-GCST90001711	rs9614288	BAFF-R on IgD- C	22	44297686
ebi-a-GCST90001711	rs6000850	BAFF-R on IgD- C	22	38032090
ebi-a-GCST90001711	rs113530150	BAFF-R on IgD- C	22	40567196
ebi-a-GCST90001711	rs150612106	BAFF-R on IgD- C	22	40175781
ebi-a-GCST90001712	rs4657041	BAFF-R on IgD- C	1	161478859
ebi-a-GCST90001712	rs116470335	BAFF-R on IgD- C	4	24492147
ebi-a-GCST90001712	rs62567851	BAFF-R on IgD- C	9	115279319
ebi-a-GCST90001712	rs56020760	BAFF-R on IgD- C	10	72391681
ebi-a-GCST90001712	rs118159778	BAFF-R on IgD- C	10	15099583
ebi-a-GCST90001712	rs61879160	BAFF-R on IgD- C	11	622044
ebi-a-GCST90001712	rs76400748	BAFF-R on IgD- C	11	131814009
ebi-a-GCST90001712	rs143106266	BAFF-R on IgD- C	13	29908421

ebi-a-GCST90001712	rs2110699	BAFF-R on IgD- C	14	89904438
ebi-a-GCST90001712	rs2142827	BAFF-R on IgD- C	22	42874781
ebi-a-GCST90001712	rs73165129	BAFF-R on IgD- C	22	42315371
ebi-a-GCST90001712	rs17032	BAFF-R on IgD- C	22	39130843
ebi-a-GCST90001712	rs5758649	BAFF-R on IgD- C	22	42605548
ebi-a-GCST90001712	rs528009599	BAFF-R on IgD- C	22	40568020
ebi-a-GCST90001712	rs75056525	BAFF-R on IgD- C	22	43349884
ebi-a-GCST90001712	rs117086217	BAFF-R on IgD- C	22	39876838
ebi-a-GCST90001712	rs142780018	BAFF-R on IgD- C	22	44395926
ebi-a-GCST90001712	rs9614288	BAFF-R on IgD- C	22	44297686
ebi-a-GCST90001713	rs2479085	BAFF-R on IgD- C	1	15667443
ebi-a-GCST90001713	rs643428	BAFF-R on IgD- C	1	54728858
ebi-a-GCST90001713	rs17828306	BAFF-R on IgD- C	4	62162828
ebi-a-GCST90001713	rs10071113	BAFF-R on IgD- C	5	178512644
ebi-a-GCST90001713	rs73269620	BAFF-R on IgD- C	7	13273064
ebi-a-GCST90001713	rs147645513	BAFF-R on IgD- C	7	32376606
ebi-a-GCST90001713	rs66717547	BAFF-R on IgD- C	8	122963696
ebi-a-GCST90001713	rs79868028	BAFF-R on IgD- C	10	133645848
ebi-a-GCST90001713	rs17878304	BAFF-R on IgD- C	19	4956161
ebi-a-GCST90001713	rs561920	BAFF-R on IgD- C	20	922125
ebi-a-GCST90001713	rs146069723	BAFF-R on IgD- C	22	43339331
ebi-a-GCST90001713	rs139784925	BAFF-R on IgD- C	22	41079533
ebi-a-GCST90001713	rs4822134	BAFF-R on IgD- C	22	42867332
ebi-a-GCST90001713	rs149192849	BAFF-R on IgD- C	22	42270864
ebi-a-GCST90001713	rs73165129	BAFF-R on IgD- C	22	42315371
ebi-a-GCST90001714	rs4657041	BAFF-R on IgD- C	1	161478859
ebi-a-GCST90001714	rs9434824	BAFF-R on IgD- C	1	7137637
ebi-a-GCST90001714	rs2925323	BAFF-R on IgD- C	3	101658926
ebi-a-GCST90001714	rs116470335	BAFF-R on IgD- C	4	24492147
ebi-a-GCST90001715	rs1801274	BAFF-R on memor	1	161479745
ebi-a-GCST90001715	rs72658947	BAFF-R on memor	1	30372026
ebi-a-GCST90001715	rs6680891	BAFF-R on memor	1	164083691
ebi-a-GCST90001715	rs2925323	BAFF-R on memor	3	101658926
ebi-a-GCST90001715	rs116470335	BAFF-R on memor	4	24492147
ebi-a-GCST90001715	rs56020760	BAFF-R on memor	10	72391681
ebi-a-GCST90001715	rs143106266	BAFF-R on memor	13	29908421
ebi-a-GCST90001715	rs13058590	BAFF-R on memor	22	39641412
ebi-a-GCST90001715	rs187315152	BAFF-R on memor	22	41305795
ebi-a-GCST90001715	rs146069723	BAFF-R on memor	22	43339331
ebi-a-GCST90001715	rs189503542	BAFF-R on memor	22	44698304
ebi-a-GCST90001715	rs528009599	BAFF-R on memor	22	40568020
ebi-a-GCST90001715	rs117086217	BAFF-R on memor	22	39876838
ebi-a-GCST90001715	rs149228586	BAFF-R on memor	22	41608058
ebi-a-GCST90001715	rs73165129	BAFF-R on memor	22	42315371
ebi-a-GCST90001715	rs5758649	BAFF-R on memor	22	42605548
ebi-a-GCST90001715	rs2142827	BAFF-R on memor	22	42874781
ebi-a-GCST90001715	rs9614288	BAFF-R on memor	22	44297686
ebi-a-GCST90001716	rs6680891	BAFF-R on naive-n	1	164083691

ebi-a-GCST90001716	rs1801274	BAFF-R on naive-n	1	161479745
ebi-a-GCST90001716	rs72658947	BAFF-R on naive-n	1	30372026
ebi-a-GCST90001716	rs149153670	BAFF-R on naive-n	2	142280835
ebi-a-GCST90001716	rs2925323	BAFF-R on naive-n	3	101658926
ebi-a-GCST90001716	rs116470335	BAFF-R on naive-n	4	24492147
ebi-a-GCST90001716	rs12529650	BAFF-R on naive-n	6	1751206
ebi-a-GCST90001716	rs56020760	BAFF-R on naive-n	10	72391681
ebi-a-GCST90001716	rs1563991	BAFF-R on naive-n	11	37670518
ebi-a-GCST90001716	rs143106266	BAFF-R on naive-n	13	29908421
ebi-a-GCST90001716	rs35226928	BAFF-R on naive-n	14	33787067
ebi-a-GCST90001716	rs148563640	BAFF-R on naive-n	17	48145906
ebi-a-GCST90001716	rs116674320	BAFF-R on naive-n	19	33884065
ebi-a-GCST90001716	rs149398740	BAFF-R on naive-n	22	23924845
ebi-a-GCST90001716	rs1081003	BAFF-R on naive-n	22	42525756
ebi-a-GCST90001716	rs76146864	BAFF-R on naive-n	22	40246055
ebi-a-GCST90001716	rs1894588	BAFF-R on naive-n	22	40629980
ebi-a-GCST90001716	rs117430146	BAFF-R on naive-n	22	41998660
ebi-a-GCST90001716	rs149806587	BAFF-R on naive-n	22	40774205
ebi-a-GCST90001716	rs117705111	BAFF-R on naive-n	22	41957510
ebi-a-GCST90001716	rs5759079	BAFF-R on naive-n	22	43393437
ebi-a-GCST90001716	rs5757678	BAFF-R on naive-n	22	39843409
ebi-a-GCST90001716	rs73165129	BAFF-R on naive-n	22	42315371
ebi-a-GCST90001717	rs1436776	BAFF-R on unsw m	1	216235074
ebi-a-GCST90001717	rs4657041	BAFF-R on unsw m	1	161478859
ebi-a-GCST90001717	rs6680891	BAFF-R on unsw m	1	164083691
ebi-a-GCST90001717	rs113510401	BAFF-R on unsw m	2	51662218
ebi-a-GCST90001717	rs2925323	BAFF-R on unsw m	3	101658926
ebi-a-GCST90001717	rs116470335	BAFF-R on unsw m	4	24492147
ebi-a-GCST90001717	rs190757416	BAFF-R on unsw m	6	45358923
ebi-a-GCST90001717	rs56020760	BAFF-R on unsw m	10	72391681
ebi-a-GCST90001717	rs116897208	BAFF-R on unsw m	10	15100608
ebi-a-GCST90001717	rs74643433	BAFF-R on unsw m	11	109385593
ebi-a-GCST90001717	rs143106266	BAFF-R on unsw m	13	29908421
ebi-a-GCST90001717	rs2110699	BAFF-R on unsw m	14	89904438
ebi-a-GCST90001717	rs1885397	BAFF-R on unsw m	14	55233098
ebi-a-GCST90001717	rs141410339	BAFF-R on unsw m	21	40298978
ebi-a-GCST90001717	rs187315152	BAFF-R on unsw m	22	41305795
ebi-a-GCST90001717	rs149228586	BAFF-R on unsw m	22	41608058
ebi-a-GCST90001717	rs5758649	BAFF-R on unsw m	22	42605548
ebi-a-GCST90001717	rs146069723	BAFF-R on unsw m	22	43339331
ebi-a-GCST90001717	rs13058590	BAFF-R on unsw m	22	39641412
ebi-a-GCST90001717	rs73165129	BAFF-R on unsw m	22	42315371
ebi-a-GCST90001717	rs2142827	BAFF-R on unsw m	22	42874781
ebi-a-GCST90001717	rs528009599	BAFF-R on unsw m	22	40568020
ebi-a-GCST90001717	rs9614288	BAFF-R on unsw m	22	44297686
ebi-a-GCST90001717	rs189503542	BAFF-R on unsw m	22	44698304
ebi-a-GCST90001717	rs117086217	BAFF-R on unsw m	22	39876838
ebi-a-GCST90001718	rs4657041	BAFF-R on sw mer	1	161478859

ebi-a-GCST90001718	rs9869373	BAFF-R on sw mer	3	101645001
ebi-a-GCST90001718	rs116470335	BAFF-R on sw mer	4	24492147
ebi-a-GCST90001718	rs10243011	BAFF-R on sw mer	7	121282913
ebi-a-GCST90001718	rs116897208	BAFF-R on sw mer	10	15100608
ebi-a-GCST90001718	rs76400748	BAFF-R on sw mer	11	131814009
ebi-a-GCST90001718	rs143106266	BAFF-R on sw mer	13	29908421
ebi-a-GCST90001718	rs2142827	BAFF-R on sw mer	22	42874781
ebi-a-GCST90001718	rs143944768	BAFF-R on sw mer	22	44378670
ebi-a-GCST90001718	rs149228586	BAFF-R on sw mer	22	41608058
ebi-a-GCST90001718	rs9614288	BAFF-R on sw mer	22	44297686
ebi-a-GCST90001718	rs5758649	BAFF-R on sw mer	22	42605548
ebi-a-GCST90001718	rs146069723	BAFF-R on sw mer	22	43339331
ebi-a-GCST90001718	rs117086217	BAFF-R on sw mer	22	39876838
ebi-a-GCST90001718	rs528009599	BAFF-R on sw mer	22	40568020
ebi-a-GCST90001718	rs187315152	BAFF-R on sw mer	22	41305795
ebi-a-GCST90001718	rs73165129	BAFF-R on sw mer	22	42315371
ebi-a-GCST90001718	rs149398740	BAFF-R on sw mer	22	23924845
ebi-a-GCST90001718	rs185794015	BAFF-R on sw mer	22	44440180
ebi-a-GCST90001718	rs13058590	BAFF-R on sw mer	22	39641412
ebi-a-GCST90001719	rs6666916	BAFF-R on IgD+	1	86007429
ebi-a-GCST90001719	rs72658947	BAFF-R on IgD+	1	30372026
ebi-a-GCST90001719	rs6680891	BAFF-R on IgD+	1	164083691
ebi-a-GCST90001719	rs1801274	BAFF-R on IgD+	1	161479745
ebi-a-GCST90001719	rs114318843	BAFF-R on IgD+	2	164755835
ebi-a-GCST90001719	rs149153670	BAFF-R on IgD+	2	142280835
ebi-a-GCST90001719	rs2925323	BAFF-R on IgD+	3	101658926
ebi-a-GCST90001719	rs116470335	BAFF-R on IgD+	4	24492147
ebi-a-GCST90001719	rs1563991	BAFF-R on IgD+	11	37670518
ebi-a-GCST90001719	rs143106266	BAFF-R on IgD+	13	29908421
ebi-a-GCST90001719	rs1609762	BAFF-R on IgD+	14	33787438
ebi-a-GCST90001719	rs1885397	BAFF-R on IgD+	14	55233098
ebi-a-GCST90001719	rs35754161	BAFF-R on IgD+	15	51655048
ebi-a-GCST90001719	rs148563640	BAFF-R on IgD+	17	48145906
ebi-a-GCST90001719	rs76146864	BAFF-R on IgD+	22	40246055
ebi-a-GCST90001719	rs5757678	BAFF-R on IgD+	22	39843409
ebi-a-GCST90001719	rs73165129	BAFF-R on IgD+	22	42315371
ebi-a-GCST90001719	rs1894588	BAFF-R on IgD+	22	40629980
ebi-a-GCST90001719	rs1081003	BAFF-R on IgD+	22	42525756
ebi-a-GCST90001719	rs117705111	BAFF-R on IgD+	22	41957510
ebi-a-GCST90001719	rs117430146	BAFF-R on IgD+	22	41998660
ebi-a-GCST90001719	rs149806587	BAFF-R on IgD+	22	40774205
ebi-a-GCST90001719	rs5759079	BAFF-R on IgD+	22	43393437
ebi-a-GCST90001719	rs149398740	BAFF-R on IgD+	22	23924845
ebi-a-GCST90001720	rs1801274	BAFF-R on transiti	1	161479745
ebi-a-GCST90001720	rs114318843	BAFF-R on transiti	2	164755835
ebi-a-GCST90001720	rs141130967	BAFF-R on transiti	3	101706582
ebi-a-GCST90001720	rs116023377	BAFF-R on transiti	4	18858102
ebi-a-GCST90001720	rs57756059	BAFF-R on transiti	4	77500796

ebi-a-GCST90001720	rs4252435	BAFF-R on transiti	7	142622714
ebi-a-GCST90001720	rs11761810	BAFF-R on transiti	7	152779596
ebi-a-GCST90001720	rs34006338	BAFF-R on transiti	7	45957318
ebi-a-GCST90001720	rs6998646	BAFF-R on transiti	8	127759740
ebi-a-GCST90001720	rs77803489	BAFF-R on transiti	10	14893495
ebi-a-GCST90001720	rs1563991	BAFF-R on transiti	11	37670518
ebi-a-GCST90001720	rs143106266	BAFF-R on transiti	13	29908421
ebi-a-GCST90001720	rs56172578	BAFF-R on transiti	14	22597739
ebi-a-GCST90001720	rs16957279	BAFF-R on transiti	15	33954227
ebi-a-GCST90001720	rs148563640	BAFF-R on transiti	17	48145906
ebi-a-GCST90001720	rs117499379	BAFF-R on transiti	18	4343023
ebi-a-GCST90001720	rs1081003	BAFF-R on transiti	22	42525756
ebi-a-GCST90001720	rs5759079	BAFF-R on transiti	22	43393437
ebi-a-GCST90001720	rs1894588	BAFF-R on transiti	22	40629980
ebi-a-GCST90001720	rs117705111	BAFF-R on transiti	22	41957510
ebi-a-GCST90001720	rs76146864	BAFF-R on transiti	22	40246055
ebi-a-GCST90001720	rs5757678	BAFF-R on transiti	22	39843409
ebi-a-GCST90001720	rs117430146	BAFF-R on transiti	22	41998660
ebi-a-GCST90001720	rs149806587	BAFF-R on transiti	22	40774205
ebi-a-GCST90001720	rs73165129	BAFF-R on transiti	22	42315371
ebi-a-GCST90001721	rs664654	CD19 on CD20-	1	12185036
ebi-a-GCST90001721	rs536003	CD19 on CD20-	1	40154898
ebi-a-GCST90001721	rs13390788	CD19 on CD20-	2	235624835
ebi-a-GCST90001721	rs7580866	CD19 on CD20-	2	202479783
ebi-a-GCST90001721	rs111494817	CD19 on CD20-	3	49120006
ebi-a-GCST90001721	rs72901497	CD19 on CD20-	4	115677333
ebi-a-GCST90001721	rs2098676	CD19 on CD20-	5	172763674
ebi-a-GCST90001721	rs183942882	CD19 on CD20-	5	26009776
ebi-a-GCST90001721	rs11748905	CD19 on CD20-	5	80934634
ebi-a-GCST90001721	rs149522199	CD19 on CD20-	7	134114882
ebi-a-GCST90001721	rs55973216	CD19 on CD20-	7	21380419
ebi-a-GCST90001721	rs13429	CD19 on CD20-	8	440424
ebi-a-GCST90001721	rs16927639	CD19 on CD20-	8	62521732
ebi-a-GCST90001721	rs17248848	CD19 on CD20-	8	107222079
ebi-a-GCST90001721	rs1541174	CD19 on CD20-	10	130287175
ebi-a-GCST90001721	rs6583759	CD19 on CD20-	10	92976570
ebi-a-GCST90001721	rs41324346	CD19 on CD20-	10	89070745
ebi-a-GCST90001721	rs12318478	CD19 on CD20-	12	20830424
ebi-a-GCST90001721	rs997173	CD19 on CD20-	12	55355033
ebi-a-GCST90001721	rs1239945	CD19 on CD20-	13	51107757
ebi-a-GCST90001721	rs77441302	CD19 on CD20-	18	40519517
ebi-a-GCST90001721	rs164020	CD19 on CD20-	19	6818628
ebi-a-GCST90001722	rs6658147	CD19 on CD20- CI	1	18905298
ebi-a-GCST90001722	rs11161637	CD19 on CD20- CI	1	86021169
ebi-a-GCST90001722	rs41267263	CD19 on CD20- CI	1	36335349
ebi-a-GCST90001722	rs13391592	CD19 on CD20- CI	2	180409011
ebi-a-GCST90001722	rs113045391	CD19 on CD20- CI	2	201721041
ebi-a-GCST90001722	rs78401267	CD19 on CD20- CI	3	189064197

ebi-a-GCST90001722	rs62290470	CD19 on CD20- CI	4	5132403
ebi-a-GCST90001722	rs62413604	CD19 on CD20- CI	6	49652991
ebi-a-GCST90001722	rs9402148	CD19 on CD20- CI	6	129929521
ebi-a-GCST90001722	rs116862171	CD19 on CD20- CI	6	112145789
ebi-a-GCST90001722	rs17480133	CD19 on CD20- CI	7	145783861
ebi-a-GCST90001722	rs150625190	CD19 on CD20- CI	13	92727041
ebi-a-GCST90001722	rs72664677	CD19 on CD20- CI	14	32638300
ebi-a-GCST90001722	rs75455523	CD19 on CD20- CI	15	96941966
ebi-a-GCST90001722	rs12438058	CD19 on CD20- CI	15	39819219
ebi-a-GCST90001722	rs1966917	CD19 on CD20- CI	17	72108055
ebi-a-GCST90001722	rs113932257	CD19 on CD20- CI	18	3127438
ebi-a-GCST90001722	rs67130681	CD19 on CD20- CI	18	71598971
ebi-a-GCST90001722	rs10426452	CD19 on CD20- CI	19	49907690
ebi-a-GCST90001722	rs7280618	CD19 on CD20- CI	21	41565479
ebi-a-GCST90001722	rs219712	CD19 on CD20- CI	21	27751176
ebi-a-GCST90001722	rs113004536	CD19 on CD20- CI	22	18923443
ebi-a-GCST90001723	rs6678796	CD19 on CD24+ C	1	247157835
ebi-a-GCST90001723	rs10917581	CD19 on CD24+ C	1	162624504
ebi-a-GCST90001723	rs113812519	CD19 on CD24+ C	1	207276336
ebi-a-GCST90001723	rs112417310	CD19 on CD24+ C	1	206855483
ebi-a-GCST90001723	rs589151	CD19 on CD24+ C	1	10567711
ebi-a-GCST90001723	rs4649079	CD19 on CD24+ C	1	25535823
ebi-a-GCST90001723	rs144743804	CD19 on CD24+ C	1	207018206
ebi-a-GCST90001723	rs80268044	CD19 on CD24+ C	2	43810948
ebi-a-GCST90001723	rs1439005	CD19 on CD24+ C	3	61094800
ebi-a-GCST90001723	rs7665261	CD19 on CD24+ C	4	26464043
ebi-a-GCST90001723	rs281142	CD19 on CD24+ C	5	70969845
ebi-a-GCST90001723	rs7718237	CD19 on CD24+ C	5	173972852
ebi-a-GCST90001723	rs764603	CD19 on CD24+ C	6	36793516
ebi-a-GCST90001723	rs35553650	CD19 on CD24+ C	6	37456130
ebi-a-GCST90001723	rs9350187	CD19 on CD24+ C	6	19794487
ebi-a-GCST90001723	rs76162621	CD19 on CD24+ C	6	68486690
ebi-a-GCST90001723	rs78007482	CD19 on CD24+ C	8	133189482
ebi-a-GCST90001723	rs12554596	CD19 on CD24+ C	9	37002142
ebi-a-GCST90001723	rs560933055	CD19 on CD24+ C	10	1717689
ebi-a-GCST90001723	rs17706571	CD19 on CD24+ C	12	29271392
ebi-a-GCST90001723	rs149168550	CD19 on CD24+ C	12	111354000
ebi-a-GCST90001723	rs118134186	CD19 on CD24+ C	13	29813364
ebi-a-GCST90001723	rs6575228	CD19 on CD24+ C	14	92561395
ebi-a-GCST90001723	rs138920008	CD19 on CD24+ C	16	32422808
ebi-a-GCST90001723	rs11643297	CD19 on CD24+ C	16	28973268
ebi-a-GCST90001723	rs1424083	CD19 on CD24+ C	16	74304865
ebi-a-GCST90001723	rs112846834	CD19 on CD24+ C	17	7725660
ebi-a-GCST90001723	rs72988602	CD19 on CD24+ C	19	7114038
ebi-a-GCST90001723	rs2903728	CD19 on CD24+ C	19	56408295
ebi-a-GCST90001723	rs117606004	CD19 on CD24+ C	20	44812456
ebi-a-GCST90001723	rs5762000	CD19 on CD24+ C	22	27530934
ebi-a-GCST90001723	rs113612962	CD19 on CD24+ C	22	43167446

ebi-a-GCST90001724	rs55712453	CD19 on IgD+ CD2	1	206902437
ebi-a-GCST90001724	rs6678796	CD19 on IgD+ CD2	1	247157835
ebi-a-GCST90001724	rs4649080	CD19 on IgD+ CD2	1	25737116
ebi-a-GCST90001724	rs113812519	CD19 on IgD+ CD2	1	207276336
ebi-a-GCST90001724	rs144743804	CD19 on IgD+ CD2	1	207018206
ebi-a-GCST90001724	rs140108418	CD19 on IgD+ CD2	2	13573112
ebi-a-GCST90001724	rs80268044	CD19 on IgD+ CD2	2	43810948
ebi-a-GCST90001724	rs9814135	CD19 on IgD+ CD2	3	194094110
ebi-a-GCST90001724	rs1595602	CD19 on IgD+ CD2	3	61090473
ebi-a-GCST90001724	rs7665261	CD19 on IgD+ CD2	4	26464043
ebi-a-GCST90001724	rs7718237	CD19 on IgD+ CD2	5	173972852
ebi-a-GCST90001724	rs77175237	CD19 on IgD+ CD2	5	72039498
ebi-a-GCST90001724	rs764603	CD19 on IgD+ CD2	6	36793516
ebi-a-GCST90001724	rs76162621	CD19 on IgD+ CD2	6	68486690
ebi-a-GCST90001724	rs7766104	CD19 on IgD+ CD2	6	105665214
ebi-a-GCST90001724	rs73393708	CD19 on IgD+ CD2	7	90292336
ebi-a-GCST90001724	rs78007482	CD19 on IgD+ CD2	8	133189482
ebi-a-GCST90001724	rs12554596	CD19 on IgD+ CD2	9	37002142
ebi-a-GCST90001724	rs10999014	CD19 on IgD+ CD2	10	71648522
ebi-a-GCST90001724	rs117006645	CD19 on IgD+ CD2	10	108143796
ebi-a-GCST90001724	rs28560225	CD19 on IgD+ CD2	10	125700760
ebi-a-GCST90001724	rs6602758	CD19 on IgD+ CD2	10	14778920
ebi-a-GCST90001724	rs560933055	CD19 on IgD+ CD2	10	1717689
ebi-a-GCST90001724	rs118075417	CD19 on IgD+ CD2	10	109625907
ebi-a-GCST90001724	rs871361	CD19 on IgD+ CD2	12	124563662
ebi-a-GCST90001724	rs4942349	CD19 on IgD+ CD2	13	31564105
ebi-a-GCST90001724	rs6575228	CD19 on IgD+ CD2	14	92561395
ebi-a-GCST90001724	rs7195635	CD19 on IgD+ CD2	16	58513959
ebi-a-GCST90001724	rs138920008	CD19 on IgD+ CD2	16	32422808
ebi-a-GCST90001724	rs11643297	CD19 on IgD+ CD2	16	28973268
ebi-a-GCST90001724	rs9892181	CD19 on IgD+ CD2	17	76534817
ebi-a-GCST90001724	rs148381451	CD19 on IgD+ CD2	17	63085557
ebi-a-GCST90001724	rs72988602	CD19 on IgD+ CD2	19	7114038
ebi-a-GCST90001724	rs77697711	CD19 on IgD+ CD2	22	45116977
ebi-a-GCST90001724	rs113612962	CD19 on IgD+ CD2	22	43167446
ebi-a-GCST90001724	rs5762000	CD19 on IgD+ CD2	22	27530934
ebi-a-GCST90001725	rs79856590	CD19 on IgD+ CD2	1	205241642
ebi-a-GCST90001725	rs113812519	CD19 on IgD+ CD2	1	207276336
ebi-a-GCST90001725	rs11120838	CD19 on IgD+ CD2	1	7149639
ebi-a-GCST90001725	rs142109788	CD19 on IgD+ CD2	1	149815561
ebi-a-GCST90001725	rs112417310	CD19 on IgD+ CD2	1	206855483
ebi-a-GCST90001725	rs1345010	CD19 on IgD+ CD2	2	174113733
ebi-a-GCST90001725	rs145392098	CD19 on IgD+ CD2	2	95682916
ebi-a-GCST90001725	rs1449311	CD19 on IgD+ CD2	3	100703124
ebi-a-GCST90001725	rs115727814	CD19 on IgD+ CD2	4	170699036
ebi-a-GCST90001725	rs764603	CD19 on IgD+ CD2	6	36793516
ebi-a-GCST90001725	rs78895027	CD19 on IgD+ CD2	6	112306917
ebi-a-GCST90001725	rs9639817	CD19 on IgD+ CD2	7	40085765

ebi-a-GCST90001725	rs12216684	CD19 on IgD+ CD2	7	56291533
ebi-a-GCST90001725	rs80273434	CD19 on IgD+ CD2	8	141772905
ebi-a-GCST90001725	rs75912463	CD19 on IgD+ CD2	9	133354402
ebi-a-GCST90001725	rs34798965	CD19 on IgD+ CD2	10	71647692
ebi-a-GCST90001725	rs67316035	CD19 on IgD+ CD2	11	132221605
ebi-a-GCST90001725	rs17493333	CD19 on IgD+ CD2	12	19098939
ebi-a-GCST90001725	rs754067	CD19 on IgD+ CD2	16	17323672
ebi-a-GCST90001725	rs138920008	CD19 on IgD+ CD2	16	32422808
ebi-a-GCST90001725	rs148381451	CD19 on IgD+ CD2	17	63085557
ebi-a-GCST90001725	rs1290397	CD19 on IgD+ CD2	17	77948063
ebi-a-GCST90001725	rs1986507	CD19 on IgD+ CD2	17	10082301
ebi-a-GCST90001725	rs8109226	CD19 on IgD+ CD2	19	710050
ebi-a-GCST90001725	rs2903728	CD19 on IgD+ CD2	19	56408295
ebi-a-GCST90001725	rs112152495	CD19 on IgD+ CD2	19	7096773
ebi-a-GCST90001726	rs55712453	CD19 on IgD+ CD3	1	206902437
ebi-a-GCST90001726	rs34762764	CD19 on IgD+ CD3	1	47996882
ebi-a-GCST90001726	rs142109788	CD19 on IgD+ CD3	1	149815561
ebi-a-GCST90001726	rs113812519	CD19 on IgD+ CD3	1	207276336
ebi-a-GCST90001726	rs79856590	CD19 on IgD+ CD3	1	205241642
ebi-a-GCST90001726	rs11733521	CD19 on IgD+ CD3	4	68968349
ebi-a-GCST90001726	rs115727814	CD19 on IgD+ CD3	4	170699036
ebi-a-GCST90001726	rs6554147	CD19 on IgD+ CD3	4	54958646
ebi-a-GCST90001726	rs186239326	CD19 on IgD+ CD3	6	2511082
ebi-a-GCST90001726	rs6908848	CD19 on IgD+ CD3	6	14717247
ebi-a-GCST90001726	rs11764670	CD19 on IgD+ CD3	7	76455763
ebi-a-GCST90001726	rs118033073	CD19 on IgD+ CD3	8	9086777
ebi-a-GCST90001726	rs62524107	CD19 on IgD+ CD3	8	141805137
ebi-a-GCST90001726	rs12554596	CD19 on IgD+ CD3	9	37002142
ebi-a-GCST90001726	rs10738775	CD19 on IgD+ CD3	9	27514318
ebi-a-GCST90001726	rs75912463	CD19 on IgD+ CD3	9	133354402
ebi-a-GCST90001726	rs6602758	CD19 on IgD+ CD3	10	14778920
ebi-a-GCST90001726	rs112802874	CD19 on IgD+ CD3	10	89640021
ebi-a-GCST90001726	rs11051699	CD19 on IgD+ CD3	12	32107411
ebi-a-GCST90001726	rs74518629	CD19 on IgD+ CD3	12	130845523
ebi-a-GCST90001726	rs75382958	CD19 on IgD+ CD3	13	70194834
ebi-a-GCST90001726	rs117004076	CD19 on IgD+ CD3	14	97702226
ebi-a-GCST90001726	rs7496584	CD19 on IgD+ CD3	15	23877607
ebi-a-GCST90001726	rs78044532	CD19 on IgD+ CD3	16	21168467
ebi-a-GCST90001726	rs118099810	CD19 on IgD+ CD3	16	2406183
ebi-a-GCST90001726	rs138920008	CD19 on IgD+ CD3	16	32422808
ebi-a-GCST90001726	rs2356414	CD19 on IgD+ CD3	16	71378301
ebi-a-GCST90001726	rs148381451	CD19 on IgD+ CD3	17	63085557
ebi-a-GCST90001726	rs6567179	CD19 on IgD+ CD3	18	58273913
ebi-a-GCST90001726	rs73056862	CD19 on IgD+ CD3	19	52235616
ebi-a-GCST90001726	rs34488330	CD19 on IgD+ CD3	19	7105841
ebi-a-GCST90001726	rs144392728	CD19 on IgD+ CD3	22	33029820
ebi-a-GCST90001727	rs142109788	CD19 on IgD+ CD3	1	149815561
ebi-a-GCST90001727	rs75320486	CD19 on IgD+ CD3	1	206842241

ebi-a-GCST90001727	rs113812519	CD19 on IgD+ CD3	1	207276336
ebi-a-GCST90001727	rs10754999	CD19 on IgD+ CD3	2	184906937
ebi-a-GCST90001727	rs11926501	CD19 on IgD+ CD3	3	9722257
ebi-a-GCST90001727	rs402710	CD19 on IgD+ CD3	5	1320722
ebi-a-GCST90001727	rs78895027	CD19 on IgD+ CD3	6	112306917
ebi-a-GCST90001727	rs28721574	CD19 on IgD+ CD3	6	29709714
ebi-a-GCST90001727	rs16939238	CD19 on IgD+ CD3	8	77030281
ebi-a-GCST90001727	rs75912463	CD19 on IgD+ CD3	9	133354402
ebi-a-GCST90001727	rs1687384	CD19 on IgD+ CD3	9	117088120
ebi-a-GCST90001727	rs72716013	CD19 on IgD+ CD3	9	71165158
ebi-a-GCST90001727	rs117004076	CD19 on IgD+ CD3	14	97702226
ebi-a-GCST90001727	rs4074448	CD19 on IgD+ CD3	15	63888495
ebi-a-GCST90001727	rs17637675	CD19 on IgD+ CD3	16	79029574
ebi-a-GCST90001727	rs12451409	CD19 on IgD+ CD3	17	45544856
ebi-a-GCST90001727	rs80090296	CD19 on IgD+ CD3	18	42110459
ebi-a-GCST90001727	rs4022330	CD19 on IgD+ CD3	19	54781843
ebi-a-GCST90001727	rs6127744	CD19 on IgD+ CD3	20	54986274
ebi-a-GCST90001728	rs10494895	CD19 on IgD+ CD3	1	208466901
ebi-a-GCST90001728	rs2419379	CD19 on IgD+ CD3	1	190433277
ebi-a-GCST90001728	rs113812519	CD19 on IgD+ CD3	1	207276336
ebi-a-GCST90001728	rs138155833	CD19 on IgD+ CD3	1	207698087
ebi-a-GCST90001728	rs7525464	CD19 on IgD+ CD3	1	154727881
ebi-a-GCST90001728	rs55712453	CD19 on IgD+ CD3	1	206902437
ebi-a-GCST90001728	rs144743804	CD19 on IgD+ CD3	1	207018206
ebi-a-GCST90001728	rs859655	CD19 on IgD+ CD3	1	172650450
ebi-a-GCST90001728	rs72999235	CD19 on IgD+ CD3	2	231874716
ebi-a-GCST90001728	rs140108418	CD19 on IgD+ CD3	2	13573112
ebi-a-GCST90001728	rs7600804	CD19 on IgD+ CD3	2	85912030
ebi-a-GCST90001728	rs200576624	CD19 on IgD+ CD3	4	110142387
ebi-a-GCST90001728	rs140761841	CD19 on IgD+ CD3	4	747378
ebi-a-GCST90001728	rs4702668	CD19 on IgD+ CD3	5	10081219
ebi-a-GCST90001728	rs148774606	CD19 on IgD+ CD3	5	149841015
ebi-a-GCST90001728	rs77518858	CD19 on IgD+ CD3	6	15735864
ebi-a-GCST90001728	rs80168996	CD19 on IgD+ CD3	7	44882040
ebi-a-GCST90001728	rs78653777	CD19 on IgD+ CD3	8	3472861
ebi-a-GCST90001728	rs13250240	CD19 on IgD+ CD3	8	130552677
ebi-a-GCST90001728	rs11840245	CD19 on IgD+ CD3	13	111300129
ebi-a-GCST90001728	rs11617402	CD19 on IgD+ CD3	13	22970185
ebi-a-GCST90001728	rs4900483	CD19 on IgD+ CD3	14	101566637
ebi-a-GCST90001728	rs17723384	CD19 on IgD+ CD3	16	34579231
ebi-a-GCST90001728	rs61610275	CD19 on IgD+ CD3	17	13040257
ebi-a-GCST90001728	rs9945791	CD19 on IgD+ CD3	18	36882371
ebi-a-GCST90001729	rs112417310	CD19 on IgD+ CD3	1	206855483
ebi-a-GCST90001729	rs144743804	CD19 on IgD+ CD3	1	207018206
ebi-a-GCST90001729	rs2026774	CD19 on IgD+ CD3	1	86076069
ebi-a-GCST90001729	rs113812519	CD19 on IgD+ CD3	1	207276336
ebi-a-GCST90001729	rs10737202	CD19 on IgD+ CD3	1	234499381
ebi-a-GCST90001729	rs13030427	CD19 on IgD+ CD3	2	208879261

ebi-a-GCST90001729	rs9880620	CD19 on IgD+ CD3	3	61111881
ebi-a-GCST90001729	rs4518167	CD19 on IgD+ CD3	3	97446508
ebi-a-GCST90001729	rs115761380	CD19 on IgD+ CD3	3	32376405
ebi-a-GCST90001729	rs72721695	CD19 on IgD+ CD3	4	153625808
ebi-a-GCST90001729	rs76162621	CD19 on IgD+ CD3	6	68486690
ebi-a-GCST90001729	rs2516390	CD19 on IgD+ CD3	6	31529883
ebi-a-GCST90001729	rs12216684	CD19 on IgD+ CD3	7	56291533
ebi-a-GCST90001729	rs67316035	CD19 on IgD+ CD3	11	132221605
ebi-a-GCST90001729	rs117442206	CD19 on IgD+ CD3	12	25430787
ebi-a-GCST90001729	rs2204990	CD19 on IgD+ CD3	14	22791563
ebi-a-GCST90001729	rs138920008	CD19 on IgD+ CD3	16	32422808
ebi-a-GCST90001729	rs148381451	CD19 on IgD+ CD3	17	63085557
ebi-a-GCST90001729	rs187374346	CD19 on IgD+ CD3	19	7091080
ebi-a-GCST90001729	rs59977691	CD19 on IgD+ CD3	19	19012358
ebi-a-GCST90001729	rs137949497	CD19 on IgD+ CD3	21	32136760
ebi-a-GCST90001730	rs142109788	CD19 on IgD+ CD3	1	149815561
ebi-a-GCST90001730	rs113812519	CD19 on IgD+ CD3	1	207276336
ebi-a-GCST90001730	rs112417310	CD19 on IgD+ CD3	1	206855483
ebi-a-GCST90001730	rs144743804	CD19 on IgD+ CD3	1	207018206
ebi-a-GCST90001730	rs1345010	CD19 on IgD+ CD3	2	174113733
ebi-a-GCST90001730	rs143403164	CD19 on IgD+ CD3	3	111246616
ebi-a-GCST90001730	rs1449311	CD19 on IgD+ CD3	3	100703124
ebi-a-GCST90001730	rs115727814	CD19 on IgD+ CD3	4	170699036
ebi-a-GCST90001730	rs12642193	CD19 on IgD+ CD3	4	25916457
ebi-a-GCST90001730	rs13164259	CD19 on IgD+ CD3	5	82870211
ebi-a-GCST90001730	rs764603	CD19 on IgD+ CD3	6	36793516
ebi-a-GCST90001730	rs2524090	CD19 on IgD+ CD3	6	31240823
ebi-a-GCST90001730	rs12216684	CD19 on IgD+ CD3	7	56291533
ebi-a-GCST90001730	rs80273434	CD19 on IgD+ CD3	8	141772905
ebi-a-GCST90001730	rs75912463	CD19 on IgD+ CD3	9	133354402
ebi-a-GCST90001730	rs76289004	CD19 on IgD+ CD3	10	67011620
ebi-a-GCST90001730	rs11825488	CD19 on IgD+ CD3	11	72165045
ebi-a-GCST90001730	rs67316035	CD19 on IgD+ CD3	11	132221605
ebi-a-GCST90001730	rs79699942	CD19 on IgD+ CD3	12	44929593
ebi-a-GCST90001730	rs1955142	CD19 on IgD+ CD3	15	65505785
ebi-a-GCST90001730	rs754067	CD19 on IgD+ CD3	16	17323672
ebi-a-GCST90001730	rs138920008	CD19 on IgD+ CD3	16	32422808
ebi-a-GCST90001730	rs148381451	CD19 on IgD+ CD3	17	63085557
ebi-a-GCST90001730	rs1290397	CD19 on IgD+ CD3	17	77948063
ebi-a-GCST90001730	rs2903728	CD19 on IgD+ CD3	19	56408295
ebi-a-GCST90001731	rs10192011	CD19 on IgD- CD2	2	53804261
ebi-a-GCST90001731	rs10928415	CD19 on IgD- CD2	2	149553139
ebi-a-GCST90001731	rs242172	CD19 on IgD- CD2	3	60405606
ebi-a-GCST90001731	rs631691	CD19 on IgD- CD2	3	124875178
ebi-a-GCST90001731	rs71307996	CD19 on IgD- CD2	3	1103352
ebi-a-GCST90001731	rs76451648	CD19 on IgD- CD2	4	139618949
ebi-a-GCST90001731	rs73254441	CD19 on IgD- CD2	5	113295200
ebi-a-GCST90001731	rs16884870	CD19 on IgD- CD2	6	75109409

ebi-a-GCST90001731	rs148597489	CD19 on IgD- CD2	6	72550483
ebi-a-GCST90001731	rs75663256	CD19 on IgD- CD2	8	69895735
ebi-a-GCST90001731	rs10906006	CD19 on IgD- CD2	10	11781967
ebi-a-GCST90001731	rs61884722	CD19 on IgD- CD2	11	18601510
ebi-a-GCST90001731	rs1164128	CD19 on IgD- CD2	13	112057432
ebi-a-GCST90001731	rs7332415	CD19 on IgD- CD2	13	41688557
ebi-a-GCST90001731	rs148027681	CD19 on IgD- CD2	13	61899478
ebi-a-GCST90001731	rs76709166	CD19 on IgD- CD2	13	20589957
ebi-a-GCST90001731	rs17315418	CD19 on IgD- CD2	15	70028833
ebi-a-GCST90001731	rs11643297	CD19 on IgD- CD2	16	28973268
ebi-a-GCST90001731	rs1453509	CD19 on IgD- CD2	16	17014673
ebi-a-GCST90001731	rs12968867	CD19 on IgD- CD2	18	60850310
ebi-a-GCST90001731	rs2613786	CD19 on IgD- CD2	19	5137846
ebi-a-GCST90001731	rs73056862	CD19 on IgD- CD2	19	52235616
ebi-a-GCST90001731	rs75656751	CD19 on IgD- CD2	21	38055091
ebi-a-GCST90001732	rs6577565	CD19 on IgD- CD2	1	6307255
ebi-a-GCST90001732	rs113747920	CD19 on IgD- CD2	1	27453519
ebi-a-GCST90001732	rs59102092	CD19 on IgD- CD2	1	39551423
ebi-a-GCST90001732	rs1524155	CD19 on IgD- CD2	2	48878727
ebi-a-GCST90001732	rs10192011	CD19 on IgD- CD2	2	53804261
ebi-a-GCST90001732	rs7608595	CD19 on IgD- CD2	2	223088415
ebi-a-GCST90001732	rs34205222	CD19 on IgD- CD2	3	1066077
ebi-a-GCST90001732	rs242172	CD19 on IgD- CD2	3	60405606
ebi-a-GCST90001732	rs12636323	CD19 on IgD- CD2	3	100795543
ebi-a-GCST90001732	rs631691	CD19 on IgD- CD2	3	124875178
ebi-a-GCST90001732	rs115952864	CD19 on IgD- CD2	4	173244216
ebi-a-GCST90001732	rs116412781	CD19 on IgD- CD2	4	2746567
ebi-a-GCST90001732	rs144574165	CD19 on IgD- CD2	4	163336202
ebi-a-GCST90001732	rs142291539	CD19 on IgD- CD2	5	175612761
ebi-a-GCST90001732	rs9350187	CD19 on IgD- CD2	6	19794487
ebi-a-GCST90001732	rs71555251	CD19 on IgD- CD2	6	75109171
ebi-a-GCST90001732	rs62463053	CD19 on IgD- CD2	7	51994027
ebi-a-GCST90001732	rs75663256	CD19 on IgD- CD2	8	69895735
ebi-a-GCST90001732	rs10216993	CD19 on IgD- CD2	8	142521360
ebi-a-GCST90001732	rs10821075	CD19 on IgD- CD2	9	95896099
ebi-a-GCST90001732	rs61884722	CD19 on IgD- CD2	11	18601510
ebi-a-GCST90001732	rs148390689	CD19 on IgD- CD2	13	20089809
ebi-a-GCST90001732	rs114723901	CD19 on IgD- CD2	15	45169337
ebi-a-GCST90001732	rs11643297	CD19 on IgD- CD2	16	28973268
ebi-a-GCST90001732	rs138920008	CD19 on IgD- CD2	16	32422808
ebi-a-GCST90001732	rs12968867	CD19 on IgD- CD2	18	60850310
ebi-a-GCST90001732	rs113612962	CD19 on IgD- CD2	22	43167446
ebi-a-GCST90001733	rs4927044	CD19 on IgD- CD3	1	48232179
ebi-a-GCST90001733	rs112978597	CD19 on IgD- CD3	1	12881809
ebi-a-GCST90001733	rs190416390	CD19 on IgD- CD3	2	159567199
ebi-a-GCST90001733	rs72877897	CD19 on IgD- CD3	3	61116118
ebi-a-GCST90001733	rs4702668	CD19 on IgD- CD3	5	10081219
ebi-a-GCST90001733	rs67145622	CD19 on IgD- CD3	6	19588752

ebi-a-GCST90001733	rs114369132	CD19 on IgD- CD3	6	32409141
ebi-a-GCST90001733	rs16884870	CD19 on IgD- CD3	6	75109409
ebi-a-GCST90001733	rs62463053	CD19 on IgD- CD3	7	51994027
ebi-a-GCST90001733	rs273144	CD19 on IgD- CD3	7	41508880
ebi-a-GCST90001733	rs75663256	CD19 on IgD- CD3	8	69895735
ebi-a-GCST90001733	rs964936	CD19 on IgD- CD3	8	142522071
ebi-a-GCST90001733	rs12554596	CD19 on IgD- CD3	9	37002142
ebi-a-GCST90001733	rs10761194	CD19 on IgD- CD3	9	95899884
ebi-a-GCST90001733	rs143330436	CD19 on IgD- CD3	9	26801194
ebi-a-GCST90001733	rs12346584	CD19 on IgD- CD3	9	97103437
ebi-a-GCST90001733	rs112522781	CD19 on IgD- CD3	10	89650801
ebi-a-GCST90001733	rs1164128	CD19 on IgD- CD3	13	112057432
ebi-a-GCST90001733	rs61154235	CD19 on IgD- CD3	14	77350459
ebi-a-GCST90001733	rs12904834	CD19 on IgD- CD3	15	76897663
ebi-a-GCST90001733	rs7189927	CD19 on IgD- CD3	16	28913787
ebi-a-GCST90001733	rs2613786	CD19 on IgD- CD3	19	5137846
ebi-a-GCST90001733	rs10420460	CD19 on IgD- CD3	19	57259311
ebi-a-GCST90001733	rs113612962	CD19 on IgD- CD3	22	43167446
ebi-a-GCST90001733	rs5762000	CD19 on IgD- CD3	22	27530934
ebi-a-GCST90001734	rs10752607	CD19 on IgD- CD3	1	154716803
ebi-a-GCST90001734	rs72816772	CD19 on IgD- CD3	2	75815457
ebi-a-GCST90001734	rs149234257	CD19 on IgD- CD3	2	7498941
ebi-a-GCST90001734	rs6805030	CD19 on IgD- CD3	3	54576549
ebi-a-GCST90001734	rs12374328	CD19 on IgD- CD3	4	61352640
ebi-a-GCST90001734	rs254079	CD19 on IgD- CD3	5	115891083
ebi-a-GCST90001734	rs72998964	CD19 on IgD- CD3	6	150723408
ebi-a-GCST90001734	rs112139040	CD19 on IgD- CD3	7	46094636
ebi-a-GCST90001734	rs139775632	CD19 on IgD- CD3	7	6917201
ebi-a-GCST90001734	rs79290247	CD19 on IgD- CD3	7	42996587
ebi-a-GCST90001734	rs73718149	CD19 on IgD- CD3	8	144813778
ebi-a-GCST90001734	rs147763919	CD19 on IgD- CD3	8	62358083
ebi-a-GCST90001734	rs117647889	CD19 on IgD- CD3	9	1239174
ebi-a-GCST90001734	rs117553718	CD19 on IgD- CD3	10	5865812
ebi-a-GCST90001734	rs1871013	CD19 on IgD- CD3	15	27842066
ebi-a-GCST90001734	rs4968431	CD19 on IgD- CD3	17	59421778
ebi-a-GCST90001734	rs75117490	CD19 on IgD- CD3	18	58322970
ebi-a-GCST90001734	rs215237	CD19 on IgD- CD3	19	56404703
ebi-a-GCST90001735	rs112417310	CD19 on IgD- CD3	1	206855483
ebi-a-GCST90001735	rs145924813	CD19 on IgD- CD3	1	28617041
ebi-a-GCST90001735	rs141619127	CD19 on IgD- CD3	1	204227546
ebi-a-GCST90001735	rs113812519	CD19 on IgD- CD3	1	207276336
ebi-a-GCST90001735	rs80268044	CD19 on IgD- CD3	2	43810948
ebi-a-GCST90001735	rs74705306	CD19 on IgD- CD3	4	189782918
ebi-a-GCST90001735	rs7665261	CD19 on IgD- CD3	4	26464043
ebi-a-GCST90001735	rs281142	CD19 on IgD- CD3	5	70969845
ebi-a-GCST90001735	rs56044553	CD19 on IgD- CD3	5	173972294
ebi-a-GCST90001735	rs73275737	CD19 on IgD- CD3	5	148626837
ebi-a-GCST90001735	rs764603	CD19 on IgD- CD3	6	36793516

ebi-a-GCST90001735	rs2281460	CD19 on IgD- CD3	6	42130300
ebi-a-GCST90001735	rs9465443	CD19 on IgD- CD3	6	19563666
ebi-a-GCST90001735	rs114369132	CD19 on IgD- CD3	6	32409141
ebi-a-GCST90001735	rs180723221	CD19 on IgD- CD3	7	103091403
ebi-a-GCST90001735	rs62463053	CD19 on IgD- CD3	7	51994027
ebi-a-GCST90001735	rs2512433	CD19 on IgD- CD3	8	98632764
ebi-a-GCST90001735	rs560933055	CD19 on IgD- CD3	10	1717689
ebi-a-GCST90001735	rs17706571	CD19 on IgD- CD3	12	29271392
ebi-a-GCST90001735	rs2971439	CD19 on IgD- CD3	12	118282893
ebi-a-GCST90001735	rs6575228	CD19 on IgD- CD3	14	92561395
ebi-a-GCST90001735	rs11643297	CD19 on IgD- CD3	16	28973268
ebi-a-GCST90001735	rs138920008	CD19 on IgD- CD3	16	32422808
ebi-a-GCST90001735	rs6500310	CD19 on IgD- CD3	16	50422996
ebi-a-GCST90001735	rs7197237	CD19 on IgD- CD3	16	49673381
ebi-a-GCST90001735	rs6509969	CD19 on IgD- CD3	19	56415502
ebi-a-GCST90001735	rs137949497	CD19 on IgD- CD3	21	32136760
ebi-a-GCST90001735	rs113612962	CD19 on IgD- CD3	22	43167446
ebi-a-GCST90001735	rs5762000	CD19 on IgD- CD3	22	27530934
ebi-a-GCST90001736	rs113812519	CD19 on memory E	1	207276336
ebi-a-GCST90001736	rs4649079	CD19 on memory E	1	25535823
ebi-a-GCST90001736	rs10917581	CD19 on memory E	1	162624504
ebi-a-GCST90001736	rs112417310	CD19 on memory E	1	206855483
ebi-a-GCST90001736	rs79856590	CD19 on memory E	1	205241642
ebi-a-GCST90001736	rs80268044	CD19 on memory E	2	43810948
ebi-a-GCST90001736	rs1439005	CD19 on memory E	3	61094800
ebi-a-GCST90001736	rs7665261	CD19 on memory E	4	26464043
ebi-a-GCST90001736	rs7718237	CD19 on memory E	5	173972852
ebi-a-GCST90001736	rs576166	CD19 on memory E	6	33567104
ebi-a-GCST90001736	rs184318978	CD19 on memory E	6	37497511
ebi-a-GCST90001736	rs9350187	CD19 on memory E	6	19794487
ebi-a-GCST90001736	rs767666	CD19 on memory E	7	43485898
ebi-a-GCST90001736	rs73393708	CD19 on memory E	7	90292336
ebi-a-GCST90001736	rs78007482	CD19 on memory E	8	133189482
ebi-a-GCST90001736	rs75904904	CD19 on memory E	9	36949205
ebi-a-GCST90001736	rs560933055	CD19 on memory E	10	1717689
ebi-a-GCST90001736	rs7900621	CD19 on memory E	10	125702232
ebi-a-GCST90001736	rs17706571	CD19 on memory E	12	29271392
ebi-a-GCST90001736	rs766977	CD19 on memory E	12	50290056
ebi-a-GCST90001736	rs10162314	CD19 on memory E	14	70990398
ebi-a-GCST90001736	rs11643297	CD19 on memory E	16	28973268
ebi-a-GCST90001736	rs138920008	CD19 on memory E	16	32422808
ebi-a-GCST90001736	rs72988602	CD19 on memory E	19	7114038
ebi-a-GCST90001736	rs2903728	CD19 on memory E	19	56408295
ebi-a-GCST90001736	rs138273613	CD19 on memory E	22	39369842
ebi-a-GCST90001736	rs5762000	CD19 on memory E	22	27530934
ebi-a-GCST90001736	rs113612962	CD19 on memory E	22	43167446
ebi-a-GCST90001737	rs79856590	CD19 on naive-mat	1	205241642
ebi-a-GCST90001737	rs142109788	CD19 on naive-mat	1	149815561

ebi-a-GCST90001737	rs75320486	CD19 on naive-mat	1	206842241
ebi-a-GCST90001737	rs113812519	CD19 on naive-mat	1	207276336
ebi-a-GCST90001737	rs34165814	CD19 on naive-mat	2	89486664
ebi-a-GCST90001737	rs1449311	CD19 on naive-mat	3	100703124
ebi-a-GCST90001737	rs11733521	CD19 on naive-mat	4	68968349
ebi-a-GCST90001737	rs78895027	CD19 on naive-mat	6	112306917
ebi-a-GCST90001737	rs117065975	CD19 on naive-mat	6	142183647
ebi-a-GCST90001737	rs9469775	CD19 on naive-mat	6	34335091
ebi-a-GCST90001737	rs76162621	CD19 on naive-mat	6	68486690
ebi-a-GCST90001737	rs12216684	CD19 on naive-mat	7	56291533
ebi-a-GCST90001737	rs62524107	CD19 on naive-mat	8	141805137
ebi-a-GCST90001737	rs147319341	CD19 on naive-mat	8	11301733
ebi-a-GCST90001737	rs75912463	CD19 on naive-mat	9	133354402
ebi-a-GCST90001737	rs34798965	CD19 on naive-mat	10	71647692
ebi-a-GCST90001737	rs11825488	CD19 on naive-mat	11	72165045
ebi-a-GCST90001737	rs67316035	CD19 on naive-mat	11	132221605
ebi-a-GCST90001737	rs79699942	CD19 on naive-mat	12	44929593
ebi-a-GCST90001737	rs74455073	CD19 on naive-mat	12	54902972
ebi-a-GCST90001737	rs10875904	CD19 on naive-mat	12	49357889
ebi-a-GCST90001737	rs12100549	CD19 on naive-mat	14	103417030
ebi-a-GCST90001737	rs11855577	CD19 on naive-mat	15	65507482
ebi-a-GCST90001737	rs117634515	CD19 on naive-mat	15	88610884
ebi-a-GCST90001737	rs138920008	CD19 on naive-mat	16	32422808
ebi-a-GCST90001737	rs754067	CD19 on naive-mat	16	17323672
ebi-a-GCST90001737	rs148381451	CD19 on naive-mat	17	63085557
ebi-a-GCST90001737	rs2903728	CD19 on naive-mat	19	56408295
ebi-a-GCST90001737	rs593715	CD19 on naive-mat	19	50734394
ebi-a-GCST90001738	rs113812519	CD19 on unsw men	1	207276336
ebi-a-GCST90001738	rs4649079	CD19 on unsw men	1	25535823
ebi-a-GCST90001738	rs144743804	CD19 on unsw men	1	207018206
ebi-a-GCST90001738	rs55712453	CD19 on unsw men	1	206902437
ebi-a-GCST90001738	rs80268044	CD19 on unsw men	2	43810948
ebi-a-GCST90001738	rs140108418	CD19 on unsw men	2	13573112
ebi-a-GCST90001738	rs9814135	CD19 on unsw men	3	194094110
ebi-a-GCST90001738	rs1439005	CD19 on unsw men	3	61094800
ebi-a-GCST90001738	rs4862454	CD19 on unsw men	4	185816062
ebi-a-GCST90001738	rs7718237	CD19 on unsw men	5	173972852
ebi-a-GCST90001738	rs76162621	CD19 on unsw men	6	68486690
ebi-a-GCST90001738	rs764603	CD19 on unsw men	6	36793516
ebi-a-GCST90001738	rs41272381	CD19 on unsw men	8	133192184
ebi-a-GCST90001738	rs12554596	CD19 on unsw men	9	37002142
ebi-a-GCST90001738	rs560933055	CD19 on unsw men	10	1717689
ebi-a-GCST90001738	rs76223390	CD19 on unsw men	11	133434534
ebi-a-GCST90001738	rs149168550	CD19 on unsw men	12	111354000
ebi-a-GCST90001738	rs4942349	CD19 on unsw men	13	31564105
ebi-a-GCST90001738	rs6575228	CD19 on unsw men	14	92561395
ebi-a-GCST90001738	rs138920008	CD19 on unsw men	16	32422808
ebi-a-GCST90001738	rs11643297	CD19 on unsw men	16	28973268

ebi-a-GCST90001738	rs148381451	CD19 on unsw men	17	63085557
ebi-a-GCST90001738	rs149041258	CD19 on unsw men	18	74408048
ebi-a-GCST90001738	rs72988602	CD19 on unsw men	19	7114038
ebi-a-GCST90001738	rs77697711	CD19 on unsw men	22	45116977
ebi-a-GCST90001738	rs113612962	CD19 on unsw men	22	43167446
ebi-a-GCST90001739	rs11585051	CD19 on PB/PC	1	235762345
ebi-a-GCST90001739	rs78175608	CD19 on PB/PC	2	233868788
ebi-a-GCST90001739	rs181547338	CD19 on PB/PC	2	240579362
ebi-a-GCST90001739	rs6767669	CD19 on PB/PC	3	116599500
ebi-a-GCST90001739	rs76670160	CD19 on PB/PC	3	54686737
ebi-a-GCST90001739	rs79146843	CD19 on PB/PC	3	165050068
ebi-a-GCST90001739	rs115017263	CD19 on PB/PC	4	55691063
ebi-a-GCST90001739	rs6848130	CD19 on PB/PC	4	81157098
ebi-a-GCST90001739	rs138520806	CD19 on PB/PC	5	3781931
ebi-a-GCST90001739	rs11748905	CD19 on PB/PC	5	80934634
ebi-a-GCST90001739	rs72759977	CD19 on PB/PC	5	53665443
ebi-a-GCST90001739	rs117517136	CD19 on PB/PC	8	88548115
ebi-a-GCST90001739	rs56799059	CD19 on PB/PC	10	130289313
ebi-a-GCST90001739	rs10858826	CD19 on PB/PC	12	89505448
ebi-a-GCST90001739	rs139369230	CD19 on PB/PC	14	106204468
ebi-a-GCST90001739	rs7171754	CD19 on PB/PC	15	96281487
ebi-a-GCST90001739	rs117491079	CD19 on PB/PC	16	85272300
ebi-a-GCST90001739	rs9913983	CD19 on PB/PC	17	56001319
ebi-a-GCST90001739	rs740380	CD19 on PB/PC	17	14221680
ebi-a-GCST90001739	rs72839309	CD19 on PB/PC	17	66341775
ebi-a-GCST90001739	rs2612375	CD19 on PB/PC	18	73626676
ebi-a-GCST90001739	rs9961803	CD19 on PB/PC	18	61174560
ebi-a-GCST90001739	rs78258643	CD19 on PB/PC	18	40527078
ebi-a-GCST90001739	rs11701275	CD19 on PB/PC	21	16962765
ebi-a-GCST90001740	rs112417310	CD19 on sw mem	1	206855483
ebi-a-GCST90001740	rs141619127	CD19 on sw mem	1	204227546
ebi-a-GCST90001740	rs145924813	CD19 on sw mem	1	28617041
ebi-a-GCST90001740	rs113812519	CD19 on sw mem	1	207276336
ebi-a-GCST90001740	rs80268044	CD19 on sw mem	2	43810948
ebi-a-GCST90001740	rs1439005	CD19 on sw mem	3	61094800
ebi-a-GCST90001740	rs7665261	CD19 on sw mem	4	26464043
ebi-a-GCST90001740	rs281142	CD19 on sw mem	5	70969845
ebi-a-GCST90001740	rs56044553	CD19 on sw mem	5	173972294
ebi-a-GCST90001740	rs73275737	CD19 on sw mem	5	148626837
ebi-a-GCST90001740	rs116364947	CD19 on sw mem	6	36809211
ebi-a-GCST90001740	rs2281460	CD19 on sw mem	6	42130300
ebi-a-GCST90001740	rs9460382	CD19 on sw mem	6	19564461
ebi-a-GCST90001740	rs112126052	CD19 on sw mem	6	32173757
ebi-a-GCST90001740	rs2357754	CD19 on sw mem	6	120164685
ebi-a-GCST90001740	rs9368123	CD19 on sw mem	6	19798682
ebi-a-GCST90001740	rs17242358	CD19 on sw mem	8	129964873
ebi-a-GCST90001740	rs12554848	CD19 on sw mem	9	36981822
ebi-a-GCST90001740	rs560933055	CD19 on sw mem	10	1717689

ebi-a-GCST90001740	rs17706571	CD19 on sw mem	12	29271392
ebi-a-GCST90001740	rs4343243	CD19 on sw mem	15	77004600
ebi-a-GCST90001740	rs62059362	CD19 on sw mem	16	35271879
ebi-a-GCST90001740	rs77162145	CD19 on sw mem	16	48885048
ebi-a-GCST90001740	rs7189927	CD19 on sw mem	16	28913787
ebi-a-GCST90001740	rs13382005	CD19 on sw mem	18	58304032
ebi-a-GCST90001740	rs62124467	CD19 on sw mem	19	7079452
ebi-a-GCST90001740	rs78614237	CD19 on sw mem	20	47459855
ebi-a-GCST90001740	rs137949497	CD19 on sw mem	21	32136760
ebi-a-GCST90001740	rs5762000	CD19 on sw mem	22	27530934
ebi-a-GCST90001740	rs113612962	CD19 on sw mem	22	43167446
ebi-a-GCST90001741	rs112417310	CD19 on IgD+	1	206855483
ebi-a-GCST90001741	rs142109788	CD19 on IgD+	1	149815561
ebi-a-GCST90001741	rs79856590	CD19 on IgD+	1	205241642
ebi-a-GCST90001741	rs11120838	CD19 on IgD+	1	7149639
ebi-a-GCST90001741	rs113812519	CD19 on IgD+	1	207276336
ebi-a-GCST90001741	rs1862061	CD19 on IgD+	2	16800618
ebi-a-GCST90001741	rs1449311	CD19 on IgD+	3	100703124
ebi-a-GCST90001741	rs10518007	CD19 on IgD+	4	67702525
ebi-a-GCST90001741	rs79550250	CD19 on IgD+	4	13123693
ebi-a-GCST90001741	rs115727814	CD19 on IgD+	4	170699036
ebi-a-GCST90001741	rs76162621	CD19 on IgD+	6	68486690
ebi-a-GCST90001741	rs116364947	CD19 on IgD+	6	36809211
ebi-a-GCST90001741	rs17433679	CD19 on IgD+	7	78097255
ebi-a-GCST90001741	rs12216684	CD19 on IgD+	7	56291533
ebi-a-GCST90001741	rs80273434	CD19 on IgD+	8	141772905
ebi-a-GCST90001741	rs557939103	CD19 on IgD+	8	9079951
ebi-a-GCST90001741	rs75912463	CD19 on IgD+	9	133354402
ebi-a-GCST90001741	rs34798965	CD19 on IgD+	10	71647692
ebi-a-GCST90001741	rs10894538	CD19 on IgD+	11	132222058
ebi-a-GCST90001741	rs117004076	CD19 on IgD+	14	97702226
ebi-a-GCST90001741	rs138920008	CD19 on IgD+	16	32422808
ebi-a-GCST90001741	rs78044532	CD19 on IgD+	16	21168467
ebi-a-GCST90001741	rs754067	CD19 on IgD+	16	17323672
ebi-a-GCST90001741	rs1290397	CD19 on IgD+	17	77948063
ebi-a-GCST90001741	rs148381451	CD19 on IgD+	17	63085557
ebi-a-GCST90001741	rs8109226	CD19 on IgD+	19	710050
ebi-a-GCST90001741	rs2903728	CD19 on IgD+	19	56408295
ebi-a-GCST90001741	rs34589185	CD19 on IgD+	19	50761130
ebi-a-GCST90001741	rs144392728	CD19 on IgD+	22	33029820
ebi-a-GCST90001742	rs55712453	CD19 on transitiona	1	206902437
ebi-a-GCST90001742	rs2026774	CD19 on transitiona	1	86076069
ebi-a-GCST90001742	rs144743804	CD19 on transitiona	1	207018206
ebi-a-GCST90001742	rs113812519	CD19 on transitiona	1	207276336
ebi-a-GCST90001742	rs13030427	CD19 on transitiona	2	208879261
ebi-a-GCST90001742	rs116180572	CD19 on transitiona	3	61084498
ebi-a-GCST90001742	rs2290055	CD19 on transitiona	3	108549720
ebi-a-GCST90001742	rs77953960	CD19 on transitiona	5	9427816

ebi-a-GCST90001742	rs13164259	CD19 on transitiona	5	82870211
ebi-a-GCST90001742	rs9459447	CD19 on transitiona	6	165954369
ebi-a-GCST90001742	rs12216684	CD19 on transitiona	7	56291533
ebi-a-GCST90001742	rs35904103	CD19 on transitiona	7	43734219
ebi-a-GCST90001742	rs62519592	CD19 on transitiona	8	133193809
ebi-a-GCST90001742	rs11825488	CD19 on transitiona	11	72165045
ebi-a-GCST90001742	rs117442206	CD19 on transitiona	12	25430787
ebi-a-GCST90001742	rs115570551	CD19 on transitiona	14	53874261
ebi-a-GCST90001742	rs935131	CD19 on transitiona	15	65625991
ebi-a-GCST90001742	rs138920008	CD19 on transitiona	16	32422808
ebi-a-GCST90001742	rs151295797	CD19 on transitiona	16	84071182
ebi-a-GCST90001742	rs28626305	CD19 on transitiona	16	24921264
ebi-a-GCST90001742	rs933570	CD19 on transitiona	16	11760186
ebi-a-GCST90001742	rs148381451	CD19 on transitiona	17	63085557
ebi-a-GCST90001742	rs9949821	CD19 on transitiona	18	54242149
ebi-a-GCST90001742	rs1667906	CD19 on transitiona	18	74839524
ebi-a-GCST90001742	rs73146562	CD19 on transitiona	20	52477220
ebi-a-GCST90001742	rs117562840	CD19 on transitiona	22	42680877
ebi-a-GCST90001743	rs113371046	CD20 on B cell	1	17595034
ebi-a-GCST90001743	rs79003098	CD20 on B cell	2	81965038
ebi-a-GCST90001743	rs187188344	CD20 on B cell	2	178858731
ebi-a-GCST90001743	rs78296478	CD20 on B cell	2	125069044
ebi-a-GCST90001743	rs75097128	CD20 on B cell	4	113292746
ebi-a-GCST90001743	rs13109614	CD20 on B cell	4	62019413
ebi-a-GCST90001743	rs28620974	CD20 on B cell	4	137463130
ebi-a-GCST90001743	rs73245560	CD20 on B cell	4	43987474
ebi-a-GCST90001743	rs80155794	CD20 on B cell	5	178957291
ebi-a-GCST90001743	rs3933208	CD20 on B cell	5	161458204
ebi-a-GCST90001743	rs77612075	CD20 on B cell	5	162820460
ebi-a-GCST90001743	rs72836542	CD20 on B cell	6	14091682
ebi-a-GCST90001743	rs9277628	CD20 on B cell	6	33082040
ebi-a-GCST90001743	rs9375555	CD20 on B cell	6	128547994
ebi-a-GCST90001743	rs233495	CD20 on B cell	6	14806317
ebi-a-GCST90001743	rs6901175	CD20 on B cell	6	32198527
ebi-a-GCST90001743	rs35139284	CD20 on B cell	6	32561370
ebi-a-GCST90001743	rs12212931	CD20 on B cell	6	15913304
ebi-a-GCST90001743	rs2975021	CD20 on B cell	6	29878559
ebi-a-GCST90001743	rs388354	CD20 on B cell	7	40190449
ebi-a-GCST90001743	rs75071948	CD20 on B cell	9	75768836
ebi-a-GCST90001743	rs1941018	CD20 on B cell	11	60268994
ebi-a-GCST90001743	rs143129208	CD20 on B cell	11	99391820
ebi-a-GCST90001743	rs11051833	CD20 on B cell	12	32330242
ebi-a-GCST90001743	rs4768810	CD20 on B cell	12	47866824
ebi-a-GCST90001743	rs6496328	CD20 on B cell	15	86988686
ebi-a-GCST90001743	rs8025257	CD20 on B cell	15	89600226
ebi-a-GCST90001743	rs354026	CD20 on B cell	19	49841054
ebi-a-GCST90001744	rs2861007	CD20 on CD20- CI	2	66306389
ebi-a-GCST90001744	rs73015549	CD20 on CD20- CI	3	7495328

ebi-a-GCST90001744	rs572468	CD20 on CD20- CI	4	13993465
ebi-a-GCST90001744	rs116559476	CD20 on CD20- CI	4	180025316
ebi-a-GCST90001744	rs4921509	CD20 on CD20- CI	5	159000854
ebi-a-GCST90001744	rs79248632	CD20 on CD20- CI	5	158319820
ebi-a-GCST90001744	rs1558186	CD20 on CD20- CI	5	133041544
ebi-a-GCST90001744	rs34280838	CD20 on CD20- CI	6	32139565
ebi-a-GCST90001744	rs6930288	CD20 on CD20- CI	6	29098881
ebi-a-GCST90001744	rs2188517	CD20 on CD20- CI	7	90108278
ebi-a-GCST90001744	rs41329345	CD20 on CD20- CI	7	48456925
ebi-a-GCST90001744	rs2243879	CD20 on CD20- CI	10	35622558
ebi-a-GCST90001744	rs35857670	CD20 on CD20- CI	10	82607555
ebi-a-GCST90001744	rs1318389	CD20 on CD20- CI	13	35149649
ebi-a-GCST90001744	rs145619091	CD20 on CD20- CI	17	4261253
ebi-a-GCST90001744	rs12952217	CD20 on CD20- CI	17	1018426
ebi-a-GCST90001744	rs12977515	CD20 on CD20- CI	19	16391727
ebi-a-GCST90001744	rs10426452	CD20 on CD20- CI	19	49907690
ebi-a-GCST90001744	rs5997212	CD20 on CD20- CI	22	27679924
ebi-a-GCST90001745	rs187188344	CD20 on CD24+ C	2	178858731
ebi-a-GCST90001745	rs13080324	CD20 on CD24+ C	3	111952407
ebi-a-GCST90001745	rs149204679	CD20 on CD24+ C	3	27240644
ebi-a-GCST90001745	rs62340965	CD20 on CD24+ C	4	190508875
ebi-a-GCST90001745	rs2114442	CD20 on CD24+ C	4	100832060
ebi-a-GCST90001745	rs77612075	CD20 on CD24+ C	5	162820460
ebi-a-GCST90001745	rs114915558	CD20 on CD24+ C	5	98564385
ebi-a-GCST90001745	rs295654	CD20 on CD24+ C	5	58107171
ebi-a-GCST90001745	rs6459417	CD20 on CD24+ C	6	15858996
ebi-a-GCST90001745	rs9400057	CD20 on CD24+ C	6	107162216
ebi-a-GCST90001745	rs111259171	CD20 on CD24+ C	6	32640351
ebi-a-GCST90001745	rs12196019	CD20 on CD24+ C	6	31005483
ebi-a-GCST90001745	rs9461557	CD20 on CD24+ C	6	29765925
ebi-a-GCST90001745	rs6922309	CD20 on CD24+ C	6	31321238
ebi-a-GCST90001745	rs12554596	CD20 on CD24+ C	9	37002142
ebi-a-GCST90001745	rs7035906	CD20 on CD24+ C	9	95898832
ebi-a-GCST90001745	rs75071948	CD20 on CD24+ C	9	75768836
ebi-a-GCST90001745	rs78850316	CD20 on CD24+ C	10	99560231
ebi-a-GCST90001745	rs11187166	CD20 on CD24+ C	10	94516008
ebi-a-GCST90001745	rs74621542	CD20 on CD24+ C	11	60166613
ebi-a-GCST90001745	rs7128035	CD20 on CD24+ C	11	268078
ebi-a-GCST90001745	rs149695230	CD20 on CD24+ C	11	60288804
ebi-a-GCST90001745	rs111896387	CD20 on CD24+ C	12	45116844
ebi-a-GCST90001745	rs2578469	CD20 on CD24+ C	12	4101401
ebi-a-GCST90001745	rs61958965	CD20 on CD24+ C	13	72232674
ebi-a-GCST90001745	rs149214933	CD20 on CD24+ C	13	64174465
ebi-a-GCST90001745	rs62000215	CD20 on CD24+ C	15	31184858
ebi-a-GCST90001745	rs141211110	CD20 on CD24+ C	16	61118647
ebi-a-GCST90001745	rs179212	CD20 on CD24+ C	16	19308868
ebi-a-GCST90001745	rs141121779	CD20 on CD24+ C	19	49998328
ebi-a-GCST90001745	rs61731791	CD20 on CD24+ C	19	50940778

ebi-a-GCST90001745	rs55809481	CD20 on CD24+ C	19	49872521
ebi-a-GCST90001746	rs116729050	CD20 on IgD+ CD2	1	62560039
ebi-a-GCST90001746	rs149204679	CD20 on IgD+ CD2	3	27240644
ebi-a-GCST90001746	rs35525697	CD20 on IgD+ CD2	3	149086665
ebi-a-GCST90001746	rs62340965	CD20 on IgD+ CD2	4	190508875
ebi-a-GCST90001746	rs4700466	CD20 on IgD+ CD2	5	61352528
ebi-a-GCST90001746	rs114915558	CD20 on IgD+ CD2	5	98564385
ebi-a-GCST90001746	rs77121649	CD20 on IgD+ CD2	6	31812140
ebi-a-GCST90001746	rs3129750	CD20 on IgD+ CD2	6	32581515
ebi-a-GCST90001746	rs9461562	CD20 on IgD+ CD2	6	29778271
ebi-a-GCST90001746	rs1569590	CD20 on IgD+ CD2	6	15898884
ebi-a-GCST90001746	rs28572463	CD20 on IgD+ CD2	6	31209854
ebi-a-GCST90001746	rs13207893	CD20 on IgD+ CD2	6	32586922
ebi-a-GCST90001746	rs79110866	CD20 on IgD+ CD2	9	75709022
ebi-a-GCST90001746	rs76239564	CD20 on IgD+ CD2	10	6361438
ebi-a-GCST90001746	rs4746507	CD20 on IgD+ CD2	10	67086564
ebi-a-GCST90001746	rs1941033	CD20 on IgD+ CD2	11	60249526
ebi-a-GCST90001746	rs7128035	CD20 on IgD+ CD2	11	268078
ebi-a-GCST90001746	rs149214933	CD20 on IgD+ CD2	13	64174465
ebi-a-GCST90001746	rs2532969	CD20 on IgD+ CD2	13	50896167
ebi-a-GCST90001746	rs62000215	CD20 on IgD+ CD2	15	31184858
ebi-a-GCST90001746	rs66637059	CD20 on IgD+ CD2	17	17417808
ebi-a-GCST90001746	rs11662753	CD20 on IgD+ CD2	18	60657744
ebi-a-GCST90001746	rs61731791	CD20 on IgD+ CD2	19	50940778
ebi-a-GCST90001746	rs55809481	CD20 on IgD+ CD2	19	49872521
ebi-a-GCST90001746	rs62623429	CD20 on IgD+ CD2	19	51714781
ebi-a-GCST90001747	rs113371046	CD20 on IgD+ CD2	1	17595034
ebi-a-GCST90001747	rs1293260	CD20 on IgD+ CD2	1	25709104
ebi-a-GCST90001747	rs111741355	CD20 on IgD+ CD2	1	48095312
ebi-a-GCST90001747	rs149360160	CD20 on IgD+ CD2	2	229499798
ebi-a-GCST90001747	rs842631	CD20 on IgD+ CD2	2	61085723
ebi-a-GCST90001747	rs6443362	CD20 on IgD+ CD2	3	176369881
ebi-a-GCST90001747	rs13070807	CD20 on IgD+ CD2	3	56847945
ebi-a-GCST90001747	rs73245560	CD20 on IgD+ CD2	4	43987474
ebi-a-GCST90001747	rs80155794	CD20 on IgD+ CD2	5	178957291
ebi-a-GCST90001747	rs3933208	CD20 on IgD+ CD2	5	161458204
ebi-a-GCST90001747	rs72836542	CD20 on IgD+ CD2	6	14091682
ebi-a-GCST90001747	rs55643835	CD20 on IgD+ CD2	6	33947909
ebi-a-GCST90001747	rs62405562	CD20 on IgD+ CD2	6	32451834
ebi-a-GCST90001747	rs2066399	CD20 on IgD+ CD2	6	15880540
ebi-a-GCST90001747	rs233495	CD20 on IgD+ CD2	6	14806317
ebi-a-GCST90001747	rs9270560	CD20 on IgD+ CD2	6	32560741
ebi-a-GCST90001747	rs388354	CD20 on IgD+ CD2	7	40190449
ebi-a-GCST90001747	rs12771377	CD20 on IgD+ CD2	10	22717779
ebi-a-GCST90001747	rs4939384	CD20 on IgD+ CD2	11	60273642
ebi-a-GCST90001747	rs11059642	CD20 on IgD+ CD2	12	128795644
ebi-a-GCST90001747	rs11051833	CD20 on IgD+ CD2	12	32330242
ebi-a-GCST90001747	rs56132967	CD20 on IgD+ CD2	14	61644977

ebi-a-GCST90001747	rs6496328	CD20 on IgD+ CD2	15	86988686
ebi-a-GCST90001747	rs8025257	CD20 on IgD+ CD2	15	89600226
ebi-a-GCST90001747	rs7404890	CD20 on IgD+ CD2	16	11007193
ebi-a-GCST90001747	rs11083972	CD20 on IgD+ CD2	19	49896752
ebi-a-GCST90001747	rs73086355	CD20 on IgD+ CD2	20	11850850
ebi-a-GCST90001747	rs1007337	CD20 on IgD+ CD2	22	39781585
ebi-a-GCST90001748	rs116412485	CD20 on IgD+ CD3	1	40040949
ebi-a-GCST90001748	rs17380682	CD20 on IgD+ CD3	1	164447415
ebi-a-GCST90001748	rs142291033	CD20 on IgD+ CD3	1	56196958
ebi-a-GCST90001748	rs187188344	CD20 on IgD+ CD3	2	178858731
ebi-a-GCST90001748	rs2114442	CD20 on IgD+ CD3	4	100832060
ebi-a-GCST90001748	rs75097128	CD20 on IgD+ CD3	4	113292746
ebi-a-GCST90001748	rs11135082	CD20 on IgD+ CD3	5	159137396
ebi-a-GCST90001748	rs76029468	CD20 on IgD+ CD3	5	161525053
ebi-a-GCST90001748	rs61651267	CD20 on IgD+ CD3	5	143495021
ebi-a-GCST90001748	rs13190846	CD20 on IgD+ CD3	6	29905727
ebi-a-GCST90001748	rs2066399	CD20 on IgD+ CD3	6	15880540
ebi-a-GCST90001748	rs7768875	CD20 on IgD+ CD3	6	32560752
ebi-a-GCST90001748	rs78517600	CD20 on IgD+ CD3	6	50239066
ebi-a-GCST90001748	rs12660382	CD20 on IgD+ CD3	6	31443323
ebi-a-GCST90001748	rs150618362	CD20 on IgD+ CD3	8	13572692
ebi-a-GCST90001748	rs149514748	CD20 on IgD+ CD3	9	118323116
ebi-a-GCST90001748	rs12554596	CD20 on IgD+ CD3	9	37002142
ebi-a-GCST90001748	rs143959146	CD20 on IgD+ CD3	9	33898132
ebi-a-GCST90001748	rs7036970	CD20 on IgD+ CD3	9	3182850
ebi-a-GCST90001748	rs4474350	CD20 on IgD+ CD3	10	26837236
ebi-a-GCST90001748	rs182220218	CD20 on IgD+ CD3	10	75422693
ebi-a-GCST90001748	rs2070770	CD20 on IgD+ CD3	11	60230531
ebi-a-GCST90001748	rs74054746	CD20 on IgD+ CD3	14	61638400
ebi-a-GCST90001748	rs2356414	CD20 on IgD+ CD3	16	71378301
ebi-a-GCST90001748	rs7216991	CD20 on IgD+ CD3	17	45052253
ebi-a-GCST90001748	rs7259731	CD20 on IgD+ CD3	19	54841732
ebi-a-GCST90001748	rs30003	CD20 on IgD+ CD3	19	49846398
ebi-a-GCST90001749	rs138448626	CD20 on IgD+ CD3	1	24084235
ebi-a-GCST90001749	rs112695918	CD20 on IgD+ CD3	1	1104584
ebi-a-GCST90001749	rs1495924	CD20 on IgD+ CD3	2	241183284
ebi-a-GCST90001749	rs12470912	CD20 on IgD+ CD3	2	168748110
ebi-a-GCST90001749	rs11920066	CD20 on IgD+ CD3	3	88803979
ebi-a-GCST90001749	rs9808998	CD20 on IgD+ CD3	3	176405898
ebi-a-GCST90001749	rs13061533	CD20 on IgD+ CD3	3	188611158
ebi-a-GCST90001749	rs73810453	CD20 on IgD+ CD3	4	41994707
ebi-a-GCST90001749	rs189077909	CD20 on IgD+ CD3	4	57626079
ebi-a-GCST90001749	rs9269561	CD20 on IgD+ CD3	6	32544405
ebi-a-GCST90001749	rs17613271	CD20 on IgD+ CD3	6	32621778
ebi-a-GCST90001749	rs2066399	CD20 on IgD+ CD3	6	15880540
ebi-a-GCST90001749	rs143959146	CD20 on IgD+ CD3	9	33898132
ebi-a-GCST90001749	rs11199370	CD20 on IgD+ CD3	10	122262398
ebi-a-GCST90001749	rs11604619	CD20 on IgD+ CD3	11	60261376

ebi-a-GCST90001749	rs141950809	CD20 on IgD+ CD3	13	93405148
ebi-a-GCST90001749	rs111741301	CD20 on IgD+ CD3	13	71781323
ebi-a-GCST90001749	rs4270172	CD20 on IgD+ CD3	16	55157679
ebi-a-GCST90001749	rs115329330	CD20 on IgD+ CD3	21	27574452
ebi-a-GCST90001749	rs3959642	CD20 on IgD+ CD3	22	39860589
ebi-a-GCST90001750	rs2481929	CD20 on IgD+ CD3	1	24807579
ebi-a-GCST90001750	rs7598607	CD20 on IgD+ CD3	2	129870972
ebi-a-GCST90001750	rs2220549	CD20 on IgD+ CD3	3	21756408
ebi-a-GCST90001750	rs68171708	CD20 on IgD+ CD3	3	142855504
ebi-a-GCST90001750	rs76078276	CD20 on IgD+ CD3	3	187802062
ebi-a-GCST90001750	rs140761841	CD20 on IgD+ CD3	4	747378
ebi-a-GCST90001750	rs1545973	CD20 on IgD+ CD3	4	141622371
ebi-a-GCST90001750	rs62366714	CD20 on IgD+ CD3	5	105199555
ebi-a-GCST90001750	rs11742841	CD20 on IgD+ CD3	5	108992703
ebi-a-GCST90001750	rs760958	CD20 on IgD+ CD3	6	15899354
ebi-a-GCST90001750	rs9461562	CD20 on IgD+ CD3	6	29778271
ebi-a-GCST90001750	rs9469053	CD20 on IgD+ CD3	6	31755776
ebi-a-GCST90001750	rs11230344	CD20 on IgD+ CD3	11	60264409
ebi-a-GCST90001750	rs1972795	CD20 on IgD+ CD3	12	95289170
ebi-a-GCST90001750	rs79741907	CD20 on IgD+ CD3	14	94877494
ebi-a-GCST90001750	rs12898020	CD20 on IgD+ CD3	14	90835752
ebi-a-GCST90001750	rs7179991	CD20 on IgD+ CD3	15	31811797
ebi-a-GCST90001750	rs76029565	CD20 on IgD+ CD3	19	49887709
ebi-a-GCST90001750	rs73205632	CD20 on IgD+ CD3	21	35291153
ebi-a-GCST90001751	rs616645	CD20 on IgD+ CD3	1	12240824
ebi-a-GCST90001751	rs56031692	CD20 on IgD+ CD3	1	58879185
ebi-a-GCST90001751	rs7568308	CD20 on IgD+ CD3	2	61051498
ebi-a-GCST90001751	rs115361290	CD20 on IgD+ CD3	2	184424836
ebi-a-GCST90001751	rs116393510	CD20 on IgD+ CD3	2	200547937
ebi-a-GCST90001751	rs2136235	CD20 on IgD+ CD3	3	183075828
ebi-a-GCST90001751	rs73175579	CD20 on IgD+ CD3	3	185524179
ebi-a-GCST90001751	rs1313625	CD20 on IgD+ CD3	5	22458923
ebi-a-GCST90001751	rs2066399	CD20 on IgD+ CD3	6	15880540
ebi-a-GCST90001751	rs111983490	CD20 on IgD+ CD3	6	32205324
ebi-a-GCST90001751	rs7768875	CD20 on IgD+ CD3	6	32560752
ebi-a-GCST90001751	rs233493	CD20 on IgD+ CD3	6	14804334
ebi-a-GCST90001751	rs72836542	CD20 on IgD+ CD3	6	14091682
ebi-a-GCST90001751	rs9271700	CD20 on IgD+ CD3	6	32593198
ebi-a-GCST90001751	rs150896136	CD20 on IgD+ CD3	7	68325252
ebi-a-GCST90001751	rs10965813	CD20 on IgD+ CD3	9	23378556
ebi-a-GCST90001751	rs116910507	CD20 on IgD+ CD3	9	137841245
ebi-a-GCST90001751	rs17233534	CD20 on IgD+ CD3	10	107505960
ebi-a-GCST90001751	rs7939177	CD20 on IgD+ CD3	11	60266070
ebi-a-GCST90001751	rs7307681	CD20 on IgD+ CD3	12	55336327
ebi-a-GCST90001751	rs117266160	CD20 on IgD+ CD3	14	81350884
ebi-a-GCST90001751	rs532803692	CD20 on IgD+ CD3	16	20477419
ebi-a-GCST90001751	rs146797770	CD20 on IgD+ CD3	17	6772746
ebi-a-GCST90001751	rs34486765	CD20 on IgD+ CD3	19	49915911

ebi-a-GCST90001751	rs234630	CD20 on IgD+ CD3	20	57483655
ebi-a-GCST90001751	rs139106	CD20 on IgD+ CD3	22	44569390
ebi-a-GCST90001751	rs5750823	CD20 on IgD+ CD3	22	39829973
ebi-a-GCST90001752	rs113371046	CD20 on IgD+ CD3	1	17595034
ebi-a-GCST90001752	rs1293260	CD20 on IgD+ CD3	1	25709104
ebi-a-GCST90001752	rs111741355	CD20 on IgD+ CD3	1	48095312
ebi-a-GCST90001752	rs12042193	CD20 on IgD+ CD3	1	116031657
ebi-a-GCST90001752	rs12120358	CD20 on IgD+ CD3	1	161444369
ebi-a-GCST90001752	rs147586463	CD20 on IgD+ CD3	1	169204921
ebi-a-GCST90001752	rs1306395	CD20 on IgD+ CD3	2	61076272
ebi-a-GCST90001752	rs73154503	CD20 on IgD+ CD3	3	159871677
ebi-a-GCST90001752	rs77612075	CD20 on IgD+ CD3	5	162820460
ebi-a-GCST90001752	rs187747283	CD20 on IgD+ CD3	5	56487565
ebi-a-GCST90001752	rs233493	CD20 on IgD+ CD3	6	14804334
ebi-a-GCST90001752	rs72836542	CD20 on IgD+ CD3	6	14091682
ebi-a-GCST90001752	rs55643835	CD20 on IgD+ CD3	6	33947909
ebi-a-GCST90001752	rs9270560	CD20 on IgD+ CD3	6	32560741
ebi-a-GCST90001752	rs2066399	CD20 on IgD+ CD3	6	15880540
ebi-a-GCST90001752	rs9267484	CD20 on IgD+ CD3	6	31505724
ebi-a-GCST90001752	rs62405562	CD20 on IgD+ CD3	6	32451834
ebi-a-GCST90001752	rs11242780	CD20 on IgD+ CD3	6	2511559
ebi-a-GCST90001752	rs76355582	CD20 on IgD+ CD3	7	119197741
ebi-a-GCST90001752	rs388354	CD20 on IgD+ CD3	7	40190449
ebi-a-GCST90001752	rs4939384	CD20 on IgD+ CD3	11	60273642
ebi-a-GCST90001752	rs11230624	CD20 on IgD+ CD3	11	60952231
ebi-a-GCST90001752	rs12282477	CD20 on IgD+ CD3	11	90976338
ebi-a-GCST90001752	rs8025257	CD20 on IgD+ CD3	15	89600226
ebi-a-GCST90001752	rs17616173	CD20 on IgD+ CD3	15	86986287
ebi-a-GCST90001752	rs7404890	CD20 on IgD+ CD3	16	11007193
ebi-a-GCST90001752	rs17763050	CD20 on IgD+ CD3	17	43903336
ebi-a-GCST90001752	rs34486765	CD20 on IgD+ CD3	19	49915911
ebi-a-GCST90001752	rs61731791	CD20 on IgD+ CD3	19	50940778
ebi-a-GCST90001752	rs73086355	CD20 on IgD+ CD3	20	11850850
ebi-a-GCST90001752	rs1007337	CD20 on IgD+ CD3	22	39781585
ebi-a-GCST90001753	rs77463781	CD20 on IgD- CD2	1	79464773
ebi-a-GCST90001753	rs74435765	CD20 on IgD- CD2	1	185522944
ebi-a-GCST90001753	rs849532	CD20 on IgD- CD2	2	206552726
ebi-a-GCST90001753	rs10171238	CD20 on IgD- CD2	2	149568729
ebi-a-GCST90001753	rs7617301	CD20 on IgD- CD2	3	119289916
ebi-a-GCST90001753	rs62299189	CD20 on IgD- CD2	4	43192979
ebi-a-GCST90001753	rs4426778	CD20 on IgD- CD2	4	102780724
ebi-a-GCST90001753	rs78373541	CD20 on IgD- CD2	4	139616732
ebi-a-GCST90001753	rs73196454	CD20 on IgD- CD2	5	105783715
ebi-a-GCST90001753	rs13181744	CD20 on IgD- CD2	5	120594915
ebi-a-GCST90001753	rs12660382	CD20 on IgD- CD2	6	31443323
ebi-a-GCST90001753	rs6454789	CD20 on IgD- CD2	6	90677176
ebi-a-GCST90001753	rs7754613	CD20 on IgD- CD2	6	15902888
ebi-a-GCST90001753	rs11751198	CD20 on IgD- CD2	6	31753526

ebi-a-GCST90001753	rs35567463	CD20 on IgD- CD2	6	32633970
ebi-a-GCST90001753	rs35485457	CD20 on IgD- CD2	8	78679087
ebi-a-GCST90001753	rs117961939	CD20 on IgD- CD2	8	132476096
ebi-a-GCST90001753	rs1838178	CD20 on IgD- CD2	8	92858976
ebi-a-GCST90001753	rs787045	CD20 on IgD- CD2	10	26831337
ebi-a-GCST90001753	rs61884722	CD20 on IgD- CD2	11	18601510
ebi-a-GCST90001753	rs78287249	CD20 on IgD- CD2	11	60157281
ebi-a-GCST90001753	rs11051833	CD20 on IgD- CD2	12	32330242
ebi-a-GCST90001753	rs1453509	CD20 on IgD- CD2	16	17014673
ebi-a-GCST90001753	rs17136624	CD20 on IgD- CD2	16	3996282
ebi-a-GCST90001753	rs139713744	CD20 on IgD- CD2	17	30608982
ebi-a-GCST90001753	rs542436617	CD20 on IgD- CD2	17	21246554
ebi-a-GCST90001753	rs13381717	CD20 on IgD- CD2	18	352095
ebi-a-GCST90001753	rs30006	CD20 on IgD- CD2	19	49847916
ebi-a-GCST90001753	rs61731791	CD20 on IgD- CD2	19	50940778
ebi-a-GCST90001754	rs116144119	CD20 on IgD- CD2	1	165812513
ebi-a-GCST90001754	rs34713734	CD20 on IgD- CD2	2	122564113
ebi-a-GCST90001754	rs10171238	CD20 on IgD- CD2	2	149568729
ebi-a-GCST90001754	rs7608595	CD20 on IgD- CD2	2	223088415
ebi-a-GCST90001754	rs74812685	CD20 on IgD- CD2	4	2654756
ebi-a-GCST90001754	rs160509	CD20 on IgD- CD2	5	9588308
ebi-a-GCST90001754	rs4716010	CD20 on IgD- CD2	6	15898371
ebi-a-GCST90001754	rs1838178	CD20 on IgD- CD2	8	92858976
ebi-a-GCST90001754	rs10901783	CD20 on IgD- CD2	10	126294501
ebi-a-GCST90001754	rs787045	CD20 on IgD- CD2	10	26831337
ebi-a-GCST90001754	rs2493367	CD20 on IgD- CD2	10	66219102
ebi-a-GCST90001754	rs61884722	CD20 on IgD- CD2	11	18601510
ebi-a-GCST90001754	rs78368496	CD20 on IgD- CD2	11	19984329
ebi-a-GCST90001754	rs11051833	CD20 on IgD- CD2	12	32330242
ebi-a-GCST90001754	rs9962656	CD20 on IgD- CD2	18	60847262
ebi-a-GCST90001754	rs13381717	CD20 on IgD- CD2	18	352095
ebi-a-GCST90001754	rs61731791	CD20 on IgD- CD2	19	50940778
ebi-a-GCST90001754	rs30006	CD20 on IgD- CD2	19	49847916
ebi-a-GCST90001754	rs1883832	CD20 on IgD- CD2	20	44746982
ebi-a-GCST90001755	rs3123557	CD20 on IgD- CD3	1	24204073
ebi-a-GCST90001755	rs140120241	CD20 on IgD- CD3	1	166021518
ebi-a-GCST90001755	rs849532	CD20 on IgD- CD3	2	206552726
ebi-a-GCST90001755	rs187188344	CD20 on IgD- CD3	2	178858731
ebi-a-GCST90001755	rs62194499	CD20 on IgD- CD3	2	207417460
ebi-a-GCST90001755	rs7608595	CD20 on IgD- CD3	2	223088415
ebi-a-GCST90001755	rs57271503	CD20 on IgD- CD3	3	119244593
ebi-a-GCST90001755	rs9842470	CD20 on IgD- CD3	3	184221260
ebi-a-GCST90001755	rs2524184	CD20 on IgD- CD3	6	30424736
ebi-a-GCST90001755	rs12200643	CD20 on IgD- CD3	6	170846603
ebi-a-GCST90001755	rs11751198	CD20 on IgD- CD3	6	31753526
ebi-a-GCST90001755	rs111369841	CD20 on IgD- CD3	6	32921104
ebi-a-GCST90001755	rs75915436	CD20 on IgD- CD3	6	40648455
ebi-a-GCST90001755	rs4716010	CD20 on IgD- CD3	6	15898371

ebi-a-GCST90001755	rs9271146	CD20 on IgD- CD3	6	32577282
ebi-a-GCST90001755	rs2868177	CD20 on IgD- CD3	7	75589903
ebi-a-GCST90001755	rs1838178	CD20 on IgD- CD3	8	92858976
ebi-a-GCST90001755	rs75071948	CD20 on IgD- CD3	9	75768836
ebi-a-GCST90001755	rs78368496	CD20 on IgD- CD3	11	19984329
ebi-a-GCST90001755	rs10501383	CD20 on IgD- CD3	11	60164527
ebi-a-GCST90001755	rs61886501	CD20 on IgD- CD3	11	18870317
ebi-a-GCST90001755	rs2578469	CD20 on IgD- CD3	12	4101401
ebi-a-GCST90001755	rs185205613	CD20 on IgD- CD3	12	48074625
ebi-a-GCST90001755	rs9537439	CD20 on IgD- CD3	13	57128500
ebi-a-GCST90001755	rs7179991	CD20 on IgD- CD3	15	31811797
ebi-a-GCST90001755	rs16948899	CD20 on IgD- CD3	16	50866572
ebi-a-GCST90001755	rs179206	CD20 on IgD- CD3	16	19303610
ebi-a-GCST90001755	rs183569210	CD20 on IgD- CD3	18	72589044
ebi-a-GCST90001755	rs61731791	CD20 on IgD- CD3	19	50940778
ebi-a-GCST90001755	rs55809481	CD20 on IgD- CD3	19	49872521
ebi-a-GCST90001755	rs6120395	CD20 on IgD- CD3	20	32451693
ebi-a-GCST90001756	rs149234257	CD20 on IgD- CD3	2	7498941
ebi-a-GCST90001756	rs144229045	CD20 on IgD- CD3	3	132962425
ebi-a-GCST90001756	rs17552555	CD20 on IgD- CD3	4	111440302
ebi-a-GCST90001756	rs67448050	CD20 on IgD- CD3	5	91379430
ebi-a-GCST90001756	rs2735071	CD20 on IgD- CD3	6	29944979
ebi-a-GCST90001756	rs7751471	CD20 on IgD- CD3	6	155390391
ebi-a-GCST90001756	rs12530833	CD20 on IgD- CD3	7	11098867
ebi-a-GCST90001756	rs2060802	CD20 on IgD- CD3	8	39502217
ebi-a-GCST90001756	rs79868028	CD20 on IgD- CD3	10	133645848
ebi-a-GCST90001756	rs7302954	CD20 on IgD- CD3	12	92020720
ebi-a-GCST90001756	rs72665111	CD20 on IgD- CD3	13	103993749
ebi-a-GCST90001756	rs143489681	CD20 on IgD- CD3	15	94271829
ebi-a-GCST90001756	rs144576056	CD20 on IgD- CD3	17	54270936
ebi-a-GCST90001756	rs141311171	CD20 on IgD- CD3	17	77837606
ebi-a-GCST90001756	rs11668091	CD20 on IgD- CD3	19	51583553
ebi-a-GCST90001756	rs17878304	CD20 on IgD- CD3	19	4956161
ebi-a-GCST90001757	rs6693971	CD20 on IgD- CD3	1	3517573
ebi-a-GCST90001757	rs4657041	CD20 on IgD- CD3	1	161478859
ebi-a-GCST90001757	rs187188344	CD20 on IgD- CD3	2	178858731
ebi-a-GCST90001757	rs3961790	CD20 on IgD- CD3	2	80108045
ebi-a-GCST90001757	rs7608595	CD20 on IgD- CD3	2	223088415
ebi-a-GCST90001757	rs111345451	CD20 on IgD- CD3	3	141866322
ebi-a-GCST90001757	rs77612075	CD20 on IgD- CD3	5	162820460
ebi-a-GCST90001757	rs719170	CD20 on IgD- CD3	5	67432406
ebi-a-GCST90001757	rs9271146	CD20 on IgD- CD3	6	32577282
ebi-a-GCST90001757	rs9469053	CD20 on IgD- CD3	6	31755776
ebi-a-GCST90001757	rs909846	CD20 on IgD- CD3	6	15897003
ebi-a-GCST90001757	rs2524184	CD20 on IgD- CD3	6	30424736
ebi-a-GCST90001757	rs111369841	CD20 on IgD- CD3	6	32921104
ebi-a-GCST90001757	rs9473626	CD20 on IgD- CD3	6	49586550
ebi-a-GCST90001757	rs9650093	CD20 on IgD- CD3	8	127984089

ebi-a-GCST90001757	rs13275137	CD20 on IgD- CD3	8	36731742
ebi-a-GCST90001757	rs113560800	CD20 on IgD- CD3	9	29393202
ebi-a-GCST90001757	rs184418275	CD20 on IgD- CD3	10	89144616
ebi-a-GCST90001757	rs11187166	CD20 on IgD- CD3	10	94516008
ebi-a-GCST90001757	rs149908209	CD20 on IgD- CD3	11	127473611
ebi-a-GCST90001757	rs11020715	CD20 on IgD- CD3	11	93989208
ebi-a-GCST90001757	rs11230624	CD20 on IgD- CD3	11	60952231
ebi-a-GCST90001757	rs2070770	CD20 on IgD- CD3	11	60230531
ebi-a-GCST90001757	rs73025928	CD20 on IgD- CD3	11	123329504
ebi-a-GCST90001757	rs1447085	CD20 on IgD- CD3	12	127798516
ebi-a-GCST90001757	rs1955934	CD20 on IgD- CD3	14	37936489
ebi-a-GCST90001757	rs74541430	CD20 on IgD- CD3	16	26201701
ebi-a-GCST90001757	rs115308973	CD20 on IgD- CD3	16	88134541
ebi-a-GCST90001757	rs60743549	CD20 on IgD- CD3	18	60704462
ebi-a-GCST90001757	rs8110979	CD20 on IgD- CD3	19	6951925
ebi-a-GCST90001757	rs66579172	CD20 on IgD- CD3	19	50928270
ebi-a-GCST90001757	rs61731791	CD20 on IgD- CD3	19	50940778
ebi-a-GCST90001757	rs55809481	CD20 on IgD- CD3	19	49872521
ebi-a-GCST90001757	rs12480920	CD20 on IgD- CD3	20	3862580
ebi-a-GCST90001757	rs112898596	CD20 on IgD- CD3	22	42039509
ebi-a-GCST90001757	rs9625961	CD20 on IgD- CD3	22	44323219
ebi-a-GCST90001758	rs4657041	CD20 on memory E	1	161478859
ebi-a-GCST90001758	rs2492599	CD20 on memory E	1	24058449
ebi-a-GCST90001758	rs187188344	CD20 on memory E	2	178858731
ebi-a-GCST90001758	rs13080324	CD20 on memory E	3	111952407
ebi-a-GCST90001758	rs2114442	CD20 on memory E	4	100832060
ebi-a-GCST90001758	rs1422711	CD20 on memory E	5	143621838
ebi-a-GCST90001758	rs295654	CD20 on memory E	5	58107171
ebi-a-GCST90001758	rs77612075	CD20 on memory E	5	162820460
ebi-a-GCST90001758	rs111369841	CD20 on memory E	6	32921104
ebi-a-GCST90001758	rs13190846	CD20 on memory E	6	29905727
ebi-a-GCST90001758	rs13207893	CD20 on memory E	6	32586922
ebi-a-GCST90001758	rs9461562	CD20 on memory E	6	29778271
ebi-a-GCST90001758	rs233495	CD20 on memory E	6	14806317
ebi-a-GCST90001758	rs1569590	CD20 on memory E	6	15898884
ebi-a-GCST90001758	rs28572463	CD20 on memory E	6	31209854
ebi-a-GCST90001758	rs145640001	CD20 on memory E	8	97002096
ebi-a-GCST90001758	rs12554596	CD20 on memory E	9	37002142
ebi-a-GCST90001758	rs7035906	CD20 on memory E	9	95898832
ebi-a-GCST90001758	rs75071948	CD20 on memory E	9	75768836
ebi-a-GCST90001758	rs11187166	CD20 on memory E	10	94516008
ebi-a-GCST90001758	rs182220218	CD20 on memory E	10	75422693
ebi-a-GCST90001758	rs149695230	CD20 on memory E	11	60288804
ebi-a-GCST90001758	rs7128035	CD20 on memory E	11	268078
ebi-a-GCST90001758	rs2070770	CD20 on memory E	11	60230531
ebi-a-GCST90001758	rs2578469	CD20 on memory E	12	4101401
ebi-a-GCST90001758	rs193090598	CD20 on memory E	12	32460708
ebi-a-GCST90001758	rs179212	CD20 on memory E	16	19308868

ebi-a-GCST90001758	rs12460030	CD20 on memory E	19	3361489
ebi-a-GCST90001758	rs30003	CD20 on memory E	19	49846398
ebi-a-GCST90001758	rs61731791	CD20 on memory E	19	50940778
ebi-a-GCST90001759	rs111741355	CD20 on naive-mat	1	48095312
ebi-a-GCST90001759	rs12042193	CD20 on naive-mat	1	116031657
ebi-a-GCST90001759	rs113371046	CD20 on naive-mat	1	17595034
ebi-a-GCST90001759	rs149360160	CD20 on naive-mat	2	229499798
ebi-a-GCST90001759	rs114931155	CD20 on naive-mat	4	130319698
ebi-a-GCST90001759	rs73245560	CD20 on naive-mat	4	43987474
ebi-a-GCST90001759	rs3933208	CD20 on naive-mat	5	161458204
ebi-a-GCST90001759	rs72836542	CD20 on naive-mat	6	14091682
ebi-a-GCST90001759	rs233495	CD20 on naive-mat	6	14806317
ebi-a-GCST90001759	rs9270560	CD20 on naive-mat	6	32560741
ebi-a-GCST90001759	rs62405562	CD20 on naive-mat	6	32451834
ebi-a-GCST90001759	rs55643835	CD20 on naive-mat	6	33947909
ebi-a-GCST90001759	rs6910382	CD20 on naive-mat	6	15914349
ebi-a-GCST90001759	rs388354	CD20 on naive-mat	7	40190449
ebi-a-GCST90001759	rs117400501	CD20 on naive-mat	7	75444519
ebi-a-GCST90001759	rs143129208	CD20 on naive-mat	11	99391820
ebi-a-GCST90001759	rs1941018	CD20 on naive-mat	11	60268994
ebi-a-GCST90001759	rs11051833	CD20 on naive-mat	12	32330242
ebi-a-GCST90001759	rs10785682	CD20 on naive-mat	12	47837477
ebi-a-GCST90001759	rs6496328	CD20 on naive-mat	15	86988686
ebi-a-GCST90001759	rs8025257	CD20 on naive-mat	15	89600226
ebi-a-GCST90001759	rs7404890	CD20 on naive-mat	16	11007193
ebi-a-GCST90001759	rs36056466	CD20 on naive-mat	17	45083383
ebi-a-GCST90001759	rs30003	CD20 on naive-mat	19	49846398
ebi-a-GCST90001759	rs61731791	CD20 on naive-mat	19	50940778
ebi-a-GCST90001759	rs2413592	CD20 on naive-mat	22	39858196
ebi-a-GCST90001760	rs11205970	CD20 on unsw men	1	53008441
ebi-a-GCST90001760	rs187188344	CD20 on unsw men	2	178858731
ebi-a-GCST90001760	rs9310657	CD20 on unsw men	3	21797575
ebi-a-GCST90001760	rs13133046	CD20 on unsw men	4	23393356
ebi-a-GCST90001760	rs62340965	CD20 on unsw men	4	190508875
ebi-a-GCST90001760	rs2114442	CD20 on unsw men	4	100832060
ebi-a-GCST90001760	rs77612075	CD20 on unsw men	5	162820460
ebi-a-GCST90001760	rs77330263	CD20 on unsw men	6	149134235
ebi-a-GCST90001760	rs112405902	CD20 on unsw men	6	31809504
ebi-a-GCST90001760	rs2523761	CD20 on unsw men	6	29818726
ebi-a-GCST90001760	rs233495	CD20 on unsw men	6	14806317
ebi-a-GCST90001760	rs147717587	CD20 on unsw men	6	32623828
ebi-a-GCST90001760	rs6459417	CD20 on unsw men	6	15858996
ebi-a-GCST90001760	rs28572463	CD20 on unsw men	6	31209854
ebi-a-GCST90001760	rs12554596	CD20 on unsw men	9	37002142
ebi-a-GCST90001760	rs149312070	CD20 on unsw men	10	132155649
ebi-a-GCST90001760	rs10047347	CD20 on unsw men	10	26741516
ebi-a-GCST90001760	rs182220218	CD20 on unsw men	10	75422693
ebi-a-GCST90001760	rs4447195	CD20 on unsw men	11	60249734

ebi-a-GCST90001760	rs149695230	CD20 on unsw men	11	60288804
ebi-a-GCST90001760	rs149214933	CD20 on unsw men	13	64174465
ebi-a-GCST90001760	rs75146873	CD20 on unsw men	19	50953211
ebi-a-GCST90001760	rs6509428	CD20 on unsw men	19	49853881
ebi-a-GCST90001760	rs12460030	CD20 on unsw men	19	3361489
ebi-a-GCST90001760	rs78398983	CD20 on unsw men	22	32864978
ebi-a-GCST90001761	rs74446027	CD20 on sw mem	1	202171923
ebi-a-GCST90001761	rs12030328	CD20 on sw mem	1	24051614
ebi-a-GCST90001761	rs7608595	CD20 on sw mem	2	223088415
ebi-a-GCST90001761	rs137894231	CD20 on sw mem	2	29442718
ebi-a-GCST90001761	rs187188344	CD20 on sw mem	2	178858731
ebi-a-GCST90001761	rs78988304	CD20 on sw mem	3	25982156
ebi-a-GCST90001761	rs13134942	CD20 on sw mem	4	181179470
ebi-a-GCST90001761	rs77612075	CD20 on sw mem	5	162820460
ebi-a-GCST90001761	rs1569590	CD20 on sw mem	6	15898884
ebi-a-GCST90001761	rs11751198	CD20 on sw mem	6	31753526
ebi-a-GCST90001761	rs12200643	CD20 on sw mem	6	170846603
ebi-a-GCST90001761	rs9271146	CD20 on sw mem	6	32577282
ebi-a-GCST90001761	rs111369841	CD20 on sw mem	6	32921104
ebi-a-GCST90001761	rs2524184	CD20 on sw mem	6	30424736
ebi-a-GCST90001761	rs9650093	CD20 on sw mem	8	127984089
ebi-a-GCST90001761	rs11138252	CD20 on sw mem	9	82087111
ebi-a-GCST90001761	rs140207086	CD20 on sw mem	9	124822185
ebi-a-GCST90001761	rs75071948	CD20 on sw mem	9	75768836
ebi-a-GCST90001761	rs35039517	CD20 on sw mem	10	94522438
ebi-a-GCST90001761	rs73025942	CD20 on sw mem	11	123334349
ebi-a-GCST90001761	rs143129208	CD20 on sw mem	11	99391820
ebi-a-GCST90001761	rs7128035	CD20 on sw mem	11	268078
ebi-a-GCST90001761	rs11230624	CD20 on sw mem	11	60952231
ebi-a-GCST90001761	rs10501383	CD20 on sw mem	11	60164527
ebi-a-GCST90001761	rs185205613	CD20 on sw mem	12	48074625
ebi-a-GCST90001761	rs1447085	CD20 on sw mem	12	127798516
ebi-a-GCST90001761	rs187026890	CD20 on sw mem	15	93770077
ebi-a-GCST90001761	rs179206	CD20 on sw mem	16	19303610
ebi-a-GCST90001761	rs62122620	CD20 on sw mem	19	14871311
ebi-a-GCST90001761	rs55809481	CD20 on sw mem	19	49872521
ebi-a-GCST90001761	rs61731791	CD20 on sw mem	19	50940778
ebi-a-GCST90001761	rs8110979	CD20 on sw mem	19	6951925
ebi-a-GCST90001761	rs12480920	CD20 on sw mem	20	3862580
ebi-a-GCST90001761	rs112898596	CD20 on sw mem	22	42039509
ebi-a-GCST90001762	rs1293260	CD20 on IgD+	1	25709104
ebi-a-GCST90001762	rs113371046	CD20 on IgD+	1	17595034
ebi-a-GCST90001762	rs149360160	CD20 on IgD+	2	229499798
ebi-a-GCST90001762	rs79003098	CD20 on IgD+	2	81965038
ebi-a-GCST90001762	rs73245560	CD20 on IgD+	4	43987474
ebi-a-GCST90001762	rs77612075	CD20 on IgD+	5	162820460
ebi-a-GCST90001762	rs80155794	CD20 on IgD+	5	178957291
ebi-a-GCST90001762	rs3933208	CD20 on IgD+	5	161458204

ebi-a-GCST90001762	rs72836542	CD20 on IgD+	6	14091682
ebi-a-GCST90001762	rs9270560	CD20 on IgD+	6	32560741
ebi-a-GCST90001762	rs233495	CD20 on IgD+	6	14806317
ebi-a-GCST90001762	rs55643835	CD20 on IgD+	6	33947909
ebi-a-GCST90001762	rs62405562	CD20 on IgD+	6	32451834
ebi-a-GCST90001762	rs12212931	CD20 on IgD+	6	15913304
ebi-a-GCST90001762	rs388354	CD20 on IgD+	7	40190449
ebi-a-GCST90001762	rs4939384	CD20 on IgD+	11	60273642
ebi-a-GCST90001762	rs11051833	CD20 on IgD+	12	32330242
ebi-a-GCST90001762	rs11059642	CD20 on IgD+	12	128795644
ebi-a-GCST90001762	rs8025257	CD20 on IgD+	15	89600226
ebi-a-GCST90001762	rs6496328	CD20 on IgD+	15	86988686
ebi-a-GCST90001762	rs7404890	CD20 on IgD+	16	11007193
ebi-a-GCST90001762	rs30003	CD20 on IgD+	19	49846398
ebi-a-GCST90001762	rs73086355	CD20 on IgD+	20	11850850
ebi-a-GCST90001763	rs12026973	CD20 on transitiona	1	107824037
ebi-a-GCST90001763	rs72683667	CD20 on transitiona	1	78106407
ebi-a-GCST90001763	rs114212270	CD20 on transitiona	2	174337105
ebi-a-GCST90001763	rs12473410	CD20 on transitiona	2	115568051
ebi-a-GCST90001763	rs73175579	CD20 on transitiona	3	185524179
ebi-a-GCST90001763	rs114984432	CD20 on transitiona	3	42912480
ebi-a-GCST90001763	rs73252531	CD20 on transitiona	4	25162256
ebi-a-GCST90001763	rs146974582	CD20 on transitiona	5	158703283
ebi-a-GCST90001763	rs879299	CD20 on transitiona	6	15897007
ebi-a-GCST90001763	rs233495	CD20 on transitiona	6	14806317
ebi-a-GCST90001763	rs9273390	CD20 on transitiona	6	32627042
ebi-a-GCST90001763	rs72836542	CD20 on transitiona	6	14091682
ebi-a-GCST90001763	rs61011928	CD20 on transitiona	7	56434969
ebi-a-GCST90001763	rs34900401	CD20 on transitiona	7	148563716
ebi-a-GCST90001763	rs10958698	CD20 on transitiona	8	41641075
ebi-a-GCST90001763	rs76437378	CD20 on transitiona	9	83114502
ebi-a-GCST90001763	rs978366	CD20 on transitiona	10	95731909
ebi-a-GCST90001763	rs7939177	CD20 on transitiona	11	60266070
ebi-a-GCST90001763	rs11055535	CD20 on transitiona	12	13754468
ebi-a-GCST90001763	rs11627327	CD20 on transitiona	14	26441807
ebi-a-GCST90001763	rs145131147	CD20 on transitiona	14	55101477
ebi-a-GCST90001763	rs9910861	CD20 on transitiona	17	2801009
ebi-a-GCST90001763	rs117413283	CD20 on transitiona	18	47434681
ebi-a-GCST90001763	rs118186161	CD20 on transitiona	19	17549328
ebi-a-GCST90001763	rs34486765	CD20 on transitiona	19	49915911
ebi-a-GCST90001763	rs149341358	CD20 on transitiona	21	46442156
ebi-a-GCST90001764	rs34719184	CD24 on CD24+ C	2	546536
ebi-a-GCST90001764	rs113934809	CD24 on CD24+ C	2	191984672
ebi-a-GCST90001764	rs1471412	CD24 on CD24+ C	4	131810654
ebi-a-GCST90001765	rs629732	CD24 on IgD+ CD2	1	112356263
ebi-a-GCST90001765	rs143386390	CD24 on IgD+ CD2	1	6146588
ebi-a-GCST90001765	rs2902453	CD24 on IgD+ CD2	2	113923281
ebi-a-GCST90001765	rs34548572	CD24 on IgD+ CD2	3	4748193

ebi-a-GCST90001765	rs1471412	CD24 on IgD+ CD2	4	131810654
ebi-a-GCST90001765	rs4862454	CD24 on IgD+ CD2	4	185816062
ebi-a-GCST90001765	rs11930977	CD24 on IgD+ CD2	4	91342005
ebi-a-GCST90001765	rs17370695	CD24 on IgD+ CD2	5	92906685
ebi-a-GCST90001765	rs13171255	CD24 on IgD+ CD2	5	159374162
ebi-a-GCST90001765	rs12200293	CD24 on IgD+ CD2	6	101259222
ebi-a-GCST90001765	rs9273358	CD24 on IgD+ CD2	6	32626019
ebi-a-GCST90001765	rs2236073	CD24 on IgD+ CD2	6	106898421
ebi-a-GCST90001765	rs9400058	CD24 on IgD+ CD2	6	107164076
ebi-a-GCST90001765	rs150087880	CD24 on IgD+ CD2	6	66021291
ebi-a-GCST90001765	rs9478944	CD24 on IgD+ CD2	6	151400649
ebi-a-GCST90001765	rs144967366	CD24 on IgD+ CD2	6	57821837
ebi-a-GCST90001765	rs117513979	CD24 on IgD+ CD2	7	103968091
ebi-a-GCST90001765	rs147803894	CD24 on IgD+ CD2	10	28798971
ebi-a-GCST90001765	rs7116224	CD24 on IgD+ CD2	11	15734002
ebi-a-GCST90001765	rs189504940	CD24 on IgD+ CD2	12	87629976
ebi-a-GCST90001765	rs147649139	CD24 on IgD+ CD2	12	2310188
ebi-a-GCST90001765	rs4942468	CD24 on IgD+ CD2	13	46618290
ebi-a-GCST90001765	rs12930821	CD24 on IgD+ CD2	16	58054671
ebi-a-GCST90001765	rs117820766	CD24 on IgD+ CD2	18	61346454
ebi-a-GCST90001765	rs78157884	CD24 on IgD+ CD2	18	50026310
ebi-a-GCST90001765	rs285250	CD24 on IgD+ CD2	19	16416430
ebi-a-GCST90001766	rs11102943	CD24 on IgD+ CD3	1	115907459
ebi-a-GCST90001766	rs75307680	CD24 on IgD+ CD3	2	144289202
ebi-a-GCST90001766	rs77154655	CD24 on IgD+ CD3	3	16255245
ebi-a-GCST90001766	rs4435699	CD24 on IgD+ CD3	4	164019500
ebi-a-GCST90001766	rs144378737	CD24 on IgD+ CD3	4	144991868
ebi-a-GCST90001766	rs62358439	CD24 on IgD+ CD3	5	57060537
ebi-a-GCST90001766	rs1007713	CD24 on IgD+ CD3	5	79150995
ebi-a-GCST90001766	rs72782129	CD24 on IgD+ CD3	5	111200853
ebi-a-GCST90001766	rs9400058	CD24 on IgD+ CD3	6	107164076
ebi-a-GCST90001766	rs783418	CD24 on IgD+ CD3	6	106962679
ebi-a-GCST90001766	rs9479391	CD24 on IgD+ CD3	6	153107656
ebi-a-GCST90001766	rs2236073	CD24 on IgD+ CD3	6	106898421
ebi-a-GCST90001766	rs9270560	CD24 on IgD+ CD3	6	32560741
ebi-a-GCST90001766	rs11781090	CD24 on IgD+ CD3	8	144871633
ebi-a-GCST90001766	rs6481230	CD24 on IgD+ CD3	10	58477006
ebi-a-GCST90001766	rs138034828	CD24 on IgD+ CD3	10	111599201
ebi-a-GCST90001766	rs143053604	CD24 on IgD+ CD3	11	12834785
ebi-a-GCST90001766	rs11176619	CD24 on IgD+ CD3	12	40829042
ebi-a-GCST90001766	rs59866449	CD24 on IgD+ CD3	12	94556734
ebi-a-GCST90001766	rs77636548	CD24 on IgD+ CD3	14	43192207
ebi-a-GCST90001766	rs792792	CD24 on IgD+ CD3	17	51153533
ebi-a-GCST90001766	rs8089264	CD24 on IgD+ CD3	18	46221152
ebi-a-GCST90001767	rs114017670	CD24 on IgD+ CD3	1	40242025
ebi-a-GCST90001767	rs139742955	CD24 on IgD+ CD3	1	9926086
ebi-a-GCST90001767	rs34396417	CD24 on IgD+ CD3	2	121363691
ebi-a-GCST90001767	rs150910045	CD24 on IgD+ CD3	3	148279147

ebi-a-GCST90001767	rs1889263	CD24 on IgD+ CD3	3	175401058
ebi-a-GCST90001767	rs2236073	CD24 on IgD+ CD3	6	106898421
ebi-a-GCST90001767	rs9400058	CD24 on IgD+ CD3	6	107164076
ebi-a-GCST90001767	rs117218666	CD24 on IgD+ CD3	6	139700367
ebi-a-GCST90001767	rs55929002	CD24 on IgD+ CD3	6	33963845
ebi-a-GCST90001767	rs783414	CD24 on IgD+ CD3	6	106966701
ebi-a-GCST90001767	rs76624974	CD24 on IgD+ CD3	11	131992482
ebi-a-GCST90001767	rs2283164	CD24 on IgD+ CD3	11	2579163
ebi-a-GCST90001767	rs117488563	CD24 on IgD+ CD3	13	29183168
ebi-a-GCST90001767	rs35851357	CD24 on IgD+ CD3	14	88302344
ebi-a-GCST90001767	rs72682699	CD24 on IgD+ CD3	14	44731074
ebi-a-GCST90001767	rs74833732	CD24 on IgD+ CD3	14	20362976
ebi-a-GCST90001767	rs34534209	CD24 on IgD+ CD3	14	30498909
ebi-a-GCST90001767	rs16942616	CD24 on IgD+ CD3	16	87098993
ebi-a-GCST90001767	rs4569270	CD24 on IgD+ CD3	16	82557069
ebi-a-GCST90001767	rs75645526	CD24 on IgD+ CD3	16	71565056
ebi-a-GCST90001767	rs58241910	CD24 on IgD+ CD3	20	62864471
ebi-a-GCST90001767	rs78409132	CD24 on IgD+ CD3	21	16838053
ebi-a-GCST90001768	rs6666130	CD24 on IgD+ CD3	1	112374026
ebi-a-GCST90001768	rs114151424	CD24 on IgD+ CD3	1	120674028
ebi-a-GCST90001768	rs116032501	CD24 on IgD+ CD3	1	55983064
ebi-a-GCST90001768	rs59655785	CD24 on IgD+ CD3	2	152494218
ebi-a-GCST90001768	rs146379662	CD24 on IgD+ CD3	2	240272688
ebi-a-GCST90001768	rs181036992	CD24 on IgD+ CD3	3	196496410
ebi-a-GCST90001768	rs78405192	CD24 on IgD+ CD3	4	71295244
ebi-a-GCST90001768	rs10056811	CD24 on IgD+ CD3	5	74605220
ebi-a-GCST90001768	rs139160176	CD24 on IgD+ CD3	5	13902245
ebi-a-GCST90001768	rs35093039	CD24 on IgD+ CD3	5	24061216
ebi-a-GCST90001768	rs9326867	CD24 on IgD+ CD3	5	112302830
ebi-a-GCST90001768	rs9452529	CD24 on IgD+ CD3	6	94734554
ebi-a-GCST90001768	rs9398107	CD24 on IgD+ CD3	6	107155687
ebi-a-GCST90001768	rs1799693	CD24 on IgD+ CD3	6	106992464
ebi-a-GCST90001768	rs3097667	CD24 on IgD+ CD3	6	33023723
ebi-a-GCST90001768	rs7781145	CD24 on IgD+ CD3	7	23626602
ebi-a-GCST90001768	rs75059545	CD24 on IgD+ CD3	7	129584738
ebi-a-GCST90001768	rs10108273	CD24 on IgD+ CD3	8	85737573
ebi-a-GCST90001768	rs45609135	CD24 on IgD+ CD3	11	118942134
ebi-a-GCST90001768	rs192325628	CD24 on IgD+ CD3	11	121622513
ebi-a-GCST90001768	rs16923647	CD24 on IgD+ CD3	12	21451395
ebi-a-GCST90001768	rs4765854	CD24 on IgD+ CD3	12	1997152
ebi-a-GCST90001768	rs4368221	CD24 on IgD+ CD3	18	45634445
ebi-a-GCST90001768	rs117920360	CD24 on IgD+ CD3	18	58030683
ebi-a-GCST90001768	rs57680652	CD24 on IgD+ CD3	19	16362072
ebi-a-GCST90001769	rs142793352	CD24 on IgD- CD3	1	188139428
ebi-a-GCST90001769	rs10925304	CD24 on IgD- CD3	1	237235986
ebi-a-GCST90001769	rs2173295	CD24 on IgD- CD3	1	54943264
ebi-a-GCST90001769	rs72635970	CD24 on IgD- CD3	2	73868977
ebi-a-GCST90001769	rs9811806	CD24 on IgD- CD3	3	164038932

ebi-a-GCST90001769	rs2236073	CD24 on IgD- CD3	6	106898421
ebi-a-GCST90001769	rs72829705	CD24 on IgD- CD3	6	23390794
ebi-a-GCST90001769	rs9400058	CD24 on IgD- CD3	6	107164076
ebi-a-GCST90001769	rs76990892	CD24 on IgD- CD3	6	101131432
ebi-a-GCST90001769	rs1800542	CD24 on IgD- CD3	6	12292528
ebi-a-GCST90001769	rs117066281	CD24 on IgD- CD3	8	84921322
ebi-a-GCST90001769	rs17816653	CD24 on IgD- CD3	8	1284332
ebi-a-GCST90001769	rs75583168	CD24 on IgD- CD3	8	131650003
ebi-a-GCST90001769	rs74343204	CD24 on IgD- CD3	9	4327663
ebi-a-GCST90001769	rs117049974	CD24 on IgD- CD3	9	117755959
ebi-a-GCST90001769	rs111701276	CD24 on IgD- CD3	9	28274639
ebi-a-GCST90001769	rs314497	CD24 on IgD- CD3	11	115341932
ebi-a-GCST90001769	rs10777201	CD24 on IgD- CD3	12	77884772
ebi-a-GCST90001769	rs9574429	CD24 on IgD- CD3	13	80035438
ebi-a-GCST90001769	rs117080191	CD24 on IgD- CD3	13	102960469
ebi-a-GCST90001769	rs77636548	CD24 on IgD- CD3	14	43192207
ebi-a-GCST90001769	rs2895071	CD24 on IgD- CD3	15	96894306
ebi-a-GCST90001769	rs4784022	CD24 on IgD- CD3	16	58051178
ebi-a-GCST90001769	rs11568076	CD24 on IgD- CD3	17	4544520
ebi-a-GCST90001769	rs113883551	CD24 on IgD- CD3	18	24190315
ebi-a-GCST90001769	rs12968683	CD24 on IgD- CD3	18	23625106
ebi-a-GCST90001769	rs4808045	CD24 on IgD- CD3	19	16423035
ebi-a-GCST90001769	rs56913504	CD24 on IgD- CD3	20	52133353
ebi-a-GCST90001769	rs6073000	CD24 on IgD- CD3	20	41777449
ebi-a-GCST90001770	rs181819396	CD24 on IgD- CD3	1	191984411
ebi-a-GCST90001770	rs61820496	CD24 on IgD- CD3	1	158541235
ebi-a-GCST90001770	rs7532008	CD24 on IgD- CD3	1	150960350
ebi-a-GCST90001770	rs146949757	CD24 on IgD- CD3	1	177354939
ebi-a-GCST90001770	rs35629713	CD24 on IgD- CD3	2	156376531
ebi-a-GCST90001770	rs7606151	CD24 on IgD- CD3	2	88835687
ebi-a-GCST90001770	rs12642193	CD24 on IgD- CD3	4	25916457
ebi-a-GCST90001771	rs143386390	CD24 on memory E	1	6146588
ebi-a-GCST90001771	rs77875928	CD24 on memory E	2	137032557
ebi-a-GCST90001771	rs2902453	CD24 on memory E	2	113923281
ebi-a-GCST90001771	rs73214037	CD24 on memory E	3	197395630
ebi-a-GCST90001771	rs4862454	CD24 on memory E	4	185816062
ebi-a-GCST90001771	rs78023498	CD24 on memory E	4	189754606
ebi-a-GCST90001771	rs138093394	CD24 on memory E	4	39960496
ebi-a-GCST90001771	rs7705021	CD24 on memory E	5	79153359
ebi-a-GCST90001771	rs72948545	CD24 on memory E	6	107168477
ebi-a-GCST90001771	rs150394154	CD24 on memory E	6	32556019
ebi-a-GCST90001771	rs9390863	CD24 on memory E	6	96559132
ebi-a-GCST90001771	rs12200293	CD24 on memory E	6	101259222
ebi-a-GCST90001771	rs2236073	CD24 on memory E	6	106898421
ebi-a-GCST90001771	rs9392365	CD24 on memory E	6	2091975
ebi-a-GCST90001771	rs117417471	CD24 on memory E	10	56973794
ebi-a-GCST90001771	rs7078071	CD24 on memory E	10	124038072
ebi-a-GCST90001771	rs192325628	CD24 on memory E	11	121622513

ebi-a-GCST90001771	rs11023691	CD24 on memory E	11	15732077
ebi-a-GCST90001771	rs77604725	CD24 on memory E	12	11210672
ebi-a-GCST90001771	rs117918490	CD24 on memory E	12	83630901
ebi-a-GCST90001771	rs6561591	CD24 on memory E	13	51367125
ebi-a-GCST90001771	rs4942468	CD24 on memory E	13	46618290
ebi-a-GCST90001771	rs4911068	CD24 on memory E	13	85904508
ebi-a-GCST90001771	rs117084919	CD24 on memory E	13	112826783
ebi-a-GCST90001771	rs7153153	CD24 on memory E	14	89049488
ebi-a-GCST90001771	rs144371214	CD24 on memory E	14	88425584
ebi-a-GCST90001771	rs139648894	CD24 on memory E	16	8831083
ebi-a-GCST90001771	rs182058654	CD24 on memory E	16	81440009
ebi-a-GCST90001771	rs12930821	CD24 on memory E	16	58054671
ebi-a-GCST90001771	rs113242537	CD24 on memory E	18	75792336
ebi-a-GCST90001771	rs117820766	CD24 on memory E	18	61346454
ebi-a-GCST90001771	rs7246669	CD24 on memory E	19	16442023
ebi-a-GCST90001771	rs55976703	CD24 on memory E	19	35785378
ebi-a-GCST90001772	rs141846315	CD24 on unsw men	1	173414084
ebi-a-GCST90001772	rs2902453	CD24 on unsw men	2	113923281
ebi-a-GCST90001772	rs112878393	CD24 on unsw men	3	125610212
ebi-a-GCST90001772	rs4862454	CD24 on unsw men	4	185816062
ebi-a-GCST90001772	rs77062870	CD24 on unsw men	5	168607069
ebi-a-GCST90001772	rs74726196	CD24 on unsw men	5	122019220
ebi-a-GCST90001772	rs7705021	CD24 on unsw men	5	79153359
ebi-a-GCST90001772	rs783418	CD24 on unsw men	6	106962679
ebi-a-GCST90001772	rs9480626	CD24 on unsw men	6	106415916
ebi-a-GCST90001772	rs9400058	CD24 on unsw men	6	107164076
ebi-a-GCST90001772	rs28752534	CD24 on unsw men	6	32585068
ebi-a-GCST90001772	rs2236073	CD24 on unsw men	6	106898421
ebi-a-GCST90001772	rs117513979	CD24 on unsw men	7	103968091
ebi-a-GCST90001772	rs34872621	CD24 on unsw men	8	120710403
ebi-a-GCST90001772	rs7835717	CD24 on unsw men	8	134992585
ebi-a-GCST90001772	rs77899115	CD24 on unsw men	13	83968655
ebi-a-GCST90001772	rs61969021	CD24 on unsw men	13	80019033
ebi-a-GCST90001772	rs77636548	CD24 on unsw men	14	43192207
ebi-a-GCST90001772	rs7153153	CD24 on unsw men	14	89049488
ebi-a-GCST90001772	rs12930821	CD24 on unsw men	16	58054671
ebi-a-GCST90001772	rs9916833	CD24 on unsw men	17	64969001
ebi-a-GCST90001772	rs11661274	CD24 on unsw men	18	53832059
ebi-a-GCST90001772	rs117772689	CD24 on unsw men	20	40633066
ebi-a-GCST90001772	rs74273424	CD24 on unsw men	20	46848498
ebi-a-GCST90001772	rs6068718	CD24 on unsw men	20	52528229
ebi-a-GCST90001773	rs181819396	CD24 on sw mem	1	191984411
ebi-a-GCST90001773	rs61820496	CD24 on sw mem	1	158541235
ebi-a-GCST90001773	rs116584882	CD24 on sw mem	1	232401896
ebi-a-GCST90001773	rs71419048	CD24 on sw mem	2	156299791
ebi-a-GCST90001773	rs4629120	CD24 on sw mem	2	30045059
ebi-a-GCST90001773	rs17035466	CD24 on sw mem	3	1249656
ebi-a-GCST90001773	rs9838297	CD24 on sw mem	3	150777263

ebi-a-GCST90001773	rs6799202	CD24 on sw mem	3	131412454
ebi-a-GCST90001773	rs12642193	CD24 on sw mem	4	25916457
ebi-a-GCST90001773	rs2872004	CD24 on sw mem	4	131805665
ebi-a-GCST90001773	rs149975454	CD24 on sw mem	4	54436158
ebi-a-GCST90001773	rs72948545	CD24 on sw mem	6	107168477
ebi-a-GCST90001773	rs7756279	CD24 on sw mem	6	107458285
ebi-a-GCST90001773	rs28672722	CD24 on sw mem	6	32626537
ebi-a-GCST90001773	rs76990892	CD24 on sw mem	6	101131432
ebi-a-GCST90001773	rs2236073	CD24 on sw mem	6	106898421
ebi-a-GCST90001773	rs783418	CD24 on sw mem	6	106962679
ebi-a-GCST90001773	rs73602758	CD24 on sw mem	6	149812163
ebi-a-GCST90001773	rs57225931	CD24 on sw mem	7	152228997
ebi-a-GCST90001773	rs183671978	CD24 on sw mem	7	124374557
ebi-a-GCST90001773	rs111701276	CD24 on sw mem	9	28274639
ebi-a-GCST90001773	rs117896078	CD24 on sw mem	9	130751060
ebi-a-GCST90001773	rs147397792	CD24 on sw mem	10	18427081
ebi-a-GCST90001773	rs141357554	CD24 on sw mem	10	74099495
ebi-a-GCST90001773	rs4427557	CD24 on sw mem	11	40861756
ebi-a-GCST90001773	rs10750479	CD24 on sw mem	11	131277609
ebi-a-GCST90001773	rs147031010	CD24 on sw mem	12	100147551
ebi-a-GCST90001773	rs6561591	CD24 on sw mem	13	51367125
ebi-a-GCST90001773	rs73251366	CD24 on sw mem	13	83905396
ebi-a-GCST90001773	rs9574429	CD24 on sw mem	13	80035438
ebi-a-GCST90001773	rs77636548	CD24 on sw mem	14	43192207
ebi-a-GCST90001773	rs2895071	CD24 on sw mem	15	96894306
ebi-a-GCST90001773	rs8046913	CD24 on sw mem	16	58039513
ebi-a-GCST90001773	rs11568076	CD24 on sw mem	17	4544520
ebi-a-GCST90001773	rs7214354	CD24 on sw mem	17	51813250
ebi-a-GCST90001773	rs11660990	CD24 on sw mem	18	50763049
ebi-a-GCST90001773	rs4808045	CD24 on sw mem	19	16423035
ebi-a-GCST90001773	rs6073000	CD24 on sw mem	20	41777449
ebi-a-GCST90001773	rs73301642	CD24 on sw mem	20	59456250
ebi-a-GCST90001774	rs35378339	CD24 on transitiona	1	32639057
ebi-a-GCST90001774	rs140315354	CD24 on transitiona	1	161617673
ebi-a-GCST90001774	rs76224749	CD24 on transitiona	1	238632927
ebi-a-GCST90001774	rs115246881	CD24 on transitiona	2	155206362
ebi-a-GCST90001774	rs116100555	CD24 on transitiona	2	7324854
ebi-a-GCST90001774	rs17778282	CD24 on transitiona	4	2947538
ebi-a-GCST90001774	rs73018738	CD24 on transitiona	4	189769692
ebi-a-GCST90001774	rs10491456	CD24 on transitiona	5	79152076
ebi-a-GCST90001774	rs12206866	CD24 on transitiona	6	107159822
ebi-a-GCST90001774	rs9390863	CD24 on transitiona	6	96559132
ebi-a-GCST90001774	rs783418	CD24 on transitiona	6	106962679
ebi-a-GCST90001774	rs55846017	CD24 on transitiona	6	106902438
ebi-a-GCST90001774	rs10245878	CD24 on transitiona	7	78032692
ebi-a-GCST90001774	rs17709988	CD24 on transitiona	9	14420767
ebi-a-GCST90001774	rs117417471	CD24 on transitiona	10	56973794
ebi-a-GCST90001774	rs74749898	CD24 on transitiona	11	128808891

ebi-a-GCST90001774	rs192325628	CD24 on transitiona	11	121622513
ebi-a-GCST90001774	rs16944983	CD24 on transitiona	15	62700919
ebi-a-GCST90001774	rs148635335	CD24 on transitiona	18	21787537
ebi-a-GCST90001774	rs117772689	CD24 on transitiona	20	40633066
ebi-a-GCST90001775	rs9425892	CD25 on B cell	1	34421513
ebi-a-GCST90001775	rs72944931	CD25 on B cell	2	178116262
ebi-a-GCST90001775	rs2139146	CD25 on B cell	2	15014620
ebi-a-GCST90001775	rs62175769	CD25 on B cell	2	173865326
ebi-a-GCST90001775	rs62157931	CD25 on B cell	2	67743098
ebi-a-GCST90001775	rs1529188	CD25 on B cell	3	109505175
ebi-a-GCST90001775	rs112043339	CD25 on B cell	3	174935606
ebi-a-GCST90001775	rs116525939	CD25 on B cell	5	97611924
ebi-a-GCST90001775	rs2119002	CD25 on B cell	5	124530767
ebi-a-GCST90001775	rs4869953	CD25 on B cell	6	151209997
ebi-a-GCST90001775	rs72997862	CD25 on B cell	6	144585977
ebi-a-GCST90001775	rs149662107	CD25 on B cell	7	47871972
ebi-a-GCST90001775	rs3793662	CD25 on B cell	9	95886051
ebi-a-GCST90001775	rs7869376	CD25 on B cell	9	72118434
ebi-a-GCST90001775	rs147487472	CD25 on B cell	10	6191376
ebi-a-GCST90001775	rs41294937	CD25 on B cell	10	6106621
ebi-a-GCST90001775	rs11051398	CD25 on B cell	12	31504316
ebi-a-GCST90001775	rs11615354	CD25 on B cell	12	2130438
ebi-a-GCST90001775	rs2248769	CD25 on B cell	13	54938910
ebi-a-GCST90001775	rs112782756	CD25 on B cell	13	34506073
ebi-a-GCST90001775	rs11617559	CD25 on B cell	13	24752225
ebi-a-GCST90001775	rs557577736	CD25 on B cell	13	46224092
ebi-a-GCST90001775	rs8028696	CD25 on B cell	15	33466839
ebi-a-GCST90001775	rs117156856	CD25 on B cell	16	46798678
ebi-a-GCST90001775	rs139491278	CD25 on B cell	17	34986043
ebi-a-GCST90001775	rs17002085	CD25 on B cell	21	27750139
ebi-a-GCST90001776	rs1731253	CD25 on CD20- CI	2	26876429
ebi-a-GCST90001776	rs12492348	CD25 on CD20- CI	3	187826332
ebi-a-GCST90001776	rs2470644	CD25 on CD20- CI	3	5776857
ebi-a-GCST90001776	rs13435269	CD25 on CD20- CI	4	95543425
ebi-a-GCST90001776	rs6838669	CD25 on CD20- CI	4	182081065
ebi-a-GCST90001776	rs4446675	CD25 on CD20- CI	7	103397333
ebi-a-GCST90001776	rs141602962	CD25 on CD20- CI	9	7815826
ebi-a-GCST90001776	rs140714148	CD25 on CD20- CI	12	2859329
ebi-a-GCST90001776	rs75543866	CD25 on CD20- CI	12	64338105
ebi-a-GCST90001776	rs35926650	CD25 on CD20- CI	12	32550835
ebi-a-GCST90001776	rs1628296	CD25 on CD20- CI	12	123309183
ebi-a-GCST90001776	rs188435636	CD25 on CD20- CI	12	78750126
ebi-a-GCST90001776	rs9595021	CD25 on CD20- CI	13	44387580
ebi-a-GCST90001776	rs66495237	CD25 on CD20- CI	14	74734614
ebi-a-GCST90001776	rs138027128	CD25 on CD20- CI	16	7282705
ebi-a-GCST90001776	rs553329467	CD25 on CD20- CI	16	15087287
ebi-a-GCST90001776	rs62066495	CD25 on CD20- CI	17	16893180
ebi-a-GCST90001776	rs58969602	CD25 on CD20- CI	20	47890347

ebi-a-GCST90001776	rs146320332	CD25 on CD20- CI	20	10238099
ebi-a-GCST90001776	rs144856909	CD25 on CD20- CI	22	29988779
ebi-a-GCST90001777	rs12139506	CD25 on CD24+ C.	1	61376720
ebi-a-GCST90001777	rs1660369	CD25 on CD24+ C.	1	214407674
ebi-a-GCST90001777	rs191775575	CD25 on CD24+ C.	2	119062858
ebi-a-GCST90001777	rs62135678	CD25 on CD24+ C.	2	33169113
ebi-a-GCST90001777	rs2139146	CD25 on CD24+ C.	2	15014620
ebi-a-GCST90001777	rs78990890	CD25 on CD24+ C.	4	161318933
ebi-a-GCST90001777	rs7708967	CD25 on CD24+ C.	5	8139657
ebi-a-GCST90001777	rs12192925	CD25 on CD24+ C.	6	37488754
ebi-a-GCST90001777	rs2999572	CD25 on CD24+ C.	7	25590299
ebi-a-GCST90001777	rs140833732	CD25 on CD24+ C.	8	36436637
ebi-a-GCST90001777	rs77303104	CD25 on CD24+ C.	9	108634724
ebi-a-GCST90001777	rs3793662	CD25 on CD24+ C.	9	95886051
ebi-a-GCST90001777	rs11245200	CD25 on CD24+ C.	10	126182752
ebi-a-GCST90001777	rs2853765	CD25 on CD24+ C.	10	7830675
ebi-a-GCST90001777	rs11598494	CD25 on CD24+ C.	10	6178941
ebi-a-GCST90001777	rs4747882	CD25 on CD24+ C.	10	6162476
ebi-a-GCST90001777	rs1887027	CD25 on CD24+ C.	10	6113782
ebi-a-GCST90001777	rs59278703	CD25 on CD24+ C.	11	35069685
ebi-a-GCST90001777	rs12886102	CD25 on CD24+ C.	14	100108322
ebi-a-GCST90001777	rs183975337	CD25 on CD24+ C.	14	89659141
ebi-a-GCST90001777	rs12907742	CD25 on CD24+ C.	15	101525737
ebi-a-GCST90001777	rs10852336	CD25 on CD24+ C.	16	11665465
ebi-a-GCST90001777	rs111912558	CD25 on CD24+ C.	17	44356694
ebi-a-GCST90001777	rs28365327	CD25 on CD24+ C.	18	25753552
ebi-a-GCST90001777	rs536374	CD25 on CD24+ C.	18	34917959
ebi-a-GCST90001777	rs3862036	CD25 on CD24+ C.	21	44262209
ebi-a-GCST90001777	rs144182927	CD25 on CD24+ C.	21	23113704
ebi-a-GCST90001777	rs200381543	CD25 on CD24+ C.	22	22547864
ebi-a-GCST90001778	rs12739411	CD25 on IgD+ CD2	1	68752573
ebi-a-GCST90001778	rs12139506	CD25 on IgD+ CD2	1	61376720
ebi-a-GCST90001778	rs72944931	CD25 on IgD+ CD2	2	178116262
ebi-a-GCST90001778	rs72840831	CD25 on IgD+ CD2	2	119131895
ebi-a-GCST90001778	rs11712686	CD25 on IgD+ CD2	3	132485194
ebi-a-GCST90001778	rs62282025	CD25 on IgD+ CD2	3	109521072
ebi-a-GCST90001778	rs6789981	CD25 on IgD+ CD2	3	173712563
ebi-a-GCST90001778	rs149905818	CD25 on IgD+ CD2	4	106231334
ebi-a-GCST90001778	rs10903228	CD25 on IgD+ CD2	5	178077656
ebi-a-GCST90001778	rs181153632	CD25 on IgD+ CD2	7	30546081
ebi-a-GCST90001778	rs2999572	CD25 on IgD+ CD2	7	25590299
ebi-a-GCST90001778	rs145755586	CD25 on IgD+ CD2	7	151448932
ebi-a-GCST90001778	rs75711329	CD25 on IgD+ CD2	8	75494833
ebi-a-GCST90001778	rs3793662	CD25 on IgD+ CD2	9	95886051
ebi-a-GCST90001778	rs2853765	CD25 on IgD+ CD2	10	7830675
ebi-a-GCST90001778	rs11245200	CD25 on IgD+ CD2	10	126182752
ebi-a-GCST90001778	rs4747882	CD25 on IgD+ CD2	10	6162476
ebi-a-GCST90001778	rs1887027	CD25 on IgD+ CD2	10	6113782

ebi-a-GCST90001778	rs11598494	CD25 on IgD+ CD2	10	6178941
ebi-a-GCST90001778	rs59278703	CD25 on IgD+ CD2	11	35069685
ebi-a-GCST90001778	rs4764609	CD25 on IgD+ CD2	12	5968848
ebi-a-GCST90001778	rs12428279	CD25 on IgD+ CD2	13	74434331
ebi-a-GCST90001778	rs62013832	CD25 on IgD+ CD2	15	86804118
ebi-a-GCST90001778	rs8079462	CD25 on IgD+ CD2	17	47002447
ebi-a-GCST90001778	rs28365327	CD25 on IgD+ CD2	18	25753552
ebi-a-GCST90001778	rs536374	CD25 on IgD+ CD2	18	34917959
ebi-a-GCST90001778	rs75727877	CD25 on IgD+ CD2	20	50852063
ebi-a-GCST90001779	rs9425892	CD25 on IgD+ CD2	1	34421513
ebi-a-GCST90001779	rs10495086	CD25 on IgD+ CD2	1	218109675
ebi-a-GCST90001779	rs111741355	CD25 on IgD+ CD2	1	48095312
ebi-a-GCST90001779	rs338614	CD25 on IgD+ CD2	3	28137648
ebi-a-GCST90001779	rs12638268	CD25 on IgD+ CD2	3	11933438
ebi-a-GCST90001779	rs112043339	CD25 on IgD+ CD2	3	174935606
ebi-a-GCST90001779	rs17460904	CD25 on IgD+ CD2	4	36354057
ebi-a-GCST90001779	rs35704968	CD25 on IgD+ CD2	4	8011229
ebi-a-GCST90001779	rs34518596	CD25 on IgD+ CD2	5	86111282
ebi-a-GCST90001779	rs4869953	CD25 on IgD+ CD2	6	151209997
ebi-a-GCST90001779	rs1688627	CD25 on IgD+ CD2	6	119162482
ebi-a-GCST90001779	rs149662107	CD25 on IgD+ CD2	7	47871972
ebi-a-GCST90001779	rs3793662	CD25 on IgD+ CD2	9	95886051
ebi-a-GCST90001779	rs17403348	CD25 on IgD+ CD2	10	5827901
ebi-a-GCST90001779	rs7906697	CD25 on IgD+ CD2	10	125554970
ebi-a-GCST90001779	rs147487472	CD25 on IgD+ CD2	10	6191376
ebi-a-GCST90001779	rs142537458	CD25 on IgD+ CD2	10	5982082
ebi-a-GCST90001779	rs62626323	CD25 on IgD+ CD2	10	6115678
ebi-a-GCST90001779	rs59278703	CD25 on IgD+ CD2	11	35069685
ebi-a-GCST90001779	rs11051398	CD25 on IgD+ CD2	12	31504316
ebi-a-GCST90001779	rs112328981	CD25 on IgD+ CD2	13	34477079
ebi-a-GCST90001779	rs709589	CD25 on IgD+ CD2	14	106306841
ebi-a-GCST90001779	rs118088397	CD25 on IgD+ CD2	16	11709659
ebi-a-GCST90001779	rs148048180	CD25 on IgD+ CD2	19	9181620
ebi-a-GCST90001779	rs62206862	CD25 on IgD+ CD2	20	61967801
ebi-a-GCST90001779	rs13051777	CD25 on IgD+ CD2	21	20241615
ebi-a-GCST90001780	rs12139506	CD25 on IgD+ CD3	1	61376720
ebi-a-GCST90001780	rs185124612	CD25 on IgD+ CD3	2	107128298
ebi-a-GCST90001780	rs74643586	CD25 on IgD+ CD3	2	80478582
ebi-a-GCST90001780	rs2139146	CD25 on IgD+ CD3	2	15014620
ebi-a-GCST90001780	rs80196851	CD25 on IgD+ CD3	2	173789113
ebi-a-GCST90001780	rs77154655	CD25 on IgD+ CD3	3	16255245
ebi-a-GCST90001780	rs115861292	CD25 on IgD+ CD3	3	126541810
ebi-a-GCST90001780	rs146092993	CD25 on IgD+ CD3	3	28261321
ebi-a-GCST90001780	rs138409352	CD25 on IgD+ CD3	3	10050183
ebi-a-GCST90001780	rs3103947	CD25 on IgD+ CD3	3	195552071
ebi-a-GCST90001780	rs74882681	CD25 on IgD+ CD3	4	80724150
ebi-a-GCST90001780	rs149905818	CD25 on IgD+ CD3	4	106231334
ebi-a-GCST90001780	rs12655234	CD25 on IgD+ CD3	5	150592481

ebi-a-GCST90001780	rs13161074	CD25 on IgD+ CD3	5	178057466
ebi-a-GCST90001780	rs262753	CD25 on IgD+ CD3	5	165060876
ebi-a-GCST90001780	rs73433764	CD25 on IgD+ CD3	7	122201968
ebi-a-GCST90001780	rs149662107	CD25 on IgD+ CD3	7	47871972
ebi-a-GCST90001780	rs11516157	CD25 on IgD+ CD3	9	136406758
ebi-a-GCST90001780	rs3793662	CD25 on IgD+ CD3	9	95886051
ebi-a-GCST90001780	rs4747846	CD25 on IgD+ CD3	10	6074451
ebi-a-GCST90001780	rs41295093	CD25 on IgD+ CD3	10	6120173
ebi-a-GCST90001780	rs11597205	CD25 on IgD+ CD3	10	132601293
ebi-a-GCST90001780	rs59745557	CD25 on IgD+ CD3	11	35069686
ebi-a-GCST90001780	rs7926898	CD25 on IgD+ CD3	11	133650237
ebi-a-GCST90001780	rs1718125	CD25 on IgD+ CD3	12	121593019
ebi-a-GCST90001780	rs117310389	CD25 on IgD+ CD3	13	58019264
ebi-a-GCST90001780	rs140678325	CD25 on IgD+ CD3	13	42323712
ebi-a-GCST90001780	rs2665962	CD25 on IgD+ CD3	17	74022213
ebi-a-GCST90001780	rs75727877	CD25 on IgD+ CD3	20	50852063
ebi-a-GCST90001781	rs696306	CD25 on IgD+ CD3	1	7854998
ebi-a-GCST90001781	rs10920477	CD25 on IgD+ CD3	1	190053879
ebi-a-GCST90001781	rs113066333	CD25 on IgD+ CD3	2	28889318
ebi-a-GCST90001781	rs72948393	CD25 on IgD+ CD3	2	215843408
ebi-a-GCST90001781	rs114964656	CD25 on IgD+ CD3	2	182116922
ebi-a-GCST90001781	rs2139146	CD25 on IgD+ CD3	2	15014620
ebi-a-GCST90001781	rs76504408	CD25 on IgD+ CD3	2	11202300
ebi-a-GCST90001781	rs76032804	CD25 on IgD+ CD3	3	173656076
ebi-a-GCST90001781	rs2616542	CD25 on IgD+ CD3	3	11688393
ebi-a-GCST90001781	rs111926422	CD25 on IgD+ CD3	3	157871039
ebi-a-GCST90001781	rs9293516	CD25 on IgD+ CD3	5	88747297
ebi-a-GCST90001781	rs12655234	CD25 on IgD+ CD3	5	150592481
ebi-a-GCST90001781	rs147734841	CD25 on IgD+ CD3	5	112536717
ebi-a-GCST90001781	rs4593388	CD25 on IgD+ CD3	6	159340868
ebi-a-GCST90001781	rs578114150	CD25 on IgD+ CD3	7	65244440
ebi-a-GCST90001781	rs142906903	CD25 on IgD+ CD3	8	82141410
ebi-a-GCST90001781	rs139976226	CD25 on IgD+ CD3	8	78082998
ebi-a-GCST90001781	rs35504411	CD25 on IgD+ CD3	8	6766251
ebi-a-GCST90001781	rs2275848	CD25 on IgD+ CD3	9	95887320
ebi-a-GCST90001781	rs41294937	CD25 on IgD+ CD3	10	6106621
ebi-a-GCST90001781	rs10843928	CD25 on IgD+ CD3	12	31502991
ebi-a-GCST90001781	rs709589	CD25 on IgD+ CD3	14	106306841
ebi-a-GCST90001781	rs118088397	CD25 on IgD+ CD3	16	11709659
ebi-a-GCST90001781	rs716408	CD25 on IgD+ CD3	17	32309424
ebi-a-GCST90001781	rs2267601	CD25 on IgD+ CD3	22	44448338
ebi-a-GCST90001782	rs9426902	CD25 on IgD+ CD3	1	153718746
ebi-a-GCST90001782	rs143132954	CD25 on IgD+ CD3	1	61322867
ebi-a-GCST90001782	rs77910234	CD25 on IgD+ CD3	1	224393629
ebi-a-GCST90001782	rs12122434	CD25 on IgD+ CD3	1	63261652
ebi-a-GCST90001782	rs11673894	CD25 on IgD+ CD3	2	118876072
ebi-a-GCST90001782	rs113066333	CD25 on IgD+ CD3	2	28889318
ebi-a-GCST90001782	rs17043899	CD25 on IgD+ CD3	2	115757709

ebi-a-GCST90001782	rs191448128	CD25 on IgD+ CD3	3	129963207
ebi-a-GCST90001782	rs116270586	CD25 on IgD+ CD3	3	121050031
ebi-a-GCST90001782	rs114201769	CD25 on IgD+ CD3	3	48005507
ebi-a-GCST90001782	rs62246874	CD25 on IgD+ CD3	3	66354137
ebi-a-GCST90001782	rs58327586	CD25 on IgD+ CD3	6	364476
ebi-a-GCST90001782	rs117315714	CD25 on IgD+ CD3	7	46669974
ebi-a-GCST90001782	rs72657101	CD25 on IgD+ CD3	8	62471902
ebi-a-GCST90001782	rs139703496	CD25 on IgD+ CD3	10	1676361
ebi-a-GCST90001782	rs2853765	CD25 on IgD+ CD3	10	7830675
ebi-a-GCST90001782	rs12218872	CD25 on IgD+ CD3	10	6177617
ebi-a-GCST90001782	rs10905716	CD25 on IgD+ CD3	10	6114010
ebi-a-GCST90001782	rs73084854	CD25 on IgD+ CD3	12	39565803
ebi-a-GCST90001782	rs2908671	CD25 on IgD+ CD3	16	5056498
ebi-a-GCST90001782	rs9932192	CD25 on IgD+ CD3	16	14510543
ebi-a-GCST90001782	rs2052087	CD25 on IgD+ CD3	17	56242749
ebi-a-GCST90001782	rs113410977	CD25 on IgD+ CD3	18	42381085
ebi-a-GCST90001782	rs8097813	CD25 on IgD+ CD3	18	67173624
ebi-a-GCST90001782	rs148503715	CD25 on IgD+ CD3	20	61958726
ebi-a-GCST90001783	rs2566772	CD25 on IgD+ CD3	1	68704099
ebi-a-GCST90001783	rs145198352	CD25 on IgD+ CD3	2	44009835
ebi-a-GCST90001783	rs1828160	CD25 on IgD+ CD3	3	7379480
ebi-a-GCST90001783	rs6550133	CD25 on IgD+ CD3	3	32472280
ebi-a-GCST90001783	rs4721574	CD25 on IgD+ CD3	7	2350451
ebi-a-GCST90001783	rs12341626	CD25 on IgD+ CD3	9	17383792
ebi-a-GCST90001783	rs3793662	CD25 on IgD+ CD3	9	95886051
ebi-a-GCST90001783	rs4129608	CD25 on IgD+ CD3	9	18327215
ebi-a-GCST90001783	rs62626323	CD25 on IgD+ CD3	10	6115678
ebi-a-GCST90001783	rs10765088	CD25 on IgD+ CD3	10	133336906
ebi-a-GCST90001783	rs11616609	CD25 on IgD+ CD3	13	35106024
ebi-a-GCST90001783	rs9596941	CD25 on IgD+ CD3	13	54874198
ebi-a-GCST90001783	rs709589	CD25 on IgD+ CD3	14	106306841
ebi-a-GCST90001783	rs10047878	CD25 on IgD+ CD3	14	22787690
ebi-a-GCST90001783	rs8044922	CD25 on IgD+ CD3	16	928708
ebi-a-GCST90001783	rs6092493	CD25 on IgD+ CD3	20	56109595
ebi-a-GCST90001784	rs111741355	CD25 on IgD+ CD3	1	48095312
ebi-a-GCST90001784	rs9425892	CD25 on IgD+ CD3	1	34421513
ebi-a-GCST90001784	rs72944931	CD25 on IgD+ CD3	2	178116262
ebi-a-GCST90001784	rs112043339	CD25 on IgD+ CD3	3	174935606
ebi-a-GCST90001784	rs35704968	CD25 on IgD+ CD3	4	8011229
ebi-a-GCST90001784	rs116525939	CD25 on IgD+ CD3	5	97611924
ebi-a-GCST90001784	rs4869953	CD25 on IgD+ CD3	6	151209997
ebi-a-GCST90001784	rs149662107	CD25 on IgD+ CD3	7	47871972
ebi-a-GCST90001784	rs116970311	CD25 on IgD+ CD3	7	50262040
ebi-a-GCST90001784	rs3793662	CD25 on IgD+ CD3	9	95886051
ebi-a-GCST90001784	rs62626323	CD25 on IgD+ CD3	10	6115678
ebi-a-GCST90001784	rs147487472	CD25 on IgD+ CD3	10	6191376
ebi-a-GCST90001784	rs144941792	CD25 on IgD+ CD3	10	5777776
ebi-a-GCST90001784	rs59757547	CD25 on IgD+ CD3	11	123262785

ebi-a-GCST90001784	rs76687530	CD25 on IgD+ CD3	11	123711887
ebi-a-GCST90001784	rs11051398	CD25 on IgD+ CD3	12	31504316
ebi-a-GCST90001784	rs11615354	CD25 on IgD+ CD3	12	2130438
ebi-a-GCST90001784	rs709589	CD25 on IgD+ CD3	14	106306841
ebi-a-GCST90001784	rs8028696	CD25 on IgD+ CD3	15	33466839
ebi-a-GCST90001784	rs117156856	CD25 on IgD+ CD3	16	46798678
ebi-a-GCST90001784	rs62206862	CD25 on IgD+ CD3	20	61967801
ebi-a-GCST90001784	rs34351616	CD25 on IgD+ CD3	21	20240985
ebi-a-GCST90001784	rs146720368	CD25 on IgD+ CD3	22	32003463
ebi-a-GCST90001784	rs117382470	CD25 on IgD+ CD3	22	30950549
ebi-a-GCST90001785	rs17327745	CD25 on IgD- CD2	1	95917846
ebi-a-GCST90001785	rs35830739	CD25 on IgD- CD2	1	229725893
ebi-a-GCST90001785	rs72809405	CD25 on IgD- CD2	2	61173382
ebi-a-GCST90001785	rs4073186	CD25 on IgD- CD2	2	30043619
ebi-a-GCST90001785	rs72973530	CD25 on IgD- CD2	2	235335429
ebi-a-GCST90001785	rs11688172	CD25 on IgD- CD2	2	150793155
ebi-a-GCST90001785	rs75007695	CD25 on IgD- CD2	2	241875782
ebi-a-GCST90001785	rs73224863	CD25 on IgD- CD2	4	29267406
ebi-a-GCST90001785	rs6940205	CD25 on IgD- CD2	6	107141246
ebi-a-GCST90001785	rs12529059	CD25 on IgD- CD2	6	64695023
ebi-a-GCST90001785	rs2290263	CD25 on IgD- CD2	7	25887278
ebi-a-GCST90001785	rs7863711	CD25 on IgD- CD2	9	93616303
ebi-a-GCST90001785	rs11245200	CD25 on IgD- CD2	10	126182752
ebi-a-GCST90001785	rs41294937	CD25 on IgD- CD2	10	6106621
ebi-a-GCST90001785	rs146277285	CD25 on IgD- CD2	10	107243367
ebi-a-GCST90001785	rs147487472	CD25 on IgD- CD2	10	6191376
ebi-a-GCST90001785	rs2732439	CD25 on IgD- CD2	12	48744199
ebi-a-GCST90001785	rs9573934	CD25 on IgD- CD2	13	77338074
ebi-a-GCST90001785	rs1370277	CD25 on IgD- CD2	15	80445815
ebi-a-GCST90001785	rs111500944	CD25 on IgD- CD2	15	80676525
ebi-a-GCST90001785	rs11636705	CD25 on IgD- CD2	15	96605912
ebi-a-GCST90001785	rs4787827	CD25 on IgD- CD2	16	26306929
ebi-a-GCST90001785	rs11548656	CD25 on IgD- CD2	16	81916912
ebi-a-GCST90001785	rs9892531	CD25 on IgD- CD2	17	72336957
ebi-a-GCST90001785	rs4099100	CD25 on IgD- CD2	18	24488604
ebi-a-GCST90001785	rs350822	CD25 on IgD- CD2	19	4083560
ebi-a-GCST90001786	rs35830739	CD25 on IgD- CD2	1	229725893
ebi-a-GCST90001786	rs13005268	CD25 on IgD- CD2	2	49699762
ebi-a-GCST90001786	rs72807491	CD25 on IgD- CD2	2	61154510
ebi-a-GCST90001786	rs1397934	CD25 on IgD- CD2	4	40187596
ebi-a-GCST90001786	rs35979946	CD25 on IgD- CD2	4	102720551
ebi-a-GCST90001786	rs10806538	CD25 on IgD- CD2	6	67249077
ebi-a-GCST90001786	rs181382386	CD25 on IgD- CD2	7	49690154
ebi-a-GCST90001786	rs885063	CD25 on IgD- CD2	9	133304189
ebi-a-GCST90001786	rs111419261	CD25 on IgD- CD2	9	91520493
ebi-a-GCST90001786	rs11245200	CD25 on IgD- CD2	10	126182752
ebi-a-GCST90001786	rs41295093	CD25 on IgD- CD2	10	6120173
ebi-a-GCST90001786	rs4747882	CD25 on IgD- CD2	10	6162476

ebi-a-GCST90001786	rs3886263	CD25 on IgD- CD2	12	130450765
ebi-a-GCST90001786	rs1570675	CD25 on IgD- CD2	13	43406556
ebi-a-GCST90001786	rs117404788	CD25 on IgD- CD2	14	51310988
ebi-a-GCST90001786	rs11548656	CD25 on IgD- CD2	16	81916912
ebi-a-GCST90001786	rs141670644	CD25 on IgD- CD2	17	35618525
ebi-a-GCST90001786	rs116540618	CD25 on IgD- CD2	19	49487555
ebi-a-GCST90001786	rs10401190	CD25 on IgD- CD2	19	17987864
ebi-a-GCST90001786	rs1883832	CD25 on IgD- CD2	20	44746982
ebi-a-GCST90001786	rs6034999	CD25 on IgD- CD2	20	18208277
ebi-a-GCST90001786	rs13049974	CD25 on IgD- CD2	21	32810944
ebi-a-GCST90001787	rs883170	CD25 on IgD- CD3	2	152830901
ebi-a-GCST90001787	rs11694298	CD25 on IgD- CD3	2	11175178
ebi-a-GCST90001787	rs74911560	CD25 on IgD- CD3	2	14883839
ebi-a-GCST90001787	rs4073186	CD25 on IgD- CD3	2	30043619
ebi-a-GCST90001787	rs115945675	CD25 on IgD- CD3	3	127810569
ebi-a-GCST90001787	rs2180045	CD25 on IgD- CD3	6	164317117
ebi-a-GCST90001787	rs78038432	CD25 on IgD- CD3	6	113488026
ebi-a-GCST90001787	rs117534066	CD25 on IgD- CD3	8	139664271
ebi-a-GCST90001787	rs12342539	CD25 on IgD- CD3	9	4327960
ebi-a-GCST90001787	rs11245200	CD25 on IgD- CD3	10	126182752
ebi-a-GCST90001787	rs12722531	CD25 on IgD- CD3	10	6076076
ebi-a-GCST90001787	rs706779	CD25 on IgD- CD3	10	6098824
ebi-a-GCST90001787	rs187593378	CD25 on IgD- CD3	11	67809980
ebi-a-GCST90001787	rs11036291	CD25 on IgD- CD3	11	41231092
ebi-a-GCST90001787	rs77750506	CD25 on IgD- CD3	11	115771425
ebi-a-GCST90001787	rs1570675	CD25 on IgD- CD3	13	43406556
ebi-a-GCST90001787	rs16957211	CD25 on IgD- CD3	15	46897868
ebi-a-GCST90001787	rs73374860	CD25 on IgD- CD3	18	4513432
ebi-a-GCST90001787	rs80243585	CD25 on IgD- CD3	18	28337909
ebi-a-GCST90001787	rs10401190	CD25 on IgD- CD3	19	17987864
ebi-a-GCST90001787	rs144182927	CD25 on IgD- CD3	21	23113704
ebi-a-GCST90001787	rs73173391	CD25 on IgD- CD3	22	46164098
ebi-a-GCST90001788	rs3958466	CD25 on IgD- CD3	1	47241376
ebi-a-GCST90001788	rs284289	CD25 on IgD- CD3	1	10763815
ebi-a-GCST90001788	rs11124936	CD25 on IgD- CD3	2	43653000
ebi-a-GCST90001788	rs1553309	CD25 on IgD- CD3	3	121910706
ebi-a-GCST90001788	rs140397359	CD25 on IgD- CD3	3	8278605
ebi-a-GCST90001788	rs7692931	CD25 on IgD- CD3	4	118326937
ebi-a-GCST90001788	rs80056168	CD25 on IgD- CD3	4	89684474
ebi-a-GCST90001788	rs62485164	CD25 on IgD- CD3	7	136341447
ebi-a-GCST90001788	rs79744969	CD25 on IgD- CD3	8	68654406
ebi-a-GCST90001788	rs149114716	CD25 on IgD- CD3	8	3816956
ebi-a-GCST90001788	rs10756960	CD25 on IgD- CD3	9	18383606
ebi-a-GCST90001788	rs78997789	CD25 on IgD- CD3	9	76715113
ebi-a-GCST90001788	rs4509665	CD25 on IgD- CD3	10	4941911
ebi-a-GCST90001788	rs407948	CD25 on IgD- CD3	12	130766040
ebi-a-GCST90001788	rs1982142	CD25 on IgD- CD3	13	102272356
ebi-a-GCST90001788	rs75264624	CD25 on IgD- CD3	14	80644758

ebi-a-GCST90001788	rs61986645	CD25 on IgD- CD3	14	86928722
ebi-a-GCST90001788	rs7166343	CD25 on IgD- CD3	15	29885297
ebi-a-GCST90001788	rs9897177	CD25 on IgD- CD3	17	3784367
ebi-a-GCST90001788	rs6035444	CD25 on IgD- CD3	20	19792012
ebi-a-GCST90001788	rs73168598	CD25 on IgD- CD3	22	34045501
ebi-a-GCST90001789	rs140813228	CD25 on IgD- CD3	1	26723064
ebi-a-GCST90001789	rs191775575	CD25 on IgD- CD3	2	119062858
ebi-a-GCST90001789	rs1816221	CD25 on IgD- CD3	2	71977094
ebi-a-GCST90001789	rs112937432	CD25 on IgD- CD3	2	72182946
ebi-a-GCST90001789	rs1397934	CD25 on IgD- CD3	4	40187596
ebi-a-GCST90001789	rs6866974	CD25 on IgD- CD3	5	178081193
ebi-a-GCST90001789	rs9372361	CD25 on IgD- CD3	6	113707049
ebi-a-GCST90001789	rs2999572	CD25 on IgD- CD3	7	25590299
ebi-a-GCST90001789	rs142063706	CD25 on IgD- CD3	8	141253242
ebi-a-GCST90001789	rs3793662	CD25 on IgD- CD3	9	95886051
ebi-a-GCST90001789	rs61839442	CD25 on IgD- CD3	10	6321360
ebi-a-GCST90001789	rs41294937	CD25 on IgD- CD3	10	6106621
ebi-a-GCST90001789	rs7078614	CD25 on IgD- CD3	10	6075831
ebi-a-GCST90001789	rs188753875	CD25 on IgD- CD3	10	24537541
ebi-a-GCST90001789	rs11036291	CD25 on IgD- CD3	11	41231092
ebi-a-GCST90001789	rs138920863	CD25 on IgD- CD3	13	85642896
ebi-a-GCST90001789	rs72772213	CD25 on IgD- CD3	16	9444808
ebi-a-GCST90001789	rs8066711	CD25 on IgD- CD3	17	73885038
ebi-a-GCST90001789	rs1795955	CD25 on IgD- CD3	17	77129985
ebi-a-GCST90001789	rs526811	CD25 on IgD- CD3	18	2090450
ebi-a-GCST90001789	rs536374	CD25 on IgD- CD3	18	34917959
ebi-a-GCST90001789	rs61745051	CD25 on IgD- CD3	20	33584197
ebi-a-GCST90001789	rs75727877	CD25 on IgD- CD3	20	50852063
ebi-a-GCST90001790	rs12139506	CD25 on memory E	1	61376720
ebi-a-GCST90001790	rs1660369	CD25 on memory E	1	214407674
ebi-a-GCST90001790	rs141644939	CD25 on memory E	2	149979124
ebi-a-GCST90001790	rs2139146	CD25 on memory E	2	15014620
ebi-a-GCST90001790	rs138409352	CD25 on memory E	3	10050183
ebi-a-GCST90001790	rs149522679	CD25 on memory E	4	142001552
ebi-a-GCST90001790	rs17252295	CD25 on memory E	5	8141537
ebi-a-GCST90001790	rs149662107	CD25 on memory E	7	47871972
ebi-a-GCST90001790	rs2999572	CD25 on memory E	7	25590299
ebi-a-GCST90001790	rs3793662	CD25 on memory E	9	95886051
ebi-a-GCST90001790	rs2853765	CD25 on memory E	10	7830675
ebi-a-GCST90001790	rs11245200	CD25 on memory E	10	126182752
ebi-a-GCST90001790	rs1887027	CD25 on memory E	10	6113782
ebi-a-GCST90001790	rs4747882	CD25 on memory E	10	6162476
ebi-a-GCST90001790	rs11598494	CD25 on memory E	10	6178941
ebi-a-GCST90001790	rs12886102	CD25 on memory E	14	100108322
ebi-a-GCST90001790	rs183975337	CD25 on memory E	14	89659141
ebi-a-GCST90001790	rs11632374	CD25 on memory E	15	33478617
ebi-a-GCST90001790	rs62013832	CD25 on memory E	15	86804118
ebi-a-GCST90001790	rs9972778	CD25 on memory E	16	25516463

ebi-a-GCST90001790	rs111912558	CD25 on memory E	17	44356694
ebi-a-GCST90001790	rs8097813	CD25 on memory E	18	67173624
ebi-a-GCST90001790	rs8100828	CD25 on memory E	19	11184104
ebi-a-GCST90001790	rs75727877	CD25 on memory E	20	50852063
ebi-a-GCST90001790	rs144182927	CD25 on memory E	21	23113704
ebi-a-GCST90001790	rs3862036	CD25 on memory E	21	44262209
ebi-a-GCST90001790	rs200381543	CD25 on memory E	22	22547864
ebi-a-GCST90001791	rs139257812	CD25 on naive-mat	1	8194962
ebi-a-GCST90001791	rs9425892	CD25 on naive-mat	1	34421513
ebi-a-GCST90001791	rs13019590	CD25 on naive-mat	2	61099420
ebi-a-GCST90001791	rs112017455	CD25 on naive-mat	3	157651153
ebi-a-GCST90001791	rs1398243	CD25 on naive-mat	4	145514461
ebi-a-GCST90001791	rs34518596	CD25 on naive-mat	5	86111282
ebi-a-GCST90001791	rs4869953	CD25 on naive-mat	6	151209997
ebi-a-GCST90001791	rs149662107	CD25 on naive-mat	7	47871972
ebi-a-GCST90001791	rs1812673	CD25 on naive-mat	7	25865542
ebi-a-GCST90001791	rs3793662	CD25 on naive-mat	9	95886051
ebi-a-GCST90001791	rs7028305	CD25 on naive-mat	9	654681
ebi-a-GCST90001791	rs17403348	CD25 on naive-mat	10	5827901
ebi-a-GCST90001791	rs147487472	CD25 on naive-mat	10	6191376
ebi-a-GCST90001791	rs41294937	CD25 on naive-mat	10	6106621
ebi-a-GCST90001791	rs116930842	CD25 on naive-mat	10	99652174
ebi-a-GCST90001791	rs59278703	CD25 on naive-mat	11	35069685
ebi-a-GCST90001791	rs77869769	CD25 on naive-mat	11	70397340
ebi-a-GCST90001791	rs474490	CD25 on naive-mat	11	117287794
ebi-a-GCST90001791	rs73070033	CD25 on naive-mat	12	17419208
ebi-a-GCST90001791	rs11051398	CD25 on naive-mat	12	31504316
ebi-a-GCST90001791	rs117788286	CD25 on naive-mat	13	19370199
ebi-a-GCST90001791	rs709589	CD25 on naive-mat	14	106306841
ebi-a-GCST90001791	rs8028696	CD25 on naive-mat	15	33466839
ebi-a-GCST90001791	rs118088397	CD25 on naive-mat	16	11709659
ebi-a-GCST90001791	rs148048180	CD25 on naive-mat	19	9181620
ebi-a-GCST90001791	rs1555271	CD25 on naive-mat	20	20803779
ebi-a-GCST90001791	rs17002085	CD25 on naive-mat	21	27750139
ebi-a-GCST90001792	rs1660369	CD25 on unsw men	1	214407674
ebi-a-GCST90001792	rs12739411	CD25 on unsw men	1	68752573
ebi-a-GCST90001792	rs12139506	CD25 on unsw men	1	61376720
ebi-a-GCST90001792	rs75784903	CD25 on unsw men	2	173874264
ebi-a-GCST90001792	rs2139146	CD25 on unsw men	2	15014620
ebi-a-GCST90001792	rs62149023	CD25 on unsw men	2	33764503
ebi-a-GCST90001792	rs149905818	CD25 on unsw men	4	106231334
ebi-a-GCST90001792	rs74726196	CD25 on unsw men	5	122019220
ebi-a-GCST90001792	rs2999572	CD25 on unsw men	7	25590299
ebi-a-GCST90001792	rs140833732	CD25 on unsw men	8	36436637
ebi-a-GCST90001792	rs183392197	CD25 on unsw men	9	132487598
ebi-a-GCST90001792	rs3793662	CD25 on unsw men	9	95886051
ebi-a-GCST90001792	rs4747882	CD25 on unsw men	10	6162476
ebi-a-GCST90001792	rs11245200	CD25 on unsw men	10	126182752

ebi-a-GCST90001792	rs1887027	CD25 on unsw men	10	6113782
ebi-a-GCST90001792	rs11598494	CD25 on unsw men	10	6178941
ebi-a-GCST90001792	rs2853765	CD25 on unsw men	10	7830675
ebi-a-GCST90001792	rs563803689	CD25 on unsw men	11	102922239
ebi-a-GCST90001792	rs183975337	CD25 on unsw men	14	89659141
ebi-a-GCST90001792	rs62013832	CD25 on unsw men	15	86804118
ebi-a-GCST90001792	rs7186545	CD25 on unsw men	16	14508423
ebi-a-GCST90001792	rs28374524	CD25 on unsw men	16	25533172
ebi-a-GCST90001792	rs73332250	CD25 on unsw men	17	47014571
ebi-a-GCST90001792	rs28365327	CD25 on unsw men	18	25753552
ebi-a-GCST90001792	rs8097813	CD25 on unsw men	18	67173624
ebi-a-GCST90001792	rs3862036	CD25 on unsw men	21	44262209
ebi-a-GCST90001793	rs4073186	CD25 on sw mem	2	30043619
ebi-a-GCST90001793	rs75038650	CD25 on sw mem	2	5553075
ebi-a-GCST90001793	rs77603125	CD25 on sw mem	5	179275092
ebi-a-GCST90001793	rs9372361	CD25 on sw mem	6	113707049
ebi-a-GCST90001793	rs2999572	CD25 on sw mem	7	25590299
ebi-a-GCST90001793	rs80287371	CD25 on sw mem	8	5413535
ebi-a-GCST90001793	rs3793662	CD25 on sw mem	9	95886051
ebi-a-GCST90001793	rs12342539	CD25 on sw mem	9	4327960
ebi-a-GCST90001793	rs609291	CD25 on sw mem	9	127809664
ebi-a-GCST90001793	rs41294937	CD25 on sw mem	10	6106621
ebi-a-GCST90001793	rs7078614	CD25 on sw mem	10	6075831
ebi-a-GCST90001793	rs61839442	CD25 on sw mem	10	6321360
ebi-a-GCST90001793	rs217211	CD25 on sw mem	11	1975919
ebi-a-GCST90001793	rs12830810	CD25 on sw mem	12	47930410
ebi-a-GCST90001793	rs12886102	CD25 on sw mem	14	100108322
ebi-a-GCST90001793	rs12907742	CD25 on sw mem	15	101525737
ebi-a-GCST90001793	rs61745051	CD25 on sw mem	20	33584197
ebi-a-GCST90001793	rs2252677	CD25 on sw mem	20	18206348
ebi-a-GCST90001793	rs60525628	CD25 on sw mem	21	47558586
ebi-a-GCST90001793	rs144182927	CD25 on sw mem	21	23113704
ebi-a-GCST90001793	rs200381543	CD25 on sw mem	22	22547864
ebi-a-GCST90001794	rs9425892	CD25 on IgD+	1	34421513
ebi-a-GCST90001794	rs540760708	CD25 on IgD+	1	7474014
ebi-a-GCST90001794	rs111741355	CD25 on IgD+	1	48095312
ebi-a-GCST90001794	rs72944931	CD25 on IgD+	2	178116262
ebi-a-GCST90001794	rs12465275	CD25 on IgD+	2	4082267
ebi-a-GCST90001794	rs62175769	CD25 on IgD+	2	173865326
ebi-a-GCST90001794	rs112043339	CD25 on IgD+	3	174935606
ebi-a-GCST90001794	rs12638268	CD25 on IgD+	3	11933438
ebi-a-GCST90001794	rs114737782	CD25 on IgD+	4	177026245
ebi-a-GCST90001794	rs113492859	CD25 on IgD+	4	185217641
ebi-a-GCST90001794	rs4869953	CD25 on IgD+	6	151209997
ebi-a-GCST90001794	rs149662107	CD25 on IgD+	7	47871972
ebi-a-GCST90001794	rs3793662	CD25 on IgD+	9	95886051
ebi-a-GCST90001794	rs71501657	CD25 on IgD+	9	113797266
ebi-a-GCST90001794	rs147487472	CD25 on IgD+	10	6191376

ebi-a-GCST90001794	rs41294937	CD25 on IgD+	10	6106621
ebi-a-GCST90001794	rs11615354	CD25 on IgD+	12	2130438
ebi-a-GCST90001794	rs11051398	CD25 on IgD+	12	31504316
ebi-a-GCST90001794	rs557577736	CD25 on IgD+	13	46224092
ebi-a-GCST90001794	rs2248769	CD25 on IgD+	13	54938910
ebi-a-GCST90001794	rs112328981	CD25 on IgD+	13	34477079
ebi-a-GCST90001794	rs2753488	CD25 on IgD+	14	106305260
ebi-a-GCST90001794	rs11632374	CD25 on IgD+	15	33478617
ebi-a-GCST90001794	rs117156856	CD25 on IgD+	16	46798678
ebi-a-GCST90001794	rs62206862	CD25 on IgD+	20	61967801
ebi-a-GCST90001794	rs75727877	CD25 on IgD+	20	50852063
ebi-a-GCST90001794	rs13051777	CD25 on IgD+	21	20241615
ebi-a-GCST90001794	rs146720368	CD25 on IgD+	22	32003463
ebi-a-GCST90001795	rs6550133	CD25 on transitiona	3	32472280
ebi-a-GCST90001795	rs11099673	CD25 on transitiona	4	148899082
ebi-a-GCST90001795	rs181825	CD25 on transitiona	5	80131532
ebi-a-GCST90001795	rs4721574	CD25 on transitiona	7	2350451
ebi-a-GCST90001795	rs3793662	CD25 on transitiona	9	95886051
ebi-a-GCST90001795	rs4129608	CD25 on transitiona	9	18327215
ebi-a-GCST90001795	rs117473115	CD25 on transitiona	10	54503355
ebi-a-GCST90001795	rs62626323	CD25 on transitiona	10	6115678
ebi-a-GCST90001795	rs7899857	CD25 on transitiona	10	133280976
ebi-a-GCST90001795	rs34513850	CD25 on transitiona	11	113437313
ebi-a-GCST90001795	rs474490	CD25 on transitiona	11	117287794
ebi-a-GCST90001795	rs76153918	CD25 on transitiona	11	70398780
ebi-a-GCST90001795	rs12822478	CD25 on transitiona	12	31514566
ebi-a-GCST90001795	rs74709342	CD25 on transitiona	13	107000655
ebi-a-GCST90001795	rs148702440	CD25 on transitiona	13	22419220
ebi-a-GCST90001795	rs61945276	CD25 on transitiona	13	22645864
ebi-a-GCST90001795	rs876043	CD25 on transitiona	14	92357145
ebi-a-GCST90001795	rs10047878	CD25 on transitiona	14	22787690
ebi-a-GCST90001795	rs709589	CD25 on transitiona	14	106306841
ebi-a-GCST90001795	rs151006498	CD25 on transitiona	15	63642578
ebi-a-GCST90001795	rs144997167	CD25 on transitiona	16	46608478
ebi-a-GCST90001795	rs140685335	CD25 on transitiona	18	1584719
ebi-a-GCST90001795	rs78716865	CD25 on transitiona	19	52158206
ebi-a-GCST90001796	rs6658353	CD27 on CD20-	1	161469054
ebi-a-GCST90001796	rs116588562	CD27 on CD20-	1	201089372
ebi-a-GCST90001796	rs10914353	CD27 on CD20-	1	30235009
ebi-a-GCST90001796	rs66732006	CD27 on CD20-	2	102157635
ebi-a-GCST90001796	rs4446062	CD27 on CD20-	2	57498544
ebi-a-GCST90001796	rs148362575	CD27 on CD20-	2	187165335
ebi-a-GCST90001796	rs10175058	CD27 on CD20-	2	162276981
ebi-a-GCST90001796	rs2935486	CD27 on CD20-	5	89576140
ebi-a-GCST90001796	rs7015229	CD27 on CD20-	8	124077305
ebi-a-GCST90001796	rs4752656	CD27 on CD20-	10	123899507
ebi-a-GCST90001796	rs878511	CD27 on CD20-	11	43543339
ebi-a-GCST90001796	rs117208568	CD27 on CD20-	15	54126596

ebi-a-GCST90001796	rs80322600	CD27 on CD20-	16	55456633
ebi-a-GCST90001796	rs8064682	CD27 on CD20-	17	75246854
ebi-a-GCST90001796	rs2939611	CD27 on CD20-	18	46871075
ebi-a-GCST90001796	rs74330899	CD27 on CD20-	19	29552282
ebi-a-GCST90001796	rs2910346	CD27 on CD20-	19	8929031
ebi-a-GCST90001797	rs909973	CD27 on CD20- CI	1	40901979
ebi-a-GCST90001797	rs74432706	CD27 on CD20- CI	2	197887417
ebi-a-GCST90001797	rs113532966	CD27 on CD20- CI	2	50627626
ebi-a-GCST90001797	rs61566441	CD27 on CD20- CI	3	155044125
ebi-a-GCST90001797	rs115412883	CD27 on CD20- CI	3	26147574
ebi-a-GCST90001797	rs10042952	CD27 on CD20- CI	5	67196963
ebi-a-GCST90001797	rs147680281	CD27 on CD20- CI	5	127113029
ebi-a-GCST90001797	rs74551235	CD27 on CD20- CI	6	150575442
ebi-a-GCST90001797	rs118135815	CD27 on CD20- CI	7	146514190
ebi-a-GCST90001797	rs7007180	CD27 on CD20- CI	8	6947493
ebi-a-GCST90001797	rs147153707	CD27 on CD20- CI	9	31165407
ebi-a-GCST90001797	rs10763431	CD27 on CD20- CI	10	58623676
ebi-a-GCST90001797	rs10740344	CD27 on CD20- CI	10	72155840
ebi-a-GCST90001797	rs7114298	CD27 on CD20- CI	11	65582028
ebi-a-GCST90001797	rs4578504	CD27 on CD20- CI	13	27510490
ebi-a-GCST90001797	rs8072367	CD27 on CD20- CI	17	75901729
ebi-a-GCST90001797	rs9914679	CD27 on CD20- CI	17	13675196
ebi-a-GCST90001797	rs1785249	CD27 on CD20- CI	18	3362511
ebi-a-GCST90001797	rs2024568	CD27 on CD20- CI	20	44732089
ebi-a-GCST90001797	rs139998	CD27 on CD20- CI	22	36862501
ebi-a-GCST90001798	rs1801274	CD27 on CD24+ C	1	161479745
ebi-a-GCST90001798	rs34101571	CD27 on CD24+ C	1	31211894
ebi-a-GCST90001798	rs113382987	CD27 on CD24+ C	2	4112823
ebi-a-GCST90001798	rs76929865	CD27 on CD24+ C	3	31134534
ebi-a-GCST90001798	rs142111770	CD27 on CD24+ C	3	34933016
ebi-a-GCST90001798	rs114401515	CD27 on CD24+ C	3	46865159
ebi-a-GCST90001798	rs144642376	CD27 on CD24+ C	3	36349691
ebi-a-GCST90001798	rs116522219	CD27 on CD24+ C	3	36625286
ebi-a-GCST90001798	rs7621492	CD27 on CD24+ C	3	32302456
ebi-a-GCST90001798	rs75918255	CD27 on CD24+ C	3	32945761
ebi-a-GCST90001798	rs9823348	CD27 on CD24+ C	3	400357
ebi-a-GCST90001798	rs35574803	CD27 on CD24+ C	3	32533246
ebi-a-GCST90001798	rs78083636	CD27 on CD24+ C	4	153842791
ebi-a-GCST90001798	rs4700827	CD27 on CD24+ C	5	178897725
ebi-a-GCST90001798	rs148536836	CD27 on CD24+ C	5	131696266
ebi-a-GCST90001798	rs79871977	CD27 on CD24+ C	5	11538463
ebi-a-GCST90001798	rs245668	CD27 on CD24+ C	5	36713399
ebi-a-GCST90001798	rs12664485	CD27 on CD24+ C	6	143890569
ebi-a-GCST90001798	rs141876897	CD27 on CD24+ C	9	94241794
ebi-a-GCST90001798	rs143164248	CD27 on CD24+ C	10	28945318
ebi-a-GCST90001798	rs230665	CD27 on CD24+ C	11	77881098
ebi-a-GCST90001798	rs25680	CD27 on CD24+ C	12	6554628
ebi-a-GCST90001798	rs147714617	CD27 on CD24+ C	12	6605299

ebi-a-GCST90001798	rs185264204	CD27 on CD24+ C	18	74212885
ebi-a-GCST90001798	rs162076	CD27 on CD24+ C	20	46785499
ebi-a-GCST90001798	rs8116311	CD27 on CD24+ C	20	44836231
ebi-a-GCST90001798	rs11086984	CD27 on CD24+ C	20	44511627
ebi-a-GCST90001798	rs4810496	CD27 on CD24+ C	20	44954283
ebi-a-GCST90001798	rs1883832	CD27 on CD24+ C	20	44746982
ebi-a-GCST90001798	rs72645230	CD27 on CD24+ C	20	46242688
ebi-a-GCST90001798	rs9989924	CD27 on CD24+ C	20	42019380
ebi-a-GCST90001799	rs79633764	CD27 on T cell	1	229986965
ebi-a-GCST90001799	rs1801274	CD27 on T cell	1	161479745
ebi-a-GCST90001799	rs180734121	CD27 on T cell	2	31487659
ebi-a-GCST90001799	rs35574803	CD27 on T cell	3	32533246
ebi-a-GCST90001799	rs17272388	CD27 on T cell	4	94346696
ebi-a-GCST90001799	rs148064748	CD27 on T cell	4	98085948
ebi-a-GCST90001799	rs73252247	CD27 on T cell	4	21549438
ebi-a-GCST90001799	rs62396405	CD27 on T cell	6	38807887
ebi-a-GCST90001799	rs78033918	CD27 on T cell	7	139037580
ebi-a-GCST90001799	rs61590681	CD27 on T cell	8	3733148
ebi-a-GCST90001799	rs111986434	CD27 on T cell	9	132142740
ebi-a-GCST90001799	rs7893773	CD27 on T cell	10	52916173
ebi-a-GCST90001799	rs10996192	CD27 on T cell	10	66846516
ebi-a-GCST90001799	rs4937631	CD27 on T cell	11	131356267
ebi-a-GCST90001799	rs184776859	CD27 on T cell	12	124564198
ebi-a-GCST90001799	rs10785556	CD27 on T cell	12	45593682
ebi-a-GCST90001799	rs9520817	CD27 on T cell	13	108846787
ebi-a-GCST90001799	rs75211095	CD27 on T cell	13	67666819
ebi-a-GCST90001799	rs35116758	CD27 on T cell	16	56916197
ebi-a-GCST90001799	rs2848587	CD27 on T cell	18	33793953
ebi-a-GCST90001799	rs11698768	CD27 on T cell	20	58825491
ebi-a-GCST90001800	rs866069	CD27 on IgD+ CD2	1	157006976
ebi-a-GCST90001800	rs481818	CD27 on IgD+ CD2	1	234426306
ebi-a-GCST90001800	rs2783440	CD27 on IgD+ CD2	1	111748488
ebi-a-GCST90001800	rs76385721	CD27 on IgD+ CD2	2	111411515
ebi-a-GCST90001800	rs7621492	CD27 on IgD+ CD2	3	32302456
ebi-a-GCST90001800	rs116551266	CD27 on IgD+ CD2	3	36350049
ebi-a-GCST90001800	rs12637802	CD27 on IgD+ CD2	3	409221
ebi-a-GCST90001800	rs75918255	CD27 on IgD+ CD2	3	32945761
ebi-a-GCST90001800	rs35574803	CD27 on IgD+ CD2	3	32533246
ebi-a-GCST90001800	rs76929865	CD27 on IgD+ CD2	3	31134534
ebi-a-GCST90001800	rs111825302	CD27 on IgD+ CD2	4	100692569
ebi-a-GCST90001800	rs73236188	CD27 on IgD+ CD2	4	56539558
ebi-a-GCST90001800	rs6881246	CD27 on IgD+ CD2	5	38617277
ebi-a-GCST90001800	rs73716743	CD27 on IgD+ CD2	6	1056091
ebi-a-GCST90001800	rs1100578	CD27 on IgD+ CD2	6	22371990
ebi-a-GCST90001800	rs11769145	CD27 on IgD+ CD2	7	70704068
ebi-a-GCST90001800	rs12541523	CD27 on IgD+ CD2	8	124577287
ebi-a-GCST90001800	rs951067	CD27 on IgD+ CD2	10	128856644
ebi-a-GCST90001800	rs10833056	CD27 on IgD+ CD2	11	19105332

ebi-a-GCST90001800	rs147714617	CD27 on IgD+ CD2	12	6605299
ebi-a-GCST90001800	rs143553941	CD27 on IgD+ CD2	12	109368119
ebi-a-GCST90001800	rs25680	CD27 on IgD+ CD2	12	6554628
ebi-a-GCST90001800	rs709589	CD27 on IgD+ CD2	14	106306841
ebi-a-GCST90001800	rs150399795	CD27 on IgD+ CD2	16	59131195
ebi-a-GCST90001800	rs75005829	CD27 on IgD+ CD2	17	53317228
ebi-a-GCST90001800	rs1883832	CD27 on IgD+ CD2	20	44746982
ebi-a-GCST90001800	rs12625834	CD27 on IgD+ CD2	20	44949039
ebi-a-GCST90001800	rs145656540	CD27 on IgD+ CD2	20	57966338
ebi-a-GCST90001800	rs235031	CD27 on IgD+ CD2	20	48248326
ebi-a-GCST90001800	rs11086984	CD27 on IgD+ CD2	20	44511627
ebi-a-GCST90001800	rs162076	CD27 on IgD+ CD2	20	46785499
ebi-a-GCST90001800	rs8139510	CD27 on IgD+ CD2	22	21448500
ebi-a-GCST90001801	rs138487400	CD27 on IgD+ CD3	1	65088698
ebi-a-GCST90001801	rs115870915	CD27 on IgD+ CD3	1	114134030
ebi-a-GCST90001801	rs62161465	CD27 on IgD+ CD3	2	107721414
ebi-a-GCST90001801	rs11718356	CD27 on IgD+ CD3	3	30308520
ebi-a-GCST90001801	rs56237381	CD27 on IgD+ CD3	3	32924804
ebi-a-GCST90001801	rs76929865	CD27 on IgD+ CD3	3	31134534
ebi-a-GCST90001801	rs35574803	CD27 on IgD+ CD3	3	32533246
ebi-a-GCST90001801	rs75918255	CD27 on IgD+ CD3	3	32945761
ebi-a-GCST90001801	rs76894736	CD27 on IgD+ CD3	5	36745459
ebi-a-GCST90001801	rs17097461	CD27 on IgD+ CD3	5	141077992
ebi-a-GCST90001801	rs11746129	CD27 on IgD+ CD3	5	83088178
ebi-a-GCST90001801	rs10733309	CD27 on IgD+ CD3	9	16253322
ebi-a-GCST90001801	rs11220465	CD27 on IgD+ CD3	11	126257779
ebi-a-GCST90001801	rs25680	CD27 on IgD+ CD3	12	6554628
ebi-a-GCST90001801	rs11829119	CD27 on IgD+ CD3	12	19193531
ebi-a-GCST90001801	rs61927746	CD27 on IgD+ CD3	12	47671332
ebi-a-GCST90001801	rs140684212	CD27 on IgD+ CD3	13	19367953
ebi-a-GCST90001801	rs148107267	CD27 on IgD+ CD3	15	33896396
ebi-a-GCST90001801	rs72794809	CD27 on IgD+ CD3	16	33377221
ebi-a-GCST90001801	rs13336159	CD27 on IgD+ CD3	16	8832515
ebi-a-GCST90001801	rs72645230	CD27 on IgD+ CD3	20	46242688
ebi-a-GCST90001801	rs4810485	CD27 on IgD+ CD3	20	44747947
ebi-a-GCST90001801	rs73301560	CD27 on IgD+ CD3	20	45088788
ebi-a-GCST90001801	rs6066198	CD27 on IgD+ CD3	20	45732275
ebi-a-GCST90001801	rs4815533	CD27 on IgD+ CD3	20	2684924
ebi-a-GCST90001802	rs1801274	CD27 on IgD- CD3	1	161479745
ebi-a-GCST90001802	rs114911864	CD27 on IgD- CD3	1	16508223
ebi-a-GCST90001802	rs34101571	CD27 on IgD- CD3	1	31211894
ebi-a-GCST90001802	rs1988454	CD27 on IgD- CD3	1	82973317
ebi-a-GCST90001802	rs7517211	CD27 on IgD- CD3	1	99969159
ebi-a-GCST90001802	rs114674204	CD27 on IgD- CD3	2	207963323
ebi-a-GCST90001802	rs62163304	CD27 on IgD- CD3	2	111847100
ebi-a-GCST90001802	rs77986179	CD27 on IgD- CD3	2	8336242
ebi-a-GCST90001802	rs35574803	CD27 on IgD- CD3	3	32533246
ebi-a-GCST90001802	rs4453793	CD27 on IgD- CD3	3	122718575

ebi-a-GCST90001802	rs2526388	CD27 on IgD- CD3	3	50174886
ebi-a-GCST90001802	rs75918255	CD27 on IgD- CD3	3	32945761
ebi-a-GCST90001802	rs76929865	CD27 on IgD- CD3	3	31134534
ebi-a-GCST90001802	rs144642376	CD27 on IgD- CD3	3	36349691
ebi-a-GCST90001802	rs75901184	CD27 on IgD- CD3	4	65101031
ebi-a-GCST90001802	rs36111462	CD27 on IgD- CD3	5	109948893
ebi-a-GCST90001802	rs717954	CD27 on IgD- CD3	5	148981029
ebi-a-GCST90001802	rs486182	CD27 on IgD- CD3	8	20806496
ebi-a-GCST90001802	rs147388542	CD27 on IgD- CD3	9	94318432
ebi-a-GCST90001802	rs230665	CD27 on IgD- CD3	11	77881098
ebi-a-GCST90001802	rs11064198	CD27 on IgD- CD3	12	6558720
ebi-a-GCST90001802	rs577575	CD27 on IgD- CD3	12	10045919
ebi-a-GCST90001802	rs2250246	CD27 on IgD- CD3	12	6553016
ebi-a-GCST90001802	rs4899049	CD27 on IgD- CD3	14	61938155
ebi-a-GCST90001802	rs7179434	CD27 on IgD- CD3	15	22523362
ebi-a-GCST90001802	rs6508496	CD27 on IgD- CD3	18	25250464
ebi-a-GCST90001802	rs4987790	CD27 on IgD- CD3	18	60861693
ebi-a-GCST90001802	rs150306315	CD27 on IgD- CD3	19	16824730
ebi-a-GCST90001802	rs11086984	CD27 on IgD- CD3	20	44511627
ebi-a-GCST90001802	rs973446	CD27 on IgD- CD3	20	44094375
ebi-a-GCST90001802	rs1883832	CD27 on IgD- CD3	20	44746982
ebi-a-GCST90001802	rs162076	CD27 on IgD- CD3	20	46785499
ebi-a-GCST90001802	rs6012007	CD27 on IgD- CD3	20	45312244
ebi-a-GCST90001803	rs10789188	CD27 on IgD- CD3	1	66053791
ebi-a-GCST90001803	rs72770527	CD27 on IgD- CD3	2	16073977
ebi-a-GCST90001803	rs13111916	CD27 on IgD- CD3	4	13780721
ebi-a-GCST90001803	rs4537081	CD27 on IgD- CD3	5	165579358
ebi-a-GCST90001803	rs9386093	CD27 on IgD- CD3	6	145274168
ebi-a-GCST90001803	rs73202627	CD27 on IgD- CD3	8	12580949
ebi-a-GCST90001803	rs12552851	CD27 on IgD- CD3	9	24488925
ebi-a-GCST90001803	rs140193270	CD27 on IgD- CD3	10	4243950
ebi-a-GCST90001803	rs2250246	CD27 on IgD- CD3	12	6553016
ebi-a-GCST90001803	rs11171232	CD27 on IgD- CD3	12	55446669
ebi-a-GCST90001803	rs2669400	CD27 on IgD- CD3	12	54880954
ebi-a-GCST90001803	rs1563304	CD27 on IgD- CD3	17	44874453
ebi-a-GCST90001803	rs112824549	CD27 on IgD- CD3	17	73082360
ebi-a-GCST90001803	rs2587637	CD27 on IgD- CD3	18	39020074
ebi-a-GCST90001803	rs1883832	CD27 on IgD- CD3	20	44746982
ebi-a-GCST90001804	rs12727962	CD27 on IgD- CD3	1	31686211
ebi-a-GCST90001804	rs116327214	CD27 on IgD- CD3	1	32083093
ebi-a-GCST90001804	rs1801274	CD27 on IgD- CD3	1	161479745
ebi-a-GCST90001804	rs34101571	CD27 on IgD- CD3	1	31211894
ebi-a-GCST90001804	rs6667752	CD27 on IgD- CD3	1	177936599
ebi-a-GCST90001804	rs9825547	CD27 on IgD- CD3	3	28883059
ebi-a-GCST90001804	rs182174914	CD27 on IgD- CD3	3	80085882
ebi-a-GCST90001804	rs41533545	CD27 on IgD- CD3	3	30298480
ebi-a-GCST90001804	rs41347750	CD27 on IgD- CD3	3	29246192
ebi-a-GCST90001804	rs2576344	CD27 on IgD- CD3	3	104073058

ebi-a-GCST90001804	rs76929865	CD27 on IgD- CD3	3	31134534
ebi-a-GCST90001804	rs75918255	CD27 on IgD- CD3	3	32945761
ebi-a-GCST90001804	rs112889714	CD27 on IgD- CD3	3	127303399
ebi-a-GCST90001804	rs35574803	CD27 on IgD- CD3	3	32533246
ebi-a-GCST90001804	rs2122701	CD27 on IgD- CD3	4	137067847
ebi-a-GCST90001804	rs112339885	CD27 on IgD- CD3	5	79389016
ebi-a-GCST90001804	rs147388542	CD27 on IgD- CD3	9	94318432
ebi-a-GCST90001804	rs158690	CD27 on IgD- CD3	9	93656985
ebi-a-GCST90001804	rs12380722	CD27 on IgD- CD3	9	6865096
ebi-a-GCST90001804	rs113711447	CD27 on IgD- CD3	9	96510568
ebi-a-GCST90001804	rs73030401	CD27 on IgD- CD3	11	131947318
ebi-a-GCST90001804	rs25680	CD27 on IgD- CD3	12	6554628
ebi-a-GCST90001804	rs1532372	CD27 on IgD- CD3	12	55016563
ebi-a-GCST90001804	rs147714617	CD27 on IgD- CD3	12	6605299
ebi-a-GCST90001804	rs12708463	CD27 on IgD- CD3	15	60628064
ebi-a-GCST90001804	rs888294	CD27 on IgD- CD3	18	25214592
ebi-a-GCST90001804	rs183836759	CD27 on IgD- CD3	19	15323100
ebi-a-GCST90001804	rs11086984	CD27 on IgD- CD3	20	44511627
ebi-a-GCST90001804	rs12625834	CD27 on IgD- CD3	20	44949039
ebi-a-GCST90001804	rs1883832	CD27 on IgD- CD3	20	44746982
ebi-a-GCST90001804	rs162076	CD27 on IgD- CD3	20	46785499
ebi-a-GCST90001804	rs847078	CD27 on IgD- CD3	20	45170213
ebi-a-GCST90001805	rs141269963	CD27 on memory E	1	99804475
ebi-a-GCST90001805	rs481818	CD27 on memory E	1	234426306
ebi-a-GCST90001805	rs75918255	CD27 on memory E	3	32945761
ebi-a-GCST90001805	rs182174914	CD27 on memory E	3	80085882
ebi-a-GCST90001805	rs56237381	CD27 on memory E	3	32924804
ebi-a-GCST90001805	rs2140755	CD27 on memory E	3	65613051
ebi-a-GCST90001805	rs76929865	CD27 on memory E	3	31134534
ebi-a-GCST90001805	rs2526388	CD27 on memory E	3	50174886
ebi-a-GCST90001805	rs35574803	CD27 on memory E	3	32533246
ebi-a-GCST90001805	rs34177560	CD27 on memory E	4	11141629
ebi-a-GCST90001805	rs114976873	CD27 on memory E	5	36783531
ebi-a-GCST90001805	rs4716387	CD27 on memory E	6	170136777
ebi-a-GCST90001805	rs486182	CD27 on memory E	8	20806496
ebi-a-GCST90001805	rs79308430	CD27 on memory E	10	124542720
ebi-a-GCST90001805	rs2445333	CD27 on memory E	11	5080234
ebi-a-GCST90001805	rs147714617	CD27 on memory E	12	6605299
ebi-a-GCST90001805	rs25680	CD27 on memory E	12	6554628
ebi-a-GCST90001805	rs11865074	CD27 on memory E	16	87903640
ebi-a-GCST90001805	rs528269417	CD27 on memory E	17	48258285
ebi-a-GCST90001805	rs3174744	CD27 on memory E	17	7322245
ebi-a-GCST90001805	rs150306315	CD27 on memory E	19	16824730
ebi-a-GCST90001805	rs973446	CD27 on memory E	20	44094375
ebi-a-GCST90001805	rs11086984	CD27 on memory E	20	44511627
ebi-a-GCST90001805	rs1883832	CD27 on memory E	20	44746982
ebi-a-GCST90001805	rs72485816	CD27 on memory E	20	45314435
ebi-a-GCST90001805	rs79645041	CD27 on memory E	20	57678574

ebi-a-GCST90001806	rs112625026	CD27 on unsw men	1	212877210
ebi-a-GCST90001806	rs34101571	CD27 on unsw men	1	31211894
ebi-a-GCST90001806	rs1801274	CD27 on unsw men	1	161479745
ebi-a-GCST90001806	rs72625293	CD27 on unsw men	2	185499138
ebi-a-GCST90001806	rs75765646	CD27 on unsw men	2	6446274
ebi-a-GCST90001806	rs76929865	CD27 on unsw men	3	31134534
ebi-a-GCST90001806	rs35574803	CD27 on unsw men	3	32533246
ebi-a-GCST90001806	rs75918255	CD27 on unsw men	3	32945761
ebi-a-GCST90001806	rs144642376	CD27 on unsw men	3	36349691
ebi-a-GCST90001806	rs56237381	CD27 on unsw men	3	32924804
ebi-a-GCST90001806	rs6777433	CD27 on unsw men	3	33165436
ebi-a-GCST90001806	rs12651722	CD27 on unsw men	5	161994889
ebi-a-GCST90001806	rs36667	CD27 on unsw men	5	36746983
ebi-a-GCST90001806	rs11769145	CD27 on unsw men	7	70704068
ebi-a-GCST90001806	rs6997361	CD27 on unsw men	8	8349757
ebi-a-GCST90001806	rs11007079	CD27 on unsw men	10	28679045
ebi-a-GCST90001806	rs143164248	CD27 on unsw men	10	28945318
ebi-a-GCST90001806	rs25680	CD27 on unsw men	12	6554628
ebi-a-GCST90001806	rs147714617	CD27 on unsw men	12	6605299
ebi-a-GCST90001806	rs138260228	CD27 on unsw men	14	29256374
ebi-a-GCST90001806	rs709589	CD27 on unsw men	14	106306841
ebi-a-GCST90001806	rs72812418	CD27 on unsw men	16	46869339
ebi-a-GCST90001806	rs72802982	CD27 on unsw men	16	78165161
ebi-a-GCST90001806	rs369017459	CD27 on unsw men	16	33471549
ebi-a-GCST90001806	rs117896307	CD27 on unsw men	18	70433020
ebi-a-GCST90001806	rs162076	CD27 on unsw men	20	46785499
ebi-a-GCST90001806	rs6112686	CD27 on unsw men	20	2047479
ebi-a-GCST90001806	rs11086984	CD27 on unsw men	20	44511627
ebi-a-GCST90001806	rs4810485	CD27 on unsw men	20	44747947
ebi-a-GCST90001806	rs72645230	CD27 on unsw men	20	46242688
ebi-a-GCST90001806	rs4253791	CD27 on unsw men	22	46585927
ebi-a-GCST90001807	rs112481165	CD27 on PB/PC	1	247244688
ebi-a-GCST90001807	rs183873886	CD27 on PB/PC	1	79837189
ebi-a-GCST90001807	rs115601228	CD27 on PB/PC	1	195070886
ebi-a-GCST90001807	rs4657041	CD27 on PB/PC	1	161478859
ebi-a-GCST90001807	rs10910352	CD27 on PB/PC	1	234267536
ebi-a-GCST90001807	rs34020577	CD27 on PB/PC	4	175917268
ebi-a-GCST90001807	rs76112278	CD27 on PB/PC	4	15915069
ebi-a-GCST90001807	rs112881275	CD27 on PB/PC	5	16006239
ebi-a-GCST90001807	rs6882346	CD27 on PB/PC	5	121749877
ebi-a-GCST90001807	rs12528717	CD27 on PB/PC	6	39028606
ebi-a-GCST90001807	rs34850955	CD27 on PB/PC	7	69588970
ebi-a-GCST90001807	rs73202627	CD27 on PB/PC	8	12580949
ebi-a-GCST90001807	rs11782223	CD27 on PB/PC	8	139337902
ebi-a-GCST90001807	rs960984	CD27 on PB/PC	12	55344683
ebi-a-GCST90001807	rs56221723	CD27 on PB/PC	14	31280307
ebi-a-GCST90001807	rs8059711	CD27 on PB/PC	16	87016628
ebi-a-GCST90001807	rs1077349	CD27 on PB/PC	20	57072098

ebi-a-GCST90001807	rs185015514	CD27 on PB/PC	20	32288479
ebi-a-GCST90001807	rs10427757	CD27 on PB/PC	22	37419582
ebi-a-GCST90001808	rs34101571	CD27 on sw mem	1	31211894
ebi-a-GCST90001808	rs115383596	CD27 on sw mem	1	99399788
ebi-a-GCST90001808	rs12727962	CD27 on sw mem	1	31686211
ebi-a-GCST90001808	rs75392984	CD27 on sw mem	1	20290573
ebi-a-GCST90001808	rs12129123	CD27 on sw mem	1	10083709
ebi-a-GCST90001808	rs1801274	CD27 on sw mem	1	161479745
ebi-a-GCST90001808	rs144642376	CD27 on sw mem	3	36349691
ebi-a-GCST90001808	rs115933247	CD27 on sw mem	3	107086449
ebi-a-GCST90001808	rs12495781	CD27 on sw mem	3	127619207
ebi-a-GCST90001808	rs35574803	CD27 on sw mem	3	32533246
ebi-a-GCST90001808	rs75918255	CD27 on sw mem	3	32945761
ebi-a-GCST90001808	rs7621492	CD27 on sw mem	3	32302456
ebi-a-GCST90001808	rs76929865	CD27 on sw mem	3	31134534
ebi-a-GCST90001808	rs116522219	CD27 on sw mem	3	36625286
ebi-a-GCST90001808	rs9885107	CD27 on sw mem	5	117207047
ebi-a-GCST90001808	rs112339885	CD27 on sw mem	5	79389016
ebi-a-GCST90001808	rs10816146	CD27 on sw mem	9	9594355
ebi-a-GCST90001808	rs141876897	CD27 on sw mem	9	94241794
ebi-a-GCST90001808	rs143164248	CD27 on sw mem	10	28945318
ebi-a-GCST90001808	rs73030401	CD27 on sw mem	11	131947318
ebi-a-GCST90001808	rs34139704	CD27 on sw mem	12	106279933
ebi-a-GCST90001808	rs147714617	CD27 on sw mem	12	6605299
ebi-a-GCST90001808	rs61927692	CD27 on sw mem	12	47529273
ebi-a-GCST90001808	rs25680	CD27 on sw mem	12	6554628
ebi-a-GCST90001808	rs7179434	CD27 on sw mem	15	22523362
ebi-a-GCST90001808	rs11086984	CD27 on sw mem	20	44511627
ebi-a-GCST90001808	rs1883832	CD27 on sw mem	20	44746982
ebi-a-GCST90001808	rs4810496	CD27 on sw mem	20	44954283
ebi-a-GCST90001808	rs9989924	CD27 on sw mem	20	42019380
ebi-a-GCST90001808	rs973446	CD27 on sw mem	20	44094375
ebi-a-GCST90001808	rs162076	CD27 on sw mem	20	46785499
ebi-a-GCST90001809	rs2811620	CD38 on CD20-	1	37871985
ebi-a-GCST90001809	rs61742836	CD38 on CD20-	3	100364914
ebi-a-GCST90001809	rs76310795	CD38 on CD20-	4	122189884
ebi-a-GCST90001809	rs79971636	CD38 on CD20-	4	35097278
ebi-a-GCST90001809	rs56693306	CD38 on CD20-	4	4063900
ebi-a-GCST90001809	rs7679041	CD38 on CD20-	4	13722729
ebi-a-GCST90001809	rs55821920	CD38 on CD20-	5	146552726
ebi-a-GCST90001809	rs3117290	CD38 on CD20-	6	29649944
ebi-a-GCST90001809	rs28452034	CD38 on CD20-	8	97029284
ebi-a-GCST90001809	rs7018225	CD38 on CD20-	8	131361477
ebi-a-GCST90001809	rs28756677	CD38 on CD20-	9	140135216
ebi-a-GCST90001809	rs55720255	CD38 on CD20-	9	113328755
ebi-a-GCST90001809	rs35709687	CD38 on CD20-	11	123396674
ebi-a-GCST90001809	rs10743387	CD38 on CD20-	12	20807481
ebi-a-GCST90001809	rs117775298	CD38 on CD20-	12	103684673

ebi-a-GCST90001809	rs1838357	CD38 on CD20-	13	93740906
ebi-a-GCST90001809	rs34259829	CD38 on CD20-	17	60126189
ebi-a-GCST90001809	rs11663828	CD38 on CD20-	18	64848183
ebi-a-GCST90001809	rs2628482	CD38 on CD20-	20	3507317
ebi-a-GCST90001809	rs5996804	CD38 on CD20-	22	25369319
ebi-a-GCST90001810	rs78814315	CD38 on CD3- CD	1	23729223
ebi-a-GCST90001810	rs7522863	CD38 on CD3- CD	1	39385086
ebi-a-GCST90001810	rs79113171	CD38 on CD3- CD	3	145525293
ebi-a-GCST90001810	rs76112278	CD38 on CD3- CD	4	15915069
ebi-a-GCST90001810	rs76476334	CD38 on CD3- CD	4	14111959
ebi-a-GCST90001810	rs9292443	CD38 on CD3- CD	5	31805341
ebi-a-GCST90001810	rs139249541	CD38 on CD3- CD	6	102294761
ebi-a-GCST90001810	rs79251210	CD38 on CD3- CD	6	100468602
ebi-a-GCST90001810	rs3807839	CD38 on CD3- CD	7	8191966
ebi-a-GCST90001810	rs2050811	CD38 on CD3- CD	10	128915719
ebi-a-GCST90001810	rs112430743	CD38 on CD3- CD	10	23725269
ebi-a-GCST90001810	rs7919742	CD38 on CD3- CD	10	61273488
ebi-a-GCST90001810	rs548316	CD38 on CD3- CD	11	128077683
ebi-a-GCST90001810	rs1920695	CD38 on CD3- CD	12	42348524
ebi-a-GCST90001810	rs4781564	CD38 on CD3- CD	16	14128524
ebi-a-GCST90001810	rs12979358	CD38 on CD3- CD	19	54906099
ebi-a-GCST90001810	rs62121123	CD38 on CD3- CD	19	18166559
ebi-a-GCST90001810	rs9637082	CD38 on CD3- CD	21	21213236
ebi-a-GCST90001811	rs12032479	CD38 on IgD+ CD	1	194997589
ebi-a-GCST90001811	rs142962704	CD38 on IgD+ CD	3	128075585
ebi-a-GCST90001811	rs79300919	CD38 on IgD+ CD	3	14288322
ebi-a-GCST90001811	rs572542	CD38 on IgD+ CD	4	14029370
ebi-a-GCST90001811	rs76112278	CD38 on IgD+ CD	4	15915069
ebi-a-GCST90001811	rs28393044	CD38 on IgD+ CD	4	157437839
ebi-a-GCST90001811	rs115062208	CD38 on IgD+ CD	4	15956227
ebi-a-GCST90001811	rs76354299	CD38 on IgD+ CD	4	15037408
ebi-a-GCST90001811	rs6895789	CD38 on IgD+ CD	5	91363908
ebi-a-GCST90001811	rs2516708	CD38 on IgD+ CD	6	30228721
ebi-a-GCST90001811	rs1878808	CD38 on IgD+ CD	7	3020375
ebi-a-GCST90001811	rs72715691	CD38 on IgD+ CD	9	15034791
ebi-a-GCST90001811	rs11042077	CD38 on IgD+ CD	11	8825966
ebi-a-GCST90001811	rs9599916	CD38 on IgD+ CD	13	72594895
ebi-a-GCST90001811	rs17179337	CD38 on IgD+ CD	14	38174359
ebi-a-GCST90001811	rs147970068	CD38 on IgD+ CD	14	87885710
ebi-a-GCST90001811	rs78111008	CD38 on IgD+ CD	20	12142943
ebi-a-GCST90001812	rs115109055	CD38 on IgD+ CD	1	17640731
ebi-a-GCST90001812	rs137987320	CD38 on IgD+ CD	4	8469228
ebi-a-GCST90001812	rs186090691	CD38 on IgD+ CD	4	8970919
ebi-a-GCST90001812	rs76112278	CD38 on IgD+ CD	4	15915069
ebi-a-GCST90001812	rs138847726	CD38 on IgD+ CD	4	13020910
ebi-a-GCST90001812	rs807784	CD38 on IgD+ CD	4	13906853
ebi-a-GCST90001812	rs115062208	CD38 on IgD+ CD	4	15956227
ebi-a-GCST90001812	rs527979176	CD38 on IgD+ CD	6	67366861

ebi-a-GCST90001812	rs113933327	CD38 on IgD+ CD3	6	162656478
ebi-a-GCST90001812	rs139833260	CD38 on IgD+ CD3	7	119003006
ebi-a-GCST90001812	rs186547410	CD38 on IgD+ CD3	10	30612367
ebi-a-GCST90001812	rs145666904	CD38 on IgD+ CD3	11	119050896
ebi-a-GCST90001812	rs4842778	CD38 on IgD+ CD3	12	91960674
ebi-a-GCST90001812	rs7139178	CD38 on IgD+ CD3	12	129446340
ebi-a-GCST90001812	rs79666361	CD38 on IgD+ CD3	17	10055216
ebi-a-GCST90001812	rs62060138	CD38 on IgD+ CD3	17	48408733
ebi-a-GCST90001812	rs2058344	CD38 on IgD+ CD3	18	59117964
ebi-a-GCST90001812	rs34022324	CD38 on IgD+ CD3	19	14567159
ebi-a-GCST90001812	rs79277914	CD38 on IgD+ CD3	20	61698841
ebi-a-GCST90001812	rs73367318	CD38 on IgD+ CD3	21	43441479
ebi-a-GCST90001813	rs10737766	CD38 on IgD+ CD3	1	116770372
ebi-a-GCST90001813	rs16858021	CD38 on IgD+ CD3	1	181677633
ebi-a-GCST90001813	rs151139674	CD38 on IgD+ CD3	1	36764904
ebi-a-GCST90001813	rs140874823	CD38 on IgD+ CD3	2	5755140
ebi-a-GCST90001813	rs55770731	CD38 on IgD+ CD3	2	211744617
ebi-a-GCST90001813	rs16867231	CD38 on IgD+ CD3	2	181050023
ebi-a-GCST90001813	rs115062208	CD38 on IgD+ CD3	4	15956227
ebi-a-GCST90001813	rs76112278	CD38 on IgD+ CD3	4	15915069
ebi-a-GCST90001813	rs115636025	CD38 on IgD+ CD3	4	9452383
ebi-a-GCST90001813	rs76354299	CD38 on IgD+ CD3	4	15037408
ebi-a-GCST90001813	rs78416268	CD38 on IgD+ CD3	4	55880433
ebi-a-GCST90001813	rs572542	CD38 on IgD+ CD3	4	14029370
ebi-a-GCST90001813	rs4235009	CD38 on IgD+ CD3	4	26233819
ebi-a-GCST90001813	rs76551277	CD38 on IgD+ CD3	5	91499761
ebi-a-GCST90001813	rs62405562	CD38 on IgD+ CD3	6	32451834
ebi-a-GCST90001813	rs117455593	CD38 on IgD+ CD3	6	169449285
ebi-a-GCST90001813	rs11763462	CD38 on IgD+ CD3	7	130342978
ebi-a-GCST90001813	rs79340798	CD38 on IgD+ CD3	8	90861273
ebi-a-GCST90001813	rs12764658	CD38 on IgD+ CD3	10	69890943
ebi-a-GCST90001813	rs79411094	CD38 on IgD+ CD3	11	20181876
ebi-a-GCST90001813	rs138459438	CD38 on IgD+ CD3	12	18290606
ebi-a-GCST90001813	rs80148042	CD38 on IgD+ CD3	13	72776004
ebi-a-GCST90001813	rs1409571	CD38 on IgD+ CD3	13	72672465
ebi-a-GCST90001813	rs75416382	CD38 on IgD+ CD3	15	89292965
ebi-a-GCST90001814	rs141365416	CD38 on IgD- CD3	1	102236814
ebi-a-GCST90001814	rs116152899	CD38 on IgD- CD3	2	1833668
ebi-a-GCST90001814	rs141403650	CD38 on IgD- CD3	3	3381223
ebi-a-GCST90001814	rs115860771	CD38 on IgD- CD3	5	176167900
ebi-a-GCST90001814	rs706295	CD38 on IgD- CD3	5	14767502
ebi-a-GCST90001814	rs145546485	CD38 on IgD- CD3	5	56047410
ebi-a-GCST90001814	rs145388152	CD38 on IgD- CD3	6	4347705
ebi-a-GCST90001814	rs144135856	CD38 on IgD- CD3	9	129618074
ebi-a-GCST90001814	rs7933950	CD38 on IgD- CD3	11	97355697
ebi-a-GCST90001814	rs58557469	CD38 on IgD- CD3	11	37862120
ebi-a-GCST90001814	rs6420407	CD38 on IgD- CD3	16	78856580
ebi-a-GCST90001814	rs145853751	CD38 on IgD- CD3	17	65601110

ebi-a-GCST90001814	rs11668091	CD38 on IgD- CD3	19	51583553
ebi-a-GCST90001814	rs10421176	CD38 on IgD- CD3	19	13425338
ebi-a-GCST90001814	rs17777402	CD38 on IgD- CD3	20	5256339
ebi-a-GCST90001815	rs35188742	CD38 on IgD- CD3	2	221344098
ebi-a-GCST90001815	rs73230764	CD38 on IgD- CD3	4	17289610
ebi-a-GCST90001815	rs115062208	CD38 on IgD- CD3	4	15956227
ebi-a-GCST90001815	rs76112278	CD38 on IgD- CD3	4	15915069
ebi-a-GCST90001815	rs76354299	CD38 on IgD- CD3	4	15037408
ebi-a-GCST90001815	rs151006904	CD38 on IgD- CD3	5	126906954
ebi-a-GCST90001815	rs9378446	CD38 on IgD- CD3	6	5895137
ebi-a-GCST90001815	rs13209828	CD38 on IgD- CD3	6	92746910
ebi-a-GCST90001815	rs183168900	CD38 on IgD- CD3	7	22539219
ebi-a-GCST90001815	rs75699914	CD38 on IgD- CD3	8	114698755
ebi-a-GCST90001815	rs141885962	CD38 on IgD- CD3	10	66791343
ebi-a-GCST90001815	rs80089599	CD38 on IgD- CD3	12	18183604
ebi-a-GCST90001815	rs143554453	CD38 on IgD- CD3	12	12727024
ebi-a-GCST90001815	rs74694612	CD38 on IgD- CD3	13	36783592
ebi-a-GCST90001815	rs9544468	CD38 on IgD- CD3	13	77933504
ebi-a-GCST90001815	rs17410241	CD38 on IgD- CD3	14	34190494
ebi-a-GCST90001815	rs13331697	CD38 on IgD- CD3	16	81910840
ebi-a-GCST90001815	rs55943003	CD38 on IgD- CD3	18	52754086
ebi-a-GCST90001815	rs6115163	CD38 on IgD- CD3	20	2590100
ebi-a-GCST90001815	rs373925792	CD38 on IgD- CD3	21	46707932
ebi-a-GCST90001815	rs147563555	CD38 on IgD- CD3	22	30044036
ebi-a-GCST90001816	rs10737766	CD38 on naive-mat	1	116770372
ebi-a-GCST90001816	rs12032479	CD38 on naive-mat	1	194997589
ebi-a-GCST90001816	rs7579256	CD38 on naive-mat	2	36938218
ebi-a-GCST90001816	rs139476939	CD38 on naive-mat	2	28422630
ebi-a-GCST90001816	rs76354299	CD38 on naive-mat	4	15037408
ebi-a-GCST90001816	rs115062208	CD38 on naive-mat	4	15956227
ebi-a-GCST90001816	rs13118578	CD38 on naive-mat	4	26202275
ebi-a-GCST90001816	rs76112278	CD38 on naive-mat	4	15915069
ebi-a-GCST90001816	rs572542	CD38 on naive-mat	4	14029370
ebi-a-GCST90001816	rs6895789	CD38 on naive-mat	5	91363908
ebi-a-GCST90001816	rs34893127	CD38 on naive-mat	5	67123177
ebi-a-GCST90001816	rs10073475	CD38 on naive-mat	5	77504427
ebi-a-GCST90001816	rs78026509	CD38 on naive-mat	6	30074310
ebi-a-GCST90001816	rs73012119	CD38 on naive-mat	6	158951296
ebi-a-GCST90001816	rs753875	CD38 on naive-mat	6	170583341
ebi-a-GCST90001816	rs76209777	CD38 on naive-mat	8	1277613
ebi-a-GCST90001816	rs72715691	CD38 on naive-mat	9	15034791
ebi-a-GCST90001816	rs146407097	CD38 on naive-mat	9	34935516
ebi-a-GCST90001816	rs11042077	CD38 on naive-mat	11	8825966
ebi-a-GCST90001816	rs12428439	CD38 on naive-mat	13	59808190
ebi-a-GCST90001816	rs9599916	CD38 on naive-mat	13	72594895
ebi-a-GCST90001816	rs77636066	CD38 on naive-mat	13	94914513
ebi-a-GCST90001816	rs12435334	CD38 on naive-mat	14	38169407
ebi-a-GCST90001816	rs11853686	CD38 on naive-mat	15	24189756

ebi-a-GCST90001816	rs112236163	CD38 on naive-mat	18	51428440
ebi-a-GCST90001816	rs78111008	CD38 on naive-mat	20	12142943
ebi-a-GCST90001817	rs1801274	CD38 on PB/PC	1	161479745
ebi-a-GCST90001817	rs112012521	CD38 on PB/PC	2	34922080
ebi-a-GCST90001817	rs72878268	CD38 on PB/PC	2	171318563
ebi-a-GCST90001817	rs9826331	CD38 on PB/PC	3	139044466
ebi-a-GCST90001817	rs7617631	CD38 on PB/PC	3	67287001
ebi-a-GCST90001817	rs335080	CD38 on PB/PC	4	182981534
ebi-a-GCST90001817	rs55985999	CD38 on PB/PC	4	58491837
ebi-a-GCST90001817	rs818892	CD38 on PB/PC	9	6997867
ebi-a-GCST90001817	rs10991116	CD38 on PB/PC	9	106896351
ebi-a-GCST90001817	rs9423546	CD38 on PB/PC	10	4823794
ebi-a-GCST90001817	rs2671612	CD38 on PB/PC	10	61489710
ebi-a-GCST90001817	rs1975261	CD38 on PB/PC	13	26808803
ebi-a-GCST90001817	rs141081775	CD38 on PB/PC	14	95092885
ebi-a-GCST90001817	rs17621486	CD38 on PB/PC	17	50682160
ebi-a-GCST90001817	rs4789573	CD38 on PB/PC	17	76279427
ebi-a-GCST90001817	rs35436350	CD38 on PB/PC	20	57093605
ebi-a-GCST90001818	rs13305986	CD38 on IgD+	1	11908505
ebi-a-GCST90001818	rs1164064	CD38 on IgD+	3	109462400
ebi-a-GCST90001818	rs80303855	CD38 on IgD+	4	64334348
ebi-a-GCST90001818	rs1511039	CD38 on IgD+	4	13905535
ebi-a-GCST90001818	rs76112278	CD38 on IgD+	4	15915069
ebi-a-GCST90001818	rs34843244	CD38 on IgD+	5	90267368
ebi-a-GCST90001818	rs74728241	CD38 on IgD+	6	125895275
ebi-a-GCST90001818	rs62405562	CD38 on IgD+	6	32451834
ebi-a-GCST90001818	rs11763462	CD38 on IgD+	7	130342978
ebi-a-GCST90001818	rs4929931	CD38 on IgD+	11	8746684
ebi-a-GCST90001818	rs138459438	CD38 on IgD+	12	18290606
ebi-a-GCST90001818	rs12435334	CD38 on IgD+	14	38169407
ebi-a-GCST90001818	rs3108001	CD38 on IgD+	18	27918590
ebi-a-GCST90001818	rs150215963	CD38 on IgD+	18	64825222
ebi-a-GCST90001818	rs55685624	CD38 on IgD+	21	39668476
ebi-a-GCST90001818	rs144392728	CD38 on IgD+	22	33029820
ebi-a-GCST90001819	rs41310410	CD38 on transitiona	1	19203961
ebi-a-GCST90001819	rs140192272	CD38 on transitiona	1	99413231
ebi-a-GCST90001819	rs197429	CD38 on transitiona	1	112267500
ebi-a-GCST90001819	rs57408613	CD38 on transitiona	2	181635654
ebi-a-GCST90001819	rs78282820	CD38 on transitiona	2	170932700
ebi-a-GCST90001819	rs9845482	CD38 on transitiona	3	184801494
ebi-a-GCST90001819	rs113248969	CD38 on transitiona	3	31815483
ebi-a-GCST90001819	rs2070478	CD38 on transitiona	3	37526985
ebi-a-GCST90001819	rs115062208	CD38 on transitiona	4	15956227
ebi-a-GCST90001819	rs76112278	CD38 on transitiona	4	15915069
ebi-a-GCST90001819	rs140447118	CD38 on transitiona	4	24413705
ebi-a-GCST90001819	rs572542	CD38 on transitiona	4	14029370
ebi-a-GCST90001819	rs149852991	CD38 on transitiona	4	15044338
ebi-a-GCST90001819	rs138612228	CD38 on transitiona	5	27458351

ebi-a-GCST90001819	rs140554292	CD38 on transitiona	5	87562189
ebi-a-GCST90001819	rs10231147	CD38 on transitiona	7	144509395
ebi-a-GCST90001819	rs11005889	CD38 on transitiona	10	59422142
ebi-a-GCST90001819	rs4288784	CD38 on transitiona	11	113745608
ebi-a-GCST90001819	rs75796463	CD38 on transitiona	14	105086574
ebi-a-GCST90001819	rs62002679	CD38 on transitiona	15	32004133
ebi-a-GCST90001819	rs62063474	CD38 on transitiona	17	6279415
ebi-a-GCST90001819	rs34379433	CD38 on transitiona	18	54232162
ebi-a-GCST90001819	rs2311849	CD38 on transitiona	19	47317539
ebi-a-GCST90001819	rs7251792	CD38 on transitiona	19	1021725
ebi-a-GCST90001820	rs147051683	IgD on IgD+ CD24	1	170103711
ebi-a-GCST90001820	rs72871227	IgD on IgD+ CD24	2	151000647
ebi-a-GCST90001820	rs72944511	IgD on IgD+ CD24	2	216742657
ebi-a-GCST90001820	rs144583907	IgD on IgD+ CD24	2	217837059
ebi-a-GCST90001820	rs9288041	IgD on IgD+ CD24	2	180583707
ebi-a-GCST90001820	rs12505403	IgD on IgD+ CD24	4	130367811
ebi-a-GCST90001820	rs73031919	IgD on IgD+ CD24	5	1980101
ebi-a-GCST90001820	rs10076053	IgD on IgD+ CD24	5	150649861
ebi-a-GCST90001820	rs1794511	IgD on IgD+ CD24	6	32671207
ebi-a-GCST90001820	rs139084170	IgD on IgD+ CD24	6	43351686
ebi-a-GCST90001820	rs9265515	IgD on IgD+ CD24	6	31297744
ebi-a-GCST90001820	rs7746553	IgD on IgD+ CD24	6	31895973
ebi-a-GCST90001820	rs147854460	IgD on IgD+ CD24	6	143010177
ebi-a-GCST90001820	rs112421400	IgD on IgD+ CD24	6	28205826
ebi-a-GCST90001820	rs73022545	IgD on IgD+ CD24	6	164591715
ebi-a-GCST90001820	rs1181899	IgD on IgD+ CD24	7	76993184
ebi-a-GCST90001820	rs850492	IgD on IgD+ CD24	7	145314534
ebi-a-GCST90001820	rs149401019	IgD on IgD+ CD24	7	2250057
ebi-a-GCST90001820	rs80149865	IgD on IgD+ CD24	8	120027617
ebi-a-GCST90001820	rs74143009	IgD on IgD+ CD24	10	69860366
ebi-a-GCST90001820	rs55675835	IgD on IgD+ CD24	12	210957
ebi-a-GCST90001820	rs2819455	IgD on IgD+ CD24	14	105445412
ebi-a-GCST90001820	rs709589	IgD on IgD+ CD24	14	106306841
ebi-a-GCST90001820	rs11621990	IgD on IgD+ CD24	14	106875768
ebi-a-GCST90001820	rs58259062	IgD on IgD+ CD24	14	107284188
ebi-a-GCST90001820	rs28866515	IgD on IgD+ CD24	15	40971835
ebi-a-GCST90001820	rs4796030	IgD on IgD+ CD24	17	33330150
ebi-a-GCST90001820	rs2630640	IgD on IgD+ CD24	17	68538485
ebi-a-GCST90001820	rs151050474	IgD on IgD+ CD24	19	57875874
ebi-a-GCST90001821	rs74720509	IgD on IgD+ CD24	1	11891626
ebi-a-GCST90001821	rs72871227	IgD on IgD+ CD24	2	151000647
ebi-a-GCST90001821	rs933793	IgD on IgD+ CD24	2	106511846
ebi-a-GCST90001821	rs139357193	IgD on IgD+ CD24	3	51971998
ebi-a-GCST90001821	rs13096753	IgD on IgD+ CD24	3	74511065
ebi-a-GCST90001821	rs56783728	IgD on IgD+ CD24	6	29459769
ebi-a-GCST90001821	rs9467989	IgD on IgD+ CD24	6	27064275
ebi-a-GCST90001821	rs4416670	IgD on IgD+ CD24	6	43950453
ebi-a-GCST90001821	rs9639820	IgD on IgD+ CD24	7	40094641

ebi-a-GCST90001821	rs41274268	IgD on IgD+ CD24	9	100886920
ebi-a-GCST90001821	rs10987840	IgD on IgD+ CD24	9	130793178
ebi-a-GCST90001821	rs72819694	IgD on IgD+ CD24	10	84605536
ebi-a-GCST90001821	rs78102093	IgD on IgD+ CD24	10	12691374
ebi-a-GCST90001821	rs2398397	IgD on IgD+ CD24	12	128804435
ebi-a-GCST90001821	rs11841715	IgD on IgD+ CD24	13	60244803
ebi-a-GCST90001821	rs2403197	IgD on IgD+ CD24	14	104053764
ebi-a-GCST90001821	rs56112295	IgD on IgD+ CD24	14	105877057
ebi-a-GCST90001821	rs28723652	IgD on IgD+ CD24	14	106644507
ebi-a-GCST90001821	rs72715996	IgD on IgD+ CD24	14	105277950
ebi-a-GCST90001821	rs709589	IgD on IgD+ CD24	14	106306841
ebi-a-GCST90001821	rs78844770	IgD on IgD+ CD24	15	87829544
ebi-a-GCST90001821	rs28634695	IgD on IgD+ CD24	15	40856113
ebi-a-GCST90001821	rs719611	IgD on IgD+ CD24	15	63277356
ebi-a-GCST90001821	rs142302290	IgD on IgD+ CD24	16	55222535
ebi-a-GCST90001821	rs35501848	IgD on IgD+ CD24	17	62021003
ebi-a-GCST90001821	rs887387	IgD on IgD+ CD24	17	3828702
ebi-a-GCST90001821	rs117797158	IgD on IgD+ CD24	17	78954840
ebi-a-GCST90001821	rs2061780	IgD on IgD+ CD24	19	28481413
ebi-a-GCST90001821	rs71314904	IgD on IgD+ CD24	22	34855476
ebi-a-GCST90001821	rs28523633	IgD on IgD+ CD24	22	49098031
ebi-a-GCST90001822	rs80179084	IgD on IgD+ CD38	1	168838486
ebi-a-GCST90001822	rs115447005	IgD on IgD+ CD38	2	215247577
ebi-a-GCST90001822	rs75131268	IgD on IgD+ CD38	4	179196108
ebi-a-GCST90001822	rs875214	IgD on IgD+ CD38	5	76713643
ebi-a-GCST90001822	rs11759718	IgD on IgD+ CD38	6	112572117
ebi-a-GCST90001822	rs3134971	IgD on IgD+ CD38	6	32654149
ebi-a-GCST90001822	rs11969118	IgD on IgD+ CD38	6	27700822
ebi-a-GCST90001822	rs2071800	IgD on IgD+ CD38	6	32714143
ebi-a-GCST90001822	rs571829761	IgD on IgD+ CD38	7	47012316
ebi-a-GCST90001822	rs6995633	IgD on IgD+ CD38	8	128440651
ebi-a-GCST90001822	rs12554596	IgD on IgD+ CD38	9	37002142
ebi-a-GCST90001822	rs72743463	IgD on IgD+ CD38	9	96108427
ebi-a-GCST90001822	rs7081413	IgD on IgD+ CD38	10	68875634
ebi-a-GCST90001822	rs875706	IgD on IgD+ CD38	11	126315673
ebi-a-GCST90001822	rs2473909	IgD on IgD+ CD38	11	34213286
ebi-a-GCST90001822	rs73192651	IgD on IgD+ CD38	13	50212885
ebi-a-GCST90001822	rs709589	IgD on IgD+ CD38	14	106306841
ebi-a-GCST90001822	rs28723652	IgD on IgD+ CD38	14	106644507
ebi-a-GCST90001822	rs147653204	IgD on IgD+ CD38	14	107117890
ebi-a-GCST90001822	rs4775581	IgD on IgD+ CD38	15	63267877
ebi-a-GCST90001822	rs7162750	IgD on IgD+ CD38	15	38002606
ebi-a-GCST90001822	rs8033829	IgD on IgD+ CD38	15	41038693
ebi-a-GCST90001822	rs183915	IgD on IgD+ CD38	15	40119703
ebi-a-GCST90001822	rs2280736	IgD on IgD+ CD38	16	5323457
ebi-a-GCST90001822	rs9923814	IgD on IgD+ CD38	16	48808506
ebi-a-GCST90001822	rs76252198	IgD on IgD+ CD38	18	61939282
ebi-a-GCST90001822	rs111323173	IgD on IgD+ CD38	19	16773301

ebi-a-GCST90001822	rs17733969	IgD on IgD+ CD38	20	51360154
ebi-a-GCST90001822	rs139047	IgD on IgD+ CD38	22	44323074
ebi-a-GCST90001823	rs568035	IgD on IgD+ CD38	1	156110167
ebi-a-GCST90001823	rs7754054	IgD on IgD+ CD38	6	29392503
ebi-a-GCST90001823	rs3093971	IgD on IgD+ CD38	6	31424607
ebi-a-GCST90001823	rs56247432	IgD on IgD+ CD38	6	74955867
ebi-a-GCST90001823	rs2735071	IgD on IgD+ CD38	6	29944979
ebi-a-GCST90001823	rs2040406	IgD on IgD+ CD38	6	32603007
ebi-a-GCST90001823	rs546103177	IgD on IgD+ CD38	6	42913071
ebi-a-GCST90001823	rs10277136	IgD on IgD+ CD38	7	153304156
ebi-a-GCST90001823	rs111505634	IgD on IgD+ CD38	7	10147518
ebi-a-GCST90001823	rs919590	IgD on IgD+ CD38	8	13061713
ebi-a-GCST90001823	rs10823811	IgD on IgD+ CD38	10	73404650
ebi-a-GCST90001823	rs72863094	IgD on IgD+ CD38	11	11497311
ebi-a-GCST90001823	rs74717895	IgD on IgD+ CD38	11	9145922
ebi-a-GCST90001823	rs79841057	IgD on IgD+ CD38	12	103294260
ebi-a-GCST90001823	rs709589	IgD on IgD+ CD38	14	106306841
ebi-a-GCST90001823	rs28723652	IgD on IgD+ CD38	14	106644507
ebi-a-GCST90001823	rs117303087	IgD on IgD+ CD38	14	103824600
ebi-a-GCST90001823	rs78288740	IgD on IgD+ CD38	15	60339435
ebi-a-GCST90001823	rs539975	IgD on IgD+ CD38	18	7562317
ebi-a-GCST90001824	rs7530780	IgD on IgD+ CD38	1	93130268
ebi-a-GCST90001824	rs1037633	IgD on IgD+ CD38	3	23750521
ebi-a-GCST90001824	rs1227420	IgD on IgD+ CD38	4	93132438
ebi-a-GCST90001824	rs139488523	IgD on IgD+ CD38	4	169621111
ebi-a-GCST90001824	rs72757668	IgD on IgD+ CD38	5	67542189
ebi-a-GCST90001824	rs73398969	IgD on IgD+ CD38	6	29410258
ebi-a-GCST90001824	rs200468	IgD on IgD+ CD38	6	27757855
ebi-a-GCST90001824	rs793853	IgD on IgD+ CD38	6	24196925
ebi-a-GCST90001824	rs62442539	IgD on IgD+ CD38	7	2352594
ebi-a-GCST90001824	rs7853163	IgD on IgD+ CD38	9	615904
ebi-a-GCST90001824	rs76714110	IgD on IgD+ CD38	9	130880041
ebi-a-GCST90001824	rs3915868	IgD on IgD+ CD38	11	8833800
ebi-a-GCST90001824	rs12788641	IgD on IgD+ CD38	11	57851179
ebi-a-GCST90001824	rs149919568	IgD on IgD+ CD38	11	88104460
ebi-a-GCST90001824	rs118089299	IgD on IgD+ CD38	14	104674020
ebi-a-GCST90001824	rs709589	IgD on IgD+ CD38	14	106306841
ebi-a-GCST90001824	rs2018404	IgD on IgD+ CD38	14	105695446
ebi-a-GCST90001824	rs12436535	IgD on IgD+ CD38	14	106407257
ebi-a-GCST90001824	rs2338377	IgD on IgD+ CD38	14	106671275
ebi-a-GCST90001824	rs56658995	IgD on IgD+ CD38	14	107056278
ebi-a-GCST90001824	rs28669075	IgD on IgD+ CD38	15	40856521
ebi-a-GCST90001824	rs72764109	IgD on IgD+ CD38	15	87727196
ebi-a-GCST90001824	rs73569760	IgD on IgD+ CD38	16	63997246
ebi-a-GCST90001824	rs74017080	IgD on IgD+ CD38	16	48816092
ebi-a-GCST90001824	rs9614208	IgD on IgD+ CD38	22	44568078
ebi-a-GCST90001825	rs12126649	IgD on IgD+ CD38	1	4153262
ebi-a-GCST90001825	rs141507756	IgD on IgD+ CD38	3	106999706

ebi-a-GCST90001825	rs77606890	IgD on IgD+ CD38	3	74792288
ebi-a-GCST90001825	rs116643893	IgD on IgD+ CD38	4	137149414
ebi-a-GCST90001825	rs9443423	IgD on IgD+ CD38	6	78306504
ebi-a-GCST90001825	rs79925536	IgD on IgD+ CD38	6	29413972
ebi-a-GCST90001825	rs9639820	IgD on IgD+ CD38	7	40094641
ebi-a-GCST90001825	rs10228863	IgD on IgD+ CD38	7	20587690
ebi-a-GCST90001825	rs78102093	IgD on IgD+ CD38	10	12691374
ebi-a-GCST90001825	rs2601762	IgD on IgD+ CD38	10	33794680
ebi-a-GCST90001825	rs72819694	IgD on IgD+ CD38	10	84605536
ebi-a-GCST90001825	rs3903770	IgD on IgD+ CD38	13	50394478
ebi-a-GCST90001825	rs28723652	IgD on IgD+ CD38	14	106644507
ebi-a-GCST90001825	rs72715996	IgD on IgD+ CD38	14	105277950
ebi-a-GCST90001825	rs7143148	IgD on IgD+ CD38	14	105927433
ebi-a-GCST90001825	rs709589	IgD on IgD+ CD38	14	106306841
ebi-a-GCST90001825	rs28669075	IgD on IgD+ CD38	15	40856521
ebi-a-GCST90001825	rs142302290	IgD on IgD+ CD38	16	55222535
ebi-a-GCST90001825	rs35501848	IgD on IgD+ CD38	17	62021003
ebi-a-GCST90001825	rs117797158	IgD on IgD+ CD38	17	78954840
ebi-a-GCST90001825	rs150521764	IgD on IgD+ CD38	18	67714307
ebi-a-GCST90001825	rs6107127	IgD on IgD+ CD38	20	25979030
ebi-a-GCST90001826	rs9870291	IgD on unsw mem	3	8652528
ebi-a-GCST90001826	rs78261673	IgD on unsw mem	4	7463428
ebi-a-GCST90001826	rs75544266	IgD on unsw mem	4	104584997
ebi-a-GCST90001826	rs55861228	IgD on unsw mem	5	2501680
ebi-a-GCST90001826	rs73022545	IgD on unsw mem	6	164591715
ebi-a-GCST90001826	rs9276702	IgD on unsw mem	6	32756741
ebi-a-GCST90001826	rs241409	IgD on unsw mem	6	32861920
ebi-a-GCST90001826	rs3132571	IgD on unsw mem	6	30905313
ebi-a-GCST90001826	rs9267517	IgD on unsw mem	6	31573110
ebi-a-GCST90001826	rs78172850	IgD on unsw mem	6	47149877
ebi-a-GCST90001826	rs139084170	IgD on unsw mem	6	43351686
ebi-a-GCST90001826	rs1181899	IgD on unsw mem	7	76993184
ebi-a-GCST90001826	rs2465073	IgD on unsw mem	7	137928696
ebi-a-GCST90001826	rs147471736	IgD on unsw mem	9	97195653
ebi-a-GCST90001826	rs11014404	IgD on unsw mem	10	25408128
ebi-a-GCST90001826	rs147653204	IgD on unsw mem	14	107117890
ebi-a-GCST90001826	rs28723652	IgD on unsw mem	14	106644507
ebi-a-GCST90001826	rs76784736	IgD on unsw mem	14	71778339
ebi-a-GCST90001826	rs709589	IgD on unsw mem	14	106306841
ebi-a-GCST90001826	rs9923814	IgD on unsw mem	16	48808506
ebi-a-GCST90001826	rs10871327	IgD on unsw mem	16	76820976
ebi-a-GCST90001826	rs12609856	IgD on unsw mem	19	11807010
ebi-a-GCST90001826	rs142964241	IgD on unsw mem	20	20874741
ebi-a-GCST90001827	rs12745074	IgD on IgD+	1	7337805
ebi-a-GCST90001827	rs62175190	IgD on IgD+	2	180573329
ebi-a-GCST90001827	rs11721614	IgD on IgD+	4	1500753
ebi-a-GCST90001827	rs149936983	IgD on IgD+	5	141470423
ebi-a-GCST90001827	rs34312683	IgD on IgD+	6	67018244

ebi-a-GCST90001827	rs74322648	IgD on IgD+	6	19598450
ebi-a-GCST90001827	rs79925536	IgD on IgD+	6	29413972
ebi-a-GCST90001827	rs9443423	IgD on IgD+	6	78306504
ebi-a-GCST90001827	rs809558	IgD on IgD+	7	40011350
ebi-a-GCST90001827	rs403185	IgD on IgD+	8	26476721
ebi-a-GCST90001827	rs6476402	IgD on IgD+	9	33148552
ebi-a-GCST90001827	rs2601762	IgD on IgD+	10	33794680
ebi-a-GCST90001827	rs75459915	IgD on IgD+	12	24400511
ebi-a-GCST90001827	rs73192651	IgD on IgD+	13	50212885
ebi-a-GCST90001827	rs28723652	IgD on IgD+	14	106644507
ebi-a-GCST90001827	rs709589	IgD on IgD+	14	106306841
ebi-a-GCST90001827	rs72715996	IgD on IgD+	14	105277950
ebi-a-GCST90001827	rs737562	IgD on IgD+	15	63276177
ebi-a-GCST90001827	rs28669075	IgD on IgD+	15	40856521
ebi-a-GCST90001827	rs4968675	IgD on IgD+	17	62015230
ebi-a-GCST90001827	rs150521764	IgD on IgD+	18	67714307
ebi-a-GCST90001827	rs139534045	IgD on IgD+	20	21561213
ebi-a-GCST90001827	rs68173597	IgD on IgD+	21	21000598
ebi-a-GCST90001828	rs11164796	IgD on transitional	1	93144080
ebi-a-GCST90001828	rs4849052	IgD on transitional	2	112948678
ebi-a-GCST90001828	rs58758530	IgD on transitional	3	181465929
ebi-a-GCST90001828	rs2322165	IgD on transitional	3	71895073
ebi-a-GCST90001828	rs1037633	IgD on transitional	3	23750521
ebi-a-GCST90001828	rs16852581	IgD on transitional	3	168419832
ebi-a-GCST90001828	rs138793837	IgD on transitional	4	161937169
ebi-a-GCST90001828	rs115835855	IgD on transitional	4	131384142
ebi-a-GCST90001828	rs72919056	IgD on transitional	4	102701632
ebi-a-GCST90001828	rs9369356	IgD on transitional	6	42238899
ebi-a-GCST90001828	rs1392916	IgD on transitional	6	74518202
ebi-a-GCST90001828	rs79924684	IgD on transitional	6	144315102
ebi-a-GCST90001828	rs200474	IgD on transitional	6	27767640
ebi-a-GCST90001828	rs2819081	IgD on transitional	6	156913511
ebi-a-GCST90001828	rs646935	IgD on transitional	6	168112848
ebi-a-GCST90001828	rs118108236	IgD on transitional	6	94238065
ebi-a-GCST90001828	rs55871547	IgD on transitional	7	2354352
ebi-a-GCST90001828	rs142817955	IgD on transitional	7	33903759
ebi-a-GCST90001828	rs6998850	IgD on transitional	8	61848028
ebi-a-GCST90001828	rs34574485	IgD on transitional	9	38587724
ebi-a-GCST90001828	rs76714110	IgD on transitional	9	130880041
ebi-a-GCST90001828	rs3915868	IgD on transitional	11	8833800
ebi-a-GCST90001828	rs775001	IgD on transitional	12	24434976
ebi-a-GCST90001828	rs11047379	IgD on transitional	12	24492761
ebi-a-GCST90001828	rs142881434	IgD on transitional	14	107129280
ebi-a-GCST90001828	rs2983767	IgD on transitional	14	106226801
ebi-a-GCST90001828	rs2018404	IgD on transitional	14	105695446
ebi-a-GCST90001828	rs709589	IgD on transitional	14	106306841
ebi-a-GCST90001828	rs7184473	IgD on transitional	16	17732625
ebi-a-GCST90001828	rs118171108	IgD on transitional	17	3853980

ebi-a-GCST90001828	rs117309996	IgD on transitional	17	59009664
ebi-a-GCST90001828	rs9614208	IgD on transitional	22	44568078
ebi-a-GCST90001829	rs6680891	BAFF-R on B cell	1	164083691
ebi-a-GCST90001829	rs72658947	BAFF-R on B cell	1	30372026
ebi-a-GCST90001829	rs1801274	BAFF-R on B cell	1	161479745
ebi-a-GCST90001829	rs149153670	BAFF-R on B cell	2	142280835
ebi-a-GCST90001829	rs2925323	BAFF-R on B cell	3	101658926
ebi-a-GCST90001829	rs116470335	BAFF-R on B cell	4	24492147
ebi-a-GCST90001829	rs12529650	BAFF-R on B cell	6	1751206
ebi-a-GCST90001829	rs56020760	BAFF-R on B cell	10	72391681
ebi-a-GCST90001829	rs1563991	BAFF-R on B cell	11	37670518
ebi-a-GCST90001829	rs143106266	BAFF-R on B cell	13	29908421
ebi-a-GCST90001829	rs148563640	BAFF-R on B cell	17	48145906
ebi-a-GCST90001829	rs5757678	BAFF-R on B cell	22	39843409
ebi-a-GCST90001829	rs1894588	BAFF-R on B cell	22	40629980
ebi-a-GCST90001829	rs149806587	BAFF-R on B cell	22	40774205
ebi-a-GCST90001829	rs73165129	BAFF-R on B cell	22	42315371
ebi-a-GCST90001829	rs149398740	BAFF-R on B cell	22	23924845
ebi-a-GCST90001829	rs117430146	BAFF-R on B cell	22	41998660
ebi-a-GCST90001829	rs76146864	BAFF-R on B cell	22	40246055
ebi-a-GCST90001829	rs1081003	BAFF-R on B cell	22	42525756
ebi-a-GCST90001829	rs117705111	BAFF-R on B cell	22	41957510
ebi-a-GCST90001829	rs5759079	BAFF-R on B cell	22	43393437
ebi-a-GCST90001830	rs2066978	BAFF-R on CD20-	1	7928759
ebi-a-GCST90001830	rs35368132	BAFF-R on CD20-	2	183179545
ebi-a-GCST90001830	rs72683736	BAFF-R on CD20-	4	162422182
ebi-a-GCST90001830	rs57396181	BAFF-R on CD20-	6	155520414
ebi-a-GCST90001830	rs7826226	BAFF-R on CD20-	8	61322563
ebi-a-GCST90001830	rs79373846	BAFF-R on CD20-	8	127988956
ebi-a-GCST90001830	rs182211529	BAFF-R on CD20-	11	3574110
ebi-a-GCST90001830	rs140785480	BAFF-R on CD20-	13	61781933
ebi-a-GCST90001830	rs145320860	BAFF-R on CD20-	13	62765406
ebi-a-GCST90001830	rs73346463	BAFF-R on CD20-	14	100063947
ebi-a-GCST90001830	rs142808203	BAFF-R on CD20-	17	53123747
ebi-a-GCST90001830	rs5996087	BAFF-R on CD20-	22	42321591
ebi-a-GCST90001830	rs149192849	BAFF-R on CD20-	22	42270864
ebi-a-GCST90001830	rs191405504	BAFF-R on CD20-	22	41305796
ebi-a-GCST90001831	rs12724134	CD62L on CD62L+	1	95075566
ebi-a-GCST90001831	rs2990641	CD62L on CD62L+	1	170237066
ebi-a-GCST90001831	rs143375069	CD62L on CD62L+	1	157915841
ebi-a-GCST90001831	rs12075684	CD62L on CD62L+	1	169620673
ebi-a-GCST90001831	rs6427213	CD62L on CD62L+	1	169713146
ebi-a-GCST90001831	rs76305105	CD62L on CD62L+	1	168573042
ebi-a-GCST90001831	rs75145851	CD62L on CD62L+	2	96804588
ebi-a-GCST90001831	rs114141182	CD62L on CD62L+	6	13173850
ebi-a-GCST90001831	rs2536092	CD62L on CD62L+	7	151378090
ebi-a-GCST90001831	rs6539095	CD62L on CD62L+	12	104092085
ebi-a-GCST90001831	rs2038655	CD62L on CD62L+	13	26228943

ebi-a-GCST90001831	rs58315932	CD62L on CD62L+	15	79777547
ebi-a-GCST90001831	rs7202624	CD62L on CD62L+	16	86016175
ebi-a-GCST90001831	rs9890410	CD62L on CD62L+	17	16279572
ebi-a-GCST90001831	rs7234534	CD62L on CD62L+	18	23407436
ebi-a-GCST90001832	rs4987358	CD62L on CD62L+	1	169665551
ebi-a-GCST90001832	rs343813	CD62L on CD62L+	1	111334781
ebi-a-GCST90001832	rs3917761	CD62L on CD62L+	1	169574729
ebi-a-GCST90001832	rs12040885	CD62L on CD62L+	1	167057553
ebi-a-GCST90001832	rs140935876	CD62L on CD62L+	2	50722912
ebi-a-GCST90001832	rs62236308	CD62L on CD62L+	3	31286954
ebi-a-GCST90001832	rs148535663	CD62L on CD62L+	3	125563415
ebi-a-GCST90001832	rs17058091	CD62L on CD62L+	6	98341767
ebi-a-GCST90001832	rs4731265	CD62L on CD62L+	7	125443474
ebi-a-GCST90001832	rs11773763	CD62L on CD62L+	7	50271499
ebi-a-GCST90001832	rs140893422	CD62L on CD62L+	9	131575934
ebi-a-GCST90001832	rs111528892	CD62L on CD62L+	13	27228287
ebi-a-GCST90001832	rs12864970	CD62L on CD62L+	13	26218284
ebi-a-GCST90001832	rs149154085	CD62L on CD62L+	14	79979025
ebi-a-GCST90001832	rs613872	CD62L on CD62L+	18	53210302
ebi-a-GCST90001832	rs150038452	CD62L on CD62L+	21	27810403
ebi-a-GCST90001833	rs12041883	CD62L on CD62L+	1	111320129
ebi-a-GCST90001833	rs742800	CD62L on CD62L+	1	29718191
ebi-a-GCST90001833	rs3917768	CD62L on CD62L+	1	169572947
ebi-a-GCST90001833	rs12040885	CD62L on CD62L+	1	167057553
ebi-a-GCST90001833	rs7526645	CD62L on CD62L+	1	169728916
ebi-a-GCST90001833	rs11692722	CD62L on CD62L+	2	23715189
ebi-a-GCST90001833	rs75943000	CD62L on CD62L+	3	56956793
ebi-a-GCST90001833	rs62236308	CD62L on CD62L+	3	31286954
ebi-a-GCST90001833	rs113947601	CD62L on CD62L+	3	32267533
ebi-a-GCST90001833	rs140597204	CD62L on CD62L+	4	43926627
ebi-a-GCST90001833	rs112295585	CD62L on CD62L+	4	55258914
ebi-a-GCST90001833	rs115980202	CD62L on CD62L+	4	42489975
ebi-a-GCST90001833	rs4731265	CD62L on CD62L+	7	125443474
ebi-a-GCST90001833	rs11773763	CD62L on CD62L+	7	50271499
ebi-a-GCST90001833	rs10435954	CD62L on CD62L+	9	90196882
ebi-a-GCST90001833	rs2428305	CD62L on CD62L+	10	24651970
ebi-a-GCST90001833	rs72997833	CD62L on CD62L+	11	108363562
ebi-a-GCST90001833	rs11029935	CD62L on CD62L+	11	27337967
ebi-a-GCST90001833	rs703648	CD62L on CD62L+	12	104080975
ebi-a-GCST90001833	rs35443755	CD62L on CD62L+	12	20469483
ebi-a-GCST90001833	rs12864970	CD62L on CD62L+	13	26218284
ebi-a-GCST90001833	rs58315932	CD62L on CD62L+	15	79777547
ebi-a-GCST90001833	rs149982484	CD62L on CD62L+	20	48349220
ebi-a-GCST90001833	rs75307154	CD62L on CD62L+	20	17824351
ebi-a-GCST90001833	rs150038452	CD62L on CD62L+	21	27810403
ebi-a-GCST90001833	rs133635	CD62L on CD62L+	22	48708374
ebi-a-GCST90001834	rs4987369	CD62L on monocyt	1	169662742
ebi-a-GCST90001834	rs7540208	CD62L on monocyt	1	104331283

ebi-a-GCST90001834	rs4987389	CD62L on monocyt	1	169658509
ebi-a-GCST90001834	rs235869	CD62L on monocyt	1	171610959
ebi-a-GCST90001834	rs10127711	CD62L on monocyt	1	168483806
ebi-a-GCST90001834	rs7516777	CD62L on monocyt	1	199788171
ebi-a-GCST90001834	rs2404215	CD62L on monocyt	1	53634228
ebi-a-GCST90001834	rs6694645	CD62L on monocyt	1	95111100
ebi-a-GCST90001834	rs73879235	CD62L on monocyt	3	170626704
ebi-a-GCST90001834	rs169046	CD62L on monocyt	3	38417367
ebi-a-GCST90001834	rs34142414	CD62L on monocyt	3	106555382
ebi-a-GCST90001834	rs447362	CD62L on monocyt	5	165250043
ebi-a-GCST90001834	rs12215799	CD62L on monocyt	6	164104976
ebi-a-GCST90001834	rs184665042	CD62L on monocyt	6	42298065
ebi-a-GCST90001834	rs79734694	CD62L on monocyt	8	68787748
ebi-a-GCST90001834	rs77224604	CD62L on monocyt	8	137328288
ebi-a-GCST90001834	rs118080101	CD62L on monocyt	9	138899879
ebi-a-GCST90001834	rs117965901	CD62L on monocyt	10	30770530
ebi-a-GCST90001834	rs11597201	CD62L on monocyt	10	88469837
ebi-a-GCST90001834	rs189683893	CD62L on monocyt	10	89153882
ebi-a-GCST90001834	rs7900893	CD62L on monocyt	10	130132360
ebi-a-GCST90001834	rs11225485	CD62L on monocyt	11	102807731
ebi-a-GCST90001834	rs10842393	CD62L on monocyt	12	24831709
ebi-a-GCST90001834	rs73462775	CD62L on monocyt	15	95884078
ebi-a-GCST90001834	rs55633105	CD62L on monocyt	17	72159072
ebi-a-GCST90001834	rs141452540	CD62L on monocyt	20	47237403
ebi-a-GCST90001835	rs145336413	CD62L on granuloc	1	66609144
ebi-a-GCST90001835	rs295137	CD62L on granuloc	2	201150040
ebi-a-GCST90001835	rs9845059	CD62L on granuloc	3	35308691
ebi-a-GCST90001835	rs7691737	CD62L on granuloc	4	108522779
ebi-a-GCST90001835	rs140597204	CD62L on granuloc	4	43926627
ebi-a-GCST90001835	rs142693862	CD62L on granuloc	4	154572780
ebi-a-GCST90001835	rs12656231	CD62L on granuloc	5	8150877
ebi-a-GCST90001835	rs2260907	CD62L on granuloc	5	134616218
ebi-a-GCST90001835	rs1821205	CD62L on granuloc	5	57127877
ebi-a-GCST90001835	rs17282486	CD62L on granuloc	6	108320502
ebi-a-GCST90001835	rs445	CD62L on granuloc	7	92408370
ebi-a-GCST90001835	rs7863065	CD62L on granuloc	9	16246539
ebi-a-GCST90001835	rs674218	CD62L on granuloc	11	126109060
ebi-a-GCST90001835	rs138669346	CD62L on granuloc	13	24958892
ebi-a-GCST90001835	rs13336680	CD62L on granuloc	16	5615903
ebi-a-GCST90001835	rs3826331	CD62L on granuloc	17	38150492
ebi-a-GCST90001835	rs148133987	CD62L on granuloc	20	56356566
ebi-a-GCST90001835	rs62243093	CD62L on granuloc	22	35377900
ebi-a-GCST90001836	rs149073662	CD66b on CD66b+	1	9801707
ebi-a-GCST90001836	rs13006137	CD66b on CD66b+	2	154986727
ebi-a-GCST90001836	rs139348851	CD66b on CD66b+	3	50280843
ebi-a-GCST90001836	rs11167675	CD66b on CD66b+	5	153735817
ebi-a-GCST90001836	rs306580	CD66b on CD66b+	5	94060445
ebi-a-GCST90001836	rs4361470	CD66b on CD66b+	5	55418144

ebi-a-GCST90001836	rs60449345	CD66b on CD66b+	5	162617092
ebi-a-GCST90001836	rs145836694	CD66b on CD66b+	6	106222810
ebi-a-GCST90001836	rs12200259	CD66b on CD66b+	6	12516242
ebi-a-GCST90001836	rs77955327	CD66b on CD66b+	6	39069699
ebi-a-GCST90001836	rs117312711	CD66b on CD66b+	7	112322389
ebi-a-GCST90001836	rs79450248	CD66b on CD66b+	9	19369066
ebi-a-GCST90001836	rs77600362	CD66b on CD66b+	11	6574904
ebi-a-GCST90001836	rs12146661	CD66b on CD66b+	11	132240457
ebi-a-GCST90001836	rs149541654	CD66b on CD66b+	14	64186810
ebi-a-GCST90001836	rs12587697	CD66b on CD66b+	14	20470737
ebi-a-GCST90001836	rs72696815	CD66b on CD66b+	14	95115530
ebi-a-GCST90001836	rs11632595	CD66b on CD66b+	15	24995686
ebi-a-GCST90001836	rs12102694	CD66b on CD66b+	16	76417625
ebi-a-GCST90001836	rs78927876	CD66b on CD66b+	17	78878134
ebi-a-GCST90001836	rs116864786	CD66b on CD66b+	18	39475934
ebi-a-GCST90001836	rs2160808	CD66b on CD66b+	18	28147641
ebi-a-GCST90001836	rs4801859	CD66b on CD66b+	19	39221947
ebi-a-GCST90001836	rs10420273	CD66b on CD66b+	19	15177126
ebi-a-GCST90001836	rs62206422	CD66b on CD66b+	20	36239708
ebi-a-GCST90001836	rs73195476	CD66b on CD66b+	21	33469682
ebi-a-GCST90001836	rs8143049	CD66b on CD66b+	22	43455246
ebi-a-GCST90001837	rs74149325	CD66b on Gr MDS	1	240277599
ebi-a-GCST90001837	rs320439	CD66b on Gr MDS	1	213629078
ebi-a-GCST90001837	rs10864377	CD66b on Gr MDS	1	9066695
ebi-a-GCST90001837	rs56190007	CD66b on Gr MDS	2	62196278
ebi-a-GCST90001837	rs12496184	CD66b on Gr MDS	3	32299237
ebi-a-GCST90001837	rs1404536	CD66b on Gr MDS	3	65517450
ebi-a-GCST90001837	rs139608920	CD66b on Gr MDS	5	138031822
ebi-a-GCST90001837	rs181426303	CD66b on Gr MDS	5	141954297
ebi-a-GCST90001837	rs13189938	CD66b on Gr MDS	5	60852845
ebi-a-GCST90001837	rs9296300	CD66b on Gr MDS	6	39458150
ebi-a-GCST90001837	rs62442507	CD66b on Gr MDS	7	2279645
ebi-a-GCST90001837	rs61194862	CD66b on Gr MDS	8	97223147
ebi-a-GCST90001837	rs11136666	CD66b on Gr MDS	8	3663762
ebi-a-GCST90001837	rs35066493	CD66b on Gr MDS	9	86717077
ebi-a-GCST90001837	rs2724786	CD66b on Gr MDS	10	12451399
ebi-a-GCST90001837	rs7135763	CD66b on Gr MDS	12	32521809
ebi-a-GCST90001837	rs6489371	CD66b on Gr MDS	12	2563657
ebi-a-GCST90001837	rs62025068	CD66b on Gr MDS	15	91189400
ebi-a-GCST90001837	rs111896566	CD66b on Gr MDS	16	20551336
ebi-a-GCST90001837	rs113799614	CD66b on Gr MDS	17	79158110
ebi-a-GCST90001837	rs2160808	CD66b on Gr MDS	18	28147641
ebi-a-GCST90001837	rs8133479	CD66b on Gr MDS	21	18668216
ebi-a-GCST90001838	rs4657430	CD3 on naive CD8l	1	165238114
ebi-a-GCST90001838	rs7416513	CD3 on naive CD8l	1	26647949
ebi-a-GCST90001838	rs4396110	CD3 on naive CD8l	1	118266529
ebi-a-GCST90001838	rs61814886	CD3 on naive CD8l	1	167136405
ebi-a-GCST90001838	rs4657649	CD3 on naive CD8l	1	167173140

ebi-a-GCST90001838	rs78132817	CD3 on naive CD8l	1	165191695
ebi-a-GCST90001838	rs4657706	CD3 on naive CD8l	1	167728447
ebi-a-GCST90001838	rs2995089	CD3 on naive CD8l	1	167431193
ebi-a-GCST90001838	rs12994487	CD3 on naive CD8l	2	45448525
ebi-a-GCST90001838	rs148965436	CD3 on naive CD8l	4	53322015
ebi-a-GCST90001838	rs115280990	CD3 on naive CD8l	4	73709210
ebi-a-GCST90001838	rs112171230	CD3 on naive CD8l	7	70434581
ebi-a-GCST90001838	rs28906376	CD3 on naive CD8l	8	89116077
ebi-a-GCST90001838	rs6479155	CD3 on naive CD8l	9	105868652
ebi-a-GCST90001838	rs74744824	CD3 on naive CD8l	9	22044904
ebi-a-GCST90001838	rs111780443	CD3 on naive CD8l	11	132210362
ebi-a-GCST90001838	rs118065562	CD3 on naive CD8l	12	2419083
ebi-a-GCST90001838	rs4646296	CD3 on naive CD8l	14	70243505
ebi-a-GCST90001838	rs75624489	CD3 on naive CD8l	14	52876003
ebi-a-GCST90001838	rs12921174	CD3 on naive CD8l	16	430304
ebi-a-GCST90001838	rs62028293	CD3 on naive CD8l	16	50264054
ebi-a-GCST90001838	rs2714019	CD3 on naive CD8l	17	70789650
ebi-a-GCST90001838	rs16940758	CD3 on naive CD8l	17	44040626
ebi-a-GCST90001838	rs66774667	CD3 on naive CD8l	20	60172299
ebi-a-GCST90001838	rs2413113	CD3 on naive CD8l	22	32779227
ebi-a-GCST90001839	rs2480679	CD3 on EM CD8br	1	167407517
ebi-a-GCST90001839	rs858545	CD3 on EM CD8br	1	167461391
ebi-a-GCST90001839	rs2988279	CD3 on EM CD8br	1	167419839
ebi-a-GCST90001839	rs17879522	CD3 on EM CD8br	2	215812660
ebi-a-GCST90001839	rs115636363	CD3 on EM CD8br	4	34352645
ebi-a-GCST90001839	rs139305287	CD3 on EM CD8br	5	166579156
ebi-a-GCST90001839	rs7782316	CD3 on EM CD8br	7	5305788
ebi-a-GCST90001839	rs817854	CD3 on EM CD8br	9	110113919
ebi-a-GCST90001839	rs117820643	CD3 on EM CD8br	10	480161
ebi-a-GCST90001839	rs10838150	CD3 on EM CD8br	11	5483133
ebi-a-GCST90001839	rs327018	CD3 on EM CD8br	11	20849758
ebi-a-GCST90001839	rs10893076	CD3 on EM CD8br	11	123569986
ebi-a-GCST90001839	rs7300908	CD3 on EM CD8br	12	132405421
ebi-a-GCST90001839	rs516590	CD3 on EM CD8br	12	65100884
ebi-a-GCST90001839	rs10848778	CD3 on EM CD8br	12	3170906
ebi-a-GCST90001839	rs10778410	CD3 on EM CD8br	12	105926700
ebi-a-GCST90001839	rs62029957	CD3 on EM CD8br	16	50213168
ebi-a-GCST90001839	rs12918439	CD3 on EM CD8br	16	50674103
ebi-a-GCST90001839	rs117524375	CD3 on EM CD8br	16	1790546
ebi-a-GCST90001839	rs17664049	CD3 on EM CD8br	18	48685445
ebi-a-GCST90001840	rs2949669	CD3 on TD CD8br	1	167417884
ebi-a-GCST90001840	rs472594	CD3 on TD CD8br	1	228601638
ebi-a-GCST90001840	rs114241106	CD3 on TD CD8br	2	47600591
ebi-a-GCST90001840	rs11678793	CD3 on TD CD8br	2	241562105
ebi-a-GCST90001840	rs116008125	CD3 on TD CD8br	2	229111257
ebi-a-GCST90001840	rs6419642	CD3 on TD CD8br	2	68100469
ebi-a-GCST90001840	rs141566851	CD3 on TD CD8br	3	170163167
ebi-a-GCST90001840	rs115208823	CD3 on TD CD8br	3	101677297

ebi-a-GCST90001840	rs4689428	CD3 on TD CD8br	4	6388191
ebi-a-GCST90001840	rs74596366	CD3 on TD CD8br	4	1635134
ebi-a-GCST90001840	rs147934227	CD3 on TD CD8br	4	144501508
ebi-a-GCST90001840	rs72785901	CD3 on TD CD8br	5	120282213
ebi-a-GCST90001840	rs57740770	CD3 on TD CD8br	6	32067435
ebi-a-GCST90001840	rs146793472	CD3 on TD CD8br	6	115127012
ebi-a-GCST90001840	rs77727789	CD3 on TD CD8br	9	116135431
ebi-a-GCST90001840	rs7905582	CD3 on TD CD8br	10	133327489
ebi-a-GCST90001840	rs6584354	CD3 on TD CD8br	10	102007714
ebi-a-GCST90001840	rs10860204	CD3 on TD CD8br	12	97805978
ebi-a-GCST90001840	rs73158979	CD3 on TD CD8br	12	132107081
ebi-a-GCST90001840	rs111666485	CD3 on TD CD8br	12	15638143
ebi-a-GCST90001840	rs945668	CD3 on TD CD8br	13	23675156
ebi-a-GCST90001840	rs55823840	CD3 on TD CD8br	14	21114258
ebi-a-GCST90001840	rs182175045	CD3 on TD CD8br	17	276230
ebi-a-GCST90001840	rs117286362	CD3 on TD CD8br	19	31209621
ebi-a-GCST90001841	rs61814872	CD3 on CM CD4+	1	167113055
ebi-a-GCST90001841	rs4396110	CD3 on CM CD4+	1	118266529
ebi-a-GCST90001841	rs3753932	CD3 on CM CD4+	1	167725004
ebi-a-GCST90001841	rs67402791	CD3 on CM CD4+	1	26645970
ebi-a-GCST90001841	rs2949661	CD3 on CM CD4+	1	167424924
ebi-a-GCST90001841	rs146780525	CD3 on CM CD4+	1	26780027
ebi-a-GCST90001841	rs1317631	CD3 on CM CD4+	1	165904360
ebi-a-GCST90001841	rs4657649	CD3 on CM CD4+	1	167173140
ebi-a-GCST90001841	rs6751481	CD3 on CM CD4+	2	38897810
ebi-a-GCST90001841	rs11715223	CD3 on CM CD4+	3	64581733
ebi-a-GCST90001841	rs12497487	CD3 on CM CD4+	3	78194307
ebi-a-GCST90001841	rs57319220	CD3 on CM CD4+	3	45919992
ebi-a-GCST90001841	rs72666260	CD3 on CM CD4+	4	113334844
ebi-a-GCST90001841	rs188669685	CD3 on CM CD4+	5	2669391
ebi-a-GCST90001841	rs117466579	CD3 on CM CD4+	7	85315630
ebi-a-GCST90001841	rs144834954	CD3 on CM CD4+	8	109416365
ebi-a-GCST90001841	rs34201586	CD3 on CM CD4+	9	110688268
ebi-a-GCST90001841	rs74744824	CD3 on CM CD4+	9	22044904
ebi-a-GCST90001841	rs118065562	CD3 on CM CD4+	12	2419083
ebi-a-GCST90001841	rs28649534	CD3 on CM CD4+	13	48439005
ebi-a-GCST90001841	rs12884372	CD3 on CM CD4+	14	86036273
ebi-a-GCST90001841	rs117171372	CD3 on CM CD4+	15	27777649
ebi-a-GCST90001841	rs59302791	CD3 on CM CD4+	16	17996315
ebi-a-GCST90001841	rs62035375	CD3 on CM CD4+	16	2872116
ebi-a-GCST90001841	rs9925166	CD3 on CM CD4+	16	50221264
ebi-a-GCST90001841	rs141467190	CD3 on CM CD4+	17	41589741
ebi-a-GCST90001841	rs3809841	CD3 on CM CD4+	17	6024786
ebi-a-GCST90001841	rs62232208	CD3 on CM CD4+	22	45187219
ebi-a-GCST90001842	rs4657649	CD3 on Naive CD4	1	167173140
ebi-a-GCST90001842	rs2949661	CD3 on Naive CD4	1	167424924
ebi-a-GCST90001842	rs4396110	CD3 on Naive CD4	1	118266529
ebi-a-GCST90001842	rs4925475	CD3 on Naive CD4	1	229528427

ebi-a-GCST90001842	rs146330992	CD3 on Naive CD4	1	165888466
ebi-a-GCST90001842	rs61814872	CD3 on Naive CD4	1	167113055
ebi-a-GCST90001842	rs7530127	CD3 on Naive CD4	1	165671820
ebi-a-GCST90001842	rs76066554	CD3 on Naive CD4	1	95285730
ebi-a-GCST90001842	rs139257983	CD3 on Naive CD4	2	56038405
ebi-a-GCST90001842	rs28607988	CD3 on Naive CD4	3	45913338
ebi-a-GCST90001842	rs11715223	CD3 on Naive CD4	3	64581733
ebi-a-GCST90001842	rs28535434	CD3 on Naive CD4	4	183742136
ebi-a-GCST90001842	rs12502302	CD3 on Naive CD4	4	186735780
ebi-a-GCST90001842	rs113731203	CD3 on Naive CD4	6	64965028
ebi-a-GCST90001842	rs62465786	CD3 on Naive CD4	7	51722488
ebi-a-GCST90001842	rs112171230	CD3 on Naive CD4	7	70434581
ebi-a-GCST90001842	rs34064330	CD3 on Naive CD4	7	48898926
ebi-a-GCST90001842	rs74744824	CD3 on Naive CD4	9	22044904
ebi-a-GCST90001842	rs146993166	CD3 on Naive CD4	10	82439265
ebi-a-GCST90001842	rs141784254	CD3 on Naive CD4	11	120364753
ebi-a-GCST90001842	rs74620952	CD3 on Naive CD4	11	117753136
ebi-a-GCST90001842	rs118065562	CD3 on Naive CD4	12	2419083
ebi-a-GCST90001842	rs9318267	CD3 on Naive CD4	13	75011432
ebi-a-GCST90001842	rs2003830	CD3 on Naive CD4	16	430469
ebi-a-GCST90001842	rs62035375	CD3 on Naive CD4	16	2872116
ebi-a-GCST90001842	rs7502332	CD3 on Naive CD4	17	70790532
ebi-a-GCST90001842	rs113208949	CD3 on Naive CD4	20	9979604
ebi-a-GCST90001842	rs2413113	CD3 on Naive CD4	22	32779227
ebi-a-GCST90001842	rs2213429	CD3 on Naive CD4	22	37217172
ebi-a-GCST90001842	rs62232208	CD3 on Naive CD4	22	45187219
ebi-a-GCST90001843	rs74139031	CD3 on EM CD4+	1	213852620
ebi-a-GCST90001843	rs6684311	CD3 on EM CD4+	1	166485891
ebi-a-GCST90001843	rs150528455	CD3 on EM CD4+	1	26740555
ebi-a-GCST90001843	rs2995089	CD3 on EM CD4+	1	167431193
ebi-a-GCST90001843	rs4656440	CD3 on EM CD4+	1	165218634
ebi-a-GCST90001843	rs4657649	CD3 on EM CD4+	1	167173140
ebi-a-GCST90001843	rs7519927	CD3 on EM CD4+	1	167100361
ebi-a-GCST90001843	rs4514905	CD3 on EM CD4+	2	3469702
ebi-a-GCST90001843	rs7595804	CD3 on EM CD4+	2	8424313
ebi-a-GCST90001843	rs727908	CD3 on EM CD4+	3	64819050
ebi-a-GCST90001843	rs2244434	CD3 on EM CD4+	3	158948969
ebi-a-GCST90001843	rs115265205	CD3 on EM CD4+	4	63231400
ebi-a-GCST90001843	rs72940579	CD3 on EM CD4+	11	66182450
ebi-a-GCST90001843	rs4288797	CD3 on EM CD4+	12	20796638
ebi-a-GCST90001843	rs28649534	CD3 on EM CD4+	13	48439005
ebi-a-GCST90001843	rs62028293	CD3 on EM CD4+	16	50264054
ebi-a-GCST90001843	rs62046402	CD3 on EM CD4+	16	18003476
ebi-a-GCST90001843	rs73068237	CD3 on EM CD4+	20	4574025
ebi-a-GCST90001843	rs226503	CD3 on EM CD4+	22	45705241
ebi-a-GCST90001844	rs61814872	CD3 on TD CD4+	1	167113055
ebi-a-GCST90001844	rs4657649	CD3 on TD CD4+	1	167173140
ebi-a-GCST90001844	rs1723018	CD3 on TD CD4+	1	167433420

ebi-a-GCST90001844	rs141365483	CD3 on TD CD4+	1	215951513
ebi-a-GCST90001844	rs34375977	CD3 on TD CD4+	1	17973179
ebi-a-GCST90001844	rs17779587	CD3 on TD CD4+	2	196205940
ebi-a-GCST90001844	rs142679567	CD3 on TD CD4+	2	173083726
ebi-a-GCST90001844	rs11124860	CD3 on TD CD4+	2	42337670
ebi-a-GCST90001844	rs116132148	CD3 on TD CD4+	2	188910093
ebi-a-GCST90001844	rs4973976	CD3 on TD CD4+	3	41638984
ebi-a-GCST90001844	rs7686404	CD3 on TD CD4+	4	110748461
ebi-a-GCST90001844	rs992038	CD3 on TD CD4+	4	70674836
ebi-a-GCST90001844	rs6535624	CD3 on TD CD4+	4	149587905
ebi-a-GCST90001844	rs114070694	CD3 on TD CD4+	5	133183956
ebi-a-GCST90001844	rs75334371	CD3 on TD CD4+	5	19788744
ebi-a-GCST90001844	rs62475031	CD3 on TD CD4+	7	76379476
ebi-a-GCST90001844	rs2719321	CD3 on TD CD4+	8	34186859
ebi-a-GCST90001844	rs6982159	CD3 on TD CD4+	8	33292469
ebi-a-GCST90001844	rs59977015	CD3 on TD CD4+	9	133301395
ebi-a-GCST90001844	rs149122933	CD3 on TD CD4+	9	115604900
ebi-a-GCST90001844	rs139058742	CD3 on TD CD4+	9	2784214
ebi-a-GCST90001844	rs72993540	CD3 on TD CD4+	11	108746675
ebi-a-GCST90001844	rs66621175	CD3 on TD CD4+	12	6965805
ebi-a-GCST90001844	rs12932298	CD3 on TD CD4+	16	17841327
ebi-a-GCST90001844	rs3922850	CD3 on TD CD4+	16	81955238
ebi-a-GCST90001844	rs141595889	CD3 on TD CD4+	18	25077062
ebi-a-GCST90001844	rs71339188	CD3 on TD CD4+	19	3484982
ebi-a-GCST90001845	rs4657649	CD3 on CD45RA-	1	167173140
ebi-a-GCST90001845	rs7536105	CD3 on CD45RA-	1	117818054
ebi-a-GCST90001845	rs4396110	CD3 on CD45RA-	1	118266529
ebi-a-GCST90001845	rs1317631	CD3 on CD45RA-	1	165904360
ebi-a-GCST90001845	rs2949661	CD3 on CD45RA-	1	167424924
ebi-a-GCST90001845	rs61814872	CD3 on CD45RA-	1	167113055
ebi-a-GCST90001845	rs12141563	CD3 on CD45RA-	1	26589636
ebi-a-GCST90001845	rs7595804	CD3 on CD45RA-	2	8424313
ebi-a-GCST90001845	rs12497487	CD3 on CD45RA-	3	78194307
ebi-a-GCST90001845	rs138838112	CD3 on CD45RA-	3	31848405
ebi-a-GCST90001845	rs57319220	CD3 on CD45RA-	3	45919992
ebi-a-GCST90001845	rs1077544	CD3 on CD45RA-	3	158945280
ebi-a-GCST90001845	rs11715223	CD3 on CD45RA-	3	64581733
ebi-a-GCST90001845	rs188669685	CD3 on CD45RA-	5	2669391
ebi-a-GCST90001845	rs62423936	CD3 on CD45RA-	6	168668486
ebi-a-GCST90001845	rs117466579	CD3 on CD45RA-	7	85315630
ebi-a-GCST90001845	rs34201586	CD3 on CD45RA-	9	110688268
ebi-a-GCST90001845	rs11015146	CD3 on CD45RA-	10	26804638
ebi-a-GCST90001845	rs72940579	CD3 on CD45RA-	11	66182450
ebi-a-GCST90001845	rs138909872	CD3 on CD45RA-	12	64916291
ebi-a-GCST90001845	rs28649534	CD3 on CD45RA-	13	48439005
ebi-a-GCST90001845	rs12884372	CD3 on CD45RA-	14	86036273
ebi-a-GCST90001845	rs117171372	CD3 on CD45RA-	15	27777649
ebi-a-GCST90001845	rs72732798	CD3 on CD45RA-	15	54670902

ebi-a-GCST90001845	rs59302791	CD3 on CD45RA- (	16	17996315
ebi-a-GCST90001845	rs9925166	CD3 on CD45RA- (	16	50221264
ebi-a-GCST90001845	rs148932595	CD3 on CD45RA- (	16	4725694
ebi-a-GCST90001845	rs3809841	CD3 on CD45RA- (	17	6024786
ebi-a-GCST90001845	rs141467190	CD3 on CD45RA- (	17	41589741
ebi-a-GCST90001845	rs62232208	CD3 on CD45RA- (	22	45187219
ebi-a-GCST90001846	rs858545	CD3 on CM CD8br	1	167461391
ebi-a-GCST90001846	rs116757920	CD3 on CM CD8br	1	117441813
ebi-a-GCST90001846	rs1723018	CD3 on CM CD8br	1	167433420
ebi-a-GCST90001846	rs61814886	CD3 on CM CD8br	1	167136405
ebi-a-GCST90001846	rs111281575	CD3 on CM CD8br	1	166737851
ebi-a-GCST90001846	rs12133696	CD3 on CM CD8br	1	165062200
ebi-a-GCST90001846	rs141851092	CD3 on CM CD8br	4	178824850
ebi-a-GCST90001846	rs192330157	CD3 on CM CD8br	4	161357515
ebi-a-GCST90001846	rs7710248	CD3 on CM CD8br	5	178194669
ebi-a-GCST90001846	rs34597764	CD3 on CM CD8br	10	97262994
ebi-a-GCST90001846	rs2156444	CD3 on CM CD8br	11	123175580
ebi-a-GCST90001846	rs146936815	CD3 on CM CD8br	11	67489988
ebi-a-GCST90001846	rs79117150	CD3 on CM CD8br	14	100649692
ebi-a-GCST90001846	rs8024202	CD3 on CM CD8br	15	97334659
ebi-a-GCST90001846	rs62029957	CD3 on CM CD8br	16	50213168
ebi-a-GCST90001846	rs3809841	CD3 on CM CD8br	17	6024786
ebi-a-GCST90001846	rs59436607	CD3 on CM CD8br	19	21356827
ebi-a-GCST90001846	rs12986020	CD3 on CM CD8br	19	36659629
ebi-a-GCST90001846	rs4814102	CD3 on CM CD8br	20	1291928
ebi-a-GCST90001847	rs1723018	CD3 on HLA DR+	1	167433420
ebi-a-GCST90001847	rs111281575	CD3 on HLA DR+	1	166737851
ebi-a-GCST90001847	rs12128267	CD3 on HLA DR+	1	222089002
ebi-a-GCST90001847	rs61814886	CD3 on HLA DR+	1	167136405
ebi-a-GCST90001847	rs953808	CD3 on HLA DR+	1	167403844
ebi-a-GCST90001847	rs74723288	CD3 on HLA DR+	2	106644972
ebi-a-GCST90001847	rs4668614	CD3 on HLA DR+	2	5593978
ebi-a-GCST90001847	rs435917	CD3 on HLA DR+	3	286552
ebi-a-GCST90001847	rs7708945	CD3 on HLA DR+	5	26402250
ebi-a-GCST90001847	rs152772	CD3 on HLA DR+	5	13129431
ebi-a-GCST90001847	rs181802824	CD3 on HLA DR+	5	40515928
ebi-a-GCST90001847	rs17159201	CD3 on HLA DR+	7	111686301
ebi-a-GCST90001847	rs78179244	CD3 on HLA DR+	8	5720711
ebi-a-GCST90001847	rs79022968	CD3 on HLA DR+	8	125638873
ebi-a-GCST90001847	rs1955077	CD3 on HLA DR+	8	118278954
ebi-a-GCST90001847	rs75535842	CD3 on HLA DR+	8	138415335
ebi-a-GCST90001847	rs17605113	CD3 on HLA DR+	10	37182452
ebi-a-GCST90001847	rs73167431	CD3 on HLA DR+	13	21343664
ebi-a-GCST90001847	rs118106483	CD3 on HLA DR+	13	22483321
ebi-a-GCST90001847	rs9318267	CD3 on HLA DR+	13	75011432
ebi-a-GCST90001847	rs28413717	CD3 on HLA DR+	14	94742681
ebi-a-GCST90001847	rs288415	CD3 on HLA DR+	15	98166209
ebi-a-GCST90001847	rs12596540	CD3 on HLA DR+	16	10970476

ebi-a-GCST90001847	rs4889018	CD3 on HLA DR+	16	79773291
ebi-a-GCST90001847	rs76073410	CD3 on HLA DR+	17	42400157
ebi-a-GCST90001847	rs73283178	CD3 on HLA DR+	17	1152909
ebi-a-GCST90001847	rs75407914	CD3 on HLA DR+	18	65188496
ebi-a-GCST90001847	rs2742060	CD3 on HLA DR+	19	55650687
ebi-a-GCST90001848	rs59533906	CD3 on NKT	1	199139803
ebi-a-GCST90001848	rs13009946	CD3 on NKT	2	219007752
ebi-a-GCST90001848	rs79439047	CD3 on NKT	2	109771818
ebi-a-GCST90001848	rs111570648	CD3 on NKT	3	90325908
ebi-a-GCST90001848	rs115485809	CD3 on NKT	4	174374287
ebi-a-GCST90001848	rs6928357	CD3 on NKT	6	100913553
ebi-a-GCST90001848	rs117913408	CD3 on NKT	6	102173117
ebi-a-GCST90001848	rs79086781	CD3 on NKT	6	100526652
ebi-a-GCST90001848	rs73429561	CD3 on NKT	7	122844882
ebi-a-GCST90001848	rs7798325	CD3 on NKT	7	62717148
ebi-a-GCST90001848	rs78369669	CD3 on NKT	11	93085233
ebi-a-GCST90001848	rs489642	CD3 on NKT	12	63783732
ebi-a-GCST90001848	rs59727684	CD3 on NKT	16	20090636
ebi-a-GCST90001848	rs61572747	CD3 on NKT	17	43460891
ebi-a-GCST90001848	rs7239036	CD3 on NKT	18	52411825
ebi-a-GCST90001848	rs2288522	CD3 on NKT	19	55708475
ebi-a-GCST90001848	rs13345102	CD3 on NKT	19	17535188
ebi-a-GCST90001849	rs145799392	CD3 on HLA DR+	1	30362673
ebi-a-GCST90001849	rs114102310	CD3 on HLA DR+	1	165023848
ebi-a-GCST90001849	rs10753692	CD3 on HLA DR+	1	165805459
ebi-a-GCST90001849	rs1723018	CD3 on HLA DR+	1	167433420
ebi-a-GCST90001849	rs6684311	CD3 on HLA DR+	1	166485891
ebi-a-GCST90001849	rs55952666	CD3 on HLA DR+	2	240269751
ebi-a-GCST90001849	rs10195369	CD3 on HLA DR+	2	3601711
ebi-a-GCST90001849	rs6765892	CD3 on HLA DR+	3	74263840
ebi-a-GCST90001849	rs13072593	CD3 on HLA DR+	3	150288489
ebi-a-GCST90001849	rs10933796	CD3 on HLA DR+	3	104667511
ebi-a-GCST90001849	rs27265	CD3 on HLA DR+	5	50161106
ebi-a-GCST90001849	rs13170201	CD3 on HLA DR+	5	30950823
ebi-a-GCST90001849	rs16867696	CD3 on HLA DR+	5	88794820
ebi-a-GCST90001849	rs188731571	CD3 on HLA DR+	7	25033008
ebi-a-GCST90001849	rs147209445	CD3 on HLA DR+	8	17728096
ebi-a-GCST90001849	rs75535842	CD3 on HLA DR+	8	138415335
ebi-a-GCST90001849	rs9408777	CD3 on HLA DR+	9	105794
ebi-a-GCST90001849	rs59738549	CD3 on HLA DR+	10	116394226
ebi-a-GCST90001849	rs11042750	CD3 on HLA DR+	11	10372959
ebi-a-GCST90001849	rs75771396	CD3 on HLA DR+	11	124869047
ebi-a-GCST90001849	rs73167431	CD3 on HLA DR+	13	21343664
ebi-a-GCST90001849	rs4787901	CD3 on HLA DR+	16	27027966
ebi-a-GCST90001849	rs113710453	CD3 on HLA DR+	16	89099756
ebi-a-GCST90001849	rs12596540	CD3 on HLA DR+	16	10970476
ebi-a-GCST90001849	rs62079658	CD3 on HLA DR+	17	41439784
ebi-a-GCST90001849	rs2010043	CD3 on HLA DR+	17	73735326

ebi-a-GCST90001849	rs2742060	CD3 on HLA DR+	19	55650687
ebi-a-GCST90001849	rs1529714	CD3 on HLA DR+	19	19793484
ebi-a-GCST90001850	rs1773542	CD3 on HLA DR+	1	167432949
ebi-a-GCST90001850	rs12141641	CD3 on HLA DR+	1	167408565
ebi-a-GCST90001850	rs2863272	CD3 on HLA DR+	1	167797924
ebi-a-GCST90001850	rs12032130	CD3 on HLA DR+	1	167156419
ebi-a-GCST90001850	rs56383740	CD3 on HLA DR+	1	29694550
ebi-a-GCST90001850	rs4668614	CD3 on HLA DR+	2	5593978
ebi-a-GCST90001850	rs4608913	CD3 on HLA DR+	5	131760418
ebi-a-GCST90001850	rs181802824	CD3 on HLA DR+	5	40515928
ebi-a-GCST90001850	rs864075	CD3 on HLA DR+	5	11004941
ebi-a-GCST90001850	rs2036009	CD3 on HLA DR+	5	26389125
ebi-a-GCST90001850	rs57740770	CD3 on HLA DR+	6	32067435
ebi-a-GCST90001850	rs7788673	CD3 on HLA DR+	7	79908084
ebi-a-GCST90001850	rs7782316	CD3 on HLA DR+	7	5305788
ebi-a-GCST90001850	rs4742559	CD3 on HLA DR+	9	9015895
ebi-a-GCST90001850	rs78539923	CD3 on HLA DR+	9	38673280
ebi-a-GCST90001850	rs11233873	CD3 on HLA DR+	11	83711222
ebi-a-GCST90001850	rs76442794	CD3 on HLA DR+	11	122175217
ebi-a-GCST90001850	rs11170960	CD3 on HLA DR+	12	54905021
ebi-a-GCST90001850	rs73167431	CD3 on HLA DR+	13	21343664
ebi-a-GCST90001850	rs11624277	CD3 on HLA DR+	14	38072374
ebi-a-GCST90001850	rs288415	CD3 on HLA DR+	15	98166209
ebi-a-GCST90001850	rs6498116	CD3 on HLA DR+	16	10969282
ebi-a-GCST90001850	rs8057798	CD3 on HLA DR+	16	25865790
ebi-a-GCST90001850	rs79048891	CD3 on HLA DR+	18	70950759
ebi-a-GCST90001850	rs112025123	CD3 on HLA DR+	18	46992743
ebi-a-GCST90001850	rs116911862	CD3 on HLA DR+	18	755834
ebi-a-GCST90001850	rs115825160	CD3 on HLA DR+	22	22817792
ebi-a-GCST90001851	rs4657649	CD3 on T cell	1	167173140
ebi-a-GCST90001851	rs1617988	CD3 on T cell	1	167436300
ebi-a-GCST90001851	rs114672530	CD3 on T cell	1	168306279
ebi-a-GCST90001851	rs148114970	CD3 on T cell	1	85390287
ebi-a-GCST90001851	rs12729972	CD3 on T cell	1	118256346
ebi-a-GCST90001851	rs10801951	CD3 on T cell	1	117781742
ebi-a-GCST90001851	rs6684311	CD3 on T cell	1	166485891
ebi-a-GCST90001851	rs75169391	CD3 on T cell	1	165284481
ebi-a-GCST90001851	rs116128731	CD3 on T cell	2	215390543
ebi-a-GCST90001851	rs75466245	CD3 on T cell	2	44973335
ebi-a-GCST90001851	rs11885872	CD3 on T cell	2	104570579
ebi-a-GCST90001851	rs2122146	CD3 on T cell	3	158951696
ebi-a-GCST90001851	rs72987628	CD3 on T cell	3	131994846
ebi-a-GCST90001851	rs6769221	CD3 on T cell	3	78188083
ebi-a-GCST90001851	rs41289614	CD3 on T cell	3	45972492
ebi-a-GCST90001851	rs4456952	CD3 on T cell	4	20480323
ebi-a-GCST90001851	rs144695228	CD3 on T cell	5	79114377
ebi-a-GCST90001851	rs185805208	CD3 on T cell	6	118163760
ebi-a-GCST90001851	rs35750035	CD3 on T cell	7	130765457

ebi-a-GCST90001851	rs61618798	CD3 on T cell	7	38070274
ebi-a-GCST90001851	rs28636970	CD3 on T cell	8	75889021
ebi-a-GCST90001851	rs6479155	CD3 on T cell	9	105868652
ebi-a-GCST90001851	rs10996702	CD3 on T cell	10	67597707
ebi-a-GCST90001851	rs113763504	CD3 on T cell	10	81110536
ebi-a-GCST90001851	rs59684757	CD3 on T cell	14	86146234
ebi-a-GCST90001851	rs12591149	CD3 on T cell	15	25176517
ebi-a-GCST90001851	rs17614424	CD3 on T cell	15	36139906
ebi-a-GCST90001851	rs2520957	CD3 on T cell	16	18293116
ebi-a-GCST90001851	rs55799444	CD3 on T cell	16	90107716
ebi-a-GCST90001851	rs62029957	CD3 on T cell	16	50213168
ebi-a-GCST90001851	rs141421236	CD3 on T cell	16	4780201
ebi-a-GCST90001851	rs1502523	CD3 on T cell	17	51481273
ebi-a-GCST90001851	rs71381322	CD3 on T cell	17	48900824
ebi-a-GCST90001851	rs1529714	CD3 on T cell	19	19793484
ebi-a-GCST90001852	rs704859	CD3 on CD39+ rest	1	167500815
ebi-a-GCST90001852	rs1723016	CD3 on CD39+ rest	1	167433056
ebi-a-GCST90001852	rs16859347	CD3 on CD39+ rest	1	167603757
ebi-a-GCST90001852	rs143008344	CD3 on CD39+ rest	3	64946405
ebi-a-GCST90001852	rs145172244	CD3 on CD39+ rest	4	172921509
ebi-a-GCST90001852	rs73001512	CD3 on CD39+ rest	4	174188120
ebi-a-GCST90001852	rs150879948	CD3 on CD39+ rest	6	90475932
ebi-a-GCST90001852	rs12215509	CD3 on CD39+ rest	6	16006964
ebi-a-GCST90001852	rs7741756	CD3 on CD39+ rest	6	118123272
ebi-a-GCST90001852	rs185763975	CD3 on CD39+ rest	7	30998786
ebi-a-GCST90001852	rs16921089	CD3 on CD39+ rest	9	104759303
ebi-a-GCST90001852	rs16929000	CD3 on CD39+ rest	9	9165321
ebi-a-GCST90001852	rs61874707	CD3 on CD39+ rest	10	121310166
ebi-a-GCST90001852	rs79844317	CD3 on CD39+ rest	10	100127250
ebi-a-GCST90001852	rs78574867	CD3 on CD39+ rest	11	133249133
ebi-a-GCST90001852	rs140395477	CD3 on CD39+ rest	13	79717709
ebi-a-GCST90001852	rs55799444	CD3 on CD39+ rest	16	90107716
ebi-a-GCST90001852	rs12449842	CD3 on CD39+ rest	17	48077390
ebi-a-GCST90001852	rs146187364	CD3 on CD39+ rest	19	10553511
ebi-a-GCST90001852	rs2832129	CD3 on CD39+ rest	21	30275535
ebi-a-GCST90001853	rs12138291	CD3 on activated T	1	166464476
ebi-a-GCST90001853	rs12401936	CD3 on activated T	1	163328756
ebi-a-GCST90001853	rs552999	CD3 on activated T	1	168700134
ebi-a-GCST90001853	rs4396110	CD3 on activated T	1	118266529
ebi-a-GCST90001853	rs35055340	CD3 on activated T	1	165314063
ebi-a-GCST90001853	rs72758215	CD3 on activated T	1	234250218
ebi-a-GCST90001853	rs2949661	CD3 on activated T	1	167424924
ebi-a-GCST90001853	rs114672530	CD3 on activated T	1	168306279
ebi-a-GCST90001853	rs4728966	CD3 on activated T	7	90981869
ebi-a-GCST90001853	rs78694250	CD3 on activated T	7	156067251
ebi-a-GCST90001853	rs148303369	CD3 on activated T	9	28514040
ebi-a-GCST90001853	rs141784254	CD3 on activated T	11	120364753
ebi-a-GCST90001853	rs79041458	CD3 on activated T	11	30021259

ebi-a-GCST90001853	rs4767280	CD3 on activated T	12	115363953
ebi-a-GCST90001853	rs55740714	CD3 on activated T	12	95185923
ebi-a-GCST90001853	rs59684757	CD3 on activated T	14	86146234
ebi-a-GCST90001853	rs78043002	CD3 on activated T	15	36111764
ebi-a-GCST90001853	rs8024202	CD3 on activated T	15	97334659
ebi-a-GCST90001853	rs59302791	CD3 on activated T	16	17996315
ebi-a-GCST90001853	rs77724325	CD3 on activated T	16	52834997
ebi-a-GCST90001853	rs138786273	CD3 on activated T	17	21944338
ebi-a-GCST90001853	rs142664794	CD3 on activated T	21	37681268
ebi-a-GCST90001854	rs12064796	CD3 on CD39+ acti	1	20141920
ebi-a-GCST90001854	rs12138291	CD3 on CD39+ acti	1	166464476
ebi-a-GCST90001854	rs35055340	CD3 on CD39+ acti	1	165314063
ebi-a-GCST90001854	rs552999	CD3 on CD39+ acti	1	168700134
ebi-a-GCST90001854	rs186524272	CD3 on CD39+ acti	1	167573289
ebi-a-GCST90001854	rs12401936	CD3 on CD39+ acti	1	163328756
ebi-a-GCST90001854	rs2949661	CD3 on CD39+ acti	1	167424924
ebi-a-GCST90001854	rs74425152	CD3 on CD39+ acti	2	241271589
ebi-a-GCST90001854	rs4685239	CD3 on CD39+ acti	3	15202886
ebi-a-GCST90001854	rs1389168	CD3 on CD39+ acti	4	108801664
ebi-a-GCST90001854	rs10491313	CD3 on CD39+ acti	5	141216310
ebi-a-GCST90001854	rs140156664	CD3 on CD39+ acti	7	70488258
ebi-a-GCST90001854	rs78694250	CD3 on CD39+ acti	7	156067251
ebi-a-GCST90001854	rs2240626	CD3 on CD39+ acti	7	151089130
ebi-a-GCST90001854	rs55772918	CD3 on CD39+ acti	8	101654508
ebi-a-GCST90001854	rs7868444	CD3 on CD39+ acti	9	28515256
ebi-a-GCST90001854	rs10882659	CD3 on CD39+ acti	10	97510704
ebi-a-GCST90001854	rs79041458	CD3 on CD39+ acti	11	30021259
ebi-a-GCST90001854	rs6589911	CD3 on CD39+ acti	11	99014189
ebi-a-GCST90001854	rs74377561	CD3 on CD39+ acti	12	103510349
ebi-a-GCST90001854	rs55740714	CD3 on CD39+ acti	12	95185923
ebi-a-GCST90001854	rs59302791	CD3 on CD39+ acti	16	17996315
ebi-a-GCST90001854	rs77724325	CD3 on CD39+ acti	16	52834997
ebi-a-GCST90001854	rs181383275	CD3 on CD39+ acti	17	3860393
ebi-a-GCST90001854	rs73063535	CD3 on CD39+ acti	19	49422652
ebi-a-GCST90001854	rs11698085	CD3 on CD39+ acti	20	62036301
ebi-a-GCST90001854	rs142664794	CD3 on CD39+ acti	21	37681268
ebi-a-GCST90001855	rs869139	CD3 on secreting T	1	26527699
ebi-a-GCST90001855	rs114672530	CD3 on secreting T	1	168306279
ebi-a-GCST90001855	rs2949661	CD3 on secreting T	1	167424924
ebi-a-GCST90001855	rs4396110	CD3 on secreting T	1	118266529
ebi-a-GCST90001855	rs115839712	CD3 on secreting T	1	75888556
ebi-a-GCST90001855	rs6684311	CD3 on secreting T	1	166485891
ebi-a-GCST90001855	rs4656440	CD3 on secreting T	1	165218634
ebi-a-GCST90001855	rs142130759	CD3 on secreting T	1	166075360
ebi-a-GCST90001855	rs4657649	CD3 on secreting T	1	167173140
ebi-a-GCST90001855	rs75466245	CD3 on secreting T	2	44973335
ebi-a-GCST90001855	rs57844740	CD3 on secreting T	3	127163016
ebi-a-GCST90001855	rs116168222	CD3 on secreting T	4	181401728

ebi-a-GCST90001855	rs1324073	CD3 on secreting T	6	88822616
ebi-a-GCST90001855	rs142935756	CD3 on secreting T	6	68280346
ebi-a-GCST90001855	rs7094641	CD3 on secreting T	10	67598745
ebi-a-GCST90001855	rs74620952	CD3 on secreting T	11	117753136
ebi-a-GCST90001855	rs4763361	CD3 on secreting T	12	14119860
ebi-a-GCST90001855	rs12884372	CD3 on secreting T	14	86036273
ebi-a-GCST90001855	rs78042544	CD3 on secreting T	15	36121803
ebi-a-GCST90001855	rs8024202	CD3 on secreting T	15	97334659
ebi-a-GCST90001855	rs55799444	CD3 on secreting T	16	90107716
ebi-a-GCST90001855	rs141467190	CD3 on secreting T	17	41589741
ebi-a-GCST90001856	rs72697323	CD3 on CD39+ sec	1	166617824
ebi-a-GCST90001856	rs76540213	CD3 on CD39+ sec	1	166035343
ebi-a-GCST90001856	rs61814872	CD3 on CD39+ sec	1	167113055
ebi-a-GCST90001856	rs79881821	CD3 on CD39+ sec	1	209224498
ebi-a-GCST90001856	rs75831635	CD3 on CD39+ sec	1	91068234
ebi-a-GCST90001856	rs74118738	CD3 on CD39+ sec	1	156667074
ebi-a-GCST90001856	rs2949661	CD3 on CD39+ sec	1	167424924
ebi-a-GCST90001856	rs12742947	CD3 on CD39+ sec	1	165193662
ebi-a-GCST90001856	rs2055631	CD3 on CD39+ sec	2	114898402
ebi-a-GCST90001856	rs11686866	CD3 on CD39+ sec	2	88740296
ebi-a-GCST90001856	rs164962	CD3 on CD39+ sec	3	8690338
ebi-a-GCST90001856	rs145438716	CD3 on CD39+ sec	3	127159039
ebi-a-GCST90001856	rs28547902	CD3 on CD39+ sec	4	7893585
ebi-a-GCST90001856	rs73238001	CD3 on CD39+ sec	4	42867440
ebi-a-GCST90001856	rs12502302	CD3 on CD39+ sec	4	186735780
ebi-a-GCST90001856	rs79338266	CD3 on CD39+ sec	5	52158890
ebi-a-GCST90001856	rs17170861	CD3 on CD39+ sec	7	37113953
ebi-a-GCST90001856	rs112692180	CD3 on CD39+ sec	8	19027810
ebi-a-GCST90001856	rs35922476	CD3 on CD39+ sec	9	8934768
ebi-a-GCST90001856	rs2026334	CD3 on CD39+ sec	9	137822101
ebi-a-GCST90001856	rs11188673	CD3 on CD39+ sec	10	97988763
ebi-a-GCST90001856	rs55740714	CD3 on CD39+ sec	12	95185923
ebi-a-GCST90001856	rs138565853	CD3 on CD39+ sec	13	42836319
ebi-a-GCST90001856	rs8024202	CD3 on CD39+ sec	15	97334659
ebi-a-GCST90001856	rs181229284	CD3 on CD39+ sec	17	51757682
ebi-a-GCST90001856	rs113711222	CD3 on CD39+ sec	19	22042048
ebi-a-GCST90001856	rs6099186	CD3 on CD39+ sec	20	55107273
ebi-a-GCST90001856	rs6085139	CD3 on CD39+ sec	20	5376309
ebi-a-GCST90001857	rs2949661	CD3 on activated &	1	167424924
ebi-a-GCST90001857	rs12138291	CD3 on activated &	1	166464476
ebi-a-GCST90001857	rs869139	CD3 on activated &	1	26527699
ebi-a-GCST90001857	rs35055340	CD3 on activated &	1	165314063
ebi-a-GCST90001857	rs12401936	CD3 on activated &	1	163328756
ebi-a-GCST90001857	rs552999	CD3 on activated &	1	168700134
ebi-a-GCST90001857	rs4396110	CD3 on activated &	1	118266529
ebi-a-GCST90001857	rs114672530	CD3 on activated &	1	168306279
ebi-a-GCST90001857	rs142394936	CD3 on activated &	3	31829907
ebi-a-GCST90001857	rs57844740	CD3 on activated &	3	127163016

ebi-a-GCST90001857	rs2595260	CD3 on activated &	3	158937938
ebi-a-GCST90001857	rs78694250	CD3 on activated &	7	156067251
ebi-a-GCST90001857	rs148303369	CD3 on activated &	9	28514040
ebi-a-GCST90001857	rs79041458	CD3 on activated &	11	30021259
ebi-a-GCST90001857	rs74620952	CD3 on activated &	11	117753136
ebi-a-GCST90001857	rs59684757	CD3 on activated &	14	86146234
ebi-a-GCST90001857	rs8024202	CD3 on activated &	15	97334659
ebi-a-GCST90001857	rs78043002	CD3 on activated &	15	36111764
ebi-a-GCST90001857	rs77724325	CD3 on activated &	16	52834997
ebi-a-GCST90001857	rs59302791	CD3 on activated &	16	17996315
ebi-a-GCST90001857	rs55799444	CD3 on activated &	16	90107716
ebi-a-GCST90001857	rs138786273	CD3 on activated &	17	21944338
ebi-a-GCST90001858	rs61814872	CD3 on CD45RA+	1	167113055
ebi-a-GCST90001858	rs76066554	CD3 on CD45RA+	1	95285730
ebi-a-GCST90001858	rs4657649	CD3 on CD45RA+	1	167173140
ebi-a-GCST90001858	rs4396110	CD3 on CD45RA+	1	118266529
ebi-a-GCST90001858	rs7530127	CD3 on CD45RA+	1	165671820
ebi-a-GCST90001858	rs146330992	CD3 on CD45RA+	1	165888466
ebi-a-GCST90001858	rs2949661	CD3 on CD45RA+	1	167424924
ebi-a-GCST90001858	rs12027506	CD3 on CD45RA+	1	167493088
ebi-a-GCST90001858	rs6751481	CD3 on CD45RA+	2	38897810
ebi-a-GCST90001858	rs7594170	CD3 on CD45RA+	2	51237767
ebi-a-GCST90001858	rs34135810	CD3 on CD45RA+	2	234652185
ebi-a-GCST90001858	rs79179998	CD3 on CD45RA+	3	45947066
ebi-a-GCST90001858	rs4683146	CD3 on CD45RA+	3	45898221
ebi-a-GCST90001858	rs12512585	CD3 on CD45RA+	4	44375745
ebi-a-GCST90001858	rs143262728	CD3 on CD45RA+	4	137926368
ebi-a-GCST90001858	rs72729208	CD3 on CD45RA+	5	10956252
ebi-a-GCST90001858	rs62430462	CD3 on CD45RA+	6	83569632
ebi-a-GCST90001858	rs150857694	CD3 on CD45RA+	7	76111831
ebi-a-GCST90001858	rs144004160	CD3 on CD45RA+	8	19478029
ebi-a-GCST90001858	rs2808525	CD3 on CD45RA+	9	101320250
ebi-a-GCST90001858	rs79844317	CD3 on CD45RA+	10	100127250
ebi-a-GCST90001858	rs72817532	CD3 on CD45RA+	10	30531504
ebi-a-GCST90001858	rs74620952	CD3 on CD45RA+	11	117753136
ebi-a-GCST90001858	rs141784254	CD3 on CD45RA+	11	120364753
ebi-a-GCST90001858	rs76841186	CD3 on CD45RA+	14	102428341
ebi-a-GCST90001858	rs10139012	CD3 on CD45RA+	14	95415204
ebi-a-GCST90001858	rs78042544	CD3 on CD45RA+	15	36121803
ebi-a-GCST90001858	rs62029957	CD3 on CD45RA+	16	50213168
ebi-a-GCST90001858	rs62035375	CD3 on CD45RA+	16	2872116
ebi-a-GCST90001858	rs12921174	CD3 on CD45RA+	16	430304
ebi-a-GCST90001858	rs2714019	CD3 on CD45RA+	17	70789650
ebi-a-GCST90001858	rs187281020	CD3 on CD45RA+	19	43490062
ebi-a-GCST90001858	rs2839523	CD3 on CD45RA+	21	43870045
ebi-a-GCST90001859	rs12132394	CD3 on CD8br	1	235247955
ebi-a-GCST90001859	rs140965084	CD3 on CD8br	1	236324551
ebi-a-GCST90001859	rs116757920	CD3 on CD8br	1	117441813

ebi-a-GCST90001859	rs76066554	CD3 on CD8br	1	95285730
ebi-a-GCST90001859	rs10494441	CD3 on CD8br	1	165250607
ebi-a-GCST90001859	rs1723018	CD3 on CD8br	1	167433420
ebi-a-GCST90001859	rs858545	CD3 on CD8br	1	167461391
ebi-a-GCST90001859	rs2949670	CD3 on CD8br	1	167406513
ebi-a-GCST90001859	rs12144916	CD3 on CD8br	1	167154534
ebi-a-GCST90001859	rs12476279	CD3 on CD8br	2	229680074
ebi-a-GCST90001859	rs143008344	CD3 on CD8br	3	64946405
ebi-a-GCST90001859	rs76757975	CD3 on CD8br	3	78658127
ebi-a-GCST90001859	rs6479155	CD3 on CD8br	9	105868652
ebi-a-GCST90001859	rs75572339	CD3 on CD8br	10	34500210
ebi-a-GCST90001859	rs10828773	CD3 on CD8br	10	18783325
ebi-a-GCST90001859	rs61916018	CD3 on CD8br	11	101769251
ebi-a-GCST90001859	rs10083033	CD3 on CD8br	12	105899685
ebi-a-GCST90001859	rs7174332	CD3 on CD8br	15	33309800
ebi-a-GCST90001859	rs142477005	CD3 on CD8br	16	4504398
ebi-a-GCST90001859	rs7195854	CD3 on CD8br	16	83608598
ebi-a-GCST90001859	rs7203336	CD3 on CD8br	16	75217897
ebi-a-GCST90001859	rs71381322	CD3 on CD8br	17	48900824
ebi-a-GCST90001859	rs1529714	CD3 on CD8br	19	19793484
ebi-a-GCST90001859	rs188662274	CD3 on CD8br	22	38091241
ebi-a-GCST90001860	rs2949661	CD3 on CD39+ CD	1	167424924
ebi-a-GCST90001860	rs4656440	CD3 on CD39+ CD	1	165218634
ebi-a-GCST90001860	rs12077809	CD3 on CD39+ CD	1	167104790
ebi-a-GCST90001860	rs12035159	CD3 on CD39+ CD	1	169011173
ebi-a-GCST90001860	rs72697615	CD3 on CD39+ CD	1	107526739
ebi-a-GCST90001860	rs12138291	CD3 on CD39+ CD	1	166464476
ebi-a-GCST90001860	rs858496	CD3 on CD39+ CD	1	167500918
ebi-a-GCST90001860	rs6697436	CD3 on CD39+ CD	1	26545277
ebi-a-GCST90001860	rs74425152	CD3 on CD39+ CD	2	241271589
ebi-a-GCST90001860	rs139257983	CD3 on CD39+ CD	2	56038405
ebi-a-GCST90001860	rs75443629	CD3 on CD39+ CD	3	141971977
ebi-a-GCST90001860	rs4456952	CD3 on CD39+ CD	4	20480323
ebi-a-GCST90001860	rs78694250	CD3 on CD39+ CD	7	156067251
ebi-a-GCST90001860	rs41434450	CD3 on CD39+ CD	8	101646390
ebi-a-GCST90001860	rs61845257	CD3 on CD39+ CD	10	31348951
ebi-a-GCST90001860	rs141328863	CD3 on CD39+ CD	10	54799428
ebi-a-GCST90001860	rs4763361	CD3 on CD39+ CD	12	14119860
ebi-a-GCST90001860	rs117734680	CD3 on CD39+ CD	12	12952560
ebi-a-GCST90001860	rs113842802	CD3 on CD39+ CD	13	67931948
ebi-a-GCST90001860	rs2700	CD3 on CD39+ CD	14	20825965
ebi-a-GCST90001860	rs61991437	CD3 on CD39+ CD	14	86185512
ebi-a-GCST90001860	rs78042544	CD3 on CD39+ CD	15	36121803
ebi-a-GCST90001860	rs59302791	CD3 on CD39+ CD	16	17996315
ebi-a-GCST90001860	rs9894706	CD3 on CD39+ CD	17	70796437
ebi-a-GCST90001860	rs4796568	CD3 on CD39+ CD	17	6001918
ebi-a-GCST90001860	rs7251035	CD3 on CD39+ CD	19	19866371
ebi-a-GCST90001860	rs142664794	CD3 on CD39+ CD	21	37681268

ebi-a-GCST90001860	rs10483184	CD3 on CD39+ CD	22	35170735
ebi-a-GCST90001861	rs61814886	CD3 on CD28+ CD	1	167136405
ebi-a-GCST90001861	rs4657649	CD3 on CD28+ CD	1	167173140
ebi-a-GCST90001861	rs10801951	CD3 on CD28+ CD	1	117781742
ebi-a-GCST90001861	rs4396110	CD3 on CD28+ CD	1	118266529
ebi-a-GCST90001861	rs2988277	CD3 on CD28+ CD	1	167431352
ebi-a-GCST90001861	rs1922987	CD3 on CD28+ CD	1	101753101
ebi-a-GCST90001861	rs183817699	CD3 on CD28+ CD	2	186714238
ebi-a-GCST90001861	rs115423998	CD3 on CD28+ CD	3	194363700
ebi-a-GCST90001861	rs41289614	CD3 on CD28+ CD	3	45972492
ebi-a-GCST90001861	rs144137185	CD3 on CD28+ CD	3	19582653
ebi-a-GCST90001861	rs75149813	CD3 on CD28+ CD	3	78205852
ebi-a-GCST90001861	rs185805208	CD3 on CD28+ CD	6	118163760
ebi-a-GCST90001861	rs61618798	CD3 on CD28+ CD	7	38070274
ebi-a-GCST90001861	rs73147660	CD3 on CD28+ CD	7	130751643
ebi-a-GCST90001861	rs11787185	CD3 on CD28+ CD	8	4145796
ebi-a-GCST90001861	rs78457979	CD3 on CD28+ CD	9	101856724
ebi-a-GCST90001861	rs113763504	CD3 on CD28+ CD	10	81110536
ebi-a-GCST90001861	rs2728811	CD3 on CD28+ CD	12	17687728
ebi-a-GCST90001861	rs78042544	CD3 on CD28+ CD	15	36121803
ebi-a-GCST90001861	rs62001340	CD3 on CD28+ CD	15	27613525
ebi-a-GCST90001861	rs142628481	CD3 on CD28+ CD	16	4720632
ebi-a-GCST90001861	rs2520957	CD3 on CD28+ CD	16	18293116
ebi-a-GCST90001861	rs73048160	CD3 on CD28+ CD	19	40977235
ebi-a-GCST90001861	rs113348527	CD3 on CD28+ CD	21	24255270
ebi-a-GCST90001861	rs62237850	CD3 on CD28+ CD	22	32030587
ebi-a-GCST90001862	rs851812	CD3 on CD28+ DN	1	118123782
ebi-a-GCST90001862	rs1840657	CD3 on CD28+ DN	2	138676552
ebi-a-GCST90001862	rs75255002	CD3 on CD28+ DN	4	154590624
ebi-a-GCST90001862	rs115371416	CD3 on CD28+ DN	5	89412916
ebi-a-GCST90001862	rs72827833	CD3 on CD28+ DN	6	16748948
ebi-a-GCST90001862	rs1264457	CD3 on CD28+ DN	6	30458064
ebi-a-GCST90001862	rs117404466	CD3 on CD28+ DN	7	148293966
ebi-a-GCST90001862	rs116969543	CD3 on CD28+ DN	8	99091321
ebi-a-GCST90001862	rs117458787	CD3 on CD28+ DN	10	100803548
ebi-a-GCST90001862	rs61862032	CD3 on CD28+ DN	10	124887125
ebi-a-GCST90001862	rs7949056	CD3 on CD28+ DN	11	35999988
ebi-a-GCST90001862	rs113707507	CD3 on CD28+ DN	14	78276958
ebi-a-GCST90001862	rs76548676	CD3 on CD28+ DN	15	92143848
ebi-a-GCST90001862	rs62039948	CD3 on CD28+ DN	16	57886567
ebi-a-GCST90001862	rs1406814	CD3 on CD28+ DN	16	778024
ebi-a-GCST90001862	rs192244506	CD3 on CD28+ DN	18	49186824
ebi-a-GCST90001862	rs7257674	CD3 on CD28+ DN	19	4331621
ebi-a-GCST90001863	rs1723018	CD3 on CD28+ CD	1	167433420
ebi-a-GCST90001863	rs6684311	CD3 on CD28+ CD	1	166485891
ebi-a-GCST90001863	rs2949670	CD3 on CD28+ CD	1	167406513
ebi-a-GCST90001863	rs12132394	CD3 on CD28+ CD	1	235247955
ebi-a-GCST90001863	rs12069993	CD3 on CD28+ CD	1	167705094

ebi-a-GCST90001863	rs858545	CD3 on CD28+ CD	1	167461391
ebi-a-GCST90001863	rs139997835	CD3 on CD28+ CD	2	220485977
ebi-a-GCST90001863	rs17049496	CD3 on CD28+ CD	3	8774388
ebi-a-GCST90001863	rs56046025	CD3 on CD28+ CD	3	71933383
ebi-a-GCST90001863	rs75603751	CD3 on CD28+ CD	6	151129499
ebi-a-GCST90001863	rs71522952	CD3 on CD28+ CD	8	85083506
ebi-a-GCST90001863	rs327018	CD3 on CD28+ CD	11	20849758
ebi-a-GCST90001863	rs11219278	CD3 on CD28+ CD	11	123626762
ebi-a-GCST90001863	rs78043002	CD3 on CD28+ CD	15	36111764
ebi-a-GCST90001863	rs8024202	CD3 on CD28+ CD	15	97334659
ebi-a-GCST90001863	rs117524375	CD3 on CD28+ CD	16	1790546
ebi-a-GCST90001863	rs62028293	CD3 on CD28+ CD	16	50264054
ebi-a-GCST90001863	rs117544452	CD3 on CD28+ CD	17	21757807
ebi-a-GCST90001863	rs6010775	CD3 on CD28+ CD	20	61504457
ebi-a-GCST90001863	rs8128515	CD3 on CD28+ CD	21	30299903
ebi-a-GCST90001864	rs2995089	CD3 on CD28+ CD	1	167431193
ebi-a-GCST90001864	rs7519927	CD3 on CD28+ CD	1	167100361
ebi-a-GCST90001864	rs12729972	CD3 on CD28+ CD	1	118256346
ebi-a-GCST90001864	rs4656440	CD3 on CD28+ CD	1	165218634
ebi-a-GCST90001864	rs12138291	CD3 on CD28+ CD	1	166464476
ebi-a-GCST90001864	rs4657649	CD3 on CD28+ CD	1	167173140
ebi-a-GCST90001864	rs12990220	CD3 on CD28+ CD	2	45451862
ebi-a-GCST90001864	rs79617518	CD3 on CD28+ CD	2	100208697
ebi-a-GCST90001864	rs116266532	CD3 on CD28+ CD	4	42485295
ebi-a-GCST90001864	rs2235394	CD3 on CD28+ CD	6	11763351
ebi-a-GCST90001864	rs73099996	CD3 on CD28+ CD	7	52060488
ebi-a-GCST90001864	rs6479155	CD3 on CD28+ CD	9	105868652
ebi-a-GCST90001864	rs79844317	CD3 on CD28+ CD	10	100127250
ebi-a-GCST90001864	rs111780443	CD3 on CD28+ CD	11	132210362
ebi-a-GCST90001864	rs117079896	CD3 on CD28+ CD	12	101971560
ebi-a-GCST90001864	rs140019589	CD3 on CD28+ CD	13	62183054
ebi-a-GCST90001864	rs4646296	CD3 on CD28+ CD	14	70243505
ebi-a-GCST90001864	rs78043002	CD3 on CD28+ CD	15	36111764
ebi-a-GCST90001864	rs62035375	CD3 on CD28+ CD	16	2872116
ebi-a-GCST90001864	rs62028293	CD3 on CD28+ CD	16	50264054
ebi-a-GCST90001864	rs12921174	CD3 on CD28+ CD	16	430304
ebi-a-GCST90001864	rs148876959	CD3 on CD28+ CD	17	2592302
ebi-a-GCST90001864	rs2713986	CD3 on CD28+ CD	17	70794367
ebi-a-GCST90001864	rs1442353	CD3 on CD28+ CD	18	40992955
ebi-a-GCST90001864	rs73526900	CD3 on CD28+ CD	19	2615996
ebi-a-GCST90001865	rs77294768	CD3 on CD28- CD8	1	6861035
ebi-a-GCST90001865	rs9787219	CD3 on CD28- CD8	1	67080159
ebi-a-GCST90001865	rs2988279	CD3 on CD28- CD8	1	167419839
ebi-a-GCST90001865	rs947505	CD3 on CD28- CD8	1	173302097
ebi-a-GCST90001865	rs62293633	CD3 on CD28- CD8	3	163557077
ebi-a-GCST90001865	rs141566851	CD3 on CD28- CD8	3	170163167
ebi-a-GCST90001865	rs4689428	CD3 on CD28- CD8	4	6388191
ebi-a-GCST90001865	rs1542545	CD3 on CD28- CD8	4	189530591

ebi-a-GCST90001865	rs59837469	CD3 on CD28- CD8	5	169629818
ebi-a-GCST90001865	rs10042722	CD3 on CD28- CD8	5	67121250
ebi-a-GCST90001865	rs10280171	CD3 on CD28- CD8	7	155971101
ebi-a-GCST90001865	rs143007860	CD3 on CD28- CD8	7	13007100
ebi-a-GCST90001865	rs62574602	CD3 on CD28- CD8	9	96839069
ebi-a-GCST90001865	rs12570957	CD3 on CD28- CD8	10	101981274
ebi-a-GCST90001865	rs72975607	CD3 on CD28- CD8	11	103167130
ebi-a-GCST90001865	rs10861453	CD3 on CD28- CD8	12	105931980
ebi-a-GCST90001865	rs7334537	CD3 on CD28- CD8	13	76014918
ebi-a-GCST90001865	rs6564225	CD3 on CD28- CD8	16	75229833
ebi-a-GCST90001865	rs6120663	CD3 on CD28- CD8	20	33081906
ebi-a-GCST90001865	rs28620385	CD3 on CD28- CD8	21	46488731
ebi-a-GCST90001866	rs12138291	CD3 on CD39+ CD	1	166464476
ebi-a-GCST90001866	rs1723018	CD3 on CD39+ CD	1	167433420
ebi-a-GCST90001866	rs858545	CD3 on CD39+ CD	1	167461391
ebi-a-GCST90001866	rs10202116	CD3 on CD39+ CD	2	209108758
ebi-a-GCST90001866	rs1918101	CD3 on CD39+ CD	3	73976238
ebi-a-GCST90001866	rs2448966	CD3 on CD39+ CD	3	99129283
ebi-a-GCST90001866	rs6853238	CD3 on CD39+ CD	4	113212607
ebi-a-GCST90001866	rs11733367	CD3 on CD39+ CD	4	5078910
ebi-a-GCST90001866	rs4958545	CD3 on CD39+ CD	5	151846830
ebi-a-GCST90001866	rs78061697	CD3 on CD39+ CD	7	105599615
ebi-a-GCST90001866	rs144727554	CD3 on CD39+ CD	8	80078162
ebi-a-GCST90001866	rs61352150	CD3 on CD39+ CD	9	111159988
ebi-a-GCST90001866	rs7077566	CD3 on CD39+ CD	10	97617943
ebi-a-GCST90001866	rs72638719	CD3 on CD39+ CD	10	7006748
ebi-a-GCST90001866	rs16942056	CD3 on CD39+ CD	18	23583315
ebi-a-GCST90001866	rs77460005	CD3 on CD39+ CD	22	23474579
ebi-a-GCST90001867	rs11803833	CD3 on CD4+	1	166813933
ebi-a-GCST90001867	rs4657649	CD3 on CD4+	1	167173140
ebi-a-GCST90001867	rs10801951	CD3 on CD4+	1	117781742
ebi-a-GCST90001867	rs12138291	CD3 on CD4+	1	166464476
ebi-a-GCST90001867	rs114672530	CD3 on CD4+	1	168306279
ebi-a-GCST90001867	rs1922987	CD3 on CD4+	1	101753101
ebi-a-GCST90001867	rs1214596	CD3 on CD4+	1	167427170
ebi-a-GCST90001867	rs4396110	CD3 on CD4+	1	118266529
ebi-a-GCST90001867	rs183817699	CD3 on CD4+	2	186714238
ebi-a-GCST90001867	rs41289614	CD3 on CD4+	3	45972492
ebi-a-GCST90001867	rs115423998	CD3 on CD4+	3	194363700
ebi-a-GCST90001867	rs75149813	CD3 on CD4+	3	78205852
ebi-a-GCST90001867	rs144137185	CD3 on CD4+	3	19582653
ebi-a-GCST90001867	rs72729208	CD3 on CD4+	5	10956252
ebi-a-GCST90001867	rs1324073	CD3 on CD4+	6	88822616
ebi-a-GCST90001867	rs185805208	CD3 on CD4+	6	118163760
ebi-a-GCST90001867	rs61618798	CD3 on CD4+	7	38070274
ebi-a-GCST90001867	rs73147660	CD3 on CD4+	7	130751643
ebi-a-GCST90001867	rs11787185	CD3 on CD4+	8	4145796
ebi-a-GCST90001867	rs78457979	CD3 on CD4+	9	101856724

ebi-a-GCST90001867	rs113763504	CD3 on CD4+	10	81110536
ebi-a-GCST90001867	rs2728811	CD3 on CD4+	12	17687728
ebi-a-GCST90001867	rs56107218	CD3 on CD4+	14	69787001
ebi-a-GCST90001867	rs78042544	CD3 on CD4+	15	36121803
ebi-a-GCST90001867	rs148932595	CD3 on CD4+	16	4725694
ebi-a-GCST90001867	rs2520957	CD3 on CD4+	16	18293116
ebi-a-GCST90001867	rs71381322	CD3 on CD4+	17	48900824
ebi-a-GCST90001867	rs2713986	CD3 on CD4+	17	70794367
ebi-a-GCST90001867	rs73048160	CD3 on CD4+	19	40977235
ebi-a-GCST90001867	rs113348527	CD3 on CD4+	21	24255270
ebi-a-GCST90001867	rs62237850	CD3 on CD4+	22	32030587
ebi-a-GCST90001868	rs6684311	CD3 on CD4 Treg	1	166485891
ebi-a-GCST90001868	rs552999	CD3 on CD4 Treg	1	168700134
ebi-a-GCST90001868	rs114672530	CD3 on CD4 Treg	1	168306279
ebi-a-GCST90001868	rs73072986	CD3 on CD4 Treg	1	208074125
ebi-a-GCST90001868	rs4396110	CD3 on CD4 Treg	1	118266529
ebi-a-GCST90001868	rs35055340	CD3 on CD4 Treg	1	165314063
ebi-a-GCST90001868	rs2949661	CD3 on CD4 Treg	1	167424924
ebi-a-GCST90001868	rs6697436	CD3 on CD4 Treg	1	26545277
ebi-a-GCST90001868	rs142394936	CD3 on CD4 Treg	3	31829907
ebi-a-GCST90001868	rs57844740	CD3 on CD4 Treg	3	127163016
ebi-a-GCST90001868	rs2595260	CD3 on CD4 Treg	3	158937938
ebi-a-GCST90001869	rs1214596	CD3 on resting Treg	1	167427170
ebi-a-GCST90001869	rs73036765	CD3 on resting Treg	1	179457254
ebi-a-GCST90001869	rs6658665	CD3 on resting Treg	1	166460696
ebi-a-GCST90001869	rs73072986	CD3 on resting Treg	1	208074125
ebi-a-GCST90001869	rs61814886	CD3 on resting Treg	1	167136405
ebi-a-GCST90001869	rs6668517	CD3 on resting Treg	1	26556870
ebi-a-GCST90001869	rs4657608	CD3 on resting Treg	1	166828464
ebi-a-GCST90001869	rs209858	CD3 on resting Treg	1	114732793
ebi-a-GCST90001869	rs12085166	CD3 on resting Treg	1	116870434
ebi-a-GCST90001869	rs12096911	CD3 on resting Treg	1	167532812
ebi-a-GCST90001869	rs12752576	CD3 on resting Treg	1	168605042
ebi-a-GCST90001869	rs147902311	CD3 on resting Treg	2	218949086
ebi-a-GCST90001869	rs146597227	CD3 on resting Treg	2	57424952
ebi-a-GCST90001869	rs1398059	CD3 on resting Treg	2	188151932
ebi-a-GCST90001869	rs2122146	CD3 on resting Treg	3	158951696
ebi-a-GCST90001869	rs73136665	CD3 on resting Treg	3	99600236
ebi-a-GCST90001869	rs3756564	CD3 on resting Treg	5	178423803
ebi-a-GCST90001869	rs4707319	CD3 on resting Treg	6	87510647
ebi-a-GCST90001869	rs72928038	CD3 on resting Treg	6	90976768
ebi-a-GCST90001869	rs1324073	CD3 on resting Treg	6	88822616
ebi-a-GCST90001869	rs631487	CD3 on resting Treg	6	10479783
ebi-a-GCST90001869	rs74863989	CD3 on resting Treg	6	144281703
ebi-a-GCST90001869	rs73099996	CD3 on resting Treg	7	52060488
ebi-a-GCST90001869	rs79486472	CD3 on resting Treg	7	70509663
ebi-a-GCST90001869	rs79844317	CD3 on resting Treg	10	100127250
ebi-a-GCST90001869	rs74620952	CD3 on resting Treg	11	117753136

ebi-a-GCST90001869	rs141784254	CD3 on resting Treg	11	120364753
ebi-a-GCST90001869	rs1537547	CD3 on resting Treg	13	67932513
ebi-a-GCST90001869	rs150306980	CD3 on resting Treg	15	100531503
ebi-a-GCST90001869	rs78042544	CD3 on resting Treg	15	36121803
ebi-a-GCST90001869	rs55799444	CD3 on resting Treg	16	90107716
ebi-a-GCST90001869	rs115662161	CD3 on resting Treg	16	11511391
ebi-a-GCST90001869	rs2015682	CD3 on resting Treg	16	2854907
ebi-a-GCST90001869	rs181383275	CD3 on resting Treg	17	3860393
ebi-a-GCST90001869	rs62084754	CD3 on resting Treg	17	66093841
ebi-a-GCST90001869	rs187281020	CD3 on resting Treg	19	43490062
ebi-a-GCST90001869	rs722898	CD3 on resting Treg	20	40714524
ebi-a-GCST90001869	rs59604901	CD3 on resting Treg	20	25444618
ebi-a-GCST90001869	rs6586224	CD3 on resting Treg	21	43122243
ebi-a-GCST90001870	rs17451898	CD34 on HSC	1	81770460
ebi-a-GCST90001870	rs586030	CD34 on HSC	1	208170487
ebi-a-GCST90001870	rs6427732	CD34 on HSC	1	163371663
ebi-a-GCST90001870	rs139489968	CD34 on HSC	1	208017826
ebi-a-GCST90001870	rs12623399	CD34 on HSC	2	46349271
ebi-a-GCST90001870	rs12621242	CD34 on HSC	2	23022235
ebi-a-GCST90001870	rs4406271	CD34 on HSC	6	109598213
ebi-a-GCST90001870	rs3735683	CD34 on HSC	7	1025533
ebi-a-GCST90001870	rs56168975	CD34 on HSC	10	11298964
ebi-a-GCST90001870	rs139622960	CD34 on HSC	11	66287586
ebi-a-GCST90001870	rs117755089	CD34 on HSC	14	28026419
ebi-a-GCST90001870	rs36102158	CD34 on HSC	15	50363583
ebi-a-GCST90001870	rs574328396	CD34 on HSC	19	21062578
ebi-a-GCST90001870	rs11701279	CD34 on HSC	21	44552070
ebi-a-GCST90001871	rs1499672	HVEM on T cell	1	56037629
ebi-a-GCST90001871	rs1886730	HVEM on T cell	1	2488608
ebi-a-GCST90001871	rs140639767	HVEM on T cell	1	146998040
ebi-a-GCST90001871	rs62140870	HVEM on T cell	2	47713921
ebi-a-GCST90001871	rs13014690	HVEM on T cell	2	153797694
ebi-a-GCST90001871	rs116783256	HVEM on T cell	2	209940257
ebi-a-GCST90001871	rs73227426	HVEM on T cell	3	112068503
ebi-a-GCST90001871	rs9870329	HVEM on T cell	3	126102109
ebi-a-GCST90001871	rs2731518	HVEM on T cell	3	1896695
ebi-a-GCST90001871	rs114178658	HVEM on T cell	4	35188325
ebi-a-GCST90001871	rs10008912	HVEM on T cell	4	7148069
ebi-a-GCST90001871	rs7731342	HVEM on T cell	5	104549911
ebi-a-GCST90001871	rs7738988	HVEM on T cell	6	83760703
ebi-a-GCST90001871	rs78389375	HVEM on T cell	7	81160961
ebi-a-GCST90001871	rs6577804	HVEM on T cell	8	138522864
ebi-a-GCST90001871	rs2246952	HVEM on T cell	10	70782592
ebi-a-GCST90001871	rs1552721	HVEM on T cell	11	7000368
ebi-a-GCST90001871	rs11212429	HVEM on T cell	11	107761682
ebi-a-GCST90001871	rs12284339	HVEM on T cell	11	107699559
ebi-a-GCST90001871	rs12818999	HVEM on T cell	12	5133216
ebi-a-GCST90001871	rs10747856	HVEM on T cell	12	60262116

ebi-a-GCST90001871	rs73449242	HVEM on T cell	13	24033981
ebi-a-GCST90001871	rs3138035	HVEM on T cell	17	32645949
ebi-a-GCST90001871	rs79388518	HVEM on T cell	18	1705002
ebi-a-GCST90001871	rs73049692	HVEM on T cell	19	51995348
ebi-a-GCST90001871	rs313856	HVEM on T cell	19	47298263
ebi-a-GCST90001872	rs28422844	HVEM on naive CI	1	28831071
ebi-a-GCST90001872	rs2234161	HVEM on naive CI	1	2491205
ebi-a-GCST90001872	rs142774716	HVEM on naive CI	1	95096608
ebi-a-GCST90001872	rs12476985	HVEM on naive CI	2	184183623
ebi-a-GCST90001872	rs13013216	HVEM on naive CI	2	153797688
ebi-a-GCST90001872	rs73851787	HVEM on naive CI	4	117567589
ebi-a-GCST90001872	rs2964678	HVEM on naive CI	5	116104935
ebi-a-GCST90001872	rs2523589	HVEM on naive CI	6	31327334
ebi-a-GCST90001872	rs561768	HVEM on naive CI	6	168203927
ebi-a-GCST90001872	rs2844594	HVEM on naive CI	6	31258393
ebi-a-GCST90001872	rs112421400	HVEM on naive CI	6	28205826
ebi-a-GCST90001872	rs17532418	HVEM on naive CI	7	121741943
ebi-a-GCST90001872	rs11981290	HVEM on naive CI	7	154686456
ebi-a-GCST90001872	rs28904611	HVEM on naive CI	8	89243591
ebi-a-GCST90001872	rs118151702	HVEM on naive CI	8	138502532
ebi-a-GCST90001872	rs10090960	HVEM on naive CI	8	136809038
ebi-a-GCST90001872	rs524655	HVEM on naive CI	11	107670625
ebi-a-GCST90001872	rs17105684	HVEM on naive CI	11	106267999
ebi-a-GCST90001872	rs4898532	HVEM on naive CI	12	50223579
ebi-a-GCST90001872	rs1353750	HVEM on naive CI	12	17510433
ebi-a-GCST90001872	rs144299393	HVEM on naive CI	13	109533726
ebi-a-GCST90001872	rs17614093	HVEM on naive CI	17	32509256
ebi-a-GCST90001873	rs1886730	HVEM on EM CD8	1	2488608
ebi-a-GCST90001873	rs11810872	HVEM on EM CD8	1	44793667
ebi-a-GCST90001873	rs16834073	HVEM on EM CD8	2	154560519
ebi-a-GCST90001873	rs11707883	HVEM on EM CD8	3	126414066
ebi-a-GCST90001873	rs73807141	HVEM on EM CD8	3	4772145
ebi-a-GCST90001873	rs139631207	HVEM on EM CD8	3	136490521
ebi-a-GCST90001873	rs13136753	HVEM on EM CD8	4	17244840
ebi-a-GCST90001873	rs139827714	HVEM on EM CD8	4	47461569
ebi-a-GCST90001873	rs76121075	HVEM on EM CD8	6	27296440
ebi-a-GCST90001873	rs6918295	HVEM on EM CD8	6	119522751
ebi-a-GCST90001873	rs28367600	HVEM on EM CD8	6	31266495
ebi-a-GCST90001873	rs7792156	HVEM on EM CD8	7	152855875
ebi-a-GCST90001873	rs28808959	HVEM on EM CD8	8	57257758
ebi-a-GCST90001873	rs1875440	HVEM on EM CD8	9	2835973
ebi-a-GCST90001873	rs356246	HVEM on EM CD8	11	127449726
ebi-a-GCST90001873	rs7962432	HVEM on EM CD8	12	105760206
ebi-a-GCST90001873	rs80228817	HVEM on EM CD8	14	41599648
ebi-a-GCST90001873	rs112619557	HVEM on EM CD8	15	31777164
ebi-a-GCST90001873	rs72913358	HVEM on EM CD8	18	42590961
ebi-a-GCST90001874	rs2258734	HVEM on TD CD8	1	2483961
ebi-a-GCST90001874	rs74414172	HVEM on TD CD8	1	210532944

ebi-a-GCST90001874	rs16834073	HVEM on TD CD8	2	154560519
ebi-a-GCST90001874	rs2862874	HVEM on TD CD8	2	58990485
ebi-a-GCST90001874	rs112418980	HVEM on TD CD8	2	34001527
ebi-a-GCST90001874	rs80056411	HVEM on TD CD8	3	60109679
ebi-a-GCST90001874	rs17354029	HVEM on TD CD8	4	83660842
ebi-a-GCST90001874	rs227452	HVEM on TD CD8	6	165479017
ebi-a-GCST90001874	rs6903232	HVEM on TD CD8	6	121724473
ebi-a-GCST90001874	rs9489809	HVEM on TD CD8	6	120117845
ebi-a-GCST90001874	rs80235303	HVEM on TD CD8	6	159828798
ebi-a-GCST90001874	rs111696805	HVEM on TD CD8	7	54565976
ebi-a-GCST90001874	rs529163274	HVEM on TD CD8	7	34495959
ebi-a-GCST90001874	rs9696823	HVEM on TD CD8	9	26754888
ebi-a-GCST90001874	rs75776206	HVEM on TD CD8	9	95793215
ebi-a-GCST90001874	rs73597199	HVEM on TD CD8	11	134924887
ebi-a-GCST90001874	rs117738211	HVEM on TD CD8	11	93741127
ebi-a-GCST90001874	rs61622210	HVEM on TD CD8	11	57036822
ebi-a-GCST90001874	rs10842705	HVEM on TD CD8	12	26469016
ebi-a-GCST90001874	rs4075681	HVEM on TD CD8	12	50214637
ebi-a-GCST90001874	rs6489587	HVEM on TD CD8	12	638012
ebi-a-GCST90001874	rs2374621	HVEM on TD CD8	12	106929985
ebi-a-GCST90001874	rs76481969	HVEM on TD CD8	13	55625565
ebi-a-GCST90001874	rs1483108	HVEM on TD CD8	14	57322045
ebi-a-GCST90001874	rs78305076	HVEM on TD CD8	14	34314839
ebi-a-GCST90001874	rs140287246	HVEM on TD CD8	15	96917459
ebi-a-GCST90001874	rs8057943	HVEM on TD CD8	16	80431900
ebi-a-GCST90001874	rs12972416	HVEM on TD CD8	19	44220733
ebi-a-GCST90001875	rs226073	HVEM on CD4+	1	44835020
ebi-a-GCST90001875	rs904604	HVEM on CD4+	1	56042915
ebi-a-GCST90001875	rs1886730	HVEM on CD4+	1	2488608
ebi-a-GCST90001875	rs4657680	HVEM on CD4+	1	167575104
ebi-a-GCST90001875	rs1714947	HVEM on CD4+	2	235253930
ebi-a-GCST90001875	rs79573167	HVEM on CD4+	2	33965374
ebi-a-GCST90001875	rs141874683	HVEM on CD4+	2	5836918
ebi-a-GCST90001875	rs62246799	HVEM on CD4+	3	2053929
ebi-a-GCST90001875	rs2074489	HVEM on CD4+	6	31240128
ebi-a-GCST90001875	rs11153673	HVEM on CD4+	6	117863943
ebi-a-GCST90001875	rs62471911	HVEM on CD4+	7	154798490
ebi-a-GCST90001875	rs28712645	HVEM on CD4+	7	157863000
ebi-a-GCST90001875	rs7896504	HVEM on CD4+	10	1634042
ebi-a-GCST90001875	rs12284339	HVEM on CD4+	11	107699559
ebi-a-GCST90001875	rs11212429	HVEM on CD4+	11	107761682
ebi-a-GCST90001875	rs4589374	HVEM on CD4+	12	53204002
ebi-a-GCST90001875	rs144299393	HVEM on CD4+	13	109533726
ebi-a-GCST90001875	rs73449242	HVEM on CD4+	13	24033981
ebi-a-GCST90001875	rs56091264	HVEM on CD4+	16	28619179
ebi-a-GCST90001875	rs79388518	HVEM on CD4+	18	1705002
ebi-a-GCST90001875	rs148349980	HVEM on CD4+	21	15328061
ebi-a-GCST90001876	rs147981351	HVEM on CM CD4	1	3030338

ebi-a-GCST90001876	rs1886730	HVEM on CM CD4	1	2488608
ebi-a-GCST90001876	rs226073	HVEM on CM CD4	1	44835020
ebi-a-GCST90001876	rs149830152	HVEM on CM CD4	2	59392272
ebi-a-GCST90001876	rs141874683	HVEM on CM CD4	2	5836918
ebi-a-GCST90001876	rs143967899	HVEM on CM CD4	3	33728588
ebi-a-GCST90001876	rs3772836	HVEM on CM CD4	3	124535635
ebi-a-GCST90001876	rs28367673	HVEM on CM CD4	6	31269215
ebi-a-GCST90001876	rs62471910	HVEM on CM CD4	7	154798440
ebi-a-GCST90001876	rs1613049	HVEM on CM CD4	7	152987872
ebi-a-GCST90001876	rs2686206	HVEM on CM CD4	8	11620315
ebi-a-GCST90001876	rs524655	HVEM on CM CD4	11	107670625
ebi-a-GCST90001876	rs73449242	HVEM on CM CD4	13	24033981
ebi-a-GCST90001876	rs77731409	HVEM on CM CD4	14	26718656
ebi-a-GCST90001876	rs114558014	HVEM on CM CD4	16	28512884
ebi-a-GCST90001876	rs17542274	HVEM on CM CD4	17	32529159
ebi-a-GCST90001876	rs77231600	HVEM on CM CD4	18	5850612
ebi-a-GCST90001876	rs1579540	HVEM on CM CD4	18	49076557
ebi-a-GCST90001876	rs75432705	HVEM on CM CD4	20	44951484
ebi-a-GCST90001877	rs2234161	HVEM on naive CI	1	2491205
ebi-a-GCST90001877	rs13434241	HVEM on naive CI	3	134924639
ebi-a-GCST90001877	rs7430694	HVEM on naive CI	3	129730125
ebi-a-GCST90001877	rs11760108	HVEM on naive CI	6	46849057
ebi-a-GCST90001877	rs536853	HVEM on naive CI	6	23965983
ebi-a-GCST90001877	rs7385030	HVEM on naive CI	7	157857174
ebi-a-GCST90001877	rs17532418	HVEM on naive CI	7	121741943
ebi-a-GCST90001877	rs28904611	HVEM on naive CI	8	89243591
ebi-a-GCST90001877	rs72730570	HVEM on naive CI	9	104812918
ebi-a-GCST90001877	rs7896504	HVEM on naive CI	10	1634042
ebi-a-GCST90001877	rs524655	HVEM on naive CI	11	107670625
ebi-a-GCST90001877	rs144299393	HVEM on naive CI	13	109533726
ebi-a-GCST90001877	rs73449242	HVEM on naive CI	13	24033981
ebi-a-GCST90001877	rs117656327	HVEM on naive CI	14	23450548
ebi-a-GCST90001877	rs117618257	HVEM on naive CI	16	423808
ebi-a-GCST90001877	rs56091264	HVEM on naive CI	16	28619179
ebi-a-GCST90001877	rs150797289	HVEM on naive CI	17	13805082
ebi-a-GCST90001877	rs62076816	HVEM on naive CI	18	5843287
ebi-a-GCST90001878	rs1772154	HVEM on EM CD4	1	205745932
ebi-a-GCST90001878	rs2182176	HVEM on EM CD4	1	2499254
ebi-a-GCST90001878	rs4657680	HVEM on EM CD4	1	167575104
ebi-a-GCST90001878	rs2002586	HVEM on EM CD4	2	19497003
ebi-a-GCST90001878	rs116838007	HVEM on EM CD4	2	241352278
ebi-a-GCST90001878	rs12053385	HVEM on EM CD4	2	209630745
ebi-a-GCST90001878	rs73150365	HVEM on EM CD4	3	100152337
ebi-a-GCST90001878	rs115176674	HVEM on EM CD4	3	174773888
ebi-a-GCST90001878	rs115725884	HVEM on EM CD4	3	139112247
ebi-a-GCST90001878	rs6882776	HVEM on EM CD4	5	172664163
ebi-a-GCST90001878	rs11758857	HVEM on EM CD4	6	117867335
ebi-a-GCST90001878	rs7754903	HVEM on EM CD4	6	31254496

ebi-a-GCST90001878	rs142133424	HVEM on EM CD4	6	132490260
ebi-a-GCST90001878	rs117761782	HVEM on EM CD4	8	22024980
ebi-a-GCST90001878	rs2959645	HVEM on EM CD4	8	99010895
ebi-a-GCST90001878	rs7864163	HVEM on EM CD4	9	2029454
ebi-a-GCST90001878	rs61873707	HVEM on EM CD4	10	99831579
ebi-a-GCST90001878	rs524655	HVEM on EM CD4	11	107670625
ebi-a-GCST90001878	rs74257706	HVEM on EM CD4	18	63124981
ebi-a-GCST90001878	rs557685	HVEM on EM CD4	18	610273
ebi-a-GCST90001878	rs4803979	HVEM on EM CD4	19	47040463
ebi-a-GCST90001878	rs147371997	HVEM on EM CD4	19	36235084
ebi-a-GCST90001879	rs2227313	HVEM on TD CD4	1	2487766
ebi-a-GCST90001879	rs10888005	HVEM on TD CD4	1	17473323
ebi-a-GCST90001879	rs759400	HVEM on TD CD4	2	10709816
ebi-a-GCST90001879	rs145042920	HVEM on TD CD4	2	88351748
ebi-a-GCST90001879	rs56365633	HVEM on TD CD4	4	164927486
ebi-a-GCST90001879	rs7735724	HVEM on TD CD4	5	53194393
ebi-a-GCST90001879	rs78318630	HVEM on TD CD4	6	36237023
ebi-a-GCST90001879	rs34500563	HVEM on TD CD4	6	10747254
ebi-a-GCST90001879	rs78703228	HVEM on TD CD4	7	133783811
ebi-a-GCST90001879	rs2158382	HVEM on TD CD4	7	19764946
ebi-a-GCST90001879	rs1541415	HVEM on TD CD4	7	147205421
ebi-a-GCST90001879	rs7024801	HVEM on TD CD4	9	93360653
ebi-a-GCST90001879	rs12378405	HVEM on TD CD4	9	7820271
ebi-a-GCST90001879	rs2382040	HVEM on TD CD4	9	9681323
ebi-a-GCST90001879	rs76025942	HVEM on TD CD4	9	96367900
ebi-a-GCST90001879	rs11014115	HVEM on TD CD4	10	24786510
ebi-a-GCST90001879	rs11592466	HVEM on TD CD4	10	14316154
ebi-a-GCST90001879	rs4880998	HVEM on TD CD4	10	2506524
ebi-a-GCST90001879	rs7918482	HVEM on TD CD4	10	131682181
ebi-a-GCST90001879	rs11232580	HVEM on TD CD4	11	81008355
ebi-a-GCST90001879	rs7122006	HVEM on TD CD4	11	128827397
ebi-a-GCST90001879	rs12833432	HVEM on TD CD4	12	130668546
ebi-a-GCST90001879	rs117920606	HVEM on TD CD4	13	70336135
ebi-a-GCST90001879	rs9549072	HVEM on TD CD4	13	40707448
ebi-a-GCST90001879	rs11659521	HVEM on TD CD4	18	5862557
ebi-a-GCST90001879	rs1399834	HVEM on TD CD4	19	51797326
ebi-a-GCST90001880	rs80147656	HVEM on CD45RA	1	97830828
ebi-a-GCST90001880	rs4657680	HVEM on CD45RA	1	167575104
ebi-a-GCST90001880	rs1886730	HVEM on CD45RA	1	2488608
ebi-a-GCST90001880	rs226073	HVEM on CD45RA	1	44835020
ebi-a-GCST90001880	rs904604	HVEM on CD45RA	1	56042915
ebi-a-GCST90001880	rs149830152	HVEM on CD45RA	2	59392272
ebi-a-GCST90001880	rs2002586	HVEM on CD45RA	2	19497003
ebi-a-GCST90001880	rs792072	HVEM on CD45RA	2	5593302
ebi-a-GCST90001880	rs141874683	HVEM on CD45RA	2	5836918
ebi-a-GCST90001880	rs79573167	HVEM on CD45RA	2	33965374
ebi-a-GCST90001880	rs2074489	HVEM on CD45RA	6	31240128
ebi-a-GCST90001880	rs11153673	HVEM on CD45RA	6	117863943

ebi-a-GCST90001880	rs62471911	HVEM on CD45RA	7	154798490
ebi-a-GCST90001880	rs28712645	HVEM on CD45RA	7	157863000
ebi-a-GCST90001880	rs74680174	HVEM on CD45RA	9	117077793
ebi-a-GCST90001880	rs4752368	HVEM on CD45RA	10	121771245
ebi-a-GCST90001880	rs524655	HVEM on CD45RA	11	107670625
ebi-a-GCST90001880	rs56091264	HVEM on CD45RA	16	28619179
ebi-a-GCST90001880	rs77231600	HVEM on CD45RA	18	5850612
ebi-a-GCST90001880	rs147371997	HVEM on CD45RA	19	36235084
ebi-a-GCST90001881	rs2227313	HVEM on CD8br	1	2487766
ebi-a-GCST90001881	rs61731213	HVEM on CD8br	1	203690537
ebi-a-GCST90001881	rs16834073	HVEM on CD8br	2	154560519
ebi-a-GCST90001881	rs182735817	HVEM on CD8br	2	129316586
ebi-a-GCST90001881	rs150482210	HVEM on CD8br	3	135398834
ebi-a-GCST90001881	rs75214693	HVEM on CD8br	4	93244732
ebi-a-GCST90001881	rs2219729	HVEM on CD8br	5	101440775
ebi-a-GCST90001881	rs35211801	HVEM on CD8br	5	80948384
ebi-a-GCST90001881	rs9267111	HVEM on CD8br	6	31416379
ebi-a-GCST90001881	rs80126152	HVEM on CD8br	7	83616304
ebi-a-GCST90001881	rs117761782	HVEM on CD8br	8	22024980
ebi-a-GCST90001881	rs10090960	HVEM on CD8br	8	136809038
ebi-a-GCST90001881	rs10508717	HVEM on CD8br	10	26672328
ebi-a-GCST90001881	rs2338998	HVEM on CD8br	17	32670901
ebi-a-GCST90001881	rs7210730	HVEM on CD8br	17	78629025
ebi-a-GCST90001881	rs9966806	HVEM on CD8br	18	64825431
ebi-a-GCST90001882	rs61826205	HVEM on CM CD8	1	170559626
ebi-a-GCST90001882	rs11807062	HVEM on CM CD8	1	3153237
ebi-a-GCST90001882	rs1886730	HVEM on CM CD8	1	2488608
ebi-a-GCST90001882	rs4593803	HVEM on CM CD8	1	91231232
ebi-a-GCST90001882	rs58461262	HVEM on CM CD8	2	5859854
ebi-a-GCST90001882	rs62246799	HVEM on CM CD8	3	2053929
ebi-a-GCST90001882	rs2937054	HVEM on CM CD8	5	116099168
ebi-a-GCST90001882	rs536853	HVEM on CM CD8	6	23965983
ebi-a-GCST90001882	rs72923012	HVEM on CM CD8	6	98060503
ebi-a-GCST90001882	rs73390208	HVEM on CM CD8	6	31326131
ebi-a-GCST90001882	rs62471910	HVEM on CM CD8	7	154798440
ebi-a-GCST90001882	rs28904611	HVEM on CM CD8	8	89243591
ebi-a-GCST90001882	rs2740433	HVEM on CM CD8	8	11621450
ebi-a-GCST90001882	rs4977976	HVEM on CM CD8	9	21156118
ebi-a-GCST90001882	rs524655	HVEM on CM CD8	11	107670625
ebi-a-GCST90001882	rs113260310	HVEM on CM CD8	11	77477036
ebi-a-GCST90001882	rs9509382	HVEM on CM CD8	13	19256068
ebi-a-GCST90001882	rs2733310	HVEM on CM CD8	15	57498691
ebi-a-GCST90001882	rs837640	HVEM on CM CD8	19	49283494
ebi-a-GCST90001883	rs138425921	CD16-CD56 on NK	1	22721951
ebi-a-GCST90001883	rs28411259	CD16-CD56 on NK	1	230179351
ebi-a-GCST90001883	rs114688494	CD16-CD56 on NK	2	23343715
ebi-a-GCST90001883	rs141668845	CD16-CD56 on NK	3	33950996
ebi-a-GCST90001883	rs237883	CD16-CD56 on NK	3	8788485

ebi-a-GCST90001883	rs115442185	CD16-CD56 on NK	4	109239280
ebi-a-GCST90001883	rs12501477	CD16-CD56 on NK	4	182906397
ebi-a-GCST90001883	rs138112111	CD16-CD56 on NK	6	97227252
ebi-a-GCST90001883	rs115536607	CD16-CD56 on NK	6	101417409
ebi-a-GCST90001883	rs9488834	CD16-CD56 on NK	6	97889936
ebi-a-GCST90001883	rs75710318	CD16-CD56 on NK	6	69685408
ebi-a-GCST90001883	rs79251210	CD16-CD56 on NK	6	100468602
ebi-a-GCST90001883	rs117147408	CD16-CD56 on NK	6	101980039
ebi-a-GCST90001883	rs75616233	CD16-CD56 on NK	7	103955121
ebi-a-GCST90001883	rs73209471	CD16-CD56 on NK	8	20867564
ebi-a-GCST90001883	rs532733980	CD16-CD56 on NK	9	138754221
ebi-a-GCST90001883	rs145334185	CD16-CD56 on NK	10	22123801
ebi-a-GCST90001883	rs12573757	CD16-CD56 on NK	10	83824559
ebi-a-GCST90001883	rs11214436	CD16-CD56 on NK	11	112830782
ebi-a-GCST90001883	rs77738700	CD16-CD56 on NK	11	112751407
ebi-a-GCST90001883	rs17729025	CD16-CD56 on NK	11	110827812
ebi-a-GCST90001883	rs45602031	CD16-CD56 on NK	11	113117261
ebi-a-GCST90001883	rs10219323	CD16-CD56 on NK	11	111984131
ebi-a-GCST90001883	rs192922707	CD16-CD56 on NK	13	40183073
ebi-a-GCST90001883	rs142268019	CD16-CD56 on NK	13	70784181
ebi-a-GCST90001883	rs7157523	CD16-CD56 on NK	14	93132015
ebi-a-GCST90001883	rs36015524	CD16-CD56 on NK	18	35141783
ebi-a-GCST90001883	rs9950268	CD16-CD56 on NK	18	4925256
ebi-a-GCST90001883	rs10401901	CD16-CD56 on NK	19	54956254
ebi-a-GCST90001883	rs150921517	CD16-CD56 on NK	21	41897898
ebi-a-GCST90001884	rs146713480	CD16-CD56 on NK	1	161121300
ebi-a-GCST90001884	rs10919544	CD16-CD56 on NK	1	161508763
ebi-a-GCST90001884	rs140904849	CD16-CD56 on NK	1	32442768
ebi-a-GCST90001884	rs139795227	CD16-CD56 on NK	1	92842367
ebi-a-GCST90001884	rs75586628	CD16-CD56 on NK	3	144642201
ebi-a-GCST90001884	rs144290638	CD16-CD56 on NK	4	31794715
ebi-a-GCST90001884	rs143549694	CD16-CD56 on NK	4	161858005
ebi-a-GCST90001884	rs12644590	CD16-CD56 on NK	4	16657392
ebi-a-GCST90001884	rs75500437	CD16-CD56 on NK	4	58092490
ebi-a-GCST90001884	rs71607232	CD16-CD56 on NK	4	60815666
ebi-a-GCST90001884	rs2844642	CD16-CD56 on NK	6	31019806
ebi-a-GCST90001884	rs7760353	CD16-CD56 on NK	6	102207604
ebi-a-GCST90001884	rs186982732	CD16-CD56 on NK	9	87447998
ebi-a-GCST90001884	rs7023353	CD16-CD56 on NK	9	110692719
ebi-a-GCST90001884	rs56341765	CD16-CD56 on NK	10	6270618
ebi-a-GCST90001884	rs117655461	CD16-CD56 on NK	11	114307356
ebi-a-GCST90001884	rs111579151	CD16-CD56 on NK	11	111848073
ebi-a-GCST90001884	rs148849191	CD16-CD56 on NK	11	113153223
ebi-a-GCST90001884	rs17504675	CD16-CD56 on NK	11	110737507
ebi-a-GCST90001884	rs77291736	CD16-CD56 on NK	11	112749800
ebi-a-GCST90001884	rs117533070	CD16-CD56 on NK	11	112710664
ebi-a-GCST90001884	rs61900380	CD16-CD56 on NK	11	83760635
ebi-a-GCST90001884	rs117853201	CD16-CD56 on NK	11	80035628

ebi-a-GCST90001884	rs182003559	CD16-CD56 on NK	11	110491411
ebi-a-GCST90001884	rs7947051	CD16-CD56 on NK	11	129495547
ebi-a-GCST90001884	rs11214436	CD16-CD56 on NK	11	112830782
ebi-a-GCST90001884	rs189384754	CD16-CD56 on NK	12	122677843
ebi-a-GCST90001884	rs73207892	CD16-CD56 on NK	13	69429461
ebi-a-GCST90001884	rs142394087	CD16-CD56 on NK	14	66348437
ebi-a-GCST90001884	rs12944882	CD16-CD56 on NK	17	37983492
ebi-a-GCST90001884	rs151212014	CD16-CD56 on NK	19	4195225
ebi-a-GCST90001885	rs147640330	CD16-CD56 on HL	1	161536758
ebi-a-GCST90001885	rs72815294	CD16-CD56 on HL	2	26780538
ebi-a-GCST90001885	rs75586628	CD16-CD56 on HL	3	144642201
ebi-a-GCST90001885	rs62247496	CD16-CD56 on HL	3	9905680
ebi-a-GCST90001885	rs4683278	CD16-CD56 on HL	3	47026596
ebi-a-GCST90001885	rs13132013	CD16-CD56 on HL	4	96713626
ebi-a-GCST90001885	rs17146863	CD16-CD56 on HL	5	119959773
ebi-a-GCST90001885	rs4866395	CD16-CD56 on HL	5	33866241
ebi-a-GCST90001885	rs2463678	CD16-CD56 on HL	7	85124635
ebi-a-GCST90001885	rs149897374	CD16-CD56 on HL	7	108731721
ebi-a-GCST90001885	rs78309366	CD16-CD56 on HL	8	3406535
ebi-a-GCST90001885	rs72693216	CD16-CD56 on HL	9	2285910
ebi-a-GCST90001885	rs1590409	CD16-CD56 on HL	9	13601074
ebi-a-GCST90001885	rs2260397	CD16-CD56 on HL	10	94163116
ebi-a-GCST90001885	rs4963162	CD16-CD56 on HL	11	769015
ebi-a-GCST90001885	rs78358948	CD16-CD56 on HL	11	112751974
ebi-a-GCST90001885	rs11229021	CD16-CD56 on HL	11	55061239
ebi-a-GCST90001885	rs369336153	CD16-CD56 on HL	11	1119270
ebi-a-GCST90001885	rs12580221	CD16-CD56 on HL	12	125621066
ebi-a-GCST90001885	rs543548673	CD16-CD56 on HL	12	42267311
ebi-a-GCST90001885	rs79850036	CD16-CD56 on HL	12	5507432
ebi-a-GCST90001885	rs117051465	CD16-CD56 on HL	14	71723360
ebi-a-GCST90001885	rs11634090	CD16-CD56 on HL	15	100538763
ebi-a-GCST90001885	rs62015176	CD16-CD56 on HL	15	68042518
ebi-a-GCST90001885	rs224230	CD16-CD56 on HL	16	3308357
ebi-a-GCST90001885	rs74783293	CD16-CD56 on HL	20	43204977
ebi-a-GCST90001886	rs181148368	CD28 on CD39+ ac	1	47339027
ebi-a-GCST90001886	rs77594357	CD28 on CD39+ ac	2	204328474
ebi-a-GCST90001886	rs702874	CD28 on CD39+ ac	2	65005540
ebi-a-GCST90001886	rs3116493	CD28 on CD39+ ac	2	204589281
ebi-a-GCST90001886	rs72968907	CD28 on CD39+ ac	3	123701658
ebi-a-GCST90001886	rs75966592	CD28 on CD39+ ac	4	25806035
ebi-a-GCST90001886	rs78069822	CD28 on CD39+ ac	5	31664604
ebi-a-GCST90001886	rs13170880	CD28 on CD39+ ac	5	81137543
ebi-a-GCST90001886	rs113069636	CD28 on CD39+ ac	6	71917703
ebi-a-GCST90001886	rs75477465	CD28 on CD39+ ac	8	68794331
ebi-a-GCST90001886	rs79881476	CD28 on CD39+ ac	10	86422393
ebi-a-GCST90001886	rs2226163	CD28 on CD39+ ac	10	97628994
ebi-a-GCST90001886	rs12146493	CD28 on CD39+ ac	11	65547333
ebi-a-GCST90001886	rs144419206	CD28 on CD39+ ac	11	24126713

ebi-a-GCST90001886	rs7294586	CD28 on CD39+ ac	12	100890455
ebi-a-GCST90001886	rs111860462	CD28 on CD39+ ac	14	82047636
ebi-a-GCST90001886	rs79450701	CD28 on CD39+ ac	14	55670402
ebi-a-GCST90001886	rs2521501	CD28 on CD39+ ac	15	91437388
ebi-a-GCST90001886	rs137875117	CD28 on CD39+ ac	16	25627987
ebi-a-GCST90001886	rs147329602	CD28 on CD39+ ac	19	6350406
ebi-a-GCST90001887	rs34460712	CD28 on secreting '	2	38935236
ebi-a-GCST90001887	rs62183989	CD28 on secreting '	2	204609402
ebi-a-GCST90001887	rs116479405	CD28 on secreting '	2	49884156
ebi-a-GCST90001887	rs78066273	CD28 on secreting '	3	113291855
ebi-a-GCST90001887	rs187188	CD28 on secreting '	3	55114197
ebi-a-GCST90001887	rs6766082	CD28 on secreting '	3	14637898
ebi-a-GCST90001887	rs1349999	CD28 on secreting '	4	15059988
ebi-a-GCST90001887	rs139007177	CD28 on secreting '	4	83560688
ebi-a-GCST90001887	rs76308995	CD28 on secreting '	5	125362860
ebi-a-GCST90001887	rs3757247	CD28 on secreting '	6	90957463
ebi-a-GCST90001887	rs144665636	CD28 on secreting '	8	51406956
ebi-a-GCST90001887	rs142599237	CD28 on secreting '	10	114914004
ebi-a-GCST90001887	rs4906123	CD28 on secreting '	14	101938382
ebi-a-GCST90001887	rs189158196	CD28 on secreting '	14	43725613
ebi-a-GCST90001887	rs2270288	CD28 on secreting '	16	8875529
ebi-a-GCST90001887	rs2341310	CD28 on secreting '	17	69802656
ebi-a-GCST90001887	rs144535716	CD28 on secreting '	18	64170750
ebi-a-GCST90001888	rs114072022	CD28 on CD39+ se	1	57351172
ebi-a-GCST90001888	rs141663136	CD28 on CD39+ se	2	3592725
ebi-a-GCST90001888	rs115086130	CD28 on CD39+ se	2	217086534
ebi-a-GCST90001888	rs114508332	CD28 on CD39+ se	2	163595996
ebi-a-GCST90001888	rs3116488	CD28 on CD39+ se	2	204586076
ebi-a-GCST90001888	rs115168681	CD28 on CD39+ se	2	92074020
ebi-a-GCST90001888	rs10205835	CD28 on CD39+ se	2	181320278
ebi-a-GCST90001888	rs142863502	CD28 on CD39+ se	3	78828398
ebi-a-GCST90001888	rs17462867	CD28 on CD39+ se	4	47665870
ebi-a-GCST90001888	rs2504703	CD28 on CD39+ se	6	51264082
ebi-a-GCST90001888	rs12778618	CD28 on CD39+ se	10	95633120
ebi-a-GCST90001888	rs112358299	CD28 on CD39+ se	10	95281076
ebi-a-GCST90001888	rs117458836	CD28 on CD39+ se	10	96773382
ebi-a-GCST90001888	rs10748649	CD28 on CD39+ se	10	97601703
ebi-a-GCST90001888	rs10882701	CD28 on CD39+ se	10	97741071
ebi-a-GCST90001888	rs80340485	CD28 on CD39+ se	12	15096323
ebi-a-GCST90001888	rs7301227	CD28 on CD39+ se	12	15687096
ebi-a-GCST90001888	rs9530273	CD28 on CD39+ se	13	74651013
ebi-a-GCST90001888	rs11017	CD28 on CD39+ se	15	75137252
ebi-a-GCST90001888	rs2071382	CD28 on CD39+ se	15	91428197
ebi-a-GCST90001888	rs61754918	CD28 on CD39+ se	16	89300425
ebi-a-GCST90001888	rs72873666	CD28 on CD39+ se	18	11480019
ebi-a-GCST90001888	rs6998	CD28 on CD39+ se	19	51363661
ebi-a-GCST90001888	rs71337061	CD28 on CD39+ se	19	1907674
ebi-a-GCST90001889	rs901666	CD28 on activated (	1	245092832

ebi-a-GCST90001889	rs62183989	CD28 on activated ‹	2	204609402
ebi-a-GCST90001889	rs116479405	CD28 on activated ‹	2	49884156
ebi-a-GCST90001889	rs145947216	CD28 on activated ‹	2	38596674
ebi-a-GCST90001889	rs4850102	CD28 on activated ‹	2	131733848
ebi-a-GCST90001889	rs34460712	CD28 on activated ‹	2	38935236
ebi-a-GCST90001889	rs72923074	CD28 on activated ‹	2	203112661
ebi-a-GCST90001889	rs6766082	CD28 on activated ‹	3	14637898
ebi-a-GCST90001889	rs358085	CD28 on activated ‹	3	55114477
ebi-a-GCST90001889	rs188669656	CD28 on activated ‹	4	134181609
ebi-a-GCST90001889	rs139007177	CD28 on activated ‹	4	83560688
ebi-a-GCST90001889	rs76308995	CD28 on activated ‹	5	125362860
ebi-a-GCST90001889	rs11755527	CD28 on activated ‹	6	90958231
ebi-a-GCST90001889	rs6942910	CD28 on activated ‹	7	38590558
ebi-a-GCST90001889	rs72639339	CD28 on activated ‹	8	37144660
ebi-a-GCST90001889	rs142519862	CD28 on activated ‹	8	51447942
ebi-a-GCST90001889	rs62542671	CD28 on activated ‹	9	27018375
ebi-a-GCST90001889	rs77013534	CD28 on activated ‹	9	26484227
ebi-a-GCST90001889	rs143888465	CD28 on activated ‹	12	52010108
ebi-a-GCST90001889	rs7304328	CD28 on activated ‹	12	100887301
ebi-a-GCST90001889	rs111860462	CD28 on activated ‹	14	82047636
ebi-a-GCST90001889	rs72764027	CD28 on activated ‹	15	96077835
ebi-a-GCST90001889	rs9806408	CD28 on activated ‹	15	101556948
ebi-a-GCST90001889	rs7226934	CD28 on activated ‹	18	76904665
ebi-a-GCST90001889	rs35493791	CD28 on activated ‹	20	59912066
ebi-a-GCST90001890	rs2922755	CD28 on CD45RA-	2	225917580
ebi-a-GCST90001890	rs115108917	CD28 on CD45RA-	2	173221757
ebi-a-GCST90001890	rs62184016	CD28 on CD45RA-	2	204623481
ebi-a-GCST90001890	rs13091429	CD28 on CD45RA-	3	118956779
ebi-a-GCST90001890	rs4959028	CD28 on CD45RA-	6	32383138
ebi-a-GCST90001890	rs4601112	CD28 on CD45RA-	6	32426271
ebi-a-GCST90001890	rs72928038	CD28 on CD45RA-	6	90976768
ebi-a-GCST90001890	rs138868484	CD28 on CD45RA-	9	22226319
ebi-a-GCST90001890	rs2804865	CD28 on CD45RA-	10	33808118
ebi-a-GCST90001890	rs112388922	CD28 on CD45RA-	12	106627038
ebi-a-GCST90001890	rs7305693	CD28 on CD45RA-	12	16767167
ebi-a-GCST90001890	rs140680300	CD28 on CD45RA-	14	40290794
ebi-a-GCST90001890	rs139057975	CD28 on CD45RA-	18	10278252
ebi-a-GCST90001890	rs118035444	CD28 on CD45RA-	19	56365335
ebi-a-GCST90001891	rs62174625	CD28 on CD45RA-	2	173772983
ebi-a-GCST90001891	rs13390252	CD28 on CD45RA-	2	204596729
ebi-a-GCST90001891	rs59639671	CD28 on CD45RA-	2	39003961
ebi-a-GCST90001891	rs72923074	CD28 on CD45RA-	2	203112661
ebi-a-GCST90001891	rs2889923	CD28 on CD45RA-	3	189596662
ebi-a-GCST90001891	rs28591324	CD28 on CD45RA-	4	29419166
ebi-a-GCST90001891	rs45553631	CD28 on CD45RA-	6	90981657
ebi-a-GCST90001891	rs62542710	CD28 on CD45RA-	9	27088757
ebi-a-GCST90001891	rs7872397	CD28 on CD45RA-	9	26516738
ebi-a-GCST90001891	rs2154080	CD28 on CD45RA-	10	130170037

ebi-a-GCST90001891	rs3133072	CD28 on CD45RA-	11	134196629
ebi-a-GCST90001891	rs60249166	CD28 on CD45RA-	13	32084901
ebi-a-GCST90001891	rs9529740	CD28 on CD45RA-	13	71009507
ebi-a-GCST90001891	rs368620678	CD28 on CD45RA-	14	105780514
ebi-a-GCST90001892	rs752641	CD28 on CD39+ C	1	156352013
ebi-a-GCST90001892	rs147613017	CD28 on CD39+ C	1	17764133
ebi-a-GCST90001892	rs7426056	CD28 on CD39+ C	2	204612058
ebi-a-GCST90001892	rs13170880	CD28 on CD39+ C	5	81137543
ebi-a-GCST90001892	rs78977206	CD28 on CD39+ C	5	125356459
ebi-a-GCST90001892	rs72709963	CD28 on CD39+ C	8	121934830
ebi-a-GCST90001892	rs117458836	CD28 on CD39+ C	10	96773382
ebi-a-GCST90001892	rs79881476	CD28 on CD39+ C	10	86422393
ebi-a-GCST90001892	rs10882701	CD28 on CD39+ C	10	97741071
ebi-a-GCST90001892	rs7084635	CD28 on CD39+ C	10	97062753
ebi-a-GCST90001892	rs12778618	CD28 on CD39+ C	10	95633120
ebi-a-GCST90001892	rs1539433	CD28 on CD39+ C	10	97519645
ebi-a-GCST90001892	rs144419206	CD28 on CD39+ C	11	24126713
ebi-a-GCST90001892	rs11227302	CD28 on CD39+ C	11	65554502
ebi-a-GCST90001892	rs143888465	CD28 on CD39+ C	12	52010108
ebi-a-GCST90001892	rs7960927	CD28 on CD39+ C	12	100894131
ebi-a-GCST90001892	rs11110796	CD28 on CD39+ C	12	101831141
ebi-a-GCST90001892	rs2427152	CD28 on CD39+ C	20	60089959
ebi-a-GCST90001892	rs34648998	CD28 on CD39+ C	20	21432927
ebi-a-GCST90001893	rs11210252	CD28 on CD28+ C	1	73919673
ebi-a-GCST90001893	rs56216383	CD28 on CD28+ C	2	204631712
ebi-a-GCST90001893	rs4674774	CD28 on CD28+ C	2	224128542
ebi-a-GCST90001893	rs2889923	CD28 on CD28+ C	3	189596662
ebi-a-GCST90001893	rs116778614	CD28 on CD28+ C	3	834951
ebi-a-GCST90001893	rs149008245	CD28 on CD28+ C	5	110580004
ebi-a-GCST90001893	rs7725278	CD28 on CD28+ C	5	174760947
ebi-a-GCST90001893	rs6459408	CD28 on CD28+ C	6	15582562
ebi-a-GCST90001893	rs9353524	CD28 on CD28+ C	6	88837616
ebi-a-GCST90001893	rs17714754	CD28 on CD28+ C	6	17754961
ebi-a-GCST90001893	rs45553631	CD28 on CD28+ C	6	90981657
ebi-a-GCST90001893	rs610195	CD28 on CD28+ C	9	27098490
ebi-a-GCST90001893	rs17056193	CD28 on CD28+ C	13	59815446
ebi-a-GCST90001893	rs4327031	CD28 on CD28+ C	15	101075569
ebi-a-GCST90001893	rs75520054	CD28 on CD28+ C	16	5568266
ebi-a-GCST90001893	rs117830609	CD28 on CD28+ C	17	71694056
ebi-a-GCST90001893	rs74859954	CD28 on CD28+ C	17	71726191
ebi-a-GCST90001893	rs1482537	CD28 on CD28+ C	18	65833632
ebi-a-GCST90001893	rs149149608	CD28 on CD28+ C	19	14599430
ebi-a-GCST90001894	rs138089143	CD28 on CD28+ C	1	32615715
ebi-a-GCST90001894	rs79296624	CD28 on CD28+ C	2	203870108
ebi-a-GCST90001894	rs13023055	CD28 on CD28+ C	2	20856144
ebi-a-GCST90001894	rs1973872	CD28 on CD28+ C	2	204631016
ebi-a-GCST90001894	rs360711	CD28 on CD28+ C	3	138691912
ebi-a-GCST90001894	rs7685596	CD28 on CD28+ C	4	8533012

ebi-a-GCST90001894	rs115478283	CD28 on CD28+ C	4	13704839
ebi-a-GCST90001894	rs116298583	CD28 on CD28+ C	4	24489778
ebi-a-GCST90001894	rs6836990	CD28 on CD28+ C	4	29416600
ebi-a-GCST90001894	rs77397796	CD28 on CD28+ C	5	5367152
ebi-a-GCST90001894	rs59159369	CD28 on CD28+ C	5	125344924
ebi-a-GCST90001894	rs3128990	CD28 on CD28+ C	6	29878483
ebi-a-GCST90001894	rs72928038	CD28 on CD28+ C	6	90976768
ebi-a-GCST90001894	rs6557390	CD28 on CD28+ C	6	155272013
ebi-a-GCST90001894	rs62458220	CD28 on CD28+ C	7	43252572
ebi-a-GCST90001894	rs55637775	CD28 on CD28+ C	8	72575417
ebi-a-GCST90001894	rs111970139	CD28 on CD28+ C	8	137644190
ebi-a-GCST90001894	rs62542710	CD28 on CD28+ C	9	27088757
ebi-a-GCST90001894	rs142599237	CD28 on CD28+ C	10	114914004
ebi-a-GCST90001894	rs17640797	CD28 on CD28+ C	11	56654147
ebi-a-GCST90001894	rs3133072	CD28 on CD28+ C	11	134196629
ebi-a-GCST90001894	rs11060804	CD28 on CD28+ C	12	130767902
ebi-a-GCST90001894	rs117505260	CD28 on CD28+ C	13	113724248
ebi-a-GCST90001894	rs9516901	CD28 on CD28+ C	13	97958649
ebi-a-GCST90001894	rs1123285	CD28 on CD28+ C	14	57274519
ebi-a-GCST90001894	rs16954045	CD28 on CD28+ C	15	70299168
ebi-a-GCST90001894	rs998490	CD28 on CD28+ C	21	16860484
ebi-a-GCST90001894	rs5751902	CD28 on CD28+ C	22	24996630
ebi-a-GCST90001895	rs873041	CD28 on CD28+ D	1	29718378
ebi-a-GCST90001895	rs79457820	CD28 on CD28+ D	1	178098674
ebi-a-GCST90001895	rs2992756	CD28 on CD28+ D	1	18807339
ebi-a-GCST90001896	rs75321567	CD28 on CD28+ C	1	231523742
ebi-a-GCST90001896	rs3845728	CD28 on CD28+ C	2	169704333
ebi-a-GCST90001896	rs788008	CD28 on CD28+ C	2	198232639
ebi-a-GCST90001896	rs112581826	CD28 on CD28+ C	2	204650296
ebi-a-GCST90001896	rs78307830	CD28 on CD28+ C	2	204596768
ebi-a-GCST90001896	rs74321916	CD28 on CD28+ C	3	148125567
ebi-a-GCST90001896	rs13147958	CD28 on CD28+ C	4	187705839
ebi-a-GCST90001896	rs10447212	CD28 on CD28+ C	5	155756837
ebi-a-GCST90001896	rs72820300	CD28 on CD28+ C	6	9342441
ebi-a-GCST90001896	rs2734904	CD28 on CD28+ C	6	29909840
ebi-a-GCST90001896	rs72928038	CD28 on CD28+ C	6	90976768
ebi-a-GCST90001896	rs17521485	CD28 on CD28+ C	6	91423027
ebi-a-GCST90001896	rs4747821	CD28 on CD28+ C	10	9270831
ebi-a-GCST90001896	rs79921170	CD28 on CD28+ C	15	68612190
ebi-a-GCST90001896	rs11074594	CD28 on CD28+ C	16	23973371
ebi-a-GCST90001896	rs28421958	CD28 on CD28+ C	19	8097404
ebi-a-GCST90001897	rs3116493	CD28 on CD39+ C	2	204589281
ebi-a-GCST90001897	rs77594357	CD28 on CD39+ C	2	204328474
ebi-a-GCST90001897	rs17490604	CD28 on CD39+ C	4	11450961
ebi-a-GCST90001897	rs187222855	CD28 on CD39+ C	5	71850168
ebi-a-GCST90001897	rs77213229	CD28 on CD39+ C	7	139869032
ebi-a-GCST90001897	rs10237725	CD28 on CD39+ C	7	30558616
ebi-a-GCST90001897	rs79635002	CD28 on CD39+ C	9	9466703

ebi-a-GCST90001897	rs62533434	CD28 on CD39+ C	9	25711618
ebi-a-GCST90001897	rs116972489	CD28 on CD39+ C	10	69651954
ebi-a-GCST90001897	rs139513469	CD28 on CD39+ C	10	66332109
ebi-a-GCST90001897	rs7088961	CD28 on CD39+ C	10	97608114
ebi-a-GCST90001897	rs10847448	CD28 on CD39+ C	12	128258849
ebi-a-GCST90001897	rs143273680	CD28 on CD39+ C	12	14071670
ebi-a-GCST90001897	rs902972	CD28 on CD39+ C	12	62608096
ebi-a-GCST90001897	rs7177745	CD28 on CD39+ C	15	78148518
ebi-a-GCST90001897	rs11070382	CD28 on CD39+ C	15	43151911
ebi-a-GCST90001897	rs151077900	CD28 on CD39+ C	16	1037963
ebi-a-GCST90001897	rs75380201	CD28 on CD39+ C	18	39778311
ebi-a-GCST90001897	rs148345105	CD28 on CD39+ C	18	14441608
ebi-a-GCST90001897	rs72871333	CD28 on CD39+ C	18	4638106
ebi-a-GCST90001897	rs2028827	CD28 on CD39+ C	19	4024020
ebi-a-GCST90001897	rs624763	CD28 on CD39+ C	21	40902905
ebi-a-GCST90001898	rs35771002	CD28 on CD4+	1	117276978
ebi-a-GCST90001898	rs138089143	CD28 on CD4+	1	32615715
ebi-a-GCST90001898	rs1973872	CD28 on CD4+	2	204631016
ebi-a-GCST90001898	rs7423567	CD28 on CD4+	2	204378226
ebi-a-GCST90001898	rs4541428	CD28 on CD4+	3	20399142
ebi-a-GCST90001898	rs16823817	CD28 on CD4+	3	115239407
ebi-a-GCST90001898	rs141494298	CD28 on CD4+	3	190052936
ebi-a-GCST90001898	rs62273311	CD28 on CD4+	3	167771624
ebi-a-GCST90001898	rs28572519	CD28 on CD4+	4	10975237
ebi-a-GCST90001898	rs115478283	CD28 on CD4+	4	13704839
ebi-a-GCST90001898	rs28591324	CD28 on CD4+	4	29419166
ebi-a-GCST90001898	rs7656433	CD28 on CD4+	4	155510224
ebi-a-GCST90001898	rs59159369	CD28 on CD4+	5	125344924
ebi-a-GCST90001898	rs77397796	CD28 on CD4+	5	5367152
ebi-a-GCST90001898	rs72928038	CD28 on CD4+	6	90976768
ebi-a-GCST90001898	rs62458220	CD28 on CD4+	7	43252572
ebi-a-GCST90001898	rs2977738	CD28 on CD4+	8	72600833
ebi-a-GCST90001898	rs62647604	CD28 on CD4+	11	25673248
ebi-a-GCST90001898	rs3133072	CD28 on CD4+	11	134196629
ebi-a-GCST90001898	rs17640797	CD28 on CD4+	11	56654147
ebi-a-GCST90001898	rs10848070	CD28 on CD4+	12	130784674
ebi-a-GCST90001898	rs78883235	CD28 on CD4+	13	39737161
ebi-a-GCST90001898	rs9516901	CD28 on CD4+	13	97958649
ebi-a-GCST90001898	rs4779516	CD28 on CD4+	15	31625040
ebi-a-GCST90001898	rs56764046	CD28 on CD4+	15	96648129
ebi-a-GCST90001898	rs16954045	CD28 on CD4+	15	70299168
ebi-a-GCST90001898	rs998490	CD28 on CD4+	21	16860484
ebi-a-GCST90001898	rs5751902	CD28 on CD4+	22	24996630
ebi-a-GCST90001899	rs901666	CD28 on CD4 Treg	1	245092832
ebi-a-GCST90001899	rs7552165	CD28 on CD4 Treg	1	16461336
ebi-a-GCST90001899	rs72887430	CD28 on CD4 Treg	2	46601171
ebi-a-GCST90001899	rs56209416	CD28 on CD4 Treg	2	204627500
ebi-a-GCST90001899	rs13017710	CD28 on CD4 Treg	2	131710625

ebi-a-GCST90001899	rs6722943	CD28 on CD4 Treg	2	121025601
ebi-a-GCST90001899	rs12152264	CD28 on CD4 Treg	3	54889054
ebi-a-GCST90001899	rs6840739	CD28 on CD4 Treg	4	55359146
ebi-a-GCST90001899	rs77455008	CD28 on CD4 Treg	5	66383812
ebi-a-GCST90001899	rs11755527	CD28 on CD4 Treg	6	90958231
ebi-a-GCST90001899	rs79907437	CD28 on CD4 Treg	7	42401568
ebi-a-GCST90001899	rs547142523	CD28 on CD4 Treg	7	106446376
ebi-a-GCST90001899	rs11990425	CD28 on CD4 Treg	8	21557285
ebi-a-GCST90001899	rs145288523	CD28 on CD4 Treg	9	21854434
ebi-a-GCST90001899	rs142599237	CD28 on CD4 Treg	10	114914004
ebi-a-GCST90001899	rs11246934	CD28 on CD4 Treg	12	132036029
ebi-a-GCST90001899	rs143888465	CD28 on CD4 Treg	12	52010108
ebi-a-GCST90001899	rs9603530	CD28 on CD4 Treg	13	39958972
ebi-a-GCST90001899	rs189158196	CD28 on CD4 Treg	14	43725613
ebi-a-GCST90001899	rs111860462	CD28 on CD4 Treg	14	82047636
ebi-a-GCST90001899	rs758407	CD28 on CD4 Treg	17	32944070
ebi-a-GCST90001899	rs8066746	CD28 on CD4 Treg	17	4151820
ebi-a-GCST90001899	rs117868196	CD28 on CD4 Treg	17	3576181
ebi-a-GCST90001899	rs6142835	CD28 on CD4 Treg	20	59979495
ebi-a-GCST90001899	rs78859673	CD28 on CD4 Treg	21	16844033
ebi-a-GCST90001900	rs62184016	CD28 on resting Tr	2	204623481
ebi-a-GCST90001900	rs112900587	CD28 on resting Tr	2	195082880
ebi-a-GCST90001900	rs138480662	CD28 on resting Tr	2	205918886
ebi-a-GCST90001900	rs72923091	CD28 on resting Tr	2	203143683
ebi-a-GCST90001900	rs79223025	CD28 on resting Tr	3	147829547
ebi-a-GCST90001900	rs7665484	CD28 on resting Tr	4	167499125
ebi-a-GCST90001901	rs141247369	CD28 on CD39+ re	1	3272937
ebi-a-GCST90001901	rs114565907	CD28 on CD39+ re	1	177284570
ebi-a-GCST90001901	rs12068559	CD28 on CD39+ re	1	6127983
ebi-a-GCST90001901	rs115841111	CD28 on CD39+ re	1	242105339
ebi-a-GCST90001901	rs13404978	CD28 on CD39+ re	2	204653886
ebi-a-GCST90001901	rs12614511	CD28 on CD39+ re	2	39471924
ebi-a-GCST90001901	rs114550156	CD28 on CD39+ re	4	129186460
ebi-a-GCST90001901	rs78365755	CD28 on CD39+ re	6	13328984
ebi-a-GCST90001901	rs4707609	CD28 on CD39+ re	6	90946479
ebi-a-GCST90001901	rs623219	CD28 on CD39+ re	6	137512886
ebi-a-GCST90001901	rs7846567	CD28 on CD39+ re	8	143612149
ebi-a-GCST90001901	rs117957417	CD28 on CD39+ re	10	96597621
ebi-a-GCST90001901	rs284615	CD28 on CD39+ re	10	61151603
ebi-a-GCST90001901	rs118122984	CD28 on CD39+ re	10	122084798
ebi-a-GCST90001901	rs117653391	CD28 on CD39+ re	10	97551952
ebi-a-GCST90001901	rs138199636	CD28 on CD39+ re	11	37444749
ebi-a-GCST90001901	rs17107510	CD28 on CD39+ re	12	70389055
ebi-a-GCST90001901	rs182187758	CD28 on CD39+ re	13	80391747
ebi-a-GCST90001901	rs447600	CD28 on CD39+ re	14	48325261
ebi-a-GCST90001901	rs114579738	CD28 on CD39+ re	15	87662869
ebi-a-GCST90001901	rs117389073	CD28 on CD39+ re	16	634475
ebi-a-GCST90001901	rs28921682	CD28 on CD39+ re	17	11968129

ebi-a-GCST90001901	rs6008485	CD28 on CD39+ re	22	48371416
ebi-a-GCST90001902	rs9288325	CD28 on activated '	2	202946868
ebi-a-GCST90001902	rs62183989	CD28 on activated '	2	204609402
ebi-a-GCST90001902	rs12713441	CD28 on activated '	2	21590196
ebi-a-GCST90001902	rs55930426	CD28 on activated '	2	46222978
ebi-a-GCST90001902	rs145947216	CD28 on activated '	2	38596674
ebi-a-GCST90001902	rs72968907	CD28 on activated '	3	123701658
ebi-a-GCST90001902	rs468118	CD28 on activated '	3	55110241
ebi-a-GCST90001902	rs830643	CD28 on activated '	3	71667063
ebi-a-GCST90001902	rs6840739	CD28 on activated '	4	55359146
ebi-a-GCST90001902	rs76308995	CD28 on activated '	5	125362860
ebi-a-GCST90001902	rs11755527	CD28 on activated '	6	90958231
ebi-a-GCST90001902	rs6942910	CD28 on activated '	7	38590558
ebi-a-GCST90001902	rs72639339	CD28 on activated '	8	37144660
ebi-a-GCST90001902	rs4877468	CD28 on activated '	9	91258912
ebi-a-GCST90001902	rs143888465	CD28 on activated '	12	52010108
ebi-a-GCST90001902	rs7304328	CD28 on activated '	12	100887301
ebi-a-GCST90001902	rs111860462	CD28 on activated '	14	82047636
ebi-a-GCST90001902	rs7143529	CD28 on activated '	14	92878020
ebi-a-GCST90001902	rs72764027	CD28 on activated '	15	96077835
ebi-a-GCST90001902	rs137875117	CD28 on activated '	16	25627987
ebi-a-GCST90001903	rs71632979	CD86 on myeloid D	1	161526397
ebi-a-GCST90001903	rs71639910	CD86 on myeloid D	1	161937774
ebi-a-GCST90001903	rs115805162	CD86 on myeloid D	1	161410253
ebi-a-GCST90001903	rs11240608	CD86 on myeloid D	1	203575887
ebi-a-GCST90001903	rs6434686	CD86 on myeloid D	2	154380301
ebi-a-GCST90001903	rs7591875	CD86 on myeloid D	2	118023763
ebi-a-GCST90001903	rs76114682	CD86 on myeloid D	2	234940795
ebi-a-GCST90001903	rs59167197	CD86 on myeloid D	3	181521530
ebi-a-GCST90001903	rs2029226	CD86 on myeloid D	4	111255086
ebi-a-GCST90001903	rs138596085	CD86 on myeloid D	4	22730743
ebi-a-GCST90001903	rs72822776	CD86 on myeloid D	5	174078104
ebi-a-GCST90001903	rs13232168	CD86 on myeloid D	7	155165806
ebi-a-GCST90001903	rs79989067	CD86 on myeloid D	9	85770092
ebi-a-GCST90001903	rs1411982	CD86 on myeloid D	9	89242001
ebi-a-GCST90001903	rs4750846	CD86 on myeloid D	10	132853310
ebi-a-GCST90001903	rs11611231	CD86 on myeloid D	12	974326
ebi-a-GCST90001903	rs143271161	CD86 on myeloid D	15	35493491
ebi-a-GCST90001903	rs7194233	CD86 on myeloid D	16	64116331
ebi-a-GCST90001903	rs56372685	CD86 on myeloid D	19	18091720
ebi-a-GCST90001903	rs186130451	CD86 on myeloid D	20	3457280
ebi-a-GCST90001903	rs6034780	CD86 on myeloid D	20	17231638
ebi-a-GCST90001903	rs78643996	CD86 on myeloid D	20	55639856
ebi-a-GCST90001903	rs8127024	CD86 on myeloid D	21	38835288
ebi-a-GCST90001904	rs12076726	CD86 on CD62L+ r	1	38545541
ebi-a-GCST90001904	rs151105922	CD86 on CD62L+ r	2	225124699
ebi-a-GCST90001904	rs143092602	CD86 on CD62L+ r	2	177306191
ebi-a-GCST90001904	rs112932872	CD86 on CD62L+ r	2	41526880

ebi-a-GCST90001904	rs9848900	CD86 on CD62L+ ı	3	121831497
ebi-a-GCST90001904	rs9834116	CD86 on CD62L+ ı	3	186214151
ebi-a-GCST90001904	rs6830352	CD86 on CD62L+ ı	4	30425631
ebi-a-GCST90001904	rs76831090	CD86 on CD62L+ ı	4	165317503
ebi-a-GCST90001904	rs116499957	CD86 on CD62L+ ı	4	23334338
ebi-a-GCST90001904	rs112834744	CD86 on CD62L+ ı	4	166364132
ebi-a-GCST90001904	rs116105787	CD86 on CD62L+ ı	4	167699242
ebi-a-GCST90001904	rs139387290	CD86 on CD62L+ ı	7	145251741
ebi-a-GCST90001904	rs62469564	CD86 on CD62L+ ı	7	95016032
ebi-a-GCST90001904	rs72771961	CD86 on CD62L+ ı	9	138859840
ebi-a-GCST90001904	rs759125	CD86 on CD62L+ ı	9	123041996
ebi-a-GCST90001904	rs2105590	CD86 on CD62L+ ı	11	117925668
ebi-a-GCST90001904	rs7307939	CD86 on CD62L+ ı	12	131440935
ebi-a-GCST90001904	rs2737282	CD86 on CD62L+ ı	16	78904218
ebi-a-GCST90001904	rs56286676	CD86 on CD62L+ ı	17	49364140
ebi-a-GCST90001904	rs55989590	CD86 on CD62L+ ı	18	52521534
ebi-a-GCST90001904	rs112891649	CD86 on CD62L+ ı	19	23466054
ebi-a-GCST90001904	rs2835746	CD86 on CD62L+ ı	21	38815830
ebi-a-GCST90001904	rs76607076	CD86 on CD62L+ ı	22	25087059
ebi-a-GCST90001905	rs150649461	CD86 on monocyte	1	92925654
ebi-a-GCST90001905	rs72663570	CD86 on monocyte	1	40095534
ebi-a-GCST90001905	rs7565243	CD86 on monocyte	2	15261044
ebi-a-GCST90001905	rs10169719	CD86 on monocyte	2	124939359
ebi-a-GCST90001905	rs61300354	CD86 on monocyte	3	132156322
ebi-a-GCST90001905	rs116524758	CD86 on monocyte	3	118271900
ebi-a-GCST90001905	rs6438880	CD86 on monocyte	3	124834289
ebi-a-GCST90001905	rs143443965	CD86 on monocyte	8	142893697
ebi-a-GCST90001905	rs6559641	CD86 on monocyte	9	84351351
ebi-a-GCST90001905	rs56283543	CD86 on monocyte	9	89798284
ebi-a-GCST90001905	rs7857137	CD86 on monocyte	9	72328727
ebi-a-GCST90001905	rs117561616	CD86 on monocyte	10	45720975
ebi-a-GCST90001905	rs150328798	CD86 on monocyte	11	123151235
ebi-a-GCST90001905	rs74907279	CD86 on monocyte	12	24562705
ebi-a-GCST90001905	rs73592377	CD86 on monocyte	12	658993
ebi-a-GCST90001905	rs12974366	CD86 on monocyte	19	46380610
ebi-a-GCST90001905	rs138552617	CD86 on monocyte	19	47321626
ebi-a-GCST90001906	rs138095875	CD86 on granulocy	3	98484408
ebi-a-GCST90001906	rs7663443	CD86 on granulocy	4	15308694
ebi-a-GCST90001906	rs187015	CD86 on granulocy	4	60840845
ebi-a-GCST90001906	rs74336201	CD86 on granulocy	6	83240504
ebi-a-GCST90001906	rs915125	CD86 on granulocy	6	82463376
ebi-a-GCST90001906	rs445	CD86 on granulocy	7	92408370
ebi-a-GCST90001906	rs7002813	CD86 on granulocy	8	6903815
ebi-a-GCST90001906	rs12675298	CD86 on granulocy	8	6828813
ebi-a-GCST90001906	rs62493338	CD86 on granulocy	8	14775908
ebi-a-GCST90001906	rs62546664	CD86 on granulocy	9	26584433
ebi-a-GCST90001906	rs117974128	CD86 on granulocy	11	100371319
ebi-a-GCST90001906	rs17473967	CD86 on granulocy	12	23886853

ebi-a-GCST90001906	rs17613315	CD86 on granulocy	13	59609619
ebi-a-GCST90001906	rs147621180	CD86 on granulocy	16	4475064
ebi-a-GCST90001906	rs139095969	CD86 on granulocy	17	55921141
ebi-a-GCST90001906	rs886929	CD86 on granulocy	17	55800643
ebi-a-GCST90001906	rs11653491	CD86 on granulocy	17	67953507
ebi-a-GCST90001906	rs114119279	CD86 on granulocy	18	52230593
ebi-a-GCST90001906	rs62109982	CD86 on granulocy	19	28207523
ebi-a-GCST90001906	rs6123226	CD86 on granulocy	20	51523530
ebi-a-GCST90001906	rs951076	CD86 on granulocy	21	21580019
ebi-a-GCST90001907	rs28668175	CCR7 on naive CD	1	26653983
ebi-a-GCST90001907	rs1515251	CCR7 on naive CD	2	146831133
ebi-a-GCST90001907	rs7565507	CCR7 on naive CD	2	39767074
ebi-a-GCST90001907	rs13013272	CCR7 on naive CD	2	75011686
ebi-a-GCST90001907	rs62230160	CCR7 on naive CD	3	3218066
ebi-a-GCST90001907	rs6533756	CCR7 on naive CD	4	79458537
ebi-a-GCST90001907	rs72732720	CCR7 on naive CD	5	38890571
ebi-a-GCST90001907	rs77025222	CCR7 on naive CD	8	26160128
ebi-a-GCST90001907	rs140136541	CCR7 on naive CD	9	92824281
ebi-a-GCST90001907	rs12217994	CCR7 on naive CD	10	45253052
ebi-a-GCST90001907	rs61968809	CCR7 on naive CD	13	106399307
ebi-a-GCST90001907	rs12593799	CCR7 on naive CD	15	82392146
ebi-a-GCST90001907	rs5020353	CCR7 on naive CD	16	423423
ebi-a-GCST90001907	rs9934320	CCR7 on naive CD	16	79755966
ebi-a-GCST90001907	rs2228015	CCR7 on naive CD	17	38715186
ebi-a-GCST90001907	rs75983909	CCR7 on naive CD	17	38723925
ebi-a-GCST90001907	rs76868109	CCR7 on naive CD	17	41776943
ebi-a-GCST90001907	rs189899503	CCR7 on naive CD	18	74173063
ebi-a-GCST90001907	rs114153301	CCR7 on naive CD	18	66074898
ebi-a-GCST90001907	rs11665125	CCR7 on naive CD	18	33295639
ebi-a-GCST90001907	rs355325	CCR7 on naive CD	18	33070491
ebi-a-GCST90001907	rs17833525	CCR7 on naive CD	18	72546030
ebi-a-GCST90001907	rs6076512	CCR7 on naive CD	20	3347297
ebi-a-GCST90001907	rs185134274	CCR7 on naive CD	21	45395803
ebi-a-GCST90001907	rs9614090	CCR7 on naive CD	22	30269907
ebi-a-GCST90001907	rs111855096	CCR7 on naive CD	22	48693531
ebi-a-GCST90001908	rs146790974	CCR7 on naive CD	2	152246402
ebi-a-GCST90001908	rs7629505	CCR7 on naive CD	3	31921624
ebi-a-GCST90001908	rs2204128	CCR7 on naive CD	3	61688122
ebi-a-GCST90001908	rs187179931	CCR7 on naive CD	4	138000655
ebi-a-GCST90001908	rs114222516	CCR7 on naive CD	4	137459015
ebi-a-GCST90001908	rs188524489	CCR7 on naive CD	4	65000122
ebi-a-GCST90001908	rs460267	CCR7 on naive CD	5	76722891
ebi-a-GCST90001908	rs146494321	CCR7 on naive CD	5	32490061
ebi-a-GCST90001908	rs6925194	CCR7 on naive CD	6	156369631
ebi-a-GCST90001908	rs3806092	CCR7 on naive CD	6	70982817
ebi-a-GCST90001908	rs112171230	CCR7 on naive CD	7	70434581
ebi-a-GCST90001908	rs13261112	CCR7 on naive CD	8	82954367
ebi-a-GCST90001908	rs842297	CCR7 on naive CD	9	7722331

ebi-a-GCST90001908	rs13295814	CCR7 on naive CD8	9	35969765
ebi-a-GCST90001908	rs7924111	CCR7 on naive CD8	10	3161871
ebi-a-GCST90001908	rs12593799	CCR7 on naive CD8	15	82392146
ebi-a-GCST90001908	rs56043797	CCR7 on naive CD8	15	76068559
ebi-a-GCST90001908	rs139036545	CCR7 on naive CD8	16	86333892
ebi-a-GCST90001908	rs1492564	CCR7 on naive CD8	16	73774321
ebi-a-GCST90001908	rs116872842	CCR7 on naive CD8	16	51249917
ebi-a-GCST90001908	rs5020353	CCR7 on naive CD8	16	423423
ebi-a-GCST90001908	rs2228015	CCR7 on naive CD8	17	38715186
ebi-a-GCST90001908	rs8179096	CCR7 on naive CD8	17	76921649
ebi-a-GCST90001908	rs143859887	CCR7 on naive CD8	20	58399063
ebi-a-GCST90001908	rs9614090	CCR7 on naive CD8	22	30269907
ebi-a-GCST90001909	rs267708	CD45 on CD14+ m	1	30445631
ebi-a-GCST90001909	rs12131677	CD45 on CD14+ m	1	90315734
ebi-a-GCST90001909	rs142767469	CD45 on CD14+ m	5	26288128
ebi-a-GCST90001909	rs6884558	CD45 on CD14+ m	5	124281556
ebi-a-GCST90001909	rs34858919	CD45 on CD14+ m	7	66829653
ebi-a-GCST90001909	rs74798265	CD45 on CD14+ m	7	29493428
ebi-a-GCST90001909	rs7005676	CD45 on CD14+ m	8	134449939
ebi-a-GCST90001909	rs117509040	CD45 on CD14+ m	8	93803650
ebi-a-GCST90001909	rs11141907	CD45 on CD14+ m	9	90213762
ebi-a-GCST90001909	rs182879147	CD45 on CD14+ m	9	127081265
ebi-a-GCST90001909	rs113853911	CD45 on CD14+ m	11	5263771
ebi-a-GCST90001909	rs12364299	CD45 on CD14+ m	11	5403108
ebi-a-GCST90001909	rs2511241	CD45 on CD14+ m	11	72945341
ebi-a-GCST90001909	rs79265501	CD45 on CD14+ m	11	5747642
ebi-a-GCST90001909	rs7323586	CD45 on CD14+ m	13	113478654
ebi-a-GCST90001909	rs58552272	CD45 on CD14+ m	16	10871095
ebi-a-GCST90001909	rs867396	CD45 on CD14+ m	17	79167116
ebi-a-GCST90001909	rs77687616	CD45 on CD14+ m	20	54392972
ebi-a-GCST90001910	rs559835585	CD45 on B cell	1	237699750
ebi-a-GCST90001910	rs4952746	CD45 on B cell	2	45397753
ebi-a-GCST90001910	rs111395861	CD45 on B cell	2	14940150
ebi-a-GCST90001910	rs2517515	CD45 on B cell	6	31029258
ebi-a-GCST90001910	rs819501	CD45 on B cell	6	47012997
ebi-a-GCST90001910	rs17613271	CD45 on B cell	6	32621778
ebi-a-GCST90001910	rs118055407	CD45 on B cell	7	134999862
ebi-a-GCST90001910	rs13244416	CD45 on B cell	7	98408686
ebi-a-GCST90001910	rs149349110	CD45 on B cell	7	289399
ebi-a-GCST90001910	rs4442111	CD45 on B cell	8	99120383
ebi-a-GCST90001910	rs11830137	CD45 on B cell	12	10379272
ebi-a-GCST90001910	rs7332754	CD45 on B cell	13	106729299
ebi-a-GCST90001910	rs142266193	CD45 on B cell	13	30673693
ebi-a-GCST90001910	rs2440320	CD45 on B cell	15	53075734
ebi-a-GCST90001910	rs11637245	CD45 on B cell	15	40846615
ebi-a-GCST90001910	rs12939172	CD45 on B cell	17	14526450
ebi-a-GCST90001910	rs80231522	CD45 on B cell	17	52584481
ebi-a-GCST90001910	rs138082872	CD45 on B cell	19	44344058

ebi-a-GCST90001910	rs5750829	CD45 on B cell	22	39838018
ebi-a-GCST90001911	rs10917571	CD45 on NK	1	161519411
ebi-a-GCST90001911	rs183053322	CD45 on NK	1	25076059
ebi-a-GCST90001911	rs75312377	CD45 on NK	1	24836544
ebi-a-GCST90001911	rs6702066	CD45 on NK	1	29711271
ebi-a-GCST90001911	rs2377027	CD45 on NK	1	226420536
ebi-a-GCST90001911	rs150065473	CD45 on NK	1	158359489
ebi-a-GCST90001911	rs181102707	CD45 on NK	1	198147822
ebi-a-GCST90001911	rs2163548	CD45 on NK	2	201226862
ebi-a-GCST90001911	rs6721978	CD45 on NK	2	232290157
ebi-a-GCST90001911	rs142823250	CD45 on NK	3	17817424
ebi-a-GCST90001911	rs2331416	CD45 on NK	4	69492629
ebi-a-GCST90001911	rs72756114	CD45 on NK	5	54862828
ebi-a-GCST90001911	rs72502555	CD45 on NK	6	31234208
ebi-a-GCST90001911	rs190229330	CD45 on NK	6	82039242
ebi-a-GCST90001911	rs117491214	CD45 on NK	7	26905913
ebi-a-GCST90001911	rs34664922	CD45 on NK	7	56100176
ebi-a-GCST90001911	rs71576013	CD45 on NK	8	134855565
ebi-a-GCST90001911	rs149803985	CD45 on NK	8	110654552
ebi-a-GCST90001911	rs3780495	CD45 on NK	9	33125942
ebi-a-GCST90001911	rs150425620	CD45 on NK	9	72572853
ebi-a-GCST90001911	rs10781534	CD45 on NK	9	139933589
ebi-a-GCST90001911	rs1182654	CD45 on NK	9	19731896
ebi-a-GCST90001911	rs10882100	CD45 on NK	10	94460687
ebi-a-GCST90001911	rs751514	CD45 on NK	10	126366931
ebi-a-GCST90001911	rs11613388	CD45 on NK	12	54342563
ebi-a-GCST90001911	rs72677702	CD45 on NK	14	40030520
ebi-a-GCST90001911	rs146515193	CD45 on NK	15	69715791
ebi-a-GCST90001911	rs12593800	CD45 on NK	15	79575521
ebi-a-GCST90001911	rs12928699	CD45 on NK	16	5027948
ebi-a-GCST90001911	rs4327090	CD45 on NK	17	43902541
ebi-a-GCST90001911	rs77592206	CD45 on NK	22	39163362
ebi-a-GCST90001912	rs2495272	CD45 on HLA DR+	1	2897465
ebi-a-GCST90001912	rs13096090	CD45 on HLA DR+	3	197271476
ebi-a-GCST90001912	rs11129873	CD45 on HLA DR+	3	40295706
ebi-a-GCST90001912	rs7698455	CD45 on HLA DR+	4	6690481
ebi-a-GCST90001912	rs114669227	CD45 on HLA DR+	5	101613375
ebi-a-GCST90001912	rs142947331	CD45 on HLA DR+	6	13780175
ebi-a-GCST90001912	rs116881034	CD45 on HLA DR+	7	37661413
ebi-a-GCST90001912	rs78892713	CD45 on HLA DR+	9	77011032
ebi-a-GCST90001912	rs12772032	CD45 on HLA DR+	10	125376027
ebi-a-GCST90001912	rs7097637	CD45 on HLA DR+	10	129339038
ebi-a-GCST90001912	rs7482118	CD45 on HLA DR+	11	972603
ebi-a-GCST90001912	rs1860345	CD45 on HLA DR+	12	4910812
ebi-a-GCST90001912	rs117580642	CD45 on HLA DR+	18	9097811
ebi-a-GCST90001913	rs77353040	CD45 on granulocy	1	92438297
ebi-a-GCST90001913	rs708772	CD45 on granulocy	1	226912891
ebi-a-GCST90001913	rs2070901	CD45 on granulocy	1	161185058

ebi-a-GCST90001913	rs76564275	CD45 on granulocy	1	198077256
ebi-a-GCST90001913	rs12120358	CD45 on granulocy	1	161444369
ebi-a-GCST90001913	rs141251504	CD45 on granulocy	2	106554170
ebi-a-GCST90001913	rs79009131	CD45 on granulocy	5	9930209
ebi-a-GCST90001913	rs76530868	CD45 on granulocy	6	31275410
ebi-a-GCST90001913	rs2523857	CD45 on granulocy	6	31021504
ebi-a-GCST90001913	rs79200728	CD45 on granulocy	8	2082753
ebi-a-GCST90001913	rs4838178	CD45 on granulocy	9	127220635
ebi-a-GCST90001913	rs77547877	CD45 on granulocy	9	72613948
ebi-a-GCST90001913	rs12364299	CD45 on granulocy	11	5403108
ebi-a-GCST90001913	rs188470738	CD45 on granulocy	18	3276401
ebi-a-GCST90001913	rs11084094	CD45 on granulocy	19	52119546
ebi-a-GCST90001913	rs138030758	CD45 on granulocy	20	7731470
ebi-a-GCST90001913	rs117414770	CD45 on granulocy	20	40628317
ebi-a-GCST90001914	rs147201606	CD45 on lymphocy	1	30780553
ebi-a-GCST90001914	rs75643253	CD45 on lymphocy	1	27415326
ebi-a-GCST90001914	rs9434622	CD45 on lymphocy	1	8205085
ebi-a-GCST90001914	rs75954342	CD45 on lymphocy	2	98567907
ebi-a-GCST90001914	rs11894144	CD45 on lymphocy	2	166034305
ebi-a-GCST90001914	rs143934712	CD45 on lymphocy	3	75826490
ebi-a-GCST90001914	rs4073271	CD45 on lymphocy	4	1573141
ebi-a-GCST90001914	rs3778139	CD45 on lymphocy	6	129467732
ebi-a-GCST90001914	rs73160264	CD45 on lymphocy	7	132417677
ebi-a-GCST90001914	rs118021489	CD45 on lymphocy	7	136665573
ebi-a-GCST90001914	rs2517278	CD45 on lymphocy	8	17441115
ebi-a-GCST90001914	rs34441251	CD45 on lymphocy	9	17678842
ebi-a-GCST90001914	rs3740869	CD45 on lymphocy	11	106002997
ebi-a-GCST90001914	rs7139068	CD45 on lymphocy	12	53597092
ebi-a-GCST90001914	rs117527144	CD45 on lymphocy	12	3303278
ebi-a-GCST90001914	rs12882220	CD45 on lymphocy	14	24869512
ebi-a-GCST90001914	rs62002014	CD45 on lymphocy	15	25371646
ebi-a-GCST90001914	rs8040122	CD45 on lymphocy	15	86809852
ebi-a-GCST90001914	rs17307464	CD45 on lymphocy	16	70755144
ebi-a-GCST90001914	rs75928238	CD45 on lymphocy	16	21089895
ebi-a-GCST90001914	rs34291514	CD45 on lymphocy	17	1058299
ebi-a-GCST90001914	rs191772295	CD45 on lymphocy	18	21222946
ebi-a-GCST90001914	rs6129733	CD45 on lymphocy	20	39662225
ebi-a-GCST90001914	rs5763842	CD45 on lymphocy	22	30568738
ebi-a-GCST90001915	rs147201606	CD45 on T cell	1	30780553
ebi-a-GCST90001915	rs115861687	CD45 on T cell	1	42697317
ebi-a-GCST90001915	rs77025195	CD45 on T cell	1	172481043
ebi-a-GCST90001915	rs469141	CD45 on T cell	1	91546799
ebi-a-GCST90001915	rs41317509	CD45 on T cell	1	186159947
ebi-a-GCST90001915	rs11676095	CD45 on T cell	2	206656142
ebi-a-GCST90001915	rs3778139	CD45 on T cell	6	129467732
ebi-a-GCST90001915	rs142492586	CD45 on T cell	6	33202756
ebi-a-GCST90001915	rs7744304	CD45 on T cell	6	32425487
ebi-a-GCST90001915	rs34441251	CD45 on T cell	9	17678842

ebi-a-GCST90001915	rs7074916	CD45 on T cell	10	8256695
ebi-a-GCST90001915	rs7952636	CD45 on T cell	11	103495231
ebi-a-GCST90001915	rs17307464	CD45 on T cell	16	70755144
ebi-a-GCST90001915	rs34291514	CD45 on T cell	17	1058299
ebi-a-GCST90001915	rs1530599	CD45 on T cell	18	23831649
ebi-a-GCST90001916	rs78970139	CD45 on CD4+	2	52204804
ebi-a-GCST90001916	rs73187865	CD45 on CD4+	3	119105402
ebi-a-GCST90001916	rs11944009	CD45 on CD4+	4	1568839
ebi-a-GCST90001916	rs7657718	CD45 on CD4+	4	21222071
ebi-a-GCST90001916	rs3104376	CD45 on CD4+	6	32600003
ebi-a-GCST90001916	rs113149369	CD45 on CD4+	6	43645107
ebi-a-GCST90001916	rs3998189	CD45 on CD4+	6	32450357
ebi-a-GCST90001916	rs9363908	CD45 on CD4+	6	69075429
ebi-a-GCST90001916	rs902920	CD45 on CD4+	8	96301882
ebi-a-GCST90001916	rs2185450	CD45 on CD4+	14	38566955
ebi-a-GCST90001916	rs17307464	CD45 on CD4+	16	70755144
ebi-a-GCST90001916	rs189409686	CD45 on CD4+	17	3612334
ebi-a-GCST90001916	rs112736938	CD45 on CD4+	18	64359675
ebi-a-GCST90001916	rs62120820	CD45 on CD4+	19	56697803
ebi-a-GCST90001917	rs76544033	CD45 on CD8br	1	165287038
ebi-a-GCST90001917	rs116030815	CD45 on CD8br	1	42691989
ebi-a-GCST90001917	rs147201606	CD45 on CD8br	1	30780553
ebi-a-GCST90001917	rs17557937	CD45 on CD8br	1	91901382
ebi-a-GCST90001917	rs4849134	CD45 on CD8br	2	111615015
ebi-a-GCST90001917	rs7559619	CD45 on CD8br	2	38892207
ebi-a-GCST90001917	rs7643142	CD45 on CD8br	3	175109345
ebi-a-GCST90001917	rs11707721	CD45 on CD8br	3	67272268
ebi-a-GCST90001917	rs543582225	CD45 on CD8br	4	94602439
ebi-a-GCST90001917	rs77771245	CD45 on CD8br	4	20154192
ebi-a-GCST90001917	rs11100525	CD45 on CD8br	4	164804342
ebi-a-GCST90001917	rs115625722	CD45 on CD8br	4	21411107
ebi-a-GCST90001917	rs114726846	CD45 on CD8br	4	85362264
ebi-a-GCST90001917	rs2409763	CD45 on CD8br	5	59832250
ebi-a-GCST90001917	rs60321540	CD45 on CD8br	5	143601136
ebi-a-GCST90001917	rs62404145	CD45 on CD8br	6	32641178
ebi-a-GCST90001917	rs147779398	CD45 on CD8br	7	42610990
ebi-a-GCST90001917	rs77188735	CD45 on CD8br	9	77312021
ebi-a-GCST90001917	rs783460	CD45 on CD8br	9	23797136
ebi-a-GCST90001917	rs150904881	CD45 on CD8br	9	68222950
ebi-a-GCST90001917	rs147885318	CD45 on CD8br	9	32795573
ebi-a-GCST90001917	rs117933675	CD45 on CD8br	10	121098011
ebi-a-GCST90001917	rs7897761	CD45 on CD8br	10	93662774
ebi-a-GCST90001917	rs1015952	CD45 on CD8br	12	77741488
ebi-a-GCST90001917	rs117878489	CD45 on CD8br	12	125913115
ebi-a-GCST90001917	rs7179582	CD45 on CD8br	15	34128532
ebi-a-GCST90001917	rs71357170	CD45 on CD8br	17	1046040
ebi-a-GCST90001917	rs141418004	CD45 on CD8br	18	49028375
ebi-a-GCST90001918	rs77647448	CD45 on HLA DR+	1	198669823

ebi-a-GCST90001918	rs4287807	CD45 on HLA DR+	2	1310118
ebi-a-GCST90001918	rs45490493	CD45 on HLA DR+	3	9002572
ebi-a-GCST90001918	rs77299479	CD45 on HLA DR+	3	195101176
ebi-a-GCST90001918	rs56073410	CD45 on HLA DR+	4	188847101
ebi-a-GCST90001918	rs76771115	CD45 on HLA DR+	5	16169518
ebi-a-GCST90001918	rs142492586	CD45 on HLA DR+	6	33202756
ebi-a-GCST90001918	rs79909944	CD45 on HLA DR+	6	151771153
ebi-a-GCST90001918	rs71543398	CD45 on HLA DR+	7	140888195
ebi-a-GCST90001918	rs11195975	CD45 on HLA DR+	10	114254390
ebi-a-GCST90001918	rs7296607	CD45 on HLA DR+	12	85162390
ebi-a-GCST90001918	rs200751912	CD45 on HLA DR+	12	63154160
ebi-a-GCST90001918	rs12884891	CD45 on HLA DR+	14	90027519
ebi-a-GCST90001918	rs141959698	CD45 on HLA DR+	15	81676142
ebi-a-GCST90001918	rs12924236	CD45 on HLA DR+	16	10974423
ebi-a-GCST90001918	rs34970051	CD45 on HLA DR+	17	1055106
ebi-a-GCST90001918	rs10411493	CD45 on HLA DR+	19	30617388
ebi-a-GCST90001918	rs2002814	CD45 on HLA DR+	22	21399278
ebi-a-GCST90001919	rs75454391	CD45 on NKT	1	244910968
ebi-a-GCST90001919	rs116030815	CD45 on NKT	1	42691989
ebi-a-GCST90001919	rs12145132	CD45 on NKT	1	198636736
ebi-a-GCST90001919	rs147981444	CD45 on NKT	1	155359033
ebi-a-GCST90001919	rs742800	CD45 on NKT	1	29718191
ebi-a-GCST90001919	rs72974727	CD45 on NKT	2	237031702
ebi-a-GCST90001919	rs11676095	CD45 on NKT	2	206656142
ebi-a-GCST90001919	rs58905133	CD45 on NKT	2	38887964
ebi-a-GCST90001919	rs151179737	CD45 on NKT	3	75549506
ebi-a-GCST90001919	rs141044941	CD45 on NKT	3	168694784
ebi-a-GCST90001919	rs34993786	CD45 on NKT	3	66434668
ebi-a-GCST90001919	rs115618992	CD45 on NKT	4	5432284
ebi-a-GCST90001919	rs60321540	CD45 on NKT	5	143601136
ebi-a-GCST90001919	rs196695	CD45 on NKT	6	80154074
ebi-a-GCST90001919	rs62440365	CD45 on NKT	6	160760868
ebi-a-GCST90001919	rs140611829	CD45 on NKT	6	14271982
ebi-a-GCST90001919	rs114672192	CD45 on NKT	6	15377905
ebi-a-GCST90001919	rs117697165	CD45 on NKT	6	113791493
ebi-a-GCST90001919	rs10952012	CD45 on NKT	7	1335231
ebi-a-GCST90001919	rs546423	CD45 on NKT	9	135344611
ebi-a-GCST90001919	rs163389	CD45 on NKT	17	15579474
ebi-a-GCST90001919	rs7217404	CD45 on NKT	17	13490432
ebi-a-GCST90001919	rs62120820	CD45 on NKT	19	56697803
ebi-a-GCST90001919	rs117385292	CD45 on NKT	20	393747
ebi-a-GCST90001920	rs148669043	CD45 on HLA DR+	2	133292614
ebi-a-GCST90001920	rs730220	CD45 on HLA DR+	2	240672330
ebi-a-GCST90001920	rs112341592	CD45 on HLA DR+	3	87411172
ebi-a-GCST90001920	rs139435103	CD45 on HLA DR+	3	9027874
ebi-a-GCST90001920	rs939285	CD45 on HLA DR+	3	14647282
ebi-a-GCST90001920	rs117486308	CD45 on HLA DR+	6	147964200
ebi-a-GCST90001920	rs71575076	CD45 on HLA DR+	6	156577959

ebi-a-GCST90001920	rs2092305	CD45 on HLA DR+	6	33100189
ebi-a-GCST90001920	rs78073915	CD45 on HLA DR+	6	80863600
ebi-a-GCST90001920	rs9271376	CD45 on HLA DR+	6	32587113
ebi-a-GCST90001920	rs537480011	CD45 on HLA DR+	6	149188427
ebi-a-GCST90001920	rs66546311	CD45 on HLA DR+	7	127039480
ebi-a-GCST90001920	rs150842259	CD45 on HLA DR+	9	118843067
ebi-a-GCST90001920	rs3764232	CD45 on HLA DR+	15	34133130
ebi-a-GCST90001920	rs79583791	CD45 on HLA DR+	15	74317702
ebi-a-GCST90001920	rs62032949	CD45 on HLA DR+	16	17653439
ebi-a-GCST90001920	rs72770017	CD45 on HLA DR+	16	10973612
ebi-a-GCST90001920	rs2226358	CD45 on HLA DR+	21	39213937
ebi-a-GCST90001921	rs12127496	CD45 on HLA DR+	1	198723915
ebi-a-GCST90001921	rs137878176	CD45 on HLA DR+	2	51742547
ebi-a-GCST90001921	rs75453573	CD45 on HLA DR+	2	192777430
ebi-a-GCST90001921	rs7559619	CD45 on HLA DR+	2	38892207
ebi-a-GCST90001921	rs111625920	CD45 on HLA DR+	3	195814264
ebi-a-GCST90001921	rs2582093	CD45 on HLA DR+	3	183533376
ebi-a-GCST90001921	rs185352219	CD45 on HLA DR+	3	129008805
ebi-a-GCST90001921	rs75760442	CD45 on HLA DR+	3	122783073
ebi-a-GCST90001921	rs17132647	CD45 on HLA DR+	4	2491695
ebi-a-GCST90001921	rs72773782	CD45 on HLA DR+	5	75531539
ebi-a-GCST90001921	rs3116989	CD45 on HLA DR+	6	33099538
ebi-a-GCST90001921	rs578355	CD45 on HLA DR+	8	102483098
ebi-a-GCST90001921	rs10105402	CD45 on HLA DR+	8	131518417
ebi-a-GCST90001921	rs16925130	CD45 on HLA DR+	9	100660122
ebi-a-GCST90001921	rs12335987	CD45 on HLA DR+	9	13235045
ebi-a-GCST90001921	rs10823981	CD45 on HLA DR+	10	53792294
ebi-a-GCST90001921	rs12820360	CD45 on HLA DR+	12	82099799
ebi-a-GCST90001921	rs8010367	CD45 on HLA DR+	14	31905918
ebi-a-GCST90001921	rs10151675	CD45 on HLA DR+	14	77594914
ebi-a-GCST90001921	rs141959698	CD45 on HLA DR+	15	81676142
ebi-a-GCST90001921	rs8071409	CD45 on HLA DR+	17	36030219
ebi-a-GCST90001921	rs10415710	CD45 on HLA DR+	19	45014722
ebi-a-GCST90001922	rs41302806	CD127 on T cell	1	97280132
ebi-a-GCST90001922	rs7571217	CD127 on T cell	2	192327964
ebi-a-GCST90001922	rs16869627	CD127 on T cell	5	2447673
ebi-a-GCST90001922	rs13176824	CD127 on T cell	5	170977932
ebi-a-GCST90001922	rs13180666	CD127 on T cell	5	35836386
ebi-a-GCST90001922	rs55748070	CD127 on T cell	5	55194112
ebi-a-GCST90001922	rs17577280	CD127 on T cell	6	110728379
ebi-a-GCST90001922	rs7801342	CD127 on T cell	7	31163003
ebi-a-GCST90001922	rs78487989	CD127 on T cell	12	6408626
ebi-a-GCST90001922	rs2951771	CD127 on T cell	12	11601027
ebi-a-GCST90001922	rs12370077	CD127 on T cell	12	55201765
ebi-a-GCST90001922	rs11627110	CD127 on T cell	14	95845572
ebi-a-GCST90001922	rs74644924	CD127 on T cell	16	1010272
ebi-a-GCST90001922	rs11078935	CD127 on T cell	17	38192137
ebi-a-GCST90001922	rs116757507	CD127 on T cell	17	5844216

ebi-a-GCST90001922	rs148709019	CD127 on T cell	19	23371494
ebi-a-GCST90001922	rs117217795	CD127 on T cell	19	49000393
ebi-a-GCST90001922	rs6107545	CD127 on T cell	20	4853544
ebi-a-GCST90001923	rs75904097	CD127 on CD45RA	1	33089735
ebi-a-GCST90001923	rs6703836	CD127 on CD45RA	1	174068813
ebi-a-GCST90001923	rs143650607	CD127 on CD45RA	2	192303701
ebi-a-GCST90001923	rs12634099	CD127 on CD45RA	3	112869208
ebi-a-GCST90001923	rs12513323	CD127 on CD45RA	4	185517125
ebi-a-GCST90001923	rs140978083	CD127 on CD45RA	4	114960229
ebi-a-GCST90001923	rs16899999	CD127 on CD45RA	5	35886731
ebi-a-GCST90001923	rs62369573	CD127 on CD45RA	5	64518731
ebi-a-GCST90001923	rs17739576	CD127 on CD45RA	6	73187327
ebi-a-GCST90001923	rs17064297	CD127 on CD45RA	8	1777571
ebi-a-GCST90001923	rs118179243	CD127 on CD45RA	11	134914590
ebi-a-GCST90001923	rs112348392	CD127 on CD45RA	11	47832118
ebi-a-GCST90001923	rs12230438	CD127 on CD45RA	12	55296757
ebi-a-GCST90001923	rs149514573	CD127 on CD45RA	13	84155288
ebi-a-GCST90001923	rs3848363	CD127 on CD45RA	16	370484
ebi-a-GCST90001923	rs8081659	CD127 on CD45RA	17	27213990
ebi-a-GCST90001923	rs6038366	CD127 on CD45RA	20	6111569
ebi-a-GCST90001923	rs5999348	CD127 on CD45RA	22	34818466
ebi-a-GCST90001924	rs55872368	CD127 on CD28+ C	1	159176856
ebi-a-GCST90001924	rs28680958	CD127 on CD28+ C	1	173848808
ebi-a-GCST90001924	rs12138848	CD127 on CD28+ C	1	210808177
ebi-a-GCST90001924	rs75904097	CD127 on CD28+ C	1	33089735
ebi-a-GCST90001924	rs12513323	CD127 on CD28+ C	4	185517125
ebi-a-GCST90001924	rs2961796	CD127 on CD28+ C	5	3009195
ebi-a-GCST90001924	rs16899999	CD127 on CD28+ C	5	35886731
ebi-a-GCST90001924	rs17773747	CD127 on CD28+ C	5	136145783
ebi-a-GCST90001924	rs17739576	CD127 on CD28+ C	6	73187327
ebi-a-GCST90001924	rs1551777	CD127 on CD28+ C	6	2690349
ebi-a-GCST90001924	rs150735276	CD127 on CD28+ C	7	21186016
ebi-a-GCST90001924	rs17064297	CD127 on CD28+ C	8	1777571
ebi-a-GCST90001924	rs78763541	CD127 on CD28+ C	8	144226020
ebi-a-GCST90001924	rs78061906	CD127 on CD28+ C	8	119499029
ebi-a-GCST90001924	rs84125	CD127 on CD28+ C	8	40204262
ebi-a-GCST90001924	rs72652037	CD127 on CD28+ C	8	71958635
ebi-a-GCST90001924	rs149514573	CD127 on CD28+ C	13	84155288
ebi-a-GCST90001924	rs4426311	CD127 on CD28+ C	15	38704057
ebi-a-GCST90001924	rs142123008	CD127 on CD28+ C	16	32639782
ebi-a-GCST90001924	rs1573734	CD127 on CD28+ C	16	423619
ebi-a-GCST90001924	rs111717712	CD127 on CD28+ C	19	20758619
ebi-a-GCST90001924	rs130970	CD127 on CD28+ C	22	34774585
ebi-a-GCST90001925	rs72656535	CD127 on CD28+ I	1	28291321
ebi-a-GCST90001925	rs873041	CD127 on CD28+ I	1	29718378
ebi-a-GCST90001925	rs59577227	CD127 on CD28+ I	1	208733117
ebi-a-GCST90001925	rs56234206	CD127 on CD28+ I	2	198215995
ebi-a-GCST90001925	rs12995587	CD127 on CD28+ I	2	189302808

ebi-a-GCST90001925	rs9873703	CD127 on CD28+ I	3	153109199
ebi-a-GCST90001925	rs76041591	CD127 on CD28+ I	3	71873670
ebi-a-GCST90001925	rs114486110	CD127 on CD28+ I	3	43667446
ebi-a-GCST90001925	rs4624569	CD127 on CD28+ I	3	175271277
ebi-a-GCST90001925	rs138908372	CD127 on CD28+ I	4	173906273
ebi-a-GCST90001925	rs75977566	CD127 on CD28+ I	4	26052897
ebi-a-GCST90001925	rs1877237	CD127 on CD28+ I	4	140625847
ebi-a-GCST90001925	rs7692704	CD127 on CD28+ I	4	118364292
ebi-a-GCST90001925	rs1961426	CD127 on CD28+ I	5	35810561
ebi-a-GCST90001925	rs117913408	CD127 on CD28+ I	6	102173117
ebi-a-GCST90001925	rs186008830	CD127 on CD28+ I	7	81170544
ebi-a-GCST90001925	rs6964799	CD127 on CD28+ I	7	52158967
ebi-a-GCST90001925	rs2688332	CD127 on CD28+ I	8	3880864
ebi-a-GCST90001925	rs148451514	CD127 on CD28+ I	8	21213574
ebi-a-GCST90001925	rs139832224	CD127 on CD28+ I	9	72035000
ebi-a-GCST90001925	rs73183591	CD127 on CD28+ I	13	40745424
ebi-a-GCST90001925	rs12900372	CD127 on CD28+ I	15	38673895
ebi-a-GCST90001925	rs9806909	CD127 on CD28+ I	16	83933597
ebi-a-GCST90001925	rs145432806	CD127 on CD28+ I	19	595812
ebi-a-GCST90001925	rs187144484	CD127 on CD28+ I	19	22624919
ebi-a-GCST90001925	rs6038487	CD127 on CD28+ I	20	6407136
ebi-a-GCST90001926	rs80205330	CD127 on granuloc	2	133708302
ebi-a-GCST90001926	rs78526927	CD127 on granuloc	2	175370076
ebi-a-GCST90001926	rs3774298	CD127 on granuloc	3	187460099
ebi-a-GCST90001926	rs3857014	CD127 on granuloc	4	166362221
ebi-a-GCST90001926	rs12659322	CD127 on granuloc	5	31925216
ebi-a-GCST90001926	rs77648394	CD127 on granuloc	5	179719608
ebi-a-GCST90001926	rs915125	CD127 on granuloc	6	82463376
ebi-a-GCST90001926	rs73045828	CD127 on granuloc	6	169053049
ebi-a-GCST90001926	rs9376098	CD127 on granuloc	6	135499460
ebi-a-GCST90001926	rs4272	CD127 on granuloc	7	92236829
ebi-a-GCST90001926	rs78124306	CD127 on granuloc	7	22121992
ebi-a-GCST90001926	rs445	CD127 on granuloc	7	92408370
ebi-a-GCST90001926	rs62445273	CD127 on granuloc	7	21716643
ebi-a-GCST90001926	rs4433170	CD127 on granuloc	8	6891971
ebi-a-GCST90001926	rs12675298	CD127 on granuloc	8	6828813
ebi-a-GCST90001926	rs62546664	CD127 on granuloc	9	26584433
ebi-a-GCST90001926	rs140819873	CD127 on granuloc	9	116014845
ebi-a-GCST90001926	rs3824801	CD127 on granuloc	10	126356840
ebi-a-GCST90001926	rs74490964	CD127 on granuloc	12	89458555
ebi-a-GCST90001926	rs959275	CD127 on granuloc	12	82430283
ebi-a-GCST90001926	rs9519933	CD127 on granuloc	13	106715057
ebi-a-GCST90001926	rs146130460	CD127 on granuloc	17	61576413
ebi-a-GCST90001926	rs77771777	CD127 on granuloc	17	55927801
ebi-a-GCST90001926	rs113425015	CD127 on granuloc	17	58951391
ebi-a-GCST90001926	rs142587417	CD127 on granuloc	17	58777014
ebi-a-GCST90001926	rs138284624	CD127 on granuloc	17	56385573
ebi-a-GCST90001926	rs147173440	CD127 on granuloc	17	59945942

ebi-a-GCST90001926	rs115426429	CD127 on granuloc	17	59341923
ebi-a-GCST90001926	rs139132726	CD127 on granuloc	17	55643849
ebi-a-GCST90001926	rs140712754	CD127 on granuloc	18	45108661
ebi-a-GCST90001926	rs144728338	CD127 on granuloc	20	54118523
ebi-a-GCST90001927	rs7533594	CD127 on CD8br	1	205558402
ebi-a-GCST90001927	rs4925660	CD127 on CD8br	1	247251150
ebi-a-GCST90001927	rs72824431	CD127 on CD8br	2	104853841
ebi-a-GCST90001927	rs28869816	CD127 on CD8br	2	116742575
ebi-a-GCST90001927	rs4668705	CD127 on CD8br	2	11152503
ebi-a-GCST90001927	rs7643142	CD127 on CD8br	3	175109345
ebi-a-GCST90001927	rs10024641	CD127 on CD8br	4	188051690
ebi-a-GCST90001927	rs1377028	CD127 on CD8br	4	75738792
ebi-a-GCST90001927	rs13165333	CD127 on CD8br	5	82275571
ebi-a-GCST90001927	rs2009425	CD127 on CD8br	5	174034800
ebi-a-GCST90001927	rs457979	CD127 on CD8br	5	2382270
ebi-a-GCST90001927	rs159608	CD127 on CD8br	5	59174103
ebi-a-GCST90001927	rs3130901	CD127 on CD8br	6	31419065
ebi-a-GCST90001927	rs74785296	CD127 on CD8br	7	156120842
ebi-a-GCST90001927	rs7801342	CD127 on CD8br	7	31163003
ebi-a-GCST90001927	rs727397	CD127 on CD8br	7	105951894
ebi-a-GCST90001927	rs7813990	CD127 on CD8br	8	37671140
ebi-a-GCST90001927	rs1911151	CD127 on CD8br	11	60546636
ebi-a-GCST90001927	rs117239506	CD127 on CD8br	12	16351043
ebi-a-GCST90001927	rs9739743	CD127 on CD8br	12	54596797
ebi-a-GCST90001927	rs73354288	CD127 on CD8br	17	79157596
ebi-a-GCST90001927	rs73561877	CD127 on CD8br	19	6365399
ebi-a-GCST90001927	rs148460149	CD127 on CD8br	20	50115542
ebi-a-GCST90001927	rs5770311	CD127 on CD8br	22	49767656
ebi-a-GCST90001927	rs5770659	CD127 on CD8br	22	50083852
ebi-a-GCST90001928	rs541367304	CD127 on CD28+ C	1	198283058
ebi-a-GCST90001928	rs147561645	CD127 on CD28+ C	2	21495577
ebi-a-GCST90001928	rs17822001	CD127 on CD28+ C	2	195873850
ebi-a-GCST90001928	rs2319326	CD127 on CD28+ C	3	147385623
ebi-a-GCST90001928	rs114103379	CD127 on CD28+ C	4	114954389
ebi-a-GCST90001928	rs116423144	CD127 on CD28+ C	5	174310054
ebi-a-GCST90001928	rs17605660	CD127 on CD28+ C	6	111492432
ebi-a-GCST90001928	rs77111030	CD127 on CD28+ C	6	32023011
ebi-a-GCST90001928	rs139665786	CD127 on CD28+ C	6	21668701
ebi-a-GCST90001928	rs117293308	CD127 on CD28+ C	11	43599281
ebi-a-GCST90001928	rs117562886	CD127 on CD28+ C	12	16449988
ebi-a-GCST90001928	rs8009540	CD127 on CD28+ C	14	20892947
ebi-a-GCST90001928	rs77266206	CD127 on CD28+ C	15	24042401
ebi-a-GCST90001928	rs12597545	CD127 on CD28+ C	16	1394430
ebi-a-GCST90001928	rs4968420	CD127 on CD28+ C	17	60353246
ebi-a-GCST90001928	rs56062431	CD127 on CD28+ C	17	52131640
ebi-a-GCST90001928	rs11670885	CD127 on CD28+ C	19	50552467
ebi-a-GCST90001928	rs7275441	CD127 on CD28+ C	21	18825905
ebi-a-GCST90001928	rs75934614	CD127 on CD28+ C	22	48129274

ebi-a-GCST90001929	rs4999658	CD127 on CD28+ (	2	117820115
ebi-a-GCST90001929	rs68104921	CD127 on CD28+ (	2	41984318
ebi-a-GCST90001929	rs191662454	CD127 on CD28+ (	2	74799096
ebi-a-GCST90001929	rs115564010	CD127 on CD28+ (	3	113178303
ebi-a-GCST90001929	rs1069981	CD127 on CD28+ (	3	64102768
ebi-a-GCST90001929	rs9826381	CD127 on CD28+ (	3	72079577
ebi-a-GCST90001929	rs66570838	CD127 on CD28+ (	5	35840724
ebi-a-GCST90001929	rs3776565	CD127 on CD28+ (	5	36631653
ebi-a-GCST90001929	rs112984211	CD127 on CD28+ (	6	32617889
ebi-a-GCST90001929	rs114609087	CD127 on CD28+ (	6	66170982
ebi-a-GCST90001929	rs111837808	CD127 on CD28+ (	7	137927280
ebi-a-GCST90001929	rs148058371	CD127 on CD28+ (	9	5035205
ebi-a-GCST90001929	rs10733856	CD127 on CD28+ (	10	71603177
ebi-a-GCST90001929	rs116867125	CD127 on CD28+ (	10	17033504
ebi-a-GCST90001929	rs9600216	CD127 on CD28+ (	13	74575348
ebi-a-GCST90001929	rs1424885	CD127 on CD28+ (	14	45250418
ebi-a-GCST90001929	rs2304993	CD127 on CD28+ (	15	79312330
ebi-a-GCST90001929	rs117037628	CD127 on CD28+ (	16	423809
ebi-a-GCST90001929	rs7219820	CD127 on CD28+ (	17	36908512
ebi-a-GCST90001929	rs55767800	CD127 on CD28+ (	17	17674967
ebi-a-GCST90001929	rs77766863	CD127 on CD28+ (	18	70193745
ebi-a-GCST90001929	rs73364364	CD127 on CD28+ (	21	42236763
ebi-a-GCST90001929	rs62242696	CD127 on CD28+ (	21	18828524
ebi-a-GCST90001930	rs72766093	CD127 on CD28- C	1	241717927
ebi-a-GCST90001930	rs115849418	CD127 on CD28- C	1	56800633
ebi-a-GCST90001930	rs12141053	CD127 on CD28- C	1	28882908
ebi-a-GCST90001930	rs4668832	CD127 on CD28- C	2	14665380
ebi-a-GCST90001930	rs145877418	CD127 on CD28- C	2	10634848
ebi-a-GCST90001930	rs62166481	CD127 on CD28- C	2	142337524
ebi-a-GCST90001930	rs62246789	CD127 on CD28- C	3	72493066
ebi-a-GCST90001930	rs7644746	CD127 on CD28- C	3	183596184
ebi-a-GCST90001930	rs9968551	CD127 on CD28- C	4	84654761
ebi-a-GCST90001930	rs727397	CD127 on CD28- C	7	105951894
ebi-a-GCST90001930	rs78886869	CD127 on CD28- C	7	106452071
ebi-a-GCST90001930	rs16939611	CD127 on CD28- C	8	79036414
ebi-a-GCST90001930	rs2762598	CD127 on CD28- C	10	7251026
ebi-a-GCST90001930	rs4760840	CD127 on CD28- C	12	71279650
ebi-a-GCST90001930	rs117712600	CD127 on CD28- C	15	29470184
ebi-a-GCST90001930	rs6500257	CD127 on CD28- C	16	49824897
ebi-a-GCST90001930	rs8062396	CD127 on CD28- C	16	88618795
ebi-a-GCST90001930	rs1874014	CD127 on CD28- C	16	84855516
ebi-a-GCST90001930	rs113302343	CD127 on CD28- C	16	83299602
ebi-a-GCST90001930	rs56095010	CD127 on CD28- C	16	3949935
ebi-a-GCST90001930	rs218703	CD127 on CD28- C	17	3750503
ebi-a-GCST90001930	rs117467123	CD127 on CD28- C	19	16407691
ebi-a-GCST90001931	rs28680958	CD127 on CD4+	1	173848808
ebi-a-GCST90001931	rs12138848	CD127 on CD4+	1	210808177
ebi-a-GCST90001931	rs12402082	CD127 on CD4+	1	231966624

ebi-a-GCST90001931	rs930760	CD127 on CD4+	2	20806336
ebi-a-GCST90001931	rs2671762	CD127 on CD4+	3	5135111
ebi-a-GCST90001931	rs12634099	CD127 on CD4+	3	112869208
ebi-a-GCST90001931	rs12513323	CD127 on CD4+	4	185517125
ebi-a-GCST90001931	rs2961795	CD127 on CD4+	5	3009377
ebi-a-GCST90001932	rs140742144	CD127 on CD45RA	1	210874433
ebi-a-GCST90001932	rs6691053	CD127 on CD45RA	1	173868955
ebi-a-GCST90001932	rs930760	CD127 on CD45RA	2	20806336
ebi-a-GCST90001932	rs180717968	CD127 on CD45RA	2	204124526
ebi-a-GCST90001932	rs6727096	CD127 on CD45RA	2	161307677
ebi-a-GCST90001932	rs115564010	CD127 on CD45RA	3	113178303
ebi-a-GCST90001932	rs144125132	CD127 on CD45RA	5	160293643
ebi-a-GCST90001932	rs11567754	CD127 on CD45RA	5	35872190
ebi-a-GCST90001932	rs138540869	CD127 on CD45RA	5	62602530
ebi-a-GCST90001932	rs9271772	CD127 on CD45RA	6	32594275
ebi-a-GCST90001932	rs6901175	CD127 on CD45RA	6	32198527
ebi-a-GCST90001932	rs3129891	CD127 on CD45RA	6	32415080
ebi-a-GCST90001932	rs10089520	CD127 on CD45RA	8	11686089
ebi-a-GCST90001932	rs7046475	CD127 on CD45RA	9	21582326
ebi-a-GCST90001932	rs34285518	CD127 on CD45RA	10	6136107
ebi-a-GCST90001932	rs7115390	CD127 on CD45RA	11	10757993
ebi-a-GCST90001932	rs2059578	CD127 on CD45RA	11	60066799
ebi-a-GCST90001932	rs67627362	CD127 on CD45RA	12	63155497
ebi-a-GCST90001932	rs6571878	CD127 on CD45RA	14	39350523
ebi-a-GCST90001932	rs76683776	CD127 on CD45RA	16	20131662
ebi-a-GCST90001932	rs1573735	CD127 on CD45RA	16	423696
ebi-a-GCST90001932	rs7264992	CD127 on CD45RA	20	57814866
ebi-a-GCST90001933	rs181741769	CD25 on CD45RA	1	26999658
ebi-a-GCST90001933	rs139210967	CD25 on CD45RA	1	157286941
ebi-a-GCST90001933	rs4669451	CD25 on CD45RA	2	9898172
ebi-a-GCST90001933	rs35894198	CD25 on CD45RA	2	9361836
ebi-a-GCST90001933	rs143987752	CD25 on CD45RA	2	33587355
ebi-a-GCST90001933	rs149326960	CD25 on CD45RA	3	21012581
ebi-a-GCST90001933	rs67403544	CD25 on CD45RA	4	21653587
ebi-a-GCST90001933	rs16884012	CD25 on CD45RA	5	10034219
ebi-a-GCST90001933	rs9480540	CD25 on CD45RA	6	151021074
ebi-a-GCST90001933	rs80026829	CD25 on CD45RA	7	8618736
ebi-a-GCST90001933	rs181516190	CD25 on CD45RA	7	35264731
ebi-a-GCST90001933	rs79062365	CD25 on CD45RA	8	82262167
ebi-a-GCST90001933	rs61839660	CD25 on CD45RA	10	6094697
ebi-a-GCST90001933	rs62626343	CD25 on CD45RA	10	6128374
ebi-a-GCST90001933	rs10509573	CD25 on CD45RA	10	91189221
ebi-a-GCST90001933	rs116928928	CD25 on CD45RA	11	32030595
ebi-a-GCST90001933	rs4075635	CD25 on CD45RA	11	28511643
ebi-a-GCST90001933	rs118110714	CD25 on CD45RA	12	16060925
ebi-a-GCST90001933	rs139194198	CD25 on CD45RA	13	82921880
ebi-a-GCST90001933	rs175717	CD25 on CD45RA	14	75985604
ebi-a-GCST90001933	rs144239147	CD25 on CD45RA	17	48154668

ebi-a-GCST90001933	rs74553250	CD25 on CD45RA	17	40063525
ebi-a-GCST90001933	rs7406899	CD25 on CD45RA	17	39766504
ebi-a-GCST90001933	rs145627854	CD25 on CD45RA	18	67524006
ebi-a-GCST90001933	rs1000411	CD25 on CD45RA	20	39634616
ebi-a-GCST90001933	rs4080481	CD25 on CD45RA	22	37905285
ebi-a-GCST90001934	rs74458586	CD25 on CD45RA-	1	167160690
ebi-a-GCST90001934	rs145161741	CD25 on CD45RA-	1	245170523
ebi-a-GCST90001934	rs1869339	CD25 on CD45RA-	2	75102130
ebi-a-GCST90001934	rs75432369	CD25 on CD45RA-	2	134396058
ebi-a-GCST90001934	rs55751874	CD25 on CD45RA-	3	143929156
ebi-a-GCST90001934	rs9838853	CD25 on CD45RA-	3	141930006
ebi-a-GCST90001934	rs1385469	CD25 on CD45RA-	3	8390226
ebi-a-GCST90001934	rs35614472	CD25 on CD45RA-	7	18687542
ebi-a-GCST90001934	rs9298702	CD25 on CD45RA-	9	14659034
ebi-a-GCST90001934	rs34644690	CD25 on CD45RA-	9	137333688
ebi-a-GCST90001934	rs16917989	CD25 on CD45RA-	9	101647469
ebi-a-GCST90001934	rs10905719	CD25 on CD45RA-	10	6115171
ebi-a-GCST90001934	rs2279434	CD25 on CD45RA-	10	45955064
ebi-a-GCST90001934	rs116411520	CD25 on CD45RA-	10	91533071
ebi-a-GCST90001934	rs79506783	CD25 on CD45RA-	16	85734699
ebi-a-GCST90001934	rs9921355	CD25 on CD45RA-	16	56571706
ebi-a-GCST90001934	rs62026958	CD25 on CD45RA-	16	12639025
ebi-a-GCST90001934	rs936056	CD25 on CD45RA-	17	73587784
ebi-a-GCST90001934	rs34779614	CD25 on CD45RA-	17	43085415
ebi-a-GCST90001934	rs494642	CD25 on CD45RA-	19	5988263
ebi-a-GCST90001934	rs1699228	CD25 on CD45RA-	20	5846927
ebi-a-GCST90001934	rs73112886	CD25 on CD45RA-	20	44737407
ebi-a-GCST90001935	rs6434720	CD25 on CD39+ C	2	195806052
ebi-a-GCST90001935	rs139265102	CD25 on CD39+ C	2	238325747
ebi-a-GCST90001935	rs1506123	CD25 on CD39+ C	5	8180669
ebi-a-GCST90001935	rs209493	CD25 on CD39+ C	6	53183509
ebi-a-GCST90001935	rs11968423	CD25 on CD39+ C	6	25422353
ebi-a-GCST90001935	rs71526028	CD25 on CD39+ C	7	23773150
ebi-a-GCST90001935	rs205775	CD25 on CD39+ C	7	130643318
ebi-a-GCST90001935	rs113703605	CD25 on CD39+ C	9	136787946
ebi-a-GCST90001935	rs6584030	CD25 on CD39+ C	10	97566052
ebi-a-GCST90001935	rs182193538	CD25 on CD39+ C	10	91754083
ebi-a-GCST90001935	rs7477011	CD25 on CD39+ C	10	6040594
ebi-a-GCST90001935	rs7078614	CD25 on CD39+ C	10	6075831
ebi-a-GCST90001935	rs61960319	CD25 on CD39+ C	13	83537406
ebi-a-GCST90001935	rs17516976	CD25 on CD39+ C	15	96452800
ebi-a-GCST90001935	rs75122374	CD25 on CD39+ C	16	1295355
ebi-a-GCST90001935	rs11861637	CD25 on CD39+ C	16	68656715
ebi-a-GCST90001935	rs1840456	CD25 on CD39+ C	18	11351562
ebi-a-GCST90001935	rs77535422	CD25 on CD39+ C	20	59751404
ebi-a-GCST90001936	rs17602422	CD25 on CD4 Treg	1	215125675
ebi-a-GCST90001936	rs141518333	CD25 on CD4 Treg	1	31011676
ebi-a-GCST90001936	rs80185999	CD25 on CD4 Treg	2	178647768

ebi-a-GCST90001936	rs7601164	CD25 on CD4 Treg	2	14678648
ebi-a-GCST90001936	rs73178594	CD25 on CD4 Treg	3	94912880
ebi-a-GCST90001936	rs115584720	CD25 on CD4 Treg	4	189537163
ebi-a-GCST90001936	rs115155204	CD25 on CD4 Treg	4	91991072
ebi-a-GCST90001936	rs4109437	CD25 on CD4 Treg	4	190769223
ebi-a-GCST90001936	rs6939307	CD25 on CD4 Treg	6	129838002
ebi-a-GCST90001936	rs141933481	CD25 on CD4 Treg	7	98045067
ebi-a-GCST90001936	rs117223635	CD25 on CD4 Treg	9	90443364
ebi-a-GCST90001936	rs9422867	CD25 on CD4 Treg	10	126960279
ebi-a-GCST90001936	rs80267112	CD25 on CD4 Treg	10	66599595
ebi-a-GCST90001936	rs11598494	CD25 on CD4 Treg	10	6178941
ebi-a-GCST90001936	rs12244238	CD25 on CD4 Treg	10	6083239
ebi-a-GCST90001936	rs1601402	CD25 on CD4 Treg	12	30968187
ebi-a-GCST90001936	rs9596433	CD25 on CD4 Treg	13	51639441
ebi-a-GCST90001936	rs7324524	CD25 on CD4 Treg	13	30357303
ebi-a-GCST90001936	rs45556043	CD25 on CD4 Treg	16	11019577
ebi-a-GCST90001936	rs4791980	CD25 on CD4 Treg	17	10259440
ebi-a-GCST90001936	rs75991976	CD25 on CD4 Treg	18	70998473
ebi-a-GCST90001936	rs148961261	CD25 on CD4 Treg	20	51271541
ebi-a-GCST90001937	rs12692532	CD25 on resting Tr	2	14682498
ebi-a-GCST90001937	rs4894809	CD25 on resting Tr	3	171844658
ebi-a-GCST90001937	rs71314568	CD25 on resting Tr	3	140618909
ebi-a-GCST90001937	rs2523882	CD25 on resting Tr	6	31042217
ebi-a-GCST90001937	rs453375	CD25 on resting Tr	6	29942487
ebi-a-GCST90001937	rs9265683	CD25 on resting Tr	6	31301240
ebi-a-GCST90001937	rs55877366	CD25 on resting Tr	6	129834798
ebi-a-GCST90001937	rs4871528	CD25 on resting Tr	8	120712611
ebi-a-GCST90001937	rs10819198	CD25 on resting Tr	9	129478793
ebi-a-GCST90001937	rs72780733	CD25 on resting Tr	10	17607698
ebi-a-GCST90001937	rs147487472	CD25 on resting Tr	10	6191376
ebi-a-GCST90001937	rs12722600	CD25 on resting Tr	10	6054765
ebi-a-GCST90001937	rs10742701	CD25 on resting Tr	11	44299909
ebi-a-GCST90001937	rs77326419	CD25 on resting Tr	11	44372275
ebi-a-GCST90001937	rs117486405	CD25 on resting Tr	12	32082360
ebi-a-GCST90001937	rs11631647	CD25 on resting Tr	15	63256904
ebi-a-GCST90001937	rs75647790	CD25 on resting Tr	16	48484960
ebi-a-GCST90001937	rs11654002	CD25 on resting Tr	17	6630436
ebi-a-GCST90001937	rs8075955	CD25 on resting Tr	17	9625559
ebi-a-GCST90001937	rs11661947	CD25 on resting Tr	18	61892007
ebi-a-GCST90001937	rs12965398	CD25 on resting Tr	18	33222414
ebi-a-GCST90001938	rs6681289	CD25 on CD39+ re	1	119421209
ebi-a-GCST90001938	rs113948643	CD25 on CD39+ re	1	63568945
ebi-a-GCST90001938	rs76433036	CD25 on CD39+ re	1	5264834
ebi-a-GCST90001938	rs139668159	CD25 on CD39+ re	3	151248680
ebi-a-GCST90001938	rs3095996	CD25 on CD39+ re	5	157370372
ebi-a-GCST90001938	rs72804108	CD25 on CD39+ re	5	134635583
ebi-a-GCST90001938	rs6477716	CD25 on CD39+ re	9	112390854
ebi-a-GCST90001938	rs117447663	CD25 on CD39+ re	10	68626431

ebi-a-GCST90001938	rs77328910	CD25 on CD39+ re	10	19334047
ebi-a-GCST90001938	rs540469	CD25 on CD39+ re	11	117346513
ebi-a-GCST90001938	rs4758500	CD25 on CD39+ re	11	3454330
ebi-a-GCST90001938	rs62043866	CD25 on CD39+ re	16	81444190
ebi-a-GCST90001938	rs78950918	CD25 on CD39+ re	19	51786661
ebi-a-GCST90001938	rs28478882	CD25 on CD39+ re	19	49021549
ebi-a-GCST90001938	rs17184074	CD25 on CD39+ re	20	37805444
ebi-a-GCST90001939	rs77993403	CD25 on activated '	1	154316996
ebi-a-GCST90001939	rs113116201	CD25 on activated '	1	198830942
ebi-a-GCST90001939	rs11578578	CD25 on activated '	1	22917278
ebi-a-GCST90001939	rs111871083	CD25 on activated '	1	27823957
ebi-a-GCST90001939	rs112819427	CD25 on activated '	2	38588385
ebi-a-GCST90001939	rs16843666	CD25 on activated '	2	159955595
ebi-a-GCST90001939	rs145947216	CD25 on activated '	2	38596674
ebi-a-GCST90001939	rs78257733	CD25 on activated '	3	152588864
ebi-a-GCST90001939	rs73215328	CD25 on activated '	4	29722257
ebi-a-GCST90001939	rs76542581	CD25 on activated '	5	39250293
ebi-a-GCST90001939	rs16903407	CD25 on activated '	5	2165975
ebi-a-GCST90001939	rs6939307	CD25 on activated '	6	129838002
ebi-a-GCST90001939	rs114746521	CD25 on activated '	6	33720059
ebi-a-GCST90001939	rs1332159	CD25 on activated '	9	25552600
ebi-a-GCST90001939	rs7999263	CD25 on activated '	13	104070037
ebi-a-GCST90001939	rs77205061	CD25 on activated '	16	11133225
ebi-a-GCST90001939	rs117690070	CD25 on activated '	18	11450416
ebi-a-GCST90001939	rs254283	CD25 on activated '	19	54687334
ebi-a-GCST90001940	rs76651564	CD25 on CD39+ ac	1	11771496
ebi-a-GCST90001940	rs80100661	CD25 on CD39+ ac	2	200338980
ebi-a-GCST90001940	rs615854	CD25 on CD39+ ac	3	61803428
ebi-a-GCST90001940	rs13106248	CD25 on CD39+ ac	4	5775106
ebi-a-GCST90001940	rs11097003	CD25 on CD39+ ac	4	39796980
ebi-a-GCST90001940	rs73215328	CD25 on CD39+ ac	4	29722257
ebi-a-GCST90001940	rs11968423	CD25 on CD39+ ac	6	25422353
ebi-a-GCST90001940	rs6584030	CD25 on CD39+ ac	10	97566052
ebi-a-GCST90001940	rs7078614	CD25 on CD39+ ac	10	6075831
ebi-a-GCST90001940	rs117086655	CD25 on CD39+ ac	13	114821897
ebi-a-GCST90001940	rs145139270	CD25 on CD39+ ac	15	100841329
ebi-a-GCST90001940	rs62065216	CD25 on CD39+ ac	17	38218773
ebi-a-GCST90001940	rs73154186	CD25 on CD39+ ac	22	26421469
ebi-a-GCST90001941	rs114116419	CD25 on secreting '	1	24521634
ebi-a-GCST90001941	rs113116201	CD25 on secreting '	1	198830942
ebi-a-GCST90001941	rs141518333	CD25 on secreting '	1	31011676
ebi-a-GCST90001941	rs76863805	CD25 on secreting '	1	226932737
ebi-a-GCST90001941	rs113465498	CD25 on secreting '	1	59360338
ebi-a-GCST90001941	rs12692532	CD25 on secreting '	2	14682498
ebi-a-GCST90001941	rs145947216	CD25 on secreting '	2	38596674
ebi-a-GCST90001941	rs16843666	CD25 on secreting '	2	159955595
ebi-a-GCST90001941	rs59279177	CD25 on secreting '	6	129842380
ebi-a-GCST90001941	rs148887938	CD25 on secreting '	7	131793986

ebi-a-GCST90001941	rs113165806	CD25 on secreting '	7	96426791
ebi-a-GCST90001941	rs7809749	CD25 on secreting '	7	152731084
ebi-a-GCST90001941	rs4747846	CD25 on secreting '	10	6074451
ebi-a-GCST90001941	rs9596433	CD25 on secreting '	13	51639441
ebi-a-GCST90001941	rs78677147	CD25 on secreting '	18	61967509
ebi-a-GCST90001941	rs111399540	CD25 on secreting '	18	9573841
ebi-a-GCST90001942	rs113116201	CD25 on CD39+ se	1	198830942
ebi-a-GCST90001942	rs10489950	CD25 on CD39+ se	1	78842265
ebi-a-GCST90001942	rs7366082	CD25 on CD39+ se	1	22048891
ebi-a-GCST90001942	rs113465498	CD25 on CD39+ se	1	59360338
ebi-a-GCST90001942	rs34645541	CD25 on CD39+ se	1	195875605
ebi-a-GCST90001942	rs114116419	CD25 on CD39+ se	1	24521634
ebi-a-GCST90001942	rs34394397	CD25 on CD39+ se	4	92182072
ebi-a-GCST90001942	rs141181706	CD25 on CD39+ se	6	153211456
ebi-a-GCST90001942	rs1900955	CD25 on CD39+ se	7	96427656
ebi-a-GCST90001942	rs79795219	CD25 on CD39+ se	7	70613403
ebi-a-GCST90001942	rs7796815	CD25 on CD39+ se	7	152731524
ebi-a-GCST90001942	rs73554143	CD25 on CD39+ se	9	137270712
ebi-a-GCST90001942	rs4747846	CD25 on CD39+ se	10	6074451
ebi-a-GCST90001942	rs80267112	CD25 on CD39+ se	10	66599595
ebi-a-GCST90001942	rs1871898	CD25 on CD39+ se	12	129849900
ebi-a-GCST90001942	rs4982176	CD25 on CD39+ se	14	34867304
ebi-a-GCST90001942	rs61005827	CD25 on CD39+ se	17	43122065
ebi-a-GCST90001942	rs75991976	CD25 on CD39+ se	18	70998473
ebi-a-GCST90001942	rs254283	CD25 on CD39+ se	19	54687334
ebi-a-GCST90001942	rs7265470	CD25 on CD39+ se	20	1478662
ebi-a-GCST90001943	rs141518333	CD25 on activated	1	31011676
ebi-a-GCST90001943	rs17602422	CD25 on activated	1	215125675
ebi-a-GCST90001943	rs76863805	CD25 on activated	1	226932737
ebi-a-GCST90001943	rs113116201	CD25 on activated	1	198830942
ebi-a-GCST90001943	rs80185999	CD25 on activated	2	178647768
ebi-a-GCST90001943	rs115584720	CD25 on activated	4	189537163
ebi-a-GCST90001943	rs78090485	CD25 on activated	4	86292367
ebi-a-GCST90001943	rs115155204	CD25 on activated	4	91991072
ebi-a-GCST90001943	rs4109437	CD25 on activated	4	190769223
ebi-a-GCST90001943	rs35769904	CD25 on activated	5	6778750
ebi-a-GCST90001943	rs58024	CD25 on activated	9	20317330
ebi-a-GCST90001943	rs4747846	CD25 on activated	10	6074451
ebi-a-GCST90001943	rs80267112	CD25 on activated	10	66599595
ebi-a-GCST90001943	rs927891	CD25 on activated	10	126959090
ebi-a-GCST90001943	rs76076322	CD25 on activated	12	12036763
ebi-a-GCST90001943	rs1601402	CD25 on activated	12	30968187
ebi-a-GCST90001943	rs9596433	CD25 on activated	13	51639441
ebi-a-GCST90001943	rs140682448	CD25 on activated	15	38957925
ebi-a-GCST90001943	rs45556043	CD25 on activated	16	11019577
ebi-a-GCST90001943	rs75991976	CD25 on activated	18	70998473
ebi-a-GCST90001943	rs148961261	CD25 on activated	20	51271541
ebi-a-GCST90001944	rs79213920	CD123 on plasmacy	1	225471349

ebi-a-GCST90001944	rs149848284	CD123 on plasmacy	2	176302664
ebi-a-GCST90001944	rs75317870	CD123 on plasmacy	2	141073584
ebi-a-GCST90001944	rs2324617	CD123 on plasmacy	3	76493619
ebi-a-GCST90001944	rs3119997	CD123 on plasmacy	3	175496605
ebi-a-GCST90001944	rs7658518	CD123 on plasmacy	4	84160279
ebi-a-GCST90001944	rs17063892	CD123 on plasmacy	5	174168279
ebi-a-GCST90001944	rs76945221	CD123 on plasmacy	5	125054477
ebi-a-GCST90001944	rs10485052	CD123 on plasmacy	6	48165228
ebi-a-GCST90001944	rs62447171	CD123 on plasmacy	7	50309890
ebi-a-GCST90001944	rs78797637	CD123 on plasmacy	7	139644484
ebi-a-GCST90001944	rs73147828	CD123 on plasmacy	7	98434427
ebi-a-GCST90001944	rs147712748	CD123 on plasmacy	8	5010714
ebi-a-GCST90001944	rs4609257	CD123 on plasmacy	9	78552389
ebi-a-GCST90001944	rs7099805	CD123 on plasmacy	10	97235286
ebi-a-GCST90001944	rs7334194	CD123 on plasmacy	13	106834880
ebi-a-GCST90001944	rs117840260	CD123 on plasmacy	15	71910865
ebi-a-GCST90001944	rs148659293	CD123 on plasmacy	17	79284358
ebi-a-GCST90001945	rs79213920	CD123 on CD62L+	1	225471349
ebi-a-GCST90001945	rs75317870	CD123 on CD62L+	2	141073584
ebi-a-GCST90001945	rs149848284	CD123 on CD62L+	2	176302664
ebi-a-GCST90001945	rs3119997	CD123 on CD62L+	3	175496605
ebi-a-GCST90001945	rs2324617	CD123 on CD62L+	3	76493619
ebi-a-GCST90001945	rs7658518	CD123 on CD62L+	4	84160279
ebi-a-GCST90001945	rs76945221	CD123 on CD62L+	5	125054477
ebi-a-GCST90001945	rs17063892	CD123 on CD62L+	5	174168279
ebi-a-GCST90001945	rs10485052	CD123 on CD62L+	6	48165228
ebi-a-GCST90001945	rs62447171	CD123 on CD62L+	7	50309890
ebi-a-GCST90001945	rs73147828	CD123 on CD62L+	7	98434427
ebi-a-GCST90001945	rs78797637	CD123 on CD62L+	7	139644484
ebi-a-GCST90001945	rs147712748	CD123 on CD62L+	8	5010714
ebi-a-GCST90001945	rs4609257	CD123 on CD62L+	9	78552389
ebi-a-GCST90001945	rs7099805	CD123 on CD62L+	10	97235286
ebi-a-GCST90001945	rs7334194	CD123 on CD62L+	13	106834880
ebi-a-GCST90001945	rs117840260	CD123 on CD62L+	15	71910865
ebi-a-GCST90001945	rs148659293	CD123 on CD62L+	17	79284358
ebi-a-GCST90001946	rs113378129	CD33 on CD14+ m	2	72403927
ebi-a-GCST90001946	rs10164761	CD33 on CD14+ m	2	220066209
ebi-a-GCST90001946	rs9858561	CD33 on CD14+ m	3	120507751
ebi-a-GCST90001946	rs2242310	CD33 on CD14+ m	4	53824071
ebi-a-GCST90001946	rs11741119	CD33 on CD14+ m	5	773420
ebi-a-GCST90001946	rs11755998	CD33 on CD14+ m	6	82463790
ebi-a-GCST90001946	rs78886656	CD33 on CD14+ m	7	99777245
ebi-a-GCST90001946	rs12337816	CD33 on CD14+ m	9	117125236
ebi-a-GCST90001946	rs7047105	CD33 on CD14+ m	9	105951980
ebi-a-GCST90001946	rs183353458	CD33 on CD14+ m	10	20005831
ebi-a-GCST90001946	rs138138479	CD33 on CD14+ m	10	22339808
ebi-a-GCST90001946	rs114090793	CD33 on CD14+ m	10	132935005
ebi-a-GCST90001946	rs75216873	CD33 on CD14+ m	14	53742598

ebi-a-GCST90001946	rs139876813	CD33 on CD14+ m	17	2689271
ebi-a-GCST90001946	rs78949787	CD33 on CD14+ m	19	51591138
ebi-a-GCST90001946	rs7351079	CD33 on CD14+ m	19	51574038
ebi-a-GCST90001946	rs17828251	CD33 on CD14+ m	19	2553602
ebi-a-GCST90001946	rs58483275	CD33 on CD14+ m	19	51868443
ebi-a-GCST90001946	rs11882720	CD33 on CD14+ m	19	52897142
ebi-a-GCST90001946	rs3865444	CD33 on CD14+ m	19	51727962
ebi-a-GCST90001946	rs558625031	CD33 on CD14+ m	19	51858911
ebi-a-GCST90001946	rs4802858	CD33 on CD14+ m	19	52253150
ebi-a-GCST90001946	rs3911857	CD33 on CD14+ m	20	58100977
ebi-a-GCST90001947	rs6679331	CD33 on CD33br H	1	244420967
ebi-a-GCST90001947	rs2362605	CD33 on CD33br H	3	190480177
ebi-a-GCST90001947	rs1868503	CD33 on CD33br H	3	54426911
ebi-a-GCST90001947	rs77738348	CD33 on CD33br H	4	97655232
ebi-a-GCST90001947	rs77011252	CD33 on CD33br H	5	159069094
ebi-a-GCST90001947	rs13181441	CD33 on CD33br H	5	6869027
ebi-a-GCST90001947	rs6892757	CD33 on CD33br H	5	743878
ebi-a-GCST90001947	rs72957739	CD33 on CD33br H	6	115952566
ebi-a-GCST90001947	rs149970449	CD33 on CD33br H	7	33577760
ebi-a-GCST90001947	rs1622346	CD33 on CD33br H	9	98494874
ebi-a-GCST90001947	rs7047105	CD33 on CD33br H	9	105951980
ebi-a-GCST90001947	rs114090793	CD33 on CD33br H	10	132935005
ebi-a-GCST90001947	rs111918250	CD33 on CD33br H	10	21637249
ebi-a-GCST90001947	rs75216873	CD33 on CD33br H	14	53742598
ebi-a-GCST90001947	rs4442789	CD33 on CD33br H	15	98373329
ebi-a-GCST90001947	rs11073171	CD33 on CD33br H	15	36675994
ebi-a-GCST90001947	rs140051672	CD33 on CD33br H	16	86460135
ebi-a-GCST90001947	rs139876813	CD33 on CD33br H	17	2689271
ebi-a-GCST90001947	rs11078779	CD33 on CD33br H	17	8952193
ebi-a-GCST90001947	rs1397071	CD33 on CD33br H	18	68477432
ebi-a-GCST90001947	rs558625031	CD33 on CD33br H	19	51858911
ebi-a-GCST90001947	rs7351079	CD33 on CD33br H	19	51574038
ebi-a-GCST90001947	rs78949787	CD33 on CD33br H	19	51591138
ebi-a-GCST90001947	rs3865444	CD33 on CD33br H	19	51727962
ebi-a-GCST90001947	rs58301181	CD33 on CD33br H	19	52097261
ebi-a-GCST90001947	rs73051934	CD33 on CD33br H	19	48582916
ebi-a-GCST90001947	rs11907700	CD33 on CD33br H	20	52417816
ebi-a-GCST90001947	rs2849905	CD33 on CD33br H	21	18957035
ebi-a-GCST90001948	rs1801274	CD33 on CD33dim	1	161479745
ebi-a-GCST90001948	rs13004897	CD33 on CD33dim	2	143238505
ebi-a-GCST90001948	rs11720960	CD33 on CD33dim	3	123957942
ebi-a-GCST90001948	rs9461212	CD33 on CD33dim	6	25783622
ebi-a-GCST90001948	rs9493075	CD33 on CD33dim	6	132038487
ebi-a-GCST90001948	rs10953963	CD33 on CD33dim	7	122553227
ebi-a-GCST90001948	rs113460642	CD33 on CD33dim	8	66569270
ebi-a-GCST90001948	rs10869456	CD33 on CD33dim	9	77499330
ebi-a-GCST90001948	rs7047105	CD33 on CD33dim	9	105951980
ebi-a-GCST90001948	rs12337816	CD33 on CD33dim	9	117125236

ebi-a-GCST90001948	rs114090793	CD33 on CD33dim	10	132935005
ebi-a-GCST90001948	rs75216873	CD33 on CD33dim	14	53742598
ebi-a-GCST90001948	rs139876813	CD33 on CD33dim	17	2689271
ebi-a-GCST90001948	rs138564050	CD33 on CD33dim	18	2873692
ebi-a-GCST90001948	rs11882720	CD33 on CD33dim	19	52897142
ebi-a-GCST90001948	rs78949787	CD33 on CD33dim	19	51591138
ebi-a-GCST90001948	rs3865444	CD33 on CD33dim	19	51727962
ebi-a-GCST90001948	rs558625031	CD33 on CD33dim	19	51858911
ebi-a-GCST90001948	rs58483275	CD33 on CD33dim	19	51868443
ebi-a-GCST90001948	rs4802858	CD33 on CD33dim	19	52253150
ebi-a-GCST90001948	rs7351079	CD33 on CD33dim	19	51574038
ebi-a-GCST90001948	rs17828251	CD33 on CD33dim	19	2553602
ebi-a-GCST90001948	rs112807165	CD33 on CD33dim	20	38092873
ebi-a-GCST90001948	rs6100482	CD33 on CD33dim	20	58103645
ebi-a-GCST90001949	rs1801274	CD33 on CD33dim	1	161479745
ebi-a-GCST90001949	rs11720960	CD33 on CD33dim	3	123957942
ebi-a-GCST90001949	rs10033650	CD33 on CD33dim	4	53954014
ebi-a-GCST90001949	rs187981347	CD33 on CD33dim	5	172080662
ebi-a-GCST90001949	rs9493075	CD33 on CD33dim	6	132038487
ebi-a-GCST90001949	rs7798098	CD33 on CD33dim	7	36494338
ebi-a-GCST90001949	rs113460642	CD33 on CD33dim	8	66569270
ebi-a-GCST90001949	rs10869456	CD33 on CD33dim	9	77499330
ebi-a-GCST90001949	rs35305059	CD33 on CD33dim	12	128658240
ebi-a-GCST90001949	rs75216873	CD33 on CD33dim	14	53742598
ebi-a-GCST90001949	rs4442789	CD33 on CD33dim	15	98373329
ebi-a-GCST90001949	rs139876813	CD33 on CD33dim	17	2689271
ebi-a-GCST90001949	rs138564050	CD33 on CD33dim	18	2873692
ebi-a-GCST90001949	rs73051934	CD33 on CD33dim	19	48582916
ebi-a-GCST90001949	rs58301181	CD33 on CD33dim	19	52097261
ebi-a-GCST90001949	rs7351079	CD33 on CD33dim	19	51574038
ebi-a-GCST90001949	rs3865444	CD33 on CD33dim	19	51727962
ebi-a-GCST90001949	rs76647540	CD33 on CD33dim	19	51088199
ebi-a-GCST90001949	rs558625031	CD33 on CD33dim	19	51858911
ebi-a-GCST90001949	rs4802858	CD33 on CD33dim	19	52253150
ebi-a-GCST90001949	rs78949787	CD33 on CD33dim	19	51591138
ebi-a-GCST90001949	rs6100482	CD33 on CD33dim	20	58103645
ebi-a-GCST90001949	rs112807165	CD33 on CD33dim	20	38092873
ebi-a-GCST90001950	rs2243196	CD33 on Gr MDSC	1	207011019
ebi-a-GCST90001950	rs138913343	CD33 on Gr MDSC	2	109128545
ebi-a-GCST90001950	rs60467414	CD33 on Gr MDSC	4	163340178
ebi-a-GCST90001950	rs4148886	CD33 on Gr MDSC	4	100064649
ebi-a-GCST90001950	rs7729727	CD33 on Gr MDSC	5	73692240
ebi-a-GCST90001950	rs74725916	CD33 on Gr MDSC	6	116418776
ebi-a-GCST90001950	rs11762203	CD33 on Gr MDSC	7	120912956
ebi-a-GCST90001950	rs149890580	CD33 on Gr MDSC	9	123280236
ebi-a-GCST90001950	rs10759715	CD33 on Gr MDSC	9	117277295
ebi-a-GCST90001950	rs72833791	CD33 on Gr MDSC	10	115406173
ebi-a-GCST90001950	rs1164675	CD33 on Gr MDSC	11	49325972

ebi-a-GCST90001950	rs1466426	CD33 on Gr MDSC	11	10518289
ebi-a-GCST90001950	rs10876353	CD33 on Gr MDSC	12	53217056
ebi-a-GCST90001950	rs71468862	CD33 on Gr MDSC	15	37805271
ebi-a-GCST90001950	rs139876813	CD33 on Gr MDSC	17	2689271
ebi-a-GCST90001950	rs7351079	CD33 on Gr MDSC	19	51574038
ebi-a-GCST90001950	rs558625031	CD33 on Gr MDSC	19	51858911
ebi-a-GCST90001950	rs12459503	CD33 on Gr MDSC	19	52161035
ebi-a-GCST90001950	rs78949787	CD33 on Gr MDSC	19	51591138
ebi-a-GCST90001950	rs7245846	CD33 on Gr MDSC	19	51731176
ebi-a-GCST90001951	rs77246389	CD33 on CD66b++	1	45733581
ebi-a-GCST90001951	rs76026141	CD33 on CD66b++	1	88174972
ebi-a-GCST90001951	rs79910465	CD33 on CD66b++	1	233086394
ebi-a-GCST90001951	rs144205288	CD33 on CD66b++	2	34988773
ebi-a-GCST90001951	rs10510189	CD33 on CD66b++	3	1316884
ebi-a-GCST90001951	rs139845236	CD33 on CD66b++	3	187673519
ebi-a-GCST90001951	rs17409678	CD33 on CD66b++	6	76105426
ebi-a-GCST90001951	rs7853685	CD33 on CD66b++	9	77507034
ebi-a-GCST90001951	rs61900137	CD33 on CD66b++	11	83237517
ebi-a-GCST90001951	rs72681960	CD33 on CD66b++	14	23350478
ebi-a-GCST90001951	rs139876813	CD33 on CD66b++	17	2689271
ebi-a-GCST90001951	rs4802858	CD33 on CD66b++	19	52253150
ebi-a-GCST90001951	rs12459503	CD33 on CD66b++	19	52161035
ebi-a-GCST90001951	rs78949787	CD33 on CD66b++	19	51591138
ebi-a-GCST90001951	rs73026163	CD33 on CD66b++	19	31994813
ebi-a-GCST90001951	rs7351079	CD33 on CD66b++	19	51574038
ebi-a-GCST90001951	rs76647540	CD33 on CD66b++	19	51088199
ebi-a-GCST90001951	rs3865444	CD33 on CD66b++	19	51727962
ebi-a-GCST90001951	rs558625031	CD33 on CD66b++	19	51858911
ebi-a-GCST90001952	rs10164761	CD33 on Mo MDS(	2	220066209
ebi-a-GCST90001952	rs2362605	CD33 on Mo MDS(	3	190480177
ebi-a-GCST90001952	rs9867350	CD33 on Mo MDS(	3	129451519
ebi-a-GCST90001952	rs403286	CD33 on Mo MDS(	3	55264003
ebi-a-GCST90001952	rs6828373	CD33 on Mo MDS(	4	154476924
ebi-a-GCST90001952	rs10022829	CD33 on Mo MDS(	4	506471
ebi-a-GCST90001952	rs6879785	CD33 on Mo MDS(	5	75450373
ebi-a-GCST90001952	rs73015201	CD33 on Mo MDS(	6	148351645
ebi-a-GCST90001952	rs6974483	CD33 on Mo MDS(	7	52705752
ebi-a-GCST90001952	rs7047105	CD33 on Mo MDS(	9	105951980
ebi-a-GCST90001952	rs138138479	CD33 on Mo MDS(	10	22339808
ebi-a-GCST90001952	rs4747079	CD33 on Mo MDS(	10	72466435
ebi-a-GCST90001952	rs73259850	CD33 on Mo MDS(	10	56897405
ebi-a-GCST90001952	rs10861528	CD33 on Mo MDS(	12	106266488
ebi-a-GCST90001952	rs117150385	CD33 on Mo MDS(	17	9157491
ebi-a-GCST90001952	rs58301181	CD33 on Mo MDS(	19	52097261
ebi-a-GCST90001952	rs3865444	CD33 on Mo MDS(	19	51727962
ebi-a-GCST90001952	rs8108687	CD33 on Mo MDS(	19	927971
ebi-a-GCST90001952	rs7351079	CD33 on Mo MDS(	19	51574038
ebi-a-GCST90001952	rs62114147	CD33 on Mo MDS(	19	51588018

ebi-a-GCST90001952	rs73051934	CD33 on Mo MDSC	19	48582916
ebi-a-GCST90001953	rs79349965	CD33 on CD33dim	1	248009263
ebi-a-GCST90001953	rs56043070	CD33 on CD33dim	1	247719769
ebi-a-GCST90001953	rs57883729	CD33 on CD33dim	2	162100169
ebi-a-GCST90001953	rs4709427	CD33 on CD33dim	6	158023339
ebi-a-GCST90001953	rs11762203	CD33 on CD33dim	7	120912956
ebi-a-GCST90001953	rs6974483	CD33 on CD33dim	7	52705752
ebi-a-GCST90001953	rs6979650	CD33 on CD33dim	7	36536896
ebi-a-GCST90001953	rs3807849	CD33 on CD33dim	7	8157710
ebi-a-GCST90001953	rs58337014	CD33 on CD33dim	10	53354043
ebi-a-GCST90001953	rs117140964	CD33 on CD33dim	10	101609403
ebi-a-GCST90001953	rs34497847	CD33 on CD33dim	11	76766923
ebi-a-GCST90001953	rs4567661	CD33 on CD33dim	15	38786114
ebi-a-GCST90001953	rs77635331	CD33 on CD33dim	16	8691661
ebi-a-GCST90001953	rs8069226	CD33 on CD33dim	17	8938562
ebi-a-GCST90001953	rs548194341	CD33 on CD33dim	17	9130998
ebi-a-GCST90001953	rs3865444	CD33 on CD33dim	19	51727962
ebi-a-GCST90001953	rs11669394	CD33 on CD33dim	19	53262327
ebi-a-GCST90001953	rs11882720	CD33 on CD33dim	19	52897142
ebi-a-GCST90001953	rs75773078	CD33 on CD33dim	19	51742145
ebi-a-GCST90001953	rs138712525	CD33 on CD33dim	19	51544665
ebi-a-GCST90001953	rs7351079	CD33 on CD33dim	19	51574038
ebi-a-GCST90001954	rs56043070	CD33 on basophil	1	247719769
ebi-a-GCST90001954	rs79349965	CD33 on basophil	1	248009263
ebi-a-GCST90001954	rs57883729	CD33 on basophil	2	162100169
ebi-a-GCST90001954	rs72640540	CD33 on basophil	4	67038487
ebi-a-GCST90001954	rs1836704	CD33 on basophil	4	30413400
ebi-a-GCST90001954	rs10040068	CD33 on basophil	5	17802169
ebi-a-GCST90001954	rs6974483	CD33 on basophil	7	52705752
ebi-a-GCST90001954	rs6979650	CD33 on basophil	7	36536896
ebi-a-GCST90001954	rs58337014	CD33 on basophil	10	53354043
ebi-a-GCST90001954	rs117140964	CD33 on basophil	10	101609403
ebi-a-GCST90001954	rs4567661	CD33 on basophil	15	38786114
ebi-a-GCST90001954	rs77635331	CD33 on basophil	16	8691661
ebi-a-GCST90001954	rs462331	CD33 on basophil	16	83832299
ebi-a-GCST90001954	rs548194341	CD33 on basophil	17	9130998
ebi-a-GCST90001954	rs8069226	CD33 on basophil	17	8938562
ebi-a-GCST90001954	rs111695695	CD33 on basophil	19	51897760
ebi-a-GCST90001954	rs79377141	CD33 on basophil	19	51538952
ebi-a-GCST90001954	rs7245846	CD33 on basophil	19	51731176
ebi-a-GCST90001954	rs75773078	CD33 on basophil	19	51742145
ebi-a-GCST90001954	rs114475810	CD33 on basophil	19	36056431
ebi-a-GCST90001954	rs141122591	CD33 on basophil	19	51900858
ebi-a-GCST90001954	rs7351079	CD33 on basophil	19	51574038
ebi-a-GCST90001954	rs58483275	CD33 on basophil	19	51868443
ebi-a-GCST90001955	rs56043070	CD33 on Im MDSC	1	247719769
ebi-a-GCST90001955	rs2722603	CD33 on Im MDSC	2	5890815
ebi-a-GCST90001955	rs74707127	CD33 on Im MDSC	2	174284380

ebi-a-GCST90001955	rs10022829	CD33 on Im MDSC	4	506471
ebi-a-GCST90001955	rs10040068	CD33 on Im MDSC	5	17802169
ebi-a-GCST90001955	rs11765781	CD33 on Im MDSC	7	4764861
ebi-a-GCST90001955	rs117140964	CD33 on Im MDSC	10	101609403
ebi-a-GCST90001955	rs1922127	CD33 on Im MDSC	10	53348048
ebi-a-GCST90001955	rs77040155	CD33 on Im MDSC	11	5402836
ebi-a-GCST90001955	rs1421461	CD33 on Im MDSC	12	106320886
ebi-a-GCST90001955	rs1218959	CD33 on Im MDSC	13	28193877
ebi-a-GCST90001955	rs79965644	CD33 on Im MDSC	13	79484369
ebi-a-GCST90001955	rs4567661	CD33 on Im MDSC	15	38786114
ebi-a-GCST90001955	rs77635331	CD33 on Im MDSC	16	8691661
ebi-a-GCST90001955	rs548194341	CD33 on Im MDSC	17	9130998
ebi-a-GCST90001955	rs1397071	CD33 on Im MDSC	18	68477432
ebi-a-GCST90001955	rs79377141	CD33 on Im MDSC	19	51538952
ebi-a-GCST90001955	rs7351079	CD33 on Im MDSC	19	51574038
ebi-a-GCST90001955	rs75773078	CD33 on Im MDSC	19	51742145
ebi-a-GCST90001955	rs141122591	CD33 on Im MDSC	19	51900858
ebi-a-GCST90001955	rs8105208	CD33 on Im MDSC	19	29522573
ebi-a-GCST90001955	rs7245846	CD33 on Im MDSC	19	51731176
ebi-a-GCST90001955	rs58483275	CD33 on Im MDSC	19	51868443
ebi-a-GCST90001955	rs111695695	CD33 on Im MDSC	19	51897760
ebi-a-GCST90001955	rs117893093	CD33 on Im MDSC	20	36543774
ebi-a-GCST90001956	rs6679331	CD33 on CD33br H	1	244420967
ebi-a-GCST90001956	rs6679371	CD33 on CD33br H	1	246566874
ebi-a-GCST90001956	rs1868503	CD33 on CD33br H	3	54426911
ebi-a-GCST90001956	rs2362605	CD33 on CD33br H	3	190480177
ebi-a-GCST90001956	rs17580821	CD33 on CD33br H	4	114358371
ebi-a-GCST90001956	rs6892757	CD33 on CD33br H	5	743878
ebi-a-GCST90001956	rs72957739	CD33 on CD33br H	6	115952566
ebi-a-GCST90001956	rs77462109	CD33 on CD33br H	7	33186065
ebi-a-GCST90001956	rs7047105	CD33 on CD33br H	9	105951980
ebi-a-GCST90001956	rs114090793	CD33 on CD33br H	10	132935005
ebi-a-GCST90001956	rs111918250	CD33 on CD33br H	10	21637249
ebi-a-GCST90001956	rs75216873	CD33 on CD33br H	14	53742598
ebi-a-GCST90001956	rs4442789	CD33 on CD33br H	15	98373329
ebi-a-GCST90001956	rs140051672	CD33 on CD33br H	16	86460135
ebi-a-GCST90001956	rs139876813	CD33 on CD33br H	17	2689271
ebi-a-GCST90001956	rs11078779	CD33 on CD33br H	17	8952193
ebi-a-GCST90001956	rs1397071	CD33 on CD33br H	18	68477432
ebi-a-GCST90001956	rs558625031	CD33 on CD33br H	19	51858911
ebi-a-GCST90001956	rs78949787	CD33 on CD33br H	19	51591138
ebi-a-GCST90001956	rs58301181	CD33 on CD33br H	19	52097261
ebi-a-GCST90001956	rs7351079	CD33 on CD33br H	19	51574038
ebi-a-GCST90001956	rs3865444	CD33 on CD33br H	19	51727962
ebi-a-GCST90001956	rs73051934	CD33 on CD33br H	19	48582916
ebi-a-GCST90001956	rs2849905	CD33 on CD33br H	21	18957035
ebi-a-GCST90001957	rs6679331	CD33 on CD33br H	1	244420967
ebi-a-GCST90001957	rs1868503	CD33 on CD33br H	3	54426911

ebi-a-GCST90001957	rs2362605	CD33 on CD33br H	3	190480177
ebi-a-GCST90001957	rs17580821	CD33 on CD33br H	4	114358371
ebi-a-GCST90001957	rs6892757	CD33 on CD33br H	5	743878
ebi-a-GCST90001957	rs72957739	CD33 on CD33br H	6	115952566
ebi-a-GCST90001957	rs77462109	CD33 on CD33br H	7	33186065
ebi-a-GCST90001957	rs7047105	CD33 on CD33br H	9	105951980
ebi-a-GCST90001957	rs111918250	CD33 on CD33br H	10	21637249
ebi-a-GCST90001957	rs114090793	CD33 on CD33br H	10	132935005
ebi-a-GCST90001957	rs78521312	CD33 on CD33br H	11	132496896
ebi-a-GCST90001957	rs75216873	CD33 on CD33br H	14	53742598
ebi-a-GCST90001957	rs4442789	CD33 on CD33br H	15	98373329
ebi-a-GCST90001957	rs139876813	CD33 on CD33br H	17	2689271
ebi-a-GCST90001957	rs11078779	CD33 on CD33br H	17	8952193
ebi-a-GCST90001957	rs1397071	CD33 on CD33br H	18	68477432
ebi-a-GCST90001957	rs7351079	CD33 on CD33br H	19	51574038
ebi-a-GCST90001957	rs558625031	CD33 on CD33br H	19	51858911
ebi-a-GCST90001957	rs58301181	CD33 on CD33br H	19	52097261
ebi-a-GCST90001957	rs3865444	CD33 on CD33br H	19	51727962
ebi-a-GCST90001957	rs73051934	CD33 on CD33br H	19	48582916
ebi-a-GCST90001957	rs78949787	CD33 on CD33br H	19	51591138
ebi-a-GCST90001958	rs56754976	CD4 on monocyte	1	180568712
ebi-a-GCST90001958	rs661686	CD4 on monocyte	1	85909210
ebi-a-GCST90001958	rs1801274	CD4 on monocyte	1	161479745
ebi-a-GCST90001958	rs1365928	CD4 on monocyte	2	19779555
ebi-a-GCST90001958	rs116366652	CD4 on monocyte	2	160856904
ebi-a-GCST90001958	rs34551852	CD4 on monocyte	4	23605540
ebi-a-GCST90001958	rs150579420	CD4 on monocyte	4	154119021
ebi-a-GCST90001958	rs4073463	CD4 on monocyte	4	65562649
ebi-a-GCST90001958	rs141048893	CD4 on monocyte	5	175815591
ebi-a-GCST90001958	rs112496462	CD4 on monocyte	6	34155071
ebi-a-GCST90001958	rs11225443	CD4 on monocyte	11	102742951
ebi-a-GCST90001958	rs7976804	CD4 on monocyte	12	6467961
ebi-a-GCST90001958	rs7296859	CD4 on monocyte	12	6894164
ebi-a-GCST90001958	rs9302635	CD4 on monocyte	16	72144174
ebi-a-GCST90001958	rs117535998	CD4 on monocyte	16	71579132
ebi-a-GCST90001958	rs78206220	CD4 on monocyte	18	27682817
ebi-a-GCST90001959	rs10518646	CD4 on HLA DR+	1	81127816
ebi-a-GCST90001959	rs10047088	CD4 on HLA DR+	1	228004140
ebi-a-GCST90001959	rs10179730	CD4 on HLA DR+	2	188410351
ebi-a-GCST90001959	rs11929631	CD4 on HLA DR+	3	164980538
ebi-a-GCST90001959	rs13316780	CD4 on HLA DR+	3	108851829
ebi-a-GCST90001959	rs17002700	CD4 on HLA DR+	4	78417537
ebi-a-GCST90001959	rs184989157	CD4 on HLA DR+	4	4214108
ebi-a-GCST90001959	rs10517576	CD4 on HLA DR+	4	154561623
ebi-a-GCST90001959	rs35032648	CD4 on HLA DR+	5	3225418
ebi-a-GCST90001959	rs166223	CD4 on HLA DR+	5	86031019
ebi-a-GCST90001959	rs9357097	CD4 on HLA DR+	6	30285121
ebi-a-GCST90001959	rs10946649	CD4 on HLA DR+	6	23957018

ebi-a-GCST90001959	rs2524081	CD4 on HLA DR+	6	31242083
ebi-a-GCST90001959	rs4371876	CD4 on HLA DR+	6	31067550
ebi-a-GCST90001959	rs17088862	CD4 on HLA DR+	8	22835142
ebi-a-GCST90001959	rs7819104	CD4 on HLA DR+	8	121748105
ebi-a-GCST90001959	rs12684941	CD4 on HLA DR+	9	37654930
ebi-a-GCST90001959	rs75559970	CD4 on HLA DR+	10	100518962
ebi-a-GCST90001959	rs72822577	CD4 on HLA DR+	10	97168100
ebi-a-GCST90001959	rs11614716	CD4 on HLA DR+	12	6904146
ebi-a-GCST90001959	rs35996890	CD4 on HLA DR+	15	50180635
ebi-a-GCST90001959	rs75036101	CD4 on HLA DR+	16	66348814
ebi-a-GCST90001960	rs12095517	CD25 on CD4+	1	11797186
ebi-a-GCST90001960	rs71673415	CD25 on CD4+	1	111786150
ebi-a-GCST90001960	rs16860924	CD25 on CD4+	2	14031175
ebi-a-GCST90001960	rs13060740	CD25 on CD4+	3	151674909
ebi-a-GCST90001960	rs906242	CD25 on CD4+	4	11050147
ebi-a-GCST90001960	rs79647440	CD25 on CD4+	7	142438302
ebi-a-GCST90001960	rs112393619	CD25 on CD4+	7	21800888
ebi-a-GCST90001960	rs72666777	CD25 on CD4+	8	69587840
ebi-a-GCST90001960	rs80020399	CD25 on CD4+	9	124757711
ebi-a-GCST90001960	rs17814462	CD25 on CD4+	9	4598881
ebi-a-GCST90001960	rs77506115	CD25 on CD4+	9	35553793
ebi-a-GCST90001960	rs61839660	CD25 on CD4+	10	6094697
ebi-a-GCST90001960	rs61875444	CD25 on CD4+	11	12418190
ebi-a-GCST90001960	rs118110714	CD25 on CD4+	12	16060925
ebi-a-GCST90001960	rs9603769	CD25 on CD4+	13	41104740
ebi-a-GCST90001960	rs661825	CD25 on CD4+	19	7616474
ebi-a-GCST90001960	rs142428460	CD25 on CD4+	20	12005003
ebi-a-GCST90001961	rs143264473	CD25 on CD39+ C	1	226979781
ebi-a-GCST90001961	rs72753706	CD25 on CD39+ C	1	234064576
ebi-a-GCST90001961	rs115446744	CD25 on CD39+ C	1	206716987
ebi-a-GCST90001961	rs28763878	CD25 on CD39+ C	2	189868535
ebi-a-GCST90001961	rs56048248	CD25 on CD39+ C	3	118862208
ebi-a-GCST90001961	rs6841503	CD25 on CD39+ C	4	25987977
ebi-a-GCST90001961	rs77797161	CD25 on CD39+ C	7	158434884
ebi-a-GCST90001961	rs36066449	CD25 on CD39+ C	8	140875665
ebi-a-GCST90001961	rs10964730	CD25 on CD39+ C	9	20826029
ebi-a-GCST90001961	rs148377476	CD25 on CD39+ C	10	86408004
ebi-a-GCST90001961	rs141109119	CD25 on CD39+ C	10	93071426
ebi-a-GCST90001961	rs11187515	CD25 on CD39+ C	10	95327429
ebi-a-GCST90001961	rs4075310	CD25 on CD39+ C	10	97574689
ebi-a-GCST90001961	rs7079761	CD25 on CD39+ C	10	97842115
ebi-a-GCST90001961	rs35025323	CD25 on CD39+ C	10	97089991
ebi-a-GCST90001961	rs113594239	CD25 on CD39+ C	10	98103215
ebi-a-GCST90001961	rs12778618	CD25 on CD39+ C	10	95633120
ebi-a-GCST90001961	rs116354946	CD25 on CD39+ C	11	9339804
ebi-a-GCST90001961	rs142179684	CD25 on CD39+ C	15	80416513
ebi-a-GCST90001961	rs112207505	CD25 on CD39+ C	16	87927291
ebi-a-GCST90001961	rs9892509	CD25 on CD39+ C	17	60434343

ebi-a-GCST90001961	rs74821035	CD25 on CD39+ C	18	42881701
ebi-a-GCST90001961	rs1618134	CD25 on CD39+ C	21	25490355
ebi-a-GCST90001962	rs115939244	CD25 on CD28+ C	1	3165628
ebi-a-GCST90001962	rs71673415	CD25 on CD28+ C	1	111786150
ebi-a-GCST90001962	rs7535297	CD25 on CD28+ C	1	25513931
ebi-a-GCST90001962	rs4632330	CD25 on CD28+ C	2	220737809
ebi-a-GCST90001962	rs13060740	CD25 on CD28+ C	3	151674909
ebi-a-GCST90001963	rs55971447	FSC-A on myeloid	1	161515326
ebi-a-GCST90001963	rs180827789	FSC-A on myeloid	2	205277145
ebi-a-GCST90001963	rs1837001	FSC-A on myeloid	2	5717130
ebi-a-GCST90001963	rs72926952	FSC-A on myeloid	2	203485086
ebi-a-GCST90001963	rs114452046	FSC-A on myeloid	2	109542930
ebi-a-GCST90001963	rs4456651	FSC-A on myeloid	2	236068863
ebi-a-GCST90001963	rs1364526	FSC-A on myeloid	2	57315946
ebi-a-GCST90001963	rs73187869	FSC-A on myeloid	3	119113344
ebi-a-GCST90001963	rs34437438	FSC-A on myeloid	3	95531331
ebi-a-GCST90001963	rs28424875	FSC-A on myeloid	4	42664508
ebi-a-GCST90001963	rs66968042	FSC-A on myeloid	7	82021397
ebi-a-GCST90001963	rs4278118	FSC-A on myeloid	8	127712638
ebi-a-GCST90001963	rs12680377	FSC-A on myeloid	8	5739227
ebi-a-GCST90001963	rs17457355	FSC-A on myeloid	9	132098004
ebi-a-GCST90001963	rs117740023	FSC-A on myeloid	10	52741522
ebi-a-GCST90001963	rs117292692	FSC-A on myeloid	10	72720949
ebi-a-GCST90001963	rs149524008	FSC-A on myeloid	12	104048446
ebi-a-GCST90001963	rs10492689	FSC-A on myeloid	13	39544533
ebi-a-GCST90001963	rs1163654	FSC-A on myeloid	13	112246767
ebi-a-GCST90001963	rs4769841	FSC-A on myeloid	13	31108727
ebi-a-GCST90001963	rs2956237	FSC-A on myeloid	15	101268827
ebi-a-GCST90001964	rs146943490	FSC-A on plasmacy	1	218248849
ebi-a-GCST90001964	rs74664695	FSC-A on plasmacy	1	102643305
ebi-a-GCST90001964	rs77019448	FSC-A on plasmacy	2	4345667
ebi-a-GCST90001964	rs115700682	FSC-A on plasmacy	2	11423312
ebi-a-GCST90001964	rs112068187	FSC-A on plasmacy	2	34890769
ebi-a-GCST90001964	rs2084848	FSC-A on plasmacy	2	169211909
ebi-a-GCST90001964	rs2289321	FSC-A on plasmacy	2	111870220
ebi-a-GCST90001964	rs4431216	FSC-A on plasmacy	4	84150313
ebi-a-GCST90001964	rs149624533	FSC-A on plasmacy	5	174332776
ebi-a-GCST90001964	rs9293809	FSC-A on plasmacy	5	79565071
ebi-a-GCST90001964	rs116474260	FSC-A on plasmacy	5	151202476
ebi-a-GCST90001964	rs77658339	FSC-A on plasmacy	5	107075296
ebi-a-GCST90001964	rs57869679	FSC-A on plasmacy	7	142512769
ebi-a-GCST90001964	rs73219706	FSC-A on plasmacy	7	89767780
ebi-a-GCST90001964	rs4317514	FSC-A on plasmacy	7	127095652
ebi-a-GCST90001964	rs2618014	FSC-A on plasmacy	9	26317973
ebi-a-GCST90001964	rs79205010	FSC-A on plasmacy	12	103996660
ebi-a-GCST90001964	rs9519437	FSC-A on plasmacy	13	105303025
ebi-a-GCST90001964	rs142005014	FSC-A on plasmacy	13	64051675
ebi-a-GCST90001964	rs242761	FSC-A on plasmacy	14	90077960

ebi-a-GCST90001964	rs62045817	FSC-A on plasmacy	16	89045233
ebi-a-GCST90001964	rs59715840	FSC-A on plasmacy	16	79180312
ebi-a-GCST90001964	rs722926	FSC-A on plasmacy	17	8677215
ebi-a-GCST90001964	rs144843975	FSC-A on plasmacy	17	63066415
ebi-a-GCST90001964	rs117067704	FSC-A on plasmacy	17	62128083
ebi-a-GCST90001964	rs4801716	FSC-A on plasmacy	19	56858865
ebi-a-GCST90001964	rs35534776	FSC-A on plasmacy	19	55107308
ebi-a-GCST90001964	rs9626174	FSC-A on plasmacy	22	45991939
ebi-a-GCST90001965	rs150649461	FSC-A on monocyt	1	92925654
ebi-a-GCST90001965	rs7519224	FSC-A on monocyt	1	15559482
ebi-a-GCST90001965	rs7557343	FSC-A on monocyt	2	5733323
ebi-a-GCST90001965	rs112698900	FSC-A on monocyt	2	175649304
ebi-a-GCST90001965	rs77762937	FSC-A on monocyt	2	203467470
ebi-a-GCST90001965	rs535652693	FSC-A on monocyt	3	6519552
ebi-a-GCST90001965	rs2013828	FSC-A on monocyt	3	1262474
ebi-a-GCST90001965	rs12646158	FSC-A on monocyt	4	14345720
ebi-a-GCST90001965	rs28424875	FSC-A on monocyt	4	42664508
ebi-a-GCST90001965	rs76378762	FSC-A on monocyt	5	87683717
ebi-a-GCST90001965	rs116492121	FSC-A on monocyt	5	20399
ebi-a-GCST90001965	rs77658339	FSC-A on monocyt	5	107075296
ebi-a-GCST90001965	rs532311082	FSC-A on monocyt	6	108592381
ebi-a-GCST90001965	rs2106353	FSC-A on monocyt	7	126506598
ebi-a-GCST90001965	rs141889079	FSC-A on monocyt	8	68736534
ebi-a-GCST90001965	rs4910344	FSC-A on monocyt	11	11450673
ebi-a-GCST90001965	rs1800973	FSC-A on monocyt	12	69744014
ebi-a-GCST90001965	rs35241999	FSC-A on monocyt	12	11184504
ebi-a-GCST90001965	rs12868834	FSC-A on monocyt	13	103681773
ebi-a-GCST90001965	rs10492689	FSC-A on monocyt	13	39544533
ebi-a-GCST90001965	rs2326401	FSC-A on monocyt	16	84929974
ebi-a-GCST90001965	rs3787199	FSC-A on monocyt	20	50096687
ebi-a-GCST90001966	rs58985199	FSC-A on granuloc	1	37569165
ebi-a-GCST90001966	rs145847454	FSC-A on granuloc	2	241090399
ebi-a-GCST90001966	rs3179891	FSC-A on granuloc	2	43319978
ebi-a-GCST90001966	rs13411484	FSC-A on granuloc	2	147467507
ebi-a-GCST90001966	rs112157475	FSC-A on granuloc	2	195118976
ebi-a-GCST90001966	rs72615608	FSC-A on granuloc	3	145320303
ebi-a-GCST90001966	rs12486053	FSC-A on granuloc	3	186182741
ebi-a-GCST90001966	rs146287173	FSC-A on granuloc	4	14288498
ebi-a-GCST90001966	rs153514	FSC-A on granuloc	5	142980181
ebi-a-GCST90001966	rs915125	FSC-A on granuloc	6	82463376
ebi-a-GCST90001966	rs445	FSC-A on granuloc	7	92408370
ebi-a-GCST90001966	rs80177268	FSC-A on granuloc	7	90911672
ebi-a-GCST90001966	rs42031	FSC-A on granuloc	7	92237396
ebi-a-GCST90001966	rs12675298	FSC-A on granuloc	8	6828813
ebi-a-GCST90001966	rs2978899	FSC-A on granuloc	8	6691598
ebi-a-GCST90001966	rs2306975	FSC-A on granuloc	8	48353258
ebi-a-GCST90001966	rs113949273	FSC-A on granuloc	12	115749077
ebi-a-GCST90001966	rs142088303	FSC-A on granuloc	13	40929342

ebi-a-GCST90001966	rs144666992	FSC-A on granuloc	13	45184855
ebi-a-GCST90001966	rs72821867	FSC-A on granuloc	17	38833815
ebi-a-GCST90001966	rs73016817	FSC-A on granuloc	19	30803794
ebi-a-GCST90001966	rs421685	FSC-A on granuloc	19	54768528
ebi-a-GCST90001966	rs11697729	FSC-A on granuloc	20	45732252
ebi-a-GCST90001967	rs192378299	FSC-A on CD14+ r	2	40719499
ebi-a-GCST90001967	rs144745307	FSC-A on CD14+ r	3	1506086
ebi-a-GCST90001967	rs74558876	FSC-A on CD14+ r	3	180757108
ebi-a-GCST90001967	rs139494294	FSC-A on CD14+ r	3	27837769
ebi-a-GCST90001967	rs115639631	FSC-A on CD14+ r	4	157594374
ebi-a-GCST90001967	rs10488833	FSC-A on CD14+ r	4	97953756
ebi-a-GCST90001967	rs2501623	FSC-A on CD14+ r	6	118068345
ebi-a-GCST90001967	rs529563	FSC-A on CD14+ r	6	148252713
ebi-a-GCST90001967	rs34880703	FSC-A on CD14+ r	6	102985583
ebi-a-GCST90001967	rs55914843	FSC-A on CD14+ r	7	21111081
ebi-a-GCST90001967	rs143798663	FSC-A on CD14+ r	8	129392532
ebi-a-GCST90001967	rs309859	FSC-A on CD14+ r	8	29876365
ebi-a-GCST90001967	rs7894142	FSC-A on CD14+ r	10	15158267
ebi-a-GCST90001967	rs142456232	FSC-A on CD14+ r	11	4960640
ebi-a-GCST90001967	rs78736654	FSC-A on CD14+ r	11	5544477
ebi-a-GCST90001967	rs12364299	FSC-A on CD14+ r	11	5403108
ebi-a-GCST90001967	rs28659991	FSC-A on CD14+ r	11	68878311
ebi-a-GCST90001967	rs117045193	FSC-A on CD14+ r	13	49315399
ebi-a-GCST90001967	rs142074591	FSC-A on CD14+ r	13	59830957
ebi-a-GCST90001967	rs150082330	FSC-A on CD14+ r	15	24528125
ebi-a-GCST90001967	rs682039	FSC-A on CD14+ r	15	22527143
ebi-a-GCST90001967	rs72801520	FSC-A on CD14+ r	16	84971669
ebi-a-GCST90001967	rs72843128	FSC-A on CD14+ r	17	65761112
ebi-a-GCST90001967	rs11652123	FSC-A on CD14+ r	17	18870524
ebi-a-GCST90001967	rs6082136	FSC-A on CD14+ r	20	20725119
ebi-a-GCST90001967	rs3787953	FSC-A on CD14+ r	21	43175027
ebi-a-GCST90001968	rs12073480	FSC-A on B cell	1	166711398
ebi-a-GCST90001968	rs116325392	FSC-A on B cell	1	4629608
ebi-a-GCST90001968	rs75166756	FSC-A on B cell	3	150053008
ebi-a-GCST90001968	rs72616681	FSC-A on B cell	3	149784568
ebi-a-GCST90001968	rs113029775	FSC-A on B cell	5	98524035
ebi-a-GCST90001968	rs12214648	FSC-A on B cell	6	144814850
ebi-a-GCST90001968	rs36071966	FSC-A on B cell	7	48174275
ebi-a-GCST90001968	rs74680712	FSC-A on B cell	7	97313850
ebi-a-GCST90001968	rs7076599	FSC-A on B cell	10	26815673
ebi-a-GCST90001968	rs7119012	FSC-A on B cell	11	134632636
ebi-a-GCST90001968	rs73462585	FSC-A on B cell	11	21126948
ebi-a-GCST90001968	rs9666963	FSC-A on B cell	11	69213299
ebi-a-GCST90001968	rs78092605	FSC-A on B cell	11	4806674
ebi-a-GCST90001968	rs117694934	FSC-A on B cell	12	106509867
ebi-a-GCST90001968	rs8000574	FSC-A on B cell	13	109364348
ebi-a-GCST90001968	rs11861296	FSC-A on B cell	16	71850632
ebi-a-GCST90001968	rs6030209	FSC-A on B cell	20	41026002

ebi-a-GCST90001968	rs6096232	FSC-A on B cell	20	49637974
ebi-a-GCST90001968	rs6041273	FSC-A on B cell	20	12321372
ebi-a-GCST90001968	rs78491019	FSC-A on B cell	22	49819964
ebi-a-GCST90001969	rs10919543	FSC-A on NK	1	161508617
ebi-a-GCST90001969	rs6694654	FSC-A on NK	1	86178676
ebi-a-GCST90001969	rs2774933	FSC-A on NK	1	62637062
ebi-a-GCST90001969	rs2068330	FSC-A on NK	2	163237390
ebi-a-GCST90001969	rs73112545	FSC-A on NK	3	2699329
ebi-a-GCST90001969	rs145419574	FSC-A on NK	6	17386586
ebi-a-GCST90001969	rs1735091	FSC-A on NK	7	152996005
ebi-a-GCST90001969	rs12542554	FSC-A on NK	8	5579734
ebi-a-GCST90001969	rs79678187	FSC-A on NK	9	91859293
ebi-a-GCST90001969	rs1006368	FSC-A on NK	10	126346603
ebi-a-GCST90001969	rs146765347	FSC-A on NK	11	11413255
ebi-a-GCST90001969	rs12425743	FSC-A on NK	12	86546401
ebi-a-GCST90001969	rs78273995	FSC-A on NK	14	79113570
ebi-a-GCST90001969	rs16970975	FSC-A on NK	17	33770706
ebi-a-GCST90001969	rs34700818	FSC-A on NK	17	48945753
ebi-a-GCST90001969	rs9912354	FSC-A on NK	17	33805730
ebi-a-GCST90001969	rs117893932	FSC-A on NK	18	56570039
ebi-a-GCST90001969	rs111460281	FSC-A on NK	21	46437156
ebi-a-GCST90001969	rs55708007	FSC-A on NK	21	46813143
ebi-a-GCST90001969	rs5768144	FSC-A on NK	22	48307233
ebi-a-GCST90001970	rs569911	FSC-A on HLA DR	1	160798209
ebi-a-GCST90001970	rs113248693	FSC-A on HLA DR	1	157464822
ebi-a-GCST90001970	rs115805162	FSC-A on HLA DR	1	161410253
ebi-a-GCST90001970	rs71632979	FSC-A on HLA DR	1	161526397
ebi-a-GCST90001970	rs13416971	FSC-A on HLA DR	2	87833081
ebi-a-GCST90001970	rs11891318	FSC-A on HLA DR	2	130608376
ebi-a-GCST90001970	rs35536653	FSC-A on HLA DR	2	60291461
ebi-a-GCST90001970	rs12524004	FSC-A on HLA DR	6	144027987
ebi-a-GCST90001970	rs56365312	FSC-A on HLA DR	9	82500743
ebi-a-GCST90001970	rs117063850	FSC-A on HLA DR	9	7361263
ebi-a-GCST90001970	rs118133760	FSC-A on HLA DR	10	131453508
ebi-a-GCST90001970	rs1255169	FSC-A on HLA DR	11	95513497
ebi-a-GCST90001970	rs61896987	FSC-A on HLA DR	11	72765846
ebi-a-GCST90001970	rs147173736	FSC-A on HLA DR	11	512255
ebi-a-GCST90001970	rs35620656	FSC-A on HLA DR	12	125629103
ebi-a-GCST90001970	rs78518201	FSC-A on HLA DR	14	91817389
ebi-a-GCST90001970	rs34649904	FSC-A on HLA DR	14	60029747
ebi-a-GCST90001970	rs141070453	FSC-A on HLA DR	15	29516794
ebi-a-GCST90001970	rs11074932	FSC-A on HLA DR	16	10968336
ebi-a-GCST90001970	rs7206658	FSC-A on HLA DR	16	68916118
ebi-a-GCST90001970	rs139392177	FSC-A on HLA DR	22	18954840
ebi-a-GCST90001971	rs190188611	FSC-A on lymphoc	1	177556743
ebi-a-GCST90001971	rs10169473	FSC-A on lymphoc	2	224307687
ebi-a-GCST90001971	rs10191966	FSC-A on lymphoc	2	58932674
ebi-a-GCST90001971	rs76883038	FSC-A on lymphoc	2	115150773

ebi-a-GCST90001971	rs146445010	FSC-A on lymphoc	2	6597596
ebi-a-GCST90001971	rs3104369	FSC-A on lymphoc	6	32602482
ebi-a-GCST90001971	rs143298995	FSC-A on lymphoc	7	48232877
ebi-a-GCST90001971	rs144027086	FSC-A on lymphoc	7	15148913
ebi-a-GCST90001971	rs1842502	FSC-A on lymphoc	8	136208763
ebi-a-GCST90001971	rs2252167	FSC-A on lymphoc	10	10534818
ebi-a-GCST90001971	rs142476157	FSC-A on lymphoc	11	101727658
ebi-a-GCST90001971	rs77789294	FSC-A on lymphoc	14	29178686
ebi-a-GCST90001971	rs7213345	FSC-A on lymphoc	17	30211831
ebi-a-GCST90001971	rs1823592	FSC-A on lymphoc	18	22484814
ebi-a-GCST90001971	rs192157885	FSC-A on lymphoc	20	25983166
ebi-a-GCST90001971	rs12166583	FSC-A on lymphoc	22	30483964
ebi-a-GCST90001971	rs35851175	FSC-A on lymphoc	22	37462632
ebi-a-GCST90001972	rs190188611	FSC-A on T cell	1	177556743
ebi-a-GCST90001972	rs115752428	FSC-A on T cell	1	205560814
ebi-a-GCST90001972	rs75668319	FSC-A on T cell	2	60404215
ebi-a-GCST90001972	rs10191966	FSC-A on T cell	2	58932674
ebi-a-GCST90001972	rs76883038	FSC-A on T cell	2	115150773
ebi-a-GCST90001972	rs10169473	FSC-A on T cell	2	224307687
ebi-a-GCST90001972	rs35313751	FSC-A on T cell	2	129026457
ebi-a-GCST90001972	rs9825166	FSC-A on T cell	3	72227397
ebi-a-GCST90001972	rs7451962	FSC-A on T cell	6	32582435
ebi-a-GCST90001972	rs17071867	FSC-A on T cell	6	110886518
ebi-a-GCST90001972	rs143298995	FSC-A on T cell	7	48232877
ebi-a-GCST90001972	rs144027086	FSC-A on T cell	7	15148913
ebi-a-GCST90001972	rs1842502	FSC-A on T cell	8	136208763
ebi-a-GCST90001972	rs10786450	FSC-A on T cell	10	100399366
ebi-a-GCST90001972	rs1910504	FSC-A on T cell	11	11109296
ebi-a-GCST90001972	rs12434214	FSC-A on T cell	14	98114570
ebi-a-GCST90001972	rs77789294	FSC-A on T cell	14	29178686
ebi-a-GCST90001972	rs11652705	FSC-A on T cell	17	41761741
ebi-a-GCST90001972	rs1823592	FSC-A on T cell	18	22484814
ebi-a-GCST90001972	rs145826240	FSC-A on T cell	19	41158229
ebi-a-GCST90001972	rs192157885	FSC-A on T cell	20	25983166
ebi-a-GCST90001972	rs12166583	FSC-A on T cell	22	30483964
ebi-a-GCST90001973	rs190188611	FSC-A on CD4+	1	177556743
ebi-a-GCST90001973	rs76585217	FSC-A on CD4+	2	72027474
ebi-a-GCST90001973	rs62142825	FSC-A on CD4+	2	60508669
ebi-a-GCST90001973	rs10169473	FSC-A on CD4+	2	224307687
ebi-a-GCST90001973	rs114801141	FSC-A on CD4+	3	191847125
ebi-a-GCST90001973	rs11571882	FSC-A on CD4+	3	42298834
ebi-a-GCST90001973	rs114690517	FSC-A on CD4+	3	116324393
ebi-a-GCST90001973	rs13191523	FSC-A on CD4+	6	31009094
ebi-a-GCST90001973	rs9271673	FSC-A on CD4+	6	32592833
ebi-a-GCST90001973	rs6922309	FSC-A on CD4+	6	31321238
ebi-a-GCST90001973	rs7745305	FSC-A on CD4+	6	30913475
ebi-a-GCST90001973	rs143298995	FSC-A on CD4+	7	48232877
ebi-a-GCST90001973	rs144027086	FSC-A on CD4+	7	15148913

ebi-a-GCST90001973	rs1842502	FSC-A on CD4+	8	136208763
ebi-a-GCST90001973	rs41294719	FSC-A on CD4+	10	6085227
ebi-a-GCST90001973	rs117287835	FSC-A on CD4+	11	96273624
ebi-a-GCST90001973	rs12434214	FSC-A on CD4+	14	98114570
ebi-a-GCST90001973	rs6564958	FSC-A on CD4+	16	82051115
ebi-a-GCST90001973	rs11652705	FSC-A on CD4+	17	41761741
ebi-a-GCST90001973	rs112736938	FSC-A on CD4+	18	64359675
ebi-a-GCST90001973	rs34362479	FSC-A on CD4+	18	35106736
ebi-a-GCST90001973	rs192157885	FSC-A on CD4+	20	25983166
ebi-a-GCST90001973	rs62204954	FSC-A on CD4+	20	56276822
ebi-a-GCST90001973	rs2154499	FSC-A on CD4+	21	33538451
ebi-a-GCST90001973	rs12166583	FSC-A on CD4+	22	30483964
ebi-a-GCST90001974	rs78269918	FSC-A on CD8br	1	166494041
ebi-a-GCST90001974	rs1891421	FSC-A on CD8br	1	208036644
ebi-a-GCST90001974	rs7556141	FSC-A on CD8br	1	44947308
ebi-a-GCST90001974	rs2629824	FSC-A on CD8br	1	114687316
ebi-a-GCST90001974	rs10191966	FSC-A on CD8br	2	58932674
ebi-a-GCST90001974	rs10183338	FSC-A on CD8br	2	111610816
ebi-a-GCST90001974	rs115682796	FSC-A on CD8br	2	127997576
ebi-a-GCST90001974	rs10433403	FSC-A on CD8br	3	117827774
ebi-a-GCST90001974	rs144027086	FSC-A on CD8br	7	15148913
ebi-a-GCST90001974	rs143298995	FSC-A on CD8br	7	48232877
ebi-a-GCST90001974	rs343066	FSC-A on CD8br	7	35557420
ebi-a-GCST90001974	rs7852045	FSC-A on CD8br	9	91833343
ebi-a-GCST90001974	rs75425029	FSC-A on CD8br	9	95851910
ebi-a-GCST90001974	rs10786450	FSC-A on CD8br	10	100399366
ebi-a-GCST90001974	rs240534	FSC-A on CD8br	11	126214363
ebi-a-GCST90001974	rs77789294	FSC-A on CD8br	14	29178686
ebi-a-GCST90001974	rs7503544	FSC-A on CD8br	17	16876852
ebi-a-GCST90001974	rs855786	FSC-A on CD8br	22	37474713
ebi-a-GCST90001975	rs10183967	FSC-A on HLA DR	2	71797176
ebi-a-GCST90001975	rs720390	FSC-A on HLA DR	3	185548683
ebi-a-GCST90001975	rs3114972	FSC-A on HLA DR	3	175507453
ebi-a-GCST90001975	rs144027086	FSC-A on HLA DR	7	15148913
ebi-a-GCST90001975	rs144869562	FSC-A on HLA DR	7	33734811
ebi-a-GCST90001975	rs73715304	FSC-A on HLA DR	7	101194461
ebi-a-GCST90001975	rs80093546	FSC-A on HLA DR	7	148433716
ebi-a-GCST90001975	rs182033296	FSC-A on HLA DR	7	88712897
ebi-a-GCST90001975	rs66481406	FSC-A on HLA DR	7	48817681
ebi-a-GCST90001975	rs76260706	FSC-A on HLA DR	7	5250140
ebi-a-GCST90001975	rs942133	FSC-A on HLA DR	9	14376836
ebi-a-GCST90001975	rs3008396	FSC-A on HLA DR	10	134996013
ebi-a-GCST90001975	rs788291	FSC-A on HLA DR	10	43358147
ebi-a-GCST90001975	rs117916891	FSC-A on HLA DR	12	40564222
ebi-a-GCST90001975	rs7964408	FSC-A on HLA DR	12	95304634
ebi-a-GCST90001975	rs10142082	FSC-A on HLA DR	14	32375359
ebi-a-GCST90001975	rs568499003	FSC-A on HLA DR	17	2596832
ebi-a-GCST90001975	rs2467240	FSC-A on HLA DR	17	72491040

ebi-a-GCST90001975	rs797969	FSC-A on HLA DR	17	27648638
ebi-a-GCST90001975	rs62074942	FSC-A on HLA DR	18	3130089
ebi-a-GCST90001976	rs55723607	FSC-A on NKT	1	39487995
ebi-a-GCST90001976	rs200789118	FSC-A on NKT	1	62675673
ebi-a-GCST90001976	rs17861816	FSC-A on NKT	4	118297081
ebi-a-GCST90001976	rs4705195	FSC-A on NKT	5	147171592
ebi-a-GCST90001976	rs56310961	FSC-A on NKT	5	63593803
ebi-a-GCST90001976	rs140611829	FSC-A on NKT	6	14271982
ebi-a-GCST90001976	rs802242	FSC-A on NKT	6	124532234
ebi-a-GCST90001976	rs7458202	FSC-A on NKT	7	156136658
ebi-a-GCST90001976	rs6469957	FSC-A on NKT	8	121855078
ebi-a-GCST90001976	rs113047054	FSC-A on NKT	9	12210926
ebi-a-GCST90001976	rs10739941	FSC-A on NKT	9	95767562
ebi-a-GCST90001976	rs60499622	FSC-A on NKT	10	126551228
ebi-a-GCST90001976	rs7124198	FSC-A on NKT	11	5552904
ebi-a-GCST90001976	rs144044889	FSC-A on NKT	11	94415091
ebi-a-GCST90001976	rs146765347	FSC-A on NKT	11	11413255
ebi-a-GCST90001976	rs9560551	FSC-A on NKT	13	90739532
ebi-a-GCST90001976	rs2216310	FSC-A on NKT	14	77533344
ebi-a-GCST90001976	rs149563576	FSC-A on NKT	14	29143463
ebi-a-GCST90001976	rs12442698	FSC-A on NKT	15	93856756
ebi-a-GCST90001976	rs75087849	FSC-A on NKT	16	20171265
ebi-a-GCST90001976	rs8079387	FSC-A on NKT	17	43926777
ebi-a-GCST90001976	rs10425426	FSC-A on NKT	19	8782089
ebi-a-GCST90001976	rs111953444	FSC-A on NKT	21	36130326
ebi-a-GCST90001977	rs146688461	FSC-A on HLA DR	1	204545064
ebi-a-GCST90001977	rs150750018	FSC-A on HLA DR	1	242338190
ebi-a-GCST90001977	rs2379150	FSC-A on HLA DR	1	11494512
ebi-a-GCST90001977	rs4950037	FSC-A on HLA DR	1	97950934
ebi-a-GCST90001977	rs112728592	FSC-A on HLA DR	2	179019068
ebi-a-GCST90001977	rs7582130	FSC-A on HLA DR	2	71779032
ebi-a-GCST90001977	rs142882340	FSC-A on HLA DR	3	98779043
ebi-a-GCST90001977	rs113508249	FSC-A on HLA DR	4	82455099
ebi-a-GCST90001977	rs76260706	FSC-A on HLA DR	7	5250140
ebi-a-GCST90001977	rs57276252	FSC-A on HLA DR	7	104411433
ebi-a-GCST90001977	rs75929528	FSC-A on HLA DR	9	15258639
ebi-a-GCST90001977	rs142909976	FSC-A on HLA DR	9	31120417
ebi-a-GCST90001977	rs7939069	FSC-A on HLA DR	11	47688459
ebi-a-GCST90001977	rs2946832	FSC-A on HLA DR	12	102718009
ebi-a-GCST90001977	rs142667430	FSC-A on HLA DR	13	19454846
ebi-a-GCST90001977	rs147422381	FSC-A on HLA DR	15	38054883
ebi-a-GCST90001977	rs12482528	FSC-A on HLA DR	21	15710293
ebi-a-GCST90001978	rs142804909	FSC-A on HLA DR	1	64510605
ebi-a-GCST90001978	rs149994217	FSC-A on HLA DR	1	157817340
ebi-a-GCST90001978	rs6660527	FSC-A on HLA DR	1	194357571
ebi-a-GCST90001978	rs190188611	FSC-A on HLA DR	1	177556743
ebi-a-GCST90001978	rs116744406	FSC-A on HLA DR	2	117613346
ebi-a-GCST90001978	rs73175547	FSC-A on HLA DR	3	185327012

ebi-a-GCST90001978	rs116220030	FSC-A on HLA DR	4	68602634
ebi-a-GCST90001978	rs12500764	FSC-A on HLA DR	4	164276557
ebi-a-GCST90001978	rs12662469	FSC-A on HLA DR	6	107248127
ebi-a-GCST90001978	rs75917881	FSC-A on HLA DR	7	101528077
ebi-a-GCST90001978	rs2135750	FSC-A on HLA DR	8	59118408
ebi-a-GCST90001978	rs79034032	FSC-A on HLA DR	10	125629883
ebi-a-GCST90001978	rs11232360	FSC-A on HLA DR	11	80547943
ebi-a-GCST90001978	rs1398442	FSC-A on HLA DR	12	85809907
ebi-a-GCST90001978	rs35237896	FSC-A on HLA DR	13	75021456
ebi-a-GCST90001978	rs62058363	FSC-A on HLA DR	17	13012605
ebi-a-GCST90001978	rs62074941	FSC-A on HLA DR	18	3123416
ebi-a-GCST90001978	rs6513961	FSC-A on HLA DR	20	35824589
ebi-a-GCST90001979	rs12239946	CD16 on CD14- CI	1	160319055
ebi-a-GCST90001979	rs61806480	CD16 on CD14- CI	1	175775602
ebi-a-GCST90001979	rs12737621	CD16 on CD14- CI	1	18698947
ebi-a-GCST90001979	rs74341264	CD16 on CD14- CI	1	161561151
ebi-a-GCST90001979	rs112655418	CD16 on CD14- CI	1	56261771
ebi-a-GCST90001979	rs17400517	CD16 on CD14- CI	1	161485259
ebi-a-GCST90001979	rs4644525	CD16 on CD14- CI	1	61954826
ebi-a-GCST90001979	rs140966292	CD16 on CD14- CI	1	175408164
ebi-a-GCST90001979	rs6671271	CD16 on CD14- CI	1	162250009
ebi-a-GCST90001979	rs3856266	CD16 on CD14- CI	1	157973889
ebi-a-GCST90001979	rs72700199	CD16 on CD14- CI	1	162324646
ebi-a-GCST90001979	rs2502806	CD16 on CD14- CI	1	161135559
ebi-a-GCST90001979	rs78804199	CD16 on CD14- CI	2	37732880
ebi-a-GCST90001979	rs35804175	CD16 on CD14- CI	3	82492118
ebi-a-GCST90001979	rs2638546	CD16 on CD14- CI	6	118994715
ebi-a-GCST90001979	rs2184197	CD16 on CD14- CI	6	170194467
ebi-a-GCST90001979	rs78639067	CD16 on CD14- CI	8	41217478
ebi-a-GCST90001979	rs17817999	CD16 on CD14- CI	8	25869608
ebi-a-GCST90001979	rs139731096	CD16 on CD14- CI	9	140753656
ebi-a-GCST90001979	rs11860582	CD16 on CD14- CI	16	62812389
ebi-a-GCST90001979	rs79433597	CD16 on CD14- CI	19	35015143
ebi-a-GCST90001979	rs139085673	CD16 on CD14- CI	21	37863897
ebi-a-GCST90001979	rs73152567	CD16 on CD14- CI	22	25105502
ebi-a-GCST90001980	rs77869433	CD40 on CD14+ C	1	23199772
ebi-a-GCST90001980	rs3813611	CD40 on CD14+ C	1	161068240
ebi-a-GCST90001980	rs1801274	CD40 on CD14+ C	1	161479745
ebi-a-GCST90001980	rs149990497	CD40 on CD14+ C	1	163990105
ebi-a-GCST90001980	rs6713465	CD40 on CD14+ C	2	231251562
ebi-a-GCST90001980	rs116203109	CD40 on CD14+ C	2	5943436
ebi-a-GCST90001980	rs4894541	CD40 on CD14+ C	3	172015532
ebi-a-GCST90001980	rs188101908	CD40 on CD14+ C	6	87886806
ebi-a-GCST90001980	rs117608122	CD40 on CD14+ C	7	152197572
ebi-a-GCST90001980	rs78012242	CD40 on CD14+ C	9	94713706
ebi-a-GCST90001980	rs60629714	CD40 on CD14+ C	9	95889160
ebi-a-GCST90001980	rs3780804	CD40 on CD14+ C	9	136598128
ebi-a-GCST90001980	rs10887024	CD40 on CD14+ C	10	123688152

ebi-a-GCST90001980	rs76949305	CD40 on CD14+ C]	10	24729568
ebi-a-GCST90001980	rs118170720	CD40 on CD14+ C]	11	26568364
ebi-a-GCST90001980	rs112876754	CD40 on CD14+ C]	12	54934521
ebi-a-GCST90001980	rs144545136	CD40 on CD14+ C]	17	31062466
ebi-a-GCST90001980	rs9954245	CD40 on CD14+ C]	18	73881725
ebi-a-GCST90001980	rs111929038	CD40 on CD14+ C]	19	2660053
ebi-a-GCST90001980	rs34658867	CD40 on CD14+ C]	20	44977184
ebi-a-GCST90001980	rs57041960	CD40 on CD14+ C]	20	44074651
ebi-a-GCST90001980	rs75309031	CD40 on CD14+ C]	20	44305107
ebi-a-GCST90001980	rs745307	CD40 on CD14+ C]	20	44747086
ebi-a-GCST90001980	rs117295081	CD40 on CD14+ C]	20	45333273
ebi-a-GCST90001981	rs1801274	CD40 on CD14+ C]	1	161479745
ebi-a-GCST90001981	rs72927495	CD40 on CD14+ C]	1	17880459
ebi-a-GCST90001981	rs6687275	CD40 on CD14+ C]	1	161515638
ebi-a-GCST90001981	rs113063605	CD40 on CD14+ C]	1	161246613
ebi-a-GCST90001981	rs77649171	CD40 on CD14+ C]	3	171098461
ebi-a-GCST90001981	rs60072425	CD40 on CD14+ C]	4	77781605
ebi-a-GCST90001981	rs12517933	CD40 on CD14+ C]	5	173665451
ebi-a-GCST90001981	rs138540869	CD40 on CD14+ C]	5	62602530
ebi-a-GCST90001981	rs141130258	CD40 on CD14+ C]	7	101436472
ebi-a-GCST90001981	rs149632680	CD40 on CD14+ C]	8	24034923
ebi-a-GCST90001981	rs11992049	CD40 on CD14+ C]	8	78906008
ebi-a-GCST90001981	rs186177055	CD40 on CD14+ C]	15	81421309
ebi-a-GCST90001981	rs139839882	CD40 on CD14+ C]	15	30291728
ebi-a-GCST90001981	rs72749639	CD40 on CD14+ C]	15	70326266
ebi-a-GCST90001981	rs12443555	CD40 on CD14+ C]	16	81615369
ebi-a-GCST90001981	rs117572234	CD40 on CD14+ C]	16	59420596
ebi-a-GCST90001981	rs745307	CD40 on CD14+ C]	20	44747086
ebi-a-GCST90001981	rs148922794	CD40 on CD14+ C]	20	44930372
ebi-a-GCST90001981	rs11906777	CD40 on CD14+ C]	20	39374731
ebi-a-GCST90001981	rs2664523	CD40 on CD14+ C]	20	45318042
ebi-a-GCST90001981	rs4810481	CD40 on CD14+ C]	20	44571079
ebi-a-GCST90001982	rs75340063	CCR2 on CD14- CI	1	162034572
ebi-a-GCST90001982	rs144126567	CCR2 on CD14- CI	1	161510516
ebi-a-GCST90001982	rs71645288	CCR2 on CD14- CI	1	176456030
ebi-a-GCST90001982	rs78823993	CCR2 on CD14- CI	1	38868362
ebi-a-GCST90001982	rs1752020	CCR2 on CD14- CI	1	15572133
ebi-a-GCST90001982	rs76650090	CCR2 on CD14- CI	1	237415220
ebi-a-GCST90001982	rs62167242	CCR2 on CD14- CI	2	146135036
ebi-a-GCST90001982	rs12614991	CCR2 on CD14- CI	2	45342215
ebi-a-GCST90001982	rs7623367	CCR2 on CD14- CI	3	192230559
ebi-a-GCST90001982	rs6768910	CCR2 on CD14- CI	3	74574226
ebi-a-GCST90001982	rs142752828	CCR2 on CD14- CI	4	14542063
ebi-a-GCST90001982	rs148775569	CCR2 on CD14- CI	4	9727374
ebi-a-GCST90001982	rs116231488	CCR2 on CD14- CI	5	101620120
ebi-a-GCST90001982	rs2053045	CCR2 on CD14- CI	5	145612264
ebi-a-GCST90001982	rs12193797	CCR2 on CD14- CI	6	41748352
ebi-a-GCST90001982	rs59470545	CCR2 on CD14- CI	6	5974506

ebi-a-GCST90001982	rs2296509	CCR2 on CD14- CI	6	158489778
ebi-a-GCST90001982	rs112619771	CCR2 on CD14- CI	7	143427146
ebi-a-GCST90001982	rs148096757	CCR2 on CD14- CI	8	11715710
ebi-a-GCST90001982	rs117062237	CCR2 on CD14- CI	11	20572262
ebi-a-GCST90001982	rs1800973	CCR2 on CD14- CI	12	69744014
ebi-a-GCST90001982	rs139922750	CCR2 on CD14- CI	12	64437951
ebi-a-GCST90001982	rs55808110	CCR2 on CD14- CI	13	90652564
ebi-a-GCST90001982	rs187783209	CCR2 on CD14- CI	13	70642363
ebi-a-GCST90001982	rs149322792	CCR2 on CD14- CI	14	95498399
ebi-a-GCST90001982	rs1545208	CCR2 on CD14- CI	16	60271139
ebi-a-GCST90001982	rs72792415	CCR2 on CD14- CI	16	58686602
ebi-a-GCST90001982	rs143246579	CCR2 on CD14- CI	18	64589063
ebi-a-GCST90001983	rs61759062	CD14 on CD14+ C	1	30558818
ebi-a-GCST90001983	rs6671847	CD14 on CD14+ C	1	161478810
ebi-a-GCST90001983	rs10071982	CD14 on CD14+ C	5	62703251
ebi-a-GCST90001983	rs71586370	CD14 on CD14+ C	5	102625786
ebi-a-GCST90001983	rs26894	CD14 on CD14+ C	5	80417663
ebi-a-GCST90001983	rs2569177	CD14 on CD14+ C	5	139992162
ebi-a-GCST90001983	rs190803173	CD14 on CD14+ C	6	378290
ebi-a-GCST90001983	rs13248932	CD14 on CD14+ C	8	58311929
ebi-a-GCST90001983	rs9406865	CD14 on CD14+ C	9	25219707
ebi-a-GCST90001983	rs12262055	CD14 on CD14+ C	10	7633337
ebi-a-GCST90001983	rs4291738	CD14 on CD14+ C	12	40611618
ebi-a-GCST90001983	rs61922057	CD14 on CD14+ C	12	12505767
ebi-a-GCST90001983	rs17111403	CD14 on CD14+ C	14	41540685
ebi-a-GCST90001983	rs12593041	CD14 on CD14+ C	15	52443107
ebi-a-GCST90001983	rs62037895	CD14 on CD14+ C	15	31653646
ebi-a-GCST90001983	rs117306778	CD14 on CD14+ C	16	77769277
ebi-a-GCST90001983	rs13043397	CD14 on CD14+ C	20	42848689
ebi-a-GCST90001984	rs2297606	HLA DR on CD14-	1	154321084
ebi-a-GCST90001984	rs139411978	HLA DR on CD14-	2	16339295
ebi-a-GCST90001984	rs6808893	HLA DR on CD14-	3	188133439
ebi-a-GCST90001984	rs13076004	HLA DR on CD14-	3	34698655
ebi-a-GCST90001984	rs577546	HLA DR on CD14-	5	6557042
ebi-a-GCST90001984	rs142856632	HLA DR on CD14-	5	150300374
ebi-a-GCST90001984	rs6917212	HLA DR on CD14-	6	32583005
ebi-a-GCST90001984	rs61742525	HLA DR on CD14-	6	33072522
ebi-a-GCST90001984	rs9269109	HLA DR on CD14-	6	32443266
ebi-a-GCST90001984	rs7748976	HLA DR on CD14-	6	33890451
ebi-a-GCST90001984	rs116671810	HLA DR on CD14-	6	31450274
ebi-a-GCST90001984	rs412492	HLA DR on CD14-	6	32213831
ebi-a-GCST90001984	rs118138479	HLA DR on CD14-	10	122312157
ebi-a-GCST90001984	rs74970695	HLA DR on CD14-	12	27651592
ebi-a-GCST90001984	rs7297160	HLA DR on CD14-	12	92681286
ebi-a-GCST90001984	rs9652602	HLA DR on CD14-	16	11338086
ebi-a-GCST90001984	rs8057874	HLA DR on CD14-	16	10706593
ebi-a-GCST90001984	rs73499473	HLA DR on CD14-	16	10978885
ebi-a-GCST90001984	rs56353032	HLA DR on CD14-	20	17369072

ebi-a-GCST90001985	rs77869433	CD40 on monocyte:	1	23199772
ebi-a-GCST90001985	rs1801274	CD40 on monocyte:	1	161479745
ebi-a-GCST90001985	rs2070901	CD40 on monocyte:	1	161185058
ebi-a-GCST90001985	rs113063605	CD40 on monocyte:	1	161246613
ebi-a-GCST90001985	rs78882596	CD40 on monocyte:	3	111702977
ebi-a-GCST90001985	rs140379607	CD40 on monocyte:	3	4758271
ebi-a-GCST90001985	rs6816965	CD40 on monocyte:	4	180975530
ebi-a-GCST90001985	rs55662279	CD40 on monocyte:	5	175204768
ebi-a-GCST90001985	rs80264041	CD40 on monocyte:	7	49695025
ebi-a-GCST90001985	rs117608122	CD40 on monocyte:	7	152197572
ebi-a-GCST90001985	rs113231250	CD40 on monocyte:	8	107146016
ebi-a-GCST90001985	rs60629714	CD40 on monocyte:	9	95889160
ebi-a-GCST90001985	rs10887024	CD40 on monocyte:	10	123688152
ebi-a-GCST90001985	rs61548896	CD40 on monocyte:	10	75300277
ebi-a-GCST90001985	rs118170720	CD40 on monocyte:	11	26568364
ebi-a-GCST90001985	rs12225010	CD40 on monocyte:	11	75378674
ebi-a-GCST90001985	rs112876754	CD40 on monocyte:	12	54934521
ebi-a-GCST90001985	rs76003695	CD40 on monocyte:	15	82406919
ebi-a-GCST90001985	rs117799048	CD40 on monocyte:	16	80007142
ebi-a-GCST90001985	rs144545136	CD40 on monocyte:	17	31062466
ebi-a-GCST90001985	rs9954245	CD40 on monocyte:	18	73881725
ebi-a-GCST90001985	rs113466200	CD40 on monocyte:	19	54250532
ebi-a-GCST90001985	rs111929038	CD40 on monocyte:	19	2660053
ebi-a-GCST90001985	rs117295081	CD40 on monocyte:	20	45333273
ebi-a-GCST90001985	rs57041960	CD40 on monocyte:	20	44074651
ebi-a-GCST90001985	rs34658867	CD40 on monocyte:	20	44977184
ebi-a-GCST90001985	rs745307	CD40 on monocyte:	20	44747086
ebi-a-GCST90001985	rs144062689	CD40 on monocyte:	22	25308118
ebi-a-GCST90001985	rs41298798	CD40 on monocyte:	22	19749508
ebi-a-GCST90001986	rs4660041	CD14 on CD14+ C	1	241344611
ebi-a-GCST90001986	rs2040433	CD14 on CD14+ C	1	7448154
ebi-a-GCST90001986	rs567921813	CD14 on CD14+ C	1	1095620
ebi-a-GCST90001986	rs6713968	CD14 on CD14+ C	2	78616884
ebi-a-GCST90001986	rs58027502	CD14 on CD14+ C	5	139978241
ebi-a-GCST90001986	rs26894	CD14 on CD14+ C	5	80417663
ebi-a-GCST90001986	rs4128876	CD14 on CD14+ C	6	156303873
ebi-a-GCST90001986	rs9481732	CD14 on CD14+ C	6	118124789
ebi-a-GCST90001986	rs12663127	CD14 on CD14+ C	6	139560140
ebi-a-GCST90001986	rs77624779	CD14 on CD14+ C	9	122726431
ebi-a-GCST90001986	rs117925981	CD14 on CD14+ C	10	112247155
ebi-a-GCST90001986	rs1041345	CD14 on CD14+ C	12	41197785
ebi-a-GCST90001986	rs139408196	CD14 on CD14+ C	12	40575173
ebi-a-GCST90001986	rs111820782	CD14 on CD14+ C	14	28914556
ebi-a-GCST90001986	rs79186565	CD14 on CD14+ C	14	23677385
ebi-a-GCST90001986	rs76903175	CD14 on CD14+ C	18	73892447
ebi-a-GCST90001987	rs144823175	CD64 on CD14+ C	1	149858520
ebi-a-GCST90001987	rs76405727	CD64 on CD14+ C	1	119909416
ebi-a-GCST90001987	rs1801274	CD64 on CD14+ C	1	161479745

ebi-a-GCST90001987	rs116275309	CD64 on CD14+ C	1	150863650
ebi-a-GCST90001987	rs116004327	CD64 on CD14+ C	1	117459175
ebi-a-GCST90001987	rs150145799	CD64 on CD14+ C	1	149738590
ebi-a-GCST90001987	rs12135207	CD64 on CD14+ C	1	160944691
ebi-a-GCST90001987	rs112755578	CD64 on CD14+ C	1	149716369
ebi-a-GCST90001987	rs188268415	CD64 on CD14+ C	1	150401679
ebi-a-GCST90001987	rs587759315	CD64 on CD14+ C	1	121480104
ebi-a-GCST90001987	rs192899694	CD64 on CD14+ C	1	146480265
ebi-a-GCST90001987	rs766041	CD64 on CD14+ C	1	165432190
ebi-a-GCST90001987	rs72475593	CD64 on CD14+ C	1	146529499
ebi-a-GCST90001987	rs145828126	CD64 on CD14+ C	1	120233380
ebi-a-GCST90001987	rs610249	CD64 on CD14+ C	1	109692641
ebi-a-GCST90001987	rs72705789	CD64 on CD14+ C	1	162501593
ebi-a-GCST90001987	rs139244126	CD64 on CD14+ C	2	69117194
ebi-a-GCST90001987	rs6780007	CD64 on CD14+ C	3	55074425
ebi-a-GCST90001987	rs1560982	CD64 on CD14+ C	4	184826026
ebi-a-GCST90001987	rs11934532	CD64 on CD14+ C	4	182164089
ebi-a-GCST90001987	rs140329252	CD64 on CD14+ C	4	131026425
ebi-a-GCST90001987	rs190928917	CD64 on CD14+ C	6	157071062
ebi-a-GCST90001987	rs79003328	CD64 on CD14+ C	6	71947935
ebi-a-GCST90001987	rs142811849	CD64 on CD14+ C	6	45627057
ebi-a-GCST90001987	rs192225504	CD64 on CD14+ C	7	1597362
ebi-a-GCST90001987	rs80178684	CD64 on CD14+ C	8	59726631
ebi-a-GCST90001987	rs1919458	CD64 on CD14+ C	10	13876322
ebi-a-GCST90001987	rs6480509	CD64 on CD14+ C	10	73071462
ebi-a-GCST90001987	rs627319	CD64 on CD14+ C	11	62247621
ebi-a-GCST90001987	rs1146114	CD64 on CD14+ C	12	63713121
ebi-a-GCST90001987	rs58902422	CD64 on CD14+ C	14	39342923
ebi-a-GCST90001987	rs1320440	CD64 on CD14+ C	14	101333700
ebi-a-GCST90001987	rs72774507	CD64 on CD14+ C	16	6170178
ebi-a-GCST90001987	rs16960068	CD64 on CD14+ C	17	64692068
ebi-a-GCST90001987	rs406130	CD64 on CD14+ C	19	11940761
ebi-a-GCST90001988	rs150649461	HLA DR on CD14+	1	92925654
ebi-a-GCST90001988	rs148974843	HLA DR on CD14+	1	237971715
ebi-a-GCST90001988	rs146897285	HLA DR on CD14+	1	213984208
ebi-a-GCST90001988	rs112651313	HLA DR on CD14+	2	212886345
ebi-a-GCST90001988	rs113337372	HLA DR on CD14+	2	121677690
ebi-a-GCST90001988	rs113555953	HLA DR on CD14+	4	97898706
ebi-a-GCST90001988	rs13168479	HLA DR on CD14+	5	164249532
ebi-a-GCST90001988	rs146479897	HLA DR on CD14+	5	65595525
ebi-a-GCST90001988	rs2394447	HLA DR on CD14+	6	30826835
ebi-a-GCST90001988	rs9469245	HLA DR on CD14+	6	32692084
ebi-a-GCST90001988	rs9271768	HLA DR on CD14+	6	32594188
ebi-a-GCST90001988	rs80032720	HLA DR on CD14+	6	32384933
ebi-a-GCST90001988	rs9270585	HLA DR on CD14+	6	32561300
ebi-a-GCST90001988	rs150542843	HLA DR on CD14+	6	33677931
ebi-a-GCST90001988	rs9261756	HLA DR on CD14+	6	30372414
ebi-a-GCST90001988	rs184592417	HLA DR on CD14+	8	140310541

ebi-a-GCST90001988	rs11788133	HLA DR on CD14+	9	137907399
ebi-a-GCST90001988	rs35848415	HLA DR on CD14+	11	102879843
ebi-a-GCST90001988	rs143858253	HLA DR on CD14+	12	54497116
ebi-a-GCST90001988	rs1800973	HLA DR on CD14+	12	69744014
ebi-a-GCST90001988	rs62005962	HLA DR on CD14+	15	54040290
ebi-a-GCST90001988	rs8043889	HLA DR on CD14+	16	27733429
ebi-a-GCST90001988	rs188394267	HLA DR on CD14+	19	12466408
ebi-a-GCST90001989	rs148765873	CD40 on CD14- CI	1	161221713
ebi-a-GCST90001989	rs6687275	CD40 on CD14- CI	1	161515638
ebi-a-GCST90001989	rs12122366	CD40 on CD14- CI	1	162563661
ebi-a-GCST90001989	rs139376787	CD40 on CD14- CI	1	161847528
ebi-a-GCST90001989	rs61784602	CD40 on CD14- CI	1	46796909
ebi-a-GCST90001989	rs1801274	CD40 on CD14- CI	1	161479745
ebi-a-GCST90001989	rs12233381	CD40 on CD14- CI	3	194232621
ebi-a-GCST90001989	rs77649171	CD40 on CD14- CI	3	171098461
ebi-a-GCST90001989	rs55872505	CD40 on CD14- CI	3	87076367
ebi-a-GCST90001989	rs75698615	CD40 on CD14- CI	3	186653458
ebi-a-GCST90001989	rs60072425	CD40 on CD14- CI	4	77781605
ebi-a-GCST90001989	rs56330830	CD40 on CD14- CI	4	19891513
ebi-a-GCST90001989	rs146963227	CD40 on CD14- CI	4	17530633
ebi-a-GCST90001989	rs78824072	CD40 on CD14- CI	5	96185002
ebi-a-GCST90001989	rs77774351	CD40 on CD14- CI	6	22713558
ebi-a-GCST90001989	rs35989582	CD40 on CD14- CI	6	170460783
ebi-a-GCST90001989	rs10762188	CD40 on CD14- CI	10	69876930
ebi-a-GCST90001989	rs11032433	CD40 on CD14- CI	11	33895527
ebi-a-GCST90001989	rs139922750	CD40 on CD14- CI	12	64437951
ebi-a-GCST90001989	rs4766578	CD40 on CD14- CI	12	111904371
ebi-a-GCST90001989	rs78162364	CD40 on CD14- CI	14	98109448
ebi-a-GCST90001989	rs140839053	CD40 on CD14- CI	16	21541396
ebi-a-GCST90001989	rs745307	CD40 on CD14- CI	20	44747086
ebi-a-GCST90001989	rs34658867	CD40 on CD14- CI	20	44977184
ebi-a-GCST90001989	rs143602490	CD40 on CD14- CI	20	44010127
ebi-a-GCST90001989	rs6073077	CD40 on CD14- CI	20	42024411
ebi-a-GCST90001989	rs6032211	CD40 on CD14- CI	20	44092301
ebi-a-GCST90001989	rs111436326	CD40 on CD14- CI	20	42701487
ebi-a-GCST90001989	rs3788395	CD40 on CD14- CI	22	26738732
ebi-a-GCST90001990	rs34710782	CD64 on CD14- CI	1	11903241
ebi-a-GCST90001990	rs150375532	CD64 on CD14- CI	1	38146353
ebi-a-GCST90001990	rs115383270	CD64 on CD14- CI	1	161531340
ebi-a-GCST90001990	rs150145799	CD64 on CD14- CI	1	149738590
ebi-a-GCST90001990	rs2197098	CD64 on CD14- CI	2	154685822
ebi-a-GCST90001990	rs4681723	CD64 on CD14- CI	3	57213255
ebi-a-GCST90001990	rs146963227	CD64 on CD14- CI	4	17530633
ebi-a-GCST90001990	rs10036919	CD64 on CD14- CI	5	160219464
ebi-a-GCST90001990	rs13161932	CD64 on CD14- CI	5	42074341
ebi-a-GCST90001990	rs111279951	CD64 on CD14- CI	5	18068020
ebi-a-GCST90001990	rs117283705	CD64 on CD14- CI	6	102881264
ebi-a-GCST90001990	rs9467953	CD64 on CD14- CI	6	26965804

ebi-a-GCST90001990	rs11772145	CD64 on CD14- CI	7	67584032
ebi-a-GCST90001990	rs35944461	CD64 on CD14- CI	8	990572
ebi-a-GCST90001990	rs35309480	CD64 on CD14- CI	8	97002090
ebi-a-GCST90001990	rs947853	CD64 on CD14- CI	11	70567194
ebi-a-GCST90001990	rs11065979	CD64 on CD14- CI	12	112059557
ebi-a-GCST90001990	rs74970695	CD64 on CD14- CI	12	27651592
ebi-a-GCST90001990	rs1932805	CD64 on CD14- CI	13	82498699
ebi-a-GCST90001990	rs1333255	CD64 on CD14- CI	13	94844743
ebi-a-GCST90001990	rs1138358	CD64 on CD14- CI	15	80263345
ebi-a-GCST90001990	rs117446496	CD64 on CD14- CI	17	78773924
ebi-a-GCST90001991	rs146897285	HLA DR on CD14+	1	213984208
ebi-a-GCST90001991	rs148974843	HLA DR on CD14+	1	237971715
ebi-a-GCST90001991	rs113337372	HLA DR on CD14+	2	121677690
ebi-a-GCST90001991	rs112651313	HLA DR on CD14+	2	212886345
ebi-a-GCST90001991	rs113555953	HLA DR on CD14+	4	97898706
ebi-a-GCST90001991	rs11737889	HLA DR on CD14+	4	64395080
ebi-a-GCST90001991	rs146479897	HLA DR on CD14+	5	65595525
ebi-a-GCST90001991	rs13168479	HLA DR on CD14+	5	164249532
ebi-a-GCST90001991	rs9261756	HLA DR on CD14+	6	30372414
ebi-a-GCST90001991	rs9270585	HLA DR on CD14+	6	32561300
ebi-a-GCST90001991	rs9271768	HLA DR on CD14+	6	32594188
ebi-a-GCST90001991	rs80032720	HLA DR on CD14+	6	32384933
ebi-a-GCST90001991	rs9469245	HLA DR on CD14+	6	32692084
ebi-a-GCST90001991	rs2394447	HLA DR on CD14+	6	30826835
ebi-a-GCST90001991	rs184592417	HLA DR on CD14+	8	140310541
ebi-a-GCST90001991	rs147628007	HLA DR on CD14+	9	81009018
ebi-a-GCST90001991	rs11788133	HLA DR on CD14+	9	137907399
ebi-a-GCST90001991	rs35848415	HLA DR on CD14+	11	102879843
ebi-a-GCST90001991	rs143858253	HLA DR on CD14+	12	54497116
ebi-a-GCST90001991	rs1800973	HLA DR on CD14+	12	69744014
ebi-a-GCST90001991	rs62005962	HLA DR on CD14+	15	54040290
ebi-a-GCST90001991	rs8043889	HLA DR on CD14+	16	27733429
ebi-a-GCST90001991	rs188394267	HLA DR on CD14+	19	12466408
ebi-a-GCST90001992	rs115383270	CCR2 on CD14+ C	1	161531340
ebi-a-GCST90001992	rs74890470	CCR2 on CD14+ C	1	236911296
ebi-a-GCST90001992	rs144845257	CCR2 on CD14+ C	2	227026744
ebi-a-GCST90001992	rs114766366	CCR2 on CD14+ C	2	1326700
ebi-a-GCST90001992	rs139144024	CCR2 on CD14+ C	2	88635490
ebi-a-GCST90001992	rs2036234	CCR2 on CD14+ C	3	1261465
ebi-a-GCST90001992	rs2361832	CCR2 on CD14+ C	3	190260037
ebi-a-GCST90001992	rs9850642	CCR2 on CD14+ C	3	46200823
ebi-a-GCST90001992	rs77927026	CCR2 on CD14+ C	4	159974748
ebi-a-GCST90001992	rs34162749	CCR2 on CD14+ C	5	171532755
ebi-a-GCST90001992	rs75117507	CCR2 on CD14+ C	5	102937489
ebi-a-GCST90001992	rs9312875	CCR2 on CD14+ C	5	14909219
ebi-a-GCST90001992	rs116231488	CCR2 on CD14+ C	5	101620120
ebi-a-GCST90001992	rs9397486	CCR2 on CD14+ C	6	152449900
ebi-a-GCST90001992	rs2745595	CCR2 on CD14+ C	6	1604845

ebi-a-GCST90001992	rs11753009	CCR2 on CD14+ C	6	123999692
ebi-a-GCST90001992	rs78093069	CCR2 on CD14+ C	7	147981012
ebi-a-GCST90001992	rs146496206	CCR2 on CD14+ C	7	116000806
ebi-a-GCST90001992	rs117427319	CCR2 on CD14+ C	7	69208295
ebi-a-GCST90001992	rs117340589	CCR2 on CD14+ C	8	138634227
ebi-a-GCST90001992	rs76641435	CCR2 on CD14+ C	9	1393540
ebi-a-GCST90001992	rs11592564	CCR2 on CD14+ C	10	115126137
ebi-a-GCST90001992	rs76082466	CCR2 on CD14+ C	11	43196874
ebi-a-GCST90001992	rs1019348	CCR2 on CD14+ C	12	127402727
ebi-a-GCST90001992	rs2046587	CCR2 on CD14+ C	12	32360576
ebi-a-GCST90001992	rs75270021	CCR2 on CD14+ C	13	70725465
ebi-a-GCST90001992	rs72653608	CCR2 on CD14+ C	13	103344593
ebi-a-GCST90001992	rs17351300	CCR2 on CD14+ C	15	82273904
ebi-a-GCST90001992	rs62042644	CCR2 on CD14+ C	16	58478227
ebi-a-GCST90001992	rs77640671	CCR2 on CD14+ C	16	58607804
ebi-a-GCST90001992	rs139288206	CCR2 on CD14+ C	17	14094524
ebi-a-GCST90001992	rs34205394	CCR2 on CD14+ C	17	55095409
ebi-a-GCST90001992	rs4547380	CCR2 on CD14+ C	17	32670982
ebi-a-GCST90001992	rs111661013	CCR2 on CD14+ C	18	25869222
ebi-a-GCST90001992	rs72951070	CCR2 on CD14+ C	18	30847362
ebi-a-GCST90001992	rs140019375	CCR2 on CD14+ C	19	54863920
ebi-a-GCST90001992	rs34690145	CCR2 on CD14+ C	19	3759702
ebi-a-GCST90001993	rs62126479	PDL-1 on CD14+ C	2	19062526
ebi-a-GCST90001993	rs143930325	PDL-1 on CD14+ C	4	146544530
ebi-a-GCST90001993	rs778582	PDL-1 on CD14+ C	5	140004816
ebi-a-GCST90001993	rs148958034	PDL-1 on CD14+ C	6	140202191
ebi-a-GCST90001993	rs10281189	PDL-1 on CD14+ C	7	5422545
ebi-a-GCST90001993	rs147810622	PDL-1 on CD14+ C	7	151910695
ebi-a-GCST90001993	rs10276990	PDL-1 on CD14+ C	7	16903052
ebi-a-GCST90001993	rs140886794	PDL-1 on CD14+ C	10	44984983
ebi-a-GCST90001993	rs72779216	PDL-1 on CD14+ C	16	1400283
ebi-a-GCST90001993	rs740026	PDL-1 on CD14+ C	17	63561681
ebi-a-GCST90001993	rs12953239	PDL-1 on CD14+ C	17	35199795
ebi-a-GCST90001993	rs8089422	PDL-1 on CD14+ C	18	54525353
ebi-a-GCST90001993	rs149793797	PDL-1 on CD14+ C	19	1781867
ebi-a-GCST90001993	rs112555198	PDL-1 on CD14+ C	19	2147301
ebi-a-GCST90001993	rs144941477	PDL-1 on CD14+ C	19	47853865
ebi-a-GCST90001993	rs150687633	PDL-1 on CD14+ C	20	49270830
ebi-a-GCST90001994	rs6661135	CX3CR1 on CD14-	1	150999414
ebi-a-GCST90001994	rs139062294	CX3CR1 on CD14-	1	35600792
ebi-a-GCST90001994	rs144126567	CX3CR1 on CD14-	1	161510516
ebi-a-GCST90001994	rs61801809	CX3CR1 on CD14-	1	161557512
ebi-a-GCST90001994	rs4648363	CX3CR1 on CD14-	1	3052501
ebi-a-GCST90001994	rs12614991	CX3CR1 on CD14-	2	45342215
ebi-a-GCST90001994	rs35579164	CX3CR1 on CD14-	2	20818883
ebi-a-GCST90001994	rs115232009	CX3CR1 on CD14-	2	181113420
ebi-a-GCST90001994	rs146027392	CX3CR1 on CD14-	3	143772423
ebi-a-GCST90001994	rs9823718	CX3CR1 on CD14-	3	39293757

ebi-a-GCST90001994	rs6813694	CX3CR1 on CD14-	4	187112141
ebi-a-GCST90001994	rs34331363	CX3CR1 on CD14-	6	32568439
ebi-a-GCST90001994	rs62435155	CX3CR1 on CD14-	6	164012301
ebi-a-GCST90001994	rs117377596	CX3CR1 on CD14-	6	69443667
ebi-a-GCST90001994	rs876036	CX3CR1 on CD14-	7	50307710
ebi-a-GCST90001994	rs71521373	CX3CR1 on CD14-	8	30021461
ebi-a-GCST90001994	rs61847551	CX3CR1 on CD14-	10	53766414
ebi-a-GCST90001994	rs17672079	CX3CR1 on CD14-	16	10331360
ebi-a-GCST90001994	rs3826742	CX3CR1 on CD14-	19	7913752
ebi-a-GCST90001994	rs147571616	CX3CR1 on CD14-	19	36362737
ebi-a-GCST90001994	rs117379843	CX3CR1 on CD14-	19	56718736
ebi-a-GCST90001995	rs1801274	CX3CR1 on monoc	1	161479745
ebi-a-GCST90001995	rs6666038	CX3CR1 on monoc	1	162640142
ebi-a-GCST90001995	rs4622126	CX3CR1 on monoc	1	228017395
ebi-a-GCST90001995	rs6726288	CX3CR1 on monoc	2	158585869
ebi-a-GCST90001995	rs17556553	CX3CR1 on monoc	2	170821289
ebi-a-GCST90001995	rs77705296	CX3CR1 on monoc	2	158703713
ebi-a-GCST90001995	rs73056479	CX3CR1 on monoc	3	38700237
ebi-a-GCST90001995	rs9823718	CX3CR1 on monoc	3	39293757
ebi-a-GCST90001995	rs72865957	CX3CR1 on monoc	3	39355919
ebi-a-GCST90001995	rs9879669	CX3CR1 on monoc	3	7682013
ebi-a-GCST90001995	rs17736094	CX3CR1 on monoc	5	5711054
ebi-a-GCST90001995	rs17521541	CX3CR1 on monoc	5	24624788
ebi-a-GCST90001995	rs115689956	CX3CR1 on monoc	7	78974417
ebi-a-GCST90001995	rs10951824	CX3CR1 on monoc	7	45618083
ebi-a-GCST90001995	rs4548126	CX3CR1 on monoc	7	167799
ebi-a-GCST90001995	rs28428470	CX3CR1 on monoc	8	139593211
ebi-a-GCST90001995	rs41307457	CX3CR1 on monoc	9	90340916
ebi-a-GCST90001995	rs17095454	CX3CR1 on monoc	10	113333619
ebi-a-GCST90001995	rs117498337	CX3CR1 on monoc	11	123076792
ebi-a-GCST90001995	rs9584784	CX3CR1 on monoc	13	98918942
ebi-a-GCST90001995	rs61957406	CX3CR1 on monoc	13	23374374
ebi-a-GCST90001995	rs8026803	CX3CR1 on monoc	15	80260014
ebi-a-GCST90001995	rs8039434	CX3CR1 on monoc	15	33869977
ebi-a-GCST90001995	rs75827471	CX3CR1 on monoc	16	4267170
ebi-a-GCST90001995	rs71387414	CX3CR1 on monoc	17	53769223
ebi-a-GCST90001995	rs6039829	CX3CR1 on monoc	20	10289371
ebi-a-GCST90001996	rs1801274	CX3CR1 on CD14-	1	161479745
ebi-a-GCST90001996	rs16843658	CX3CR1 on CD14-	1	162672134
ebi-a-GCST90001996	rs149100	CX3CR1 on CD14-	1	162441037
ebi-a-GCST90001996	rs1376624	CX3CR1 on CD14-	2	187771135
ebi-a-GCST90001996	rs62170788	CX3CR1 on CD14-	2	170958389
ebi-a-GCST90001996	rs72918158	CX3CR1 on CD14-	3	87078837
ebi-a-GCST90001996	rs58215329	CX3CR1 on CD14-	3	196465812
ebi-a-GCST90001996	rs143002526	CX3CR1 on CD14-	3	184649632
ebi-a-GCST90001996	rs28491136	CX3CR1 on CD14-	3	106964051
ebi-a-GCST90001996	rs9823718	CX3CR1 on CD14-	3	39293757
ebi-a-GCST90001996	rs2649752	CX3CR1 on CD14-	3	39351805

ebi-a-GCST90001996	rs10011142	CX3CR1 on CD14-	4	5015486
ebi-a-GCST90001996	rs4957969	CX3CR1 on CD14-	5	110958260
ebi-a-GCST90001996	rs372655475	CX3CR1 on CD14-	6	68886782
ebi-a-GCST90001996	rs11768517	CX3CR1 on CD14-	7	54919768
ebi-a-GCST90001996	rs10499824	CX3CR1 on CD14-	7	75171091
ebi-a-GCST90001996	rs6952061	CX3CR1 on CD14-	7	30459852
ebi-a-GCST90001996	rs7791415	CX3CR1 on CD14-	7	44433778
ebi-a-GCST90001996	rs148057702	CX3CR1 on CD14-	7	97552526
ebi-a-GCST90001996	rs117940302	CX3CR1 on CD14-	8	123834904
ebi-a-GCST90001996	rs117871360	CX3CR1 on CD14-	8	3690065
ebi-a-GCST90001996	rs16867973	CX3CR1 on CD14-	8	102610279
ebi-a-GCST90001996	rs17095454	CX3CR1 on CD14-	10	113333619
ebi-a-GCST90001996	rs74574092	CX3CR1 on CD14-	11	100513751
ebi-a-GCST90001996	rs894107	CX3CR1 on CD14-	11	71019939
ebi-a-GCST90001996	rs117043578	CX3CR1 on CD14-	13	104381481
ebi-a-GCST90001996	rs9542786	CX3CR1 on CD14-	13	72514633
ebi-a-GCST90001996	rs12372861	CX3CR1 on CD14-	14	96662640
ebi-a-GCST90001996	rs182244806	CX3CR1 on CD14-	15	68651749
ebi-a-GCST90001996	rs59871438	CX3CR1 on CD14-	15	97432077
ebi-a-GCST90001996	rs17549676	CX3CR1 on CD14-	16	63344732
ebi-a-GCST90001996	rs112450242	CX3CR1 on CD14-	17	75272996
ebi-a-GCST90001996	rs117896307	CX3CR1 on CD14-	18	70433020
ebi-a-GCST90001996	rs139952138	CX3CR1 on CD14-	22	33593799
ebi-a-GCST90001997	rs141845371	CX3CR1 on CD14-	1	194738786
ebi-a-GCST90001997	rs1801274	CX3CR1 on CD14-	1	161479745
ebi-a-GCST90001997	rs67252300	CX3CR1 on CD14-	1	82669375
ebi-a-GCST90001997	rs4622126	CX3CR1 on CD14-	1	228017395
ebi-a-GCST90001997	rs12023218	CX3CR1 on CD14-	1	161269897
ebi-a-GCST90001997	rs62172045	CX3CR1 on CD14-	2	170899008
ebi-a-GCST90001997	rs77705296	CX3CR1 on CD14-	2	158703713
ebi-a-GCST90001997	rs13396271	CX3CR1 on CD14-	2	17055946
ebi-a-GCST90001997	rs115129679	CX3CR1 on CD14-	2	77793865
ebi-a-GCST90001997	rs9823718	CX3CR1 on CD14-	3	39293757
ebi-a-GCST90001997	rs72865957	CX3CR1 on CD14-	3	39355919
ebi-a-GCST90001997	rs73056479	CX3CR1 on CD14-	3	38700237
ebi-a-GCST90001997	rs143813477	CX3CR1 on CD14-	3	44507750
ebi-a-GCST90001997	rs9879669	CX3CR1 on CD14-	3	7682013
ebi-a-GCST90001997	rs6414558	CX3CR1 on CD14-	3	63548238
ebi-a-GCST90001997	rs4957969	CX3CR1 on CD14-	5	110958260
ebi-a-GCST90001997	rs17736094	CX3CR1 on CD14-	5	5711054
ebi-a-GCST90001997	rs17521541	CX3CR1 on CD14-	5	24624788
ebi-a-GCST90001997	rs4729041	CX3CR1 on CD14-	7	92063500
ebi-a-GCST90001997	rs10951824	CX3CR1 on CD14-	7	45618083
ebi-a-GCST90001997	rs115689956	CX3CR1 on CD14-	7	78974417
ebi-a-GCST90001997	rs17095454	CX3CR1 on CD14-	10	113333619
ebi-a-GCST90001997	rs1571526	CX3CR1 on CD14-	13	98918488
ebi-a-GCST90001997	rs72770576	CX3CR1 on CD14-	16	22948093
ebi-a-GCST90001997	rs75827471	CX3CR1 on CD14-	16	4267170

ebi-a-GCST90001997	rs8064010	CX3CR1 on CD14-	16	78685218
ebi-a-GCST90001997	rs146719695	CX3CR1 on CD14-	22	44817424
ebi-a-GCST90001998	rs12025396	PDL-1 on CD14+ C	1	107729118
ebi-a-GCST90001998	rs61782288	PDL-1 on CD14+ C	1	67561056
ebi-a-GCST90001998	rs61759109	PDL-1 on CD14+ C	1	30574812
ebi-a-GCST90001998	rs1801274	PDL-1 on CD14+ C	1	161479745
ebi-a-GCST90001998	rs142118225	PDL-1 on CD14+ C	2	173198472
ebi-a-GCST90001998	rs191830506	PDL-1 on CD14+ C	2	218615431
ebi-a-GCST90001998	rs74782463	PDL-1 on CD14+ C	3	190713424
ebi-a-GCST90001998	rs1402451	PDL-1 on CD14+ C	3	132795714
ebi-a-GCST90001998	rs342002	PDL-1 on CD14+ C	3	5307071
ebi-a-GCST90001998	rs11952327	PDL-1 on CD14+ C	5	32672017
ebi-a-GCST90001998	rs139016775	PDL-1 on CD14+ C	5	172936151
ebi-a-GCST90001998	rs62477578	PDL-1 on CD14+ C	7	75430887
ebi-a-GCST90001998	rs1419935	PDL-1 on CD14+ C	7	151327301
ebi-a-GCST90001998	rs4737476	PDL-1 on CD14+ C	8	58709282
ebi-a-GCST90001998	rs6992027	PDL-1 on CD14+ C	8	136670964
ebi-a-GCST90001998	rs28509412	PDL-1 on CD14+ C	8	138210917
ebi-a-GCST90001998	rs34805084	PDL-1 on CD14+ C	9	71230197
ebi-a-GCST90001998	rs4750710	PDL-1 on CD14+ C	10	130347772
ebi-a-GCST90001998	rs12793538	PDL-1 on CD14+ C	11	103476499
ebi-a-GCST90001998	rs61932869	PDL-1 on CD14+ C	12	74304374
ebi-a-GCST90001998	rs17261924	PDL-1 on CD14+ C	13	38290910
ebi-a-GCST90001998	rs9527890	PDL-1 on CD14+ C	13	59355631
ebi-a-GCST90001998	rs77640671	PDL-1 on CD14+ C	16	58607804
ebi-a-GCST90001998	rs16950913	PDL-1 on CD14+ C	17	14952998
ebi-a-GCST90001998	rs8089422	PDL-1 on CD14+ C	18	54525353
ebi-a-GCST90001998	rs144426024	PDL-1 on CD14+ C	18	56897917
ebi-a-GCST90001998	rs34690145	PDL-1 on CD14+ C	19	3759702
ebi-a-GCST90001998	rs35585770	PDL-1 on CD14+ C	20	3634123
ebi-a-GCST90001999	rs150839274	PDL-1 on CD14- C	1	63779172
ebi-a-GCST90001999	rs35384695	PDL-1 on CD14- C	1	233457799
ebi-a-GCST90001999	rs74341264	PDL-1 on CD14- C	1	161561151
ebi-a-GCST90001999	rs71632989	PDL-1 on CD14- C	1	161533055
ebi-a-GCST90001999	rs1883578	PDL-1 on CD14- C	1	176446562
ebi-a-GCST90001999	rs72700199	PDL-1 on CD14- C	1	162324646
ebi-a-GCST90001999	rs113565695	PDL-1 on CD14- C	2	175008676
ebi-a-GCST90001999	rs200614043	PDL-1 on CD14- C	2	79447312
ebi-a-GCST90001999	rs114864027	PDL-1 on CD14- C	5	152958194
ebi-a-GCST90001999	rs12524781	PDL-1 on CD14- C	6	154891396
ebi-a-GCST90001999	rs4737476	PDL-1 on CD14- C	8	58709282
ebi-a-GCST90001999	rs7841427	PDL-1 on CD14- C	8	142112218
ebi-a-GCST90001999	rs72766757	PDL-1 on CD14- C	10	1716746
ebi-a-GCST90001999	rs72882056	PDL-1 on CD14- C	11	5740015
ebi-a-GCST90001999	rs117552079	PDL-1 on CD14- C	11	73714261
ebi-a-GCST90001999	rs111824642	PDL-1 on CD14- C	12	120526148
ebi-a-GCST90001999	rs12367061	PDL-1 on CD14- C	12	119294464
ebi-a-GCST90001999	rs11107060	PDL-1 on CD14- C	12	93916757

ebi-a-GCST90001999	rs710697	PDL-1 on CD14- C	12	70371053
ebi-a-GCST90001999	rs74363387	PDL-1 on CD14- C	20	48899907
ebi-a-GCST90001999	rs113887836	PDL-1 on CD14- C	22	46233179
ebi-a-GCST90002000	rs139529340	PDL-1 on CD14- C	1	161563554
ebi-a-GCST90002000	rs12042800	PDL-1 on CD14- C	1	245930713
ebi-a-GCST90002000	rs77455113	PDL-1 on CD14- C	1	198518569
ebi-a-GCST90002000	rs9728174	PDL-1 on CD14- C	1	240600690
ebi-a-GCST90002000	rs62649771	PDL-1 on CD14- C	2	5649823
ebi-a-GCST90002000	rs72928949	PDL-1 on CD14- C	2	203570761
ebi-a-GCST90002000	rs4973409	PDL-1 on CD14- C	2	232311871
ebi-a-GCST90002000	rs149427550	PDL-1 on CD14- C	2	240353586
ebi-a-GCST90002000	rs6770996	PDL-1 on CD14- C	3	147470053
ebi-a-GCST90002000	rs719508	PDL-1 on CD14- C	3	65696503
ebi-a-GCST90002000	rs77153881	PDL-1 on CD14- C	3	38673487
ebi-a-GCST90002000	rs183608856	PDL-1 on CD14- C	4	77258306
ebi-a-GCST90002000	rs139016879	PDL-1 on CD14- C	4	77737913
ebi-a-GCST90002000	rs2654849	PDL-1 on CD14- C	4	100396690
ebi-a-GCST90002000	rs143753691	PDL-1 on CD14- C	4	80579882
ebi-a-GCST90002000	rs35141864	PDL-1 on CD14- C	4	7148099
ebi-a-GCST90002000	rs78511754	PDL-1 on CD14- C	7	78911065
ebi-a-GCST90002000	rs73168946	PDL-1 on CD14- C	7	138590465
ebi-a-GCST90002000	rs2969497	PDL-1 on CD14- C	7	4926535
ebi-a-GCST90002000	rs682812	PDL-1 on CD14- C	8	2821756
ebi-a-GCST90002000	rs143917558	PDL-1 on CD14- C	9	106448354
ebi-a-GCST90002000	rs12256905	PDL-1 on CD14- C	10	32700539
ebi-a-GCST90002000	rs4980645	PDL-1 on CD14- C	11	70616492
ebi-a-GCST90002000	rs1800973	PDL-1 on CD14- C	12	69744014
ebi-a-GCST90002000	rs1628382	PDL-1 on CD14- C	13	95764061
ebi-a-GCST90002000	rs4885909	PDL-1 on CD14- C	13	84709903
ebi-a-GCST90002000	rs73516783	PDL-1 on CD14- C	13	70618933
ebi-a-GCST90002000	rs79262480	PDL-1 on CD14- C	15	70503605
ebi-a-GCST90002000	rs2331414	PDL-1 on CD14- C	17	62161454
ebi-a-GCST90002001	rs150145799	CD64 on CD14- CI	1	149738590
ebi-a-GCST90002001	rs72930744	CD64 on CD14- CI	2	203597269
ebi-a-GCST90002001	rs4077641	CD64 on CD14- CI	2	161551327
ebi-a-GCST90002001	rs9830508	CD64 on CD14- CI	3	177553362
ebi-a-GCST90002001	rs6834422	CD64 on CD14- CI	4	131175275
ebi-a-GCST90002001	rs7714855	CD64 on CD14- CI	5	147431095
ebi-a-GCST90002001	rs558721	CD64 on CD14- CI	6	32570573
ebi-a-GCST90002001	rs183506865	CD64 on CD14- CI	6	137921821
ebi-a-GCST90002001	rs7002380	CD64 on CD14- CI	8	124722391
ebi-a-GCST90002001	rs189868170	CD64 on CD14- CI	9	8755271
ebi-a-GCST90002001	rs74149061	CD64 on CD14- CI	10	92919256
ebi-a-GCST90002001	rs116872398	CD64 on CD14- CI	11	108805701
ebi-a-GCST90002001	rs1566303	CD64 on CD14- CI	12	63704970
ebi-a-GCST90002001	rs1800973	CD64 on CD14- CI	12	69744014
ebi-a-GCST90002001	rs189043718	CD64 on CD14- CI	13	28118710
ebi-a-GCST90002001	rs9597833	CD64 on CD14- CI	13	59334076

ebi-a-GCST90002001	rs112698696	CD64 on CD14- CI	17	25745048
ebi-a-GCST90002002	rs4908637	PDL-1 on monocyte	1	7482757
ebi-a-GCST90002002	rs9427401	PDL-1 on monocyte	1	161492175
ebi-a-GCST90002002	rs2569177	PDL-1 on monocyte	5	139992162
ebi-a-GCST90002002	rs74538501	PDL-1 on monocyte	5	23367000
ebi-a-GCST90002002	rs10276990	PDL-1 on monocyte	7	16903052
ebi-a-GCST90002002	rs73160029	PDL-1 on monocyte	7	151523758
ebi-a-GCST90002002	rs147810622	PDL-1 on monocyte	7	151910695
ebi-a-GCST90002002	rs10103683	PDL-1 on monocyte	8	106272376
ebi-a-GCST90002002	rs11038817	PDL-1 on monocyte	11	46226004
ebi-a-GCST90002002	rs578253340	PDL-1 on monocyte	16	57140525
ebi-a-GCST90002002	rs17851876	PDL-1 on monocyte	16	67979423
ebi-a-GCST90002002	rs12953239	PDL-1 on monocyte	17	35199795
ebi-a-GCST90002002	rs10454715	PDL-1 on monocyte	18	48996522
ebi-a-GCST90002002	rs8089422	PDL-1 on monocyte	18	54525353
ebi-a-GCST90002002	rs75567064	PDL-1 on monocyte	20	48898441
ebi-a-GCST90002002	rs4809811	PDL-1 on monocyte	20	49351740
ebi-a-GCST90002003	rs146537837	CCR2 on CD14- CI	1	98705577
ebi-a-GCST90002003	rs17608966	CCR2 on CD14- CI	2	15278190
ebi-a-GCST90002003	rs762789	CCR2 on CD14- CI	3	46402627
ebi-a-GCST90002003	rs28692786	CCR2 on CD14- CI	3	119522753
ebi-a-GCST90002003	rs9825805	CCR2 on CD14- CI	3	177552702
ebi-a-GCST90002003	rs144199265	CCR2 on CD14- CI	4	63222990
ebi-a-GCST90002003	rs2973741	CCR2 on CD14- CI	5	177821665
ebi-a-GCST90002003	rs188482110	CCR2 on CD14- CI	5	68260933
ebi-a-GCST90002003	rs77465361	CCR2 on CD14- CI	5	159009803
ebi-a-GCST90002003	rs3793333	CCR2 on CD14- CI	7	156801023
ebi-a-GCST90002003	rs117383806	CCR2 on CD14- CI	8	140934719
ebi-a-GCST90002003	rs7131521	CCR2 on CD14- CI	11	108956889
ebi-a-GCST90002003	rs144044548	CCR2 on CD14- CI	12	30923098
ebi-a-GCST90002003	rs1800973	CCR2 on CD14- CI	12	69744014
ebi-a-GCST90002003	rs11655786	CCR2 on CD14- CI	17	21074153
ebi-a-GCST90002003	rs139270458	CCR2 on CD14- CI	17	16283736
ebi-a-GCST90002003	rs6417105	CCR2 on CD14- CI	18	47013723
ebi-a-GCST90002003	rs141988029	CCR2 on CD14- CI	18	61962829
ebi-a-GCST90002003	rs140264318	CCR2 on CD14- CI	19	29692512
ebi-a-GCST90002003	rs12981940	CCR2 on CD14- CI	19	35789377
ebi-a-GCST90002004	rs114744368	CCR2 on CD14+ C	1	240779226
ebi-a-GCST90002004	rs12613485	CCR2 on CD14+ C	2	102154764
ebi-a-GCST90002004	rs2683681	CCR2 on CD14+ C	2	67747535
ebi-a-GCST90002004	rs2160337	CCR2 on CD14+ C	2	36964751
ebi-a-GCST90002004	rs116078872	CCR2 on CD14+ C	2	239553302
ebi-a-GCST90002004	rs1386098	CCR2 on CD14+ C	3	97446764
ebi-a-GCST90002004	rs4683184	CCR2 on CD14+ C	3	46187707
ebi-a-GCST90002004	rs79825014	CCR2 on CD14+ C	3	46880193
ebi-a-GCST90002004	rs13060505	CCR2 on CD14+ C	3	46968023
ebi-a-GCST90002004	rs2270454	CCR2 on CD14+ C	3	10292140
ebi-a-GCST90002004	rs141411805	CCR2 on CD14+ C	3	53544015

ebi-a-GCST90002004	rs184445588	CCR2 on CD14+ C	3	47210979
ebi-a-GCST90002004	rs777404	CCR2 on CD14+ C	4	71016615
ebi-a-GCST90002004	rs62327180	CCR2 on CD14+ C	4	103540762
ebi-a-GCST90002004	rs111162334	CCR2 on CD14+ C	7	51430245
ebi-a-GCST90002004	rs28561969	CCR2 on CD14+ C	7	231461
ebi-a-GCST90002004	rs7000391	CCR2 on CD14+ C	8	12709650
ebi-a-GCST90002004	rs56407854	CCR2 on CD14+ C	8	136669344
ebi-a-GCST90002004	rs147404212	CCR2 on CD14+ C	9	100014987
ebi-a-GCST90002004	rs10081735	CCR2 on CD14+ C	9	105486830
ebi-a-GCST90002004	rs190371975	CCR2 on CD14+ C	9	106439903
ebi-a-GCST90002004	rs7896883	CCR2 on CD14+ C	10	98787354
ebi-a-GCST90002004	rs78109162	CCR2 on CD14+ C	11	129986854
ebi-a-GCST90002004	rs10878258	CCR2 on CD14+ C	12	40641692
ebi-a-GCST90002004	rs12185230	CCR2 on CD14+ C	17	79591388
ebi-a-GCST90002004	rs112976913	CCR2 on CD14+ C	17	69699532
ebi-a-GCST90002004	rs79049673	CCR2 on CD14+ C	19	3274527
ebi-a-GCST90002004	rs2426939	CCR2 on CD14+ C	20	59484022
ebi-a-GCST90002005	rs71632979	CD16 on CD14+ C	1	161526397
ebi-a-GCST90002005	rs115805162	CD16 on CD14+ C	1	161410253
ebi-a-GCST90002005	rs187197560	CD16 on CD14+ C	1	161006723
ebi-a-GCST90002005	rs147678401	CD16 on CD14+ C	1	161035780
ebi-a-GCST90002005	rs143140559	CD16 on CD14+ C	1	160972255
ebi-a-GCST90002005	rs73008073	CD16 on CD14+ C	1	149241581
ebi-a-GCST90002005	rs71639910	CD16 on CD14+ C	1	161937774
ebi-a-GCST90002005	rs113857680	CD16 on CD14+ C	1	56634923
ebi-a-GCST90002005	rs35583090	CD16 on CD14+ C	1	160450885
ebi-a-GCST90002005	rs3842968	CD16 on CD14+ C	1	95320281
ebi-a-GCST90002005	rs250957	CD16 on CD14+ C	2	234992975
ebi-a-GCST90002005	rs73976590	CD16 on CD14+ C	2	173072824
ebi-a-GCST90002005	rs2342702	CD16 on CD14+ C	2	45411188
ebi-a-GCST90002005	rs79338412	CD16 on CD14+ C	3	41079463
ebi-a-GCST90002005	rs13361130	CD16 on CD14+ C	5	177378903
ebi-a-GCST90002005	rs141952082	CD16 on CD14+ C	6	3541155
ebi-a-GCST90002005	rs6899613	CD16 on CD14+ C	6	148553992
ebi-a-GCST90002005	rs6955633	CD16 on CD14+ C	7	71270245
ebi-a-GCST90002005	rs817258	CD16 on CD14+ C	8	2779954
ebi-a-GCST90002005	rs117725134	CD16 on CD14+ C	11	106624331
ebi-a-GCST90002005	rs72778152	CD16 on CD14+ C	16	3741396
ebi-a-GCST90002005	rs8052828	CD16 on CD14+ C	16	1526293
ebi-a-GCST90002005	rs112843603	CD16 on CD14+ C	19	52648160
ebi-a-GCST90002005	rs75068893	CD16 on CD14+ C	19	55794439
ebi-a-GCST90002006	rs12023218	CD64 on monocyte	1	161269897
ebi-a-GCST90002006	rs3007144	CD64 on monocyte	1	160929890
ebi-a-GCST90002006	rs587759315	CD64 on monocyte	1	121480104
ebi-a-GCST90002006	rs192899694	CD64 on monocyte	1	146480265
ebi-a-GCST90002006	rs141156871	CD64 on monocyte	1	150447458
ebi-a-GCST90002006	rs145828126	CD64 on monocyte	1	120233380
ebi-a-GCST90002006	rs188268415	CD64 on monocyte	1	150401679

ebi-a-GCST90002006	rs1801274	CD64 on monocyte	1	161479745
ebi-a-GCST90002006	rs144823175	CD64 on monocyte	1	149858520
ebi-a-GCST90002006	rs150145799	CD64 on monocyte	1	149738590
ebi-a-GCST90002006	rs116004327	CD64 on monocyte	1	117459175
ebi-a-GCST90002006	rs112755578	CD64 on monocyte	1	149716369
ebi-a-GCST90002006	rs116770889	CD64 on monocyte	1	116993158
ebi-a-GCST90002006	rs116275309	CD64 on monocyte	1	150863650
ebi-a-GCST90002006	rs114066620	CD64 on monocyte	2	67612820
ebi-a-GCST90002006	rs76258977	CD64 on monocyte	2	219052326
ebi-a-GCST90002006	rs6780007	CD64 on monocyte	3	55074425
ebi-a-GCST90002006	rs11934532	CD64 on monocyte	4	182164089
ebi-a-GCST90002006	rs190928917	CD64 on monocyte	6	157071062
ebi-a-GCST90002006	rs192225504	CD64 on monocyte	7	1597362
ebi-a-GCST90002006	rs10276990	CD64 on monocyte	7	16903052
ebi-a-GCST90002006	rs192997418	CD64 on monocyte	8	99553474
ebi-a-GCST90002006	rs80178684	CD64 on monocyte	8	59726631
ebi-a-GCST90002006	rs6480509	CD64 on monocyte	10	73071462
ebi-a-GCST90002006	rs142408635	CD64 on monocyte	11	92180250
ebi-a-GCST90002006	rs11065979	CD64 on monocyte	12	112059557
ebi-a-GCST90002006	rs58902422	CD64 on monocyte	14	39342923
ebi-a-GCST90002006	rs16960068	CD64 on monocyte	17	64692068
ebi-a-GCST90002006	rs9941795	CD64 on monocyte	21	19063114
ebi-a-GCST90002007	rs7514594	HLA DR on CD14+	1	209819061
ebi-a-GCST90002007	rs77131320	HLA DR on CD14+	1	191529098
ebi-a-GCST90002007	rs144126567	HLA DR on CD14+	1	161510516
ebi-a-GCST90002007	rs62176112	HLA DR on CD14+	2	179315726
ebi-a-GCST90002007	rs1015298	HLA DR on CD14+	3	8652215
ebi-a-GCST90002007	rs6772253	HLA DR on CD14+	3	151060301
ebi-a-GCST90002007	rs12485444	HLA DR on CD14+	3	188135783
ebi-a-GCST90002007	rs147095653	HLA DR on CD14+	3	42422119
ebi-a-GCST90002007	rs1044722	HLA DR on CD14+	4	164447976
ebi-a-GCST90002007	rs118167703	HLA DR on CD14+	6	131880206
ebi-a-GCST90002007	rs6934244	HLA DR on CD14+	6	33024032
ebi-a-GCST90002007	rs12198220	HLA DR on CD14+	6	21330293
ebi-a-GCST90002007	rs9275511	HLA DR on CD14+	6	32674329
ebi-a-GCST90002007	rs6917212	HLA DR on CD14+	6	32583005
ebi-a-GCST90002007	rs9275639	HLA DR on CD14+	6	32684831
ebi-a-GCST90002007	rs62395272	HLA DR on CD14+	6	31394424
ebi-a-GCST90002007	rs12196486	HLA DR on CD14+	6	3041467
ebi-a-GCST90002007	rs4918496	HLA DR on CD14+	10	112087579
ebi-a-GCST90002007	rs117870500	HLA DR on CD14+	10	111066806
ebi-a-GCST90002007	rs60924036	HLA DR on CD14+	15	35035866
ebi-a-GCST90002007	rs74525750	HLA DR on CD14+	16	82812242
ebi-a-GCST90002007	rs12930083	HLA DR on CD14+	16	26883757
ebi-a-GCST90002008	rs74124962	CCR2 on monocyte	1	161408398
ebi-a-GCST90002008	rs17017001	CCR2 on monocyte	1	207019530
ebi-a-GCST90002008	rs149527001	CCR2 on monocyte	1	4901170
ebi-a-GCST90002008	rs12613485	CCR2 on monocyte	2	102154764

ebi-a-GCST90002008	rs35526800	CCR2 on monocyte	2	6628808
ebi-a-GCST90002008	rs4683184	CCR2 on monocyte	3	46187707
ebi-a-GCST90002008	rs147120184	CCR2 on monocyte	3	189165767
ebi-a-GCST90002008	rs13060505	CCR2 on monocyte	3	46968023
ebi-a-GCST90002008	rs141411805	CCR2 on monocyte	3	53544015
ebi-a-GCST90002008	rs79641389	CCR2 on monocyte	3	46882338
ebi-a-GCST90002008	rs78246754	CCR2 on monocyte	3	133776890
ebi-a-GCST90002008	rs777415	CCR2 on monocyte	4	71028584
ebi-a-GCST90002008	rs150805883	CCR2 on monocyte	6	30200969
ebi-a-GCST90002008	rs144682094	CCR2 on monocyte	7	6359771
ebi-a-GCST90002008	rs192191749	CCR2 on monocyte	8	9002502
ebi-a-GCST90002008	rs113111100	CCR2 on monocyte	10	129087440
ebi-a-GCST90002008	rs625761	CCR2 on monocyte	11	100644569
ebi-a-GCST90002008	rs11606662	CCR2 on monocyte	11	75224900
ebi-a-GCST90002008	rs10878258	CCR2 on monocyte	12	40641692
ebi-a-GCST90002008	rs34463381	CCR2 on monocyte	12	131501922
ebi-a-GCST90002008	rs116966304	CCR2 on monocyte	14	101368491
ebi-a-GCST90002008	rs12185230	CCR2 on monocyte	17	79591388
ebi-a-GCST90002008	rs77772425	CCR2 on monocyte	17	30219703
ebi-a-GCST90002008	rs187328631	CCR2 on monocyte	19	16862057
ebi-a-GCST90002008	rs79049673	CCR2 on monocyte	19	3274527
ebi-a-GCST90002009	rs12466446	HLA DR on CD14-	2	212326642
ebi-a-GCST90002009	rs113688693	HLA DR on CD14-	2	235115726
ebi-a-GCST90002009	rs62176112	HLA DR on CD14-	2	179315726
ebi-a-GCST90002009	rs17486987	HLA DR on CD14-	3	169164828
ebi-a-GCST90002009	rs139738120	HLA DR on CD14-	3	167150368
ebi-a-GCST90002009	rs6790767	HLA DR on CD14-	3	39296105
ebi-a-GCST90002009	rs17008807	HLA DR on CD14-	4	91154964
ebi-a-GCST90002009	rs72502555	HLA DR on CD14-	6	31234208
ebi-a-GCST90002009	rs9268430	HLA DR on CD14-	6	32345429
ebi-a-GCST90002009	rs1415809	HLA DR on CD14-	6	145648573
ebi-a-GCST90002009	rs10485346	HLA DR on CD14-	6	33866059
ebi-a-GCST90002009	rs34291045	HLA DR on CD14-	6	32560385
ebi-a-GCST90002009	rs116683794	HLA DR on CD14-	6	33034114
ebi-a-GCST90002009	rs111407432	HLA DR on CD14-	6	152585213
ebi-a-GCST90002009	rs2734573	HLA DR on CD14-	6	31494738
ebi-a-GCST90002009	rs75289830	HLA DR on CD14-	7	781754
ebi-a-GCST90002009	rs78417437	HLA DR on CD14-	10	80345436
ebi-a-GCST90002009	rs189320558	HLA DR on CD14-	10	125333759
ebi-a-GCST90002009	rs117789335	HLA DR on CD14-	10	125617894
ebi-a-GCST90002009	rs78010789	HLA DR on CD14-	12	113317826
ebi-a-GCST90002009	rs11071444	HLA DR on CD14-	15	59740740
ebi-a-GCST90002009	rs2109574	HLA DR on CD14-	15	97464581
ebi-a-GCST90002009	rs142006783	HLA DR on CD14-	16	74651733
ebi-a-GCST90002009	rs4780334	HLA DR on CD14-	16	11002626
ebi-a-GCST90002009	rs147554267	HLA DR on CD14-	18	59045629
ebi-a-GCST90002009	rs7354407	HLA DR on CD14-	20	52844986
ebi-a-GCST90002009	rs6070460	HLA DR on CD14-	20	56988186

ebi-a-GCST90002009	rs6139723	HLA DR on CD14-	20	5397579
ebi-a-GCST90002010	rs148974843	HLA DR on monoc	1	237971715
ebi-a-GCST90002010	rs11581732	HLA DR on monoc	1	25527587
ebi-a-GCST90002010	rs113337372	HLA DR on monoc	2	121677690
ebi-a-GCST90002010	rs11737889	HLA DR on monoc	4	64395080
ebi-a-GCST90002010	rs113555953	HLA DR on monoc	4	97898706
ebi-a-GCST90002010	rs9469245	HLA DR on monoc	6	32692084
ebi-a-GCST90002010	rs72990264	HLA DR on monoc	6	143148113
ebi-a-GCST90002010	rs62395272	HLA DR on monoc	6	31394424
ebi-a-GCST90002010	rs115363051	HLA DR on monoc	6	31444174
ebi-a-GCST90002010	rs9270588	HLA DR on monoc	6	32561327
ebi-a-GCST90002010	rs9271768	HLA DR on monoc	6	32594188
ebi-a-GCST90002010	rs1449650	HLA DR on monoc	9	104844038
ebi-a-GCST90002010	rs12235220	HLA DR on monoc	9	123971122
ebi-a-GCST90002010	rs1800973	HLA DR on monoc	12	69744014
ebi-a-GCST90002010	rs7321974	HLA DR on monoc	13	95665658
ebi-a-GCST90002010	rs62005962	HLA DR on monoc	15	54040290
ebi-a-GCST90002010	rs142006783	HLA DR on monoc	16	74651733
ebi-a-GCST90002010	rs142573049	HLA DR on monoc	20	60176623
ebi-a-GCST90002011	rs7543360	CD64 on CD14+ C	1	38659743
ebi-a-GCST90002011	rs16869776	CD64 on CD14+ C	4	20594904
ebi-a-GCST90002011	rs7685215	CD64 on CD14+ C	4	182195837
ebi-a-GCST90002011	rs146963227	CD64 on CD14+ C	4	17530633
ebi-a-GCST90002011	rs326052	CD64 on CD14+ C	5	1004879
ebi-a-GCST90002011	rs190928917	CD64 on CD14+ C	6	157071062
ebi-a-GCST90002011	rs80178684	CD64 on CD14+ C	8	59726631
ebi-a-GCST90002011	rs34113791	CD64 on CD14+ C	8	97002092
ebi-a-GCST90002011	rs627319	CD64 on CD14+ C	11	62247621
ebi-a-GCST90002011	rs11065979	CD64 on CD14+ C	12	112059557
ebi-a-GCST90002011	rs7333071	CD64 on CD14+ C	13	59333719
ebi-a-GCST90002011	rs71404123	CD64 on CD14+ C	16	83953190
ebi-a-GCST90002011	rs16960068	CD64 on CD14+ C	17	64692068
ebi-a-GCST90002011	rs57732730	CD64 on CD14+ C	21	19054955
ebi-a-GCST90002012	rs1801274	CX3CR1 on CD14-	1	161479745
ebi-a-GCST90002012	rs149100	CX3CR1 on CD14-	1	162441037
ebi-a-GCST90002012	rs10779294	CX3CR1 on CD14-	1	217426519
ebi-a-GCST90002012	rs76913544	CX3CR1 on CD14-	2	201190732
ebi-a-GCST90002012	rs6754954	CX3CR1 on CD14-	2	141839471
ebi-a-GCST90002012	rs9867963	CX3CR1 on CD14-	3	11469126
ebi-a-GCST90002012	rs9877538	CX3CR1 on CD14-	3	39289664
ebi-a-GCST90002012	rs145135505	CX3CR1 on CD14-	3	38928831
ebi-a-GCST90002012	rs58215329	CX3CR1 on CD14-	3	196465812
ebi-a-GCST90002012	rs140808371	CX3CR1 on CD14-	4	23114161
ebi-a-GCST90002012	rs75379034	CX3CR1 on CD14-	4	182251306
ebi-a-GCST90002012	rs1024619	CX3CR1 on CD14-	5	11054713
ebi-a-GCST90002012	rs12655590	CX3CR1 on CD14-	5	169085561
ebi-a-GCST90002012	rs1208168	CX3CR1 on CD14-	7	144102599
ebi-a-GCST90002012	rs9415987	CX3CR1 on CD14-	10	73440628

ebi-a-GCST90002012	rs74389435	CX3CR1 on CD14-	11	101949821
ebi-a-GCST90002012	rs2292701	CX3CR1 on CD14-	12	20876574
ebi-a-GCST90002012	rs10130058	CX3CR1 on CD14-	14	22045954
ebi-a-GCST90002012	rs112450242	CX3CR1 on CD14-	17	75272996
ebi-a-GCST90002012	rs72932967	CX3CR1 on CD14-	18	8922463
ebi-a-GCST90002012	rs41314914	CX3CR1 on CD14-	20	48892942
ebi-a-GCST90002013	rs55971447	CCR2 on myeloid I	1	161515326
ebi-a-GCST90002013	rs16827093	CCR2 on myeloid I	1	22744534
ebi-a-GCST90002013	rs2169052	CCR2 on myeloid I	1	161557154
ebi-a-GCST90002013	rs116800975	CCR2 on myeloid I	1	162238738
ebi-a-GCST90002013	rs10914660	CCR2 on myeloid I	1	33597788
ebi-a-GCST90002013	rs4384829	CCR2 on myeloid I	2	30466802
ebi-a-GCST90002013	rs35587265	CCR2 on myeloid I	3	46259296
ebi-a-GCST90002013	rs542104164	CCR2 on myeloid I	4	35634867
ebi-a-GCST90002013	rs147275564	CCR2 on myeloid I	4	135382945
ebi-a-GCST90002013	rs139808988	CCR2 on myeloid I	6	50581099
ebi-a-GCST90002013	rs11775437	CCR2 on myeloid I	8	139675584
ebi-a-GCST90002013	rs969808	CCR2 on myeloid I	8	72599402
ebi-a-GCST90002013	rs143366996	CCR2 on myeloid I	10	83561663
ebi-a-GCST90002013	rs2856092	CCR2 on myeloid I	11	1075194
ebi-a-GCST90002014	rs139258218	CCR2 on CD62L+	1	118620702
ebi-a-GCST90002014	rs6687275	CCR2 on CD62L+	1	161515638
ebi-a-GCST90002014	rs16827093	CCR2 on CD62L+	1	22744534
ebi-a-GCST90002014	rs61802333	CCR2 on CD62L+	1	161589982
ebi-a-GCST90002014	rs3813658	CCR2 on CD62L+	2	30473522
ebi-a-GCST90002014	rs35587265	CCR2 on CD62L+	3	46259296
ebi-a-GCST90002014	rs115044799	CCR2 on CD62L+	3	47053135
ebi-a-GCST90002014	rs115100307	CCR2 on CD62L+	4	31408232
ebi-a-GCST90002014	rs9470090	CCR2 on CD62L+	6	35737527
ebi-a-GCST90002014	rs969808	CCR2 on CD62L+	8	72599402
ebi-a-GCST90002014	rs10977191	CCR2 on CD62L+	9	862889
ebi-a-GCST90002014	rs61896808	CCR2 on CD62L+	11	111183251
ebi-a-GCST90002014	rs12573934	CCR2 on CD62L+	11	4702567
ebi-a-GCST90002014	rs138016528	CCR2 on CD62L+	16	72111834
ebi-a-GCST90002014	rs2011807	CCR2 on CD62L+	16	83976564
ebi-a-GCST90002015	rs67765474	CCR2 on plasmacy	2	64168437
ebi-a-GCST90002015	rs356643	CCR2 on plasmacy	2	101536192
ebi-a-GCST90002015	rs138130091	CCR2 on plasmacy	3	45509296
ebi-a-GCST90002015	rs3092963	CCR2 on plasmacy	3	46396938
ebi-a-GCST90002015	rs6793219	CCR2 on plasmacy	3	54557443
ebi-a-GCST90002015	rs3852012	CCR2 on plasmacy	3	44045981
ebi-a-GCST90002015	rs116403890	CCR2 on plasmacy	4	189033600
ebi-a-GCST90002015	rs62331379	CCR2 on plasmacy	4	158465150
ebi-a-GCST90002015	rs142651372	CCR2 on plasmacy	6	20032295
ebi-a-GCST90002015	rs143407337	CCR2 on plasmacy	7	1095701
ebi-a-GCST90002015	rs13276925	CCR2 on plasmacy	8	62682295
ebi-a-GCST90002015	rs111309798	CCR2 on plasmacy	8	138508508
ebi-a-GCST90002015	rs10759524	CCR2 on plasmacy	9	114834318

ebi-a-GCST90002015	rs7075746	CCR2 on plasmacyt	10	73527925
ebi-a-GCST90002015	rs142099221	CCR2 on plasmacyt	10	85389078
ebi-a-GCST90002015	rs1466802	CCR2 on plasmacyt	11	85927213
ebi-a-GCST90002015	rs78825186	CCR2 on plasmacyt	11	74883559
ebi-a-GCST90002015	rs7303538	CCR2 on plasmacyt	12	119070518
ebi-a-GCST90002015	rs1698491	CCR2 on plasmacyt	14	43236053
ebi-a-GCST90002015	rs145634669	CCR2 on plasmacyt	16	29694391
ebi-a-GCST90002016	rs67765474	CCR2 on CD62L+	2	64168437
ebi-a-GCST90002016	rs356643	CCR2 on CD62L+	2	101536192
ebi-a-GCST90002016	rs148970617	CCR2 on CD62L+	3	51940494
ebi-a-GCST90002016	rs138130091	CCR2 on CD62L+	3	45509296
ebi-a-GCST90002016	rs3092963	CCR2 on CD62L+	3	46396938
ebi-a-GCST90002016	rs3852012	CCR2 on CD62L+	3	44045981
ebi-a-GCST90002016	rs116403890	CCR2 on CD62L+	4	189033600
ebi-a-GCST90002016	rs62331379	CCR2 on CD62L+	4	158465150
ebi-a-GCST90002016	rs142651372	CCR2 on CD62L+	6	20032295
ebi-a-GCST90002016	rs143407337	CCR2 on CD62L+	7	1095701
ebi-a-GCST90002016	rs111309798	CCR2 on CD62L+	8	138508508
ebi-a-GCST90002016	rs13276925	CCR2 on CD62L+	8	62682295
ebi-a-GCST90002016	rs10759524	CCR2 on CD62L+	9	114834318
ebi-a-GCST90002016	rs7075746	CCR2 on CD62L+	10	73527925
ebi-a-GCST90002016	rs142099221	CCR2 on CD62L+	10	85389078
ebi-a-GCST90002016	rs78825186	CCR2 on CD62L+	11	74883559
ebi-a-GCST90002016	rs1466802	CCR2 on CD62L+	11	85927213
ebi-a-GCST90002016	rs7303538	CCR2 on CD62L+	12	119070518
ebi-a-GCST90002016	rs1698491	CCR2 on CD62L+	14	43236053
ebi-a-GCST90002016	rs145634669	CCR2 on CD62L+	16	29694391
ebi-a-GCST90002017	rs1801274	CCR2 on monocyte	1	161479745
ebi-a-GCST90002017	rs111854415	CCR2 on monocyte	1	3399168
ebi-a-GCST90002017	rs1071849	CCR2 on monocyte	1	26646726
ebi-a-GCST90002017	rs11926254	CCR2 on monocyte	3	43380049
ebi-a-GCST90002017	rs13060505	CCR2 on monocyte	3	46968023
ebi-a-GCST90002017	rs144804416	CCR2 on monocyte	3	46861400
ebi-a-GCST90002017	rs112391343	CCR2 on monocyte	3	165060342
ebi-a-GCST90002017	rs35605067	CCR2 on monocyte	3	45153051
ebi-a-GCST90002017	rs114839966	CCR2 on monocyte	3	47357047
ebi-a-GCST90002017	rs353091	CCR2 on monocyte	3	15194949
ebi-a-GCST90002017	rs4683184	CCR2 on monocyte	3	46187707
ebi-a-GCST90002017	rs147217826	CCR2 on monocyte	4	6193223
ebi-a-GCST90002017	rs299597	CCR2 on monocyte	5	34031186
ebi-a-GCST90002017	rs17748581	CCR2 on monocyte	6	150264626
ebi-a-GCST90002017	rs112216269	CCR2 on monocyte	7	119847090
ebi-a-GCST90002017	rs182030050	CCR2 on monocyte	7	12999190
ebi-a-GCST90002017	rs56382705	CCR2 on monocyte	8	26837933
ebi-a-GCST90002017	rs147002093	CCR2 on monocyte	8	146201448
ebi-a-GCST90002017	rs17314600	CCR2 on monocyte	10	109456317
ebi-a-GCST90002017	rs17657502	CCR2 on monocyte	10	18227452
ebi-a-GCST90002017	rs117908977	CCR2 on monocyte	11	124197747

ebi-a-GCST90002017	rs141436692	CCR2 on monocyte	14	102948100
ebi-a-GCST90002017	rs117265129	CCR2 on monocyte	15	92575288
ebi-a-GCST90002017	rs76532688	CCR2 on monocyte	16	61115686
ebi-a-GCST90002017	rs7254242	CCR2 on monocyte	19	16268666
ebi-a-GCST90002018	rs139407311	CCR2 on granulocy	1	182527138
ebi-a-GCST90002018	rs55670480	CCR2 on granulocy	1	69727128
ebi-a-GCST90002018	rs112157475	CCR2 on granulocy	2	195118976
ebi-a-GCST90002018	rs10200651	CCR2 on granulocy	2	241986377
ebi-a-GCST90002018	rs13024798	CCR2 on granulocy	2	3028279
ebi-a-GCST90002018	rs7611080	CCR2 on granulocy	3	184120723
ebi-a-GCST90002018	rs184966737	CCR2 on granulocy	3	35282215
ebi-a-GCST90002018	rs1493142	CCR2 on granulocy	4	28732101
ebi-a-GCST90002018	rs17344000	CCR2 on granulocy	4	129589625
ebi-a-GCST90002018	rs33191	CCR2 on granulocy	5	158103815
ebi-a-GCST90002018	rs74893841	CCR2 on granulocy	6	151948668
ebi-a-GCST90002018	rs445	CCR2 on granulocy	7	92408370
ebi-a-GCST90002018	rs42031	CCR2 on granulocy	7	92237396
ebi-a-GCST90002018	rs114008100	CCR2 on granulocy	7	151516164
ebi-a-GCST90002018	rs78538699	CCR2 on granulocy	9	31399149
ebi-a-GCST90002018	rs7033141	CCR2 on granulocy	9	72226015
ebi-a-GCST90002018	rs11637579	CCR2 on granulocy	15	52729171
ebi-a-GCST90002018	rs11863231	CCR2 on granulocy	16	78366298
ebi-a-GCST90002018	rs1999663	CCR2 on granulocy	20	31897554
ebi-a-GCST90002019	rs10917759	CD14 on Mo MDS(	1	163435598
ebi-a-GCST90002019	rs113721058	CD14 on Mo MDS(	1	228597022
ebi-a-GCST90002019	rs142486612	CD14 on Mo MDS(	1	164824389
ebi-a-GCST90002019	rs6660065	CD14 on Mo MDS(	1	241159848
ebi-a-GCST90002019	rs6818126	CD14 on Mo MDS(	4	131748538
ebi-a-GCST90002019	rs6891742	CD14 on Mo MDS(	5	163345711
ebi-a-GCST90002019	rs72798896	CD14 on Mo MDS(	5	139978645
ebi-a-GCST90002019	rs6453485	CD14 on Mo MDS(	5	79151752
ebi-a-GCST90002019	rs75305369	CD14 on Mo MDS(	6	3991198
ebi-a-GCST90002019	rs138194426	CD14 on Mo MDS(	6	124668998
ebi-a-GCST90002019	rs17429020	CD14 on Mo MDS(	7	132316317
ebi-a-GCST90002019	rs3823806	CD14 on Mo MDS(	7	77670637
ebi-a-GCST90002019	rs987435	CD14 on Mo MDS(	7	78599583
ebi-a-GCST90002019	rs62542759	CD14 on Mo MDS(	9	26635464
ebi-a-GCST90002019	rs111912592	CD14 on Mo MDS(	10	10790518
ebi-a-GCST90002019	rs10883096	CD14 on Mo MDS(	10	100203516
ebi-a-GCST90002019	rs1520811	CD14 on Mo MDS(	12	117765067
ebi-a-GCST90002019	rs1174651	CD14 on Mo MDS(	12	58632584
ebi-a-GCST90002019	rs7999168	CD14 on Mo MDS(	13	46680264
ebi-a-GCST90002019	rs11848927	CD14 on Mo MDS(	14	49309905
ebi-a-GCST90002019	rs117556192	CD14 on Mo MDS(	16	49667299
ebi-a-GCST90002019	rs62036290	CD14 on Mo MDS(	16	25854816
ebi-a-GCST90002019	rs11150478	CD14 on Mo MDS(	16	82592211
ebi-a-GCST90002019	rs6048146	CD14 on Mo MDS(	20	22488267
ebi-a-GCST90002019	rs138125418	CD14 on Mo MDS(	20	45988729

ebi-a-GCST90002020	rs7539387	CD14 on CD33br H	1	102040284
ebi-a-GCST90002020	rs4657282	CD14 on CD33br H	1	163440621
ebi-a-GCST90002020	rs76728302	CD14 on CD33br H	2	47849107
ebi-a-GCST90002020	rs1976058	CD14 on CD33br H	2	111792734
ebi-a-GCST90002020	rs4673986	CD14 on CD33br H	2	216180100
ebi-a-GCST90002020	rs13111223	CD14 on CD33br H	4	188692178
ebi-a-GCST90002020	rs182186600	CD14 on CD33br H	4	109674936
ebi-a-GCST90002020	rs149153393	CD14 on CD33br H	5	222611
ebi-a-GCST90002020	rs2230186	CD14 on CD33br H	7	134645319
ebi-a-GCST90002020	rs3114703	CD14 on CD33br H	7	22456928
ebi-a-GCST90002020	rs74896244	CD14 on CD33br H	8	25036512
ebi-a-GCST90002020	rs11007733	CD14 on CD33br H	10	30059784
ebi-a-GCST90002020	rs11003240	CD14 on CD33br H	10	54641214
ebi-a-GCST90002020	rs74785984	CD14 on CD33br H	12	22230399
ebi-a-GCST90002020	rs9596613	CD14 on CD33br H	13	52492525
ebi-a-GCST90002020	rs79880157	CD14 on CD33br H	13	84038649
ebi-a-GCST90002020	rs11624046	CD14 on CD33br H	14	57370548
ebi-a-GCST90002020	rs11117273	CD14 on CD33br H	16	87483838
ebi-a-GCST90002020	rs2726036	CD14 on CD33br H	16	28347140
ebi-a-GCST90002020	rs73014922	CD14 on CD33br H	19	17916591
ebi-a-GCST90002021	rs7539387	CD14 on CD33dim	1	102040284
ebi-a-GCST90002021	rs1801274	CD14 on CD33dim	1	161479745
ebi-a-GCST90002021	rs74453600	CD14 on CD33dim	1	87319317
ebi-a-GCST90002021	rs4657282	CD14 on CD33dim	1	163440621
ebi-a-GCST90002021	rs11687130	CD14 on CD33dim	2	56343677
ebi-a-GCST90002021	rs72664120	CD14 on CD33dim	4	105646485
ebi-a-GCST90002021	rs57599368	CD14 on CD33dim	5	139992113
ebi-a-GCST90002021	rs116871970	CD14 on CD33dim	6	115908744
ebi-a-GCST90002021	rs6459426	CD14 on CD33dim	6	15911190
ebi-a-GCST90002021	rs117907831	CD14 on CD33dim	7	77830890
ebi-a-GCST90002021	rs143380208	CD14 on CD33dim	7	157541950
ebi-a-GCST90002021	rs77166057	CD14 on CD33dim	8	37782883
ebi-a-GCST90002021	rs7125721	CD14 on CD33dim	11	108966171
ebi-a-GCST90002021	rs491465	CD14 on CD33dim	11	64830949
ebi-a-GCST90002021	rs9568631	CD14 on CD33dim	13	52113042
ebi-a-GCST90002021	rs4555095	CD14 on CD33dim	15	46669609
ebi-a-GCST90002021	rs67814263	CD14 on CD33dim	16	26227878
ebi-a-GCST90002021	rs6087047	CD14 on CD33dim	20	9871771
ebi-a-GCST90002021	rs134558	CD14 on CD33dim	22	29457622
ebi-a-GCST90002022	rs11587381	CD4 on CD4+	1	226377250
ebi-a-GCST90002022	rs6659442	CD4 on CD4+	1	244370950
ebi-a-GCST90002022	rs10200667	CD4 on CD4+	2	153552714
ebi-a-GCST90002022	rs149025564	CD4 on CD4+	3	150265658
ebi-a-GCST90002022	rs9827923	CD4 on CD4+	3	154165486
ebi-a-GCST90002022	rs144911886	CD4 on CD4+	4	190091645
ebi-a-GCST90002022	rs9992090	CD4 on CD4+	4	150982870
ebi-a-GCST90002022	rs74604607	CD4 on CD4+	5	52143523
ebi-a-GCST90002022	rs3129267	CD4 on CD4+	6	33098896

ebi-a-GCST90002022	rs4959028	CD4 on CD4+	6	32383138
ebi-a-GCST90002022	rs28584364	CD4 on CD4+	6	32512048
ebi-a-GCST90002022	rs2960460	CD4 on CD4+	7	77638859
ebi-a-GCST90002022	rs118120903	CD4 on CD4+	8	30276043
ebi-a-GCST90002022	rs10970000	CD4 on CD4+	9	3106474
ebi-a-GCST90002022	rs4573693	CD4 on CD4+	11	58699767
ebi-a-GCST90002022	rs4766578	CD4 on CD4+	12	111904371
ebi-a-GCST90002022	rs11615628	CD4 on CD4+	12	6903631
ebi-a-GCST90002022	rs6580816	CD4 on CD4+	12	51622418
ebi-a-GCST90002022	rs61951572	CD4 on CD4+	13	70982795
ebi-a-GCST90002022	rs11628611	CD4 on CD4+	14	65828525
ebi-a-GCST90002022	rs62027494	CD4 on CD4+	15	94203188
ebi-a-GCST90002022	rs1833936	CD4 on CD4+	16	65817386
ebi-a-GCST90002022	rs73965521	CD4 on CD4+	18	63972550
ebi-a-GCST90002022	rs6041696	CD4 on CD4+	20	188030
ebi-a-GCST90002022	rs6068656	CD4 on CD4+	20	52386803
ebi-a-GCST90002023	rs11584966	CD4 on CM CD4 +	1	226382922
ebi-a-GCST90002023	rs181188506	CD4 on CM CD4 +	2	74231575
ebi-a-GCST90002023	rs72825704	CD4 on CM CD4 +	2	99239164
ebi-a-GCST90002023	rs9992090	CD4 on CM CD4 +	4	150982870
ebi-a-GCST90002023	rs3129267	CD4 on CM CD4 +	6	33098896
ebi-a-GCST90002023	rs28584364	CD4 on CM CD4 +	6	32512048
ebi-a-GCST90002023	rs4495271	CD4 on CM CD4 +	6	53049236
ebi-a-GCST90002023	rs4487854	CD4 on CM CD4 +	9	137844723
ebi-a-GCST90002023	rs7908436	CD4 on CM CD4 +	10	106139081
ebi-a-GCST90002023	rs11615628	CD4 on CM CD4 +	12	6903631
ebi-a-GCST90002023	rs6580816	CD4 on CM CD4 +	12	51622418
ebi-a-GCST90002023	rs4766578	CD4 on CM CD4 +	12	111904371
ebi-a-GCST90002023	rs547243038	CD4 on CM CD4 +	13	52619467
ebi-a-GCST90002023	rs8009128	CD4 on CM CD4 +	14	33560928
ebi-a-GCST90002023	rs7145500	CD4 on CM CD4 +	14	65884212
ebi-a-GCST90002023	rs368550319	CD4 on CM CD4 +	14	23466191
ebi-a-GCST90002023	rs62027494	CD4 on CM CD4 +	15	94203188
ebi-a-GCST90002023	rs6063990	CD4 on CM CD4 +	20	52386784
ebi-a-GCST90002024	rs139225197	CD4 on naive CD4·	2	11978499
ebi-a-GCST90002024	rs1261235	CD4 on naive CD4·	2	79183004
ebi-a-GCST90002024	rs9833698	CD4 on naive CD4·	3	154166611
ebi-a-GCST90002024	rs139306421	CD4 on naive CD4·	6	109298437
ebi-a-GCST90002024	rs2844608	CD4 on naive CD4·	6	31247414
ebi-a-GCST90002024	rs12204644	CD4 on naive CD4·	6	91794933
ebi-a-GCST90002024	rs17205675	CD4 on naive CD4·	6	32637489
ebi-a-GCST90002024	rs7457754	CD4 on naive CD4·	7	28128002
ebi-a-GCST90002024	rs72702672	CD4 on naive CD4·	9	12803588
ebi-a-GCST90002024	rs12000134	CD4 on naive CD4·	9	3108492
ebi-a-GCST90002024	rs141120003	CD4 on naive CD4·	10	36139903
ebi-a-GCST90002024	rs10749528	CD4 on naive CD4·	10	87460984
ebi-a-GCST90002024	rs10876154	CD4 on naive CD4·	12	51607890
ebi-a-GCST90002024	rs951818	CD4 on naive CD4·	12	6896055

ebi-a-GCST90002024	rs28920471	CD4 on naive CD4-	12	6915160
ebi-a-GCST90002024	rs3184504	CD4 on naive CD4-	12	111884608
ebi-a-GCST90002024	rs61951572	CD4 on naive CD4-	13	70982795
ebi-a-GCST90002024	rs11158593	CD4 on naive CD4-	14	65859984
ebi-a-GCST90002024	rs75181293	CD4 on naive CD4-	19	1068808
ebi-a-GCST90002024	rs6063990	CD4 on naive CD4-	20	52386784
ebi-a-GCST90002025	rs10927163	CD4 on EM CD4+	1	244377870
ebi-a-GCST90002025	rs72712872	CD4 on EM CD4+	1	160982770
ebi-a-GCST90002025	rs12052946	CD4 on EM CD4+	2	153567946
ebi-a-GCST90002025	rs61741426	CD4 on EM CD4+	2	137852550
ebi-a-GCST90002025	rs78350981	CD4 on EM CD4+	3	102203257
ebi-a-GCST90002025	rs9992090	CD4 on EM CD4+	4	150982870
ebi-a-GCST90002025	rs530397075	CD4 on EM CD4+	4	63964498
ebi-a-GCST90002025	rs60970806	CD4 on EM CD4+	5	152607414
ebi-a-GCST90002025	rs9264532	CD4 on EM CD4+	6	31234381
ebi-a-GCST90002025	rs9272242	CD4 on EM CD4+	6	32602790
ebi-a-GCST90002025	rs3129267	CD4 on EM CD4+	6	33098896
ebi-a-GCST90002025	rs34560282	CD4 on EM CD4+	6	69075534
ebi-a-GCST90002025	rs1569213	CD4 on EM CD4+	8	19870735
ebi-a-GCST90002025	rs118120903	CD4 on EM CD4+	8	30276043
ebi-a-GCST90002025	rs4487854	CD4 on EM CD4+	9	137844723
ebi-a-GCST90002025	rs10122230	CD4 on EM CD4+	9	37686238
ebi-a-GCST90002025	rs4878712	CD4 on EM CD4+	9	37654257
ebi-a-GCST90002025	rs4910721	CD4 on EM CD4+	11	5194616
ebi-a-GCST90002025	rs7137828	CD4 on EM CD4+	12	111932800
ebi-a-GCST90002025	rs11615628	CD4 on EM CD4+	12	6903631
ebi-a-GCST90002025	rs12870415	CD4 on EM CD4+	13	105791075
ebi-a-GCST90002025	rs12147140	CD4 on EM CD4+	14	107282437
ebi-a-GCST90002025	rs368550319	CD4 on EM CD4+	14	23466191
ebi-a-GCST90002026	rs9439747	CD4 on TD CD4+	1	19111912
ebi-a-GCST90002026	rs828892	CD4 on TD CD4+	2	74390595
ebi-a-GCST90002026	rs13024840	CD4 on TD CD4+	2	38895920
ebi-a-GCST90002026	rs35947652	CD4 on TD CD4+	3	129318594
ebi-a-GCST90002026	rs187985913	CD4 on TD CD4+	4	152837976
ebi-a-GCST90002026	rs6853862	CD4 on TD CD4+	4	32955410
ebi-a-GCST90002026	rs149775976	CD4 on TD CD4+	4	108032717
ebi-a-GCST90002026	rs10463714	CD4 on TD CD4+	5	97797651
ebi-a-GCST90002026	rs78069551	CD4 on TD CD4+	6	169334009
ebi-a-GCST90002026	rs9480502	CD4 on TD CD4+	6	150949998
ebi-a-GCST90002026	rs424001	CD4 on TD CD4+	6	13715997
ebi-a-GCST90002026	rs719498	CD4 on TD CD4+	7	144876947
ebi-a-GCST90002026	rs78863652	CD4 on TD CD4+	7	31168474
ebi-a-GCST90002026	rs79658047	CD4 on TD CD4+	7	46453848
ebi-a-GCST90002026	rs10229549	CD4 on TD CD4+	7	14668100
ebi-a-GCST90002026	rs111931508	CD4 on TD CD4+	8	9938243
ebi-a-GCST90002026	rs146861616	CD4 on TD CD4+	8	8216308
ebi-a-GCST90002026	rs72640514	CD4 on TD CD4+	8	49593467
ebi-a-GCST90002026	rs11033545	CD4 on TD CD4+	11	36327582

ebi-a-GCST90002026	rs4414181	CD4 on TD CD4+	11	90950783
ebi-a-GCST90002026	rs11026566	CD4 on TD CD4+	11	22472189
ebi-a-GCST90002026	rs4771059	CD4 on TD CD4+	13	27588660
ebi-a-GCST90002026	rs713431	CD4 on TD CD4+	17	39138045
ebi-a-GCST90002026	rs183650419	CD4 on TD CD4+	21	28265035
ebi-a-GCST90002026	rs74432937	CD4 on TD CD4+	21	28786756
ebi-a-GCST90002026	rs9306514	CD4 on TD CD4+	22	46837114
ebi-a-GCST90002027	rs11584966	CD4 on CD45RA+	1	226382922
ebi-a-GCST90002027	rs72712872	CD4 on CD45RA+	1	160982770
ebi-a-GCST90002027	rs11807869	CD4 on CD45RA+	1	244370098
ebi-a-GCST90002027	rs184697850	CD4 on CD45RA+	2	80753761
ebi-a-GCST90002027	rs16831529	CD4 on CD45RA+	2	153519768
ebi-a-GCST90002027	rs56253058	CD4 on CD45RA+	3	5485591
ebi-a-GCST90002027	rs530397075	CD4 on CD45RA+	4	63964498
ebi-a-GCST90002027	rs9992090	CD4 on CD45RA+	4	150982870
ebi-a-GCST90002027	rs144911886	CD4 on CD45RA+	4	190091645
ebi-a-GCST90002027	rs74604607	CD4 on CD45RA+	5	52143523
ebi-a-GCST90002027	rs28584364	CD4 on CD45RA+	6	32512048
ebi-a-GCST90002027	rs3129267	CD4 on CD45RA+	6	33098896
ebi-a-GCST90002027	rs2960460	CD4 on CD45RA+	7	77638859
ebi-a-GCST90002027	rs118120903	CD4 on CD45RA+	8	30276043
ebi-a-GCST90002027	rs7827166	CD4 on CD45RA+	8	15773120
ebi-a-GCST90002027	rs4487854	CD4 on CD45RA+	9	137844723
ebi-a-GCST90002027	rs10115790	CD4 on CD45RA+	9	7345304
ebi-a-GCST90002027	rs147954895	CD4 on CD45RA+	10	3259982
ebi-a-GCST90002027	rs10501464	CD4 on CD45RA+	11	80053454
ebi-a-GCST90002027	rs6580816	CD4 on CD45RA+	12	51622418
ebi-a-GCST90002027	rs11615628	CD4 on CD45RA+	12	6903631
ebi-a-GCST90002027	rs11569383	CD4 on CD45RA+	12	6557758
ebi-a-GCST90002027	rs4766578	CD4 on CD45RA+	12	111904371
ebi-a-GCST90002027	rs61951572	CD4 on CD45RA+	13	70982795
ebi-a-GCST90002027	rs547243038	CD4 on CD45RA+	13	52619467
ebi-a-GCST90002027	rs368550319	CD4 on CD45RA+	14	23466191
ebi-a-GCST90002027	rs8009128	CD4 on CD45RA+	14	33560928
ebi-a-GCST90002027	rs8011224	CD4 on CD45RA+	14	65969670
ebi-a-GCST90002027	rs62027494	CD4 on CD45RA+	15	94203188
ebi-a-GCST90002027	rs11073860	CD4 on CD45RA+	15	89934406
ebi-a-GCST90002027	rs9939476	CD4 on CD45RA+	16	65846028
ebi-a-GCST90002028	rs2179634	CD19 on B cell	1	208063067
ebi-a-GCST90002028	rs6698444	CD19 on B cell	1	167583141
ebi-a-GCST90002028	rs143306403	CD19 on B cell	2	232217689
ebi-a-GCST90002028	rs73181184	CD19 on B cell	3	178371750
ebi-a-GCST90002028	rs149686480	CD19 on B cell	3	124399196
ebi-a-GCST90002028	rs145412732	CD19 on B cell	4	119168128
ebi-a-GCST90002028	rs17516457	CD19 on B cell	5	131590387
ebi-a-GCST90002028	rs3828914	CD19 on B cell	6	31465819
ebi-a-GCST90002028	rs28383305	CD19 on B cell	6	32586721
ebi-a-GCST90002028	rs571514	CD19 on B cell	7	73769459

ebi-a-GCST90002028	rs2030975	CD19 on B cell	7	105177611
ebi-a-GCST90002028	rs117541942	CD19 on B cell	8	58507470
ebi-a-GCST90002028	rs182569768	CD19 on B cell	8	52764429
ebi-a-GCST90002028	rs2977855	CD19 on B cell	8	41408227
ebi-a-GCST90002028	rs55670656	CD19 on B cell	9	27581489
ebi-a-GCST90002028	rs76929802	CD19 on B cell	11	125505878
ebi-a-GCST90002028	rs1222321	CD19 on B cell	11	30528400
ebi-a-GCST90002028	rs149010460	CD19 on B cell	12	23512909
ebi-a-GCST90002028	rs9543559	CD19 on B cell	13	74865635
ebi-a-GCST90002028	rs117314912	CD19 on B cell	14	92966472
ebi-a-GCST90002028	rs74628013	CD19 on B cell	15	79819451
ebi-a-GCST90002028	rs111853663	CD19 on B cell	16	29181468
ebi-a-GCST90002028	rs2904880	CD19 on B cell	16	28944396
ebi-a-GCST90002028	rs145738076	CD19 on B cell	16	28007096
ebi-a-GCST90002028	rs2284147	CD19 on B cell	19	35765041
ebi-a-GCST90002028	rs910655	CD19 on B cell	20	3787550
ebi-a-GCST90002028	rs2232181	CD19 on B cell	22	31533137
ebi-a-GCST90002029	rs11674407	CD39 on CD39+ C	2	1464530
ebi-a-GCST90002029	rs116646957	CD39 on CD39+ C	2	115975810
ebi-a-GCST90002029	rs13070466	CD39 on CD39+ C	3	87382102
ebi-a-GCST90002029	rs9882438	CD39 on CD39+ C	3	36776413
ebi-a-GCST90002029	rs2120877	CD39 on CD39+ C	3	106178942
ebi-a-GCST90002029	rs111933386	CD39 on CD39+ C	3	119262389
ebi-a-GCST90002029	rs78570145	CD39 on CD39+ C	4	158695975
ebi-a-GCST90002029	rs2167204	CD39 on CD39+ C	4	121827202
ebi-a-GCST90002029	rs78933037	CD39 on CD39+ C	6	1424645
ebi-a-GCST90002029	rs72866625	CD39 on CD39+ C	6	47410742
ebi-a-GCST90002029	rs76983442	CD39 on CD39+ C	7	101186788
ebi-a-GCST90002029	rs118169282	CD39 on CD39+ C	7	52373730
ebi-a-GCST90002029	rs12237839	CD39 on CD39+ C	9	135281367
ebi-a-GCST90002029	rs12415115	CD39 on CD39+ C	10	82712186
ebi-a-GCST90002029	rs10882701	CD39 on CD39+ C	10	97741071
ebi-a-GCST90002029	rs11815047	CD39 on CD39+ C	10	1112149
ebi-a-GCST90002029	rs2901833	CD39 on CD39+ C	10	96998591
ebi-a-GCST90002029	rs7088961	CD39 on CD39+ C	10	97608114
ebi-a-GCST90002029	rs728713	CD39 on CD39+ C	10	105552449
ebi-a-GCST90002029	rs113261188	CD39 on CD39+ C	12	107834932
ebi-a-GCST90002029	rs11107237	CD39 on CD39+ C	12	94282149
ebi-a-GCST90002029	rs8014341	CD39 on CD39+ C	14	89487689
ebi-a-GCST90002029	rs4341854	CD39 on CD39+ C	19	52260567
ebi-a-GCST90002029	rs10422831	CD39 on CD39+ C	19	2019930
ebi-a-GCST90002029	rs8178474	CD39 on CD39+ C	21	34649337
ebi-a-GCST90002030	rs4567963	CD39 on CD39+ ac	2	79257886
ebi-a-GCST90002030	rs66685711	CD39 on CD39+ ac	2	221447582
ebi-a-GCST90002030	rs2171655	CD39 on CD39+ ac	2	127229166
ebi-a-GCST90002030	rs74346764	CD39 on CD39+ ac	3	145230069
ebi-a-GCST90002030	rs74788351	CD39 on CD39+ ac	4	129075134
ebi-a-GCST90002030	rs1528104	CD39 on CD39+ ac	7	137136419

ebi-a-GCST90002030	rs111253358	CD39 on CD39+ ac	10	64441033
ebi-a-GCST90002030	rs145557266	CD39 on CD39+ ac	10	95850055
ebi-a-GCST90002030	rs147121076	CD39 on CD39+ ac	10	95924521
ebi-a-GCST90002030	rs17442418	CD39 on CD39+ ac	10	99777109
ebi-a-GCST90002030	rs113440812	CD39 on CD39+ ac	10	100221844
ebi-a-GCST90002030	rs1410600	CD39 on CD39+ ac	10	97070694
ebi-a-GCST90002030	rs12778797	CD39 on CD39+ ac	10	96998688
ebi-a-GCST90002030	rs4918960	CD39 on CD39+ ac	10	97472570
ebi-a-GCST90002030	rs114844216	CD39 on CD39+ ac	10	98158952
ebi-a-GCST90002030	rs61886297	CD39 on CD39+ ac	10	95830221
ebi-a-GCST90002030	rs12252311	CD39 on CD39+ ac	10	79971872
ebi-a-GCST90002030	rs61859411	CD39 on CD39+ ac	10	82519788
ebi-a-GCST90002030	rs10882701	CD39 on CD39+ ac	10	97741071
ebi-a-GCST90002030	rs11653180	CD39 on CD39+ ac	17	3689318
ebi-a-GCST90002030	rs17782405	CD39 on CD39+ ac	17	31751232
ebi-a-GCST90002030	rs308035	CD39 on CD39+ ac	19	3099555
ebi-a-GCST90002030	rs77176206	CD39 on CD39+ ac	19	35278942
ebi-a-GCST90002031	rs112736587	CD39 on CD39+ se	1	68140344
ebi-a-GCST90002031	rs76866779	CD39 on CD39+ se	1	115344429
ebi-a-GCST90002031	rs62142767	CD39 on CD39+ se	2	60359556
ebi-a-GCST90002031	rs116180260	CD39 on CD39+ se	2	127180882
ebi-a-GCST90002031	rs74788351	CD39 on CD39+ se	4	129075134
ebi-a-GCST90002031	rs58294041	CD39 on CD39+ se	4	177219904
ebi-a-GCST90002031	rs12778797	CD39 on CD39+ se	10	96998688
ebi-a-GCST90002031	rs141018090	CD39 on CD39+ se	10	98169221
ebi-a-GCST90002031	rs10882701	CD39 on CD39+ se	10	97741071
ebi-a-GCST90002031	rs10882655	CD39 on CD39+ se	10	97474371
ebi-a-GCST90002031	rs10886720	CD39 on CD39+ se	10	122355414
ebi-a-GCST90002031	rs557334	CD39 on CD39+ se	10	99765397
ebi-a-GCST90002031	rs1410600	CD39 on CD39+ se	10	97070694
ebi-a-GCST90002031	rs10882793	CD39 on CD39+ se	10	98217526
ebi-a-GCST90002031	rs10788360	CD39 on CD39+ se	10	86499074
ebi-a-GCST90002031	rs10998783	CD39 on CD39+ se	10	71204033
ebi-a-GCST90002031	rs61858556	CD39 on CD39+ se	10	98340476
ebi-a-GCST90002031	rs72896749	CD39 on CD39+ se	11	45659591
ebi-a-GCST90002031	rs10501444	CD39 on CD39+ se	11	79137378
ebi-a-GCST90002031	rs190744827	CD39 on CD39+ se	14	55569054
ebi-a-GCST90002031	rs80146165	CD39 on CD39+ se	15	68273646
ebi-a-GCST90002031	rs17782405	CD39 on CD39+ se	17	31751232
ebi-a-GCST90002031	rs7259646	CD39 on CD39+ se	19	53873606
ebi-a-GCST90002031	rs2424506	CD39 on CD39+ se	20	23036651
ebi-a-GCST90002032	rs79502391	CD39 on CD39+ C	2	75610667
ebi-a-GCST90002032	rs62138982	CD39 on CD39+ C	2	65095286
ebi-a-GCST90002032	rs2886870	CD39 on CD39+ C	3	192616700
ebi-a-GCST90002032	rs7646534	CD39 on CD39+ C	3	62850140
ebi-a-GCST90002032	rs11929716	CD39 on CD39+ C	4	28719206
ebi-a-GCST90002032	rs6815832	CD39 on CD39+ C	4	177211552
ebi-a-GCST90002032	rs191035403	CD39 on CD39+ C	5	178729984

ebi-a-GCST90002032	rs28687532	CD39 on CD39+ C	7	137130450
ebi-a-GCST90002032	rs147765250	CD39 on CD39+ C	7	153196368
ebi-a-GCST90002032	rs2045028	CD39 on CD39+ C	8	27922723
ebi-a-GCST90002032	rs12269710	CD39 on CD39+ C	10	100218017
ebi-a-GCST90002032	rs10882701	CD39 on CD39+ C	10	97741071
ebi-a-GCST90002032	rs10883000	CD39 on CD39+ C	10	99565268
ebi-a-GCST90002032	rs10882655	CD39 on CD39+ C	10	97474371
ebi-a-GCST90002032	rs2901833	CD39 on CD39+ C	10	96998591
ebi-a-GCST90002032	rs1410600	CD39 on CD39+ C	10	97070694
ebi-a-GCST90002032	rs11188782	CD39 on CD39+ C	10	98236565
ebi-a-GCST90002032	rs148810393	CD39 on CD39+ C	12	39003459
ebi-a-GCST90002032	rs111290040	CD39 on CD39+ C	16	63795817
ebi-a-GCST90002032	rs12603021	CD39 on CD39+ C	17	3202903
ebi-a-GCST90002032	rs308035	CD39 on CD39+ C	19	3099555
ebi-a-GCST90002032	rs55760306	CD39 on CD39+ C	22	49885801
ebi-a-GCST90002033	rs2070901	CD39 on granulocy	1	161185058
ebi-a-GCST90002033	rs146832619	CD39 on granulocy	1	182145692
ebi-a-GCST90002033	rs114593719	CD39 on granulocy	1	120146042
ebi-a-GCST90002033	rs75826267	CD39 on granulocy	2	25151545
ebi-a-GCST90002033	rs116660215	CD39 on granulocy	2	17527024
ebi-a-GCST90002033	rs11719519	CD39 on granulocy	3	186104558
ebi-a-GCST90002033	rs7630948	CD39 on granulocy	3	30732534
ebi-a-GCST90002033	rs61286411	CD39 on granulocy	3	25586839
ebi-a-GCST90002033	rs11720783	CD39 on granulocy	3	159259315
ebi-a-GCST90002033	rs116817680	CD39 on granulocy	4	20867272
ebi-a-GCST90002033	rs28791580	CD39 on granulocy	4	60032561
ebi-a-GCST90002033	rs867924	CD39 on granulocy	5	143087656
ebi-a-GCST90002033	rs351433	CD39 on granulocy	5	68414152
ebi-a-GCST90002033	rs115457551	CD39 on granulocy	5	80156939
ebi-a-GCST90002033	rs2817775	CD39 on granulocy	6	110840020
ebi-a-GCST90002033	rs445	CD39 on granulocy	7	92408370
ebi-a-GCST90002033	rs188989638	CD39 on granulocy	7	51951285
ebi-a-GCST90002033	rs11766311	CD39 on granulocy	7	77134132
ebi-a-GCST90002033	rs42031	CD39 on granulocy	7	92237396
ebi-a-GCST90002033	rs12553288	CD39 on granulocy	9	105037632
ebi-a-GCST90002033	rs7073937	CD39 on granulocy	10	97469109
ebi-a-GCST90002033	rs72892358	CD39 on granulocy	11	31876312
ebi-a-GCST90002033	rs506053	CD39 on granulocy	11	59829804
ebi-a-GCST90002033	rs35247100	CD39 on granulocy	15	99433803
ebi-a-GCST90002033	rs7177153	CD39 on granulocy	15	53764662
ebi-a-GCST90002033	rs139849385	CD39 on granulocy	16	4693648
ebi-a-GCST90002033	rs1941617	CD39 on granulocy	18	22584904
ebi-a-GCST90002033	rs371927	CD39 on granulocy	20	43263036
ebi-a-GCST90002034	rs75935102	CD39 on monocyte	1	44477960
ebi-a-GCST90002034	rs138028805	CD39 on monocyte	1	38546640
ebi-a-GCST90002034	rs115897087	CD39 on monocyte	1	43450801
ebi-a-GCST90002034	rs181076711	CD39 on monocyte	1	105207158
ebi-a-GCST90002034	rs3213851	CD39 on monocyte	2	38812656

ebi-a-GCST90002034	rs72867988	CD39 on monocyte	2	153968014
ebi-a-GCST90002034	rs11896747	CD39 on monocyte	2	43764398
ebi-a-GCST90002034	rs115014362	CD39 on monocyte	3	20181904
ebi-a-GCST90002034	rs34992898	CD39 on monocyte	4	77697071
ebi-a-GCST90002034	rs35764061	CD39 on monocyte	7	73285441
ebi-a-GCST90002034	rs117286316	CD39 on monocyte	7	110522802
ebi-a-GCST90002034	rs28810746	CD39 on monocyte	7	73054573
ebi-a-GCST90002034	rs73113464	CD39 on monocyte	7	53205505
ebi-a-GCST90002034	rs11592078	CD39 on monocyte	10	30653567
ebi-a-GCST90002034	rs77346511	CD39 on monocyte	11	5268498
ebi-a-GCST90002034	rs67652251	CD39 on monocyte	13	114957688
ebi-a-GCST90002034	rs8050653	CD39 on monocyte	16	82300059
ebi-a-GCST90002034	rs247062	CD39 on monocyte	16	57888792
ebi-a-GCST90002034	rs2337147	CD39 on monocyte	18	46483810
ebi-a-GCST90002034	rs77687616	CD39 on monocyte	20	54392972
ebi-a-GCST90002034	rs6031492	CD39 on monocyte	20	42897549
ebi-a-GCST90002034	rs4050551	CD39 on monocyte	22	22991747
ebi-a-GCST90002035	rs71632979	CD80 on myeloid D	1	161526397
ebi-a-GCST90002035	rs139795227	CD80 on myeloid D	1	92842367
ebi-a-GCST90002035	rs1146465	CD80 on myeloid D	1	95401613
ebi-a-GCST90002035	rs71639910	CD80 on myeloid D	1	161937774
ebi-a-GCST90002035	rs79015439	CD80 on myeloid D	1	94803124
ebi-a-GCST90002035	rs76064946	CD80 on myeloid D	1	93926697
ebi-a-GCST90002035	rs34687967	CD80 on myeloid D	2	155359340
ebi-a-GCST90002035	rs6787493	CD80 on myeloid D	3	52528000
ebi-a-GCST90002035	rs79299158	CD80 on myeloid D	3	194861480
ebi-a-GCST90002035	rs12640679	CD80 on myeloid D	4	155764038
ebi-a-GCST90002035	rs7757423	CD80 on myeloid D	6	149222646
ebi-a-GCST90002035	rs74562173	CD80 on myeloid D	6	87279281
ebi-a-GCST90002035	rs79470163	CD80 on myeloid D	6	43733897
ebi-a-GCST90002035	rs2325259	CD80 on myeloid D	6	90850164
ebi-a-GCST90002035	rs2052324	CD80 on myeloid D	7	35419263
ebi-a-GCST90002035	rs35291101	CD80 on myeloid D	8	4443670
ebi-a-GCST90002035	rs9650736	CD80 on myeloid D	9	121270755
ebi-a-GCST90002035	rs651398	CD80 on myeloid D	10	80446485
ebi-a-GCST90002035	rs329521	CD80 on myeloid D	11	29440261
ebi-a-GCST90002035	rs77891122	CD80 on myeloid D	11	132943074
ebi-a-GCST90002035	rs11178308	CD80 on myeloid D	12	70975329
ebi-a-GCST90002035	rs111689986	CD80 on myeloid D	12	51535753
ebi-a-GCST90002035	rs9524710	CD80 on myeloid D	13	95612174
ebi-a-GCST90002035	rs146503240	CD80 on myeloid D	17	6496912
ebi-a-GCST90002035	rs55752165	CD80 on myeloid D	19	17830975
ebi-a-GCST90002035	rs12609290	CD80 on myeloid D	19	17910564
ebi-a-GCST90002035	rs398462	CD80 on myeloid D	19	5996439
ebi-a-GCST90002035	rs6516794	CD80 on myeloid D	21	29285838
ebi-a-GCST90002036	rs972360	CD80 on CD62L+ ı	1	94937670
ebi-a-GCST90002036	rs139795227	CD80 on CD62L+ ı	1	92842367
ebi-a-GCST90002036	rs1146465	CD80 on CD62L+ ı	1	95401613

ebi-a-GCST90002036	rs149094296	CD80 on CD62L+ ı	3	128701091
ebi-a-GCST90002036	rs145983670	CD80 on CD62L+ ı	3	129775019
ebi-a-GCST90002036	rs9812720	CD80 on CD62L+ ı	3	25085794
ebi-a-GCST90002036	rs142175829	CD80 on CD62L+ ı	3	128313034
ebi-a-GCST90002036	rs9875875	CD80 on CD62L+ ı	3	10560942
ebi-a-GCST90002036	rs6445484	CD80 on CD62L+ ı	3	52503800
ebi-a-GCST90002036	rs116671753	CD80 on CD62L+ ı	4	138723841
ebi-a-GCST90002036	rs79470163	CD80 on CD62L+ ı	6	43733897
ebi-a-GCST90002036	rs7757423	CD80 on CD62L+ ı	6	149222646
ebi-a-GCST90002036	rs12199079	CD80 on CD62L+ ı	6	90852258
ebi-a-GCST90002036	rs78587782	CD80 on CD62L+ ı	8	16839699
ebi-a-GCST90002036	rs12237829	CD80 on CD62L+ ı	9	25815735
ebi-a-GCST90002036	rs10733750	CD80 on CD62L+ ı	9	96630555
ebi-a-GCST90002036	rs145212011	CD80 on CD62L+ ı	10	55883783
ebi-a-GCST90002036	rs35142187	CD80 on CD62L+ ı	10	90553571
ebi-a-GCST90002036	rs77891122	CD80 on CD62L+ ı	11	132943074
ebi-a-GCST90002036	rs73434317	CD80 on CD62L+ ı	11	26598705
ebi-a-GCST90002036	rs111689986	CD80 on CD62L+ ı	12	51535753
ebi-a-GCST90002036	rs11178308	CD80 on CD62L+ ı	12	70975329
ebi-a-GCST90002036	rs9524710	CD80 on CD62L+ ı	13	95612174
ebi-a-GCST90002036	rs143918998	CD80 on CD62L+ ı	15	67269845
ebi-a-GCST90002036	rs146503240	CD80 on CD62L+ ı	17	6496912
ebi-a-GCST90002036	rs398462	CD80 on CD62L+ ı	19	5996439
ebi-a-GCST90002036	rs10416809	CD80 on CD62L+ ı	19	1252311
ebi-a-GCST90002036	rs117930014	CD80 on CD62L+ ı	20	2609471
ebi-a-GCST90002037	rs78700726	CD80 on plasmacyt	1	59673110
ebi-a-GCST90002037	rs79213920	CD80 on plasmacyt	1	225471349
ebi-a-GCST90002037	rs379605	CD80 on plasmacyt	1	30988530
ebi-a-GCST90002037	rs12239268	CD80 on plasmacyt	1	202051197
ebi-a-GCST90002037	rs115568119	CD80 on plasmacyt	1	118721918
ebi-a-GCST90002037	rs7573831	CD80 on plasmacyt	2	38099030
ebi-a-GCST90002037	rs78312719	CD80 on plasmacyt	3	16614400
ebi-a-GCST90002037	rs7658518	CD80 on plasmacyt	4	84160279
ebi-a-GCST90002037	rs6921419	CD80 on plasmacyt	6	48113358
ebi-a-GCST90002037	rs7761641	CD80 on plasmacyt	6	41956475
ebi-a-GCST90002037	rs62447171	CD80 on plasmacyt	7	50309890
ebi-a-GCST90002037	rs7812595	CD80 on plasmacyt	8	135493606
ebi-a-GCST90002037	rs75061768	CD80 on plasmacyt	11	87840236
ebi-a-GCST90002037	rs1878266	CD80 on plasmacyt	11	83552659
ebi-a-GCST90002037	rs2786227	CD80 on plasmacyt	13	35698790
ebi-a-GCST90002037	rs116989551	CD80 on plasmacyt	14	85248677
ebi-a-GCST90002037	rs7185638	CD80 on plasmacyt	16	86741000
ebi-a-GCST90002037	rs34388196	CD80 on plasmacyt	17	28534469
ebi-a-GCST90002037	rs639092	CD80 on plasmacyt	17	76514568
ebi-a-GCST90002037	rs8094829	CD80 on plasmacyt	18	6023963
ebi-a-GCST90002037	rs138318679	CD80 on plasmacyt	21	46094596
ebi-a-GCST90002038	rs78700726	CD80 on CD62L+]	1	59673110
ebi-a-GCST90002038	rs379605	CD80 on CD62L+]	1	30988530

ebi-a-GCST90002038	rs115568119	CD80 on CD62L+]	1	118721918
ebi-a-GCST90002038	rs12239268	CD80 on CD62L+]	1	202051197
ebi-a-GCST90002038	rs79213920	CD80 on CD62L+]	1	225471349
ebi-a-GCST90002038	rs7573831	CD80 on CD62L+]	2	38099030
ebi-a-GCST90002038	rs78312719	CD80 on CD62L+]	3	16614400
ebi-a-GCST90002038	rs7658518	CD80 on CD62L+]	4	84160279
ebi-a-GCST90002038	rs6921419	CD80 on CD62L+]	6	48113358
ebi-a-GCST90002038	rs7761641	CD80 on CD62L+]	6	41956475
ebi-a-GCST90002038	rs144549482	CD80 on CD62L+]	7	113212504
ebi-a-GCST90002038	rs62447171	CD80 on CD62L+]	7	50309890
ebi-a-GCST90002038	rs7812595	CD80 on CD62L+]	8	135493606
ebi-a-GCST90002038	rs75061768	CD80 on CD62L+]	11	87840236
ebi-a-GCST90002038	rs1878266	CD80 on CD62L+]	11	83552659
ebi-a-GCST90002038	rs2786227	CD80 on CD62L+]	13	35698790
ebi-a-GCST90002038	rs7185638	CD80 on CD62L+]	16	86741000
ebi-a-GCST90002038	rs34388196	CD80 on CD62L+]	17	28534469
ebi-a-GCST90002038	rs639092	CD80 on CD62L+]	17	76514568
ebi-a-GCST90002038	rs8094829	CD80 on CD62L+]	18	6023963
ebi-a-GCST90002038	rs138318679	CD80 on CD62L+]	21	46094596
ebi-a-GCST90002039	rs139795227	CD80 on monocyte	1	92842367
ebi-a-GCST90002039	rs141264358	CD80 on monocyte	1	90530314
ebi-a-GCST90002039	rs76064946	CD80 on monocyte	1	93926697
ebi-a-GCST90002039	rs79015439	CD80 on monocyte	1	94803124
ebi-a-GCST90002039	rs114839459	CD80 on monocyte	1	92412978
ebi-a-GCST90002039	rs149889574	CD80 on monocyte	2	195749242
ebi-a-GCST90002039	rs758800	CD80 on monocyte	3	52529266
ebi-a-GCST90002039	rs116371507	CD80 on monocyte	3	52895436
ebi-a-GCST90002039	rs114253672	CD80 on monocyte	3	11967862
ebi-a-GCST90002039	rs73154858	CD80 on monocyte	3	162785650
ebi-a-GCST90002039	rs6764053	CD80 on monocyte	3	194095527
ebi-a-GCST90002039	rs35075155	CD80 on monocyte	3	53273195
ebi-a-GCST90002039	rs7625554	CD80 on monocyte	3	137724122
ebi-a-GCST90002039	rs113543675	CD80 on monocyte	4	190041767
ebi-a-GCST90002039	rs10520274	CD80 on monocyte	4	175130244
ebi-a-GCST90002039	rs4867493	CD80 on monocyte	5	32983457
ebi-a-GCST90002039	rs74425990	CD80 on monocyte	7	17172484
ebi-a-GCST90002039	rs36159865	CD80 on monocyte	7	416852
ebi-a-GCST90002039	rs11764289	CD80 on monocyte	7	47373313
ebi-a-GCST90002039	rs10230431	CD80 on monocyte	7	116586829
ebi-a-GCST90002039	rs13254130	CD80 on monocyte	8	1679058
ebi-a-GCST90002039	rs10857716	CD80 on monocyte	10	135256327
ebi-a-GCST90002039	rs751724	CD80 on monocyte	20	59623127
ebi-a-GCST90002040	rs1886654	CD80 on granulocy	1	236105910
ebi-a-GCST90002040	rs11102883	CD80 on granulocy	1	115612702
ebi-a-GCST90002040	rs10936226	CD80 on granulocy	3	94179989
ebi-a-GCST90002040	rs17745206	CD80 on granulocy	3	9321015
ebi-a-GCST90002040	rs148627425	CD80 on granulocy	3	62649750
ebi-a-GCST90002040	rs1489849	CD80 on granulocy	3	79404844

ebi-a-GCST90002040	rs116690299	CD80 on granulocy	3	16516486
ebi-a-GCST90002040	rs115469709	CD80 on granulocy	4	64778416
ebi-a-GCST90002040	rs12507081	CD80 on granulocy	4	153889606
ebi-a-GCST90002040	rs3012550	CD80 on granulocy	6	74567610
ebi-a-GCST90002040	rs319333	CD80 on granulocy	7	95543624
ebi-a-GCST90002040	rs445	CD80 on granulocy	7	92408370
ebi-a-GCST90002040	rs12675298	CD80 on granulocy	8	6828813
ebi-a-GCST90002040	rs73327307	CD80 on granulocy	8	111918474
ebi-a-GCST90002040	rs78322649	CD80 on granulocy	8	6762655
ebi-a-GCST90002040	rs2981398	CD80 on granulocy	8	6801712
ebi-a-GCST90002040	rs138383232	CD80 on granulocy	9	86761155
ebi-a-GCST90002040	rs1018850	CD80 on granulocy	9	108335541
ebi-a-GCST90002040	rs2801527	CD80 on granulocy	10	90904076
ebi-a-GCST90002040	rs186823180	CD80 on granulocy	11	22731651
ebi-a-GCST90002040	rs7924850	CD80 on granulocy	11	127055116
ebi-a-GCST90002040	rs3825097	CD80 on granulocy	12	122804034
ebi-a-GCST90002040	rs17481181	CD80 on granulocy	12	24172546
ebi-a-GCST90002040	rs79603423	CD80 on granulocy	12	113919973
ebi-a-GCST90002040	rs12585407	CD80 on granulocy	13	23667413
ebi-a-GCST90002040	rs10150976	CD80 on granulocy	14	107005926
ebi-a-GCST90002040	rs111503035	CD80 on granulocy	16	60468825
ebi-a-GCST90002040	rs7350970	CD80 on granulocy	17	67933411
ebi-a-GCST90002040	rs62102263	CD80 on granulocy	18	46540942
ebi-a-GCST90002040	rs56161266	CD80 on granulocy	19	58816642
ebi-a-GCST90002040	rs142529172	CD80 on granulocy	19	4814248
ebi-a-GCST90002040	rs1023388	CD80 on granulocy	21	19717241
ebi-a-GCST90002040	rs117718780	CD80 on granulocy	22	28335603
ebi-a-GCST90002041	rs113430136	CD45 on lymphocy	1	204258208
ebi-a-GCST90002041	rs145947216	CD45 on lymphocy	2	38596674
ebi-a-GCST90002041	rs11706901	CD45 on lymphocy	3	10663917
ebi-a-GCST90002041	rs1004687	CD45 on lymphocy	3	103810095
ebi-a-GCST90002041	rs1553663	CD45 on lymphocy	4	55280100
ebi-a-GCST90002041	rs149609625	CD45 on lymphocy	5	53714969
ebi-a-GCST90002041	rs77704135	CD45 on lymphocy	6	26293012
ebi-a-GCST90002041	rs79267062	CD45 on lymphocy	6	26176010
ebi-a-GCST90002041	rs282088	CD45 on lymphocy	6	154260029
ebi-a-GCST90002041	rs117772551	CD45 on lymphocy	10	56075362
ebi-a-GCST90002041	rs184651667	CD45 on lymphocy	10	16847686
ebi-a-GCST90002041	rs74789069	CD45 on lymphocy	12	130989861
ebi-a-GCST90002041	rs9567870	CD45 on lymphocy	13	48040480
ebi-a-GCST90002041	rs9559563	CD45 on lymphocy	13	110000129
ebi-a-GCST90002041	rs368565398	CD45 on lymphocy	16	89952846
ebi-a-GCST90002041	rs138535375	CD45 on lymphocy	18	22152471
ebi-a-GCST90002041	rs1660269	CD45 on lymphocy	18	62549208
ebi-a-GCST90002041	rs56165924	CD45 on lymphocy	19	562874
ebi-a-GCST90002041	rs11696662	CD45 on lymphocy	20	39790435
ebi-a-GCST90002041	rs150795	CD45 on lymphocy	21	43462379
ebi-a-GCST90002042	rs72677097	CD45 on CD33br H	1	61134076

ebi-a-GCST90002042	rs149629736	CD45 on CD33br H	1	204981132
ebi-a-GCST90002042	rs72761150	CD45 on CD33br H	1	245515377
ebi-a-GCST90002042	rs9809259	CD45 on CD33br H	3	146815969
ebi-a-GCST90002042	rs7722937	CD45 on CD33br H	5	79153866
ebi-a-GCST90002042	rs62366654	CD45 on CD33br H	5	104939173
ebi-a-GCST90002042	rs114094080	CD45 on CD33br H	5	26499129
ebi-a-GCST90002042	rs1181554	CD45 on CD33br H	6	106926228
ebi-a-GCST90002042	rs73264490	CD45 on CD33br H	7	1329138
ebi-a-GCST90002042	rs17429361	CD45 on CD33br H	7	27415715
ebi-a-GCST90002042	rs75276852	CD45 on CD33br H	11	97917799
ebi-a-GCST90002042	rs11234542	CD45 on CD33br H	11	85803238
ebi-a-GCST90002042	rs36154713	CD45 on CD33br H	12	109350576
ebi-a-GCST90002042	rs10744547	CD45 on CD33br H	12	1779545
ebi-a-GCST90002042	rs143599997	CD45 on CD33br H	12	130798660
ebi-a-GCST90002042	rs1580715	CD45 on CD33br H	12	103045553
ebi-a-GCST90002042	rs3825436	CD45 on CD33br H	13	99056020
ebi-a-GCST90002042	rs12970861	CD45 on CD33br H	18	66283602
ebi-a-GCST90002043	rs380901	CD45 on CD33br H	1	60846104
ebi-a-GCST90002043	rs6675501	CD45 on CD33br H	1	24672641
ebi-a-GCST90002043	rs35895413	CD45 on CD33br H	1	85318028
ebi-a-GCST90002043	rs6760298	CD45 on CD33br H	2	54865820
ebi-a-GCST90002043	rs1983079	CD45 on CD33br H	3	113961934
ebi-a-GCST90002043	rs1580170	CD45 on CD33br H	5	29803516
ebi-a-GCST90002043	rs73264490	CD45 on CD33br H	7	1329138
ebi-a-GCST90002043	rs10252843	CD45 on CD33br H	7	133674795
ebi-a-GCST90002043	rs116900648	CD45 on CD33br H	8	144503650
ebi-a-GCST90002043	rs182091184	CD45 on CD33br H	9	32566749
ebi-a-GCST90002043	rs76777625	CD45 on CD33br H	10	12701296
ebi-a-GCST90002043	rs9421560	CD45 on CD33br H	10	88794870
ebi-a-GCST90002043	rs921674	CD45 on CD33br H	11	68869308
ebi-a-GCST90002043	rs713522	CD45 on CD33br H	17	44065263
ebi-a-GCST90002043	rs111988158	CD45 on CD33br H	18	368602
ebi-a-GCST90002043	rs62132587	CD45 on CD33br H	19	561816
ebi-a-GCST90002044	rs4676406	CD45 on CD33dim	2	241579108
ebi-a-GCST90002044	rs2406420	CD45 on CD33dim	5	120560841
ebi-a-GCST90002044	rs1354185	CD45 on CD33dim	5	13761068
ebi-a-GCST90002044	rs4142311	CD45 on CD33dim	6	169034116
ebi-a-GCST90002044	rs75267165	CD45 on CD33dim	7	44180044
ebi-a-GCST90002044	rs376418616	CD45 on CD33dim	10	14610624
ebi-a-GCST90002044	rs1784661	CD45 on CD33dim	11	110154710
ebi-a-GCST90002044	rs55823336	CD45 on CD33dim	15	86320164
ebi-a-GCST90002044	rs12459419	CD45 on CD33dim	19	51728477
ebi-a-GCST90002044	rs2824282	CD45 on CD33dim	21	18723671
ebi-a-GCST90002045	rs10919544	CD45 on CD33- HI	1	161508763
ebi-a-GCST90002045	rs112370962	CD45 on CD33- HI	1	15693424
ebi-a-GCST90002045	rs138593127	CD45 on CD33- HI	2	126466187
ebi-a-GCST90002045	rs12614599	CD45 on CD33- HI	2	232290640
ebi-a-GCST90002045	rs144954155	CD45 on CD33- HI	3	177691462

ebi-a-GCST90002045	rs9814135	CD45 on CD33- HI	3	194094110
ebi-a-GCST90002045	rs1562059	CD45 on CD33- HI	4	55253342
ebi-a-GCST90002045	rs139602776	CD45 on CD33- HI	4	103564713
ebi-a-GCST90002045	rs115428824	CD45 on CD33- HI	4	125753562
ebi-a-GCST90002045	rs114152229	CD45 on CD33- HI	4	92853329
ebi-a-GCST90002045	rs111649552	CD45 on CD33- HI	8	117456157
ebi-a-GCST90002045	rs117072933	CD45 on CD33- HI	9	84909178
ebi-a-GCST90002045	rs140313648	CD45 on CD33- HI	10	29037194
ebi-a-GCST90002045	rs34710654	CD45 on CD33- HI	13	96096633
ebi-a-GCST90002045	rs2358778	CD45 on CD33- HI	16	31868109
ebi-a-GCST90002045	rs4386182	CD45 on CD33- HI	17	54816038
ebi-a-GCST90002046	rs1326284	CD45 on CD33- HI	1	198553794
ebi-a-GCST90002046	rs143150964	CD45 on CD33- HI	1	238709665
ebi-a-GCST90002046	rs12523072	CD45 on CD33- HI	5	104197397
ebi-a-GCST90002046	rs149228799	CD45 on CD33- HI	6	7917872
ebi-a-GCST90002046	rs117317282	CD45 on CD33- HI	6	32497719
ebi-a-GCST90002046	rs17552904	CD45 on CD33- HI	7	50318308
ebi-a-GCST90002046	rs145631483	CD45 on CD33- HI	8	4517297
ebi-a-GCST90002046	rs116900648	CD45 on CD33- HI	8	144503650
ebi-a-GCST90002046	rs34386977	CD45 on CD33- HI	8	134200209
ebi-a-GCST90002046	rs10971439	CD45 on CD33- HI	9	33180813
ebi-a-GCST90002046	rs142398114	CD45 on CD33- HI	9	106075712
ebi-a-GCST90002046	rs3124947	CD45 on CD33- HI	9	137762081
ebi-a-GCST90002046	rs73664150	CD45 on CD33- HI	9	137710319
ebi-a-GCST90002046	rs11222974	CD45 on CD33- HI	11	132073382
ebi-a-GCST90002046	rs4980766	CD45 on CD33- HI	11	69946996
ebi-a-GCST90002046	rs117745284	CD45 on CD33- HI	11	95746999
ebi-a-GCST90002046	rs11232456	CD45 on CD33- HI	11	80811894
ebi-a-GCST90002046	rs10735302	CD45 on CD33- HI	12	93068616
ebi-a-GCST90002046	rs71466481	CD45 on CD33- HI	12	130568569
ebi-a-GCST90002046	rs576712691	CD45 on CD33- HI	12	17715604
ebi-a-GCST90002046	rs1678025	CD45 on CD33- HI	14	102351858
ebi-a-GCST90002046	rs34358471	CD45 on CD33- HI	16	51353423
ebi-a-GCST90002046	rs9892517	CD45 on CD33- HI	17	6592054
ebi-a-GCST90002046	rs33978622	CD45 on CD33- HI	19	51736383
ebi-a-GCST90002047	rs320439	CD45 on Gr MDSC	1	213629078
ebi-a-GCST90002047	rs144971347	CD45 on Gr MDSC	1	175964456
ebi-a-GCST90002047	rs2197801	CD45 on Gr MDSC	2	177451264
ebi-a-GCST90002047	rs56907657	CD45 on Gr MDSC	5	124407733
ebi-a-GCST90002047	rs12333289	CD45 on Gr MDSC	6	6263446
ebi-a-GCST90002047	rs10224609	CD45 on Gr MDSC	7	20505412
ebi-a-GCST90002047	rs11506039	CD45 on Gr MDSC	7	50344544
ebi-a-GCST90002047	rs1534770	CD45 on Gr MDSC	7	21987804
ebi-a-GCST90002047	rs36048875	CD45 on Gr MDSC	8	128479961
ebi-a-GCST90002047	rs35066493	CD45 on Gr MDSC	9	86717077
ebi-a-GCST90002047	rs2377426	CD45 on Gr MDSC	9	78546389
ebi-a-GCST90002047	rs146131093	CD45 on Gr MDSC	11	113379377
ebi-a-GCST90002047	rs2731088	CD45 on Gr MDSC	12	48960058

ebi-a-GCST90002047	rs2148972	CD45 on Gr MDSC	13	75259853
ebi-a-GCST90002047	rs111800240	CD45 on Gr MDSC	14	43631525
ebi-a-GCST90002047	rs74545928	CD45 on Gr MDSC	15	54615098
ebi-a-GCST90002047	rs9922435	CD45 on Gr MDSC	16	86622639
ebi-a-GCST90002047	rs111896566	CD45 on Gr MDSC	16	20551336
ebi-a-GCST90002048	rs114313091	CD45 on CD66b++	1	226903189
ebi-a-GCST90002048	rs34661403	CD45 on CD66b++	1	86716001
ebi-a-GCST90002048	rs3755285	CD45 on CD66b++	2	102844020
ebi-a-GCST90002048	rs62275033	CD45 on CD66b++	3	148889235
ebi-a-GCST90002048	rs4494933	CD45 on CD66b++	3	164582905
ebi-a-GCST90002048	rs62257614	CD45 on CD66b++	3	52464827
ebi-a-GCST90002048	rs138540634	CD45 on CD66b++	4	91238751
ebi-a-GCST90002048	rs67708934	CD45 on CD66b++	7	82015343
ebi-a-GCST90002048	rs12555208	CD45 on CD66b++	9	130531142
ebi-a-GCST90002048	rs75424883	CD45 on CD66b++	10	23872652
ebi-a-GCST90002048	rs61907767	CD45 on CD66b++	11	128417551
ebi-a-GCST90002048	rs111573488	CD45 on CD66b++	12	82723650
ebi-a-GCST90002048	rs2095320	CD45 on CD66b++	14	96663840
ebi-a-GCST90002048	rs72696815	CD45 on CD66b++	14	95115530
ebi-a-GCST90002048	rs117014682	CD45 on CD66b++	16	19693627
ebi-a-GCST90002048	rs74813486	CD45 on CD66b++	17	49958870
ebi-a-GCST90002049	rs41270829	CD45 on Mo MDSC	1	40011690
ebi-a-GCST90002049	rs6696776	CD45 on Mo MDSC	1	107930205
ebi-a-GCST90002049	rs17719731	CD45 on Mo MDSC	2	209453912
ebi-a-GCST90002049	rs139648605	CD45 on Mo MDSC	2	59488592
ebi-a-GCST90002049	rs145290654	CD45 on Mo MDSC	3	46613040
ebi-a-GCST90002049	rs147320610	CD45 on Mo MDSC	4	179767370
ebi-a-GCST90002049	rs79805545	CD45 on Mo MDSC	5	152737877
ebi-a-GCST90002049	rs142164490	CD45 on Mo MDSC	6	102083682
ebi-a-GCST90002049	rs145907787	CD45 on Mo MDSC	6	133082731
ebi-a-GCST90002049	rs73116504	CD45 on Mo MDSC	7	52470831
ebi-a-GCST90002049	rs76204403	CD45 on Mo MDSC	8	121868335
ebi-a-GCST90002049	rs111704828	CD45 on Mo MDSC	10	2108255
ebi-a-GCST90002049	rs7983916	CD45 on Mo MDSC	13	22642894
ebi-a-GCST90002049	rs9566131	CD45 on Mo MDSC	13	86734039
ebi-a-GCST90002049	rs11070797	CD45 on Mo MDSC	15	31376696
ebi-a-GCST90002049	rs8091698	CD45 on Mo MDSC	18	62407267
ebi-a-GCST90002049	rs10410260	CD45 on Mo MDSC	19	7150938
ebi-a-GCST90002050	rs143331027	CD45 on CD33dim	1	28663941
ebi-a-GCST90002050	rs149863711	CD45 on CD33dim	3	2628309
ebi-a-GCST90002050	rs28527224	CD45 on CD33dim	4	125843057
ebi-a-GCST90002050	rs1588987	CD45 on CD33dim	4	92428380
ebi-a-GCST90002050	rs4864443	CD45 on CD33dim	4	53969870
ebi-a-GCST90002050	rs1352606	CD45 on CD33dim	5	66683389
ebi-a-GCST90002050	rs73000803	CD45 on CD33dim	6	132835314
ebi-a-GCST90002050	rs138738554	CD45 on CD33dim	6	18228165
ebi-a-GCST90002050	rs183052978	CD45 on CD33dim	7	88592152
ebi-a-GCST90002050	rs150735276	CD45 on CD33dim	7	21186016

ebi-a-GCST90002050	rs4585735	CD45 on CD33dim	8	143909216
ebi-a-GCST90002050	rs117961017	CD45 on CD33dim	8	128170250
ebi-a-GCST90002050	rs11140803	CD45 on CD33dim	9	87512669
ebi-a-GCST90002050	rs72743016	CD45 on CD33dim	9	85834645
ebi-a-GCST90002050	rs681136	CD45 on CD33dim	11	77856505
ebi-a-GCST90002050	rs6598209	CD45 on CD33dim	12	132703347
ebi-a-GCST90002050	rs80337742	CD45 on CD33dim	13	22789793
ebi-a-GCST90002050	rs117492745	CD45 on CD33dim	18	7523191
ebi-a-GCST90002050	rs116940177	CD45 on CD33dim	20	410531
ebi-a-GCST90002051	rs4916296	CD45 on basophil	1	172989052
ebi-a-GCST90002051	rs149863711	CD45 on basophil	3	2628309
ebi-a-GCST90002051	rs4864443	CD45 on basophil	4	53969870
ebi-a-GCST90002051	rs1352606	CD45 on basophil	5	66683389
ebi-a-GCST90002051	rs73000803	CD45 on basophil	6	132835314
ebi-a-GCST90002051	rs149715992	CD45 on basophil	6	133440965
ebi-a-GCST90002051	rs59203915	CD45 on basophil	7	5621740
ebi-a-GCST90002051	rs72690741	CD45 on basophil	8	142891200
ebi-a-GCST90002051	rs72752359	CD45 on basophil	9	98105210
ebi-a-GCST90002051	rs147913115	CD45 on basophil	9	124657581
ebi-a-GCST90002051	rs16937506	CD45 on basophil	9	19413670
ebi-a-GCST90002051	rs1246274	CD45 on basophil	9	86770003
ebi-a-GCST90002051	rs9629947	CD45 on basophil	10	135447375
ebi-a-GCST90002051	rs12415341	CD45 on basophil	10	117921247
ebi-a-GCST90002051	rs10764985	CD45 on basophil	10	132597423
ebi-a-GCST90002051	rs12804356	CD45 on basophil	11	65315980
ebi-a-GCST90002051	rs74372761	CD45 on basophil	13	63277576
ebi-a-GCST90002051	rs17128008	CD45 on basophil	14	55308210
ebi-a-GCST90002051	rs1526598	CD45 on basophil	17	38870443
ebi-a-GCST90002052	rs115457429	CD45 on Im MDSC	3	28872973
ebi-a-GCST90002052	rs6793627	CD45 on Im MDSC	3	189044299
ebi-a-GCST90002052	rs114052849	CD45 on Im MDSC	5	16610399
ebi-a-GCST90002052	rs73238009	CD45 on Im MDSC	8	29828802
ebi-a-GCST90002052	rs117224922	CD45 on Im MDSC	12	5441979
ebi-a-GCST90002052	rs9557033	CD45 on Im MDSC	13	99343094
ebi-a-GCST90002052	rs12884697	CD45 on Im MDSC	14	61935996
ebi-a-GCST90002052	rs273640	CD45 on Im MDSC	19	51725824
ebi-a-GCST90002052	rs7351079	CD45 on Im MDSC	19	51574038
ebi-a-GCST90002052	rs111695695	CD45 on Im MDSC	19	51897760
ebi-a-GCST90002052	rs34070496	CD45 on Im MDSC	20	16970768
ebi-a-GCST90002053	rs72761150	CD45 on CD33br H	1	245515377
ebi-a-GCST90002053	rs7533281	CD45 on CD33br H	1	24698029
ebi-a-GCST90002053	rs72677097	CD45 on CD33br H	1	61134076
ebi-a-GCST90002053	rs2330139	CD45 on CD33br H	5	29787554
ebi-a-GCST90002053	rs114094080	CD45 on CD33br H	5	26499129
ebi-a-GCST90002053	rs2540593	CD45 on CD33br H	7	91148402
ebi-a-GCST90002053	rs73264490	CD45 on CD33br H	7	1329138
ebi-a-GCST90002053	rs139335095	CD45 on CD33br H	11	62711027
ebi-a-GCST90002053	rs61907325	CD45 on CD33br H	11	85802779

ebi-a-GCST90002053	rs11612383	CD45 on CD33br H	12	1831355
ebi-a-GCST90002053	rs703593	CD45 on CD33br H	12	103064247
ebi-a-GCST90002053	rs143599997	CD45 on CD33br H	12	130798660
ebi-a-GCST90002053	rs35843682	CD45 on CD33br H	14	87968127
ebi-a-GCST90002053	rs56097305	CD45 on CD33br H	17	13961007
ebi-a-GCST90002053	rs12970861	CD45 on CD33br H	18	66283602
ebi-a-GCST90002053	rs147597282	CD45 on CD33br H	19	49965537
ebi-a-GCST90002054	rs138809534	CD8 on CM CD8br	2	11541120
ebi-a-GCST90002054	rs3020726	CD8 on CM CD8br	2	87016506
ebi-a-GCST90002054	rs56214822	CD8 on CM CD8br	2	53947717
ebi-a-GCST90002054	rs77434118	CD8 on CM CD8br	3	144965710
ebi-a-GCST90002054	rs6882406	CD8 on CM CD8br	5	3098494
ebi-a-GCST90002054	rs9379064	CD8 on CM CD8br	6	6818562
ebi-a-GCST90002054	rs4081559	CD8 on CM CD8br	6	31323677
ebi-a-GCST90002054	rs1633108	CD8 on CM CD8br	6	29783305
ebi-a-GCST90002054	rs72657634	CD8 on CM CD8br	6	29849073
ebi-a-GCST90002054	rs2711500	CD8 on CM CD8br	7	68310336
ebi-a-GCST90002054	rs16889563	CD8 on CM CD8br	8	40408479
ebi-a-GCST90002054	rs41287409	CD8 on CM CD8br	9	71320912
ebi-a-GCST90002054	rs1791808	CD8 on CM CD8br	11	114103610
ebi-a-GCST90002054	rs4760640	CD8 on CM CD8br	12	49203355
ebi-a-GCST90002054	rs144117835	CD8 on CM CD8br	13	57475168
ebi-a-GCST90002054	rs1230475	CD8 on CM CD8br	13	46774761
ebi-a-GCST90002055	rs1977332	CD8 on naive CD8l	1	29805041
ebi-a-GCST90002055	rs35626322	CD8 on naive CD8l	2	87030421
ebi-a-GCST90002055	rs115452662	CD8 on naive CD8l	2	88794200
ebi-a-GCST90002055	rs555594976	CD8 on naive CD8l	2	92267258
ebi-a-GCST90002055	rs73186411	CD8 on naive CD8l	3	189784957
ebi-a-GCST90002055	rs28619960	CD8 on naive CD8l	4	14887657
ebi-a-GCST90002055	rs116211259	CD8 on naive CD8l	4	152943585
ebi-a-GCST90002055	rs76631647	CD8 on naive CD8l	4	187610871
ebi-a-GCST90002055	rs115155396	CD8 on naive CD8l	5	89015995
ebi-a-GCST90002055	rs4081559	CD8 on naive CD8l	6	31323677
ebi-a-GCST90002055	rs2516429	CD8 on naive CD8l	6	31426881
ebi-a-GCST90002055	rs13196568	CD8 on naive CD8l	6	31098400
ebi-a-GCST90002055	rs552412	CD8 on naive CD8l	6	31777077
ebi-a-GCST90002055	rs79937884	CD8 on naive CD8l	7	14896868
ebi-a-GCST90002055	rs77535524	CD8 on naive CD8l	11	122690399
ebi-a-GCST90002055	rs11038843	CD8 on naive CD8l	11	46288623
ebi-a-GCST90002055	rs12184937	CD8 on naive CD8l	13	23274737
ebi-a-GCST90002055	rs9672901	CD8 on naive CD8l	15	93058567
ebi-a-GCST90002056	rs3000791	CD8 on EM CD8br	1	227599174
ebi-a-GCST90002056	rs184295970	CD8 on EM CD8br	1	95836523
ebi-a-GCST90002056	rs62153285	CD8 on EM CD8br	2	81780122
ebi-a-GCST90002056	rs3020726	CD8 on EM CD8br	2	87016506
ebi-a-GCST90002056	rs12478003	CD8 on EM CD8br	2	47479869
ebi-a-GCST90002056	rs114486180	CD8 on EM CD8br	2	193161497
ebi-a-GCST90002056	rs60701118	CD8 on EM CD8br	2	224854203

ebi-a-GCST90002056	rs4860179	CD8 on EM CD8br	4	66143879
ebi-a-GCST90002056	rs73774928	CD8 on EM CD8br	5	102855858
ebi-a-GCST90002056	rs13171873	CD8 on EM CD8br	5	3080656
ebi-a-GCST90002056	rs17201560	CD8 on EM CD8br	6	32047268
ebi-a-GCST90002056	rs115795061	CD8 on EM CD8br	6	32731861
ebi-a-GCST90002056	rs2571390	CD8 on EM CD8br	6	29923554
ebi-a-GCST90002056	rs66465252	CD8 on EM CD8br	6	63625302
ebi-a-GCST90002056	rs2734989	CD8 on EM CD8br	6	29812643
ebi-a-GCST90002056	rs11771058	CD8 on EM CD8br	7	53008596
ebi-a-GCST90002056	rs16889563	CD8 on EM CD8br	8	40408479
ebi-a-GCST90002056	rs117197627	CD8 on EM CD8br	10	18473835
ebi-a-GCST90002056	rs1701094	CD8 on EM CD8br	12	44468877
ebi-a-GCST90002056	rs35677476	CD8 on EM CD8br	14	33784800
ebi-a-GCST90002056	rs77561506	CD8 on EM CD8br	14	55423735
ebi-a-GCST90002056	rs58193384	CD8 on EM CD8br	14	68235542
ebi-a-GCST90002056	rs4782640	CD8 on EM CD8br	16	84637174
ebi-a-GCST90002056	rs16995213	CD8 on EM CD8br	20	15005337
ebi-a-GCST90002057	rs9332511	CD8 on TD CD8br	1	169545822
ebi-a-GCST90002057	rs960502	CD8 on TD CD8br	1	55612278
ebi-a-GCST90002057	rs1746718	CD8 on TD CD8br	1	16941207
ebi-a-GCST90002057	rs1079009	CD8 on TD CD8br	2	121365377
ebi-a-GCST90002057	rs72795179	CD8 on TD CD8br	2	38931153
ebi-a-GCST90002057	rs115313377	CD8 on TD CD8br	2	156954164
ebi-a-GCST90002057	rs3020726	CD8 on TD CD8br	2	87016506
ebi-a-GCST90002057	rs13068900	CD8 on TD CD8br	3	175739566
ebi-a-GCST90002057	rs56393413	CD8 on TD CD8br	3	73016333
ebi-a-GCST90002057	rs144350282	CD8 on TD CD8br	6	1689657
ebi-a-GCST90002057	rs2571390	CD8 on TD CD8br	6	29923554
ebi-a-GCST90002057	rs2523887	CD8 on TD CD8br	6	31040725
ebi-a-GCST90002057	rs114373132	CD8 on TD CD8br	6	32106813
ebi-a-GCST90002057	rs75312675	CD8 on TD CD8br	8	133229489
ebi-a-GCST90002057	rs12894741	CD8 on TD CD8br	14	53791493
ebi-a-GCST90002057	rs4454952	CD8 on TD CD8br	15	100798686
ebi-a-GCST90002057	rs111824350	CD8 on TD CD8br	17	20661020
ebi-a-GCST90002057	rs73058568	CD8 on TD CD8br	20	202468
ebi-a-GCST90002057	rs2472787	CD8 on TD CD8br	22	45673477
ebi-a-GCST90002058	rs3020726	CD8 on CD8br	2	87016506
ebi-a-GCST90002058	rs111939240	CD8 on CD8br	2	42654159
ebi-a-GCST90002058	rs35701411	CD8 on CD8br	2	88472249
ebi-a-GCST90002058	rs11126482	CD8 on CD8br	2	75977801
ebi-a-GCST90002058	rs149303959	CD8 on CD8br	2	97046283
ebi-a-GCST90002058	rs17389736	CD8 on CD8br	2	8286509
ebi-a-GCST90002058	rs149618549	CD8 on CD8br	3	180210710
ebi-a-GCST90002058	rs74737596	CD8 on CD8br	5	95116760
ebi-a-GCST90002058	rs9450269	CD8 on CD8br	6	86114684
ebi-a-GCST90002058	rs294880	CD8 on CD8br	6	159603722
ebi-a-GCST90002058	rs11964370	CD8 on CD8br	6	75445964
ebi-a-GCST90002058	rs2531817	CD8 on CD8br	6	28448894

ebi-a-GCST90002058	rs1050420	CD8 on CD8br	6	31239518
ebi-a-GCST90002058	rs2395041	CD8 on CD8br	6	31461795
ebi-a-GCST90002058	rs9405084	CD8 on CD8br	6	31326251
ebi-a-GCST90002058	rs77657145	CD8 on CD8br	7	92167577
ebi-a-GCST90002058	rs9987823	CD8 on CD8br	9	38394541
ebi-a-GCST90002058	rs116049155	CD8 on CD8br	9	120333000
ebi-a-GCST90002058	rs73305072	CD8 on CD8br	12	50034058
ebi-a-GCST90002058	rs71581981	CD8 on CD8br	12	21327906
ebi-a-GCST90002058	rs6538668	CD8 on CD8br	12	96128771
ebi-a-GCST90002058	rs149065025	CD8 on CD8br	15	33826794
ebi-a-GCST90002058	rs34026630	CD8 on CD8br	16	79776245
ebi-a-GCST90002058	rs750126	CD8 on CD8br	16	88813371
ebi-a-GCST90002058	rs140845172	CD8 on CD8br	19	520519
ebi-a-GCST90002058	rs657944	CD8 on CD8br	19	15685999
ebi-a-GCST90002058	rs392871	CD8 on CD8br	20	15228544
ebi-a-GCST90002058	rs76477385	CD8 on CD8br	20	59336213
ebi-a-GCST90002058	rs73158117	CD8 on CD8br	22	37259534
ebi-a-GCST90002058	rs6005542	CD8 on CD8br	22	28043820
ebi-a-GCST90002058	rs4819981	CD8 on CD8br	22	17725304
ebi-a-GCST90002059	rs12408387	CD8 on NKT	1	119528329
ebi-a-GCST90002059	rs34613828	CD8 on NKT	2	87022759
ebi-a-GCST90002059	rs114165927	CD8 on NKT	2	163117904
ebi-a-GCST90002059	rs369008323	CD8 on NKT	2	100182724
ebi-a-GCST90002059	rs77495878	CD8 on NKT	3	148979844
ebi-a-GCST90002059	rs72625020	CD8 on NKT	3	185596778
ebi-a-GCST90002059	rs146192023	CD8 on NKT	4	25802933
ebi-a-GCST90002059	rs143651731	CD8 on NKT	5	22407099
ebi-a-GCST90002059	rs138458033	CD8 on NKT	6	161660100
ebi-a-GCST90002059	rs6915999	CD8 on NKT	6	31377851
ebi-a-GCST90002059	rs2395191	CD8 on NKT	6	32447085
ebi-a-GCST90002059	rs2610941	CD8 on NKT	8	21151009
ebi-a-GCST90002059	rs2249975	CD8 on NKT	10	108103587
ebi-a-GCST90002059	rs117884995	CD8 on NKT	11	11712983
ebi-a-GCST90002059	rs7126488	CD8 on NKT	11	24190138
ebi-a-GCST90002059	rs11050506	CD8 on NKT	12	30013127
ebi-a-GCST90002059	rs56331909	CD8 on NKT	16	84582423
ebi-a-GCST90002059	rs1871698	CD8 on NKT	18	65187856
ebi-a-GCST90002059	rs2286004	CD8 on NKT	19	1792190
ebi-a-GCST90002060	rs3020726	CD8 on HLA DR+	2	87016506
ebi-a-GCST90002060	rs55778875	CD8 on HLA DR+	2	12312863
ebi-a-GCST90002060	rs1376775	CD8 on HLA DR+	2	121385142
ebi-a-GCST90002060	rs11128659	CD8 on HLA DR+	3	13833365
ebi-a-GCST90002060	rs79120070	CD8 on HLA DR+	3	96895394
ebi-a-GCST90002060	rs2523402	CD8 on HLA DR+	6	29699156
ebi-a-GCST90002060	rs115127002	CD8 on HLA DR+	6	159442296
ebi-a-GCST90002060	rs2523882	CD8 on HLA DR+	6	31042217
ebi-a-GCST90002060	rs36100818	CD8 on HLA DR+	6	123397736
ebi-a-GCST90002060	rs11964370	CD8 on HLA DR+	6	75445964

ebi-a-GCST90002060	rs3131290	CD8 on HLA DR+	6	32183175
ebi-a-GCST90002060	rs117731852	CD8 on HLA DR+	7	86744636
ebi-a-GCST90002060	rs10234570	CD8 on HLA DR+	7	10875345
ebi-a-GCST90002060	rs1401480	CD8 on HLA DR+	8	4117811
ebi-a-GCST90002060	rs9987823	CD8 on HLA DR+	9	38394541
ebi-a-GCST90002060	rs75208356	CD8 on HLA DR+	10	67768125
ebi-a-GCST90002060	rs148307858	CD8 on HLA DR+	10	114431196
ebi-a-GCST90002060	rs71482199	CD8 on HLA DR+	11	40291897
ebi-a-GCST90002060	rs117660790	CD8 on HLA DR+	14	28384801
ebi-a-GCST90002060	rs149065025	CD8 on HLA DR+	15	33826794
ebi-a-GCST90002060	rs9926812	CD8 on HLA DR+	16	88811418
ebi-a-GCST90002060	rs12602418	CD8 on HLA DR+	17	9678294
ebi-a-GCST90002060	rs140845172	CD8 on HLA DR+	19	520519
ebi-a-GCST90002060	rs7258589	CD8 on HLA DR+	19	18701499
ebi-a-GCST90002060	rs56350470	CD8 on HLA DR+	19	53813407
ebi-a-GCST90002061	rs12027949	CD4 on CD39+ CD	1	218448425
ebi-a-GCST90002061	rs41266967	CD4 on CD39+ CD	2	241529785
ebi-a-GCST90002061	rs13127505	CD4 on CD39+ CD	4	141880770
ebi-a-GCST90002061	rs7747800	CD4 on CD39+ CD	6	30927617
ebi-a-GCST90002061	rs78933037	CD4 on CD39+ CD	6	1424645
ebi-a-GCST90002061	rs3016018	CD4 on CD39+ CD	6	31314106
ebi-a-GCST90002061	rs9264532	CD4 on CD39+ CD	6	31234381
ebi-a-GCST90002061	rs12200389	CD4 on CD39+ CD	6	38122097
ebi-a-GCST90002061	rs184085789	CD4 on CD39+ CD	7	20296885
ebi-a-GCST90002061	rs16938946	CD4 on CD39+ CD	8	75687887
ebi-a-GCST90002061	rs139181081	CD4 on CD39+ CD	8	96559364
ebi-a-GCST90002061	rs17151969	CD4 on CD39+ CD	8	10301469
ebi-a-GCST90002061	rs433305	CD4 on CD39+ CD	9	19209546
ebi-a-GCST90002061	rs1836233	CD4 on CD39+ CD	9	8797798
ebi-a-GCST90002061	rs2451473	CD4 on CD39+ CD	10	106573032
ebi-a-GCST90002061	rs117458836	CD4 on CD39+ CD	10	96773382
ebi-a-GCST90002061	rs118019481	CD4 on CD39+ CD	10	95151086
ebi-a-GCST90002061	rs7084635	CD4 on CD39+ CD	10	97062753
ebi-a-GCST90002061	rs2168739	CD4 on CD39+ CD	10	109402407
ebi-a-GCST90002061	rs10786281	CD4 on CD39+ CD	10	98116936
ebi-a-GCST90002061	rs2761317	CD4 on CD39+ CD	10	95056698
ebi-a-GCST90002061	rs12778618	CD4 on CD39+ CD	10	95633120
ebi-a-GCST90002061	rs7086986	CD4 on CD39+ CD	10	97507312
ebi-a-GCST90002061	rs4293064	CD4 on CD39+ CD	10	107757380
ebi-a-GCST90002061	rs117190770	CD4 on CD39+ CD	10	86457358
ebi-a-GCST90002061	rs73426887	CD4 on CD39+ CD	11	13768946
ebi-a-GCST90002061	rs11615628	CD4 on CD39+ CD	12	6903631
ebi-a-GCST90002061	rs9552362	CD4 on CD39+ CD	13	21693572
ebi-a-GCST90002061	rs142814565	CD4 on CD39+ CD	17	75698827
ebi-a-GCST90002061	rs118123748	CD4 on CD39+ CD	18	11805408
ebi-a-GCST90002062	rs634639	CD4 on CD28+ CD	1	153025933
ebi-a-GCST90002062	rs12471945	CD4 on CD28+ CD	2	19654868
ebi-a-GCST90002062	rs6724611	CD4 on CD28+ CD	2	46180188

ebi-a-GCST90002062	rs183012056	CD4 on CD28+ CD	4	4711458
ebi-a-GCST90002062	rs831657	CD4 on CD28+ CD	5	16286973
ebi-a-GCST90002062	rs1762718	CD4 on CD28+ CD	6	107319615
ebi-a-GCST90002062	rs3130215	CD4 on CD28+ CD	6	33074963
ebi-a-GCST90002062	rs12664217	CD4 on CD28+ CD	6	108418406
ebi-a-GCST90002062	rs114729093	CD4 on CD28+ CD	6	55192621
ebi-a-GCST90002062	rs2844608	CD4 on CD28+ CD	6	31247414
ebi-a-GCST90002062	rs16938946	CD4 on CD28+ CD	8	75687887
ebi-a-GCST90002062	rs140084476	CD4 on CD28+ CD	8	24184495
ebi-a-GCST90002062	rs62568944	CD4 on CD28+ CD	9	87813612
ebi-a-GCST90002062	rs17143738	CD4 on CD28+ CD	10	8505721
ebi-a-GCST90002062	rs146956135	CD4 on CD28+ CD	10	86432367
ebi-a-GCST90002062	rs79998393	CD4 on CD28+ CD	10	25354093
ebi-a-GCST90002062	rs11615628	CD4 on CD28+ CD	12	6903631
ebi-a-GCST90002062	rs10138570	CD4 on CD28+ CD	14	65860038
ebi-a-GCST90002062	rs1079042	CD4 on CD28+ CD	15	27842037
ebi-a-GCST90002062	rs9906657	CD4 on CD28+ CD	17	40557958
ebi-a-GCST90002062	rs11655205	CD4 on CD28+ CD	17	6630904
ebi-a-GCST90002062	rs117058083	CD4 on CD28+ CD	22	26210585
ebi-a-GCST90002063	rs10911945	CD4 on CD4 Treg	1	186852597
ebi-a-GCST90002063	rs12039573	CD4 on CD4 Treg	1	20941515
ebi-a-GCST90002063	rs41266967	CD4 on CD4 Treg	2	241529785
ebi-a-GCST90002063	rs78697264	CD4 on CD4 Treg	4	39277538
ebi-a-GCST90002063	rs146641318	CD4 on CD4 Treg	4	9024208
ebi-a-GCST90002063	rs831657	CD4 on CD4 Treg	5	16286973
ebi-a-GCST90002063	rs60409747	CD4 on CD4 Treg	5	39751811
ebi-a-GCST90002063	rs2844608	CD4 on CD4 Treg	6	31247414
ebi-a-GCST90002063	rs7744589	CD4 on CD4 Treg	6	6051514
ebi-a-GCST90002063	rs116138399	CD4 on CD4 Treg	6	31325478
ebi-a-GCST90002063	rs196695	CD4 on CD4 Treg	6	80154074
ebi-a-GCST90002063	rs140684041	CD4 on CD4 Treg	6	169725886
ebi-a-GCST90002063	rs150643215	CD4 on CD4 Treg	7	20291018
ebi-a-GCST90002063	rs56216313	CD4 on CD4 Treg	7	141053545
ebi-a-GCST90002063	rs7831103	CD4 on CD4 Treg	8	22762175
ebi-a-GCST90002063	rs16938946	CD4 on CD4 Treg	8	75687887
ebi-a-GCST90002063	rs116421623	CD4 on CD4 Treg	8	119192827
ebi-a-GCST90002063	rs74800155	CD4 on CD4 Treg	9	24765046
ebi-a-GCST90002063	rs189810664	CD4 on CD4 Treg	9	97001952
ebi-a-GCST90002063	rs79998393	CD4 on CD4 Treg	10	25354093
ebi-a-GCST90002063	rs117421233	CD4 on CD4 Treg	10	85630450
ebi-a-GCST90002063	rs117190770	CD4 on CD4 Treg	10	86457358
ebi-a-GCST90002063	rs4747882	CD4 on CD4 Treg	10	6162476
ebi-a-GCST90002063	rs73457945	CD4 on CD4 Treg	11	21414632
ebi-a-GCST90002063	rs75040387	CD4 on CD4 Treg	11	86960608
ebi-a-GCST90002063	rs11615628	CD4 on CD4 Treg	12	6903631
ebi-a-GCST90002063	rs2299912	CD4 on CD4 Treg	14	78012732
ebi-a-GCST90002063	rs9898118	CD4 on CD4 Treg	17	77121278
ebi-a-GCST90002063	rs34154026	CD4 on CD4 Treg	17	40564630

ebi-a-GCST90002063	rs62111177	CD4 on CD4 Treg	19	13471119
ebi-a-GCST90002064	rs2368500	CD4 on resting Treg	2	241021276
ebi-a-GCST90002064	rs6724611	CD4 on resting Treg	2	46180188
ebi-a-GCST90002064	rs116477286	CD4 on resting Treg	4	23267234
ebi-a-GCST90002064	rs112402469	CD4 on resting Treg	5	141172502
ebi-a-GCST90002064	rs2844608	CD4 on resting Treg	6	31247414
ebi-a-GCST90002064	rs114729093	CD4 on resting Treg	6	55192621
ebi-a-GCST90002064	rs9294327	CD4 on resting Treg	6	85782234
ebi-a-GCST90002064	rs145783964	CD4 on resting Treg	6	169725888
ebi-a-GCST90002064	rs117384636	CD4 on resting Treg	9	7145483
ebi-a-GCST90002064	rs61908390	CD4 on resting Treg	12	6015632
ebi-a-GCST90002064	rs11615628	CD4 on resting Treg	12	6903631
ebi-a-GCST90002064	rs8011224	CD4 on resting Treg	14	65969670
ebi-a-GCST90002064	rs11646104	CD4 on resting Treg	16	82621799
ebi-a-GCST90002064	rs4793163	CD4 on resting Treg	17	40680934
ebi-a-GCST90002065	rs184800017	CD4 on CD39+ rest	1	3275382
ebi-a-GCST90002065	rs72852573	CD4 on CD39+ rest	2	142742594
ebi-a-GCST90002065	rs1543082	CD4 on CD39+ rest	2	9212749
ebi-a-GCST90002065	rs142122078	CD4 on CD39+ rest	3	36456475
ebi-a-GCST90002065	rs2523887	CD4 on CD39+ rest	6	31040725
ebi-a-GCST90002065	rs6601591	CD4 on CD39+ rest	8	11316692
ebi-a-GCST90002065	rs117153869	CD4 on CD39+ rest	8	32077693
ebi-a-GCST90002065	rs117450546	CD4 on CD39+ rest	8	37371755
ebi-a-GCST90002065	rs138782600	CD4 on CD39+ rest	9	35195975
ebi-a-GCST90002065	rs79998393	CD4 on CD39+ rest	10	25354093
ebi-a-GCST90002065	rs147763768	CD4 on CD39+ rest	10	97548303
ebi-a-GCST90002065	rs12223055	CD4 on CD39+ rest	11	121778965
ebi-a-GCST90002065	rs2239094	CD4 on CD39+ rest	12	2610538
ebi-a-GCST90002065	rs17110593	CD4 on CD39+ rest	14	26533159
ebi-a-GCST90002065	rs56075460	CD4 on CD39+ rest	16	27901250
ebi-a-GCST90002065	rs382106	CD4 on CD39+ rest	16	22651209
ebi-a-GCST90002066	rs12039573	CD4 on activated T	1	20941515
ebi-a-GCST90002066	rs78697264	CD4 on activated T	4	39277538
ebi-a-GCST90002066	rs139628646	CD4 on activated T	4	8953933
ebi-a-GCST90002066	rs13127505	CD4 on activated T	4	141880770
ebi-a-GCST90002066	rs60409747	CD4 on activated T	5	39751811
ebi-a-GCST90002066	rs73800736	CD4 on activated T	5	169629109
ebi-a-GCST90002066	rs831657	CD4 on activated T	5	16286973
ebi-a-GCST90002066	rs3130215	CD4 on activated T	6	33074963
ebi-a-GCST90002066	rs9264532	CD4 on activated T	6	31234381
ebi-a-GCST90002066	rs9266064	CD4 on activated T	6	31319457
ebi-a-GCST90002066	rs7744589	CD4 on activated T	6	6051514
ebi-a-GCST90002066	rs221124	CD4 on activated T	7	28969250
ebi-a-GCST90002066	rs116421623	CD4 on activated T	8	119192827
ebi-a-GCST90002066	rs6986066	CD4 on activated T	8	75692400
ebi-a-GCST90002066	rs75646069	CD4 on activated T	9	12007250
ebi-a-GCST90002066	rs189810664	CD4 on activated T	9	97001952
ebi-a-GCST90002066	rs79998393	CD4 on activated T	10	25354093

ebi-a-GCST90002066	rs17143738	CD4 on activated T	10	8505721
ebi-a-GCST90002066	rs117421233	CD4 on activated T	10	85630450
ebi-a-GCST90002066	rs117190770	CD4 on activated T	10	86457358
ebi-a-GCST90002066	rs80210412	CD4 on activated T	11	21419333
ebi-a-GCST90002066	rs10507053	CD4 on activated T	12	95703285
ebi-a-GCST90002066	rs11615628	CD4 on activated T	12	6903631
ebi-a-GCST90002066	rs73084602	CD4 on activated T	12	22956864
ebi-a-GCST90002066	rs2299912	CD4 on activated T	14	78012732
ebi-a-GCST90002066	rs9898118	CD4 on activated T	17	77121278
ebi-a-GCST90002067	rs12404655	CD4 on CD39+ acti	1	20943195
ebi-a-GCST90002067	rs1385611	CD4 on CD39+ acti	1	207221409
ebi-a-GCST90002067	rs71420675	CD4 on CD39+ acti	2	196049654
ebi-a-GCST90002067	rs73205076	CD4 on CD39+ acti	3	126057162
ebi-a-GCST90002067	rs139628646	CD4 on CD39+ acti	4	8953933
ebi-a-GCST90002067	rs13127505	CD4 on CD39+ acti	4	141880770
ebi-a-GCST90002067	rs183012056	CD4 on CD39+ acti	4	4711458
ebi-a-GCST90002067	rs10044521	CD4 on CD39+ acti	5	116035707
ebi-a-GCST90002067	rs2253487	CD4 on CD39+ acti	6	31249127
ebi-a-GCST90002067	rs9263475	CD4 on CD39+ acti	6	31050348
ebi-a-GCST90002067	rs1625485	CD4 on CD39+ acti	6	29894389
ebi-a-GCST90002067	rs3757517	CD4 on CD39+ acti	7	8242803
ebi-a-GCST90002067	rs221124	CD4 on CD39+ acti	7	28969250
ebi-a-GCST90002067	rs116421623	CD4 on CD39+ acti	8	119192827
ebi-a-GCST90002067	rs67057217	CD4 on CD39+ acti	8	74255588
ebi-a-GCST90002067	rs149080865	CD4 on CD39+ acti	8	80655797
ebi-a-GCST90002067	rs189810664	CD4 on CD39+ acti	9	97001952
ebi-a-GCST90002067	rs7095711	CD4 on CD39+ acti	10	8507223
ebi-a-GCST90002067	rs11200863	CD4 on CD39+ acti	10	85875791
ebi-a-GCST90002067	rs73457945	CD4 on CD39+ acti	11	21414632
ebi-a-GCST90002067	rs11615628	CD4 on CD39+ acti	12	6903631
ebi-a-GCST90002067	rs73084602	CD4 on CD39+ acti	12	22956864
ebi-a-GCST90002067	rs61954768	CD4 on CD39+ acti	13	54907331
ebi-a-GCST90002067	rs10130428	CD4 on CD39+ acti	14	95659768
ebi-a-GCST90002067	rs4127403	CD4 on CD39+ acti	18	6988423
ebi-a-GCST90002068	rs10911945	CD4 on secreting T	1	186852597
ebi-a-GCST90002068	rs9646681	CD4 on secreting T	2	145671913
ebi-a-GCST90002068	rs12185614	CD4 on secreting T	2	26464408
ebi-a-GCST90002068	rs80349754	CD4 on secreting T	3	193078262
ebi-a-GCST90002068	rs77346685	CD4 on secreting T	3	111025555
ebi-a-GCST90002068	rs76560336	CD4 on secreting T	4	33455497
ebi-a-GCST90002068	rs6898033	CD4 on secreting T	5	172247417
ebi-a-GCST90002068	rs116694668	CD4 on secreting T	5	57174385
ebi-a-GCST90002068	rs6875686	CD4 on secreting T	5	157352502
ebi-a-GCST90002068	rs196695	CD4 on secreting T	6	80154074
ebi-a-GCST90002068	rs571270084	CD4 on secreting T	6	32509457
ebi-a-GCST90002068	rs56216313	CD4 on secreting T	7	141053545
ebi-a-GCST90002068	rs2699463	CD4 on secreting T	7	22050169
ebi-a-GCST90002068	rs1793733	CD4 on secreting T	8	118251913

ebi-a-GCST90002068	rs62520866	CD4 on secreting T	8	124586000
ebi-a-GCST90002068	rs75029212	CD4 on secreting T	9	7364861
ebi-a-GCST90002068	rs79998393	CD4 on secreting T	10	25354093
ebi-a-GCST90002068	rs61839660	CD4 on secreting T	10	6094697
ebi-a-GCST90002068	rs80210412	CD4 on secreting T	11	21419333
ebi-a-GCST90002068	rs11615628	CD4 on secreting T	12	6903631
ebi-a-GCST90002068	rs11610038	CD4 on secreting T	12	4770669
ebi-a-GCST90002068	rs78653337	CD4 on secreting T	13	89576465
ebi-a-GCST90002068	rs112860404	CD4 on secreting T	14	72853129
ebi-a-GCST90002068	rs1840913	CD4 on secreting T	17	52190962
ebi-a-GCST90002068	rs12150088	CD4 on secreting T	17	40564303
ebi-a-GCST90002068	rs28706562	CD4 on secreting T	19	55104921
ebi-a-GCST90002068	rs62111177	CD4 on secreting T	19	13471119
ebi-a-GCST90002068	rs141913575	CD4 on secreting T	21	34716713
ebi-a-GCST90002069	rs11584259	CD4 on CD39+ sec	1	22732386
ebi-a-GCST90002069	rs71644931	CD4 on CD39+ sec	1	238537221
ebi-a-GCST90002069	rs41266967	CD4 on CD39+ sec	2	241529785
ebi-a-GCST90002069	rs139628646	CD4 on CD39+ sec	4	8953933
ebi-a-GCST90002069	rs116694668	CD4 on CD39+ sec	5	57174385
ebi-a-GCST90002069	rs10044521	CD4 on CD39+ sec	5	116035707
ebi-a-GCST90002069	rs9263475	CD4 on CD39+ sec	6	31050348
ebi-a-GCST90002069	rs9265953	CD4 on CD39+ sec	6	31315312
ebi-a-GCST90002069	rs115907820	CD4 on CD39+ sec	7	37068759
ebi-a-GCST90002069	rs149080865	CD4 on CD39+ sec	8	80655797
ebi-a-GCST90002069	rs62568944	CD4 on CD39+ sec	9	87813612
ebi-a-GCST90002069	rs10819527	CD4 on CD39+ sec	9	132242279
ebi-a-GCST90002069	rs7072664	CD4 on CD39+ sec	10	126759321
ebi-a-GCST90002069	rs12778618	CD4 on CD39+ sec	10	95633120
ebi-a-GCST90002069	rs117458836	CD4 on CD39+ sec	10	96773382
ebi-a-GCST90002069	rs10882655	CD4 on CD39+ sec	10	97474371
ebi-a-GCST90002069	rs80014012	CD4 on CD39+ sec	10	86318511
ebi-a-GCST90002069	rs7968358	CD4 on CD39+ sec	12	100033308
ebi-a-GCST90002069	rs73053728	CD4 on CD39+ sec	12	6896546
ebi-a-GCST90002069	rs11610038	CD4 on CD39+ sec	12	4770669
ebi-a-GCST90002069	rs139059955	CD4 on CD39+ sec	13	53615745
ebi-a-GCST90002069	rs55877972	CD4 on CD39+ sec	14	94754259
ebi-a-GCST90002069	rs446663	CD4 on CD39+ sec	16	77670157
ebi-a-GCST90002069	rs136883	CD4 on CD39+ sec	22	45040462
ebi-a-GCST90002070	rs10911945	CD4 on activated &	1	186852597
ebi-a-GCST90002070	rs12039573	CD4 on activated &	1	20941515
ebi-a-GCST90002070	rs41266967	CD4 on activated &	2	241529785
ebi-a-GCST90002070	rs139628646	CD4 on activated &	4	8953933
ebi-a-GCST90002070	rs78697264	CD4 on activated &	4	39277538
ebi-a-GCST90002070	rs60409747	CD4 on activated &	5	39751811
ebi-a-GCST90002070	rs6875686	CD4 on activated &	5	157352502
ebi-a-GCST90002070	rs831657	CD4 on activated &	5	16286973
ebi-a-GCST90002070	rs442391	CD4 on activated &	6	31681289
ebi-a-GCST90002070	rs7744589	CD4 on activated &	6	6051514

ebi-a-GCST90002070	rs9264532	CD4 on activated &	6	31234381
ebi-a-GCST90002070	rs12529806	CD4 on activated &	6	108416847
ebi-a-GCST90002070	rs150643215	CD4 on activated &	7	20291018
ebi-a-GCST90002070	rs56216313	CD4 on activated &	7	141053545
ebi-a-GCST90002070	rs116421623	CD4 on activated &	8	119192827
ebi-a-GCST90002070	rs16938946	CD4 on activated &	8	75687887
ebi-a-GCST90002070	rs189810664	CD4 on activated &	9	97001952
ebi-a-GCST90002070	rs74800155	CD4 on activated &	9	24765046
ebi-a-GCST90002070	rs143928375	CD4 on activated &	10	90242054
ebi-a-GCST90002070	rs12772333	CD4 on activated &	10	107779867
ebi-a-GCST90002070	rs79998393	CD4 on activated &	10	25354093
ebi-a-GCST90002070	rs117190770	CD4 on activated &	10	86457358
ebi-a-GCST90002070	rs117421233	CD4 on activated &	10	85630450
ebi-a-GCST90002070	rs80210412	CD4 on activated &	11	21419333
ebi-a-GCST90002070	rs75040387	CD4 on activated &	11	86960608
ebi-a-GCST90002070	rs11615628	CD4 on activated &	12	6903631
ebi-a-GCST90002070	rs10507053	CD4 on activated &	12	95703285
ebi-a-GCST90002070	rs2299912	CD4 on activated &	14	78012732
ebi-a-GCST90002070	rs72815248	CD4 on activated &	16	54493872
ebi-a-GCST90002070	rs9898118	CD4 on activated &	17	77121278
ebi-a-GCST90002070	rs34154026	CD4 on activated &	17	40564630
ebi-a-GCST90002070	rs7249811	CD4 on activated &	19	55087313
ebi-a-GCST90002070	rs62111177	CD4 on activated &	19	13471119
ebi-a-GCST90002071	rs139529340	SSC-A on myeloid	1	161563554
ebi-a-GCST90002071	rs113138131	SSC-A on myeloid	2	203545127
ebi-a-GCST90002071	rs146844975	SSC-A on myeloid	4	70229142
ebi-a-GCST90002071	rs73018616	SSC-A on myeloid	4	181607963
ebi-a-GCST90002071	rs9265898	SSC-A on myeloid	6	31313377
ebi-a-GCST90002071	rs115780495	SSC-A on myeloid	6	32501627
ebi-a-GCST90002071	rs189299852	SSC-A on myeloid	7	88479003
ebi-a-GCST90002071	rs4977889	SSC-A on myeloid	9	23786433
ebi-a-GCST90002071	rs7032562	SSC-A on myeloid	9	100295921
ebi-a-GCST90002071	rs459007	SSC-A on myeloid	9	98478678
ebi-a-GCST90002071	rs139527316	SSC-A on myeloid	10	104987693
ebi-a-GCST90002071	rs4910742	SSC-A on myeloid	11	5306509
ebi-a-GCST90002071	rs80297185	SSC-A on myeloid	11	5488177
ebi-a-GCST90002071	rs10859795	SSC-A on myeloid	12	95317957
ebi-a-GCST90002071	rs139947059	SSC-A on myeloid	12	25434182
ebi-a-GCST90002071	rs1800973	SSC-A on myeloid	12	69744014
ebi-a-GCST90002071	rs16948856	SSC-A on myeloid	16	78946587
ebi-a-GCST90002071	rs139684430	SSC-A on myeloid	16	5551924
ebi-a-GCST90002071	rs772967	SSC-A on myeloid	16	27859096
ebi-a-GCST90002071	rs11554159	SSC-A on myeloid	19	18285944
ebi-a-GCST90002071	rs58470944	SSC-A on myeloid	20	61416034
ebi-a-GCST90002072	rs12410542	SSC-A on plasmacy	1	77233774
ebi-a-GCST90002072	rs11164360	SSC-A on plasmacy	1	102472108
ebi-a-GCST90002072	rs10193587	SSC-A on plasmacy	2	136925439
ebi-a-GCST90002072	rs71423107	SSC-A on plasmacy	2	242975103

ebi-a-GCST90002072	rs2084848	SSC-A on plasmacy	2	169211909
ebi-a-GCST90002072	rs2903918	SSC-A on plasmacy	4	84157443
ebi-a-GCST90002072	rs13162242	SSC-A on plasmacy	5	74298665
ebi-a-GCST90002072	rs79711662	SSC-A on plasmacy	6	95562104
ebi-a-GCST90002072	rs17791425	SSC-A on plasmacy	6	84970341
ebi-a-GCST90002072	rs80067671	SSC-A on plasmacy	7	24079081
ebi-a-GCST90002072	rs17552904	SSC-A on plasmacy	7	50318308
ebi-a-GCST90002072	rs6987643	SSC-A on plasmacy	8	118028546
ebi-a-GCST90002072	rs537539184	SSC-A on plasmacy	10	42747410
ebi-a-GCST90002072	rs142456232	SSC-A on plasmacy	11	4960640
ebi-a-GCST90002072	rs78736654	SSC-A on plasmacy	11	5544477
ebi-a-GCST90002072	rs776409	SSC-A on plasmacy	12	70018656
ebi-a-GCST90002072	rs9669659	SSC-A on plasmacy	12	52279540
ebi-a-GCST90002072	rs8022420	SSC-A on plasmacy	14	101652466
ebi-a-GCST90002072	rs2923129	SSC-A on plasmacy	16	57726123
ebi-a-GCST90002072	rs71388573	SSC-A on plasmacy	16	4436279
ebi-a-GCST90002072	rs55733613	SSC-A on plasmacy	16	89141723
ebi-a-GCST90002072	rs6564921	SSC-A on plasmacy	16	74142321
ebi-a-GCST90002072	rs6500136	SSC-A on plasmacy	16	60631203
ebi-a-GCST90002072	rs117067704	SSC-A on plasmacy	17	62128083
ebi-a-GCST90002072	rs144843975	SSC-A on plasmacy	17	63066415
ebi-a-GCST90002072	rs722926	SSC-A on plasmacy	17	8677215
ebi-a-GCST90002072	rs2058319	SSC-A on plasmacy	19	56863306
ebi-a-GCST90002072	rs144283125	SSC-A on plasmacy	21	34218354
ebi-a-GCST90002073	rs12129442	SSC-A on monocyt	1	15139272
ebi-a-GCST90002073	rs6761913	SSC-A on monocyt	2	43665245
ebi-a-GCST90002073	rs72928949	SSC-A on monocyt	2	203570761
ebi-a-GCST90002073	rs4663559	SSC-A on monocyt	2	236180958
ebi-a-GCST90002073	rs113146287	SSC-A on monocyt	4	21201947
ebi-a-GCST90002073	rs445	SSC-A on monocyt	7	92408370
ebi-a-GCST90002073	rs75117710	SSC-A on monocyt	7	54774655
ebi-a-GCST90002073	rs11136335	SSC-A on monocyt	8	145003777
ebi-a-GCST90002073	rs148146302	SSC-A on monocyt	12	70360198
ebi-a-GCST90002073	rs17301388	SSC-A on monocyt	12	80271645
ebi-a-GCST90002073	rs80216229	SSC-A on monocyt	12	67839087
ebi-a-GCST90002073	rs10784763	SSC-A on monocyt	12	69524025
ebi-a-GCST90002073	rs1800973	SSC-A on monocyt	12	69744014
ebi-a-GCST90002073	rs75994780	SSC-A on monocyt	12	70949085
ebi-a-GCST90002073	rs1381043	SSC-A on monocyt	12	74806815
ebi-a-GCST90002073	rs1152928	SSC-A on monocyt	12	69247557
ebi-a-GCST90002073	rs117623495	SSC-A on monocyt	12	95459971
ebi-a-GCST90002073	rs144373670	SSC-A on monocyt	14	96537074
ebi-a-GCST90002073	rs4411465	SSC-A on monocyt	15	94187437
ebi-a-GCST90002073	rs73587552	SSC-A on monocyt	16	80912403
ebi-a-GCST90002073	rs59386234	SSC-A on monocyt	17	41715057
ebi-a-GCST90002073	rs181913273	SSC-A on monocyt	18	24264307
ebi-a-GCST90002073	rs11554159	SSC-A on monocyt	19	18285944
ebi-a-GCST90002073	rs151082686	SSC-A on monocyt	19	6847167

ebi-a-GCST90002073	rs487473	SSC-A on monocyt	20	889869
ebi-a-GCST90002073	rs553371437	SSC-A on monocyt	22	16868681
ebi-a-GCST90002074	rs72736734	SSC-A on CD14+ n	1	219869305
ebi-a-GCST90002074	rs10202811	SSC-A on CD14+ n	2	218930120
ebi-a-GCST90002074	rs12478601	SSC-A on CD14+ n	2	43721508
ebi-a-GCST90002074	rs35637068	SSC-A on CD14+ n	2	203467998
ebi-a-GCST90002074	rs10488833	SSC-A on CD14+ n	4	97953756
ebi-a-GCST90002074	rs13231864	SSC-A on CD14+ n	7	14761122
ebi-a-GCST90002074	rs4947752	SSC-A on CD14+ n	7	52979778
ebi-a-GCST90002074	rs445	SSC-A on CD14+ n	7	92408370
ebi-a-GCST90002074	rs12276856	SSC-A on CD14+ n	11	122628500
ebi-a-GCST90002074	rs11228503	SSC-A on CD14+ n	11	68879044
ebi-a-GCST90002074	rs75994780	SSC-A on CD14+ n	12	70949085
ebi-a-GCST90002074	rs57059400	SSC-A on CD14+ n	12	19933239
ebi-a-GCST90002074	rs1152928	SSC-A on CD14+ n	12	69247557
ebi-a-GCST90002074	rs12370645	SSC-A on CD14+ n	12	19824999
ebi-a-GCST90002074	rs7309974	SSC-A on CD14+ n	12	93519967
ebi-a-GCST90002074	rs80216229	SSC-A on CD14+ n	12	67839087
ebi-a-GCST90002074	rs10784763	SSC-A on CD14+ n	12	69524025
ebi-a-GCST90002074	rs1800973	SSC-A on CD14+ n	12	69744014
ebi-a-GCST90002074	rs148146302	SSC-A on CD14+ n	12	70360198
ebi-a-GCST90002074	rs10774278	SSC-A on CD14+ n	12	629655
ebi-a-GCST90002074	rs73175542	SSC-A on CD14+ n	13	47478865
ebi-a-GCST90002074	rs62026668	SSC-A on CD14+ n	15	79868517
ebi-a-GCST90002074	rs62070801	SSC-A on CD14+ n	17	50801881
ebi-a-GCST90002074	rs11554159	SSC-A on CD14+ n	19	18285944
ebi-a-GCST90002074	rs9616832	SSC-A on CD14+ n	22	51219387
ebi-a-GCST90002075	rs116325392	SSC-A on B cell	1	4629608
ebi-a-GCST90002075	rs61180620	SSC-A on B cell	2	36008717
ebi-a-GCST90002075	rs1317900	SSC-A on B cell	2	131607875
ebi-a-GCST90002075	rs6763382	SSC-A on B cell	3	32156283
ebi-a-GCST90002075	rs144261817	SSC-A on B cell	5	40009649
ebi-a-GCST90002075	rs72784338	SSC-A on B cell	5	106222436
ebi-a-GCST90002075	rs32475	SSC-A on B cell	5	171191605
ebi-a-GCST90002075	rs1635750	SSC-A on B cell	7	74360068
ebi-a-GCST90002075	rs11777012	SSC-A on B cell	8	51754521
ebi-a-GCST90002075	rs72879624	SSC-A on B cell	11	5144391
ebi-a-GCST90002075	rs10459209	SSC-A on B cell	12	106623009
ebi-a-GCST90002075	rs139142972	SSC-A on B cell	18	29231102
ebi-a-GCST90002075	rs6134523	SSC-A on B cell	20	12351462
ebi-a-GCST90002075	rs6096232	SSC-A on B cell	20	49637974
ebi-a-GCST90002076	rs7575742	SSC-A on NK	2	135429002
ebi-a-GCST90002076	rs75215140	SSC-A on NK	2	62135307
ebi-a-GCST90002076	rs147596499	SSC-A on NK	4	96489377
ebi-a-GCST90002076	rs6850820	SSC-A on NK	4	149325342
ebi-a-GCST90002076	rs77154024	SSC-A on NK	4	72546264
ebi-a-GCST90002076	rs6863338	SSC-A on NK	5	127586596
ebi-a-GCST90002076	rs79025422	SSC-A on NK	5	80236683

ebi-a-GCST90002076	rs188107682	SSC-A on NK	5	170062959
ebi-a-GCST90002076	rs767896	SSC-A on NK	6	33791515
ebi-a-GCST90002076	rs2915158	SSC-A on NK	7	9912333
ebi-a-GCST90002076	rs1006368	SSC-A on NK	10	126346603
ebi-a-GCST90002076	rs2198787	SSC-A on NK	12	62296066
ebi-a-GCST90002076	rs12594783	SSC-A on NK	15	88040400
ebi-a-GCST90002076	rs74856421	SSC-A on NK	16	3773110
ebi-a-GCST90002076	rs9912354	SSC-A on NK	17	33805730
ebi-a-GCST90002076	rs10512469	SSC-A on NK	17	33770802
ebi-a-GCST90002076	rs11081793	SSC-A on NK	18	19722536
ebi-a-GCST90002076	rs72901909	SSC-A on NK	18	39079198
ebi-a-GCST90002076	rs2869804	SSC-A on NK	18	14141133
ebi-a-GCST90002076	rs17606146	SSC-A on NK	19	3631134
ebi-a-GCST90002076	rs28691703	SSC-A on NK	19	43649306
ebi-a-GCST90002076	rs35150190	SSC-A on NK	19	40286227
ebi-a-GCST90002076	rs873220	SSC-A on NK	21	33382162
ebi-a-GCST90002076	rs11703193	SSC-A on NK	22	18542023
ebi-a-GCST90002077	rs569911	SSC-A on HLA DR	1	160798209
ebi-a-GCST90002077	rs115805162	SSC-A on HLA DR	1	161410253
ebi-a-GCST90002077	rs71632979	SSC-A on HLA DR	1	161526397
ebi-a-GCST90002077	rs183249728	SSC-A on HLA DR	1	158205680
ebi-a-GCST90002077	rs72698314	SSC-A on HLA DR	1	164795440
ebi-a-GCST90002077	rs4970518	SSC-A on HLA DR	1	27647082
ebi-a-GCST90002077	rs116660215	SSC-A on HLA DR	2	17527024
ebi-a-GCST90002077	rs11891318	SSC-A on HLA DR	2	130608376
ebi-a-GCST90002077	rs333491	SSC-A on HLA DR	3	78808850
ebi-a-GCST90002077	rs112085672	SSC-A on HLA DR	3	182309260
ebi-a-GCST90002077	rs6847175	SSC-A on HLA DR	4	88481387
ebi-a-GCST90002077	rs77898356	SSC-A on HLA DR	6	147107666
ebi-a-GCST90002077	rs12524004	SSC-A on HLA DR	6	144027987
ebi-a-GCST90002077	rs11980037	SSC-A on HLA DR	7	88594684
ebi-a-GCST90002077	rs6977893	SSC-A on HLA DR	7	130890843
ebi-a-GCST90002077	rs140589274	SSC-A on HLA DR	9	138894488
ebi-a-GCST90002077	rs7859270	SSC-A on HLA DR	9	116135261
ebi-a-GCST90002077	rs11000435	SSC-A on HLA DR	10	74638979
ebi-a-GCST90002077	rs117691019	SSC-A on HLA DR	11	78351106
ebi-a-GCST90002077	rs2896705	SSC-A on HLA DR	11	24553602
ebi-a-GCST90002077	rs148866760	SSC-A on HLA DR	11	4509339
ebi-a-GCST90002077	rs41473449	SSC-A on HLA DR	12	125621419
ebi-a-GCST90002077	rs142321106	SSC-A on HLA DR	13	96037026
ebi-a-GCST90002077	rs11639511	SSC-A on HLA DR	16	10977844
ebi-a-GCST90002077	rs7206658	SSC-A on HLA DR	16	68916118
ebi-a-GCST90002077	rs142666685	SSC-A on HLA DR	17	1769354
ebi-a-GCST90002077	rs2155742	SSC-A on HLA DR	18	13952635
ebi-a-GCST90002077	rs7292569	SSC-A on HLA DR	22	27418259
ebi-a-GCST90002078	rs112900587	SSC-A on granuloc	2	195082880
ebi-a-GCST90002078	rs56200756	SSC-A on granuloc	2	218938244
ebi-a-GCST90002078	rs58700409	SSC-A on granuloc	2	43347932

ebi-a-GCST90002078	rs10172031	SSC-A on granuloc	2	235450958
ebi-a-GCST90002078	rs3774304	SSC-A on granuloc	3	187454731
ebi-a-GCST90002078	rs2236947	SSC-A on granuloc	3	50371432
ebi-a-GCST90002078	rs11568958	SSC-A on granuloc	4	110884941
ebi-a-GCST90002078	rs114254337	SSC-A on granuloc	5	173631361
ebi-a-GCST90002078	rs75752883	SSC-A on granuloc	6	155636964
ebi-a-GCST90002078	rs72984093	SSC-A on granuloc	6	136080855
ebi-a-GCST90002078	rs42031	SSC-A on granuloc	7	92237396
ebi-a-GCST90002078	rs445	SSC-A on granuloc	7	92408370
ebi-a-GCST90002078	rs17346868	SSC-A on granuloc	8	77468435
ebi-a-GCST90002078	rs2738144	SSC-A on granuloc	8	6826043
ebi-a-GCST90002078	rs117932393	SSC-A on granuloc	9	109152709
ebi-a-GCST90002078	rs17775854	SSC-A on granuloc	10	115676623
ebi-a-GCST90002078	rs1533765	SSC-A on granuloc	11	75901019
ebi-a-GCST90002078	rs137910312	SSC-A on granuloc	11	78834505
ebi-a-GCST90002078	rs12286701	SSC-A on granuloc	11	31863361
ebi-a-GCST90002078	rs12788263	SSC-A on granuloc	11	22490590
ebi-a-GCST90002078	rs473765	SSC-A on granuloc	11	100829176
ebi-a-GCST90002078	rs28620656	SSC-A on granuloc	12	22247373
ebi-a-GCST90002078	rs9567796	SSC-A on granuloc	13	47738561
ebi-a-GCST90002078	rs182697774	SSC-A on granuloc	15	53722503
ebi-a-GCST90002078	rs12906244	SSC-A on granuloc	15	72199230
ebi-a-GCST90002078	rs1444301	SSC-A on granuloc	15	86683981
ebi-a-GCST90002078	rs12459815	SSC-A on granuloc	19	52142313
ebi-a-GCST90002078	rs4808397	SSC-A on granuloc	19	15977304
ebi-a-GCST90002079	rs115752428	SSC-A on lymphoc	1	205560814
ebi-a-GCST90002079	rs150148686	SSC-A on lymphoc	1	214244868
ebi-a-GCST90002079	rs10169473	SSC-A on lymphoc	2	224307687
ebi-a-GCST90002079	rs763957	SSC-A on lymphoc	2	72047819
ebi-a-GCST90002079	rs4476566	SSC-A on lymphoc	4	92685794
ebi-a-GCST90002079	rs140946844	SSC-A on lymphoc	4	126113940
ebi-a-GCST90002079	rs9268832	SSC-A on lymphoc	6	32427789
ebi-a-GCST90002079	rs79281322	SSC-A on lymphoc	6	32621047
ebi-a-GCST90002079	rs33964890	SSC-A on lymphoc	6	32583919
ebi-a-GCST90002079	rs567832763	SSC-A on lymphoc	7	5876741
ebi-a-GCST90002079	rs1842502	SSC-A on lymphoc	8	136208763
ebi-a-GCST90002079	rs776782	SSC-A on lymphoc	9	18653773
ebi-a-GCST90002079	rs112485823	SSC-A on lymphoc	10	10530822
ebi-a-GCST90002079	rs3907999	SSC-A on lymphoc	11	82027114
ebi-a-GCST90002079	rs78270456	SSC-A on lymphoc	11	5229028
ebi-a-GCST90002079	rs11607999	SSC-A on lymphoc	11	44623672
ebi-a-GCST90002079	rs4883958	SSC-A on lymphoc	13	74511054
ebi-a-GCST90002079	rs77789294	SSC-A on lymphoc	14	29178686
ebi-a-GCST90002079	rs8029959	SSC-A on lymphoc	15	26028154
ebi-a-GCST90002079	rs873857	SSC-A on lymphoc	16	84697706
ebi-a-GCST90002079	rs1823592	SSC-A on lymphoc	18	22484814
ebi-a-GCST90002079	rs12166583	SSC-A on lymphoc	22	30483964
ebi-a-GCST90002080	rs75643253	SSC-A on T cell	1	27415326

ebi-a-GCST90002080	rs61768373	SSC-A on T cell	1	44148499
ebi-a-GCST90002080	rs115752428	SSC-A on T cell	1	205560814
ebi-a-GCST90002080	rs4654566	SSC-A on T cell	1	4657087
ebi-a-GCST90002080	rs763957	SSC-A on T cell	2	72047819
ebi-a-GCST90002080	rs12635110	SSC-A on T cell	3	129503109
ebi-a-GCST90002080	rs9878926	SSC-A on T cell	3	103913196
ebi-a-GCST90002080	rs112262410	SSC-A on T cell	3	134400604
ebi-a-GCST90002080	rs141625995	SSC-A on T cell	4	5686368
ebi-a-GCST90002080	rs72634890	SSC-A on T cell	4	58645104
ebi-a-GCST90002080	rs5004278	SSC-A on T cell	6	32582401
ebi-a-GCST90002080	rs261949	SSC-A on T cell	6	30270080
ebi-a-GCST90002080	rs1842502	SSC-A on T cell	8	136208763
ebi-a-GCST90002080	rs143674649	SSC-A on T cell	8	24246646
ebi-a-GCST90002080	rs776782	SSC-A on T cell	9	18653773
ebi-a-GCST90002080	rs11607999	SSC-A on T cell	11	44623672
ebi-a-GCST90002080	rs77789294	SSC-A on T cell	14	29178686
ebi-a-GCST90002080	rs2188353	SSC-A on T cell	16	26444828
ebi-a-GCST90002080	rs873857	SSC-A on T cell	16	84697706
ebi-a-GCST90002080	rs1823592	SSC-A on T cell	18	22484814
ebi-a-GCST90002080	rs76782803	SSC-A on T cell	20	23665192
ebi-a-GCST90002080	rs5749002	SSC-A on T cell	22	17749096
ebi-a-GCST90002080	rs113026874	SSC-A on T cell	22	30400641
ebi-a-GCST90002081	rs145630857	SSC-A on CD4+	2	1273653
ebi-a-GCST90002081	rs10169473	SSC-A on CD4+	2	224307687
ebi-a-GCST90002081	rs2625989	SSC-A on CD4+	3	129345155
ebi-a-GCST90002081	rs62274365	SSC-A on CD4+	3	103225168
ebi-a-GCST90002081	rs114690517	SSC-A on CD4+	3	116324393
ebi-a-GCST90002081	rs139637230	SSC-A on CD4+	3	75966167
ebi-a-GCST90002081	rs77201915	SSC-A on CD4+	3	165438990
ebi-a-GCST90002081	rs112057082	SSC-A on CD4+	6	31875523
ebi-a-GCST90002081	rs9351713	SSC-A on CD4+	6	69075736
ebi-a-GCST90002081	rs9271536	SSC-A on CD4+	6	32589978
ebi-a-GCST90002081	rs113243185	SSC-A on CD4+	6	32585071
ebi-a-GCST90002081	rs79493037	SSC-A on CD4+	6	31405825
ebi-a-GCST90002081	rs2245214	SSC-A on CD4+	6	106662741
ebi-a-GCST90002081	rs148031710	SSC-A on CD4+	6	31148873
ebi-a-GCST90002081	rs12704990	SSC-A on CD4+	7	77093722
ebi-a-GCST90002081	rs1842502	SSC-A on CD4+	8	136208763
ebi-a-GCST90002081	rs10975141	SSC-A on CD4+	9	5496918
ebi-a-GCST90002081	rs62577878	SSC-A on CD4+	9	104134117
ebi-a-GCST90002081	rs2184012	SSC-A on CD4+	9	114152636
ebi-a-GCST90002081	rs776782	SSC-A on CD4+	9	18653773
ebi-a-GCST90002081	rs12356750	SSC-A on CD4+	10	7450221
ebi-a-GCST90002081	rs67169771	SSC-A on CD4+	10	133344600
ebi-a-GCST90002081	rs117236744	SSC-A on CD4+	11	13091906
ebi-a-GCST90002081	rs140567398	SSC-A on CD4+	13	99878398
ebi-a-GCST90002081	rs75548299	SSC-A on CD4+	14	101441602
ebi-a-GCST90002081	rs79312638	SSC-A on CD4+	15	84477705

ebi-a-GCST90002081	rs1994719	SSC-A on CD4+	17	72945894
ebi-a-GCST90002081	rs11078501	SSC-A on CD4+	17	4287261
ebi-a-GCST90002081	rs1823592	SSC-A on CD4+	18	22484814
ebi-a-GCST90002081	rs80099003	SSC-A on CD4+	19	34352141
ebi-a-GCST90002081	rs12166583	SSC-A on CD4+	22	30483964
ebi-a-GCST90002082	rs115752428	SSC-A on CD8br	1	205560814
ebi-a-GCST90002082	rs13019266	SSC-A on CD8br	2	111604642
ebi-a-GCST90002082	rs112262410	SSC-A on CD8br	3	134400604
ebi-a-GCST90002082	rs4550979	SSC-A on CD8br	4	189591165
ebi-a-GCST90002082	rs62435615	SSC-A on CD8br	6	147532536
ebi-a-GCST90002082	rs9266801	SSC-A on CD8br	6	31376051
ebi-a-GCST90002082	rs147779398	SSC-A on CD8br	7	42610990
ebi-a-GCST90002082	rs144027086	SSC-A on CD8br	7	15148913
ebi-a-GCST90002082	rs11192024	SSC-A on CD8br	10	106126407
ebi-a-GCST90002082	rs147187095	SSC-A on CD8br	10	9454654
ebi-a-GCST90002082	rs77789294	SSC-A on CD8br	14	29178686
ebi-a-GCST90002082	rs873857	SSC-A on CD8br	16	84697706
ebi-a-GCST90002082	rs7192172	SSC-A on CD8br	16	57127753
ebi-a-GCST90002082	rs138038426	SSC-A on CD8br	17	42576434
ebi-a-GCST90002082	rs191772295	SSC-A on CD8br	18	21222946
ebi-a-GCST90002082	rs72943263	SSC-A on CD8br	18	54275565
ebi-a-GCST90002082	rs117482121	SSC-A on CD8br	18	75269360
ebi-a-GCST90002082	rs62221356	SSC-A on CD8br	22	49522943
ebi-a-GCST90002082	rs5762053	SSC-A on CD8br	22	27675461
ebi-a-GCST90002083	rs11801958	SSC-A on HLA DR	1	7176679
ebi-a-GCST90002083	rs4971333	SSC-A on HLA DR	2	931745
ebi-a-GCST90002083	rs4686701	SSC-A on HLA DR	3	185550565
ebi-a-GCST90002083	rs34705953	SSC-A on HLA DR	3	19997111
ebi-a-GCST90002083	rs35775925	SSC-A on HLA DR	3	71332550
ebi-a-GCST90002083	rs115620709	SSC-A on HLA DR	4	148953372
ebi-a-GCST90002083	rs34001932	SSC-A on HLA DR	5	133304889
ebi-a-GCST90002083	rs16901165	SSC-A on HLA DR	5	31439859
ebi-a-GCST90002083	rs7740132	SSC-A on HLA DR	6	89807320
ebi-a-GCST90002083	rs140095418	SSC-A on HLA DR	6	120793422
ebi-a-GCST90002083	rs9272259	SSC-A on HLA DR	6	32603192
ebi-a-GCST90002083	rs9267655	SSC-A on HLA DR	6	31841055
ebi-a-GCST90002083	rs56012404	SSC-A on HLA DR	7	148180566
ebi-a-GCST90002083	rs2374643	SSC-A on HLA DR	7	93045939
ebi-a-GCST90002083	rs186882253	SSC-A on HLA DR	9	101270987
ebi-a-GCST90002083	rs10742994	SSC-A on HLA DR	11	1810188
ebi-a-GCST90002083	rs2683466	SSC-A on HLA DR	12	32210668
ebi-a-GCST90002083	rs4597229	SSC-A on HLA DR	14	38400344
ebi-a-GCST90002083	rs4924305	SSC-A on HLA DR	15	39159349
ebi-a-GCST90002083	rs114959443	SSC-A on HLA DR	15	35835193
ebi-a-GCST90002083	rs3813581	SSC-A on HLA DR	16	79749341
ebi-a-GCST90002083	rs12924236	SSC-A on HLA DR	16	10974423
ebi-a-GCST90002083	rs8089887	SSC-A on HLA DR	18	67573810
ebi-a-GCST90002084	rs10749775	SSC-A on NKT	1	67822087

ebi-a-GCST90002084	rs6694654	SSC-A on NKT	1	86178676
ebi-a-GCST90002084	rs138822248	SSC-A on NKT	2	152970607
ebi-a-GCST90002084	rs6783507	SSC-A on NKT	3	123518258
ebi-a-GCST90002084	rs79523120	SSC-A on NKT	6	66414139
ebi-a-GCST90002084	rs6918518	SSC-A on NKT	6	158930696
ebi-a-GCST90002084	rs4707956	SSC-A on NKT	6	72648340
ebi-a-GCST90002084	rs2109396	SSC-A on NKT	7	14933955
ebi-a-GCST90002084	rs35060274	SSC-A on NKT	8	58338298
ebi-a-GCST90002084	rs12716653	SSC-A on NKT	9	89886037
ebi-a-GCST90002084	rs1006368	SSC-A on NKT	10	126346603
ebi-a-GCST90002084	rs10765790	SSC-A on NKT	11	95852734
ebi-a-GCST90002084	rs78201778	SSC-A on NKT	12	45872353
ebi-a-GCST90002084	rs2198787	SSC-A on NKT	12	62296066
ebi-a-GCST90002084	rs55657662	SSC-A on NKT	13	102674496
ebi-a-GCST90002084	rs118042158	SSC-A on NKT	13	97456459
ebi-a-GCST90002084	rs7183698	SSC-A on NKT	15	64870152
ebi-a-GCST90002084	rs276951	SSC-A on NKT	16	86243187
ebi-a-GCST90002084	rs11644532	SSC-A on NKT	16	70657660
ebi-a-GCST90002084	rs140151332	SSC-A on NKT	17	18368011
ebi-a-GCST90002084	rs74777495	SSC-A on NKT	18	58910278
ebi-a-GCST90002084	rs10422871	SSC-A on NKT	19	8789227
ebi-a-GCST90002085	rs79811607	SSC-A on HLA DR	1	111760061
ebi-a-GCST90002085	rs4326672	SSC-A on HLA DR	1	95581713
ebi-a-GCST90002085	rs6697448	SSC-A on HLA DR	1	12594521
ebi-a-GCST90002085	rs3115859	SSC-A on HLA DR	1	754503
ebi-a-GCST90002085	rs138424232	SSC-A on HLA DR	1	248075249
ebi-a-GCST90002085	rs4686701	SSC-A on HLA DR	3	185550565
ebi-a-GCST90002085	rs13159652	SSC-A on HLA DR	5	86130997
ebi-a-GCST90002085	rs115781665	SSC-A on HLA DR	6	67256822
ebi-a-GCST90002085	rs3104376	SSC-A on HLA DR	6	32600003
ebi-a-GCST90002085	rs78131752	SSC-A on HLA DR	6	63218235
ebi-a-GCST90002085	rs78800399	SSC-A on HLA DR	7	25515946
ebi-a-GCST90002085	rs7805250	SSC-A on HLA DR	7	32126481
ebi-a-GCST90002085	rs151184302	SSC-A on HLA DR	7	76839222
ebi-a-GCST90002085	rs72679223	SSC-A on HLA DR	8	96005870
ebi-a-GCST90002085	rs138293681	SSC-A on HLA DR	9	19994802
ebi-a-GCST90002085	rs353628	SSC-A on HLA DR	11	35188507
ebi-a-GCST90002085	rs117045668	SSC-A on HLA DR	11	21178226
ebi-a-GCST90002085	rs4941916	SSC-A on HLA DR	13	39834151
ebi-a-GCST90002085	rs4924305	SSC-A on HLA DR	15	39159349
ebi-a-GCST90002085	rs6498113	SSC-A on HLA DR	16	10961882
ebi-a-GCST90002085	rs79890386	SSC-A on HLA DR	19	18006749
ebi-a-GCST90002085	rs12482528	SSC-A on HLA DR	21	15710293
ebi-a-GCST90002085	rs11910199	SSC-A on HLA DR	21	31589957
ebi-a-GCST90002086	rs142804909	SSC-A on HLA DR	1	64510605
ebi-a-GCST90002086	rs11801958	SSC-A on HLA DR	1	7176679
ebi-a-GCST90002086	rs114955332	SSC-A on HLA DR	1	77859645
ebi-a-GCST90002086	rs62161922	SSC-A on HLA DR	2	34784052

ebi-a-GCST90002086	rs4273217	SSC-A on HLA DR	2	152638498
ebi-a-GCST90002086	rs4686701	SSC-A on HLA DR	3	185550565
ebi-a-GCST90002086	rs78160435	SSC-A on HLA DR	4	164937109
ebi-a-GCST90002086	rs4833232	SSC-A on HLA DR	4	122684367
ebi-a-GCST90002086	rs614008	SSC-A on HLA DR	6	31840794
ebi-a-GCST90002086	rs3129034	SSC-A on HLA DR	6	29555809
ebi-a-GCST90002086	rs117967604	SSC-A on HLA DR	7	154651450
ebi-a-GCST90002086	rs74843612	SSC-A on HLA DR	7	9681264
ebi-a-GCST90002086	rs76157970	SSC-A on HLA DR	9	7325127
ebi-a-GCST90002086	rs2147883	SSC-A on HLA DR	10	68734898
ebi-a-GCST90002086	rs78335055	SSC-A on HLA DR	12	87425162
ebi-a-GCST90002086	rs74827160	SSC-A on HLA DR	12	88353146
ebi-a-GCST90002086	rs75133898	SSC-A on HLA DR	12	124981979
ebi-a-GCST90002086	rs1552390	SSC-A on HLA DR	12	13227095
ebi-a-GCST90002086	rs117767550	SSC-A on HLA DR	12	98809414
ebi-a-GCST90002086	rs75242390	SSC-A on HLA DR	13	107412166
ebi-a-GCST90002086	rs10145270	SSC-A on HLA DR	14	105349891
ebi-a-GCST90002086	rs117410119	SSC-A on HLA DR	15	89996482
ebi-a-GCST90002086	rs79260068	SSC-A on HLA DR	17	11304316
ebi-a-GCST90002086	rs1017102	SSC-A on HLA DR	19	56892632
ebi-a-GCST90002087	rs17481824	CD11c on myeloid	1	54559794
ebi-a-GCST90002087	rs72873714	CD11c on myeloid	1	25875650
ebi-a-GCST90002087	rs78520150	CD11c on myeloid	1	19786101
ebi-a-GCST90002087	rs71652355	CD11c on myeloid	1	25505556
ebi-a-GCST90002087	rs61802329	CD11c on myeloid	1	161589626
ebi-a-GCST90002087	rs72662658	CD11c on myeloid	1	26010884
ebi-a-GCST90002087	rs79355481	CD11c on myeloid	1	40087162
ebi-a-GCST90002087	rs79968431	CD11c on myeloid	2	67456752
ebi-a-GCST90002087	rs75841521	CD11c on myeloid	2	59592691
ebi-a-GCST90002087	rs11708131	CD11c on myeloid	3	104415905
ebi-a-GCST90002087	rs9856506	CD11c on myeloid	3	8718629
ebi-a-GCST90002087	rs72732223	CD11c on myeloid	5	16754930
ebi-a-GCST90002087	rs778233	CD11c on myeloid	5	84617476
ebi-a-GCST90002087	rs16881169	CD11c on myeloid	6	18769716
ebi-a-GCST90002087	rs56210513	CD11c on myeloid	6	112990257
ebi-a-GCST90002087	rs7795869	CD11c on myeloid	7	36710341
ebi-a-GCST90002087	rs13255633	CD11c on myeloid	8	138231016
ebi-a-GCST90002087	rs7869020	CD11c on myeloid	9	138107011
ebi-a-GCST90002087	rs2541232	CD11c on myeloid	10	72520930
ebi-a-GCST90002087	rs11004890	CD11c on myeloid	10	57257180
ebi-a-GCST90002087	rs148343003	CD11c on myeloid	10	101863324
ebi-a-GCST90002087	rs138069865	CD11c on myeloid	10	115183723
ebi-a-GCST90002087	rs4770411	CD11c on myeloid	13	23783140
ebi-a-GCST90002087	rs9562128	CD11c on myeloid	13	114917127
ebi-a-GCST90002087	rs67898294	CD11c on myeloid	16	31362711
ebi-a-GCST90002087	rs80318951	CD11c on myeloid	16	30829668
ebi-a-GCST90002087	rs113167560	CD11c on myeloid	16	51444521
ebi-a-GCST90002087	rs79298699	CD11c on myeloid	16	31402042

ebi-a-GCST90002087	rs113404848	CD11c on myeloid	16	48040615
ebi-a-GCST90002087	rs35945038	CD11c on myeloid	18	20245248
ebi-a-GCST90002087	rs56116087	CD11c on myeloid	19	10090323
ebi-a-GCST90002088	rs79355481	CD11c on CD62L+	1	40087162
ebi-a-GCST90002088	rs17481824	CD11c on CD62L+	1	54559794
ebi-a-GCST90002088	rs78520150	CD11c on CD62L+	1	19786101
ebi-a-GCST90002088	rs72662658	CD11c on CD62L+	1	26010884
ebi-a-GCST90002088	rs72873714	CD11c on CD62L+	1	25875650
ebi-a-GCST90002088	rs185038925	CD11c on CD62L+	1	238659551
ebi-a-GCST90002088	rs71652355	CD11c on CD62L+	1	25505556
ebi-a-GCST90002088	rs149922594	CD11c on CD62L+	2	24117291
ebi-a-GCST90002088	rs75841521	CD11c on CD62L+	2	59592691
ebi-a-GCST90002088	rs17314359	CD11c on CD62L+	2	83597897
ebi-a-GCST90002088	rs9856506	CD11c on CD62L+	3	8718629
ebi-a-GCST90002088	rs72672205	CD11c on CD62L+	4	111950141
ebi-a-GCST90002088	rs115914905	CD11c on CD62L+	4	6588450
ebi-a-GCST90002088	rs778233	CD11c on CD62L+	5	84617476
ebi-a-GCST90002088	rs72732223	CD11c on CD62L+	5	16754930
ebi-a-GCST90002088	rs79792171	CD11c on CD62L+	6	124596494
ebi-a-GCST90002088	rs12529747	CD11c on CD62L+	6	112955454
ebi-a-GCST90002088	rs16881169	CD11c on CD62L+	6	18769716
ebi-a-GCST90002088	rs13255633	CD11c on CD62L+	8	138231016
ebi-a-GCST90002088	rs148343003	CD11c on CD62L+	10	101863324
ebi-a-GCST90002088	rs2541232	CD11c on CD62L+	10	72520930
ebi-a-GCST90002088	rs138069865	CD11c on CD62L+	10	115183723
ebi-a-GCST90002088	rs1887757	CD11c on CD62L+	13	23788362
ebi-a-GCST90002088	rs12927491	CD11c on CD62L+	16	31596543
ebi-a-GCST90002088	rs76225503	CD11c on CD62L+	16	47980553
ebi-a-GCST90002088	rs4493054	CD11c on CD62L+	16	31361988
ebi-a-GCST90002088	rs113404848	CD11c on CD62L+	16	48040615
ebi-a-GCST90002088	rs4592764	CD11c on CD62L+	18	20258482
ebi-a-GCST90002089	rs16827406	CD11c on monocyt	1	41059499
ebi-a-GCST90002089	rs1801274	CD11c on monocyt	1	161479745
ebi-a-GCST90002089	rs74585014	CD11c on monocyt	2	75208727
ebi-a-GCST90002089	rs2699961	CD11c on monocyt	3	109042419
ebi-a-GCST90002089	rs149159018	CD11c on monocyt	3	132077351
ebi-a-GCST90002089	rs35764061	CD11c on monocyt	7	73285441
ebi-a-GCST90002089	rs116977022	CD11c on monocyt	8	131045527
ebi-a-GCST90002089	rs146665828	CD11c on monocyt	8	15677874
ebi-a-GCST90002089	rs117387675	CD11c on monocyt	9	105955248
ebi-a-GCST90002089	rs7118182	CD11c on monocyt	11	7445905
ebi-a-GCST90002089	rs12421743	CD11c on monocyt	11	124765894
ebi-a-GCST90002089	rs11608886	CD11c on monocyt	12	105713710
ebi-a-GCST90002089	rs1887757	CD11c on monocyt	13	23788362
ebi-a-GCST90002089	rs1808890	CD11c on monocyt	15	101227478
ebi-a-GCST90002089	rs9932817	CD11c on monocyt	16	48503912
ebi-a-GCST90002089	rs117968915	CD11c on monocyt	16	47869527
ebi-a-GCST90002089	rs11642757	CD11c on monocyt	16	79753467

ebi-a-GCST90002089	rs7199570	CD11c on monocyt	16	31365464
ebi-a-GCST90002089	rs12924903	CD11c on monocyt	16	30928970
ebi-a-GCST90002089	rs116873976	CD11c on monocyt	17	21255388
ebi-a-GCST90002089	rs77687616	CD11c on monocyt	20	54392972
ebi-a-GCST90002089	rs11913696	CD11c on monocyt	22	19827328
ebi-a-GCST90002090	rs1801274	CD11c on granuloc	1	161479745
ebi-a-GCST90002090	rs114393604	CD11c on granuloc	1	9765679
ebi-a-GCST90002090	rs144638810	CD11c on granuloc	1	161968404
ebi-a-GCST90002090	rs12122013	CD11c on granuloc	1	162860370
ebi-a-GCST90002090	rs35852864	CD11c on granuloc	1	181217088
ebi-a-GCST90002090	rs55828113	CD11c on granuloc	2	195660273
ebi-a-GCST90002090	rs11719519	CD11c on granuloc	3	186104558
ebi-a-GCST90002090	rs79027362	CD11c on granuloc	4	72155939
ebi-a-GCST90002090	rs13110782	CD11c on granuloc	4	186808581
ebi-a-GCST90002090	rs34744703	CD11c on granuloc	4	188861213
ebi-a-GCST90002090	rs138075266	CD11c on granuloc	7	28584086
ebi-a-GCST90002090	rs2026075	CD11c on granuloc	9	520886
ebi-a-GCST90002090	rs4743771	CD11c on granuloc	9	107722282
ebi-a-GCST90002090	rs1231330	CD11c on granuloc	9	25717386
ebi-a-GCST90002090	rs10756625	CD11c on granuloc	9	1501109
ebi-a-GCST90002090	rs78304446	CD11c on granuloc	10	133662338
ebi-a-GCST90002090	rs11841867	CD11c on granuloc	13	29022875
ebi-a-GCST90002090	rs16947237	CD11c on granuloc	13	92923296
ebi-a-GCST90002090	rs9671321	CD11c on granuloc	14	23151953
ebi-a-GCST90002090	rs139788044	CD11c on granuloc	15	24527620
ebi-a-GCST90002090	rs28432694	CD11c on granuloc	16	48666310
ebi-a-GCST90002090	rs9923916	CD11c on granuloc	16	1322605
ebi-a-GCST90002090	rs8046381	CD11c on granuloc	16	31379355
ebi-a-GCST90002090	rs321911	CD11c on granuloc	19	52811620
ebi-a-GCST90002090	rs113861918	CD11c on granuloc	19	52129097
ebi-a-GCST90002090	rs117596617	CD11c on granuloc	20	52794798
ebi-a-GCST90002091	rs3813611	CD11b on CD14+ r	1	161068240
ebi-a-GCST90002091	rs1801274	CD11b on CD14+ r	1	161479745
ebi-a-GCST90002091	rs12073174	CD11b on CD14+ r	1	4002766
ebi-a-GCST90002091	rs80051878	CD11b on CD14+ r	1	87979893
ebi-a-GCST90002091	rs80033019	CD11b on CD14+ r	2	122609228
ebi-a-GCST90002091	rs148027358	CD11b on CD14+ r	4	190410040
ebi-a-GCST90002091	rs7341115	CD11b on CD14+ r	5	90510809
ebi-a-GCST90002091	rs142164490	CD11b on CD14+ r	6	102083682
ebi-a-GCST90002091	rs56063308	CD11b on CD14+ r	6	91511685
ebi-a-GCST90002091	rs6591744	CD11b on CD14+ r	11	62880909
ebi-a-GCST90002091	rs142456232	CD11b on CD14+ r	11	4960640
ebi-a-GCST90002091	rs11232457	CD11b on CD14+ r	11	80812245
ebi-a-GCST90002091	rs285589	CD11b on CD14+ r	12	41944407
ebi-a-GCST90002091	rs12147583	CD11b on CD14+ r	14	87896781
ebi-a-GCST90002091	rs112645067	CD11b on CD14+ r	15	99970786
ebi-a-GCST90002091	rs62032592	CD11b on CD14+ r	15	87411994
ebi-a-GCST90002091	rs62077141	CD11b on CD14+ r	17	75798962

ebi-a-GCST90002091	rs60887432	CD11b on CD14+ r	17	70177652
ebi-a-GCST90002091	rs72835003	CD11b on CD14+ r	17	4714471
ebi-a-GCST90002091	rs7268939	CD11b on CD14+ r	20	18896895
ebi-a-GCST90002092	rs78378365	CD11b on Gr MDS	1	79154051
ebi-a-GCST90002092	rs140875293	CD11b on Gr MDS	2	190360243
ebi-a-GCST90002092	rs147394990	CD11b on Gr MDS	4	144217222
ebi-a-GCST90002093	rs3905243	CD11b on CD66b+	1	115355974
ebi-a-GCST90002093	rs35028965	CD11b on CD66b+	2	213535240
ebi-a-GCST90002093	rs115257843	CD11b on CD66b+	2	25875950
ebi-a-GCST90002093	rs12471268	CD11b on CD66b+	2	153464633
ebi-a-GCST90002093	rs73218196	CD11b on CD66b+	4	12016687
ebi-a-GCST90002093	rs12186328	CD11b on CD66b+	4	154428112
ebi-a-GCST90002093	rs12204943	CD11b on CD66b+	6	12516245
ebi-a-GCST90002093	rs7790850	CD11b on CD66b+	7	154511099
ebi-a-GCST90002093	rs138210464	CD11b on CD66b+	7	62685602
ebi-a-GCST90002093	rs148435116	CD11b on CD66b+	8	120898056
ebi-a-GCST90002093	rs12345519	CD11b on CD66b+	9	134450016
ebi-a-GCST90002093	rs2377426	CD11b on CD66b+	9	78546389
ebi-a-GCST90002093	rs78741790	CD11b on CD66b+	11	40291201
ebi-a-GCST90002093	rs7111235	CD11b on CD66b+	11	133658661
ebi-a-GCST90002093	rs78885473	CD11b on CD66b+	12	26396679
ebi-a-GCST90002093	rs116849735	CD11b on CD66b+	12	44943100
ebi-a-GCST90002093	rs117878089	CD11b on CD66b+	14	56195815
ebi-a-GCST90002093	rs80037173	CD11b on CD66b+	19	39164081
ebi-a-GCST90002094	rs12073174	CD11b on Mo MDS	1	4002766
ebi-a-GCST90002094	rs1801274	CD11b on Mo MDS	1	161479745
ebi-a-GCST90002094	rs7579789	CD11b on Mo MDS	2	176610873
ebi-a-GCST90002094	rs77400868	CD11b on Mo MDS	3	3150964
ebi-a-GCST90002094	rs146739860	CD11b on Mo MDS	5	174996664
ebi-a-GCST90002094	rs74545702	CD11b on Mo MDS	5	155307756
ebi-a-GCST90002094	rs142164490	CD11b on Mo MDS	6	102083682
ebi-a-GCST90002094	rs148428675	CD11b on Mo MDS	6	112788900
ebi-a-GCST90002094	rs7750629	CD11b on Mo MDS	6	150915881
ebi-a-GCST90002094	rs17157063	CD11b on Mo MDS	7	103530694
ebi-a-GCST90002094	rs35417041	CD11b on Mo MDS	8	94254378
ebi-a-GCST90002094	rs2997983	CD11b on Mo MDS	10	1461116
ebi-a-GCST90002094	rs11232457	CD11b on Mo MDS	11	80812245
ebi-a-GCST90002094	rs1499516	CD11b on Mo MDS	11	36082067
ebi-a-GCST90002094	rs62032592	CD11b on Mo MDS	15	87411994
ebi-a-GCST90002094	rs72835003	CD11b on Mo MDS	17	4714471
ebi-a-GCST90002095	rs114446732	CD11b on CD33dir	1	170916456
ebi-a-GCST90002095	rs56391734	CD11b on CD33dir	1	67982239
ebi-a-GCST90002095	rs11922267	CD11b on CD33dir	3	65401156
ebi-a-GCST90002095	rs73214121	CD11b on CD33dir	3	134675768
ebi-a-GCST90002095	rs79689885	CD11b on CD33dir	4	38170624
ebi-a-GCST90002095	rs13143110	CD11b on CD33dir	4	143105070
ebi-a-GCST90002095	rs72943419	CD11b on CD33dir	6	106580040
ebi-a-GCST90002095	rs9381139	CD11b on CD33dir	6	42076788

ebi-a-GCST90002095	rs2015467	CD11b on CD33dir	6	120034199
ebi-a-GCST90002095	rs73084604	CD11b on CD33dir	7	24364831
ebi-a-GCST90002095	rs149090801	CD11b on CD33dir	7	48224317
ebi-a-GCST90002095	rs2462681	CD11b on CD33dir	7	113164548
ebi-a-GCST90002095	rs117431630	CD11b on CD33dir	9	7853019
ebi-a-GCST90002095	rs1547784	CD11b on CD33dir	10	112465154
ebi-a-GCST90002095	rs7896853	CD11b on CD33dir	10	99476915
ebi-a-GCST90002095	rs72837738	CD11b on CD33dir	10	122530940
ebi-a-GCST90002095	rs11114574	CD11b on CD33dir	12	81094063
ebi-a-GCST90002095	rs8018158	CD11b on CD33dir	14	99782579
ebi-a-GCST90002095	rs62040131	CD11b on CD33dir	16	79108056
ebi-a-GCST90002095	rs2457797	CD11b on CD33dir	19	22046425
ebi-a-GCST90002095	rs78744187	CD11b on CD33dir	19	33754548
ebi-a-GCST90002095	rs114757228	CD11b on CD33dir	21	19500806
ebi-a-GCST90002095	rs760462	CD11b on CD33dir	21	46328099
ebi-a-GCST90002096	rs139324834	CD11b on basophil	1	161588466
ebi-a-GCST90002096	rs12135207	CD11b on basophil	1	160944691
ebi-a-GCST90002096	rs115866025	CD11b on basophil	2	23861433
ebi-a-GCST90002096	rs73214121	CD11b on basophil	3	134675768
ebi-a-GCST90002096	rs139087540	CD11b on basophil	4	14387882
ebi-a-GCST90002096	rs7716442	CD11b on basophil	5	55780435
ebi-a-GCST90002096	rs7737891	CD11b on basophil	5	31021947
ebi-a-GCST90002096	rs410683	CD11b on basophil	6	88008920
ebi-a-GCST90002096	rs150751001	CD11b on basophil	6	94714285
ebi-a-GCST90002096	rs138132338	CD11b on basophil	7	34480553
ebi-a-GCST90002096	rs17151708	CD11b on basophil	7	10516229
ebi-a-GCST90002096	rs150791226	CD11b on basophil	9	93305784
ebi-a-GCST90002096	rs72837738	CD11b on basophil	10	122530940
ebi-a-GCST90002096	rs8018158	CD11b on basophil	14	99782579
ebi-a-GCST90002096	rs34550882	CD11b on basophil	16	31274875
ebi-a-GCST90002096	rs12451379	CD11b on basophil	17	70268997
ebi-a-GCST90002096	rs78744187	CD11b on basophil	19	33754548
ebi-a-GCST90002096	rs66604253	CD11b on basophil	21	46709509
ebi-a-GCST90002096	rs760462	CD11b on basophil	21	46328099
ebi-a-GCST90002097	rs1801274	CD11b on CD33br	1	161479745
ebi-a-GCST90002097	rs76769365	CD11b on CD33br	2	201489162
ebi-a-GCST90002097	rs142436127	CD11b on CD33br	3	118883955
ebi-a-GCST90002097	rs3943404	CD11b on CD33br	4	55977759
ebi-a-GCST90002097	rs142470748	CD11b on CD33br	7	55375099
ebi-a-GCST90002097	rs79788679	CD11b on CD33br	7	92609625
ebi-a-GCST90002097	rs846294	CD11b on CD33br	7	42123925
ebi-a-GCST90002097	rs62561188	CD11b on CD33br	9	87310577
ebi-a-GCST90002097	rs1930423	CD11b on CD33br	9	101407173
ebi-a-GCST90002097	rs11015080	CD11b on CD33br	10	26677896
ebi-a-GCST90002097	rs66811768	CD11b on CD33br	10	9580495
ebi-a-GCST90002097	rs61952076	CD11b on CD33br	12	133089630
ebi-a-GCST90002097	rs57761043	CD11b on CD33br	12	218152
ebi-a-GCST90002097	rs10850001	CD11b on CD33br	12	112553032

ebi-a-GCST90002097	rs140129370	CD11b on CD33br	13	74097996
ebi-a-GCST90002097	rs146147816	CD11b on CD33br	14	73295385
ebi-a-GCST90002097	rs75948515	CD11b on CD33br	15	51443825
ebi-a-GCST90002097	rs7166641	CD11b on CD33br	15	84637928
ebi-a-GCST90002097	rs80184675	CD11b on CD33br	16	83304908
ebi-a-GCST90002097	rs72815426	CD11b on CD33br	17	11788269
ebi-a-GCST90002097	rs55978635	CD11b on CD33br	17	75786314
ebi-a-GCST90002097	rs2552498	CD11b on CD33br	18	38747705
ebi-a-GCST90002097	rs72983710	CD11b on CD33br	18	75884528
ebi-a-GCST90002098	rs187089343	CD45RA on naive (	1	182091538
ebi-a-GCST90002098	rs146899923	CD45RA on naive (	1	195919849
ebi-a-GCST90002098	rs147071297	CD45RA on naive (	1	191648600
ebi-a-GCST90002098	rs74967706	CD45RA on naive (	1	195466150
ebi-a-GCST90002098	rs17583875	CD45RA on naive (	1	197924770
ebi-a-GCST90002098	rs138915779	CD45RA on naive (	1	190079170
ebi-a-GCST90002098	rs12730924	CD45RA on naive (	1	10870863
ebi-a-GCST90002098	rs79747818	CD45RA on naive (	2	41757305
ebi-a-GCST90002098	rs42803	CD45RA on naive (	2	40837037
ebi-a-GCST90002098	rs145947216	CD45RA on naive (	2	38596674
ebi-a-GCST90002098	rs138283413	CD45RA on naive (	2	40480936
ebi-a-GCST90002098	rs113885925	CD45RA on naive (	2	38094107
ebi-a-GCST90002098	rs182240059	CD45RA on naive (	2	38955419
ebi-a-GCST90002098	rs7605193	CD45RA on naive (	2	38698112
ebi-a-GCST90002098	rs147692482	CD45RA on naive (	2	39864242
ebi-a-GCST90002098	rs139544135	CD45RA on naive (	2	37885284
ebi-a-GCST90002098	rs34558770	CD45RA on naive (	2	38933040
ebi-a-GCST90002098	rs9834739	CD45RA on naive (	3	11614492
ebi-a-GCST90002098	rs35592432	CD45RA on naive (	3	71355240
ebi-a-GCST90002098	rs35550194	CD45RA on naive (	3	10639764
ebi-a-GCST90002098	rs115645435	CD45RA on naive (	4	22091595
ebi-a-GCST90002098	rs62328019	CD45RA on naive (	4	147691255
ebi-a-GCST90002098	rs10022333	CD45RA on naive (	4	19362367
ebi-a-GCST90002098	rs531042	CD45RA on naive (	6	124879453
ebi-a-GCST90002098	rs28590272	CD45RA on naive (	7	50360663
ebi-a-GCST90002098	rs917116	CD45RA on naive (	7	28172739
ebi-a-GCST90002098	rs79000121	CD45RA on naive (	7	10126364
ebi-a-GCST90002098	rs117626397	CD45RA on naive (	10	131423918
ebi-a-GCST90002098	rs72859264	CD45RA on naive (	11	11304233
ebi-a-GCST90002098	rs61938012	CD45RA on naive (	12	119526748
ebi-a-GCST90002098	rs79505210	CD45RA on naive (	12	132969270
ebi-a-GCST90002098	rs61913600	CD45RA on naive (	12	12884357
ebi-a-GCST90002098	rs1641548	CD45RA on naive (	17	7574779
ebi-a-GCST90002098	rs55733272	CD45RA on naive (	18	4765016
ebi-a-GCST90002098	rs75425544	CD45RA on naive (	20	31591228
ebi-a-GCST90002099	rs116794175	CD4RA on TD CD4	1	194010208
ebi-a-GCST90002099	rs11579717	CD4RA on TD CD4	1	199999121
ebi-a-GCST90002099	rs34645541	CD4RA on TD CD4	1	195875605
ebi-a-GCST90002099	rs822833	CD4RA on TD CD4	1	234954585

ebi-a-GCST90002099	rs141541045	CD4RA on TD CD4	1	182131075
ebi-a-GCST90002099	rs541367304	CD4RA on TD CD4	1	198283058
ebi-a-GCST90002099	rs4670907	CD4RA on TD CD4	2	39278066
ebi-a-GCST90002099	rs147201746	CD4RA on TD CD4	2	15706963
ebi-a-GCST90002099	rs7559619	CD4RA on TD CD4	2	38892207
ebi-a-GCST90002099	rs1861266	CD4RA on TD CD4	2	40283459
ebi-a-GCST90002099	rs116345427	CD4RA on TD CD4	3	146702237
ebi-a-GCST90002099	rs4974277	CD4RA on TD CD4	3	70625844
ebi-a-GCST90002099	rs78528853	CD4RA on TD CD4	3	26352969
ebi-a-GCST90002099	rs1504496	CD4RA on TD CD4	4	45951996
ebi-a-GCST90002099	rs17777079	CD4RA on TD CD4	5	100081982
ebi-a-GCST90002099	rs111916169	CD4RA on TD CD4	5	78137524
ebi-a-GCST90002099	rs929785	CD4RA on TD CD4	5	159153268
ebi-a-GCST90002099	rs181435089	CD4RA on TD CD4	6	69234703
ebi-a-GCST90002099	rs117931526	CD4RA on TD CD4	6	117716047
ebi-a-GCST90002099	rs931816	CD4RA on TD CD4	7	9679455
ebi-a-GCST90002099	rs78948498	CD4RA on TD CD4	8	77159185
ebi-a-GCST90002099	rs13269202	CD4RA on TD CD4	8	70924735
ebi-a-GCST90002099	rs13292669	CD4RA on TD CD4	9	132659104
ebi-a-GCST90002099	rs10114587	CD4RA on TD CD4	9	133795282
ebi-a-GCST90002099	rs10815513	CD4RA on TD CD4	9	7088522
ebi-a-GCST90002099	rs1328601	CD4RA on TD CD4	10	125153605
ebi-a-GCST90002099	rs7894393	CD4RA on TD CD4	10	100120872
ebi-a-GCST90002099	rs76295302	CD4RA on TD CD4	10	2556079
ebi-a-GCST90002099	rs56049135	CD4RA on TD CD4	12	102381732
ebi-a-GCST90002099	rs2188219	CD4RA on TD CD4	17	5923525
ebi-a-GCST90002099	rs11652274	CD4RA on TD CD4	17	26010931
ebi-a-GCST90002099	rs78804476	CD4RA on TD CD4	19	662832
ebi-a-GCST90002099	rs4261817	CD4RA on TD CD4	22	49045798
ebi-a-GCST90002100	rs6587385	CD45RA on naive C	1	248047256
ebi-a-GCST90002100	rs12027761	CD45RA on naive C	1	247974959
ebi-a-GCST90002100	rs2993668	CD45RA on naive C	1	11394782
ebi-a-GCST90002100	rs116517602	CD45RA on naive C	1	198922068
ebi-a-GCST90002100	rs147071297	CD45RA on naive C	1	191648600
ebi-a-GCST90002100	rs150981153	CD45RA on naive C	2	57533997
ebi-a-GCST90002100	rs185202879	CD45RA on naive C	2	72252030
ebi-a-GCST90002100	rs971274	CD45RA on naive C	2	38495461
ebi-a-GCST90002100	rs12712613	CD45RA on naive C	2	38948860
ebi-a-GCST90002100	rs116497662	CD45RA on naive C	3	17996736
ebi-a-GCST90002100	rs79003654	CD45RA on naive C	5	180639067
ebi-a-GCST90002100	rs3812450	CD45RA on naive C	8	69349596
ebi-a-GCST90002100	rs3762058	CD45RA on naive C	9	116842333
ebi-a-GCST90002100	rs61875926	CD45RA on naive C	11	8705528
ebi-a-GCST90002100	rs144760211	CD45RA on naive C	11	62424847
ebi-a-GCST90002100	rs2620762	CD45RA on naive C	12	68003886
ebi-a-GCST90002100	rs2160419	CD45RA on naive C	13	72314346
ebi-a-GCST90002100	rs1709408	CD45RA on naive C	15	22517225
ebi-a-GCST90002100	rs72845213	CD45RA on naive C	17	61280212

ebi-a-GCST90002100	rs7217643	CD45RA on naive (	17	62402769
ebi-a-GCST90002100	rs74440646	CD45RA on naive (	18	6162001
ebi-a-GCST90002100	rs7239526	CD45RA on naive (	18	65414267
ebi-a-GCST90002100	rs117074487	CD45RA on naive (	18	64698349
ebi-a-GCST90002101	rs1861404	CD45RA on TD CI	2	64833920
ebi-a-GCST90002101	rs7583259	CD45RA on TD CI	2	38921934
ebi-a-GCST90002101	rs192211982	CD45RA on TD CI	2	201907127
ebi-a-GCST90002101	rs61141933	CD45RA on TD CI	2	39388479
ebi-a-GCST90002101	rs800067	CD45RA on TD CI	3	144389644
ebi-a-GCST90002101	rs111772667	CD45RA on TD CI	4	130243636
ebi-a-GCST90002101	rs6962836	CD45RA on TD CI	7	22705474
ebi-a-GCST90002101	rs74516300	CD45RA on TD CI	9	22968922
ebi-a-GCST90002101	rs141423324	CD45RA on TD CI	9	78911401
ebi-a-GCST90002101	rs141550154	CD45RA on TD CI	10	19011440
ebi-a-GCST90002101	rs11177189	CD45RA on TD CI	12	68758131
ebi-a-GCST90002101	rs141602247	CD45RA on TD CI	13	53730995
ebi-a-GCST90002101	rs62025666	CD45RA on TD CI	15	99720770
ebi-a-GCST90002101	rs11634765	CD45RA on TD CI	15	92817659
ebi-a-GCST90002101	rs634097	CD45RA on TD CI	18	561109
ebi-a-GCST90002102	rs140807368	CD45RA on resting	1	194379933
ebi-a-GCST90002102	rs34645541	CD45RA on resting	1	195875605
ebi-a-GCST90002102	rs138915779	CD45RA on resting	1	190079170
ebi-a-GCST90002102	rs187089343	CD45RA on resting	1	182091538
ebi-a-GCST90002102	rs6587385	CD45RA on resting	1	248047256
ebi-a-GCST90002102	rs11579717	CD45RA on resting	1	199999121
ebi-a-GCST90002102	rs541367304	CD45RA on resting	1	198283058
ebi-a-GCST90002102	rs72805573	CD45RA on resting	2	62708494
ebi-a-GCST90002102	rs10188010	CD45RA on resting	2	41782340
ebi-a-GCST90002102	rs13024840	CD45RA on resting	2	38895920
ebi-a-GCST90002102	rs11124653	CD45RA on resting	2	39112166
ebi-a-GCST90002102	rs2565661	CD45RA on resting	2	38132650
ebi-a-GCST90002102	rs78689347	CD45RA on resting	2	40773763
ebi-a-GCST90002102	rs146873217	CD45RA on resting	3	43370758
ebi-a-GCST90002102	rs72819716	CD45RA on resting	6	10433404
ebi-a-GCST90002102	rs12663871	CD45RA on resting	6	68875765
ebi-a-GCST90002102	rs138790946	CD45RA on resting	7	35166829
ebi-a-GCST90002102	rs76643193	CD45RA on resting	7	130703888
ebi-a-GCST90002102	rs149694073	CD45RA on resting	8	139700141
ebi-a-GCST90002102	rs146962738	CD45RA on resting	8	122930949
ebi-a-GCST90002102	rs34376819	CD45RA on resting	13	86243281
ebi-a-GCST90002102	rs191186600	CD45RA on resting	14	69609044
ebi-a-GCST90002102	rs141148183	CD45RA on resting	16	54755529
ebi-a-GCST90002102	rs72868954	CD45RA on resting	17	68520771
ebi-a-GCST90002102	rs359735	CD45RA on resting	18	73770282
ebi-a-GCST90002102	rs568546879	CD45RA on resting	18	33878112
ebi-a-GCST90002102	rs56747968	CD45RA on resting	19	2059322
ebi-a-GCST90002102	rs34096085	CD45RA on resting	21	39344345
ebi-a-GCST90002102	rs7283704	CD45RA on resting	21	39816617

ebi-a-GCST90002103	rs191753228	CD45RA on CD39-	1	196321168
ebi-a-GCST90002103	rs114590935	CD45RA on CD39-	1	210056681
ebi-a-GCST90002103	rs72761048	CD45RA on CD39-	1	248058067
ebi-a-GCST90002103	rs80125540	CD45RA on CD39-	1	80096051
ebi-a-GCST90002103	rs72805573	CD45RA on CD39-	2	62708494
ebi-a-GCST90002103	rs183860421	CD45RA on CD39-	2	38042606
ebi-a-GCST90002103	rs145947216	CD45RA on CD39-	2	38596674
ebi-a-GCST90002103	rs1405788	CD45RA on CD39-	3	39789191
ebi-a-GCST90002103	rs116682768	CD45RA on CD39-	3	22257995
ebi-a-GCST90002103	rs114748919	CD45RA on CD39-	4	122946186
ebi-a-GCST90002104	rs55971447	HLA DR on myeloi	1	161515326
ebi-a-GCST90002104	rs72973336	HLA DR on myeloi	2	228879579
ebi-a-GCST90002104	rs116607596	HLA DR on myeloi	3	125955606
ebi-a-GCST90002104	rs9853546	HLA DR on myeloi	3	73377280
ebi-a-GCST90002104	rs3756577	HLA DR on myeloi	5	149628644
ebi-a-GCST90002104	rs35525122	HLA DR on myeloi	6	32561283
ebi-a-GCST90002104	rs9267650	HLA DR on myeloi	6	31824912
ebi-a-GCST90002104	rs1634738	HLA DR on myeloi	6	31284067
ebi-a-GCST90002104	rs116007826	HLA DR on myeloi	6	31569745
ebi-a-GCST90002104	rs6925683	HLA DR on myeloi	6	33894292
ebi-a-GCST90002104	rs29221	HLA DR on myeloi	6	29589124
ebi-a-GCST90002104	rs2858885	HLA DR on myeloi	6	32699782
ebi-a-GCST90002104	rs144823164	HLA DR on myeloi	6	22385857
ebi-a-GCST90002104	rs34618458	HLA DR on myeloi	7	806948
ebi-a-GCST90002104	rs11979208	HLA DR on myeloi	7	80529639
ebi-a-GCST90002104	rs142640535	HLA DR on myeloi	8	122566151
ebi-a-GCST90002104	rs74134806	HLA DR on myeloi	10	54787061
ebi-a-GCST90002104	rs9533322	HLA DR on myeloi	13	43546618
ebi-a-GCST90002104	rs42362	HLA DR on myeloi	16	9438363
ebi-a-GCST90002105	rs62176112	HLA DR on plasma	2	179315726
ebi-a-GCST90002105	rs11731221	HLA DR on plasma	4	84168891
ebi-a-GCST90002105	rs115770777	HLA DR on plasma	4	26859610
ebi-a-GCST90002105	rs952839	HLA DR on plasma	5	86099942
ebi-a-GCST90002105	rs9296042	HLA DR on plasma	6	32736005
ebi-a-GCST90002105	rs9267650	HLA DR on plasma	6	31824912
ebi-a-GCST90002105	rs2496370	HLA DR on plasma	6	49127467
ebi-a-GCST90002105	rs16894020	HLA DR on plasma	6	28340245
ebi-a-GCST90002105	rs200819	HLA DR on plasma	6	97857419
ebi-a-GCST90002105	rs116007826	HLA DR on plasma	6	31569745
ebi-a-GCST90002105	rs7748976	HLA DR on plasma	6	33890451
ebi-a-GCST90002105	rs34039593	HLA DR on plasma	6	32570311
ebi-a-GCST90002105	rs29221	HLA DR on plasma	6	29589124
ebi-a-GCST90002105	rs16890461	HLA DR on plasma	8	41189599
ebi-a-GCST90002105	rs10812812	HLA DR on plasma	9	28412352
ebi-a-GCST90002105	rs11615578	HLA DR on plasma	12	121714935
ebi-a-GCST90002105	rs11615028	HLA DR on plasma	12	41040386
ebi-a-GCST90002105	rs144816332	HLA DR on plasma	14	67537526
ebi-a-GCST90002105	rs11644019	HLA DR on plasma	16	10863414

ebi-a-GCST90002105	rs1646067	HLA DR on plasma	16	11319045
ebi-a-GCST90002105	rs7204799	HLA DR on plasma	16	11004549
ebi-a-GCST90002105	rs118111544	HLA DR on plasma	17	55035000
ebi-a-GCST90002105	rs12607423	HLA DR on plasma	18	67084661
ebi-a-GCST90002105	rs146863538	HLA DR on plasma	19	19396439
ebi-a-GCST90002106	rs55971447	HLA DR on DC	1	161515326
ebi-a-GCST90002106	rs12140470	HLA DR on DC	1	115683097
ebi-a-GCST90002106	rs13064098	HLA DR on DC	3	73320945
ebi-a-GCST90002106	rs149978880	HLA DR on DC	5	66320166
ebi-a-GCST90002106	rs9267650	HLA DR on DC	6	31824912
ebi-a-GCST90002106	rs9296042	HLA DR on DC	6	32736005
ebi-a-GCST90002106	rs116007826	HLA DR on DC	6	31569745
ebi-a-GCST90002106	rs34039593	HLA DR on DC	6	32570311
ebi-a-GCST90002106	rs7748976	HLA DR on DC	6	33890451
ebi-a-GCST90002106	rs362522	HLA DR on DC	6	29545208
ebi-a-GCST90002106	rs66680090	HLA DR on DC	6	18520529
ebi-a-GCST90002106	rs34618458	HLA DR on DC	7	806948
ebi-a-GCST90002106	rs4085480	HLA DR on DC	8	62151870
ebi-a-GCST90002106	rs117673964	HLA DR on DC	8	60190262
ebi-a-GCST90002106	rs11251654	HLA DR on DC	10	3075772
ebi-a-GCST90002106	rs9532060	HLA DR on DC	13	38046720
ebi-a-GCST90002106	rs191565346	HLA DR on DC	13	90363666
ebi-a-GCST90002106	rs7204799	HLA DR on DC	16	11004549
ebi-a-GCST90002106	rs17794301	HLA DR on DC	16	6085213
ebi-a-GCST90002106	rs140657974	HLA DR on DC	16	8010586
ebi-a-GCST90002106	rs11644019	HLA DR on DC	16	10863414
ebi-a-GCST90002107	rs56947008	HLA DR on HSC	2	104722210
ebi-a-GCST90002107	rs79232278	HLA DR on HSC	3	68515938
ebi-a-GCST90002107	rs6779181	HLA DR on HSC	3	10960562
ebi-a-GCST90002107	rs114459693	HLA DR on HSC	3	150331124
ebi-a-GCST90002107	rs9834298	HLA DR on HSC	3	81341469
ebi-a-GCST90002107	rs148887724	HLA DR on HSC	3	101266753
ebi-a-GCST90002107	rs142397031	HLA DR on HSC	5	8721320
ebi-a-GCST90002108	rs76743559	HLA DR on CD33b	1	247591691
ebi-a-GCST90002108	rs114267024	HLA DR on CD33b	2	39668818
ebi-a-GCST90002108	rs1424773	HLA DR on CD33b	2	108017810
ebi-a-GCST90002108	rs146788953	HLA DR on CD33b	3	19007547
ebi-a-GCST90002108	rs57623845	HLA DR on CD33b	4	114672008
ebi-a-GCST90002108	rs71600299	HLA DR on CD33b	5	6766557
ebi-a-GCST90002108	rs523727	HLA DR on CD33b	6	31768800
ebi-a-GCST90002108	rs116007826	HLA DR on CD33b	6	31569745
ebi-a-GCST90002108	rs9269027	HLA DR on CD33b	6	32437466
ebi-a-GCST90002108	rs77072921	HLA DR on CD33b	6	52346566
ebi-a-GCST90002108	rs238873	HLA DR on CD33b	6	29351641
ebi-a-GCST90002108	rs6925683	HLA DR on CD33b	6	33894292
ebi-a-GCST90002108	rs9270588	HLA DR on CD33b	6	32561327
ebi-a-GCST90002108	rs847842	HLA DR on CD33b	6	34915073
ebi-a-GCST90002108	rs77506340	HLA DR on CD33b	7	128764737

ebi-a-GCST90002108	rs147951036	HLA DR on CD33b	7	78355906
ebi-a-GCST90002108	rs117798915	HLA DR on CD33b	12	103202798
ebi-a-GCST90002108	rs9526485	HLA DR on CD33b	13	49246943
ebi-a-GCST90002108	rs7325866	HLA DR on CD33b	13	51608785
ebi-a-GCST90002108	rs78122038	HLA DR on CD33b	14	107253542
ebi-a-GCST90002108	rs36037794	HLA DR on CD33b	16	5929011
ebi-a-GCST90002108	rs71363011	HLA DR on CD33b	18	46154251
ebi-a-GCST90002108	rs112096735	HLA DR on CD33b	19	11733838
ebi-a-GCST90002108	rs79269061	HLA DR on CD33b	20	5759975
ebi-a-GCST90002108	rs16987905	HLA DR on CD33b	20	37940679
ebi-a-GCST90002108	rs186879638	HLA DR on CD33b	21	22100611
ebi-a-GCST90002109	rs76111665	HLA DR on CD33b	1	240278560
ebi-a-GCST90002109	rs12987451	HLA DR on CD33b	2	108014254
ebi-a-GCST90002109	rs146788953	HLA DR on CD33b	3	19007547
ebi-a-GCST90002109	rs9846278	HLA DR on CD33b	3	54068119
ebi-a-GCST90002109	rs112878149	HLA DR on CD33b	4	184499212
ebi-a-GCST90002109	rs7667073	HLA DR on CD33b	4	126165609
ebi-a-GCST90002109	rs72667899	HLA DR on CD33b	4	108205128
ebi-a-GCST90002109	rs613165	HLA DR on CD33b	6	31834398
ebi-a-GCST90002109	rs111404935	HLA DR on CD33b	6	66907682
ebi-a-GCST90002109	rs9346529	HLA DR on CD33b	6	168576827
ebi-a-GCST90002109	rs6912516	HLA DR on CD33b	6	152114121
ebi-a-GCST90002109	rs9270588	HLA DR on CD33b	6	32561327
ebi-a-GCST90002109	rs142186496	HLA DR on CD33b	6	31473707
ebi-a-GCST90002109	rs9269027	HLA DR on CD33b	6	32437466
ebi-a-GCST90002109	rs142241717	HLA DR on CD33b	8	82111249
ebi-a-GCST90002109	rs2094597	HLA DR on CD33b	9	31813280
ebi-a-GCST90002109	rs7478464	HLA DR on CD33b	10	5615210
ebi-a-GCST90002109	rs393073	HLA DR on CD33b	10	55249364
ebi-a-GCST90002109	rs79485969	HLA DR on CD33b	14	34680388
ebi-a-GCST90002109	rs2115412	HLA DR on CD33b	16	9425538
ebi-a-GCST90002109	rs34813869	HLA DR on CD33b	19	51737121
ebi-a-GCST90002109	rs11701225	HLA DR on CD33b	21	44598827
ebi-a-GCST90002110	rs1801274	HLA DR on CD33d	1	161479745
ebi-a-GCST90002110	rs76870855	HLA DR on CD33d	1	183457090
ebi-a-GCST90002110	rs2887457	HLA DR on CD33d	2	185272346
ebi-a-GCST90002110	rs13074783	HLA DR on CD33d	3	4037838
ebi-a-GCST90002110	rs1461277	HLA DR on CD33d	3	174918975
ebi-a-GCST90002110	rs12485444	HLA DR on CD33d	3	188135783
ebi-a-GCST90002110	rs412492	HLA DR on CD33d	6	32213831
ebi-a-GCST90002110	rs61742525	HLA DR on CD33d	6	33072522
ebi-a-GCST90002110	rs116632210	HLA DR on CD33d	6	37856213
ebi-a-GCST90002110	rs182790	HLA DR on CD33d	6	100053560
ebi-a-GCST90002110	rs9269109	HLA DR on CD33d	6	32443266
ebi-a-GCST90002110	rs6917212	HLA DR on CD33d	6	32583005
ebi-a-GCST90002110	rs118175684	HLA DR on CD33d	7	121443863
ebi-a-GCST90002110	rs2237423	HLA DR on CD33d	7	42100425
ebi-a-GCST90002110	rs10511609	HLA DR on CD33d	9	15335996

ebi-a-GCST90002110	rs61835728	HLA DR on CD33d	10	10550265
ebi-a-GCST90002110	rs78082697	HLA DR on CD33d	12	133777338
ebi-a-GCST90002110	rs147171620	HLA DR on CD33d	13	46828483
ebi-a-GCST90002110	rs72737163	HLA DR on CD33d	15	39300811
ebi-a-GCST90002110	rs11074934	HLA DR on CD33d	16	10979440
ebi-a-GCST90002110	rs12929277	HLA DR on CD33d	16	10700928
ebi-a-GCST90002110	rs142006783	HLA DR on CD33d	16	74651733
ebi-a-GCST90002110	rs5016173	HLA DR on CD33d	20	49233671
ebi-a-GCST90002110	rs113773853	HLA DR on CD33d	22	39607103
ebi-a-GCST90002111	rs9790174	HLA DR on CD33d	3	174906509
ebi-a-GCST90002111	rs56386567	HLA DR on CD33d	3	4868895
ebi-a-GCST90002111	rs12485444	HLA DR on CD33d	3	188135783
ebi-a-GCST90002111	rs2517696	HLA DR on CD33d	6	29927611
ebi-a-GCST90002111	rs6917212	HLA DR on CD33d	6	32583005
ebi-a-GCST90002111	rs7747856	HLA DR on CD33d	6	33889952
ebi-a-GCST90002111	rs9269109	HLA DR on CD33d	6	32443266
ebi-a-GCST90002111	rs61742525	HLA DR on CD33d	6	33072522
ebi-a-GCST90002111	rs79018836	HLA DR on CD33d	7	29344179
ebi-a-GCST90002111	rs2237423	HLA DR on CD33d	7	42100425
ebi-a-GCST90002111	rs13226058	HLA DR on CD33d	7	150536031
ebi-a-GCST90002111	rs7797098	HLA DR on CD33d	7	141012015
ebi-a-GCST90002111	rs117936815	HLA DR on CD33d	10	92271546
ebi-a-GCST90002111	rs77638176	HLA DR on CD33d	11	5106022
ebi-a-GCST90002111	rs12929277	HLA DR on CD33d	16	10700928
ebi-a-GCST90002111	rs55798945	HLA DR on CD33d	16	669708
ebi-a-GCST90002111	rs11074934	HLA DR on CD33d	16	10979440
ebi-a-GCST90002111	rs33978622	HLA DR on CD33d	19	51736383
ebi-a-GCST90002111	rs4811082	HLA DR on CD33d	20	49216966
ebi-a-GCST90002112	rs9310124	HLA DR on CD33-	3	68608477
ebi-a-GCST90002112	rs78909221	HLA DR on CD33-	4	8219482
ebi-a-GCST90002112	rs9368784	HLA DR on CD33-	6	33895001
ebi-a-GCST90002112	rs2760980	HLA DR on CD33-	6	32565201
ebi-a-GCST90002112	rs1474618	HLA DR on CD33-	6	18655570
ebi-a-GCST90002112	rs9279789	HLA DR on CD33-	6	32571079
ebi-a-GCST90002112	rs114068468	HLA DR on CD33-	6	33054463
ebi-a-GCST90002112	rs9268430	HLA DR on CD33-	6	32345429
ebi-a-GCST90002112	rs11576012	HLA DR on CD33-	6	31795619
ebi-a-GCST90002112	rs140697652	HLA DR on CD33-	7	101713394
ebi-a-GCST90002112	rs28714544	HLA DR on CD33-	7	213189
ebi-a-GCST90002112	rs79778131	HLA DR on CD33-	11	94044559
ebi-a-GCST90002112	rs775443	HLA DR on CD33-	12	70122623
ebi-a-GCST90002112	rs9545905	HLA DR on CD33-	13	82450885
ebi-a-GCST90002112	rs928189	HLA DR on CD33-	13	94559274
ebi-a-GCST90002113	rs17512470	HLA DR on HLA I	1	60755909
ebi-a-GCST90002113	rs609101	HLA DR on HLA I	1	181413584
ebi-a-GCST90002113	rs34590422	HLA DR on HLA I	2	192127400
ebi-a-GCST90002113	rs4588375	HLA DR on HLA I	3	161826111
ebi-a-GCST90002113	rs74633806	HLA DR on HLA I	4	76114786

ebi-a-GCST90002113	rs4565150	HLA DR on	HLA l	4	6517066
ebi-a-GCST90002113	rs11931648	HLA DR on	HLA l	4	117545645
ebi-a-GCST90002113	rs2526231	HLA DR on	HLA l	5	128296872
ebi-a-GCST90002113	rs11771690	HLA DR on	HLA l	7	42031304
ebi-a-GCST90002113	rs117406069	HLA DR on	HLA l	10	130262493
ebi-a-GCST90002113	rs116985795	HLA DR on	HLA l	13	105513838
ebi-a-GCST90002113	rs2027912	HLA DR on	HLA l	14	107045318
ebi-a-GCST90002113	rs146706846	HLA DR on	HLA l	15	100671214
ebi-a-GCST90002113	rs12449199	HLA DR on	HLA l	16	10968440
ebi-a-GCST90002113	rs246230	HLA DR on	HLA l	16	16132880
ebi-a-GCST90002113	rs7211380	HLA DR on	HLA l	17	64206768
ebi-a-GCST90002113	rs2584732	HLA DR on	HLA l	18	11025325
ebi-a-GCST90002113	rs62131098	HLA DR on	HLA l	19	11396934
ebi-a-GCST90002113	rs55899966	HLA DR on	HLA l	20	361668
ebi-a-GCST90002114	rs115943613	HLA DR on	HLA l	2	40943702
ebi-a-GCST90002114	rs78191148	HLA DR on	HLA l	4	89583183
ebi-a-GCST90002114	rs13175786	HLA DR on	HLA l	5	7447525
ebi-a-GCST90002114	rs12206101	HLA DR on	HLA l	6	77973938
ebi-a-GCST90002114	rs9391881	HLA DR on	HLA l	6	32443432
ebi-a-GCST90002114	rs9480594	HLA DR on	HLA l	6	106183999
ebi-a-GCST90002114	rs112529964	HLA DR on	HLA l	6	135284472
ebi-a-GCST90002114	rs76903247	HLA DR on	HLA l	6	163955747
ebi-a-GCST90002114	rs17639764	HLA DR on	HLA l	7	104606411
ebi-a-GCST90002114	rs544627737	HLA DR on	HLA l	7	22746599
ebi-a-GCST90002114	rs138947942	HLA DR on	HLA l	9	96558710
ebi-a-GCST90002114	rs61852195	HLA DR on	HLA l	10	57794952
ebi-a-GCST90002114	rs183943359	HLA DR on	HLA l	11	112685081
ebi-a-GCST90002114	rs6591057	HLA DR on	HLA l	11	103771107
ebi-a-GCST90002114	rs148090091	HLA DR on	HLA l	12	131767379
ebi-a-GCST90002114	rs75576656	HLA DR on	HLA l	15	31542075
ebi-a-GCST90002114	rs10438450	HLA DR on	HLA l	15	94577671
ebi-a-GCST90002114	rs12449199	HLA DR on	HLA l	16	10968440
ebi-a-GCST90002114	rs4310973	HLA DR on	HLA l	18	20707622
ebi-a-GCST90002114	rs76600563	HLA DR on	HLA l	18	864027
ebi-a-GCST90002114	rs55899966	HLA DR on	HLA l	20	361668
ebi-a-GCST90002114	rs5994871	HLA DR on	HLA l	22	22091244
ebi-a-GCST90002115	rs76623705	HLA DR on	HLA l	2	67624799
ebi-a-GCST90002115	rs115681509	HLA DR on	HLA l	4	25796677
ebi-a-GCST90002115	rs9388722	HLA DR on	HLA l	6	129982285
ebi-a-GCST90002115	rs62450275	HLA DR on	HLA l	7	15298727
ebi-a-GCST90002115	rs7026621	HLA DR on	HLA l	9	29448508
ebi-a-GCST90002115	rs10815977	HLA DR on	HLA l	9	8860355
ebi-a-GCST90002115	rs2820900	HLA DR on	HLA l	9	7206216
ebi-a-GCST90002115	rs73211935	HLA DR on	HLA l	12	118047225
ebi-a-GCST90002115	rs111484143	HLA DR on	HLA l	13	93687655
ebi-a-GCST90002115	rs1927344	HLA DR on	HLA l	13	111053219
ebi-a-GCST90002115	rs142342151	HLA DR on	HLA l	14	104847658
ebi-a-GCST90002115	rs6498116	HLA DR on	HLA l	16	10969282

ebi-a-GCST90002115	rs73513895	HLA DR on HLA I	16	10811814
ebi-a-GCST90002115	rs11651897	HLA DR on HLA I	17	64739971
ebi-a-GCST90002115	rs7406446	HLA DR on HLA I	17	80979162
ebi-a-GCST90002115	rs11651658	HLA DR on HLA I	17	64198640
ebi-a-GCST90002115	rs147748599	HLA DR on HLA I	17	63501173
ebi-a-GCST90002115	rs403027	HLA DR on HLA I	19	11406458
ebi-a-GCST90002115	rs74171655	HLA DR on HLA I	20	17906490
ebi-a-GCST90002115	rs9976375	HLA DR on HLA I	21	18010539
ebi-a-GCST90002116	rs111281233	HLA DR on B cell	2	230286959
ebi-a-GCST90002116	rs1021867	HLA DR on B cell	2	8599660
ebi-a-GCST90002116	rs144091827	HLA DR on B cell	2	69187142
ebi-a-GCST90002116	rs144782687	HLA DR on B cell	4	167696884
ebi-a-GCST90002116	rs74405933	HLA DR on B cell	6	14095755
ebi-a-GCST90002116	rs9379818	HLA DR on B cell	6	26023206
ebi-a-GCST90002116	rs9271768	HLA DR on B cell	6	32594188
ebi-a-GCST90002116	rs147210938	HLA DR on B cell	6	13892767
ebi-a-GCST90002116	rs143305120	HLA DR on B cell	6	13991643
ebi-a-GCST90002116	rs17616631	HLA DR on B cell	6	12544548
ebi-a-GCST90002116	rs3869145	HLA DR on B cell	6	31851354
ebi-a-GCST90002116	rs111983490	HLA DR on B cell	6	32205324
ebi-a-GCST90002116	rs11969859	HLA DR on B cell	6	44605416
ebi-a-GCST90002116	rs9270599	HLA DR on B cell	6	32561656
ebi-a-GCST90002116	rs72880510	HLA DR on B cell	6	33729188
ebi-a-GCST90002116	rs2511702	HLA DR on B cell	8	103704029
ebi-a-GCST90002116	rs117640098	HLA DR on B cell	9	91723702
ebi-a-GCST90002116	rs79709127	HLA DR on B cell	9	126838406
ebi-a-GCST90002116	rs944963	HLA DR on B cell	10	131688008
ebi-a-GCST90002116	rs76548433	HLA DR on B cell	13	77027095
ebi-a-GCST90002116	rs7192768	HLA DR on B cell	16	11004905
ebi-a-GCST90002116	rs2286975	HLA DR on B cell	16	11114006
ebi-a-GCST90002117	rs74341264	HLA DR on HLA D	1	161561151
ebi-a-GCST90002117	rs3845488	HLA DR on HLA D	1	37419029
ebi-a-GCST90002117	rs56199187	HLA DR on HLA D	1	161520931
ebi-a-GCST90002117	rs11901763	HLA DR on HLA D	2	235192580
ebi-a-GCST90002117	rs113346886	HLA DR on HLA D	3	43788371
ebi-a-GCST90002117	rs138970824	HLA DR on HLA D	3	4179058
ebi-a-GCST90002117	rs17039221	HLA DR on HLA D	3	14308924
ebi-a-GCST90002117	rs114705966	HLA DR on HLA D	5	150304121
ebi-a-GCST90002117	rs805288	HLA DR on HLA D	6	31678028
ebi-a-GCST90002117	rs9268628	HLA DR on HLA D	6	32405865
ebi-a-GCST90002117	rs885947	HLA DR on HLA D	6	29696656
ebi-a-GCST90002117	rs73747338	HLA DR on HLA D	6	33798041
ebi-a-GCST90002117	rs419434	HLA DR on HLA D	6	32986749
ebi-a-GCST90002117	rs4959613	HLA DR on HLA D	6	1835403
ebi-a-GCST90002117	rs139174173	HLA DR on HLA D	6	32592834
ebi-a-GCST90002117	rs111660650	HLA DR on HLA D	6	135147576
ebi-a-GCST90002117	rs117172940	HLA DR on HLA D	7	26706043
ebi-a-GCST90002117	rs9297556	HLA DR on HLA D	8	117469352

ebi-a-GCST90002117	rs2065798	HLA DR on HLA I	13	20793579
ebi-a-GCST90002117	rs55813952	HLA DR on HLA I	14	101223876
ebi-a-GCST90002117	rs74876321	HLA DR on HLA I	16	78808938
ebi-a-GCST90002117	rs12598451	HLA DR on HLA I	16	10982607
ebi-a-GCST90002117	rs12929277	HLA DR on HLA I	16	10700928
ebi-a-GCST90002117	rs739438	HLA DR on HLA I	17	26689712
ebi-a-GCST90002117	rs55831773	HLA DR on HLA I	17	7559037
ebi-a-GCST90002117	rs11260062	HLA DR on HLA I	19	8214970
ebi-a-GCST90002117	rs13052008	HLA DR on HLA I	21	25055193
ebi-a-GCST90002117	rs738649	HLA DR on HLA I	22	26623368
ebi-a-GCST90002118	rs12046688	CD8 on CD28+ CD	1	47599360
ebi-a-GCST90002118	rs1235080	CD8 on CD28+ CD	1	192838446
ebi-a-GCST90002118	rs13004867	CD8 on CD28+ CD	2	86982504
ebi-a-GCST90002118	rs10181413	CD8 on CD28+ CD	2	80959711
ebi-a-GCST90002118	rs10185095	CD8 on CD28+ CD	2	85517590
ebi-a-GCST90002118	rs59998168	CD8 on CD28+ CD	2	145331724
ebi-a-GCST90002118	rs145728549	CD8 on CD28+ CD	2	101907011
ebi-a-GCST90002118	rs58007649	CD8 on CD28+ CD	3	128289541
ebi-a-GCST90002118	rs4241667	CD8 on CD28+ CD	4	66441067
ebi-a-GCST90002118	rs1616538	CD8 on CD28+ CD	6	31276042
ebi-a-GCST90002118	rs9263601	CD8 on CD28+ CD	6	31071758
ebi-a-GCST90002118	rs77892262	CD8 on CD28+ CD	6	30738211
ebi-a-GCST90002118	rs56262516	CD8 on CD28+ CD	6	29930939
ebi-a-GCST90002118	rs34862764	CD8 on CD28+ CD	8	135741562
ebi-a-GCST90002118	rs80304304	CD8 on CD28+ CD	10	80313233
ebi-a-GCST90002118	rs117410826	CD8 on CD28+ CD	20	17336549
ebi-a-GCST90002119	rs184303531	CD8 on CD28+ CD	1	237230229
ebi-a-GCST90002119	rs517004	CD8 on CD28+ CD	1	18664626
ebi-a-GCST90002119	rs1356625	CD8 on CD28+ CD	2	86022747
ebi-a-GCST90002119	rs938487	CD8 on CD28+ CD	2	87009488
ebi-a-GCST90002119	rs149943125	CD8 on CD28+ CD	2	102826146
ebi-a-GCST90002119	rs13024436	CD8 on CD28+ CD	2	86534521
ebi-a-GCST90002119	rs10205992	CD8 on CD28+ CD	2	87279109
ebi-a-GCST90002119	rs4691190	CD8 on CD28+ CD	4	166319020
ebi-a-GCST90002119	rs116619343	CD8 on CD28+ CD	5	9335253
ebi-a-GCST90002119	rs12660712	CD8 on CD28+ CD	6	29734634
ebi-a-GCST90002119	rs28747043	CD8 on CD28+ CD	6	32655508
ebi-a-GCST90002119	rs130065	CD8 on CD28+ CD	6	31122500
ebi-a-GCST90002119	rs58853189	CD8 on CD28+ CD	6	41532904
ebi-a-GCST90002119	rs1264570	CD8 on CD28+ CD	6	30365210
ebi-a-GCST90002119	rs6903896	CD8 on CD28+ CD	6	31168029
ebi-a-GCST90002119	rs10091979	CD8 on CD28+ CD	8	124435289
ebi-a-GCST90002119	rs56250120	CD8 on CD28+ CD	9	120599704
ebi-a-GCST90002119	rs191051785	CD8 on CD28+ CD	10	32003966
ebi-a-GCST90002119	rs7935549	CD8 on CD28+ CD	11	134312341
ebi-a-GCST90002119	rs201585667	CD8 on CD28+ CD	11	24881116
ebi-a-GCST90002119	rs404977	CD8 on CD28+ CD	11	35132267
ebi-a-GCST90002119	rs76872865	CD8 on CD28+ CD	12	10727673

ebi-a-GCST90002119	rs141200631	CD8 on CD28+ CD	13	94482351
ebi-a-GCST90002119	rs80190728	CD8 on CD28+ CD	17	47474902
ebi-a-GCST90002120	rs11121500	CD8 on CD28- CD	1	6279616
ebi-a-GCST90002120	rs960502	CD8 on CD28- CD	1	55612278
ebi-a-GCST90002120	rs7425430	CD8 on CD28- CD	2	87056480
ebi-a-GCST90002120	rs183795272	CD8 on CD28- CD	2	225921583
ebi-a-GCST90002120	rs533695182	CD8 on CD28- CD	2	88045094
ebi-a-GCST90002120	rs9998963	CD8 on CD28- CD	4	155857432
ebi-a-GCST90002120	rs9884179	CD8 on CD28- CD	4	116408927
ebi-a-GCST90002120	rs13157900	CD8 on CD28- CD	5	79654971
ebi-a-GCST90002120	rs78689026	CD8 on CD28- CD	6	30740038
ebi-a-GCST90002120	rs56262516	CD8 on CD28- CD	6	29930939
ebi-a-GCST90002120	rs9263601	CD8 on CD28- CD	6	31071758
ebi-a-GCST90002120	rs12537451	CD8 on CD28- CD	7	49270809
ebi-a-GCST90002120	rs143440491	CD8 on CD28- CD	7	46541742
ebi-a-GCST90002120	rs147279479	CD8 on CD28- CD	7	145302741
ebi-a-GCST90002120	rs2588120	CD8 on CD28- CD	8	17465263
ebi-a-GCST90002120	rs11014701	CD8 on CD28- CD	10	25999240
ebi-a-GCST90002120	rs189927142	CD8 on CD28- CD	10	27516755
ebi-a-GCST90002120	rs80155370	CD8 on CD28- CD	11	35632392
ebi-a-GCST90002120	rs7134487	CD8 on CD28- CD	12	108486680
ebi-a-GCST90002120	rs62034403	CD8 on CD28- CD	16	78368600
ebi-a-GCST90002120	rs6139531	CD8 on CD28- CD	20	4707066
ebi-a-GCST90002121	rs144661184	CD8 on CD39+ CD	1	67934641
ebi-a-GCST90002121	rs13017213	CD8 on CD39+ CD	2	79976286
ebi-a-GCST90002121	rs76748045	CD8 on CD39+ CD	2	21986200
ebi-a-GCST90002121	rs75482992	CD8 on CD39+ CD	2	237394959
ebi-a-GCST90002121	rs72847706	CD8 on CD39+ CD	2	87055981
ebi-a-GCST90002121	rs75071804	CD8 on CD39+ CD	2	14847536
ebi-a-GCST90002121	rs148989317	CD8 on CD39+ CD	3	59931183
ebi-a-GCST90002121	rs75329937	CD8 on CD39+ CD	3	194563616
ebi-a-GCST90002121	rs9284960	CD8 on CD39+ CD	5	17107957
ebi-a-GCST90002121	rs3128900	CD8 on CD39+ CD	6	29833057
ebi-a-GCST90002121	rs55740958	CD8 on CD39+ CD	6	32073700
ebi-a-GCST90002121	rs17197199	CD8 on CD39+ CD	6	31138472
ebi-a-GCST90002121	rs9357097	CD8 on CD39+ CD	6	30285121
ebi-a-GCST90002121	rs111359007	CD8 on CD39+ CD	6	31761201
ebi-a-GCST90002121	rs140045313	CD8 on CD39+ CD	7	96366491
ebi-a-GCST90002121	rs10974005	CD8 on CD39+ CD	9	38733270
ebi-a-GCST90002121	rs11008428	CD8 on CD39+ CD	10	31536806
ebi-a-GCST90002121	rs117519014	CD8 on CD39+ CD	10	97403248
ebi-a-GCST90002121	rs4237773	CD8 on CD39+ CD	11	6031584
ebi-a-GCST90002121	rs17635782	CD8 on CD39+ CD	11	38978717
ebi-a-GCST90002121	rs1493960	CD8 on CD39+ CD	11	12344362
ebi-a-GCST90002121	rs34134566	CD8 on CD39+ CD	14	91842928
ebi-a-GCST90002121	rs112485259	CD8 on CD39+ CD	16	52061326
ebi-a-GCST90002121	rs71382764	CD8 on CD39+ CD	16	49736424
ebi-a-GCST90002121	rs17634993	CD8 on CD39+ CD	17	38679607

l variables (IVs) for immune cells

effect_allele	other_allele	beta	SE	EAF	samplesi	P value	R2
T	C	0.5864	0.12	0.01	3658	1.09E-06	0.006474959
T	A	-0.2355	0.053	0.0547	3658	7.91E-06	0.005443782
G	T	0.1829	0.041	0.0998	3658	9.67E-06	0.005335444
G	A	1.402	0.3	0.0019	3658	2.99E-06	0.00594687
G	A	0.9378	0.188	0.9956	3658	6.10E-07	0.006777891
T	A	0.1542	0.028	0.284	3658	2.38E-08	0.00847917
G	C	0.49	0.105	0.0142	3658	2.83E-06	0.005974662
G	A	0.1454	0.03	0.4718	3658	1.92E-06	0.006178438
C	T	0.2426	0.055	0.0522	3658	1.00E-05	0.005319469
C	T	0.1184	0.026	0.6006	3658	3.84E-06	0.005813633
T	C	-0.152	0.032	0.816	3658	1.65E-06	0.006257791
T	C	0.4421	0.094	0.0179	3658	2.43E-06	0.006056701
T	C	0.3904	0.081	0.0231	3658	1.49E-06	0.006315044
T	G	-0.4199	0.094	0.0171	3658	8.75E-06	0.005388822
T	C	0.1273	0.029	0.2508	3658	9.63E-06	0.005342173
C	T	-0.174	0.035	0.1471	3658	6.08E-07	0.006780202
A	T	-0.4088	0.089	0.0193	3658	4.75E-06	0.005707751
G	A	0.1675	0.038	0.108	3656	8.94E-06	0.005381697
T	C	0.6503	0.142	0.0068	3656	4.44E-06	0.005743898
T	C	5.387	1.184	0.0004	3656	5.51E-06	0.005630309
T	C	0.3487	0.074	0.0265	3656	2.71E-06	0.006002857
C	T	-0.1382	0.027	0.7254	3656	2.10E-07	0.007340074
C	T	1.249	0.257	0.0023	3656	1.29E-06	0.006399022
C	G	-0.3262	0.072	0.0286	3656	6.29E-06	0.005566045
T	G	-0.1396	0.027	0.2637	3656	2.85E-07	0.007184779
G	A	1.533	0.321	0.0014	3656	1.80E-06	0.006215034
C	T	-0.3568	0.063	0.0368	3656	1.57E-08	0.008707945
G	T	0.6111	0.131	0.0098	3656	3.02E-06	0.00594399
C	T	-0.1116	0.025	0.3362	3656	9.06E-06	0.005373888
A	G	-0.4579	0.086	0.0194	3656	1.09E-07	0.007685685
C	T	-0.2956	0.063	0.0361	3656	2.36E-06	0.00607353
A	G	-0.2576	0.044	0.0814	3656	6.16E-09	0.009196773
A	G	1.665	0.362	0.001	3656	4.39E-06	0.005753067
A	G	-0.1264	0.028	0.2299	3656	8.15E-06	0.005434537
A	T	-0.4238	0.095	0.0159	3656	8.67E-06	0.005398073
G	A	-0.2229	0.049	0.9375	3656	6.15E-06	0.00557604
C	T	-0.4626	0.096	0.0157	3656	1.42E-06	0.006342689
A	C	-0.1256	0.024	0.5309	3656	2.06E-07	0.007356172
C	T	0.4146	0.085	0.0198	3656	1.23E-06	0.006417335
G	T	-0.1279	0.027	0.2657	3656	1.69E-06	0.006255891
T	C	-0.1118	0.025	0.6458	3656	8.81E-06	0.005393063
A	G	0.1453	0.031	0.1886	3656	2.24E-06	0.006097588
T	C	0.3555	0.078	0.0231	3656	5.55E-06	0.005628127
G	T	1.518	0.34	0.0011	3656	8.23E-06	0.005425905
T	G	-0.1526	0.032	0.8166	3656	2.40E-06	0.006068124

G	A	-0.2007	0.044	0.9219	3656	5.46E-06	0.005638321
C	T	0.2489	0.054	0.0494	3656	4.56E-06	0.005728873
T	A	-0.1364	0.027	0.4267	3656	3.47E-07	0.007077259
T	C	0.3861	0.086	0.0201	3657	7.14E-06	0.005497907
C	T	-0.1211	0.027	0.3054	3657	6.02E-06	0.005589606
A	C	0.115	0.026	0.6459	3657	7.16E-06	0.005496371
G	A	0.1189	0.025	0.4701	3657	1.79E-06	0.006216249
G	T	-0.187	0.04	0.1097	3657	2.94E-06	0.005958632
C	G	0.1101	0.025	0.4346	3657	9.18E-06	0.005369193
G	A	0.1204	0.027	0.3018	3657	7.46E-06	0.005476483
C	T	0.763	0.167	0.0051	3657	4.75E-06	0.005709642
G	A	-0.3635	0.081	0.0283	3657	6.80E-06	0.005522837
A	T	1.216	0.266	0.0022	3657	4.82E-06	0.005703338
T	C	-0.2771	0.053	0.0582	3657	2.34E-07	0.007284965
A	G	0.1107	0.025	0.5022	3657	7.24E-06	0.005493499
C	T	0.1327	0.027	0.3154	3657	7.23E-07	0.00669425
G	A	-0.1265	0.025	0.5425	3657	2.90E-07	0.007173092
G	A	0.1261	0.025	0.4837	3657	3.13E-07	0.007128123
C	T	0.2092	0.047	0.0764	3657	7.39E-06	0.005480747
T	C	0.2934	0.059	0.044	3657	7.22E-07	0.00669427
T	C	1.476	0.332	0.0014	3656	9.05E-06	0.005377109
G	A	0.5256	0.117	0.0115	3656	7.72E-06	0.005461727
G	A	0.6732	0.141	0.0075	3656	1.78E-06	0.006222758
C	T	-0.2225	0.044	0.081	3656	5.37E-07	0.006849602
A	G	-0.1966	0.042	0.087	3656	3.57E-06	0.005862805
T	C	2.534	0.572	0.0004	3656	9.63E-06	0.005343084
T	C	1.849	0.398	0.001	3656	3.49E-06	0.005871678
T	A	0.2476	0.056	0.049	3656	9.25E-06	0.005362394
A	G	2.216	0.443	0.0007	3656	5.96E-07	0.006794678
C	T	-0.4549	0.096	0.0157	3656	2.38E-06	0.006071404
G	T	0.327	0.074	0.028	3656	8.89E-06	0.005383345
G	T	-0.1253	0.027	0.2657	3656	3.03E-06	0.005943434
C	T	0.1175	0.024	0.5621	3656	1.13E-06	0.006465157
T	C	-0.1942	0.043	0.084	3656	5.88E-06	0.005599716
C	T	0.3801	0.084	0.0208	3656	5.63E-06	0.005622485
T	C	-0.4319	0.09	0.0176	3656	1.64E-06	0.006265155
A	G	0.13	0.027	0.2619	3656	1.99E-06	0.006164103
A	G	0.5528	0.106	0.0126	3656	2.09E-07	0.007342816
A	G	0.1764	0.038	0.1091	3656	3.91E-06	0.005807871
C	T	0.2458	0.055	0.049	3656	6.93E-06	0.00551279
C	G	1.912	0.393	0.001	3656	1.19E-06	0.006435784
T	C	-0.1385	0.031	0.185	3656	6.01E-06	0.005586677
T	A	-0.1486	0.027	0.4148	3656	5.31E-08	0.008068266
T	G	0.1093	0.024	0.4699	3656	4.42E-06	0.005750046
A	G	0.1183	0.024	0.4571	3656	7.70E-07	0.00665682
C	T	-0.2175	0.047	0.0699	3656	3.31E-06	0.005895554
A	G	-0.1408	0.031	0.1916	3657	5.94E-06	0.005595002
A	G	0.202	0.039	0.1084	3657	2.07E-07	0.007349589

T	C	0.4948	0.11	0.0133	3657	7.21E-06	0.005492472
G	T	-0.4914	0.106	0.0137	3657	3.88E-06	0.005809629
T	A	0.308	0.068	0.033	3657	6.81E-06	0.005521977
G	A	-0.4129	0.092	0.0175	3657	7.76E-06	0.005454159
T	C	0.1498	0.032	0.2901	3657	2.13E-06	0.006126761
C	A	0.1793	0.033	0.1687	3657	3.91E-08	0.00822395
A	G	0.2539	0.054	0.0734	3657	3.09E-06	0.005934392
T	G	0.1333	0.029	0.2404	3657	4.40E-06	0.005748245
G	A	0.1168	0.026	0.628	3657	5.74E-06	0.005607594
T	A	0.1209	0.027	0.2759	3657	8.58E-06	0.005404986
G	A	-0.1169	0.026	0.5384	3657	7.72E-06	0.005451503
C	T	0.1407	0.031	0.1874	3657	6.25E-06	0.005565684
T	C	-2.911	0.647	0.0004	3657	7.01E-06	0.005506644
G	A	0.9733	0.216	0.0033	3657	6.84E-06	0.005521491
T	A	0.112	0.025	0.3589	3657	9.81E-06	0.00533446
C	G	0.3388	0.057	0.045	3657	4.10E-09	0.009410701
A	G	-0.4608	0.091	0.018	3657	4.35E-07	0.006959743
C	T	-2.756	0.619	0.0004	3657	8.78E-06	0.005387971
C	G	-0.1169	0.024	0.5386	3657	1.78E-06	0.006217176
A	G	-0.5276	0.11	0.0126	3657	1.73E-06	0.006240098
G	A	0.1246	0.024	0.5425	3657	3.63E-07	0.007051478
C	T	-0.2138	0.046	0.0764	3657	4.01E-06	0.005799493
T	G	0.3463	0.077	0.0268	3657	7.54E-06	0.00546657
G	A	-0.1336	0.029	0.233	3656	5.43E-06	0.005643192
G	C	-0.3526	0.078	0.0263	3656	5.85E-06	0.005604016
A	G	0.1854	0.039	0.1184	3656	2.74E-06	0.006001844
C	A	-0.278	0.062	0.0406	3656	8.09E-06	0.005434207
A	G	0.3025	0.064	0.0383	3656	2.59E-06	0.006028475
T	A	0.174	0.038	0.1554	3656	4.88E-06	0.005699206
T	C	-0.1686	0.036	0.1415	3656	2.99E-06	0.005953701
C	T	-0.2247	0.049	0.0687	3656	5.07E-06	0.005677409
T	C	0.6074	0.136	0.0086	3656	8.70E-06	0.005394677
A	G	-0.1114	0.025	0.4751	3656	6.49E-06	0.00554639
C	T	0.1258	0.028	0.7213	3656	6.38E-06	0.005553898
A	G	-0.165	0.037	0.1358	3656	8.79E-06	0.00538976
T	G	-0.3372	0.074	0.0291	3656	4.77E-06	0.005707024
G	A	0.1363	0.027	0.3256	3656	3.42E-07	0.007082742
C	G	0.3229	0.058	0.045	3656	2.85E-08	0.008389134
A	G	-0.4107	0.092	0.0181	3656	8.84E-06	0.005387507
G	T	0.1176	0.025	0.5581	3656	3.21E-06	0.005916781
A	G	0.4628	0.094	0.0175	3656	8.58E-07	0.00660324
T	C	-0.1152	0.026	0.5153	3657	7.81E-06	0.00545163
T	G	1.017	0.222	0.0027	3657	4.87E-06	0.005695714
T	C	-0.4409	0.087	0.0201	3657	4.15E-07	0.006981894
A	T	0.1254	0.028	0.2961	3657	5.45E-06	0.005633437
C	T	0.2907	0.063	0.0392	3657	4.39E-06	0.005748472
G	A	-0.1728	0.038	0.1354	3657	4.98E-06	0.005685029
A	G	0.2054	0.043	0.0913	3657	2.31E-06	0.006084791

G	A	0.294	0.063	0.0421	3657	2.81E-06	0.005983873
G	A	-0.2606	0.058	0.0488	3657	8.19E-06	0.005426601
G	A	-0.1978	0.044	0.0905	3657	7.53E-06	0.005468548
C	T	0.9467	0.208	0.0036	3657	5.23E-06	0.005659768
A	G	-0.1186	0.026	0.3683	3657	5.78E-06	0.005606045
C	T	0.1675	0.035	0.1509	3657	1.85E-06	0.00620267
T	C	-0.1204	0.025	0.5621	3657	2.03E-06	0.006154684
T	C	-0.5404	0.115	0.0115	3657	2.47E-06	0.00605421
T	C	-0.1402	0.031	0.4003	3657	8.41E-06	0.0054116
T	C	-0.1252	0.026	0.5945	3657	9.97E-07	0.006523184
G	A	-0.137	0.03	0.2419	3657	3.55E-06	0.00586298
A	T	-0.5815	0.128	0.0096	3657	5.76E-06	0.005611907
C	G	0.5187	0.109	0.013	3657	2.09E-06	0.006131843
T	A	0.1229	0.027	0.299	3657	6.24E-06	0.005563863
T	C	-0.1256	0.027	0.3275	3657	2.61E-06	0.00601915
T	C	0.5551	0.09	0.02	3657	7.31E-10	0.010320231
T	C	-0.1335	0.03	0.2052	3657	8.72E-06	0.005396523
A	G	0.2174	0.048	0.0708	3657	7.48E-06	0.005470985
G	A	0.4523	0.102	0.0146	3657	9.53E-06	0.005348093
C	G	0.3067	0.058	0.045	3657	1.46E-07	0.007531387
A	G	0.2759	0.058	0.0503	3657	1.96E-06	0.006170681
G	T	0.2459	0.054	0.0552	3657	5.34E-06	0.005646625
A	G	0.1208	0.026	0.6259	3657	3.06E-06	0.005936112
C	T	-0.1236	0.027	0.6318	3657	4.24E-06	0.005774037
A	G	-1.906	0.417	0.0007	3657	4.94E-06	0.005688481
T	C	0.852	0.192	0.0042	3657	9.66E-06	0.005339132
T	C	-0.3814	0.086	0.0201	3656	8.58E-06	0.005403282
T	C	-0.2347	0.052	0.0584	3656	7.43E-06	0.00547603
G	C	0.3026	0.068	0.0334	3656	8.29E-06	0.005422098
A	T	-0.1331	0.027	0.3005	3656	9.73E-07	0.006535556
T	C	0.5999	0.135	0.0086	3656	8.70E-06	0.005395933
G	A	0.2056	0.045	0.077	3656	6.41E-06	0.005556334
G	T	-0.2912	0.063	0.0414	3656	4.00E-06	0.005798863
G	T	0.1114	0.024	0.4521	3656	5.42E-06	0.005641496
A	G	-0.1468	0.031	0.1972	3656	2.19E-06	0.006111974
G	A	0.1523	0.026	0.3256	3656	8.62E-09	0.009027684
G	A	1.924	0.385	0.001	3656	6.09E-07	0.006788128
T	G	0.4126	0.089	0.0194	3656	3.43E-06	0.00588227
T	C	0.4968	0.088	0.02	3656	2.09E-08	0.008555194
C	G	0.3067	0.057	0.045	3656	8.87E-08	0.007794273
A	G	0.268	0.057	0.0502	3656	2.71E-06	0.006004008
C	T	-0.123	0.025	0.5737	3656	6.71E-07	0.006731706
T	C	-0.1813	0.04	0.1034	3656	6.63E-06	0.00553532
C	T	-0.1112	0.024	0.475	3656	4.21E-06	0.005780045
A	G	0.1184	0.025	0.6291	3656	2.95E-06	0.005964103
A	G	-0.2602	0.058	0.0435	3656	7.43E-06	0.005476677
A	G	-0.1345	0.026	0.6359	3656	3.66E-07	0.007044197
T	C	0.214	0.047	0.074	3656	5.76E-06	0.00561006

T	G	-0.6497	0.144	0.0068	3657	6.61E-06	0.005535604
T	C	0.3455	0.074	0.029	3657	3.14E-06	0.00592393
A	G	-0.1134	0.025	0.554	3657	6.97E-06	0.005511182
C	T	-0.3526	0.054	0.0562	3657	5.92E-11	0.011647725
A	G	0.7602	0.164	0.006	3657	3.81E-06	0.005827019
T	C	0.1158	0.025	0.5505	3657	4.73E-06	0.005709459
G	A	0.1466	0.024	0.5339	3657	2.13E-09	0.009758716
A	G	-0.1297	0.028	0.4751	3657	2.61E-06	0.006019693
G	A	-0.1678	0.036	0.1362	3657	2.77E-06	0.00599156
G	C	-0.12	0.027	0.2786	3657	9.41E-06	0.00535269
C	T	0.4671	0.097	0.0161	3657	1.70E-06	0.006245804
A	G	-0.1226	0.027	0.2811	3657	5.52E-06	0.00562714
G	T	0.3898	0.026	0.6314	3657	7.20E-50	0.058538321
A	G	0.1776	0.037	0.12	3657	2.21E-06	0.006108917
T	C	-0.1811	0.035	0.1514	3657	2.37E-07	0.007276136
T	C	0.2217	0.05	0.0649	3657	8.39E-06	0.005411741
C	A	-0.3043	0.061	0.0406	3657	7.40E-07	0.006682484
A	G	2.08	0.449	0.0007	3657	3.74E-06	0.005831437
C	T	0.1254	0.027	0.2963	3657	4.39E-06	0.005753241
A	G	-0.2583	0.057	0.0485	3657	6.22E-06	0.005568405
G	C	-0.3853	0.085	0.0221	3657	5.73E-06	0.00561222
T	C	-0.2314	0.044	0.0839	3657	1.28E-07	0.007595093
A	C	0.1185	0.026	0.3173	3657	5.12E-06	0.005669799
G	A	0.1309	0.028	0.7123	3657	2.71E-06	0.006004658
T	C	0.8269	0.17	0.0055	3657	1.15E-06	0.006450699
G	A	-0.9845	0.221	0.0037	3657	8.43E-06	0.005411842
A	T	0.1284	0.024	0.5405	3657	1.53E-07	0.007503138
T	G	-0.1332	0.027	0.3033	3657	6.86E-07	0.006714477
A	G	0.1525	0.033	0.1557	3657	3.47E-06	0.005872787
C	G	1.831	0.403	0.001	3657	5.76E-06	0.00560749
T	G	-0.1212	0.024	0.5919	3657	7.77E-07	0.00665279
T	A	-0.1669	0.029	0.4148	3657	5.12E-09	0.009290611
A	C	0.2021	0.045	0.0759	3657	9.10E-06	0.005373014
A	G	0.1175	0.025	0.6292	3657	3.43E-06	0.005877333
T	C	0.522	0.117	0.0107	3657	8.15E-06	0.005432063
T	G	-0.1238	0.027	0.3135	3656	4.45E-06	0.005751484
A	G	0.2278	0.049	0.9334	3656	3.21E-06	0.005915253
T	C	-0.3205	0.07	0.0312	3656	4.89E-06	0.005694785
T	C	-0.1387	0.031	0.2221	3656	7.36E-06	0.005480787
G	C	0.2313	0.05	0.0643	3656	4.18E-06	0.005777862
A	G	-0.2567	0.057	0.0472	3656	8.21E-06	0.005425619
C	T	-0.1216	0.025	0.5196	3656	1.24E-06	0.006409142
A	T	-0.1375	0.03	0.7882	3656	6.15E-06	0.00557547
A	G	-0.2787	0.057	0.0509	3656	1.03E-06	0.006507957
C	T	-0.2808	0.054	0.0562	3656	2.02E-07	0.007360692
G	A	-0.263	0.059	0.0466	3656	9.96E-06	0.005326333
T	A	0.173	0.039	0.1171	3656	8.37E-06	0.005413932
A	G	0.6631	0.146	0.0066	3656	5.96E-06	0.00559526

A	G	0.3826	0.08	0.0234	3656	1.62E-06	0.006273187
A	G	0.2796	0.056	0.0528	3656	5.77E-07	0.006813398
A	G	-0.3304	0.074	0.0279	3656	9.28E-06	0.005362403
G	T	0.6463	0.143	0.009	3656	6.19E-06	0.005571591
C	T	-0.1795	0.04	0.1146	3656	8.65E-06	0.005397123
T	G	-0.1467	0.032	0.176	3656	6.50E-06	0.005545485
G	A	0.1359	0.025	0.5341	3656	3.13E-08	0.008338912
C	A	-0.1109	0.025	0.4944	3656	8.20E-06	0.005431105
A	C	-0.2897	0.063	0.9583	3656	3.97E-06	0.005805333
C	T	-0.1204	0.026	0.6704	3656	5.04E-06	0.005673941
A	T	-0.2296	0.05	0.0643	3656	5.33E-06	0.005653325
G	C	0.1135	0.024	0.5327	3656	3.39E-06	0.0058836
C	T	0.3825	0.08	0.0239	3656	1.95E-06	0.00617403
C	T	0.2841	0.026	0.6002	3656	1.64E-27	0.031790729
G	C	-0.1107	0.025	0.4683	3656	9.36E-06	0.005355694
G	A	-0.1733	0.035	0.1421	3656	6.95E-07	0.006714448
T	C	-0.5733	0.121	0.01	3657	2.37E-06	0.006071173
C	T	0.1189	0.026	0.5387	3657	3.59E-06	0.005859591
G	C	0.1327	0.03	0.2363	3657	7.14E-06	0.005499
G	A	-1.348	0.302	0.0019	3657	8.49E-06	0.005407837
T	G	1.008	0.211	0.0037	3657	1.75E-06	0.006231279
T	A	-0.1414	0.028	0.2841	3657	4.40E-07	0.006949945
C	T	-0.1286	0.029	0.7383	3657	6.85E-06	0.005517494
C	T	-0.1543	0.032	0.4717	3657	1.10E-06	0.006477545
T	C	0.1522	0.032	0.816	3657	2.25E-06	0.00610232
T	C	-0.4624	0.095	0.0179	3657	1.11E-06	0.006467716
A	G	-0.1181	0.026	0.4881	3657	6.11E-06	0.005580363
T	C	-0.407	0.082	0.0231	3657	7.31E-07	0.006686591
G	A	0.1985	0.043	0.0958	3657	3.74E-06	0.005833825
C	T	0.1769	0.035	0.1471	3657	5.43E-07	0.006843474
T	C	-0.2707	0.059	0.0435	3656	5.51E-06	0.005631573
G	C	0.3537	0.075	0.0286	3656	2.20E-06	0.006114424
C	T	0.1511	0.031	0.1957	3656	1.35E-06	0.006366244
C	T	0.1458	0.031	0.1893	3656	3.02E-06	0.005945207
C	T	0.1794	0.04	0.1041	3656	6.66E-06	0.005532225
T	C	0.3583	0.079	0.0235	3656	5.91E-06	0.005597773
A	T	-1.838	0.401	0.0008	3656	4.61E-06	0.005724921
C	G	0.481	0.101	0.0141	3656	1.88E-06	0.006189655
A	G	0.1212	0.027	0.7103	3656	7.07E-06	0.005501557
T	G	0.1223	0.028	0.2705	3656	9.50E-06	0.00534969
A	T	-0.1251	0.027	0.3005	3656	4.54E-06	0.005731642
G	C	0.1297	0.029	0.286	3656	8.87E-06	0.005389491
C	T	-0.1161	0.025	0.5137	3656	3.37E-06	0.005883104
C	T	-0.1238	0.025	0.5715	3656	6.83E-07	0.006721348
T	C	-0.2543	0.054	0.058	3656	2.15E-06	0.006125966
C	T	-0.1356	0.028	0.283	3656	1.02E-06	0.006507359
A	G	0.7964	0.161	0.006	3656	7.87E-07	0.006648254
T	C	0.16	0.033	0.1703	3656	1.78E-06	0.006222863

G	A	0.1323	0.025	0.5044	3656	7.43E-08	0.007887247
T	G	-0.1273	0.025	0.592	3656	2.65E-07	0.00721875
A	C	-0.2923	0.063	0.9583	3656	3.03E-06	0.005943259
A	G	0.9179	0.204	0.0034	3656	7.28E-06	0.005491064
C	A	-0.7487	0.164	0.0056	3656	5.05E-06	0.005682075
G	C	-0.5466	0.111	0.0126	3656	9.03E-07	0.00657717
G	C	-0.1828	0.04	0.103	3656	6.52E-06	0.005544165
A	G	-0.9069	0.192	0.004	3658	2.52E-06	0.006037194
A	G	-0.1287	0.025	0.5671	3658	3.15E-07	0.007130372
G	A	-1.507	0.333	0.0012	3658	6.01E-06	0.005584284
T	A	0.4869	0.106	0.0149	3658	4.45E-06	0.00574568
T	C	0.3286	0.071	0.0316	3658	3.47E-06	0.005872426
A	G	0.1711	0.037	0.1223	3658	4.75E-06	0.005710226
C	T	-0.1749	0.037	0.1211	3658	3.16E-06	0.005920924
T	A	0.1159	0.026	0.6506	3658	5.95E-06	0.005589426
A	G	-0.2427	0.05	0.0633	3658	1.31E-06	0.006379516
G	A	0.5	0.109	0.0124	3658	4.85E-06	0.005698612
C	T	0.2127	0.048	0.0681	3658	8.67E-06	0.005397294
G	A	0.5592	0.12	0.0104	3658	3.54E-06	0.005862517
C	T	0.3429	0.075	0.0279	3658	4.69E-06	0.005713677
C	G	0.3534	0.064	0.0376	3658	3.71E-08	0.008251202
A	G	-0.1199	0.026	0.3324	3658	3.10E-06	0.005928713
T	C	-0.2223	0.049	0.0678	3658	6.49E-06	0.005545452
A	C	-0.1669	0.033	0.1607	3658	3.78E-07	0.007028426
C	T	-0.257	0.056	0.051	3658	4.23E-06	0.005769691
T	C	-0.4648	0.103	0.0142	3658	6.25E-06	0.0055683
A	G	1.708	0.345	0.0015	3658	7.61E-07	0.006659533
A	G	0.1576	0.034	0.1595	3658	3.02E-06	0.005943197
C	G	0.2879	0.063	0.0381	3658	4.52E-06	0.005734349
C	G	-0.2036	0.045	0.0832	3658	5.43E-06	0.005639501
A	C	-0.1407	0.028	0.7365	3658	4.57E-07	0.006934005
A	G	0.4702	0.105	0.0137	3658	7.46E-06	0.005472876
C	T	0.154	0.032	0.1706	3658	1.80E-06	0.006210274
T	C	-0.1288	0.029	0.2243	3658	7.74E-06	0.005449231
G	A	0.1338	0.027	0.2881	3658	6.52E-07	0.006742821
T	C	1.467	0.308	0.0015	3657	1.91E-06	0.006185154
A	G	0.1124	0.025	0.5844	3657	5.95E-06	0.005590106
A	G	2.692	0.575	0.0004	3657	2.90E-06	0.005968227
T	A	2.566	0.549	0.0008	3657	3.03E-06	0.005946841
T	C	0.7633	0.17	0.0051	3657	7.02E-06	0.005508268
T	C	1.671	0.368	0.0011	3657	5.68E-06	0.005615585
C	A	-0.2902	0.065	0.036	3657	7.45E-06	0.0054762
C	T	0.3653	0.079	0.0227	3657	3.53E-06	0.005864386
C	T	-0.1773	0.038	0.1061	3657	4.06E-06	0.005786705
C	T	0.1236	0.027	0.7401	3657	7.10E-06	0.00549754
G	A	0.5137	0.107	0.0124	3657	1.74E-06	0.006240022
C	A	-0.1344	0.03	0.8043	3657	8.54E-06	0.005400811
T	C	0.4466	0.098	0.0157	3657	5.43E-06	0.005638769

T	C	0.4919	0.104	0.0135	3657	2.10E-06	0.006138663
A	G	-0.1239	0.026	0.2815	3657	2.79E-06	0.00599141
A	C	-0.16	0.032	0.1608	3657	7.19E-07	0.006698002
C	T	0.1086	0.024	0.5559	3657	6.62E-06	0.005535689
C	T	-0.3422	0.071	0.0304	3657	1.34E-06	0.006367149
G	C	-0.1228	0.027	0.732	3657	6.56E-06	0.005538633
G	A	0.3865	0.086	0.0189	3657	7.58E-06	0.005467371
C	G	0.1932	0.043	0.0807	3657	8.82E-06	0.00538474
A	C	-0.1145	0.026	0.3229	3657	7.79E-06	0.005453179
A	G	-0.1186	0.026	0.3112	3657	4.30E-06	0.005758475
G	C	0.1172	0.024	0.466	3657	1.19E-06	0.006435957
A	T	-0.1141	0.026	0.3179	3657	8.86E-06	0.005385982
T	C	-0.468	0.086	0.0201	3657	6.60E-08	0.007948234
C	T	-0.1354	0.026	0.3805	3657	1.73E-07	0.007434957
T	C	-1.002	0.224	0.0027	3657	7.99E-06	0.005441826
T	C	-0.138	0.025	0.5153	3657	6.03E-08	0.007994589
G	T	0.3572	0.061	0.0444	3657	5.45E-09	0.009262248
A	G	0.426	0.096	0.0161	3657	9.69E-06	0.005338028
A	G	0.2178	0.043	0.0913	3657	4.67E-07	0.006921719
A	G	-0.1745	0.038	0.1181	3657	4.12E-06	0.005784605
T	C	0.1306	0.029	0.7517	3657	6.30E-06	0.005564733
G	A	-0.2768	0.059	0.0447	3657	3.10E-06	0.005932617
G	T	-0.6233	0.126	0.0107	3657	7.27E-07	0.006689215
A	G	-0.1351	0.026	0.3683	3657	2.13E-07	0.007334594
A	C	0.1701	0.036	0.8588	3657	2.44E-06	0.006061162
G	A	0.9926	0.201	0.0037	3657	8.61E-07	0.006598262
C	T	0.1256	0.026	0.4032	3657	1.28E-06	0.00638956
A	G	-0.1488	0.032	0.5249	3657	4.80E-06	0.005702925
C	T	0.3141	0.061	0.9556	3657	2.75E-07	0.007195691
T	A	0.115	0.026	0.3414	3657	7.96E-06	0.005441229
T	C	-0.4176	0.094	0.0179	3657	8.96E-06	0.005376979
T	C	-1.082	0.224	0.003	3657	1.49E-06	0.006317301
A	C	-0.1775	0.039	0.1135	3657	6.93E-06	0.005513627
A	G	0.1294	0.029	0.2428	3657	6.72E-06	0.005528055
T	C	-0.4739	0.102	0.0149	3657	3.63E-06	0.005845217
C	T	0.3691	0.077	0.0257	3657	1.75E-06	0.006231106
G	A	0.4292	0.088	0.0206	3657	1.14E-06	0.006452487
A	G	0.2839	0.051	0.0662	3657	2.26E-08	0.008514552
C	G	0.2733	0.058	0.045	3657	2.73E-06	0.005995782
A	G	0.4209	0.093	0.018	3657	6.45E-06	0.005549627
G	A	0.1104	0.025	0.5425	3657	8.73E-06	0.005394003
A	G	0.538	0.116	0.0108	3656	3.45E-06	0.005879369
G	A	0.1198	0.027	0.3441	3656	8.09E-06	0.00543589
A	C	2.504	0.522	0.0005	3656	1.68E-06	0.006254558
T	C	-0.1641	0.036	0.1415	3656	5.39E-06	0.005648132
A	G	0.1371	0.031	0.2009	3656	7.27E-06	0.005489201
G	T	0.1235	0.028	0.2798	3656	8.97E-06	0.005380627
C	T	0.1259	0.028	0.7213	3656	6.27E-06	0.005566658

A	G	-0.1301	0.028	0.2884	3656	2.84E-06	0.005976119
T	C	0.305	0.067	0.0356	3656	5.81E-06	0.005606243
G	A	1.829	0.389	0.001	3656	2.69E-06	0.006007336
G	A	0.173	0.027	0.3256	3656	9.14E-11	0.011428893
G	C	-0.2972	0.061	0.0432	3656	1.36E-06	0.006365607
C	G	0.2713	0.058	0.045	3656	2.95E-06	0.005959238
G	A	1.204	0.252	0.0025	3656	1.90E-06	0.006190354
T	G	-0.1161	0.025	0.592	3656	2.94E-06	0.005968372
A	G	0.6102	0.121	0.0103	3656	4.46E-07	0.006942217
G	T	-0.4235	0.087	0.9792	3656	1.22E-06	0.006420485
C	T	0.1371	0.026	0.3805	3657	1.28E-07	0.007603884
T	C	0.1312	0.025	0.5153	3657	2.75E-07	0.007203525
T	C	0.463	0.087	0.0201	3657	9.55E-08	0.00775568
T	C	1.014	0.224	0.0027	3657	6.31E-06	0.005557406
A	G	-0.2131	0.043	0.0913	3657	8.50E-07	0.006606852
T	C	0.2616	0.056	0.0528	3657	3.62E-06	0.005852631
G	T	-0.3399	0.061	0.0444	3657	3.01E-08	0.008361534
A	G	0.1678	0.038	0.1181	3657	9.81E-06	0.005334415
G	A	0.2687	0.059	0.0447	3657	6.17E-06	0.005573674
G	T	0.6035	0.126	0.0107	3657	1.68E-06	0.006253797
G	A	-1.013	0.202	0.0037	3657	5.37E-07	0.006856851
A	C	-0.1667	0.036	0.8588	3657	3.99E-06	0.005803439
A	G	0.1314	0.026	0.3683	3657	4.65E-07	0.00691993
C	T	-0.1251	0.026	0.4032	3657	1.46E-06	0.006324544
C	T	-0.3012	0.061	0.9556	3657	8.68E-07	0.006599104
A	G	0.1477	0.033	0.5261	3657	6.89E-06	0.005517601
T	A	-0.1163	0.026	0.3414	3657	6.46E-06	0.005547077
T	C	1.077	0.225	0.003	3657	1.72E-06	0.006242817
A	G	-0.1306	0.029	0.2428	3657	5.66E-06	0.005614906
A	G	0.1801	0.039	0.114	3657	4.91E-06	0.005689755
T	C	0.4852	0.102	0.0149	3657	2.19E-06	0.006113671
G	A	-0.4222	0.088	0.0206	3657	1.76E-06	0.00622535
C	T	-0.388	0.077	0.0257	3657	5.20E-07	0.006865145
T	C	0.1119	0.025	0.5659	3657	9.30E-06	0.005358658
C	G	-0.2722	0.058	0.045	3657	3.10E-06	0.005929653
A	G	-0.1154	0.025	0.5141	3657	4.08E-06	0.005792734
A	G	-0.2677	0.051	0.0662	3657	1.42E-07	0.007548107
A	G	-0.4239	0.093	0.018	3657	5.72E-06	0.005612987
G	C	-2.179	0.482	0.0005	3657	6.42E-06	0.005550571
T	C	-0.1159	0.025	0.382	3656	4.37E-06	0.005757009
T	C	-0.2134	0.047	0.0744	3656	6.01E-06	0.005588252
T	C	2.627	0.579	0.0004	3656	5.91E-06	0.005599101
A	G	0.233	0.052	0.0613	3656	7.40E-06	0.005478359
C	T	-0.2366	0.046	0.081	3656	2.36E-07	0.007278108
C	T	0.1232	0.027	0.2964	3656	7.30E-06	0.005487492
T	C	0.1584	0.034	0.1536	3656	3.71E-06	0.005836651
A	G	-0.2668	0.057	0.0486	3656	3.51E-06	0.005868561
C	T	-0.4624	0.099	0.0157	3656	3.30E-06	0.005900797

G	T	-0.1445	0.028	0.2657	3656	1.74E-07	0.007436269
C	T	0.1251	0.025	0.5621	3656	4.60E-07	0.006928464
T	C	0.7902	0.171	0.0055	3656	3.92E-06	0.005813685
A	G	-0.4129	0.092	0.0178	3656	8.13E-06	0.005431009
A	G	0.138	0.028	0.2619	3656	9.07E-07	0.006576889
A	G	0.1183	0.027	0.32	3656	8.96E-06	0.005380923
G	A	0.1702	0.036	0.1302	3656	2.84E-06	0.005977176
G	A	-0.1163	0.025	0.6075	3656	3.63E-06	0.005851883
T	G	-0.1229	0.027	0.3032	3656	5.33E-06	0.005647756
A	G	-0.1886	0.041	0.0989	3656	5.34E-06	0.005649832
C	G	1.819	0.406	0.001	3656	7.65E-06	0.005465814
A	G	-0.3088	0.067	0.0325	3656	4.34E-06	0.005757926
T	A	-0.1605	0.029	0.4148	3656	2.35E-08	0.008493393
G	C	0.1382	0.03	0.2083	3656	3.31E-06	0.005895412
C	T	-0.2347	0.048	0.0699	3656	1.08E-06	0.006483482
A	G	-0.1407	0.025	0.567	3657	1.49E-08	0.008731752
A	T	-0.1078	0.024	0.5554	3657	7.79E-06	0.005454876
A	G	0.1185	0.024	0.4619	3657	9.61E-07	0.006546134
G	A	0.3884	0.081	0.0219	3657	1.88E-06	0.006193178
G	A	0.1247	0.028	0.7616	3657	8.56E-06	0.00539823
A	C	0.3165	0.07	0.0294	3657	6.10E-06	0.005578114
G	A	0.4094	0.088	0.0209	3657	3.22E-06	0.005912947
T	A	-0.5032	0.108	0.0123	3657	3.40E-06	0.005879506
C	T	-0.3437	0.066	0.034	3657	2.04E-07	0.007354375
A	G	-0.1235	0.027	0.2801	3657	5.46E-06	0.005638633
C	T	-0.118	0.024	0.5103	3657	1.39E-06	0.006354638
G	A	0.44	0.098	0.0142	3657	7.33E-06	0.005482023
C	G	0.3227	0.063	0.0376	3657	3.64E-07	0.007054299
C	G	-0.6636	0.141	0.007	3657	2.76E-06	0.005995039
A	T	0.2024	0.045	0.0766	3657	8.53E-06	0.005403065
C	A	0.1745	0.035	0.1333	3657	6.44E-07	0.00675514
A	G	-0.1152	0.025	0.369	3657	4.40E-06	0.005745337
G	C	-0.1257	0.027	0.732	3657	4.44E-06	0.005742879
G	C	0.1701	0.037	0.12	3657	3.95E-06	0.005808421
T	C	-0.4552	0.087	0.0201	3657	1.99E-07	0.007367891
C	T	-0.1336	0.03	0.2311	3657	9.70E-06	0.005340571
G	A	0.2797	0.063	0.0421	3657	9.25E-06	0.005360982
C	G	0.3685	0.082	0.0235	3657	7.49E-06	0.005470748
A	G	-0.1214	0.027	0.6924	3657	8.46E-06	0.005405838
G	A	0.4368	0.091	0.064	3657	1.52E-06	0.006303406
C	T	-0.1674	0.037	0.1333	3657	5.28E-06	0.00565705
T	A	-0.2834	0.063	0.0418	3657	7.05E-06	0.005504707
C	T	-0.1237	0.027	0.3121	3657	3.92E-06	0.005813461
C	T	-0.1289	0.029	0.2762	3657	7.63E-06	0.005466691
A	G	-0.4312	0.091	0.0172	3657	2.51E-06	0.006042725
T	C	-0.4808	0.103	0.0149	3657	3.24E-06	0.005911689
G	A	0.1313	0.027	0.3257	3657	1.20E-06	0.006425066
T	C	-0.1353	0.027	0.3266	3657	4.79E-07	0.006905891

G	A	0.4747	0.089	0.0206	3657	1.08E-07	0.007688236
T	G	0.4126	0.091	0.0194	3657	5.50E-06	0.005633062
C	G	0.2816	0.059	0.045	3657	1.57E-06	0.00628562
T	C	0.2453	0.055	0.0582	3657	7.15E-06	0.005495019
A	T	-0.1204	0.027	0.704	3657	9.00E-06	0.005380325
A	T	0.1207	0.026	0.3234	3657	4.73E-06	0.00570915
T	A	0.2862	0.063	0.0418	3657	6.54E-06	0.005543177
G	T	-0.1868	0.038	0.8907	3656	1.17E-06	0.006441064
G	T	0.1323	0.03	0.2261	3656	7.52E-06	0.005471253
A	G	-0.1191	0.026	0.6385	3656	3.30E-06	0.005899108
T	C	-0.3012	0.063	0.039	3656	1.51E-06	0.00631038
C	A	0.197	0.042	0.9123	3656	3.08E-06	0.005939419
G	A	0.1176	0.026	0.3155	3656	7.86E-06	0.005447353
T	C	0.2175	0.049	0.0636	3656	9.71E-06	0.005338566
T	A	-0.1304	0.029	0.2247	3656	6.47E-06	0.005549306
C	A	-0.1831	0.04	0.1027	3656	5.67E-06	0.005617325
A	G	0.3121	0.06	0.0402	3656	2.14E-07	0.007334295
C	T	0.3005	0.06	0.0418	3656	5.23E-07	0.006864038
G	A	1.819	0.382	0.001	3656	1.92E-06	0.00617985
T	G	0.4292	0.088	0.0194	3656	1.02E-06	0.006512886
G	A	-0.1608	0.035	0.8553	3656	3.93E-06	0.005812661
G	A	0.1963	0.026	0.3256	3656	5.62E-14	0.015340509
T	C	0.4428	0.087	0.02	3656	4.05E-07	0.006997263
C	T	-0.1136	0.024	0.5737	3656	3.57E-06	0.0058652
C	G	0.291	0.057	0.045	3656	3.22E-07	0.007115698
A	G	-0.1118	0.025	0.4579	3656	5.80E-06	0.005608654
A	G	-0.1821	0.04	0.1034	3656	4.68E-06	0.005719039
T	G	-0.125	0.024	0.592	3656	2.83E-07	0.007191579
A	G	-0.2566	0.058	0.0435	3656	8.81E-06	0.005389661
G	T	-0.4122	0.085	0.9792	3656	1.47E-06	0.006323088
C	T	1.055	0.225	0.003	3657	2.81E-06	0.005986591
G	A	0.1136	0.025	0.4754	3657	5.25E-06	0.005659371
A	G	-2.752	0.567	0.0004	3657	1.29E-06	0.006391584
A	G	-2.77	0.567	0.0004	3657	1.09E-06	0.006479463
C	T	-0.6384	0.136	0.0085	3657	2.76E-06	0.005989272
G	A	0.3561	0.075	0.0286	3657	2.26E-06	0.006099201
A	G	-1.841	0.389	0.0016	3657	2.25E-06	0.00610298
G	A	0.2678	0.055	0.0555	3657	1.27E-06	0.006394872
T	C	-0.1605	0.036	0.1396	3657	9.04E-06	0.005373167
G	A	-0.3971	0.086	0.0219	3657	3.76E-06	0.005831334
C	T	0.4712	0.101	0.0152	3657	2.97E-06	0.005951606
A	G	0.1908	0.036	0.1349	3657	1.43E-07	0.007543383
C	A	0.466	0.105	0.0148	3657	8.84E-06	0.005387748
T	C	0.146	0.032	0.182	3657	5.38E-06	0.005645947
T	C	0.1597	0.035	0.1593	3657	5.36E-06	0.005644823
A	T	-0.2029	0.044	0.0919	3657	4.27E-06	0.005765526
C	T	-0.3098	0.063	0.959	3657	8.16E-07	0.006627321
A	G	-0.2776	0.062	0.0421	3657	7.96E-06	0.005439783

G	C	0.2344	0.051	0.0643	3656	4.20E-06	0.005773919
C	T	1.052	0.226	0.003	3656	3.35E-06	0.00588654
A	G	0.2343	0.051	0.0647	3656	5.51E-06	0.005631705
C	T	0.1425	0.032	0.1956	3656	8.85E-06	0.005391435
T	G	0.2069	0.046	0.0792	3656	6.77E-06	0.005529307
A	G	0.1246	0.027	0.6779	3656	3.53E-06	0.005868891
G	A	0.3458	0.076	0.0286	3656	5.62E-06	0.005623365
C	T	-0.1924	0.043	0.0909	3656	8.66E-06	0.005396182
A	G	0.6821	0.149	0.0066	3656	4.74E-06	0.005714721
A	G	-0.9851	0.214	0.0033	3656	4.47E-06	0.005741222
C	T	0.4601	0.102	0.0152	3656	6.28E-06	0.005567136
G	A	0.2658	0.06	0.0464	3656	9.47E-06	0.005349838
G	A	-0.1482	0.03	0.2157	3656	1.07E-06	0.006488114
G	A	0.3337	0.072	0.031	3656	4.14E-06	0.005783491
A	G	0.1331	0.027	0.6422	3656	5.69E-07	0.006822143
A	C	-0.2921	0.064	0.9583	3656	4.54E-06	0.00573467
A	T	-0.2323	0.051	0.0643	3656	5.75E-06	0.005609934
C	T	-0.146	0.03	0.7674	3656	8.52E-07	0.006610521
T	C	-0.1946	0.04	0.1095	3657	1.08E-06	0.006485067
T	C	0.1263	0.026	0.5153	3657	1.11E-06	0.006470443
T	C	0.483	0.088	0.0201	3657	3.86E-08	0.008229612
G	A	-0.2927	0.063	0.0421	3657	3.85E-06	0.005820055
C	T	-0.6051	0.136	0.009	3657	8.84E-06	0.005384024
A	G	-0.1728	0.038	0.1326	3657	4.84E-06	0.005703022
A	G	-0.1408	0.031	0.7849	3657	6.89E-06	0.005520471
G	A	0.2735	0.06	0.0447	3657	5.36E-06	0.005644102
A	G	0.1159	0.026	0.4199	3657	5.71E-06	0.005617139
C	G	0.3029	0.066	0.0387	3657	4.43E-06	0.005745557
T	C	-0.1439	0.032	0.816	3657	8.69E-06	0.005394776
G	C	-0.142	0.03	0.7737	3657	2.97E-06	0.005954248
T	C	0.4404	0.095	0.0179	3657	3.80E-06	0.005826355
T	C	1.016	0.227	0.003	3657	7.68E-06	0.005452791
A	G	-0.2773	0.051	0.0662	3657	7.30E-08	0.007895975
T	C	1.363	0.3	0.0018	3657	5.85E-06	0.005601657
C	T	-0.1632	0.035	0.1471	3657	4.14E-06	0.005781441
G	A	-0.1247	0.026	0.6266	3657	2.37E-06	0.006068559
T	C	0.2723	0.06	0.044	3657	6.74E-06	0.00552518
T	C	-0.3021	0.067	0.0343	3657	7.53E-06	0.005468421
C	A	-0.2989	0.061	0.0406	3656	1.21E-06	0.006425687
C	T	-0.2103	0.046	0.081	3656	3.99E-06	0.005801631
A	G	1.503	0.33	0.0012	3656	5.31E-06	0.005652131
T	C	2.619	0.577	0.0004	3656	5.73E-06	0.005613342
A	G	2.118	0.45	0.0007	3656	2.63E-06	0.006020122
C	T	0.138	0.027	0.2964	3656	4.42E-07	0.00694573
C	T	-0.4464	0.099	0.0157	3656	6.51E-06	0.00554719
G	T	0.3363	0.075	0.028	3656	8.65E-06	0.005400476
T	A	-0.2677	0.057	0.048	3656	2.94E-06	0.005963599
G	T	-0.1335	0.028	0.2657	3656	1.26E-06	0.006404725

T	C	-0.2005	0.044	0.084	3656	5.03E-06	0.005680847
A	G	0.4148	0.081	0.022	3656	3.64E-07	0.007054313
G	A	0.1269	0.028	0.7124	3656	5.63E-06	0.005622747
T	C	0.7675	0.17	0.0055	3656	6.72E-06	0.005531241
A	G	0.132	0.028	0.2619	3656	2.39E-06	0.006068
T	C	-0.3693	0.08	0.023	3656	4.44E-06	0.005747692
A	G	0.3166	0.07	0.0298	3656	5.74E-06	0.00561185
A	G	0.4915	0.109	0.0126	3656	6.25E-06	0.005571278
T	G	-0.1264	0.027	0.3032	3656	2.60E-06	0.006025245
C	G	1.816	0.404	0.001	3656	7.18E-06	0.005496284
C	T	-0.1616	0.035	0.1469	3656	3.10E-06	0.005937998
A	G	-0.3049	0.067	0.0325	3656	5.23E-06	0.005662776
T	A	-0.1593	0.029	0.4148	3656	2.61E-08	0.008437794
C	T	-0.2158	0.048	0.0699	3656	6.70E-06	0.005530226
A	G	-0.56	0.123	0.0097	3657	5.91E-06	0.005599921
T	C	-0.1301	0.025	0.5153	3657	3.41E-07	0.007084092
G	A	-0.2365	0.051	0.0641	3657	3.67E-06	0.005843612
C	T	-0.1269	0.026	0.3805	3657	1.01E-06	0.00651165
T	C	-0.4865	0.087	0.0201	3657	2.05E-08	0.008563888
G	A	0.4657	0.105	0.0138	3657	9.92E-06	0.005330096
G	A	0.3527	0.062	0.0421	3657	1.71E-08	0.008660425
A	G	0.2066	0.043	0.0913	3657	1.83E-06	0.006209562
T	C	-0.737	0.164	0.006	3657	7.48E-06	0.005472073
A	G	-0.2083	0.043	0.0906	3657	1.15E-06	0.00644717
A	G	-0.1708	0.038	0.1181	3657	6.71E-06	0.005528701
G	A	-0.293	0.059	0.0447	3657	8.18E-07	0.006629271
G	A	0.9325	0.202	0.0037	3657	3.93E-06	0.005810721
C	T	0.9614	0.207	0.9966	3657	3.52E-06	0.005863925
C	T	0.2715	0.061	0.9556	3657	9.28E-06	0.005361509
C	G	0.9264	0.207	0.0034	3657	7.60E-06	0.005462761
C	G	0.3676	0.081	0.022	3657	6.15E-06	0.005574338
T	C	-0.1852	0.042	0.0941	3657	9.38E-06	0.005351968
A	G	-0.1205	0.025	0.4035	3657	2.21E-06	0.006111915
T	C	0.1143	0.026	0.3411	3657	9.30E-06	0.005358946
A	C	1.249	0.278	0.0021	3657	7.44E-06	0.005473659
G	A	0.4892	0.088	0.0206	3657	3.03E-08	0.008358956
G	A	0.4553	0.102	0.0146	3657	8.56E-06	0.005397815
C	G	0.2675	0.058	0.045	3657	4.60E-06	0.005727813
A	G	0.2482	0.051	0.0662	3657	1.06E-06	0.006490308
A	G	-0.1563	0.034	0.1574	3657	4.30E-06	0.005762403
T	C	-0.4055	0.086	0.0201	3656	2.57E-06	0.00603174
A	G	0.5129	0.115	0.0108	3656	8.98E-06	0.00537412
A	G	0.1752	0.039	0.1184	3656	8.47E-06	0.005406578
A	C	-0.1111	0.025	0.4714	3656	7.48E-06	0.005476909
T	C	-0.4318	0.096	0.0167	3656	6.67E-06	0.005533025
A	C	2.539	0.52	0.0005	3656	1.09E-06	0.006476252
G	A	0.1188	0.027	0.3155	3656	8.93E-06	0.00537789
C	T	0.1344	0.03	0.2211	3656	6.18E-06	0.005573712

T	C	0.312	0.067	0.0349	3656	3.23E-06	0.005910397
A	T	-0.1404	0.027	0.3005	3656	2.84E-07	0.00718767
T	C	0.6162	0.136	0.0086	3656	5.75E-06	0.005608348
T	C	1.162	0.261	0.0021	3656	8.56E-06	0.005400564
G	T	-0.2901	0.063	0.0414	3656	4.98E-06	0.005685254
C	T	0.1133	0.026	0.6198	3656	9.51E-06	0.005345694
T	C	0.1488	0.03	0.772	3656	6.09E-07	0.006782575
A	G	-0.4374	0.094	0.0174	3656	3.11E-06	0.005931331
G	A	1.818	0.388	0.001	3656	2.87E-06	0.005975347
G	A	0.1691	0.027	0.3256	3656	2.04E-10	0.010990238
T	C	0.4411	0.089	0.02	3656	7.38E-07	0.006684341
T	G	0.283	0.062	0.042	3656	5.54E-06	0.00563213
C	G	0.2815	0.058	0.045	3656	1.13E-06	0.006459259
T	G	-0.118	0.025	0.592	3656	1.84E-06	0.006203849
A	G	0.1546	0.035	0.8487	3656	8.75E-06	0.00539392
A	G	-0.2606	0.059	0.0435	3656	8.64E-06	0.005398585
G	A	-0.1712	0.038	0.121	3656	7.09E-06	0.005498105
A	G	0.5664	0.12	0.0103	3656	2.60E-06	0.006026758
A	G	-0.4047	0.083	0.0254	3658	1.01E-06	0.006516479
A	G	-0.9447	0.193	0.004	3658	1.08E-06	0.006480486
T	C	0.3316	0.073	0.0299	3658	6.20E-06	0.005569624
A	G	0.1401	0.029	0.2246	3658	2.08E-06	0.006140409
T	C	0.3083	0.068	0.0316	3658	5.11E-06	0.005667222
C	G	0.2235	0.05	0.0645	3658	6.61E-06	0.005537814
C	G	0.2993	0.065	0.0372	3658	3.97E-06	0.005799994
T	C	0.1364	0.029	0.6083	3658	2.10E-06	0.006132635
C	T	-0.1917	0.043	0.9097	3658	7.46E-06	0.005472071
T	G	0.2453	0.055	0.0524	3658	8.60E-06	0.005396707
T	C	0.2118	0.047	0.073	3658	6.42E-06	0.005551376
C	T	0.1137	0.025	0.3779	3658	5.83E-06	0.005596003
G	A	-0.2663	0.054	0.0547	3658	1.03E-06	0.006508263
C	A	0.2407	0.053	0.9422	3658	5.84E-06	0.005600491
G	T	-0.1233	0.026	0.3414	3658	2.67E-06	0.006008963
A	C	-1.694	0.353	0.0011	3658	1.64E-06	0.006263215
G	A	-0.1372	0.031	0.7989	3658	7.06E-06	0.005497761
A	C	0.2021	0.044	0.0853	3658	4.18E-06	0.005778685
G	C	-0.2182	0.046	0.0778	3658	2.36E-06	0.006071407
C	A	0.4562	0.1	0.015	3658	5.65E-06	0.005612484
T	C	-0.6197	0.124	0.0081	3656	6.33E-07	0.006763419
A	G	-0.108	0.024	0.5671	3656	9.60E-06	0.005343226
A	G	-1.339	0.283	0.0018	3656	2.33E-06	0.006077445
C	G	0.5637	0.126	0.0086	3656	7.82E-06	0.005453353
T	A	2.541	0.529	0.0008	3656	1.64E-06	0.00626662
A	G	-0.1466	0.03	0.1991	3656	7.43E-07	0.006678042
A	G	0.3828	0.085	0.02	3656	6.99E-06	0.005509189
C	T	0.3438	0.077	0.0227	3656	8.48E-06	0.005410697
T	A	0.3462	0.078	0.0239	3656	8.27E-06	0.00542294
A	G	-0.1707	0.038	0.1076	3656	8.03E-06	0.00543494

T	C	-0.6658	0.145	0.0068	3656	4.47E-06	0.005741744
T	G	0.2526	0.054	0.0511	3656	2.76E-06	0.005993564
C	G	-2.494	0.526	0.9996	3656	2.16E-06	0.006123135
A	G	0.1457	0.032	0.1637	3656	5.16E-06	0.005670088
C	G	0.1973	0.044	0.0799	3656	6.69E-06	0.005534522
C	A	0.1689	0.034	0.1332	3656	8.86E-07	0.006584798
A	G	-0.5657	0.122	0.0094	3656	3.92E-06	0.005808625
G	A	-0.1355	0.03	0.1916	3656	7.11E-06	0.005497834
A	G	-0.3311	0.072	0.0264	3656	4.91E-06	0.00569423
A	G	-0.1142	0.025	0.6518	3656	4.15E-06	0.005780379
A	G	0.1928	0.041	0.093	3656	2.87E-06	0.005974314
C	G	0.3465	0.078	0.0248	3656	8.31E-06	0.00542116
T	G	0.3592	0.079	0.0246	3657	6.32E-06	0.005561049
C	G	-0.1546	0.029	0.2252	3657	1.48E-07	0.007519829
T	C	-0.1694	0.038	0.1154	3657	8.03E-06	0.005436074
T	A	0.1193	0.026	0.6555	3657	4.11E-06	0.005781558
C	G	0.2348	0.049	0.0645	3657	2.03E-06	0.006152008
A	G	-0.2732	0.059	0.0446	3657	3.24E-06	0.005906365
T	A	1.728	0.382	0.999	3657	6.26E-06	0.005567223
G	A	1.508	0.281	0.0018	3657	8.31E-08	0.007824776
T	C	0.177	0.035	0.5975	3657	3.17E-07	0.007125573
A	G	0.1274	0.029	0.7447	3657	9.78E-06	0.005333492
T	G	0.249	0.055	0.0524	3657	5.77E-06	0.005609866
T	C	-0.1541	0.033	0.1693	3657	3.21E-06	0.00590967
C	T	-0.1182	0.026	0.6508	3657	4.35E-06	0.005759851
G	A	-0.1128	0.025	0.3646	3657	9.72E-06	0.005338897
T	C	0.1881	0.04	0.102	3657	3.50E-06	0.005866805
G	T	-0.1412	0.031	0.8048	3657	6.08E-06	0.005580083
A	G	-0.1127	0.025	0.3704	3657	6.51E-06	0.00554837
C	G	-0.1249	0.027	0.2863	3657	3.52E-06	0.005860608
C	G	-0.1932	0.042	0.0946	3657	3.49E-06	0.005868998
A	G	0.1091	0.024	0.5643	3657	6.61E-06	0.005531512
G	C	-0.1465	0.032	0.1719	3657	5.92E-06	0.005590385
C	T	-0.1164	0.025	0.6221	3657	3.75E-06	0.005832511
C	T	0.3071	0.066	0.0358	3657	3.23E-06	0.005912183
G	C	-0.1123	0.025	0.4371	3657	5.17E-06	0.00566626
C	G	-0.1147	0.026	0.3131	3657	9.67E-06	0.005338425
G	T	-0.2476	0.052	0.0585	3659	1.93E-06	0.006177013
A	G	0.127	0.028	0.236	3659	6.54E-06	0.005535871
G	T	-0.4902	0.11	0.0127	3659	8.62E-06	0.005398189
A	G	0.1157	0.026	0.6682	3659	6.00E-06	0.005586119
T	C	0.6255	0.131	0.0089	3659	2.01E-06	0.006154889
A	G	0.524	0.112	0.0118	3659	3.07E-06	0.005936128
T	C	-0.1398	0.032	0.1735	3659	9.86E-06	0.005323955
A	G	-0.1471	0.029	0.7803	3659	3.12E-07	0.007128403
G	A	0.2113	0.048	0.0696	3659	9.12E-06	0.005365576
T	C	0.2125	0.042	0.6663	3659	4.19E-07	0.006973866
T	C	0.1557	0.034	0.1469	3659	5.72E-06	0.005606495

C	G	0.1351	0.03	0.2044	3659	6.77E-06	0.005519267
A	T	0.2799	0.061	0.0402	3659	5.45E-06	0.005632776
T	C	-1.505	0.339	0.0012	3659	9.20E-06	0.005360842
C	G	-0.1463	0.031	0.1838	3659	2.90E-06	0.005965706
T	G	0.1355	0.028	0.2346	3659	1.46E-06	0.006319164
A	G	-1.069	0.221	0.0033	3659	1.42E-06	0.006336791
T	C	-1.342	0.29	0.0016	3659	3.73E-06	0.005830504
C	T	-0.8348	0.149	0.0066	3659	2.48E-08	0.008460795
C	T	-0.2409	0.052	0.0559	3659	3.09E-06	0.005930647
C	A	-0.2551	0.057	0.05	3659	8.79E-06	0.005383957
G	A	0.1278	0.029	0.2288	3659	9.10E-06	0.005371364
C	T	0.1332	0.03	0.2062	3659	8.60E-06	0.005394541
T	G	0.293	0.062	0.0377	3659	2.57E-06	0.006025977
A	G	0.1488	0.033	0.1596	3659	8.08E-06	0.005430639
A	G	-0.1186	0.025	0.5914	3659	1.55E-06	0.006297015
T	C	0.6026	0.132	0.0089	3658	5.38E-06	0.005639476
T	C	-0.1537	0.032	0.1736	3658	1.34E-06	0.006365656
A	G	-0.1438	0.029	0.7802	3658	6.60E-07	0.006736664
T	C	0.2112	0.042	0.6662	3658	4.25E-07	0.006966971
A	T	0.1893	0.041	0.903	3658	3.29E-06	0.005899216
T	C	-0.1548	0.033	0.1643	3658	2.27E-06	0.006092762
C	G	0.139	0.03	0.2045	3658	4.09E-06	0.00578835
A	T	0.2899	0.062	0.0402	3658	2.87E-06	0.005971899
A	G	-0.3072	0.066	0.0349	3658	3.45E-06	0.005873545
C	T	-0.1305	0.028	0.7571	3658	4.63E-06	0.005723023
G	A	0.1435	0.028	0.2347	3658	3.99E-07	0.007004393
C	T	-0.6917	0.15	0.0066	3658	4.40E-06	0.005748999
T	G	0.3095	0.062	0.0377	3658	7.67E-07	0.006661223
A	C	-0.2548	0.057	0.0484	3658	6.74E-06	0.005527102
C	T	-0.2131	0.048	0.0794	3658	8.02E-06	0.005435607
A	G	-0.1099	0.025	0.5914	3658	9.60E-06	0.005344062
A	G	0.1498	0.033	0.1597	3658	7.70E-06	0.005455963
C	T	2.746	0.589	0.0004	3657	3.27E-06	0.005904441
G	T	-0.2827	0.057	0.0494	3657	7.43E-07	0.006676715
T	G	0.614	0.136	0.0081	3657	6.97E-06	0.005510402
C	A	-0.3021	0.066	0.0357	3657	4.76E-06	0.005706806
C	G	-0.2101	0.042	0.1131	3657	6.78E-07	0.006722881
A	T	-0.1651	0.036	0.1318	3657	4.85E-06	0.005699482
A	G	0.2879	0.063	0.0377	3657	5.20E-06	0.005662019
T	A	-0.2739	0.056	0.0481	3657	1.25E-06	0.006403289
G	T	-0.1285	0.027	0.2657	3657	2.24E-06	0.006101603
C	G	0.2839	0.064	0.0387	3657	8.16E-06	0.005427641
A	G	0.1101	0.024	0.5273	3657	3.11E-06	0.00593126
C	T	0.3546	0.079	0.0249	3657	6.84E-06	0.005520759
G	A	-1.032	0.218	0.0037	3657	2.22E-06	0.006101837
G	A	0.1898	0.026	0.3257	3657	3.43E-13	0.014373654
C	G	0.1596	0.034	0.1523	3657	2.10E-06	0.006135472
T	C	-0.1468	0.024	0.4882	3657	5.52E-10	0.010469652

T	A	-0.142	0.027	0.4267	3657	2.45E-07	0.007253917
G	A	-0.2576	0.058	0.0469	3657	8.75E-06	0.005390913
G	A	0.1165	0.025	0.6218	3657	3.24E-06	0.005907736
G	A	-0.1212	0.026	0.6172	3657	2.47E-06	0.006054147
A	G	-0.1191	0.025	0.6517	3656	1.65E-06	0.006258743
C	T	0.192	0.041	0.0929	3656	2.60E-06	0.006023721
T	A	2.567	0.538	0.0008	3656	1.93E-06	0.006179359
A	T	0.1297	0.029	0.2133	3656	6.13E-06	0.005574383
A	G	-1.324	0.281	0.0018	3656	2.50E-06	0.006044253
A	G	-0.1404	0.029	0.1991	3656	1.98E-06	0.006165766
T	C	1.619	0.361	0.0011	3656	7.48E-06	0.005471299
A	G	0.3801	0.082	0.0197	3656	4.16E-06	0.005780903
A	G	0.2925	0.062	0.0379	3656	2.32E-06	0.006084105
A	C	0.3028	0.068	0.0294	3656	9.29E-06	0.005361358
A	T	-0.6902	0.155	0.0059	3656	8.89E-06	0.005387337
A	C	1.582	0.356	0.001	3656	8.96E-06	0.005381404
C	T	0.1238	0.027	0.7503	3656	5.92E-06	0.005593373
T	C	-0.4998	0.112	0.0129	3656	8.62E-06	0.005398211
C	A	-2.09	0.45	0.0007	3656	3.53E-06	0.00586552
A	G	-1.987	0.403	0.0008	3656	8.32E-07	0.006618477
T	G	0.3296	0.07	0.0294	3656	2.48E-06	0.006051665
T	C	-0.6459	0.145	0.0068	3656	8.26E-06	0.005420345
C	T	-0.311	0.064	0.0341	3656	1.47E-06	0.006320858
G	C	0.2558	0.054	0.0518	3656	1.91E-06	0.00618881
A	T	0.1975	0.044	0.0766	3656	8.62E-06	0.005402266
C	A	0.1561	0.034	0.1332	3656	5.11E-06	0.005669337
G	A	-0.1069	0.024	0.4229	3656	6.51E-06	0.005548001
T	C	0.1168	0.024	0.5412	3656	1.09E-06	0.006484774
G	A	-0.1438	0.03	0.1916	3656	1.65E-06	0.006261811
A	G	-0.3233	0.072	0.0264	3656	7.68E-06	0.005460522
T	C	0.2941	0.061	0.0382	3656	1.64E-06	0.006264705
A	G	-0.1167	0.026	0.2986	3656	7.15E-06	0.005501296
T	C	-0.1289	0.025	0.635	3656	1.92E-07	0.007388115
C	G	0.3562	0.077	0.0248	3656	4.12E-06	0.005784822
T	G	0.3941	0.082	0.0207	3656	1.75E-06	0.006232921
T	C	-0.1164	0.026	0.7061	3656	9.55E-06	0.005349481
T	A	0.556	0.124	0.0105	3656	7.80E-06	0.005451627
T	C	1.034	0.225	0.0027	3656	4.55E-06	0.005738318
C	T	0.516	0.104	0.0164	3656	8.16E-07	0.006637431
G	A	0.1644	0.03	0.2069	3656	4.86E-08	0.008109504
C	T	0.1571	0.03	0.1893	3656	2.51E-07	0.007246939
G	A	0.5092	0.109	0.0126	3656	2.94E-06	0.005955506
A	C	0.1691	0.038	0.1104	3656	9.93E-06	0.005325747
G	A	2.925	0.649	0.0005	3656	6.81E-06	0.005523532
T	A	-0.1275	0.027	0.2842	3656	2.28E-06	0.006089286
T	C	1.843	0.384	0.0008	3656	1.66E-06	0.006257913
T	G	-0.1185	0.025	0.3885	3656	1.74E-06	0.006231111
T	G	0.2746	0.06	0.0414	3656	4.78E-06	0.005705994

G	A	-0.1408	0.024	0.5715	3656	8.25E-09	0.0090404
G	A	0.1383	0.031	0.1882	3656	6.89E-06	0.005516658
C	G	0.4384	0.093	0.0172	3656	2.24E-06	0.006101239
C	T	-0.2055	0.041	0.1013	3656	6.51E-07	0.006752413
T	C	-0.1218	0.027	0.7001	3656	6.31E-06	0.005559962
G	A	0.1281	0.024	0.5044	3656	1.05E-07	0.007706608
G	A	0.38	0.083	0.0213	3656	4.61E-06	0.005726658
T	G	-0.1257	0.024	0.592	3656	1.93E-07	0.007379943
T	C	0.4981	0.11	0.0116	3656	6.64E-06	0.005537036
C	T	-0.1194	0.027	0.2946	3656	7.05E-06	0.005509707
T	C	0.1091	0.024	0.5678	3656	6.04E-06	0.005588014
T	C	0.3766	0.083	0.0215	3656	5.86E-06	0.005600975
A	C	0.1517	0.029	0.23	3656	1.84E-07	0.007408703
A	G	-0.2855	0.065	0.0387	3656	9.95E-06	0.00532554
C	T	0.1428	0.031	0.2113	3656	3.17E-06	0.00592145
G	A	0.1358	0.03	0.2082	3656	7.37E-06	0.00548581
T	C	-0.1296	0.029	0.2408	3656	6.94E-06	0.005512137
A	G	-0.1193	0.027	0.677	3656	7.22E-06	0.005488182
G	A	0.1403	0.027	0.2924	3656	2.84E-07	0.007177508
A	G	-0.2723	0.057	0.0509	3656	1.87E-06	0.006197016
C	T	0.2178	0.048	0.0673	3656	6.07E-06	0.005583796
C	G	-0.853	0.189	0.0045	3656	6.27E-06	0.005563977
T	C	-1.231	0.269	0.0021	3656	4.86E-06	0.00569962
T	C	0.1731	0.038	0.8835	3656	6.41E-06	0.005556108
A	G	0.1211	0.027	0.7103	3656	8.37E-06	0.005416313
C	G	-0.165	0.037	0.1325	3656	7.97E-06	0.005445135
T	A	-0.1322	0.028	0.2842	3656	1.89E-06	0.006191557
T	C	0.617	0.137	0.0086	3656	6.50E-06	0.005549399
C	T	-0.1246	0.025	0.5715	3656	6.83E-07	0.006721796
C	T	0.391	0.077	0.0272	3656	3.59E-07	0.007059809
C	T	0.3985	0.088	0.0208	3656	6.00E-06	0.005587809
T	C	0.204	0.044	0.0907	3656	4.40E-06	0.005751301
C	T	-0.1119	0.025	0.5685	3656	8.39E-06	0.005415525
A	G	-0.3825	0.086	0.0207	3656	9.03E-06	0.005376684
G	C	-0.1952	0.043	0.0989	3656	4.95E-06	0.005688882
C	G	0.1207	0.025	0.4402	3656	1.44E-06	0.006335315
G	A	0.1408	0.027	0.3256	3656	1.38E-07	0.007560172
A	G	0.1116	0.025	0.5167	3656	5.63E-06	0.005615904
T	G	-0.1169	0.025	0.592	3656	2.78E-06	0.005992579
C	T	-0.1195	0.027	0.3155	3656	9.61E-06	0.005345179
C	T	0.1163	0.025	0.5922	3656	3.33E-06	0.005898572
G	C	-0.5436	0.112	0.0126	3656	1.24E-06	0.006413546
T	G	0.1953	0.042	0.0956	3657	3.85E-06	0.005819878
C	T	-2.636	0.594	0.0004	3657	9.31E-06	0.005361631
T	C	-0.7345	0.164	0.0055	3657	7.23E-06	0.005488239
C	G	-0.5857	0.114	0.0109	3657	2.81E-07	0.007191286
A	G	0.1484	0.031	0.1987	3657	2.49E-06	0.00604389
G	A	-1.341	0.296	0.0019	3657	6.06E-06	0.005581084

C	T	0.3323	0.071	0.0334	3657	2.82E-06	0.005982672
A	G	-0.2872	0.062	0.0388	3657	4.41E-06	0.005750089
G	A	3.03	0.67	0.0005	3657	6.22E-06	0.005569721
T	C	-0.1389	0.029	0.2374	3657	1.51E-06	0.006302938
T	C	-0.3593	0.08	0.0247	3657	6.50E-06	0.005547447
A	G	0.6398	0.125	0.0093	3657	3.46E-07	0.007079067
T	C	-0.1154	0.026	0.5576	3657	9.41E-06	0.005353953
T	C	-0.4565	0.102	0.0148	3657	8.36E-06	0.005415605
T	C	-0.136	0.028	0.248	3657	1.41E-06	0.006342098
G	T	-0.246	0.055	0.0524	3657	8.23E-06	0.005422982
A	G	-0.3698	0.072	0.029	3657	3.31E-07	0.007100963
A	G	-0.1167	0.025	0.4035	3657	4.11E-06	0.005788922
C	A	0.321	0.073	0.0305	3657	9.93E-06	0.005326117
C	A	-0.1293	0.029	0.7518	3657	8.82E-06	0.005391765
A	G	0.1177	0.025	0.359	3657	3.36E-06	0.005887971
G	C	0.651	0.145	0.0078	3657	7.42E-06	0.00547417
G	A	-0.1701	0.037	0.1288	3657	3.48E-06	0.005871701
T	C	0.2621	0.056	0.0515	3657	2.74E-06	0.005994786
C	A	-0.2358	0.053	0.0593	3657	8.89E-06	0.005385544
C	G	0.887	0.196	0.0038	3657	6.22E-06	0.005569097
G	A	0.1734	0.038	0.108	3656	5.73E-06	0.005613104
C	T	-0.1278	0.026	0.3243	3656	9.11E-07	0.006570223
C	T	-0.1658	0.037	0.1232	3656	9.60E-06	0.005343925
C	T	-0.3597	0.064	0.0368	3656	2.07E-08	0.008560704
G	A	0.3352	0.074	0.9739	3656	6.67E-06	0.005533255
A	G	-0.4243	0.087	0.0194	3656	1.26E-06	0.006403651
C	T	-0.283	0.063	0.0361	3656	8.56E-06	0.005405082
A	G	1.792	0.366	0.001	3656	1.01E-06	0.006517861
A	G	-0.2327	0.045	0.0814	3656	2.47E-07	0.007257801
A	G	-0.1368	0.029	0.2299	3656	2.03E-06	0.006158989
A	G	-0.1653	0.036	0.1722	3656	6.07E-06	0.005581629
A	T	-0.4644	0.097	0.0159	3656	1.59E-06	0.006283137
A	G	-0.2347	0.05	0.9375	3656	2.96E-06	0.005957394
C	A	0.126	0.027	0.7321	3656	2.99E-06	0.005956487
A	C	-0.1213	0.025	0.5309	3656	7.76E-07	0.006654708
T	A	0.1232	0.028	0.2789	3656	7.75E-06	0.005451854
T	C	-0.116	0.026	0.6458	3656	5.66E-06	0.005615167
A	G	0.1495	0.032	0.1722	3656	3.74E-06	0.005836281
T	C	0.3875	0.079	0.0231	3656	1.09E-06	0.006477422
A	C	-0.4624	0.097	0.0161	3656	1.89E-06	0.006192464
G	C	0.1102	0.024	0.4739	3656	6.49E-06	0.005543794
G	A	-0.4731	0.106	0.0129	3656	7.93E-06	0.005439507
T	C	-0.5382	0.12	0.0107	3656	7.07E-06	0.005499181
A	T	0.1771	0.038	0.1157	3656	2.50E-06	0.006044311
C	T	-0.1525	0.031	0.8141	3656	8.88E-07	0.006588405
A	G	0.1158	0.024	0.5022	3656	1.86E-06	0.006198507
G	A	-0.3886	0.087	0.0185	3656	8.59E-06	0.005402735
C	T	2.959	0.584	0.0004	3656	4.28E-07	0.006965882

A	G	0.1449	0.033	0.1607	3656	9.69E-06	0.005342061
T	C	0.6348	0.143	0.007	3656	8.94E-06	0.005383631
A	T	2.439	0.55	0.0004	3656	9.59E-06	0.005346237
T	C	-0.1262	0.028	0.2486	3656	5.62E-06	0.005625183
T	C	0.1411	0.031	0.1982	3656	4.74E-06	0.005707691
A	C	0.1272	0.028	0.2433	3656	7.89E-06	0.005449376
G	T	0.1165	0.025	0.4702	3656	2.42E-06	0.006062704
A	T	-0.2112	0.046	0.0788	3656	5.32E-06	0.005651913
A	T	-0.2012	0.044	0.0826	3656	5.29E-06	0.005653529
C	T	0.1502	0.032	0.1737	3656	3.75E-06	0.005833095
C	G	-0.1912	0.043	0.1131	3656	7.93E-06	0.005441613
C	G	0.1106	0.025	0.4346	3656	7.12E-06	0.005498438
T	A	-0.2544	0.057	0.048	3656	7.26E-06	0.005489644
T	A	0.2018	0.042	0.0927	3656	1.42E-06	0.006337679
C	T	-0.3385	0.075	0.0265	3656	5.97E-06	0.005592627
C	T	-0.1537	0.03	0.1978	3656	3.96E-07	0.007007285
C	G	0.3011	0.064	0.0387	3656	2.32E-06	0.006083707
A	G	-0.4228	0.089	0.0204	3656	1.91E-06	0.006185964
C	A	0.2233	0.049	0.0639	3656	4.30E-06	0.005764703
A	C	0.348	0.071	0.0302	3656	1.09E-06	0.006479116
C	T	0.3651	0.079	0.0249	3656	3.91E-06	0.005813944
G	A	1.581	0.351	0.0011	3656	6.69E-06	0.00553127
A	G	0.503	0.108	0.0126	3656	3.13E-06	0.005930823
G	A	0.1366	0.026	0.3256	3656	1.90E-07	0.007397131
A	G	-0.2347	0.05	0.0662	3656	2.40E-06	0.006065127
A	C	0.1233	0.024	0.4851	3656	4.78E-07	0.00690799
A	G	0.1214	0.024	0.5022	3656	6.26E-07	0.006763935
T	C	-0.1167	0.024	0.4884	3656	8.84E-07	0.006588221
A	G	0.1953	0.044	0.0793	3656	9.57E-06	0.005345417
A	G	0.1258	0.024	0.4571	3656	2.19E-07	0.007319104
G	A	-0.1184	0.026	0.6172	3656	4.95E-06	0.005692322
G	A	-0.4222	0.087	0.0185	3656	1.40E-06	0.006348035
A	C	-0.4492	0.1	0.015	3656	7.21E-06	0.00549324
T	C	0.2463	0.052	0.0577	3656	2.72E-06	0.005999973
G	C	-0.3548	0.078	0.0263	3656	5.08E-06	0.00567522
A	C	-0.1352	0.028	0.7471	3656	2.16E-06	0.006122029
C	T	-0.199	0.043	0.0946	3656	3.30E-06	0.00590021
G	T	-0.5085	0.108	0.0137	3656	2.50E-06	0.006049271
A	T	-0.2682	0.058	0.0495	3656	3.36E-06	0.005891133
C	T	-0.1427	0.031	0.2014	3656	4.75E-06	0.00570742
T	C	0.6141	0.136	0.0086	3656	6.90E-06	0.005513687
C	T	0.1272	0.028	0.7213	3656	5.05E-06	0.005677496
C	T	0.1892	0.041	0.0964	3656	4.66E-06	0.005718564
G	A	-0.2515	0.052	0.0618	3656	1.34E-06	0.006372203
C	T	-0.1789	0.037	0.1288	3656	1.66E-06	0.006256107
G	A	0.1714	0.027	0.3256	3656	1.39E-10	0.011204171
G	A	3.09	0.683	0.0003	3656	6.25E-06	0.005567302
T	C	-0.1589	0.036	0.1413	3656	8.77E-06	0.00538962

C	G	0.3532	0.058	0.045	3656	1.25E-09	0.010041445
A	G	0.448	0.094	0.0179	3656	1.98E-06	0.006166709
T	C	-0.9621	0.216	0.0036	3656	8.36E-06	0.005417235
A	G	-0.4323	0.092	0.0181	3656	2.89E-06	0.005970775
G	C	-0.1135	0.024	0.4918	3656	2.77E-06	0.005990506
A	G	0.4662	0.094	0.0175	3656	7.14E-07	0.006702803
T	C	-0.3003	0.066	0.0379	3656	4.81E-06	0.005706126
T	C	-0.3961	0.085	0.0201	3656	3.52E-06	0.005867529
G	A	0.1461	0.032	0.1761	3656	6.30E-06	0.005565006
T	C	-0.1494	0.032	0.1838	3656	3.62E-06	0.005850129
T	C	0.5993	0.134	0.0086	3656	8.30E-06	0.005417156
C	T	-0.3254	0.067	0.9661	3656	1.47E-06	0.006323843
G	A	-0.3185	0.068	0.0319	3656	3.35E-06	0.005892234
G	A	-0.1601	0.035	0.1435	3656	5.68E-06	0.005617006
G	T	0.1189	0.024	0.4521	3656	1.12E-06	0.006468863
A	G	0.3622	0.082	0.9784	3656	9.91E-06	0.005327622
C	T	-0.1182	0.026	0.3087	3656	7.88E-06	0.005444926
A	C	-0.1638	0.037	0.1296	3656	9.00E-06	0.005378242
C	G	0.501	0.107	0.013	3656	2.96E-06	0.005960809
G	A	-0.2536	0.051	0.0618	3656	7.31E-07	0.006691671
T	C	0.5455	0.088	0.02	3656	6.54E-10	0.010384695
G	A	0.1827	0.026	0.3256	3656	4.02E-12	0.013076627
G	A	1.799	0.384	0.001	3656	2.86E-06	0.005973709
C	G	0.3287	0.057	0.045	3656	8.75E-09	0.009010732
C	T	-0.1203	0.025	0.5737	3656	1.08E-06	0.006482934
G	C	0.416	0.088	0.0174	3656	2.57E-06	0.006030276
C	A	-0.1271	0.024	0.4903	3656	1.16E-07	0.0076507
A	G	-0.2695	0.058	0.0435	3656	3.21E-06	0.005915339
A	G	-0.1525	0.026	0.6359	3656	7.20E-09	0.009119554
G	A	-0.3702	0.083	0.0228	3656	7.92E-06	0.00544583
T	C	0.2264	0.047	0.074	3656	1.47E-06	0.006322747
C	T	-0.2462	0.048	0.0706	3656	3.33E-07	0.00709747
A	G	-0.2734	0.057	0.0472	3656	1.97E-06	0.006167092
T	C	-0.3248	0.07	0.0312	3656	3.80E-06	0.005827849
A	T	-0.1469	0.03	0.7882	3656	1.42E-06	0.006342221
C	T	-0.3044	0.054	0.0562	3656	1.83E-08	0.008626125
C	T	0.4221	0.087	0.0194	3656	1.45E-06	0.006330638
T	C	-0.2241	0.048	0.9301	3656	3.31E-06	0.005897358
T	C	-0.3731	0.082	0.0257	3656	4.87E-06	0.00569685
G	T	0.114	0.025	0.5868	3656	7.13E-06	0.005496816
G	A	-0.113	0.025	0.6033	3656	9.39E-06	0.005350838
A	G	-0.3442	0.074	0.0279	3656	3.97E-06	0.005806182
G	A	0.1325	0.025	0.5341	3656	7.13E-08	0.007910922
T	A	-0.239	0.054	0.0543	3656	8.42E-06	0.005412863
A	G	-0.1285	0.026	0.6726	3656	1.20E-06	0.006428852
T	C	-0.1612	0.034	0.1551	3656	1.87E-06	0.006193889
C	T	-0.2227	0.047	0.0722	3656	2.50E-06	0.006047113
C	T	0.3695	0.08	0.0239	3656	4.23E-06	0.005771013

G	T	0.317	0.026	0.6313	3656	4.98E-33	0.038443402
C	T	0.1684	0.031	0.1893	3656	8.92E-08	0.007790985
C	T	0.1674	0.032	0.1957	3656	1.34E-07	0.007579319
T	C	0.1649	0.036	0.1493	3656	3.54E-06	0.005867092
T	C	0.3178	0.071	0.03	3656	7.56E-06	0.005468556
A	G	0.1313	0.029	0.2492	3656	6.23E-06	0.005568055
T	A	-0.1343	0.028	0.2842	3656	1.44E-06	0.00633844
C	T	0.3656	0.077	0.0272	3656	2.20E-06	0.006111145
G	A	-0.1437	0.025	0.5715	3656	1.21E-08	0.008843583
T	C	0.199	0.045	0.0907	3656	9.68E-06	0.005341801
T	C	1.115	0.228	0.0025	3656	1.08E-06	0.006481977
G	C	-0.2182	0.043	0.0989	3656	4.52E-07	0.006942483
T	C	0.1133	0.025	0.4387	3656	6.36E-06	0.005564355
G	A	0.1567	0.025	0.5044	3656	2.91E-10	0.010810807
T	G	-0.1491	0.025	0.592	3656	2.30E-09	0.009719802
T	C	0.5148	0.114	0.0116	3656	6.92E-06	0.00551792
A	C	0.311	0.07	0.0319	3656	8.78E-06	0.0053915
T	C	0.17	0.038	0.1219	3656	6.95E-06	0.00551349
T	C	-0.7635	0.172	0.0049	3656	8.80E-06	0.005391824
T	C	2.328	0.496	0.0005	3657	2.79E-06	0.005985429
T	A	2.608	0.553	0.0008	3657	2.47E-06	0.006053845
T	C	1.756	0.375	0.0011	3657	2.92E-06	0.005963422
C	T	0.1298	0.028	0.7401	3657	3.67E-06	0.00584203
G	A	0.5223	0.109	0.0124	3657	1.65E-06	0.006262231
T	G	0.2681	0.058	0.0447	3657	4.70E-06	0.005714327
A	G	-0.1218	0.027	0.2815	3657	5.98E-06	0.005595567
C	T	0.351	0.075	0.0279	3657	2.65E-06	0.006010737
T	C	-0.2314	0.049	0.0678	3657	2.62E-06	0.006017326
A	C	-0.1755	0.033	0.1608	3657	9.04E-08	0.007786577
A	G	-0.158	0.036	0.1359	3657	9.27E-06	0.005363419
G	T	-0.1183	0.027	0.3001	3657	8.57E-06	0.005403679
A	G	1.624	0.347	0.0015	3657	2.91E-06	0.005967474
A	G	0.1516	0.034	0.1596	3657	7.12E-06	0.005499975
A	G	0.1167	0.026	0.3142	3657	9.94E-06	0.005322912
T	C	-0.1403	0.026	0.6349	3657	4.62E-08	0.008133646
C	T	-0.1601	0.035	0.853	3657	4.95E-06	0.005689103
T	C	-0.1294	0.029	0.2244	3657	7.04E-06	0.00550514
G	A	0.1295	0.027	0.2882	3657	1.45E-06	0.006325483
A	G	0.2864	0.064	0.0354	3656	8.20E-06	0.005427369
T	C	-0.3971	0.085	0.0201	3656	3.46E-06	0.005875079
A	G	-0.1135	0.025	0.5429	3656	6.74E-06	0.005531085
G	T	0.1242	0.027	0.3031	3656	2.98E-06	0.005949989
A	G	-0.5299	0.116	0.0115	3656	4.79E-06	0.005704667
A	C	2.518	0.521	0.0005	3656	1.41E-06	0.006343555
C	T	-0.1889	0.042	0.0946	3656	7.88E-06	0.005448217
T	A	-0.1473	0.033	0.8254	3656	6.39E-06	0.00555323
C	T	-0.2033	0.04	0.1057	3656	3.08E-07	0.00714312
G	A	-0.144	0.032	0.8222	3656	7.39E-06	0.005481042

T	C	0.6141	0.135	0.0086	3656	5.31E-06	0.005652946
T	C	-0.2635	0.058	0.9547	3656	5.25E-06	0.005660248
A	G	0.1453	0.03	0.2153	3656	1.24E-06	0.006413541
C	T	0.132	0.027	0.7213	3656	1.64E-06	0.006267032
G	A	-0.1793	0.039	0.1134	3656	4.28E-06	0.005765689
A	G	0.1112	0.025	0.5364	3656	5.86E-06	0.005603133
T	C	1.357	0.305	0.0015	3656	8.92E-06	0.005378267
G	A	1.799	0.385	0.001	3656	3.11E-06	0.005933685
G	A	0.2221	0.026	0.3256	3656	3.31E-17	0.019276794
C	G	0.112	0.025	0.4402	3656	5.93E-06	0.005596933
T	C	0.442	0.088	0.02	3656	5.44E-07	0.006842276
C	G	0.3049	0.057	0.045	3656	1.13E-07	0.007666491
A	G	0.1136	0.025	0.5141	3656	4.17E-06	0.005780314
G	A	1.237	0.25	0.0025	3656	7.49E-07	0.006678546
T	G	-0.1359	0.025	0.592	3656	3.17E-08	0.008338912
A	G	-0.2668	0.058	0.0435	3656	4.68E-06	0.005717138
T	C	-0.5751	0.124	0.01	3656	3.62E-06	0.005849106
G	T	-0.1132	0.025	0.5444	3656	4.24E-06	0.005772476
T	A	0.1493	0.032	0.83	3656	4.01E-06	0.005795742
G	T	-0.3841	0.086	0.9792	3656	8.81E-06	0.005387831
G	A	0.271	0.061	0.0465	3656	9.18E-06	0.005366008
C	A	-0.3046	0.067	0.0357	3656	6.34E-06	0.005560309
T	G	-0.479	0.103	0.0161	3656	3.27E-06	0.005903467
C	T	-0.2266	0.046	0.081	3656	8.39E-07	0.006616477
C	G	-0.1954	0.044	0.1131	3656	9.12E-06	0.005372673
T	C	0.1611	0.034	0.1536	3656	2.83E-06	0.00598381
A	G	0.2916	0.065	0.0377	3656	6.51E-06	0.005547424
T	C	0.4648	0.102	0.0153	3656	4.94E-06	0.005691931
G	T	-0.1504	0.028	0.2657	3656	6.15E-08	0.00798767
C	T	0.3335	0.074	0.9725	3656	6.97E-06	0.005511453
C	G	0.3348	0.065	0.0387	3656	2.50E-07	0.007250839
C	T	0.1509	0.032	0.1969	3656	1.85E-06	0.006198658
A	C	0.3341	0.073	0.0302	3656	4.39E-06	0.00575136
C	G	0.1156	0.026	0.3832	3656	6.48E-06	0.005550748
A	G	-0.3176	0.066	0.0343	3656	1.68E-06	0.00625436
A	C	0.2756	0.057	0.0501	3656	1.42E-06	0.00634054
A	G	-0.2342	0.051	0.0662	3656	3.92E-06	0.005811736
G	A	0.3445	0.071	0.031	3656	1.46E-06	0.00632555
T	A	-0.1308	0.029	0.4267	3656	5.42E-06	0.005641356
T	G	-0.1122	0.025	0.592	3656	6.17E-06	0.005576329
G	A	-0.1599	0.033	0.823	3656	1.42E-06	0.006346469
G	A	0.1132	0.026	0.6219	3656	9.63E-06	0.005344628
C	T	-0.1135	0.025	0.4132	3656	5.88E-06	0.005601683
G	A	-0.3974	0.089	0.0185	3656	8.24E-06	0.005425061
T	C	0.405	0.082	0.0219	3656	9.04E-07	0.006576993
C	T	0.14	0.03	0.1893	3656	4.09E-06	0.00578645
G	C	0.3388	0.073	0.0286	3656	3.29E-06	0.005903653
A	T	0.141	0.029	0.2246	3656	1.12E-06	0.006459827

T	C	0.3656	0.077	0.0235	3656	1.96E-06	0.006174649
C	T	0.1715	0.039	0.1041	3656	9.56E-06	0.005342857
G	A	-0.1267	0.024	0.5715	3656	1.85E-07	0.007411274
C	T	-0.1298	0.027	0.2824	3656	1.56E-06	0.006290983
G	C	-0.1579	0.034	0.1496	3656	3.84E-06	0.005820367
T	C	0.3355	0.07	0.0295	3656	1.79E-06	0.006219249
G	C	0.3309	0.071	0.0289	3656	3.63E-06	0.00585023
T	C	-0.1927	0.043	0.0886	3656	7.15E-06	0.005498678
T	C	0.1583	0.033	0.1703	3656	1.24E-06	0.006415908
A	G	0.8492	0.156	0.006	3656	5.92E-08	0.008009462
G	A	0.1117	0.024	0.5044	3656	3.14E-06	0.005919343
G	A	0.1277	0.028	0.2382	3656	7.64E-06	0.005465258
T	G	-0.1121	0.024	0.592	3656	3.29E-06	0.005907472
A	G	0.8913	0.199	0.0034	3656	7.49E-06	0.005473464
G	A	-0.119	0.026	0.7021	3656	5.45E-06	0.005636658
A	G	0.6139	0.132	0.0081	3656	3.48E-06	0.005872534
A	G	-0.2606	0.058	0.0475	3656	6.86E-06	0.005518013
T	C	-0.4103	0.086	0.0201	3656	1.82E-06	0.006211729
C	A	-0.1746	0.037	0.8771	3656	1.87E-06	0.006192941
C	G	0.3639	0.081	0.0235	3656	6.68E-06	0.005533708
A	G	0.1127	0.025	0.3734	3656	7.14E-06	0.005501532
G	A	-0.1736	0.039	0.1134	3656	9.36E-06	0.00536023
A	G	0.2998	0.061	0.0402	3656	8.90E-07	0.00658711
G	A	0.2335	0.026	0.3256	3656	1.09E-18	0.021073938
T	C	0.2109	0.047	0.0777	3656	6.71E-06	0.005528646
T	C	0.4819	0.089	0.02	3656	6.07E-08	0.00799629
C	G	0.3082	0.057	0.045	3656	8.52E-08	0.007818507
C	T	-0.11	0.025	0.5737	3656	9.60E-06	0.005348066
C	T	-0.1428	0.024	0.475	3656	3.70E-09	0.00946512
A	G	-0.263	0.058	0.0435	3656	6.40E-06	0.005558239
G	T	-0.1107	0.025	0.5444	3656	7.52E-06	0.005468465
A	C	0.1339	0.029	0.2311	3656	5.57E-06	0.00562258
C	T	0.1417	0.03	0.2289	3656	2.22E-06	0.006105642
C	T	1.002	0.226	0.003	3656	9.47E-06	0.005347904
C	T	0.2499	0.056	0.0553	3656	8.30E-06	0.005419328
A	G	0.1576	0.033	0.1711	3656	1.93E-06	0.006177452
C	T	-0.1917	0.043	0.0909	3656	9.30E-06	0.005364606
G	A	0.3433	0.076	0.0286	3656	6.45E-06	0.005550053
A	G	-1.768	0.393	0.0016	3656	7.05E-06	0.005505233
G	T	0.1202	0.026	0.5868	3656	3.00E-06	0.005956867
G	A	0.2699	0.056	0.0555	3656	1.36E-06	0.006363122
T	C	-0.328	0.071	0.0325	3656	3.94E-06	0.005806846
A	G	-0.1156	0.025	0.5149	3656	5.20E-06	0.005660191
C	T	-1.07	0.237	0.0029	3656	6.60E-06	0.005539693
T	C	0.2673	0.056	0.0555	3656	1.58E-06	0.006286599
T	A	-0.1184	0.027	0.557	3656	9.09E-06	0.005373888
A	G	-0.2174	0.048	0.0788	3656	5.58E-06	0.005626092
G	A	0.3254	0.072	0.031	3656	7.02E-06	0.005506984

A	G	0.2573	0.057	0.0506	3656	6.28E-06	0.005563888
A	G	0.1187	0.027	0.6422	3656	8.15E-06	0.005429349
A	T	-0.2581	0.056	0.0517	3656	4.05E-06	0.005791057
A	G	0.4267	0.09	0.0187	3656	2.46E-06	0.006054422
A	C	-0.1174	0.025	0.4768	3656	2.72E-06	0.006000448
C	T	-0.4244	0.091	0.0198	3656	3.31E-06	0.005898585
T	C	-0.1264	0.027	0.6704	3656	2.24E-06	0.006101803
C	T	-0.1313	0.03	0.7674	3656	9.51E-06	0.005353142
G	T	-0.2649	0.057	0.0494	3656	3.95E-06	0.005807854
A	T	-0.1136	0.026	0.3489	3656	9.49E-06	0.005348882
T	A	0.1567	0.032	0.1644	3656	1.45E-06	0.006333959
C	T	-0.2015	0.045	0.081	3656	8.14E-06	0.005432724
C	A	-0.2982	0.066	0.0357	3656	6.99E-06	0.005509433
T	G	-0.4604	0.101	0.0161	3656	5.09E-06	0.005673777
T	C	0.1447	0.032	0.1864	3656	4.58E-06	0.005727805
C	T	0.1258	0.027	0.2964	3656	3.44E-06	0.005881104
C	G	-0.2163	0.042	0.1131	3656	3.57E-07	0.007061353
A	G	0.2933	0.063	0.0377	3656	3.86E-06	0.005819753
T	A	-0.282	0.057	0.048	3656	7.03E-07	0.00670633
C	A	-0.1572	0.031	0.1943	3656	2.72E-07	0.007208955
C	T	0.1404	0.031	0.1969	3656	6.34E-06	0.005557822
C	G	0.3428	0.064	0.0387	3656	8.20E-08	0.0078395
A	G	0.6298	0.14	0.0078	3656	6.90E-06	0.005512682
C	T	0.3524	0.079	0.0249	3656	8.62E-06	0.005400954
A	G	0.3251	0.069	0.0298	3656	2.90E-06	0.005969786
C	G	0.1312	0.028	0.2462	3656	3.38E-06	0.005889872
A	G	-0.232	0.05	0.0662	3656	3.28E-06	0.005903561
C	T	-0.1133	0.025	0.6041	3656	4.06E-06	0.005796685
T	A	-0.1318	0.028	0.4267	3656	1.87E-06	0.006194315
C	T	-0.1549	0.034	0.1469	3656	6.68E-06	0.00553139
G	A	-0.152	0.032	0.823	3656	2.86E-06	0.005976564
G	A	-0.1285	0.026	0.6172	3656	6.64E-07	0.006739441
T	C	-0.4263	0.085	0.0201	3656	5.73E-07	0.00681543
T	C	1.557	0.342	0.0011	3656	5.46E-06	0.005640483
A	T	0.1322	0.029	0.2246	3656	7.23E-06	0.005492618
A	G	0.6143	0.133	0.0082	3656	3.73E-06	0.005836141
G	T	0.1237	0.026	0.3031	3656	3.03E-06	0.005942444
A	C	-0.1241	0.027	0.2961	3656	3.51E-06	0.005869928
A	C	2.517	0.52	0.0005	3656	1.32E-06	0.006379836
C	T	0.1315	0.029	0.2211	3656	7.92E-06	0.005449649
A	T	-0.1287	0.027	0.3005	3656	1.96E-06	0.006176361
C	T	-0.1838	0.04	0.1057	3656	3.48E-06	0.0058756
T	C	0.6284	0.134	0.0086	3656	2.95E-06	0.00596162
G	A	0.1127	0.024	0.4707	3656	2.41E-06	0.006065388
C	T	-0.1323	0.029	0.7592	3656	5.48E-06	0.005637248
A	G	-0.1445	0.032	0.1885	3656	5.68E-06	0.005619543
G	T	-0.1102	0.025	0.5003	3656	7.94E-06	0.005441342
A	G	0.3787	0.081	0.9772	3656	2.70E-06	0.006005015

G	A	-0.2708	0.051	0.0618	3656	1.24E-07	0.007620042
G	A	0.2114	0.026	0.3256	3656	8.09E-16	0.017574868
G	A	1.814	0.384	0.001	3656	2.37E-06	0.006073134
T	C	0.5056	0.088	0.02	3656	8.75E-09	0.009013112
G	A	2.99	0.673	0.0003	3656	8.99E-06	0.005376273
G	A	-0.1323	0.028	0.2676	3656	2.49E-06	0.006052308
C	G	0.3074	0.057	0.045	3656	8.06E-08	0.007845927
C	A	-0.1352	0.024	0.4903	3656	1.81E-08	0.008626772
A	G	-0.2739	0.058	0.0435	3656	2.40E-06	0.006067063
T	G	0.3643	0.079	0.0246	3657	4.71E-06	0.005714881
T	C	-0.1695	0.038	0.1154	3657	7.91E-06	0.005442459
T	C	0.3067	0.068	0.0316	3657	6.12E-06	0.005577501
T	G	0.4826	0.105	0.0142	3657	3.97E-06	0.005798179
A	C	0.1097	0.025	0.4584	3657	7.83E-06	0.005447901
T	A	0.5621	0.126	0.01	3657	7.85E-06	0.005446919
T	C	0.179	0.034	0.5975	3657	1.61E-07	0.007478462
G	A	1.534	0.282	0.0018	3657	5.81E-08	0.008020882
T	G	0.2658	0.059	0.0447	3657	6.38E-06	0.005556616
A	T	-0.1242	0.028	0.2594	3657	8.97E-06	0.005374339
A	C	-0.1598	0.035	0.138	3657	5.40E-06	0.005645447
A	T	0.513	0.106	0.0149	3657	1.38E-06	0.006352016
T	C	-0.7403	0.158	0.0059	3657	3.02E-06	0.005944827
C	G	-0.1257	0.027	0.2863	3657	3.00E-06	0.00594426
C	T	-0.1712	0.037	0.118	3657	4.57E-06	0.005727569
G	T	-0.1244	0.026	0.3414	3657	1.99E-06	0.006168998
C	G	0.1079	0.024	0.5648	3657	8.41E-06	0.005415605
G	A	0.4085	0.088	0.9802	3657	3.15E-06	0.005924645
C	T	0.1453	0.032	0.1705	3657	6.52E-06	0.005540531
T	C	0.6312	0.141	0.0077	3657	8.27E-06	0.005419387
G	C	-0.1132	0.025	0.4371	3657	4.25E-06	0.005761577
G	T	-0.2457	0.052	0.0585	3658	2.38E-06	0.006068529
C	A	0.1217	0.027	0.6963	3658	8.78E-06	0.005387587
C	A	0.1135	0.025	0.3682	3658	6.04E-06	0.005585305
A	G	0.5046	0.112	0.0118	3658	7.41E-06	0.005479387
T	C	0.6295	0.132	0.0089	3658	1.89E-06	0.006188171
T	C	-0.1405	0.032	0.1736	3658	9.44E-06	0.005348225
A	G	-0.1476	0.029	0.7802	3658	3.12E-07	0.007134052
T	C	0.2022	0.042	0.6662	3658	1.22E-06	0.00641706
A	T	0.1807	0.041	0.903	3658	8.55E-06	0.005404628
T	C	0.4968	0.11	0.0115	3658	6.54E-06	0.005535212
T	C	-0.1469	0.033	0.1643	3658	6.84E-06	0.005513544
A	T	0.2924	0.062	0.0402	3658	2.21E-06	0.006106078
C	G	-0.1443	0.031	0.1837	3658	4.19E-06	0.005769425
C	T	-0.1261	0.028	0.7571	3658	8.97E-06	0.005371907
T	G	0.1512	0.028	0.2346	3658	8.46E-08	0.007814105
A	G	-1.031	0.223	0.0033	3658	3.85E-06	0.005819804
C	T	-0.7613	0.15	0.0066	3658	4.02E-07	0.007001855
C	T	0.1354	0.03	0.2063	3658	6.72E-06	0.005530491

G	A	0.2263	0.05	0.0596	3658	7.01E-06	0.005505093
T	G	0.3075	0.062	0.0377	3658	8.46E-07	0.006607449
A	C	-0.2605	0.056	0.0484	3658	3.92E-06	0.005806318
C	T	-0.2259	0.048	0.0794	3658	2.08E-06	0.006139898
A	G	-0.1164	0.025	0.5914	3658	2.61E-06	0.006024776
A	G	-0.2517	0.055	0.0496	3658	5.54E-06	0.005627397
A	G	0.1556	0.033	0.1597	3658	3.19E-06	0.00591924
A	C	0.1305	0.029	0.2301	3657	7.45E-06	0.005476741
A	G	-0.4062	0.091	0.0194	3657	7.96E-06	0.005440299
C	G	-0.9757	0.207	0.9962	3657	2.53E-06	0.006038603
G	T	-0.1903	0.04	0.103	3657	2.65E-06	0.006015823
T	A	-0.1414	0.028	0.2841	3657	3.47E-07	0.007080145
C	T	-0.2629	0.056	0.0502	3657	2.82E-06	0.005979985
T	C	-0.1444	0.031	0.4642	3657	3.98E-06	0.005804717
T	C	0.1668	0.032	0.816	3657	1.61E-07	0.007481203
T	C	-0.4466	0.094	0.0179	3657	2.10E-06	0.006135871
G	A	-0.5468	0.123	0.0096	3657	8.75E-06	0.005392462
T	C	-0.3746	0.081	0.0231	3657	4.21E-06	0.005771857
C	G	-0.1137	0.025	0.4839	3657	4.95E-06	0.00569199
G	A	0.1743	0.036	0.1328	3657	1.78E-06	0.006224126
G	A	-0.2726	0.06	0.0472	3656	4.82E-06	0.005702824
A	C	0.142	0.029	0.23	3656	1.41E-06	0.006340354
A	G	-0.2926	0.058	0.0509	3656	4.08E-07	0.006991902
A	T	-1.898	0.408	0.0008	3656	3.41E-06	0.005884408
C	G	0.6003	0.135	0.0083	3656	9.57E-06	0.005347668
C	T	0.2161	0.049	0.0673	3656	8.82E-06	0.005387656
G	C	-1.445	0.291	0.0018	3656	7.04E-07	0.006712962
A	G	0.1401	0.027	0.7103	3656	3.39E-07	0.007089981
T	C	0.6143	0.138	0.0086	3656	8.96E-06	0.005382987
T	C	-0.2685	0.055	0.058	3656	8.91E-07	0.006583009
T	C	0.7592	0.168	0.0055	3656	6.02E-06	0.005587828
C	T	-0.4181	0.093	0.0197	3656	6.39E-06	0.00555713
A	C	-0.3138	0.064	0.9583	3656	8.60E-07	0.006602214
G	C	-0.627	0.113	0.0126	3656	3.14E-08	0.0083362
A	G	0.5677	0.123	0.0103	3656	3.78E-06	0.005830571
T	A	0.2307	0.048	0.0666	3657	1.79E-06	0.006220223
T	G	0.2748	0.062	0.0368	3657	9.38E-06	0.005356904
A	G	-0.547	0.114	0.0108	3657	1.60E-06	0.006278126
C	T	-0.3401	0.064	0.0368	3657	9.31E-08	0.007768436
A	G	-0.4136	0.087	0.0194	3657	2.27E-06	0.006093347
G	C	0.3089	0.067	0.0334	3657	4.25E-06	0.005768611
A	G	1.714	0.372	0.001	3657	4.16E-06	0.005774696
T	A	0.6715	0.147	0.0063	3657	4.69E-06	0.005712207
A	G	-0.2113	0.042	0.0913	3657	6.72E-07	0.006732702
A	T	-0.4299	0.096	0.0159	3657	8.30E-06	0.005419973
C	A	0.8693	0.196	0.0037	3657	9.46E-06	0.00535023
A	C	-0.188	0.042	0.0928	3657	6.50E-06	0.005545781
A	T	0.4342	0.096	0.0156	3657	6.42E-06	0.005551244

T	C	0.1803	0.041	0.1922	3657	8.88E-06	0.005382319
A	G	-1.169	0.259	0.0023	3657	6.53E-06	0.005544029
C	G	-0.2556	0.052	0.0556	3657	1.14E-06	0.006456895
T	G	-0.1953	0.04	0.1036	3657	1.28E-06	0.00639677
G	A	0.1564	0.035	0.1389	3657	8.44E-06	0.005412126
T	C	0.3556	0.079	0.0231	3657	7.13E-06	0.005498833
A	C	-0.4658	0.096	0.0161	3657	1.40E-06	0.006347821
A	G	-0.1368	0.027	0.6906	3657	5.78E-07	0.006804601
C	T	-0.676	0.152	0.0057	3657	8.60E-06	0.005400643
A	C	0.3538	0.078	0.0246	3657	5.42E-06	0.005639032
G	C	-0.6854	0.139	0.0074	3657	8.88E-07	0.006585899
C	T	0.517	0.115	0.0115	3657	7.21E-06	0.005496255
C	T	-0.1464	0.031	0.8141	3657	2.26E-06	0.0060929
A	G	-0.122	0.026	0.6504	3657	2.29E-06	0.006091333
T	C	-0.33	0.068	0.0335	3657	1.24E-06	0.00641189
T	G	-0.1756	0.038	0.1422	3020	4.06E-06	0.007010261
G	T	0.1562	0.03	0.7464	3020	2.98E-07	0.008660534
G	A	0.9648	0.202	0.0041	3020	1.95E-06	0.007467775
T	C	-0.1552	0.034	0.2225	3020	3.98E-06	0.007019356
T	C	0.5582	0.125	0.0124	3020	7.77E-06	0.006601764
A	G	0.6966	0.157	0.0073	3020	9.01E-06	0.006509389
T	G	0.2511	0.056	0.0594	3020	9.05E-06	0.006504543
A	G	0.1506	0.034	0.1901	3020	9.65E-06	0.006465984
A	G	-0.3546	0.078	0.0305	3020	5.92E-06	0.006772863
T	C	-0.1429	0.031	0.2432	3020	4.55E-06	0.006942424
G	C	0.8103	0.178	0.0058	3020	5.74E-06	0.006792382
G	T	-0.1256	0.028	0.3124	3020	9.77E-06	0.006452803
T	C	0.2681	0.06	0.05	3020	8.05E-06	0.006576546
A	T	-0.3991	0.086	0.0268	3020	3.38E-06	0.007123359
C	T	-0.2994	0.063	0.0472	3020	2.17E-06	0.007406659
G	T	-0.4778	0.105	0.0151	3020	5.93E-06	0.00677139
A	G	-0.2321	0.052	0.0919	3020	8.62E-06	0.006531138
A	G	0.25	0.052	0.0674	3020	1.60E-06	0.00759258
G	A	0.4346	0.08	0.0292	3043	7.01E-08	0.009506074
A	T	0.4772	0.102	0.0168	3043	3.11E-06	0.00712756
T	C	0.6366	0.125	0.0125	3043	3.37E-07	0.008518774
C	T	0.1661	0.037	0.1456	3043	8.53E-06	0.006488206
A	G	-0.2147	0.045	0.0971	3043	1.77E-06	0.007477754
T	A	-0.1934	0.043	0.104	3043	7.00E-06	0.006616062
T	C	0.545	0.12	0.0122	3043	6.05E-06	0.006710547
G	A	-0.28	0.061	0.0483	3043	4.95E-06	0.006829566
A	G	0.591	0.128	0.0115	3043	4.23E-06	0.006924714
G	T	0.1214	0.027	0.3971	3043	7.71E-06	0.006551522
T	C	0.4148	0.088	0.023	3043	2.38E-06	0.007289603
C	G	-0.575	0.13	0.0107	3043	9.30E-06	0.00643709
T	C	0.1211	0.027	0.3899	3043	7.22E-06	0.006596549
T	C	-0.1616	0.036	0.1687	3043	6.73E-06	0.006640391
G	C	-0.124	0.027	0.4358	3043	4.53E-06	0.006893714

C	G	0.3158	0.063	0.0462	3043	4.67E-07	0.008312284
A	G	-0.4763	0.096	0.0189	3043	6.82E-07	0.008074477
T	C	-0.6761	0.141	0.0089	3043	1.81E-06	0.007457107
G	A	0.1924	0.042	0.1119	3043	5.51E-06	0.006768698
A	G	-0.1936	0.044	0.1032	3043	9.62E-06	0.006414294
G	A	0.1465	0.03	0.7212	3043	8.11E-07	0.007969572
A	G	-0.1569	0.035	0.1661	3043	7.20E-06	0.006598088
C	T	0.1579	0.033	0.198	3043	1.80E-06	0.007463077
C	A	-0.3696	0.065	0.0459	3019	1.22E-08	0.010693579
T	G	0.2378	0.03	0.2774	3019	3.61E-15	0.020308231
A	G	-0.347	0.075	0.0341	3019	3.48E-06	0.007108116
T	C	0.269	0.059	0.0581	3019	4.62E-06	0.006929123
T	G	0.1274	0.028	0.3596	3019	5.45E-06	0.006820373
C	G	0.1842	0.036	0.1686	3019	3.50E-07	0.008564232
A	G	0.1235	0.027	0.5561	3019	6.87E-06	0.006679472
A	G	-0.2033	0.045	0.1014	3019	6.96E-06	0.006670974
A	G	-0.1525	0.026	0.4581	3019	7.66E-09	0.010997706
G	T	1.724	0.353	0.0015	3019	1.05E-06	0.007860783
G	T	0.859	0.183	0.0056	3019	2.75E-06	0.007253277
A	G	-0.1386	0.03	0.7173	3019	4.37E-06	0.00696495
C	T	0.1627	0.033	0.2211	3019	6.13E-07	0.008202874
T	C	2.05	0.404	0.0013	3019	4.12E-07	0.00846071
A	G	0.352	0.079	0.0301	3019	9.30E-06	0.006488991
A	G	0.3974	0.09	0.0235	3019	9.33E-06	0.006488131
A	C	0.222	0.049	0.083	3019	5.12E-06	0.006861223
C	A	0.139	0.029	0.3475	3019	1.21E-06	0.007774144
G	T	0.4639	0.104	0.0171	3019	8.44E-06	0.006547359
C	T	-0.1417	0.031	0.2448	3019	6.08E-06	0.006755801
T	G	0.295	0.061	0.0517	3019	1.43E-06	0.00766726
G	A	0.3359	0.03	0.2763	3042	4.37E-29	0.040377625
C	A	-0.3294	0.065	0.0457	3042	3.93E-07	0.008420377
C	T	0.3492	0.079	0.0306	3042	9.03E-06	0.006458092
C	T	0.5758	0.13	0.0113	3042	9.35E-06	0.006437237
A	C	-0.1976	0.043	0.106	3042	5.66E-06	0.006749846
A	T	0.228	0.05	0.0769	3042	6.40E-06	0.006674582
A	G	0.227	0.042	0.1205	3042	4.89E-08	0.009735056
G	A	-0.1512	0.032	0.2516	3042	2.34E-06	0.007303772
A	G	0.1467	0.03	0.2855	3042	7.77E-07	0.007999134
G	C	-0.2904	0.058	0.0569	3042	5.02E-07	0.008269484
A	G	-0.1315	0.026	0.4578	3042	6.30E-07	0.008126751
G	A	-0.1315	0.027	0.4988	3042	1.37E-06	0.007635973
T	C	0.4905	0.109	0.0155	3042	7.02E-06	0.006612785
T	C	-0.3108	0.069	0.0396	3042	7.45E-06	0.006579935
G	C	-0.4256	0.094	0.0212	3042	6.39E-06	0.006675428
C	T	0.1614	0.033	0.2206	3042	7.28E-07	0.00803238
T	C	0.2982	0.067	0.0403	3042	9.82E-06	0.006406892
T	C	0.2947	0.054	0.0676	3042	4.96E-08	0.009731427
G	A	0.5719	0.12	0.013	3042	2.07E-06	0.007374543

A	G	-0.1913	0.039	0.132	3042	1.28E-06	0.00767838
A	T	0.6623	0.12	0.0135	3042	3.74E-08	0.009897927
T	C	-0.1787	0.036	0.1624	3042	9.95E-07	0.007835026
T	C	-0.2057	0.04	0.1333	3042	2.34E-07	0.008748073
A	C	0.1341	0.029	0.283	3042	5.31E-06	0.006792718
C	G	0.2127	0.048	0.0866	3042	8.94E-06	0.006461618
G	T	0.1532	0.03	0.7463	3017	3.60E-07	0.00855266
C	T	0.201	0.045	0.9123	3017	9.81E-06	0.006460601
T	C	-0.1809	0.031	0.231	3017	4.95E-09	0.011275823
T	C	-0.2006	0.043	0.1018	3017	3.61E-06	0.007092945
G	A	0.9448	0.2	0.0041	3017	2.47E-06	0.007327943
T	C	-0.1559	0.033	0.2224	3017	2.81E-06	0.00724701
A	G	0.7057	0.155	0.0073	3017	5.50E-06	0.006823824
T	G	0.2484	0.056	0.0595	3017	9.11E-06	0.006504673
G	T	-1.36	0.29	0.002	3017	2.95E-06	0.007217114
C	G	0.225	0.049	0.0766	3017	4.02E-06	0.007019656
G	A	0.117	0.026	0.5486	3017	5.51E-06	0.00681744
G	A	0.3583	0.08	0.027	3017	8.13E-06	0.006577014
T	G	0.1808	0.04	0.1178	3017	5.15E-06	0.006861844
T	A	-0.1217	0.027	0.3378	3017	7.99E-06	0.006586863
A	G	1.307	0.284	0.0023	3017	4.24E-06	0.006990637
T	C	0.3099	0.059	0.05	3017	1.68E-07	0.009028325
T	C	-0.5233	0.109	0.0146	3017	1.76E-06	0.007540474
A	G	-0.2264	0.051	0.0746	3017	7.66E-06	0.006617762
C	A	0.1241	0.027	0.3474	3017	6.44E-06	0.006719283
C	G	0.2509	0.055	0.06	3017	4.70E-06	0.006925216
T	A	-0.1526	0.033	0.7957	3017	3.01E-06	0.007201566
A	T	-0.4108	0.085	0.0268	3017	1.36E-06	0.007709403
G	A	-0.1581	0.035	0.8397	3017	6.42E-06	0.0067254
A	G	0.2439	0.051	0.0673	3017	2.27E-06	0.007382179
T	C	0.1774	0.039	0.121	3017	7.30E-06	0.006647845
A	G	0.245	0.055	0.0661	3040	9.40E-06	0.00643836
A	G	-0.6187	0.135	0.0105	3040	4.37E-06	0.006912412
G	A	0.3846	0.081	0.0293	3040	2.30E-06	0.007318383
A	G	0.207	0.045	0.0993	3040	3.52E-06	0.007051773
T	G	-0.195	0.039	0.1418	3040	4.68E-07	0.008316568
A	G	0.2479	0.054	0.0673	3040	5.50E-06	0.006774715
T	C	0.1244	0.027	0.387	3040	5.94E-06	0.006725146
T	C	0.5956	0.126	0.0125	3040	2.52E-06	0.007262124
C	T	0.1689	0.038	0.1456	3040	7.32E-06	0.006590334
A	G	-0.2161	0.047	0.0895	3040	3.69E-06	0.007024295
G	A	-0.2769	0.062	0.0482	3040	7.23E-06	0.006602886
T	C	0.4544	0.089	0.0229	3040	3.43E-07	0.008515155
T	C	0.1227	0.027	0.3901	3040	7.18E-06	0.006610682
T	A	0.2989	0.065	0.9564	3040	3.91E-06	0.00698444
C	G	0.3077	0.063	0.0462	3040	1.22E-06	0.007717626
A	C	0.25	0.048	0.0831	3040	2.11E-07	0.008822473
G	C	-0.1298	0.027	0.4359	3040	2.05E-06	0.007392049

T	C	-0.6373	0.143	0.0089	3040	8.25E-06	0.006518178
G	A	0.1991	0.043	0.1118	3040	3.12E-06	0.007124153
A	G	-0.1976	0.044	0.1033	3040	7.82E-06	0.006552055
G	A	0.1484	0.03	0.7286	3040	9.13E-07	0.007901081
T	C	0.3042	0.067	0.0396	3018	5.91E-06	0.006778085
C	A	0.5986	0.134	0.0096	3018	8.27E-06	0.006568745
T	C	0.1401	0.032	0.2179	3018	9.92E-06	0.006450585
G	C	-0.2927	0.066	0.0409	3018	8.98E-06	0.006511861
T	C	-0.1209	0.027	0.6269	3018	8.65E-06	0.006537105
C	T	0.1137	0.026	0.4606	3018	9.53E-06	0.006473588
G	A	-0.2094	0.046	0.0911	3018	6.35E-06	0.006731915
T	C	-0.1197	0.027	0.4032	3018	8.75E-06	0.006527791
A	G	-0.1284	0.028	0.3118	3018	5.90E-06	0.006774633
A	G	0.1276	0.029	0.3101	3018	8.36E-06	0.006561433
A	G	0.2396	0.05	0.0747	3018	1.47E-06	0.007654273
C	T	0.1364	0.027	0.6473	3018	6.94E-07	0.008126697
A	G	-0.4212	0.09	0.0209	3018	2.86E-06	0.007235265
C	A	0.1267	0.028	0.3474	3018	4.53E-06	0.006939153
A	G	0.3934	0.083	0.0255	3018	2.17E-06	0.007411804
T	G	0.3569	0.079	0.0295	3018	6.31E-06	0.006732494
A	T	-0.1416	0.031	0.2169	3018	7.06E-06	0.006659414
C	G	-0.1231	0.028	0.6678	3018	8.89E-06	0.006515379
T	C	0.1995	0.04	0.1209	3018	5.05E-07	0.008331137
T	A	0.4559	0.103	0.0178	3041	9.40E-06	0.006438381
G	C	-0.1352	0.03	0.271	3041	5.53E-06	0.006763699
A	G	0.2764	0.059	0.0524	3041	2.63E-06	0.007230826
C	T	0.1271	0.029	0.329	3041	9.97E-06	0.006399084
T	C	-0.176	0.039	0.8576	3041	5.88E-06	0.006727623
A	G	0.1248	0.026	0.4513	3041	2.24E-06	0.007333552
A	T	0.1287	0.027	0.4742	3041	1.67E-06	0.007515311
G	A	-0.2473	0.051	0.0702	3041	1.43E-06	0.007613298
C	T	-0.8168	0.173	0.0059	3041	2.31E-06	0.007310497
G	C	-0.2995	0.067	0.0406	3041	8.15E-06	0.006524176
T	A	-0.1541	0.034	0.1981	3041	5.29E-06	0.00679685
A	T	0.807	0.176	0.0064	3041	4.49E-06	0.006897248
A	G	-0.2955	0.065	0.0449	3041	6.49E-06	0.006666614
C	T	0.3488	0.074	0.0344	3041	2.81E-06	0.007189105
G	T	0.1523	0.032	0.2292	3041	1.40E-06	0.007623651
T	A	0.1333	0.027	0.479	3041	6.66E-07	0.008093611
G	T	0.1258	0.028	0.632	3019	7.66E-06	0.006613647
G	C	1.163	0.251	0.0035	3019	3.61E-06	0.007089101
T	C	-0.1271	0.028	0.3741	3019	5.89E-06	0.006774072
T	C	-0.198	0.044	0.1017	3019	7.14E-06	0.006656815
G	T	0.1466	0.031	0.7464	3019	1.80E-06	0.007520821
T	C	1.839	0.403	0.0012	3019	5.23E-06	0.006853605
T	C	-0.1285	0.027	0.5711	3019	2.31E-06	0.007370731
C	T	-0.502	0.106	0.0187	3019	2.23E-06	0.007388085
T	C	0.5811	0.126	0.0124	3019	3.84E-06	0.007040297

T	G	-0.5072	0.106	0.0157	3019	1.71E-06	0.007554931
A	G	0.1631	0.034	0.19	3019	1.92E-06	0.007481441
A	G	-0.3754	0.079	0.0305	3019	1.92E-06	0.007484008
T	C	-0.1584	0.031	0.2433	3019	4.47E-07	0.008406492
T	G	0.1863	0.04	0.1179	3019	4.00E-06	0.007018548
T	C	-0.4377	0.097	0.0197	3019	7.08E-06	0.006659654
A	G	-0.4097	0.091	0.0209	3019	6.97E-06	0.006669294
A	T	-0.3853	0.086	0.0268	3019	8.47E-06	0.006547202
T	C	-0.1651	0.037	0.1423	3019	9.13E-06	0.006503001
T	C	0.189	0.04	0.1211	3019	2.68E-06	0.007275609
T	C	0.2544	0.055	0.0661	3042	4.46E-06	0.006901539
G	A	0.3732	0.081	0.0293	3042	4.75E-06	0.006859188
T	C	-0.1582	0.027	0.5717	3042	8.47E-09	0.010847581
T	C	0.6697	0.126	0.0125	3042	1.26E-07	0.009143615
A	G	3.194	0.717	0.0003	3042	8.76E-06	0.006475711
A	G	-0.2309	0.045	0.0971	3042	3.82E-07	0.008438771
T	A	0.1238	0.027	0.4196	3042	5.39E-06	0.006783696
G	A	0.4043	0.087	0.0251	3042	3.79E-06	0.007001136
A	C	-0.1459	0.031	0.7429	3042	2.32E-06	0.007303635
T	C	-0.1412	0.032	0.2431	3042	8.47E-06	0.006496178
T	A	0.304	0.065	0.9564	3042	2.80E-06	0.007191833
A	G	0.6992	0.155	0.0074	3042	6.47E-06	0.006670469
G	A	-0.3015	0.065	0.044	3042	3.21E-06	0.007105337
A	G	-0.447	0.097	0.0189	3042	3.95E-06	0.006975301
A	G	-0.1267	0.027	0.3922	3042	3.59E-06	0.007035913
T	C	-0.1798	0.038	0.1418	3042	1.75E-06	0.007488564
C	T	0.1304	0.029	0.2936	3042	7.41E-06	0.006580161
G	A	-0.4681	0.104	0.0168	3042	7.46E-06	0.00657784
A	G	0.8749	0.184	0.005	3387	2.15E-06	0.006609536
T	C	-0.2265	0.032	0.2083	3387	2.75E-12	0.014319287
G	A	0.6546	0.028	0.2449	3387	2.29E-112	0.13920411
A	G	0.319	0.063	0.0437	3387	3.69E-07	0.007603704
C	T	0.1529	0.026	0.4349	3387	5.43E-09	0.010000543
C	T	0.7935	0.163	0.0061	3387	1.25E-06	0.006914507
C	T	-0.2007	0.043	0.0977	3387	3.18E-06	0.006387892
A	C	0.5531	0.117	0.012	3387	2.27E-06	0.006577193
T	C	-0.5608	0.124	0.0118	3387	6.54E-06	0.005983444
G	A	-0.1157	0.026	0.4244	3387	7.97E-06	0.005870857
T	C	-0.4793	0.107	0.0136	3387	6.98E-06	0.005944442
A	G	0.574	0.127	0.0106	3387	5.91E-06	0.006042205
G	C	-0.8214	0.182	0.0049	3387	6.25E-06	0.006010667
A	G	0.1205	0.025	0.4846	3387	1.81E-06	0.006705572
T	C	0.1297	0.028	0.2938	3387	2.61E-06	0.006501131
T	C	-1.325	0.291	0.0018	3387	5.29E-06	0.006100533
C	T	-0.1438	0.032	0.8078	3387	8.21E-06	0.005857459
A	T	-0.1158	0.026	0.428	3387	7.76E-06	0.00589
G	A	0.3915	0.088	0.0214	3387	9.55E-06	0.005771801
T	C	-0.1238	0.028	0.3106	3387	7.34E-06	0.005917997

T	A	0.1978	0.045	0.9117	3387	9.85E-06	0.005750585
A	G	-1.922	0.43	0.0009	3387	7.94E-06	0.005874948
G	A	-0.1264	0.027	0.6811	3387	3.17E-06	0.006391414
G	C	0.9423	0.203	0.0038	3387	3.64E-06	0.006309095
T	C	0.6438	0.129	0.0094	3410	5.78E-07	0.00729601
A	G	0.2526	0.051	0.0688	3410	9.22E-07	0.00703812
G	A	0.6258	0.029	0.2447	3410	8.46E-99	0.12236739
A	G	-0.2585	0.04	0.1218	3410	7.62E-11	0.012341927
T	C	-0.2154	0.046	0.0839	3410	3.48E-06	0.006296249
C	T	0.7369	0.165	0.006	3410	8.07E-06	0.005829205
A	G	-0.1227	0.026	0.4757	3410	2.15E-06	0.006568822
T	C	-0.2542	0.056	0.0531	3410	5.90E-06	0.006002
C	G	-0.1682	0.037	0.1276	3410	5.00E-06	0.006095632
T	G	0.2972	0.06	0.0501	3410	7.27E-07	0.007174598
T	C	0.3577	0.076	0.0292	3410	2.93E-06	0.006388912
G	A	0.1185	0.027	0.3971	3410	9.16E-06	0.005751822
C	G	-0.6159	0.131	0.0097	3410	2.44E-06	0.006489592
C	T	0.1584	0.03	0.2645	3410	9.42E-08	0.008322421
T	C	-0.3324	0.07	0.0362	3410	2.39E-06	0.006504373
G	T	-0.1378	0.028	0.2875	3410	9.67E-07	0.007012833
C	T	1.198	0.262	0.0023	3410	5.09E-06	0.006084757
T	C	-0.2037	0.046	0.0855	3410	8.53E-06	0.005797627
A	G	-0.3648	0.079	0.0265	3410	4.40E-06	0.006164592
G	T	0.1193	0.026	0.5802	3410	4.35E-06	0.006174003
T	G	0.1266	0.026	0.4535	3410	1.03E-06	0.006973982
G	A	-0.1364	0.03	0.7411	3410	5.31E-06	0.00606179
T	C	0.2116	0.047	0.0842	3410	6.22E-06	0.005971898
T	C	-0.223	0.049	0.0783	3410	5.63E-06	0.006027384
T	C	0.5111	0.114	0.0136	3410	7.71E-06	0.005849761
T	A	-0.2695	0.06	0.0466	3410	6.58E-06	0.005938575
A	G	-0.5066	0.11	0.0136	3410	4.44E-06	0.006159275
T	C	-0.1675	0.036	0.1474	3410	3.35E-06	0.006318899
T	C	-0.1841	0.041	0.0957	3374	8.41E-06	0.005866093
G	A	0.1169	0.024	0.4784	3374	1.24E-06	0.006948078
A	G	0.1321	0.026	0.7121	3374	5.57E-07	0.007405099
G	T	-0.6432	0.142	0.0071	3374	6.51E-06	0.006010475
T	C	-0.7983	0.166	0.0052	3374	1.59E-06	0.006807761
C	T	0.1937	0.026	0.3244	3374	2.12E-13	0.015846246
T	C	-1.742	0.387	0.0012	3374	6.78E-06	0.00598474
C	T	0.2251	0.047	0.071	3374	1.79E-06	0.006735456
A	T	0.1163	0.024	0.4813	3374	1.05E-06	0.007033316
T	C	0.5481	0.103	0.0133	3374	1.25E-07	0.00825908
G	C	-1.824	0.396	0.0009	3374	4.17E-06	0.006261296
C	T	-0.2167	0.048	0.0702	3374	5.69E-06	0.006084846
T	C	-0.3111	0.068	0.0325	3374	4.42E-06	0.00622699
C	G	-0.1971	0.028	0.2619	3374	9.51E-13	0.014975399
A	T	2.012	0.415	0.0007	3374	1.32E-06	0.006911687
C	A	0.1139	0.024	0.4092	3374	3.14E-06	0.006422153

T	C	-0.1453	0.031	0.818	3374	2.82E-06	0.006481562
G	C	-0.4462	0.1	0.0141	3374	8.93E-06	0.005831392
C	T	1.044	0.226	0.0028	3374	3.81E-06	0.006312663
C	T	1.338	0.295	0.0015	3374	5.87E-06	0.006068323
C	G	0.3807	0.084	0.0202	3374	5.74E-06	0.006079729
C	T	0.4301	0.091	0.0169	3374	2.54E-06	0.006541507
G	T	-0.2103	0.047	0.0637	3374	7.32E-06	0.005938992
T	C	0.1614	0.033	0.1457	3374	1.42E-06	0.006869346
C	T	0.3131	0.069	0.0313	3374	5.55E-06	0.006097272
C	A	-0.7037	0.155	0.0056	3374	5.85E-06	0.006071858
G	T	0.3127	0.069	0.0319	3374	6.69E-06	0.005994903
A	G	0.152	0.034	0.1432	3374	8.07E-06	0.005888698
T	C	-1.205	0.234	0.0027	3374	2.81E-07	0.007785043
C	G	-1.178	0.262	0.0019	3374	7.07E-06	0.005960438
G	A	0.1138	0.024	0.4413	3374	2.73E-06	0.006500679
A	G	0.6722	0.127	0.0096	3374	1.28E-07	0.008234806
A	G	0.1199	0.026	0.5697	3374	3.09E-06	0.006424545
T	C	0.5889	0.028	0.2358	3374	1.20E-89	0.112611337
C	T	0.3483	0.057	0.0532	3374	9.74E-10	0.011017948
T	C	0.1591	0.036	0.1464	3374	8.00E-06	0.005894676
T	A	0.1983	0.044	0.0917	3374	5.88E-06	0.006068658
T	C	0.1262	0.028	0.3254	3374	5.77E-06	0.006075054
C	T	-0.195	0.043	0.098	3374	4.68E-06	0.006194965
C	G	0.1158	0.025	0.4502	3374	4.08E-06	0.006268931
A	G	0.1846	0.041	0.1101	3374	5.74E-06	0.006081009
A	T	-0.3782	0.082	0.0258	3374	3.84E-06	0.006304953
T	C	0.3124	0.067	0.0362	3374	3.75E-06	0.006319585
A	G	0.5945	0.125	0.0107	3374	1.98E-06	0.006680645
A	G	0.1328	0.029	0.7432	3374	4.61E-06	0.006206551
G	C	-0.7956	0.18	0.0049	3374	9.74E-06	0.005782468
T	G	-0.3287	0.072	0.0313	3374	5.95E-06	0.00605869
A	G	0.1282	0.027	0.2964	3374	2.45E-06	0.006560139
A	G	0.125	0.025	0.4843	3374	5.48E-07	0.007407954
T	C	0.1545	0.034	0.1679	3374	4.40E-06	0.006227594
C	G	-0.1534	0.032	0.3072	3374	1.89E-06	0.006706432
A	G	-0.1209	0.027	0.3103	3374	9.58E-06	0.005787593
G	C	-0.252	0.054	0.0603	3374	3.43E-06	0.006368586
G	C	0.9392	0.2	0.0039	3374	2.78E-06	0.006487097
A	G	-0.3652	0.039	0.1219	3387	1.18E-20	0.025311637
T	C	0.7418	0.128	0.0094	3387	8.28E-09	0.009758199
G	A	0.7524	0.027	0.2449	3387	1.40E-150	0.182634912
A	G	0.3283	0.063	0.0437	3387	1.83E-07	0.008001657
A	G	0.3586	0.051	0.0686	3387	2.05E-12	0.014493276
T	A	0.2057	0.044	0.0921	3387	3.38E-06	0.006351047
C	G	-1.139	0.245	0.0024	3387	3.39E-06	0.006351005
G	T	0.1447	0.032	0.8091	3387	8.68E-06	0.005825757
C	T	-4.152	0.876	0.0001	3387	2.24E-06	0.006586023
A	C	-1.176	0.258	0.0025	3387	5.12E-06	0.006120385

A	G	0.2383	0.054	0.0602	3387	9.91E-06	0.005748542
T	C	0.2767	0.057	0.0512	3387	1.09E-06	0.006989571
C	T	-0.36	0.076	0.0272	3387	1.94E-06	0.00666792
A	G	0.1337	0.028	0.287	3387	1.87E-06	0.006691538
A	G	0.128	0.027	0.3276	3387	3.04E-06	0.006411277
A	C	-2.086	0.463	0.0009	3387	6.98E-06	0.005947176
A	G	0.3107	0.07	0.0353	3387	9.94E-06	0.005748646
T	G	-0.2246	0.05	0.0697	3387	7.70E-06	0.005891727
G	A	-0.1219	0.027	0.6811	3387	7.55E-06	0.005907987
A	G	-0.1257	0.027	0.435	3410	2.34E-06	0.006515792
T	C	0.6557	0.13	0.0094	3410	5.15E-07	0.007360248
A	G	-0.3829	0.04	0.1218	3410	1.43E-21	0.026335007
G	A	0.4142	0.075	0.0326	3410	3.97E-08	0.008806659
A	G	-0.2072	0.037	0.8488	3410	2.54E-08	0.009059222
G	A	0.2373	0.052	0.0679	3410	5.64E-06	0.006028467
A	G	-0.137	0.029	0.7375	3410	3.25E-06	0.006331826
T	C	0.7548	0.028	0.2441	3410	2.11E-144	0.174741458
A	G	-0.3312	0.073	0.0334	3410	6.81E-06	0.005922543
T	C	-0.855	0.188	0.0059	3410	5.73E-06	0.00601614
C	A	0.1381	0.027	0.3707	3410	3.32E-07	0.007613534
T	C	-0.9935	0.211	0.0031	3410	2.68E-06	0.006441335
G	T	1.355	0.292	0.0023	3410	3.50E-06	0.006287991
T	G	-1.697	0.378	0.0012	3410	7.60E-06	0.005863448
T	C	-1.039	0.231	0.0031	3410	7.06E-06	0.005897716
G	A	0.2232	0.049	0.0783	3410	4.41E-06	0.0061624
T	A	0.2575	0.058	0.0516	3410	8.35E-06	0.005808567
C	T	0.4062	0.091	0.021	3410	7.61E-06	0.005858969
C	T	-0.3592	0.077	0.0271	3410	2.88E-06	0.006402229
G	A	0.1217	0.027	0.3752	3410	6.40E-06	0.005953342
A	G	0.1266	0.028	0.3702	3410	4.35E-06	0.006167785
G	C	-1.426	0.308	0.0019	3410	3.95E-06	0.006230767
T	C	0.1625	0.035	0.1736	3410	3.13E-06	0.006353685
C	T	-0.1368	0.031	0.7776	3410	8.77E-06	0.005777981
A	G	0.2153	0.046	0.0884	3387	2.92E-06	0.006440148
T	C	0.1639	0.035	0.1673	3387	2.45E-06	0.006540092
A	G	0.3116	0.064	0.0437	3387	1.30E-06	0.006892226
T	C	-0.5561	0.117	0.0124	3387	2.28E-06	0.006580924
A	G	0.3667	0.052	0.0686	3387	2.25E-12	0.014442656
T	C	0.1405	0.03	0.2499	3387	2.09E-06	0.006625848
G	A	0.8088	0.028	0.2449	3387	9.71E-166	0.199365861
A	G	-0.6058	0.129	0.0103	3387	2.51E-06	0.006519234
C	T	-0.3862	0.085	0.023	3387	6.16E-06	0.006017166
A	G	-0.355	0.04	0.1219	3387	1.25E-18	0.022660275
T	C	0.7391	0.13	0.0094	3387	1.55E-08	0.009395852
T	A	-0.3927	0.083	0.0245	3387	2.50E-06	0.00652671
G	T	-0.1444	0.032	0.2149	3387	7.18E-06	0.005935443
T	C	-0.6969	0.144	0.0075	3387	1.45E-06	0.00682991
A	T	-0.1177	0.026	0.4074	3387	8.53E-06	0.005834299

T	C	0.1278	0.028	0.2938	3387	5.94E-06	0.006035806
A	G	-0.2492	0.055	0.0576	3387	6.53E-06	0.005985634
A	G	0.1673	0.038	0.1333	3387	9.41E-06	0.005777596
C	T	-1.333	0.297	0.0021	3387	7.24E-06	0.005928169
G	T	-0.7215	0.157	0.0066	3387	4.53E-06	0.006188836
T	C	-2.409	0.511	0.0006	3387	2.48E-06	0.006529079
G	C	-0.2163	0.046	0.0865	3387	2.26E-06	0.006584868
T	C	-0.3544	0.078	0.0282	3387	5.47E-06	0.006084538
T	C	0.6732	0.131	0.0094	3410	2.80E-07	0.007708284
T	C	0.1357	0.03	0.2501	3410	4.64E-06	0.006133902
A	G	0.3612	0.052	0.0688	3410	4.90E-12	0.013899111
C	T	-0.3803	0.086	0.0229	3410	8.94E-06	0.005768377
A	G	-0.6667	0.129	0.0103	3410	2.34E-07	0.007808096
A	G	-0.3508	0.04	0.1218	3410	3.34E-18	0.021950127
T	C	-0.6222	0.118	0.0123	3410	1.30E-07	0.008142175
G	A	0.8089	0.028	0.2447	3410	4.22E-165	0.197530075
C	A	0.1227	0.026	0.4919	3410	2.01E-06	0.006604321
T	A	-1.864	0.391	0.0009	3410	1.95E-06	0.006620626
G	A	-0.1204	0.026	0.4776	3410	4.07E-06	0.006211228
A	G	-0.3741	0.082	0.026	3410	4.63E-06	0.006134894
T	C	0.2353	0.053	0.0633	3410	9.64E-06	0.005727557
C	A	-0.443	0.097	0.0177	3410	4.68E-06	0.006130812
C	A	1.672	0.372	0.001	3410	7.10E-06	0.005895646
G	A	-0.225	0.049	0.0767	3410	5.26E-06	0.006063829
C	G	-2.6	0.556	0.0004	3410	3.08E-06	0.006362766
A	C	-2.515	0.516	0.0007	3410	1.12E-06	0.006926419
T	C	-1.586	0.347	0.0015	3410	5.19E-06	0.006074996
A	C	0.5691	0.119	0.0128	3410	1.67E-06	0.006707038
T	C	-2.273	0.512	0.0006	3410	9.34E-06	0.005744246
A	C	-0.5139	0.11	0.0141	3410	3.13E-06	0.006359846
G	A	0.2938	0.065	0.0428	3410	6.31E-06	0.005961106
G	A	0.2261	0.05	0.0755	3410	6.10E-06	0.005982266
C	T	-0.1223	0.026	0.3543	3374	2.87E-06	0.006475469
C	T	-0.117	0.025	0.5529	3374	3.14E-06	0.006419006
C	T	0.3946	0.08	0.0252	3374	7.45E-07	0.007236295
G	T	0.3098	0.068	0.0368	3374	5.42E-06	0.006112368
G	T	-0.1133	0.025	0.5221	3374	5.53E-06	0.006103879
G	A	-0.1473	0.028	0.266	3374	2.17E-07	0.007943232
T	A	-0.3011	0.066	0.0375	3374	4.44E-06	0.006222293
A	G	-1.091	0.241	0.0028	3374	6.19E-06	0.006032291
G	A	0.2575	0.055	0.0578	3374	2.52E-06	0.00654417
G	A	-0.2618	0.051	0.0649	3374	2.84E-07	0.007779769
G	T	0.1234	0.027	0.3251	3374	4.67E-06	0.006198411
A	G	0.7025	0.151	0.0067	3374	3.17E-06	0.006416217
C	A	0.2639	0.055	0.0587	3374	1.53E-06	0.006826494
C	T	0.9624	0.198	0.0037	3374	1.19E-06	0.006967501
G	A	-0.2713	0.059	0.0491	3374	4.77E-06	0.006184013
T	C	0.2729	0.058	0.0505	3374	2.36E-06	0.006581752

C	A	0.1603	0.033	0.1768	3374	1.29E-06	0.006924071
G	C	-0.4937	0.111	0.0123	3374	8.31E-06	0.005871021
T	C	-0.2171	0.049	0.0702	3374	8.45E-06	0.005862708
G	C	0.3632	0.08	0.0252	3374	5.90E-06	0.006065828
T	C	-0.2739	0.029	0.2345	3410	1.98E-20	0.024853969
T	C	0.1173	0.026	0.3594	3410	9.19E-06	0.005756079
T	C	-0.1537	0.031	0.2186	3410	6.63E-07	0.007221937
A	G	-1.201	0.271	0.0021	3410	9.94E-06	0.005714041
T	C	0.1318	0.027	0.2944	3410	9.80E-07	0.007006282
A	G	-0.3759	0.079	0.0265	3410	1.77E-06	0.006674377
C	T	0.1569	0.031	0.2025	3410	3.85E-07	0.007533187
G	T	0.3227	0.068	0.0364	3410	2.34E-06	0.006517082
T	C	-0.1504	0.032	0.1789	3410	3.81E-06	0.006244839
A	G	-0.1275	0.027	0.3198	3410	1.69E-06	0.006702478
C	G	-0.9025	0.182	0.0045	3410	7.20E-07	0.007175051
C	T	-0.1321	0.027	0.3085	3410	1.36E-06	0.00682449
A	G	-1.154	0.243	0.0028	3410	2.17E-06	0.006559518
C	T	-0.186	0.041	0.1062	3410	7.12E-06	0.005892962
G	A	0.277	0.055	0.0578	3410	3.93E-07	0.00751582
G	C	0.2457	0.054	0.0588	3410	6.55E-06	0.005942244
T	C	-0.4408	0.097	0.0172	3410	5.28E-06	0.006064197
G	A	-0.2768	0.051	0.0648	3410	5.54E-08	0.008621387
A	G	-0.2631	0.055	0.0554	3410	1.45E-06	0.00678544
G	C	0.2649	0.058	0.0501	3410	4.38E-06	0.006164235
G	T	0.131	0.027	0.326	3410	1.07E-06	0.006958029
C	G	-0.114	0.026	0.3865	3410	9.75E-06	0.005719357
C	A	0.2436	0.055	0.0587	3410	8.61E-06	0.005788696
C	T	0.9606	0.201	0.0037	3410	1.73E-06	0.00668634
A	T	-0.1122	0.025	0.4499	3410	5.78E-06	0.006014747
C	A	0.1585	0.033	0.1771	3410	1.72E-06	0.006687427
C	T	-0.2097	0.045	0.0845	3410	3.18E-06	0.006344715
G	C	-0.4931	0.111	0.0125	3410	8.45E-06	0.00579537
C	G	-0.8171	0.184	0.0047	3410	8.85E-06	0.00577478
T	C	0.1192	0.026	0.6037	3410	3.67E-06	0.00625935
A	G	-0.4066	0.04	0.1219	3387	5.49E-24	0.029675827
G	A	0.8094	0.028	0.2449	3387	9.84E-166	0.199373424
T	A	0.2161	0.045	0.0921	3387	1.84E-06	0.00669459
T	C	0.8211	0.13	0.0094	3387	3.34E-10	0.011588464
A	G	0.401	0.052	0.0686	3387	1.66E-14	0.01724168
A	G	0.3306	0.064	0.0437	3387	2.86E-07	0.007746868
T	A	-0.2364	0.053	0.0633	3387	9.83E-06	0.005755105
T	C	-0.1908	0.04	0.1233	3387	1.77E-06	0.006719529
T	C	0.1304	0.027	0.3691	3387	1.62E-06	0.006769713
T	C	0.2902	0.058	0.0512	3387	5.39E-07	0.007392682
C	T	0.1346	0.028	0.2917	3387	2.29E-06	0.006569814
T	C	-0.314	0.07	0.0371	3387	7.12E-06	0.005936066
T	C	-0.6001	0.135	0.0084	3387	9.04E-06	0.005800137
A	G	0.1281	0.028	0.3276	3387	5.04E-06	0.006128681

T	G	-0.2301	0.051	0.0697	3387	7.06E-06	0.005941669
T	C	0.1694	0.038	0.1305	3387	8.04E-06	0.005869956
C	T	-0.1397	0.031	0.7772	3387	6.08E-06	0.006025675
A	G	-0.1661	0.037	0.8488	3410	6.56E-06	0.00593885
A	G	-0.4263	0.039	0.1218	3410	5.29E-27	0.033387636
A	G	0.3059	0.063	0.0437	3410	1.48E-06	0.006772158
C	G	0.2273	0.046	0.0846	3410	7.51E-07	0.007155616
T	C	0.7929	0.129	0.0094	3410	1.01E-09	0.010890759
T	A	0.1995	0.045	0.0919	3410	8.32E-06	0.005810053
G	A	0.8232	0.027	0.2447	3410	8.53E-178	0.211121879
A	G	0.4169	0.051	0.0688	3410	5.86E-16	0.019028674
T	A	-0.2427	0.053	0.0632	3410	4.04E-06	0.006209247
A	G	-1.817	0.407	0.001	3410	8.18E-06	0.005816479
G	A	-1.124	0.24	0.0026	3410	2.84E-06	0.006401633
G	A	0.1393	0.03	0.2437	3410	3.94E-06	0.006225143
G	A	-1.019	0.217	0.0038	3410	2.73E-06	0.006430918
C	A	0.1382	0.027	0.3707	3410	2.52E-07	0.007760789
G	T	1.34	0.289	0.0023	3410	3.68E-06	0.00626513
C	T	0.2018	0.045	0.9157	3410	9.14E-06	0.00575555
T	C	-0.748	0.168	0.0056	3410	9.20E-06	0.005752533
C	T	-0.3397	0.076	0.0271	3410	8.41E-06	0.00580341
T	C	0.273	0.057	0.0512	3410	1.82E-06	0.006654187
C	T	-0.1337	0.03	0.7405	3410	8.52E-06	0.005798552
C	A	-0.1278	0.029	0.7111	3410	7.75E-06	0.005849998
G	C	0.2287	0.052	0.0682	3410	9.51E-06	0.005736587
T	C	-0.1776	0.04	0.1226	3410	7.45E-06	0.005869799
T	C	1.099	0.239	0.0031	3410	4.40E-06	0.00616768
G	A	0.4063	0.091	0.0198	3410	7.40E-06	0.005874721
C	T	-0.1514	0.03	0.7776	3410	6.95E-07	0.007192874
C	T	-0.1397	0.03	0.2011	3374	3.76E-06	0.006314603
G	A	-0.1861	0.041	0.0947	3374	6.89E-06	0.005979059
C	T	-0.129	0.028	0.2945	3374	2.87E-06	0.006465541
A	G	0.3356	0.062	0.0413	3374	5.39E-08	0.008725959
C	A	0.6561	0.147	0.007	3374	7.90E-06	0.00590142
A	G	0.111	0.024	0.525	3374	5.43E-06	0.006116203
G	A	-0.2628	0.043	0.0865	3374	1.38E-09	0.010824474
C	T	-0.2794	0.025	0.3435	3374	5.62E-28	0.035018999
C	T	-0.1252	0.025	0.6057	3374	5.45E-07	0.007407869
T	G	0.7317	0.156	0.0061	3374	2.87E-06	0.006469881
T	A	-0.1362	0.031	0.2024	3374	8.37E-06	0.00586792
A	C	0.252	0.056	0.0508	3374	7.45E-06	0.005932228
A	G	-0.1501	0.033	0.1635	3374	5.98E-06	0.006054254
A	G	-0.1835	0.039	0.1119	3374	2.53E-06	0.006545299
T	C	0.3081	0.069	0.0325	3374	8.88E-06	0.005832548
A	G	0.2985	0.062	0.0422	3374	1.60E-06	0.006801372
C	G	0.5376	0.115	0.0111	3374	3.19E-06	0.006413193
C	T	0.1874	0.04	0.1046	3374	2.44E-06	0.006560764
C	T	0.1364	0.028	0.2503	3374	1.40E-06	0.006876586

A	G	1.17	0.247	0.0022	3374	2.15E-06	0.006632887
A	G	0.1127	0.024	0.507	3374	3.16E-06	0.006413193
T	G	0.1321	0.03	0.2107	3374	8.01E-06	0.00589214
A	T	0.209	0.046	0.0772	3374	4.71E-06	0.006192998
T	C	0.8677	0.191	0.004	3374	5.63E-06	0.00609233
C	T	-0.1401	0.031	0.2009	3410	5.46E-06	0.00604275
T	C	-0.3011	0.029	0.2352	3410	6.70E-25	0.030665111
A	G	0.2959	0.063	0.0411	3410	2.74E-06	0.006427675
C	T	-0.3282	0.072	0.0324	3410	6.11E-06	0.005980274
A	G	-0.2638	0.059	0.0462	3410	7.97E-06	0.005830407
C	A	0.6724	0.148	0.0072	3410	5.62E-06	0.006024767
A	C	0.1816	0.039	0.1132	3410	3.31E-06	0.006321451
T	C	-0.2731	0.061	0.0456	3410	8.53E-06	0.005792556
C	T	-0.3029	0.026	0.344	3410	1.35E-31	0.039317684
G	A	-0.2664	0.044	0.0867	3410	1.72E-09	0.010583254
A	G	1.06	0.232	0.0029	3410	5.07E-06	0.006084583
T	C	0.1261	0.028	0.7415	3410	6.44E-06	0.005950645
A	G	-0.1787	0.04	0.1116	3410	6.77E-06	0.005924434
T	A	-0.1115	0.025	0.4751	3410	9.15E-06	0.00575363
A	G	0.7951	0.178	0.005	3410	8.29E-06	0.005810726
A	G	1.182	0.245	0.0025	3410	1.42E-06	0.006795965
A	G	0.1292	0.028	0.2843	3410	3.13E-06	0.006353081
A	G	1.949	0.408	0.0009	3410	1.86E-06	0.006647409
C	T	0.1095	0.025	0.4141	3410	9.80E-06	0.005716574
G	A	0.1206	0.026	0.3408	3410	4.62E-06	0.006137842
A	G	0.3417	0.064	0.0437	3387	1.10E-07	0.008291885
A	G	-0.4509	0.04	0.1219	3387	3.72E-29	0.036422946
A	G	-0.1286	0.027	0.6884	3387	2.71E-06	0.006480536
A	G	0.425	0.052	0.0686	3387	3.96E-16	0.01937731
G	T	0.1553	0.035	0.1676	3387	8.03E-06	0.00586561
A	G	-0.6066	0.128	0.0103	3387	2.38E-06	0.006556611
G	A	0.9207	0.027	0.2449	3387	1.00E-200	0.256845871
T	C	0.1319	0.03	0.2499	3387	8.54E-06	0.005836278
C	A	-0.4223	0.082	0.0279	3387	3.13E-07	0.007698868
T	C	0.7645	0.13	0.0094	3387	4.22E-09	0.010138295
T	A	-0.2434	0.053	0.0633	3387	5.34E-06	0.006096599
T	C	-0.4935	0.11	0.0136	3387	6.81E-06	0.005961207
A	G	-0.1544	0.032	0.2046	3387	1.06E-06	0.007008109
A	T	-0.4201	0.095	0.0186	3387	9.12E-06	0.005796081
T	C	-0.691	0.144	0.0075	3387	1.69E-06	0.00674333
A	G	-1.385	0.31	0.0016	3387	8.27E-06	0.005855049
A	C	-2.339	0.511	0.0007	3387	4.81E-06	0.006155058
T	C	-0.4885	0.11	0.0133	3387	9.19E-06	0.005789047
G	A	0.2686	0.055	0.0616	3387	8.75E-07	0.007117741
T	C	-0.3134	0.07	0.0371	3387	7.35E-06	0.005918591
A	G	0.1435	0.031	0.2166	3387	4.46E-06	0.006195064
A	G	-0.1283	0.027	0.6886	3410	2.49E-06	0.006487151
A	G	0.3215	0.064	0.0437	3410	4.97E-07	0.007384801

A	G	0.4245	0.052	0.0688	3410	3.16E-16	0.019372604
A	G	-0.6516	0.128	0.0103	3410	3.54E-07	0.007577442
A	G	-0.1706	0.037	0.8488	3410	4.48E-06	0.006156104
G	A	0.5118	0.112	0.017	3410	5.00E-06	0.006097193
A	G	-0.4505	0.04	0.1218	3410	2.65E-29	0.036370198
G	A	0.929	0.027	0.2447	3410	1.00E-200	0.260561178
C	A	-0.4247	0.082	0.028	3410	2.31E-07	0.007816462
T	C	0.71	0.129	0.0094	3410	3.88E-08	0.008818801
T	A	-0.2429	0.053	0.0632	3410	5.00E-06	0.006094382
A	G	-0.1411	0.031	0.2048	3410	7.10E-06	0.005901697
A	C	-2.506	0.506	0.0007	3410	7.49E-07	0.007152799
T	C	0.5823	0.13	0.0101	3410	7.56E-06	0.005858268
T	G	0.1382	0.031	0.2557	3410	6.85E-06	0.005915268
G	A	0.244	0.054	0.0616	3410	6.66E-06	0.005929905
T	C	0.1553	0.033	0.1861	3410	3.19E-06	0.006341588
G	C	0.4006	0.09	0.0218	3410	9.03E-06	0.005763782
A	G	0.1195	0.026	0.5667	3410	4.38E-06	0.00617091
C	T	-0.1396	0.031	0.7776	3410	5.18E-06	0.006070342
T	G	-0.2262	0.049	0.0707	3410	3.86E-06	0.006238396
G	A	-0.3966	0.073	0.0326	3410	5.11E-08	0.008666014
A	G	0.2502	0.039	0.1218	3410	1.12E-10	0.01213374
T	C	-0.5887	0.028	0.2441	3410	1.97E-92	0.114829817
C	A	0.1305	0.025	0.4749	3410	2.52E-07	0.007766296
C	T	0.1236	0.028	0.3238	3410	8.76E-06	0.005775839
G	A	0.2108	0.046	0.085	3410	3.78E-06	0.006249706
T	G	-0.2775	0.059	0.0501	3410	2.21E-06	0.006546568
C	G	0.1657	0.036	0.1276	3410	4.48E-06	0.00615062
C	T	0.184	0.042	0.9029	3410	1.00E-05	0.00570986
G	A	-0.1221	0.026	0.3971	3410	2.91E-06	0.006391608
C	T	-0.1835	0.029	0.2645	3410	2.25E-10	0.011724762
C	G	0.6212	0.127	0.0097	3410	1.14E-06	0.006923922
A	G	-0.2853	0.062	0.0422	3410	4.66E-06	0.006133889
A	G	-0.2618	0.059	0.0477	3410	9.85E-06	0.005715835
T	G	-0.1258	0.025	0.4535	3410	6.93E-07	0.007198274
T	C	-1.939	0.416	0.0009	3410	3.26E-06	0.006327745
C	G	-0.868	0.19	0.0048	3410	5.31E-06	0.006057764
T	C	0.679	0.151	0.007	3410	7.05E-06	0.005902496
T	A	0.1222	0.027	0.677	3410	8.32E-06	0.005803317
G	A	0.1338	0.029	0.7411	3410	4.92E-06	0.006103016
T	C	-0.2075	0.046	0.0842	3410	6.00E-06	0.005991151
T	C	0.3577	0.079	0.0264	3410	6.78E-06	0.005922437
T	C	0.2104	0.047	0.0745	3410	7.24E-06	0.005884716
A	G	-0.1217	0.025	0.5792	3410	1.24E-06	0.006874097
C	T	0.1622	0.037	0.1367	3595	9.60E-06	0.005433435
C	T	-0.1231	0.026	0.3644	3595	2.85E-06	0.006075488
G	T	0.1374	0.031	0.7944	3595	8.53E-06	0.005501679
A	G	-1.812	0.392	0.0008	3595	3.85E-06	0.005917426
T	C	-0.5052	0.094	0.0185	3595	8.31E-08	0.007962297

T	C	-0.2782	0.062	0.0421	3595	7.75E-06	0.005546222
T	C	-0.1735	0.039	0.1139	3595	9.57E-06	0.005436146
T	C	0.2039	0.046	0.0771	3595	9.83E-06	0.005423933
T	C	-0.2295	0.037	0.2819	3595	3.91E-10	0.010836392
T	C	-0.3029	0.03	0.5808	3595	5.37E-23	0.026770418
G	A	-0.2475	0.032	0.4356	3595	1.68E-14	0.016237543
T	C	-0.1923	0.029	0.5013	3595	5.86E-11	0.011855989
C	G	0.3326	0.052	0.0861	3595	1.35E-10	0.011407499
T	C	0.2678	0.054	0.0633	3595	6.64E-07	0.006852608
T	A	0.1173	0.026	0.6394	3595	9.67E-06	0.005432796
C	G	-0.1283	0.028	0.2826	3595	4.20E-06	0.005872971
T	C	-0.5588	0.125	0.0103	3595	8.39E-06	0.005501938
T	A	0.5024	0.113	0.0129	3595	9.26E-06	0.005458809
T	A	-0.1366	0.027	0.531	3595	5.35E-07	0.006971812
C	T	0.5761	0.123	0.9897	3595	2.94E-06	0.006065192
C	T	-0.1742	0.037	0.1305	3595	2.68E-06	0.006108374
T	C	0.1843	0.041	0.1067	3595	5.65E-06	0.00571323
C	A	-0.3757	0.08	0.0243	3595	2.67E-06	0.006114137
T	A	-0.1324	0.025	0.4732	3595	1.62E-07	0.00760201
C	G	-0.7033	0.151	0.0065	3595	3.52E-06	0.005966663
T	C	-0.4683	0.095	0.0175	3595	8.93E-07	0.006695712
G	A	-0.2168	0.033	0.4356	3595	3.72E-11	0.012101349
T	C	-0.1829	0.037	0.2819	3595	1.07E-06	0.006601528
T	C	-0.2715	0.031	0.5793	3595	7.44E-18	0.020410602
T	C	-0.1611	0.03	0.5013	3595	6.34E-08	0.008106986
A	T	1.447	0.321	0.0017	3595	6.64E-06	0.005634523
A	G	0.1791	0.04	0.1097	3595	9.20E-06	0.005461204
A	G	0.2562	0.057	0.0482	3579	8.27E-06	0.005539404
T	C	0.1115	0.025	0.5617	3579	7.81E-06	0.005571382
T	C	-0.4684	0.094	0.0176	3579	5.98E-07	0.006938208
T	G	-0.9382	0.185	0.0047	3579	4.03E-07	0.007150046
A	C	0.3833	0.08	0.025	3579	1.75E-06	0.006365317
G	A	0.1903	0.041	0.1018	3579	4.21E-06	0.005900056
A	G	0.8519	0.189	0.0043	3579	6.48E-06	0.005668438
T	A	-0.2957	0.065	0.0379	3579	4.98E-06	0.005809505
A	G	-0.1649	0.03	0.5423	3579	2.66E-08	0.008608519
T	C	-0.1767	0.035	0.2812	3579	5.79E-07	0.006952357
C	T	0.2356	0.031	0.6292	3579	1.67E-14	0.016324868
T	C	-1.116	0.249	0.0022	3579	7.59E-06	0.005585785
T	C	0.1685	0.033	0.1692	3579	2.83E-07	0.007342013
C	A	1.226	0.269	0.0022	3579	5.31E-06	0.00577034
A	G	-0.1396	0.031	0.202	3579	5.62E-06	0.005747935
T	G	0.1193	0.027	0.2923	3579	8.23E-06	0.005539034
G	A	0.3959	0.088	0.9719	3579	7.00E-06	0.00562589
G	C	-0.1431	0.026	0.5222	3579	6.40E-08	0.008136408
A	G	-0.3396	0.073	0.0296	3579	3.72E-06	0.0059633
G	A	-1.644	0.34	0.0013	3579	1.34E-06	0.006505371
A	G	0.2123	0.045	0.0807	3579	2.28E-06	0.006221612

C	A	-1.803	0.38	0.001	3579	2.11E-06	0.006267232
C	G	-1.754	0.382	0.0011	3579	4.49E-06	0.005862355
G	C	-0.2801	0.059	0.0451	3579	2.03E-06	0.006285475
T	C	0.1874	0.039	0.1133	3579	2.15E-06	0.006259164
T	C	-0.3484	0.075	0.0323	3579	3.59E-06	0.005983716
C	T	1.241	0.268	0.002	3437	3.74E-06	0.006204626
T	C	-0.3267	0.067	0.0346	3437	1.32E-06	0.006783539
T	C	0.5648	0.124	0.0103	3437	5.34E-06	0.006009653
C	G	-0.1117	0.025	0.4693	3437	8.30E-06	0.005765554
G	A	-0.1134	0.025	0.6123	3437	8.31E-06	0.005765909
T	C	-0.307	0.067	0.0371	3437	4.58E-06	0.00609508
C	A	-0.1531	0.033	0.3251	3437	4.66E-06	0.006087049
A	G	0.3869	0.083	0.0253	3437	2.83E-06	0.006358283
A	C	0.1876	0.037	0.1484	3437	5.62E-07	0.007255776
T	G	0.1934	0.043	0.112	3437	7.27E-06	0.00583504
G	A	0.2422	0.034	0.1766	3437	1.11E-12	0.014634129
A	C	0.2173	0.039	0.1155	3437	3.34E-08	0.008834571
A	G	-0.1478	0.033	0.1785	3437	7.56E-06	0.005820007
C	G	-0.2858	0.054	0.0598	3437	1.52E-07	0.007989903
T	C	1.04	0.227	0.0031	3437	4.79E-06	0.006070035
T	A	-0.2227	0.049	0.0708	3437	5.48E-06	0.005995891
T	C	-0.1713	0.039	0.1251	3437	9.77E-06	0.005676941
T	C	0.2845	0.061	0.045	3437	3.38E-06	0.006260467
A	T	-0.1357	0.028	0.2784	3437	1.08E-06	0.006889704
G	A	0.1213	0.026	0.3688	3437	4.57E-06	0.00609108
T	C	-1.849	0.39	0.0009	3437	2.15E-06	0.006513901
A	G	0.3211	0.065	0.0412	3437	7.22E-07	0.007119639
A	G	0.1335	0.029	0.262	3437	6.11E-06	0.005935228
T	G	-0.1628	0.034	0.4342	3437	1.42E-06	0.006744202
T	C	0.1181	0.026	0.4588	3437	4.56E-06	0.006097086
T	C	-1.047	0.235	0.0033	3437	8.49E-06	0.005751914
C	T	0.46	0.104	0.0148	3437	9.87E-06	0.005670682
A	C	0.1917	0.04	0.1155	3437	1.99E-06	0.00655656
G	A	0.2054	0.035	0.1766	3437	4.17E-09	0.01000004
G	A	0.1334	0.03	0.2629	3437	6.98E-06	0.005859003
G	C	-0.1567	0.035	0.1616	3437	7.89E-06	0.005794957
A	G	-0.2479	0.054	0.0634	3437	4.06E-06	0.006157707
T	A	-0.2506	0.05	0.0708	3437	5.54E-07	0.007264363
C	T	0.323	0.069	0.0358	3437	3.11E-06	0.006308018
G	C	-0.1191	0.026	0.4364	3437	3.89E-06	0.006190561
T	C	0.122	0.027	0.334	3437	8.89E-06	0.005730942
T	A	0.1394	0.03	0.7704	3437	4.62E-06	0.00608861
A	G	0.1425	0.032	0.2074	3437	6.67E-06	0.005881851
G	A	-0.3482	0.075	0.0248	3405	3.24E-06	0.006344068
C	T	0.8994	0.181	0.0043	3405	6.68E-07	0.00723105
A	G	1.651	0.211	0.0029	3405	6.94E-15	0.01764689
C	T	0.2177	0.023	0.4699	3405	3.10E-20	0.024624941
T	C	0.2841	0.064	0.0352	3405	9.93E-06	0.005718271

T	C	0.1892	0.038	0.1084	3405	8.66E-07	0.007082751
A	C	-0.2089	0.031	0.814	3405	1.33E-11	0.0133471
A	G	0.6777	0.149	0.0056	3405	5.62E-06	0.006038857
G	A	-0.1339	0.028	0.7852	3405	2.40E-06	0.006517948
A	G	-0.3186	0.062	0.0363	3405	2.77E-07	0.007727608
A	G	-0.1784	0.037	0.2229	3405	1.37E-06	0.006828894
A	G	0.1633	0.03	0.7012	3405	7.10E-08	0.008491572
A	G	0.1509	0.033	0.3771	3405	4.38E-06	0.006177638
A	G	1.635	0.321	0.0013	3405	3.81E-07	0.007547561
C	T	0.3441	0.068	0.0295	3405	4.69E-07	0.007429412
C	T	0.1935	0.041	0.0793	3405	3.14E-06	0.006365632
G	C	-0.1238	0.026	0.2803	3405	2.45E-06	0.00650466
C	T	-0.1773	0.037	0.1094	3405	2.03E-06	0.006605964
G	T	-0.1819	0.037	0.89	3405	9.74E-07	0.00702171
C	G	-0.2006	0.036	0.1217	3405	3.02E-08	0.008977036
G	A	-0.1712	0.038	0.1026	3405	8.01E-06	0.005836834
G	A	0.2877	0.064	0.0341	3405	7.22E-06	0.005897907
G	A	-0.429	0.094	0.0142	3405	5.80E-06	0.006019873
G	A	0.2852	0.055	0.0467	3405	1.94E-07	0.007923395
C	T	-0.5089	0.113	0.0103	3405	7.09E-06	0.005910823
T	C	-0.476	0.097	0.0144	3405	8.56E-07	0.00709204
C	A	0.9806	0.171	0.0047	3437	1.00E-08	0.009510147
A	G	0.2555	0.057	0.0444	3437	7.66E-06	0.005809913
C	T	2.691	0.569	0.0003	3437	2.37E-06	0.006458793
G	A	0.3706	0.069	0.0289	3437	9.35E-08	0.008259221
G	A	0.7179	0.116	0.0096	3437	6.33E-10	0.011058642
C	T	0.7278	0.125	0.0089	3437	7.08E-09	0.00970541
T	C	0.4297	0.077	0.0212	3437	3.08E-08	0.008883205
A	G	1.292	0.179	0.0039	3437	6.14E-13	0.014964505
A	G	-0.463	0.075	0.9748	3437	5.95E-10	0.011094947
A	G	2.714	0.202	0.0029	3437	3.22E-40	0.049994688
A	C	1.793	0.206	0.0035	3437	4.93E-18	0.021545943
T	A	-0.4624	0.104	0.0111	3437	8.95E-06	0.005718718
G	A	0.2951	0.061	0.0352	3437	1.54E-06	0.006701947
G	A	-0.2777	0.03	0.8154	3437	1.47E-20	0.024821942
T	C	0.2445	0.052	0.0528	3437	2.94E-06	0.006340281
C	A	-0.1476	0.032	0.1481	3437	5.06E-06	0.006038896
A	G	0.2968	0.066	0.0304	3437	7.69E-06	0.005805609
C	T	0.2946	0.023	0.4709	3437	3.40E-38	0.047429267
T	C	0.209	0.037	0.1084	3437	2.14E-08	0.009085797
C	A	2.013	0.455	0.0006	3437	9.71E-06	0.005675036
G	A	-0.1194	0.025	0.3224	3437	1.36E-06	0.006769257
A	G	-0.1327	0.029	0.5121	3437	5.13E-06	0.006030373
T	C	-0.1758	0.035	0.3326	3437	6.80E-07	0.007152428
A	G	0.831	0.187	0.0033	3437	8.78E-06	0.005731097
C	G	-0.1329	0.03	0.8356	3437	9.37E-06	0.005692558
G	A	-0.2244	0.048	0.0611	3437	3.35E-06	0.006266744
T	C	1.588	0.318	0.0016	3437	6.00E-07	0.007221259

G	A	0.7514	0.166	0.0047	3437	6.33E-06	0.005911881
A	G	0.4351	0.095	0.0145	3437	5.17E-06	0.006026932
C	T	0.3448	0.072	0.0249	3437	1.92E-06	0.006577377
T	C	0.2321	0.049	0.0541	3437	2.23E-06	0.006496171
T	A	-0.5364	0.107	0.0111	3437	5.94E-07	0.007231963
A	G	-0.3827	0.077	0.9748	3437	6.28E-07	0.007196968
C	T	0.6595	0.129	0.0089	3437	3.36E-07	0.007547108
G	T	0.6122	0.121	0.0092	3437	4.73E-07	0.007356627
A	G	1.123	0.185	0.0039	3437	1.40E-09	0.010618664
C	T	2.897	0.595	0.0003	3437	1.18E-06	0.006847836
G	A	0.6418	0.119	0.0096	3437	7.60E-08	0.008378041
A	G	2.309	0.209	0.0029	3437	8.26E-28	0.034167773
T	G	0.2405	0.053	0.0524	3437	6.28E-06	0.00591752
A	C	1.439	0.213	0.0035	3437	1.72E-11	0.013093364
A	C	0.2552	0.052	0.0541	3437	8.37E-07	0.007039353
T	C	0.1996	0.038	0.1084	3437	1.64E-07	0.007942715
C	T	0.2762	0.023	0.4709	3437	4.78E-32	0.039604275
A	C	-0.2655	0.03	0.8142	3437	4.22E-18	0.02165471
G	A	0.2882	0.063	0.0352	3437	4.73E-06	0.006076791
A	G	-0.2906	0.062	0.0361	3437	2.70E-06	0.006386029
G	A	-0.1546	0.034	0.8586	3437	4.82E-06	0.006068042
T	C	-0.1474	0.032	0.5928	3437	3.12E-06	0.006310553
G	A	-0.155	0.03	0.4537	3437	1.83E-07	0.007888509
T	A	-0.1068	0.024	0.4773	3437	7.87E-06	0.005790765
G	A	-0.1891	0.041	0.0841	3437	5.12E-06	0.006030677
C	T	0.2055	0.046	0.0716	3437	8.13E-06	0.005778159
T	C	1.539	0.327	0.0016	3437	2.54E-06	0.006419018
G	A	-0.1913	0.041	0.09	3437	3.61E-06	0.006227609
G	T	-0.1657	0.037	0.8897	3437	7.34E-06	0.005829593
A	C	0.2778	0.058	0.0425	3437	2.12E-06	0.006520496
T	C	0.3703	0.081	0.0212	3437	4.62E-06	0.006088754
T	G	-0.2123	0.047	0.0653	3437	5.47E-06	0.005994849
T	C	-0.3187	0.07	0.0273	3437	5.91E-06	0.00595418
A	G	-0.1499	0.033	0.1507	3437	4.94E-06	0.006047439
A	C	0.3318	0.073	0.0278	3437	5.15E-06	0.006028881
A	C	0.5355	0.117	0.0105	3437	4.91E-06	0.006057993
G	A	2.017	0.442	0.0006	3437	5.21E-06	0.00601962
G	C	0.1481	0.032	0.1477	3437	3.86E-06	0.006185761
A	G	-0.1445	0.032	0.1621	3437	5.40E-06	0.006001716
C	A	0.855	0.187	0.0047	3405	5.22E-06	0.006076158
A	G	2.134	0.223	0.0029	3405	2.17E-21	0.026121555
A	G	0.6136	0.121	0.0101	3405	4.24E-07	0.00748347
C	T	0.8565	0.137	0.009	3405	4.29E-10	0.011381361
A	C	1.188	0.228	0.0034	3405	1.89E-07	0.007944906
A	G	-0.1566	0.028	0.2875	3405	2.88E-08	0.009007001
C	A	1.521	0.329	0.0013	3405	3.85E-06	0.006249128
G	A	0.1912	0.027	0.2972	3405	4.16E-12	0.014008205
C	T	-0.1138	0.025	0.4137	3405	7.22E-06	0.005897543

T	A	1.024	0.204	0.0034	3405	5.49E-07	0.007338343
T	G	0.2238	0.049	0.0712	3405	5.77E-06	0.006023019
C	T	-0.1276	0.028	0.2797	3405	4.10E-06	0.006206717
T	G	0.7719	0.064	0.0408	3405	3.06E-33	0.041504082
A	C	0.6017	0.121	0.0107	3405	6.32E-07	0.007257465
A	G	0.7212	0.056	0.0554	3405	3.75E-37	0.046526743
T	C	0.3601	0.061	0.0464	3405	3.22E-09	0.010243601
A	G	0.5828	0.059	0.046	3405	1.03E-22	0.02786704
C	T	0.6358	0.079	0.0263	3405	1.07E-15	0.018713954
G	T	-1.021	0.214	0.0031	3405	1.83E-06	0.006659223
T	A	0.4945	0.072	0.0311	3405	1.00E-11	0.013515392
T	G	0.5292	0.087	0.021	3405	1.10E-09	0.010852961
G	C	0.8068	0.021	0.4264	3405	1.00E-200	0.292758526
T	C	0.4336	0.05	0.0675	3405	3.04E-18	0.022029336
T	C	-0.1716	0.037	0.8774	3405	4.56E-06	0.006151191
T	C	0.6066	0.13	0.0094	3405	3.29E-06	0.006334407
A	G	0.1371	0.031	0.2137	3405	8.24E-06	0.005826746
G	C	-0.3394	0.07	0.0329	3405	1.38E-06	0.006823857
T	C	-0.3521	0.079	0.0266	3405	8.19E-06	0.005824984
C	T	-0.2253	0.05	0.0626	3405	7.27E-06	0.005890126
A	G	1.259	0.199	0.0036	3437	2.82E-10	0.011511644
C	T	0.66	0.137	0.0089	3437	1.45E-06	0.006736523
C	T	1.767	0.375	0.001	3437	2.53E-06	0.006421917
T	C	-0.1662	0.035	0.1589	3437	1.64E-06	0.006660793
T	C	0.2854	0.061	0.0425	3437	3.41E-06	0.006259131
C	A	-0.1237	0.028	0.7296	3437	9.78E-06	0.005678789
C	T	-0.1175	0.025	0.4149	3437	2.90E-06	0.00634063
G	A	0.5307	0.113	0.0124	3437	2.91E-06	0.006343009
T	C	0.8281	0.183	0.0049	3437	6.29E-06	0.005916058
C	A	-0.1457	0.032	0.1977	3437	3.94E-06	0.006178394
T	C	-0.3623	0.081	0.0239	3437	8.52E-06	0.005751819
T	G	-0.1424	0.031	0.2054	3437	4.27E-06	0.00613718
G	T	-0.9654	0.214	0.0031	3437	6.68E-06	0.005886316
T	A	0.5858	0.071	0.0313	3437	3.07E-16	0.019235174
A	G	-0.3339	0.075	0.9711	3437	8.37E-06	0.005759612
G	A	0.647	0.127	0.0095	3437	3.50E-07	0.007518186
C	A	-0.8878	0.02	0.5738	3437	1.00E-200	0.357982456
G	T	-0.5718	0.053	0.9453	3437	8.39E-27	0.032876045
C	A	-0.3681	0.024	0.5141	3437	6.66E-50	0.06220061
A	C	0.2434	0.043	0.0899	3437	1.98E-08	0.009126553
C	T	0.2009	0.027	0.3595	3437	4.85E-14	0.016398261
G	A	0.5451	0.053	0.0588	3437	7.07E-25	0.030400531
T	C	0.125	0.027	0.2733	3437	4.62E-06	0.006089388
G	C	-0.1198	0.027	0.6881	3437	7.93E-06	0.005793141
G	T	0.4488	0.099	0.0163	3437	5.98E-06	0.005946222
G	C	0.2848	0.063	0.0378	3437	6.49E-06	0.005901463
T	A	1.009	0.226	0.0031	3437	8.20E-06	0.005776158
C	T	-0.223	0.05	0.0628	3437	7.54E-06	0.005818801

A	T	0.4208	0.084	0.0215	3437	6.01E-07	0.007222963
A	C	0.7843	0.129	0.0095	3437	1.29E-09	0.010656781
C	T	2.837	0.624	0.0003	3437	5.74E-06	0.005970526
C	T	1.311	0.191	0.0042	3437	6.98E-12	0.013592281
G	A	2.267	0.217	0.0032	3437	3.79E-25	0.030749626
A	G	1.718	0.184	0.0044	3437	1.35E-20	0.02486896
C	A	0.8819	0.186	0.0047	3437	2.32E-06	0.006470652
C	A	1.581	0.328	0.0013	3437	1.47E-06	0.00672667
G	A	0.201	0.027	0.2975	3437	1.83E-13	0.015651042
A	C	0.6189	0.134	0.0077	3437	3.83E-06	0.006195829
A	G	-0.1612	0.028	0.2866	3437	7.94E-09	0.009639839
G	A	-0.1114	0.025	0.4491	3437	6.84E-06	0.005869297
G	T	0.1241	0.025	0.412	3437	9.15E-07	0.006990096
T	C	0.2772	0.063	0.0418	3437	9.87E-06	0.005669069
G	C	0.3075	0.068	0.9674	3437	6.32E-06	0.005917941
T	C	-0.3668	0.081	0.0239	3437	6.37E-06	0.005912089
C	T	-0.1275	0.027	0.28	3437	3.30E-06	0.006278751
T	C	0.6998	0.072	0.0317	3437	4.24E-22	0.026822702
A	G	0.8426	0.021	0.4267	3437	1.00E-200	0.323583558
T	C	0.3811	0.06	0.0464	3437	2.30E-10	0.011628667
G	T	-1.108	0.214	0.0031	3437	2.25E-07	0.007775249
A	C	0.5464	0.074	0.0295	3437	1.95E-13	0.015606744
T	A	-0.1514	0.034	0.165	3437	8.81E-06	0.005732738
T	C	-0.2216	0.025	0.4069	3437	3.59E-18	0.021732961
C	T	0.1965	0.027	0.3595	3437	1.55E-13	0.015733981
T	G	0.8056	0.063	0.0409	3437	1.06E-36	0.045538471
C	T	0.6853	0.05	0.0666	3437	1.86E-41	0.0515694
A	G	0.6067	0.058	0.0464	3437	4.44E-25	0.030658339
T	C	0.1209	0.027	0.2733	3437	9.09E-06	0.005711215
C	T	0.1246	0.028	0.2827	3437	7.80E-06	0.005802498
T	C	0.6146	0.13	0.0093	3437	2.18E-06	0.006500745
G	C	-0.3327	0.069	0.033	3437	1.53E-06	0.006701553
T	A	1.095	0.225	0.0031	3437	1.25E-06	0.006819758
C	T	-0.2311	0.05	0.0628	3437	3.35E-06	0.006269034
C	A	0.1309	0.03	0.22	3437	9.81E-06	0.005673095
G	A	-1.414	0.228	0.0032	3405	5.88E-10	0.011198625
G	C	-0.6234	0.136	0.0085	3405	4.38E-06	0.00617798
A	C	-1.288	0.235	0.0034	3405	4.23E-08	0.008782093
C	T	0.2633	0.059	0.0533	3405	9.30E-06	0.005756625
T	C	0.9001	0.19	0.0044	3405	2.27E-06	0.006541089
A	G	0.5526	0.125	0.0101	3405	9.99E-06	0.005715974
A	C	0.1467	0.033	0.1816	3405	8.95E-06	0.005780792
C	G	-0.5075	0.113	0.0138	3405	7.64E-06	0.005868216
T	A	-0.1265	0.026	0.4164	3405	1.48E-06	0.006784391
C	A	-2.9	0.633	0.0004	3405	4.78E-06	0.006126362
T	C	-0.4684	0.104	0.0206	3405	6.25E-06	0.005979041
C	T	-0.4526	0.099	0.0166	3405	5.05E-06	0.006097087
T	C	-0.1164	0.026	0.3626	3405	8.58E-06	0.005802933

G	A	0.1569	0.035	0.1517	3405	7.65E-06	0.005867283
G	A	-0.1186	0.027	0.647	3405	8.75E-06	0.005795778
T	C	0.1817	0.039	0.1232	3405	3.55E-06	0.006295818
A	G	-1.532	0.224	0.0029	3437	9.63E-12	0.013414943
A	C	-1.347	0.224	0.0035	3437	2.06E-09	0.01040233
C	T	0.2723	0.055	0.0546	3437	7.50E-07	0.007101654
A	G	-0.2757	0.06	0.0432	3437	4.45E-06	0.006107671
A	G	0.4628	0.08	0.9748	3437	9.52E-09	0.009541297
G	A	-0.2394	0.051	0.0615	3437	2.65E-06	0.006397591
A	G	-0.964	0.197	0.0039	3437	1.02E-06	0.00693271
T	C	0.9223	0.204	0.0039	3437	6.43E-06	0.005906174
G	T	-0.8837	0.192	0.0041	3437	4.31E-06	0.006125748
A	G	0.1469	0.032	0.1852	3437	3.29E-06	0.006279943
A	C	-0.1095	0.024	0.475	3437	7.91E-06	0.005787687
C	T	0.3951	0.083	0.0223	3437	2.31E-06	0.006473587
C	T	0.1526	0.033	0.1609	3437	4.08E-06	0.006157138
G	A	-0.1369	0.031	0.1957	3437	8.37E-06	0.005759808
C	T	0.8536	0.188	0.0044	3437	5.82E-06	0.005962336
C	G	-0.1829	0.04	0.1231	3437	3.87E-06	0.006186987
G	C	-0.5506	0.123	0.0106	3437	7.97E-06	0.005787036
T	C	0.2021	0.043	0.0889	3437	2.58E-06	0.006412717
T	C	0.1893	0.042	0.0918	3437	7.70E-06	0.005809563
C	G	-0.3153	0.069	0.0335	3437	4.78E-06	0.006068333
A	G	0.1611	0.035	0.1423	3437	4.45E-06	0.006112524
C	G	0.1524	0.031	0.1759	3437	9.79E-07	0.006951497
C	T	-0.1248	0.028	0.263	3437	7.27E-06	0.00584189
T	G	0.1158	0.026	0.6676	3437	7.95E-06	0.005786993
T	A	0.3286	0.072	0.0297	3437	5.77E-06	0.005964313
A	G	-0.1254	0.026	0.4658	3437	1.15E-06	0.006858193
A	G	-0.3664	0.081	0.0233	3437	6.58E-06	0.005893499
C	T	0.1108	0.024	0.4406	3437	5.63E-06	0.005978397
A	G	0.1908	0.042	0.0946	3437	6.32E-06	0.005915365
A	G	-0.7579	0.171	0.0052	3437	9.59E-06	0.00568299
T	A	0.3619	0.075	0.0285	3437	1.51E-06	0.006711094
C	T	0.2547	0.056	0.0546	3437	5.65E-06	0.00597632
A	G	0.2145	0.044	0.0898	3437	1.47E-06	0.006723739
C	T	-0.6866	0.133	0.0089	3437	2.56E-07	0.00769432
A	G	-0.8482	0.188	0.0044	3437	6.72E-06	0.005881358
A	C	-1.152	0.229	0.0035	3437	5.25E-07	0.007302844
G	C	-0.6029	0.133	0.0084	3437	6.29E-06	0.005916618
A	G	-0.354	0.077	0.0276	3437	4.46E-06	0.006108841
A	G	0.1452	0.03	0.2376	3437	1.43E-06	0.006742757
A	G	-0.7478	0.148	0.0083	3437	4.42E-07	0.007392986
T	G	0.4769	0.097	0.0156	3437	1.04E-06	0.006918425
G	C	-0.128	0.029	0.2991	3437	8.39E-06	0.00576199
C	T	-0.1278	0.028	0.263	3437	6.79E-06	0.005873694
T	G	0.1375	0.026	0.3413	3437	1.96E-07	0.007842543
G	A	0.1264	0.027	0.5349	3437	2.54E-06	0.006420953

T	C	0.4076	0.089	0.0198	3437	5.19E-06	0.006025059
A	G	-0.2311	0.052	0.0634	3437	8.06E-06	0.00577992
C	T	-0.1138	0.025	0.5797	3437	6.56E-06	0.005893755
T	A	0.3571	0.077	0.0285	3437	3.19E-06	0.006294978
C	T	0.121	0.027	0.2814	3437	9.99E-06	0.005666647
C	A	0.2441	0.05	0.068	3405	1.20E-06	0.006904327
C	G	-0.401	0.085	0.0231	3405	2.53E-06	0.006484782
G	A	-0.5171	0.106	0.0145	3405	1.02E-06	0.006992887
T	C	-0.2705	0.059	0.0515	3405	4.78E-06	0.006125046
C	T	0.1184	0.027	0.6101	3405	8.52E-06	0.005806682
A	G	0.2212	0.047	0.0846	3405	2.29E-06	0.006540301
A	G	0.1591	0.034	0.1783	3405	3.37E-06	0.006323017
C	T	-0.3155	0.065	0.0414	3405	1.08E-06	0.006962826
A	T	-0.1467	0.031	0.2395	3405	1.57E-06	0.006748429
G	A	-0.3443	0.072	0.0391	3405	1.65E-06	0.006722748
A	T	-0.4955	0.11	0.014	3405	6.42E-06	0.005966916
T	C	0.1205	0.027	0.3335	3405	9.23E-06	0.005764569
T	G	0.8768	0.021	0.4687	3405	1.00E-200	0.33522034
T	C	0.3373	0.051	0.0675	3405	4.74E-11	0.012634318
T	C	-0.197	0.043	0.1021	3405	4.28E-06	0.006186367
C	A	-0.3631	0.025	0.514	3405	1.80E-45	0.057126135
C	A	-0.2494	0.031	0.2213	3405	3.06E-15	0.018122399
T	C	-0.3653	0.078	0.0476	3405	3.26E-06	0.006342059
A	G	-0.189	0.029	0.2894	3405	5.63E-11	0.012532953
A	G	-0.1316	0.027	0.3366	3405	1.46E-06	0.00679304
G	A	0.4192	0.046	0.0878	3405	1.02E-19	0.023961484
A	G	0.1497	0.032	0.2137	3405	2.12E-06	0.006580919
G	A	0.125	0.027	0.3192	3405	3.85E-06	0.006241539
G	C	0.2605	0.057	0.0542	3405	5.66E-06	0.00603129
A	G	0.5601	0.119	0.0115	3405	2.54E-06	0.006485676
G	A	0.1372	0.03	0.7573	3405	3.77E-06	0.006261711
T	C	2.666	0.571	0.0004	3405	3.16E-06	0.006359284
A	G	0.1337	0.029	0.2515	3405	5.09E-06	0.006086284
T	C	0.3109	0.06	0.0457	3405	2.51E-07	0.00777987
T	C	-0.7453	0.168	0.0057	3405	9.64E-06	0.005733187
A	G	0.1348	0.026	0.4019	3405	2.65E-07	0.007749485
A	G	1.942	0.401	0.0007	3437	1.36E-06	0.00676755
C	A	0.3024	0.057	0.0508	3437	1.30E-07	0.008077507
G	C	-0.4465	0.098	0.0159	3437	5.65E-06	0.005976681
C	T	-0.1184	0.027	0.3174	3437	9.13E-06	0.005714347
C	A	-0.1875	0.041	0.1079	3437	4.30E-06	0.006131074
G	A	-1.135	0.255	0.0026	3437	8.69E-06	0.005740019
T	C	1.098	0.242	0.0026	3437	5.90E-06	0.005953892
T	G	-0.3945	0.088	0.0185	3437	8.01E-06	0.005784435
A	G	0.1828	0.041	0.1013	3437	8.63E-06	0.005744859
A	G	-0.125	0.025	0.5692	3437	9.70E-07	0.006959047
G	A	-0.5101	0.104	0.0145	3437	9.19E-07	0.0069908
C	T	0.174	0.038	0.1266	3437	6.16E-06	0.005932261

A	G	3.675	0.828	0.0001	3437	9.29E-06	0.005703026
A	G	-0.138	0.029	0.23	3437	2.14E-06	0.006509588
T	C	0.4874	0.105	0.0141	3437	3.86E-06	0.006183247
G	A	1.849	0.414	0.001	3437	8.32E-06	0.005767291
C	G	0.1497	0.032	0.1759	3437	2.83E-06	0.006354737
T	C	0.3615	0.05	0.0675	3437	6.86E-13	0.014898731
T	C	-0.1886	0.027	0.3439	3437	2.05E-12	0.014288309
T	C	-0.3505	0.077	0.0474	3437	5.68E-06	0.005973932
G	A	0.4416	0.045	0.0877	3437	2.46E-22	0.02712623
A	C	0.3533	0.078	0.0294	3437	5.94E-06	0.005950485
T	G	0.4456	0.055	0.0528	3437	1.08E-15	0.018527827
T	G	0.4454	0.088	0.0227	3437	4.87E-07	0.007340205
A	G	-0.9139	0.02	0.5319	3437	1.00E-200	0.370258684
C	A	-0.3538	0.025	0.5141	3437	1.32E-44	0.055523579
A	G	0.6005	0.129	0.0106	3437	3.23E-06	0.006284588
A	G	-0.1864	0.028	0.2888	3437	5.61E-11	0.012412956
G	A	1.041	0.22	0.0029	3437	2.34E-06	0.006472269
T	C	0.1589	0.036	0.1459	3437	9.46E-06	0.005692953
C	T	0.2868	0.064	0.041	3437	6.98E-06	0.005859694
A	G	0.1447	0.032	0.1898	3437	5.26E-06	0.006018241
A	G	-0.9656	0.217	0.0029	3437	9.04E-06	0.005717486
C	T	0.1177	0.026	0.3926	3437	6.03E-06	0.005945307
A	G	-1.201	0.193	0.0044	3405	5.94E-10	0.011198633
A	C	-1.455	0.237	0.0034	3405	9.40E-10	0.010938781
G	A	-2.032	0.228	0.0032	3405	8.69E-19	0.022756312
A	C	-0.3478	0.075	0.0301	3405	3.37E-06	0.006321179
A	G	-0.5613	0.124	0.0103	3405	6.22E-06	0.005981691
C	A	-2.391	0.519	0.0006	3405	4.30E-06	0.006185056
G	A	-3.209	0.72	0.0007	3405	8.56E-06	0.005801643
C	G	-3.936	0.843	0.0003	3405	3.15E-06	0.006360099
C	G	0.694	0.151	0.007	3405	4.21E-06	0.006198002
C	T	1.384	0.305	0.0022	3405	5.69E-06	0.006026568
A	G	-0.1271	0.027	0.6416	3405	2.32E-06	0.006528211
C	T	0.3868	0.082	0.0267	3405	2.44E-06	0.006504915
C	T	-2.993	0.676	0.0003	3405	9.88E-06	0.005722451
T	C	0.3436	0.057	0.0542	3405	2.27E-09	0.010435611
A	G	1.709	0.379	0.001	3405	6.61E-06	0.005942356
A	G	0.722	0.159	0.0063	3405	5.52E-06	0.006049447
T	C	0.2636	0.058	0.0523	3405	4.97E-06	0.006102624
C	T	-0.1297	0.028	0.5289	3405	4.40E-06	0.006174118
T	C	-0.5134	0.105	0.0206	3405	9.43E-07	0.007038731
A	G	-0.1209	0.026	0.4314	3405	2.86E-06	0.006417613
G	A	1.442	0.313	0.0019	3405	4.32E-06	0.006186921
C	T	0.2109	0.044	0.1035	3405	1.66E-06	0.006720282
T	A	-0.1244	0.028	0.3065	3405	8.88E-06	0.005780052
A	G	-1.776	0.177	0.0044	3437	2.93E-23	0.028334525
C	T	-1.417	0.184	0.0042	3437	1.78E-14	0.016944572
C	A	-0.8547	0.181	0.0047	3437	2.39E-06	0.006452963

T	C	1.612	0.341	0.0012	3437	2.39E-06	0.006452398
A	C	-0.6193	0.125	0.0095	3437	7.60E-07	0.007091074
T	C	-0.4131	0.082	0.0212	3437	4.56E-07	0.007374638
A	G	0.1243	0.026	0.2979	3437	1.55E-06	0.006692642
G	A	-2.54	0.209	0.0032	3437	3.14E-33	0.041089197
G	A	0.212	0.046	0.0711	3437	3.98E-06	0.006171172
T	C	-0.2213	0.039	0.1084	3437	1.44E-08	0.009309565
G	A	0.2955	0.031	0.8154	3437	3.31E-21	0.025659218
C	T	-0.2879	0.024	0.4709	3437	3.53E-33	0.041034156
C	T	-0.2131	0.048	0.0701	3437	7.55E-06	0.00581459
C	A	0.1532	0.034	0.1481	3437	6.30E-06	0.005913917
T	C	-0.814	0.176	0.0049	3437	3.83E-06	0.00619213
C	T	-0.3801	0.083	0.0214	3437	4.42E-06	0.006114511
G	A	0.1188	0.026	0.3224	3437	4.48E-06	0.006107615
C	T	0.1387	0.031	0.5804	3437	8.15E-06	0.005775828
T	G	0.1091	0.024	0.5701	3437	6.77E-06	0.005878664
C	T	-0.2123	0.047	0.0716	3437	6.73E-06	0.005881468
C	G	0.1507	0.031	0.8356	3437	1.76E-06	0.006627741
C	G	-0.4074	0.084	0.0221	3437	1.12E-06	0.006875201
C	T	-0.7938	0.179	0.0044	3437	9.51E-06	0.005689298
A	G	-0.5285	0.116	0.0097	3437	5.29E-06	0.006013455
C	T	0.1629	0.034	0.1562	3437	1.25E-06	0.006816571
A	G	0.1495	0.033	0.1565	3437	7.07E-06	0.005854531
T	A	0.3416	0.076	0.0256	3437	6.30E-06	0.005917722
G	A	-0.2105	0.046	0.9326	3437	5.36E-06	0.00600896
G	C	0.2934	0.065	0.0342	3437	5.98E-06	0.005943942
A	G	0.1197	0.025	0.3714	3437	1.54E-06	0.006700186
A	T	-1.008	0.214	0.0035	3437	2.67E-06	0.006390103
G	A	-2.18	0.221	0.0032	3437	9.94E-23	0.027628336
T	C	-2.172	0.472	0.0007	3437	4.35E-06	0.006123343
G	T	-0.1919	0.039	0.8829	3437	7.73E-07	0.007084987
C	T	-0.5212	0.104	0.0141	3437	5.04E-07	0.00732413
A	G	-1.625	0.187	0.0044	3437	4.49E-18	0.021611303
A	C	-1.514	0.229	0.0035	3437	4.11E-11	0.012601213
G	A	-0.1818	0.028	0.2975	3437	5.43E-11	0.012439705
A	C	0.1191	0.026	0.5911	3437	4.79E-06	0.006068117
G	C	-0.7233	0.161	0.0063	3437	7.27E-06	0.005837983
C	A	-0.1809	0.037	0.8749	3437	1.35E-06	0.006771743
A	G	-0.1137	0.026	0.5422	3437	9.10E-06	0.005715458
C	T	0.1279	0.027	0.3134	3437	2.44E-06	0.006438987
T	G	-0.1153	0.025	0.4529	3437	3.60E-06	0.006234613
G	A	-2.133	0.469	0.0006	3437	5.52E-06	0.005989674
T	C	-0.1342	0.028	0.7284	3437	2.00E-06	0.0065506
C	G	-3.842	0.814	0.0003	3437	2.46E-06	0.006436778
T	C	-0.1178	0.026	0.342	3437	6.72E-06	0.005883045
C	A	-2.942	0.502	0.0006	3437	4.97E-09	0.009901994
C	A	-0.1407	0.032	0.1834	3437	9.89E-06	0.005667081
T	G	0.3405	0.076	0.0308	3437	7.95E-06	0.005788101

T	C	0.1999	0.036	0.1525	3437	4.14E-08	0.008712824
A	C	0.2126	0.039	0.1155	3437	5.28E-08	0.008585027
C	T	0.2816	0.035	0.1562	3437	1.37E-15	0.018393171
T	C	0.1904	0.037	0.1286	3437	3.55E-07	0.007512138
G	T	-2.824	0.598	0.0004	3437	2.45E-06	0.006442435
G	A	0.2115	0.047	0.0751	3437	5.91E-06	0.005955099
G	A	0.3538	0.074	0.0304	3405	1.91E-06	0.006639965
C	T	0.5826	0.129	0.012	3405	6.23E-06	0.0059822
C	T	-0.2948	0.065	0.0414	3405	6.14E-06	0.005988262
A	G	-0.4852	0.106	0.015	3405	4.47E-06	0.006161867
A	G	0.2585	0.058	0.0512	3405	8.78E-06	0.005786023
T	C	0.3162	0.071	0.0342	3405	8.48E-06	0.005805811
A	G	0.2257	0.047	0.0846	3405	1.71E-06	0.006704273
T	A	-0.4106	0.081	0.0295	3405	4.89E-07	0.007404532
C	T	0.5538	0.119	0.0123	3405	3.09E-06	0.00637346
T	C	-0.1185	0.027	0.3499	3405	9.67E-06	0.005730285
C	T	-0.1594	0.035	0.1671	3405	5.24E-06	0.006075293
A	G	-0.1932	0.029	0.2894	3405	3.39E-11	0.012814588
C	T	-0.6337	0.138	0.011	3405	4.64E-06	0.006145901
T	C	-0.1723	0.038	0.1317	3405	6.88E-06	0.005920856
T	C	0.4322	0.051	0.0675	3405	4.98E-17	0.020451315
A	G	-0.9594	0.02	0.532	3405	1.00E-200	0.392377942
C	A	-0.2549	0.032	0.2213	3405	1.33E-15	0.018589151
G	A	0.4715	0.046	0.0878	3405	3.68E-24	0.029755835
C	A	-0.4069	0.025	0.514	3405	3.19E-56	0.07070615
G	C	0.1946	0.043	0.9091	3405	7.81E-06	0.00585382
C	T	0.2069	0.044	0.1035	3405	2.51E-06	0.006487004
G	A	0.1294	0.028	0.2902	3405	3.74E-06	0.006259958
T	A	-0.1636	0.037	0.1373	3405	9.74E-06	0.005730532
C	T	-1.066	0.24	0.0028	3405	9.47E-06	0.00574153
T	C	-0.7497	0.169	0.0057	3405	9.25E-06	0.00575976
T	C	-0.2143	0.048	0.0762	3405	7.40E-06	0.005883014
G	A	-0.2011	0.042	0.8974	3405	1.62E-06	0.006735672
T	C	0.1314	0.028	0.3099	3437	2.23E-06	0.006495233
C	T	-0.772	0.167	0.0063	3437	3.69E-06	0.006216108
A	G	1.987	0.413	0.0007	3437	1.54E-06	0.006699278
C	T	0.6139	0.135	0.9916	3437	5.70E-06	0.005971779
T	A	2.399	0.504	0.0007	3437	1.98E-06	0.00655921
C	A	0.2773	0.059	0.0508	3437	2.30E-06	0.006477378
C	T	-0.2836	0.061	0.0451	3437	3.65E-06	0.006219144
A	G	-0.5969	0.129	0.0102	3437	3.53E-06	0.006238776
G	A	0.131	0.027	0.3363	3437	9.06E-07	0.006991553
A	G	-0.6056	0.133	0.0089	3437	5.13E-06	0.006032224
G	T	1.778	0.37	0.0015	3437	1.66E-06	0.006663051
T	G	-0.3999	0.09	0.0185	3437	9.73E-06	0.005675084
G	A	-0.1352	0.03	0.2277	3437	5.84E-06	0.005957142
T	C	0.1234	0.027	0.6721	3437	6.53E-06	0.005892332
G	A	-0.2856	0.063	0.0435	3437	6.34E-06	0.005913936

C	T	0.6107	0.06	0.0464	3437	7.35E-24	0.029090468
A	T	-0.9412	0.02	0.4862	3437	1.00E-200	0.385485989
C	A	-0.3085	0.054	0.0634	3437	9.17E-09	0.009560435
A	G	0.2459	0.032	0.2076	3437	1.42E-14	0.017078743
G	T	0.4594	0.082	0.0262	3437	2.63E-08	0.008973472
G	C	0.4419	0.097	0.0166	3437	5.90E-06	0.00595451
T	C	0.4259	0.041	0.1114	3437	3.91E-25	0.030729838
G	A	0.7359	0.09	0.0211	3437	5.55E-16	0.01890771
C	A	-0.4034	0.025	0.5141	3437	2.04E-56	0.070315826
G	T	0.4852	0.103	0.0167	3437	2.59E-06	0.006414941
C	G	0.466	0.095	0.0179	3437	1.11E-06	0.006881413
A	G	0.3531	0.08	0.0257	3437	9.24E-06	0.005706845
C	T	-1.107	0.239	0.0028	3437	3.59E-06	0.006223911
C	A	0.1277	0.026	0.6062	3437	8.34E-07	0.0070449
T	C	0.1225	0.027	0.3099	3437	7.93E-06	0.005790336
A	G	1.921	0.408	0.0007	3437	2.53E-06	0.006421097
C	A	0.2606	0.058	0.0508	3437	6.91E-06	0.005867548
T	A	2.598	0.497	0.0007	3437	1.81E-07	0.007887636
A	G	-0.5655	0.127	0.0102	3437	8.66E-06	0.005744608
C	G	0.2334	0.052	0.0626	3437	6.83E-06	0.005872254
A	G	-0.5873	0.131	0.0089	3437	7.56E-06	0.005813873
G	T	1.694	0.366	0.0015	3437	3.77E-06	0.006200953
G	A	-0.1367	0.029	0.2277	3437	3.46E-06	0.006246634
A	G	-0.4063	0.086	0.0223	3437	2.21E-06	0.006497131
T	C	0.2934	0.061	0.0425	3437	1.62E-06	0.006670802
C	T	0.5653	0.06	0.0464	3437	3.98E-21	0.025557421
T	C	0.4056	0.04	0.1114	3437	1.80E-23	0.028586696
T	C	-0.3713	0.077	0.0294	3437	1.33E-06	0.006780958
G	A	0.7299	0.089	0.0211	3437	4.09E-16	0.019075435
A	G	0.2468	0.031	0.2076	3437	5.28E-15	0.017634834
C	A	-0.3962	0.025	0.5141	3437	8.03E-56	0.069542309
C	A	-0.3048	0.053	0.0634	3437	8.96E-09	0.00957033
A	G	-0.1276	0.027	0.684	3437	3.13E-06	0.006306853
G	T	0.4681	0.081	0.0262	3437	9.29E-09	0.009553177
A	T	-0.9321	0.02	0.4862	3437	1.00E-200	0.387712558
C	T	-1.062	0.236	0.0028	3437	6.75E-06	0.005877045
T	C	-0.2086	0.047	0.0781	3437	7.72E-06	0.00580375
A	G	0.4385	0.085	0.0211	3405	3.01E-07	0.007679885
C	T	0.1507	0.025	0.4699	3405	1.68E-09	0.010609242
A	G	0.1242	0.028	0.262	3405	7.98E-06	0.005840237
A	C	-0.164	0.032	0.814	3405	4.71E-07	0.007427343
A	G	0.5612	0.121	0.0101	3405	3.65E-06	0.006277872
G	A	-0.1389	0.031	0.358	3405	8.45E-06	0.005812959
T	C	0.1634	0.032	0.7159	3405	3.67E-07	0.007571106
C	T	0.354	0.072	0.0295	3405	9.09E-07	0.007057182
C	A	0.2204	0.044	0.0803	3405	4.85E-07	0.007411628
A	G	1.629	0.341	0.0013	3405	1.82E-06	0.006673105
A	G	-0.1624	0.035	0.1427	3405	4.32E-06	0.006187971

A	G	0.2633	0.055	0.0548	3405	1.55E-06	0.00675627
C	G	-0.2043	0.038	0.122	3405	8.71E-08	0.00838243
G	T	-0.6053	0.13	0.0085	3405	3.41E-06	0.006317096
C	T	-0.1109	0.024	0.486	3405	5.73E-06	0.006030308
T	C	-0.1151	0.025	0.3626	3405	5.03E-06	0.006099092
C	G	-0.1302	0.028	0.7336	3405	2.54E-06	0.006483842
G	A	0.2954	0.059	0.0442	3405	6.33E-07	0.007259325
A	G	0.1319	0.028	0.2535	3405	3.58E-06	0.006290562
C	T	0.125	0.026	0.7012	3437	1.48E-06	0.006715973
G	A	2.569	0.21	0.0032	3437	1.28E-33	0.041611349
A	G	1.815	0.178	0.0044	3437	5.33E-24	0.029266518
C	A	0.8446	0.182	0.0047	3437	3.53E-06	0.006240454
T	C	-1.588	0.343	0.0012	3437	3.78E-06	0.006197738
T	C	0.4221	0.082	0.0212	3437	2.95E-07	0.007620929
C	T	1.427	0.185	0.0042	3437	1.62E-14	0.016998468
A	C	0.6092	0.126	0.0095	3437	1.30E-06	0.006787532
G	A	-0.2161	0.046	0.0711	3437	2.92E-06	0.006344522
C	A	-0.1538	0.034	0.1481	3437	6.48E-06	0.005897595
C	T	0.2886	0.024	0.4709	3437	5.49E-33	0.040796152
T	C	0.218	0.039	0.1084	3437	2.77E-08	0.00894066
G	A	-0.2936	0.031	0.8154	3437	9.54E-21	0.025070433
T	C	0.7983	0.177	0.0049	3437	6.55E-06	0.005896844
C	T	0.3732	0.083	0.0214	3437	7.34E-06	0.005832527
G	A	-0.1187	0.026	0.3224	3437	5.10E-06	0.006032282
C	T	-0.1408	0.031	0.5804	3437	6.59E-06	0.005890482
T	G	-0.1086	0.024	0.5701	3437	8.28E-06	0.005763491
C	G	-0.1485	0.032	0.8356	3437	2.82E-06	0.006368334
C	G	0.4155	0.084	0.0221	3437	7.80E-07	0.007076798
C	T	0.2151	0.047	0.0716	3437	5.71E-06	0.005973464
A	G	0.5324	0.117	0.0097	3437	5.06E-06	0.006039678
C	T	-0.1646	0.034	0.1562	3437	1.09E-06	0.006889067
A	G	-0.1503	0.033	0.1565	3437	7.03E-06	0.005857256
G	A	0.2102	0.046	0.9326	3437	6.15E-06	0.005927975
T	A	-0.3405	0.076	0.0256	3437	7.52E-06	0.005818459
G	C	-0.2939	0.065	0.0342	3437	6.49E-06	0.005902291
A	G	-0.1195	0.025	0.3714	3437	1.81E-06	0.006609124
A	T	1.032	0.216	0.0035	3437	1.74E-06	0.006628223
C	T	-0.4841	0.103	0.0141	3437	2.48E-06	0.006435643
C	T	-0.2458	0.056	0.055	3437	9.80E-06	0.00567449
A	G	1.827	0.224	0.0029	3437	4.64E-16	0.019004498
C	T	0.9735	0.191	0.0042	3437	3.75E-07	0.007486059
A	G	0.5843	0.121	0.01	3437	1.56E-06	0.0066948
T	A	1.603	0.339	0.0013	3437	2.37E-06	0.006459775
C	T	0.2086	0.025	0.4709	3437	5.19E-17	0.020233471
A	C	-0.2228	0.032	0.8142	3437	5.06E-12	0.013771971
A	G	0.1161	0.025	0.4951	3437	3.15E-06	0.006300693
C	T	-0.1467	0.033	0.1666	3437	8.08E-06	0.005779447
G	C	-0.1107	0.025	0.5835	3437	9.68E-06	0.005681414

T	C	-0.2939	0.066	0.0346	3437	7.82E-06	0.005797291
G	A	-0.1319	0.029	0.7496	3437	5.93E-06	0.005946105
C	T	0.459	0.1	0.0147	3437	4.60E-06	0.006092448
T	C	-0.1995	0.042	0.0912	3437	2.67E-06	0.006391101
T	C	0.1562	0.034	0.1529	3437	4.93E-06	0.006053666
T	C	0.5437	0.118	0.0106	3437	3.90E-06	0.006180619
A	G	0.4712	0.103	0.0177	3437	4.64E-06	0.006087491
T	C	1.044	0.229	0.0029	3437	5.35E-06	0.006005584
A	T	-0.1626	0.036	0.8519	3437	5.68E-06	0.005969499
T	A	-0.1155	0.025	0.4773	3437	4.36E-06	0.006118229
T	G	0.3934	0.086	0.022	3437	4.82E-06	0.006064013
A	G	-0.5131	0.102	0.0167	3437	5.39E-07	0.007280301
A	T	-0.4186	0.093	0.0198	3437	6.94E-06	0.0058638
C	T	-0.1167	0.025	0.6045	3437	3.37E-06	0.006265043
T	C	0.1384	0.029	0.2232	3437	2.79E-06	0.006371792
C	T	0.5039	0.111	0.0129	3437	5.27E-06	0.00601402
C	T	0.7724	0.166	0.0055	3437	3.59E-06	0.006229946
C	G	-0.2529	0.053	0.0598	3437	1.76E-06	0.006623246
A	G	0.726	0.156	0.0081	3437	3.32E-06	0.006270034
T	A	-0.3468	0.078	0.0256	3437	8.89E-06	0.005724555
A	C	0.352	0.077	0.0278	3437	4.45E-06	0.006108025
C	T	-0.1207	0.027	0.7186	3437	9.50E-06	0.005692443
A	G	-0.1276	0.027	0.2789	3437	2.47E-06	0.0064373
A	G	-1.179	0.193	0.0044	3405	1.18E-09	0.010807558
G	A	-2.018	0.228	0.0032	3405	1.47E-18	0.022450837
A	C	-0.3574	0.075	0.0301	3405	1.78E-06	0.006679691
A	G	-0.683	0.15	0.0076	3405	5.26E-06	0.006076224
A	C	-1.518	0.237	0.0034	3405	1.67E-10	0.011914907
G	T	-3.269	0.72	0.0007	3405	5.74E-06	0.006025959
C	A	-2.389	0.519	0.0006	3405	4.36E-06	0.006181872
C	G	-3.873	0.843	0.0003	3405	4.47E-06	0.006163731
C	T	1.467	0.304	0.0022	3405	1.50E-06	0.006774878
A	G	0.8482	0.174	0.0053	3405	1.11E-06	0.006946285
C	T	0.3923	0.082	0.0267	3405	1.73E-06	0.006696468
T	C	-0.5369	0.119	0.0119	3405	7.18E-06	0.00590323
T	C	0.3316	0.057	0.0542	3405	7.93E-09	0.009729752
G	A	-0.1232	0.027	0.3802	3405	6.75E-06	0.005936861
C	T	-2.992	0.676	0.0003	3405	9.87E-06	0.005723699
T	C	0.2653	0.058	0.0523	3405	4.28E-06	0.006187502
T	C	-0.5371	0.104	0.0206	3405	2.85E-07	0.007713109
A	G	-0.1207	0.026	0.4314	3405	2.91E-06	0.006401467
G	A	1.397	0.313	0.0019	3405	8.39E-06	0.005812698
G	A	-1.375	0.294	0.0018	3405	2.95E-06	0.006391459
C	T	0.2078	0.044	0.1035	3405	2.33E-06	0.006531358
A	C	0.1855	0.042	0.1065	3405	8.83E-06	0.005780815
C	G	-0.1302	0.029	0.7336	3405	6.99E-06	0.00590927
G	A	-0.356	0.068	0.0289	3437	2.13E-07	0.007799479
A	G	-1.821	0.166	0.0044	3437	1.87E-27	0.033710345

T	C	-0.1135	0.025	0.2699	3437	6.64E-06	0.005886092
A	C	-1.784	0.204	0.0035	3437	3.70E-18	0.021724993
T	C	-0.4053	0.077	0.0212	3437	1.37E-07	0.00804628
C	T	-2.624	0.568	0.0003	3437	4.02E-06	0.006164634
C	T	-0.7496	0.124	0.0089	3437	1.68E-09	0.01050389
C	A	-0.9956	0.169	0.0047	3437	4.52E-09	0.009961583
G	A	-2.621	0.196	0.0032	3437	6.33E-40	0.049599741
A	G	-0.4303	0.092	0.016	3437	2.74E-06	0.006378205
A	G	0.4611	0.074	0.9748	3437	4.50E-10	0.01125144
T	C	-0.2345	0.052	0.0528	3437	5.65E-06	0.005977775
C	T	0.1141	0.025	0.2959	3437	4.62E-06	0.006086795
A	G	4.109	0.904	0.0003	3437	5.65E-06	0.005977838
G	A	0.2748	0.029	0.8154	3437	1.16E-20	0.024954203
C	T	0.1316	0.029	0.1718	3437	8.08E-06	0.005784055
T	C	-0.4234	0.096	0.0153	3437	9.86E-06	0.005668698
G	A	-0.2867	0.061	0.0352	3437	2.31E-06	0.006470108
T	C	-0.2077	0.037	0.1084	3437	1.70E-08	0.009217853
C	T	-0.2903	0.022	0.4709	3437	6.29E-38	0.04711039
A	G	-2.709	0.595	0.0003	3437	5.51E-06	0.005993051
C	A	-2.124	0.451	0.0006	3437	2.59E-06	0.006411842
G	A	0.1214	0.024	0.3224	3437	6.51E-07	0.007180098
A	C	-2.712	0.596	0.0003	3437	5.61E-06	0.005982247
A	G	0.1281	0.028	0.5121	3437	6.75E-06	0.005884626
T	C	-0.2112	0.047	0.0592	3437	7.66E-06	0.00581121
T	C	0.1731	0.035	0.3326	3437	5.81E-07	0.007242012
C	G	0.1317	0.03	0.8356	3437	9.00E-06	0.005715306
A	G	-0.8373	0.185	0.0033	3437	6.33E-06	0.005911879
C	T	-0.1959	0.044	0.0716	3437	9.82E-06	0.005672682
G	A	0.215	0.048	0.0611	3437	6.33E-06	0.005913174
T	C	-1.546	0.314	0.0016	3437	9.19E-07	0.006986019
A	G	1.967	0.423	0.001	3437	3.51E-06	0.006243279
G	A	-0.7442	0.165	0.0047	3437	6.52E-06	0.005898156
A	G	-0.4419	0.094	0.0145	3437	2.92E-06	0.006343269
G	A	-0.8475	0.187	0.0041	3437	6.35E-06	0.005915399
C	G	-0.7463	0.168	0.0049	3437	8.80E-06	0.005729094
G	A	0.6921	0.153	0.0061	3437	5.90E-06	0.005956946
T	C	-0.2336	0.047	0.063	3437	6.94E-07	0.007139103
A	G	-1.573	0.186	0.0044	3437	3.35E-17	0.020492634
C	T	-2.494	0.546	0.0006	3437	5.09E-06	0.006038318
G	T	-0.1944	0.039	0.8829	3437	4.77E-07	0.007355869
T	C	-2.089	0.469	0.0007	3437	8.89E-06	0.005729487
A	C	-1.565	0.227	0.0035	3437	6.75E-12	0.013616896
G	A	-2.11	0.22	0.0032	3437	1.31E-21	0.02618149
C	T	-0.5302	0.103	0.0141	3437	2.72E-07	0.007665268
G	A	-0.1572	0.028	0.2975	3437	1.21E-08	0.009404271
G	C	-0.7413	0.16	0.0063	3437	3.77E-06	0.006199051
G	C	-0.11	0.025	0.4693	3437	7.85E-06	0.005793204
C	A	-0.1805	0.037	0.8749	3437	1.23E-06	0.006817927

C	G	-3.73	0.81	0.0003	3437	4.23E-06	0.006136442
G	A	-2.136	0.466	0.0006	3437	4.75E-06	0.006075813
T	C	-0.131	0.028	0.7284	3437	3.09E-06	0.006314888
C	A	-2.923	0.499	0.0006	3437	5.13E-09	0.009888608
T	C	-0.1149	0.026	0.342	3437	9.94E-06	0.005658713
C	G	-2.446	0.546	0.0006	3437	7.65E-06	0.005807346
T	C	0.1992	0.036	0.1525	3437	3.87E-08	0.008752346
C	A	0.1207	0.025	0.4751	3437	1.94E-06	0.006568189
T	C	0.4097	0.055	0.0543	3437	1.38E-13	0.015797499
G	C	-0.1567	0.026	0.3848	3437	2.64E-09	0.010253987
G	T	-2.841	0.595	0.0004	3437	1.85E-06	0.006596186
T	C	-0.1382	0.031	0.2104	3437	6.53E-06	0.005899619
A	G	-0.1728	0.037	0.1318	3437	2.81E-06	0.006367459
A	G	-1.986	0.415	0.0009	3437	1.83E-06	0.006606437
T	C	-2.838	0.595	0.0004	3437	1.89E-06	0.006582354
A	G	-0.1198	0.027	0.6891	3437	7.43E-06	0.00582338
G	A	-2.484	0.546	0.0006	3437	5.59E-06	0.005983741
G	C	0.2033	0.045	0.0845	3437	8.13E-06	0.005780119
G	A	0.2327	0.052	0.0602	3437	8.35E-06	0.005761845
A	G	1.332	0.292	0.0016	3437	5.15E-06	0.006026058
T	C	0.1447	0.031	0.2026	3437	4.43E-06	0.006109793
G	T	-2.662	0.478	0.0006	3437	2.68E-08	0.008957774
A	C	0.1666	0.037	0.1391	3405	7.06E-06	0.005909509
T	G	-0.1911	0.041	0.1154	3405	3.80E-06	0.006257619
T	A	0.2998	0.067	0.0396	3405	8.49E-06	0.005806186
A	G	-0.4772	0.104	0.0167	3405	4.53E-06	0.006157018
G	A	0.6588	0.138	0.0091	3405	1.84E-06	0.006658252
T	C	-0.183	0.041	0.1075	3405	9.98E-06	0.005719302
A	G	-0.7243	0.163	0.006	3405	8.76E-06	0.005786612
A	G	0.6264	0.141	0.0084	3405	8.93E-06	0.00577915
T	C	-0.1647	0.036	0.146	3405	6.57E-06	0.005953948
C	G	0.7221	0.15	0.007	3405	1.50E-06	0.00677798
C	T	0.3864	0.081	0.0267	3405	2.10E-06	0.006587069
T	C	0.3573	0.078	0.0276	3405	4.66E-06	0.006142
T	C	0.4625	0.057	0.0542	3405	4.05E-16	0.019259355
C	T	-0.9764	0.212	0.0035	3405	4.22E-06	0.006196934
A	G	1.709	0.378	0.001	3405	6.44E-06	0.00595798
A	G	1.533	0.343	0.0016	3405	8.13E-06	0.005828904
T	C	-0.4767	0.104	0.0206	3405	4.82E-06	0.00612076
A	G	-0.3824	0.086	0.0238	3405	9.09E-06	0.005766408
A	G	0.4896	0.101	0.016	3405	1.23E-06	0.00689449
T	G	0.2962	0.066	0.0388	3405	7.79E-06	0.005852117
T	C	0.6427	0.143	0.0085	3405	7.48E-06	0.005872852
C	T	0.2152	0.044	0.1035	3405	8.48E-07	0.007100688
C	T	0.1763	0.038	0.132	3405	3.21E-06	0.006351358
C	G	-0.3605	0.075	0.0311	3405	1.67E-06	0.006716452
A	G	0.1203	0.026	0.4972	3405	3.72E-06	0.006262417
T	C	-0.4015	0.088	0.0207	3437	4.77E-06	0.006070737

C	T	1.191	0.268	0.002	3437	9.38E-06	0.00570059
A	C	-0.5788	0.12	0.0132	3437	1.46E-06	0.006723343
T	C	0.1222	0.028	0.3	3437	9.99E-06	0.005663033
T	C	-1.787	0.388	0.0009	3437	4.30E-06	0.006124446
T	C	0.2161	0.048	0.0772	3437	6.95E-06	0.005865079
T	C	0.1359	0.03	0.2112	3437	8.25E-06	0.00576579
G	A	-0.1308	0.029	0.2912	3437	9.37E-06	0.005698934
C	T	-0.157	0.033	0.7472	3437	1.95E-06	0.006562202
A	G	0.1491	0.032	0.1852	3437	3.60E-06	0.006230321
T	G	0.2309	0.049	0.07	3437	2.45E-06	0.006440054
C	T	-0.1867	0.042	0.9024	3437	9.57E-06	0.005684036
A	T	-0.4481	0.095	0.0198	3437	2.76E-06	0.006372839
A	C	-0.1358	0.026	0.3695	3437	2.97E-07	0.007616869
T	C	0.592	0.055	0.0543	3437	1.64E-26	0.032506223
A	G	-0.476	0.098	0.0159	3437	1.40E-06	0.006752799
T	C	-0.1372	0.031	0.2104	3437	9.74E-06	0.005674069
C	T	-0.2994	0.065	0.0407	3437	4.27E-06	0.006133267
C	T	0.176	0.038	0.8718	3437	3.74E-06	0.006209132
A	G	0.3244	0.072	0.0317	3437	7.57E-06	0.005815301
A	G	0.1524	0.033	0.2227	3437	5.17E-06	0.006028257
G	C	0.206	0.046	0.0845	3437	8.26E-06	0.005766181
T	C	-0.1839	0.04	0.1135	3437	4.85E-06	0.006063941
T	C	0.64	0.141	0.0084	3437	5.95E-06	0.00595024
T	A	0.4545	0.092	0.0188	3437	9.02E-07	0.00699785
C	G	0.3302	0.066	0.0368	3437	6.42E-07	0.007184491
C	G	-0.335	0.074	0.031	3437	6.38E-06	0.005909928
A	G	-0.4633	0.102	0.0172	3437	5.25E-06	0.006013649
A	C	-0.5916	0.121	0.0132	3437	9.74E-07	0.006952678
T	C	0.2544	0.055	0.0634	3437	3.27E-06	0.006279036
T	C	-2.022	0.39	0.0009	3437	2.28E-07	0.007760152
G	C	-0.1174	0.025	0.5601	3437	4.21E-06	0.006143593
G	C	-0.1753	0.038	0.1269	3437	4.93E-06	0.006055114
C	G	-0.1329	0.029	0.2518	3437	5.97E-06	0.00595036
G	A	-0.1445	0.03	0.2912	3437	1.10E-06	0.006881458
C	T	-0.5202	0.116	0.0125	3437	7.30E-06	0.00583716
T	C	0.1213	0.027	0.3473	3437	5.08E-06	0.006036476
A	C	-0.9208	0.196	0.0036	3437	2.63E-06	0.006400015
G	A	-0.8322	0.184	0.0049	3437	6.23E-06	0.005922874
T	G	0.277	0.062	0.046	3437	7.27E-06	0.005837546
T	C	0.1265	0.027	0.35	3437	2.71E-06	0.006383666
T	C	0.5156	0.056	0.0543	3437	3.21E-20	0.024392672
G	C	0.1454	0.031	0.214	3437	2.44E-06	0.0064423
C	T	0.3799	0.086	0.022	3437	9.92E-06	0.005666459
A	T	-0.235	0.051	0.0652	3437	5.20E-06	0.006026345
C	G	0.4872	0.105	0.0147	3437	3.51E-06	0.006236873
G	C	-0.1182	0.025	0.4364	3437	3.22E-06	0.006285806
T	C	-0.2661	0.058	0.049	3437	4.55E-06	0.006095344
G	C	0.2541	0.057	0.0527	3437	8.46E-06	0.005754808

A	G	-0.1757	0.037	0.134	3437	2.03E-06	0.006542664
A	C	0.4366	0.084	0.0251	3405	2.04E-07	0.007897636
T	A	0.7554	0.158	0.0066	3405	1.89E-06	0.00665159
C	T	0.7176	0.145	0.0078	3405	8.28E-07	0.007112407
G	A	0.5142	0.114	0.0131	3405	7.18E-06	0.005898286
C	T	1.029	0.2	0.0043	3405	2.65E-07	0.007744901
T	C	1.722	0.191	0.0041	3405	3.67E-19	0.02324371
A	C	-0.1861	0.027	0.5907	3405	3.74E-12	0.014066969
T	A	3.538	0.793	0.0006	3405	8.42E-06	0.005810477
G	A	0.2347	0.028	0.2972	3405	2.15E-16	0.019622249
T	C	0.1969	0.042	0.1084	3405	3.34E-06	0.006326198
A	G	-0.1896	0.043	0.1041	3405	9.31E-06	0.005757001
T	C	-0.2899	0.064	0.0399	3405	6.61E-06	0.005945365
T	C	0.4242	0.096	0.0191	3405	9.56E-06	0.005739605
G	A	-0.8126	0.162	0.0063	3405	5.37E-07	0.007353164
T	C	-0.9619	0.199	0.0043	3405	1.46E-06	0.006794651
T	C	-0.3121	0.068	0.0372	3405	4.63E-06	0.006144976
A	G	0.1573	0.032	0.2093	3405	1.26E-06	0.006878926
C	G	-0.205	0.04	0.1217	3405	2.94E-07	0.007696742
C	T	1.573	0.332	0.0013	3405	2.32E-06	0.006533883
G	T	0.4748	0.097	0.0175	3405	1.15E-06	0.00692911
C	G	-0.1593	0.035	0.8327	3405	5.00E-06	0.006105871
C	A	0.3206	0.072	0.0322	3405	7.93E-06	0.00584563
G	A	0.2509	0.057	0.0546	3405	9.64E-06	0.005735875
G	T	0.3096	0.065	0.0439	3405	1.84E-06	0.00666141
A	G	0.3347	0.068	0.0377	3405	1.03E-06	0.006987005
G	C	0.1319	0.029	0.7458	3405	7.41E-06	0.005884448
G	A	-0.1531	0.033	0.2043	3405	4.77E-06	0.006132939
G	C	1.067	0.206	0.0042	3437	2.33E-07	0.007745296
T	C	1.941	0.191	0.0041	3437	4.76E-24	0.029319587
C	G	-0.1628	0.036	0.1466	3437	8.32E-06	0.005761165
C	T	0.7622	0.144	0.0077	3437	1.38E-07	0.00804114
G	A	0.6318	0.107	0.0145	3437	4.42E-09	0.009968281
T	A	0.7445	0.157	0.0065	3437	2.28E-06	0.006483654
A	G	0.342	0.072	0.0326	3437	2.35E-06	0.00646634
A	C	-0.2138	0.026	0.5911	3437	5.23E-16	0.018949578
A	T	0.2764	0.029	0.2587	3437	4.05E-21	0.025560366
T	A	3.688	0.786	0.0006	3437	2.81E-06	0.006366401
C	T	-0.2323	0.044	0.9022	3437	1.13E-07	0.008158253
T	G	0.1237	0.028	0.2963	3437	7.92E-06	0.005793757
G	T	0.127	0.029	0.6989	3437	9.34E-06	0.005696494
C	G	3.89	0.845	0.0003	3437	4.33E-06	0.006123929
G	A	-0.7343	0.161	0.0063	3437	5.26E-06	0.006015826
T	C	-0.8787	0.198	0.0042	3437	9.15E-06	0.005709034
A	G	-0.6809	0.151	0.0065	3437	6.70E-06	0.005881275
C	T	0.225	0.05	0.0716	3437	8.33E-06	0.00576064
G	A	-0.1147	0.026	0.4776	3437	7.00E-06	0.005852183
T	C	0.1141	0.026	0.5914	3437	9.69E-06	0.005675813

T	C	0.4488	0.1	0.0166	3437	7.43E-06	0.005826241
G	A	3.007	0.586	0.0004	3437	3.10E-07	0.00759258
A	G	0.1841	0.032	0.2082	3437	7.06E-09	0.00970568
A	G	-0.1482	0.032	0.1907	3437	5.12E-06	0.00603198
C	G	-0.1771	0.039	0.1219	3437	6.58E-06	0.005891601
A	T	0.4045	0.088	0.0212	3437	4.52E-06	0.006101578
A	G	0.4908	0.107	0.0145	3437	5.00E-06	0.00603935
G	T	0.4559	0.096	0.0175	3437	2.32E-06	0.006469299
C	G	-0.1536	0.034	0.8323	3437	7.77E-06	0.005804167
T	G	0.1378	0.027	0.3945	3437	4.11E-07	0.007428505
C	A	0.2701	0.053	0.0599	3437	4.26E-07	0.007413455
C	T	1.783	0.374	0.001	3437	1.96E-06	0.006565788
G	A	2.218	0.485	0.0006	3437	4.88E-06	0.006055634
C	T	0.1936	0.043	0.0989	3437	8.76E-06	0.005735274
C	T	0.1331	0.03	0.7597	3437	7.11E-06	0.005852447
C	T	0.7369	0.144	0.0077	3437	3.48E-07	0.00753047
G	A	0.6084	0.107	0.0145	3437	1.58E-08	0.009250267
A	C	-0.3904	0.088	0.0215	3437	9.80E-06	0.005671894
A	C	0.4042	0.082	0.0253	3437	9.02E-07	0.006996078
T	C	1.875	0.191	0.0041	3437	1.50E-22	0.027385333
A	C	-0.1952	0.026	0.5911	3437	1.36E-13	0.015810252
T	A	-0.1472	0.033	0.1865	3437	8.32E-06	0.005762685
A	T	0.2465	0.029	0.2587	3437	4.22E-17	0.020354039
T	C	0.1985	0.041	0.1084	3437	1.70E-06	0.006644245
C	G	-0.7964	0.177	0.0055	3437	7.28E-06	0.00583611
T	A	3.735	0.785	0.0006	3437	2.05E-06	0.006536897
C	G	3.798	0.845	0.0003	3437	7.19E-06	0.005844858
T	C	-0.8751	0.198	0.0042	3437	9.83E-06	0.005668312
G	A	-0.7381	0.161	0.0063	3437	4.64E-06	0.006085382
A	T	0.1911	0.043	0.0979	3437	8.77E-06	0.005734884
T	G	-0.4787	0.102	0.0185	3437	2.96E-06	0.0063305
T	C	0.1172	0.026	0.5914	3437	5.47E-06	0.005995796
G	A	2.958	0.586	0.0004	3437	4.72E-07	0.007356423
A	G	0.1734	0.032	0.2082	3437	4.88E-08	0.008619708
G	A	0.2382	0.051	0.0649	3437	3.74E-06	0.006202529
C	G	-0.1904	0.039	0.1219	3437	1.24E-06	0.00682074
C	T	1.473	0.332	0.0013	3437	9.58E-06	0.005681063
G	T	0.4553	0.096	0.0175	3437	2.36E-06	0.006460382
T	G	0.1357	0.027	0.3945	3437	6.15E-07	0.007215979
C	T	1.778	0.374	0.001	3437	2.06E-06	0.006536196
C	A	0.3547	0.071	0.0319	3437	6.17E-07	0.007205117
A	C	0.332	0.072	0.0343	3437	4.41E-06	0.006114467
G	A	2.205	0.484	0.0006	3437	5.53E-06	0.005992651
A	G	-0.4785	0.105	0.0167	3405	4.84E-06	0.006119954
A	G	-1.039	0.194	0.0044	3405	8.77E-08	0.008362026
T	G	-0.189	0.042	0.1154	3405	5.47E-06	0.006048629
G	A	-1.681	0.23	0.0032	3405	3.03E-13	0.015511844
A	C	-1.153	0.238	0.0034	3405	1.31E-06	0.006851205

G	A	0.6392	0.139	0.0091	3405	4.21E-06	0.006198735
A	G	-0.5649	0.121	0.0113	3405	3.02E-06	0.00638134
A	T	0.3065	0.064	0.043	3405	1.59E-06	0.006744977
A	G	1.211	0.274	0.0023	3405	9.98E-06	0.005716521
T	G	2.339	0.523	0.0007	3405	8.12E-06	0.005830907
A	G	-0.1448	0.031	0.2401	3405	2.67E-06	0.006453404
T	C	0.5547	0.057	0.0542	3405	2.40E-22	0.027386693
C	T	-0.9749	0.213	0.0035	3405	4.96E-06	0.006103372
A	G	-0.1913	0.043	0.1004	3405	7.88E-06	0.005849194
A	G	1.578	0.345	0.0016	3405	4.99E-06	0.006106586
T	C	0.9382	0.198	0.004	3405	2.23E-06	0.00655072
A	G	-2.405	0.5	0.0006	3405	1.59E-06	0.006746206
A	G	0.4965	0.101	0.016	3405	1.01E-06	0.007005663
T	C	0.7075	0.144	0.0085	3405	9.40E-07	0.007039515
A	G	0.1475	0.032	0.2085	3405	3.01E-06	0.006386114
T	A	0.88	0.192	0.0047	3405	4.87E-06	0.006118936
C	T	0.1755	0.038	0.132	3405	4.06E-06	0.006218759
C	G	-0.3793	0.076	0.0311	3405	5.43E-07	0.007348126
A	G	-1.737	0.231	0.0029	3437	7.08E-14	0.016171108
C	T	-1.101	0.197	0.0042	3437	2.46E-08	0.009015103
A	G	0.1997	0.045	0.0912	3437	9.85E-06	0.00566972
A	G	-0.3462	0.078	0.0301	3437	9.41E-06	0.005696155
A	G	-0.5247	0.119	0.0113	3437	9.82E-06	0.005671991
A	G	0.2379	0.053	0.0602	3437	7.28E-06	0.005838942
A	G	-0.1201	0.026	0.5921	3437	3.42E-06	0.006260414
C	T	-0.233	0.052	0.0646	3437	6.75E-06	0.005879311
T	C	-0.2794	0.062	0.0441	3437	6.25E-06	0.005923248
C	T	-0.1554	0.033	0.7472	3437	2.74E-06	0.006383705
A	G	-0.3221	0.07	0.0364	3437	3.70E-06	0.006210502
A	G	-0.3386	0.073	0.0298	3437	3.99E-06	0.006171862
T	C	0.1893	0.039	0.1189	3437	1.27E-06	0.006804629
T	C	0.6146	0.055	0.0543	3437	2.78E-28	0.034776307
C	T	-0.2998	0.065	0.0407	3437	4.52E-06	0.006102827
A	G	-0.2286	0.05	0.0662	3437	4.82E-06	0.006064319
A	G	-0.1309	0.027	0.6852	3437	1.75E-06	0.006625456
A	G	-0.2876	0.057	0.0499	3437	5.18E-07	0.007304226
A	G	-2.405	0.495	0.0006	3437	1.24E-06	0.006815836
G	A	0.118	0.027	0.6539	3437	8.94E-06	0.005722917
T	C	0.6387	0.142	0.0084	3437	6.73E-06	0.005884703
T	A	0.5009	0.093	0.0188	3437	6.83E-08	0.008436065
C	G	0.3191	0.066	0.0368	3437	1.65E-06	0.006660707
T	C	0.3478	0.07	0.0343	3437	7.13E-07	0.007125343
A	G	-1.396	0.212	0.0033	3437	4.86E-11	0.012505318
C	T	-0.9442	0.197	0.0042	3437	1.72E-06	0.00663931
A	C	-0.5412	0.12	0.0132	3437	7.19E-06	0.005844367
A	G	-0.4562	0.101	0.0172	3437	6.97E-06	0.005866202
A	G	-1.078	0.191	0.0044	3437	1.83E-08	0.009173482
A	G	0.212	0.045	0.0912	3437	2.66E-06	0.006396321

A	G	-0.3506	0.078	0.0301	3437	7.15E-06	0.005848464
G	C	-0.119	0.025	0.5601	3437	2.95E-06	0.006335821
T	C	-1.731	0.39	0.0009	3437	9.11E-06	0.005710702
C	T	-0.2433	0.052	0.0646	3437	2.56E-06	0.006417064
C	G	-0.1391	0.029	0.2518	3437	2.04E-06	0.006545833
G	A	-0.1371	0.03	0.2912	3437	3.61E-06	0.00622399
C	T	-0.1651	0.037	0.1373	3437	7.27E-06	0.005841066
G	A	-0.1352	0.03	0.2205	3437	8.40E-06	0.005759438
G	C	-0.1789	0.039	0.1263	3437	4.18E-06	0.006144367
T	C	0.1125	0.025	0.4594	3437	6.90E-06	0.005866584
G	A	-0.8361	0.184	0.0049	3437	5.38E-06	0.00600412
G	C	0.1194	0.026	0.3883	3437	4.75E-06	0.006075292
T	C	0.1253	0.027	0.35	3437	3.19E-06	0.006291722
A	G	0.183	0.039	0.1158	3437	3.42E-06	0.006253294
T	C	0.5758	0.055	0.0543	3437	5.32E-25	0.030557751
C	T	-0.2908	0.065	0.0407	3437	8.53E-06	0.00574924
A	G	-0.2579	0.057	0.0499	3437	6.73E-06	0.005881944
A	G	-2.27	0.495	0.0006	3437	4.70E-06	0.006079084
G	C	0.3552	0.078	0.0276	3437	6.01E-06	0.00594277
C	G	0.4724	0.105	0.0147	3437	6.57E-06	0.005888194
T	G	-0.2786	0.062	0.0454	3437	6.68E-06	0.005885785
T	C	0.6334	0.142	0.0084	3437	7.92E-06	0.005788007
T	A	0.4275	0.093	0.0188	3437	4.13E-06	0.006151023
T	C	-0.2771	0.058	0.049	3437	1.72E-06	0.006638116
T	C	0.3269	0.07	0.0343	3437	3.15E-06	0.006301742
C	G	-0.329	0.074	0.031	3437	9.92E-06	0.005664781
A	G	-0.165	0.037	0.134	3437	7.90E-06	0.005796329
G	T	-2.169	0.488	0.0006	3437	9.08E-06	0.005712601
A	C	-0.3536	0.073	0.0301	3405	1.44E-06	0.0067972
C	T	-0.5716	0.129	0.0097	3405	8.90E-06	0.005777562
A	G	1.113	0.249	0.0028	3405	8.28E-06	0.005824254
A	G	0.8168	0.17	0.0053	3405	1.61E-06	0.006734138
G	A	0.1675	0.038	0.1235	3405	9.22E-06	0.005760899
T	C	-0.16	0.036	0.146	3405	7.97E-06	0.005841717
C	T	0.391	0.08	0.0267	3405	9.69E-07	0.007020516
G	T	0.6586	0.144	0.0078	3405	4.77E-06	0.006131149
T	C	0.3563	0.077	0.0276	3405	3.39E-06	0.00631894
A	C	0.6083	0.133	0.0093	3405	5.31E-06	0.006069638
T	C	0.2717	0.056	0.0542	3405	1.10E-06	0.006946918
C	G	-0.175	0.039	0.122	3405	7.11E-06	0.005905602
T	C	0.2496	0.056	0.0523	3405	9.40E-06	0.005749404
C	T	-0.1235	0.027	0.5289	3405	5.13E-06	0.006084595
T	C	-0.4993	0.102	0.0206	3405	1.10E-06	0.006947475
A	G	0.1988	0.041	0.1051	3405	1.07E-06	0.006968357
A	G	-0.1224	0.025	0.4314	3405	1.20E-06	0.006908109
T	G	0.3047	0.065	0.0388	3405	2.87E-06	0.006412204
C	T	0.2109	0.043	0.1035	3405	8.46E-07	0.007096932
A	C	0.1885	0.041	0.1065	3405	3.80E-06	0.006254108

G	A	0.2751	0.061	0.0442	3405	5.83E-06	0.006017842
C	G	-0.1376	0.028	0.7336	3405	1.15E-06	0.006929123
A	G	0.4172	0.093	0.0307	1858	7.05E-06	0.010806921
C	T	0.2177	0.045	0.1485	1858	1.49E-06	0.012385274
C	T	-0.5029	0.103	0.9771	1858	1.20E-06	0.012619516
A	G	1.543	0.175	0.0086	1858	2.30E-18	0.040293836
G	A	1.453	0.181	0.0081	1858	2.00E-15	0.033378544
G	A	-0.7371	0.161	0.0102	1858	4.75E-06	0.01121037
T	C	0.152	0.033	0.5823	1858	3.25E-06	0.011586298
T	C	0.1703	0.036	0.7374	1858	2.34E-06	0.011927055
G	A	-0.4209	0.09	0.0334	1858	2.87E-06	0.011719205
G	C	0.9085	0.198	0.0062	1858	4.59E-06	0.011237835
A	G	-0.2775	0.062	0.0721	1858	7.83E-06	0.010701053
T	C	-0.276	0.061	0.0751	1858	7.11E-06	0.010796398
C	T	0.2534	0.051	0.1082	1858	6.39E-07	0.01326119
G	C	-0.3134	0.07	0.9451	1858	7.43E-06	0.010752106
C	T	0.1647	0.037	0.2484	1858	9.10E-06	0.010546268
T	G	0.1444	0.032	0.4029	1858	8.32E-06	0.010629309
T	C	-1.326	0.277	0.0038	1858	1.78E-06	0.012217944
C	T	0.1462	0.032	0.4378	1858	5.25E-06	0.011102707
G	A	0.2384	0.053	0.0974	1858	5.95E-06	0.010976273
A	G	-0.3383	0.07	0.0584	1857	1.47E-06	0.012414312
G	T	0.499	0.112	0.0221	1857	8.25E-06	0.010651484
G	T	-0.1767	0.038	0.7124	1857	2.84E-06	0.011740714
T	C	-0.1586	0.035	0.6675	1857	7.80E-06	0.010705438
T	G	-0.5264	0.101	0.0293	1857	1.90E-07	0.01450163
T	A	-0.3866	0.074	0.0582	1857	1.79E-07	0.014573917
G	A	0.6111	0.134	0.0148	1857	5.39E-06	0.011091938
T	C	-0.1512	0.034	0.5078	1857	6.94E-06	0.010831654
T	C	-0.1953	0.044	0.1753	1857	8.53E-06	0.010612178
T	C	0.151	0.034	0.5555	1857	9.74E-06	0.010479309
T	C	0.2687	0.056	0.9047	1857	1.86E-06	0.01216866
T	C	-0.8322	0.177	0.0083	1857	2.92E-06	0.0117117
T	C	0.1487	0.033	0.4631	1857	9.06E-06	0.010554772
T	A	-1.55	0.318	0.0027	1857	1.19E-06	0.012624279
T	A	0.1499	0.034	0.4922	1857	9.06E-06	0.010560738
A	G	-1.579	0.355	0.0022	1857	8.97E-06	0.010564823
G	A	-0.7864	0.145	0.0137	1857	6.37E-08	0.015634867
T	G	0.1655	0.037	0.297	1857	8.05E-06	0.010676369
T	C	0.278	0.052	0.1201	1857	8.12E-08	0.015384265
G	A	0.3938	0.032	0.5197	1857	1.25E-32	0.073395487
G	A	0.2453	0.049	0.1314	1857	5.45E-07	0.013423761
T	C	-0.2816	0.047	0.1527	1857	2.20E-09	0.019083854
C	T	-1.442	0.319	0.9973	1857	6.67E-06	0.010870413
T	C	-0.3098	0.069	0.0649	1857	7.86E-06	0.010699075
A	G	-0.2383	0.048	0.1373	1857	9.09E-07	0.012901626
A	C	-0.5379	0.097	0.0307	1857	2.92E-08	0.016436118
T	A	-0.3737	0.072	0.0582	1857	2.72E-07	0.014140097

C	T	-0.167	0.036	0.3024	1857	2.98E-06	0.011685328
C	T	0.2747	0.062	0.074	1857	8.86E-06	0.010578416
C	T	-0.8503	0.186	0.0078	1857	5.36E-06	0.011093333
C	T	-1.976	0.433	0.0019	1857	5.21E-06	0.01111566
G	T	-1.066	0.238	0.9943	1857	7.76E-06	0.010714351
G	A	-0.4335	0.091	0.0369	1857	2.19E-06	0.012004944
T	C	0.5458	0.122	0.0199	1857	7.81E-06	0.010715079
A	G	-0.9907	0.223	0.0059	1857	9.22E-06	0.010535191
T	C	0.4464	0.101	0.0267	1857	9.87E-06	0.010471427
T	A	-1.503	0.314	0.0027	1857	1.85E-06	0.012172362
T	C	-0.675	0.145	0.0132	1857	3.59E-06	0.011503698
T	C	0.1528	0.034	0.6292	1857	8.57E-06	0.010616604
T	G	-0.2101	0.047	0.1373	1857	9.68E-06	0.010491074
T	C	-0.2163	0.046	0.1527	1857	3.25E-06	0.011606306
G	C	0.2248	0.048	0.1314	1857	3.31E-06	0.011582631
G	C	0.5143	0.116	0.0213	1857	9.92E-06	0.010456625
G	A	0.2522	0.033	0.5197	1857	1.86E-14	0.031111413
T	C	-0.1594	0.033	0.5312	1857	1.86E-06	0.012174053
A	G	0.2287	0.051	0.1206	1857	6.70E-06	0.010863982
T	C	0.7558	0.161	0.0105	1858	2.81E-06	0.011750667
G	A	0.1593	0.033	0.4128	1858	1.48E-06	0.012401239
G	A	0.2285	0.047	0.1453	1858	9.69E-07	0.012829594
A	G	-1.096	0.246	0.004	1858	8.54E-06	0.010613011
C	T	0.1643	0.035	0.3103	1858	3.02E-06	0.011668443
A	C	-0.7744	0.168	0.01	1858	4.15E-06	0.011346527
T	G	3.847	0.801	0.0005	1858	1.67E-06	0.012268443
G	T	0.1792	0.037	0.2801	1858	1.01E-06	0.012793133
A	C	0.1809	0.038	0.2672	1858	1.93E-06	0.012119553
C	G	0.4335	0.095	0.0293	1858	5.42E-06	0.011073468
A	C	-0.6389	0.143	0.0137	1858	8.27E-06	0.010644081
G	A	1.586	0.336	0.0022	1858	2.54E-06	0.011842673
A	G	0.564	0.125	0.0178	1858	6.98E-06	0.010821132
G	A	0.6932	0.156	0.01	1858	9.79E-06	0.010475625
A	G	-1.666	0.37	0.0019	1858	7.06E-06	0.01080568
T	A	1.986	0.445	0.9984	1858	8.36E-06	0.010629872
C	T	-2.346	0.438	0.0019	1858	9.67E-08	0.015192075
G	A	-0.1958	0.043	0.1889	1858	4.78E-06	0.011205734
T	C	0.3546	0.074	0.0533	1858	1.59E-06	0.012325878
T	G	-0.1538	0.032	0.4594	1858	2.16E-06	0.012004294
T	A	3.416	0.67	0.0005	1858	3.80E-07	0.013789571
A	G	0.2229	0.047	0.1418	1858	2.00E-06	0.012092423
A	C	0.2117	0.045	0.1671	1858	2.35E-06	0.011928052
G	A	-0.155	0.034	0.6284	1858	6.65E-06	0.010865052
T	G	3.05	0.671	0.0005	1858	5.79E-06	0.011010789
T	C	0.2079	0.044	0.1593	1858	3.10E-06	0.011647229
G	C	0.1693	0.035	0.6631	1858	1.26E-06	0.012563763
A	C	0.1771	0.039	0.2279	1858	5.45E-06	0.011066242
T	C	-0.2048	0.039	0.2177	1858	2.25E-07	0.014333484

G	C	-0.3814	0.083	0.0401	1858	4.13E-06	0.011353044
A	C	-0.3696	0.039	0.2131	1858	4.36E-21	0.046678223
G	T	2.149	0.479	0.0011	1858	7.70E-06	0.010717083
T	C	0.7944	0.162	0.0105	1858	1.06E-06	0.012730094
C	G	0.307	0.068	0.0649	1858	7.38E-06	0.01076327
A	G	-1.134	0.248	0.004	1858	5.19E-06	0.011119131
G	A	0.2171	0.047	0.1453	1858	4.29E-06	0.011310363
C	A	-0.172	0.036	0.6983	1858	1.41E-06	0.012455961
A	G	0.1616	0.035	0.3388	1858	3.77E-06	0.01144022
T	G	3.845	0.806	0.0005	1858	1.97E-06	0.012103091
A	G	0.1855	0.038	0.2672	1858	1.43E-06	0.012429482
C	G	0.4261	0.096	0.0293	1858	9.94E-06	0.010457382
G	A	1.56	0.34	0.0022	1858	4.66E-06	0.011229582
G	A	0.7	0.158	0.01	1858	9.28E-06	0.010519533
C	T	-2.363	0.448	0.0019	1858	1.45E-07	0.014778671
T	A	2.053	0.455	0.9984	1858	6.77E-06	0.010843397
T	A	-0.1477	0.033	0.4233	1858	9.39E-06	0.010514837
T	C	0.4332	0.091	0.0355	1858	1.83E-06	0.012181789
T	G	-0.1489	0.033	0.4594	1858	6.06E-06	0.010956752
C	A	2.986	0.659	0.0005	1858	6.24E-06	0.010925972
A	C	3.028	0.683	0.0005	1858	9.74E-06	0.010476885
C	T	0.3336	0.07	0.0603	1858	2.20E-06	0.011991524
T	A	3.174	0.676	0.0005	1858	2.89E-06	0.01171578
T	A	0.2063	0.044	0.1674	1858	3.77E-06	0.011450303
A	G	-1.602	0.351	0.0022	1858	5.44E-06	0.011068511
G	A	-0.1615	0.035	0.6284	1858	3.43E-06	0.011537228
G	A	0.1822	0.04	0.7734	1858	6.23E-06	0.010929745
G	A	0.2146	0.045	0.152	1858	2.25E-06	0.011965767
G	C	0.1663	0.035	0.6631	1858	2.58E-06	0.011830568
T	G	3.21	0.676	0.0005	1858	2.17E-06	0.012007902
G	T	-1.887	0.422	0.0013	1858	8.04E-06	0.010671937
A	C	0.1559	0.033	0.5326	1858	2.98E-06	0.011686827
A	C	-0.3767	0.039	0.2131	1858	2.13E-21	0.047417743
C	A	0.2421	0.054	0.1074	1858	7.10E-06	0.010793216
A	G	-0.1496	0.034	0.5067	1858	9.41E-06	0.010513351
C	T	-0.262	0.048	0.1389	1858	5.40E-08	0.015788605
C	T	-0.196	0.041	0.2199	1858	2.32E-06	0.011936588
C	T	0.4192	0.09	0.0342	1858	3.58E-06	0.011493691
G	A	0.3392	0.069	0.937	1858	9.57E-07	0.012847068
A	G	-0.2317	0.051	0.1243	1858	5.20E-06	0.011120015
A	G	-0.4891	0.104	0.0269	1858	2.83E-06	0.01174137
G	C	1.103	0.236	0.0054	1858	3.14E-06	0.011619977
A	G	0.1697	0.037	0.267	1858	5.75E-06	0.011017647
A	G	0.1825	0.041	0.2096	1858	9.16E-06	0.010541113
C	A	-1.297	0.285	0.004	1858	5.71E-06	0.011016129
G	A	2.17	0.484	0.0013	1858	7.83E-06	0.010698728
G	A	-0.1819	0.04	0.2333	1858	4.43E-06	0.011273583
A	G	0.2193	0.044	0.1747	1858	6.02E-07	0.013312601

G	A	-1.707	0.365	0.0019	1858	3.02E-06	0.011666213
A	G	-0.3128	0.039	0.2236	1858	2.63E-15	0.033101928
A	C	-0.2749	0.061	0.0823	1858	7.57E-06	0.010735678
T	C	-0.2378	0.051	0.1173	1858	4.02E-06	0.011380025
T	C	-0.3	0.066	0.0659	1858	6.57E-06	0.01087686
G	A	0.2123	0.047	0.1453	1858	5.95E-06	0.010972701
C	T	0.159	0.035	0.3103	1858	6.96E-06	0.010813581
G	T	-0.3759	0.076	0.0492	1858	7.96E-07	0.013032647
A	G	-0.7381	0.148	0.0118	1858	6.82E-07	0.013191898
G	C	-0.662	0.146	0.0126	1858	6.49E-06	0.01088516
G	A	0.9302	0.209	0.0057	1858	8.90E-06	0.010568942
G	A	-0.5483	0.121	0.0196	1858	5.67E-06	0.011020567
A	G	3.667	0.81	0.0005	1858	6.43E-06	0.010899775
C	A	-0.2028	0.046	0.1596	1858	8.88E-06	0.010569898
A	G	-1.725	0.373	0.0019	1858	3.96E-06	0.011392125
T	C	0.5732	0.12	0.0215	1858	1.74E-06	0.012231675
T	C	0.5837	0.13	0.0172	1858	7.04E-06	0.010799607
G	C	0.6809	0.154	0.0118	1858	9.84E-06	0.010465726
C	T	-2.178	0.437	0.0019	1858	6.68E-07	0.01321674
G	C	-0.2209	0.042	0.2034	1858	1.46E-07	0.014773751
T	A	3.383	0.676	0.0005	1858	6.23E-07	0.013288311
A	T	0.292	0.065	0.0675	1858	7.65E-06	0.010722023
T	A	-0.2236	0.05	0.8743	1858	7.09E-06	0.01080233
T	C	0.1603	0.033	0.4774	1858	1.31E-06	0.012525443
A	G	0.4989	0.108	0.025	1858	3.94E-06	0.011396355
C	T	0.158	0.034	0.4241	1858	4.73E-06	0.011213706
A	G	-0.3177	0.07	0.0616	1858	5.24E-06	0.011105659
T	C	-0.2252	0.04	0.2177	1858	1.45E-08	0.017150823
G	C	-0.3785	0.083	0.0401	1858	5.37E-06	0.011079242
A	G	0.1922	0.039	0.2279	1858	9.12E-07	0.012889975
A	C	-0.3278	0.039	0.2131	1858	1.08E-16	0.036359968
A	G	-0.3852	0.084	0.0385	1858	5.22E-06	0.011112672
T	C	0.9334	0.2	0.0065	1858	3.32E-06	0.011575485
A	G	0.2212	0.049	0.1367	1858	7.31E-06	0.010775047
A	G	1.355	0.267	0.0038	1858	4.32E-07	0.013661894
T	C	0.1782	0.034	0.4451	1858	2.09E-07	0.014393522
C	A	0.4352	0.096	0.0307	1858	5.69E-06	0.011023756
C	T	-0.4851	0.102	0.0277	1858	2.18E-06	0.012003851
G	T	1.621	0.336	0.0024	1858	1.55E-06	0.012342825
C	T	0.1569	0.034	0.6122	1858	5.49E-06	0.011053478
G	A	0.3369	0.074	0.0541	1858	6.03E-06	0.010962089
A	G	0.4605	0.104	0.0269	1858	9.88E-06	0.010462005
A	G	0.173	0.035	0.3251	1858	1.03E-06	0.012776295
T	C	1.244	0.224	0.0057	1858	3.04E-08	0.016386119
G	T	0.3264	0.065	0.0705	1858	5.63E-07	0.013385718
A	G	0.1629	0.034	0.4249	1858	2.02E-06	0.01207745
T	G	-0.2597	0.056	0.9058	1858	3.92E-06	0.01140229
G	T	0.1502	0.034	0.5853	1858	9.86E-06	0.010467368

A	T	1.402	0.294	0.0032	1858	1.98E-06	0.012091285
C	T	-0.1645	0.033	0.4467	1858	9.82E-07	0.012826515
C	T	-0.1538	0.034	0.4131	1858	6.87E-06	0.010836303
A	G	-0.8814	0.185	0.0086	1858	2.06E-06	0.012056456
G	C	0.1857	0.04	0.2317	1858	2.79E-06	0.011749813
G	A	0.1584	0.036	0.3173	1858	9.11E-06	0.010542937
T	C	0.6437	0.135	0.0156	1858	2.13E-06	0.012018022
T	C	-0.1839	0.04	0.2115	1858	5.23E-06	0.011105046
A	G	-0.7418	0.162	0.014	1858	4.92E-06	0.011172613
T	C	-0.1576	0.035	0.6459	1858	7.78E-06	0.010709935
G	A	0.1688	0.038	0.2718	1858	7.85E-06	0.010702765
C	G	-0.4264	0.085	0.0371	1858	5.57E-07	0.01340355
T	C	-0.2339	0.051	0.8819	1858	5.67E-06	0.011026612
T	C	-0.27	0.059	0.0845	1858	5.98E-06	0.01096857
G	A	-0.8101	0.174	0.0102	1858	3.37E-06	0.011558013
T	C	-0.309	0.068	0.0667	1858	5.38E-06	0.011081482
A	G	-0.1626	0.037	0.6983	1858	9.21E-06	0.010533773
A	G	0.2146	0.046	0.1534	1858	3.26E-06	0.011593124
A	G	-0.3475	0.077	0.0576	1858	6.21E-06	0.010938296
A	G	0.3739	0.082	0.9553	1858	5.20E-06	0.011117247
A	G	-0.1585	0.033	0.5081	1858	2.35E-06	0.011925893
A	G	-0.3044	0.068	0.0635	1858	7.36E-06	0.010757527
G	A	0.505	0.112	0.0223	1858	7.31E-06	0.010766544
T	C	0.3374	0.072	0.0562	1858	2.66E-06	0.011797212
C	T	-0.6084	0.137	0.0159	1858	9.06E-06	0.010548487
T	G	-0.1904	0.034	0.5737	1858	1.59E-08	0.017048808
T	A	0.6723	0.145	0.0135	1858	3.61E-06	0.011484888
T	C	0.2769	0.062	0.0778	1858	9.37E-06	0.010513688
G	C	-1.312	0.278	0.0038	1858	2.61E-06	0.01182038
G	C	0.4225	0.081	0.046	1858	2.13E-07	0.014386378
G	A	-0.1664	0.036	0.3114	1858	4.19E-06	0.011337014
C	T	-0.648	0.142	0.014	1858	4.97E-06	0.011161353
T	C	1.824	0.412	0.0016	1858	9.93E-06	0.010458925
A	G	-0.4064	0.091	0.0463	1858	9.20E-06	0.010533207
T	G	-0.1588	0.034	0.4325	1858	4.10E-06	0.011358864
A	G	0.1961	0.042	0.2008	1858	3.69E-06	0.011461713
A	G	-2.591	0.56	0.0011	1858	3.99E-06	0.011386351
T	C	-0.1515	0.034	0.3891	1858	9.43E-06	0.010511904
T	A	-1.157	0.257	0.0046	1858	7.18E-06	0.010790536
A	C	-0.1623	0.037	0.2618	1858	9.90E-06	0.010461342
C	G	-1.079	0.225	0.0059	1858	1.70E-06	0.012258416
T	A	0.2837	0.061	0.0786	1858	3.50E-06	0.011522582
G	A	-0.9064	0.202	0.0073	1858	7.83E-06	0.010699415
T	C	-0.4903	0.104	0.0258	1858	2.59E-06	0.011820806
A	G	0.3782	0.084	0.0441	1858	7.83E-06	0.010699158
T	C	2.595	0.585	0.0008	1858	9.67E-06	0.010479536
C	T	-0.2533	0.037	0.2635	1858	1.49E-11	0.024231848
T	G	-0.2077	0.041	0.2088	1858	4.46E-07	0.013630478

A	G	-1.641	0.36	0.0022	1858	5.59E-06	0.011041322
T	C	0.6759	0.14	0.0153	1858	1.55E-06	0.012354474
C	A	0.7117	0.157	0.0118	1858	6.28E-06	0.01092509
T	C	-0.7561	0.16	0.0113	1858	2.46E-06	0.01187638
T	A	0.7644	0.163	0.0105	1858	3.10E-06	0.011641423
A	G	-0.6165	0.137	0.0161	1858	7.21E-06	0.010781312
G	T	-1.575	0.352	0.9976	1858	8.00E-06	0.010678448
A	G	-0.1801	0.038	0.2382	1858	2.96E-06	0.011694571
A	T	-0.6925	0.156	0.0113	1858	9.60E-06	0.010494527
A	G	-0.6728	0.151	0.0126	1858	8.25E-06	0.010641599
G	C	0.678	0.145	0.0143	1858	2.99E-06	0.011678176
A	G	0.3306	0.072	0.0562	1858	5.39E-06	0.011079615
A	C	-1.57	0.322	0.0032	1858	1.13E-06	0.012672228
G	C	-1.037	0.233	0.9949	1858	8.72E-06	0.010593528
T	C	-0.5549	0.118	0.021	1858	2.52E-06	0.011861128
T	C	0.2569	0.056	0.0977	1858	4.06E-06	0.011371942
A	G	-2.109	0.473	0.0013	1858	8.70E-06	0.010595619
A	G	-1.079	0.233	0.0048	1858	3.76E-06	0.011449242
T	A	-0.3009	0.064	0.0721	1858	2.98E-06	0.011684874
A	T	-0.4103	0.032	0.5191	1858	1.73E-35	0.079861356
A	G	-0.1942	0.036	0.6889	1858	6.13E-08	0.015668038
T	A	-0.2049	0.044	0.1792	1858	3.17E-06	0.011615157
T	G	0.1662	0.035	0.2874	1856	1.75E-06	0.01223016
G	A	0.3229	0.072	0.0533	1856	8.62E-06	0.01060935
T	C	-0.5272	0.11	0.9801	1856	1.79E-06	0.012224906
G	A	0.8776	0.18	0.0075	1856	1.18E-06	0.012645711
G	A	-0.1499	0.033	0.6183	1856	6.03E-06	0.010981848
C	T	0.4013	0.09	0.0302	1856	8.99E-06	0.010570676
C	T	-0.271	0.057	0.9173	1856	1.86E-06	0.012171268
A	T	-0.4783	0.106	0.0234	1856	6.41E-06	0.010912084
A	T	0.444	0.099	0.0286	1856	7.01E-06	0.010822442
T	C	0.2723	0.061	0.0725	1856	7.87E-06	0.01070551
A	T	0.579	0.129	0.0145	1856	7.47E-06	0.010754186
C	T	0.1516	0.032	0.4278	1856	2.59E-06	0.011838223
A	C	-0.3815	0.086	0.0334	1856	9.05E-06	0.010559334
A	G	1.784	0.355	0.0019	1856	5.34E-07	0.013454006
T	G	-0.1502	0.034	0.6754	1856	8.33E-06	0.01064576
C	T	0.1463	0.032	0.5229	1856	5.80E-06	0.011020363
T	C	0.3493	0.071	0.0523	1856	1.07E-06	0.012744972
G	A	0.1797	0.038	0.2163	1856	2.76E-06	0.011770492
C	T	-0.2198	0.047	0.1266	1856	3.31E-06	0.011587904
A	C	-0.2687	0.038	0.2128	1856	1.28E-12	0.02676507
T	C	-0.2007	0.036	0.2659	1856	2.20E-08	0.016725104
G	A	0.2617	0.035	0.3334	1858	8.34E-14	0.029554527
T	G	1.666	0.376	0.0019	1858	9.66E-06	0.010483527
T	C	0.1686	0.037	0.7274	1858	4.71E-06	0.011219257
G	A	-0.2861	0.063	0.0753	1858	5.16E-06	0.011120479
G	T	-0.205	0.046	0.1502	1858	7.14E-06	0.010793288

T	C	1.142	0.247	0.0043	1858	3.90E-06	0.011410803
T	C	0.1544	0.035	0.3149	1858	8.78E-06	0.010579714
C	T	0.2853	0.058	0.0826	1858	1.12E-06	0.012682042
T	G	0.1934	0.039	0.7764	1858	5.86E-07	0.013351511
G	A	0.1837	0.038	0.2411	1858	1.30E-06	0.012532073
G	C	-0.5888	0.127	0.0167	1858	3.69E-06	0.011472032
T	C	-0.3383	0.068	0.0597	1858	6.87E-07	0.013184231
T	G	0.8135	0.173	0.0091	1858	2.61E-06	0.011828335
T	C	0.1862	0.038	0.2511	1858	7.68E-07	0.013068095
C	G	-0.3748	0.078	0.0455	1858	1.68E-06	0.012274408
C	A	-0.7728	0.167	0.0094	1858	3.78E-06	0.011434645
G	C	0.514	0.111	0.024	1858	3.94E-06	0.011388799
T	C	-0.5266	0.1	0.0277	1858	1.36E-07	0.014842722
G	A	0.2768	0.06	0.0797	1858	3.81E-06	0.011433995
C	T	1.043	0.221	0.0057	1858	2.66E-06	0.011803505
T	C	0.2146	0.045	0.158	1858	2.13E-06	0.012023466
A	G	0.4746	0.093	0.0307	1858	3.41E-07	0.01390238
T	C	-0.1627	0.033	0.5509	1858	8.79E-07	0.012937082
G	A	-0.721	0.159	0.0102	1858	6.12E-06	0.010945879
A	G	-0.6579	0.135	0.0137	1858	1.28E-06	0.012547379
C	T	-0.1483	0.032	0.5073	1858	5.33E-06	0.011095804
A	G	0.5875	0.115	0.0202	1858	3.95E-07	0.013757581
A	G	-0.4313	0.074	0.9459	1858	7.11E-09	0.01787882
G	A	0.5511	0.034	0.3334	1858	2.77E-54	0.121554458
T	C	0.1883	0.042	0.2021	1858	6.65E-06	0.010865627
T	C	-0.9307	0.19	0.0073	1858	1.07E-06	0.012736282
G	A	-0.3897	0.081	0.0439	1858	1.51E-06	0.012376952
G	C	-0.4467	0.096	0.0355	1858	3.70E-06	0.011462207
A	G	1.001	0.22	0.0065	1858	5.59E-06	0.011029487
A	G	-0.5334	0.113	0.0237	1858	2.34E-06	0.011933551
C	A	-0.1689	0.034	0.4255	1858	8.65E-07	0.012949318
A	G	-0.3509	0.075	0.053	1858	3.12E-06	0.011638122
T	C	-0.1805	0.039	0.2374	1858	5.15E-06	0.011124893
T	G	0.4015	0.09	0.035	1858	9.06E-06	0.010546663
T	A	0.1728	0.037	0.292	1858	4.20E-06	0.011334941
A	T	0.449	0.1	0.0398	1858	7.78E-06	0.010712756
C	T	-0.5933	0.132	0.0161	1858	7.11E-06	0.010788506
A	G	0.6326	0.14	0.0135	1858	6.35E-06	0.010915739
C	G	0.6622	0.145	0.0135	1858	5.53E-06	0.011055373
C	G	0.1619	0.034	0.5353	1858	1.85E-06	0.012162315
G	A	1.816	0.399	0.0016	1858	5.79E-06	0.011004346
C	G	0.2262	0.049	0.145	1858	4.99E-06	0.011158664
A	G	0.3088	0.069	0.0665	1858	9.24E-06	0.010525528
C	T	0.3643	0.08	0.0455	1858	5.93E-06	0.010980458
C	T	0.1845	0.04	0.7505	1858	4.01E-06	0.011388393
T	C	0.361	0.04	0.2209	1858	7.44E-19	0.041458754
A	G	0.1628	0.035	0.3601	1858	4.68E-06	0.011223511
G	C	0.1618	0.036	0.6765	1858	6.26E-06	0.010934525

G	A	0.316	0.068	0.0568	1858	3.63E-06	0.011479244
G	A	-0.1792	0.034	0.3334	1858	1.40E-07	0.014816581
T	C	-1.386	0.307	0.0024	1858	6.79E-06	0.010836929
A	C	-1.045	0.235	0.0046	1858	9.53E-06	0.010504056
T	C	0.1488	0.032	0.4088	1858	3.83E-06	0.011432896
T	C	0.1587	0.034	0.6709	1858	3.22E-06	0.011596847
T	C	-0.4484	0.1	0.0248	1858	8.23E-06	0.010642336
G	A	-0.2554	0.057	0.0918	1858	6.69E-06	0.010858981
C	T	-0.1531	0.033	0.3886	1858	4.51E-06	0.011255407
A	G	-0.1468	0.033	0.644	1858	7.35E-06	0.010763173
C	G	-0.7808	0.168	0.0089	1858	3.38E-06	0.011559915
G	T	-0.3125	0.067	0.0603	1858	3.18E-06	0.011617607
G	T	0.5178	0.11	0.021	1858	2.46E-06	0.011891991
T	C	-0.1483	0.032	0.588	1858	4.50E-06	0.011266659
C	A	0.702	0.147	0.0124	1858	1.87E-06	0.012158048
T	C	-0.1545	0.034	0.3025	1858	6.98E-06	0.010820767
T	G	2.823	0.633	0.0005	1858	8.81E-06	0.01057795
T	C	-0.1474	0.033	0.3356	1858	9.26E-06	0.010528955
A	G	-0.6561	0.119	0.0175	1858	3.83E-08	0.016150653
G	A	-0.9278	0.205	0.0059	1858	6.64E-06	0.010862229
C	G	0.2683	0.06	0.0692	1858	8.77E-06	0.010587972
A	C	0.186	0.042	0.1685	1858	9.07E-06	0.010549443
T	A	-0.5979	0.126	0.0153	1858	2.06E-06	0.012068431
A	G	-0.2949	0.063	0.0659	1858	2.87E-06	0.01172161
A	G	-0.3414	0.038	0.2209	1858	2.20E-19	0.042703668
C	T	0.732	0.164	0.0091	1858	8.27E-06	0.010647076
C	G	-0.7235	0.159	0.0102	1858	5.72E-06	0.01102108
T	C	3.054	0.635	0.0005	1858	1.61E-06	0.012315368
T	C	-0.1576	0.031	0.5054	1858	5.88E-07	0.013343435
A	G	0.4291	0.074	0.9459	1858	8.70E-09	0.01767201
T	C	0.9251	0.19	0.0073	1858	1.26E-06	0.01255929
G	A	-0.5517	0.034	0.3334	1858	2.53E-54	0.121662646
G	A	0.3752	0.081	0.0439	1858	3.72E-06	0.011458162
A	G	0.5212	0.113	0.0237	1858	4.03E-06	0.011380062
G	C	0.4393	0.096	0.0355	1858	5.44E-06	0.011071555
C	A	0.1559	0.034	0.4255	1858	5.68E-06	0.01101559
A	G	0.3458	0.075	0.053	1858	4.38E-06	0.01128822
C	T	1.63	0.363	0.0022	1858	7.72E-06	0.010712289
A	G	0.1667	0.036	0.6359	1858	2.87E-06	0.011722042
T	C	-0.3179	0.07	0.0619	1858	5.84E-06	0.010997187
C	A	0.6389	0.142	0.014	1858	6.93E-06	0.010823166
C	T	1.058	0.235	0.0054	1858	6.85E-06	0.010836968
A	G	-0.625	0.14	0.0135	1858	8.32E-06	0.010642732
C	G	-0.6684	0.145	0.0135	1858	4.58E-06	0.011245706
G	C	-0.4089	0.089	0.0439	1858	4.64E-06	0.011228181
A	G	-0.1552	0.034	0.5347	1858	5.11E-06	0.011135426
C	G	-0.2299	0.049	0.145	1858	3.53E-06	0.011513241
G	A	-1.847	0.4	0.0016	1858	4.07E-06	0.011367682

T	C	0.2458	0.054	0.109	1858	6.00E-06	0.010972108
C	T	-0.1808	0.04	0.7505	1858	6.27E-06	0.010919474
T	C	-0.3541	0.04	0.2209	1858	3.71E-18	0.039818746
T	C	0.9203	0.204	0.0073	1858	6.69E-06	0.010855863
A	G	-0.1656	0.035	0.3601	1858	3.23E-06	0.011601907
G	C	-0.162	0.036	0.6765	1858	6.18E-06	0.01094308
T	C	-0.1795	0.04	0.7826	1858	9.54E-06	0.010497661
T	G	0.189	0.036	0.2871	1858	2.37E-07	0.014271787
C	T	0.2179	0.048	0.1426	1858	5.73E-06	0.011015073
A	C	0.2119	0.046	0.1512	1858	4.19E-06	0.011340639
G	C	0.1999	0.042	0.8089	1858	2.50E-06	0.011866002
A	G	1.002	0.223	0.0051	1858	7.65E-06	0.010730403
A	T	0.4988	0.104	0.0285	1858	1.54E-06	0.012346141
T	A	-0.1528	0.033	0.5579	1858	4.73E-06	0.011218498
T	G	-0.3584	0.08	0.0436	1858	7.25E-06	0.010777148
A	G	1.74	0.372	0.0019	1858	3.10E-06	0.011644308
G	A	0.3401	0.07	0.0584	1858	1.08E-06	0.012720758
A	C	0.1498	0.034	0.5982	1858	8.96E-06	0.010559795
G	A	0.2036	0.04	0.2182	1858	4.56E-07	0.013604321
T	C	0.3421	0.075	0.0522	1858	5.59E-06	0.011041875
T	C	-0.1502	0.034	0.387	1858	8.83E-06	0.010578298
C	A	0.8998	0.199	0.0073	1858	6.16E-06	0.01093827
G	A	0.1792	0.034	0.5	1858	1.01E-07	0.01515821
T	C	0.2566	0.058	0.0912	1858	8.65E-06	0.010593841
A	G	-0.2997	0.068	0.0614	1858	9.83E-06	0.010464948
T	C	-0.1687	0.036	0.3032	1855	2.96E-06	0.011706005
C	T	0.485	0.108	0.0245	1855	7.85E-06	0.010715349
G	A	-0.2164	0.047	0.8423	1855	3.99E-06	0.011399488
G	A	-0.1561	0.034	0.4941	1855	3.91E-06	0.011427544
C	T	1.28	0.276	0.0035	1855	3.63E-06	0.011502923
G	A	0.3668	0.04	0.4372	1855	8.99E-20	0.043677951
T	C	0.4161	0.039	0.5755	1855	5.46E-26	0.058182098
T	C	-0.2405	0.038	0.3892	1855	2.30E-10	0.0214451
A	G	-0.4389	0.07	0.0749	1855	4.31E-10	0.020799647
A	G	-0.1533	0.033	0.5245	1855	3.33E-06	0.011596833
T	C	-0.3141	0.068	0.0652	1855	3.55E-06	0.011528233
A	G	0.1972	0.044	0.1717	1855	7.76E-06	0.010722049
C	G	-0.1727	0.036	0.2984	1855	2.20E-06	0.012009008
A	C	0.3114	0.061	0.0806	1855	3.45E-07	0.013912404
T	A	0.2114	0.048	0.858	1855	9.41E-06	0.010525416
A	C	-0.1714	0.037	0.6873	1855	2.93E-06	0.011722481
T	C	0.2011	0.044	0.1644	1855	5.40E-06	0.011095614
T	C	0.1531	0.033	0.5539	1855	4.72E-06	0.011226674
G	T	-0.4306	0.038	0.2237	1855	1.72E-28	0.063949966
T	C	2.431	0.422	0.0016	1856	9.41E-09	0.017598663
C	A	0.5622	0.126	0.0178	1856	8.75E-06	0.010596127
G	T	-0.1921	0.038	0.2487	1856	6.47E-07	0.013256734
A	C	-0.4834	0.108	0.0253	1856	8.73E-06	0.010601034

A	G	0.5996	0.133	0.0154	1856	7.23E-06	0.010799931
G	A	0.1522	0.034	0.4092	1856	8.00E-06	0.010687655
G	A	2.721	0.578	0.0008	1856	2.69E-06	0.011795598
C	G	2.675	0.575	0.0008	1856	3.55E-06	0.011522577
C	T	0.1861	0.039	0.7368	1856	2.18E-06	0.012009876
C	T	0.1713	0.037	0.2788	1856	4.13E-06	0.011362145
A	G	0.1756	0.035	0.3661	1856	6.59E-07	0.013238679
A	G	0.1574	0.034	0.4634	1856	3.26E-06	0.011603462
G	A	3.19	0.709	0.0005	1856	7.22E-06	0.010795483
G	A	2.433	0.495	0.0011	1856	9.75E-07	0.012833934
T	C	-0.8296	0.181	0.0084	1856	4.90E-06	0.011192169
T	C	-0.5472	0.112	0.0251	1856	1.19E-06	0.012630898
A	C	-0.4877	0.106	0.0269	1856	4.64E-06	0.011234962
G	A	0.1884	0.04	0.7826	1856	3.35E-06	0.011570099
A	G	2.118	0.477	0.0016	1856	9.48E-06	0.010515461
G	A	-0.2752	0.057	0.0975	1856	1.22E-06	0.012608124
C	G	-0.4747	0.039	0.215	1856	3.74E-33	0.074629843
T	C	-0.5108	0.067	0.0673	1856	5.44E-14	0.030025699
C	T	0.183	0.039	0.2492	1856	2.66E-06	0.011813567
A	G	-0.1645	0.033	0.4701	1856	8.35E-07	0.012992941
A	G	0.4891	0.11	0.0218	1856	8.87E-06	0.010577775
T	C	2.421	0.42	0.0016	1857	9.84E-09	0.017545459
A	C	-0.48	0.108	0.0253	1857	9.47E-06	0.010505879
G	T	-0.1968	0.038	0.2488	1857	3.13E-07	0.013997125
T	C	-0.1465	0.033	0.441	1857	8.40E-06	0.010628575
C	G	2.755	0.573	0.0008	1857	1.64E-06	0.012299821
G	A	2.771	0.576	0.0008	1857	1.65E-06	0.012296751
G	A	-0.1558	0.035	0.6319	1857	9.78E-06	0.010480716
A	G	0.1788	0.035	0.3659	1857	3.75E-07	0.013812077
C	T	0.1906	0.039	0.7369	1857	1.15E-06	0.012666473
A	G	-0.164	0.036	0.3045	1857	4.90E-06	0.011180709
A	G	0.1573	0.034	0.4631	1857	3.04E-06	0.011664639
G	A	3.266	0.707	0.0005	1857	4.07E-06	0.011370611
G	A	2.432	0.494	0.0011	1857	8.99E-07	0.012909162
A	C	-0.5318	0.106	0.0269	1857	5.45E-07	0.013422842
T	C	-0.5889	0.112	0.025	1857	1.59E-07	0.014695404
A	G	2.14	0.475	0.0016	1857	7.06E-06	0.010807528
A	C	-0.4855	0.039	0.2132	1857	7.51E-35	0.078465469
G	A	-0.2977	0.056	0.0975	1857	1.37E-07	0.014838524
A	G	-0.2897	0.062	0.0765	1857	2.77E-06	0.011762591
T	C	-0.5045	0.067	0.0673	1857	9.46E-14	0.029439869
G	A	-0.1881	0.038	0.2722	1857	6.70E-07	0.013221247
C	T	0.1818	0.039	0.2493	1857	2.87E-06	0.011732209
A	G	-0.1591	0.033	0.4698	1857	1.72E-06	0.012244718
A	G	0.496	0.109	0.0218	1857	6.13E-06	0.010948028
G	T	-0.1914	0.043	0.1734	1857	9.09E-06	0.01055179
A	G	0.2733	0.061	0.0805	1857	9.18E-06	0.010539549
A	G	0.1763	0.037	0.2765	1857	1.50E-06	0.012387395

T	C	0.1576	0.033	0.479	1857	1.74E-06	0.012242778
A	T	-0.7043	0.156	0.0108	1857	6.82E-06	0.010843332
G	C	-0.5982	0.13	0.0159	1857	4.75E-06	0.011222527
G	A	-0.2561	0.056	0.9025	1857	5.06E-06	0.011144848
T	G	0.157	0.033	0.4297	1857	2.10E-06	0.012049197
G	A	-0.4165	0.087	0.0382	1857	1.79E-06	0.01221077
T	C	0.1791	0.038	0.2477	1857	3.49E-06	0.011525215
T	C	0.1482	0.033	0.4432	1857	6.39E-06	0.010913435
C	T	-0.1764	0.04	0.2216	1857	9.82E-06	0.010478066
A	C	0.3003	0.067	0.0652	1857	8.37E-06	0.010636212
T	C	-0.7626	0.171	0.0089	1857	8.69E-06	0.010596512
C	T	0.2465	0.053	0.1042	1857	4.20E-06	0.011340276
G	A	-0.2756	0.037	0.2722	1857	1.37E-13	0.029071656
T	G	0.8056	0.148	0.0129	1857	5.32E-08	0.015809643
A	C	-0.5183	0.038	0.2132	1857	1.14E-40	0.091583184
T	C	0.2541	0.046	0.1527	1857	3.85E-08	0.016152216
A	C	0.1584	0.033	0.5533	1857	2.31E-06	0.011945562
G	C	-0.1436	0.032	0.448	1857	9.20E-06	0.010538043
C	T	-2.99	0.662	0.0003	3395	6.48E-06	0.005974689
A	G	-1.857	0.267	0.0032	3395	4.24E-12	0.014048089
C	T	0.1354	0.027	0.466	3395	3.57E-07	0.007602461
T	C	0.2941	0.064	0.0442	3395	3.97E-06	0.006247342
G	A	-0.3399	0.074	0.0308	3395	4.49E-06	0.006179338
C	A	0.1712	0.037	0.1445	3395	4.93E-06	0.006130862
C	T	-0.138	0.027	0.6415	3395	3.51E-07	0.007619118
T	G	2.335	0.527	0.0007	3395	9.82E-06	0.005742715
G	C	0.2331	0.049	0.076	3395	1.99E-06	0.006635111
C	T	-0.9595	0.214	0.0035	3395	7.47E-06	0.005897473
G	A	0.1756	0.036	0.1558	3395	9.68E-07	0.007040723
T	C	-0.1619	0.028	0.5287	3395	1.18E-08	0.009528027
A	G	-0.1295	0.026	0.4315	3395	6.29E-07	0.007282004
G	C	-0.2354	0.047	0.0829	3395	5.98E-07	0.007316111
A	G	0.7099	0.159	0.0069	3395	7.78E-06	0.005874047
C	T	-0.3058	0.064	0.0439	3395	1.74E-06	0.006711027
G	A	-0.3758	0.085	0.0237	3395	9.62E-06	0.005751446
C	T	-0.8093	0.182	0.0042	3427	9.42E-06	0.005711729
A	G	-0.1566	0.031	0.1882	3427	6.00E-07	0.007242178
C	G	-0.3913	0.085	0.0201	3427	4.70E-06	0.006097392
C	G	-0.4267	0.094	0.0168	3427	6.01E-06	0.005961705
G	C	-0.3839	0.085	0.0193	3427	6.77E-06	0.005893614
T	G	-2.021	0.266	0.0032	3427	3.46E-14	0.016626769
T	C	-0.4158	0.094	0.0159	3427	9.44E-06	0.005710879
A	G	-1.081	0.176	0.0044	3427	8.87E-10	0.010900479
C	T	-0.1402	0.03	0.1959	3427	3.14E-06	0.006320011
A	C	0.1345	0.024	0.5909	3427	3.92E-08	0.008767166
G	T	0.2888	0.062	0.0376	3427	3.94E-06	0.006193853
G	A	-0.1902	0.026	0.2969	3427	3.13E-13	0.015387208
T	C	-0.425	0.092	0.0171	3427	3.81E-06	0.006212732

A	G	0.192	0.04	0.0943	3427	1.89E-06	0.006602544
T	C	-0.1246	0.028	0.2613	3427	9.25E-06	0.005716725
G	A	0.1329	0.029	0.2022	3427	5.94E-06	0.005971671
A	C	-0.1075	0.024	0.4181	3427	6.87E-06	0.005883493
G	A	-0.1619	0.034	0.782	3427	1.51E-06	0.006725304
T	C	0.4277	0.092	0.0177	3427	3.35E-06	0.006285979
A	G	0.1892	0.042	0.087	3427	6.61E-06	0.00590897
T	C	0.6089	0.134	0.9931	3427	5.37E-06	0.00602477
C	G	0.6888	0.139	0.0067	3427	8.18E-07	0.007073975
G	T	-0.1145	0.026	0.6733	3427	7.58E-06	0.005835172
C	G	-0.2543	0.056	0.0461	3427	4.95E-06	0.006067169
A	G	-0.1661	0.037	0.1103	3427	8.12E-06	0.005796277
T	C	-0.1119	0.024	0.3826	3427	5.04E-06	0.00606015
T	C	0.3128	0.065	0.0328	3427	1.82E-06	0.006624892
G	T	0.1373	0.031	0.1773	3427	7.22E-06	0.005859389
T	C	-0.1152	0.024	0.5057	3427	1.77E-06	0.006639655
T	A	-0.1648	0.032	0.1533	3427	3.05E-07	0.007618292
A	G	-0.9339	0.179	0.0044	3427	1.79E-07	0.007915392
T	G	-1.991	0.269	0.0032	3427	1.78E-13	0.015699414
G	C	-0.3206	0.068	0.0306	3427	2.42E-06	0.006465236
G	T	-0.13	0.026	0.3166	3427	5.66E-07	0.007281048
G	A	-0.1468	0.031	0.2015	3427	1.90E-06	0.006606464
A	G	0.1112	0.024	0.4444	3427	5.39E-06	0.006014289
G	A	-0.1632	0.026	0.2969	3427	7.82E-10	0.010978731
G	A	-3.149	0.65	0.0003	3427	1.30E-06	0.006812463
G	C	-0.112	0.024	0.5601	3427	3.31E-06	0.006288572
G	T	-0.1152	0.025	0.6344	3427	4.36E-06	0.006143176
A	G	0.1825	0.041	0.0944	3427	8.06E-06	0.005798824
A	G	-2.198	0.439	0.0006	3427	5.73E-07	0.007271708
A	G	-0.3731	0.083	0.0207	3427	8.03E-06	0.005799064
G	A	-0.1842	0.034	0.782	3427	9.54E-08	0.008273299
C	T	-0.7845	0.169	0.0048	3427	3.41E-06	0.00627061
T	C	0.1684	0.033	0.7693	3427	4.55E-07	0.007402763
T	C	-4.107	0.77	0.0004	3427	1.02E-07	0.008235223
A	G	-0.3683	0.08	0.0229	3427	4.45E-06	0.006126748
G	A	0.1562	0.029	0.7785	3427	8.59E-08	0.008325957
C	A	0.4998	0.109	0.0117	3427	5.00E-06	0.006064518
A	G	-0.4646	0.101	0.0144	3427	4.12E-06	0.006172998
T	G	0.3353	0.075	0.0261	3427	9.16E-06	0.005726774
T	A	0.1317	0.03	0.2235	3427	8.76E-06	0.005755038
C	T	-0.6934	0.143	0.0077	3427	1.37E-06	0.006785847
T	C	-0.3734	0.084	0.0225	3427	9.51E-06	0.005707261
T	C	0.1905	0.041	0.0925	3427	3.28E-06	0.006293608
A	G	0.1388	0.029	0.2164	3427	1.64E-06	0.006685837
G	A	-0.6057	0.125	0.0083	3427	1.32E-06	0.006804804
C	T	-0.1697	0.038	0.1106	3427	7.31E-06	0.00585297
T	C	0.1224	0.026	0.4633	3395	1.65E-06	0.006740705
A	G	-1.329	0.261	0.0032	3395	3.83E-07	0.007567714

C	T	0.1343	0.03	0.2443	3395	5.73E-06	0.00604732
A	G	-0.1204	0.027	0.5577	3395	9.06E-06	0.005788879
A	G	1.777	0.383	0.001	3395	3.63E-06	0.006300754
A	T	0.3714	0.083	0.0233	3395	7.74E-06	0.005874443
A	G	-0.6098	0.115	0.0115	3395	1.07E-07	0.008285349
A	G	0.1821	0.035	0.7477	3395	1.83E-07	0.007982577
C	T	-0.1483	0.032	0.2954	3395	2.98E-06	0.006409226
C	T	-0.2038	0.044	0.0887	3395	4.42E-06	0.006186967
T	A	0.2519	0.053	0.0617	3395	1.87E-06	0.006669614
A	T	0.2327	0.051	0.0663	3395	5.77E-06	0.006038158
C	T	0.4171	0.08	0.0267	3395	1.87E-07	0.007968903
G	A	0.2507	0.056	0.0539	3395	8.64E-06	0.005812771
A	G	-0.2997	0.067	0.0368	3395	8.59E-06	0.005817607
C	G	0.6116	0.131	0.0091	3395	2.87E-06	0.006427965
C	T	-0.667	0.146	0.9922	3395	5.40E-06	0.006076906
C	T	-0.1609	0.027	0.5292	3395	2.98E-09	0.01031419
G	A	0.1592	0.035	0.1558	3395	4.98E-06	0.006119599
C	T	0.4832	0.09	0.0206	3395	7.65E-08	0.008476741
T	C	-0.4084	0.088	0.0209	3395	3.84E-06	0.006267199
C	T	-0.1525	0.034	0.8293	3395	8.03E-06	0.005856528
T	C	0.2554	0.057	0.0495	3395	6.84E-06	0.005945012
A	G	-0.1159	0.025	0.4315	3395	4.48E-06	0.00617734
A	G	-0.2959	0.067	0.0387	3395	9.04E-06	0.005787656
T	C	0.1872	0.041	0.1081	3395	4.09E-06	0.006229225
A	G	0.3231	0.066	0.0376	3395	1.18E-06	0.006932173
G	A	-0.2251	0.05	0.0645	3395	8.44E-06	0.005825153
T	C	0.4463	0.099	0.0169	3395	6.42E-06	0.005980462
T	G	0.4028	0.085	0.0219	3427	2.47E-06	0.006455699
A	G	0.3172	0.069	0.035	3427	3.94E-06	0.006196569
A	G	-1.093	0.211	0.0034	3427	2.35E-07	0.007769159
T	C	-0.2079	0.041	0.1058	3427	5.22E-07	0.007322377
T	C	-0.7811	0.168	0.0054	3427	3.56E-06	0.006253495
G	A	0.4075	0.087	0.022	3427	2.60E-06	0.006426977
C	T	0.1714	0.037	0.8567	3427	4.26E-06	0.006150007
T	C	-0.5822	0.126	0.0104	3427	4.17E-06	0.006162247
C	T	-0.1726	0.026	0.4704	3427	1.82E-11	0.013090757
C	T	0.1451	0.032	0.1897	3427	5.64E-06	0.00599355
A	G	0.3214	0.07	0.033	3427	4.19E-06	0.006159288
T	G	-0.2065	0.041	0.1048	3427	5.52E-07	0.007294687
A	G	0.1142	0.026	0.5505	3427	8.65E-06	0.005755397
A	G	0.2024	0.044	0.0909	3427	3.47E-06	0.006266141
G	C	0.1162	0.026	0.4145	3427	7.35E-06	0.005848201
T	G	0.2645	0.057	0.0486	3427	3.78E-06	0.006220182
G	A	0.1154	0.026	0.4365	3427	6.46E-06	0.005917498
G	T	0.2815	0.06	0.0474	3427	3.05E-06	0.006337882
C	T	0.4483	0.099	0.0166	3427	5.83E-06	0.005975447
T	C	-6.344	1.431	0.0001	3427	9.64E-06	0.005702292
C	T	0.4028	0.089	0.0209	3427	6.96E-06	0.005878309

C	A	0.2116	0.047	0.0751	3427	7.96E-06	0.005805854
C	T	0.5892	0.129	0.0098	3427	4.95E-06	0.00606926
C	A	-0.1611	0.035	0.1574	3427	3.74E-06	0.006225232
A	G	0.119	0.026	0.3947	3427	4.88E-06	0.00608021
T	C	0.4341	0.094	0.02	3427	3.91E-06	0.006199065
A	G	-0.3446	0.073	0.0303	3427	2.36E-06	0.006479735
T	A	0.346	0.067	0.0369	3427	3.01E-07	0.007631175
T	C	0.1856	0.04	0.1091	3427	3.99E-06	0.006187659
A	T	0.9885	0.219	0.0034	3427	6.48E-06	0.005920599
T	G	0.1529	0.034	0.1711	3427	5.70E-06	0.005988556
A	G	-1.253	0.264	0.0023	3427	2.17E-06	0.006530325
T	C	-0.1216	0.027	0.3204	3427	9.03E-06	0.005739265
T	C	-0.1924	0.042	0.1058	3427	4.79E-06	0.006086199
T	C	0.1218	0.026	0.3881	3427	2.25E-06	0.006506409
T	G	1.287	0.291	0.0018	3427	9.83E-06	0.005690799
C	T	-0.1403	0.026	0.4704	3427	7.73E-08	0.008393156
G	T	0.2413	0.05	0.0667	3427	1.33E-06	0.006801481
A	G	0.2134	0.043	0.098	3427	6.09E-07	0.007232082
G	C	0.4581	0.094	0.0196	3427	1.13E-06	0.006889859
T	C	-0.131	0.03	0.2572	3427	9.36E-06	0.005713567
T	G	0.3613	0.081	0.0251	3427	8.94E-06	0.005741103
T	C	-0.1343	0.029	0.2669	3427	5.38E-06	0.006019506
A	G	0.2494	0.053	0.0603	3427	2.39E-06	0.006473209
T	C	0.1194	0.026	0.4281	3427	3.30E-06	0.006292971
G	A	0.4412	0.097	0.0179	3427	5.04E-06	0.006057641
G	A	0.1171	0.026	0.4365	3427	6.46E-06	0.005915859
C	T	0.1821	0.041	0.1126	3427	8.35E-06	0.005779208
T	A	-0.1353	0.029	0.3014	3427	2.62E-06	0.006421081
G	T	0.3096	0.061	0.0474	3427	4.21E-07	0.00744367
C	G	-0.2293	0.05	0.0702	3427	5.45E-06	0.006017916
C	T	0.4485	0.091	0.0209	3427	7.92E-07	0.007089144
G	A	0.257	0.058	0.0515	3427	9.74E-06	0.005694639
T	A	0.1443	0.032	0.2235	3427	6.11E-06	0.005946538
A	G	0.1198	0.026	0.3947	3427	5.94E-06	0.005972964
C	A	-0.1373	0.029	0.3439	3427	1.71E-06	0.006656976
T	G	0.1552	0.034	0.1711	3427	5.76E-06	0.005987225
A	G	0.1465	0.033	0.1901	3427	9.11E-06	0.005728334
A	G	0.182	0.04	0.0946	3395	4.33E-06	0.006201944
C	T	0.5648	0.126	0.0088	3395	7.13E-06	0.005920948
G	T	0.1278	0.028	0.785	3395	6.67E-06	0.005954271
T	G	0.2011	0.043	0.082	3395	3.18E-06	0.006374616
G	A	-0.235	0.052	0.0524	3395	7.41E-06	0.005898308
A	G	0.1144	0.026	0.2875	3395	7.75E-06	0.005870478
G	C	1.462	0.329	0.001	3395	9.05E-06	0.005782883
T	C	-0.1878	0.037	0.4189	3395	2.88E-07	0.007724746
T	G	0.7369	0.166	0.0053	3395	9.75E-06	0.005743401
A	G	0.2514	0.034	0.7751	3395	2.62E-13	0.015630802
A	G	-0.2185	0.034	0.2239	3395	9.33E-11	0.01228847

C	T	0.2896	0.064	0.0368	3395	6.56E-06	0.00596712
C	T	-0.306	0.069	0.0321	3395	9.83E-06	0.005739786
A	G	0.2105	0.041	0.0891	3395	3.80E-07	0.007568225
G	A	0.3446	0.076	0.0256	3395	5.66E-06	0.006052439
C	G	-0.1717	0.036	0.1214	3395	2.63E-06	0.006482858
C	T	-0.1176	0.026	0.5292	3395	6.62E-06	0.005962506
G	A	0.1126	0.025	0.6707	3395	8.93E-06	0.005791447
T	G	0.1204	0.027	0.2483	3395	8.61E-06	0.005818749
A	G	0.1158	0.026	0.295	3395	7.48E-06	0.005894322
T	C	-0.4664	0.102	0.014	3395	4.76E-06	0.006144759
G	C	-0.3213	0.066	0.0337	3395	1.18E-06	0.00693014
C	T	0.3114	0.063	0.0358	3395	9.13E-07	0.007075693
C	A	0.2122	0.043	0.0811	3395	1.01E-06	0.007021252
A	G	-1.11	0.243	0.0024	3395	4.87E-06	0.006133533
T	G	0.251	0.056	0.0457	3395	6.33E-06	0.005988432
A	G	0.8342	0.172	0.0044	3427	1.33E-06	0.006793526
T	G	1.495	0.26	0.0032	3427	1.01E-08	0.009526409
A	G	0.141	0.031	0.1882	3427	4.31E-06	0.00614544
C	G	0.4343	0.083	0.0201	3427	1.98E-07	0.007863829
C	G	0.4446	0.092	0.0168	3427	1.39E-06	0.006775925
G	A	0.1457	0.025	0.2969	3427	1.18E-08	0.009451094
A	C	-0.1137	0.024	0.5909	3427	2.04E-06	0.006560733
G	T	-0.2955	0.061	0.0376	3427	1.36E-06	0.006790013
C	G	0.1061	0.024	0.3843	3427	9.02E-06	0.005732119
G	A	0.2463	0.054	0.0474	3427	5.79E-06	0.005980949
G	C	0.1421	0.032	0.152	3427	9.94E-06	0.005685749
T	G	0.1596	0.034	0.132	3427	3.23E-06	0.006307304
C	G	0.6384	0.132	0.0071	3427	1.39E-06	0.006779063
C	G	-0.1823	0.037	0.1094	3427	6.46E-07	0.007198989
G	A	-0.1115	0.024	0.3713	3427	2.66E-06	0.006411785
C	T	-0.2454	0.051	0.1083	3427	1.45E-06	0.006747486
T	A	0.2324	0.033	0.7798	3427	3.60E-12	0.014005015
C	G	-0.2104	0.036	0.1996	3427	8.06E-09	0.00966045
T	C	-0.1661	0.035	0.4183	3427	2.53E-06	0.006440908
C	G	0.3616	0.076	0.0251	3427	1.93E-06	0.006591564
A	G	-0.6472	0.145	0.0061	3427	8.04E-06	0.005803608
C	G	-0.6285	0.136	0.0067	3427	4.18E-06	0.006166213
A	G	0.1668	0.036	0.1103	3427	4.53E-06	0.006120112
T	C	0.1091	0.024	0.3826	3427	5.25E-06	0.006028702
T	C	0.1185	0.026	0.2526	3427	7.30E-06	0.005858019
T	C	-0.3196	0.064	0.0328	3427	6.03E-07	0.007239929
G	A	0.799	0.18	0.0039	3427	8.98E-06	0.005735683
C	A	0.1072	0.024	0.3251	3427	9.92E-06	0.005679304
T	C	-0.1222	0.024	0.4246	3427	2.80E-07	0.007672237
G	A	-0.1384	0.031	0.1707	3427	7.64E-06	0.005831027
G	C	-0.286	0.064	0.0341	3427	9.38E-06	0.005711495
G	C	-0.1455	0.031	0.8462	3427	3.56E-06	0.006250158
C	T	0.284	0.062	0.0357	3427	4.51E-06	0.006118692

G	A	0.1057	0.024	0.6265	3427	9.13E-06	0.005732124
T	G	0.3785	0.084	0.0212	3427	6.39E-06	0.005924713
T	C	0.1592	0.035	0.1228	3427	4.97E-06	0.006062803
A	G	-1.792	0.264	0.0032	3395	1.25E-11	0.013429918
A	G	-0.8572	0.192	0.0044	3395	8.25E-06	0.005836866
C	T	-3.037	0.653	0.0003	3395	3.45E-06	0.006327045
T	A	-0.1485	0.026	0.5459	3395	1.24E-08	0.009495574
C	T	-0.4619	0.102	0.0169	3395	5.93E-06	0.006027469
C	G	-0.3012	0.062	0.0479	3395	1.16E-06	0.006936934
T	C	-0.6847	0.153	0.0069	3395	7.33E-06	0.005902692
G	A	0.1635	0.036	0.151	3395	7.27E-06	0.005910945
A	C	0.2305	0.049	0.0725	3395	3.24E-06	0.006364262
A	T	-0.3061	0.069	0.0446	3395	8.37E-06	0.005832101
G	A	-0.3637	0.073	0.0308	3395	6.56E-07	0.007262291
C	T	-0.1312	0.027	0.6415	3395	9.34E-07	0.007067252
T	C	-2.355	0.52	0.0004	3395	6.21E-06	0.005998209
G	A	-0.5672	0.124	0.01	3395	4.78E-06	0.006144895
A	G	-0.3035	0.064	0.0411	3395	2.36E-06	0.006539704
A	G	-0.3431	0.077	0.0296	3395	7.96E-06	0.005859475
A	T	-0.241	0.054	0.058	3395	9.55E-06	0.005758206
A	G	-0.3605	0.081	0.0252	3395	9.53E-06	0.005758133
T	C	0.3907	0.088	0.0228	3395	8.32E-06	0.005833018
A	G	-0.3375	0.076	0.0293	3395	9.33E-06	0.005769136
T	C	-0.1714	0.038	0.1283	3395	7.12E-06	0.005919671
T	G	-0.2226	0.048	0.076	3395	3.90E-06	0.006255939
C	G	0.1203	0.026	0.4315	3395	3.07E-06	0.006397709
C	T	-0.3209	0.063	0.0439	3395	3.68E-07	0.007586632
G	A	-0.3978	0.082	0.0247	3395	1.27E-06	0.006891009
A	G	-1.535	0.338	0.0012	3427	5.96E-06	0.005968184
T	G	-1.617	0.27	0.0032	3427	2.30E-09	0.010365139
A	G	-0.9188	0.179	0.0044	3427	2.82E-07	0.007663443
C	G	-0.4464	0.086	0.0201	3427	2.55E-07	0.00772392
G	A	-0.1199	0.026	0.2969	3427	6.12E-06	0.005951466
A	G	-0.135	0.027	0.2628	3427	6.93E-07	0.007168189
G	A	-0.1735	0.038	0.1112	3427	5.97E-06	0.005964811
A	G	-0.2812	0.062	0.0379	3427	6.61E-06	0.005907826
A	G	0.1136	0.026	0.6487	3427	8.85E-06	0.005744325
T	C	-1.26	0.263	0.0018	3427	1.66E-06	0.006673132
T	A	0.2057	0.043	0.1729	3427	1.67E-06	0.006670193
A	G	0.2059	0.044	0.0848	3427	2.43E-06	0.006459706
G	A	0.2111	0.034	0.2288	3427	3.35E-10	0.011447568
A	G	-0.2094	0.038	0.7954	3427	5.03E-08	0.008633677
C	G	-0.3626	0.079	0.0251	3427	4.18E-06	0.006162388
A	C	0.1143	0.024	0.4021	3427	2.88E-06	0.006367669
A	G	0.155	0.032	0.1698	3427	1.84E-06	0.006617654
C	T	-0.2986	0.067	0.034	3427	8.95E-06	0.005740267
G	A	0.1904	0.042	0.0896	3427	7.31E-06	0.005852532
G	A	0.1986	0.039	0.1023	3427	3.93E-07	0.007479537

G	T	0.1371	0.03	0.1959	3427	7.09E-06	0.00586914
G	A	0.1226	0.027	0.2666	3427	5.33E-06	0.006024734
A	G	0.159	0.034	0.15	3427	2.44E-06	0.006465127
T	C	-0.6925	0.154	0.0057	3427	7.53E-06	0.005835647
A	G	0.1903	0.04	0.1029	3427	1.74E-06	0.006653433
T	C	1.402	0.304	0.002	3427	4.06E-06	0.006180164
C	T	-0.2896	0.064	0.0357	3427	6.49E-06	0.005915397
G	C	0.3026	0.067	0.0341	3427	6.18E-06	0.005945128
G	A	-0.1192	0.025	0.6265	3427	1.38E-06	0.006777215
C	G	-0.4079	0.085	0.0201	3427	1.49E-06	0.006734612
T	G	-1.711	0.264	0.0032	3427	1.02E-10	0.012117483
A	G	-0.8849	0.175	0.0044	3427	4.33E-07	0.007422596
A	G	0.2599	0.054	0.0499	3427	1.87E-06	0.006611508
G	A	0.1158	0.025	0.3528	3427	3.79E-06	0.006221749
C	G	0.2134	0.037	0.1996	3427	8.19E-09	0.009649505
T	A	0.2376	0.042	0.1729	3427	1.55E-08	0.009295969
T	C	-0.2505	0.056	0.0451	3427	9.39E-06	0.005713275
T	A	-0.2368	0.034	0.7798	3427	2.95E-12	0.014120168
A	G	-0.1537	0.033	0.2101	3427	3.76E-06	0.006218847
G	A	-0.1158	0.026	0.7151	3427	7.23E-06	0.005862163
C	T	-0.6696	0.14	0.007	3427	1.81E-06	0.006630883
A	G	-0.3674	0.076	0.0231	3427	1.62E-06	0.006690623
A	G	0.1688	0.032	0.1681	3427	1.22E-07	0.008134622
C	T	-0.3023	0.066	0.034	3427	4.33E-06	0.006143552
A	G	-0.1686	0.03	0.8064	3427	1.26E-08	0.009403488
A	G	-0.1726	0.037	0.1103	3427	2.92E-06	0.006360938
G	A	0.1787	0.038	0.1023	3427	3.13E-06	0.006325418
T	A	-0.1206	0.025	0.3513	3427	9.23E-07	0.007003788
A	T	0.2964	0.063	0.0382	3427	3.16E-06	0.006319438
T	C	-0.7539	0.151	0.0057	3427	6.23E-07	0.007221243
G	C	0.2933	0.065	0.0341	3427	7.57E-06	0.005832854
T	G	-0.1114	0.024	0.3594	3427	5.02E-06	0.006055514
C	T	1.029	0.207	0.0037	3395	7.17E-07	0.007219119
G	A	0.183	0.028	0.2966	3395	1.22E-10	0.012132807
G	T	-0.2177	0.048	0.0733	3395	6.51E-06	0.005970382
T	C	0.2448	0.053	0.0627	3395	4.08E-06	0.006230661
G	A	0.1696	0.037	0.138	3395	6.05E-06	0.006011098
G	A	-0.4751	0.107	0.0146	3395	9.88E-06	0.005741563
C	T	0.1576	0.034	0.5704	3395	2.84E-06	0.006438561
T	C	-0.1866	0.031	0.633	3395	2.26E-09	0.010472566
T	C	-0.2777	0.054	0.0613	3395	2.42E-07	0.007827049
G	C	0.236	0.053	0.0642	3395	7.36E-06	0.005903399
C	T	-0.7224	0.151	0.0072	3395	1.76E-06	0.006705256
A	G	-0.1207	0.026	0.463	3395	3.36E-06	0.006341716
T	C	0.1129	0.025	0.5511	3427	7.51E-06	0.005841283
G	A	1.234	0.224	0.0032	3427	3.65E-08	0.008816883
T	C	0.1133	0.025	0.4308	3427	6.31E-06	0.005933972
C	T	0.1478	0.03	0.7505	3427	7.36E-07	0.007131575

A	T	0.2065	0.028	0.2578	3427	4.89E-13	0.01512987
C	T	-0.1246	0.028	0.7336	3427	9.97E-06	0.005680449
T	C	0.2331	0.051	0.0624	3427	5.43E-06	0.006016571
C	T	-0.1797	0.038	0.1153	3427	2.12E-06	0.006541227
G	A	0.307	0.048	0.1087	3427	1.37E-10	0.011947799
G	A	0.2256	0.048	0.098	3427	2.79E-06	0.006388708
C	G	0.2773	0.036	0.1996	3427	2.88E-14	0.016725107
T	G	0.2124	0.039	0.1503	3427	6.06E-08	0.008532918
T	A	-0.2806	0.034	0.7798	3427	7.94E-17	0.020050087
A	G	0.1528	0.034	0.1554	3427	7.53E-06	0.005838486
C	G	-0.2868	0.06	0.0451	3427	2.04E-06	0.006562034
A	G	-0.1252	0.026	0.3742	3427	1.14E-06	0.006888151
C	G	-0.3911	0.085	0.0219	3427	4.11E-06	0.006171435
C	T	-0.6334	0.142	0.0076	3427	7.80E-06	0.005812959
G	A	-0.7003	0.153	0.0066	3427	5.01E-06	0.006060335
G	A	0.113	0.025	0.5518	3427	5.46E-06	0.006012019
T	C	0.1181	0.026	0.3507	3427	8.20E-06	0.005783825
C	T	-0.1281	0.027	0.3108	3427	2.36E-06	0.006477735
A	G	-0.8285	0.18	0.0048	3427	4.44E-06	0.006130427
T	C	0.1111	0.025	0.5511	3427	9.13E-06	0.005734347
A	G	1.299	0.226	0.0029	3427	9.52E-09	0.009556556
C	G	0.1718	0.038	0.1176	3427	7.49E-06	0.005837034
G	A	-0.2037	0.043	0.091	3427	2.62E-06	0.006422369
C	T	0.1445	0.03	0.7505	3427	1.09E-06	0.006910667
T	G	0.1924	0.04	0.1069	3427	1.17E-06	0.00686861
A	T	0.2049	0.028	0.2578	3427	5.23E-13	0.015097719
G	A	-0.124	0.028	0.2699	3427	7.34E-06	0.005851239
G	C	-2.989	0.633	0.0003	3427	2.41E-06	0.006466215
A	G	-0.1813	0.039	0.1094	3427	3.84E-06	0.00620937
T	A	-0.291	0.033	0.7798	3427	3.30E-18	0.021861942
C	G	0.2854	0.036	0.1996	3427	3.28E-15	0.017960249
G	A	0.2415	0.048	0.098	3427	4.41E-07	0.007417981
G	A	0.3169	0.047	0.1087	3427	2.55E-11	0.012907194
T	G	0.2185	0.039	0.1503	3427	2.00E-08	0.009150358
C	G	-2.17	0.49	0.0006	3427	9.61E-06	0.005699545
C	G	-0.2895	0.06	0.0451	3427	1.38E-06	0.006776594
A	G	-0.1274	0.026	0.3742	3427	6.19E-07	0.007225278
G	A	0.1097	0.025	0.5518	3427	8.86E-06	0.005741328
C	T	-0.6438	0.141	0.0076	3427	4.76E-06	0.006089512
A	C	-1.199	0.266	0.0023	3427	7.00E-06	0.005876196
G	A	-0.6778	0.152	0.0066	3427	8.72E-06	0.005753785
T	A	-0.1817	0.041	0.1021	3427	8.03E-06	0.005799125
T	C	0.1189	0.026	0.3507	3427	6.24E-06	0.005942116
C	T	-0.1225	0.027	0.3108	3427	5.54E-06	0.006006088
A	G	0.1209	0.026	0.3296	3427	5.17E-06	0.006046009
A	G	-0.1132	0.025	0.4578	3427	5.27E-06	0.006028362
G	A	-1.226	0.212	0.0032	3395	7.49E-09	0.009782058
A	T	1.152	0.259	0.0019	3395	9.09E-06	0.005780196

C	A	1.095	0.233	0.0024	3395	2.78E-06	0.006446887
A	G	-0.2957	0.058	0.0421	3395	4.23E-07	0.007507724
C	T	-0.1856	0.042	0.0918	3395	8.18E-06	0.005842909
A	T	-0.2907	0.061	0.0378	3395	1.84E-06	0.006677584
T	C	-0.1264	0.028	0.2432	3395	5.39E-06	0.006074106
A	C	-0.227	0.046	0.0857	3395	6.81E-07	0.007243269
T	C	0.1417	0.03	0.1971	3395	3.16E-06	0.00637974
A	C	0.2082	0.046	0.0725	3395	7.46E-06	0.005897988
G	A	0.4974	0.11	0.0127	3395	6.20E-06	0.005997412
G	A	0.361	0.077	0.0259	3395	2.75E-06	0.006455962
A	G	-0.1712	0.038	0.1124	3395	7.00E-06	0.005930665
T	G	-0.5185	0.114	0.0102	3395	5.45E-06	0.006077511
A	G	-0.1806	0.04	0.0973	3395	6.96E-06	0.005933206
T	C	0.2214	0.049	0.0651	3395	5.74E-06	0.006043521
T	C	0.2903	0.064	0.0361	3395	6.74E-06	0.005951481
C	T	-0.1945	0.041	0.09	3395	2.85E-06	0.006434554
T	C	-0.2599	0.05	0.0586	3395	2.47E-07	0.007811763
T	C	-0.1186	0.024	0.4717	3395	5.72E-07	0.007346791
A	G	-0.2046	0.042	0.0851	3395	1.11E-06	0.006967746
T	C	0.2465	0.055	0.0482	3395	6.89E-06	0.005939583
C	T	0.1104	0.025	0.6321	3395	8.01E-06	0.005854719
T	G	0.2259	0.051	0.0601	3395	9.82E-06	0.005741312
G	A	0.3118	0.068	0.0323	3395	5.24E-06	0.006092303
A	G	-0.1168	0.025	0.6521	3395	2.69E-06	0.006465115
C	G	0.2852	0.059	0.0415	3395	1.20E-06	0.006926237
A	G	-0.3646	0.076	0.0274	3427	1.92E-06	0.006596555
A	G	-1.186	0.259	0.0032	3427	4.95E-06	0.006067463
T	C	0.4829	0.094	0.0177	3427	3.18E-07	0.007598723
C	G	-0.16	0.032	0.1936	3427	5.49E-07	0.007296403
T	C	-0.1755	0.039	0.1224	3427	8.97E-06	0.00573598
G	A	-0.1373	0.03	0.2305	3427	4.13E-06	0.006172656
A	G	0.3128	0.067	0.0427	3427	2.80E-06	0.006386115
C	T	0.2568	0.058	0.0522	3427	9.71E-06	0.005693634
G	A	0.4004	0.083	0.0239	3427	1.42E-06	0.006761121
G	A	-0.1351	0.029	0.2454	3427	4.67E-06	0.006099218
T	C	0.248	0.056	0.0527	3427	8.44E-06	0.005769928
A	T	-0.1854	0.041	0.0992	3427	7.03E-06	0.005877083
G	A	0.1422	0.032	0.1911	3427	9.92E-06	0.005683141
G	A	-0.113	0.025	0.5455	3427	4.62E-06	0.006104564
T	C	-0.1193	0.025	0.5225	3427	2.03E-06	0.006569648
A	G	-0.1233	0.025	0.4613	3427	6.84E-07	0.007172708
T	G	0.5813	0.126	0.0099	3427	4.27E-06	0.006153006
C	T	-0.3202	0.072	0.9718	3427	9.90E-06	0.005684545
A	G	-0.1456	0.032	0.8039	3427	4.07E-06	0.006176171
G	T	0.3273	0.071	0.0321	3427	4.27E-06	0.006150718
A	G	0.5404	0.117	0.0109	3427	3.96E-06	0.00619709
G	T	0.5202	0.114	0.0117	3427	5.20E-06	0.006039293
G	A	0.2645	0.059	0.0455	3427	8.67E-06	0.005756383

G	A	-0.3411	0.076	0.0279	3427	6.79E-06	0.005892781
G	A	-0.1208	0.026	0.4316	3427	2.64E-06	0.0064255
T	C	-0.1224	0.025	0.4721	3427	8.98E-07	0.007018404
T	G	-0.1237	0.027	0.3115	3427	4.76E-06	0.006092081
G	A	0.1176	0.026	0.6526	3427	7.01E-06	0.005875738
C	T	0.1638	0.037	0.1344	3427	8.97E-06	0.005738664
C	G	-0.2005	0.041	0.8971	3427	7.93E-07	0.007086934
C	T	0.1519	0.032	0.1722	3427	2.79E-06	0.00638461
G	A	0.3458	0.071	0.0308	3427	1.28E-06	0.006824491
T	C	-0.2959	0.067	0.0363	3427	9.04E-06	0.005733923
A	G	-1.307	0.254	0.0032	3427	2.84E-07	0.007655061
C	T	-0.1296	0.027	0.3097	3427	1.28E-06	0.006817919
T	C	0.3907	0.087	0.0203	3427	7.86E-06	0.005811822
G	A	0.1865	0.041	0.9044	3427	5.73E-06	0.005987001
C	T	0.1577	0.034	0.1512	3427	4.85E-06	0.006077478
G	A	-0.191	0.043	0.0903	3427	8.94E-06	0.005737561
T	G	0.5711	0.124	0.0099	3427	4.28E-06	0.006151585
A	G	-0.117	0.024	0.4613	3427	1.69E-06	0.006664597
G	A	-0.109	0.025	0.5133	3427	8.88E-06	0.00574256
G	A	0.3586	0.08	0.0258	3427	6.97E-06	0.005879951
A	C	-0.2129	0.045	0.0778	3427	2.39E-06	0.006474802
G	T	0.2977	0.066	0.0372	3427	7.20E-06	0.005861127
G	A	-0.1558	0.035	0.1369	3427	9.64E-06	0.005700166
T	C	0.3679	0.083	0.0241	3427	9.78E-06	0.005689505
A	G	-0.1405	0.031	0.1853	3427	7.12E-06	0.00586387
T	G	0.1247	0.028	0.2721	3427	8.49E-06	0.005766632
A	G	0.4428	0.1	0.0156	3427	9.41E-06	0.005712668
A	G	-0.1586	0.035	0.1412	3427	6.67E-06	0.005898995
T	C	-0.1242	0.025	0.4116	3427	9.31E-07	0.006999517
T	C	-0.1238	0.026	0.3637	3427	1.69E-06	0.006668763
A	G	0.2929	0.065	0.0366	3427	6.05E-06	0.00595563
C	G	0.292	0.06	0.0417	3427	1.38E-06	0.006780388
G	C	-0.1096	0.023	0.8445	3395	2.63E-06	0.006486184
T	G	-2.234	0.465	0.0003	3395	1.62E-06	0.006752709
G	A	-0.1736	0.039	0.0508	3395	8.91E-06	0.005793475
T	G	0.1859	0.038	0.0533	3395	1.28E-06	0.006887997
C	T	-0.1229	0.027	0.115	3395	4.40E-06	0.006197497
T	C	-0.08848	0.018	0.3289	3395	1.69E-06	0.006728614
G	A	0.1004	0.018	0.6455	3395	1.86E-08	0.009284036
C	T	0.7465	0.155	0.0034	3395	1.62E-06	0.006751121
C	A	0.4005	0.09	0.009	3395	9.81E-06	0.00574179
A	G	0.435	0.094	0.0074	3395	3.61E-06	0.006302935
C	T	-0.08275	0.019	0.2934	3395	9.75E-06	0.005746976
T	C	0.1395	0.028	0.1417	3395	8.74E-07	0.007096256
T	C	0.2077	0.033	0.7906	3395	4.33E-10	0.011417089
A	G	0.1479	0.033	0.9247	3395	9.57E-06	0.00575622
T	C	-0.1216	0.027	0.1144	3395	7.01E-06	0.005934623
T	C	-0.08657	0.019	0.7244	3395	4.00E-06	0.006246469

C	T	-0.1563	0.03	0.109	3395	1.71E-07	0.008021836
G	A	0.1409	0.03	0.0887	3395	2.90E-06	0.006429879
G	A	0.1387	0.029	0.0931	3395	2.13E-06	0.006597438
C	G	-0.07849	0.017	0.6085	3395	7.25E-06	0.005910559
T	A	0.303	0.066	0.0168	3395	4.78E-06	0.006145697
G	T	0.2391	0.047	0.0314	3395	4.83E-07	0.007432864
C	A	-0.1058	0.022	0.1817	3395	9.57E-07	0.007043109
C	A	0.1089	0.024	0.151	3395	5.90E-06	0.006032919
G	A	-0.116	0.023	0.1705	3395	3.60E-07	0.007606401
A	T	0.1577	0.034	0.0688	3395	2.93E-06	0.006420076
A	G	1.618	0.364	0.0004	3395	9.07E-06	0.005783052
T	C	-0.2553	0.05	0.0286	3395	4.14E-07	0.007521924
G	C	-2.447	0.507	0.0001	3395	1.45E-06	0.006814634
C	G	0.2183	0.048	0.0407	3427	4.62E-06	0.006107542
G	A	-0.1251	0.024	0.8226	3427	2.12E-07	0.007827024
G	C	-0.5394	0.114	0.0067	3427	2.17E-06	0.006524452
G	C	0.5039	0.099	0.0089	3427	3.98E-07	0.007472976
T	C	0.09386	0.019	0.3786	3427	8.57E-07	0.007041162
T	C	3.437	0.711	0.0001	3427	1.38E-06	0.006778265
A	G	-0.1062	0.019	0.328	3427	4.56E-08	0.008695266
C	G	-0.1394	0.031	0.9004	3427	8.23E-06	0.005787661
T	C	1.648	0.37	0.0006	3427	8.55E-06	0.005767985
T	C	1.36	0.306	0.0012	3427	9.07E-06	0.005727203
C	G	-0.0871	0.019	0.3611	3427	7.06E-06	0.005871567
T	C	1.382	0.277	0.0012	3427	6.34E-07	0.007216236
A	G	2.318	0.5	0.0004	3427	3.71E-06	0.006229956
T	C	0.3912	0.086	0.0112	3427	5.15E-06	0.006046316
C	G	0.1093	0.023	0.4561	3427	2.68E-06	0.0064075
C	T	-0.1204	0.024	0.4708	3427	4.30E-07	0.007430897
T	G	-0.1443	0.031	0.8995	3427	2.68E-06	0.006409618
T	C	0.1693	0.036	0.0697	3427	3.52E-06	0.006259162
C	G	1.367	0.308	0.0009	3427	8.98E-06	0.005733706
G	A	-0.2144	0.048	0.04	3427	7.27E-06	0.005853331
G	T	1.964	0.443	0.0004	3427	9.38E-06	0.005712916
A	G	0.3549	0.078	0.0169	3427	5.42E-06	0.006016986
T	C	0.5275	0.115	0.0069	3427	4.24E-06	0.006155147
T	A	0.1571	0.035	0.0741	3427	8.66E-06	0.00575924
A	G	-0.2178	0.048	0.0369	3427	6.72E-06	0.005900882
G	A	2.033	0.42	0.0004	3427	1.35E-06	0.006790518
A	G	-0.5805	0.129	0.0048	3427	7.27E-06	0.005856182
G	A	0.1098	0.024	0.1819	3427	4.64E-06	0.006105839
C	T	0.08333	0.018	0.4765	3427	4.75E-06	0.006093216
G	T	-0.08846	0.02	0.3077	3427	8.46E-06	0.005773242
T	C	0.1436	0.032	0.0919	3427	6.76E-06	0.005896678
T	A	2.18	0.481	0.0003	3427	6.11E-06	0.005948338
C	T	-0.09626	0.02	0.3208	3427	1.49E-06	0.006740919
G	A	0.1025	0.022	0.2155	3427	4.35E-06	0.006143514
A	G	0.4915	0.106	0.0074	3427	3.66E-06	0.00623455

G	C	2.976	0.673	0.0001	3427	9.90E-06	0.005681886
A	C	-0.09028	0.019	0.6472	3427	3.58E-06	0.006247525
G	A	1.268	0.285	0.0009	3427	8.93E-06	0.005738923
C	T	0.3428	0.074	0.016	3427	3.63E-06	0.006243009
G	A	0.07817	0.017	0.4758	3427	4.71E-06	0.006096235
T	G	0.1206	0.022	0.1892	3427	2.66E-08	0.008989709
A	G	-0.08993	0.018	0.328	3427	3.67E-07	0.007518446
T	G	-2.257	0.462	0.0003	3427	1.10E-06	0.00690406
T	C	-0.1199	0.023	0.1547	3427	2.97E-07	0.007635225
T	C	0.1374	0.03	0.091	3427	4.35E-06	0.006140514
A	G	-0.1427	0.03	0.1129	3427	1.61E-06	0.006695697
G	A	-1.192	0.244	0.0012	3427	1.14E-06	0.006893375
G	A	0.3952	0.066	0.0191	3427	2.71E-09	0.01027382
A	C	0.1946	0.044	0.1036	3427	9.77E-06	0.005693371
C	G	1.487	0.28	0.0009	3427	1.12E-07	0.00818585
T	C	0.0837	0.018	0.6698	3427	2.83E-06	0.006375039
C	T	0.244	0.052	0.0266	3427	2.80E-06	0.006383774
G	A	0.1558	0.03	0.089	3427	1.98E-07	0.007860518
A	G	0.1151	0.025	0.1312	3427	6.43E-06	0.005928399
G	C	-0.07929	0.018	0.6347	3427	6.64E-06	0.005907531
C	T	-0.1128	0.025	0.8665	3427	4.76E-06	0.006088018
A	G	-0.529	0.118	0.0048	3427	7.13E-06	0.005859923
C	T	0.09414	0.02	0.2256	3427	3.42E-06	0.006273073
A	G	1.124	0.239	0.0012	3427	2.55E-06	0.006439263
T	G	0.1964	0.044	0.0376	3427	6.72E-06	0.005899603
C	A	-0.1005	0.021	0.1819	3427	2.93E-06	0.006358989
T	C	0.1191	0.025	0.1326	3427	1.79E-06	0.006636936
G	A	-0.1202	0.023	0.1707	3427	1.16E-07	0.008158015
A	G	0.4392	0.097	0.0074	3427	5.54E-06	0.006005648
C	G	0.09839	0.021	0.2041	3427	4.39E-06	0.00613611
G	A	0.1064	0.02	0.2155	3427	1.60E-07	0.007983789
C	G	-0.1114	0.025	0.1328	3427	9.40E-06	0.005715046
G	C	-2.438	0.5	0.0001	3427	1.12E-06	0.006892603
A	T	0.6951	0.141	0.0034	3427	8.59E-07	0.007041631
A	G	-0.1203	0.026	0.4127	3395	4.25E-06	0.006209263
A	G	-1.51	0.234	0.0029	3395	1.15E-10	0.012157828
C	T	-2.912	0.656	0.0003	3395	9.34E-06	0.005768863
G	C	-0.7049	0.137	0.009	3395	2.97E-07	0.007703985
A	G	-0.3399	0.076	0.0297	3395	7.13E-06	0.005918897
G	A	0.1375	0.028	0.353	3395	6.19E-07	0.007288877
A	C	-0.3126	0.07	0.0365	3395	7.79E-06	0.005871451
A	G	-0.6238	0.133	0.0103	3395	2.85E-06	0.006437877
A	C	-0.2651	0.059	0.0501	3395	6.15E-06	0.00600425
T	C	0.1277	0.026	0.539	3395	7.85E-07	0.007164402
C	T	-0.2346	0.047	0.0841	3395	4.90E-07	0.007425742
G	A	0.236	0.048	0.0761	3395	1.11E-06	0.006963017
G	A	-0.1435	0.031	0.2296	3395	3.15E-06	0.006377898
T	C	0.1803	0.032	0.3439	3395	1.62E-08	0.009350884

G	T	0.1735	0.034	0.3116	3395	3.59E-07	0.00759841
T	C	0.1235	0.026	0.5351	3395	2.11E-06	0.006606975
A	C	0.373	0.079	0.0277	3395	2.51E-06	0.006507133
A	G	0.1783	0.04	0.1193	3395	8.00E-06	0.005859172
G	C	-0.1259	0.028	0.3985	3395	7.29E-06	0.005911538
C	T	-0.1356	0.027	0.5872	3395	6.17E-07	0.00729925
T	C	0.1811	0.041	0.1134	3395	9.77E-06	0.005747409
C	A	0.1852	0.042	0.1016	3395	9.97E-06	0.005732538
T	C	-0.1185	0.027	0.4044	3395	8.93E-06	0.005798677
A	G	0.3181	0.069	0.0365	3395	3.71E-06	0.006286288
T	C	-0.3401	0.074	0.0212	3427	3.99E-06	0.0061872
C	T	-2.989	0.556	0.0003	3427	8.23E-08	0.008356632
A	C	-0.5996	0.113	0.0093	3427	1.19E-07	0.008148895
A	C	-2.298	0.483	0.0004	3427	2.00E-06	0.006572754
G	C	0.131	0.029	0.8439	3427	8.23E-06	0.00578348
C	T	-0.7915	0.166	0.0042	3427	1.96E-06	0.006582341
A	G	-1.619	0.194	0.0029	3427	1.14E-16	0.019857439
C	G	-0.2549	0.022	0.3707	3427	2.39E-30	0.037531415
T	C	1.154	0.203	0.9971	3427	1.46E-08	0.009323564
A	C	-0.1102	0.024	0.2607	3427	6.28E-06	0.005936203
C	A	-0.16	0.022	0.3669	3427	9.44E-13	0.014747121
C	G	-0.2971	0.065	0.028	3427	5.38E-06	0.006024278
C	T	-0.1193	0.026	0.2117	3427	5.96E-06	0.005959354
A	C	-0.2168	0.043	0.0696	3427	5.09E-07	0.007335933
G	T	-0.1251	0.027	0.1913	3427	3.30E-06	0.006294621
C	T	0.3142	0.067	0.0267	3427	2.75E-06	0.006397182
T	C	0.3999	0.081	0.0198	3427	9.56E-07	0.006984935
C	T	-0.2751	0.061	0.0474	3427	7.10E-06	0.005867249
T	A	-0.2268	0.049	0.0471	3427	4.28E-06	0.006147593
T	C	0.1832	0.04	0.0762	3427	5.91E-06	0.005973342
T	C	0.1329	0.029	0.1628	3427	6.45E-06	0.005923327
G	A	-0.1156	0.025	0.234	3427	4.10E-06	0.006175835
T	G	-0.9345	0.21	0.0025	3427	8.41E-06	0.005772477
T	C	-0.675	0.152	0.005	3427	9.58E-06	0.005699168
C	T	0.1109	0.022	0.6526	3427	8.29E-07	0.007064001
T	A	-0.2927	0.065	0.0353	3427	7.69E-06	0.005825079
A	G	-1.502	0.222	0.0029	3427	1.56E-11	0.013169557
A	C	0.3547	0.08	0.0241	3427	8.65E-06	0.005759232
G	C	-0.6669	0.131	0.0089	3427	3.53E-07	0.007539962
G	A	0.1775	0.026	0.3528	3427	1.19E-11	0.013326252
T	A	0.2483	0.053	0.0661	3427	2.76E-06	0.006394911
G	A	0.1582	0.03	0.7794	3427	1.99E-07	0.007860821
G	A	-0.1301	0.028	0.2604	3427	4.67E-06	0.006103364
T	C	-1.62	0.365	0.0012	3427	9.50E-06	0.005709103
A	G	0.4285	0.093	0.0204	3427	4.01E-06	0.006184307
G	A	0.1437	0.032	0.5659	3427	8.45E-06	0.005770785
T	C	0.2725	0.057	0.0489	3427	1.60E-06	0.006692423
A	G	-0.1409	0.031	0.402	3427	7.73E-06	0.00582278

A	G	-2.133	0.423	0.0007	3427	4.77E-07	0.007375443
C	T	-0.1403	0.031	0.1897	3427	6.81E-06	0.005888425
A	G	-0.1472	0.033	0.1931	3395	7.01E-06	0.005929688
T	C	1.017	0.229	0.0041	3395	8.89E-06	0.005801002
T	C	0.3087	0.068	0.0362	3395	5.73E-06	0.006044347
G	C	0.2816	0.026	0.5024	3395	8.64E-28	0.034544274
G	A	0.1912	0.028	0.3009	3395	1.45E-11	0.01334102
G	A	0.4153	0.091	0.0214	3395	5.62E-06	0.006053689
A	C	0.2215	0.048	0.0756	3395	4.72E-06	0.006151401
T	C	0.2936	0.066	0.0396	3395	8.07E-06	0.005851367
A	G	-0.1397	0.03	0.5399	3395	2.68E-06	0.006470362
G	T	-0.6831	0.151	0.0077	3395	6.52E-06	0.005968307
T	C	-0.3715	0.079	0.0277	3395	2.42E-06	0.006523889
C	T	0.2185	0.049	0.0747	3395	8.79E-06	0.005803981
A	G	0.1928	0.039	0.1311	3395	6.25E-07	0.007287411
G	T	0.118	0.026	0.6189	3395	6.93E-06	0.00593929
T	A	0.383	0.085	0.0239	3395	6.78E-06	0.005950273
T	C	-0.2482	0.053	0.9393	3395	3.21E-06	0.006370376
A	G	-0.1751	0.039	0.1183	3395	8.26E-06	0.005833845
C	T	-0.1756	0.038	0.134	3395	4.44E-06	0.006182467
G	A	0.2286	0.049	0.9264	3395	2.77E-06	0.006450932
G	C	0.3408	0.074	0.0311	3395	4.69E-06	0.00615719
T	C	0.1774	0.037	0.1255	3427	2.13E-06	0.006532816
A	G	-0.1459	0.031	0.1926	3427	3.61E-06	0.006240744
A	G	1.79	0.256	0.0032	3427	3.42E-12	0.01403321
C	T	-0.3874	0.087	0.0206	3427	8.98E-06	0.005738123
A	G	0.8882	0.186	0.0044	3427	1.96E-06	0.006581846
C	T	0.1837	0.033	0.1792	3427	2.87E-08	0.008950462
T	G	-0.3142	0.048	0.0732	3427	4.97E-11	0.012528441
T	G	0.2684	0.059	0.0467	3427	5.07E-06	0.006057483
G	T	0.2376	0.052	0.06	3427	5.65E-06	0.00599554
G	C	0.352	0.024	0.5023	3427	2.39E-46	0.057875898
G	A	0.246	0.027	0.3011	3427	1.21E-19	0.023718723
C	A	-0.8184	0.178	0.0045	3427	4.64E-06	0.006103355
A	G	-0.1328	0.03	0.4804	3427	7.23E-06	0.005854947
T	A	-0.6519	0.145	0.0092	3427	7.39E-06	0.005847465
G	T	-0.1628	0.037	0.8674	3427	9.33E-06	0.005715402
T	C	-0.3363	0.075	0.0279	3427	8.36E-06	0.00577752
A	G	0.1906	0.037	0.1309	3427	2.94E-07	0.007638703
T	G	-0.5202	0.117	0.0114	3427	8.88E-06	0.00574507
T	C	-0.4005	0.088	0.0222	3427	5.17E-06	0.006044517
T	C	-0.2307	0.051	0.9389	3427	6.43E-06	0.005921614
C	T	-0.1994	0.043	0.0878	3427	3.32E-06	0.006287869
T	C	-0.1333	0.029	0.7734	3427	6.19E-06	0.005942744
C	T	-0.2461	0.05	0.0657	3427	1.12E-06	0.006898467
T	C	0.131	0.029	0.2447	3427	6.42E-06	0.00592314
C	T	-0.1568	0.035	0.8564	3427	6.65E-06	0.005902655
T	G	1.574	0.287	0.0032	3427	4.57E-08	0.008682343

C	T	0.1566	0.034	0.1792	3427	3.40E-06	0.006276345
G	A	0.1972	0.028	0.3011	3427	1.03E-12	0.014709252
G	C	0.3014	0.025	0.5023	3427	5.06E-33	0.040969201
G	T	0.2413	0.053	0.06	3427	5.90E-06	0.005969532
T	G	-0.2555	0.049	0.0732	3427	1.56E-07	0.00799378
C	T	0.7352	0.164	0.0055	3427	7.83E-06	0.005815906
A	C	-0.1138	0.025	0.4819	3427	6.51E-06	0.005920186
C	T	-0.1178	0.027	0.6455	3427	9.28E-06	0.005720192
C	T	0.2153	0.049	0.0708	3427	9.50E-06	0.00570572
C	A	-0.9121	0.197	0.0038	3427	3.66E-06	0.006235133
T	A	0.3695	0.083	0.0238	3427	9.05E-06	0.005733325
A	G	0.1814	0.038	0.1309	3427	1.66E-06	0.006678769
G	A	0.2155	0.048	0.074	3427	8.88E-06	0.005744464
A	G	0.2251	0.049	0.068	3427	5.17E-06	0.00604657
T	C	-0.2324	0.052	0.9389	3427	7.99E-06	0.005801308
A	G	-0.2085	0.044	0.0878	3427	1.78E-06	0.006637897
G	A	0.1278	0.027	0.6878	3427	2.46E-06	0.006452356
C	T	-0.1463	0.032	0.1942	3427	5.27E-06	0.006032233
C	T	-0.2433	0.055	0.0406	3395	9.47E-06	0.005760021
A	G	3.806	0.835	0.0001	3395	5.34E-06	0.006080956
C	A	0.1216	0.023	0.3664	3395	9.48E-08	0.008359547
C	G	0.197	0.023	0.371	3395	3.31E-18	0.022043246
C	T	0.9887	0.198	0.0034	3395	6.22E-07	0.007290896
A	G	1.982	0.443	0.0006	3395	8.00E-06	0.005858846
T	C	3.706	0.827	0.0001	3395	7.70E-06	0.005876051
T	C	0.2664	0.055	0.0396	3395	1.64E-06	0.006745533
T	C	-0.1555	0.034	0.1144	3395	6.51E-06	0.005972352
C	T	-0.1586	0.028	0.2996	3395	1.48E-08	0.009408477
C	T	0.1747	0.029	0.7732	3395	1.22E-09	0.010825959
T	C	-0.4537	0.102	0.0109	3395	9.41E-06	0.005760201
A	T	0.1253	0.027	0.2028	3395	4.11E-06	0.006225453
T	G	3.282	0.695	0.0003	3395	2.40E-06	0.006533129
G	A	-0.1222	0.027	0.7779	3395	4.16E-06	0.006219759
A	G	-0.616	0.136	0.0062	3395	5.91E-06	0.00602418
A	G	4.001	0.806	0.0001	3395	7.32E-07	0.007198775
T	A	3.818	0.841	0.0001	3395	5.77E-06	0.006039796
T	C	0.1248	0.027	0.204	3395	4.52E-06	0.006176174
G	A	0.3584	0.081	0.0196	3395	8.84E-06	0.005801786
C	T	0.1782	0.04	0.0813	3395	7.26E-06	0.005908505
A	T	-0.1561	0.034	0.1165	3395	6.13E-06	0.006004377
A	G	0.4151	0.094	0.013	3395	9.53E-06	0.005757326
G	T	-0.6898	0.149	0.0052	3395	3.81E-06	0.006273371
G	A	0.0983	0.022	0.4177	3395	6.44E-06	0.005980584
C	T	0.1285	0.025	0.2498	3395	2.65E-07	0.007771096
G	A	0.1995	0.041	0.9264	3395	1.28E-06	0.006885557
A	G	0.5352	0.106	0.0101	3427	4.28E-07	0.007425594
C	T	0.8233	0.167	0.0042	3427	8.11E-07	0.007075682
A	G	1.755	0.195	0.0029	3427	3.06E-19	0.023182898

A	C	0.5786	0.113	0.0093	3427	3.55E-07	0.007539282
C	T	3.098	0.558	0.0003	3427	3.03E-08	0.008917555
C	A	0.174	0.022	0.3669	3427	9.93E-15	0.017332877
T	C	-1.139	0.204	0.9971	3427	2.52E-08	0.009023237
C	G	0.2701	0.022	0.3707	3427	1.04E-33	0.041838457
A	C	0.1091	0.024	0.2607	3427	8.38E-06	0.005776459
C	G	0.2951	0.065	0.028	3427	6.66E-06	0.005902449
C	A	0.2162	0.048	0.0525	3427	6.21E-06	0.005939064
C	A	-0.1668	0.037	0.0913	3427	6.31E-06	0.005936709
C	T	0.3476	0.074	0.0238	3427	2.43E-06	0.006464799
C	T	-0.3103	0.067	0.0267	3427	3.94E-06	0.006197913
A	G	0.2369	0.051	0.0463	3427	3.82E-06	0.006213105
T	C	-0.404	0.082	0.0198	3427	8.04E-07	0.00708118
G	A	-0.663	0.147	0.0053	3427	6.62E-06	0.00590875
G	A	1.199	0.264	0.0016	3427	5.57E-06	0.006000942
T	C	0.3649	0.08	0.0179	3427	4.67E-06	0.006102319
A	G	3.615	0.793	0.0001	3427	5.32E-06	0.00603043
T	C	-0.1344	0.03	0.1628	3427	5.40E-06	0.006016242
G	A	0.119	0.025	0.234	3427	2.28E-06	0.006500734
T	G	0.9647	0.21	0.0025	3427	4.58E-06	0.006114413
T	C	-0.3175	0.072	0.0223	3427	9.66E-06	0.005697176
T	C	0.6906	0.153	0.005	3427	6.38E-06	0.005925314
C	T	-0.1103	0.023	0.6526	3427	1.04E-06	0.006945248
T	G	0.4162	0.091	0.9854	3427	4.39E-06	0.006133676
A	G	0.5194	0.116	0.0093	3427	7.63E-06	0.005826198
G	A	0.1612	0.036	0.1107	3427	9.06E-06	0.005730926
T	G	1.455	0.253	0.0032	3427	1.00E-08	0.009536304
A	G	-0.4365	0.098	0.0127	3427	7.96E-06	0.005805028
C	T	2.695	0.579	0.0003	3427	3.39E-06	0.00628001
G	C	0.2378	0.022	0.5023	3427	1.64E-26	0.032595298
G	A	0.1592	0.024	0.3011	3427	8.11E-11	0.012249767
C	T	0.4924	0.107	0.0107	3427	4.36E-06	0.006141564
A	G	-0.1749	0.039	0.0889	3427	6.50E-06	0.00591857
A	G	2.01	0.451	0.0006	3427	8.55E-06	0.005767639
C	A	0.2292	0.05	0.0525	3427	3.82E-06	0.00621223
G	T	1.051	0.214	0.0025	3427	9.85E-07	0.006963172
A	G	-0.4375	0.093	0.0136	3427	2.73E-06	0.006398454
A	T	-0.1242	0.027	0.7847	3427	5.49E-06	0.006012035
T	G	0.1499	0.026	0.5099	3427	1.65E-08	0.009257338
C	T	-0.1461	0.027	0.3844	3427	1.04E-07	0.008222099
T	C	0.2816	0.057	0.0403	3427	7.31E-07	0.007131129
A	G	-0.1017	0.022	0.5156	3427	4.66E-06	0.006108398
T	G	3.594	0.699	0.0003	3427	2.87E-07	0.007657265
C	T	2.855	0.586	0.0003	3427	1.18E-06	0.006869365
T	C	-0.2601	0.058	0.0378	3427	6.80E-06	0.005890446
T	C	0.1123	0.023	0.6698	3427	1.74E-06	0.006653208
T	C	-0.2825	0.063	0.0318	3427	6.52E-06	0.005914974
G	A	1.346	0.273	0.0016	3427	8.67E-07	0.007038257

A	G	3.896	0.822	0.0001	3427	2.21E-06	0.006515573
T	C	0.1244	0.028	0.2037	3427	8.09E-06	0.005792489
A	T	0.1585	0.033	0.1358	3427	1.49E-06	0.006735129
A	G	1.706	0.385	0.0007	3427	9.71E-06	0.005693994
T	G	0.1393	0.029	0.1716	3427	2.07E-06	0.006556807
G	T	-0.7578	0.153	0.0051	3427	7.00E-07	0.00715381
A	G	2.28	0.507	0.0004	3427	7.16E-06	0.005861973
T	C	-0.104	0.023	0.5899	3427	5.59E-06	0.006003232
A	C	0.3634	0.081	0.0194	3427	7.03E-06	0.005875051
A	T	0.916	0.187	0.0034	3427	9.60E-07	0.006982492
G	C	-0.1874	0.042	0.0708	3395	6.51E-06	0.005970388
A	G	1.671	0.372	0.0012	3395	7.42E-06	0.00589557
C	T	0.2144	0.048	0.0539	3395	8.83E-06	0.005798964
G	A	1.119	0.224	0.0027	3395	5.77E-07	0.007329435
A	G	0.1558	0.035	0.1297	3427	7.60E-06	0.005828043
T	C	0.5755	0.125	0.008	3427	4.45E-06	0.006127714
G	C	-0.2083	0.045	0.0712	3427	3.76E-06	0.006221687
T	A	-0.157	0.034	0.1299	3427	5.08E-06	0.006055375
T	C	0.1661	0.037	0.1078	3427	9.72E-06	0.005695263
A	G	1.724	0.384	0.0009	3427	7.40E-06	0.005844216
A	G	-0.2629	0.056	0.0465	3427	2.29E-06	0.006495687
A	C	0.1113	0.025	0.3224	3427	6.49E-06	0.00591857
T	C	0.6456	0.133	0.0082	3427	1.29E-06	0.006818447
T	C	-0.8579	0.191	0.0039	3427	7.18E-06	0.005864729
G	A	0.1234	0.027	0.2418	3427	5.54E-06	0.006009515
T	C	0.1141	0.025	0.3055	3427	6.95E-06	0.005881419
C	T	0.1106	0.024	0.644	3427	6.42E-06	0.005920989
T	C	0.5006	0.097	0.0147	3427	2.90E-07	0.00764918
G	A	0.1487	0.033	0.1405	3427	9.13E-06	0.005730074
C	T	0.2665	0.058	0.0432	3427	4.74E-06	0.006091542
A	T	0.1129	0.025	0.7161	3427	5.26E-06	0.006040088
G	C	0.1238	0.027	0.8016	3427	5.98E-06	0.005964906
T	C	0.1288	0.026	0.226	3427	1.08E-06	0.006923712
C	G	-0.2947	0.067	0.0287	3427	9.67E-06	0.005697984
A	G	0.7854	0.171	0.0041	3427	4.69E-06	0.006096732
T	C	0.2836	0.064	0.0298	3427	8.54E-06	0.00576501
A	G	0.1225	0.027	0.1936	3427	7.41E-06	0.005841019
C	T	-0.2766	0.062	0.0324	3427	8.31E-06	0.005781616
G	A	0.1075	0.024	0.2902	3427	9.92E-06	0.00568759
C	G	-0.3243	0.063	0.0312	3427	2.62E-07	0.007706747
A	G	-0.1639	0.036	0.0964	3427	5.67E-06	0.005995448
C	T	1.998	0.387	0.0007	3427	2.62E-07	0.007709831
T	G	0.1091	0.024	0.3062	3427	4.70E-06	0.00609434
C	T	1.323	0.283	0.0015	3427	3.14E-06	0.006319065
T	C	0.1607	0.036	0.1002	3427	8.47E-06	0.00576814
C	A	1.761	0.359	0.0009	3427	9.72E-07	0.006980037
A	G	-0.1999	0.045	0.0533	3395	8.14E-06	0.005845849
T	G	-0.1029	0.022	0.7144	3395	4.51E-06	0.006182858

C	T	-0.3638	0.082	0.0159	3395	9.43E-06	0.005765707
A	G	0.3132	0.066	0.0233	3395	2.40E-06	0.00653223
A	G	-0.4473	0.096	0.0106	3395	3.65E-06	0.006296518
G	A	-0.6641	0.148	0.005	3395	7.19E-06	0.005919537
T	C	-0.7602	0.168	0.0034	3395	5.96E-06	0.006023433
C	T	-0.1841	0.039	0.1029	3395	2.43E-06	0.006527397
C	T	-0.1356	0.026	0.2641	3395	1.62E-07	0.008052293
A	T	0.1925	0.041	0.0636	3395	3.54E-06	0.006309778
A	T	0.5594	0.124	0.0066	3395	6.43E-06	0.005978054
A	G	0.1027	0.021	0.3542	3395	1.14E-06	0.006949338
C	G	-0.3311	0.074	0.0202	3395	7.61E-06	0.005885913
G	T	-0.1944	0.044	0.0567	3395	9.53E-06	0.005755812
C	T	0.2589	0.058	0.0311	3395	7.55E-06	0.005887168
G	A	0.1429	0.028	0.1529	3395	4.44E-07	0.007485694
G	T	0.2769	0.057	0.0314	3395	1.02E-06	0.00701269
T	C	-0.1339	0.028	0.1535	3395	1.34E-06	0.006860274
G	A	0.1406	0.032	0.1165	3395	8.61E-06	0.005811997
C	T	-0.09212	0.021	0.351	3395	9.44E-06	0.005760842
G	A	0.1138	0.023	0.5573	3427	5.41E-07	0.007299375
T	C	0.8619	0.185	0.0035	3427	3.31E-06	0.006293818
T	C	-0.6498	0.143	0.0063	3427	5.42E-06	0.006022583
T	C	0.5738	0.124	0.008	3427	4.12E-06	0.006169903
C	T	-0.1222	0.027	0.7693	3427	5.94E-06	0.005963662
T	C	1.751	0.348	0.0012	3427	5.04E-07	0.007341737
A	G	0.494	0.107	0.0108	3427	4.24E-06	0.006158393
A	G	0.3892	0.081	0.0198	3427	1.69E-06	0.006665657
A	G	0.1325	0.029	0.7569	3427	5.42E-06	0.006013291
A	C	-0.2442	0.05	0.0598	3427	1.07E-06	0.006923332
C	T	-0.148	0.033	0.1348	3427	9.04E-06	0.00573435
C	T	-0.1284	0.027	0.2221	3427	2.73E-06	0.006399536
A	T	0.1064	0.023	0.3748	3427	4.63E-06	0.006110551
T	C	0.1481	0.027	0.2228	3427	7.23E-08	0.008434624
A	T	0.6688	0.14	0.0066	3427	1.99E-06	0.006577761
C	T	-0.1317	0.03	0.8226	3427	9.03E-06	0.005731887
A	G	-0.1024	0.023	0.5327	3427	8.82E-06	0.005745793
G	T	0.4381	0.095	0.0146	3427	3.96E-06	0.006193237
C	T	-0.126	0.027	0.7776	3427	4.73E-06	0.006097298
A	G	0.3673	0.082	0.0198	3427	7.20E-06	0.005860281
G	A	-0.1196	0.026	0.2569	3427	4.79E-06	0.006085323
C	T	-0.1307	0.03	0.1866	3427	9.68E-06	0.005695247
T	C	-0.1076	0.023	0.3783	3427	3.36E-06	0.006280563
T	G	-0.103	0.023	0.7146	3427	6.79E-06	0.005889016
G	A	0.1914	0.041	0.0676	3427	3.47E-06	0.006261212
A	G	0.1096	0.024	0.2426	3427	5.32E-06	0.006033526
A	G	0.2583	0.058	0.0334	3427	8.92E-06	0.005742218
T	C	-0.7687	0.172	0.0034	3427	8.51E-06	0.005767837
C	T	-0.1522	0.026	0.2642	3427	6.10E-09	0.009817854
C	T	-0.2052	0.039	0.1032	3427	1.73E-07	0.00793651

A	T	0.2055	0.042	0.0638	3427	1.24E-06	0.006833455
C	T	0.0944	0.021	0.6389	3427	9.48E-06	0.005709524
C	T	-0.1193	0.025	0.2221	3427	1.55E-06	0.006712568
T	G	1.153	0.26	0.0015	3427	9.22E-06	0.005723246
C	T	0.901	0.201	0.0028	3427	7.46E-06	0.005840689
A	T	0.565	0.127	0.0066	3427	9.00E-06	0.005733168
A	G	-0.2417	0.054	0.0369	3427	7.66E-06	0.005826937
A	G	-0.1557	0.034	0.1029	3427	4.17E-06	0.006164749
T	C	-0.1173	0.026	0.2069	3427	5.37E-06	0.00602339
T	C	0.8254	0.183	0.0032	3427	6.95E-06	0.005882038
G	A	0.1503	0.029	0.1533	3427	1.87E-07	0.007900947
C	T	0.2638	0.059	0.0314	3427	7.82E-06	0.005813404
C	T	-0.11	0.023	0.726	3427	1.92E-06	0.006595969
G	A	0.1436	0.032	0.117	3427	8.28E-06	0.005780626
T	G	0.1694	0.035	0.0959	3427	1.45E-06	0.006750822
G	C	0.2319	0.05	0.0446	3427	4.18E-06	0.00616405
A	G	1.588	0.356	0.0009	3427	8.44E-06	0.005772615
A	G	-1.32	0.248	0.0032	3395	1.02E-07	0.008308721
A	G	0.4732	0.086	0.0193	3395	4.71E-08	0.008749907
G	A	-0.2618	0.057	0.052	3395	3.73E-06	0.006282203
A	G	0.177	0.03	0.1985	3395	5.45E-09	0.009970798
G	A	0.3469	0.077	0.0255	3395	6.41E-06	0.005981451
G	A	-0.1192	0.025	0.5956	3395	1.59E-06	0.006769555
T	C	-0.1307	0.027	0.2758	3395	9.92E-07	0.007024315
T	C	0.1848	0.04	0.0975	3395	3.57E-06	0.006310283
A	G	-0.1139	0.025	0.5449	3395	3.83E-06	0.006264729
T	C	0.2901	0.058	0.0449	3395	5.82E-07	0.007327483
G	A	0.3984	0.071	0.0314	3395	1.85E-08	0.009282127
A	G	0.1414	0.032	0.1873	3395	7.40E-06	0.005896494
C	T	-0.4447	0.098	0.0147	3395	6.42E-06	0.00597756
A	G	-0.2527	0.057	0.0457	3395	9.33E-06	0.005772008
T	C	0.12	0.027	0.2608	3395	6.60E-06	0.005963325
C	T	-0.6792	0.151	0.0078	3395	7.20E-06	0.005916292
T	G	0.1758	0.039	0.1093	3395	5.98E-06	0.006016742
G	A	0.1522	0.026	0.6739	3395	2.84E-09	0.010336073
A	G	0.9711	0.216	0.0028	3395	7.12E-06	0.005923829
G	T	-0.1626	0.035	0.133	3395	4.12E-06	0.006228302
T	G	-0.1306	0.027	0.5436	3395	2.04E-06	0.006623322
G	A	0.1376	0.026	0.3737	3427	1.75E-07	0.007930194
A	C	-0.6664	0.134	0.0093	3427	7.31E-07	0.00713337
A	G	0.5134	0.092	0.0193	3427	2.65E-08	0.008991635
T	C	-0.6924	0.154	0.0067	3427	6.72E-06	0.005902179
A	G	0.4531	0.084	0.9748	3427	6.25E-08	0.008510852
A	G	-2.131	0.263	0.0032	3427	8.22E-16	0.018741514
C	A	0.1204	0.026	0.4672	3427	2.90E-06	0.006368483
C	T	-0.1294	0.026	0.4692	3427	6.57E-07	0.00719243
G	A	-0.6622	0.13	0.0096	3427	3.84E-07	0.007491632
C	T	-0.1206	0.026	0.4704	3427	2.93E-06	0.006364882

T	C	-0.2034	0.026	0.3844	3427	1.09E-14	0.017282787
A	G	0.1485	0.029	0.7509	3427	2.95E-07	0.007640323
G	C	-0.1546	0.035	0.1637	3427	7.75E-06	0.005822092
C	T	-0.1623	0.035	0.3442	3427	3.15E-06	0.006321343
T	A	-0.1217	0.025	0.5179	3427	1.77E-06	0.006643867
T	C	0.1731	0.037	0.3324	3427	2.79E-06	0.006390714
T	C	-0.1337	0.028	0.2988	3427	1.47E-06	0.006742549
T	C	-0.445	0.097	0.0162	3427	4.88E-06	0.006076419
T	A	0.1349	0.03	0.2276	3427	7.60E-06	0.005834623
C	T	0.3815	0.086	0.0241	3427	9.41E-06	0.005712054
A	G	0.2343	0.042	0.1008	3427	3.32E-08	0.008864863
C	T	0.1319	0.029	0.2804	3427	6.05E-06	0.005955209
G	C	0.1263	0.026	0.6269	3427	1.85E-06	0.006614356
C	T	0.1842	0.041	0.1062	3427	7.41E-06	0.00584393
C	T	-0.256	0.055	0.0591	3427	3.77E-06	0.006216763
A	G	0.1705	0.037	0.1297	3427	5.47E-06	0.006015099
A	C	-0.182	0.04	0.1148	3427	4.66E-06	0.006098316
T	G	-1.506	0.335	0.0015	3427	7.32E-06	0.005852207
T	C	-0.7787	0.173	0.0054	3427	6.97E-06	0.005877248
C	T	-0.4117	0.093	0.0194	3427	9.46E-06	0.005710374
C	T	0.1362	0.026	0.4215	3427	2.05E-07	0.00784771
T	C	-0.2684	0.057	0.0505	3427	2.40E-06	0.006471155
A	G	-1.24	0.252	0.0032	3427	9.05E-07	0.007015693
A	G	0.4328	0.088	0.0193	3427	8.35E-07	0.007059639
A	G	0.2019	0.031	0.1923	3427	5.06E-11	0.012519673
G	A	0.1258	0.026	0.3077	3427	1.78E-06	0.006637033
A	G	1.494	0.337	0.0012	3427	9.40E-06	0.005708953
T	C	0.2578	0.056	0.0519	3427	3.58E-06	0.006249907
T	C	0.1964	0.04	0.0976	3427	1.11E-06	0.006896296
G	A	0.2447	0.055	0.0543	3427	7.79E-06	0.005816211
T	C	0.4707	0.071	0.0312	3427	4.24E-11	0.012613447
A	G	0.1501	0.032	0.2151	3427	3.52E-06	0.006262015
T	C	0.3476	0.058	0.0449	3427	2.83E-09	0.010249191
T	A	-0.1278	0.027	0.2946	3427	1.55E-06	0.006710691
A	G	0.6931	0.151	0.0064	3427	4.57E-06	0.006110292
A	G	0.2288	0.051	0.0601	3427	8.41E-06	0.005775457
A	G	0.1973	0.04	0.1008	3427	1.09E-06	0.006907992
A	C	-0.1752	0.038	0.1148	3427	3.79E-06	0.006219734
C	A	-0.1278	0.028	0.2858	3427	3.66E-06	0.00623552
C	T	0.1213	0.027	0.272	3427	8.31E-06	0.00578242
C	T	-0.8611	0.183	0.0047	3427	2.78E-06	0.006391601
G	A	0.1159	0.025	0.4234	3427	3.09E-06	0.00632765
T	G	0.1737	0.039	0.1099	3427	9.22E-06	0.005722915
G	A	0.1315	0.026	0.6738	3427	3.91E-07	0.007483115
T	C	-0.2519	0.055	0.049	3427	5.68E-06	0.005992374
G	T	-0.1625	0.036	0.1326	3427	5.43E-06	0.0060162
C	T	0.2323	0.051	0.0632	3395	6.30E-06	0.005987306
A	G	0.9668	0.21	0.0034	3395	4.49E-06	0.006180867

T	C	0.1224	0.025	0.458	3395	1.54E-06	0.006777614
A	C	0.1405	0.031	0.7922	3395	7.92E-06	0.00585902
C	T	0.115	0.026	0.4694	3395	6.72E-06	0.005950357
T	C	0.1948	0.026	0.3848	3395	8.39E-14	0.016265571
G	T	0.1651	0.032	0.1895	3395	2.94E-07	0.007712588
G	A	0.1218	0.026	0.5203	3395	1.87E-06	0.006675222
G	T	0.1291	0.026	0.3766	3395	9.59E-07	0.007047413
A	T	-0.2948	0.067	0.0368	3395	9.63E-06	0.005750104
T	C	0.3812	0.081	0.0249	3395	2.28E-06	0.006558444
T	C	0.3442	0.076	0.0286	3395	6.58E-06	0.005967832
T	A	-0.1651	0.031	0.3775	3395	1.05E-07	0.008296092
G	T	-0.2244	0.049	0.1133	3395	5.70E-06	0.006048461
G	A	0.1133	0.025	0.5859	3395	6.12E-06	0.006008627
G	A	-0.2671	0.038	0.1973	3395	1.30E-12	0.014715517
C	T	-1.143	0.257	0.0024	3395	8.77E-06	0.005810434
A	G	0.2618	0.058	0.0513	3395	5.91E-06	0.006025217
C	G	0.3859	0.081	0.0424	3395	1.92E-06	0.006655874
C	T	0.37	0.082	0.024	3395	7.51E-06	0.005891082
A	G	0.1223	0.026	0.3866	3395	2.19E-06	0.006575196
G	T	-0.2674	0.057	0.0505	3395	3.11E-06	0.00638706
A	G	0.1742	0.038	0.1237	3395	5.91E-06	0.006028293
C	T	0.1301	0.026	0.4692	3427	6.19E-07	0.007220124
G	A	-0.1369	0.026	0.3737	3427	2.10E-07	0.0078326
A	G	-0.4531	0.084	0.9748	3427	6.35E-08	0.00850076
A	G	-0.5021	0.092	0.0193	3427	5.15E-08	0.008620244
G	A	0.6423	0.13	0.0096	3427	8.59E-07	0.007040515
A	G	2.048	0.263	0.0032	3427	9.53E-15	0.017360736
G	A	0.1603	0.028	0.2969	3427	1.26E-08	0.009406666
G	C	0.22	0.025	0.5023	3427	7.90E-18	0.02135609
A	G	-0.1564	0.029	0.7509	3427	7.03E-08	0.008444603
C	T	0.1318	0.028	0.28	3427	3.64E-06	0.006241016
T	C	-0.3101	0.068	0.0363	3427	5.17E-06	0.006044123
T	G	-0.173	0.037	0.1929	3427	2.36E-06	0.006480814
A	G	0.1318	0.029	0.2498	3427	6.44E-06	0.00591791
T	C	0.1322	0.028	0.2988	3427	1.97E-06	0.006574236
T	G	-0.3196	0.071	0.0337	3427	6.94E-06	0.00587955
C	T	-0.1225	0.026	0.6129	3427	3.84E-06	0.006206095
A	G	-0.2314	0.042	0.1008	3427	5.15E-08	0.008620367
C	T	-0.1304	0.029	0.2804	3427	8.48E-06	0.005766044
G	A	-0.123	0.028	0.3157	3427	8.41E-06	0.005774409
C	T	-0.1915	0.041	0.1062	3427	3.42E-06	0.006270741
G	T	0.275	0.055	0.0587	3427	7.56E-07	0.007118293
A	C	0.1819	0.04	0.1148	3427	4.84E-06	0.006082514
A	T	-0.2097	0.047	0.0829	3427	8.52E-06	0.005767938
G	A	0.6818	0.147	0.007	3427	3.59E-06	0.006246475
G	A	0.9363	0.208	0.0038	3427	6.86E-06	0.005889245
C	T	0.4143	0.093	0.0194	3427	8.45E-06	0.005771174
C	G	-1.071	0.236	0.0031	3427	5.64E-06	0.005998876

C	T	-0.1294	0.026	0.4215	3427	8.60E-07	0.007046214
T	C	-0.2886	0.063	0.042	3427	4.13E-06	0.006169621
C	T	0.2574	0.052	0.0629	3427	7.19E-07	0.007142658
A	G	-0.4005	0.084	0.9748	3427	1.74E-06	0.006650839
A	G	1.561	0.264	0.0032	3427	3.70E-09	0.010098936
C	G	0.2029	0.04	0.1142	3427	4.73E-07	0.007375085
A	G	0.1148	0.026	0.4943	3427	7.13E-06	0.005861044
G	T	0.1903	0.032	0.1886	3427	4.75E-09	0.009959973
A	G	-0.7933	0.173	0.0053	3427	4.80E-06	0.006084355
T	C	0.202	0.026	0.3844	3427	1.78E-14	0.016998675
C	T	0.1217	0.026	0.4704	3427	2.36E-06	0.006475765
G	A	0.1294	0.026	0.5201	3427	5.11E-07	0.007326245
G	T	0.127	0.027	0.3764	3427	1.84E-06	0.00661759
T	C	0.3462	0.077	0.0285	3427	7.26E-06	0.005855065
G	A	-0.493	0.11	0.0128	3427	7.91E-06	0.005806142
T	C	-0.2404	0.052	0.0681	3427	3.56E-06	0.006248
T	C	0.15	0.031	0.4581	3427	1.58E-06	0.006708001
A	G	-0.2166	0.037	0.2107	3427	3.74E-09	0.010089066
T	C	0.3118	0.051	0.6185	3427	1.43E-09	0.010635922
A	C	0.4277	0.094	0.0182	3427	5.02E-06	0.006060992
C	T	-1.329	0.3	0.0019	3427	9.63E-06	0.005697723
A	T	-1.179	0.258	0.0023	3427	5.09E-06	0.006052031
A	G	0.2461	0.055	0.0584	3427	8.73E-06	0.00575415
T	C	0.558	0.123	0.0114	3427	5.55E-06	0.006008362
T	A	-0.6539	0.138	0.0083	3427	2.33E-06	0.006490297
T	C	-0.1219	0.027	0.6315	3427	4.83E-06	0.00608174
T	G	-0.1748	0.038	0.1263	3427	4.95E-06	0.006069747
G	A	0.2158	0.047	0.0795	3427	5.44E-06	0.006014443
A	G	1.013	0.22	0.0029	3427	4.15E-06	0.006170947
T	G	-0.1307	0.029	0.7693	3427	7.14E-06	0.005859976
C	G	0.3918	0.087	0.0201	3427	7.46E-06	0.005841741
C	G	0.4463	0.097	0.0168	3427	3.93E-06	0.006198899
G	T	0.898	0.202	0.0035	3427	8.71E-06	0.005756391
G	T	-0.3134	0.064	0.0376	3427	1.02E-06	0.006948574
C	A	-0.5635	0.112	0.0117	3427	5.45E-07	0.007293485
G	A	0.1236	0.027	0.2969	3427	3.87E-06	0.00620507
T	C	-0.1348	0.03	0.788	3427	9.43E-06	0.005712185
G	C	0.1666	0.034	0.152	3427	7.49E-07	0.007122835
G	A	-0.1496	0.03	0.7794	3427	6.07E-07	0.007237375
T	G	0.1592	0.036	0.132	3427	9.30E-06	0.005718221
C	G	-0.1242	0.028	0.7457	3427	7.92E-06	0.005811282
C	G	0.6573	0.139	0.0071	3427	2.20E-06	0.006519974
T	C	0.124	0.028	0.2565	3427	7.20E-06	0.005859675
A	C	-0.1898	0.038	0.1087	3427	8.34E-07	0.007060063
T	C	1.203	0.269	0.0019	3427	8.12E-06	0.005793535
G	A	-0.2421	0.053	0.0528	3427	5.95E-06	0.005966496
A	G	0.1917	0.038	0.7954	3427	5.91E-07	0.007253443
G	A	-0.1174	0.025	0.3713	3427	2.39E-06	0.006481042

T	C	0.178	0.04	0.233	3427	9.91E-06	0.005682857
G	A	-0.1816	0.034	0.2288	3427	6.39E-08	0.008496945
C	T	0.3012	0.066	0.0368	3427	4.61E-06	0.006108436
T	C	-0.1265	0.028	0.2533	3427	6.42E-06	0.005924891
C	G	-0.647	0.143	0.0067	3427	6.27E-06	0.005937941
T	C	0.1127	0.025	0.3826	3427	6.98E-06	0.005876321
T	C	-0.3103	0.067	0.0328	3427	3.89E-06	0.006201585
G	A	0.1169	0.026	0.6697	3427	6.73E-06	0.00590031
T	C	-0.1391	0.025	0.4246	3427	2.20E-08	0.009096372
G	A	0.116	0.026	0.3446	3427	7.09E-06	0.005864192
T	C	-1.396	0.306	0.002	3427	5.29E-06	0.006032568
A	G	-0.1879	0.04	0.1029	3427	2.94E-06	0.006359848
C	T	0.3248	0.065	0.0357	3427	5.31E-07	0.007311289
C	T	0.1737	0.037	0.1157	3427	3.73E-06	0.006228349
G	A	0.1118	0.025	0.6265	3427	7.36E-06	0.005848217
T	C	0.163	0.036	0.1228	3427	8.10E-06	0.00579516
G	A	0.1168	0.025	0.6035	3657	3.26E-06	0.005905089
T	C	-0.1864	0.04	0.1098	3657	2.48E-06	0.006052518
T	C	0.1162	0.025	0.4013	3657	2.95E-06	0.005957836
T	C	-0.1346	0.03	0.21	3657	7.45E-06	0.005478058
A	T	-0.3365	0.076	0.0257	3657	9.50E-06	0.005347459
A	G	-0.4112	0.089	0.0194	3657	3.95E-06	0.005805874
C	T	-0.1198	0.027	0.6903	3657	7.89E-06	0.005446532
A	G	-0.148	0.031	0.3024	3657	1.93E-06	0.006178223
C	T	0.3292	0.066	0.0365	3657	6.33E-07	0.006763236
T	C	0.1892	0.041	0.1922	3657	4.93E-06	0.005689554
T	C	-0.939	0.201	0.0037	3657	2.94E-06	0.005961843
G	C	0.3019	0.066	0.0371	3657	5.12E-06	0.005671893
T	G	-0.1967	0.041	0.1036	3657	1.77E-06	0.006221262
A	C	-0.4483	0.098	0.0161	3657	5.24E-06	0.005658563
T	C	0.2444	0.053	0.0593	3657	3.77E-06	0.005831273
T	G	-0.1383	0.03	0.2214	3657	3.73E-06	0.005839535
C	T	-0.1508	0.031	0.8141	3657	1.74E-06	0.006231857
C	T	-0.1215	0.027	0.7254	3656	5.84E-06	0.005607021
T	C	0.6676	0.143	0.0068	3656	2.98E-06	0.00595095
G	A	0.1161	0.025	0.6035	3656	2.25E-06	0.006099783
C	T	-0.3106	0.063	0.0368	3656	1.00E-06	0.006521935
G	A	1.479	0.323	0.0014	3656	4.80E-06	0.005705701
T	C	0.2817	0.064	0.0386	3656	9.83E-06	0.005332387
C	G	-0.3238	0.073	0.0286	3656	8.40E-06	0.005412983
T	G	-0.1258	0.027	0.2637	3656	4.26E-06	0.005766101
G	T	0.5918	0.132	0.0098	3656	7.39E-06	0.005484342
A	G	-0.1101	0.025	0.5109	3656	7.28E-06	0.005484526
A	T	-0.1686	0.036	0.1239	3656	3.07E-06	0.005934039
A	G	-0.4544	0.087	0.0194	3656	1.69E-07	0.00745559
C	T	-0.1082	0.024	0.5315	3656	6.57E-06	0.00554701
A	T	-0.2625	0.046	0.0788	3656	9.20E-09	0.008986546
T	A	-0.1682	0.037	0.8786	3656	6.06E-06	0.005587665

A	G	0.3305	0.069	0.0321	3656	1.93E-06	0.006182681
C	T	-0.4629	0.096	0.0157	3656	1.62E-06	0.006271224
T	C	0.1135	0.024	0.4795	3656	3.59E-06	0.005854939
G	A	-0.1674	0.036	0.8777	3656	2.74E-06	0.005994795
T	C	0.2433	0.049	0.0654	3656	7.31E-07	0.006687488
G	T	-0.1334	0.027	0.2657	3656	6.93E-07	0.006716413
C	T	0.4201	0.086	0.0198	3656	1.01E-06	0.006519104
G	C	0.3278	0.065	0.0371	3656	4.14E-07	0.006984975
A	G	0.1214	0.027	0.2619	3656	9.74E-06	0.005336901
T	G	-0.2724	0.059	0.0439	3656	4.30E-06	0.005765547
T	G	-0.1458	0.032	0.8166	3656	6.78E-06	0.005521881
A	C	-0.3934	0.086	0.0187	3656	5.08E-06	0.005676531
C	T	0.2696	0.055	0.049	3656	8.94E-07	0.006583841
C	G	0.1156	0.025	0.3554	3656	3.11E-06	0.005926885
T	A	-0.1444	0.027	0.4267	3656	6.03E-08	0.007996102
A	T	0.1227	0.027	0.2438	3656	8.18E-06	0.0054236
C	T	-0.2101	0.046	0.0744	3656	4.95E-06	0.005688352
A	G	0.1093	0.024	0.4571	3656	5.26E-06	0.005655033
G	T	0.1155	0.026	0.3122	3656	8.84E-06	0.005385218
G	A	0.1231	0.025	0.6035	3656	6.40E-07	0.006758873
G	A	0.1139	0.025	0.602	3656	4.77E-06	0.005704338
C	T	-0.3216	0.064	0.0368	3656	4.95E-07	0.006895585
T	C	-0.1314	0.03	0.2101	3656	8.61E-06	0.005397468
T	G	-0.1224	0.028	0.2637	3656	8.87E-06	0.005385552
G	A	0.7354	0.162	0.0057	3656	5.84E-06	0.005604927
T	C	0.1173	0.026	0.3103	3656	9.19E-06	0.005366807
A	G	-0.4197	0.087	0.0194	3656	1.62E-06	0.006272123
A	G	0.3204	0.07	0.0321	3656	4.58E-06	0.0057286
A	T	-0.2384	0.046	0.0788	3656	2.25E-07	0.007305699
C	T	-0.2114	0.046	0.118	3656	3.74E-06	0.005836609
G	A	-0.1613	0.036	0.8777	3656	7.27E-06	0.005488348
T	C	0.2249	0.049	0.0654	3656	5.46E-06	0.005634935
A	C	-0.1301	0.027	0.2626	3656	1.48E-06	0.006324571
T	C	-0.1966	0.043	0.0946	3656	3.90E-06	0.005810842
G	C	0.33	0.065	0.0371	3656	4.17E-07	0.006981528
T	C	0.3576	0.079	0.0231	3656	6.58E-06	0.005539714
A	C	-0.4763	0.097	0.0161	3656	8.69E-07	0.006597609
T	C	0.2573	0.052	0.0594	3656	7.63E-07	0.006664967
A	T	0.1795	0.038	0.1157	3656	1.78E-06	0.00622138
T	G	-0.2004	0.045	0.0812	3656	7.71E-06	0.005457836
C	T	-0.1544	0.031	0.8141	3656	6.32E-07	0.006761153
A	C	-0.4087	0.087	0.0187	3656	2.52E-06	0.006039954
T	C	-0.1135	0.024	0.4884	3656	1.65E-06	0.006270846
T	A	-0.1251	0.027	0.4267	3656	3.19E-06	0.005920196
A	G	0.1083	0.024	0.5022	3656	7.80E-06	0.00545261
G	A	-0.1089	0.024	0.5427	3656	6.97E-06	0.00550833
C	T	0.3244	0.07	0.0303	3629	4.12E-06	0.005828473
A	G	-0.4624	0.032	0.1714	3629	3.10E-45	0.053423284

A	G	0.1979	0.037	0.1261	3629	1.26E-07	0.007668584
T	C	0.5241	0.113	0.0113	3629	3.82E-06	0.005872064
C	A	0.6791	0.148	0.0065	3629	4.53E-06	0.005776016
T	C	0.1758	0.038	0.1188	3629	4.60E-06	0.00577217
T	A	0.3159	0.07	0.0314	3629	7.50E-06	0.005513101
T	G	-0.137	0.03	0.2122	3629	4.57E-06	0.005771011
C	T	-0.1261	0.027	0.7193	3629	4.13E-06	0.005827842
T	C	0.218	0.049	0.0671	3629	8.16E-06	0.005466731
G	A	1.056	0.237	0.0025	3629	8.50E-06	0.005454676
C	G	-1.202	0.244	0.0025	3629	8.75E-07	0.006642748
C	T	-0.14	0.03	0.2123	3629	2.45E-06	0.006097863
T	G	0.3255	0.07	0.032	3629	2.96E-06	0.006001109
A	C	-2.48	0.557	0.0004	3629	8.62E-06	0.005442718
C	T	-0.1516	0.03	0.2151	3629	4.74E-07	0.006964457
A	G	-0.2658	0.057	0.9525	3629	2.83E-06	0.006025482
A	G	0.2343	0.05	0.0663	3629	2.46E-06	0.006096609
T	C	-0.1332	0.029	0.2233	3629	5.37E-06	0.005685784
A	G	0.2465	0.038	0.1261	3629	5.98E-11	0.011729288
C	T	-1.259	0.274	0.0018	3629	4.40E-06	0.005792614
A	G	-0.5671	0.127	0.0095	3629	8.13E-06	0.005473003
G	A	-0.6399	0.027	0.2479	3629	1.56E-113	0.131823694
C	A	0.3162	0.059	0.0514	3629	7.85E-08	0.007916287
A	G	-0.3061	0.048	0.0704	3629	2.25E-10	0.01102739
G	C	0.1649	0.027	0.3147	3629	7.59E-10	0.010385975
C	T	0.4261	0.096	0.0164	3629	9.65E-06	0.005382618
G	T	0.2999	0.063	0.0415	3629	1.92E-06	0.006231096
G	A	-0.6138	0.136	0.0087	3629	6.34E-06	0.005606161
T	C	0.1089	0.024	0.5433	3629	8.83E-06	0.005427966
T	G	0.2327	0.052	0.0626	3629	7.94E-06	0.005483742
T	C	-0.1335	0.029	0.2451	3629	4.24E-06	0.005809633
C	T	0.321	0.069	0.0324	3629	3.53E-06	0.005908023
C	T	0.3006	0.067	0.0343	3629	7.69E-06	0.00550148
T	A	-0.1205	0.025	0.5477	3629	1.39E-06	0.006401796
C	G	-0.5822	0.121	0.0107	3629	1.64E-06	0.006318289
A	G	-1.045	0.235	0.0025	3629	8.72E-06	0.00542857
A	C	-0.1389	0.027	0.4964	3629	1.87E-07	0.007457672
T	G	-0.373	0.082	0.0241	3629	5.35E-06	0.005690037
T	C	-0.1219	0.026	0.318	3629	2.93E-06	0.006002385
C	T	-0.1223	0.026	0.3387	3629	2.54E-06	0.006083327
A	T	-0.1679	0.038	0.8794	3629	9.14E-06	0.005410092
G	C	0.3274	0.032	0.1737	3629	9.29E-24	0.027449471
G	A	-0.2879	0.061	0.044	3629	2.03E-06	0.006197246
T	C	-0.2609	0.046	0.0743	3629	2.04E-08	0.008633212
T	C	-0.5466	0.114	0.0121	3629	1.54E-06	0.00633919
T	C	-1.372	0.308	0.0019	3629	8.53E-06	0.005448707
C	T	0.1119	0.025	0.5503	3629	5.75E-06	0.005655633
C	T	-0.1144	0.025	0.6044	3629	5.01E-06	0.005723354
G	A	1.197	0.269	0.0023	3629	9.03E-06	0.005414654

C	T	-2.368	0.5	0.0006	3629	2.30E-06	0.006135397
C	T	0.1393	0.031	0.1857	3629	9.71E-06	0.005380309
T	C	-0.1786	0.031	0.1941	3629	6.35E-09	0.009251848
A	G	-0.4023	0.083	0.0218	3629	1.47E-06	0.006369448
A	G	-0.806	0.178	0.0047	3629	5.97E-06	0.005630763
T	C	0.1413	0.032	0.1877	3629	7.69E-06	0.005500202
C	A	0.1659	0.037	0.1232	3629	9.08E-06	0.005415818
A	G	-0.7414	0.164	0.0059	3629	6.67E-06	0.00557298
A	C	0.3084	0.045	0.0798	3629	6.79E-12	0.012889952
T	C	-0.2121	0.045	0.9212	3629	2.81E-06	0.006025712
A	G	0.2183	0.046	0.0789	3629	1.68E-06	0.006300311
A	G	0.1365	0.028	0.7411	3629	1.12E-06	0.006515444
G	A	0.1416	0.03	0.214	3629	3.29E-06	0.00594686
A	T	5.611	1.234	0.0001	3629	5.58E-06	0.005664949
G	A	-0.1574	0.034	0.1547	3629	4.00E-06	0.005840159
T	G	-0.1857	0.04	0.8917	3629	4.41E-06	0.005791176
C	G	-0.1946	0.041	0.0915	3629	2.65E-06	0.006060211
C	G	0.2921	0.064	0.0459	3629	4.83E-06	0.00574472
C	T	0.1094	0.025	0.4241	3629	9.53E-06	0.005389672
T	C	-0.1157	0.026	0.6645	3629	6.99E-06	0.005545272
A	G	0.1517	0.025	0.3781	3629	9.92E-10	0.010237966
G	T	-0.2044	0.045	0.081	3629	6.32E-06	0.005605943
G	T	-0.1471	0.031	0.1768	3629	2.90E-06	0.006011192
C	T	0.3004	0.066	0.0338	3629	5.91E-06	0.005637004
G	A	-0.1442	0.032	0.1637	3629	7.78E-06	0.005492501
C	T	-2.572	0.566	0.0004	3629	5.67E-06	0.005661912
C	T	0.1703	0.033	0.1536	3629	2.75E-07	0.007254573
A	T	-0.1071	0.024	0.4675	3629	9.87E-06	0.005368137
G	A	0.1613	0.032	0.1674	3629	4.45E-07	0.007000383
T	C	-0.1354	0.029	0.2097	3629	4.34E-06	0.005802799
T	C	-0.1822	0.03	0.1941	3629	1.36E-09	0.010068436
G	A	-0.137	0.028	0.7531	3629	9.09E-07	0.006628684
A	T	0.3431	0.076	0.0263	3629	6.83E-06	0.005562782
C	T	0.3132	0.063	0.0366	3629	6.53E-07	0.006796483
A	G	-0.1487	0.024	0.5236	3629	1.16E-09	0.010147014
A	G	-0.6203	0.135	0.0076	3629	4.58E-06	0.00577551
C	T	-0.1276	0.027	0.2827	3629	1.68E-06	0.006296244
G	A	0.7646	0.165	0.0056	3629	3.47E-06	0.00591796
C	T	-0.5694	0.128	0.0087	3629	9.36E-06	0.005398137
A	G	-0.1145	0.025	0.6503	3629	6.70E-06	0.005568414
G	T	-0.1782	0.036	0.1291	3629	7.11E-07	0.006754949
G	C	0.1288	0.027	0.4118	3629	2.55E-06	0.006078547
G	A	0.1284	0.029	0.7778	3629	7.82E-06	0.005488979
G	T	-0.1154	0.024	0.5141	3629	1.82E-06	0.006252682
C	G	-0.1931	0.043	0.0805	3629	7.37E-06	0.005521185
G	A	-0.3633	0.081	0.0222	3629	8.33E-06	0.005456391
T	C	-1.458	0.324	0.0012	3629	6.81E-06	0.005566156
C	T	-1.305	0.268	0.0018	3629	1.19E-06	0.006476952

T	C	-0.1319	0.029	0.2392	3629	5.02E-06	0.005726862
C	G	0.3032	0.064	0.0459	3629	2.09E-06	0.006183016
G	A	0.1482	0.032	0.1736	3629	3.16E-06	0.005964177
A	G	0.127	0.025	0.3781	3629	3.26E-07	0.007168733
C	T	-0.1359	0.03	0.2001	3629	6.02E-06	0.005626632
C	A	-0.1122	0.025	0.4555	3629	5.41E-06	0.005681238
G	T	-0.2002	0.045	0.081	3629	9.80E-06	0.005372058
C	T	-2.819	0.566	0.0004	3629	6.57E-07	0.006793862
C	T	0.1693	0.033	0.1536	3629	3.29E-07	0.007161631
G	A	0.1594	0.032	0.1674	3629	6.17E-07	0.006829046
T	C	-0.1523	0.03	0.1941	3629	4.25E-07	0.007028468
T	C	-0.332	0.072	0.0285	3629	4.65E-06	0.005764232
T	C	0.1356	0.029	0.8002	3629	4.36E-06	0.005800238
C	G	0.2863	0.065	0.0361	3629	9.51E-06	0.00538991
A	T	0.362	0.078	0.0263	3629	3.63E-06	0.005895748
T	G	-0.2237	0.045	0.0778	3629	6.49E-07	0.006799488
T	C	-0.1378	0.024	0.5233	3629	1.77E-08	0.008712274
G	C	-0.1399	0.032	0.1795	3629	9.31E-06	0.005399144
T	C	0.8181	0.18	0.0047	3629	5.57E-06	0.005672518
A	G	-0.6011	0.135	0.0076	3629	9.08E-06	0.005417438
A	C	0.1409	0.03	0.1962	3629	2.10E-06	0.006184313
T	C	0.2687	0.056	0.0477	3629	1.48E-06	0.006364982
C	T	-0.1213	0.027	0.2827	3629	5.36E-06	0.005684815
G	A	1.359	0.295	0.0015	3629	4.09E-06	0.005829714
T	A	1.306	0.287	0.0017	3629	5.48E-06	0.005673676
G	T	-0.1189	0.024	0.5141	3629	8.91E-07	0.006629703
G	T	-0.1604	0.036	0.1291	3629	8.19E-06	0.005467765
G	A	-0.361	0.081	0.0222	3629	9.64E-06	0.005382632
T	C	0.2339	0.045	0.081	3629	1.95E-07	0.007435559
A	G	-0.4468	0.032	0.1714	3629	2.52E-43	0.05113319
T	C	2.173	0.482	0.0006	3629	6.71E-06	0.005574056
C	G	-0.712	0.154	0.0062	3629	3.68E-06	0.005886088
T	C	0.3784	0.083	0.0204	3629	5.69E-06	0.005659494
A	G	0.1176	0.024	0.5355	3629	1.31E-06	0.006428171
G	A	-0.1476	0.033	0.1571	3629	9.42E-06	0.005391027
T	C	-0.1399	0.028	0.2454	3629	6.93E-07	0.006759987
T	C	-0.9516	0.215	0.0034	3629	9.95E-06	0.005364202
A	G	-0.5784	0.126	0.011	3629	4.81E-06	0.005745929
A	C	0.1193	0.026	0.3002	3629	6.30E-06	0.005608299
G	A	0.1627	0.036	0.136	3629	5.77E-06	0.005652948
A	C	0.2763	0.059	0.0441	3629	3.53E-06	0.005910962
A	G	0.2965	0.062	0.0404	3629	1.58E-06	0.006333401
C	G	-1.147	0.242	0.0025	3629	2.25E-06	0.006142086
T	C	0.1113	0.025	0.3847	3629	8.08E-06	0.005475454
T	C	-0.9518	0.213	0.0032	3629	8.07E-06	0.005477316
T	C	0.3805	0.084	0.023	3629	5.38E-06	0.00568811
A	G	-0.1117	0.025	0.623	3629	7.56E-06	0.005510262
T	C	-0.1739	0.03	0.2136	3629	5.41E-09	0.009340003

C	T	0.1099	0.024	0.4486	3629	6.96E-06	0.005554613
C	T	0.1474	0.031	0.8207	3629	2.18E-06	0.006163684
A	G	0.3961	0.087	0.019	3629	5.62E-06	0.00566396
A	G	-0.2863	0.049	0.0704	3629	4.58E-09	0.009426014
A	G	0.2376	0.038	0.1261	3629	4.60E-10	0.010647138
G	C	0.1677	0.027	0.3147	3629	6.14E-10	0.010487861
G	A	-0.7013	0.027	0.2479	3629	1.41E-134	0.154629261
G	C	-0.5907	0.133	0.0094	3629	9.22E-06	0.005406167
A	G	-0.6517	0.128	0.0095	3629	3.99E-07	0.007059572
C	T	0.3741	0.072	0.0331	3629	1.94E-07	0.0074395
C	T	0.1541	0.034	0.8355	3629	5.52E-06	0.00567509
A	G	0.3676	0.079	0.0258	3629	3.37E-06	0.005932472
C	T	0.4909	0.097	0.0164	3629	4.64E-07	0.006978082
G	A	0.5828	0.13	0.0088	3629	8.02E-06	0.005474104
G	C	0.4909	0.11	0.0134	3629	9.03E-06	0.005418776
G	T	0.2898	0.064	0.0415	3629	5.43E-06	0.005683431
G	T	0.2625	0.057	0.0495	3629	4.94E-06	0.005731954
T	C	-0.1412	0.031	0.2006	3629	4.11E-06	0.00582552
A	G	-0.3263	0.071	0.0331	3629	3.83E-06	0.005864999
C	G	0.1179	0.027	0.336	3629	9.27E-06	0.005404532
T	C	0.22	0.049	0.0668	3629	7.85E-06	0.005490605
A	G	0.4933	0.101	0.0163	3629	9.82E-07	0.006582202
T	C	0.1652	0.031	0.1941	3629	1.06E-07	0.007759727
C	T	0.3233	0.07	0.0324	3629	3.91E-06	0.005853607
C	T	0.3399	0.068	0.0343	3629	5.70E-07	0.006871903
C	G	-0.5996	0.123	0.0107	3629	1.06E-06	0.006537305
G	A	-0.1131	0.025	0.3907	3629	5.98E-06	0.005634965
A	C	-0.1416	0.027	0.4964	3629	1.51E-07	0.007572009
A	G	0.3235	0.068	0.0339	3629	1.93E-06	0.006225151
T	C	-3.05	0.655	0.0001	3629	3.29E-06	0.005944817
G	C	-0.166	0.035	0.1556	3629	1.55E-06	0.006339123
G	A	-0.1142	0.025	0.5178	3629	4.71E-06	0.005753646
T	C	-2.132	0.48	0.0006	3629	9.34E-06	0.005397974
T	C	-0.7379	0.159	0.0054	3629	3.54E-06	0.005907275
A	G	0.5518	0.032	0.1714	3629	3.21E-65	0.077015534
A	G	-0.2169	0.037	0.1261	3629	5.60E-09	0.009320716
A	G	-0.1135	0.024	0.5355	3629	3.36E-06	0.005936785
G	A	0.1521	0.033	0.1571	3629	5.37E-06	0.005688806
A	G	0.3065	0.069	0.0335	3629	8.25E-06	0.005464366
A	G	-0.3023	0.066	0.036	3629	5.41E-06	0.005685912
A	G	0.172	0.037	0.121	3629	3.90E-06	0.005856433
A	G	0.6107	0.136	0.0076	3629	6.83E-06	0.005566294
A	G	0.5387	0.115	0.0116	3629	2.89E-06	0.006010259
T	G	0.1458	0.033	0.164	3629	9.00E-06	0.005415296
T	A	-0.1892	0.042	0.0953	3629	5.74E-06	0.005654091
T	C	-2.112	0.466	0.0007	3629	6.10E-06	0.005623506
C	T	0.2405	0.051	0.0592	3629	3.08E-06	0.005980394
T	G	-0.3205	0.069	0.032	3629	3.85E-06	0.005862727

C	T	0.132	0.03	0.2123	3629	8.30E-06	0.005461099
A	G	-1.209	0.265	0.0019	3629	5.09E-06	0.005715686
A	G	0.1575	0.03	0.2136	3629	1.49E-07	0.007577874
C	T	-0.1438	0.031	0.8207	3629	4.10E-06	0.005830658
C	T	0.122	0.026	0.6667	3629	2.81E-06	0.006035193
C	A	-1.287	0.281	0.0018	3629	4.99E-06	0.00573094
A	G	-0.4021	0.087	0.019	3629	4.35E-06	0.005797408
T	C	0.7119	0.159	0.0054	3629	7.76E-06	0.005493692
A	G	0.2168	0.037	0.1261	3629	5.67E-09	0.009307235
G	A	0.1155	0.025	0.5178	3629	3.68E-06	0.00588461
G	C	0.1659	0.035	0.1556	3629	1.59E-06	0.006327891
A	G	-0.5431	0.032	0.1714	3629	4.06E-63	0.074568583
A	C	-0.4106	0.093	0.0167	3629	9.57E-06	0.005384052
T	C	0.3715	0.083	0.0204	3629	8.67E-06	0.005437891
A	G	0.1127	0.024	0.5355	3629	3.97E-06	0.005849107
T	C	-0.1264	0.028	0.2454	3629	7.94E-06	0.005486319
G	A	-0.1513	0.033	0.1571	3629	6.06E-06	0.005626104
A	G	-0.1714	0.037	0.121	3629	4.24E-06	0.00580967
A	G	0.302	0.066	0.036	3629	5.56E-06	0.005671297
A	G	-0.6208	0.136	0.0076	3629	4.83E-06	0.005742433
A	G	-0.5341	0.115	0.0116	3629	3.54E-06	0.005908657
C	T	-0.2421	0.051	0.0592	3629	2.68E-06	0.006057408
A	G	0.2916	0.062	0.0404	3629	2.59E-06	0.006072126
T	C	0.1122	0.025	0.3847	3629	7.43E-06	0.005519693
C	G	-1.095	0.243	0.0025	3629	6.96E-06	0.005550598
C	T	-0.1424	0.032	0.1779	3629	6.82E-06	0.005568123
A	G	-0.1632	0.03	0.2136	3629	5.21E-08	0.008131752
C	T	0.1482	0.031	0.8207	3629	2.07E-06	0.006186742
C	T	-0.1248	0.026	0.6667	3629	1.64E-06	0.006308803
T	C	0.1303	0.029	0.2138	3629	8.30E-06	0.005460791
A	G	0.3913	0.087	0.019	3629	7.91E-06	0.005486857
C	A	-0.1977	0.042	0.0979	3653	3.05E-06	0.005946999
C	G	-0.1843	0.041	0.1043	3653	5.87E-06	0.005603774
T	G	-0.5367	0.112	0.0115	3653	1.71E-06	0.006246777
A	C	-0.1205	0.025	0.422	3653	1.88E-06	0.006200752
A	G	-0.3246	0.071	0.0307	3653	5.41E-06	0.005648015
G	A	0.1089	0.024	0.5182	3653	8.75E-06	0.005396883
T	G	-0.1356	0.03	0.2067	3653	8.27E-06	0.005427708
T	G	-0.4832	0.106	0.0133	3653	5.83E-06	0.005614045
A	C	0.1318	0.027	0.3099	3653	8.19E-07	0.00664112
C	T	0.1408	0.03	0.2247	3653	3.15E-06	0.00593075
G	A	-0.1469	0.032	0.8199	3653	5.67E-06	0.005619982
A	G	-0.1591	0.036	0.1422	3653	9.91E-06	0.00533594
T	C	0.2029	0.045	0.0835	3653	6.88E-06	0.005522299
G	A	0.143	0.028	0.2644	3653	2.66E-07	0.007227241
G	T	-0.3757	0.084	0.0233	3653	7.82E-06	0.005455349
C	G	0.354	0.075	0.0266	3653	2.77E-06	0.005997931
A	G	0.1501	0.034	0.156	3653	7.67E-06	0.005462407

G	A	-0.2716	0.061	0.0426	3653	9.57E-06	0.005350375
G	A	1.056	0.198	0.0037	3653	1.09E-07	0.007695552
G	T	0.134	0.025	0.4286	3653	6.18E-08	0.007986053
T	G	-0.1166	0.025	0.431	3653	3.62E-06	0.005854192
G	A	-0.2201	0.027	0.3195	3653	1.43E-16	0.018520478
T	C	0.1182	0.025	0.4989	3653	2.30E-06	0.006091815
G	A	-0.118	0.027	0.3058	3653	9.37E-06	0.005362195
G	C	-0.1467	0.026	0.467	3653	1.81E-08	0.008639635
C	T	0.1572	0.025	0.597	3653	2.18E-10	0.010975399
T	C	-0.515	0.115	0.012	3653	8.11E-06	0.005431762
T	G	0.1157	0.026	0.3527	3653	5.90E-06	0.005603973
A	G	-0.5434	0.119	0.0107	3653	4.88E-06	0.005704318
A	T	-0.138	0.026	0.3904	3653	7.53E-08	0.007885895
C	T	-0.1735	0.039	0.1113	3652	9.07E-06	0.005379057
T	C	0.1564	0.035	0.1465	3652	7.83E-06	0.005456582
A	G	-0.2075	0.046	0.0772	3652	7.34E-06	0.005490884
G	C	-0.1442	0.031	0.2063	3652	3.48E-06	0.005882392
C	T	-0.1395	0.03	0.7809	3652	3.56E-06	0.005866424
G	A	0.2925	0.061	0.0435	3652	2.04E-06	0.006157854
C	T	0.1905	0.034	0.3456	3652	2.39E-08	0.008493089
G	T	-0.1347	0.03	0.3758	3652	7.10E-06	0.005508229
A	G	0.8545	0.186	0.0041	3652	4.36E-06	0.005758295
A	T	0.4252	0.095	0.983	3652	7.64E-06	0.00546921
T	C	-0.117	0.026	0.3527	3652	7.98E-06	0.005447449
G	C	0.1243	0.026	0.553	3652	1.64E-06	0.006276945
G	C	0.4504	0.1	0.0189	3652	7.37E-06	0.005491269
G	A	0.3049	0.065	0.0357	3652	3.10E-06	0.005939769
T	C	0.1148	0.026	0.4018	3652	7.37E-06	0.005484832
A	G	-0.121	0.026	0.3472	3652	3.26E-06	0.005913638
A	G	-0.1404	0.032	0.1785	3652	9.07E-06	0.005379743
G	C	0.2783	0.06	0.0439	3652	3.81E-06	0.005831406
C	T	0.1612	0.036	0.8612	3652	7.86E-06	0.005451259
A	T	-0.3345	0.074	0.0305	3652	6.40E-06	0.005562349
C	A	0.1209	0.027	0.7223	3652	9.49E-06	0.005357193
C	G	0.1181	0.026	0.3493	3652	5.39E-06	0.005652457
A	G	0.132	0.027	0.2685	3652	1.06E-06	0.006492583
G	A	-0.324	0.069	0.0312	3652	3.12E-06	0.005936151
A	G	0.1877	0.035	0.774	3652	1.01E-07	0.007738896
G	C	1.633	0.359	0.001	3652	5.58E-06	0.005633769
A	G	-0.1631	0.034	0.2249	3652	2.32E-06	0.006089615
C	T	0.2456	0.049	0.073	3652	4.34E-07	0.006967036
A	G	0.1352	0.03	0.2054	3652	7.94E-06	0.005447211
G	C	0.1776	0.037	0.1247	3652	2.14E-06	0.006136752
T	G	1.531	0.342	0.0016	3652	7.76E-06	0.005463816
G	A	0.3565	0.08	0.0245	3652	9.38E-06	0.005362772
C	T	-0.9366	0.19	0.004	3652	8.72E-07	0.006602922
A	G	-0.2776	0.061	0.0412	3652	4.88E-06	0.005703774
C	T	-0.1838	0.027	0.5305	3652	7.66E-12	0.012753043

A	G	-1.654	0.358	0.0012	3652	3.88E-06	0.005820592
C	T	0.1133	0.026	0.3482	3652	9.78E-06	0.005343198
A	G	-0.1136	0.024	0.4295	3652	3.37E-06	0.005890724
A	G	0.1352	0.029	0.2367	3652	3.73E-06	0.005843951
C	T	0.1324	0.028	0.7529	3652	1.81E-06	0.006221411
C	T	0.145	0.032	0.1779	3668	7.58E-06	0.005450709
A	G	0.5431	0.117	0.0109	3668	3.75E-06	0.005810366
A	G	0.8837	0.197	0.004	3668	7.67E-06	0.005444969
G	A	-0.1299	0.027	0.2854	3668	1.31E-06	0.006359524
C	G	-0.1382	0.031	0.1936	3668	9.86E-06	0.005310438
G	A	-0.5469	0.112	0.012	3668	1.16E-06	0.006424332
A	T	0.5956	0.133	0.0083	3668	7.19E-06	0.00547851
G	C	-0.2714	0.06	0.0432	3668	7.07E-06	0.005483374
C	T	-0.1368	0.03	0.7814	3668	4.38E-06	0.00573539
T	C	-0.5192	0.117	0.011	3668	9.29E-06	0.005349112
G	A	0.1665	0.034	0.3092	3668	1.35E-06	0.006346252
T	C	-0.3507	0.072	0.0315	3668	1.11E-06	0.00644966
A	G	-0.1388	0.03	0.4903	3668	3.24E-06	0.005891498
T	C	0.2398	0.053	0.059	3668	5.66E-06	0.005600417
T	C	-0.2316	0.05	0.0667	3668	3.55E-06	0.005843187
C	T	-0.2963	0.065	0.0367	3668	5.34E-06	0.005633183
G	C	0.1237	0.025	0.5532	3668	1.13E-06	0.006439667
C	G	-0.1105	0.025	0.3851	3668	8.05E-06	0.005422361
A	G	0.1324	0.03	0.217	3668	9.88E-06	0.005317272
C	G	0.4469	0.101	0.0147	3668	9.83E-06	0.005309293
A	G	1.04	0.227	0.0026	3668	4.58E-06	0.005709928
T	C	0.1252	0.027	0.2902	3668	3.23E-06	0.005897197
G	C	-0.1197	0.024	0.4388	3668	1.03E-06	0.006481373
C	T	-0.1982	0.045	0.0792	3668	9.74E-06	0.005321923
T	C	-0.2038	0.045	0.0789	3668	7.33E-06	0.005466109
C	T	0.36	0.076	0.0245	3652	2.54E-06	0.006044608
G	A	-0.6212	0.131	0.0084	3652	2.24E-06	0.006110334
T	C	0.6899	0.146	0.0063	3652	2.32E-06	0.006093572
A	G	-0.5533	0.124	0.0101	3652	8.68E-06	0.005404975
C	T	-0.1209	0.024	0.4618	3652	3.96E-07	0.007010479
T	C	0.1887	0.028	0.3428	3652	3.52E-11	0.011944221
G	T	0.2774	0.062	0.0403	3652	7.73E-06	0.005463872
T	C	0.1703	0.035	0.1314	3652	1.46E-06	0.006332717
T	C	0.1842	0.03	0.3161	3652	1.43E-09	0.009979053
A	G	-0.1436	0.028	0.5394	3652	2.36E-07	0.007284338
T	C	3.671	0.806	0.0004	3652	5.43E-06	0.00564678
T	C	-0.1099	0.024	0.4633	3652	5.01E-06	0.005685364
G	A	-0.1417	0.029	0.7751	3652	1.10E-06	0.006481727
T	G	0.2221	0.045	0.0754	3652	7.32E-07	0.006696811
G	A	-0.1135	0.025	0.5531	3652	4.34E-06	0.005762524
C	A	0.1796	0.039	0.102	3652	3.91E-06	0.005820873
C	T	-0.11	0.025	0.3586	3652	9.76E-06	0.005341037
A	G	-0.2742	0.06	0.0413	3652	4.29E-06	0.005772004

G	A	0.1229	0.026	0.2868	3652	3.33E-06	0.005908129
T	C	-0.1209	0.025	0.4018	3652	1.02E-06	0.006522852
G	C	1.76	0.368	0.0011	3652	1.77E-06	0.006234352
G	A	0.2056	0.045	0.9259	3652	5.54E-06	0.005638548
A	C	-0.1266	0.025	0.3924	3652	3.18E-07	0.007130694
G	T	0.9785	0.212	0.0037	3668	4.24E-06	0.005752779
A	G	0.2079	0.046	0.077	3668	5.63E-06	0.005605648
T	C	-0.1603	0.035	0.1465	3668	3.59E-06	0.005837815
G	C	0.1508	0.031	0.2058	3668	8.24E-07	0.006607552
T	G	-0.2001	0.045	0.0841	3668	7.71E-06	0.00544081
C	T	0.135	0.03	0.7814	3668	5.76E-06	0.005593768
T	C	-0.1132	0.025	0.613	3668	6.62E-06	0.005518979
G	A	0.135	0.03	0.7927	3668	5.74E-06	0.005593768
C	T	-0.1934	0.032	0.3458	3668	2.76E-09	0.009591107
G	T	0.1401	0.029	0.3762	3668	1.26E-06	0.006379314
G	A	-0.2919	0.061	0.0433	3668	1.53E-06	0.006283661
A	G	-0.8197	0.185	0.0041	3668	9.44E-06	0.005335232
A	G	0.2698	0.059	0.0447	3668	5.47E-06	0.005617437
T	C	-0.3983	0.09	0.0188	3668	8.91E-06	0.00536682
T	C	0.1232	0.026	0.3525	3668	1.91E-06	0.006163935
A	T	-0.2386	0.053	0.0624	3668	6.65E-06	0.005515668
G	C	-0.4398	0.099	0.0189	3668	9.19E-06	0.005350484
G	C	-0.121	0.025	0.5532	3668	1.89E-06	0.006173
T	G	-0.372	0.084	0.0211	3668	9.50E-06	0.00533356
G	A	0.1876	0.04	0.8993	3668	3.33E-06	0.005876008
A	G	-0.1374	0.031	0.1884	3668	8.02E-06	0.005420738
G	A	-0.2968	0.064	0.0357	3668	4.24E-06	0.0057502
A	G	0.1233	0.026	0.3472	3668	1.61E-06	0.006260294
T	C	-0.2993	0.067	0.0348	3668	7.81E-06	0.005433569
T	A	0.1231	0.028	0.2833	3652	7.90E-06	0.005448984
T	G	0.5894	0.129	0.0096	3652	4.97E-06	0.005692517
G	A	0.6861	0.144	0.0074	3652	2.03E-06	0.006160695
A	G	0.1612	0.036	0.1353	3652	7.96E-06	0.005445246
C	T	-0.3786	0.085	0.0216	3652	9.12E-06	0.005376603
G	A	-0.2338	0.053	0.0589	3652	9.07E-06	0.005378726
A	G	0.1385	0.027	0.3097	3652	3.35E-07	0.007101256
C	A	-0.3127	0.065	0.0377	3652	1.82E-06	0.006217208
A	G	-0.4601	0.099	0.0153	3652	3.45E-06	0.005881888
G	A	-0.2471	0.054	0.0561	3652	4.23E-06	0.005779389
A	G	-0.4567	0.083	0.022	3652	3.35E-08	0.008315362
G	A	0.273	0.028	0.2581	3652	1.03E-22	0.026017736
T	G	-0.1177	0.025	0.4094	3652	3.96E-06	0.00580889
C	T	-0.3783	0.083	0.0219	3652	5.15E-06	0.005672466
C	T	0.1768	0.04	0.8951	3652	9.02E-06	0.005385127
T	C	0.1146	0.026	0.3499	3652	7.82E-06	0.005457352
A	T	-0.127	0.026	0.55	3652	7.58E-07	0.006683532
G	A	-0.1506	0.032	0.8111	3652	2.34E-06	0.006088646
G	T	-0.9906	0.215	0.0033	3652	4.40E-06	0.005757933

C	T	-0.1144	0.025	0.5743	3652	5.16E-06	0.005678487
T	C	-0.1291	0.027	0.2807	3668	2.29E-06	0.006073057
G	A	0.1298	0.028	0.241	3668	4.57E-06	0.0057105
G	T	0.1508	0.033	0.1579	3668	6.53E-06	0.005523507
C	T	-0.1524	0.034	0.1512	3668	9.11E-06	0.005353295
C	G	-0.1981	0.043	0.0886	3668	5.39E-06	0.005627425
T	C	-0.2518	0.051	0.0585	3668	1.02E-06	0.006492623
T	C	0.3406	0.072	0.029	3668	2.45E-06	0.00603722
T	C	0.204	0.045	0.1963	3668	6.51E-06	0.005529967
T	C	0.5142	0.115	0.0112	3652	8.47E-06	0.005416466
G	T	0.2542	0.053	0.0535	3652	1.60E-06	0.006283067
A	G	-0.1862	0.038	0.1088	3652	1.37E-06	0.006367348
G	A	0.8772	0.193	0.0038	3652	5.36E-06	0.005653828
T	C	0.1223	0.027	0.7193	3652	6.44E-06	0.005562169
T	C	0.3254	0.072	0.0301	3652	6.11E-06	0.005588033
G	A	-0.1232	0.025	0.6669	3652	1.24E-06	0.006415852
A	G	0.3124	0.069	0.0301	3652	5.82E-06	0.005612339
A	T	0.1659	0.037	0.116	3652	9.74E-06	0.005341955
G	A	0.4806	0.07	0.0316	3652	8.63E-12	0.012685659
A	G	0.5877	0.112	0.0116	3652	1.46E-07	0.007536449
A	G	0.425	0.064	0.0379	3652	2.87E-11	0.012049696
G	C	-0.2579	0.058	0.0434	3652	8.93E-06	0.005390363
A	G	0.1823	0.041	0.0945	3652	8.79E-06	0.005397397
T	C	0.3911	0.087	0.02	3652	6.54E-06	0.005548703
C	T	-0.1783	0.039	0.1102	3652	5.52E-06	0.005638807
T	C	1.635	0.335	0.0012	3652	1.11E-06	0.006480248
A	T	0.2353	0.051	0.059	3652	4.45E-06	0.005752246
A	T	0.263	0.059	0.046	3652	7.34E-06	0.005490844
T	C	0.5569	0.123	0.0092	3652	6.42E-06	0.005563898
A	G	0.4269	0.092	0.0167	3652	3.83E-06	0.005828489
G	A	2.057	0.459	0.0007	3652	7.72E-06	0.005462183
T	G	0.5136	0.113	0.0114	3652	5.10E-06	0.005674697
A	G	-0.1564	0.033	0.1574	3652	2.38E-06	0.006076308
C	T	0.1339	0.024	0.5122	3652	2.41E-08	0.008486294
A	G	0.3467	0.077	0.0242	3652	7.70E-06	0.005465465
G	A	0.287	0.055	0.0493	3652	2.32E-07	0.00729514
A	G	0.1215	0.027	0.6105	3652	5.60E-06	0.005629852
G	A	0.4238	0.094	0.0194	3668	6.03E-06	0.005567483
G	A	-0.1271	0.026	0.667	3668	9.14E-07	0.006547684
T	C	0.3384	0.073	0.0301	3668	3.88E-06	0.005797492
T	C	0.4888	0.064	0.0378	3668	4.31E-14	0.015425232
G	A	0.5664	0.071	0.0315	3668	2.99E-15	0.016834348
T	C	0.1156	0.025	0.4129	3668	5.22E-06	0.005641778
T	C	0.2484	0.054	0.0581	3668	3.73E-06	0.005816834
C	T	0.2492	0.046	0.0785	3668	5.10E-08	0.008062317
A	G	0.6898	0.155	0.0063	3668	8.25E-06	0.005405132
T	C	-0.2491	0.056	0.0498	3668	8.57E-06	0.005384554
A	C	-0.2385	0.053	0.0553	3668	8.36E-06	0.005400856

A	G	-0.5896	0.125	0.0094	3668	2.25E-06	0.006077145
A	T	0.269	0.059	0.0462	3668	5.95E-06	0.005576871
T	C	-0.2478	0.052	0.06	3668	1.78E-06	0.006200299
A	T	-0.3212	0.07	0.0309	3668	4.19E-06	0.005754732
G	A	0.1214	0.027	0.2781	3668	7.82E-06	0.005429303
A	G	-0.4063	0.089	0.02	3668	5.55E-06	0.005609496
G	A	0.2822	0.057	0.0491	3668	6.43E-07	0.00673633
G	A	-0.3219	0.072	0.0294	3668	9.07E-06	0.005357505
G	T	0.1552	0.033	0.1475	3652	3.57E-06	0.005867112
G	A	-0.1256	0.024	0.5968	3652	1.92E-07	0.007394558
G	T	0.2134	0.03	0.1925	3652	7.04E-13	0.014013891
T	C	0.1152	0.026	0.2908	3652	8.15E-06	0.005438013
C	T	0.2797	0.061	0.0389	3652	5.39E-06	0.005650107
A	G	-0.1167	0.026	0.7133	3652	5.58E-06	0.00563177
A	G	-0.1032	0.023	0.5086	3652	8.62E-06	0.005407502
A	G	0.5059	0.108	0.0116	3652	3.19E-06	0.005928674
A	G	0.3598	0.073	0.0285	3652	8.52E-07	0.006615146
G	A	0.3666	0.068	0.0316	3652	8.76E-08	0.007811192
A	G	0.2997	0.062	0.0379	3652	1.36E-06	0.00636979
A	G	0.1287	0.026	0.7129	3652	7.48E-07	0.006679926
A	G	-0.2658	0.055	0.0455	3652	1.57E-06	0.006295276
C	T	-0.4534	0.096	0.0149	3652	2.18E-06	0.006123933
T	C	0.2314	0.052	0.0511	3652	7.69E-06	0.005463962
A	G	-0.1192	0.026	0.296	3652	5.63E-06	0.005627371
T	C	-0.1391	0.028	0.27	3652	1.02E-06	0.006521381
A	G	0.1278	0.028	0.2155	3652	5.65E-06	0.005632036
G	A	-0.1076	0.023	0.4302	3652	4.34E-06	0.005766247
G	A	0.1433	0.025	0.6737	3652	7.59E-09	0.009095841
C	T	-0.1358	0.029	0.6387	3652	3.61E-06	0.005859641
C	T	0.4507	0.099	0.0144	3668	5.32E-06	0.005634439
G	A	-0.1138	0.025	0.5966	3668	4.66E-06	0.005703188
A	C	0.1265	0.028	0.7655	3668	7.77E-06	0.005433022
T	C	0.246	0.031	0.1915	3668	1.70E-15	0.017138024
G	C	-0.111	0.024	0.4512	3668	5.28E-06	0.005633331
A	G	-0.1197	0.026	0.7135	3668	6.10E-06	0.005556689
T	C	0.1788	0.039	0.1006	3668	5.15E-06	0.005648569
A	G	0.1198	0.027	0.7124	3668	7.53E-06	0.005450518
G	A	0.4569	0.07	0.0315	3668	9.72E-11	0.011352956
A	G	0.3685	0.064	0.0378	3668	8.33E-09	0.009013059
C	G	0.21	0.045	0.0787	3668	3.15E-06	0.005907411
G	T	-0.1188	0.024	0.5448	3668	8.36E-07	0.006597458
C	A	-0.1225	0.025	0.3615	3668	7.53E-07	0.006650404
G	A	0.1207	0.026	0.6738	3668	2.33E-06	0.006061545
T	C	-0.2435	0.055	0.0492	3668	8.84E-06	0.005371506
G	T	-0.4874	0.108	0.0116	3668	6.99E-06	0.005491529
C	T	0.5036	0.112	0.0111	3653	6.57E-06	0.005543438
T	A	-0.8039	0.158	0.007	3653	4.09E-07	0.007001524
T	C	-0.311	0.06	0.043	3653	2.64E-07	0.007229121

T	C	-1.792	0.396	0.0008	3653	6.36E-06	0.00556614
A	G	-0.6406	0.134	0.0085	3653	1.82E-06	0.00621736
C	G	-0.4567	0.099	0.0155	3653	4.30E-06	0.005767524
A	G	0.2865	0.06	0.0411	3653	2.02E-06	0.006159973
A	C	-0.3257	0.071	0.0305	3653	4.97E-06	0.005690905
T	C	-0.3558	0.076	0.0264	3653	3.34E-06	0.005899004
A	T	0.3931	0.08	0.0229	3653	1.08E-06	0.006488635
C	T	-0.2284	0.051	0.0576	3653	9.23E-06	0.00536996
T	C	-0.3748	0.072	0.029	3653	2.15E-07	0.007334997
A	G	0.1303	0.029	0.7446	3653	5.88E-06	0.005607004
G	A	-1.046	0.227	0.0027	3653	4.03E-06	0.005799199
C	G	-0.8695	0.196	0.0038	3653	9.76E-06	0.00534223
A	G	0.1212	0.027	0.2684	3653	8.60E-06	0.005409801
A	C	0.1945	0.034	0.7416	3653	1.65E-08	0.0086904
C	T	0.2319	0.049	0.073	3653	2.07E-06	0.006151295
G	A	0.1317	0.029	0.2275	3653	8.15E-06	0.00543743
A	G	-0.1163	0.026	0.6393	3653	5.96E-06	0.005596259
G	A	0.1866	0.04	0.1068	3653	3.85E-06	0.005826121
C	T	-0.4219	0.094	0.017	3653	6.74E-06	0.005532242
G	A	-0.1543	0.027	0.5363	3653	9.39E-09	0.0089794
G	T	-0.6792	0.131	0.0083	3653	2.34E-07	0.00729392
G	A	-0.1772	0.039	0.8901	3653	6.11E-06	0.005585314
G	C	0.2811	0.063	0.0387	3653	8.66E-06	0.005401612
T	C	-0.3326	0.073	0.0281	3653	4.71E-06	0.005720428
C	T	0.1125	0.025	0.5865	3653	7.13E-06	0.005499695
T	C	-1.537	0.335	0.0011	3653	4.71E-06	0.005722655
A	T	-0.5037	0.102	0.0131	3653	7.70E-07	0.006670306
T	C	0.1591	0.035	0.4347	3653	5.76E-06	0.005615196
G	A	0.1869	0.041	0.0938	3669	6.75E-06	0.005505606
C	T	-0.1167	0.025	0.4869	3669	2.10E-06	0.00611113
A	G	-0.2852	0.063	0.039	3669	7.10E-06	0.005483374
G	T	-0.387	0.086	0.0211	3669	7.33E-06	0.005464879
T	C	0.3946	0.08	0.0232	3669	9.79E-07	0.006511206
G	A	-0.5777	0.121	0.0109	3669	1.94E-06	0.006154189
G	A	0.3672	0.079	0.0249	3669	3.71E-06	0.005820271
T	A	0.2205	0.049	0.0665	3669	8.43E-06	0.005390012
A	G	0.2088	0.047	0.0786	3669	8.88E-06	0.005366305
C	T	2.357	0.531	0.0005	3669	9.26E-06	0.005345421
G	C	0.1698	0.038	0.127	3669	7.93E-06	0.005426763
T	C	-1.181	0.255	0.0022	3669	3.61E-06	0.005834921
G	C	-0.1499	0.032	0.1924	3669	2.41E-06	0.006046188
C	A	-0.1185	0.025	0.588	3669	1.55E-06	0.006274503
C	A	-0.1678	0.036	0.8609	3669	2.56E-06	0.006015502
G	A	0.2002	0.044	0.0829	3669	6.93E-06	0.005496017
C	T	-0.1204	0.024	0.4516	3669	7.68E-07	0.006635699
C	T	-0.1561	0.034	0.1555	3669	4.17E-06	0.005755999
A	G	0.1396	0.03	0.2186	3669	2.59E-06	0.006009646
T	C	0.2168	0.047	0.0754	3669	4.87E-06	0.005676645

A	G	-0.1502	0.026	0.3227	3669	1.12E-08	0.008851224
C	T	-0.3554	0.079	0.0248	3669	8.05E-06	0.005418791
T	A	-0.6963	0.156	0.007	3653	8.78E-06	0.005396592
G	A	-0.1332	0.027	0.3063	3653	8.25E-07	0.006632949
A	G	0.1161	0.025	0.3597	3653	4.12E-06	0.005795212
T	C	-0.274	0.06	0.0452	3653	4.59E-06	0.005733313
T	C	-0.6975	0.155	0.006	3653	7.33E-06	0.005491668
C	G	0.116	0.026	0.3492	3653	6.42E-06	0.005558961
A	G	0.2154	0.034	0.7473	3653	2.14E-10	0.010982432
C	T	0.2563	0.048	0.073	3653	9.62E-08	0.007757238
C	T	-0.1488	0.031	0.297	3653	1.45E-06	0.006332403
C	T	-0.1152	0.025	0.6388	3653	5.12E-06	0.005670312
G	A	-0.1325	0.03	0.7751	3653	7.32E-06	0.005492199
G	A	0.1828	0.04	0.1024	3653	4.80E-06	0.005710212
C	T	-0.1468	0.026	0.5304	3653	2.65E-08	0.008437635
G	T	-0.6286	0.129	0.0083	3653	1.17E-06	0.00644818
T	A	-0.3225	0.072	0.0278	3653	7.32E-06	0.005489444
A	G	-0.2678	0.06	0.0413	3653	9.40E-06	0.005361449
C	T	-0.1231	0.028	0.254	3653	8.31E-06	0.005423953
A	G	-0.4299	0.089	0.0171	3653	1.31E-06	0.006387883
A	G	0.1311	0.029	0.2368	3653	5.74E-06	0.00561329
T	C	0.1568	0.034	0.4347	3653	5.55E-06	0.005632572
C	T	-0.157	0.034	0.1374	3669	5.39E-06	0.005628876
G	C	0.1497	0.032	0.1668	3669	2.58E-06	0.006011316
C	T	0.1298	0.029	0.2254	3669	9.50E-06	0.005331319
C	T	0.2948	0.065	0.4685	3669	6.38E-06	0.005536069
G	A	-0.1345	0.029	0.6419	3669	4.41E-06	0.00573383
G	A	-0.2806	0.063	0.9629	3669	8.36E-06	0.005398235
G	A	-0.1588	0.033	0.291	3669	1.42E-06	0.006317385
C	T	-0.1205	0.025	0.3684	3669	1.63E-06	0.006247458
T	G	0.2854	0.057	0.048	3669	5.24E-07	0.006836553
T	C	0.1697	0.037	0.1218	3669	5.17E-06	0.005642947
G	A	-0.2706	0.059	0.0429	3669	4.97E-06	0.005666177
C	G	0.2409	0.053	0.0534	3669	6.15E-06	0.005553383
A	G	0.3387	0.068	0.0307	3669	5.94E-07	0.006775677
T	G	0.2301	0.043	0.0822	3669	1.25E-07	0.007585757
T	G	0.1918	0.024	0.5685	3669	1.41E-15	0.017236121
C	A	0.1234	0.027	0.2521	3669	5.69E-06	0.005598923
T	C	-0.2345	0.032	0.1639	3669	4.61E-13	0.014171096
G	T	0.3394	0.076	0.0244	3669	7.37E-06	0.005463277
G	A	-0.298	0.066	0.035	3669	5.88E-06	0.005579415
T	C	-0.2644	0.058	0.0437	3669	5.40E-06	0.005624331
T	C	0.4193	0.078	0.0232	3669	8.44E-08	0.007794737
C	A	0.1521	0.033	0.1607	3669	4.27E-06	0.005742875
T	C	-0.4555	0.096	0.0157	3669	2.23E-06	0.006084729
G	A	-0.5583	0.118	0.0109	3669	2.18E-06	0.006095081
G	A	0.2324	0.05	0.9406	3669	3.79E-06	0.005805179
C	T	2.39	0.515	0.0005	3669	3.66E-06	0.005826683

G	C	-0.1111	0.024	0.5162	3669	4.64E-06	0.00570228
G	C	0.1713	0.037	0.127	3669	3.43E-06	0.005855199
T	G	-0.2011	0.044	0.0797	3669	4.93E-06	0.005668845
G	C	-0.1464	0.031	0.1924	3669	2.13E-06	0.006112326
T	G	0.2667	0.056	0.0481	3669	2.18E-06	0.006096225
C	G	-0.1253	0.027	0.7348	3669	3.53E-06	0.005848508
C	T	-0.1094	0.024	0.4516	3669	3.77E-06	0.005808042
G	A	-0.1498	0.033	0.1554	3669	5.58E-06	0.005605155
A	C	-0.1711	0.035	0.1266	3669	1.28E-06	0.006369715
C	T	0.1172	0.024	0.5124	3669	8.77E-07	0.006571361
A	G	-0.1147	0.026	0.3227	3669	7.17E-06	0.005471354
C	T	-0.3712	0.077	0.0248	3669	1.57E-06	0.006266731
C	A	0.5316	0.119	0.0106	3668	8.36E-06	0.005402125
C	T	-0.2375	0.052	0.0589	3668	4.69E-06	0.00569626
C	T	-0.1399	0.029	0.7814	3668	2.09E-06	0.006118782
T	C	-0.3968	0.071	0.0315	3668	2.73E-08	0.008389369
T	G	0.4689	0.105	0.0123	3668	8.97E-06	0.005366769
C	T	0.6955	0.15	0.0067	3668	3.82E-06	0.005803888
G	T	0.1421	0.032	0.1574	3668	9.88E-06	0.005314161
G	A	-0.8014	0.18	0.0046	3668	8.75E-06	0.005375065
C	T	0.8424	0.182	0.0045	3668	3.73E-06	0.005819489
A	C	0.1268	0.028	0.2867	3668	4.18E-06	0.005758639
G	A	0.1408	0.032	0.1831	3668	9.33E-06	0.005342973
G	A	0.123	0.027	0.7411	3668	7.88E-06	0.005428346
C	G	0.1981	0.044	0.0852	3668	8.41E-06	0.005397878
G	A	0.1177	0.025	0.3768	3668	1.74E-06	0.006217341
A	G	-0.3174	0.071	0.0288	3668	8.89E-06	0.005369106
T	C	-0.3892	0.086	0.0198	3668	6.73E-06	0.005511796
A	C	0.3831	0.084	0.0207	3668	5.01E-06	0.005664171
A	C	-0.1555	0.033	0.1602	3668	2.71E-06	0.005984544
G	C	0.1508	0.031	0.2058	3668	7.98E-07	0.006620471
A	G	0.1636	0.037	0.1279	3668	8.36E-06	0.005403018
A	C	0.2966	0.062	0.0418	3668	1.54E-06	0.006278778
A	G	0.1606	0.032	0.3028	3668	4.81E-07	0.006884031
A	G	-0.1368	0.029	0.5394	3668	2.17E-06	0.006096723
T	C	0.1801	0.03	0.3428	3668	1.16E-09	0.010045708
A	G	0.2722	0.059	0.0447	3668	4.39E-06	0.005732624
C	T	0.1831	0.04	0.1031	3668	4.53E-06	0.005719809
T	C	0.1145	0.026	0.3525	3668	9.36E-06	0.005340925
G	C	-0.1219	0.025	0.5532	3668	1.53E-06	0.006279354
G	A	0.1164	0.025	0.3978	3668	3.35E-06	0.0058754
G	C	-0.4383	0.099	0.0189	3668	9.61E-06	0.005327079
G	C	-0.2216	0.05	0.0647	3668	8.18E-06	0.00541023
G	C	-0.1216	0.027	0.6855	3668	5.17E-06	0.005652411
T	C	-0.1371	0.025	0.4017	3668	5.92E-08	0.007979716
A	G	0.1147	0.026	0.3472	3668	7.78E-06	0.005430445
A	G	0.2281	0.05	0.063	3668	5.08E-06	0.005655371
C	T	-0.1599	0.034	0.1512	3668	3.20E-06	0.005900236

C	G	-0.194	0.043	0.0886	3668	8.30E-06	0.005403085
T	C	-0.232	0.051	0.0585	3668	6.64E-06	0.005517101
T	C	0.342	0.072	0.029	3668	2.20E-06	0.006093363
A	G	0.1377	0.027	0.3101	3668	3.17E-07	0.007103689
C	T	0.3038	0.068	0.0346	3668	8.55E-06	0.005388501
C	A	-0.2978	0.065	0.0375	3668	4.76E-06	0.005688297
G	A	-0.2423	0.053	0.056	3668	5.63E-06	0.005604607
A	G	-0.4693	0.098	0.0153	3668	1.96E-06	0.006154359
A	G	-0.4277	0.082	0.0221	3668	1.91E-07	0.007369414
C	T	-0.1259	0.027	0.2916	3668	2.42E-06	0.006047742
G	A	0.2919	0.027	0.2578	3668	4.29E-26	0.029991376
C	T	-0.3724	0.082	0.0219	3668	6.04E-06	0.005568501
C	G	0.566	0.123	0.0135	3668	4.25E-06	0.005749046
G	T	0.2442	0.052	0.06	3668	2.68E-06	0.00598801
C	T	0.2018	0.039	0.8949	3668	3.21E-07	0.007097522
T	G	-0.2528	0.054	0.0578	3668	2.54E-06	0.006019014
G	A	-0.1172	0.025	0.5196	3668	4.20E-06	0.005761893
C	T	-0.2752	0.058	0.0472	3668	2.07E-06	0.006125507
C	A	-0.3758	0.08	0.0248	3668	2.70E-06	0.005984436
G	A	0.1097	0.025	0.4562	3668	7.80E-06	0.005436063
C	T	-0.1148	0.025	0.5748	3668	4.03E-06	0.005770855
A	G	-0.6422	0.145	0.0076	3668	9.19E-06	0.005348667
C	T	0.1545	0.032	0.1954	3652	1.14E-06	0.006462373
T	C	-0.3114	0.059	0.0498	3652	1.29E-07	0.007605891
C	G	-0.364	0.031	0.1955	3652	5.06E-31	0.036064727
T	C	-0.278	0.057	0.0546	3652	1.05E-06	0.006505242
C	T	0.1472	0.031	0.2534	3652	1.59E-06	0.006292407
C	T	0.5828	0.132	0.0085	3652	9.94E-06	0.005333524
T	C	0.1172	0.026	0.6424	3652	6.97E-06	0.005516203
A	G	0.3058	0.067	0.0346	3652	5.95E-06	0.005600141
G	A	0.2152	0.036	0.2493	3652	3.96E-09	0.009438986
T	G	-0.2371	0.053	0.4363	3652	8.14E-06	0.005435844
G	A	0.2258	0.034	0.23	3652	3.19E-11	0.011995557
G	A	0.2145	0.042	0.0995	3652	2.80E-07	0.007199962
C	T	0.1188	0.026	0.3413	3652	7.35E-06	0.005485363
G	T	0.1417	0.032	0.8163	3652	9.52E-06	0.005350492
C	T	0.2452	0.055	0.0545	3652	9.27E-06	0.005370057
A	G	0.8964	0.197	0.0038	3652	5.37E-06	0.005654595
C	T	-0.1147	0.025	0.5027	3652	5.28E-06	0.005658617
A	C	0.1208	0.025	0.4482	3652	1.60E-06	0.006282543
T	G	-0.1302	0.028	0.2983	3652	4.22E-06	0.005778697
T	C	0.1232	0.027	0.4017	3652	7.59E-06	0.005469646
G	A	0.138	0.031	0.1895	3668	9.12E-06	0.005352972
G	A	0.1599	0.03	0.208	3668	1.15E-07	0.007639985
T	C	-0.299	0.057	0.0496	3668	2.05E-07	0.007333102
C	G	-0.3858	0.03	0.1948	3668	1.70E-36	0.042488888
A	G	0.2782	0.062	0.0369	3668	8.24E-06	0.005406973
C	T	0.1444	0.03	0.2531	3668	1.33E-06	0.006352172

T	C	-0.5696	0.128	0.9907	3668	9.25E-06	0.005344781
T	G	0.3761	0.084	0.0213	3668	7.98E-06	0.005421521
A	G	0.2491	0.05	0.0687	3668	6.16E-07	0.006756088
G	A	0.2877	0.033	0.2297	3668	1.41E-18	0.020879421
T	G	-0.2634	0.051	0.437	3668	2.65E-07	0.007194401
G	A	0.252	0.035	0.2492	3668	8.47E-13	0.013857887
G	C	0.1278	0.029	0.2358	3668	9.29E-06	0.00534715
G	A	0.2039	0.041	0.0998	3668	5.58E-07	0.006809309
T	C	0.1116	0.025	0.4009	3668	7.90E-06	0.005424947
C	A	0.8104	0.181	0.0045	3668	7.61E-06	0.005447539
C	T	0.2386	0.054	0.0543	3668	9.89E-06	0.005313981
A	G	0.114	0.025	0.5162	3668	3.46E-06	0.005853769
A	C	-0.1117	0.025	0.5018	3668	5.65E-06	0.005598543
A	C	0.1156	0.025	0.4475	3668	2.52E-06	0.006023122
G	A	-0.119	0.026	0.3212	3668	6.03E-06	0.005567376
T	C	0.1223	0.027	0.4027	3668	4.16E-06	0.00575592
T	C	-0.1247	0.026	0.3138	3668	2.43E-06	0.006041365
C	T	0.165	0.031	0.1951	3668	1.13E-07	0.007644733
G	A	0.1391	0.031	0.1895	3668	8.41E-06	0.00539663
T	C	-0.2017	0.044	0.0879	3668	5.49E-06	0.005619885
T	C	-0.3086	0.058	0.0496	3668	9.12E-08	0.007754144
C	G	-0.3848	0.03	0.1948	3668	4.76E-36	0.041958651
C	T	0.1477	0.03	0.2531	3668	8.41E-07	0.006595449
G	A	0.2355	0.035	0.2492	3668	2.81E-11	0.012008665
G	A	0.2689	0.033	0.2297	3668	2.73E-16	0.01810186
A	G	0.2465	0.05	0.0687	3668	8.90E-07	0.006566916
T	G	-0.2658	0.051	0.437	3668	2.28E-07	0.007271352
G	A	0.2104	0.041	0.0998	3668	2.67E-07	0.007197879
C	T	0.255	0.054	0.0543	3668	2.50E-06	0.006024946
A	G	0.1163	0.025	0.5162	3668	2.41E-06	0.006046722
A	C	-0.116	0.025	0.5018	3668	2.66E-06	0.00599154
A	C	0.1141	0.025	0.4475	3668	3.67E-06	0.005826142
G	A	-0.1187	0.026	0.3212	3668	6.84E-06	0.005497776
T	C	-0.1247	0.027	0.3138	3668	2.63E-06	0.005996146
T	C	0.1211	0.027	0.4027	3668	5.63E-06	0.005602098
A	G	-0.1769	0.04	0.1086	3668	8.27E-06	0.005402881
G	A	0.4434	0.094	0.0194	3668	2.51E-06	0.006023109
G	A	2.041	0.46	0.0007	3668	9.30E-06	0.005345397
A	G	0.1531	0.034	0.1449	3668	9.22E-06	0.005346363
T	C	0.3554	0.074	0.0301	3668	1.44E-06	0.006315105
G	A	-0.1298	0.026	0.667	3668	6.57E-07	0.006723183
T	C	0.471	0.065	0.0378	3668	5.86E-13	0.014048795
G	A	0.5228	0.072	0.0315	3668	6.10E-13	0.014027793
C	T	0.2425	0.046	0.0785	3668	1.50E-07	0.007493806
A	G	0.6971	0.156	0.0063	3668	7.60E-06	0.005449129
T	C	-0.2522	0.056	0.0498	3668	7.49E-06	0.005458291
A	G	-0.5911	0.125	0.0094	3668	2.25E-06	0.006078766
A	T	0.275	0.06	0.0462	3668	4.50E-06	0.005720986

T	C	-0.2405	0.052	0.06	3668	4.01E-06	0.005780179
A	T	-0.3157	0.07	0.0309	3668	6.99E-06	0.005491274
G	A	0.1228	0.027	0.2781	3668	7.62E-06	0.005446212
T	G	0.1342	0.025	0.5684	3668	6.63E-08	0.007919889
G	A	0.2703	0.057	0.0491	3668	2.20E-06	0.006093385
A	G	0.2265	0.05	0.0611	3668	5.11E-06	0.005653035
G	A	-0.115	0.024	0.5966	3668	2.76E-06	0.005975659
C	A	2.323	0.506	0.0007	3668	4.62E-06	0.005706474
T	C	0.2327	0.03	0.1915	3668	2.49E-14	0.015722936
T	C	-0.232	0.051	0.06	3668	4.82E-06	0.005682896
A	C	0.1255	0.028	0.7655	3668	7.15E-06	0.005482152
G	C	-0.1076	0.024	0.4512	3668	7.90E-06	0.005427521
A	G	0.1299	0.026	0.7124	3668	8.73E-07	0.006572114
G	A	1.393	0.311	0.0014	3668	7.65E-06	0.005443284
A	G	0.3543	0.063	0.0378	3668	2.02E-08	0.008543383
C	G	0.2042	0.044	0.0787	3668	4.44E-06	0.005728357
G	A	0.415	0.07	0.0315	3668	2.69E-09	0.009605199
A	G	0.681	0.149	0.0063	3668	4.94E-06	0.005670298
G	T	-0.1191	0.024	0.5448	3668	5.66E-07	0.00679788
A	G	-0.2505	0.056	0.0455	3668	8.48E-06	0.005391071
T	C	0.2397	0.052	0.0511	3668	5.12E-06	0.005653163
C	T	0.1276	0.027	0.2719	3668	1.59E-06	0.006257729
G	A	0.1595	0.036	0.1299	3668	7.57E-06	0.005451928
G	A	0.1408	0.025	0.6738	3668	2.39E-08	0.008459023
T	C	-0.2405	0.054	0.0492	3668	8.81E-06	0.005374662
T	C	0.1647	0.037	0.119	3668	7.92E-06	0.005425349
A	C	-0.1514	0.033	0.1602	3668	4.90E-06	0.005674888
G	C	0.1478	0.03	0.2058	3668	1.31E-06	0.006365483
T	C	0.1093	0.024	0.4866	3668	7.53E-06	0.005454121
T	C	0.4467	0.099	0.0159	3668	6.71E-06	0.005512108
A	C	0.2864	0.062	0.0418	3668	3.46E-06	0.005856838
T	C	-0.1289	0.028	0.7415	3668	6.27E-06	0.005549766
C	T	-0.1904	0.032	0.3458	3668	4.59E-09	0.00932136
G	A	0.1546	0.031	0.5643	3668	6.87E-07	0.00670051
C	A	0.2331	0.052	0.0819	3668	7.15E-06	0.005481986
A	G	0.2907	0.059	0.0447	3668	9.26E-07	0.006541853
C	T	0.1822	0.04	0.1031	3668	4.99E-06	0.005666863
T	C	0.4447	0.09	0.0192	3668	7.19E-07	0.006675304
G	C	-0.1219	0.025	0.5532	3668	1.52E-06	0.006284286
T	C	-0.1548	0.025	0.4017	3668	9.08E-10	0.010182783
T	C	-0.128	0.029	0.2244	3668	9.64E-06	0.005323258
T	C	0.2044	0.045	0.0789	3668	6.59E-06	0.005522336
G	A	-0.1539	0.033	0.8447	3650	4.28E-06	0.005772942
C	T	-0.1373	0.029	0.2236	3650	1.77E-06	0.006239812
C	T	0.154	0.034	0.1526	3650	8.19E-06	0.005438731
A	G	-0.3381	0.075	0.0267	3650	7.63E-06	0.005472815
A	T	-1.257	0.276	0.0019	3650	5.60E-06	0.0056344
C	G	-0.1947	0.035	0.1942	3650	3.16E-08	0.008350037

A	G	0.3066	0.046	0.7905	3650	1.90E-11	0.01227139
C	T	-0.3512	0.071	0.0329	3650	7.23E-07	0.006703773
A	G	-0.2867	0.06	0.0437	3650	2.06E-06	0.006155261
A	C	0.1504	0.031	0.1959	3650	9.75E-07	0.006549449
G	A	-0.8984	0.18	0.0048	3650	6.45E-07	0.006763782
T	C	-0.1129	0.024	0.5141	3650	3.15E-06	0.005937398
A	G	0.2763	0.062	0.0395	3650	9.69E-06	0.00534626
C	T	-0.1323	0.03	0.2168	3650	7.86E-06	0.005461769
A	C	-0.1303	0.028	0.747	3650	2.85E-06	0.005987023
T	C	0.109	0.025	0.5271	3650	8.78E-06	0.005393606
A	G	0.2848	0.059	0.0449	3650	1.20E-06	0.006438427
G	T	-0.2623	0.057	0.0493	3650	4.84E-06	0.00570832
T	C	0.2644	0.056	0.0508	3650	2.89E-06	0.005978684
A	G	0.3857	0.084	0.0215	3650	4.35E-06	0.005764916
A	G	0.4148	0.091	0.0181	3650	5.40E-06	0.005652849
T	A	0.455	0.102	0.0157	3666	8.86E-06	0.005377579
A	G	-0.2029	0.045	0.9158	3666	5.38E-06	0.005628851
A	G	-0.1424	0.03	0.2218	3666	2.14E-06	0.006108352
T	C	0.1178	0.026	0.3648	3666	7.28E-06	0.005471655
T	C	-0.7025	0.157	0.0057	3666	7.66E-06	0.005445486
A	G	-0.2557	0.053	0.0544	3666	1.62E-06	0.006255053
C	T	0.544	0.117	0.0115	3666	3.16E-06	0.005902517
A	G	-0.3645	0.077	0.0267	3666	2.29E-06	0.00607226
A	G	0.363	0.071	0.415	3666	3.24E-07	0.007097614
C	T	-0.3592	0.072	0.0329	3666	7.37E-07	0.006667725
T	G	-0.2303	0.052	0.0637	3666	9.35E-06	0.005342374
C	G	0.1358	0.03	0.2158	3666	6.75E-06	0.005510727
G	T	0.1318	0.026	0.5821	3666	3.12E-07	0.007117575
C	T	-2.124	0.436	0.0008	3666	1.14E-06	0.006437795
T	C	-0.1114	0.025	0.4367	3666	8.52E-06	0.005395646
T	C	-0.1159	0.025	0.5139	3666	3.07E-06	0.005927064
T	C	-0.2419	0.054	0.0541	3666	9.30E-06	0.005347087
G	C	0.1154	0.026	0.5869	3666	6.42E-06	0.005542475
C	T	0.1769	0.038	0.1148	3666	4.30E-06	0.005746713
C	T	-0.1407	0.029	0.2234	3666	1.53E-06	0.006280611
T	A	0.4612	0.102	0.0157	3666	6.11E-06	0.005567572
A	G	-0.5993	0.135	0.0085	3666	8.94E-06	0.005370592
G	A	-0.1144	0.025	0.4594	3666	7.20E-06	0.005481468
A	G	-0.1411	0.03	0.2218	3666	2.41E-06	0.00604598
A	G	-0.2417	0.053	0.0544	3666	5.34E-06	0.005632494
C	T	0.5568	0.116	0.0115	3666	1.68E-06	0.00623484
A	G	-0.3616	0.077	0.0267	3666	2.52E-06	0.006023147
A	T	-1.268	0.283	0.0019	3666	7.45E-06	0.005461642
C	T	-0.3562	0.072	0.0329	3666	8.23E-07	0.006608214
A	G	0.3943	0.071	0.415	3666	2.49E-08	0.008439061
A	G	0.1346	0.029	0.689	3666	4.70E-06	0.005696508
C	G	0.1369	0.03	0.2158	3666	5.26E-06	0.005644483
G	T	0.1316	0.026	0.5821	3666	2.90E-07	0.007151271

C	T	-2.217	0.434	0.0008	3666	3.39E-07	0.007067716
T	C	-0.1129	0.025	0.4367	3666	5.83E-06	0.005585501
T	C	-0.1115	0.025	0.5139	3666	6.56E-06	0.005527847
T	C	1.831	0.405	0.001	3666	6.48E-06	0.005533588
T	C	0.112	0.025	0.5271	3666	7.39E-06	0.005471018
C	T	0.1762	0.038	0.1148	3666	4.28E-06	0.005746095
T	C	-2.379	0.514	0.0005	3666	3.75E-06	0.005820767
A	T	-0.1168	0.025	0.3627	3580	3.92E-06	0.005936767
A	C	0.2208	0.047	0.0739	3580	2.60E-06	0.006155637
A	G	-0.1261	0.027	0.2811	3580	4.05E-06	0.005915738
C	G	-0.1856	0.041	0.092	3580	7.20E-06	0.005612275
A	G	0.1944	0.043	0.0852	3580	6.02E-06	0.005705745
G	A	-0.945	0.211	0.0032	3580	7.80E-06	0.00556647
G	A	-0.4346	0.097	0.0156	3580	7.98E-06	0.005556637
G	A	-0.6975	0.157	0.0063	3580	8.65E-06	0.005510892
T	C	0.3263	0.045	0.2803	3580	6.53E-13	0.014335704
T	C	0.219	0.048	0.0774	3580	5.81E-06	0.005723973
C	T	-0.1621	0.033	0.1709	3580	1.02E-06	0.006650689
T	C	0.4445	0.096	0.0169	3580	3.81E-06	0.005947923
G	A	-0.4158	0.09	0.0176	3580	3.84E-06	0.005946474
A	G	-0.8376	0.166	0.0053	3580	4.46E-07	0.007095405
C	T	-0.1628	0.026	0.4168	3580	2.59E-10	0.011101646
C	T	0.1218	0.026	0.6642	3580	2.33E-06	0.006215651
A	T	-0.2226	0.043	0.9154	3580	2.71E-07	0.007358532
T	G	-0.1373	0.031	0.8108	3580	9.26E-06	0.005477642
T	G	0.2962	0.066	0.0332	3580	8.65E-06	0.005512817
T	C	1.194	0.252	0.0021	3580	2.30E-06	0.006217026
T	C	-0.4072	0.087	0.0201	3580	2.71E-06	0.006129499
T	G	0.1302	0.027	0.7331	3580	1.92E-06	0.006313397
T	C	-0.2209	0.045	0.0775	3580	1.05E-06	0.006633238
G	A	-0.2015	0.033	0.1635	3580	6.46E-10	0.010603985
A	G	-0.4355	0.027	0.7659	3580	3.62E-55	0.066094318
T	C	0.4026	0.08	0.0237	3580	4.65E-07	0.007073705
A	G	-0.1472	0.025	0.602	3580	3.32E-09	0.00972933
C	T	0.2303	0.048	0.0649	3580	2.11E-06	0.006266553
G	A	-0.1073	0.024	0.5177	3580	8.58E-06	0.005515709
C	T	1.498	0.327	0.0014	3580	4.74E-06	0.005831373
C	T	-0.1301	0.029	0.2124	3580	9.57E-06	0.005458562
A	G	0.3836	0.079	0.0225	3580	1.27E-06	0.006536305
T	C	-0.1428	0.03	0.1912	3580	2.37E-06	0.006202543
A	G	0.548	0.116	0.0097	3596	2.25E-06	0.006199745
A	G	-0.3105	0.07	0.0292	3596	9.51E-06	0.005440193
A	G	2.031	0.453	0.0006	3596	7.65E-06	0.005551509
A	G	0.3143	0.064	0.0317	3596	9.80E-07	0.00664345
C	T	-0.1411	0.031	0.1714	3596	6.53E-06	0.005637403
A	G	0.1693	0.029	0.3813	3596	9.31E-09	0.009137199
T	G	-1.414	0.303	0.0013	3596	3.08E-06	0.006039452
G	A	-0.3744	0.084	0.0178	3596	8.21E-06	0.005515028

C	T	-0.7017	0.158	0.005	3596	9.53E-06	0.005434441
G	A	-0.5382	0.12	0.009	3596	7.82E-06	0.005544268
A	G	0.1033	0.023	0.6279	3596	8.94E-06	0.005473597
A	G	0.2231	0.049	0.0628	3596	5.19E-06	0.005759813
T	C	-0.1471	0.024	0.4182	3596	1.46E-09	0.010120602
C	G	0.1347	0.03	0.8184	3596	5.39E-06	0.005741245
T	C	-0.9683	0.204	0.0028	3596	2.25E-06	0.006202067
A	G	0.2157	0.047	0.0601	3596	4.95E-06	0.005783808
A	G	-0.1849	0.039	0.0891	3596	2.43E-06	0.006161474
G	A	0.1021	0.023	0.46	3596	7.74E-06	0.00554558
T	C	-0.1125	0.023	0.5089	3596	8.19E-07	0.006742503
G	A	0.1937	0.044	0.0749	3596	9.23E-06	0.005456213
G	A	0.1028	0.023	0.3899	3596	9.23E-06	0.005453679
T	G	0.2888	0.062	0.0331	3596	3.70E-06	0.005936547
A	G	0.4774	0.107	0.0106	3596	7.56E-06	0.005556815
T	C	-0.3833	0.081	0.02	3596	2.64E-06	0.006116338
A	G	-0.4611	0.025	0.7661	3596	3.08E-70	0.083588105
G	T	-0.245	0.051	0.0517	3596	1.90E-06	0.006290584
G	A	-0.1328	0.023	0.5726	3596	9.71E-09	0.0091149
A	G	-0.2435	0.046	0.0654	3596	9.89E-08	0.007867167
A	G	-0.1065	0.023	0.3598	3596	5.73E-06	0.005703086
T	C	-0.3778	0.085	0.0204	3596	8.05E-06	0.005526893
C	T	1.483	0.307	0.0014	3596	1.39E-06	0.006455638
T	C	-0.2381	0.048	0.0598	3596	7.90E-07	0.006756823
T	C	1.729	0.34	0.0013	3596	3.95E-07	0.007123386
A	T	-0.1493	0.033	0.1402	3596	6.39E-06	0.005649659
A	G	0.5526	0.119	0.0097	3596	3.40E-06	0.005980864
A	G	-0.3197	0.072	0.0292	3596	8.95E-06	0.005471002
C	A	-1.265	0.284	0.0015	3596	8.48E-06	0.005498549
A	G	0.3005	0.066	0.0317	3596	5.16E-06	0.005761191
T	C	0.2699	0.044	0.2799	3596	1.19E-09	0.010235175
T	G	-1.445	0.311	0.0013	3596	3.43E-06	0.005982835
G	A	-0.3991	0.086	0.0178	3596	3.66E-06	0.005944995
C	T	-0.7231	0.155	0.0074	3596	3.23E-06	0.006008088
A	G	0.2474	0.05	0.0628	3596	8.50E-07	0.006719494
T	C	-0.1552	0.025	0.4182	3596	5.15E-10	0.010688046
A	G	-0.1798	0.04	0.0891	3596	8.04E-06	0.005529469
C	T	-0.1905	0.042	0.9175	3596	5.86E-06	0.005693844
A	G	0.2367	0.048	0.0601	3596	1.04E-06	0.006609757
T	C	-1.013	0.21	0.0028	3596	1.44E-06	0.006441421
T	C	-0.1084	0.023	0.5089	3596	3.70E-06	0.005942394
G	A	0.106	0.024	0.3899	3596	8.39E-06	0.005499695
A	G	0.4087	0.092	0.0161	3596	9.62E-06	0.005432175
T	G	0.3154	0.064	0.0331	3596	8.55E-07	0.006716761
T	C	-0.3761	0.084	0.02	3596	7.19E-06	0.005586127
A	G	0.5032	0.109	0.0106	3596	4.30E-06	0.005859614
C	T	-0.1296	0.029	0.2113	3596	6.31E-06	0.00565818
T	C	-0.5481	0.124	0.009	3596	9.97E-06	0.00541253

A	G	-0.4689	0.026	0.7661	3596	7.09E-69	0.082016317
T	C	0.4397	0.077	0.0236	3596	1.10E-08	0.00904229
A	G	-0.2598	0.047	0.0654	3596	3.05E-08	0.008496909
G	A	-0.1268	0.024	0.6022	3596	1.21E-07	0.00776026
T	C	1.618	0.35	0.0013	3596	3.85E-06	0.00591791
T	C	-0.2422	0.049	0.0598	3596	9.96E-07	0.006634864
T	C	-0.1659	0.037	0.1181	3653	9.12E-06	0.005374697
T	C	-0.4897	0.109	0.0127	3653	6.85E-06	0.005525165
A	G	-0.5201	0.106	0.0151	3653	9.85E-07	0.006535004
T	C	0.5615	0.119	0.0109	3653	2.66E-06	0.006017553
T	C	-0.1156	0.025	0.4316	3653	3.80E-06	0.005828308
C	A	0.8601	0.183	0.0042	3653	2.81E-06	0.005991196
A	G	0.4706	0.07	0.0328	3653	2.22E-11	0.012183457
A	G	-0.1918	0.039	0.8837	3653	9.20E-07	0.006574022
G	A	0.4624	0.073	0.0316	3653	2.44E-10	0.01091142
T	C	0.29	0.059	0.048	3653	1.08E-06	0.006491278
T	C	0.264	0.055	0.0547	3653	1.79E-06	0.006222561
C	G	0.1548	0.033	0.8214	3653	2.09E-06	0.006145814
T	C	0.1184	0.025	0.5463	3653	2.78E-06	0.005997236
C	T	0.1838	0.041	0.8968	3653	8.22E-06	0.005426459
G	A	0.1387	0.025	0.5116	3653	2.84E-08	0.008402224
G	T	0.231	0.051	0.0623	3653	6.52E-06	0.00555004
C	T	-0.1119	0.025	0.5475	3653	7.16E-06	0.00549816
A	G	-0.1122	0.025	0.5059	3653	6.02E-06	0.005589852
C	T	-0.2014	0.044	0.088	3653	5.16E-06	0.00567445
A	G	0.1234	0.027	0.6731	3653	4.19E-06	0.005778864
T	C	-0.3521	0.067	0.0345	3653	1.51E-07	0.007521279
T	C	-0.4086	0.039	0.1096	3653	2.79E-25	0.029113596
G	C	0.1721	0.031	0.2087	3653	2.78E-08	0.008409409
C	T	-0.1173	0.025	0.5672	3653	3.07E-06	0.005938356
T	A	0.7327	0.161	0.0059	3653	5.73E-06	0.005616784
T	C	0.3815	0.075	0.0279	3653	4.24E-07	0.006977639
A	G	0.117	0.026	0.3622	3653	9.44E-06	0.005355995
A	G	-0.346	0.075	0.0271	3653	4.06E-06	0.005795453
T	C	-0.207	0.041	0.1044	3653	3.80E-07	0.007041638
G	T	0.6919	0.149	0.0068	3653	3.41E-06	0.00588394
A	G	-0.1848	0.034	0.1574	3653	7.00E-08	0.007929478
C	T	0.1457	0.025	0.5123	3653	4.13E-09	0.00942024
T	C	0.3817	0.085	0.0227	3653	7.88E-06	0.005451582
C	A	0.1274	0.028	0.7178	3653	4.02E-06	0.005798895
T	C	-0.4558	0.092	0.0188	3653	8.05E-07	0.006642846
T	C	-0.3059	0.067	0.0354	3669	5.87E-06	0.005581255
A	G	-0.2072	0.045	0.0867	3669	4.25E-06	0.00574773
T	C	-0.5038	0.107	0.0134	3669	2.37E-06	0.006050877
A	G	0.4435	0.099	0.0151	3669	8.11E-06	0.005413877
G	A	-0.143	0.031	0.7822	3669	3.23E-06	0.005890215
T	C	0.3253	0.055	0.0545	3669	3.91E-09	0.009403699
A	G	0.2073	0.047	0.0777	3669	9.47E-06	0.005332751

T	A	0.2587	0.051	0.071	3669	3.80E-07	0.007005034
G	A	0.6063	0.077	0.0275	3669	5.37E-15	0.016524614
A	G	0.4184	0.066	0.0377	3669	2.83E-10	0.010789369
A	G	0.3723	0.059	0.0465	3669	3.91E-10	0.010614724
C	G	0.1585	0.033	0.8213	3669	1.30E-06	0.006366594
A	G	0.1595	0.035	0.156	3669	4.05E-06	0.005771813
G	A	0.148	0.025	0.5112	3669	3.25E-09	0.009499252
A	G	-0.119	0.025	0.5221	3669	2.22E-06	0.006084185
A	G	0.156	0.035	0.1536	3669	7.06E-06	0.005487871
C	T	-0.1174	0.025	0.5677	3669	3.15E-06	0.005903925
A	G	-0.435	0.04	0.1067	3669	1.04E-27	0.031919018
G	C	0.1713	0.031	0.2086	3669	3.41E-08	0.008269482
A	G	0.1233	0.027	0.6732	3669	4.36E-06	0.005736019
T	C	-0.3574	0.067	0.0345	3669	8.94E-08	0.007764695
T	A	0.7886	0.16	0.0059	3669	9.08E-07	0.00655305
T	C	0.3393	0.075	0.0278	3669	6.97E-06	0.005490369
C	T	-1.449	0.322	0.0012	3669	6.76E-06	0.005505913
A	G	-0.3311	0.075	0.0271	3669	9.60E-06	0.005324692
C	G	-0.4917	0.102	0.0147	3669	1.61E-06	0.006257134
T	G	-0.1885	0.039	0.1108	3669	1.59E-06	0.00625971
G	T	0.6822	0.148	0.0068	3669	4.23E-06	0.005749913
G	A	-0.1202	0.026	0.3863	3669	3.40E-06	0.005867548
G	A	-0.1591	0.034	0.1634	3669	2.44E-06	0.006034571
T	C	0.121	0.025	0.5271	3669	1.27E-06	0.006374598
T	C	0.14	0.03	0.2258	3669	2.34E-06	0.006060173
G	A	0.5042	0.113	0.012	3669	7.97E-06	0.005425596
G	A	-0.1333	0.029	0.2247	3669	5.18E-06	0.005644056
T	C	-0.4349	0.092	0.0189	3669	2.12E-06	0.006110305
C	T	0.1187	0.027	0.6529	3669	8.21E-06	0.005406168
A	G	0.452	0.1	0.0151	3669	6.13E-06	0.005557427
T	C	-0.4942	0.107	0.0134	3669	4.19E-06	0.005759169
A	G	-0.2097	0.045	0.0867	3669	3.72E-06	0.005819387
G	A	-0.1397	0.031	0.7822	3669	6.14E-06	0.005557945
A	G	0.4014	0.067	0.0377	3669	1.79E-09	0.009815144
G	A	0.5685	0.078	0.0275	3669	3.23E-13	0.014358846
T	A	0.2554	0.051	0.071	3669	6.26E-07	0.006746725
T	C	0.3109	0.055	0.0545	3669	2.25E-08	0.008489357
A	G	0.3581	0.06	0.0465	3669	2.21E-09	0.009708006
A	G	0.2118	0.047	0.0777	3669	6.81E-06	0.005502089
C	G	0.1568	0.033	0.8213	3669	1.93E-06	0.006156511
A	G	0.1541	0.035	0.156	3669	9.59E-06	0.005325119
G	A	0.1488	0.025	0.5112	3669	3.34E-09	0.009487909
C	T	-0.1142	0.025	0.5474	3669	5.25E-06	0.00563269
A	G	-0.1188	0.025	0.5221	3669	2.63E-06	0.005992501
A	G	0.1569	0.035	0.1536	3669	7.07E-06	0.005487877
G	C	0.1669	0.031	0.2086	3669	8.91E-08	0.007763615
C	T	-0.1215	0.025	0.5677	3669	1.63E-06	0.006246582
A	G	0.1221	0.027	0.6732	3669	6.14E-06	0.005559333

T	C	-0.3438	0.067	0.0345	3669	3.17E-07	0.00710222
A	G	-0.4214	0.04	0.1067	3669	8.64E-26	0.029605279
T	C	0.3614	0.076	0.0278	3669	1.93E-06	0.006157547
C	T	-1.438	0.323	0.0012	3669	8.97E-06	0.005359891
T	A	0.8085	0.161	0.0059	3669	5.57E-07	0.006809494
C	G	-0.4849	0.103	0.0147	3669	2.55E-06	0.006015965
T	G	-0.1858	0.039	0.1108	3669	2.56E-06	0.006009412
G	T	0.6865	0.149	0.0068	3669	4.19E-06	0.005752483
G	A	-0.1158	0.026	0.3863	3669	8.53E-06	0.005381624
T	C	0.1443	0.025	0.5271	3669	9.10E-09	0.008963122
G	A	-0.1866	0.034	0.1634	3669	3.82E-08	0.008204785
T	C	0.1388	0.03	0.2258	3669	3.24E-06	0.00588597
T	C	-0.2344	0.052	0.06	3669	8.03E-06	0.005418116
G	A	-0.1331	0.029	0.2247	3669	6.15E-06	0.005562656
T	C	-0.4399	0.092	0.0189	3669	1.86E-06	0.006178104
C	T	-0.2063	0.047	0.0797	3669	9.39E-06	0.005333784
A	G	-0.3548	0.074	0.0295	3579	1.53E-06	0.006439004
A	G	-0.4445	0.099	0.0148	3579	6.76E-06	0.005645225
A	C	0.1335	0.028	0.2311	3579	2.78E-06	0.006118938
T	C	-0.1129	0.024	0.5545	3579	3.69E-06	0.00597073
G	A	-0.9842	0.222	0.0027	3579	9.64E-06	0.005456719
A	G	0.3491	0.077	0.0239	3579	5.62E-06	0.005745997
G	A	0.3737	0.079	0.9761	3579	2.23E-06	0.006238404
G	A	-0.2281	0.048	0.0802	3579	1.93E-06	0.006314472
G	A	-0.1396	0.031	0.1949	3579	5.67E-06	0.005740494
G	T	-0.1766	0.04	0.1023	3579	9.98E-06	0.005441101
C	G	-0.1662	0.037	0.8806	3579	7.03E-06	0.005627182
G	A	-0.1511	0.026	0.4173	3579	4.75E-09	0.009529156
C	T	-0.1963	0.043	0.9173	3579	6.14E-06	0.005696651
A	C	0.1603	0.034	0.1357	3579	3.23E-06	0.006041082
A	G	0.1894	0.042	0.0884	3579	6.44E-06	0.00567134
C	T	0.1584	0.035	0.8639	3579	6.03E-06	0.005709736
C	G	-0.2544	0.051	0.227	3579	7.63E-07	0.006808563
T	A	0.1473	0.032	0.1707	3579	3.33E-06	0.006019339
A	G	-0.783	0.176	0.0047	3579	9.29E-06	0.005474953
G	T	-2.588	0.57	0.0004	3579	5.86E-06	0.005720953
G	T	-0.1151	0.025	0.6014	3579	3.35E-06	0.006016169
A	G	-0.2926	0.028	0.766	3579	1.40E-25	0.030086266
G	A	1.274	0.287	0.998	3579	9.48E-06	0.005467982
T	C	-0.1553	0.03	0.1913	3579	2.50E-07	0.007407358
G	C	0.1146	0.025	0.3564	3579	4.70E-06	0.005836939
G	A	-0.1415	0.031	0.1356	3595	5.86E-06	0.005696143
C	T	-1.176	0.266	0.0015	3595	9.64E-06	0.00542779
G	A	-0.1031	0.022	0.5772	3595	3.39E-06	0.005985106
A	C	0.1895	0.043	0.0691	3595	9.02E-06	0.005466487
A	C	-0.1195	0.026	0.2878	3595	3.26E-06	0.00601065
C	A	0.1268	0.026	0.3819	3595	1.28E-06	0.006507667
G	A	-0.5324	0.117	0.0088	3595	5.51E-06	0.005726789

T	A	-0.2578	0.057	0.0401	3595	5.47E-06	0.005731621
A	G	-0.4255	0.094	0.0134	3595	6.28E-06	0.005660114
A	C	-0.1399	0.03	0.1449	3595	4.52E-06	0.005837399
T	C	-0.1199	0.023	0.4182	3595	1.55E-07	0.007627176
A	G	-0.1728	0.037	0.0892	3595	4.14E-06	0.005877994
G	A	0.5775	0.13	0.0068	3595	9.41E-06	0.005442682
A	G	-0.5232	0.116	0.0086	3595	6.11E-06	0.005675456
T	C	0.1528	0.034	0.1122	3595	7.87E-06	0.005544477
T	G	0.1108	0.023	0.6476	3595	1.05E-06	0.006612458
C	T	0.1137	0.026	0.2399	3595	9.34E-06	0.005444448
A	G	-0.7092	0.158	0.0047	3595	7.70E-06	0.005552119
G	A	0.1107	0.024	0.7327	3595	6.05E-06	0.00568368
T	G	0.1124	0.025	0.6996	3595	6.26E-06	0.005658697
T	C	-0.2989	0.025	0.7744	3595	4.52E-32	0.037920199
T	C	0.1188	0.026	0.7811	3595	6.64E-06	0.005626676
T	C	-0.209	0.046	0.0598	3595	5.69E-06	0.005714351
G	A	-0.1105	0.023	0.7039	3595	2.43E-06	0.006159416
C	T	-0.1051	0.022	0.6328	3595	2.49E-06	0.006151716
A	G	-0.09863	0.022	0.611	3595	8.27E-06	0.005514753
T	C	-0.5593	0.119	0.0079	3595	2.68E-06	0.006107119
C	T	-1.26	0.273	0.0015	3595	4.06E-06	0.005890486
T	C	-0.103	0.023	0.6028	3595	5.40E-06	0.005739507
A	C	0.2016	0.044	0.0691	3595	4.41E-06	0.005847819
G	A	-0.1017	0.023	0.5775	3595	8.40E-06	0.005503974
A	G	0.1367	0.028	0.3812	3595	1.33E-06	0.00648483
G	A	-0.549	0.12	0.0088	3595	5.24E-06	0.00575978
C	T	-0.2348	0.053	0.0458	3595	8.45E-06	0.005503876
A	G	-0.4395	0.097	0.0134	3595	5.75E-06	0.005706133
T	C	-0.1334	0.023	0.4182	3595	1.39E-08	0.008921444
A	G	-0.1744	0.039	0.0892	3595	6.31E-06	0.005657907
T	G	0.1129	0.023	0.6476	3595	1.30E-06	0.006488585
A	T	0.1079	0.024	0.2983	3595	8.97E-06	0.005467934
C	T	0.1229	0.026	0.2399	3595	3.26E-06	0.006010297
T	C	0.1276	0.027	0.7811	3595	2.58E-06	0.006133558
A	G	-0.3114	0.026	0.7662	3595	1.34E-33	0.039799651
C	T	-0.2029	0.045	0.9363	3595	8.20E-06	0.005517936
G	A	-0.1153	0.024	0.7039	3595	1.75E-06	0.006337031
T	C	-0.2205	0.047	0.0598	3595	3.24E-06	0.0060112
G	A	-0.1073	0.023	0.4302	3595	2.40E-06	0.006171316
C	T	-0.1019	0.023	0.6328	3595	8.96E-06	0.005468132
T	C	-0.5613	0.122	0.0079	3595	4.67E-06	0.00581561
A	G	-0.11	0.023	0.611	3595	1.33E-06	0.006483764
G	C	-0.1377	0.03	0.7802	3579	3.22E-06	0.006042841
A	C	0.2308	0.048	0.0739	3579	1.25E-06	0.006545192
G	A	-0.2266	0.046	0.0768	3579	9.79E-07	0.006676745
C	T	0.2173	0.047	0.0742	3579	4.05E-06	0.005914582
A	G	-0.4444	0.097	0.0166	3579	4.76E-06	0.005831663
A	G	0.196	0.044	0.0852	3579	6.80E-06	0.005640471

G	A	-0.3262	0.068	0.0337	3579	1.53E-06	0.006439297
G	A	-0.1305	0.029	0.228	3579	8.32E-06	0.005534702
C	T	-0.1496	0.034	0.1707	3579	8.81E-06	0.005505123
G	T	0.2434	0.031	0.3061	3579	5.16E-15	0.016954702
T	C	0.4692	0.097	0.0169	3579	1.50E-06	0.006448708
T	G	0.1607	0.031	0.277	3579	3.43E-07	0.007237656
G	A	-0.4225	0.091	0.0176	3579	3.67E-06	0.005971223
T	G	-0.2997	0.067	0.0362	3579	6.88E-06	0.005634582
T	C	-0.2114	0.047	0.0735	3579	7.27E-06	0.00560901
C	T	-0.8815	0.171	0.005	3579	2.65E-07	0.007378743
C	T	-0.1446	0.026	0.4167	3579	3.17E-08	0.00851619
C	T	0.1243	0.026	0.6642	3579	2.01E-06	0.006297323
T	G	0.2373	0.046	0.0759	3579	2.27E-07	0.007461036
A	G	-0.1197	0.025	0.5085	3579	1.18E-06	0.006577239
A	G	0.1456	0.03	0.2195	3579	1.05E-06	0.006639119
T	C	1.176	0.256	0.0021	3579	4.39E-06	0.005870765
T	C	-0.4162	0.088	0.0201	3579	2.26E-06	0.006229416
T	G	0.1239	0.028	0.733	3579	7.93E-06	0.005563041
A	G	0.1133	0.025	0.4202	3579	4.82E-06	0.005825875
C	A	-0.2253	0.05	0.0645	3579	5.96E-06	0.005713589
G	A	-0.1331	0.025	0.5721	3579	1.06E-07	0.007870061
A	G	-0.4286	0.028	0.766	3579	5.57E-52	0.06227699
T	C	0.3535	0.071	0.0302	3579	7.79E-07	0.006796636
G	A	-0.2122	0.033	0.1636	3579	1.36E-10	0.01144864
C	T	-0.1242	0.028	0.2543	3579	9.05E-06	0.005494734
C	T	1.495	0.331	0.0014	3579	6.69E-06	0.005653966
A	G	1.513	0.336	0.0014	3579	7.06E-06	0.005623585
G	A	0.1906	0.042	0.0911	3579	6.98E-06	0.005630284
A	G	-0.1869	0.041	0.0965	3579	4.78E-06	0.005829048
A	G	0.3437	0.075	0.0259	3595	5.31E-06	0.00574968
C	G	1.339	0.295	0.0015	3595	5.71E-06	0.005709725
G	C	-0.1258	0.028	0.7801	3595	9.64E-06	0.005435887
G	A	-0.2178	0.044	0.0769	3595	1.00E-06	0.006634108
G	T	0.1984	0.03	0.3057	3595	3.79E-11	0.012091174
T	C	-0.156	0.034	0.1401	3595	3.61E-06	0.005949834
G	A	-0.1252	0.027	0.2338	3595	4.97E-06	0.005782614
T	A	-0.2187	0.049	0.0622	3595	7.26E-06	0.005585262
C	T	-0.8053	0.165	0.005	3595	1.08E-06	0.006590276
T	A	-0.1878	0.042	0.0823	3595	9.12E-06	0.005463288
A	G	-0.1339	0.025	0.4179	3595	9.86E-08	0.007866424
C	T	0.1171	0.024	0.636	3595	1.64E-06	0.006365928
C	T	0.3641	0.078	0.0257	3595	2.95E-06	0.00606163
C	G	-1.02	0.213	0.0028	3595	1.70E-06	0.00635026
G	A	0.2117	0.045	0.075	3595	3.16E-06	0.006022451
T	C	-0.1324	0.029	0.1944	3595	6.90E-06	0.005609696
T	C	-0.1295	0.024	0.5089	3595	4.62E-08	0.008278193
T	G	0.2975	0.065	0.0331	3595	4.79E-06	0.005802158
A	G	0.1329	0.029	0.2195	3595	3.59E-06	0.005954037

T	C	-0.4018	0.085	0.02	3595	2.18E-06	0.006222229
G	C	-1.263	0.274	0.0019	3595	4.11E-06	0.005884072
A	G	-0.4526	0.027	0.7662	3595	1.47E-62	0.074581594
G	A	-0.1196	0.024	0.6024	3595	8.46E-07	0.006726161
G	A	-0.1852	0.032	0.1634	3595	5.96E-09	0.009369871
T	C	0.4156	0.078	0.0236	3595	1.00E-07	0.007861118
T	C	0.3608	0.069	0.03	3595	1.69E-07	0.007583097
G	A	0.5431	0.119	0.9908	3595	5.37E-06	0.005741265
C	T	1.501	0.319	0.0014	3595	2.68E-06	0.006105664
G	A	0.1844	0.041	0.0911	3595	6.19E-06	0.005666475
A	G	1.66	0.324	0.0014	3595	3.13E-07	0.00725327
T	C	0.2936	0.066	0.0327	3595	9.48E-06	0.005439977
G	A	0.1427	0.031	0.1819	3595	4.45E-06	0.005844668
G	A	0.2643	0.056	0.0477	3595	2.18E-06	0.006221836
G	A	-0.2164	0.045	0.0769	3595	1.66E-06	0.006366218
C	G	-0.2868	0.063	0.0369	3595	5.13E-06	0.005768034
G	C	-0.1297	0.029	0.7801	3595	6.82E-06	0.005613747
G	T	0.2165	0.03	0.3057	3595	1.11E-12	0.013993451
G	A	-0.1264	0.028	0.2338	3595	5.49E-06	0.005729911
G	A	-0.3958	0.088	0.0178	3595	7.93E-06	0.00553668
T	C	-0.1636	0.034	0.1401	3595	1.68E-06	0.006358199
C	T	-0.8001	0.167	0.005	3595	1.79E-06	0.00632184
T	A	-0.2241	0.049	0.0622	3595	5.85E-06	0.00569871
C	A	-0.1165	0.025	0.347	3595	3.30E-06	0.006004232
A	G	-0.1372	0.025	0.4179	3595	7.38E-08	0.008025565
A	G	0.2485	0.052	0.0629	3595	1.50E-06	0.00641994
C	G	-1.027	0.216	0.0028	3595	2.03E-06	0.006254776
C	T	-0.2005	0.043	0.9184	3595	3.74E-06	0.005931588
T	C	-0.1259	0.024	0.5089	3595	1.61E-07	0.00760287
G	A	0.2085	0.046	0.075	3595	6.08E-06	0.005677372
A	G	0.1332	0.029	0.2195	3595	4.76E-06	0.005810154
T	G	0.3118	0.066	0.0331	3595	2.29E-06	0.006194119
T	C	-0.3949	0.086	0.02	3595	4.47E-06	0.00584039
G	C	-1.251	0.278	0.0019	3595	6.92E-06	0.005609295
A	T	0.2531	0.055	0.0494	3595	5.16E-06	0.005761992
G	T	0.1168	0.024	0.4408	3595	1.64E-06	0.006369823
T	C	0.3662	0.07	0.03	3595	1.66E-07	0.007591788
A	G	-0.4525	0.027	0.7662	3595	9.23E-61	0.072466974
G	A	-0.1929	0.032	0.1634	3595	2.31E-09	0.009878086
T	C	0.4259	0.079	0.0236	3595	7.38E-08	0.00802386
G	A	0.1841	0.041	0.0911	3595	8.72E-06	0.005488927
T	C	0.1702	0.038	0.1093	3595	7.56E-06	0.005557999
A	G	-0.1864	0.04	0.0968	3595	2.96E-06	0.006058308
T	C	1.695	0.359	0.0013	3595	2.47E-06	0.006152418
G	A	0.1464	0.031	0.1819	3595	3.42E-06	0.005980106
G	A	0.259	0.057	0.0477	3595	4.75E-06	0.005807202
T	C	0.142	0.031	0.1967	3652	5.90E-06	0.005604237
C	G	0.1451	0.032	0.1933	3652	4.57E-06	0.005736626

A	G	-1.538	0.32	0.0014	3652	1.60E-06	0.006285554
T	C	-0.1251	0.027	0.7134	3652	4.32E-06	0.005767306
A	G	0.2065	0.046	0.0776	3652	9.21E-06	0.005373414
C	T	-0.1338	0.029	0.5481	3652	2.96E-06	0.005965659
C	T	0.1671	0.03	0.3642	3652	3.31E-08	0.008324407
T	C	0.1122	0.025	0.5344	3652	7.25E-06	0.005498237
T	C	0.2742	0.058	0.0487	3652	2.03E-06	0.006162727
C	G	0.1523	0.032	0.8213	3652	2.59E-06	0.006036144
T	C	0.1169	0.025	0.5122	3652	2.89E-06	0.005975226
C	A	0.3468	0.076	0.0259	3652	5.35E-06	0.005654516
G	A	0.1325	0.027	0.3159	3652	1.35E-06	0.006376367
A	G	-0.1298	0.026	0.5795	3652	4.73E-07	0.006920272
T	C	-0.3155	0.067	0.0345	3652	2.30E-06	0.006094696
T	C	-0.3964	0.039	0.1097	3652	4.65E-24	0.027647821
G	C	0.1477	0.031	0.2088	3652	1.69E-06	0.006261566
C	T	0.3176	0.067	0.0356	3652	2.49E-06	0.006054061
T	C	-0.1237	0.025	0.4121	3652	6.76E-07	0.006733982
T	C	0.34	0.075	0.0279	3652	6.01E-06	0.005595868
A	G	0.1113	0.025	0.4261	3652	8.03E-06	0.005445507
A	G	-0.7438	0.164	0.0052	3652	5.96E-06	0.005600859
T	C	-0.4686	0.104	0.0141	3652	6.63E-06	0.005538989
G	A	-0.2038	0.04	0.1123	3652	2.84E-07	0.007193056
T	C	0.1138	0.025	0.5487	3652	4.33E-06	0.005764937
G	A	0.1228	0.028	0.7178	3652	8.20E-06	0.005430449
T	C	0.1155	0.025	0.3627	3652	6.03E-06	0.005599338
T	C	-0.5656	0.124	0.0087	3668	5.08E-06	0.005658272
A	G	-0.5427	0.118	0.0115	3668	4.58E-06	0.005714346
T	A	0.3864	0.087	0.0196	3668	9.66E-06	0.005323463
G	A	-0.193	0.042	0.0994	3668	3.79E-06	0.005811638
T	A	0.2426	0.053	0.0645	3668	5.25E-06	0.005637335
A	G	-1.451	0.316	0.0014	3668	4.62E-06	0.005704565
T	C	0.1933	0.043	0.0859	3668	9.06E-06	0.005357014
C	A	0.1762	0.039	0.1095	3668	6.67E-06	0.005517156
A	G	0.209	0.046	0.0777	3668	5.32E-06	0.005635335
G	A	0.1393	0.03	0.5643	3668	4.94E-06	0.005673192
T	C	0.2565	0.057	0.0488	3668	6.62E-06	0.005521191
A	G	0.1529	0.032	0.8246	3668	2.49E-06	0.006027455
G	A	0.1198	0.025	0.6125	3668	1.92E-06	0.006162549
T	C	-0.1196	0.025	0.5781	3668	2.63E-06	0.005998861
C	T	-0.2409	0.052	0.0609	3668	3.06E-06	0.005923031
T	C	-0.1212	0.025	0.4124	3668	8.25E-07	0.006606147
G	C	0.142	0.03	0.2087	3668	3.02E-06	0.005924845
C	T	0.1093	0.025	0.5055	3668	9.52E-06	0.005327274
C	T	0.3243	0.066	0.0361	3668	9.69E-07	0.006517641
G	A	-0.5116	0.113	0.012	3668	6.20E-06	0.005557183
T	C	-0.3117	0.066	0.0345	3668	2.18E-06	0.006098973
T	C	-0.406	0.039	0.1088	3668	1.41E-25	0.029366658
T	C	-0.4711	0.102	0.0142	3668	4.08E-06	0.005770746

T	G	-0.8148	0.177	0.0046	3668	4.27E-06	0.005744131
A	G	-0.7571	0.162	0.0052	3668	3.12E-06	0.005912021
G	A	-0.116	0.025	0.3867	3668	4.32E-06	0.005743597
G	A	-0.1133	0.025	0.6265	3668	4.12E-06	0.005768479
C	T	-0.1378	0.031	0.2019	3668	8.26E-06	0.005406583
A	G	-0.2247	0.049	0.0626	3668	5.78E-06	0.005588664
T	A	0.392	0.088	0.0196	3668	8.10E-06	0.005413636
T	C	-0.5546	0.125	0.0087	3668	8.76E-06	0.005372234
C	G	0.1419	0.031	0.1936	3668	6.12E-06	0.005561508
G	A	-0.1981	0.042	0.0994	3668	2.39E-06	0.006048603
A	G	0.2504	0.055	0.0537	3668	5.96E-06	0.005574664
A	G	-1.466	0.318	0.0014	3668	4.19E-06	0.005753503
T	C	0.1947	0.044	0.0859	3668	8.90E-06	0.005367974
C	A	0.1761	0.039	0.1095	3668	7.69E-06	0.005444192
A	G	0.2125	0.046	0.0777	3668	4.19E-06	0.005756929
C	T	0.1479	0.03	0.3721	3668	7.87E-07	0.006626394
T	C	0.2652	0.057	0.0488	3668	3.64E-06	0.005830287
C	G	0.1491	0.032	0.8213	3668	3.52E-06	0.005847482
G	A	0.1222	0.025	0.6125	3668	1.39E-06	0.006334942
T	C	-0.1213	0.026	0.5781	3668	2.18E-06	0.006097818
C	T	0.1113	0.025	0.504	3668	7.41E-06	0.005456714
T	C	-0.3039	0.066	0.0345	3668	4.44E-06	0.005727999
C	G	-0.45	0.101	0.0149	3668	8.79E-06	0.005372222
T	C	-0.1242	0.025	0.4124	3668	5.06E-07	0.006851503
T	C	-0.3961	0.039	0.1095	3668	2.18E-24	0.027921087
G	C	0.14	0.031	0.2087	3668	4.76E-06	0.005692787
C	T	0.324	0.067	0.0361	3668	1.15E-06	0.006428151
A	G	-0.7638	0.163	0.0052	3668	2.93E-06	0.005943376
T	C	-0.4752	0.103	0.0142	3668	3.84E-06	0.005803034
T	G	-0.7971	0.178	0.0046	3668	7.81E-06	0.005431293
G	A	-0.1113	0.025	0.6265	3668	6.91E-06	0.005500726
G	A	-0.1138	0.025	0.3867	3668	7.34E-06	0.005464114
T	C	-0.1928	0.039	0.117	3668	5.85E-07	0.006780038
C	T	-0.1404	0.031	0.2019	3668	6.32E-06	0.00554329
A	G	-0.4541	0.101	0.0142	3668	6.73E-06	0.005513326
A	G	-0.7877	0.175	0.0048	3652	7.16E-06	0.005504597
A	C	0.3374	0.074	0.0281	3652	5.31E-06	0.005658666
A	G	-0.1853	0.039	0.1088	3652	2.37E-06	0.006081333
T	C	0.1566	0.032	0.187	3652	7.44E-07	0.006688274
G	A	0.9023	0.196	0.0038	3652	4.18E-06	0.005781329
C	G	0.1478	0.033	0.1666	3652	8.86E-06	0.005387797
T	C	-0.3842	0.083	0.0227	3652	4.20E-06	0.005781575
A	G	0.5556	0.065	0.0379	3652	1.16E-17	0.01985272
A	G	0.3428	0.058	0.0465	3652	4.40E-09	0.009391007
A	G	0.5462	0.114	0.0116	3652	1.63E-06	0.006279369
G	A	0.6679	0.076	0.0277	3652	1.38E-18	0.020979257
T	C	0.3487	0.054	0.0548	3652	1.40E-10	0.011210849
T	G	0.1508	0.034	0.1588	3652	9.42E-06	0.005357732

C	T	0.1197	0.026	0.346	3652	4.51E-06	0.005739521
G	A	-0.2049	0.046	0.0753	3652	8.27E-06	0.00542941
G	A	0.1277	0.025	0.613	3652	4.27E-07	0.006976935
A	T	0.2371	0.052	0.059	3652	5.63E-06	0.005630385
T	C	1.516	0.341	0.0012	3652	8.92E-06	0.005386023
G	A	-0.1466	0.033	0.1733	3652	7.52E-06	0.005480076
A	G	-0.3231	0.039	0.1069	3652	2.69E-16	0.018180702
T	C	-0.1793	0.039	0.1124	3652	3.71E-06	0.005843361
G	A	-0.1507	0.033	0.1584	3652	6.34E-06	0.005570061
A	G	0.4704	0.094	0.0167	3652	5.71E-07	0.006823491
T	C	0.3268	0.069	0.0342	3652	2.37E-06	0.006078564
T	C	-0.3314	0.07	0.0315	3652	2.21E-06	0.006117233
G	A	-0.1628	0.033	0.1632	3652	1.04E-06	0.006509909
T	G	0.1472	0.025	0.5685	3652	2.20E-09	0.009748268
A	G	-0.1143	0.025	0.4225	3652	4.23E-06	0.005782819
A	G	0.1224	0.027	0.6105	3652	6.84E-06	0.005526447
T	A	0.1305	0.028	0.2848	3652	3.46E-06	0.005883594
A	C	0.329	0.074	0.0284	3668	8.80E-06	0.00537444
T	C	0.1496	0.032	0.1872	3668	2.58E-06	0.006008721
G	A	0.8992	0.197	0.0038	3668	5.42E-06	0.005625214
T	C	-0.1138	0.026	0.5634	3668	8.84E-06	0.00536682
G	A	0.7354	0.076	0.0275	3668	6.79E-22	0.024897476
T	A	0.2439	0.05	0.071	3668	1.30E-06	0.006366662
T	C	0.3978	0.055	0.0545	3668	3.73E-13	0.014290897
A	G	0.5985	0.065	0.0378	3668	5.81E-20	0.022557735
A	G	0.352	0.059	0.0465	3668	2.17E-09	0.009718129
C	T	0.1169	0.026	0.3461	3668	8.71E-06	0.005377733
G	A	0.1399	0.025	0.6125	3668	3.56E-08	0.008247815
A	G	-0.3265	0.04	0.1067	3668	2.06E-16	0.018250072
T	C	-0.435	0.093	0.0176	3668	3.25E-06	0.005888906
T	C	-0.1823	0.039	0.1122	3668	3.00E-06	0.005933644
T	C	-0.2409	0.052	0.06	3668	3.76E-06	0.005812619
G	A	-0.1579	0.034	0.1581	3668	2.63E-06	0.005995434
A	G	0.4594	0.095	0.0166	3668	1.28E-06	0.006374927
C	T	0.2544	0.056	0.0543	3668	6.10E-06	0.005559303
C	G	-0.1334	0.03	0.2199	3668	7.33E-06	0.005462681
A	G	-0.4672	0.09	0.02	3668	2.10E-07	0.007318913
T	C	0.1146	0.025	0.527	3668	3.89E-06	0.005792471
T	C	0.2127	0.047	0.0819	3668	7.27E-06	0.005471206
C	T	0.3643	0.082	0.023	3668	9.71E-06	0.00532244
T	C	-0.1159	0.025	0.5634	3668	5.50E-06	0.005613515
T	C	0.1486	0.032	0.1872	3668	2.74E-06	0.005981422
G	A	0.892	0.197	0.0038	3668	5.86E-06	0.005580882
A	G	-0.1753	0.039	0.1086	3668	8.64E-06	0.0053842
T	A	-0.16	0.036	0.1382	3668	7.60E-06	0.005449347
G	A	0.416	0.094	0.0194	3668	8.93E-06	0.00536552
A	G	0.3457	0.058	0.0465	3668	3.57E-09	0.009456273
G	A	0.7078	0.076	0.0275	3668	1.51E-20	0.023267317

T	A	0.2428	0.05	0.071	3668	1.31E-06	0.006367445
A	G	0.5857	0.065	0.0378	3668	2.47E-19	0.021793928
T	C	0.3865	0.054	0.0545	3668	1.35E-12	0.013609406
C	T	0.1164	0.026	0.3461	3668	8.71E-06	0.005380896
G	A	0.1407	0.025	0.6125	3668	2.55E-08	0.008420555
A	G	-0.3205	0.039	0.1067	3668	5.38E-16	0.017746772
G	A	-0.1467	0.033	0.1734	3668	7.87E-06	0.005430603
T	C	-0.1853	0.039	0.1122	3668	1.85E-06	0.006186016
T	C	-0.4301	0.093	0.0176	3668	3.79E-06	0.005808376
T	C	-0.241	0.052	0.06	3668	3.38E-06	0.005868898
G	A	-0.157	0.033	0.1581	3668	2.71E-06	0.005980698
A	G	0.4611	0.094	0.0166	3668	1.05E-06	0.006478864
C	T	0.4474	0.096	0.017	3668	3.47E-06	0.005854916
C	T	0.2515	0.056	0.0543	3668	7.10E-06	0.005482385
G	T	-0.4652	0.101	0.0169	3668	4.56E-06	0.005716647
T	G	0.1437	0.025	0.5684	3668	5.79E-09	0.009194829
C	G	-0.1324	0.03	0.2199	3668	7.90E-06	0.00542866
T	C	-0.3543	0.07	0.0316	3668	4.20E-07	0.006955484
A	G	0.5445	0.121	0.0102	3668	7.13E-06	0.005481395
T	A	0.1265	0.028	0.2842	3668	7.24E-06	0.005475332
T	C	0.209	0.047	0.0819	3668	9.53E-06	0.005330438
T	A	-0.2119	0.046	0.0752	3652	4.40E-06	0.005757033
G	T	0.3658	0.082	0.023	3652	7.39E-06	0.005485956
G	A	-0.1199	0.026	0.3443	3652	4.19E-06	0.005785051
C	G	0.1473	0.032	0.1825	3652	4.57E-06	0.005739922
G	A	-0.1283	0.025	0.5968	3652	3.33E-07	0.007109232
T	C	-0.1625	0.036	0.1182	3652	8.56E-06	0.005409869
G	T	0.1269	0.028	0.7555	3652	6.98E-06	0.00551433
T	A	0.2127	0.031	0.1952	3652	5.12E-12	0.012965136
G	A	-0.1853	0.042	0.0904	3652	8.65E-06	0.005403564
A	C	-0.2347	0.052	0.0583	3652	6.91E-06	0.005521815
A	G	-0.1282	0.027	0.7133	3652	1.73E-06	0.006245285
G	C	-0.2166	0.049	0.0668	3668	9.61E-06	0.005324839
G	T	0.3772	0.082	0.023	3668	4.68E-06	0.005701107
C	G	0.1565	0.032	0.1821	3668	1.48E-06	0.006301221
T	G	0.4126	0.09	0.0194	3668	4.52E-06	0.005717412
T	C	0.1238	0.028	0.7356	3668	8.93E-06	0.005362144
T	C	0.2272	0.031	0.1915	3668	6.35E-13	0.014010898
A	G	0.3624	0.074	0.029	3668	1.07E-06	0.006471756
A	G	0.4373	0.098	0.0151	3668	8.80E-06	0.005372955
G	A	-0.118	0.025	0.5966	3668	3.39E-06	0.005867868
G	A	-0.1984	0.042	0.0902	3668	2.48E-06	0.006029606
G	T	-0.2033	0.045	0.0817	3668	5.63E-06	0.005602757
A	G	-0.1293	0.027	0.7135	3668	1.83E-06	0.006186104
A	G	-0.1271	0.027	0.2779	3668	3.68E-06	0.005827809
A	G	0.3099	0.065	0.0397	3668	1.77E-06	0.006206268
T	A	0.2524	0.05	0.071	3668	4.99E-07	0.006866491
T	C	0.3694	0.054	0.0545	3668	1.25E-11	0.012432892

G	A	0.7037	0.076	0.0275	3668	2.75E-20	0.022951388
A	G	0.564	0.065	0.0378	3668	5.29E-18	0.020186026
C	T	0.1519	0.034	0.166	3668	6.54E-06	0.005527984
G	A	0.3542	0.075	0.0282	3668	2.34E-06	0.006061493
A	G	0.3806	0.058	0.0465	3668	8.38E-11	0.011435144
C	T	-0.2398	0.053	0.0568	3668	5.95E-06	0.00557517
A	G	-0.3832	0.039	0.1067	3668	2.68E-22	0.025391009
T	C	-0.3195	0.066	0.0345	3668	1.34E-06	0.00635023
A	G	-0.4738	0.107	0.0135	3668	9.22E-06	0.005346919
T	C	-0.4323	0.093	0.0176	3668	3.31E-06	0.005878917
T	A	0.3766	0.08	0.0228	3668	2.92E-06	0.005944588
G	T	0.1815	0.04	0.1091	3668	6.27E-06	0.005545879
A	G	-0.1319	0.029	0.733	3668	4.45E-06	0.00572536
T	C	0.6269	0.14	0.0076	3668	8.09E-06	0.005413702
C	T	0.2529	0.056	0.0543	3668	6.82E-06	0.005504036
G	A	0.1336	0.026	0.6738	3668	3.33E-07	0.007081914
T	C	-0.4132	0.091	0.0189	3668	5.39E-06	0.005625111
G	T	0.3751	0.082	0.023	3668	4.82E-06	0.005683418
T	C	-0.5199	0.116	0.0115	3668	7.79E-06	0.005437235
C	G	0.1528	0.032	0.1821	3668	2.37E-06	0.006056703
G	C	-0.224	0.049	0.0668	3668	4.30E-06	0.005741736
A	G	0.2338	0.051	0.0611	3668	5.11E-06	0.005652725
A	G	0.4432	0.098	0.0151	3668	6.09E-06	0.00556307
T	C	0.2197	0.031	0.1915	3668	2.88E-12	0.01321233
G	A	-0.1185	0.025	0.5966	3668	2.80E-06	0.005968767
A	G	0.3487	0.074	0.029	3668	2.43E-06	0.006039833
G	A	-0.1997	0.042	0.0902	3668	1.93E-06	0.006157771
G	T	-0.2013	0.045	0.0817	3668	6.39E-06	0.005537912
G	A	0.1799	0.039	0.1065	3668	4.16E-06	0.005758743
A	G	0.306	0.064	0.0397	3668	2.17E-06	0.006100581
A	G	0.3698	0.058	0.0465	3668	2.39E-10	0.010883206
A	G	0.5505	0.065	0.0378	3668	2.39E-17	0.019384168
T	C	0.3576	0.054	0.0545	3668	4.64E-11	0.011745652
T	A	0.2498	0.05	0.071	3668	5.90E-07	0.006780343
T	G	0.1268	0.027	0.2803	3668	2.58E-06	0.006007855
G	A	0.675	0.076	0.0275	3668	6.50E-19	0.021287972
C	T	-0.2382	0.053	0.0568	3668	6.28E-06	0.005545141
A	G	-0.3774	0.039	0.1067	3668	7.85E-22	0.024832007
T	C	-0.3106	0.066	0.0345	3668	2.38E-06	0.006050768
T	A	0.367	0.08	0.0228	3668	4.77E-06	0.005690616
T	C	-0.4258	0.092	0.0176	3668	4.25E-06	0.005749946
G	T	0.1844	0.04	0.1091	3668	4.09E-06	0.005772016
A	G	-0.1273	0.029	0.733	3668	8.73E-06	0.005375986
T	C	0.6311	0.14	0.0076	3668	6.46E-06	0.00553305
C	T	0.2508	0.056	0.0543	3668	7.49E-06	0.005457896
T	G	0.1122	0.025	0.3799	3668	8.70E-06	0.005375446
C	T	0.1378	0.024	0.5123	3668	1.54E-08	0.008683831
A	G	-0.1515	0.034	0.1576	3668	6.71E-06	0.005508836

T	C	-0.416	0.09	0.0189	3668	4.27E-06	0.005747696
G	A	0.4159	0.09	0.0201	3653	4.43E-06	0.005751932
C	T	0.1153	0.025	0.4655	3653	4.72E-06	0.005720598
G	A	-1.353	0.227	0.0027	3653	2.63E-09	0.009648266
C	A	-0.2803	0.06	0.0427	3653	3.03E-06	0.00595074
A	G	0.133	0.028	0.2836	3653	1.46E-06	0.006330269
T	C	-0.2945	0.063	0.0393	3653	3.61E-06	0.005859113
A	T	0.1998	0.042	0.101	3653	1.62E-06	0.006281102
C	T	0.1584	0.035	0.1567	3653	5.25E-06	0.005658957
C	T	-0.1251	0.027	0.2976	3653	5.26E-06	0.005665815
T	C	-0.1885	0.041	0.1046	3653	4.41E-06	0.005753069
A	T	-0.2105	0.046	0.0756	3653	5.15E-06	0.00567275
C	G	0.6203	0.14	0.0078	3653	9.56E-06	0.00535288
T	A	-0.2261	0.045	0.0829	3653	3.96E-07	0.007014219
C	A	-0.1186	0.025	0.4387	3653	3.18E-06	0.005923636
G	C	0.1184	0.026	0.467	3653	5.71E-06	0.005623274
C	T	0.1842	0.037	0.1336	3653	5.10E-07	0.006882257
A	C	-0.1278	0.026	0.318	3653	1.11E-06	0.006481102
T	C	-0.1125	0.025	0.4277	3653	8.67E-06	0.005400536
G	A	-0.2119	0.043	0.0924	3653	9.64E-07	0.006549276
C	T	0.157	0.034	0.1374	3669	5.39E-06	0.005628876
G	C	-0.1497	0.032	0.1668	3669	2.58E-06	0.006011316
C	T	-0.2948	0.065	0.4685	3669	6.38E-06	0.005536069
G	A	0.2806	0.063	0.9629	3669	8.36E-06	0.005398235
C	T	-0.1298	0.029	0.2254	3669	9.50E-06	0.005331319
G	A	0.1588	0.033	0.291	3669	1.42E-06	0.006317385
G	A	0.1345	0.029	0.6419	3669	4.41E-06	0.00573383
C	T	0.6097	0.134	0.0087	3669	5.29E-06	0.005635952
T	C	0.3983	0.09	0.0194	3669	9.38E-06	0.005336868
G	A	-1.044	0.23	0.0027	3669	5.97E-06	0.005574615
C	T	0.1702	0.036	0.1374	3669	2.20E-06	0.006088777
A	C	0.4251	0.093	0.0194	3669	4.79E-06	0.005686719
G	C	-0.1545	0.033	0.1668	3669	3.22E-06	0.00589604
C	T	-0.9915	0.221	0.003	3669	7.75E-06	0.005436445
G	A	0.2289	0.047	0.0775	3669	1.37E-06	0.006337142
A	G	0.1122	0.025	0.4484	3669	6.16E-06	0.005552223
G	A	-0.1762	0.036	0.8609	3669	1.11E-06	0.006451176
T	C	-1.173	0.261	0.0022	3669	7.15E-06	0.005474986
A	G	0.3699	0.083	0.0245	3669	7.86E-06	0.00542833
G	A	0.1221	0.027	0.319	3669	6.41E-06	0.005530736
A	C	-0.1269	0.026	0.4325	3669	8.14E-07	0.006606458
C	G	1.141	0.255	0.0022	3669	7.76E-06	0.005435726
C	A	-0.1257	0.028	0.2521	3669	9.16E-06	0.005348364
C	T	0.2194	0.037	0.134	3669	2.99E-09	0.009543512
A	G	0.1577	0.035	0.1524	3669	7.22E-06	0.00547786
A	G	-0.1324	0.027	0.3227	3669	6.96E-07	0.006692213
A	G	-0.6578	0.147	0.0072	3669	8.39E-06	0.00539876
T	A	0.1093	0.024	0.5371	3653	7.16E-06	0.005498811

T	C	0.1875	0.042	0.0965	3653	7.19E-06	0.005498821
C	G	0.1802	0.04	0.1043	3653	5.99E-06	0.005597145
G	A	0.1112	0.025	0.4294	3653	8.12E-06	0.005438645
A	C	0.1124	0.025	0.3603	3653	8.78E-06	0.005395239
G	A	-0.1187	0.024	0.5182	3653	7.30E-07	0.006695941
A	G	-0.3328	0.067	0.0342	3653	5.91E-07	0.006805294
C	A	-0.1305	0.028	0.2447	3653	3.30E-06	0.00590707
A	G	0.1243	0.028	0.2498	3653	9.78E-06	0.00533928
T	A	-0.1161	0.026	0.6789	3653	6.20E-06	0.005577135
C	T	0.144	0.032	0.8277	3653	7.22E-06	0.005499149
G	A	-0.4542	0.098	0.0159	3653	3.48E-06	0.005876789
G	T	0.2916	0.065	0.0371	3653	6.97E-06	0.005514522
G	A	0.1209	0.026	0.6731	3653	3.69E-06	0.005848444
G	T	-0.1269	0.027	0.2658	3653	3.31E-06	0.005901602
C	T	0.4493	0.085	0.0216	3653	1.21E-07	0.007638685
T	G	-0.1282	0.029	0.2278	3653	8.17E-06	0.005436231
G	A	-1.048	0.194	0.0037	3653	7.04E-08	0.007917166
G	A	0.1852	0.036	0.1313	3653	2.38E-07	0.007280782
G	A	-0.1735	0.036	0.1291	3653	1.96E-06	0.00618093
G	A	0.1537	0.024	0.4777	3653	1.83E-10	0.011066146
T	C	-0.1147	0.024	0.4989	3653	2.81E-06	0.005993273
T	C	-0.1258	0.024	0.5064	3653	2.51E-07	0.007253573
C	T	-0.129	0.029	0.2253	3653	9.87E-06	0.005332496
C	T	-0.1213	0.024	0.5591	3653	5.52E-07	0.006836299
G	C	0.1344	0.025	0.467	3653	1.35E-07	0.007576529
G	T	0.1779	0.039	0.1033	3653	6.02E-06	0.005589428
C	T	0.2608	0.055	0.0511	3653	2.26E-06	0.006102069
T	A	-0.5791	0.13	0.0086	3653	8.84E-06	0.005394531
G	A	0.1913	0.042	0.0884	3653	5.96E-06	0.005593957
T	C	0.1073	0.024	0.5761	3653	9.17E-06	0.005370531
G	A	-0.1516	0.034	0.1495	3653	8.01E-06	0.00544156
A	G	-1.553	0.346	0.0015	3653	7.28E-06	0.005494166
A	G	-0.1362	0.03	0.1993	3653	4.74E-06	0.005716329
T	C	0.3585	0.066	0.0354	3669	5.72E-08	0.007996673
T	C	0.1785	0.036	0.131	3669	8.39E-07	0.006590523
G	A	-0.1079	0.024	0.5181	3669	4.63E-06	0.005698616
T	G	-0.4144	0.09	0.0177	3669	4.03E-06	0.005777057
T	A	1.317	0.268	0.0019	3669	8.83E-07	0.006563213
T	C	-0.4344	0.091	0.017	3669	2.03E-06	0.006133573
A	G	1.16	0.238	0.0022	3669	1.10E-06	0.006449113
T	A	2.317	0.5	0.0005	3669	3.79E-06	0.005809508
A	G	2.135	0.363	0.001	3669	4.36E-09	0.009345368
A	G	-0.196	0.044	0.0849	3669	7.57E-06	0.005450411
A	G	0.1245	0.027	0.251	3669	5.51E-06	0.005611962
C	T	0.7585	0.163	0.0061	3669	3.16E-06	0.005903172
C	T	0.3137	0.07	0.0309	3669	8.86E-06	0.005368937
C	T	0.4161	0.083	0.0217	3669	5.80E-07	0.006785536
T	C	1.895	0.369	0.0014	3669	2.99E-07	0.007133024

G	A	-0.2812	0.06	0.0407	3669	2.98E-06	0.005931307
T	C	0.2303	0.045	0.0769	3669	2.67E-07	0.007189196
A	G	-0.183	0.038	0.1086	3669	2.05E-06	0.006126542
C	T	0.3064	0.068	0.03	3669	6.89E-06	0.005496768
C	T	0.3133	0.07	0.0337	3669	7.63E-06	0.00544407
G	A	0.1363	0.024	0.4999	3669	8.45E-09	0.009001708
A	C	-0.6305	0.14	0.0068	3669	6.83E-06	0.005505407
C	T	0.2493	0.056	0.0477	3669	9.32E-06	0.005340262
A	G	-0.1634	0.032	0.1623	3669	4.20E-07	0.006952426
C	T	0.5375	0.12	0.9898	3669	7.79E-06	0.005429487
T	G	0.1693	0.024	0.5685	3669	1.08E-12	0.013717371
T	C	1.901	0.395	0.0008	3669	1.55E-06	0.00627636
A	G	-0.1181	0.025	0.3684	3669	2.63E-06	0.005993052
A	T	0.1856	0.038	0.1066	3669	1.04E-06	0.006480225
C	G	0.2075	0.04	0.1047	3669	2.59E-07	0.007205757
C	A	0.2019	0.042	0.0984	3669	1.51E-06	0.006282684
G	A	0.3174	0.069	0.0331	3669	4.47E-06	0.005724266
T	G	0.5216	0.112	0.0114	3669	2.97E-06	0.005929186
A	G	0.8405	0.184	0.0044	3669	4.83E-06	0.005679483
G	A	-0.1171	0.024	0.5181	3669	1.51E-06	0.00628432
A	G	-0.4522	0.102	0.014	3669	9.95E-06	0.005307627
C	T	0.1849	0.042	0.0954	3669	9.03E-06	0.005358152
T	G	0.3025	0.068	0.0339	3669	9.10E-06	0.005353776
T	C	-0.42	0.094	0.017	3669	8.90E-06	0.00536624
T	G	-0.4331	0.093	0.0177	3669	3.14E-06	0.005905291
G	A	0.5437	0.117	0.0105	3669	3.43E-06	0.005861232
A	C	-0.1301	0.026	0.3104	3669	9.47E-07	0.006531263
A	G	0.1705	0.036	0.1423	3669	1.86E-06	0.006178358
G	A	0.1431	0.032	0.8198	3669	8.64E-06	0.005377352
G	T	-0.1413	0.028	0.2655	3669	3.44E-07	0.007062431
G	A	-0.4402	0.099	0.0151	3669	9.12E-06	0.005353337
T	C	-0.2127	0.045	0.0835	3669	2.10E-06	0.006111625
G	T	0.4121	0.083	0.0233	3669	7.62E-07	0.006643857
A	G	-0.1398	0.031	0.1907	3669	6.41E-06	0.005537265
T	C	0.1147	0.024	0.494	3669	2.61E-06	0.006001417
A	G	0.5353	0.11	0.0121	3669	1.12E-06	0.006447987
G	A	-1.044	0.194	0.0038	3669	7.51E-08	0.007863466
C	T	0.1453	0.032	0.18	3669	6.68E-06	0.005515692
G	A	0.1732	0.036	0.1312	3669	2.00E-06	0.006143068
G	T	-0.1288	0.025	0.4293	3669	1.69E-07	0.007434179
G	A	0.2272	0.026	0.319	3669	8.99E-18	0.019890326
A	G	0.1297	0.029	0.7602	3669	7.21E-06	0.005474639
T	G	0.1266	0.025	0.4302	3669	4.19E-07	0.006957456
T	C	-0.1185	0.025	0.4981	3669	1.90E-06	0.00616947
T	C	-0.1493	0.024	0.4842	3669	7.63E-10	0.01027577
G	C	0.1346	0.026	0.4667	3669	2.03E-07	0.007335401
T	C	0.5227	0.114	0.0121	3669	4.71E-06	0.005697262
G	A	0.1135	0.025	0.6307	3669	8.23E-06	0.005408532

T	G	-0.1173	0.025	0.3532	3669	3.70E-06	0.005824656
A	T	0.1356	0.025	0.3906	3669	1.04E-07	0.007678011
T	C	-0.3926	0.066	0.0356	3653	3.83E-09	0.009456759
T	C	-0.1314	0.029	0.2368	3653	4.29E-06	0.005769263
T	C	0.1119	0.024	0.4744	3653	4.76E-06	0.00571053
G	A	-1.189	0.221	0.0027	3653	8.29E-08	0.007840302
T	C	0.1787	0.039	0.8934	3653	5.30E-06	0.005656707
T	C	-0.3533	0.075	0.0272	3653	2.46E-06	0.006060354
A	T	0.1803	0.04	0.101	3653	8.19E-06	0.005430744
A	G	0.1148	0.025	0.3596	3653	6.86E-06	0.005526225
G	A	-0.1659	0.035	0.1404	3653	1.92E-06	0.006186417
A	G	0.1362	0.03	0.1904	3653	6.86E-06	0.005522503
T	C	-0.188	0.04	0.1046	3653	2.23E-06	0.006107481
G	A	-0.1204	0.026	0.5363	3653	3.05E-06	0.005949184
C	T	0.6198	0.135	0.0083	3653	4.32E-06	0.005762467
T	A	0.1561	0.032	0.1693	3653	1.30E-06	0.006392332
C	A	0.7623	0.166	0.0059	3653	4.23E-06	0.005774192
T	C	0.1177	0.025	0.3785	3653	1.83E-06	0.006207512
T	C	-0.8617	0.195	0.0037	3653	9.71E-06	0.005344364
T	A	-0.2003	0.043	0.0829	3653	3.96E-06	0.005810363
A	G	0.2022	0.033	0.1622	3653	6.55E-10	0.010389877
T	G	-0.2005	0.024	0.5686	3653	8.82E-17	0.018777943
A	G	-0.1246	0.028	0.2453	3653	6.55E-06	0.005548198
G	A	-0.1121	0.025	0.4531	3653	5.30E-06	0.005652357
T	C	-0.1667	0.036	0.131	3669	4.64E-06	0.005702561
T	C	-0.4163	0.066	0.0354	3669	3.35E-10	0.010698485
T	C	-0.1488	0.028	0.237	3669	1.77E-07	0.007400612
T	A	-1.294	0.269	0.0019	3669	1.51E-06	0.00628594
T	C	0.4242	0.092	0.017	3669	3.82E-06	0.005802456
T	G	0.4173	0.09	0.0177	3669	3.77E-06	0.005811281
A	G	-2.093	0.364	0.001	3669	9.93E-09	0.008911411
A	G	-1.199	0.239	0.0022	3669	5.33E-07	0.006835511
T	C	-0.521	0.107	0.0123	3669	1.25E-06	0.006384789
T	A	-2.321	0.502	0.0005	3669	3.99E-06	0.005783414
T	G	0.2362	0.052	0.0549	3669	6.61E-06	0.00552002
A	G	-0.1235	0.027	0.251	3669	7.10E-06	0.005478768
C	T	-0.7471	0.163	0.0061	3669	4.85E-06	0.005679315
T	C	-1.015	0.226	0.0027	3669	7.55E-06	0.005448274
T	C	0.5	0.112	0.011	3669	8.33E-06	0.005402609
C	T	-0.3941	0.083	0.0217	3669	2.44E-06	0.006039119
T	C	-1.805	0.371	0.0014	3669	1.17E-06	0.006420431
T	C	-0.2182	0.045	0.0769	3669	1.21E-06	0.006401284
C	T	-0.4344	0.09	0.0177	3669	1.30E-06	0.006362823
G	A	-0.1456	0.026	0.319	3669	1.60E-08	0.008658771
C	T	-0.5865	0.121	0.9898	3669	1.18E-06	0.006415332
T	G	-0.1909	0.024	0.5685	3669	1.21E-15	0.017304376
G	A	0.1822	0.032	0.1634	3669	1.74E-08	0.008624379
A	G	0.3791	0.08	0.0232	3669	2.47E-06	0.006030608

T	C	-1.834	0.397	0.0008	3669	3.91E-06	0.005794576
A	G	0.1175	0.025	0.3684	3669	3.24E-06	0.005885977
A	T	-0.1949	0.038	0.1066	3669	3.23E-07	0.00708912
T	C	0.2646	0.058	0.0414	3669	5.12E-06	0.005652145
T	C	-0.408	0.064	0.0354	3669	2.51E-10	0.010851187
T	C	0.5517	0.124	0.0093	3669	9.09E-06	0.005349162
C	A	0.7308	0.164	0.0053	3669	8.98E-06	0.005356894
T	A	-0.1993	0.043	0.0767	3669	4.00E-06	0.00577813
A	G	-1.131	0.235	0.0022	3669	1.58E-06	0.006257595
T	A	-2.292	0.491	0.0005	3669	3.09E-06	0.005915978
A	G	-1.682	0.358	0.001	3669	2.77E-06	0.005970489
G	A	0.105	0.024	0.4158	3669	8.17E-06	0.005411759
T	C	0.1124	0.024	0.5327	3669	1.97E-06	0.006144466
C	T	0.1676	0.036	0.1956	3669	3.76E-06	0.005808357
T	C	0.4364	0.096	0.0149	3669	5.47E-06	0.005618117
A	G	-0.2745	0.059	0.0412	3669	2.96E-06	0.005936945
A	G	0.328	0.072	0.0281	3669	6.18E-06	0.005552204
C	T	-0.4268	0.087	0.0177	3669	1.09E-06	0.006457504
T	C	-0.9938	0.218	0.0037	3669	5.50E-06	0.005616898
C	T	0.2685	0.06	0.9629	3669	6.59E-06	0.005519544
T	C	0.249	0.031	0.1639	3669	1.98E-15	0.017041984
T	G	-0.2323	0.023	0.5685	3669	1.51E-23	0.026914306
C	T	-0.9855	0.218	0.0037	3669	6.44E-06	0.005534063
A	G	0.2542	0.056	0.0448	3669	6.67E-06	0.005515862
A	G	0.1347	0.03	0.1976	3669	5.70E-06	0.00559749
A	G	-1.284	0.278	0.0016	3669	4.08E-06	0.005768243
A	G	0.1157	0.024	0.3684	3669	2.20E-06	0.006090963
T	C	0.1023	0.023	0.5067	3669	8.95E-06	0.005358422
A	C	-0.1923	0.041	0.0837	3669	2.61E-06	0.006000677
A	C	-0.1329	0.03	0.1877	3669	7.04E-06	0.005486457
A	T	-0.1857	0.037	0.1066	3669	6.25E-07	0.006742464
A	G	-0.3019	0.049	0.0686	3580	1.15E-09	0.01029591
A	G	0.3386	0.038	0.1218	3580	5.43E-19	0.021899358
G	T	-0.6984	0.027	0.2473	3580	2.05E-136	0.158551812
C	T	-0.118	0.026	0.3411	3580	6.22E-06	0.005694453
C	G	-0.1445	0.031	0.1989	3580	4.22E-06	0.005895661
C	T	-0.1378	0.029	0.2398	3580	1.68E-06	0.006380606
C	A	0.261	0.058	0.049	3580	6.01E-06	0.005708464
A	G	0.1342	0.03	0.8018	3580	9.35E-06	0.005474715
A	G	-0.1575	0.035	0.1412	3580	8.20E-06	0.005542424
A	G	-0.1305	0.027	0.6793	3580	1.32E-06	0.006516672
A	G	-0.1834	0.041	0.1052	3580	7.77E-06	0.00556621
G	A	-0.2256	0.05	0.0693	3580	5.38E-06	0.005763992
C	T	-0.2773	0.061	0.0464	3580	6.30E-06	0.005681716
A	G	0.301	0.067	0.0342	3580	6.91E-06	0.005631118
T	A	-0.2666	0.033	0.1786	3580	1.99E-15	0.017475425
G	A	-0.3144	0.068	0.0349	3580	4.09E-06	0.00591157
A	C	0.117	0.025	0.6053	3580	3.24E-06	0.006037503

A	G	0.2213	0.049	0.069	3580	6.37E-06	0.005674473
G	A	0.2071	0.046	0.0739	3580	7.87E-06	0.005562474
G	A	-0.1371	0.025	0.5004	3596	4.97E-08	0.008234983
G	T	-0.6848	0.028	0.2474	3596	1.30E-124	0.145091224
G	C	0.3669	0.076	0.0261	3596	1.43E-06	0.006444417
A	G	0.3328	0.039	0.1218	3596	1.13E-17	0.020181081
C	T	0.1389	0.026	0.6023	3596	1.32E-07	0.007708561
T	C	0.8583	0.178	0.0046	3596	1.48E-06	0.006424207
T	C	-0.1619	0.034	0.1623	3596	2.49E-06	0.006146794
G	A	-0.1222	0.026	0.642	3596	2.77E-06	0.006096106
T	G	0.1416	0.03	0.2045	3596	3.26E-06	0.006008965
T	C	-0.3236	0.072	0.0327	3596	7.91E-06	0.005535409
A	G	-0.14	0.028	0.6792	3596	3.87E-07	0.007140225
T	G	-0.3593	0.079	0.0263	3596	6.33E-06	0.005652318
A	T	-0.6443	0.133	0.9914	3596	1.23E-06	0.006522701
T	A	0.1502	0.033	0.8231	3596	6.95E-06	0.005609003
T	C	-0.5233	0.112	0.0128	3596	3.28E-06	0.006002162
A	T	-0.4439	0.089	0.0209	3596	7.00E-07	0.006823027
A	C	-1.166	0.258	0.0024	3596	6.35E-06	0.005647786
T	G	0.2936	0.062	0.0449	3596	2.40E-06	0.006165728
A	G	0.2832	0.063	0.0414	3596	8.15E-06	0.005519776
A	G	-0.2525	0.03	0.2162	3596	8.74E-17	0.019081382
A	G	-0.1367	0.03	0.7729	3596	4.26E-06	0.005864556
T	G	0.1457	0.025	0.5698	3596	6.72E-09	0.009312663
C	T	-0.112	0.025	0.4573	3596	8.44E-06	0.005502079
T	C	-0.5897	0.123	0.0096	3596	1.80E-06	0.006320664
G	C	0.3452	0.075	0.0261	3596	3.73E-06	0.005935046
C	T	-0.1254	0.026	0.3411	3596	1.59E-06	0.006383313
A	G	0.3424	0.038	0.1218	3596	2.53E-19	0.022216247
G	A	-0.7124	0.027	0.2474	3596	9.98E-142	0.163614761
A	G	-0.2746	0.05	0.0685	3596	3.27E-08	0.008461611
C	A	0.2654	0.058	0.0488	3596	4.41E-06	0.005847002
T	C	0.7938	0.175	0.0046	3596	5.61E-06	0.005715114
A	G	-0.2009	0.044	0.0829	3596	6.08E-06	0.005673901
G	A	-0.1168	0.026	0.642	3596	4.84E-06	0.005791382
A	G	-0.1461	0.027	0.6792	3596	6.50E-08	0.008088533
T	G	-0.3596	0.078	0.0263	3596	4.04E-06	0.005890862
A	T	-0.586	0.13	0.9914	3596	6.84E-06	0.005610191
T	A	0.1447	0.033	0.8231	3596	9.97E-06	0.005412517
A	T	-0.4035	0.088	0.0209	3596	4.25E-06	0.005864159
G	C	-0.1148	0.025	0.5535	3596	6.40E-06	0.005648545
A	G	0.3054	0.066	0.0367	3596	3.82E-06	0.005920837
T	G	0.2779	0.061	0.0449	3596	5.32E-06	0.00574599
C	G	-0.5148	0.116	0.0122	3596	8.76E-06	0.005484704
T	C	-0.6247	0.137	0.0079	3596	5.20E-06	0.005757168
A	G	-0.1228	0.027	0.2771	3596	7.39E-06	0.005566768
T	A	-0.2729	0.033	0.1783	3596	4.22E-16	0.018226602
C	T	-0.1225	0.026	0.6379	3596	2.35E-06	0.006182436

A	G	0.1225	0.025	0.4053	3596	1.61E-06	0.006381642
A	C	0.1116	0.025	0.605	3596	8.69E-06	0.005489121
A	G	0.6357	0.135	0.0074	3596	2.60E-06	0.006128405
G	A	-0.1188	0.026	0.3407	3596	4.50E-06	0.005830166
A	G	0.3966	0.08	0.9759	3653	8.35E-07	0.006625172
T	C	-0.3362	0.067	0.0357	3653	4.61E-07	0.006933729
C	T	-0.2653	0.053	0.0594	3653	5.34E-07	0.006861237
C	T	0.3326	0.072	0.0319	3653	3.62E-06	0.005857688
G	A	-0.3358	0.076	0.0279	3653	9.11E-06	0.00537474
G	C	-0.1133	0.025	0.4592	3653	7.59E-06	0.005468579
T	C	-0.6398	0.141	0.0081	3653	5.86E-06	0.005604793
T	C	0.1222	0.025	0.4094	3653	1.05E-06	0.006508368
C	T	-0.1142	0.025	0.4454	3653	4.68E-06	0.005725195
T	C	-0.3375	0.074	0.029	3653	4.87E-06	0.005701752
T	G	-0.1104	0.025	0.5125	3653	9.97E-06	0.00532272
G	T	0.1403	0.03	0.785	3653	2.89E-06	0.005971328
T	C	0.1203	0.026	0.3804	3653	4.20E-06	0.005782054
A	G	-0.7378	0.165	0.0057	3653	7.52E-06	0.005476598
C	T	0.1104	0.025	0.4511	3653	7.81E-06	0.005461053
T	C	-0.1888	0.039	0.1173	3653	1.56E-06	0.006297277
A	C	0.1811	0.04	0.1129	3653	7.08E-06	0.005508593
C	T	-0.251	0.055	0.0541	3653	5.66E-06	0.005626159
A	G	-0.1644	0.037	0.1377	3653	7.16E-06	0.00549885
T	C	-0.1161	0.026	0.4885	3653	9.35E-06	0.005367033
A	G	0.1262	0.027	0.3221	3653	2.24E-06	0.006114897
A	G	-1.782	0.393	0.001	3653	6.05E-06	0.005588356
C	G	0.2719	0.058	0.0483	3653	3.22E-06	0.005917051
A	G	-0.1259	0.026	0.3408	3653	1.83E-06	0.006215306
T	C	-0.8628	0.184	0.0048	3653	2.75E-06	0.006002574
T	C	-0.9599	0.214	0.0034	3669	7.74E-06	0.005438655
A	G	0.2941	0.064	0.039	3669	4.52E-06	0.005717225
C	T	0.124	0.025	0.4869	3669	6.00E-07	0.006767731
T	C	-0.3169	0.066	0.0356	3669	1.93E-06	0.006156916
G	A	0.3798	0.085	0.0221	3669	8.83E-06	0.005368062
T	C	-0.1155	0.026	0.6167	3669	9.02E-06	0.00536213
G	T	-0.2011	0.045	0.0816	3669	9.45E-06	0.005333238
T	C	-0.3807	0.081	0.0232	3669	2.94E-06	0.005942311
C	T	1.127	0.251	0.0022	3669	7.36E-06	0.005460454
G	A	-0.3658	0.08	0.0249	3669	5.00E-06	0.005663384
T	C	-0.1646	0.036	0.8639	3669	6.10E-06	0.005557542
G	A	0.5425	0.122	0.0109	3669	9.64E-06	0.005325616
T	C	-0.1823	0.04	0.1141	3669	4.95E-06	0.005671512
G	C	0.1563	0.032	0.1924	3669	1.12E-06	0.006444325
T	C	0.5351	0.12	0.0099	3669	9.00E-06	0.005354728
A	G	0.4302	0.096	0.0172	3669	7.66E-06	0.005442396
G	A	-0.2032	0.045	0.0829	3669	6.20E-06	0.005551233
C	A	0.1711	0.036	0.8609	3669	2.05E-06	0.006132554
C	T	0.1239	0.025	0.4516	3669	4.72E-07	0.006894256

C	T	0.1527	0.034	0.1555	3669	8.36E-06	0.005400976
T	C	-0.1175	0.025	0.3993	3669	3.44E-06	0.005853616
T	C	-0.2627	0.057	0.0496	3669	4.18E-06	0.005755933
T	C	0.6232	0.14	0.0084	3669	9.15E-06	0.005348877
A	G	-0.1211	0.026	0.6727	3669	3.62E-06	0.005828918
A	G	0.1512	0.027	0.3227	3669	1.25E-08	0.008794835
T	C	-0.1218	0.027	0.2978	3669	7.87E-06	0.005431558
T	C	-0.7959	0.173	0.0052	3440	4.30E-06	0.006122117
A	G	-0.8439	0.183	0.0044	3440	4.02E-06	0.006163979
T	C	0.115	0.026	0.433	3440	7.83E-06	0.00579144
G	A	-0.1507	0.028	0.2719	3440	7.75E-08	0.008350456
T	G	-0.117	0.025	0.524	3440	3.58E-06	0.006222384
T	C	0.1749	0.036	0.1456	3440	1.66E-06	0.006652207
C	T	-0.1344	0.028	0.2746	3440	1.83E-06	0.006596826
G	A	-0.293	0.061	0.0448	3440	1.47E-06	0.006721117
C	T	-0.1978	0.041	0.1839	3440	1.44E-06	0.00672696
A	G	0.2703	0.06	0.0464	3440	6.33E-06	0.005910073
T	C	-1.711	0.355	0.0012	3440	1.53E-06	0.006696274
A	G	-0.4428	0.099	0.0163	3440	8.19E-06	0.005767956
T	C	0.249	0.051	0.0667	3440	1.24E-06	0.006809982
T	C	0.171	0.038	0.1186	3440	7.19E-06	0.005839949
A	T	0.2423	0.053	0.0606	3440	5.93E-06	0.005944989
C	T	0.1939	0.043	0.0922	3440	5.91E-06	0.005944775
C	T	-0.151	0.03	0.7741	3440	4.38E-07	0.00739377
T	C	-0.2774	0.061	0.0432	3440	5.21E-06	0.006014883
A	G	-0.1292	0.029	0.2651	3440	7.44E-06	0.005820337
A	C	-0.1613	0.035	0.1461	3440	5.14E-06	0.006026204
G	T	0.3233	0.072	0.0312	3440	6.84E-06	0.005864254
G	A	-0.2463	0.054	0.0596	3440	6.16E-06	0.00592369
T	C	0.2422	0.054	0.0587	3440	8.40E-06	0.005752342
G	A	0.4529	0.102	0.015	3440	8.96E-06	0.005720829
C	T	-0.2435	0.037	0.1343	3440	6.13E-11	0.012367644
G	A	-0.1505	0.033	0.1759	3440	4.12E-06	0.006142405
T	C	-0.2028	0.043	0.0985	3440	1.95E-06	0.006563306
G	A	-0.1144	0.025	0.4555	3440	5.77E-06	0.005955229
G	A	0.7811	0.176	0.0056	3408	9.71E-06	0.005726836
G	T	0.1657	0.036	0.1476	3408	4.44E-06	0.006160996
T	A	-0.9323	0.208	0.0035	3408	7.94E-06	0.005838126
C	T	0.3012	0.067	0.0383	3408	6.67E-06	0.005935566
A	G	0.1921	0.032	0.1991	3408	1.45E-09	0.010687273
G	A	-0.279	0.061	0.0462	3408	4.60E-06	0.00614283
G	A	0.3202	0.07	0.0335	3408	5.69E-06	0.006024993
C	T	-0.5102	0.115	0.0134	3408	8.83E-06	0.005782207
T	G	-0.1189	0.025	0.5246	3408	2.68E-06	0.006444048
T	C	0.1787	0.037	0.1451	3408	1.18E-06	0.006908862
G	A	-0.2241	0.047	0.0803	3408	2.11E-06	0.006579389
A	G	0.3453	0.077	0.0292	3408	8.35E-06	0.005809061
A	G	0.3063	0.067	0.0381	3408	4.60E-06	0.006142513

G	A	-0.201	0.038	0.1797	3408	1.10E-07	0.00823714
A	G	0.2698	0.06	0.0465	3408	7.25E-06	0.005890298
T	C	-1.712	0.354	0.0012	3408	1.35E-06	0.006827512
A	T	0.1241	0.027	0.3628	3408	3.41E-06	0.00631317
G	A	0.3832	0.074	0.0314	3408	2.55E-07	0.007765296
T	C	0.5394	0.12	0.0109	3408	6.85E-06	0.005923167
C	A	-0.1141	0.026	0.4062	3408	9.38E-06	0.005741549
C	T	-0.125	0.026	0.4156	3408	1.01E-06	0.006990568
A	G	-0.2717	0.06	0.0456	3408	6.40E-06	0.005959234
A	G	-0.1252	0.025	0.467	3408	9.26E-07	0.007040165
C	T	-0.1518	0.03	0.2656	3408	3.35E-07	0.007617201
A	T	0.2162	0.048	0.0747	3408	8.27E-06	0.005813674
A	C	-0.1585	0.036	0.1455	3408	8.51E-06	0.005799011
A	T	0.1199	0.027	0.5405	3408	7.16E-06	0.005899953
G	T	0.1893	0.041	0.1084	3408	4.31E-06	0.006180208
A	G	0.1361	0.031	0.2211	3408	8.71E-06	0.005786151
C	A	-0.1432	0.032	0.1963	3408	6.31E-06	0.005967128
A	G	0.3004	0.067	0.0361	3408	8.31E-06	0.005813891
G	C	-0.1497	0.032	0.2017	3408	2.30E-06	0.006533913
G	A	0.1857	0.027	0.6743	3408	5.64E-12	0.013831313
C	T	-0.1592	0.032	0.6386	3408	5.55E-07	0.007323301
C	T	0.1977	0.044	0.0711	3440	6.97E-06	0.005855711
A	G	0.3131	0.067	0.0294	3440	3.61E-06	0.006221226
A	G	0.1109	0.023	0.3994	3440	1.51E-06	0.006713109
A	G	0.1659	0.029	0.1927	3440	8.92E-09	0.009567045
T	C	0.2228	0.05	0.0574	3440	7.84E-06	0.005789492
G	A	-0.2734	0.059	0.0363	3440	4.39E-06	0.006110447
G	A	0.1142	0.026	0.3344	3440	8.81E-06	0.005724888
A	G	-0.2413	0.053	0.0499	3440	4.87E-06	0.006055255
A	G	1.008	0.228	0.0025	3440	9.81E-06	0.005669553
G	A	0.1841	0.039	0.0911	3440	2.54E-06	0.006413102
C	T	0.1872	0.041	0.0846	3440	4.35E-06	0.006118234
C	T	0.4675	0.099	0.0128	3440	2.66E-06	0.00638795
A	G	0.1531	0.031	0.2147	3440	7.08E-07	0.007122335
G	A	0.2427	0.054	0.061	3440	6.86E-06	0.005863702
A	G	0.1352	0.029	0.1836	3440	3.99E-06	0.006164022
C	T	1.736	0.358	0.0009	3440	1.31E-06	0.0067854
T	C	0.2476	0.056	0.044	3440	9.92E-06	0.005662805
G	T	-0.2002	0.044	0.0679	3440	6.07E-06	0.00593114
C	T	0.1649	0.037	0.1036	3440	7.50E-06	0.005815657
A	C	-0.1806	0.035	0.1147	3440	3.37E-07	0.007543056
T	C	0.2487	0.049	0.0592	3440	3.83E-07	0.007469211
G	A	0.9109	0.19	0.0047	3440	1.77E-06	0.006616409
C	G	0.1431	0.029	0.2028	3440	7.05E-07	0.007130658
G	A	-0.1115	0.025	0.2718	3440	9.92E-06	0.005663285
C	A	-0.204	0.044	0.0693	3440	4.65E-06	0.006080215
A	G	0.8439	0.183	0.0044	3440	4.02E-06	0.006163979
T	C	0.7959	0.173	0.0052	3440	4.30E-06	0.006122117

T	C	-0.115	0.026	0.433	3440	7.83E-06	0.00579144
G	A	0.1507	0.028	0.2719	3440	7.75E-08	0.008350456
T	G	0.117	0.025	0.524	3440	3.58E-06	0.006222384
C	T	0.1344	0.028	0.2746	3440	1.83E-06	0.006596826
T	C	-0.1749	0.036	0.1456	3440	1.66E-06	0.006652207
G	A	0.293	0.061	0.0448	3440	1.47E-06	0.006721117
C	T	0.1978	0.041	0.1839	3440	1.44E-06	0.00672696
A	G	-0.2703	0.06	0.0464	3440	6.33E-06	0.005910073
T	C	1.711	0.355	0.0012	3440	1.53E-06	0.006696274
T	C	-0.249	0.051	0.0667	3440	1.24E-06	0.006809982
A	G	0.4428	0.099	0.0163	3440	8.19E-06	0.005767956
C	T	-0.1939	0.043	0.0922	3440	5.91E-06	0.005944775
A	T	-0.2423	0.053	0.0606	3440	5.93E-06	0.005944989
T	C	-0.171	0.038	0.1186	3440	7.19E-06	0.005839949
C	T	0.151	0.03	0.7741	3440	4.38E-07	0.00739377
T	C	0.2774	0.061	0.0432	3440	5.21E-06	0.006014883
A	G	0.1292	0.029	0.2651	3440	7.44E-06	0.005820337
A	C	0.1613	0.035	0.1461	3440	5.14E-06	0.006026204
G	T	-0.3233	0.072	0.0312	3440	6.84E-06	0.005864254
T	C	-0.2422	0.054	0.0587	3440	8.40E-06	0.005752342
G	A	-0.4529	0.102	0.015	3440	8.96E-06	0.005720829
G	A	0.2463	0.054	0.0596	3440	6.16E-06	0.00592369
T	C	0.2028	0.043	0.0985	3440	1.95E-06	0.006563306
C	T	0.2435	0.037	0.1343	3440	6.13E-11	0.012367644
G	A	0.1505	0.033	0.1759	3440	4.12E-06	0.006142405
G	A	0.1144	0.025	0.4555	3440	5.77E-06	0.005955229
C	T	0.1447	0.028	0.191	3408	1.62E-07	0.008023804
G	A	0.2819	0.063	0.0293	3408	8.89E-06	0.005774906
T	C	-0.1677	0.037	0.0979	3408	6.32E-06	0.005962872
G	T	0.809	0.176	0.0038	3408	4.35E-06	0.006175462
T	C	0.1053	0.022	0.3718	3408	2.87E-06	0.006402675
G	T	-0.5354	0.114	0.0089	3408	2.54E-06	0.006475585
G	C	0.2735	0.052	0.0467	3408	1.42E-07	0.008095061
G	A	0.1469	0.032	0.132	3408	6.23E-06	0.00597372
G	A	-0.09693	0.022	0.4303	3408	8.73E-06	0.00578337
T	C	0.2194	0.047	0.0591	3408	2.49E-06	0.006487158
T	C	0.1884	0.041	0.0738	3408	3.84E-06	0.006245098
A	G	0.9508	0.185	0.0034	3408	2.88E-07	0.007699247
G	A	0.1713	0.037	0.1272	3440	4.73E-06	0.006074303
A	T	-0.625	0.135	0.0087	3440	3.49E-06	0.006237915
A	G	-0.5776	0.122	0.0102	3440	2.38E-06	0.006452709
G	A	0.2137	0.046	0.0778	3440	2.84E-06	0.006352212
T	G	0.2206	0.048	0.0727	3440	3.91E-06	0.006176508
G	A	-0.1429	0.029	0.2307	3440	8.36E-07	0.007028227
G	A	0.118	0.026	0.3177	3440	6.60E-06	0.005884358
A	G	-0.1194	0.025	0.6099	3440	1.60E-06	0.006671751
T	A	-0.3512	0.077	0.0297	3440	4.68E-06	0.006075191
C	A	-0.1588	0.035	0.1426	3440	5.51E-06	0.005985951

G	A	0.1112	0.025	0.5305	3440	6.37E-06	0.00590485
G	A	0.6746	0.086	0.0211	3440	7.61E-15	0.017417125
G	T	-0.1294	0.025	0.5113	3440	1.60E-07	0.007953544
C	T	-1.767	0.396	0.0009	3440	8.29E-06	0.005763315
T	C	-0.291	0.063	0.0417	3440	3.61E-06	0.006220766
A	T	-0.9175	0.019	0.4863	3440	1.00E-200	0.401984553
A	G	-0.3241	0.073	0.0288	3440	8.53E-06	0.005745763
C	T	0.2592	0.025	0.3836	3440	4.75E-24	0.029293108
A	G	0.4814	0.086	0.0212	3440	2.29E-08	0.009041069
C	A	-0.3969	0.024	0.5141	3440	3.17E-60	0.074913869
T	C	-0.2419	0.025	0.4068	3440	1.06E-21	0.026290289
C	T	-0.6106	0.13	0.011	3440	2.69E-06	0.006381993
C	T	0.6181	0.057	0.0464	3440	9.27E-27	0.032785557
T	C	-0.191	0.039	0.1042	3440	1.05E-06	0.006902962
C	T	-0.1396	0.031	0.7988	3440	5.16E-06	0.006025551
C	G	-2.463	0.511	0.0004	3440	1.47E-06	0.006716022
T	C	0.1314	0.029	0.2365	3440	5.49E-06	0.005990049
T	C	0.1439	0.029	0.2094	3440	8.71E-07	0.007010389
C	T	-2.978	0.635	0.0003	3440	2.89E-06	0.006345008
A	G	-2.568	0.537	0.0004	3440	1.82E-06	0.006596651
G	A	0.1288	0.028	0.2262	3440	6.00E-06	0.005939415
A	G	-0.5461	0.122	0.0099	3440	7.18E-06	0.005838353
G	C	-0.4404	0.092	0.016	3440	1.68E-06	0.006644479
C	T	0.4688	0.106	0.0121	3440	9.41E-06	0.005685785
A	G	0.2216	0.05	0.0658	3440	8.67E-06	0.005736815
T	C	-0.1215	0.026	0.2904	3440	4.32E-06	0.006119563
A	G	-0.6221	0.122	0.0102	3440	3.73E-07	0.00747756
G	A	-0.4495	0.1	0.0145	3440	7.56E-06	0.005816105
A	G	-0.1147	0.025	0.6099	3440	4.11E-06	0.006155087
C	T	-0.7852	0.175	0.0045	3440	7.01E-06	0.005851437
G	C	0.1733	0.039	0.1032	3440	9.00E-06	0.005713033
C	A	-0.1726	0.035	0.1426	3440	7.85E-07	0.007071915
T	A	-0.4543	0.094	0.0174	3440	1.38E-06	0.00675279
C	A	-0.3997	0.024	0.5141	3440	4.54E-61	0.0759531
C	T	0.6597	0.057	0.0464	3440	2.62E-30	0.03734073
T	C	-0.2881	0.063	0.0417	3440	4.52E-06	0.006096216
T	C	-0.3492	0.074	0.0294	3440	2.50E-06	0.006423052
G	A	0.7452	0.086	0.0211	3440	8.40E-18	0.021244378
C	T	0.2506	0.025	0.3836	3440	1.46E-22	0.027392061
G	T	-0.1212	0.025	0.5113	3440	9.26E-07	0.006973033
A	G	0.4649	0.086	0.0212	3440	6.83E-08	0.00842928
T	C	-0.2459	0.025	0.4068	3440	2.18E-22	0.027164195
A	T	-0.9258	0.019	0.4863	3440	1.00E-200	0.409114338
A	G	-0.3868	0.086	0.0201	3440	6.72E-06	0.005876016
T	C	-0.1824	0.039	0.1042	3440	3.17E-06	0.006292751
A	G	-0.465	0.103	0.0138	3440	7.14E-06	0.005844679
C	T	0.1695	0.034	0.1487	3440	5.63E-07	0.007248934
T	C	0.1408	0.029	0.2094	3440	1.50E-06	0.006709038

C	A	-2.79	0.586	0.0003	3440	2.01E-06	0.006546397
T	C	0.1562	0.035	0.1643	3408	9.37E-06	0.005748048
C	T	1.095	0.247	0.0028	3408	9.40E-06	0.005747606
C	T	-0.1208	0.026	0.4134	3408	3.18E-06	0.006347529
T	A	1.022	0.204	0.0034	3408	5.82E-07	0.007310637
C	T	2.034	0.446	0.0007	3408	5.37E-06	0.006057727
T	G	0.2313	0.05	0.0712	3408	4.73E-06	0.006125151
A	G	0.2554	0.056	0.0524	3408	6.27E-06	0.005968437
T	C	0.3142	0.07	0.0342	3408	7.26E-06	0.005888718
C	T	-0.3243	0.064	0.0414	3408	4.69E-07	0.007422457
A	G	0.2207	0.046	0.0848	3408	2.15E-06	0.006569367
G	A	-0.3193	0.071	0.0392	3408	7.73E-06	0.005855101
C	T	0.5331	0.117	0.0123	3408	5.28E-06	0.006065221
C	A	-0.4118	0.025	0.5139	3408	3.85E-59	0.074293009
C	T	0.6311	0.06	0.0459	3408	2.66E-25	0.031200444
A	T	-0.9198	0.021	0.4862	3408	1.00E-200	0.370897509
T	A	-0.275	0.049	0.0776	3408	2.12E-08	0.009168649
T	C	-0.199	0.043	0.1021	3408	3.36E-06	0.006320969
C	T	0.2544	0.027	0.3841	3408	3.12E-21	0.025909616
C	T	-0.6196	0.136	0.011	3408	5.74E-06	0.006018287
T	A	0.2664	0.059	0.0491	3408	7.02E-06	0.005908788
T	C	-0.1207	0.026	0.3498	3408	4.96E-06	0.006096092
G	A	0.7936	0.09	0.021	3408	2.46E-18	0.022142125
T	C	-0.2402	0.026	0.4073	3408	1.20E-19	0.023855513
G	A	-0.1393	0.029	0.7525	3408	2.08E-06	0.006588651
T	C	-1.428	0.307	0.0021	3408	3.49E-06	0.006292276
A	G	0.156	0.031	0.2139	3408	7.08E-07	0.007195002
G	A	0.5315	0.12	0.0123	3408	9.88E-06	0.005713896
C	T	0.1983	0.043	0.1034	3408	4.99E-06	0.006096928
A	G	0.1441	0.029	0.2515	3408	7.48E-07	0.00716333
C	T	-0.1181	0.026	0.4325	3408	5.48E-06	0.006045425
T	C	-1.173	0.247	0.0025	3440	2.11E-06	0.006518612
G	A	-0.1604	0.028	0.2827	3440	1.54E-08	0.009258587
G	C	0.9405	0.206	0.0039	3440	4.96E-06	0.006046153
C	T	0.2263	0.046	0.0852	3440	9.51E-07	0.00696228
A	G	-0.9685	0.216	0.0033	3440	7.86E-06	0.005789017
T	G	-0.2605	0.059	0.0531	3440	9.14E-06	0.005707939
T	C	0.1339	0.029	0.2692	3440	4.68E-06	0.006079755
A	G	-0.1312	0.027	0.3587	3440	8.81E-07	0.007006695
G	A	0.3121	0.057	0.0541	3440	6.06E-08	0.008497465
T	C	0.1229	0.026	0.4138	3440	3.07E-06	0.006312682
T	A	-0.246	0.053	0.0626	3440	3.60E-06	0.0062237
C	T	0.2818	0.06	0.0499	3440	2.61E-06	0.006399072
G	A	0.4819	0.079	0.0273	3440	1.47E-09	0.010578875
G	T	0.3132	0.066	0.961	3440	2.45E-06	0.006437678
G	A	0.1185	0.026	0.6156	3440	6.61E-06	0.005884717
C	G	-0.1262	0.026	0.3923	3440	1.89E-06	0.006579163
T	C	0.2387	0.053	0.0616	3440	6.06E-06	0.00593071

T	C	0.1183	0.026	0.5308	3440	4.40E-06	0.006112325
G	A	0.176	0.039	0.1294	3440	5.30E-06	0.006004154
A	G	0.4705	0.099	0.0161	3440	2.25E-06	0.006481319
C	T	-0.2225	0.038	0.1343	3440	3.91E-09	0.010024057
G	A	0.2598	0.058	0.0501	3440	8.52E-06	0.005745494
A	G	0.3968	0.084	0.0234	3440	2.19E-06	0.006495539
G	C	0.1285	0.029	0.7384	3440	8.38E-06	0.005757801
C	T	-0.1247	0.027	0.3499	3440	3.01E-06	0.006315055
A	G	-0.1601	0.032	0.1872	3440	4.13E-07	0.007429963
G	A	0.1123	0.025	0.4638	3440	7.27E-06	0.00583151
C	T	0.2113	0.045	0.0852	3440	2.24E-06	0.006482557
G	A	0.1625	0.036	0.1472	3440	6.99E-06	0.00585576
T	C	-1.192	0.245	0.0023	3440	1.22E-06	0.0068286
T	C	-0.1373	0.027	0.293	3440	3.98E-07	0.007450137
A	G	-0.126	0.026	0.3587	3440	1.20E-06	0.006832906
G	A	-0.1841	0.038	0.1794	3440	9.75E-07	0.006950156
T	C	0.3425	0.073	0.0317	3440	2.62E-06	0.006400131
C	T	-4.194	0.927	0.0001	3440	6.20E-06	0.005920182
T	A	0.1559	0.034	0.8317	3440	3.99E-06	0.006168173
A	T	0.2355	0.053	0.0618	3440	7.53E-06	0.005813094
G	T	-1.993	0.444	0.0009	3440	7.31E-06	0.005828314
C	T	-0.2283	0.036	0.1343	3440	4.32E-10	0.011269329
G	C	-4.194	0.927	0.0001	3440	6.19E-06	0.005920182
G	A	0.2299	0.052	0.065	3440	9.03E-06	0.005711038
T	C	-1.111	0.248	0.0025	3408	7.46E-06	0.005873123
G	A	-0.1568	0.028	0.2826	3408	3.59E-08	0.008865201
C	T	0.2114	0.046	0.0855	3408	5.00E-06	0.00609564
G	A	0.9354	0.203	0.004	3408	4.03E-06	0.006215961
A	G	0.5682	0.128	0.0103	3408	8.96E-06	0.005775711
T	C	-0.1164	0.026	0.3804	3408	7.19E-06	0.005896224
C	T	-0.1263	0.027	0.3703	3408	2.22E-06	0.006547265
C	T	-0.1732	0.037	0.1389	3408	3.82E-06	0.006246937
C	T	-0.1446	0.031	0.2961	3408	4.28E-06	0.006184201
G	A	0.4038	0.075	0.0314	3408	8.62E-08	0.008376286
G	A	0.3929	0.077	0.029	3408	3.87E-07	0.007529399
T	A	-0.243	0.053	0.0625	3408	5.36E-06	0.006055249
A	G	0.5414	0.118	0.0117	3408	4.62E-06	0.006139013
G	T	0.3043	0.066	0.9607	3408	4.82E-06	0.006115756
G	A	-0.1242	0.026	0.636	3408	2.73E-06	0.006437901
A	T	0.5035	0.111	0.0135	3408	6.41E-06	0.005958453
C	T	-0.6397	0.137	0.0091	3408	2.94E-06	0.006393902
A	G	0.2001	0.044	0.0942	3408	5.06E-06	0.006089644
T	C	1.692	0.347	0.0013	3408	1.14E-06	0.006928238
T	C	-0.128	0.029	0.2931	3408	8.57E-06	0.005794655
T	G	-0.1279	0.027	0.3787	3408	1.58E-06	0.006743205
G	A	-0.2226	0.048	0.0753	3408	3.53E-06	0.006291816
G	A	0.1712	0.036	0.8499	3408	2.12E-06	0.006574048
A	G	0.4723	0.1	0.0161	3408	2.35E-06	0.006518371

A	G	0.1662	0.026	0.5219	3408	1.05E-10	0.012169428
A	G	0.3958	0.084	0.0235	3408	2.43E-06	0.006500166
G	A	0.2783	0.058	0.0503	3408	1.99E-06	0.006612622
G	A	-0.8597	0.19	0.0044	3408	6.14E-06	0.00598405
A	G	-0.4098	0.077	0.9749	3440	9.59E-08	0.008240763
A	C	0.6208	0.122	0.0094	3440	3.99E-07	0.007446573
C	T	1.164	0.181	0.0042	3440	1.30E-10	0.011944676
A	G	2.523	0.239	0.0032	3440	9.14E-26	0.031506208
T	C	0.349	0.076	0.0253	3440	4.58E-06	0.006086349
A	G	0.1379	0.031	0.1676	3440	8.34E-06	0.005756334
A	G	1.303	0.174	0.0044	3440	1.02E-13	0.01596786
G	A	0.1724	0.026	0.2977	3440	2.27E-11	0.012922282
A	G	0.3541	0.077	0.023	3440	4.25E-06	0.006130684
T	C	-0.1252	0.027	0.235	3440	4.50E-06	0.006094577
A	G	0.1726	0.027	0.243	3440	2.21E-10	0.011637509
G	C	0.1951	0.023	0.502	3440	8.41E-17	0.019957965
T	G	-0.267	0.045	0.0731	3440	3.34E-09	0.010116822
C	T	0.1265	0.027	0.7549	3440	3.46E-06	0.006243748
T	C	3.379	0.624	0.0003	3440	6.61E-08	0.008449373
A	G	-0.4031	0.087	0.019	3440	3.98E-06	0.006166658
C	G	3.727	0.768	0.0003	3440	1.26E-06	0.006802985
T	C	0.1679	0.037	0.1099	3440	6.91E-06	0.005858768
A	G	1.018	0.217	0.0029	3440	2.80E-06	0.006362765
C	T	0.1089	0.024	0.5573	3440	4.11E-06	0.006146498
A	C	3.721	0.819	0.0001	3440	5.65E-06	0.005972032
C	T	-0.1595	0.034	0.2231	3440	2.17E-06	0.006508018
T	C	0.1843	0.035	0.1856	3440	1.45E-07	0.008009556
G	T	0.2388	0.048	0.1129	3440	5.90E-07	0.00722702
A	C	3.37	0.624	0.0003	3440	7.14E-08	0.008404801
G	T	3.04	0.625	0.0003	3440	1.22E-06	0.006821818
G	A	2.741	0.537	0.0004	3440	3.46E-07	0.007525164
T	C	0.1065	0.024	0.3737	3440	7.17E-06	0.005840723
C	T	0.1895	0.041	0.0875	3440	4.20E-06	0.006135935
C	T	2.215	0.499	0.0006	3440	9.28E-06	0.005697451
C	T	0.2338	0.052	0.0544	3440	8.03E-06	0.005780186
C	T	1.681	0.374	0.0009	3440	7.41E-06	0.005825963
A	G	0.1422	0.031	0.1741	3440	3.57E-06	0.006230402
T	C	0.1246	0.027	0.2458	3440	3.71E-06	0.006202886
G	A	-0.1308	0.027	0.768	3440	1.44E-06	0.006731405
T	C	3.343	0.737	0.0003	3440	5.89E-06	0.005948725
T	C	0.6783	0.15	0.0057	3440	6.25E-06	0.005917041
A	G	0.2933	0.059	0.0407	3440	5.87E-07	0.007227234
T	C	5.111	1.119	0.0001	3440	5.11E-06	0.006027918
A	G	2.584	0.521	0.0004	3440	7.39E-07	0.007099972
C	T	1.191	0.192	0.0042	3440	5.92E-10	0.011084776
A	C	0.6883	0.13	0.0094	3440	1.18E-07	0.008120376
A	G	1.307	0.186	0.0044	3440	2.37E-12	0.014180731
A	G	2.559	0.255	0.0032	3440	1.96E-23	0.02852955

T	C	0.1605	0.034	0.1565	3440	1.95E-06	0.006562149
C	T	-0.1133	0.025	0.3699	3440	8.83E-06	0.005732846
C	T	0.1716	0.029	0.2464	3440	2.85E-09	0.010207834
G	C	0.1741	0.025	0.502	3440	2.61E-12	0.014135246
G	C	0.5926	0.13	0.0094	3440	4.97E-06	0.0060412
G	A	0.1611	0.027	0.2977	3440	3.80E-09	0.010050645
T	C	-0.13	0.029	0.2419	3440	5.58E-06	0.005974444
T	G	-0.2194	0.048	0.0731	3440	4.61E-06	0.006084526
A	G	3.014	0.669	0.0003	3440	6.92E-06	0.005858747
T	C	0.62	0.134	0.0083	3440	3.63E-06	0.006212347
A	C	0.2365	0.05	0.0696	3440	2.02E-06	0.006542072
A	T	0.8378	0.164	0.0057	3440	3.65E-07	0.00749294
A	G	-0.1818	0.038	0.8792	3440	1.36E-06	0.006760842
C	G	3.787	0.827	0.0003	3440	4.81E-06	0.006063105
A	G	0.1667	0.034	0.3343	3440	9.01E-07	0.00698844
T	C	3.372	0.741	0.0003	3440	5.52E-06	0.005986966
A	G	-0.1759	0.036	0.3456	3440	1.28E-06	0.006790768
A	G	-0.1112	0.025	0.5038	3440	9.55E-06	0.00568677
A	T	-0.2425	0.052	0.0593	3440	3.30E-06	0.006267957
T	A	0.3265	0.069	0.0339	3440	2.48E-06	0.006429753
T	C	0.122	0.024	0.49	3440	6.47E-07	0.007179908
G	A	-0.1391	0.03	0.2328	3440	2.58E-06	0.006408799
G	A	-0.3267	0.072	0.0336	3440	5.27E-06	0.006012459
C	A	-0.1567	0.034	0.1564	3440	3.23E-06	0.006279237
T	C	0.1619	0.035	0.148	3440	4.56E-06	0.0060914
T	A	0.8456	0.171	0.0051	3408	7.70E-07	0.007140725
A	G	2.141	0.228	0.0032	3408	1.10E-20	0.025199896
T	C	0.3324	0.072	0.0255	3408	4.12E-06	0.006203132
A	G	-0.3874	0.073	0.9746	3408	1.06E-07	0.008260927
A	G	1.176	0.166	0.0044	3408	1.77E-12	0.014478354
T	C	0.9379	0.201	0.0034	3408	3.24E-06	0.006335738
C	T	1.018	0.172	0.0043	3408	3.23E-09	0.010232947
C	T	-0.1218	0.026	0.2377	3408	2.74E-06	0.006432611
A	G	0.3781	0.073	0.0232	3408	2.32E-07	0.007820838
A	G	1.428	0.306	0.0012	3408	3.09E-06	0.006362007
G	A	0.1631	0.024	0.2974	3408	2.82E-11	0.012920201
T	C	-0.1308	0.026	0.2347	3408	5.07E-07	0.007377133
A	C	0.1398	0.025	0.2664	3408	2.72E-08	0.009027655
T	G	-0.2254	0.043	0.0734	3408	1.51E-07	0.00806115
A	G	-0.3532	0.072	0.0248	3408	8.58E-07	0.007085735
G	C	0.1794	0.022	0.5021	3408	8.34E-16	0.018834942
C	T	0.9633	0.211	0.0025	3408	5.30E-06	0.006061555
T	C	3.341	0.599	0.0003	3408	2.58E-08	0.009057914
A	C	-0.1157	0.024	0.3385	3408	1.09E-06	0.006956201
T	C	0.5934	0.12	0.0082	3408	8.24E-07	0.007112293
C	T	0.1195	0.026	0.755	3408	4.02E-06	0.006222039
G	A	6.548	1.434	0.0001	3408	5.16E-06	0.006080937
C	G	3.498	0.739	0.0003	3408	2.30E-06	0.006531385

T	C	-0.239	0.051	0.0502	3408	3.36E-06	0.006321203
C	A	2.077	0.45	0.0006	3408	4.13E-06	0.006201191
A	G	-0.1044	0.023	0.3999	3408	5.47E-06	0.006040644
A	C	3.334	0.599	0.0003	3408	2.76E-08	0.00902034
A	C	3.627	0.788	0.0001	3408	4.38E-06	0.006171825
C	T	3.552	0.785	0.0001	3408	6.23E-06	0.005971806
C	T	3.826	0.817	0.0001	3408	2.95E-06	0.006390712
C	G	0.2289	0.051	0.0508	3408	8.92E-06	0.00577216
G	A	4.616	1.03	0.0001	3408	7.68E-06	0.005858759
G	T	3.637	0.784	0.0001	3408	3.68E-06	0.006268748
T	A	5.687	1.277	0.0001	3408	8.68E-06	0.005785821
G	A	1.253	0.273	0.0015	3408	4.39E-06	0.006165716
G	T	4.583	1.034	0.0001	3408	9.60E-06	0.005731427
G	C	1.37	0.293	0.0018	3408	2.96E-06	0.00638724
C	T	2.128	0.481	0.0006	3408	9.73E-06	0.005722221
G	A	-0.1189	0.026	0.7676	3408	4.43E-06	0.006164835
G	A	-0.4449	0.1	0.012	3408	8.26E-06	0.005816002
T	C	-0.1409	0.03	0.1533	3408	3.79E-06	0.006251654
T	C	3.192	0.704	0.0003	3408	5.90E-06	0.006004576
A	T	4.225	0.932	0.0003	3408	5.95E-06	0.006000319
G	A	3.931	0.888	0.0001	3408	9.84E-06	0.005719855
C	T	0.12	0.025	0.2531	3408	2.45E-06	0.006501607
C	T	1.742	0.391	0.0007	3408	8.43E-06	0.005802364
G	A	3.776	0.847	0.0001	3408	8.49E-06	0.005800643
T	C	5.042	1.068	0.0001	3408	2.46E-06	0.006497296
A	G	-0.3021	0.066	0.0283	3408	5.51E-06	0.00604444
C	T	0.2069	0.047	0.0609	3408	9.20E-06	0.005758387
A	G	2.219	0.501	0.0004	3408	9.57E-06	0.005734679
A	G	-0.9923	0.175	0.0049	3440	1.45E-08	0.009291538
A	G	-1.201	0.177	0.0044	3440	1.42E-11	0.013192374
A	G	0.8535	0.187	0.0038	3440	5.25E-06	0.00601287
A	G	4.119	0.855	0.0001	3440	1.53E-06	0.006696841
A	G	-2.266	0.243	0.0032	3440	1.74E-20	0.024714597
A	G	0.4979	0.112	0.0112	3440	8.76E-06	0.005732518
T	C	-0.1245	0.028	0.7462	3440	6.21E-06	0.005918628
A	C	-0.1636	0.035	0.1243	3440	2.95E-06	0.006336513
G	A	0.1151	0.026	0.2985	3440	8.74E-06	0.005725868
A	G	-2.756	0.607	0.0003	3440	5.88E-06	0.005949206
T	G	1.355	0.285	0.0016	3440	2.11E-06	0.006514456
G	A	0.1351	0.026	0.7048	3440	2.26E-07	0.007758082
A	C	0.1623	0.036	0.1185	3440	8.60E-06	0.005742962
C	T	0.1474	0.032	0.2999	3440	3.27E-06	0.006277374
C	T	-0.1123	0.024	0.5653	3440	3.10E-06	0.006308774
A	G	-0.5022	0.11	0.0118	3440	5.09E-06	0.006033528
G	A	-0.2652	0.055	0.0471	3440	1.82E-06	0.006598429
C	T	0.1346	0.026	0.2773	3440	3.79E-07	0.007471765
A	G	-2.955	0.634	0.0003	3440	3.27E-06	0.006273471
T	C	1.176	0.26	0.0028	3440	6.37E-06	0.005902968

T	C	-0.1268	0.028	0.2273	3440	7.98E-06	0.005781702
A	G	0.293	0.065	0.0347	3440	6.36E-06	0.005908174
T	C	0.1202	0.024	0.4352	3440	3.52E-07	0.007516093
A	G	3.821	0.861	0.0001	3440	9.29E-06	0.005696538
T	C	0.196	0.043	0.0813	3440	5.22E-06	0.006014578
A	G	0.3121	0.066	0.0318	3440	2.59E-06	0.006404331
G	A	-0.5062	0.097	0.0148	3440	2.00E-07	0.00782884
A	G	-0.8811	0.199	0.0032	3440	9.64E-06	0.005677881
C	T	-0.2776	0.058	0.9586	3440	1.68E-06	0.006644742
A	G	-2.245	0.255	0.0032	3440	2.09E-18	0.022035206
A	C	-1.146	0.227	0.0035	3440	4.68E-07	0.007354502
A	G	-1.217	0.186	0.0044	3440	6.36E-11	0.012331337
C	A	-2.221	0.484	0.0006	3440	4.66E-06	0.006079123
C	G	0.1594	0.034	0.1584	3440	3.05E-06	0.006315572
G	A	0.1586	0.031	0.1955	3440	3.80E-07	0.007474714
T	C	-0.6195	0.132	0.0083	3440	2.98E-06	0.006324017
A	G	0.4791	0.106	0.0131	3440	6.50E-06	0.005892458
G	A	-0.1828	0.036	0.1472	3440	3.77E-07	0.00748073
T	C	0.1241	0.027	0.293	3440	4.54E-06	0.006094799
T	C	1.093	0.246	0.0023	3440	8.95E-06	0.005715167
A	G	-0.1121	0.025	0.4844	3440	5.53E-06	0.005990557
G	A	-0.1189	0.027	0.3176	3440	9.22E-06	0.005697739
A	G	0.9482	0.21	0.0032	3440	6.53E-06	0.005891645
G	A	0.1721	0.037	0.1794	3440	3.37E-06	0.006256677
T	C	-0.3326	0.073	0.0317	3440	4.80E-06	0.006060847
A	C	-2.683	0.577	0.0004	3440	3.42E-06	0.006248269
A	G	-0.5618	0.115	0.0118	3440	1.01E-06	0.006925641
G	C	0.2511	0.056	0.0517	3440	7.20E-06	0.005837609
G	A	-2.612	0.569	0.0004	3440	4.58E-06	0.006090638
T	C	-0.1388	0.031	0.189	3440	9.84E-06	0.005662422
A	G	-0.205	0.045	0.0808	3440	6.45E-06	0.005897277
T	C	-0.2163	0.045	0.9174	3440	1.48E-06	0.00671291
G	C	-0.1515	0.033	0.8179	3440	3.40E-06	0.006258042
G	A	0.1154	0.026	0.3503	3440	9.87E-06	0.005668077
A	T	-0.2357	0.052	0.0618	3440	7.29E-06	0.005831744
A	T	-0.2381	0.051	0.0641	3440	2.60E-06	0.006405535
G	T	2.135	0.441	0.0009	3440	1.35E-06	0.006767234
C	T	0.1982	0.036	0.1343	3440	5.93E-08	0.008512645
G	A	-0.1189	0.026	0.3121	3440	6.71E-06	0.005879676
G	T	-3.635	0.82	0.0001	3408	9.68E-06	0.005727477
A	G	4.289	0.815	0.0001	3408	1.49E-07	0.008068732
A	C	-1.228	0.208	0.0034	3408	3.74E-09	0.01014328
A	G	-2.319	0.231	0.0032	3408	2.18E-23	0.028722438
A	C	-0.5342	0.118	0.0095	3408	6.31E-06	0.005967726
C	T	-0.1161	0.025	0.2701	3408	4.53E-06	0.006150794
A	G	-1.125	0.169	0.0044	3408	3.18E-11	0.012835756
A	G	-1.975	0.433	0.0007	3408	5.30E-06	0.006064798
A	G	-0.1214	0.026	0.7504	3408	4.54E-06	0.006152663

T	C	0.2626	0.058	0.0387	3408	6.18E-06	0.005979012
C	T	-0.1279	0.027	0.7729	3408	2.66E-06	0.006446072
C	T	-0.2541	0.056	0.059	3408	6.97E-06	0.005912354
C	T	-3.37	0.747	0.0001	3408	6.55E-06	0.005944444
A	G	-2.743	0.579	0.0003	3408	2.23E-06	0.006549248
A	G	0.1131	0.025	0.7085	3408	6.97E-06	0.005913039
G	A	0.3244	0.071	0.0251	3408	5.19E-06	0.006076346
G	C	-0.4138	0.091	0.0166	3408	5.29E-06	0.006066482
G	A	-3.829	0.848	0.0001	3408	6.55E-06	0.005945484
G	A	-4.138	0.93	0.0001	3408	8.89E-06	0.005774402
T	C	0.235	0.052	0.0489	3408	7.23E-06	0.005891591
T	C	-4.689	1.046	0.0001	3408	7.61E-06	0.005861978
T	C	-0.4332	0.097	0.0145	3408	7.41E-06	0.00587723
C	T	0.1255	0.025	0.2764	3408	7.91E-07	0.007129182
G	A	-2.362	0.519	0.0004	3408	5.57E-06	0.006038481
G	A	0.1788	0.034	0.132	3408	1.50E-07	0.008058878
T	C	-5.264	1.141	0.0001	3408	4.07E-06	0.006206646
A	G	-5.054	1.11	0.0001	3408	5.42E-06	0.006046319
T	C	-1.191	0.242	0.0022	3408	9.12E-07	0.007051171
T	G	-3.741	0.765	0.0001	3408	1.04E-06	0.006977182
A	G	0.2897	0.062	0.0349	3408	2.92E-06	0.00639839
T	A	0.1021	0.023	0.4349	3408	5.95E-06	0.006000495
T	C	-1.907	0.424	0.0007	3408	7.14E-06	0.00590065
A	G	0.2989	0.065	0.0305	3408	3.78E-06	0.006254169
G	T	-0.1068	0.023	0.3922	3408	3.08E-06	0.006369355
A	G	-2.041	0.445	0.0007	3408	4.71E-06	0.006129232
C	T	-0.2594	0.055	0.9586	3408	3.03E-06	0.006378253
T	G	0.5343	0.119	0.0089	3408	7.32E-06	0.005880516
G	A	0.2212	0.042	0.0983	3440	1.75E-07	0.007901465
A	G	-0.1358	0.03	0.2516	3440	5.49E-06	0.005988609
C	G	-0.9047	0.192	0.0041	3440	2.40E-06	0.006446212
G	A	0.1174	0.026	0.6012	3440	5.41E-06	0.006001664
C	T	0.1749	0.039	0.1241	3440	6.24E-06	0.005917586
C	A	-0.1682	0.036	0.1426	3440	3.75E-06	0.006202662
T	C	-0.3325	0.074	0.0297	3440	8.09E-06	0.005774054
G	T	-0.4394	0.087	0.023	3440	5.08E-07	0.00730883
T	C	0.6769	0.074	0.0317	3440	9.35E-20	0.023764781
T	C	1.27	0.226	0.0029	3440	2.14E-08	0.009080343
C	T	0.8332	0.061	0.0462	3440	1.23E-41	0.05175029
A	G	0.6459	0.059	0.0483	3440	1.13E-27	0.03396601
C	T	0.2205	0.027	0.3594	3440	9.08E-16	0.018611207
C	T	-0.1378	0.027	0.3432	3440	4.91E-07	0.007325415
T	G	0.7307	0.065	0.041	3440	4.66E-29	0.035741315
G	T	0.1668	0.035	0.161	3440	2.22E-06	0.006492517
C	T	0.6237	0.052	0.0667	3440	9.80E-33	0.04043951
A	T	-0.915	0.02	0.4863	3440	1.00E-200	0.369699755
A	C	0.2385	0.05	0.0731	3440	1.70E-06	0.006636519
A	G	-0.4962	0.108	0.0138	3440	4.42E-06	0.00611013

G	T	0.1129	0.025	0.4656	3440	6.83E-06	0.0058656
G	T	0.163	0.036	0.858	3440	5.32E-06	0.006003529
G	A	0.211	0.042	0.0983	3440	6.26E-07	0.007194682
T	C	-1.4	0.309	0.0016	3440	5.96E-06	0.005943407
A	G	-0.1332	0.03	0.2516	3440	8.52E-06	0.00574364
A	G	1.893	0.428	0.0009	3440	9.94E-06	0.005659737
C	A	-0.1634	0.036	0.1426	3440	7.28E-06	0.005836543
G	T	-0.4	0.088	0.023	3440	5.16E-06	0.00602461
A	C	0.2297	0.05	0.0731	3440	4.15E-06	0.006144038
A	G	0.6238	0.059	0.0483	3440	7.75E-26	0.031607887
T	C	0.4475	0.093	0.0202	3440	1.56E-06	0.006688581
A	T	-0.9043	0.021	0.4863	3440	1.00E-200	0.359497942
C	T	0.8186	0.061	0.0462	3440	5.07E-40	0.049700287
A	G	0.2205	0.027	0.383	3440	1.73E-16	0.019555356
T	G	0.7675	0.065	0.041	3440	7.27E-32	0.039333731
C	A	-0.3008	0.053	0.0635	3440	1.64E-08	0.009228427
C	T	0.6402	0.052	0.0667	3440	2.59E-34	0.042452497
T	C	0.6977	0.074	0.0317	3440	7.75E-21	0.025170518
G	T	0.1651	0.035	0.161	3440	2.82E-06	0.006358096
A	G	-0.1159	0.025	0.439	3440	5.28E-06	0.006016173
T	C	0.8524	0.182	0.0041	3440	2.75E-06	0.006370881
G	T	0.1678	0.038	0.1269	3440	8.76E-06	0.005728989
G	T	0.2028	0.046	0.0802	3440	8.63E-06	0.005736861
A	G	0.7449	0.166	0.0065	3408	7.86E-06	0.005845794
C	T	-0.1164	0.026	0.4134	3408	8.85E-06	0.005780233
A	G	1.055	0.221	0.0032	3408	1.86E-06	0.006648396
G	A	0.2206	0.043	0.0977	3408	3.16E-07	0.007649482
G	C	1.007	0.227	0.0031	3408	9.19E-06	0.005761428
G	A	0.1191	0.026	0.6012	3408	5.40E-06	0.006054459
C	A	-0.1641	0.037	0.1425	3408	9.16E-06	0.005754167
G	A	-0.1551	0.033	0.8141	3408	2.10E-06	0.006581968
C	T	0.2118	0.027	0.3841	3408	7.19E-15	0.017607543
A	G	0.6785	0.06	0.0481	3408	1.49E-29	0.036706346
T	G	-0.1908	0.04	0.8854	3408	1.76E-06	0.00668173
T	G	0.7282	0.066	0.0409	3408	5.69E-28	0.034660447
T	C	0.4798	0.094	0.0204	3408	3.56E-07	0.007578781
A	T	-0.9143	0.021	0.4862	3408	1.00E-200	0.359388682
A	C	0.2538	0.051	0.0731	3408	5.69E-07	0.007316562
G	T	-0.4226	0.09	0.0226	3408	2.59E-06	0.006462166
C	T	0.8528	0.062	0.0461	3408	3.23E-42	0.052950685
T	C	0.7156	0.075	0.0317	3408	2.79E-21	0.025977243
C	T	0.6424	0.053	0.0668	3408	1.45E-33	0.041885357
C	A	-0.2939	0.054	0.064	3408	5.24E-08	0.008658228
G	T	0.1764	0.036	0.1611	3408	8.19E-07	0.007109178
T	C	0.1771	0.039	0.1158	3408	6.53E-06	0.005950478
T	G	2.067	0.449	0.0007	3408	4.21E-06	0.006193814
T	G	0.322	0.072	0.0339	3408	7.70E-06	0.005857152
T	C	0.4576	0.094	0.018	3408	1.08E-06	0.006951149

G	C	0.4736	0.104	0.0147	3162	5.61E-06	0.006503191
T	A	-0.2225	0.047	0.0751	3162	2.82E-06	0.006911619
C	T	0.2952	0.063	0.0432	3162	3.00E-06	0.006876274
A	G	-0.2637	0.057	0.0501	3162	4.54E-06	0.006628193
A	G	0.3036	0.063	0.0424	3162	1.59E-06	0.007258862
A	T	0.1268	0.026	0.3414	3162	1.68E-06	0.007232006
G	A	0.1228	0.028	0.2978	3162	9.76E-06	0.006172666
C	T	1.51	0.326	0.0019	3162	3.76E-06	0.00674349
A	C	-0.1542	0.035	0.1898	3162	9.66E-06	0.00617459
A	G	0.1418	0.029	0.3775	3162	1.06E-06	0.007504524
A	T	-0.2121	0.04	0.1154	3162	1.00E-07	0.008932756
A	G	-0.1441	0.032	0.2098	3162	6.65E-06	0.006399998
C	T	-0.7196	0.151	0.0076	3162	2.00E-06	0.007121763
A	G	0.1906	0.042	0.1006	3162	6.19E-06	0.006440412
T	C	0.1462	0.031	0.7919	3162	2.60E-06	0.006967117
A	T	-0.1165	0.025	0.4322	3162	4.42E-06	0.006640233
T	A	0.3988	0.089	0.021	3162	6.94E-06	0.00637088
T	C	0.1189	0.025	0.5416	3162	2.71E-06	0.006936459
C	T	0.1144	0.026	0.5079	3162	8.07E-06	0.006275901
G	T	0.2486	0.049	0.0792	3162	3.23E-07	0.008220499
G	C	0.162	0.034	0.1608	3162	2.15E-06	0.007074767
C	T	0.289	0.064	0.0432	3162	5.94E-06	0.006465492
G	C	-0.1995	0.044	0.0938	3162	6.27E-06	0.006436303
T	A	0.2315	0.048	0.0789	3162	1.23E-06	0.007415602
A	G	0.2805	0.063	0.044	3162	8.93E-06	0.006220478
G	A	-0.1851	0.042	0.8947	3162	8.66E-06	0.006240213
C	T	1.53	0.329	0.0019	3162	3.43E-06	0.006797218
A	T	0.7596	0.16	0.0063	3162	2.21E-06	0.007060021
A	G	0.1997	0.042	0.1053	3162	2.29E-06	0.007042353
A	T	-0.2016	0.04	0.1154	3162	5.41E-07	0.00791041
G	A	-0.1353	0.03	0.2362	3162	8.97E-06	0.00621737
G	C	-0.6975	0.148	0.0074	3162	2.58E-06	0.006965954
A	G	1.067	0.218	0.0033	3162	1.06E-06	0.007505602
A	G	0.1893	0.043	0.1006	3162	8.82E-06	0.006226386
C	A	0.1532	0.033	0.1904	3162	3.45E-06	0.006790251
C	T	0.1203	0.026	0.6097	3162	4.88E-06	0.006588378
G	A	0.1359	0.03	0.2448	3162	4.50E-06	0.006635642
G	T	0.2433	0.049	0.0792	3162	7.41E-07	0.007717958
G	A	0.1537	0.032	0.1882	3162	2.24E-06	0.007053712
G	A	0.184	0.041	0.8895	3130	5.88E-06	0.006535248
C	T	0.3032	0.063	0.0436	3130	1.41E-06	0.007408563
T	A	0.3431	0.073	0.0323	3130	2.44E-06	0.007075257
A	G	0.2961	0.063	0.0428	3130	2.53E-06	0.007050212
T	C	3.113	0.682	0.0003	3130	5.12E-06	0.006622114
C	T	-0.6309	0.138	0.0088	3130	5.01E-06	0.006633277
C	A	-0.1279	0.029	0.7407	3130	8.75E-06	0.006291936
T	A	0.2101	0.047	0.0792	3130	8.38E-06	0.006322377
A	G	0.1749	0.039	0.1209	3130	7.68E-06	0.006377963

C	T	1.437	0.324	0.0019	3130	9.37E-06	0.006253042
T	C	0.1186	0.026	0.5169	3130	4.05E-06	0.00676316
A	G	0.1789	0.033	0.2767	3130	4.52E-08	0.009512425
G	C	-0.1603	0.033	0.1823	3130	1.42E-06	0.007406333
C	T	-0.1475	0.032	0.2102	3130	4.05E-06	0.006763178
C	T	-0.1252	0.028	0.2863	3130	8.33E-06	0.006320267
T	C	0.1111	0.025	0.4709	3130	9.99E-06	0.006215592
A	G	0.1917	0.042	0.1002	3130	5.70E-06	0.006555868
A	G	-0.1445	0.032	0.1962	3130	7.00E-06	0.006432483
A	T	-0.116	0.025	0.4323	3130	4.70E-06	0.0066715
G	T	0.4095	0.082	0.0241	3130	5.98E-07	0.007933537
T	C	0.1237	0.025	0.5425	3130	1.07E-06	0.007573716
T	A	0.3988	0.089	0.0209	3130	7.36E-06	0.006402514
G	C	0.2525	0.051	0.0642	3130	9.06E-07	0.007680601
T	C	-0.1333	0.029	0.7284	3130	3.64E-06	0.006830743
G	A	0.1155	0.026	0.5032	3130	6.82E-06	0.006451349
A	G	0.2329	0.052	0.0642	3130	9.42E-06	0.006250552
G	T	0.248	0.048	0.0796	3130	3.32E-07	0.008291211
G	T	-0.2097	0.046	0.0829	3130	6.35E-06	0.006491572
T	C	0.3812	0.083	0.024	3130	5.03E-06	0.006635148
G	A	0.1463	0.032	0.1875	3130	5.33E-06	0.006600843
T	C	0.1155	0.024	0.4154	3162	1.82E-06	0.007175969
C	T	0.4364	0.096	0.0157	3162	6.26E-06	0.006432805
C	A	0.1185	0.025	0.6097	3162	2.02E-06	0.007117426
A	T	0.1889	0.038	0.1156	3162	5.62E-07	0.007889885
A	G	0.3014	0.06	0.0424	3162	4.46E-07	0.008025615
T	C	-0.6373	0.142	0.0081	3162	7.35E-06	0.006338691
T	G	0.1056	0.024	0.5103	3162	7.48E-06	0.006329429
G	A	0.1233	0.025	0.3471	3162	7.03E-07	0.007756737
T	C	0.1165	0.024	0.4154	3162	1.49E-06	0.007299855
G	A	-0.4036	0.083	0.0259	3162	1.19E-06	0.007436697
C	T	-0.1117	0.025	0.5832	3162	7.69E-06	0.00630384
A	T	0.1681	0.038	0.1156	3162	8.84E-06	0.00622868
G	A	0.1205	0.025	0.3471	3162	1.22E-06	0.007422905
T	C	-0.3788	0.085	0.0204	3162	8.36E-06	0.006260689
A	G	-0.1137	0.026	0.6746	3162	9.76E-06	0.006166249
T	A	-0.5294	0.115	0.0115	3162	3.95E-06	0.006715352
A	G	0.1278	0.027	0.4271	3162	1.67E-06	0.007231122
A	G	0.4914	0.1	0.015	3162	9.80E-07	0.007548873
C	T	-0.1637	0.032	0.2774	3162	4.70E-07	0.007993849
T	C	0.187	0.039	0.8887	3162	1.29E-06	0.007390543
C	G	0.1117	0.025	0.6108	3162	5.86E-06	0.006467708
G	A	0.1138	0.025	0.3775	3162	5.42E-06	0.006520736
A	G	0.9161	0.204	0.0033	3162	7.68E-06	0.006312652
G	C	-0.1711	0.036	0.1241	3162	2.36E-06	0.007023282
T	C	-0.8804	0.19	0.0043	3162	3.66E-06	0.00675866
C	T	-0.1355	0.031	0.1972	3162	9.17E-06	0.006203184
A	G	0.2421	0.052	0.0565	3162	3.40E-06	0.006800744

A	G	-0.4301	0.084	0.0202	3162	3.70E-07	0.008140175
T	C	0.1148	0.025	0.6627	3162	5.10E-06	0.006561808
T	G	0.1716	0.038	0.1067	3162	7.48E-06	0.006328196
C	G	0.1305	0.028	0.2508	3162	2.79E-06	0.006925702
T	C	-0.1096	0.024	0.6047	3162	5.30E-06	0.006541285
T	C	0.3661	0.079	0.9772	3162	4.07E-06	0.006692003
A	T	-0.5155	0.113	0.0115	3162	4.84E-06	0.006584904
C	A	0.1173	0.025	0.6099	3130	2.03E-06	0.007182691
C	A	1.22	0.257	0.0021	3130	2.13E-06	0.007153674
G	A	0.7448	0.154	0.0061	3130	1.28E-06	0.007465584
C	T	-0.2804	0.063	0.0358	3130	8.86E-06	0.006289131
T	C	2.867	0.633	0.0003	3130	6.19E-06	0.00650924
A	G	0.2893	0.059	0.0428	3130	8.79E-07	0.007692699
A	T	0.1654	0.037	0.116	3130	9.48E-06	0.006249599
A	G	-0.6583	0.148	0.0064	3130	9.08E-06	0.006272786
C	T	-0.6449	0.129	0.0088	3130	6.19E-07	0.007909323
C	A	-0.1294	0.027	0.7407	3130	1.61E-06	0.007322505
G	C	0.1747	0.036	0.1217	3130	1.65E-06	0.007313549
T	C	0.1702	0.038	0.8885	3130	8.73E-06	0.006295794
C	T	0.1597	0.028	0.3593	3130	1.04E-08	0.010418443
A	G	-0.1246	0.027	0.5722	3130	4.82E-06	0.006659659
A	G	-0.3217	0.071	0.0291	3130	5.25E-06	0.006604733
T	C	-0.7914	0.168	0.0053	3130	2.45E-06	0.007073217
C	G	-0.2976	0.067	0.0326	3130	8.17E-06	0.006336972
G	A	-0.1369	0.03	0.1957	3130	5.75E-06	0.006548214
C	G	-0.1887	0.043	0.0856	3130	9.35E-06	0.006253005
G	T	0.3588	0.077	0.0241	3130	3.10E-06	0.006926797
G	C	0.2522	0.048	0.0642	3130	1.67E-07	0.008710375
T	C	0.3491	0.078	0.9772	3130	8.63E-06	0.006304369
G	A	0.1164	0.024	0.5032	3130	1.28E-06	0.007471474
G	C	-0.1812	0.04	0.0968	3130	7.88E-06	0.006360917
T	C	0.3578	0.078	0.024	3130	4.81E-06	0.006660883
A	G	0.5796	0.129	0.0103	3162	7.12E-06	0.006353615
G	A	0.127	0.027	0.6221	3162	3.57E-06	0.006768058
A	G	-0.7401	0.13	0.0096	3162	1.37E-08	0.010146197
G	C	-0.1872	0.042	0.115	3162	8.30E-06	0.006267248
A	G	-0.2071	0.046	0.0919	3162	8.46E-06	0.006250158
G	C	-0.2504	0.054	0.061	3162	4.24E-06	0.006668099
T	C	-0.1288	0.028	0.6572	3162	3.58E-06	0.006766982
G	A	-0.1422	0.031	0.2362	3162	3.89E-06	0.006717687
A	G	0.414	0.087	0.0223	3162	1.83E-06	0.007174238
A	G	1.038	0.21	0.004	3162	7.66E-07	0.00770382
T	C	0.3212	0.058	0.0541	3162	3.41E-08	0.009589588
T	G	0.1673	0.038	0.149	3162	9.24E-06	0.006199235
C	T	0.5579	0.116	0.0141	3162	1.72E-06	0.007212753
C	T	-0.1173	0.026	0.4046	3162	9.43E-06	0.006186091
C	G	-0.1786	0.039	0.1301	3162	5.20E-06	0.006548623
G	A	-1.428	0.32	0.0016	3162	8.33E-06	0.006262357

G	A	0.6093	0.122	0.0106	3162	6.69E-07	0.007788465
T	C	0.3619	0.078	0.0291	3162	3.31E-06	0.006819256
G	A	0.1286	0.027	0.6221	3162	1.66E-06	0.007240077
A	G	-0.7447	0.125	0.0103	3162	3.00E-09	0.011065215
T	C	-0.2994	0.063	0.0419	3162	2.41E-06	0.007007822
C	T	0.1878	0.042	0.1855	3162	6.76E-06	0.006388749
T	A	0.3278	0.073	0.0343	3162	7.81E-06	0.006300432
T	C	0.2962	0.06	0.0493	3162	7.66E-07	0.007701819
A	G	0.4529	0.1	0.0172	3162	5.94E-06	0.006465714
A	G	-0.3753	0.073	0.0318	3162	3.37E-07	0.008198035
T	C	-0.1472	0.032	0.198	3162	5.97E-06	0.006465577
A	G	-0.2025	0.044	0.0984	3162	5.11E-06	0.006555958
G	A	0.1712	0.038	0.1278	3162	8.90E-06	0.006224288
T	C	0.2909	0.057	0.0541	3162	3.12E-07	0.008247142
C	T	0.1268	0.026	0.4836	3162	1.53E-06	0.007286664
T	C	0.1183	0.026	0.5818	3162	6.92E-06	0.0063677
C	T	-0.3205	0.07	0.0356	3162	5.52E-06	0.006508283
T	C	1.242	0.274	0.0022	3162	6.26E-06	0.006437368
A	C	-0.1314	0.026	0.4589	3162	3.24E-07	0.008218511
G	A	-0.1246	0.027	0.6602	3162	4.59E-06	0.006621695
C	G	0.3906	0.087	0.0218	3162	6.96E-06	0.006369254
C	G	-0.1887	0.038	0.1301	3162	9.45E-07	0.007571232
G	A	0.5958	0.121	0.0106	3162	9.33E-07	0.007584508
T	C	0.1216	0.027	0.4024	3162	5.00E-06	0.006575521
T	A	0.5559	0.121	0.012	3162	4.12E-06	0.006685657
G	C	-0.1638	0.037	0.1522	3130	8.30E-06	0.006330916
T	A	-0.2815	0.06	0.0498	3130	2.80E-06	0.006988018
T	C	0.3698	0.08	0.0276	3130	3.58E-06	0.006837998
C	G	1.319	0.286	0.0021	3130	3.97E-06	0.006773007
G	T	-0.5137	0.108	0.0157	3130	2.06E-06	0.007176281
A	G	-0.7492	0.132	0.0096	3130	1.32E-08	0.010264057
G	C	-0.2529	0.054	0.0613	3130	3.53E-06	0.006847515
T	C	-0.1537	0.028	0.657	3130	3.16E-08	0.009726844
T	A	-0.4806	0.089	0.022	3130	7.57E-08	0.009195464
A	G	0.4219	0.089	0.0219	3130	1.96E-06	0.007206905
G	A	-0.1198	0.026	0.6182	3130	5.91E-06	0.006536023
G	C	0.3022	0.062	0.0454	3130	1.31E-06	0.007451824
G	C	0.2385	0.052	0.0706	3130	4.28E-06	0.006732472
C	T	0.2098	0.042	0.1102	3130	6.62E-07	0.007871731
A	G	1.07	0.214	0.0038	3130	5.87E-07	0.007946017
T	A	0.2655	0.053	0.0674	3130	6.80E-07	0.007850422
A	G	0.3208	0.067	0.0414	3130	1.54E-06	0.007351572
A	C	-0.1933	0.039	0.1358	3130	7.43E-07	0.007803317
C	A	-0.1262	0.028	0.6765	3130	9.09E-06	0.006277902
C	T	0.5321	0.116	0.0142	3130	5.03E-06	0.006632028
A	G	0.1576	0.036	0.1674	3130	9.68E-06	0.00623631
G	A	-0.2355	0.052	0.073	3130	5.01E-06	0.006633821
G	A	0.2546	0.053	0.0671	3130	1.93E-06	0.007215564

C	G	0.4369	0.09	0.0211	3130	1.17E-06	0.00752405
A	G	-0.2978	0.066	0.0411	3130	6.76E-06	0.006452796
A	C	-0.1717	0.037	0.8589	3130	4.62E-06	0.006681549
T	C	0.2696	0.047	0.0864	3130	8.82E-09	0.010517826
T	C	-0.1249	0.026	0.453	3130	1.88E-06	0.007230255
G	A	-0.1215	0.027	0.372	3130	8.10E-06	0.006339106
G	A	-0.1465	0.031	0.2187	3130	3.19E-06	0.006906559
A	G	0.1532	0.032	0.1914	3403	1.75E-06	0.006694374
A	G	0.248	0.055	0.0579	3403	6.05E-06	0.006001966
G	A	-0.1922	0.04	0.1099	3403	1.50E-06	0.006785988
T	C	0.6817	0.149	0.0072	3403	4.83E-06	0.00612165
C	T	-0.3525	0.077	0.0264	3403	5.12E-06	0.006090892
C	T	0.1817	0.034	0.2013	3403	1.20E-07	0.008202566
G	A	-0.1557	0.029	0.6402	3403	9.18E-08	0.008348089
G	A	-0.406	0.092	0.0192	3403	9.56E-06	0.005743572
C	T	0.8353	0.184	0.0046	3403	6.06E-06	0.006000103
G	C	-0.2353	0.052	0.0623	3403	5.68E-06	0.006033887
T	C	0.1252	0.028	0.6988	3403	6.31E-06	0.005976011
A	G	0.1931	0.044	0.0893	3403	9.30E-06	0.005757269
T	C	0.1255	0.025	0.4812	3403	7.36E-07	0.00717886
G	A	0.2628	0.059	0.0476	3403	9.15E-06	0.005767231
A	G	0.3035	0.067	0.0364	3403	6.45E-06	0.00596358
T	C	-0.1959	0.041	0.0948	3403	2.00E-06	0.006615832
G	A	-0.567	0.126	0.0096	3403	7.46E-06	0.005878271
G	C	1.191	0.256	0.0021	3403	3.56E-06	0.006300581
G	A	0.1332	0.03	0.1985	3403	7.93E-06	0.005844554
C	T	-0.1465	0.033	0.1581	3403	8.47E-06	0.005806961
A	C	-0.3543	0.079	0.0226	3403	8.39E-06	0.005817088
A	G	-0.1303	0.025	0.3707	3403	1.28E-07	0.008170352
G	T	0.1354	0.026	0.3247	3403	1.41E-07	0.008115646
A	G	-0.1808	0.041	0.0954	3403	8.84E-06	0.005790908
A	G	0.9211	0.199	0.0035	3403	3.60E-06	0.006287692
A	G	0.1441	0.028	0.2445	3403	2.74E-07	0.007733903
C	T	0.1675	0.036	0.1264	3403	4.25E-06	0.006194163
T	C	0.107	0.024	0.5436	3403	8.66E-06	0.005802218
C	G	-1.183	0.262	0.0021	3403	6.24E-06	0.005978058
C	T	0.3686	0.082	0.0254	3371	6.60E-06	0.006006347
G	T	-0.1467	0.033	0.1936	3371	6.93E-06	0.005978539
C	A	0.116	0.026	0.5311	3371	6.95E-06	0.005979445
T	C	-0.2739	0.061	0.0485	3371	7.65E-06	0.005924072
C	G	-0.1262	0.028	0.6565	3371	5.00E-06	0.006163914
A	G	-0.1501	0.03	0.5389	3371	7.18E-07	0.007260419
C	A	-0.1266	0.027	0.4496	3371	3.77E-06	0.006320594
T	C	0.1905	0.031	0.3449	3371	9.17E-10	0.011064092
T	C	0.6974	0.153	0.0073	3371	5.51E-06	0.006109786
A	G	0.232	0.042	0.2906	3371	5.06E-08	0.00876637
T	A	0.1209	0.026	0.4776	3371	4.59E-06	0.006210928
A	G	-0.5296	0.118	0.0113	3371	7.95E-06	0.00590016

C	T	-0.1224	0.027	0.5874	3371	6.20E-06	0.006041696
C	A	0.2008	0.042	0.1018	3371	1.85E-06	0.006722239
T	C	-0.2824	0.063	0.046	3371	8.95E-06	0.005836528
G	A	0.1289	0.029	0.2872	3371	6.63E-06	0.0060064
G	A	0.2219	0.049	0.9255	3371	5.42E-06	0.006116133
A	C	-0.1395	0.027	0.3932	3371	1.88E-07	0.008026789
A	C	-0.255	0.056	0.0504	3440	4.87E-06	0.00605799
A	G	-0.201	0.041	0.1019	3440	9.37E-07	0.006968475
C	T	0.1618	0.03	0.3626	3440	9.18E-08	0.008264291
C	T	-0.1473	0.028	0.5487	3440	2.47E-07	0.007716191
A	G	-0.2814	0.06	0.0448	3440	2.47E-06	0.006434316
G	C	-0.2393	0.051	0.0624	3440	2.58E-06	0.006406741
T	C	-0.2244	0.051	0.9391	3440	9.68E-06	0.00567581
C	T	-0.2551	0.057	0.0471	3440	8.37E-06	0.00575466
G	A	0.2626	0.058	0.0458	3440	6.41E-06	0.005901449
T	A	-0.7006	0.158	0.0061	3440	9.15E-06	0.005704713
C	T	-0.1883	0.036	0.1343	3440	2.18E-07	0.007782748
T	C	0.1177	0.025	0.5494	3440	1.63E-06	0.006658957
C	T	-0.1277	0.028	0.2491	3440	6.96E-06	0.005859475
A	G	-0.3043	0.068	0.0339	3440	8.44E-06	0.00574898
G	A	0.1299	0.026	0.325	3440	5.81E-07	0.007231578
A	G	-0.1623	0.036	0.1307	3440	8.64E-06	0.005739826
C	T	-0.3709	0.078	0.0211	3440	1.92E-06	0.00657022
A	G	0.1308	0.029	0.1788	3440	9.45E-06	0.005690151
A	G	-0.1092	0.023	0.3706	3440	3.13E-06	0.00629627
A	C	0.1255	0.026	0.2731	3440	1.94E-06	0.006560977
A	G	-0.2169	0.038	0.1019	3440	9.95E-09	0.009510574
A	G	-0.2474	0.055	0.0448	3440	7.37E-06	0.005826396
G	A	-0.1458	0.026	0.6414	3440	3.34E-08	0.008828237
C	G	-0.2599	0.055	0.1715	3440	2.73E-06	0.006377759
G	A	0.142	0.027	0.5654	3440	2.40E-07	0.0077303
C	G	-0.1298	0.026	0.5487	3440	4.28E-07	0.007406349
G	A	0.1078	0.023	0.6156	3440	3.87E-06	0.006178781
G	T	-0.1252	0.028	0.2078	3440	8.34E-06	0.005758065
T	C	-0.6275	0.139	0.007	3440	6.15E-06	0.005923283
A	G	-0.1118	0.024	0.325	3440	4.08E-06	0.006150902
G	A	0.1327	0.03	0.1814	3440	7.25E-06	0.005832063
C	G	0.4293	0.096	0.0138	3440	8.66E-06	0.005736818
T	C	0.3436	0.069	0.0273	3440	7.31E-07	0.007105772
C	A	-0.1578	0.035	0.1144	3440	7.56E-06	0.005808185
T	C	-0.2218	0.047	0.9391	3440	2.29E-06	0.006473289
A	T	0.2126	0.048	0.061	3440	9.78E-06	0.00567043
A	G	-0.4812	0.104	0.0126	3440	4.07E-06	0.006149576
T	C	0.108	0.023	0.5433	3440	2.11E-06	0.006520026
C	T	-0.2034	0.033	0.1343	3440	1.35E-09	0.010627992
A	G	0.2113	0.047	0.0592	3440	7.13E-06	0.005843652
A	G	-0.3239	0.063	0.0339	3440	2.92E-07	0.007615719
G	A	0.1211	0.024	0.325	3440	4.74E-07	0.007352994

G	T	-0.1437	0.032	0.1938	3408	6.12E-06	0.005986046
A	G	0.1558	0.034	0.1715	3408	5.13E-06	0.006084451
A	C	-0.2749	0.057	0.0502	3408	1.71E-06	0.006699083
C	G	-0.1221	0.026	0.3885	3408	2.83E-06	0.006409985
A	G	-0.22	0.038	0.2229	3408	6.68E-09	0.009821034
T	C	0.6962	0.15	0.0072	3408	3.67E-06	0.006264686
T	C	0.1894	0.03	0.3439	3408	2.56E-10	0.01165994
G	A	-0.163	0.031	0.3842	3408	1.80E-07	0.007965399
G	A	-0.6929	0.147	0.0072	3408	2.46E-06	0.006494708
A	C	-0.2399	0.052	0.0634	3408	3.77E-06	0.006254271
T	C	-0.2461	0.052	0.9396	3408	2.48E-06	0.006489644
T	C	0.1212	0.025	0.5503	3408	1.61E-06	0.006725744
C	T	-0.2103	0.037	0.1339	3408	1.90E-08	0.009226508
A	G	-0.3167	0.071	0.0335	3408	7.47E-06	0.005873203
G	A	0.1395	0.027	0.3254	3408	1.85E-07	0.007952145
C	T	-0.209	0.044	0.9083	3408	2.51E-06	0.006482893
A	C	-0.1212	0.026	0.3928	3408	3.73E-06	0.006263735
C	T	0.6358	0.123	0.0047	3440	2.30E-07	0.007744918
A	C	2.171	0.459	0.0003	3440	2.29E-06	0.006472514
T	C	1.818	0.403	0.0004	3440	6.66E-06	0.005881086
G	A	6.643	1.199	0.0001	3440	3.22E-08	0.008844504
A	G	1.943	0.405	0.0004	3440	1.71E-06	0.006639801
T	C	3.122	0.695	0.0001	3440	7.36E-06	0.005825065
G	A	3.783	0.668	0.0001	3440	1.62E-08	0.009228789
G	A	4.52	0.79	0.0001	3440	1.13E-08	0.009433606
G	A	1.609	0.329	0.0004	3440	1.06E-06	0.006896496
A	G	2.169	0.475	0.0003	3440	5.16E-06	0.006022367
G	C	-0.08589	0.018	0.3554	3440	1.66E-06	0.0066485
A	G	2.141	0.474	0.0003	3440	6.46E-06	0.005900849
T	C	1.676	0.36	0.0004	3440	3.44E-06	0.006247386
C	T	2.579	0.532	0.0003	3440	1.31E-06	0.006782691
T	C	5.454	1.049	0.0001	3440	2.12E-07	0.00779689
G	C	0.104	0.018	0.502	3440	3.71E-09	0.010059712
T	G	0.1132	0.022	0.1891	3440	4.71E-07	0.007349742
G	A	0.6373	0.128	0.0048	3440	6.14E-07	0.007199298
G	A	6.493	1.126	0.0001	3440	8.69E-09	0.009573655
C	T	-0.7833	0.165	0.0029	3440	2.01E-06	0.006548063
C	T	1.67	0.375	0.0004	3440	8.68E-06	0.005732121
T	A	1.407	0.3	0.001	3440	2.73E-06	0.006374685
T	C	2.349	0.469	0.0003	3440	5.75E-07	0.00724253
A	G	-0.1825	0.039	0.0523	3440	3.84E-06	0.006188958
C	A	3.867	0.683	0.0001	3440	1.59E-08	0.009243243
T	C	-0.1202	0.024	0.1555	3440	6.57E-07	0.007161671
A	G	0.4503	0.097	0.0073	3440	3.72E-06	0.006205362
G	T	4.272	0.754	0.0001	3440	1.60E-08	0.009238152
C	T	3.974	0.725	0.0001	3440	4.54E-08	0.00865617
C	T	3.353	0.616	0.0001	3440	5.62E-08	0.008536561
G	A	0.4024	0.068	0.0192	3440	3.97E-09	0.010021709

T	A	0.1213	0.023	0.2932	3440	2.51E-07	0.007698579
T	C	3.021	0.626	0.0001	3440	1.43E-06	0.006733107
T	C	1.818	0.298	0.0004	3440	1.17E-09	0.010710548
A	G	-0.1468	0.03	0.1125	3440	1.54E-06	0.006693635
T	C	3.533	0.651	0.0001	3440	6.06E-08	0.008496915
A	C	2.345	0.469	0.0003	3440	5.96E-07	0.007218063
C	G	1.57	0.293	0.0009	3440	8.71E-08	0.008294271
C	T	4.025	0.64	0.0001	3440	3.59E-10	0.01137058
T	C	2.468	0.522	0.0003	3440	2.40E-06	0.006448847
G	A	4.848	0.81	0.0001	3440	2.34E-09	0.010313737
G	C	2.164	0.468	0.0003	3440	4.00E-06	0.00616646
G	A	3.787	0.677	0.0001	3440	2.44E-08	0.009006187
G	T	2.985	0.617	0.0001	3440	1.35E-06	0.006764482
A	G	3.141	0.639	0.0001	3440	9.27E-07	0.006974867
T	C	0.08508	0.018	0.6699	3440	4.05E-06	0.006156922
A	C	3.717	0.67	0.0001	3440	3.11E-08	0.008867654
A	G	2.492	0.527	0.0004	3440	2.39E-06	0.006450769
A	G	3.614	0.676	0.0001	3440	9.65E-08	0.008235239
C	G	3.794	0.668	0.0001	3440	1.44E-08	0.009301332
T	A	5.704	1.002	0.0001	3440	1.35E-08	0.009332395
G	A	4.877	0.86	0.0001	3440	1.53E-08	0.009264223
G	C	2.119	0.473	0.0003	3440	7.68E-06	0.005802797
C	T	4.765	0.846	0.0001	3440	1.92E-08	0.009139904
G	A	0.1465	0.031	0.089	3440	2.38E-06	0.006454482
G	T	4.68	0.811	0.0001	3440	8.65E-09	0.009585209
T	G	0.6544	0.144	0.0036	3440	5.58E-06	0.005975902
C	A	6.235	1.209	0.0001	3440	2.66E-07	0.007672149
A	T	0.1289	0.029	0.0999	3440	6.75E-06	0.005874357
A	C	4.11	0.726	0.0001	3440	1.60E-08	0.009240572
T	G	4.416	0.847	0.0001	3440	1.95E-07	0.00784181
C	G	2.288	0.462	0.0003	3440	7.85E-07	0.007067045
T	C	-0.1678	0.038	0.0583	3440	9.82E-06	0.005666036
G	A	2.385	0.476	0.0003	3440	5.55E-07	0.007260269
A	G	3.783	0.668	0.0001	3440	1.63E-08	0.009228789
T	C	3.436	0.602	0.0001	3440	1.25E-08	0.009378179
C	T	0.4657	0.105	0.0064	3440	9.82E-06	0.005664421
C	G	4.763	0.842	0.0001	3440	1.65E-08	0.009222827
G	T	3.833	0.677	0.0001	3440	1.63E-08	0.009229678
T	C	1.613	0.356	0.0006	3440	6.03E-06	0.005932343
A	G	3.894	0.691	0.0001	3440	1.94E-08	0.009136681
A	G	4.66	0.884	0.0001	3440	1.43E-07	0.008016959
C	A	-0.1143	0.022	0.1817	3440	2.82E-07	0.007640223
A	G	0.6042	0.12	0.0048	3440	4.65E-07	0.007364284
G	T	2.587	0.526	0.0003	3440	9.13E-07	0.006982642
A	G	1.692	0.357	0.0004	3440	2.26E-06	0.006476708
T	G	0.09014	0.019	0.3041	3440	2.95E-06	0.006333675
C	A	4.463	0.792	0.0001	3440	1.90E-08	0.009141914
G	A	3.946	0.697	0.0001	3440	1.60E-08	0.009239169

T	C	2.886	0.553	0.0003	3440	1.88E-07	0.007863694
T	A	5.2	1.132	0.0001	3440	4.52E-06	0.006096766
A	G	2.115	0.455	0.0003	3440	3.42E-06	0.006247405
G	A	0.1025	0.021	0.216	3440	1.05E-06	0.006903956
G	A	-0.1174	0.023	0.1709	3440	5.52E-07	0.007270233
G	A	3.755	0.664	0.0001	3440	1.72E-08	0.009200008
C	G	0.3131	0.069	0.0169	3440	6.49E-06	0.005897218
G	T	0.08737	0.019	0.3206	3440	3.81E-06	0.006193332
T	C	3.814	0.661	0.0001	3440	8.68E-09	0.009579806
T	C	4.893	0.839	0.0001	3440	5.91E-09	0.009799533
A	G	3.533	0.637	0.0001	3440	3.12E-08	0.008868578
A	T	0.7802	0.146	0.0033	3440	1.04E-07	0.008199533
A	G	0.9979	0.173	0.0029	3440	8.82E-09	0.009568545
T	C	0.2919	0.065	0.0212	3440	7.68E-06	0.005803453
G	A	-0.1289	0.025	0.8225	3440	1.53E-07	0.00797596
T	C	0.4087	0.09	0.9894	3440	5.61E-06	0.005977427
G	C	0.5165	0.101	0.0089	3440	3.53E-07	0.007515277
A	G	-0.103	0.02	0.3275	3440	2.23E-07	0.007766208
A	G	3.535	0.752	0.0001	3440	2.70E-06	0.006384379
C	G	0.1024	0.02	0.3703	3440	2.50E-07	0.007707477
T	C	0.4644	0.103	0.0083	3440	6.10E-06	0.005931908
G	A	5.539	1.224	0.0001	3440	6.23E-06	0.005917847
T	C	0.5074	0.104	0.0074	3440	1.21E-06	0.006832761
T	C	1.504	0.307	0.0012	3440	1.00E-06	0.006933013
A	C	-1.499	0.327	0.0009	3440	4.73E-06	0.006071618
C	T	-2.308	0.508	0.0003	3440	5.69E-06	0.005969357
T	C	1.491	0.324	0.0004	3440	4.44E-06	0.006107205
C	G	0.107	0.023	0.4567	3440	3.44E-06	0.006241362
C	T	-0.117	0.023	0.4702	3440	5.69E-07	0.007245716
C	T	3.043	0.67	0.0001	3440	5.73E-06	0.005962498
T	G	-0.1499	0.031	0.8994	3440	1.73E-06	0.006627441
A	G	0.1978	0.041	0.0541	3440	1.53E-06	0.006697707
G	A	4.274	0.88	0.0001	3440	1.23E-06	0.006815089
C	T	3.525	0.697	0.0001	3440	4.42E-07	0.007388768
A	T	0.1863	0.039	0.0637	3440	1.75E-06	0.006623415
C	G	1.424	0.32	0.0009	3440	9.14E-06	0.005709392
A	G	0.1011	0.02	0.325	3440	5.89E-07	0.007229198
T	C	0.0849	0.019	0.5055	3440	9.44E-06	0.005687169
T	C	0.3597	0.079	0.0157	3440	5.80E-06	0.005958909
T	C	1.535	0.345	0.0007	3440	8.66E-06	0.005734954
T	C	0.1139	0.025	0.1624	3440	7.78E-06	0.005793372
T	C	3.271	0.654	0.0001	3440	5.92E-07	0.007223775
G	A	0.1114	0.024	0.1812	3440	5.46E-06	0.005988738
G	T	0.1012	0.023	0.2205	3440	8.05E-06	0.005774768
C	T	0.08909	0.019	0.4751	3440	1.60E-06	0.006674828
A	G	0.6077	0.13	0.0048	3440	3.12E-06	0.006302598
A	G	0.4914	0.109	0.0074	3440	6.42E-06	0.005905821
T	C	1.187	0.268	0.001	3440	9.91E-06	0.005661865

C	T	-0.09624	0.02	0.3209	3440	2.23E-06	0.006484961
A	G	-0.1733	0.039	0.0592	3440	9.72E-06	0.005675332
T	C	4.134	0.912	0.0001	3440	6.04E-06	0.005936245
G	C	-0.108	0.024	0.8443	3408	7.89E-06	0.005843701
G	A	2.995	0.673	0.0001	3408	8.72E-06	0.005786145
G	A	3.551	0.795	0.0001	3408	8.13E-06	0.005824497
G	A	0.09093	0.018	0.6455	3408	8.93E-07	0.007061603
G	A	-0.1821	0.041	0.0508	3408	7.21E-06	0.005894248
T	G	0.1904	0.04	0.0534	3408	1.79E-06	0.006673878
C	T	-0.1235	0.028	0.1152	3408	8.21E-06	0.005815632
C	G	0.1051	0.018	0.3706	3408	1.17E-08	0.009503158
T	C	-0.08388	0.018	0.3526	3408	4.10E-06	0.006207621
A	G	3.006	0.673	0.0001	3408	8.21E-06	0.005818151
C	T	0.7631	0.161	0.0034	3408	2.24E-06	0.006548746
A	G	0.1225	0.026	0.1328	3408	2.70E-06	0.006437036
G	A	5.307	1.132	0.0001	3408	2.88E-06	0.006407873
A	T	-0.4417	0.094	0.0088	3408	2.42E-06	0.006504367
A	G	0.4812	0.097	0.0073	3408	8.31E-07	0.007100923
C	T	3.557	0.798	0.0001	3408	8.49E-06	0.005800461
G	A	3.054	0.685	0.0001	3408	8.45E-06	0.005802082
G	C	0.08708	0.018	0.3618	3408	2.28E-06	0.006536208
A	G	-0.2192	0.049	0.0335	3408	8.28E-06	0.005814148
C	T	-0.158	0.031	0.1084	3408	2.83E-07	0.007707168
G	C	-0.1153	0.025	0.2132	3408	5.39E-06	0.006052547
C	T	2.765	0.62	0.0001	3408	8.38E-06	0.005807619
G	A	0.1259	0.021	0.5109	3408	2.27E-09	0.010436549
T	C	1.373	0.3	0.0004	3408	4.92E-06	0.006104502
T	C	-0.1271	0.028	0.1143	3408	5.72E-06	0.006022587
C	T	3.304	0.645	0.0001	3408	3.11E-07	0.007652414
G	A	3.905	0.814	0.0001	3408	1.67E-06	0.006709293
T	C	0.3069	0.068	0.0178	3408	7.09E-06	0.005901641
C	T	3.772	0.851	0.0001	3408	9.63E-06	0.005729085
G	A	3.854	0.865	0.0001	3408	8.73E-06	0.005787217
C	G	3.003	0.672	0.0001	3408	8.09E-06	0.005828975
C	G	0.164	0.035	0.064	3408	3.56E-06	0.00628295
T	A	4.566	1.008	0.0001	3408	6.10E-06	0.005984725
A	C	3.256	0.73	0.0001	3408	8.48E-06	0.005801996
G	T	3.704	0.816	0.0001	3408	5.86E-06	0.006006655
T	C	0.1204	0.026	0.1357	3408	3.17E-06	0.006349618
C	T	0.1377	0.03	0.0998	3408	3.67E-06	0.006272135
G	A	0.5847	0.13	0.0047	3408	6.54E-06	0.005946165
A	G	2.997	0.673	0.0001	3408	8.60E-06	0.00579383
A	G	1.985	0.438	0.0004	3408	5.96E-06	0.005998675
C	G	3.775	0.847	0.0001	3408	8.56E-06	0.005796228
G	T	0.2261	0.049	0.0314	3408	4.61E-06	0.00614131
G	T	3.036	0.681	0.0001	3408	8.60E-06	0.005793006
T	C	-0.1685	0.031	0.0929	3408	3.80E-08	0.008841716
G	A	3.142	0.701	0.0001	3408	7.64E-06	0.005860358

T	C	-0.1691	0.038	0.0587	3408	7.11E-06	0.005896746
C	A	3.543	0.797	0.0001	3408	9.08E-06	0.005763771
G	C	-0.09081	0.019	0.3257	3408	1.33E-06	0.006842999
A	G	0.08278	0.018	0.3383	3408	7.06E-06	0.005903967
C	T	2.989	0.673	0.0001	3408	9.08E-06	0.005763118
C	T	-0.08242	0.018	0.4632	3408	3.02E-06	0.006379321
G	T	0.0932	0.019	0.321	3408	9.86E-07	0.007003509
T	C	3.043	0.665	0.0001	3408	4.96E-06	0.006101147
T	C	3.748	0.844	0.0001	3408	9.26E-06	0.005751837
T	C	-0.2521	0.052	0.0285	3408	1.59E-06	0.00674085
A	G	2.835	0.641	0.0001	3408	9.94E-06	0.005712276
A	G	-0.905	0.202	0.0039	3440	7.39E-06	0.005823994
A	G	0.3879	0.083	0.9749	3440	3.42E-06	0.006250719
C	T	-1.073	0.196	0.0042	3440	4.99E-08	0.008610821
A	G	-2.154	0.229	0.0029	3440	8.60E-21	0.025117318
G	A	0.1175	0.026	0.5685	3440	4.98E-06	0.006039765
A	C	-0.5894	0.133	0.0094	3440	9.48E-06	0.00568507
T	C	-0.5154	0.087	0.0212	3440	3.04E-09	0.01016842
C	G	-0.2373	0.046	0.0808	3440	2.95E-07	0.007610884
A	G	-2.996	0.675	0.0003	3440	9.42E-06	0.005687559
C	T	0.599	0.128	0.0089	3440	3.22E-06	0.006286751
T	C	-0.1281	0.028	0.7064	3440	4.42E-06	0.006103892
G	A	-3.353	0.708	0.0007	3440	2.25E-06	0.006483142
A	C	-0.1384	0.029	0.2762	3440	1.45E-06	0.006728624
G	A	0.1122	0.025	0.5241	3440	6.82E-06	0.005867765
G	A	0.374	0.069	0.0339	3440	5.24E-08	0.008573869
A	C	-0.1299	0.026	0.4436	3440	7.54E-07	0.007089829
T	C	-3.358	0.745	0.0003	3440	6.83E-06	0.00586815
A	G	0.1572	0.031	0.4339	3440	3.38E-07	0.007539967
T	C	0.1945	0.04	0.1933	3440	9.64E-07	0.006949997
T	C	0.158	0.032	0.1831	3440	1.05E-06	0.006903562
A	T	1.451	0.323	0.0015	3440	7.40E-06	0.005821426
T	C	-0.1205	0.027	0.3249	3440	8.82E-06	0.005723026
A	C	-0.3166	0.068	0.0347	3440	3.65E-06	0.006212936
A	G	-0.3108	0.067	0.0385	3440	3.31E-06	0.006266589
T	A	0.3729	0.082	0.0225	3440	6.22E-06	0.005919688
G	T	0.1134	0.025	0.4483	3440	7.56E-06	0.005810842
T	C	0.2542	0.051	0.0667	3440	7.32E-07	0.007106351
T	G	-2.596	0.283	0.0032	3440	8.26E-20	0.023844244
T	C	-0.427	0.086	0.0212	3440	7.35E-07	0.007103911
A	G	-1.249	0.188	0.0044	3440	3.55E-11	0.01266817
A	C	-1.138	0.23	0.0035	3440	8.12E-07	0.007048009
C	G	-0.205	0.046	0.0808	3440	7.64E-06	0.005807874
T	C	-0.2404	0.052	0.0624	3440	4.46E-06	0.006102149
A	G	-3.123	0.674	0.0003	3440	3.77E-06	0.006195146
G	A	-3.299	0.704	0.0007	3440	2.83E-06	0.006352001
C	T	-0.1219	0.027	0.689	3440	5.32E-06	0.00600943
T	A	-0.2553	0.055	0.0564	3440	2.97E-06	0.00632699

A	G	-2.1	0.474	0.0006	3440	9.84E-06	0.005664009
C	G	0.1446	0.026	0.623	3440	2.58E-08	0.008979686
C	T	-0.1288	0.028	0.2948	3440	3.73E-06	0.006201291
T	C	-0.1287	0.029	0.2669	3440	6.80E-06	0.005864403
C	G	0.1267	0.026	0.3285	3440	1.42E-06	0.006742042
T	A	0.1459	0.032	0.2058	3440	4.86E-06	0.006051727
T	C	0.2395	0.033	0.7693	3440	8.82E-13	0.014744458
G	C	-0.2338	0.035	0.7833	3440	1.48E-11	0.013151877
G	A	-3.338	0.717	0.0003	3440	3.34E-06	0.006262801
G	A	-0.2958	0.058	0.0709	3440	4.35E-07	0.007397588
C	T	0.2569	0.057	0.0516	3440	6.77E-06	0.005872386
T	C	0.1197	0.026	0.3775	3440	5.04E-06	0.006035728
G	T	-0.1421	0.032	0.1933	3440	6.79E-06	0.005869823
A	G	0.1442	0.032	0.1888	3440	7.44E-06	0.005821235
A	T	0.1205	0.026	0.4347	3440	2.72E-06	0.006379697
G	A	-0.4201	0.087	0.0215	3440	1.39E-06	0.006749415
C	T	0.2164	0.048	0.0807	3440	7.08E-06	0.005847073
G	A	-0.1203	0.027	0.3267	3440	7.45E-06	0.005818951
A	T	-0.1678	0.037	0.1359	3440	5.93E-06	0.00594657
C	T	-0.2324	0.052	0.058	3440	8.76E-06	0.005731167
T	C	-0.3289	0.073	0.0282	3440	6.59E-06	0.005887182
G	A	1.272	0.281	0.0019	3440	6.10E-06	0.005929781
T	A	-0.7813	0.167	0.0069	3408	2.83E-06	0.006411984
A	G	-1.253	0.191	0.0044	3408	5.53E-11	0.012535284
A	C	-0.1174	0.026	0.4272	3408	5.05E-06	0.006085822
A	C	-1.182	0.235	0.0034	3408	5.02E-07	0.007387355
A	G	-2.393	0.261	0.0032	3408	8.39E-20	0.02405457
T	C	-0.4597	0.087	0.0214	3408	1.45E-07	0.008083377
C	G	1.269	0.286	0.0019	3408	9.27E-06	0.005751672
C	T	-0.1398	0.031	0.7729	3408	5.38E-06	0.006055724
G	A	-3.608	0.712	0.0007	3408	4.19E-07	0.00748474
T	C	-0.7363	0.16	0.0063	3408	4.54E-06	0.006152656
T	A	0.2493	0.048	0.0737	3408	2.26E-07	0.00783361
C	G	0.5675	0.125	0.0112	3408	5.73E-06	0.006021218
G	A	-0.2333	0.051	0.0651	3408	6.02E-06	0.005992544
T	C	0.1483	0.033	0.1831	3408	5.53E-06	0.006042939
G	C	-0.1226	0.027	0.3988	3408	8.41E-06	0.005802359
A	G	-0.1783	0.036	0.8628	3408	1.02E-06	0.006987414
T	C	-0.1184	0.026	0.5817	3408	5.60E-06	0.0060343
C	T	0.1862	0.04	0.1174	3408	2.99E-06	0.006381362
T	C	0.4924	0.104	0.0158	3408	2.40E-06	0.006509755
A	G	-0.1243	0.027	0.5861	3408	3.57E-06	0.00628181
G	A	0.1826	0.038	0.132	3408	1.99E-06	0.006611755
T	C	0.1545	0.035	0.1599	3408	9.15E-06	0.005756928
G	C	0.1499	0.033	0.8251	3408	7.55E-06	0.005868601
A	G	-0.2328	0.045	0.0847	3408	2.99E-07	0.007679745
A	G	-0.1977	0.04	0.1161	3408	6.69E-07	0.007224104
A	G	-0.2801	0.063	0.0417	3408	9.93E-06	0.00571436

G	A	0.249	0.048	0.0791	3408	2.10E-07	0.007873321
T	C	-0.1201	0.026	0.5781	3408	2.59E-06	0.006461749
G	A	-2.191	0.483	0.0006	3408	5.86E-06	0.006009149
G	C	0.1259	0.028	0.4369	3440	6.81E-06	0.005867922
G	A	-0.2826	0.062	0.0464	3440	5.14E-06	0.00602643
G	T	0.2159	0.033	0.189	3440	5.00E-11	0.012475901
C	T	-0.5406	0.117	0.0134	3440	3.80E-06	0.006188876
C	T	-0.8722	0.188	0.0051	3440	3.71E-06	0.006204851
A	G	0.3042	0.068	0.0392	3440	7.86E-06	0.005789006
T	C	0.1779	0.037	0.1456	3440	2.00E-06	0.006544788
G	A	-0.2243	0.048	0.0804	3440	3.00E-06	0.006320757
G	A	0.4291	0.076	0.0312	3440	1.48E-08	0.009285563
T	C	0.3371	0.067	0.0381	3440	6.03E-07	0.007215007
T	C	-1.705	0.36	0.0012	3440	2.21E-06	0.006489065
A	G	0.2727	0.061	0.0464	3440	8.41E-06	0.005749852
G	C	0.2334	0.047	0.0824	3440	7.39E-07	0.007096787
T	C	-0.1228	0.026	0.4331	3440	2.31E-06	0.006467636
C	T	-0.1255	0.026	0.4119	3440	1.61E-06	0.006671266
A	G	0.1771	0.039	0.1154	3440	6.39E-06	0.00590443
G	A	-0.1431	0.03	0.7741	3440	2.70E-06	0.006383375
C	T	-0.14	0.03	0.2654	3440	3.92E-06	0.00617178
T	C	0.3088	0.069	0.0356	3440	7.68E-06	0.005803684
T	C	-0.2123	0.046	0.0887	3440	4.45E-06	0.006103609
G	T	0.1863	0.042	0.1084	3440	8.90E-06	0.005719563
G	A	0.1773	0.039	0.1294	3440	5.15E-06	0.006024209
T	A	0.1485	0.032	0.8015	3440	4.67E-06	0.00607706
T	C	-0.2627	0.059	0.049	3440	7.76E-06	0.005796328
G	A	0.1701	0.027	0.6741	3440	5.51E-10	0.011135449
C	T	-0.2368	0.052	0.0702	3440	5.77E-06	0.005962553
C	T	-0.1482	0.032	0.6388	3440	4.70E-06	0.006071301
A	G	187	33.11	0.0016	3408	1.74E-08	0.009272979
A	G	263.3	37.24	0.0013	3408	1.89E-12	0.01445635
G	T	147.3	23.48	0.0035	3408	3.93E-10	0.011416243
T	G	328.7	56.2	0.0006	3408	5.40E-09	0.009937787
C	T	123.8	19.64	0.0043	3408	3.21E-10	0.011524567
G	A	166.5	30.94	0.0018	3408	7.96E-08	0.008425845
T	A	358.6	51.64	0.0006	3408	4.54E-12	0.01395232
C	A	160.1	33.79	0.0015	3408	2.25E-06	0.00654418
A	G	84.4	18.01	0.0059	3408	2.88E-06	0.006402775
T	C	760.9	100.5	0.0001	3408	4.68E-14	0.016541673
G	T	128.6	28.74	0.0021	3408	7.87E-06	0.005840699
T	C	180.6	39.33	0.0012	3408	4.54E-06	0.006149068
G	A	725.9	100.6	0.0001	3408	6.76E-13	0.015047806
C	T	644.1	106	0.0001	3408	1.37E-09	0.01071804
G	A	174.6	39.25	0.0012	3408	8.93E-06	0.005772915
G	A	284.3	52.5	0.0009	3408	6.56E-08	0.008531287
T	C	210.9	46.9	0.001	3408	7.17E-06	0.00589846
G	A	58.1	12.79	0.011	3408	5.74E-06	0.006018522

T	G	721.2	91.57	0.0001	3408	4.50E-15	0.017876057
C	T	378	77.39	0.0003	3408	1.09E-06	0.006951599
A	G	127.9	26.15	0.0023	3408	1.04E-06	0.006970442
T	C	75.56	16.06	0.0065	3408	2.63E-06	0.006453295
T	C	172.5	37.71	0.0012	3408	4.91E-06	0.006102491
A	G	176.7	29.35	0.0022	3408	1.92E-09	0.010523562
C	A	530.8	58.46	0.0004	3408	1.81E-19	0.023619129
T	C	38.01	8.027	0.0283	3408	2.27E-06	0.006536444
T	C	111	19.7	0.0048	3408	1.89E-08	0.009229686
C	A	259.1	57.74	0.0006	3408	7.43E-06	0.005873854
T	A	88.02	18.79	0.0051	3408	2.91E-06	0.006397675
A	G	463.8	58.31	0.0004	3408	2.46E-15	0.018225834
C	G	307.6	55.58	0.0006	3408	3.35E-08	0.008907385
G	T	114.9	22.64	0.0034	3408	4.12E-07	0.007500975
T	G	342.5	71.9	0.0003	3408	1.98E-06	0.006614267
G	C	167.9	37.24	0.0012	3408	6.74E-06	0.00592925
C	G	344	76.55	0.0003	3408	7.21E-06	0.005890621
A	G	80.28	17.31	0.006	3408	3.67E-06	0.006271749
C	T	185.1	26.88	0.0022	3408	6.76E-12	0.013723142
A	T	148.4	29.95	0.0019	3408	7.54E-07	0.007152488
T	C	485.7	72.98	0.0003	3408	3.29E-11	0.012829831
A	G	40.96	8.481	0.0257	3408	1.43E-06	0.00679773
T	C	791.9	98.43	0.0001	3408	1.17E-15	0.018638676
A	G	444.1	76.59	0.0003	3408	7.31E-09	0.009769094
A	G	213	36.73	0.0015	3408	7.23E-09	0.009771321
A	C	979.6	163.9	0.0001	3408	2.52E-09	0.010373171
A	C	187.5	39.04	0.0012	3408	1.63E-06	0.006722856
T	C	429	74.81	0.0003	3408	1.06E-08	0.009557078
C	T	183.6	36.98	0.0012	3408	7.19E-07	0.007180953
A	G	405.6	61.74	0.0004	3408	5.84E-11	0.012505406
A	T	521.2	71.33	0.0004	3408	3.40E-13	0.015424595
G	A	20.08	2.814	0.3526	3408	1.18E-12	0.014721052
A	G	270.7	56.22	0.0007	3408	1.54E-06	0.006756951
A	C	174.2	36.66	0.0012	3408	2.11E-06	0.006581788
T	C	35.09	6.871	0.0398	3408	3.46E-07	0.007594799
C	T	259.1	45.06	0.0007	3408	9.70E-09	0.009608591
C	G	24.07	5.37	0.0646	3408	7.61E-06	0.005860724
T	C	164.6	36.88	0.0012	3408	8.38E-06	0.005810951
A	G	283	46.74	0.0007	3408	1.55E-09	0.010642632
G	A	147.8	30.44	0.0023	3408	1.26E-06	0.006870147
A	G	641.1	67.08	0.0004	3408	2.24E-21	0.026102325
A	G	115.7	24.44	0.0026	3408	2.32E-06	0.006533082
G	A	227.1	47.11	0.0007	3408	1.50E-06	0.006772627
A	G	189.7	34.49	0.0015	3408	4.09E-08	0.00879854
T	C	102.4	21.6	0.0035	3408	2.21E-06	0.006551462
C	T	306.9	59.3	0.0004	3408	2.42E-07	0.00779803
A	C	282.4	62.95	0.0004	3408	7.48E-06	0.005870583
G	A	349.4	70.62	0.0003	3408	7.87E-07	0.007131527

T	C	70.05	15.71	0.0072	3408	8.56E-06	0.005800134
T	C	116.5	21.81	0.004	3408	9.78E-08	0.008302714
G	T	248.4	47.12	0.0007	3408	1.44E-07	0.008088462
A	G	179.1	36.43	0.0016	3408	9.26E-07	0.00704213
A	G	556.4	84.21	0.0004	3408	4.51E-11	0.012647927
A	G	239.7	42.01	0.001	3408	1.26E-08	0.009462417
A	C	208	41.21	0.0009	3408	4.70E-07	0.00741972
G	A	159.7	26.69	0.0025	3408	2.42E-09	0.010396196
A	G	278.7	62.95	0.0004	3408	9.84E-06	0.005718632
T	C	60.86	13.51	0.0089	3408	6.89E-06	0.005919366
T	C	751.4	100.1	0.0001	3408	7.82E-14	0.016264952
A	G	61.26	13.84	0.0092	3408	9.91E-06	0.005716006
G	T	550.9	98.17	0.0001	3408	2.17E-08	0.009155747
C	T	184.5	38.41	0.0013	3408	1.62E-06	0.006724719
A	G	760.5	100.6	0.0001	3408	5.25E-14	0.016492279
G	A	281.9	45.23	0.0009	3408	5.10E-10	0.011269774
T	A	203.6	37.91	0.0013	3408	8.35E-08	0.00839244
C	T	155.8	32.54	0.0018	3408	1.76E-06	0.006681726
A	C	473.4	81.22	0.0003	3408	6.10E-09	0.009870135
C	T	137.7	22.45	0.0035	3408	9.64E-10	0.010918617
T	C	142.3	22.5	0.0037	3408	2.87E-10	0.011600526
A	G	98.05	21.43	0.0037	3408	4.93E-06	0.006105085
C	A	55.39	9.959	0.0175	3408	2.87E-08	0.00899513
T	C	548.7	98.8	0.0004	3408	3.01E-08	0.008968993
G	A	285.1	50.77	0.0006	3408	2.11E-08	0.009168124
G	A	284.6	56.25	0.0006	3408	4.41E-07	0.007455472
A	G	186.7	36.52	0.0012	3408	3.37E-07	0.007610439
T	C	173.4	37.29	0.0012	3408	3.45E-06	0.006304738
C	A	1005	130	0.0003	3408	1.39E-14	0.017234385
C	G	318.1	62.32	0.0004	3408	3.51E-07	0.007586917
G	A	240.1	50.93	0.0006	3408	2.52E-06	0.006479096
G	C	272.3	60.5	0.0004	3408	7.03E-06	0.005908961
T	C	293.3	48.87	0.0007	3408	2.16E-09	0.010458607
G	A	112.1	23.78	0.0029	3408	2.55E-06	0.006478363
A	G	202.6	39.65	0.001	3408	3.41E-07	0.007602885
A	G	610.4	106.6	0.0001	3408	1.11E-08	0.009529206
T	C	276.6	51.66	0.0007	3408	9.20E-08	0.008341766
G	A	154.5	22.53	0.0032	3408	8.23E-12	0.013610789
A	G	76.96	15.41	0.0075	3408	6.26E-07	0.007265381
A	G	72.32	16.06	0.0072	3408	6.94E-06	0.005914931
A	G	397.6	71.92	0.0004	3408	3.50E-08	0.008888253
T	C	105.2	21.94	0.0035	3408	1.70E-06	0.006700985
C	T	269.9	57.94	0.0009	3408	3.33E-06	0.006326926
G	A	133.9	24.58	0.0028	3408	5.41E-08	0.008632419
C	A	347.8	56.2	0.0006	3408	6.81E-10	0.011113058
G	A	67.16	14.87	0.0079	3408	6.46E-06	0.005949879
A	G	202.2	40.35	0.0012	3408	5.66E-07	0.007314545
T	C	55.15	11.93	0.012	3408	3.96E-06	0.006231545

T	C	70.99	14.44	0.0084	3408	9.31E-07	0.007041923
G	A	161	35.65	0.0013	3408	6.54E-06	0.00594897
T	C	591	96.85	0.0001	3408	1.16E-09	0.01080828
T	A	383.2	58.71	0.0004	3408	7.72E-11	0.012346163
A	G	138.5	30.03	0.0028	3408	4.11E-06	0.00620279
C	T	384.3	58.05	0.0006	3408	4.14E-11	0.012696598
T	C	36.01	7.839	0.0296	3408	4.51E-06	0.006153816
A	C	46.66	9.776	0.0191	3408	1.89E-06	0.006640092
A	G	251.6	42.22	0.001	3408	2.78E-09	0.010312955
A	C	101.8	19.19	0.0047	3408	1.19E-07	0.008189824
C	G	240	48.24	0.0007	3408	6.85E-07	0.007210502
T	C	191.2	40.62	0.0012	3408	2.61E-06	0.006459249
T	G	164.9	31.9	0.0018	3408	2.47E-07	0.0077798
T	C	116.9	26.12	0.0025	3408	7.84E-06	0.005843029
A	G	38.64	8.697	0.0239	3408	9.16E-06	0.00575874
C	T	224.8	32.07	0.0018	3408	2.83E-12	0.014212756
T	G	-14.59	2.819	0.6067	3408	2.40E-07	0.007798672
C	A	74.28	11.52	0.0125	3408	1.29E-10	0.01205239
A	C	436	75.02	0.0003	3408	6.72E-09	0.009813776
G	A	102.9	21.6	0.0038	3408	1.97E-06	0.006615173
C	A	50.22	10.87	0.0147	3408	3.96E-06	0.006224195
A	G	389.5	63.7	0.0006	3408	1.08E-09	0.010851703
T	C	314.9	66.75	0.0004	3408	2.48E-06	0.006488081
G	C	242.6	49.52	0.0006	3408	1.01E-06	0.006993152
T	C	151.3	32.96	0.0016	3408	4.58E-06	0.00614507
T	C	148.2	25.16	0.0026	3408	4.22E-09	0.01007805
C	T	-22.92	4.094	0.2372	3408	2.31E-08	0.009112932
A	G	913.1	98.95	0.0001	3408	4.71E-20	0.024377398
A	G	22.81	3.563	0.2188	3408	1.74E-10	0.011883044
G	C	326.5	55.73	0.0006	3408	5.13E-09	0.009970952
T	C	111.7	25.03	0.0026	3408	8.36E-06	0.005809712
A	G	52.78	11.43	0.0129	3408	4.04E-06	0.006217817
T	A	419.1	73.98	0.0003	3408	1.60E-08	0.009329031
A	T	69.53	11.54	0.0139	3408	1.87E-09	0.010539781
A	G	281.9	47.31	0.0009	3408	2.80E-09	0.010310592
A	C	404.2	71.96	0.0003	3408	2.09E-08	0.009172941
G	A	29.71	5.912	0.0534	3408	5.29E-07	0.007355809
T	A	268.7	51.04	0.0007	3408	1.49E-07	0.008066718
A	G	211.9	42.67	0.0012	3408	7.14E-07	0.00718432
A	G	450.6	77.86	0.0003	3408	7.79E-09	0.009732103
T	C	286.9	45.35	0.0009	3408	2.84E-10	0.011607439
A	G	30.71	6.857	0.0469	3408	7.78E-06	0.005851176
T	C	595.1	71.5	0.0003	3408	1.22E-16	0.019921817
A	G	332.4	56.32	0.0006	3408	3.92E-09	0.010117675
G	A	154.3	30.79	0.0018	3408	5.72E-07	0.007315167
C	G	887.7	146	0.0001	3408	1.33E-09	0.010731033
T	A	726.8	82.02	0.0003	3408	1.26E-18	0.022521532
A	C	132.8	20.58	0.0035	3408	1.24E-10	0.012070679

C	G	271.2	48.48	0.0007	3408	2.39E-08	0.009098817
C	T	152.1	30.1	0.0022	3408	4.58E-07	0.007436765
A	G	177.3	36.41	0.0013	3408	1.17E-06	0.006909796
C	T	256.7	45.57	0.0009	3408	1.91E-08	0.009225058
T	C	521.4	86.78	0.0004	3408	2.08E-09	0.010481587
T	A	109.8	24.29	0.0031	3408	6.36E-06	0.005960105
T	C	141.3	29.8	0.0021	3408	2.21E-06	0.006553851
A	G	379.6	61.08	0.0006	3408	5.76E-10	0.011206254
G	A	198.9	42.02	0.0009	3408	2.31E-06	0.006531486
A	G	192.8	34.06	0.0022	3408	1.63E-08	0.009314531
C	T	178.9	27.52	0.0023	3408	9.07E-11	0.012248194
G	A	93.75	20.68	0.0044	3408	6.00E-06	0.005994194
T	G	447.9	85.71	0.0003	3408	1.84E-07	0.007949382
A	G	26.01	5.859	0.0556	3408	9.30E-06	0.005749498
T	C	197.4	39.3	0.001	3408	5.37E-07	0.00734863
C	T	48.75	10.58	0.0157	3408	4.21E-06	0.00619129
A	G	75.05	12.99	0.0098	3408	8.29E-09	0.009699526
T	C	251.2	41.16	0.001	3408	1.15E-09	0.010811058
C	T	24.38	4.994	0.0789	3408	1.10E-06	0.006944552
T	C	591.6	84.77	0.0003	3408	3.56E-12	0.014089957
A	G	56.51	12.39	0.0113	3408	5.26E-06	0.006066882
G	T	172.7	28.26	0.0022	3408	1.12E-09	0.010839458
C	T	375.7	59.27	0.0007	3408	2.64E-10	0.011652593
C	A	-101.9	20.76	0.9956	3408	9.65E-07	0.007019959
C	T	217	42.86	0.001	3408	4.34E-07	0.007465539
A	G	557.6	102	0.0001	3408	4.91E-08	0.008692684
C	T	50.48	10.85	0.0156	3408	3.38E-06	0.006311461
G	A	-13.2	2.596	0.5346	3408	3.84E-07	0.007529334
T	C	49.04	9.728	0.0183	3408	4.89E-07	0.007401637
A	G	420	71.13	0.0003	3408	3.87E-09	0.010126816
G	A	154.6	30.45	0.0018	3408	4.02E-07	0.0075071
T	C	96.8	19.78	0.0041	3408	1.04E-06	0.006978422
T	C	144.2	27.81	0.0023	3408	2.29E-07	0.007827376
A	G	201.5	32.14	0.0018	3408	4.11E-10	0.011401934
T	G	504.2	95.57	0.0001	3408	1.41E-07	0.008100847
A	G	68.16	15.23	0.0075	3408	7.82E-06	0.005842717
A	G	402.2	60.78	0.0004	3408	4.24E-11	0.01268582
A	C	389.2	70.79	0.0003	3408	4.14E-08	0.008791586
T	C	204.7	45.46	0.0012	3408	6.91E-06	0.005914269
G	A	308.2	58.42	0.0004	3408	1.41E-07	0.008100471
T	C	32.56	6.516	0.0439	3408	6.13E-07	0.007273387
C	T	284.3	51.52	0.0006	3408	3.69E-08	0.008856032
T	C	268.9	60.64	0.0004	3408	9.51E-06	0.005736738
A	C	138.5	21.65	0.0037	3408	1.80E-10	0.011865883
A	G	160.1	33.15	0.0018	3408	1.42E-06	0.006797572
G	A	302.9	63.85	0.0004	3408	2.19E-06	0.006560224
G	A	46.12	9.799	0.0185	3408	2.61E-06	0.006458054
A	G	118.5	25.62	0.0023	3408	3.88E-06	0.006238227

A	C	61.08	11.67	0.0135	3408	1.77E-07	0.007974065
A	G	416.6	74.65	0.0003	3408	2.58E-08	0.009055835
A	G	244.1	49.16	0.0007	3408	7.18E-07	0.007182599
A	G	295.5	63.49	0.0006	3408	3.38E-06	0.006316158
A	G	80	15.92	0.0072	3408	5.26E-07	0.007355093
A	G	352.4	49.32	0.0007	3408	1.09E-12	0.014759389
T	C	134.4	26.88	0.0025	3408	6.06E-07	0.00728226
G	A	385.7	68.87	0.0004	3408	2.31E-08	0.009119287
A	C	40.36	7.773	0.0283	3408	2.20E-07	0.0078488
C	T	364.8	66.04	0.0004	3408	3.57E-08	0.008874109
T	C	259.3	51.36	0.0007	3408	4.70E-07	0.007423681
A	T	96.27	21.36	0.0035	3408	6.80E-06	0.005925145
G	A	284.3	60.84	0.0009	3408	3.08E-06	0.006366518
G	A	356.8	65.76	0.0004	3408	6.19E-08	0.008564284
T	C	303.3	53.88	0.0006	3408	1.95E-08	0.009212355
T	A	754.3	103.6	0.0001	3408	4.02E-13	0.015316717
C	T	157.9	34.66	0.0018	3408	5.40E-06	0.006053002
C	G	180.1	33.3	0.0016	3408	6.75E-08	0.008509964
C	G	216.7	36.18	0.0013	3408	2.33E-09	0.010416779
A	G	308	49.26	0.0007	3408	4.56E-10	0.011341212
A	C	189.6	35.71	0.0013	3408	1.18E-07	0.008203889
C	G	370	67.38	0.0004	3408	4.28E-08	0.008770334
A	G	221.6	42.71	0.0009	3408	2.26E-07	0.00783725
G	A	101.4	18.3	0.0053	3408	3.25E-08	0.008928513
T	A	133.6	28.54	0.0023	3408	2.95E-06	0.006388838
A	G	122.4	25.83	0.0025	3408	2.21E-06	0.006545793
G	T	17.9	3.428	0.1828	3408	1.86E-07	0.00793714
T	G	308.9	61.24	0.0006	3408	4.82E-07	0.007410297
G	A	165.1	36.13	0.0013	3408	5.05E-06	0.006089838
T	C	71.36	10.98	0.0147	3408	9.24E-11	0.0122421
T	G	232.5	42.11	0.001	3408	3.63E-08	0.008865605
A	G	320.2	56.32	0.0006	3408	1.42E-08	0.009395461
G	A	115.3	24.76	0.0032	3408	3.34E-06	0.006322708
A	T	341.3	71.93	0.0004	3408	2.17E-06	0.006562862
G	A	379.5	72.6	0.0003	3408	1.83E-07	0.007953945
C	A	591.3	99.63	0.0001	3408	3.23E-09	0.010229873
A	G	165.4	35.54	0.0013	3408	3.41E-06	0.006315173
T	C	123.2	21.68	0.004	3408	1.42E-08	0.009386582
C	A	215.5	46.23	0.0007	3408	3.27E-06	0.006335588
T	C	442.7	63.56	0.0004	3408	3.90E-12	0.014035024
T	C	128.8	22.13	0.0034	3408	6.54E-09	0.009841786
T	C	368.8	66.09	0.0004	3408	2.59E-08	0.009054418
T	C	259.6	43.23	0.0009	3408	2.11E-09	0.010470515
A	G	229.9	46.65	0.0009	3408	8.67E-07	0.007076052
A	G	230.8	48.41	0.0007	3408	1.93E-06	0.006625443
T	G	368.8	66.09	0.0004	3408	2.59E-08	0.009054418
T	G	51.71	10.96	0.0157	3408	2.49E-06	0.006489346
T	C	169	36.86	0.0012	3408	4.71E-06	0.006130453

T	G	162.1	34.1	0.0013	3408	2.09E-06	0.006586998
T	C	30.3	6.817	0.0373	3408	9.07E-06	0.005763533
G	C	141.4	31.72	0.0016	3408	8.62E-06	0.005797062
A	G	98.24	21.32	0.0037	3408	4.20E-06	0.006191635
C	T	267	58.64	0.0004	3408	5.46E-06	0.006046459
G	A	137.6	24.38	0.0025	3408	1.82E-08	0.009260393
C	G	240.2	52.12	0.0007	3408	4.19E-06	0.006193547
G	A	240	52.59	0.0006	3408	5.22E-06	0.006073942
G	T	109	15.71	0.0072	3408	4.64E-12	0.013928661
A	G	110	22.52	0.0029	3408	1.09E-06	0.006952151
C	T	296.2	50.18	0.0007	3408	3.93E-09	0.010120259
C	T	61.29	11.2	0.0141	3408	4.77E-08	0.008710521
T	C	159.2	29.91	0.0022	3408	1.09E-07	0.008244387
G	C	28.39	6.325	0.0462	3408	7.38E-06	0.005876927
T	C	227.3	47.01	0.0007	3408	1.39E-06	0.006813178
C	G	243.9	47.03	0.0007	3408	2.28E-07	0.007829971
T	C	18.54	3.845	0.1404	3408	1.48E-06	0.006776015
A	C	92.71	20.44	0.0044	3408	5.96E-06	0.00600037
A	G	186.4	27.77	0.0022	3408	2.26E-11	0.013047779
C	G	94.76	18.9	0.0047	3408	5.61E-07	0.007322093
C	T	514.7	82.05	0.0003	3408	3.97E-10	0.011414732
A	G	402.2	71.9	0.0003	3408	2.39E-08	0.00909824
T	G	285.3	47.21	0.0007	3408	1.66E-09	0.010602463
A	G	267.4	57.73	0.0006	3408	3.75E-06	0.00625597
T	C	216.5	40.13	0.0013	3408	7.27E-08	0.008468073
T	C	87.63	15.24	0.0076	3408	9.60E-09	0.009608224
T	G	465.5	88.9	0.0003	3408	1.74E-07	0.007980981
T	C	1065	176.6	0.0001	3408	1.78E-09	0.010558647
A	G	121.8	24.04	0.0032	3408	4.27E-07	0.007475962
C	T	151.8	24.76	0.0028	3408	9.76E-10	0.010908851
A	T	195.7	35.46	0.0015	3408	3.69E-08	0.008858097
T	C	161.7	26.59	0.0025	3408	1.33E-09	0.010734862
T	G	564.5	94.08	0.0003	3408	2.18E-09	0.010453695
A	G	116.2	21.55	0.0043	3408	7.51E-08	0.008459189
C	A	416.3	43.68	0.0009	3408	2.82E-21	0.025961169
T	C	337.2	53.66	0.0007	3408	3.73E-10	0.011454363
C	T	-177.9	33.85	0.9984	3408	1.57E-07	0.008039506
G	T	654.7	114.1	0.0003	3408	1.06E-08	0.009568378
A	G	262.2	58.71	0.0004	3408	8.24E-06	0.00581845
A	G	230.6	48.53	0.0007	3408	2.09E-06	0.006581587
T	C	270.5	39.98	0.0013	3408	1.55E-11	0.013254234
A	G	210.2	30.1	0.0019	3408	3.44E-12	0.014107896
A	C	116.2	25.75	0.0026	3408	6.57E-06	0.005939787
G	A	118.8	16.87	0.0057	3408	2.27E-12	0.01434264
A	G	117.3	22.14	0.0035	3408	1.25E-07	0.008169186
T	C	-397.5	42.74	0.9991	3408	2.40E-20	0.024752579
T	C	317.4	43.13	0.0009	3408	2.34E-13	0.015642572
A	G	145.9	28.52	0.0022	3408	3.34E-07	0.007620609

G	A	262.3	44.92	0.0009	3408	5.71E-09	0.009905901
G	A	201.6	42.28	0.0012	3408	1.95E-06	0.006627104
C	T	647.9	98.9	0.0001	3408	6.57E-11	0.012436235
C	T	319.5	53.02	0.0006	3408	1.87E-09	0.01054289
T	C	37.39	6.868	0.0396	3408	5.59E-08	0.008621646
C	G	363.2	60.02	0.0004	3408	1.59E-09	0.01063062
G	A	294.5	59	0.0004	3408	6.28E-07	0.007257775
A	G	613.8	90.65	0.0001	3408	1.50E-11	0.013274402
C	T	136.9	30.24	0.0022	3408	6.23E-06	0.005977779
T	C	113	23.6	0.0032	3408	1.75E-06	0.006682231
C	A	111.4	24.93	0.0029	3408	8.10E-06	0.005824909
T	C	81.67	18.28	0.0053	3408	8.20E-06	0.005822868
T	C	88.88	15.94	0.0073	3408	2.66E-08	0.009040404
G	A	265.7	50.39	0.0007	3408	1.42E-07	0.008092191
C	T	257.3	47.25	0.0007	3408	5.52E-08	0.008626087
A	G	180	33.61	0.0019	3408	9.07E-08	0.008345813
C	T	538.9	73.99	0.0003	3408	4.03E-13	0.015327195
C	T	135.1	26.31	0.0026	3408	3.00E-07	0.007677543
A	C	472.2	70.2	0.0004	3408	2.03E-11	0.013102382
C	A	120.1	25.31	0.0029	3408	2.16E-06	0.006563602
G	T	278.5	40.8	0.0012	3408	1.04E-11	0.01348755
T	C	351.9	58.23	0.0006	3408	1.68E-09	0.010602691
T	C	371.3	81.55	0.0003	3408	5.48E-06	0.006046007
T	C	11.73	2.646	0.5461	3408	9.60E-06	0.005733497
G	C	285.3	39.59	0.0012	3408	7.09E-13	0.015009458
C	T	358.8	56.41	0.0004	3408	2.28E-10	0.011731887
T	G	304	55.65	0.0006	3408	5.06E-08	0.008680226
G	C	98.82	20.87	0.0041	3408	2.29E-06	0.00653578
T	A	-294.6	50.73	0.9994	3408	6.96E-09	0.009798504
A	C	150.9	27.91	0.0021	3408	6.90E-08	0.008504523
G	A	84.27	15.32	0.0075	3408	4.04E-08	0.00880015
C	G	166.2	37.14	0.0013	3408	7.91E-06	0.005841632
C	A	462.3	82.97	0.0003	3408	2.71E-08	0.009027503
T	C	174.4	34.67	0.0013	3408	5.20E-07	0.007370094
T	C	345.8	57.03	0.0006	3408	1.48E-09	0.010672927
T	C	557.7	103	0.0003	3408	6.61E-08	0.008529178
A	G	82	17.26	0.0057	3408	2.09E-06	0.006579301
A	T	52.6	11.15	0.0144	3408	2.49E-06	0.006487769
T	C	87.78	18.78	0.0051	3408	3.06E-06	0.006369791
A	G	32.04	7.097	0.0367	3408	6.55E-06	0.005944923
T	C	573.2	128.4	0.0001	3408	8.37E-06	0.005813672
G	A	263.2	53.88	0.0007	3408	1.09E-06	0.006953232
T	C	16.73	3.78	0.0016	3425	9.90E-06	0.005686838
T	G	55.35	10.46	0.0001	3425	1.31E-07	0.008109147
T	C	14.12	3.162	0.0023	3425	8.29E-06	0.005788471
A	G	20.89	4.337	0.001	3425	1.52E-06	0.006728303
T	G	17.93	3.806	0.0013	3425	2.55E-06	0.006438095
T	A	3.019	0.652	0.058	3425	3.71E-06	0.006230494

G	A	20.96	3.949	0.0013	3425	1.18E-07	0.008158121
C	T	53.93	8.721	0.0003	3425	6.98E-10	0.011041945
C	T	18.44	3.073	0.0022	3425	2.16E-09	0.010403851
C	A	-1.704	0.307	0.541	3425	3.08E-08	0.008909072
G	A	3.541	0.318	0.3523	3425	2.91E-28	0.03487402
A	T	15.46	3.422	0.0019	3425	6.51E-06	0.00592404
A	G	1.892	0.414	0.1546	3425	5.00E-06	0.006066775
C	A	7.293	1.623	0.0092	3425	7.22E-06	0.005860869
A	G	19.06	3.981	0.0016	3425	1.76E-06	0.006648198
A	G	11.97	2.464	0.0035	3425	1.23E-06	0.006843277
T	C	37.63	8.283	0.0004	3425	5.73E-06	0.005989952
T	C	6.375	1.362	0.012	3425	2.95E-06	0.006355891
T	C	56.58	11.01	0.0001	3425	2.91E-07	0.007651645
C	T	18.5	3.684	0.0018	3425	5.37E-07	0.007308996
C	T	7.629	1.661	0.0082	3425	4.55E-06	0.006121652
T	C	1.524	0.321	0.3556	3425	2.12E-06	0.006550242
A	G	26.75	5.484	0.0009	3425	1.12E-06	0.00689899
A	G	2.545	0.413	0.2185	3425	7.93E-10	0.010970701
C	T	-2.715	0.473	0.2372	3425	1.03E-08	0.009527941
C	T	2.095	0.367	0.6474	3425	1.26E-08	0.009414432
T	C	-2.744	0.591	0.1168	3425	3.52E-06	0.006261033
G	A	19.53	4.148	0.0012	3425	2.59E-06	0.006430793
A	G	2.722	0.584	0.0762	3425	3.28E-06	0.006302952
T	C	36.99	7.317	0.0004	3425	4.54E-07	0.007406506
T	C	47.63	9.619	0.0003	3425	7.71E-07	0.007107924
G	T	1.525	0.315	0.3676	3425	1.37E-06	0.00678383
G	A	-1.512	0.306	0.4593	3425	8.14E-07	0.007078077
C	A	38.41	8.177	0.0003	3425	2.74E-06	0.006401049
G	C	5.753	1.259	0.0153	3425	5.04E-06	0.006059504
T	A	18.12	4.089	0.0013	3425	9.62E-06	0.005700838
G	T	2.142	0.472	0.1194	3425	5.86E-06	0.005977103
T	C	14.88	2.692	0.0029	3425	3.49E-08	0.008841749
G	A	28.75	5.206	0.0009	3425	3.59E-08	0.008825854
G	A	8.664	1.937	0.0057	3425	8.01E-06	0.005807478
C	A	26.26	5.048	0.0009	3425	2.10E-07	0.007839209
A	G	26.32	4.629	0.001	3425	1.42E-08	0.00935097
T	C	28.31	6.128	0.0007	3425	4.00E-06	0.006192754
A	T	19.65	4.049	0.0015	3425	1.27E-06	0.006829561
A	G	27.72	5.936	0.0006	3425	3.13E-06	0.006326765
T	C	5.121	1.023	0.0218	3425	5.91E-07	0.007263264
C	T	44.86	8.442	0.0003	3425	1.14E-07	0.008177139
A	T	10.34	2.307	0.0041	3425	7.57E-06	0.005831033
T	C	7.798	1.675	0.0076	3425	3.36E-06	0.006288349
G	C	4.978	1.076	0.0197	3425	3.87E-06	0.006210393
A	C	36.89	8.022	0.0004	3425	4.41E-06	0.006136464
G	A	5.131	1.11	0.0187	3425	3.94E-06	0.006200068
A	C	14.23	3.197	0.002	3425	8.77E-06	0.005751207
C	G	-1.811	0.376	0.2091	3425	1.53E-06	0.006720633

C	A	13.74	2.735	0.0004	3425	5.33E-07	0.007314922
G	A	22.03	4.696	0.0001	3425	2.82E-06	0.006384554
A	G	4.375	0.843	0.0058	3425	2.23E-07	0.007800748
T	C	6.421	1.291	0.0023	3425	6.91E-07	0.007170787
T	C	16.8	3.715	0.0003	3425	6.34E-06	0.005935467
T	G	25.81	4.27	0.0001	3425	1.66E-09	0.010554834
A	G	4.653	0.973	0.0042	3425	1.80E-06	0.0066354
T	C	11.36	1.879	0.001	3425	1.67E-09	0.010559232
T	C	7.798	1.543	0.0016	3425	4.56E-07	0.007401972
C	T	31.35	4.91	0.0001	3425	1.95E-10	0.011762854
G	C	8.253	1.737	0.0012	3425	2.11E-06	0.006548025
T	C	6.026	1.334	0.0025	3425	6.45E-06	0.005922516
C	G	13.39	2.614	0.0006	3425	3.18E-07	0.007602824
C	T	4.735	0.917	0.0042	3425	2.57E-07	0.007721204
T	A	30.28	5.61	0.0001	3425	7.23E-08	0.008434259
T	C	11.07	2.327	0.0007	3425	2.04E-06	0.006564199
A	G	14.77	3.33	0.0003	3425	9.46E-06	0.005711162
A	C	47.98	7.59	0.0001	3425	2.92E-10	0.011532901
T	C	5.7	1.265	0.0023	3425	6.88E-06	0.005893064
G	A	1.152	0.131	0.3523	3425	2.08E-18	0.022113304
A	G	9.164	2.054	0.0009	3425	8.39E-06	0.005778192
A	G	9.757	1.738	0.0015	3425	2.15E-08	0.009117893
G	T	10.56	1.865	0.0012	3425	1.64E-08	0.009273917
A	G	7.12	1.607	0.0015	3425	9.72E-06	0.005698829
A	G	16.19	3.137	0.0004	3425	2.58E-07	0.007716844
A	G	13.87	2.883	0.0004	3425	1.58E-06	0.006712404
C	A	7.62	1.554	0.0013	3425	9.90E-07	0.006971226
G	A	5.448	1.215	0.0025	3425	7.54E-06	0.005836051
T	C	18.09	3.474	0.001	3425	2.02E-07	0.007854747
G	A	11.97	2.528	0.0006	3425	2.28E-06	0.006503393
C	T	32	3.541	0.0003	3425	2.61E-19	0.023289145
A	G	24.63	3.673	0.0003	3425	2.34E-11	0.012958708
A	G	1.581	0.34	0.0365	3425	3.49E-06	0.006262546
G	A	7.789	1.615	0.0013	3425	1.47E-06	0.006745564
C	T	6.75	1.257	0.0022	3425	8.36E-08	0.008349015
A	T	8.79	1.394	0.0019	3425	3.28E-10	0.011475695
T	C	6.561	1.381	0.0016	3425	2.10E-06	0.006546957
T	C	6.616	1.473	0.0019	3425	7.35E-06	0.00585564
T	C	8.58	1.833	0.0012	3425	2.96E-06	0.006356523
G	C	8.464	1.614	0.0016	3425	1.65E-07	0.007965453
G	T	21.84	4.516	0.0001	3425	1.39E-06	0.006782366
A	C	22.84	3.766	0.0003	3425	1.47E-09	0.010625072
G	A	13.13	2.827	0.0004	3425	3.56E-06	0.006258793
G	A	5.996	1.149	0.0028	3425	1.90E-07	0.007888296
A	G	29.68	4.936	0.0001	3425	2.03E-09	0.010446152
C	A	30.79	6.136	0.0003	3425	5.48E-07	0.007298052
A	G	4.697	1.007	0.0035	3425	3.21E-06	0.006312067
C	T	8.792	1.951	0.0013	3425	6.77E-06	0.005894308

A	G	10.52	2.32	0.0007	3425	6.02E-06	0.005967542
G	A	6.353	1.049	0.0032	3425	1.52E-09	0.010595466
T	C	7.387	1.446	0.0018	3425	3.40E-07	0.007562099
T	C	6.293	1.218	0.0025	3425	2.55E-07	0.007733722
C	T	13.52	2.709	0.0006	3425	6.27E-07	0.007219845
A	C	5.388	0.895	0.0047	3425	1.95E-09	0.010463793
G	C	3.612	0.768	0.0064	3425	2.66E-06	0.006418434
T	G	33.78	6.59	0.0001	3425	3.13E-07	0.007613228
T	A	15.77	2.724	0.0004	3425	7.76E-09	0.009690795
A	G	13.87	2.719	0.0006	3425	3.55E-07	0.007540269
C	T	3.013	0.679	0.0082	3425	9.39E-06	0.005716214
T	C	-3.984	0.777	0.9939	3425	3.05E-07	0.007625336
G	T	4.893	1.054	0.0038	3425	3.59E-06	0.006252943
A	G	9.284	1.98	0.001	3425	2.85E-06	0.006378233
T	C	30.16	4.486	0.0001	3425	2.09E-11	0.013025355
C	T	10.98	1.499	0.0018	3425	2.98E-13	0.015423758
T	C	3.584	0.798	0.0058	3425	7.29E-06	0.005859278
C	T	9.727	1.858	0.0012	3425	1.76E-07	0.007938609
A	G	13.93	2.882	0.0004	3425	1.39E-06	0.006774877
T	A	-0.7609	0.164	0.7495	3425	3.54E-06	0.006260935
C	T	-1.08	0.204	0.1839	3425	1.30E-07	0.008108955
A	G	2.859	0.638	0.0095	3425	7.64E-06	0.005832547
A	G	2.046	0.46	0.0182	3425	8.82E-06	0.005752876
A	G	1.086	0.169	0.2185	3425	1.37E-10	0.011968908
A	G	11.66	2.24	0.0009	3425	2.03E-07	0.007849071
A	G	15.07	2.976	0.0006	3425	4.35E-07	0.00743123
G	C	-1.022	0.229	0.127	3425	8.45E-06	0.005776636
A	G	18.92	3.324	0.0003	3425	1.37E-08	0.009370674
C	T	16.2	3.135	0.0003	3425	2.51E-07	0.007736091
A	G	12.24	2.632	0.0006	3425	3.46E-06	0.006274756
T	C	17.94	3.35	0.0003	3425	9.19E-08	0.008303733
C	G	43.28	6.761	0.0001	3425	1.74E-10	0.011822976
T	A	0.889	0.199	0.1067	3425	7.81E-06	0.005816357
A	G	10.19	2.094	0.0009	3425	1.19E-06	0.006866604
A	G	2.257	0.46	0.0191	3425	9.89E-07	0.006973808
T	C	9.254	1.615	0.0015	3425	1.08E-08	0.009495326
T	C	29.98	3.911	0.0003	3425	2.31E-14	0.016867056
T	C	10.43	2.108	0.0009	3425	7.76E-07	0.007096981
T	C	20.95	2.979	0.0004	3425	2.46E-12	0.014234431
C	T	1.209	0.234	0.0791	3425	2.36E-07	0.007766621
A	T	6.916	1.41	0.0022	3425	9.75E-07	0.006975433
T	C	12.6	2.429	0.0006	3425	2.25E-07	0.007795192
T	C	9.628	1.921	0.001	3425	5.66E-07	0.00728087
C	T	0.9898	0.206	0.1001	3425	1.52E-06	0.00672789
A	G	6.679	1.483	0.0016	3425	6.91E-06	0.005887293
T	C	4.342	0.935	0.0042	3425	3.49E-06	0.006263709
A	C	2.251	0.494	0.0165	3425	5.40E-06	0.006023333
A	C	2.78	0.542	0.0136	3425	3.13E-07	0.007614306

G	A	15.43	2.985	0.0004	3425	2.49E-07	0.007741187
T	C	3.108	0.696	0.008	3425	8.22E-06	0.005790097
T	G	2.799	0.603	0.0108	3425	3.52E-06	0.006261856
T	A	5.278	1.026	0.0035	3425	2.82E-07	0.007667271
A	G	15.24	2.947	0.0006	3425	2.47E-07	0.007747672
G	A	16.44	2.719	0.0004	3425	1.62E-09	0.010561207
T	C	1.416	0.305	0.0438	3425	3.44E-06	0.0062701
A	G	4.44	0.744	0.0072	3425	2.66E-09	0.010288484
T	C	3.801	0.807	0.0063	3425	2.54E-06	0.006443458
C	G	14.72	2.717	0.0004	3425	6.52E-08	0.008497081
G	A	13.88	2.845	0.0009	3425	1.12E-06	0.006901542
T	A	22.92	4.832	0.0001	3425	2.18E-06	0.006526362
T	A	15.12	2.825	0.0004	3425	9.28E-08	0.008294474
T	C	1.133	0.245	0.0695	3425	3.70E-06	0.006230561
A	G	10.25	2.165	0.0009	3425	2.29E-06	0.006501872
C	T	20.33	3.685	0.0004	3425	3.68E-08	0.008808396
C	T	3.203	0.688	0.0086	3425	3.35E-06	0.006290168
A	G	23.77	3.62	0.0003	3425	5.93E-11	0.012432188
C	G	8.455	1.683	0.0013	3425	5.34E-07	0.007314926
T	A	2.932	0.613	0.0098	3425	1.81E-06	0.006630919
T	C	2.408	0.513	0.0147	3425	2.81E-06	0.006389466
A	G	16.76	3.638	0.0003	3425	4.25E-06	0.00615856
C	G	19.12	3.159	0.0004	3425	1.56E-09	0.010582675
C	T	15.01	3.391	0.0003	3425	9.91E-06	0.005688108
A	G	6.448	1.387	0.0022	3425	3.49E-06	0.006270537
T	G	15.84	2.869	0.0006	3425	3.65E-08	0.008821462
T	C	4.694	1.016	0.0039	3425	4.02E-06	0.006193555
G	T	0.7447	0.16	0.1829	3425	3.32E-06	0.006293084
C	T	25.3	4.669	0.0001	3425	6.39E-08	0.008500126
C	A	20.46	3.333	0.0003	3425	9.41E-10	0.010882482
C	T	-10.71	2.24	0.9993	3425	1.81E-06	0.006630301
A	G	26.26	4.684	0.0001	3425	2.22E-08	0.009093432
G	T	0.8644	0.193	0.1194	3425	7.65E-06	0.00582862
T	C	14.98	3.003	0.0004	3425	6.36E-07	0.007212877
A	T	8.626	1.673	0.0015	3425	2.68E-07	0.007702089
T	C	11.33	2.443	0.0007	3425	3.68E-06	0.006240703
G	A	9.584	2.16	0.0007	3425	9.40E-06	0.005715264
A	G	18.44	3.292	0.0003	3425	2.28E-08	0.009077811
T	C	16.93	3.298	0.0003	3425	2.99E-07	0.007635252
T	C	7.048	1.098	0.0029	3425	1.57E-10	0.011887024
T	G	2.792	0.626	0.0098	3425	8.41E-06	0.005779902
T	C	6.283	1.4	0.0022	3425	7.42E-06	0.005846165
C	T	1.114	0.248	0.0654	3425	7.53E-06	0.005842672
G	A	11.92	2.463	0.0006	3425	1.37E-06	0.006792088
C	G	9.445	1.903	0.001	3425	7.26E-07	0.007140907
T	C	3.316	0.713	0.0076	3425	3.40E-06	0.006280853
G	A	19.07	3.318	0.0003	3425	9.78E-09	0.009552554
T	C	52.08	8.178	0.0001	3425	2.17E-10	0.011702399

T	G	22.24	4.12	0.0003	3425	7.16E-08	0.008435976
C	T	20.97	3.305	0.0003	3425	2.50E-10	0.011617651
A	G	14.23	2.741	0.0006	3425	2.22E-07	0.007807769
C	T	12.27	2.63	0.0006	3425	3.19E-06	0.006314891
G	C	4.227	0.859	0.0048	3425	9.01E-07	0.00702196
G	A	4.577	0.79	0.0057	3425	7.53E-09	0.009705357
A	G	4.857	1.009	0.0042	3425	1.53E-06	0.006719934
G	A	18.3	3.188	0.0003	3425	1.04E-08	0.009528987
C	G	15.42	2.675	0.0004	3425	8.83E-09	0.009608761
T	C	8.949	1.874	0.0013	3425	1.87E-06	0.006614047
A	G	1.889	0.395	0.0239	3425	1.80E-06	0.006633135
T	C	13.59	2.627	0.0006	3425	2.42E-07	0.007753145
T	C	12.63	2.018	0.0009	3425	4.39E-10	0.01130746
C	T	8.956	1.997	0.0009	3425	7.57E-06	0.005838061
G	C	12.74	2.693	0.0004	3425	2.34E-06	0.006491977
A	G	4.355	0.956	0.0039	3425	5.37E-06	0.006027508
G	C	29.78	4.668	0.0001	3425	2.03E-10	0.01174348
A	C	-0.5485	0.123	0.5428	3425	9.09E-06	0.005735408
G	A	6.053	1.152	0.0026	3425	1.59E-07	0.0079963
C	T	22.19	4.608	0.0001	3425	1.52E-06	0.006725097
T	C	2.358	0.526	0.0134	3425	7.68E-06	0.005826696
A	G	12.78	2.423	0.0006	3425	1.43E-07	0.008057141
T	C	27.16	5.163	0.0001	3425	1.53E-07	0.008014932
A	T	4.292	0.943	0.0041	3425	5.44E-06	0.006018309
T	C	3.575	0.749	0.0073	3425	1.87E-06	0.006611182
A	G	7.225	1.576	0.0019	3425	4.74E-06	0.006098821
C	T	28.44	3.429	0.0003	3425	1.57E-16	0.019689171
A	G	19.35	4.228	0.0001	3425	4.88E-06	0.00607832
A	G	7.137	1.611	0.0015	3425	9.68E-06	0.005697682
A	C	24.71	3.261	0.0004	3425	4.54E-14	0.016487813
T	C	18.46	3.785	0.0003	3425	1.12E-06	0.006897081
G	C	5.101	1.095	0.0032	3425	3.31E-06	0.006296204
G	T	32.49	6.401	0.0001	3425	4.07E-07	0.00746601
T	C	7.394	1.479	0.0018	3425	6.08E-07	0.007244431
G	A	2.216	0.453	0.0187	3425	1.07E-06	0.006926238
A	C	5.807	1.306	0.002	3425	9.02E-06	0.005739275
T	A	5.853	1.299	0.0023	3425	6.87E-06	0.005892661
C	T	3.936	0.851	0.0058	3425	3.91E-06	0.006202719
T	G	4.189	0.888	0.0047	3425	2.45E-06	0.006461159
C	T	111.1	23.73	0.0054	3408	2.93E-06	0.006390704
A	C	90.6	19.08	0.0088	3408	2.13E-06	0.006572585
A	G	210.4	38.51	0.0026	3408	5.03E-08	0.008682754
G	C	267.8	37.95	0.0021	3408	2.07E-12	0.014401183
C	T	465.8	51.16	0.001	3408	1.42E-19	0.02374657
C	G	617.1	61.82	0.0007	3408	3.76E-23	0.028407795
T	C	210.7	35.78	0.0025	3408	4.25E-09	0.010072843
G	A	144.8	30.72	0.0031	3408	2.54E-06	0.006476987
G	A	301	43.5	0.0016	3408	5.38E-12	0.013854658

T	G	312.5	44.32	0.0016	3408	2.16E-12	0.01437842
C	G	585.1	59.78	0.0007	3408	2.53E-22	0.027340694
C	T	360.9	45.09	0.0015	3408	1.62E-15	0.018451241
A	G	2896	127.1	0.0001	3408	5.00E-107	0.132198578
T	C	180.5	34.62	0.0023	3408	1.95E-07	0.007913168
T	C	794.2	70	0.0006	3408	2.61E-29	0.03639673
C	T	104.8	18.61	0.0095	3408	1.97E-08	0.009219507
T	C	68.03	14.85	0.0144	3408	4.77E-06	0.006120434
T	C	2073	128.3	0.0001	3408	1.15E-56	0.071152494
T	C	139.3	25.24	0.0059	3408	3.67E-08	0.008858488
A	G	342.3	48.3	0.0019	3408	1.66E-12	0.014523333
A	G	713.2	134.1	0.0001	3408	1.13E-07	0.008231441
A	G	324.4	37.63	0.0023	3408	1.01E-17	0.021341495
G	C	90.64	18.38	0.0092	3408	8.54E-07	0.007085347
C	G	648.7	61.14	0.0013	3408	6.82E-26	0.031975952
A	G	317.1	49.08	0.0013	3408	1.19E-10	0.012100314
G	C	621.6	61.6	0.0007	3408	1.28E-23	0.029011818
C	T	158.3	32.6	0.0028	3408	1.25E-06	0.0068712
A	G	731.4	69.14	0.0006	3408	9.38E-26	0.03179217
C	T	76.42	11.29	0.0251	3408	1.55E-11	0.013265599
A	C	106.8	19.44	0.0088	3408	4.27E-08	0.008778516
C	G	701.2	76.62	0.0004	3408	9.39E-20	0.0239859
A	G	287	50.66	0.0013	3408	1.60E-08	0.0093296
G	A	96.72	20.51	0.0076	3408	2.51E-06	0.006483014
T	A	86.06	17.26	0.0141	3408	6.46E-07	0.007242106
T	C	153.3	28.67	0.0038	3408	9.51E-08	0.008319574
A	G	1256	106.9	0.0004	3408	2.73E-31	0.038929561
A	G	234.6	39.65	0.0022	3408	3.60E-09	0.010167907
T	C	96.48	21.61	0.007	3408	8.29E-06	0.005814773
T	C	90.15	17.28	0.0114	3408	1.93E-07	0.007923002
A	G	69.27	13.49	0.0183	3408	2.97E-07	0.007677498
T	C	270.7	41.43	0.0018	3408	7.32E-11	0.012372015
A	G	51.21	10.5	0.0314	3408	1.12E-06	0.006931236
C	G	734.3	67.86	0.0006	3408	7.51E-27	0.033216139
A	G	166.6	29.17	0.0035	3408	1.22E-08	0.009480702
A	G	262.1	38.78	0.0021	3408	1.63E-11	0.013226241
C	A	297	38.79	0.0022	3408	2.47E-14	0.016910897
T	C	605.2	62.86	0.0007	3408	1.15E-21	0.026478599
T	C	118.6	22.12	0.007	3408	8.80E-08	0.008364722
A	G	381.7	53.18	0.0009	3408	8.73E-13	0.014891287
A	G	636.7	65.61	0.0009	3408	5.51E-22	0.026890071
G	A	77.42	16.15	0.0122	3408	1.71E-06	0.006697967
G	C	36.06	7.292	0.0697	3408	7.94E-07	0.007124483
A	G	43.94	7.987	0.0565	3408	4.03E-08	0.008802647
G	A	264	40.5	0.0018	3408	8.16E-11	0.012314503
A	G	200.7	32.7	0.0034	3408	9.35E-10	0.010932652
G	A	189.8	29.08	0.0037	3408	7.67E-11	0.012345511
C	T	134.6	22.06	0.0063	3408	1.16E-09	0.010805902

C	A	216.5	35.34	0.0026	3408	1.00E-09	0.010892476
A	G	188.1	41.28	0.0021	3408	5.37E-06	0.006055649
G	A	244	36.89	0.0021	3408	4.30E-11	0.012674277
C	T	143.9	26.11	0.005	3408	3.78E-08	0.008833945
T	A	128.9	27.43	0.004	3408	2.69E-06	0.00643798
C	T	139.9	28.75	0.0043	3408	1.19E-06	0.006900064
G	C	271.6	45.23	0.0015	3408	2.11E-09	0.010469739
G	C	156.4	29.26	0.0035	3408	9.60E-08	0.008313799
T	C	257.4	36.09	0.0022	3408	1.20E-12	0.014706501
C	T	331.8	38.87	0.0022	3408	2.06E-17	0.020933238
C	A	282.9	37.84	0.0021	3408	9.60E-14	0.016136118
G	A	138.4	29.65	0.0037	3408	3.14E-06	0.006352658
A	G	206.8	32.42	0.0026	3408	2.03E-10	0.011798344
G	C	505.9	59.97	0.0009	3408	4.85E-17	0.020454381
T	C	230.6	33.78	0.0026	3408	1.02E-11	0.013489671
C	T	1006	184.8	0.0001	3408	5.60E-08	0.008620498
G	A	1581	93.65	0.0003	3408	1.69E-61	0.077173574
A	G	158.9	28.76	0.0035	3408	3.53E-08	0.008877644
G	T	419.8	54.08	0.0013	3408	1.09E-14	0.017374014
A	G	-87.22	15.38	0.9869	3408	1.55E-08	0.009348476
T	G	127.2	27.45	0.0044	3408	3.76E-06	0.006261266
T	C	163.3	20.92	0.0073	3408	7.76E-15	0.017565202
A	G	133.6	27.52	0.0041	3408	1.26E-06	0.006867893
G	T	165.9	34.19	0.0032	3408	1.27E-06	0.006861276
G	A	1003	98.17	0.0003	3408	3.69E-24	0.029719533
C	T	221.7	49.64	0.0012	3408	8.25E-06	0.005818805
A	T	62.04	11.45	0.0246	3408	6.49E-08	0.008540982
T	C	194	37.12	0.0022	3408	1.85E-07	0.007950987
A	T	175.7	32.77	0.0031	3408	8.77E-08	0.00836456
T	C	737.2	135.8	0.0001	3408	6.09E-08	0.008572989
T	A	749.7	84.69	0.0006	3408	1.35E-18	0.02247699
T	G	181.6	22.52	0.0056	3408	1.00E-15	0.018723487
G	A	132.3	29.04	0.0038	3408	5.43E-06	0.006053269
T	C	413.3	62.46	0.0007	3408	4.25E-11	0.012684785
T	C	353	46.68	0.0013	3408	5.07E-14	0.016502935
G	A	98.72	17.57	0.0113	3408	2.08E-08	0.009178309
C	A	366	46.88	0.0018	3408	7.76E-15	0.017570685
T	C	141.4	26.91	0.0047	3408	1.58E-07	0.008036511
T	C	330.2	60.13	0.0012	3408	4.27E-08	0.008770941
T	C	210.6	33.76	0.0026	3408	4.98E-10	0.011289672
A	G	100.5	20.04	0.0078	3408	5.55E-07	0.007325612
C	T	67.88	13.44	0.0179	3408	4.64E-07	0.007429285
T	C	203	35.7	0.0029	3408	1.41E-08	0.009398425
A	G	155.1	28.13	0.0038	3408	3.76E-08	0.008841534
T	G	80.23	16.2	0.0128	3408	7.70E-07	0.00714545
T	A	363.6	48.88	0.001	3408	1.28E-13	0.015976847
T	A	56.57	11.97	0.0223	3408	2.39E-06	0.006510998
T	G	173.7	33.85	0.0031	3408	3.05E-07	0.007667257

C	T	207.3	38.94	0.0021	3408	1.09E-07	0.008247276
C	G	332.2	53.59	0.001	3408	6.35E-10	0.011149688
A	T	76.74	12.02	0.0216	3408	1.93E-10	0.011818754
T	C	55.61	12.37	0.0222	3408	7.16E-06	0.005895206
T	C	117.2	21.64	0.0065	3408	6.57E-08	0.008533344
A	G	124	27.54	0.0041	3408	6.97E-06	0.005913439
A	G	132.5	28.42	0.0041	3408	3.21E-06	0.006337572
A	G	658.4	82.54	0.0004	3408	2.02E-15	0.018328102
C	A	157.8	30.21	0.0034	3408	1.87E-07	0.007942365
G	T	353.7	39.81	0.0019	3408	1.02E-18	0.022638182
C	G	320.5	56.26	0.001	3408	1.33E-08	0.009432807
C	T	177.5	30.9	0.0034	3408	1.01E-08	0.009589488
A	C	199.4	34.68	0.0026	3408	9.74E-09	0.00960727
C	T	568.9	78.84	0.0004	3408	6.60E-13	0.015048523
C	T	100.7	21.16	0.0062	3408	2.00E-06	0.006601634
A	G	896.2	97.49	0.0003	3408	6.49E-20	0.024196499
C	T	103.4	22.87	0.0063	3408	6.30E-06	0.005962275
T	C	73.79	16.23	0.012	3408	5.64E-06	0.006028818
C	T	562.4	67.24	0.0007	3408	8.73E-17	0.020114578
T	G	461.6	100.6	0.0004	3408	4.60E-06	0.0061399
C	G	135.8	27.93	0.0043	3408	1.20E-06	0.006888995
T	C	166.4	27.44	0.0041	3408	1.46E-09	0.010675242
C	T	292.3	40.49	0.0019	3408	6.43E-13	0.015061615
A	C	278.8	42.64	0.0018	3408	7.23E-11	0.012389035
T	C	320.1	47.71	0.0012	3408	2.29E-11	0.013036293
A	G	125.6	28.14	0.0044	3408	8.39E-06	0.00581166
C	G	160	29.14	0.0037	3408	4.31E-08	0.008768722
G	A	-287.7	48.86	0.9987	3408	4.25E-09	0.010071107
T	A	225.4	39.21	0.0022	3408	9.72E-09	0.009603371
A	G	2073	128.5	0.0001	3408	1.74E-56	0.070946881
A	G	632.9	75.09	0.0004	3408	5.07E-17	0.020419566
G	A	225.9	42.94	0.0015	3408	1.52E-07	0.008055567
A	C	48.23	9.695	0.0343	3408	6.86E-07	0.007209364
T	C	313.5	41.76	0.0018	3408	7.66E-14	0.016267895
C	T	139.1	30.1	0.0034	3408	3.94E-06	0.006227428
A	C	207.9	36.33	0.0023	3408	1.15E-08	0.009517555
T	G	420	62.49	0.0012	3408	2.10E-11	0.01308155
T	C	35.49	7.68	0.0585	3408	3.97E-06	0.006226976
A	C	961.3	93.43	0.0003	3408	1.80E-24	0.030127308
C	T	212.5	36.29	0.0023	3408	5.23E-09	0.009960857
A	G	139.5	25.13	0.0054	3408	3.08E-08	0.008960962
G	A	358.8	54.73	0.0009	3408	6.39E-11	0.012454081
A	G	179.7	36.1	0.0028	3408	6.78E-07	0.007218316
T	C	2047	127.9	0.0001	3408	1.21E-55	0.069907108
C	G	89.63	19.49	0.0095	3408	4.40E-06	0.006167325
A	T	80.23	17.28	0.0103	3408	3.55E-06	0.00628562
T	G	140	31.59	0.0032	3408	9.69E-06	0.005730091
G	A	169.7	32.81	0.0031	3408	2.46E-07	0.007788537

A	T	142.6	31.72	0.0028	3408	7.13E-06	0.005895291
G	A	759.4	70.06	0.0006	3408	6.21E-27	0.033325865
A	C	548.1	67.17	0.0006	3408	4.65E-16	0.019163115
T	C	212.6	36.32	0.0022	3408	5.30E-09	0.009953841
A	T	246.7	37.5	0.0019	3408	5.47E-11	0.012539947
T	C	126.7	26.42	0.0051	3408	1.71E-06	0.006702968
A	G	255.1	29.73	0.0032	3408	1.40E-17	0.021147011
T	C	274.8	40.08	0.0018	3408	8.39E-12	0.013605951
T	A	221	34.9	0.0023	3408	2.75E-10	0.01162931
T	G	212.9	34.36	0.0023	3408	6.51E-10	0.011139874
A	G	184.3	40.11	0.0018	3408	4.50E-06	0.006156921
G	A	237.6	38.71	0.0021	3408	9.39E-10	0.010933829
T	C	160.1	23.53	0.0054	3408	1.21E-11	0.0134023
A	G	235.6	38.25	0.0028	3408	8.14E-10	0.011009817
C	T	196.5	29.94	0.0035	3408	6.06E-11	0.012481511
G	A	571.9	61.21	0.0009	3408	1.66E-20	0.024975342
C	T	570.9	70.25	0.0006	3408	6.12E-16	0.019010443
T	C	225.9	40.82	0.0018	3408	3.37E-08	0.008906387
G	A	428.3	59.76	0.0009	3408	9.36E-13	0.014848359
C	T	156.3	23.78	0.0065	3408	5.65E-11	0.012517684
A	G	115.6	25.99	0.0044	3408	8.88E-06	0.005771514
A	G	887.3	70.63	0.0006	3408	2.03E-35	0.044259154
G	C	169.8	31.47	0.0034	3408	7.29E-08	0.008470092
A	G	623.6	88.02	0.0003	3408	1.69E-12	0.014514436
T	C	41.07	8.012	0.0531	3408	3.12E-07	0.007651248
G	A	84.59	13.57	0.0185	3408	5.08E-10	0.011273402
T	C	101.3	20.12	0.0081	3408	5.01E-07	0.007383206
A	G	889.9	73.93	0.0006	3408	1.04E-32	0.040781119
C	A	338.9	40.47	0.0018	3408	7.89E-17	0.0201619
A	G	166.2	33.27	0.0031	3408	6.20E-07	0.007269229
C	T	261.1	52.46	0.0013	3408	6.74E-07	0.007216261
A	G	155.7	28.74	0.0041	3408	6.49E-08	0.008538468
G	A	118.7	22.96	0.0062	3408	2.48E-07	0.007781537
C	T	143.3	26.23	0.0045	3408	5.05E-08	0.00868179
G	A	725.8	66.51	0.0006	3408	2.81E-27	0.033763231
A	C	76.89	15.6	0.0139	3408	8.71E-07	0.007077927
T	C	168.6	26.99	0.0043	3408	4.71E-10	0.011320492
A	G	165.2	33.03	0.0031	3408	5.93E-07	0.007286638
A	G	257.5	44.78	0.0015	3408	9.76E-09	0.009609333
T	C	106.6	21.82	0.007	3408	1.07E-06	0.006954636
T	C	181.2	33.13	0.0032	3408	4.82E-08	0.008701187
A	T	720.8	85.31	0.0004	3408	4.27E-17	0.020517608
T	C	1584	97.29	0.0006	3408	1.79E-57	0.072167852
T	G	47.59	8.691	0.0433	3408	4.66E-08	0.008721437
A	C	915.9	94.17	0.0003	3408	4.51E-22	0.027007288
C	T	836.1	73.46	0.0004	3408	1.75E-29	0.036619537
G	A	510.5	107.2	0.0001	3408	1.98E-06	0.006610312
C	T	537	109.7	0.0001	3408	1.02E-06	0.006982208

T	C	1757	129.6	0.0001	3408	8.19E-41	0.051170774
T	C	184.8	39.46	0.0021	3408	2.93E-06	0.006394465
T	C	634.9	80.9	0.0004	3408	5.64E-15	0.017751514
C	T	460.1	50.43	0.0012	3408	1.21E-19	0.023842235
C	T	158.9	30.26	0.0034	3408	1.60E-07	0.008026211
T	C	315.7	41.99	0.0016	3408	7.06E-14	0.01631599
A	C	326.1	38.46	0.0019	3408	3.32E-17	0.020659377
G	A	1129	72.17	0.0006	3408	2.50E-53	0.06699737
A	G	1165	84.75	0.0004	3408	6.83E-42	0.052533543
A	G	240.6	38.16	0.0019	3408	3.26E-10	0.011530239
A	G	662.4	82.11	0.0004	3408	9.84E-16	0.018738453
A	G	438.9	52.07	0.0012	3408	5.06E-17	0.02042187
A	C	580.3	60.69	0.0007	3408	2.16E-21	0.026126066
A	G	312.3	54.42	0.0012	3408	1.03E-08	0.009570855
A	G	620.5	43.09	0.0013	3408	1.11E-45	0.057355981
C	T	259.2	48.24	0.0015	3408	8.24E-08	0.00840025
A	G	268.3	44.19	0.0015	3408	1.40E-09	0.010700926
A	G	262.7	44.6	0.0015	3408	4.22E-09	0.010077482
C	T	381.2	58.11	0.0012	3408	6.19E-11	0.012469668
T	C	156.9	34.64	0.0025	3408	6.12E-06	0.005983895
G	A	475.9	53.26	0.0012	3408	6.51E-19	0.022891399
T	C	114	19.49	0.0114	3408	5.42E-09	0.009939129
T	C	98.13	20.57	0.0073	3408	1.91E-06	0.006633533
A	T	164.5	33.86	0.0025	3408	1.23E-06	0.00687798
C	T	1117	84.15	0.0004	3408	3.23E-39	0.049159364
G	A	438.9	64.07	0.0007	3408	8.69E-12	0.013582598
G	A	160.9	28.33	0.0037	3408	1.47E-08	0.009376227
T	C	51.61	9.929	0.0335	3408	2.13E-07	0.007865524
T	C	102.2	22.59	0.0059	3408	6.33E-06	0.00596993
C	A	225.4	40.79	0.0018	3408	3.49E-08	0.008880286
T	A	154.4	21.25	0.007	3408	4.62E-13	0.015254609
G	T	46.84	9.534	0.0355	3408	9.43E-07	0.007032648
A	G	119.6	24.42	0.0053	3408	1.01E-06	0.006989167
G	A	193.9	36.98	0.0022	3408	1.67E-07	0.00800263
C	A	127.1	21.37	0.0072	3408	3.00E-09	0.010273014
A	T	93.82	16.39	0.0126	3408	1.12E-08	0.009523086
T	C	358.2	46.47	0.0013	3408	1.67E-14	0.01713562
C	T	150.2	18.37	0.0091	3408	4.15E-16	0.019239116
A	G	78.03	16.23	0.0134	3408	1.58E-06	0.006736758
C	T	159.5	18.29	0.01	3408	4.20E-18	0.021827787
G	A	320.1	60.03	0.0009	3408	1.03E-07	0.008274213
A	C	112.6	23.01	0.0063	3408	1.04E-06	0.006977551
A	G	189.6	29.36	0.004	3408	1.20E-10	0.012088792
T	C	988.1	79.27	0.0004	3408	6.58E-35	0.043603608
C	T	99.32	19.64	0.0078	3408	4.51E-07	0.007448076
T	A	93.66	19.78	0.0088	3408	2.29E-06	0.006535944
A	G	200.6	42.25	0.0016	3408	2.14E-06	0.006571214
C	T	1006	78.94	0.0006	3408	2.29E-36	0.045486668

G	T	239.1	39.88	0.0019	3408	2.24E-09	0.010437405
T	C	172.3	28.61	0.0038	3408	1.93E-09	0.010530228
G	A	266.7	46.83	0.0013	3408	1.34E-08	0.009427239
A	G	127.5	26.48	0.0044	3408	1.54E-06	0.00675679
G	T	235.6	37	0.0022	3408	2.18E-10	0.011757395
G	A	81.64	17.53	0.0106	3408	3.34E-06	0.006323935
C	G	268.3	44.14	0.0015	3408	1.34E-09	0.010724923
T	C	235.3	42.7	0.0016	3408	3.85E-08	0.008831531
G	A	299.7	64.16	0.0012	3408	3.10E-06	0.006361705
A	G	308.9	44.43	0.0018	3408	4.29E-12	0.013985148
A	G	348.1	45.47	0.0016	3408	2.50E-14	0.016906487
T	C	771.6	91.49	0.0003	3408	4.87E-17	0.020444046
C	G	79.55	15.69	0.0131	3408	4.18E-07	0.007486371
C	G	84.68	17.28	0.0116	3408	1.00E-06	0.006997213
G	A	386.6	51.06	0.0012	3408	4.77E-14	0.016543135
C	G	487.3	54.77	0.0007	3408	9.16E-19	0.0227005
T	C	111.9	22.59	0.0063	3408	7.74E-07	0.007148459
G	A	62.07	12.95	0.0198	3408	1.72E-06	0.006695864
C	T	73.14	16.19	0.0126	3408	6.45E-06	0.005952832
G	A	203.9	36.54	0.0028	3408	2.60E-08	0.00905415
A	G	1285	88.96	0.0003	3408	5.98E-46	0.0576913
C	T	141	20.2	0.0079	3408	3.50E-12	0.014095188
C	G	203.1	36.33	0.0022	3408	2.44E-08	0.009087093
T	A	85.58	16.08	0.0134	3408	1.10E-07	0.008242866
G	A	193.7	32.62	0.0031	3408	3.18E-09	0.010240506
T	C	176.3	27.54	0.004	3408	1.74E-10	0.011881903
C	T	218.4	33.18	0.0029	3408	5.41E-11	0.012553548
C	T	159.2	28.32	0.0043	3408	2.05E-08	0.009187378
T	C	466.1	53.08	0.001	3408	2.51E-18	0.022124843
C	A	301.9	44.92	0.0015	3408	2.12E-11	0.013080635
T	C	186.1	33.82	0.0026	3408	4.06E-08	0.008806521
T	C	80.73	17.7	0.0107	3408	5.30E-06	0.006067096
A	G	101.4	17.52	0.0103	3408	7.68E-09	0.009733304
A	G	263	43.72	0.0016	3408	1.99E-09	0.010506655
A	T	126	27.82	0.0048	3408	6.12E-06	0.005983028
G	A	-388.2	50.03	0.9988	3408	1.11E-14	0.017359811
T	C	208.3	32.03	0.0028	3408	8.96E-11	0.012257693
C	T	87.03	16.56	0.012	3408	1.58E-07	0.008039189
T	A	121.1	19.05	0.0079	3408	2.34E-10	0.011718697
A	G	206.9	34.55	0.0026	3408	2.34E-09	0.010413081
C	A	1445	93.44	0.0003	3408	3.23E-52	0.065571698
G	A	1994	108.9	0.0003	3408	1.48E-71	0.089566047
C	T	239.6	36.44	0.0022	3408	5.54E-11	0.012526871
C	T	171.2	33.14	0.0034	3408	2.53E-07	0.007769898
G	A	116.4	24.23	0.0054	3408	1.64E-06	0.006726181
A	G	54.47	12.15	0.0217	3408	7.61E-06	0.005862852
T	C	339.6	33.81	0.0022	3408	2.09E-23	0.028752483
T	C	378.1	57.27	0.0013	3408	4.68E-11	0.012628162

A	C	38.5	7.602	0.0553	3408	4.32E-07	0.007469815
C	T	857.2	107	0.0003	3408	1.53E-15	0.018483942
T	C	297.5	55.33	0.0009	3408	8.14E-08	0.008411712
A	G	169.6	23.32	0.0057	3408	4.38E-13	0.015282925
G	A	82.52	17.15	0.0116	3408	1.56E-06	0.006747614
G	A	48.29	10.67	0.0268	3408	6.21E-06	0.005974253
T	C	436.5	60.77	0.0009	3408	8.35E-13	0.014913
A	G	31.02	6.783	0.0748	3408	4.97E-06	0.006099347
A	G	312.7	46.04	0.0015	3408	1.30E-11	0.013355078
A	G	80.7	15.86	0.0142	3408	3.78E-07	0.007539703
T	C	175.4	31.44	0.0032	3408	2.59E-08	0.009049952
T	C	85.79	14.27	0.0161	3408	2.00E-09	0.010494076
T	C	774.8	85.45	0.0004	3408	2.02E-19	0.023556069
C	G	767.7	64.23	0.0007	3408	2.77E-32	0.040232204
C	T	219.9	33.8	0.0028	3408	8.78E-11	0.012267521
G	A	119.8	24.82	0.0051	3408	1.45E-06	0.006789717
T	A	145.3	29.57	0.0041	3408	9.38E-07	0.007034981
C	T	164.9	33.88	0.0028	3408	1.18E-06	0.006903138
T	C	118.7	25.35	0.0045	3408	2.93E-06	0.006392354
G	A	413.6	45.5	0.0015	3408	1.66E-19	0.023671972
A	G	107.8	23.39	0.0059	3408	4.19E-06	0.006194112
G	A	76.16	14.08	0.0163	3408	6.74E-08	0.008512094
T	C	131.3	29.09	0.0041	3408	6.60E-06	0.005942292
A	C	131.3	26.82	0.0044	3408	1.02E-06	0.00698343
C	T	92.68	20.57	0.0078	3408	6.82E-06	0.005921403
T	C	475	55.24	0.001	3408	1.21E-17	0.021235309
G	T	404.7	62.22	0.0007	3408	8.89E-11	0.012261645
A	G	264.9	39.21	0.0019	3408	1.67E-11	0.013215781
T	C	114.2	18.22	0.0094	3408	4.11E-10	0.01139615
A	G	85.37	17.66	0.0104	3408	1.40E-06	0.006810231
C	T	50.52	10.46	0.0296	3408	1.42E-06	0.006798313
A	G	272.3	56.05	0.0009	3408	1.24E-06	0.006877762
A	G	139.5	30.32	0.0038	3408	4.31E-06	0.00617307
C	T	228.2	34.47	0.0028	3408	4.17E-11	0.012696956
A	C	272.5	27.65	0.0035	3408	1.31E-22	0.027710152
G	T	204.6	27.04	0.004	3408	4.93E-14	0.016522012
A	G	137.5	26.75	0.0044	3408	2.89E-07	0.00769315
G	T	192.2	28.52	0.0041	3408	1.88E-11	0.013151002
T	A	243.9	46.83	0.0019	3408	2.02E-07	0.007896465
G	T	184.9	31.03	0.0032	3408	2.77E-09	0.010311203
A	G	132.5	17.32	0.0097	3408	2.61E-14	0.016882694
T	C	181.1	30.9	0.0032	3408	5.04E-09	0.009978494
A	G	118.5	26.43	0.005	3408	7.60E-06	0.005863928
T	G	155	33.08	0.0026	3408	2.88E-06	0.006400943
C	A	750.9	68.52	0.0007	3408	1.70E-27	0.034039963
T	C	419.3	64.95	0.0006	3408	1.23E-10	0.012081283
T	C	1574	113.7	0.0004	3408	1.91E-42	0.053238932
G	A	51.3	10.14	0.0335	3408	4.44E-07	0.007454349

T	C	336.7	53.43	0.0009	3408	3.31E-10	0.011518209
A	G	146.7	28.68	0.0043	3408	3.28E-07	0.007618701
T	C	299.6	41.15	0.0018	3408	4.15E-13	0.015315857
T	A	35.5	7.259	0.0651	3408	1.05E-06	0.006968934
C	T	163.2	23.63	0.006	3408	6.03E-12	0.013803109
G	A	300.6	36.78	0.0025	3408	4.15E-16	0.019223175
T	G	82.59	18.51	0.0089	3408	8.42E-06	0.005807816
T	G	303	43.46	0.0015	3408	3.75E-12	0.014062279
G	A	85.98	16.13	0.0114	3408	1.05E-07	0.008268386
T	C	76.79	16.56	0.0107	3408	3.66E-06	0.006269856
A	G	130.7	17.31	0.0116	3408	5.64E-14	0.016453278
T	C	126.2	21.64	0.0065	3408	5.99E-09	0.0098808
C	G	168.8	25.4	0.0045	3408	3.43E-11	0.012793398
G	A	301.1	42.76	0.0018	3408	2.29E-12	0.014340798
A	G	167.3	35.13	0.0025	3408	1.99E-06	0.006610821
C	T	404.4	62.01	0.0007	3408	7.99E-11	0.012325739
A	G	108.2	24.02	0.0057	3408	6.84E-06	0.005918761
A	C	395.7	44.12	0.0019	3408	4.89E-19	0.023058449
G	A	162.5	32.77	0.0028	3408	7.48E-07	0.00716361
G	C	403.5	51.12	0.001	3408	3.95E-15	0.017953042
T	C	137	20.15	0.0085	3408	1.26E-11	0.013382591
G	A	142.7	29.75	0.004	3408	1.68E-06	0.006705826
A	G	118.7	22.05	0.0065	3408	7.71E-08	0.008431549
A	G	260.9	40.57	0.0021	3408	1.43E-10	0.011989474
A	G	180.4	30.92	0.0032	3408	5.91E-09	0.009889587
G	C	196	35.52	0.0023	3408	3.68E-08	0.008855309
G	A	81.84	15.59	0.0131	3408	1.60E-07	0.008021245
A	G	1826	132.8	0.0001	3408	6.61E-42	0.052560249
C	T	118.9	25.6	0.0048	3408	3.56E-06	0.006289902
A	T	-133.6	25.24	0.995	3408	1.28E-07	0.008154152
A	G	269.8	44.42	0.0015	3408	1.39E-09	0.010709057
C	G	112.8	23.94	0.0053	3408	2.55E-06	0.006472177
T	C	164.3	21.61	0.0066	3408	3.68E-14	0.016678672
G	T	281.1	52.95	0.0013	3408	1.18E-07	0.008201881
A	G	172.5	29.41	0.0035	3408	4.95E-09	0.009993705
G	A	153.6	30.11	0.0037	3408	3.60E-07	0.007578054
C	T	56.44	12.75	0.0198	3408	9.84E-06	0.005716946
A	G	123.5	20	0.0079	3408	7.32E-10	0.011064765
G	A	85.91	17.34	0.0106	3408	7.63E-07	0.0071511
C	G	229.3	39.06	0.0021	3408	4.76E-09	0.01001093
T	C	991	183.2	0.0001	3408	6.80E-08	0.008513023
C	T	375.1	62.7	0.0009	3408	2.43E-09	0.010392557
A	C	100.5	20.65	0.0076	3408	1.19E-06	0.006902151
A	C	111	19.94	0.0079	3408	2.77E-08	0.009010834
C	G	828.9	70.61	0.0007	3408	3.17E-31	0.038864827
A	G	253.3	38.53	0.0021	3408	5.64E-11	0.012522755
A	G	206.4	37.47	0.0026	3408	3.85E-08	0.008824759
G	A	369.2	61.96	0.0007	3408	2.79E-09	0.010310975

A	G	59.92	12.97	0.0195	3408	3.97E-06	0.006223758
A	C	169.6	32.72	0.0029	3408	2.31E-07	0.007821952
T	C	116.6	21.02	0.0066	3408	3.19E-08	0.008948052
A	G	328.6	60.54	0.0009	3408	6.09E-08	0.008570625
C	T	185.6	37.99	0.0021	3408	1.08E-06	0.006954834
A	G	248.5	37.09	0.0022	3408	2.41E-11	0.013000394
C	T	196.1	35.66	0.0022	3408	4.08E-08	0.008795413
A	G	138	26.37	0.0041	3408	1.76E-07	0.007971908
A	T	310	42.47	0.0018	3408	3.59E-13	0.015392963
C	A	196.4	28.32	0.0038	3408	4.89E-12	0.013915885
T	G	494.7	55.7	0.0012	3408	1.04E-18	0.022622275
G	A	185.9	33.18	0.0031	3408	2.28E-08	0.009126924
G	T	50.94	10.79	0.0301	3408	2.45E-06	0.006497471
T	C	188.4	31.51	0.0037	3408	2.46E-09	0.010380864
A	G	130.9	20.78	0.0079	3408	3.35E-10	0.011509622
T	C	149.3	26.38	0.0044	3408	1.64E-08	0.009311243
C	T	161.3	34.68	0.0028	3408	3.43E-06	0.006307583
T	C	125.6	19.43	0.0076	3408	1.16E-10	0.012112715
T	C	751.9	78.49	0.0004	3408	1.83E-21	0.026221181
A	T	219.6	33.39	0.0026	3408	5.57E-11	0.012532994
A	C	116.1	22.12	0.0059	3408	1.62E-07	0.008018592
A	C	96.61	15.83	0.0156	3408	1.17E-09	0.010810905
G	A	215.2	39.24	0.0021	3408	4.46E-08	0.008748045
A	T	45.29	9.88	0.0351	3408	4.72E-06	0.00612804
G	C	142.7	28.63	0.0037	3408	6.51E-07	0.007236879
A	G	1008	92.78	0.0003	3408	4.73E-27	0.033475391
A	T	52.34	10.84	0.0283	3408	1.43E-06	0.006794359
G	A	112.9	22.17	0.0059	3408	3.76E-07	0.007552046
A	G	175.9	37.3	0.0021	3408	2.51E-06	0.006483203
G	A	424.9	49.05	0.0013	3408	7.01E-18	0.021544525
T	A	224.1	32	0.0028	3408	3.03E-12	0.014186618
G	A	143.3	20.28	0.0079	3408	1.89E-12	0.014439108
T	C	708.1	63.75	0.0007	3408	3.47E-28	0.034936944
G	A	69.01	14.71	0.0145	3408	2.84E-06	0.006416578
A	G	245.4	36.99	0.0025	3408	3.79E-11	0.012749937
C	T	117.9	25.73	0.0056	3408	4.79E-06	0.006123236
T	C	159.6	26.7	0.0044	3408	2.51E-09	0.01037562
A	G	359.1	49.2	0.0012	3408	3.58E-13	0.01539093
A	G	520	59.93	0.0007	3408	6.19E-18	0.02161369
T	C	177.5	35.46	0.0025	3408	5.80E-07	0.007298579
A	G	114.6	24.58	0.0053	3408	3.27E-06	0.006337889
A	G	204.4	36.08	0.0032	3408	1.58E-08	0.0093295
T	C	584.7	76.44	0.0004	3408	2.60E-14	0.016878446
A	C	31.12	6.789	0.0761	3408	4.74E-06	0.006127715
G	T	479.2	79.88	0.0004	3408	2.19E-09	0.010449509
C	T	97.68	15.15	0.0128	3408	1.30E-10	0.012050942
G	A	103.8	22.24	0.0079	3408	3.14E-06	0.006351239
T	C	192.4	28.49	0.0035	3408	1.68E-11	0.013205426

A	G	59.98	10.31	0.0318	3408	6.47E-09	0.009833413
G	A	156.1	22.14	0.0066	3408	2.15E-12	0.014376789
C	A	177.1	31.66	0.004	3408	2.42E-08	0.00909801
T	C	1232	203.7	0.0001	3408	1.63E-09	0.010619477
A	G	221.2	40.33	0.0019	3408	4.44E-08	0.008749788
A	C	336.9	45.79	0.0015	3408	2.36E-13	0.015635688
C	T	124.1	19.47	0.0092	3408	2.08E-10	0.011780547
A	G	95.85	16.51	0.0114	3408	7.06E-09	0.009793017
T	C	328.1	52.32	0.001	3408	4.02E-10	0.01140761
A	G	194.3	29.94	0.0035	3408	9.89E-11	0.012206984
T	C	453.6	47.7	0.0013	3408	3.55E-21	0.025848552
T	G	407.5	40.68	0.0019	3408	2.65E-23	0.028601652
C	G	91.26	19.62	0.0076	3408	3.41E-06	0.006308338
A	T	174.7	34.15	0.0028	3408	3.28E-07	0.007620485
A	G	77.03	13.23	0.0207	3408	6.37E-09	0.00984922
T	C	62.93	13.44	0.0169	3408	2.95E-06	0.006391936
G	A	35.31	7.897	0.0555	3408	8.00E-06	0.005832185
G	A	183.9	22.31	0.0057	3408	2.32E-16	0.019547503
T	G	387.4	65.92	0.001	3408	4.58E-09	0.010032432
T	C	454.9	54.04	0.001	3408	5.58E-17	0.020368744
T	A	206	24.92	0.0047	3408	1.92E-16	0.019656982
G	C	526.2	54.32	0.0009	3408	6.51E-22	0.02679699
C	T	144.2	29.23	0.0037	3408	8.51E-07	0.007090599
G	A	658.4	82.87	0.0004	3408	2.61E-15	0.018185071
G	A	383.1	39.46	0.0019	3408	5.34E-22	0.026913007
A	G	236.2	50.2	0.0013	3408	2.64E-06	0.006454174
T	C	549.6	57.08	0.0009	3408	1.16E-21	0.026483136
T	C	335	44.28	0.0016	3408	4.92E-14	0.016517392
T	C	263.6	31.58	0.0029	3408	1.02E-16	0.020034466
G	T	183	33.09	0.0029	3408	3.42E-08	0.00889465
C	T	163.2	34.11	0.0026	3408	1.80E-06	0.006672212
A	G	105.3	18.42	0.0094	3408	1.18E-08	0.009498024
A	G	112.6	24.86	0.0045	3408	6.06E-06	0.005983681
A	C	134.7	26.48	0.0043	3408	3.81E-07	0.007535544
T	A	152.4	26.87	0.004	3408	1.54E-08	0.009350931
T	C	95.49	13.66	0.0173	3408	3.28E-12	0.014136169
C	A	93.82	20.75	0.0075	3408	6.38E-06	0.005962902
C	A	186	31.2	0.0032	3408	2.73E-09	0.010320758
T	C	394.5	51.12	0.0013	3408	1.55E-14	0.017174696
G	C	67.61	14.06	0.0161	3408	1.57E-06	0.006739302
T	A	103.3	23.04	0.0081	3408	7.58E-06	0.005863838
A	G	212.8	37.83	0.0022	3408	2.00E-08	0.009199352
T	A	1783	131.7	0.0001	3408	9.60E-41	0.05103652
C	A	297	43.55	0.0016	3408	1.07E-11	0.013463261
A	G	187	31.68	0.0029	3408	3.98E-09	0.010120353
A	G	342.1	46.48	0.0015	3408	2.29E-13	0.015646791
T	C	100	19.92	0.0082	3408	5.37E-07	0.00734044
A	G	248.6	28.49	0.004	3408	4.08E-18	0.021853533

A	G	90.23	16.28	0.0122	3408	3.21E-08	0.008932984
A	T	269.8	41.4	0.0018	3408	8.23E-11	0.012308495
A	G	141.4	20.64	0.0073	3408	8.60E-12	0.013584379
C	G	153.2	28.56	0.004	3408	8.67E-08	0.008372403
T	C	122.6	18.08	0.01	3408	1.42E-11	0.013312636
A	T	344.4	54.89	0.001	3408	3.96E-10	0.011419635
T	C	699.1	69.55	0.0007	3408	1.90E-23	0.02879362
A	G	2721	123.4	0.0001	3408	8.41E-101	0.124855316
A	T	106.4	17.4	0.0106	3408	1.07E-09	0.010852906
A	G	188	33.74	0.0029	3408	2.71E-08	0.009027913
T	C	146.6	31.15	0.0034	3408	2.63E-06	0.00645712
G	A	834.4	80.31	0.0006	3408	6.48E-25	0.030702028
A	C	287.4	43.92	0.0015	3408	6.96E-11	0.012408707
G	A	216.9	30.85	0.0031	3408	2.49E-12	0.014297336
T	G	322	41.59	0.0016	3408	1.29E-14	0.017284707
T	A	642.9	54.5	0.0009	3408	1.66E-31	0.0392296
A	G	144.1	25.27	0.0062	3408	1.30E-08	0.009451347
G	A	398.3	60.2	0.0007	3408	4.25E-11	0.012681924
T	G	573	57.77	0.001	3408	7.11E-23	0.028057282
T	C	82	13.96	0.0173	3408	4.67E-09	0.010022649
A	C	515.7	55.97	0.0009	3408	5.40E-20	0.024305137
A	T	69.54	13.18	0.0176	3408	1.41E-07	0.008102254
A	G	157.2	30.63	0.0035	3408	3.02E-07	0.007669514
T	G	150.1	32.81	0.0029	3408	4.95E-06	0.006103661
C	T	76.34	16.34	0.0117	3408	3.12E-06	0.006363967
G	A	516.8	57.96	0.001	3408	7.67E-19	0.022796783
T	A	161.3	28.25	0.0043	3408	1.23E-08	0.009475399
C	G	206.7	42.62	0.0018	3408	1.30E-06	0.006854368
C	A	985.5	78.14	0.0004	3408	1.12E-35	0.04459188
A	G	120.8	22.15	0.0067	3408	5.33E-08	0.00865193
A	G	1200	109.5	0.0001	3408	1.70E-27	0.034040322
C	T	109.9	22.89	0.0059	3408	1.64E-06	0.006718565
T	C	345.5	42.75	0.0015	3408	8.74E-16	0.018805266
C	T	154.9	31.41	0.0031	3408	8.51E-07	0.007085641
C	T	103.1	20.58	0.0073	3408	5.71E-07	0.007310389
T	C	397	48.27	0.0015	3408	2.74E-16	0.019462167
C	T	303.6	45.76	0.0016	3408	3.79E-11	0.012751417
A	G	271.1	40.3	0.0019	3408	2.02E-11	0.013104502
C	T	249.4	42.57	0.0019	3408	5.14E-09	0.009970891
T	C	851.2	92.57	0.0003	3408	6.37E-20	0.024209167
G	A	542.5	54.89	0.001	3408	9.78E-23	0.027863813
A	G	250.3	45.06	0.0015	3408	3.01E-08	0.008972745
C	T	440.2	64.38	0.0007	3408	9.46E-12	0.013532601
T	C	437.7	69.06	0.0006	3408	2.64E-10	0.011649608
G	A	76.86	17.27	0.011	3408	8.81E-06	0.005778295
A	T	-136.8	30.35	0.9962	3408	6.79E-06	0.005926167
A	G	122	24.22	0.0059	3408	5.00E-07	0.007390101
A	T	162.6	33.14	0.0031	3408	9.77E-07	0.007014222

T	C	152.8	23.81	0.0056	3408	1.58E-10	0.011940199
A	G	279.2	36.97	0.0021	3408	5.42E-14	0.016459796
T	C	416.6	51.47	0.0012	3408	7.89E-16	0.018860848
G	T	757.5	140.6	0.0001	3408	7.55E-08	0.008445235
T	C	207	33.71	0.0029	3408	9.17E-10	0.010943212
G	A	257	38.1	0.0021	3408	1.78E-11	0.013175186
G	C	252.9	28.72	0.0037	3408	2.05E-18	0.022246344
C	T	80.46	16.92	0.0117	3408	2.05E-06	0.006591551
C	G	391.9	46.66	0.0015	3408	6.53E-17	0.020279794
G	A	272.7	56.24	0.0013	3408	1.30E-06	0.006851635
C	T	202.4	32.76	0.0031	3408	7.31E-10	0.011076343
A	G	308.6	43.15	0.0015	3408	1.04E-12	0.014786353
T	A	633	123.7	0.0001	3408	3.26E-07	0.007625077
T	G	559.9	69.63	0.0006	3408	1.22E-15	0.018619415
T	C	297.2	44.49	0.0015	3408	2.78E-11	0.012924789
A	G	353.5	42.26	0.0018	3408	8.66E-17	0.020118419
A	G	432.3	52.29	0.0012	3408	1.92E-16	0.01966119
C	T	-92.72	19.13	0.9906	3408	1.31E-06	0.006845953
C	T	79.02	15.57	0.0129	3408	4.09E-07	0.00750114
A	T	386.5	52.34	0.001	3408	1.91E-13	0.015748459
A	T	289.8	41.55	0.0018	3408	3.67E-12	0.014073421
A	T	82.56	16.61	0.0119	3408	7.03E-07	0.007197199
A	G	178.6	37.81	0.0021	3408	2.42E-06	0.006504529
T	C	77.93	15.84	0.0123	3408	9.12E-07	0.00705222
T	C	255.1	33.32	0.0028	3408	2.52E-14	0.016908508
T	A	67.87	14.62	0.015	3408	3.59E-06	0.00628382
G	A	147	32.5	0.0029	3408	6.27E-06	0.005967179
A	C	126.4	28.31	0.0037	3408	8.31E-06	0.00581543
C	G	-289.7	55.18	0.9991	3408	1.61E-07	0.008022978
T	G	442.9	54.79	0.001	3408	8.69E-16	0.018813123
A	G	337.1	43.36	0.0015	3408	9.91E-15	0.017426273
C	T	117.2	23.63	0.0056	3408	7.43E-07	0.007166459
G	A	391.1	49.95	0.0012	3408	6.43E-15	0.017671036
A	C	73.87	15.3	0.0129	3408	1.43E-06	0.006793496
C	T	829.8	104	0.0003	3408	2.04E-15	0.018337611
G	T	730	96.39	0.0003	3408	4.65E-14	0.016551366
A	G	584.1	69.32	0.0006	3408	5.22E-17	0.020408115
T	C	211.5	36.04	0.0023	3408	4.80E-09	0.010004259
A	T	116.7	25.64	0.0047	3408	5.49E-06	0.006041909
T	G	167.3	36.46	0.0022	3408	4.64E-06	0.006140222
C	A	280.3	39.64	0.0019	3408	1.85E-12	0.014459517
C	T	216.5	46.11	0.0015	3408	2.77E-06	0.006427257
T	C	97.05	20.8	0.0076	3408	3.20E-06	0.006347451
A	G	140.6	30.13	0.0032	3408	3.19E-06	0.006349019
G	T	399.5	53.53	0.0018	3408	1.07E-13	0.016080482
G	A	176.8	33.12	0.0035	3408	1.01E-07	0.008292167
G	C	308.3	54.7	0.001	3408	1.88E-08	0.009235138
C	T	192.4	36.48	0.0023	3408	1.42E-07	0.008096001

T	A	156.4	31.93	0.0029	3408	1.02E-06	0.006990839
T	C	91.47	19.33	0.0084	3408	2.32E-06	0.006527545
A	G	262.4	37.13	0.0025	3408	1.91E-12	0.014443082
T	G	306.1	48.59	0.0013	3408	3.39E-10	0.011510792
T	C	118.5	17.59	0.0103	3408	1.86E-11	0.013141959
C	A	171.9	33.06	0.0029	3408	2.13E-07	0.007870725
T	A	260.8	39.75	0.0019	3408	6.23E-11	0.01247355
T	C	606.7	63.67	0.0007	3408	2.95E-21	0.025951315
G	A	862.8	179.7	0.0001	3408	1.64E-06	0.006718877
G	C	57.45	12.21	0.0213	3408	2.62E-06	0.006454121
A	G	246	36.11	0.0032	3408	1.13E-11	0.013435112
C	T	46.42	10.44	0.0307	3408	9.02E-06	0.005767633
A	G	628.3	64.46	0.0006	3408	3.68E-22	0.027121429
A	T	64.13	13.45	0.0182	3408	1.94E-06	0.006626601
T	C	224.8	31.22	0.0034	3408	7.31E-13	0.014985453
C	T	117.6	23.53	0.006	3408	6.04E-07	0.007276117
G	C	155.4	31.61	0.0031	3408	9.28E-07	0.007041812
A	G	59.95	12.74	0.0194	3408	2.62E-06	0.006455461
G	A	159.7	36.03	0.0021	3408	9.65E-06	0.005731724
G	T	52.21	10.34	0.0326	3408	4.73E-07	0.00742557
G	A	159.7	28.3	0.004	3408	1.82E-08	0.009257594
A	G	144.4	28.08	0.0041	3408	2.86E-07	0.007699874
C	G	203.7	31.66	0.0032	3408	1.41E-10	0.012000992
T	C	416.3	45.67	0.0016	3408	1.31E-19	0.023800699
T	C	115.8	22.95	0.0065	3408	4.77E-07	0.007415148
T	C	64.03	12.28	0.0204	3408	1.95E-07	0.007914433
A	G	185	29.82	0.0037	3408	6.24E-10	0.011167382
G	A	-127.4	28.37	0.996	3408	7.38E-06	0.005882451
A	G	447.6	54.04	0.001	3408	1.71E-16	0.019733051
T	G	151.8	28.99	0.0041	3408	1.74E-07	0.007981186
A	C	64.64	14.42	0.0148	3408	7.58E-06	0.005861644
G	A	48.61	9.003	0.0415	3408	7.14E-08	0.008481603
C	T	163.6	28.37	0.0053	3408	8.71E-09	0.009663427
C	A	414.6	53.64	0.001	3408	1.41E-14	0.017227976
G	C	366.1	48.63	0.0012	3408	6.56E-14	0.016357933
A	T	60.71	13.32	0.0189	3408	5.37E-06	0.006058613
A	C	434.2	63.13	0.0006	3408	7.20E-12	0.013690574
T	A	132.9	28.79	0.004	3408	4.05E-06	0.006213839
C	T	192.1	35.2	0.0026	3408	5.15E-08	0.008663451
T	C	842.4	89.95	0.0003	3408	1.34E-20	0.025089924
A	G	51.41	11.58	0.0244	3408	9.29E-06	0.005750082
T	C	228.7	45.62	0.0013	3408	5.59E-07	0.007320341
A	G	331.7	44.7	0.0015	3408	1.46E-13	0.015900664
C	G	116.2	18	0.0106	3408	1.25E-10	0.012080618
G	C	221	37.13	0.0026	3408	2.91E-09	0.010288303
A	G	180.4	34.8	0.0028	3408	2.30E-07	0.007823551
G	A	161.6	24.83	0.0053	3408	8.64E-11	0.012276235
A	G	181.9	32.71	0.0029	3408	2.90E-08	0.008992525

C	T	202.2	44.66	0.0018	3408	6.16E-06	0.005978894
A	C	143	29.16	0.0045	3408	9.77E-07	0.007007185
A	C	274.8	38.79	0.0019	3408	1.69E-12	0.014512605
A	G	47.62	9.034	0.0401	3408	1.44E-07	0.008087095
T	C	115.8	22.98	0.0059	3408	4.90E-07	0.007395943
T	G	184	34.62	0.0029	3408	1.14E-07	0.008220478
C	A	144.7	32.17	0.0028	3408	7.10E-06	0.005901532
A	G	330.6	59.91	0.0007	3408	3.68E-08	0.008856134
T	C	496.1	55.44	0.0009	3408	5.81E-19	0.022956534
T	C	97.09	21.28	0.0072	3408	5.26E-06	0.006071018
C	T	56.72	11.4	0.0254	3408	6.89E-07	0.007211406
T	C	189.1	33.53	0.0028	3408	1.84E-08	0.009246597
A	G	591.4	73.04	0.0006	3408	7.80E-16	0.018874101
A	G	352	44.95	0.0015	3408	6.36E-15	0.017675884
G	A	785.2	70.89	0.0006	3408	4.89E-28	0.03474815
A	G	99.34	16.98	0.0116	3408	5.33E-09	0.009943365
T	C	443.1	51.97	0.001	3408	2.25E-17	0.020884892
T	C	135.5	30.29	0.0037	3408	7.97E-06	0.005837646
G	A	146.5	27.72	0.0045	3408	1.33E-07	0.008129135
A	G	211.7	33.34	0.0026	3408	2.42E-10	0.011692384
T	C	121.9	21.11	0.007	3408	8.32E-09	0.00968953
T	C	104.7	20.88	0.0072	3408	5.67E-07	0.007323866
G	A	128.7	26.9	0.0044	3408	1.79E-06	0.006671841
T	C	299.9	60.1	0.0009	3408	6.32E-07	0.00725342
A	G	165.2	30.55	0.0032	3408	6.77E-08	0.008507219
T	C	101.9	22.39	0.0059	3408	5.53E-06	0.006041003
A	G	447.6	52.44	0.001	3408	2.09E-17	0.020929987
T	C	268.4	41.66	0.0016	3408	1.35E-10	0.012032875
C	G	142.1	31.5	0.0031	3408	6.72E-06	0.005935836
A	G	140.8	29.43	0.004	3408	1.80E-06	0.006671418
G	A	103.6	21.07	0.0069	3408	9.19E-07	0.007044024
A	T	93.79	15.05	0.0135	3408	5.14E-10	0.011267288
T	C	316	49.25	0.0013	3408	1.59E-10	0.011935684
T	G	131	26.01	0.0045	3408	5.01E-07	0.007388252
A	G	311	47.64	0.0015	3408	7.72E-11	0.012350387
C	T	854.9	74.11	0.0006	3408	3.13E-30	0.037578775
A	G	-233.6	40.25	0.9974	3408	7.08E-09	0.009786852
G	T	64.3	12.79	0.02	3408	5.24E-07	0.007361602
A	G	35.6	7.782	0.0568	3408	4.94E-06	0.006103223
T	C	82.54	15.29	0.0136	3408	7.17E-08	0.008478451
A	C	207	22.8	0.006	3408	1.80E-19	0.023615242
A	G	99.23	21.38	0.0069	3408	3.60E-06	0.006281083
A	G	196.9	39.12	0.0021	3408	5.08E-07	0.007378664
T	C	122.1	24.31	0.0053	3408	5.42E-07	0.007347827
T	C	286.8	38.31	0.0025	3408	8.99E-14	0.016178957
T	A	362.8	46.21	0.0018	3408	5.51E-15	0.017765531
C	A	364.4	54.53	0.001	3408	2.74E-11	0.012933985
A	G	939.1	130.1	0.0001	3408	6.41E-13	0.015058439

A	G	234	44.62	0.0016	3408	1.66E-07	0.008005387
T	C	469.1	52.88	0.001	3408	1.14E-18	0.022570127
T	A	160.7	28.09	0.0041	3408	1.16E-08	0.009512132
G	A	139.4	30.72	0.0032	3408	5.89E-06	0.006005752
T	C	516.5	52.39	0.0018	3408	1.25E-22	0.027728838
G	C	187.4	33.91	0.0026	3408	3.51E-08	0.008881972
A	G	319.6	44.35	0.0016	3408	7.07E-13	0.015009245
T	C	1015	81.57	0.0004	3408	8.89E-35	0.043458563
T	C	131.3	26.91	0.0041	3408	1.12E-06	0.00693712
C	A	148.2	32.28	0.0029	3408	4.56E-06	0.006146841
G	T	217.6	37.05	0.0023	3408	4.72E-09	0.010020011
G	C	97.34	19.04	0.0089	3408	3.37E-07	0.007610816
T	G	110.1	20.78	0.0067	3408	1.23E-07	0.008169984
A	G	227.5	49.6	0.0012	3408	4.67E-06	0.006135178
T	C	69.9	12.06	0.0211	3408	7.48E-09	0.009761131
C	T	214.7	38.13	0.0022	3408	1.94E-08	0.009217417
G	A	111.8	18.01	0.0092	3408	6.12E-10	0.011180815
A	C	417.2	62.38	0.0007	3408	2.64E-11	0.012954935
A	G	338.4	41.59	0.0021	3408	5.64E-16	0.019055817
A	G	643.8	80.36	0.0006	3408	1.54E-15	0.018485
T	C	184.3	35.38	0.0023	3408	2.01E-07	0.007899346
C	T	1057	134.1	0.0001	3408	4.18E-15	0.017903867
A	G	149.1	21.18	0.0069	3408	2.36E-12	0.014332899
G	C	202.5	24.61	0.005	3408	2.68E-16	0.019479769
T	C	250	43.75	0.0016	3408	1.20E-08	0.009490367
T	C	826.8	69.68	0.0007	3408	7.54E-32	0.039673804
A	G	489.9	50.73	0.001	3408	8.85E-22	0.026635513
T	G	1000	72.99	0.0006	3408	1.18E-41	0.052202266
A	G	158.6	29.18	0.0037	3408	5.83E-08	0.008593851
C	T	105.4	20.01	0.0072	3408	1.47E-07	0.008075438
A	G	262.1	43.79	0.0016	3408	2.38E-09	0.010402629
G	A	165.5	32.04	0.0029	3408	2.55E-07	0.007768273
G	C	609.3	53.09	0.0012	3408	6.17E-30	0.037210761
A	G	315	62.47	0.0007	3408	4.85E-07	0.007405432
G	A	46.19	10.19	0.0323	3408	5.98E-06	0.005992906
G	A	128.9	25.73	0.0045	3408	5.77E-07	0.007310383
A	G	221.5	34.4	0.0023	3408	1.36E-10	0.012019308
T	C	160.3	31.58	0.0032	3408	4.04E-07	0.007503642
C	G	378.7	48.86	0.0012	3408	1.18E-14	0.017321894
A	C	148.1	30.2	0.0035	3408	9.78E-07	0.007007172
C	T	751.8	64.6	0.0006	3408	1.00E-30	0.038222123
A	T	267.2	40.47	0.0018	3408	4.68E-11	0.012629526
G	T	559.6	56.91	0.0009	3408	1.61E-22	0.02758854
A	T	97.1	21.94	0.0089	3408	9.90E-06	0.00571448
A	G	169.2	33.84	0.0028	3408	6.05E-07	0.00728226
G	T	197.3	29.42	0.0034	3408	2.34E-11	0.013024931
T	C	214.8	37.5	0.0021	3408	1.10E-08	0.009535541
C	T	534.9	55.24	0.001	3408	6.75E-22	0.026776323

T	C	282.1	43.4	0.0018	3408	9.20E-11	0.012245489
G	C	46.78	10.27	0.0312	3408	5.49E-06	0.006051234
T	C	172.1	31.46	0.0032	3408	4.77E-08	0.00870458
A	T	81.08	15.08	0.0145	3408	8.13E-08	0.008411178
C	T	171.6	32.8	0.0028	3408	1.78E-07	0.007967338
C	T	-548.4	57.56	0.999	3408	2.95E-21	0.025944042
T	G	37.95	8.38	0.049	3408	6.13E-06	0.005981778
T	G	167.8	35.69	0.0026	3408	2.68E-06	0.006444414
A	T	199	34.88	0.0029	3408	1.26E-08	0.009460744
T	C	-78.38	17.32	0.9887	3408	6.22E-06	0.005973285
A	G	49.89	10.56	0.0289	3408	2.38E-06	0.006506756
T	C	88.59	18.82	0.0082	3408	2.63E-06	0.006459755
A	G	122.4	24.8	0.005	3408	8.35E-07	0.007096869
A	G	355.7	52.34	0.0013	3408	1.26E-11	0.013370717
G	C	121.1	23.01	0.0059	3408	1.53E-07	0.008061948
A	C	139.4	26.93	0.0041	3408	2.41E-07	0.007801031
A	G	89.25	19.59	0.0073	3408	5.43E-06	0.006053562
A	G	197.3	38.35	0.0021	3408	2.85E-07	0.00770662
G	C	55.83	12.4	0.0214	3408	6.94E-06	0.005913119
A	C	246.8	37.81	0.0021	3408	7.63E-11	0.01234757
T	C	417.3	51.4	0.0012	3408	6.59E-16	0.018973681
T	C	-659.9	84.5	0.9994	3408	7.60E-15	0.017580858
C	T	173.5	32.21	0.0035	3408	7.70E-08	0.008441821
G	T	95.58	21.26	0.0065	3408	7.18E-06	0.005895761
T	G	18.78	4.013	0.0032	3425	2.97E-06	0.006353662
T	C	-2.987	0.661	0.0749	3425	6.48E-06	0.005921524
A	G	60.43	13.57	0.0001	3425	8.67E-06	0.005756753
G	A	18.22	3.787	0.0022	3425	1.56E-06	0.006713056
G	T	17.66	3.916	0.0019	3425	6.74E-06	0.005902884
C	G	49.13	10.45	0.0003	3425	2.68E-06	0.006412194
C	T	1.721	0.384	0.2791	3425	7.70E-06	0.005827393
G	A	-2.745	0.372	0.3508	3425	1.96E-13	0.015649058
C	T	11.65	2.417	0.0054	3425	1.48E-06	0.00673755
T	C	11.48	2.466	0.0047	3425	3.37E-06	0.006287785
A	C	27.8	6.049	0.0009	3425	4.48E-06	0.006129031
C	T	37.99	6.487	0.001	3425	5.20E-09	0.009914321
G	A	1.65	0.349	0.5321	3425	2.33E-06	0.006494916
T	C	17.82	3.901	0.0019	3425	5.13E-06	0.006055709
G	T	5.776	1.273	0.0207	3425	5.92E-06	0.005974951
A	G	36.64	6.772	0.0006	3425	6.72E-08	0.008474621
T	C	16.82	3.744	0.0023	3425	7.27E-06	0.005858252
G	A	9.772	1.873	0.0092	3425	1.93E-07	0.00788484
C	T	3.713	0.818	0.0512	3425	5.82E-06	0.005981134
C	G	6.923	1.375	0.0166	3425	5.05E-07	0.00734717
C	G	3.528	0.795	0.0558	3425	9.46E-06	0.005712767
T	C	7.144	1.487	0.0136	3425	1.62E-06	0.006693965
G	A	1.611	0.35	0.5133	3425	4.22E-06	0.00616174
C	G	1.727	0.383	0.7149	3425	6.68E-06	0.005907545

G	C	9.884	2.114	0.0069	3425	3.04E-06	0.006342082
T	C	21.72	4.38	0.0016	3425	7.47E-07	0.007128593
T	A	9.832	2.207	0.0067	3425	8.65E-06	0.005761151
T	A	64.01	13.7	0.0001	3425	3.08E-06	0.006333367
A	G	59.5	13.1	0.0001	3425	5.82E-06	0.005987186
T	C	2.222	0.489	0.147	3425	5.62E-06	0.006002139
T	C	-3.328	0.726	0.9397	3425	4.66E-06	0.006106203
C	A	10.36	2.03	0.0073	3425	3.55E-07	0.007547042
C	T	2.946	0.621	0.0867	3425	2.14E-06	0.006536321
A	G	42.58	9.265	0.0003	3425	4.46E-06	0.006129004
C	T	-2.611	0.517	0.1345	3425	4.62E-07	0.007391795
T	G	5.923	1.324	0.019	3425	8.00E-06	0.005809204
C	G	13.26	2.594	0.0007	3425	3.34E-07	0.007571566
C	A	15.6	3.164	0.0007	3425	8.58E-07	0.007047646
C	G	12.68	2.647	0.0013	3425	1.72E-06	0.006655336
T	A	2.407	0.535	0.0197	3425	6.91E-06	0.005886163
G	A	4.942	0.987	0.0063	3425	5.77E-07	0.007269723
C	T	15.69	3.028	0.0007	3425	2.33E-07	0.007778257
T	C	13.78	2.988	0.0006	3425	4.16E-06	0.006171463
C	T	2.264	0.474	0.0263	3425	1.86E-06	0.006614094
A	G	27.24	5.333	0.0001	3425	3.44E-07	0.007559878
A	G	51.58	5.751	0.0001	3425	4.82E-19	0.022947391
A	G	9.265	1.585	0.0023	3425	5.47E-09	0.009877807
C	T	13.71	2.962	0.0007	3425	3.80E-06	0.006216359
C	G	1.998	0.391	0.0377	3425	3.39E-07	0.00756621
G	C	7.853	1.619	0.002	3425	1.28E-06	0.006822504
T	G	14.34	2.896	0.0006	3425	7.73E-07	0.007107922
T	C	33.4	5.736	0.0001	3425	6.30E-09	0.009802479
C	T	9.036	1.652	0.0022	3425	4.84E-08	0.008659536
G	A	31.04	4.31	0.0003	3425	7.30E-13	0.014917665
G	A	8.874	1.564	0.0022	3425	1.51E-08	0.009311973
A	G	2.823	0.616	0.0147	3425	4.79E-06	0.006088699
G	T	0.773	0.151	0.5263	3425	3.07E-07	0.007623395
C	G	29.23	4.452	0.0003	3425	5.97E-11	0.012429534
T	C	8.302	1.533	0.0022	3425	6.58E-08	0.008490193
A	G	39.6	8.915	0.0001	3425	9.17E-06	0.00572786
G	A	35.37	4.058	0.0003	3425	4.44E-18	0.021699891
G	A	5.306	1.184	0.0038	3425	7.66E-06	0.005829501
G	T	9.274	1.681	0.0019	3425	3.74E-08	0.008808375
A	G	33.31	5.744	0.0001	3425	7.31E-09	0.00972336
T	C	10.01	2.227	0.001	3425	7.23E-06	0.005864255
A	G	18.17	3.072	0.0006	3425	3.62E-09	0.010110983
G	A	0.9087	0.205	0.1635	3425	9.10E-06	0.005731884
A	G	8.176	1.62	0.0028	3425	4.73E-07	0.007381988
G	C	8.765	1.905	0.0018	3425	4.36E-06	0.006142953
G	A	12.99	2.578	0.0009	3425	4.96E-07	0.007358416
T	C	32.81	5.716	0.0001	3425	1.02E-08	0.009528172
T	C	5.185	1.061	0.0047	3425	1.07E-06	0.0069245

A	G	26.82	4.296	0.0003	3425	4.81E-10	0.011251598
T	G	14.57	3.276	0.0004	3425	8.91E-06	0.005742087
C	T	1.87	0.419	0.0337	3425	8.16E-06	0.005795719
T	A	10.71	2.132	0.0012	3425	5.39E-07	0.007314015
T	C	8.69	1.67	0.0019	3425	2.08E-07	0.007843792
A	G	17.96	3.205	0.0006	3425	2.27E-08	0.009085155
T	C	26.23	4.191	0.0006	3425	4.38E-10	0.01130738
G	T	2.937	0.546	0.0207	3425	7.95E-08	0.008380428
A	C	25	4.08	0.0003	3425	1.00E-09	0.010843368
A	G	14.59	2.874	0.0007	3425	4.08E-07	0.007468285
A	C	7.95	1.654	0.0019	3425	1.60E-06	0.006700127
A	T	18.49	3.731	0.0004	3425	7.51E-07	0.007119667
G	A	25.15	3.141	0.0006	3425	1.59E-15	0.018374908
A	G	17.06	3.509	0.0004	3425	1.22E-06	0.006853996
G	A	3.698	0.767	0.0096	3425	1.49E-06	0.006743065
C	T	38.22	4.737	0.0001	3425	9.73E-16	0.018652491
A	G	20.36	4.31	0.0003	3425	2.42E-06	0.006473215
A	C	12.67	2.608	0.0007	3425	1.23E-06	0.006843764
G	A	15.63	2.895	0.0006	3425	7.15E-08	0.008438782
A	G	19.45	3.678	0.0004	3425	1.31E-07	0.008098854
G	A	19.81	3.224	0.0006	3425	8.99E-10	0.010903278
C	A	7.861	1.731	0.0019	3425	5.79E-06	0.005985406
T	C	49	5.703	0.0001	3425	1.26E-17	0.021099083
A	G	10.8	1.881	0.0013	3425	1.00E-08	0.00953343
T	C	7.334	1.616	0.0023	3425	5.87E-06	0.005977707
G	A	14.11	2.744	0.0012	3425	2.86E-07	0.007660997
T	C	3.271	0.713	0.0112	3425	4.64E-06	0.006109167
C	T	22.4	3.301	0.0006	3425	1.35E-11	0.013266136
A	G	19.55	3.788	0.0004	3425	2.60E-07	0.007717001
C	G	37.39	7.091	0.0001	3425	1.43E-07	0.008052377
C	T	19.78	3.684	0.0004	3425	8.40E-08	0.008346658
G	A	35.54	7.417	0.0001	3425	1.73E-06	0.006659108
C	T	3.649	0.786	0.0091	3425	3.54E-06	0.006258168
G	A	11.34	2.525	0.0009	3425	7.39E-06	0.005854539
C	T	3.834	0.762	0.0101	3425	5.04E-07	0.007344938
T	C	16.05	3.457	0.0004	3425	3.59E-06	0.006254118
T	C	27.58	5.685	0.0001	3425	1.28E-06	0.006824846
T	C	15.37	3.316	0.0006	3425	3.72E-06	0.00623365
C	G	11.37	2.328	0.0007	3425	1.07E-06	0.006916402
C	T	3.153	0.642	0.0134	3425	9.43E-07	0.006995261
A	G	10.23	2.22	0.0013	3425	4.19E-06	0.006161699
T	C	9.611	2.144	0.0012	3425	7.59E-06	0.005832925
G	A	34.45	4.832	0.0003	3425	1.22E-12	0.014623994
G	A	4.521	0.804	0.0092	3425	2.04E-08	0.009140801
C	T	24.55	4.474	0.0003	3425	4.37E-08	0.008714634
C	G	3.222	0.591	0.0166	3425	5.25E-08	0.008611925
C	A	25.69	4.095	0.0003	3425	3.97E-10	0.011360509
A	G	9.457	2.127	0.0012	3425	9.02E-06	0.005738677

A	G	21.5	3.886	0.0003	3425	3.38E-08	0.008858221
T	C	37.38	4.922	0.0004	3425	3.94E-14	0.016560838
T	C	4.563	0.909	0.0066	3425	5.48E-07	0.007300278
G	A	13.03	1.94	0.0015	3425	2.13E-11	0.012999959
C	A	14.06	2.974	0.0007	3425	2.35E-06	0.006483407
C	G	1.023	0.218	0.1425	3425	2.80E-06	0.00639426
A	C	15.07	2.977	0.0006	3425	4.40E-07	0.007426275
C	G	4.332	0.912	0.0066	3425	2.11E-06	0.006545905
A	C	6.228	1.155	0.0036	3425	7.39E-08	0.008417853
T	A	4.133	0.843	0.0079	3425	9.81E-07	0.006974047
G	T	19.66	4.1	0.0003	3425	1.70E-06	0.006668573
G	A	8.527	1.56	0.0025	3425	4.90E-08	0.0086479
T	C	19.5	4.4	0.0004	3425	9.65E-06	0.005701904
T	C	8.214	1.748	0.0018	3425	2.70E-06	0.006405824
A	G	3.909	0.732	0.0096	3425	1.01E-07	0.008248537
C	G	18.54	2.753	0.0007	3425	1.90E-11	0.01306873
T	C	5.693	1.172	0.0039	3425	1.24E-06	0.006842029
T	G	33.95	5.804	0.0001	3425	5.41E-09	0.009891157
A	T	5.888	1.203	0.0041	3425	1.04E-06	0.006945712
T	C	10.5	2.211	0.001	3425	2.10E-06	0.006541693
T	C	32.87	5.796	0.0001	3425	1.53E-08	0.009302998
T	C	4.477	0.961	0.006	3425	3.30E-06	0.00629555
A	G	36.56	6.272	0.0001	3425	6.06E-09	0.009823188
C	T	5.545	1.177	0.0039	3425	2.54E-06	0.006438495
G	A	13.01	2.45	0.0009	3425	1.16E-07	0.008165841
C	G	3.632	0.81	0.0089	3425	7.66E-06	0.005830325
C	G	15.3	3.059	0.0007	3425	5.93E-07	0.007251081
A	G	6.969	1.557	0.0026	3425	7.90E-06	0.005815274
A	C	12.51	1.85	0.0019	3425	1.58E-11	0.013175003
A	G	35.15	5.793	0.0001	3425	1.44E-09	0.01063505
A	C	12.28	2.651	0.0009	3425	3.74E-06	0.006225932
A	G	13.5	2.962	0.0006	3425	5.34E-06	0.006028521
A	C	3.047	0.664	0.0158	3425	4.55E-06	0.006117957
G	C	5.704	1.27	0.0036	3425	7.29E-06	0.005855186
A	C	9.939	2.208	0.0012	3425	7.00E-06	0.005881187
C	A	6.492	1.196	0.0038	3425	6.12E-08	0.008529311
G	A	-14.67	3.168	0.9994	3425	3.78E-06	0.006221839
G	A	9.082	1.693	0.0019	3425	8.69E-08	0.008332105
T	G	13.13	2.792	0.001	3425	2.66E-06	0.006415681
T	C	10.21	2.298	0.001	3425	9.19E-06	0.005730532
T	C	51.17	8.845	0.0001	3425	7.92E-09	0.009677236
G	A	46.68	7.967	0.0001	3425	5.07E-09	0.009923848
T	C	15.69	3.443	0.0007	3425	5.37E-06	0.006026788
A	G	5.664	1.165	0.0039	3425	1.21E-06	0.006854048
T	C	14.87	2.792	0.0007	3425	1.06E-07	0.008213884
T	G	9.768	1.725	0.0019	3425	1.62E-08	0.009275248
[truncated: 504,210 more chars]
